# Automated identification of spotted‐fever tick vectors using convolutional neural networks

**DOI:** 10.1111/mve.12822

**Published:** 2025-07-04

**Authors:** Isadora R. C. Gomes, Vinícius L. Miranda, José Fabrício C. Leal, Igor P. Oliveira, Paula J. Silva, Karla Bitencourth, Claudio M. Rodrigues, Liege R. Siqueira, Marcelo B. Labruna, Gilberto S. Gazeta, Marinete Amorim, Rodrigo Gurgel‐Gonçalves

**Affiliations:** ^1^ Programa de Pós‐graduação em Medicina Tropical, Núcleo de Medicina Tropical Faculdade de Medicina, Universidade de Brasília Brasília Brazil; ^2^ Laboratório de Parasitologia Médica e Biologia de Vetores, Faculdade de Medicina Universidade de Brasília Brasília Brazil; ^3^ Laboratório de Carrapatos e outros Artrópodes Ápteros e Coleção de Artrópodes Vetores Ápteros de Importância em Saúde das Comunidades Instituto Oswaldo Cruz, Fiocruz Rio de Janeiro Brazil; ^4^ Centro de Desenvolvimento Tecnológico em Saúde Instituto Oswaldo Cruz, Fiocruz Rio de Janeiro Brazil; ^5^ Departamento de Medicina Veterinária Preventiva e Saúde Animal, Faculdade de Medicina Veterinária e Zootecnia Universidade de São Paulo São Paulo Brazil

**Keywords:** *Amblyomma*, artificial intelligence, health surveillance, machine learning, one health, tick‐borne pathogens

## Abstract

Ticks are key ectoparasites for the One Health approach, as they are vectors of pathogens that infect humans, domestic and wild animals. The bacteria *Rickettsia rickettsii* and *R. parkeri* are the aetiological agents of tick‐borne spotted fever (SF) in South America, where *Amblyomma sculptum*, *A. aureolatum*, *A. ovale* and *A. triste* are the main vectors. Studies in the medical and biological fields show that artificial intelligence, through machine learning, has great potential to assist researchers and health professionals in image identification practices. The aim of this study was to evaluate the performance of the Convolutional Neural Networks (CNN) AlexNet, ResNet‐50 and MobileNetV2 for identifying tick species transmitting SF bioagents. We organised an image database with the following groups: females (368), males (458), dorsal (423), ventral (403), low resolution (328), high resolution (498) and all together (sex+position+resolution = 826), to identify the three main vectors of SF bioagents (*Amblyomma aureolatum*, *A. ovale* and *A. sculptum*), two other possible vectors (*A. triste* and *A. dubitatum*) and the species *A. cajennense* sensu stricto (s.s.), which has similar morphology to *A. sculptum* but no known vectorial capacity. To evaluate the network's performance, we measured accuracy, sensitivity and specificity. We used Grad‐CAM to highlight the regions of the images most relevant to the predictions. CNNs achieved accuracy rates of ~90% in identifying ticks and showed sensitivities of 59%–100% according to species, sex, position or image resolution. When considering all images, both AlexNet and MobileNetV2 recorded the best sensitivity and specificity values in identifying SF vectors. The most relevant areas for classifying species varied according to algorithms. Our results support the idea of using CNNs for the automated identification of tick species transmitting SF bioagents in South America. Our database could support the development of tick identification apps to aid public health surveillance and contribute to citizen science.

## Graphical abstract

- We evaluate the performance of convolutional neural networks (CNN) AlexNet, ResNet‐50 and MobileNetV2 for the automated identification of tick species capable of transmitting spotted fever.
- CNNs achieved accuracy rates of ~90% in identifying ticks and showed sensitivities of 59%–100% according to species, sex, position or image resolution.
- Our results support the idea of using CNNs for the automated identification of tick species transmitting SF bioagents in South America.

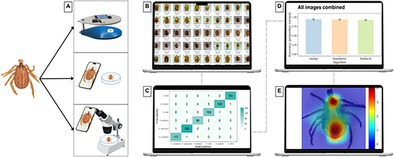

## INTRODUCTION

Ticks (Arachnida, Ixodida) are blood‐sucking ectoparasites of mammals, birds, reptiles and amphibians, represented by more than 970 species distributed worldwide (Dantas‐Torres, 2018; Guglielmone et al., 2023). Ticks are key to the One Health approach, as they are vectors of pathogens that infect humans, domestic animals and wild animals (Dantas‐Torres et al., 2012), such as the bacteria *Rickettsia rickettsii* and *R. parkeri* in South America (Faccini‐Martínez et al., 2018; Moura‐Martiniano et al., 2014), which cause spotted fever (SF).

In South America, the main vectors of SF bioagents are *Amblyomma sculptum* Berlese, 1888, *A. aureolatum* (Pallas, 1772), *A. ovale* Koch, 1844 and *A. triste* Koch, 1844 (Barbieri et al., 2019; Eremeeva & Dasch, 2015; Parola et al., 2013; Szabó et al., 2013). *A. triste* belongs to the group known as the “maculatum group” (Lado et al., 2018), which encompasses the species *A. triste*, *A. tigrinum* Koch, 1844 and *A. maculatum* Koch, 1844, which are morphologically similar. Of these species, *A. triste* is considered the vector of *R. parkeri* in Brazil (Barbieri et al., 2019). Furthermore, the species *A. dubitatum* Neumann, 1899 was also identified as a potential vector of the disease (Matias et al., 2015). The clinical manifestations of SF are strikingly similar to other acute febrile illnesses. Rapid tests for SF are lacking in hospitals, increasing the risk of severe cases and deaths due to late diagnosis (Blanton, 2019). The Brazilian Ministry of Health reported ~3500 confirmed cases and 1050 deaths from SF between 2007 and 2024. Between 2007 and 2017, an average of 150 cases of SF per year were registered in Brazil, and in recent years the number of cases has increased, reaching 420 cases by 2024. The highest numbers of cases and deaths were concentrated in the Southeast and South regions. However, the Southeast region accounted for the highest number of deaths, with 678 cases compared to 5 in the South region (Ministério da Saúde, 2024). The deaths are associated with *R. rickettsii*, and cases caused by *R. parkeri* strains have no reported deaths (Oliveira et al., 2016; Silva‐Ramos et al., 2021).

The correct identification of ticks is essential for the surveillance of SF and other diseases of public health concern, as there are differences in vectorial competence between tick species. Labruna et al. (2011) and Soares et al. (2012) demonstrated that *A. aureolatum* had a greater vectorial competence to transmit *R. rickettsii* when compared to *A. sculptum*. This is because *A. sculptum* was much less susceptible to the rickettsial infection, being unable to maintain an infection through successive tick generations. Instead, horizontal transmission from vertebrate hosts is an important means of acquiring the infection (Gerardi et al., 2019). Furthermore, ixodid species can transmit different rickettsiae, resulting in different disease conditions and outcomes. In Brazil, *R. rickettsii* causes severe SF and is transmitted by *A. sculptum* and *A. aureolatum*. In contrast, pathogenic *R. parkeri* strains cause less severe clinical cases and are associated with *A. ovale*, *A. triste* and *A. tigrinum* (Barbieri et al., 2019; Ministério da Saúde, 2024; Szabó et al., 2013). The correct identification of *Amblyomma* ticks and their rickettsial infection is important for effective surveillance and vector control, with implications for SF transmission.

Tick species are distinguished by analysing external characters and using keys (Barros‐Battesti et al., 2006; Dantas‐Torres et al., 2019; Gianizella & Nascimento, 2017; Nava et al., 2017). However, this process can be complex and difficult to understand, especially for non‐specialists. This difficulty is even more evident in morphologically similar species, such as some of the genus *Amblyomma*, which include important vectors of SF bioagents. A relevant example is the *Amblyomma cajennense* complex, composed of six visually similar species, of which only *Amblyomma sculptum* is recognised as a proven vector of SF (Martins et al., 2016). Molecular taxonomy methods have been tested for tick identification (Quadros et al., 2021; Ramos et al., 2014), but are expensive and require specialised laboratories. Therefore, innovative approaches are needed to identify *Amblyomma* ticks.

Machine learning (ML) has been used to identify insect vectors such as mosquitoes and triatomines (Araújo et al., 2024; Gurgel‐Gonçalves et al., 2017; Miranda et al., 2024; Motta et al., 2019). Furthermore, ML has been applied to identify infectious diseases transmitted by some of these insects, such as malaria and Chagas disease through the detection of parasites in blood smears and leishmaniasis through the identification of skin lesions (Leal et al., 2023; Montalbo & Alon, 2021; Morais et al., 2022). Convolutional neural networks (CNN) have been successfully applied to classify tick pictures in North America (Akbarian et al., 2021; Justen et al., 2021; Luo et al., 2022; Omodior et al., 2021). A similar study is needed for South American ticks.

There are 77 tick species registered in Brazil, most of which belong to the Ixodidae family (Labruna et al., 2024). Despite the diversity, no ML study has identified tick species in South America, especially those involved in the transmission of bioagents that cause SF. To improve tick surveillance, we need to develop new, more accessible, rapid and effective identification methods. This study evaluates the performance of the CNNs AlexNet (Krizhevsky et al., 2012), ResNet‐50 (He et al., 2016) and MobileNetV2 (Sandler et al., 2018) in identifying tick species that transmit SF bioagents. To do so, we organised and analysed an image bank of six *Amblyomma* species. CNNs achieved an accuracy rate of ~90% in identifying ticks and showed sensitivities of 59%–100% according to species, sex, position or image resolution.

## MATERIALS AND METHODS

### Tick species

**FIGURE 1:**
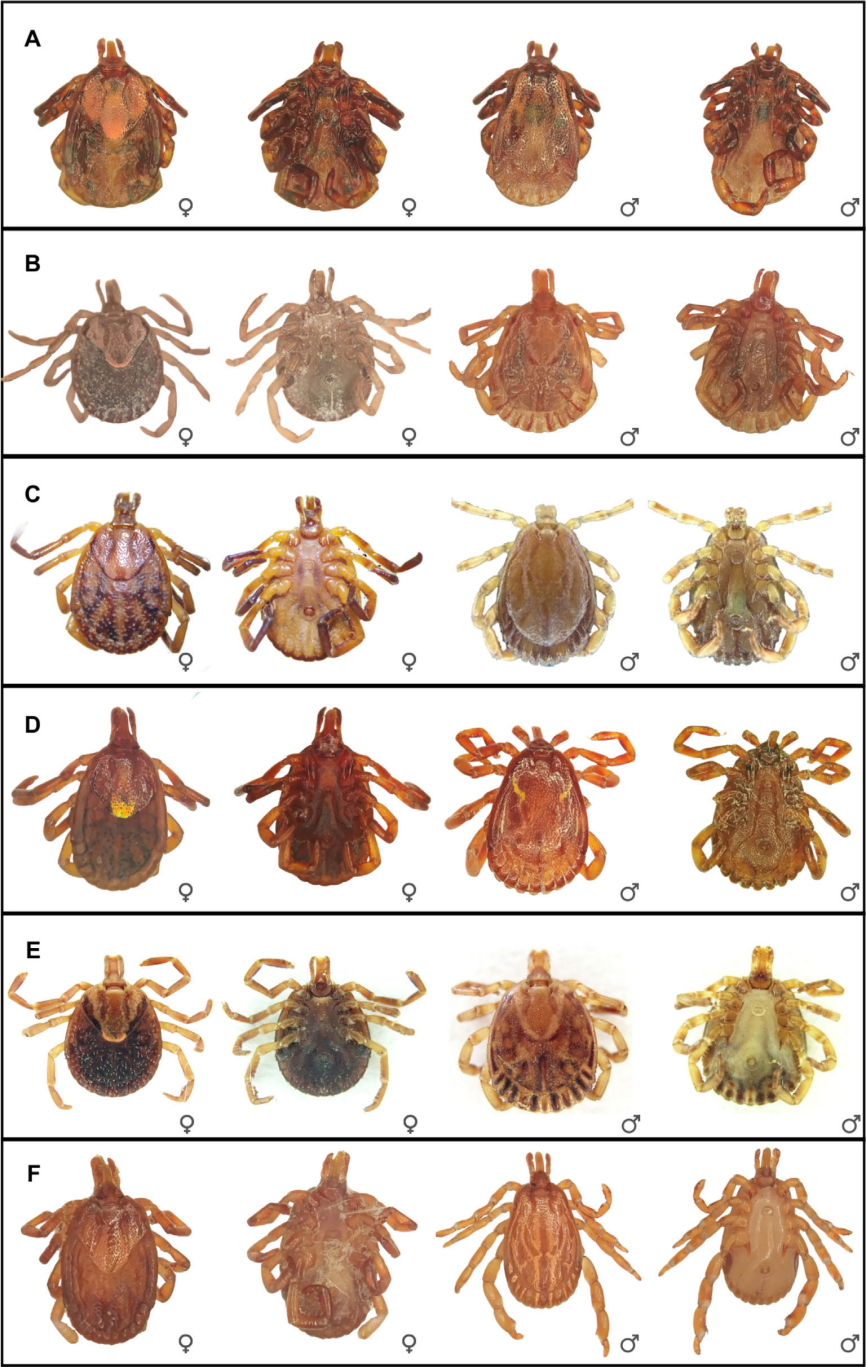
Dorsal and ventral views of female and male specimens of the tick species included in the study. (a) *Amblyomma aureolatum*. (b) *A. cajennense*. (c) *A. dubitatum*. (d) *A. ovale*. (e) *A. sculptum*. (f) *A. triste*. Images were obtained in Ribeiro (2023) and during the present study.

Our study included the species *A. sculptum*, *A. ovale*, *A. aureolatum* and *A. triste*, all vectors of SF bioagents in South America (Oliveira et al., 2016; Szabó et al., 2013; Venzal et al., 2012). The species *A. dubitatum* was also included because it usually occurs in sympatry with *A. sculptum* and may be a possible vector of SF bioagents (Matias et al., 2015). We also included the species *A. cajennense* sensu stricto (s.s.), because it is morphologically very similar to *A. sculptum*– both *A. sculptum* and *A. cajennense* s.s. (hereafter just *A. cajennense*) belong to the *Amblyomma cajennense* species complex, together with four other species; however, only *A. sculptum* is known to be an important vector of SF bioagents in Brazil (Martins et al., 2016; Nava et al., 2014). Furthermore, there are records of simultaneous occurrence of these species in Brazil (Martins et al., 2016). Ticks are involved in different SF epidemic scenarios; therefore, correct species identification is essential for acarological surveillance (Oliveira et al., 2016; Szabó et al., 2013; Venzal et al., 2012). We identified the species using printed keys (Dantas‐Torres et al., 2019). The species included in the study are shown in Figure 1.

### Picture database

We worked with 826 pictures of adult ticks (Table 1). The pictures were obtained from the Hospital Veterinário at the Universidade de Brasília (UnB), the Universidade Católica de Brasília (UCB), the Coleção de Artrópodes Vetores Ápteros de Importância em Saúde das Comunidades at the Instituto Oswaldo Cruz (CAVAISC) and the Coleção Nacional de Carrapatos Danilo Gonçalves Saraiva (CNC) in the Departamento de Medicina Veterinária Preventiva e Saúde Animal, Faculdade de Medicina Veterinária e Zootecnia at the Universidade de São Paulo (USP), with permission, and images from Ribeiro (2023). We used the device described by Gurgel‐Gonçalves et al. (2017) and mobile phone cameras (Xiaomi Mi 8 Lite, 12mp, sensor: 1/2.5, aperture: f/1.9, and Samsung Galaxy M51, 64mp, sensor: 1/1.7, aperture: f/1.8), coupled with Leica MZ16 and Zeiss Stemi DV4 optical stereomicroscopes. The locations where the specimens were collected are described in Table S1. All pictures used in our study are available (DOI: https://doi.org/10.6084/m9.figshare.28711394.v1).

**TABLE 1:** Number of pictures of ticks by species, sex, position, picture resolution and all pictures.

Species	Female	Male	Dorsal	Ventral	Low resolution	High resolution	Total
*Amblyomma aureolatum*	58	63	63	58	53	68	121
*Amblyomma cajennense*	82	83	83	82	82	83	165
*Amblyomma dubitatum*	34	75	56	52	44	65	109
*Amblyomma ovale*	79	42	61	60	55	66	121
*Amblyomma sculptum*	63	92	81	74	17	138	155
*Amblyomma triste*	52	103	79	77	77	78	155
Total	368	458	423	403	328	498	826

We grouped pictures according to sex (fed and unfed female, and male), position (dorsal and ventral) and resolution (low, for pictures taken only with the mobile phone; high, for pictures taken with the mobile phone attached to the stereomicroscope and with the device described by Gurgel‐Gonçalves et al., 2017). An additional set combines all groups together (sex + position + resolution). The sex (female and male) and position (dorsal and ventral) groups include pictures at both resolutions. Only adult ticks in different feeding states (fed or unfed) were included. In total, 283 tick specimens were photographed. Most individuals were photographed four times, including dorsal and ventral images under stereomicroscopes with yellow or white lights, and dorsal and ventral images obtained with a cell phone camera. However, some samples presented a limited number of images, ranging from a single dorsal capture under the stereomicroscope or a single image of dorsal and ventral views. Of the total specimens, 38 were fed females, with the following distribution: *A. aureolatum n* = 8, *A. cajennense n* = 5, *A. dubitatum n* = 6, *A. ovale n* = 13 and *A. triste n* = 6.

### Machine learning

We used three deep learning algorithms: AlexNet (Krizhevsky et al., 2012), ResNet‐50 (He et al., 2016) and MobileNetV2 (Sandler et al., 2018). These algorithms and their variations show high performance in identifying mosquitoes, triatomines and ticks (Araújo et al., 2024; Luo et al., 2022; Miranda et al., 2024; Omodior et al., 2021). The implementation followed a systematic approach involving image pre‐processing, data splitting, data augmentation, network training, evaluation and application of Grad‐CAM (Luo et al., 2020).

AlexNet is a CNN pre‐trained on 1.2 million high‐resolution pictures, capable of classifying pictures into 1000 categories from the ImageNet database using 60 million parameters (Krizhevsky et al., 2012). ResNet‐50 has a higher depth, reaching up to 152 layers, resulting in good quality performance. The inclusion of ‘residual connections’ allows good training progress for deeper networks (He et al., 2016). MobileNetV2 is designed for applications on smartphones using specialised convolutional layers that are separable in depth and significantly reducing the number of parameters and computations (Sandler et al., 2018).

The images were loaded using the imageDatastore function, with the IncludeSubfolders option and LabelSource set to folder names (Luo et al., 2020). All images were resized to 227 × 227 pixels for AlexNet (Krizhevsky et al., 2012) and 224 × 224 pixels for MobileNetV2 (Sandler et al., 2018) and ResNet‐50 (He et al., 2016). The data was split using cross‐validation with K‐folds (k = 5), with indices generated by the crossvalind (‘Kfold’, imds.Labels, k) function (Stone, 1974). For data augmentation, we applied random rotation between −20 and 20 degrees, translation on the X and Y axis between −5 and 5 pixels, horizontal reflection and random scaling between 0.8 and 1.2 (Shorten & Khoshgoftaar, 2019). Training options included the sgdm optimizer, initial learning rate of 0.001, mini‐batch of 64, use of graphics processing unit (GPU) and 50 epochs (Krizhevsky et al., 2012). For MobileNetV2 and ResNet‐50, we used the adam optimiser and the same learning rate and training settings (He et al., 2016; Sandler et al., 2018).

Cross‐validation ensures that each image was used for both training and validation across different folds. This approach prevents overfitting and allows a more robust evaluation of model performance without relying on a fixed split (Stone, 1974). On the images classified by the trained models, we used the Grad‐CAM (Selvaraju et al., 2017) to highlight the regions of the images most relevant to the prediction of each image group. The Grad‐CAM visualisations allow the interpretation of the results and the identification of the regions that contributed to the model's decisions.

### Data analysis

The performance of CNNs to identify six tick species was assessed based on observations of confusion matrices and heat maps (to show the number of classification errors and hits), the mean and confidence interval (CI) of accuracy, sensitivity and specificity. We use the following equations to calculate CNNs performance metrics:

$$\text{Accuracy}=\frac{\mathrm{TP}+\mathrm{TN}}{\mathrm{TP}+\mathrm{TN}+\mathrm{FP}+\mathrm{FN}}$$

$$\text{Sensitivity}=\frac{\mathrm{TP}}{\mathrm{TP}+\mathrm{FN}}$$

$$\text{Specificity}=\frac{\mathrm{TN}}{\mathrm{TN}+\mathrm{FP}}$$

where TP = true positives, TN = true negatives, FP = false positives, FN = false negatives.

Frequencies and proportions with 95% ‘Wilson’ (CIs) (Newcombe, 1998) were calculated using the R package ‘Hmisc’ (Harrell & Dupont, 2023). Data was analysed using R 4.2.1 software (R Core Team, 2022). The code and the data used is available in Data S1 and Table S2, respectively. The results were visualised in graphs representing the overall accuracy of the models, with confidence intervals indicated. Colours were used to differentiate algorithms, allowing for a clear comparison. Sensitivity and specificity graphs were also generated by group, showing the relative performance of the models (Sokolova & Lapalme, 2009).

## RESULTS

### Performance of the algorithms for tick identification considering sex, position and picture resolution

**FIGURE 2:**
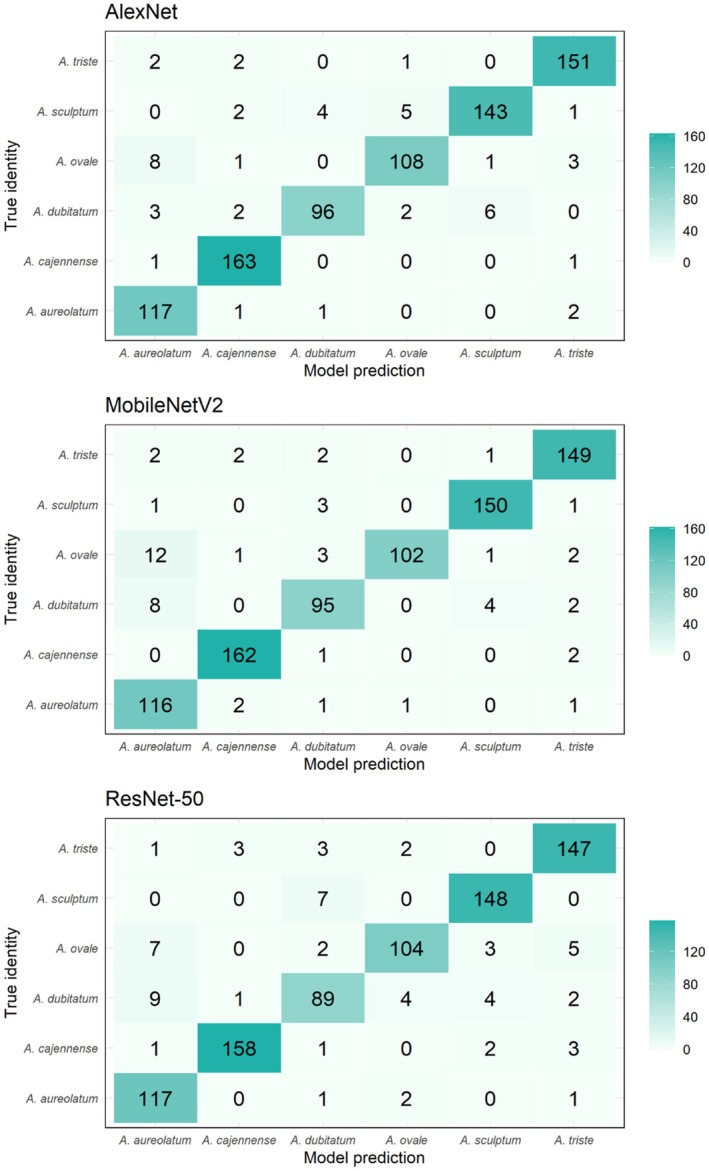
Confusion matrices with all images combined showing correct and incorrect identifications by AlexNet, MobileNetV2 and ResNet‐50 algorithms. The intensity of green represents the frequency of predictions: Darker shades indicate higher numbers, lighter shades indicate lower numbers. The main diagonal shows correct predictions, while off‐diagonal values represent confusion between classes.

**FIGURE 3:**
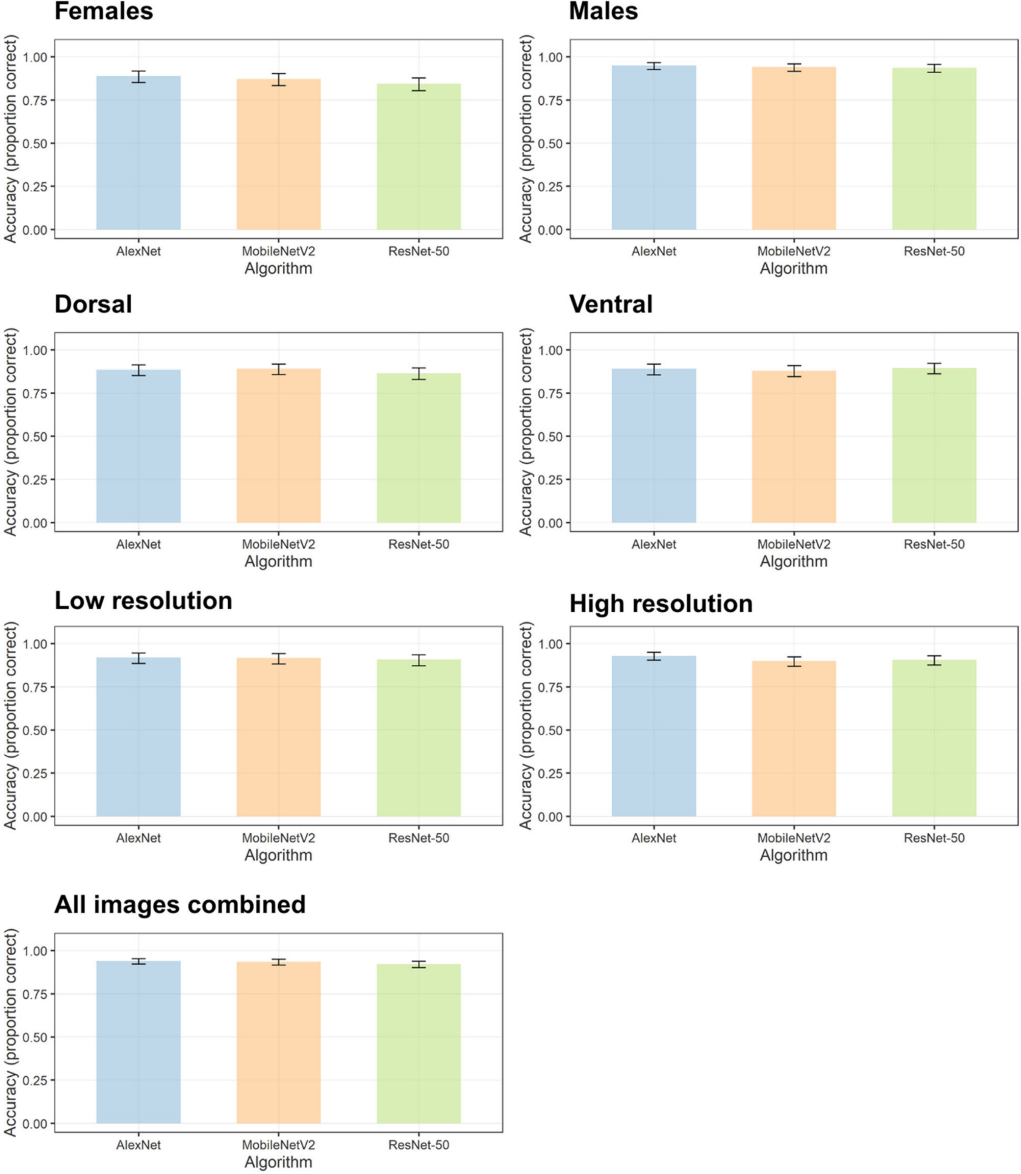
Accuracy obtained with AlexNet, MobileNetV2 and ResNet‐50, with 95% confidence intervals (bars) in the groups of images of females, males, dorsal, ventral, low resolution, high resolution and all images combined (sex+position+resolution).

We used a different number of pictures to evaluate the network's performance in terms of accuracy for sex (female and male), position (dorsal and ventral), resolution (low and high) and all pictures (Table 1, Table S2). Table S3 shows the confusion matrices for AlexNet, MobileNetV2 and ResNet‐50 models, and Figure 2 shows examples of confusion matrices considering all pictures. Figure 3 shows the accuracies obtained in each group of pictures. In general, the algorithms showed very similar accuracies regardless of the group of images used (Figure 3). In the image set considering the sex of the specimens, the AlexNet network performed similarly to the other algorithms tested, with an accuracy of 0.89 (95% CI 0.85–0.92) for females and 0.95 (95% CI 0.93–0.97) for males (Figure 3, Table S3). When considering the picture positions, AlexNet and MobileNetV2 showed similar performances in dorsal view identification, with accuracies of 0.89 (95% CI 0.85–0.91) and 0.89 (95% CI 0.86–0.92), respectively. For the ventral view, ResNet‐50 had the best performance, with an accuracy of 0.90 (95% CI 0.86–0.92) (Figure 3, Table S3). The group of images in the ventral position performed similarly to images in the dorsal position. When considering the different resolutions, AlexNet and MobileNetV2 performed similarly for low‐resolution images, with accuracies of 0.92 (95% CI 0.89–0.95) and 0.92 (95% CI 0.88–0.94), respectively. For high‐resolution images, AlexNet had a slightly higher increase in results, reaching 0.93 (95% CI 0.90–0.95), and this category showed slightly better results compared to low‐resolution images. Finally, when considering all images, both AlexNet and MobileNetV2 recorded the best performances, with an accuracy of 0.94 (95% CI 0.92–0.95) in both cases (Figure 3, Table S3). With regard to incorrect identifications made by the algorithms, the species with the highest rates were: *A. dubitatum* (most frequently identified as *A. aureolatum*, *A. cajennense* ou *A. sculptum*) and *A. sculptum* (most frequently identified as *A. dubitatum*). The other incorrect identifications are shown in Figure 2.

### Sensitivity and specificity

**FIGURE 4:**
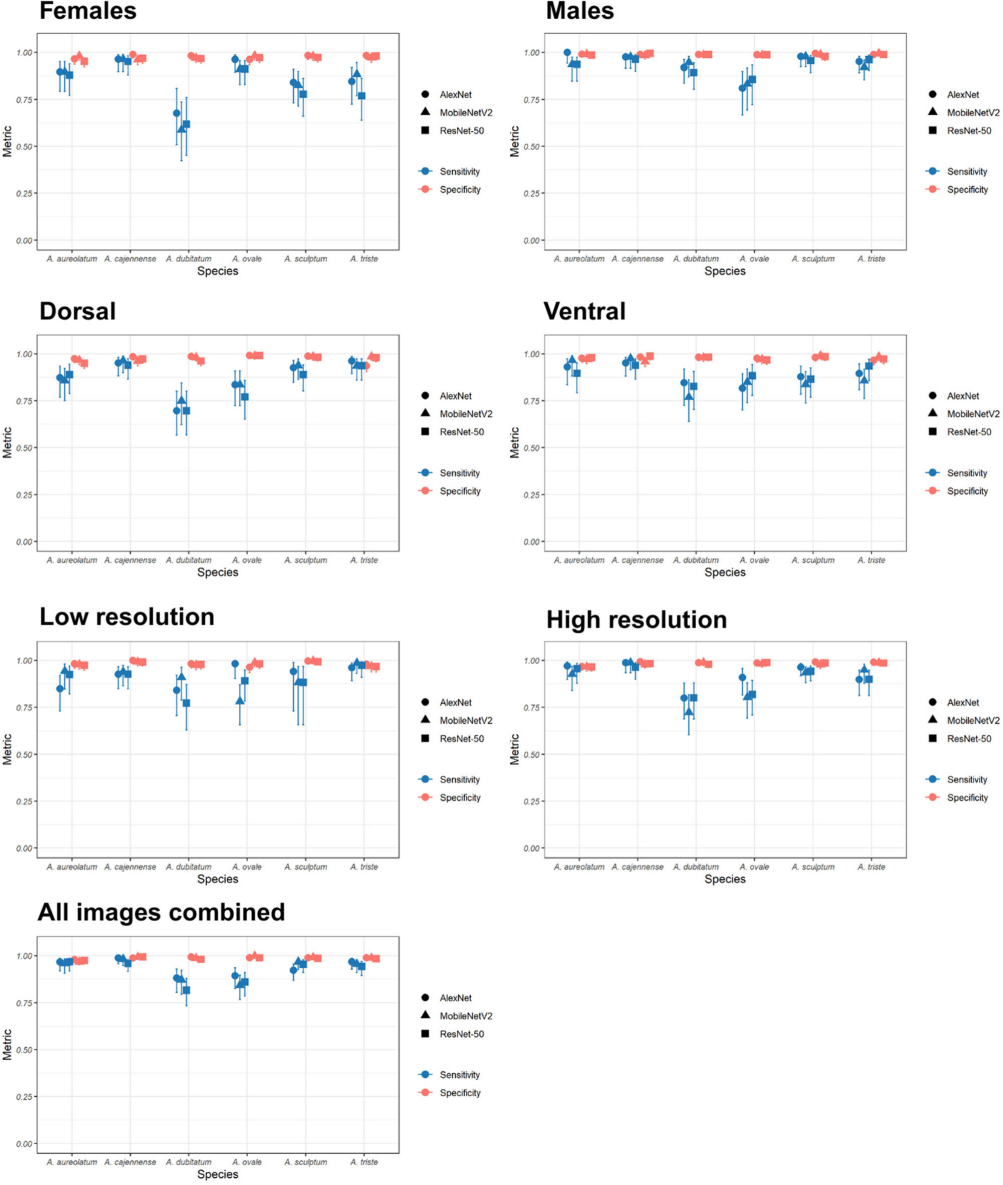
Sensitivity and specificity obtained with AlexNet, MobileNetV2 and ResNet‐50, with 95% confidence intervals (bars) in the groups of images of females, males, dorsal, ventral, low resolution, high resolution, all images combined (sex+position+resolution).

To assess the sensitivity, we used the same pictures described in Table 1 and the results of the confusion matrix (Figure 2), now taking into account the differentiation of species within each group. AlexNet showed sensitivity values ≥0.80 for all species in all image groupings, except for *A. dubitatum* in the female 0.68 (95% CI 0.51–0.81) and dorsal 0.70 (95% CI 0.57–0.80) groups. The MobileNetV2 network also displayed sensitivity values ≥0.80 in most of the image groups, except for *A. dubitatum* in the female 0.59 (95% CI 0.42–0.74), dorsal 0.75 (95% CI 0.62–0.84), ventral 0.77 (95% CI 0.64–0.86) and high resolution 0.72 (95% CI 0.60–0.82) groups, as well as *A. ovale* in the low‐resolution group 0.78 (95% CI 0.66–0.87). The ResNet‐50 network showed sensitivity values ≥0.80 for most species in all image groups, with the exception of *A. dubitatum* in the female 0.62 (95% CI 0.45–0.76), dorsal 0.70 (95% CI 0.57–0.80) and low‐resolution 0.77 (95% CI 0.63–0.87) image groups. In addition, *A. sculptum* and *A. triste* also showed values lower than 0.80 in the female group 0.78 (95% CI 0.66–0.86) and 0.77 (95% CI 0.64–0.86), respectively and *A. ovale* in dorsal 0.77 (95% CI 0.65–0.86). When considering the group with all the images of *A. sculptum*, the main vector of SF in the Americas, we observed that all the algorithms showed values >0.90 (AlexNet = 0.92, MobileNetV2: 0.97, ResNet‐50: 0.95). All the values mentioned above can be found in Figure 4 and Table S3.

**FIGURE 5:**
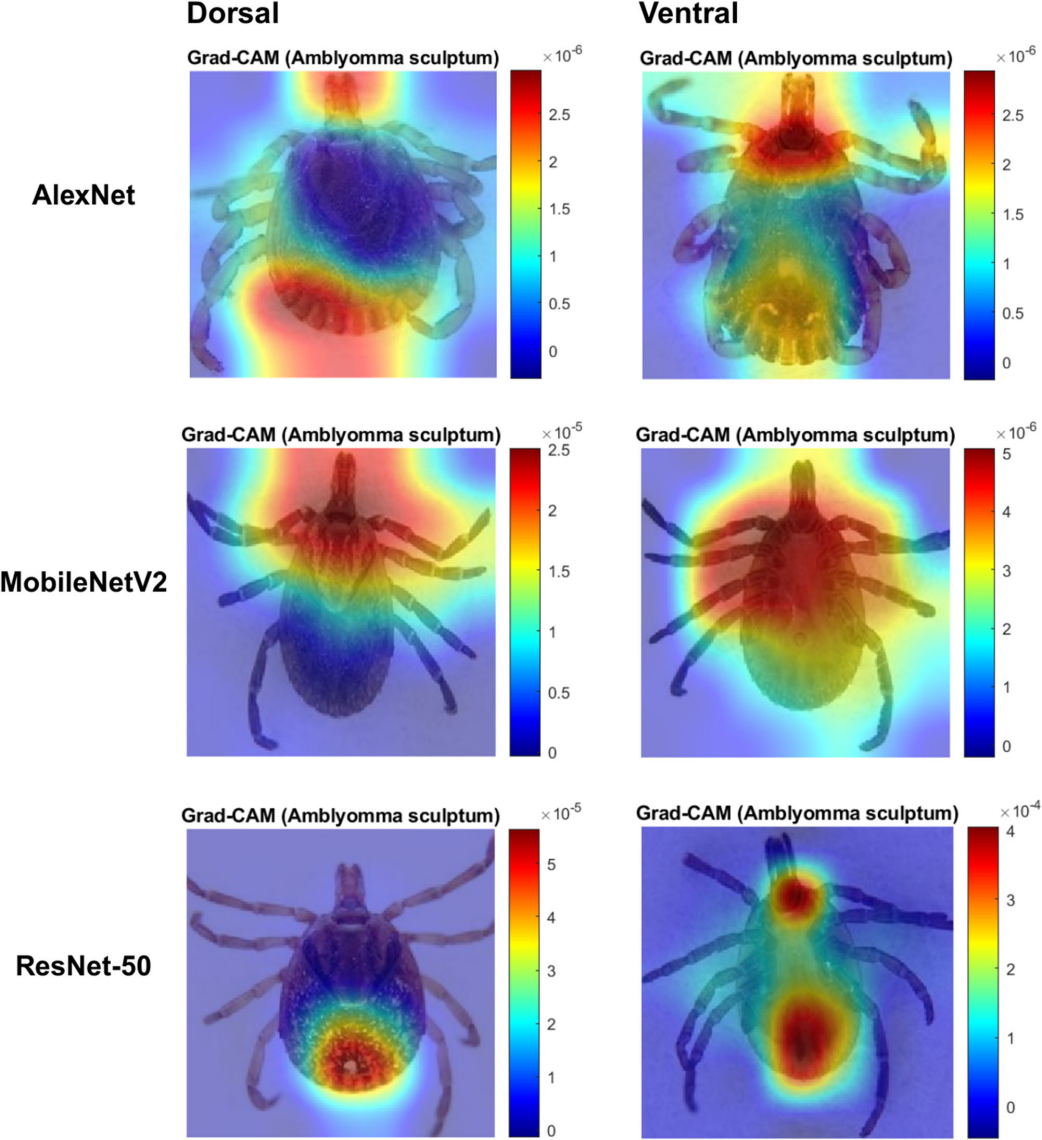
Grad‐CAM visualisations for *Amblyomma sculptum* (dorsal and ventral views) across three algorithms (AlexNet, MobileNetV2 and ResNet‐50). The images highlight the regions most important for classification. The intensity of the colouring indicates the contribution of each region in the image to the model prediction, with hotter areas representing greater relevance.

We calculated specificity using the confusion matrix (Figure 2) to assess the networks' ability to identify non‐target species. The specificity of all algorithms (>0.90) was high in identifying all tick species, regardless of the groups of images (Figure 4; Table S4). Grad‐CAM generated the heat maps shown in Figure 5 for *A. sculptum* according to the three algorithms. The most relevant areas for classifying species varied among the algorithms. AlexNet used both anterior and posterior areas of the tick body, whereas MobileNetV2 and ResNet‐50 focused on the anterior areas (Figure 5). Grad‐CAM results for all species and image groups are available (DOI: https://doi.org/10.6084/m9.figshare.28722818.v1).

## DISCUSSION

This study evaluated the performance of three CNNs in identifying SF vectors in South America. CNNs showed accuracies of ~90% for species identification and sensitivities of 59%–100% according to species, sex, position or image resolution. We also showed that the most relevant areas for classifying species varied according to algorithms.

Overall, CNNs have been successfully applied to identify pictures of arthropod vectors. AlexNet has been used to identify triatomines, mosquitoes and ticks with accuracies of 100% (Miranda et al., 2024), 90% (Araújo et al., 2024) and ~90% in our study, respectively. Studies in North America using automated tick identification have yielded similar results with other CNNs (Akbarian et al., 2021; Justen et al., 2021; Omodior et al., 2021), with accuracies of 89%–95% for different species and pictures. We showed that AlexNet, ResNet‐50 and MobileNetV2 CNNs allow reliable identification regardless of sex, position or image resolution. Justen et al. (2021) used a CNN called “TickIDNet” to identify three tick species: *A. americanum*, *Dermacentor variabilis* and *Ixodes scapularis*, considering the stage of development, sex and feeding status of the ticks. The accuracy for the three species was 87.8%. Omodior et al. (2021) compared the accuracy of a deep learning model and a surface CNN to identify four species, taking into account the stage of development. The CNN achieved 80% accuracy in species identification, compared to 75% for the ResNet‐50 model. Akbarian et al. (2021) used a CNN to discriminate *I. scapularis* from other tick species with 92% accuracy. Luo et al. (2022) compared the accuracy of five deep learning CNN models (VGG16, ResNet‐50, InceptionV3, DenseNet121 and MobileNetV2) for identifying three tick species, taking into account developmental stage, sex and feeding status. Their results indicate that all five CNNs performed exceptionally well to identify three tick species (98%–99% accuracy).

Several factors such as texture, colour and lighting conditions may have influenced the CNNs accuracies (Maitlo et al., 2024; Tian et al., 2023). Low‐resolution pictures had the lowest accuracy (89%), as expected (Justen et al., 2021). The position of the picture may also influence the results because some of the features of the ventral or dorsal region could be used more by CNNs for accurate identification. However, in general, our results did not show major differences between dorsal and ventral photographs for the identification of SF tick vectors. This suggests that ventral and dorsal pictures may display sufficiently detailed taxonomic features to accurately identify tick species. The female images obtained an accuracy of 91%, although slightly lower than that of male images (93%). This difference is probably associated with morphological changes resulting from the feeding process. Furthermore, the smaller size of the shield in females, which covers only a part of the idiosoma, may result in a reduced number of characters available for analysis. In contrast, in males, the larger shield offers more morphological characteristics, which possibly contributes to a higher accuracy in identification. The sensitivity results observed for *A. cajennense* and *A. sculptum* in this study (Figure 4, Table S4) were encouraging, given that both species belong to the same complex, share several morphological characteristics (Martins et al., 2016) and are commonly confused by taxonomists. These results demonstrate the potential of the neural network to discriminate among these species, which are highly morphologically similar but of different epidemiological importance. Sensitivity results (>90%) for *A. triste* suggest that the network could be a promising tool to monitor this species, which is a vector of *R. parkeri*, an agent of SF (Parola et al., 2013).

CNNs were able to identify female, ventral and high‐resolution images of *A. aureolatum* and *A. ovale* with a sensitivity rate of over 80%. This result is promising given the morphological similarity of these species, which often causes confusion even among taxonomists. The data demonstrate the potential of the neural network to discriminate against vector species of epidemiological importance, even with challenges of resolution, position and sex. The neural network misidentified *A. dubitatum* primarily in the low‐resolution image group. Although texture, colour and lighting of the images, as well as frequent confusion when differentiating this species from *A. sculptum*, are factors that can influence performance. These misclassifications are likely due to the limited number of images available for this species in the study. A larger number of images is crucial for training the network, as greater diversity and amount of data generally improve model performance. This limitation also applies to other species, which would probably perform better with a larger image base. Some species in this study showed high sensitivity, a key metric for evaluating machine learning in tick identification. The algorithm showed high specificity in identifying all tick species, with values above 90% (Figure 4; Table S4). CNNs correctly distinguish target species ticks from non‐target species, reducing false positives and inappropriate alarms in tick‐borne disease surveillance. Specificity, like sensitivity, is a fundamental metric for evaluating the performance of machine learning algorithms. Furthermore, CNNs have shown excellent results in other studies focusing on automated identification of arthropod pictures, confirming its effectiveness in similar contexts (Araújo et al., 2024; Luo et al., 2022; Miranda et al., 2024). In relation to the grad‐CAM data, the models do not seem to use the same areas of the body observed by taxonomists. For example, shield patterns are frequently used by taxonomists to identify species, and this characteristic was not frequently used by the CNN models, as revealed by Grad‐CAM. This can be explained by the networks' ability to take textures and lighting into account. Our database has pictures photographed with different lighting; in some pictures, the light from the magnifying glass was white, and in others, yellow. We know that light can influence the colours of tick images and consequently the recognition of the image by algorithms that learn colour patterns. This characteristic could be used to separate the species. However, we hypothesise a low effect of light variation in our study because the identification accuracies were relatively high for all species using the three evaluated algorithms. Future studies controlling this variable could measure what the relative effect of the type of light would be on the accuracy of tick identification. Moreover, new studies about automated identification of ticks should consider shortcut learning, when algorithms learn to differentiate images by differences in lighting or textures that do not necessarily represent the object being searched. Shortcut learning is a key roadblock towards trustworthy CNNs and could be associated with training data and optimisation (Geirhos et al., 2020).

This study demonstrates the feasibility of developing a tool for identifying ticks relevant to public health. It could be used by health surveillance services and communities at risk of tick exposure. Future research should focus on collecting a substantial number of pictures for each species, in addition to evaluating the performance of other automated identification networks. The aim is to identify an ideal network that could be incorporated into the development of a smartphone application. The future app for automated identification of ticks still requires a broad database and a universal system that allows the data to be received and the database to be run. We think this will be possible with the integration of networks of researchers interested in automated tick identification to discuss what this implementation will look like.

## CONCLUSIONS

Our findings indicate that AlexNet, ResNet‐50 and MobileNetV2 CNNs are reliable for identifying tick species transmitting SF bioagents, regardless of sex, position or image resolution. This performance lends further support to the concept of utilising CNNs for the automated identification of SF‐transmitting tick species in South America. Our database can facilitate the development of tick identification applications, which will prove invaluable in supporting public health surveillance and contributing to citizen science initiatives.

## AUTHOR CONTRIBUTIONS

**Isadora R. C. Gomes:** Conceptualization; data curation; formal analysis; investigation; methodology; validation; visualization; writing – original draft; writing – review and editing. **Vinícius L. Miranda:** Conceptualization; data curation; formal analysis; methodology; validation; visualization; writing – original draft; writing – review and editing. **José Fabrício C. Leal:** Conceptualization; data curation; formal analysis; methodology; validation; visualization; writing – original draft; writing – review and editing. **Igor P. Oliveira:** Conceptualization; data curation; writing – review and editing. **Paula J. Silva:** Data curation; writing – review and editing. **Karla Bitencourth:** Data curation; writing – review and editing; investigation. **Claudio M. Rodrigues:** Data curation; writing – review and editing. **Liege R. Siqueira:** Data curation; writing – review and editing. **Marcelo B. Labruna:** Data curation; writing – review and editing. **Gilberto S. Gazeta:** Data curation; writing – review and editing. **Marinete Amorim:** Data curation; writing – review and editing. **Rodrigo Gurgel‐Gonçalves:** Conceptualization; data curation; funding acquisition; project administration; supervision; writing – original draft; writing – review and editing.

## FUNDING INFORMATION

Isadora R. C. Gomes and José Fabrício C. Leal received specific funding from Coordenação de Aperfeiçoamento de Pessoal de Nível Superior https://www.capes.gov.br/ Award Number: finance code 001. Rodrigo Gurgel‐Gonçalves and Vinícius L. Miranda received funding from the National Council for Scientific and Technological Development (CNPq, Brazil, award number 314892/2021‐4 and 150659/2024‐5 respectively). The funding sources of this study had no role in the study design, data collection, data analysis, data interpretation, writing of the report or in the decision to submit the paper for publication.

## CONFLICT OF INTEREST STATEMENT

The authors declare no conflicts of interest.

## Supporting information


**Data S1.** MATLAB codes for image processing and training with AlexNet, MobileNetV2 and ResNet‐50.

**Table S1.** Number of tick specimens by sex (females and males) and collection site.

**Table S2.** Dataset used in the analysis showing the characteristics of each picture processed by *t*.

**Table S3.** Hits, accuracy and 95% confidence interval in the identification of tick species images predicted by AlexNet, MobileNetV2 and ResNet‐50.

**Table S4.** Sensitivity and specificity results observed in the study.

## Data Availability

The data that support the findings of this study are openly available in Figshare at https://doi.org/10.6084/m9.figshare.28711394 [Correction added on 11 July 2025 after first online publication: The Data Availability Statement has been updated.]
