# Supplementary material for: Automated identification of spotted‐fever tick vectors using convolutional neural networks
Source: Med Vet Entomol. 2025 Jul 4;39(4):829–41. doi: 10.1111/mve.12822 (PMC12586270; doi:10.1111/mve.12822)
Supplement: Supplementary file 1 — Data S1. MATLAB codes for image processing and training with AlexNet, MobileNetV2 and ResNet‐50. [file MVE-39-829-s002.docx]

**Supplementary information S1. MATLAB codes for image processing and training with AlexNet, MobileNetV2, and ResNet-50**

ALexNet

%% Image Resizing

clear all;

clc;

% Folder name with input files

input_folder = 'Entrada';

% Load and process images

imds = imageDatastore(input_folder, 'IncludeSubfolders', true, 'LabelSource', 'foldernames');

process(imds);

%% Training with AlexNet

% Creating an imageDatastore from the output folder

imds = imageDatastore('Saida', 'IncludeSubfolders', true, 'LabelSource', 'foldernames');

% Creating K-folds

k = 5; % Number of folds for cross-validation

indices = crossvalind('Kfold', imds.Labels, k);

% Preparing result storage

allPredictions = [];

allTrueLabels = [];

allScores = [];

allTestFiles = {}; % To store test files

for i = 1:k

% Creating train and test sets for the current fold

testIdx = (indices == i);

trainIdx = ~testIdx;

Train = subset(imds, trainIdx);

Test = subset(imds, testIdx);

% Store test files for later use

allTestFiles = [allTestFiles; Test.Files];

% Save train and test images

saveImages(Train, sprintf('fold%d/train', i));

saveImages(Test, sprintf('fold%d/test', i));

% Load AlexNet

net = alexnet;

layers = net.Layers;

% Modify the last fully connected layer to match the number of classes

fc = fullyConnectedLayer(length(unique(imds.Labels)), 'Name', 'new_fc');

layers(23) = fc;

% Replace classification layer with new classification layer

layers(end) = classificationLayer('Name', 'new_classification');

% Create data augmentation options (second version)

imageAugmenter = imageDataAugmenter( ...

'RandRotation', [-20, 20], ...

'RandXTranslation', [-5, 5], ...

'RandYTranslation', [-5, 5], ...

'RandXReflection', true, ...

'RandYReflection', false, ...

'RandXScale', [0.8, 1.2], ...

'RandYScale', [0.8, 1.2]);

% Create augmented image datastore with these options

augImdsTrain = augmentedImageDatastore([227, 227], Train, 'DataAugmentation', imageAugmenter);

% Training Options

Opts = trainingOptions('sgdm', ...

'InitialLearnRate', 0.001, ...

'MiniBatchSize', 64, ...

'ExecutionEnvironment', 'gpu', ...

'Plots', 'training-progress', ...

'MaxEpochs', 50, ...

'Verbose', true, ...

'VerboseFrequency', 50);

% Train the Network

[tickan_classifier, info] = trainNetwork(augImdsTrain, layers, Opts);

% Correct Predictions

labels_Test = Test.Labels;

[predictions, scores] = classify(tickan_classifier, Test);

% Storing predictions and true labels

allPredictions = [allPredictions; predictions];

allTrueLabels = [allTrueLabels; labels_Test];

allScores = [allScores; scores];

% Save the model for the current fold

save(sprintf('tickan_classifier_fold%d.mat', i), 'tickan_classifier');

% Save the results of the current fold to a spreadsheet

fold_results = table(Test.Files, labels_Test, predictions, 'VariableNames', {'ImageName', 'TrueClass', 'PredictedClass'});

writetable(fold_results, sprintf('classification_results_fold%d.xlsx', i));

end

% General Accuracy Analysis

percentage_correctly_classified_images = 100 * nnz(allPredictions == allTrueLabels) / numel(allPredictions);

% Confusion Matrix

[conf, names] = confusionmat(allTrueLabels, allPredictions);

figure;

h = heatmap(names, names, conf);

title('Confusion Matrix');

xlabel('Algorithm Prediction');

ylabel('True Identity');

% Save predictions and scores to a spreadsheet for ROC curve calculation

filename = 'ROC_data_crossvalidation.xlsx';

T = table(allTrueLabels, allPredictions, allScores, 'VariableNames', {'TrueLabels', 'PredictedLabels', 'Scores'});

writetable(T, filename);

% Save the final model

save('tickan_classifier.mat', 'tickan_classifier');

%% Applying Grad-CAM

% Define the network input size

inputSize = net.Layers(1).InputSize(1:2); % AlexNet expects [227, 227]

% Create folder to save test images

testImageFolder = 'imagens_teste';

if ~exist(testImageFolder, 'dir')

mkdir(testImageFolder);

end

% Create folder to save Grad-CAM results

gradCAMFolder = 'gradcam_results';

if ~exist(gradCAMFolder, 'dir')

mkdir(gradCAMFolder);

end

% Process test images and apply Grad-CAM

for i = 1:numel(allTestFiles)

% Read original image

imgOriginal = imread(allTestFiles{i});

% Resize the image to the network input size

imgResized = imresize(imgOriginal, inputSize);

% Save resized test image

[~, imageName, ext] = fileparts(allTestFiles{i});

imwrite(imgResized, fullfile(testImageFolder, [imageName, ext]));

% Classify the image

[YPred, scores] = classify(tickan_classifier, imgResized);

% Apply Grad-CAM for the predicted class

scoreMap = gradCAM(tickan_classifier, imgResized, YPred, 'ExecutionEnvironment', 'auto');

% Visualize and save the Grad-CAM map

figure;

imshow(imgResized, 'InitialMagnification', 150);

hold on;

imagesc(scoreMap, 'AlphaData', 0.5);

colormap jet;

colorbar;

title(sprintf('Grad-CAM (%s)', YPred));

% Save the image with Grad-CAM map

saveas(gcf, fullfile(gradCAMFolder, [imageName, '_gradcam.png']));

% Close figure

close(gcf);

end

disp('Processing completed.');

%% Function to process and resize images

function process(imds)

% Check if output folder exists

if exist('Saida', 'dir') == 7

rmdir('Saida', 's'); % If so, delete it

else

mkdir('Saida'); % If not, create it

end

% Initialize loop variables

files = imds.Files;

num_files = length(files);

% Initialize progress bar

progress_bar = waitbar(1/num_files, 'Processing images...');

% Loop to process and save

for k = 1:num_files

% Read image

img = readimage(imds, k);

% Resize image to 227x227

img = imresize(img, [227 227]);

% Check if class folder exists

output_folder_name = char(imds.Labels(k));

if exist(fullfile('Saida', output_folder_name), 'dir') ~= 7

mkdir(fullfile('Saida', output_folder_name)); % If not, create it

end

% Get original filename

[~, file_name, extension] = fileparts(char(imds.Files(k)));

% Convert image to uint8

img = im2uint8(img);

% Save image with original name

output_name = fullfile('Saida', output_folder_name, strcat(file_name, extension));

imwrite(img, output_name);

% Update progress bar

waitbar(k/num_files, progress_bar, 'Processing images...');

end

% Close progress bar

close(progress_bar);

end

%% Function to save train and test images

function saveImages(imds, output_folder)

% Create output folder if it doesn't exist

if ~exist(output_folder, 'dir')

mkdir(output_folder);

end

% Initialize loop variables

files = imds.Files;

num_files = length(files);

% Loop to save images

for k = 1:num_files

% Read image

img = readimage(imds, k);

% Check if class folder exists

output_folder_name = char(imds.Labels(k));

if exist(fullfile(output_folder, output_folder_name), 'dir') ~= 7

mkdir(fullfile(output_folder, output_folder_name)); % If not, create it

end

% Get original filename

[~, file_name, extension] = fileparts(char(imds.Files(k)));

% Convert image to uint8

img = im2uint8(img);

% Save image with original name

output_name = fullfile(output_folder, output_folder_name, strcat(file_name, extension));

imwrite(img, output_name);

end

end

MobileNetV2

%% Initial Settings

clc;

clear;

% Input folder path

input_folder = 'E:\Testes - Carrapatos\MobileNetV2\1 - Teste MobileNetV2\1-Fêmea\Entrada';

% Number of folds

k = 5;

% Input image size for MobileNetV2

inputSize = [224 224 3];

% Define data augmenter

imageAugmenter = imageDataAugmenter( ...

'RandRotation', [-20, 20], ... % Randomly rotate images between -20 and 20 degrees

'RandXTranslation', [-5, 5], ... % Translate images along the X axis

'RandYTranslation', [-5, 5], ... % Translate images along the Y axis

'RandXReflection', true, ... % Reflect images horizontally

'RandYReflection', false, ... % No vertical reflection

'RandXScale', [0.8, 1.2], ... % Randomly scale images along the X axis

'RandYScale', [0.8, 1.2]); % Randomly scale images along the Y axis

% Initialize results storage

allPredictions = [];

allTrueLabels = [];

allScores = [];

allTestFiles = {}; % To store test file names

for i = 1:k

% Define train and test folders for current fold

trainFolder = fullfile(input_folder, sprintf('fold%d', i), 'train');

testFolder = fullfile(input_folder, sprintf('fold%d', i), 'test');

% Create image datastores

imdsTrain = imageDatastore(trainFolder, ...

'IncludeSubfolders', true, ...

'LabelSource', 'foldernames');

imdsTest = imageDatastore(testFolder, ...

'IncludeSubfolders', true, ...

'LabelSource', 'foldernames');

% Create augmented training datastore

augmentedTrain = augmentedImageDatastore(inputSize(1:2), imdsTrain, ...

'DataAugmentation', imageAugmenter, ...

'ColorPreprocessing', 'gray2rgb');

% Create test datastore without augmentation

augmentedTest = augmentedImageDatastore(inputSize(1:2), imdsTest, ...

'ColorPreprocessing', 'gray2rgb');

% Try loading pretrained MobileNetV2

try

net = mobilenetv2();

catch ME

if strcmp(ME.identifier, 'MATLAB:undefinedVarOrClass')

fprintf('Support package is not installed.\n');

fprintf('Install the Deep Learning Toolbox Model for MobileNet-v2 Network via Add-On Explorer.\n');

fprintf('Alternatively, using MobileNetV2 without pretrained weights.\n');

net = mobilenetv2('Weights', 'none');

else

rethrow(ME);

end

end

% Adjust MobileNetV2 for dataset's number of classes

lgraph = layerGraph(net);

% Remove old classification layers, if they exist

layersToRemove = {'predictions', 'Logits', 'Logits_softmax', 'classification', 'ClassificationLayer_Logits'};

for j = 1:numel(layersToRemove)

if isLayerExist(lgraph, layersToRemove{j})

lgraph = removeLayers(lgraph, layersToRemove{j});

end

end

% Number of classes in the dataset

numClasses = numel(categories(imdsTrain.Labels));

% Add new fully connected and classification layers

newLayers = [

fullyConnectedLayer(numClasses, 'Name', 'fc', 'WeightLearnRateFactor', 10, 'BiasLearnRateFactor', 10)

softmaxLayer('Name', 'softmax')

classificationLayer('Name', 'classification')];

lgraph = addLayers(lgraph, newLayers);

% Connect new fully connected layer to the correct layer

lastLayerName = 'global_average_pooling2d_1'; % Update this name if needed

if ~isConnected(lgraph, lastLayerName, 'fc')

lgraph = connectLayers(lgraph, lastLayerName, 'fc');

end

if ~isConnected(lgraph, 'fc', 'softmax')

lgraph = connectLayers(lgraph, 'fc', 'softmax');

end

if ~isConnected(lgraph, 'softmax', 'classification')

lgraph = connectLayers(lgraph, 'softmax', 'classification');

end

% Training options

options = trainingOptions('adam', ...

'MiniBatchSize', 64, ...

'MaxEpochs', 50, ...

'InitialLearnRate', 0.001, ...

'Verbose', false, ...

'Plots', 'training-progress', ...

'ExecutionEnvironment', 'gpu');

% Train the network

try

netTransfer = trainNetwork(augmentedTrain, lgraph, options);

catch ME

fprintf('Error training network: %s\n', ME.message);

rethrow(ME);

end

% Evaluate on the test set

[predictedLabels, scores] = classify(netTransfer, augmentedTest);

accuracy = mean(predictedLabels == imdsTest.Labels);

% Display accuracy

fprintf('Test set accuracy (fold %d): %.2f%%\n', i, accuracy * 100);

% Store predictions, true labels, and scores

allPredictions = [allPredictions; predictedLabels];

allTrueLabels = [allTrueLabels; imdsTest.Labels];

allScores = [allScores; scores];

allTestFiles = [allTestFiles; imdsTest.Files];

% Save current fold results to Excel

foldResults = table(imdsTest.Files, imdsTest.Labels, predictedLabels, 'VariableNames', {'ImageName', 'TrueClass', 'PredictedClass'});

writetable(foldResults, sprintf('classification_results_fold%d.xlsx', i));

% Save current fold model

save(sprintf('model_fold%d.mat', i), 'netTransfer');

end

% Overall accuracy analysis

percent_correct = 100 * nnz(allPredictions == allTrueLabels) / numel(allPredictions);

% Confusion Matrix

[conf, names] = confusionmat(allTrueLabels, allPredictions);

figure;

h = heatmap(names, names, conf);

title('Confusion Matrix');

xlabel('Algorithm Prediction');

ylabel('True Identity');

% Save predictions and scores to Excel for ROC curve calculation

filename = 'ROC_data_crossvalidation.xlsx';

T = table(allTrueLabels, allPredictions, allScores, 'VariableNames', {'TrueLabels', 'PredictedLabels', 'Scores'});

writetable(T, filename);

% Save final model

save('tickmn_classifier_mobilenetv2.mat', 'netTransfer');

%% Apply Grad-CAM

% Create folder to save Grad-CAM results

gradCAMFolder = 'gradcam_results';

if ~exist(gradCAMFolder, 'dir')

mkdir(gradCAMFolder);

end

% Process test images and apply Grad-CAM

for i = 1:numel(allTestFiles)

% Read original image

imgOriginal = imread(allTestFiles{i});

% Resize image to network input size

imgResized = imresize(imgOriginal, inputSize(1:2));

% Save resized test image

[~, imageName, ext] = fileparts(allTestFiles{i});

imwrite(imgResized, fullfile(gradCAMFolder, [imageName, ext]));

% Classify the image

[YPred, scores] = classify(netTransfer, imgResized);

% Apply Grad-CAM for the predicted class

scoreMap = gradCAM(netTransfer, imgResized, YPred, 'ExecutionEnvironment', 'auto');

% Display and save Grad-CAM map

figure;

imshow(imgResized, 'InitialMagnification', 150);

hold on;

imagesc(scoreMap, 'AlphaData', 0.5);

colormap jet;

colorbar;

title(sprintf('Grad-CAM (%s)', YPred));

% Save Grad-CAM image

saveas(gcf, fullfile(gradCAMFolder, [imageName, '_gradcam.png']));

% Close figure

close(gcf);

end

disp('Processing complete.');

%% Helper Functions

% Helper function to check if a layer exists

function exists = isLayerExist(lgraph, layerName)

exists = any(strcmp({lgraph.Layers.Name}, layerName));

end

% Helper function to check if two layers are connected

function connected = isConnected(lgraph, layerName1, layerName2)

connections = lgraph.Connections;

connected = any(strcmp(connections.Source, layerName1) & strcmp(connections.Destination, layerName2));

end

ResNet-50

%% Initial Settings

clc;

clear;

% Input folder path

input_folder = 'E:\Testes - Carrapatos\ResNet-50\1 - Teste ResNet-50\1-Fêmea\Entrada';

% Number of folds

k = 5;

% Input image size for ResNet

inputSize = [224 224 3];

% Define the data augmenter

imageAugmenter = imageDataAugmenter( ...

'RandRotation', [-20, 20], ... % Randomly rotate images between -20 to 20 degrees

'RandXTranslation', [-5, 5], ... % Translate images along the X-axis

'RandYTranslation', [-5, 5], ... % Translate images along the Y-axis

'RandXReflection', true, ... % Reflect images horizontally

'RandYReflection', false, ... % No vertical reflection

'RandXScale', [0.8, 1.2], ... % Randomly scale images along the X-axis

'RandYScale', [0.8, 1.2]); % Randomly scale images along the Y-axis

% Prepare result storage

allPredictions = [];

allTrueLabels = [];

allScores = [];

allTestFiles = {}; % To store test file paths

for i = 1:k

% Define training and testing folder paths for the current fold

trainFolder = fullfile(input_folder, sprintf('fold%d', i), 'train');

testFolder = fullfile(input_folder, sprintf('fold%d', i), 'test');

% Create image datastores

imdsTrain = imageDatastore(trainFolder, ...

'IncludeSubfolders', true, ...

'LabelSource', 'foldernames');

imdsTest = imageDatastore(testFolder, ...

'IncludeSubfolders', true, ...

'LabelSource', 'foldernames');

% Create augmented training datastore

augmentedTrain = augmentedImageDatastore(inputSize(1:2), imdsTrain, ...

'DataAugmentation', imageAugmenter, ...

'ColorPreprocessing', 'gray2rgb');

% Create testing datastore without augmentation

augmentedTest = augmentedImageDatastore(inputSize(1:2), imdsTest, ...

'ColorPreprocessing', 'gray2rgb');

% Attempt to load pretrained ResNet

try

net = resnet50();

catch ME

if strcmp(ME.identifier, 'MATLAB:undefinedVarOrClass')

fprintf('Support package not installed.\n');

fprintf('Install the Deep Learning Toolbox Model for ResNet-50 Network via Add-On Explorer.\n');

fprintf('Alternatively, using ResNet50 without pretrained weights.\n');

net = resnet50('Weights', 'none');

else

rethrow(ME);

end

end

% Adapt ResNet for the number of dataset classes

lgraph = layerGraph(net);

% Remove existing classification layers, if present

layersToRemove = {'fc1000', 'fc1000_softmax', 'ClassificationLayer_fc1000'};

for j = 1:numel(layersToRemove)

if isLayerExist(lgraph, layersToRemove{j})

lgraph = removeLayers(lgraph, layersToRemove{j});

end

end

% Number of classes in the dataset

numClasses = numel(categories(imdsTrain.Labels));

% Add new fully connected and classification layers

newLayers = [

fullyConnectedLayer(numClasses, 'Name', 'fc', 'WeightLearnRateFactor', 10, 'BiasLearnRateFactor', 10)

softmaxLayer('Name', 'softmax')

classificationLayer('Name', 'classification')];

% Add new layers to the graph

lgraph = addLayers(lgraph, newLayers);

% Identify the name of the global pooling or final layer

lastLayerName = 'avg_pool'; % Update if needed

% Connect the new fully connected layer to the correct preceding layer

if ~isConnected(lgraph, lastLayerName, 'fc')

lgraph = connectLayers(lgraph, lastLayerName, 'fc');

end

if ~isConnected(lgraph, 'fc', 'softmax')

lgraph = connectLayers(lgraph, 'fc', 'softmax');

end

if ~isConnected(lgraph, 'softmax', 'classification')

lgraph = connectLayers(lgraph, 'softmax', 'classification');

end

% Training options

options = trainingOptions('adam', ...

'MiniBatchSize', 64, ...

'MaxEpochs', 50, ...

'InitialLearnRate', 0.001, ...

'Verbose', false, ...

'Plots', 'training-progress', ...

'ExecutionEnvironment', 'gpu');

% Train the network

try

netTransfer = trainNetwork(augmentedTrain, lgraph, options);

catch ME

fprintf('Error training the network: %s\n', ME.message);

rethrow(ME);

end

% Evaluate on the test set

[predictedLabels, scores] = classify(netTransfer, augmentedTest);

accuracy = mean(predictedLabels == imdsTest.Labels);

% Display accuracy

fprintf('Test set accuracy (fold %d): %.2f%%\n', i, accuracy * 100);

% Store predictions, true labels, and scores

allPredictions = [allPredictions; predictedLabels];

allTrueLabels = [allTrueLabels; imdsTest.Labels];

allScores = [allScores; scores];

allTestFiles = [allTestFiles; imdsTest.Files];

% Save current fold results to a spreadsheet

fold_results = table(imdsTest.Files, imdsTest.Labels, predictedLabels, 'VariableNames', {'ImageName', 'TrueClass', 'PredictedClass'});

writetable(fold_results, sprintf('classification_results_fold%d.xlsx', i));

% Save current fold model

save(sprintf('model_fold%d.mat', i), 'netTransfer');

end

% Overall Accuracy Analysis

correct_classification_percentage = 100 * nnz(allPredictions == allTrueLabels) / numel(allPredictions);

% Confusion Matrix

[conf, names] = confusionmat(allTrueLabels, allPredictions);

figure;

h = heatmap(names, names, conf);

title('Confusion Matrix');

xlabel('Algorithm Prediction');

ylabel('True Identity');

% Save predictions and scores to a spreadsheet for ROC analysis

filename = 'ROC_data_crossvalidation.xlsx';

T = table(allTrueLabels, allPredictions, allScores, 'VariableNames', {'TrueLabels', 'PredictedLabels', 'Scores'});

writetable(T, filename);

% Save the final model

save('tickrn_classifier.mat', 'netTransfer');

%% Applying Grad-CAM

% Create folder to save Grad-CAM results

gradCAMFolder = 'gradcam_results';

if ~exist(gradCAMFolder, 'dir')

mkdir(gradCAMFolder);

end

% Process test images and apply Grad-CAM

for i = 1:numel(allTestFiles)

% Read original image

imgOriginal = imread(allTestFiles{i});

% Resize image to network input size

imgResized = imresize(imgOriginal, inputSize(1:2));

% Save resized test image

[~, imageName, ext] = fileparts(allTestFiles{i});

imwrite(imgResized, fullfile(gradCAMFolder, [imageName, ext]));

% Classify the image

[YPred, scores] = classify(netTransfer, imgResized);

% Apply Grad-CAM to the predicted class

scoreMap = gradCAM(netTransfer, imgResized, YPred, 'ExecutionEnvironment', 'auto');

% Display and save Grad-CAM map

figure;

imshow(imgResized, 'InitialMagnification', 150);

hold on;

imagesc(scoreMap, 'AlphaData', 0.5);

colormap jet;

colorbar;

title(sprintf('Grad-CAM (%s)', YPred));

% Save Grad-CAM image

saveas(gcf, fullfile(gradCAMFolder, [imageName, '_gradcam.png']));

% Close the figure

close(gcf);

end

disp('Processing completed.');

%% Helper Functions

% Helper function to check if a layer exists

function exists = isLayerExist(lgraph, layerName)

exists = any(strcmp({lgraph.Layers.Name}, layerName));

end

% Helper function to check if connection between layers exists

function connected = isConnected(lgraph, layerName1, layerName2)

connections = lgraph.Connections;

connected = any(strcmp(connections.Source, layerName1) & strcmp(connections.Destination, layerName2));

end
