# Supplementary material for: Automated identification of spotted‐fever tick vectors using convolutional neural networks
Source: Med Vet Entomol. 2025 Jul 4;39(4):829–41. doi: 10.1111/mve.12822 (PMC12586270; doi:10.1111/mve.12822)
Supplement: Supplementary file 2 — Table S1. Number of tick specimens by sex (females and males) and collection site. [file MVE-39-829-s001.docx]

**Table S1.** Number of tick specimens by sex (females and males) and collection site.

| **Family** | **Genus** | **Species** | **Female** | **Male** | **Total** | **Geographical origin** | **Host** | **Collection date** | **Collection** | **Identification number** | **Photography location** |
| --- | --- | --- | --- | --- | --- | --- | --- | --- | --- | --- | --- |
| Ixodidae | *Amblyomma* | *A. aureolatum* | 12 | 12 | 24 | Piedade/São Paulo | *Puma concolor* | 5/6/2011 | CAVAISC | 1794 | Fiocruz |
|  |  | *A. aureolatum* | 3 | 5 | 8 | São Sebastião das Águas Claras/Minas Gerais | *Puma concolor* | 9/30/2021 | CNC | 4596 | USP |
|  |  | *A. aureolatum* | 1 | 1 | 2 | - | - | - | CAVAISC | - | Fiocruz |
|  |  | *A. aureolatum* | 3 | - | 3 | Planalto Catarinense/Santa Catarina | *Puma concolor* | 4/16/2008 | CAVAISC | 1146 | Fiocruz |
|  |  | *A. cajennense* | - | 1 | 1 | Belém/Pará | *Bubalus bubalis* | 10/1995 | CAVAISC | 231 | Fiocruz |
|  |  | *A. cajennense* | 5 | 2 | 7 | Marabá/Pará | *Tamandua tetradactyla* | 8/31/2010 | CAVAISC | 57 | Fiocruz |
|  |  | *A. cajennense* | - | 2 | 2 | Belém/Pará | *Tapirus terrestres* | 11/1/1994 | CAVAISC | 489 | Fiocruz |
|  |  | *A. cajennense* | 4 | 2 | 6 | Carajás/Pará | *Tapirus terrestres* | 7/8/2004 | CAVAISC | 868 | Fiocruz |
|  |  | *A. cajennense* | 1 | - | 1 | Santarém/Pará | *Homo sapiens* | 8/18/2007 | CAVAISC | 1244 | Fiocruz |
|  |  | *A. cajennense* | 1 | - | 1 | Carajás/Pará | *Cerdocyon thaus* | 9/15/2004 | CAVAISC | 876 | Fiocruz |
|  |  | *A. cajennense* | - | 1 | 1 | Carajás/Pará | *Tapirus terrestres* | 9/17/2009 | CAVAISC | 1167 | Fiocruz |
|  |  | *A. cajennense* | - | 1 | 1 | Carajás/Pará | *Tamandua tetradactyla* | 2/9/2009 | CAVAISC | 1495 | Fiocruz |
|  |  | *A. cajennense* | 10 | 12 | 22 | Lucas do Rio Verde/Mato Grosso | *Hydrochoerus hydrochaeris* | 10/2013 | CNC | 2544 | USP |
|  |  | *A. dubitatum* | 2 | 1 | 3 | Sorocaba/São Paulo | *Myocastor coypus* | 10/15/2000 | CAVAISC | 671 | Fiocruz |
|  |  | *A. dubitatum* | 6 | 18 | 24 | Sorocaba/São Paulo | *Hydrochoerus hydrochaeris* | 7/23/2006 | CAVAISC | 755 | Fiocruz |
|  |  | *A. dubitatum* | 1 | - | 1 | Brasília/Distrito Federal | Free life | - | UCB | - | UCB |
|  |  | *A. dubitatum* | 2 | 2 | 4 | Sorocaba/São Paulo | *Hydrochoerus hydrochaeris* | - | CAVAISC | - | Fiocruz |
|  |  | *A. dubitatum* | 1 | 1 | 2 | - | - | - | CAVAISC | - | Fiocruz |
|  |  | *A. dubitatum* | - | 2 | 2 | - | - | - | HVET-UnB | - | UnB |
|  |  | *A. ovale* | 1 | 1 | 2 | Paraupeba/Pará | *Cerdocyon thous* | 9/15/2004 | CAVAISC | 879 | Fiocruz |
|  |  | *A. ovale* | 5 | 2 | 7 | Cruzeiro do Sul/Acre | *Canis familiaris* | - | CAVAISC | 4205 | Fiocruz |
|  |  | *A. ovale* | 1 | 1 | 2 | Mangaratiba/Rio de Janeiro | *Canis familiaris* | 9/1/2006 | CAVAISC | 1363 | Fiocruz |
|  |  | *A. ovale* | 1 | 1 | 2 | - | - | - | CAVAISC | - | Fiocruz |
|  |  | *A. ovale* | 1 | 1 | 2 | - | - | - | HVET-UnB | - | UnB |
|  |  | *A. ovale* | 12 | 6 | 18 | Cubatão/São Paulo | *Procyon cancrivorus* | 6/17/2023 | CNC | 4782 | USP |
|  |  | *A. sculptum* | 3 | 6 | 9 | São Roque/São Paulo | *Mazama gouazoubira* | 10/10/2009 | CAVAISC | 1271 | Fiocruz |
|  |  | *A. sculptum* | 28 | 42 | 70 | Brasília/Distrito Federal | Free life | - | UCB | - | UCB |
|  |  | *A. sculptum* | 1 | 1 | 2 | - | *-* | - | CAVAISC | - | Fiocruz |
|  |  | *A. triste* | 3 | 17 | 20 | Pantanal/Mato Grosso do Sul | *Blastocerus dichotomus* | 8/9/1993 | CAVAISC | 73 | Fiocruz |
|  |  | *A. triste* | 7 | 4 | 11 | Paulicéia/São Paulo e Brasilândia/Mato Grosso do Sul | *Blastocerus dichotomus* | 04/2001 | CNC | 451 | USP |
|  |  | *A. triste* | 3 | 4 | 7 | Paulicéia/São Paulo | Free life | 1/24/2002 | CNC | 672 | USP |
|  |  | *A. triste* | 1 | 1 | 2 | - | - | - | CAVAISC | - | Fiocruz |
|  |  | *A. triste* | - | 1 | 1 | - | - | - | - | - |  |
|  |  | **Total** | **119** | **151** | **270** |  |  |  |  |  |  |

(-) no data
