# Supplementary material for: Automated identification of spotted‐fever tick vectors using convolutional neural networks
Source: Med Vet Entomol. 2025 Jul 4;39(4):829–41. doi: 10.1111/mve.12822 (PMC12586270; doi:10.1111/mve.12822)
Supplement: Supplementary file 3 — Table S2. Dataset used in the analysis showing the characteristics of each picture processed by t. [file MVE-39-829-s004.pdf]

Table S2. Dataset used in the analysis showing the characteritics of each picture processed by

Global order	Group	Algorithm	Picture	Fold	Class	Genus
1	Female	AlexNet	femea aureolatum (15).jpg	fold1	1	*Amblyomma*
2	Female	AlexNet	femea aureolatum (27).jpg	fold1	1	*Amblyomma*
3	Female	AlexNet	femea aureolatum (30).jpg	fold1	1	*Amblyomma*
4	Female	AlexNet	femea aureolatum (35).jpg	fold1	1	*Amblyomma*
5	Female	AlexNet	femea aureolatum (36).jpg	fold1	1	*Amblyomma*
6	Female	AlexNet	femea aureolatum (43).jpg	fold1	1	*Amblyomma*
7	Female	AlexNet	femea aureolatum (44).jpg	fold1	1	*Amblyomma*
8	Female	AlexNet	femea aureolatum (46).jpg	fold1	1	*Amblyomma*
9	Female	AlexNet	femea aureolatum (48).jpg	fold1	1	*Amblyomma*
10	Female	AlexNet	femea aureolatum (5).jpg	fold1	1	*Amblyomma*
11	Female	AlexNet	femea aureolatum (53).jpg	fold1	1	*Amblyomma*
12	Female	AlexNet	femea cajennense (11).jpg	fold1	2	*Amblyomma*
13	Female	AlexNet	femea cajennense (14).jpg	fold1	2	*Amblyomma*
14	Female	AlexNet	femea cajennense (17).jpg	fold1	2	*Amblyomma*
15	Female	AlexNet	femea cajennense (18).jpg	fold1	2	*Amblyomma*
16	Female	AlexNet	femea cajennense (25).jpg	fold1	2	*Amblyomma*
17	Female	AlexNet	femea cajennense (27).jpg	fold1	2	*Amblyomma*
18	Female	AlexNet	femea cajennense (36).jpg	fold1	2	*Amblyomma*
19	Female	AlexNet	femea cajennense (37).jpg	fold1	2	*Amblyomma*
20	Female	AlexNet	femea cajennense (39).jpg	fold1	2	*Amblyomma*
21	Female	AlexNet	femea cajennense (47).jpg	fold1	2	*Amblyomma*
22	Female	AlexNet	femea cajennense (55).jpg	fold1	2	*Amblyomma*
23	Female	AlexNet	femea cajennense (58).jpg	fold1	2	*Amblyomma*
24	Female	AlexNet	femea cajennense (60).jpg	fold1	2	*Amblyomma*
25	Female	AlexNet	femea cajennense (62).jpg	fold1	2	*Amblyomma*
26	Female	AlexNet	femea cajennense (68).jpg	fold1	2	*Amblyomma*
27	Female	AlexNet	femea cajennense (69).jpg	fold1	2	*Amblyomma*
28	Female	AlexNet	femea cajennense (76).jpg	fold1	2	*Amblyomma*
29	Female	AlexNet	femea dubitatum (11).jpg	fold1	3	*Amblyomma*
30	Female	AlexNet	femea dubitatum (2).jpg	fold1	3	*Amblyomma*
31	Female	AlexNet	femea dubitatum (23).jpg	fold1	3	*Amblyomma*
32	Female	AlexNet	femea dubitatum (26).jpg	fold1	3	*Amblyomma*
33	Female	AlexNet	femea dubitatum (28).jpg	fold1	3	*Amblyomma*
34	Female	AlexNet	femea dubitatum (6).jpg	fold1	3	*Amblyomma*
35	Female	AlexNet	femea dubitatum (8).jpg	fold1	3	*Amblyomma*
36	Female	AlexNet	femea ovale (15).jpg	fold1	4	*Amblyomma*
37	Female	AlexNet	femea ovale (17).jpg	fold1	4	*Amblyomma*
38	Female	AlexNet	femea ovale (20).jpg	fold1	4	*Amblyomma*
39	Female	AlexNet	femea ovale (22).jpg	fold1	4	*Amblyomma*
40	Female	AlexNet	femea ovale (25).jpg	fold1	4	*Amblyomma*
41	Female	AlexNet	femea ovale (28).jpg	fold1	4	*Amblyomma*
42	Female	AlexNet	femea ovale (33).jpg	fold1	4	*Amblyomma*
43	Female	AlexNet	femea ovale (35).jpg	fold1	4	*Amblyomma*
44	Female	AlexNet	femea ovale (44).jpg	fold1	4	*Amblyomma*
45	Female	AlexNet	femea ovale (51).jpg	fold1	4	*Amblyomma*
46	Female	AlexNet	femea ovale (53).jpg	fold1	4	*Amblyomma*
47	Female	AlexNet	femea ovale (59).jpg	fold1	4	*Amblyomma*
48	Female	AlexNet	femea ovale (61).jpg	fold1	4	*Amblyomma*
49	Female	AlexNet	femea ovale (62).jpg	fold1	4	*Amblyomma*
50	Female	AlexNet	femea ovale (74).jpg	fold1	4	*Amblyomma*
51	Female	AlexNet	femea ovale (75).jpg	fold1	4	*Amblyomma*
52	Female	AlexNet	femea sculptum (1).jpg	fold1	5	*Amblyomma*
53	Female	AlexNet	femea sculptum (11).JPG	fold1	5	*Amblyomma*
54	Female	AlexNet	femea sculptum (13).JPG	fold1	5	*Amblyomma*
55	Female	AlexNet	femea sculptum (14).JPG	fold1	5	*Amblyomma*
56	Female	AlexNet	femea sculptum (22).JPG	fold1	5	*Amblyomma*
57	Female	AlexNet	femea sculptum (39).JPG	fold1	5	*Amblyomma*
58	Female	AlexNet	femea sculptum (58).jpg	fold1	5	*Amblyomma*
59	Female	AlexNet	femea sculptum (6).jpg	fold1	5	*Amblyomma*
60	Female	AlexNet	femea sculptum (60).jpg	fold1	5	*Amblyomma*
61	Female	AlexNet	femea sculptum (61).jpg	fold1	5	*Amblyomma*
62	Female	AlexNet	femea sculptum (62).jpg	fold1	5	*Amblyomma*
63	Female	AlexNet	femea sculptum (8).jpg	fold1	5	*Amblyomma*
64	Female	AlexNet	femea sculptum (9).JPG	fold1	5	*Amblyomma*
65	Female	AlexNet	femea triste (12).jpg	fold1	6	*Amblyomma*

66	Female	AlexNet	femea triste (13).jpg	fold1	6	*Amblyomma*
67	Female	AlexNet	femea triste (14).jpg	fold1	6	*Amblyomma*
68	Female	AlexNet	femea triste (23).jpg	fold1	6	*Amblyomma*
69	Female	AlexNet	femea triste (26).jpg	fold1	6	*Amblyomma*
70	Female	AlexNet	femea triste (32).jpg	fold1	6	*Amblyomma*
71	Female	AlexNet	femea triste (35).jpg	fold1	6	*Amblyomma*
72	Female	AlexNet	femea triste (38).jpg	fold1	6	*Amblyomma*
73	Female	AlexNet	femea triste (45).jpg	fold1	6	*Amblyomma*
74	Female	AlexNet	femea triste (46).jpg	fold1	6	*Amblyomma*
75	Female	AlexNet	femea triste (47).jpg	fold1	6	*Amblyomma*
76	Female	AlexNet	femea aureolatum (1).jpg	fold2	1	*Amblyomma*
77	Female	AlexNet	femea aureolatum (10).jpg	fold2	1	*Amblyomma*
78	Female	AlexNet	femea aureolatum (12).jpg	fold2	1	*Amblyomma*
79	Female	AlexNet	femea aureolatum (16).jpg	fold2	1	*Amblyomma*
80	Female	AlexNet	femea aureolatum (22).jpg	fold2	1	*Amblyomma*
81	Female	AlexNet	femea aureolatum (28).jpg	fold2	1	*Amblyomma*
82	Female	AlexNet	femea aureolatum (31).jpg	fold2	1	*Amblyomma*
83	Female	AlexNet	femea aureolatum (37).jpg	fold2	1	*Amblyomma*
84	Female	AlexNet	femea aureolatum (39).jpg	fold2	1	*Amblyomma*
85	Female	AlexNet	femea aureolatum (50).jpg	fold2	1	*Amblyomma*
86	Female	AlexNet	femea aureolatum (52).jpg	fold2	1	*Amblyomma*
87	Female	AlexNet	femea aureolatum (8).jpg	fold2	1	*Amblyomma*
88	Female	AlexNet	femea cajennense (10).jpg	fold2	2	*Amblyomma*
89	Female	AlexNet	femea cajennense (12).jpg	fold2	2	*Amblyomma*
90	Female	AlexNet	femea cajennense (19).jpg	fold2	2	*Amblyomma*
91	Female	AlexNet	femea cajennense (23).jpg	fold2	2	*Amblyomma*
92	Female	AlexNet	femea cajennense (3).jpg	fold2	2	*Amblyomma*
93	Female	AlexNet	femea cajennense (34).jpg	fold2	2	*Amblyomma*
94	Female	AlexNet	femea cajennense (35).jpg	fold2	2	*Amblyomma*
95	Female	AlexNet	femea cajennense (53).jpg	fold2	2	*Amblyomma*
96	Female	AlexNet	femea cajennense (54).jpg	fold2	2	*Amblyomma*
97	Female	AlexNet	femea cajennense (57).jpg	fold2	2	*Amblyomma*
98	Female	AlexNet	femea cajennense (6).jpg	fold2	2	*Amblyomma*
99	Female	AlexNet	femea cajennense (63).jpg	fold2	2	*Amblyomma*
100	Female	AlexNet	femea cajennense (66).jpg	fold2	2	*Amblyomma*
101	Female	AlexNet	femea cajennense (67).jpg	fold2	2	*Amblyomma*
102	Female	AlexNet	femea cajennense (7).jpg	fold2	2	*Amblyomma*
103	Female	AlexNet	femea cajennense (77).jpg	fold2	2	*Amblyomma*
104	Female	AlexNet	femea cajennense (9).jpg	fold2	2	*Amblyomma*
105	Female	AlexNet	femea dubitatum (12).jpg	fold2	3	*Amblyomma*
106	Female	AlexNet	femea dubitatum (13).jpg	fold2	3	*Amblyomma*
107	Female	AlexNet	femea dubitatum (19).jpg	fold2	3	*Amblyomma*
108	Female	AlexNet	femea dubitatum (21).jpg	fold2	3	*Amblyomma*
109	Female	AlexNet	femea dubitatum (24).jpg	fold2	3	*Amblyomma*
110	Female	AlexNet	femea dubitatum (3).jpg	fold2	3	*Amblyomma*
111	Female	AlexNet	femea dubitatum (33).jpg	fold2	3	*Amblyomma*
112	Female	AlexNet	femea ovale (1).jpg	fold2	4	*Amblyomma*
113	Female	AlexNet	femea ovale (10).jpg	fold2	4	*Amblyomma*
114	Female	AlexNet	femea ovale (14).jpg	fold2	4	*Amblyomma*
115	Female	AlexNet	femea ovale (18).jpg	fold2	4	*Amblyomma*
116	Female	AlexNet	femea ovale (29).jpg	fold2	4	*Amblyomma*
117	Female	AlexNet	femea ovale (32).jpg	fold2	4	*Amblyomma*
118	Female	AlexNet	femea ovale (34).jpg	fold2	4	*Amblyomma*
119	Female	AlexNet	femea ovale (36).jpg	fold2	4	*Amblyomma*
120	Female	AlexNet	femea ovale (39).jpg	fold2	4	*Amblyomma*
121	Female	AlexNet	femea ovale (50).jpg	fold2	4	*Amblyomma*
122	Female	AlexNet	femea ovale (55).jpg	fold2	4	*Amblyomma*
123	Female	AlexNet	femea ovale (56).jpg	fold2	4	*Amblyomma*
124	Female	AlexNet	femea ovale (6).jpg	fold2	4	*Amblyomma*
125	Female	AlexNet	femea ovale (64).jpg	fold2	4	*Amblyomma*
126	Female	AlexNet	femea ovale (65).jpg	fold2	4	*Amblyomma*
127	Female	AlexNet	femea ovale (66).jpg	fold2	4	*Amblyomma*
128	Female	AlexNet	femea sculptum (16).JPG	fold2	5	*Amblyomma*
129	Female	AlexNet	femea sculptum (24).JPG	fold2	5	*Amblyomma*
130	Female	AlexNet	femea sculptum (25).JPG	fold2	5	*Amblyomma*
131	Female	AlexNet	femea sculptum (26).JPG	fold2	5	*Amblyomma*
132	Female	AlexNet	femea sculptum (27).JPG	fold2	5	*Amblyomma*

133	Female	AlexNet	femea sculptum (30).JPG	fold2	5 *Amblyomma*
134	Female	AlexNet	femea sculptum (34).JPG	fold2	5 *Amblyomma*
135	Female	AlexNet	femea sculptum (36).JPG	fold2	5 *Amblyomma*
136	Female	AlexNet	femea sculptum (40).JPG	fold2	5 *Amblyomma*
137	Female	AlexNet	femea sculptum (42).JPG	fold2	5 *Amblyomma*
138	Female	AlexNet	femea sculptum (44).JPG	fold2	5 *Amblyomma*
139	Female	AlexNet	femea sculptum (46).JPG	fold2	5 *Amblyomma*
140	Female	AlexNet	femea triste (19).jpg	fold2	6 *Amblyomma*
141	Female	AlexNet	femea triste (22).jpg	fold2	6 *Amblyomma*
142	Female	AlexNet	femea triste (24).jpg	fold2	6 *Amblyomma*
143	Female	AlexNet	femea triste (27).jpg	fold2	6 *Amblyomma*
144	Female	AlexNet	femea triste (30).jpg	fold2	6 *Amblyomma*
145	Female	AlexNet	femea triste (31).jpg	fold2	6 *Amblyomma*
146	Female	AlexNet	femea triste (36).jpg	fold2	6 *Amblyomma*
147	Female	AlexNet	femea triste (42).jpg	fold2	6 *Amblyomma*
148	Female	AlexNet	femea triste (43).jpg	fold2	6 *Amblyomma*
149	Female	AlexNet	femea triste (5).jpg	fold2	6 *Amblyomma*
150	Female	AlexNet	femea aureolatum (11).jpg	fold3	1 *Amblyomma*
151	Female	AlexNet	femea aureolatum (19).jpg	fold3	1 *Amblyomma*
152	Female	AlexNet	femea aureolatum (2).jpg	fold3	1 *Amblyomma*
153	Female	AlexNet	femea aureolatum (21).jpg	fold3	1 *Amblyomma*
154	Female	AlexNet	femea aureolatum (32).jpg	fold3	1 *Amblyomma*
155	Female	AlexNet	femea aureolatum (34).jpg	fold3	1 *Amblyomma*
156	Female	AlexNet	femea aureolatum (38).jpg	fold3	1 *Amblyomma*
157	Female	AlexNet	femea aureolatum (4).jpg	fold3	1 *Amblyomma*
158	Female	AlexNet	femea aureolatum (47).jpg	fold3	1 *Amblyomma*
159	Female	AlexNet	femea aureolatum (51).jpg	fold3	1 *Amblyomma*
160	Female	AlexNet	femea aureolatum (55).jpg	fold3	1 *Amblyomma*
161	Female	AlexNet	femea cajennense (1).jpg	fold3	2 *Amblyomma*
162	Female	AlexNet	femea cajennense (22).jpg	fold3	2 *Amblyomma*
163	Female	AlexNet	femea cajennense (26).jpg	fold3	2 *Amblyomma*
164	Female	AlexNet	femea cajennense (28).jpg	fold3	2 *Amblyomma*
165	Female	AlexNet	femea cajennense (32).jpg	fold3	2 *Amblyomma*
166	Female	AlexNet	femea cajennense (38).jpg	fold3	2 *Amblyomma*
167	Female	AlexNet	femea cajennense (41).jpg	fold3	2 *Amblyomma*
168	Female	AlexNet	femea cajennense (50).jpg	fold3	2 *Amblyomma*
169	Female	AlexNet	femea cajennense (51).jpg	fold3	2 *Amblyomma*
170	Female	AlexNet	femea cajennense (71).jpg	fold3	2 *Amblyomma*
171	Female	AlexNet	femea cajennense (72).jpg	fold3	2 *Amblyomma*
172	Female	AlexNet	femea cajennense (73).jpg	fold3	2 *Amblyomma*
173	Female	AlexNet	femea cajennense (78).jpg	fold3	2 *Amblyomma*
174	Female	AlexNet	femea cajennense (79).jpg	fold3	2 *Amblyomma*
175	Female	AlexNet	femea cajennense (8).jpg	fold3	2 *Amblyomma*
176	Female	AlexNet	femea cajennense (80).jpg	fold3	2 *Amblyomma*
177	Female	AlexNet	femea dubitatum (16).jpg	fold3	3 *Amblyomma*
178	Female	AlexNet	femea dubitatum (18).jpg	fold3	3 *Amblyomma*
179	Female	AlexNet	femea dubitatum (25).jpg	fold3	3 *Amblyomma*
180	Female	AlexNet	femea dubitatum (27).jpg	fold3	3 *Amblyomma*
181	Female	AlexNet	femea dubitatum (29).jpg	fold3	3 *Amblyomma*
182	Female	AlexNet	femea dubitatum (32).jpg	fold3	3 *Amblyomma*
183	Female	AlexNet	femea dubitatum (34).jpg	fold3	3 *Amblyomma*
184	Female	AlexNet	femea ovale (11).jpg	fold3	4 *Amblyomma*
185	Female	AlexNet	femea ovale (19).jpg	fold3	4 *Amblyomma*
186	Female	AlexNet	femea ovale (26).jpg	fold3	4 *Amblyomma*
187	Female	AlexNet	femea ovale (3).JPG	fold3	4 *Amblyomma*
188	Female	AlexNet	femea ovale (30).jpg	fold3	4 *Amblyomma*
189	Female	AlexNet	femea ovale (31).jpg	fold3	4 *Amblyomma*
190	Female	AlexNet	femea ovale (37).jpg	fold3	4 *Amblyomma*
191	Female	AlexNet	femea ovale (38).jpg	fold3	4 *Amblyomma*
192	Female	AlexNet	femea ovale (45).jpg	fold3	4 *Amblyomma*
193	Female	AlexNet	femea ovale (49).jpg	fold3	4 *Amblyomma*
194	Female	AlexNet	femea ovale (5).jpg	fold3	4 *Amblyomma*
195	Female	AlexNet	femea ovale (54).jpg	fold3	4 *Amblyomma*
196	Female	AlexNet	femea ovale (67).jpg	fold3	4 *Amblyomma*
197	Female	AlexNet	femea ovale (71).jpg	fold3	4 *Amblyomma*
198	Female	AlexNet	femea ovale (78).jpg	fold3	4 *Amblyomma*
199	Female	AlexNet	femea sculptum (10).JPG	fold3	5 *Amblyomma*

200	Female	AlexNet	femea sculptum (17).JPG	fold3	5	*Amblyomma*
201	Female	AlexNet	femea sculptum (18).JPG	fold3	5	*Amblyomma*
202	Female	AlexNet	femea sculptum (21).JPG	fold3	5	*Amblyomma*
203	Female	AlexNet	femea sculptum (28).JPG	fold3	5	*Amblyomma*
204	Female	AlexNet	femea sculptum (43).JPG	fold3	5	*Amblyomma*
205	Female	AlexNet	femea sculptum (45).JPG	fold3	5	*Amblyomma*
206	Female	AlexNet	femea sculptum (47).JPG	fold3	5	*Amblyomma*
207	Female	AlexNet	femea sculptum (49).JPG	fold3	5	*Amblyomma*
208	Female	AlexNet	femea sculptum (53).JPG	fold3	5	*Amblyomma*
209	Female	AlexNet	femea sculptum (54).JPG	fold3	5	*Amblyomma*
210	Female	AlexNet	femea sculptum (59).jpg	fold3	5	*Amblyomma*
211	Female	AlexNet	femea sculptum (7).jpg	fold3	5	*Amblyomma*
212	Female	AlexNet	femea triste (1).jpg	fold3	6	*Amblyomma*
213	Female	AlexNet	femea triste (10).jpg	fold3	6	*Amblyomma*
214	Female	AlexNet	femea triste (11).jpg	fold3	6	*Amblyomma*
215	Female	AlexNet	femea triste (2).jpg	fold3	6	*Amblyomma*
216	Female	AlexNet	femea triste (37).jpg	fold3	6	*Amblyomma*
217	Female	AlexNet	femea triste (4).jpg	fold3	6	*Amblyomma*
218	Female	AlexNet	femea triste (44).jpg	fold3	6	*Amblyomma*
219	Female	AlexNet	femea triste (50).jpg	fold3	6	*Amblyomma*
220	Female	AlexNet	femea triste (7).jpg	fold3	6	*Amblyomma*
221	Female	AlexNet	femea triste (9).jpg	fold3	6	*Amblyomma*
222	Female	AlexNet	femea aureolatum (14).jpg	fold4	1	*Amblyomma*
223	Female	AlexNet	femea aureolatum (17).jpg	fold4	1	*Amblyomma*
224	Female	AlexNet	femea aureolatum (20).jpg	fold4	1	*Amblyomma*
225	Female	AlexNet	femea aureolatum (29).jpg	fold4	1	*Amblyomma*
226	Female	AlexNet	femea aureolatum (3).jpg	fold4	1	*Amblyomma*
227	Female	AlexNet	femea aureolatum (40).jpg	fold4	1	*Amblyomma*
228	Female	AlexNet	femea aureolatum (42).jpg	fold4	1	*Amblyomma*
229	Female	AlexNet	femea aureolatum (49).jpg	fold4	1	*Amblyomma*
230	Female	AlexNet	femea aureolatum (54).jpg	fold4	1	*Amblyomma*
231	Female	AlexNet	femea aureolatum (6).jpg	fold4	1	*Amblyomma*
232	Female	AlexNet	femea aureolatum (7).jpg	fold4	1	*Amblyomma*
233	Female	AlexNet	femea aureolatum (9).jpg	fold4	1	*Amblyomma*
234	Female	AlexNet	femea cajennense (16).jpg	fold4	2	*Amblyomma*
235	Female	AlexNet	femea cajennense (20).jpg	fold4	2	*Amblyomma*
236	Female	AlexNet	femea cajennense (24).jpg	fold4	2	*Amblyomma*
237	Female	AlexNet	femea cajennense (30).jpg	fold4	2	*Amblyomma*
238	Female	AlexNet	femea cajennense (4).jpg	fold4	2	*Amblyomma*
239	Female	AlexNet	femea cajennense (43).jpg	fold4	2	*Amblyomma*
240	Female	AlexNet	femea cajennense (44).jpg	fold4	2	*Amblyomma*
241	Female	AlexNet	femea cajennense (45).jpg	fold4	2	*Amblyomma*
242	Female	AlexNet	femea cajennense (46).jpg	fold4	2	*Amblyomma*
243	Female	AlexNet	femea cajennense (52).jpg	fold4	2	*Amblyomma*
244	Female	AlexNet	femea cajennense (56).jpg	fold4	2	*Amblyomma*
245	Female	AlexNet	femea cajennense (61).jpg	fold4	2	*Amblyomma*
246	Female	AlexNet	femea cajennense (70).jpg	fold4	2	*Amblyomma*
247	Female	AlexNet	femea cajennense (74).jpg	fold4	2	*Amblyomma*
248	Female	AlexNet	femea cajennense (75).jpg	fold4	2	*Amblyomma*
249	Female	AlexNet	femea cajennense (81).jpg	fold4	2	*Amblyomma*
250	Female	AlexNet	femea dubitatum (10).jpg	fold4	3	*Amblyomma*
251	Female	AlexNet	femea dubitatum (15).jpg	fold4	3	*Amblyomma*
252	Female	AlexNet	femea dubitatum (17).jpg	fold4	3	*Amblyomma*
253	Female	AlexNet	femea dubitatum (22).jpg	fold4	3	*Amblyomma*
254	Female	AlexNet	femea dubitatum (30).jpg	fold4	3	*Amblyomma*
255	Female	AlexNet	femea dubitatum (7).jpg	fold4	3	*Amblyomma*
256	Female	AlexNet	femea ovale (2).jpg	fold4	4	*Amblyomma*
257	Female	AlexNet	femea ovale (23).jpg	fold4	4	*Amblyomma*
258	Female	AlexNet	femea ovale (24).jpg	fold4	4	*Amblyomma*
259	Female	AlexNet	femea ovale (4).JPG	fold4	4	*Amblyomma*
260	Female	AlexNet	femea ovale (40).jpg	fold4	4	*Amblyomma*
261	Female	AlexNet	femea ovale (46).jpg	fold4	4	*Amblyomma*
262	Female	AlexNet	femea ovale (48).jpg	fold4	4	*Amblyomma*
263	Female	AlexNet	femea ovale (52).jpg	fold4	4	*Amblyomma*
264	Female	AlexNet	femea ovale (57).jpg	fold4	4	*Amblyomma*
265	Female	AlexNet	femea ovale (58).jpg	fold4	4	*Amblyomma*
266	Female	AlexNet	femea ovale (7).jpg	fold4	4	*Amblyomma*

267	Female	AlexNet	femea ovale (70).jpg	fold4	4	*Amblyomma*
268	Female	AlexNet	femea ovale (73).jpg	fold4	4	*Amblyomma*
269	Female	AlexNet	femea ovale (76).jpg	fold4	4	*Amblyomma*
270	Female	AlexNet	femea ovale (77).jpg	fold4	4	*Amblyomma*
271	Female	AlexNet	femea ovale (9).jpg	fold4	4	*Amblyomma*
272	Female	AlexNet	femea sculptum (15).JPG	fold4	5	*Amblyomma*
273	Female	AlexNet	femea sculptum (19).JPG	fold4	5	*Amblyomma*
274	Female	AlexNet	femea sculptum (2).jpg	fold4	5	*Amblyomma*
275	Female	AlexNet	femea sculptum (23).JPG	fold4	5	*Amblyomma*
276	Female	AlexNet	femea sculptum (29).JPG	fold4	5	*Amblyomma*
277	Female	AlexNet	femea sculptum (3).jpg	fold4	5	*Amblyomma*
278	Female	AlexNet	femea sculptum (37).JPG	fold4	5	*Amblyomma*
279	Female	AlexNet	femea sculptum (38).JPG	fold4	5	*Amblyomma*
280	Female	AlexNet	femea sculptum (41).JPG	fold4	5	*Amblyomma*
281	Female	AlexNet	femea sculptum (5).jpg	fold4	5	*Amblyomma*
282	Female	AlexNet	femea sculptum (52).JPG	fold4	5	*Amblyomma*
283	Female	AlexNet	femea sculptum (56).jpg	fold4	5	*Amblyomma*
284	Female	AlexNet	femea sculptum (57).jpg	fold4	5	*Amblyomma*
285	Female	AlexNet	femea triste (15).jpg	fold4	6	*Amblyomma*
286	Female	AlexNet	femea triste (18).jpg	fold4	6	*Amblyomma*
287	Female	AlexNet	femea triste (28).jpg	fold4	6	*Amblyomma*
288	Female	AlexNet	femea triste (3).jpg	fold4	6	*Amblyomma*
289	Female	AlexNet	femea triste (33).jpg	fold4	6	*Amblyomma*
290	Female	AlexNet	femea triste (34).jpg	fold4	6	*Amblyomma*
291	Female	AlexNet	femea triste (40).jpg	fold4	6	*Amblyomma*
292	Female	AlexNet	femea triste (41).jpg	fold4	6	*Amblyomma*
293	Female	AlexNet	femea triste (51).jpg	fold4	6	*Amblyomma*
294	Female	AlexNet	femea triste (52).jpg	fold4	6	*Amblyomma*
295	Female	AlexNet	femea aureolatum (13).jpg	fold5	1	*Amblyomma*
296	Female	AlexNet	femea aureolatum (18).jpg	fold5	1	*Amblyomma*
297	Female	AlexNet	femea aureolatum (23).jpg	fold5	1	*Amblyomma*
298	Female	AlexNet	femea aureolatum (24).jpg	fold5	1	*Amblyomma*
299	Female	AlexNet	femea aureolatum (25).jpg	fold5	1	*Amblyomma*
300	Female	AlexNet	femea aureolatum (26).jpg	fold5	1	*Amblyomma*
301	Female	AlexNet	femea aureolatum (33).jpg	fold5	1	*Amblyomma*
302	Female	AlexNet	femea aureolatum (41).jpg	fold5	1	*Amblyomma*
303	Female	AlexNet	femea aureolatum (45).jpg	fold5	1	*Amblyomma*
304	Female	AlexNet	femea aureolatum (56).jpg	fold5	1	*Amblyomma*
305	Female	AlexNet	femea aureolatum (57).jpg	fold5	1	*Amblyomma*
306	Female	AlexNet	femea aureolatum (58).jpg	fold5	1	*Amblyomma*
307	Female	AlexNet	femea cajennense (13).jpg	fold5	2	*Amblyomma*
308	Female	AlexNet	femea cajennense (15).jpg	fold5	2	*Amblyomma*
309	Female	AlexNet	femea cajennense (2).jpg	fold5	2	*Amblyomma*
310	Female	AlexNet	femea cajennense (21).jpg	fold5	2	*Amblyomma*
311	Female	AlexNet	femea cajennense (29).jpg	fold5	2	*Amblyomma*
312	Female	AlexNet	femea cajennense (31).jpg	fold5	2	*Amblyomma*
313	Female	AlexNet	femea cajennense (33).jpg	fold5	2	*Amblyomma*
314	Female	AlexNet	femea cajennense (40).jpg	fold5	2	*Amblyomma*
315	Female	AlexNet	femea cajennense (42).jpg	fold5	2	*Amblyomma*
316	Female	AlexNet	femea cajennense (48).jpg	fold5	2	*Amblyomma*
317	Female	AlexNet	femea cajennense (49).jpg	fold5	2	*Amblyomma*
318	Female	AlexNet	femea cajennense (5).jpg	fold5	2	*Amblyomma*
319	Female	AlexNet	femea cajennense (59).jpg	fold5	2	*Amblyomma*
320	Female	AlexNet	femea cajennense (64).jpg	fold5	2	*Amblyomma*
321	Female	AlexNet	femea cajennense (65).jpg	fold5	2	*Amblyomma*
322	Female	AlexNet	femea cajennense (82).jpg	fold5	2	*Amblyomma*
323	Female	AlexNet	femea dubitatum (1).jpg	fold5	3	*Amblyomma*
324	Female	AlexNet	femea dubitatum (14).jpg	fold5	3	*Amblyomma*
325	Female	AlexNet	femea dubitatum (20).jpg	fold5	3	*Amblyomma*
326	Female	AlexNet	femea dubitatum (31).jpg	fold5	3	*Amblyomma*
327	Female	AlexNet	femea dubitatum (4).jpg	fold5	3	*Amblyomma*
328	Female	AlexNet	femea dubitatum (5).jpg	fold5	3	*Amblyomma*
329	Female	AlexNet	femea dubitatum (9).jpg	fold5	3	*Amblyomma*
330	Female	AlexNet	femea ovale (12).jpg	fold5	4	*Amblyomma*
331	Female	AlexNet	femea ovale (13).jpg	fold5	4	*Amblyomma*
332	Female	AlexNet	femea ovale (16).jpg	fold5	4	*Amblyomma*
333	Female	AlexNet	femea ovale (21).jpg	fold5	4	*Amblyomma*

334	Female	AlexNet	femea ovale (27).jpg	fold5	4	*Amblyomma*
335	Female	AlexNet	femea ovale (41).jpg	fold5	4	*Amblyomma*
336	Female	AlexNet	femea ovale (42).jpg	fold5	4	*Amblyomma*
337	Female	AlexNet	femea ovale (43).jpg	fold5	4	*Amblyomma*
338	Female	AlexNet	femea ovale (47).jpg	fold5	4	*Amblyomma*
339	Female	AlexNet	femea ovale (60).jpg	fold5	4	*Amblyomma*
340	Female	AlexNet	femea ovale (63).jpg	fold5	4	*Amblyomma*
341	Female	AlexNet	femea ovale (68).jpg	fold5	4	*Amblyomma*
342	Female	AlexNet	femea ovale (69).jpg	fold5	4	*Amblyomma*
343	Female	AlexNet	femea ovale (72).jpg	fold5	4	*Amblyomma*
344	Female	AlexNet	femea ovale (79).jpg	fold5	4	*Amblyomma*
345	Female	AlexNet	femea ovale (8).jpg	fold5	4	*Amblyomma*
346	Female	AlexNet	femea sculptum (12).JPG	fold5	5	*Amblyomma*
347	Female	AlexNet	femea sculptum (20).JPG	fold5	5	*Amblyomma*
348	Female	AlexNet	femea sculptum (31).JPG	fold5	5	*Amblyomma*
349	Female	AlexNet	femea sculptum (32).JPG	fold5	5	*Amblyomma*
350	Female	AlexNet	femea sculptum (33).JPG	fold5	5	*Amblyomma*
351	Female	AlexNet	femea sculptum (35).JPG	fold5	5	*Amblyomma*
352	Female	AlexNet	femea sculptum (4).jpg	fold5	5	*Amblyomma*
353	Female	AlexNet	femea sculptum (48).JPG	fold5	5	*Amblyomma*
354	Female	AlexNet	femea sculptum (50).JPG	fold5	5	*Amblyomma*
355	Female	AlexNet	femea sculptum (51).JPG	fold5	5	*Amblyomma*
356	Female	AlexNet	femea sculptum (55).JPG	fold5	5	*Amblyomma*
357	Female	AlexNet	femea sculptum (63).jpg	fold5	5	*Amblyomma*
358	Female	AlexNet	femea triste (16).jpg	fold5	6	*Amblyomma*
359	Female	AlexNet	femea triste (17).jpg	fold5	6	*Amblyomma*
360	Female	AlexNet	femea triste (20).jpg	fold5	6	*Amblyomma*
361	Female	AlexNet	femea triste (21).jpg	fold5	6	*Amblyomma*
362	Female	AlexNet	femea triste (25).jpg	fold5	6	*Amblyomma*
363	Female	AlexNet	femea triste (29).jpg	fold5	6	*Amblyomma*
364	Female	AlexNet	femea triste (39).jpg	fold5	6	*Amblyomma*
365	Female	AlexNet	femea triste (48).jpg	fold5	6	*Amblyomma*
366	Female	AlexNet	femea triste (49).jpg	fold5	6	*Amblyomma*
367	Female	AlexNet	femea triste (6).jpg	fold5	6	*Amblyomma*
368	Female	AlexNet	femea triste (8).jpg	fold5	6	*Amblyomma*
369	Male	AlexNet	macho aureolatum (11).jpg	fold1	1	*Amblyomma*
370	Male	AlexNet	macho aureolatum (12).jpg	fold1	1	*Amblyomma*
371	Male	AlexNet	macho aureolatum (16).jpg	fold1	1	*Amblyomma*
372	Male	AlexNet	macho aureolatum (17).jpg	fold1	1	*Amblyomma*
373	Male	AlexNet	macho aureolatum (35).jpg	fold1	1	*Amblyomma*
374	Male	AlexNet	macho aureolatum (36).jpg	fold1	1	*Amblyomma*
375	Male	AlexNet	macho aureolatum (42).jpg	fold1	1	*Amblyomma*
376	Male	AlexNet	macho aureolatum (44).jpg	fold1	1	*Amblyomma*
377	Male	AlexNet	macho aureolatum (51).jpg	fold1	1	*Amblyomma*
378	Male	AlexNet	macho aureolatum (58).jpg	fold1	1	*Amblyomma*
379	Male	AlexNet	macho aureolatum (61).jpg	fold1	1	*Amblyomma*
380	Male	AlexNet	macho aureolatum (9).jpg	fold1	1	*Amblyomma*
381	Male	AlexNet	macho cajennense (1).jpg	fold1	2	*Amblyomma*
382	Male	AlexNet	macho cajennense (12).jpg	fold1	2	*Amblyomma*
383	Male	AlexNet	macho cajennense (17).jpg	fold1	2	*Amblyomma*
384	Male	AlexNet	macho cajennense (23).jpg	fold1	2	*Amblyomma*
385	Male	AlexNet	macho cajennense (34).jpg	fold1	2	*Amblyomma*
386	Male	AlexNet	macho cajennense (36).jpg	fold1	2	*Amblyomma*
387	Male	AlexNet	macho cajennense (37).jpg	fold1	2	*Amblyomma*
388	Male	AlexNet	macho cajennense (39).jpg	fold1	2	*Amblyomma*
389	Male	AlexNet	macho cajennense (52).jpg	fold1	2	*Amblyomma*
390	Male	AlexNet	macho cajennense (64).jpg	fold1	2	*Amblyomma*
391	Male	AlexNet	macho cajennense (66).jpg	fold1	2	*Amblyomma*
392	Male	AlexNet	macho cajennense (71).jpg	fold1	2	*Amblyomma*
393	Male	AlexNet	macho cajennense (73).jpg	fold1	2	*Amblyomma*
394	Male	AlexNet	macho cajennense (76).jpg	fold1	2	*Amblyomma*
395	Male	AlexNet	macho cajennense (82).jpg	fold1	2	*Amblyomma*
396	Male	AlexNet	macho cajennense (83).jpg	fold1	2	*Amblyomma*
397	Male	AlexNet	macho dubitatum (11).jpg	fold1	3	*Amblyomma*
398	Male	AlexNet	macho dubitatum (16).jpg	fold1	3	*Amblyomma*
399	Male	AlexNet	macho dubitatum (18).jpg	fold1	3	*Amblyomma*
400	Male	AlexNet	macho dubitatum (2).jpg	fold1	3	*Amblyomma*

401	Male	AlexNet	macho dubitatum (21).jpg	fold1	3	*Amblyomma*
402	Male	AlexNet	macho dubitatum (22).jpg	fold1	3	*Amblyomma*
403	Male	AlexNet	macho dubitatum (31).jpg	fold1	3	*Amblyomma*
404	Male	AlexNet	macho dubitatum (38).jpg	fold1	3	*Amblyomma*
405	Male	AlexNet	macho dubitatum (40).jpg	fold1	3	*Amblyomma*
406	Male	AlexNet	macho dubitatum (47).jpg	fold1	3	*Amblyomma*
407	Male	AlexNet	macho dubitatum (50).jpg	fold1	3	*Amblyomma*
408	Male	AlexNet	macho dubitatum (57).jpg	fold1	3	*Amblyomma*
409	Male	AlexNet	macho dubitatum (69).jpg	fold1	3	*Amblyomma*
410	Male	AlexNet	macho dubitatum (74).jpg	fold1	3	*Amblyomma*
411	Male	AlexNet	macho dubitatum (9).jpg	fold1	3	*Amblyomma*
412	Male	AlexNet	macho ovale (12).jpg	fold1	4	*Amblyomma*
413	Male	AlexNet	macho ovale (14).jpg	fold1	4	*Amblyomma*
414	Male	AlexNet	macho ovale (15).jpg	fold1	4	*Amblyomma*
415	Male	AlexNet	macho ovale (2).jpg	fold1	4	*Amblyomma*
416	Male	AlexNet	macho ovale (20).jpg	fold1	4	*Amblyomma*
417	Male	AlexNet	macho ovale (30).jpg	fold1	4	*Amblyomma*
418	Male	AlexNet	macho ovale (41).jpg	fold1	4	*Amblyomma*
419	Male	AlexNet	macho ovale (7).jpg	fold1	4	*Amblyomma*
420	Male	AlexNet	macho sculptum (16).JPG	fold1	5	*Amblyomma*
421	Male	AlexNet	macho sculptum (20).JPG	fold1	5	*Amblyomma*
422	Male	AlexNet	macho sculptum (23).JPG	fold1	5	*Amblyomma*
423	Male	AlexNet	macho sculptum (3).jpg	fold1	5	*Amblyomma*
424	Male	AlexNet	macho sculptum (30).JPG	fold1	5	*Amblyomma*
425	Male	AlexNet	macho sculptum (4).jpg	fold1	5	*Amblyomma*
426	Male	AlexNet	macho sculptum (40).JPG	fold1	5	*Amblyomma*
427	Male	AlexNet	macho sculptum (43).JPG	fold1	5	*Amblyomma*
428	Male	AlexNet	macho sculptum (45).JPG	fold1	5	*Amblyomma*
429	Male	AlexNet	macho sculptum (46).JPG	fold1	5	*Amblyomma*
430	Male	AlexNet	macho sculptum (5).jpg	fold1	5	*Amblyomma*
431	Male	AlexNet	macho sculptum (53).JPG	fold1	5	*Amblyomma*
432	Male	AlexNet	macho sculptum (56).JPG	fold1	5	*Amblyomma*
433	Male	AlexNet	macho sculptum (62).JPG	fold1	5	*Amblyomma*
434	Male	AlexNet	macho sculptum (63).JPG	fold1	5	*Amblyomma*
435	Male	AlexNet	macho sculptum (71).JPG	fold1	5	*Amblyomma*
436	Male	AlexNet	macho sculptum (81).JPG	fold1	5	*Amblyomma*
437	Male	AlexNet	macho sculptum (89).jpg	fold1	5	*Amblyomma*
438	Male	AlexNet	macho triste (10).jpg	fold1	6	*Amblyomma*
439	Male	AlexNet	macho triste (13).jpg	fold1	6	*Amblyomma*
440	Male	AlexNet	macho triste (18).jpg	fold1	6	*Amblyomma*
441	Male	AlexNet	macho triste (19).jpg	fold1	6	*Amblyomma*
442	Male	AlexNet	macho triste (23).jpg	fold1	6	*Amblyomma*
443	Male	AlexNet	macho triste (29).jpg	fold1	6	*Amblyomma*
444	Male	AlexNet	macho triste (3).jpg	fold1	6	*Amblyomma*
445	Male	AlexNet	macho triste (32).jpg	fold1	6	*Amblyomma*
446	Male	AlexNet	macho triste (38).jpg	fold1	6	*Amblyomma*
447	Male	AlexNet	macho triste (43).jpg	fold1	6	*Amblyomma*
448	Male	AlexNet	macho triste (48).jpg	fold1	6	*Amblyomma*
449	Male	AlexNet	macho triste (5).jpg	fold1	6	*Amblyomma*
450	Male	AlexNet	macho triste (52).jpg	fold1	6	*Amblyomma*
451	Male	AlexNet	macho triste (56).jpg	fold1	6	*Amblyomma*
452	Male	AlexNet	macho triste (7).jpg	fold1	6	*Amblyomma*
453	Male	AlexNet	macho triste (75).jpg	fold1	6	*Amblyomma*
454	Male	AlexNet	macho triste (83).jpg	fold1	6	*Amblyomma*
455	Male	AlexNet	macho triste (88).jpg	fold1	6	*Amblyomma*
456	Male	AlexNet	macho triste (92).jpg	fold1	6	*Amblyomma*
457	Male	AlexNet	macho triste (93).jpg	fold1	6	*Amblyomma*
458	Male	AlexNet	macho aureolatum (19).jpg	fold2	1	*Amblyomma*
459	Male	AlexNet	macho aureolatum (22).jpg	fold2	1	*Amblyomma*
460	Male	AlexNet	macho aureolatum (26).jpg	fold2	1	*Amblyomma*
461	Male	AlexNet	macho aureolatum (28).jpg	fold2	1	*Amblyomma*
462	Male	AlexNet	macho aureolatum (32).jpg	fold2	1	*Amblyomma*
463	Male	AlexNet	macho aureolatum (46).jpg	fold2	1	*Amblyomma*
464	Male	AlexNet	macho aureolatum (49).jpg	fold2	1	*Amblyomma*
465	Male	AlexNet	macho aureolatum (50).jpg	fold2	1	*Amblyomma*
466	Male	AlexNet	macho aureolatum (52).jpg	fold2	1	*Amblyomma*
467	Male	AlexNet	macho aureolatum (56).jpg	fold2	1	*Amblyomma*

468	Male	AlexNet	macho aureolatum (59).jpg	fold2	1	*Amblyomma*
469	Male	AlexNet	macho aureolatum (6).jpg	fold2	1	*Amblyomma*
470	Male	AlexNet	macho aureolatum (62).jpg	fold2	1	*Amblyomma*
471	Male	AlexNet	macho cajennense (10).jpg	fold2	2	*Amblyomma*
472	Male	AlexNet	macho cajennense (2).jpg	fold2	2	*Amblyomma*
473	Male	AlexNet	macho cajennense (26).jpg	fold2	2	*Amblyomma*
474	Male	AlexNet	macho cajennense (27).jpg	fold2	2	*Amblyomma*
475	Male	AlexNet	macho cajennense (3).jpg	fold2	2	*Amblyomma*
476	Male	AlexNet	macho cajennense (30).jpg	fold2	2	*Amblyomma*
477	Male	AlexNet	macho cajennense (43).jpg	fold2	2	*Amblyomma*
478	Male	AlexNet	macho cajennense (53).jpg	fold2	2	*Amblyomma*
479	Male	AlexNet	macho cajennense (59).jpg	fold2	2	*Amblyomma*
480	Male	AlexNet	macho cajennense (60).jpg	fold2	2	*Amblyomma*
481	Male	AlexNet	macho cajennense (61).jpg	fold2	2	*Amblyomma*
482	Male	AlexNet	macho cajennense (68).jpg	fold2	2	*Amblyomma*
483	Male	AlexNet	macho cajennense (70).jpg	fold2	2	*Amblyomma*
484	Male	AlexNet	macho cajennense (72).jpg	fold2	2	*Amblyomma*
485	Male	AlexNet	macho cajennense (8).jpg	fold2	2	*Amblyomma*
486	Male	AlexNet	macho cajennense (81).jpg	fold2	2	*Amblyomma*
487	Male	AlexNet	macho cajennense (9).jpg	fold2	2	*Amblyomma*
488	Male	AlexNet	macho dubitatum (1).jpg	fold2	3	*Amblyomma*
489	Male	AlexNet	macho dubitatum (13).jpg	fold2	3	*Amblyomma*
490	Male	AlexNet	macho dubitatum (29).jpg	fold2	3	*Amblyomma*
491	Male	AlexNet	macho dubitatum (30).jpg	fold2	3	*Amblyomma*
492	Male	AlexNet	macho dubitatum (35).jpg	fold2	3	*Amblyomma*
493	Male	AlexNet	macho dubitatum (43).jpg	fold2	3	*Amblyomma*
494	Male	AlexNet	macho dubitatum (46).jpg	fold2	3	*Amblyomma*
495	Male	AlexNet	macho dubitatum (48).jpg	fold2	3	*Amblyomma*
496	Male	AlexNet	macho dubitatum (5).JPG	fold2	3	*Amblyomma*
497	Male	AlexNet	macho dubitatum (53).jpg	fold2	3	*Amblyomma*
498	Male	AlexNet	macho dubitatum (55).jpg	fold2	3	*Amblyomma*
499	Male	AlexNet	macho dubitatum (56).jpg	fold2	3	*Amblyomma*
500	Male	AlexNet	macho dubitatum (60).jpg	fold2	3	*Amblyomma*
501	Male	AlexNet	macho dubitatum (72).jpg	fold2	3	*Amblyomma*
502	Male	AlexNet	macho dubitatum (75).jpg	fold2	3	*Amblyomma*
503	Male	AlexNet	macho ovale (18).jpg	fold2	4	*Amblyomma*
504	Male	AlexNet	macho ovale (27).JPG	fold2	4	*Amblyomma*
505	Male	AlexNet	macho ovale (33).jpg	fold2	4	*Amblyomma*
506	Male	AlexNet	macho ovale (35).jpg	fold2	4	*Amblyomma*
507	Male	AlexNet	macho ovale (36).jpg	fold2	4	*Amblyomma*
508	Male	AlexNet	macho ovale (37).jpg	fold2	4	*Amblyomma*
509	Male	AlexNet	macho ovale (39).jpg	fold2	4	*Amblyomma*
510	Male	AlexNet	macho ovale (40).jpg	fold2	4	*Amblyomma*
511	Male	AlexNet	macho ovale (6).jpg	fold2	4	*Amblyomma*
512	Male	AlexNet	macho sculptum (12).JPG	fold2	5	*Amblyomma*
513	Male	AlexNet	macho sculptum (21).JPG	fold2	5	*Amblyomma*
514	Male	AlexNet	macho sculptum (24).JPG	fold2	5	*Amblyomma*
515	Male	AlexNet	macho sculptum (26).JPG	fold2	5	*Amblyomma*
516	Male	AlexNet	macho sculptum (27).JPG	fold2	5	*Amblyomma*
517	Male	AlexNet	macho sculptum (36).JPG	fold2	5	*Amblyomma*
518	Male	AlexNet	macho sculptum (42).JPG	fold2	5	*Amblyomma*
519	Male	AlexNet	macho sculptum (55).JPG	fold2	5	*Amblyomma*
520	Male	AlexNet	macho sculptum (59).JPG	fold2	5	*Amblyomma*
521	Male	AlexNet	macho sculptum (67).JPG	fold2	5	*Amblyomma*
522	Male	AlexNet	macho sculptum (7).jpg	fold2	5	*Amblyomma*
523	Male	AlexNet	macho sculptum (70).JPG	fold2	5	*Amblyomma*
524	Male	AlexNet	macho sculptum (73).JPG	fold2	5	*Amblyomma*
525	Male	AlexNet	macho sculptum (78).JPG	fold2	5	*Amblyomma*
526	Male	AlexNet	macho sculptum (82).jpg	fold2	5	*Amblyomma*
527	Male	AlexNet	macho sculptum (86).jpg	fold2	5	*Amblyomma*
528	Male	AlexNet	macho sculptum (91).jpg	fold2	5	*Amblyomma*
529	Male	AlexNet	macho sculptum (92).jpg	fold2	5	*Amblyomma*
530	Male	AlexNet	macho triste (14).jpg	fold2	6	*Amblyomma*
531	Male	AlexNet	macho triste (15).jpg	fold2	6	*Amblyomma*
532	Male	AlexNet	macho triste (17).jpg	fold2	6	*Amblyomma*
533	Male	AlexNet	macho triste (20).jpg	fold2	6	*Amblyomma*
534	Male	AlexNet	macho triste (21).jpg	fold2	6	*Amblyomma*

535	Male	AlexNet	macho triste (24).jpg	fold2	6	*Amblyomma*
536	Male	AlexNet	macho triste (49).jpg	fold2	6	*Amblyomma*
537	Male	AlexNet	macho triste (50).jpg	fold2	6	*Amblyomma*
538	Male	AlexNet	macho triste (61).jpg	fold2	6	*Amblyomma*
539	Male	AlexNet	macho triste (62).jpg	fold2	6	*Amblyomma*
540	Male	AlexNet	macho triste (63).jpg	fold2	6	*Amblyomma*
541	Male	AlexNet	macho triste (64).jpg	fold2	6	*Amblyomma*
542	Male	AlexNet	macho triste (67).jpg	fold2	6	*Amblyomma*
543	Male	AlexNet	macho triste (68).jpg	fold2	6	*Amblyomma*
544	Male	AlexNet	macho triste (73).jpg	fold2	6	*Amblyomma*
545	Male	AlexNet	macho triste (77).jpg	fold2	6	*Amblyomma*
546	Male	AlexNet	macho triste (78).jpg	fold2	6	*Amblyomma*
547	Male	AlexNet	macho triste (82).jpg	fold2	6	*Amblyomma*
548	Male	AlexNet	macho triste (9).jpg	fold2	6	*Amblyomma*
549	Male	AlexNet	macho triste (90).jpg	fold2	6	*Amblyomma*
550	Male	AlexNet	macho triste (91).jpg	fold2	6	*Amblyomma*
551	Male	AlexNet	macho aureolatum (14).jpg	fold3	1	*Amblyomma*
552	Male	AlexNet	macho aureolatum (20).jpg	fold3	1	*Amblyomma*
553	Male	AlexNet	macho aureolatum (27).jpg	fold3	1	*Amblyomma*
554	Male	AlexNet	macho aureolatum (29).jpg	fold3	1	*Amblyomma*
555	Male	AlexNet	macho aureolatum (31).jpg	fold3	1	*Amblyomma*
556	Male	AlexNet	macho aureolatum (38).jpg	fold3	1	*Amblyomma*
557	Male	AlexNet	macho aureolatum (41).jpg	fold3	1	*Amblyomma*
558	Male	AlexNet	macho aureolatum (47).jpg	fold3	1	*Amblyomma*
559	Male	AlexNet	macho aureolatum (55).jpg	fold3	1	*Amblyomma*
560	Male	AlexNet	macho aureolatum (57).jpg	fold3	1	*Amblyomma*
561	Male	AlexNet	macho aureolatum (63).jpg	fold3	1	*Amblyomma*
562	Male	AlexNet	macho aureolatum (7).jpg	fold3	1	*Amblyomma*
563	Male	AlexNet	macho cajennense (11).jpg	fold3	2	*Amblyomma*
564	Male	AlexNet	macho cajennense (14).jpg	fold3	2	*Amblyomma*
565	Male	AlexNet	macho cajennense (15).jpg	fold3	2	*Amblyomma*
566	Male	AlexNet	macho cajennense (24).jpg	fold3	2	*Amblyomma*
567	Male	AlexNet	macho cajennense (29).jpg	fold3	2	*Amblyomma*
568	Male	AlexNet	macho cajennense (33).jpg	fold3	2	*Amblyomma*
569	Male	AlexNet	macho cajennense (38).jpg	fold3	2	*Amblyomma*
570	Male	AlexNet	macho cajennense (42).jpg	fold3	2	*Amblyomma*
571	Male	AlexNet	macho cajennense (46).jpg	fold3	2	*Amblyomma*
572	Male	AlexNet	macho cajennense (5).jpg	fold3	2	*Amblyomma*
573	Male	AlexNet	macho cajennense (54).jpg	fold3	2	*Amblyomma*
574	Male	AlexNet	macho cajennense (56).jpg	fold3	2	*Amblyomma*
575	Male	AlexNet	macho cajennense (6).jpg	fold3	2	*Amblyomma*
576	Male	AlexNet	macho cajennense (63).jpg	fold3	2	*Amblyomma*
577	Male	AlexNet	macho cajennense (65).jpg	fold3	2	*Amblyomma*
578	Male	AlexNet	macho cajennense (67).jpg	fold3	2	*Amblyomma*
579	Male	AlexNet	macho cajennense (7).jpg	fold3	2	*Amblyomma*
580	Male	AlexNet	macho dubitatum (14).jpg	fold3	3	*Amblyomma*
581	Male	AlexNet	macho dubitatum (20).jpg	fold3	3	*Amblyomma*
582	Male	AlexNet	macho dubitatum (23).jpg	fold3	3	*Amblyomma*
583	Male	AlexNet	macho dubitatum (25).jpg	fold3	3	*Amblyomma*
584	Male	AlexNet	macho dubitatum (26).jpg	fold3	3	*Amblyomma*
585	Male	AlexNet	macho dubitatum (36).jpg	fold3	3	*Amblyomma*
586	Male	AlexNet	macho dubitatum (37).jpg	fold3	3	*Amblyomma*
587	Male	AlexNet	macho dubitatum (41).jpg	fold3	3	*Amblyomma*
588	Male	AlexNet	macho dubitatum (42).jpg	fold3	3	*Amblyomma*
589	Male	AlexNet	macho dubitatum (52).jpg	fold3	3	*Amblyomma*
590	Male	AlexNet	macho dubitatum (59).jpg	fold3	3	*Amblyomma*
591	Male	AlexNet	macho dubitatum (65).jpg	fold3	3	*Amblyomma*
592	Male	AlexNet	macho dubitatum (66).jpg	fold3	3	*Amblyomma*
593	Male	AlexNet	macho dubitatum (67).jpg	fold3	3	*Amblyomma*
594	Male	AlexNet	macho dubitatum (73).jpg	fold3	3	*Amblyomma*
595	Male	AlexNet	macho ovale (10).jpg	fold3	4	*Amblyomma*
596	Male	AlexNet	macho ovale (11).jpg	fold3	4	*Amblyomma*
597	Male	AlexNet	macho ovale (19).jpg	fold3	4	*Amblyomma*
598	Male	AlexNet	macho ovale (22).jpg	fold3	4	*Amblyomma*
599	Male	AlexNet	macho ovale (28).JPG	fold3	4	*Amblyomma*
600	Male	AlexNet	macho ovale (31).jpg	fold3	4	*Amblyomma*
601	Male	AlexNet	macho ovale (5).jpg	fold3	4	*Amblyomma*

602	Male	AlexNet	macho ovale (8).jpg	fold3	4	*Amblyomma*
603	Male	AlexNet	macho ovale (9).jpg	fold3	4	*Amblyomma*
604	Male	AlexNet	macho sculptum (1).jpg	fold3	5	*Amblyomma*
605	Male	AlexNet	macho sculptum (13).JPG	fold3	5	*Amblyomma*
606	Male	AlexNet	macho sculptum (14).JPG	fold3	5	*Amblyomma*
607	Male	AlexNet	macho sculptum (18).JPG	fold3	5	*Amblyomma*
608	Male	AlexNet	macho sculptum (2).jpg	fold3	5	*Amblyomma*
609	Male	AlexNet	macho sculptum (22).JPG	fold3	5	*Amblyomma*
610	Male	AlexNet	macho sculptum (31).JPG	fold3	5	*Amblyomma*
611	Male	AlexNet	macho sculptum (34).JPG	fold3	5	*Amblyomma*
612	Male	AlexNet	macho sculptum (35).JPG	fold3	5	*Amblyomma*
613	Male	AlexNet	macho sculptum (37).JPG	fold3	5	*Amblyomma*
614	Male	AlexNet	macho sculptum (39).JPG	fold3	5	*Amblyomma*
615	Male	AlexNet	macho sculptum (44).JPG	fold3	5	*Amblyomma*
616	Male	AlexNet	macho sculptum (49).JPG	fold3	5	*Amblyomma*
617	Male	AlexNet	macho sculptum (57).JPG	fold3	5	*Amblyomma*
618	Male	AlexNet	macho sculptum (83).jpg	fold3	5	*Amblyomma*
619	Male	AlexNet	macho sculptum (84).jpg	fold3	5	*Amblyomma*
620	Male	AlexNet	macho sculptum (85).jpg	fold3	5	*Amblyomma*
621	Male	AlexNet	macho sculptum (87).jpg	fold3	5	*Amblyomma*
622	Male	AlexNet	macho sculptum (90).jpg	fold3	5	*Amblyomma*
623	Male	AlexNet	macho triste (1).jpg	fold3	6	*Amblyomma*
624	Male	AlexNet	macho triste (100).jpg	fold3	6	*Amblyomma*
625	Male	AlexNet	macho triste (101).jpg	fold3	6	*Amblyomma*
626	Male	AlexNet	macho triste (16).jpg	fold3	6	*Amblyomma*
627	Male	AlexNet	macho triste (26).jpg	fold3	6	*Amblyomma*
628	Male	AlexNet	macho triste (34).jpg	fold3	6	*Amblyomma*
629	Male	AlexNet	macho triste (35).jpg	fold3	6	*Amblyomma*
630	Male	AlexNet	macho triste (40).jpg	fold3	6	*Amblyomma*
631	Male	AlexNet	macho triste (44).jpg	fold3	6	*Amblyomma*
632	Male	AlexNet	macho triste (45).jpg	fold3	6	*Amblyomma*
633	Male	AlexNet	macho triste (51).jpg	fold3	6	*Amblyomma*
634	Male	AlexNet	macho triste (59).jpg	fold3	6	*Amblyomma*
635	Male	AlexNet	macho triste (6).jpg	fold3	6	*Amblyomma*
636	Male	AlexNet	macho triste (66).jpg	fold3	6	*Amblyomma*
637	Male	AlexNet	macho triste (71).jpg	fold3	6	*Amblyomma*
638	Male	AlexNet	macho triste (76).jpg	fold3	6	*Amblyomma*
639	Male	AlexNet	macho triste (80).jpg	fold3	6	*Amblyomma*
640	Male	AlexNet	macho triste (86).jpg	fold3	6	*Amblyomma*
641	Male	AlexNet	macho triste (87).jpg	fold3	6	*Amblyomma*
642	Male	AlexNet	macho triste (94).jpg	fold3	6	*Amblyomma*
643	Male	AlexNet	macho aureolatum (1).jpg	fold4	1	*Amblyomma*
644	Male	AlexNet	macho aureolatum (10).jpg	fold4	1	*Amblyomma*
645	Male	AlexNet	macho aureolatum (15).jpg	fold4	1	*Amblyomma*
646	Male	AlexNet	macho aureolatum (2).jpg	fold4	1	*Amblyomma*
647	Male	AlexNet	macho aureolatum (23).jpg	fold4	1	*Amblyomma*
648	Male	AlexNet	macho aureolatum (24).jpg	fold4	1	*Amblyomma*
649	Male	AlexNet	macho aureolatum (25).jpg	fold4	1	*Amblyomma*
650	Male	AlexNet	macho aureolatum (33).jpg	fold4	1	*Amblyomma*
651	Male	AlexNet	macho aureolatum (37).jpg	fold4	1	*Amblyomma*
652	Male	AlexNet	macho aureolatum (39).jpg	fold4	1	*Amblyomma*
653	Male	AlexNet	macho aureolatum (4).jpg	fold4	1	*Amblyomma*
654	Male	AlexNet	macho aureolatum (48).jpg	fold4	1	*Amblyomma*
655	Male	AlexNet	macho aureolatum (60).jpg	fold4	1	*Amblyomma*
656	Male	AlexNet	macho cajennense (13).jpg	fold4	2	*Amblyomma*
657	Male	AlexNet	macho cajennense (16).jpg	fold4	2	*Amblyomma*
658	Male	AlexNet	macho cajennense (19).jpg	fold4	2	*Amblyomma*
659	Male	AlexNet	macho cajennense (20).jpg	fold4	2	*Amblyomma*
660	Male	AlexNet	macho cajennense (21).jpg	fold4	2	*Amblyomma*
661	Male	AlexNet	macho cajennense (28).jpg	fold4	2	*Amblyomma*
662	Male	AlexNet	macho cajennense (31).jpg	fold4	2	*Amblyomma*
663	Male	AlexNet	macho cajennense (32).jpg	fold4	2	*Amblyomma*
664	Male	AlexNet	macho cajennense (40).jpg	fold4	2	*Amblyomma*
665	Male	AlexNet	macho cajennense (41).jpg	fold4	2	*Amblyomma*
666	Male	AlexNet	macho cajennense (45).jpg	fold4	2	*Amblyomma*
667	Male	AlexNet	macho cajennense (47).jpg	fold4	2	*Amblyomma*
668	Male	AlexNet	macho cajennense (55).jpg	fold4	2	*Amblyomma*

669	Male	AlexNet	macho cajennense (58).jpg	fold4	2	*Amblyomma*
670	Male	AlexNet	macho cajennense (79).jpg	fold4	2	*Amblyomma*
671	Male	AlexNet	macho cajennense (80).jpg	fold4	2	*Amblyomma*
672	Male	AlexNet	macho dubitatum (10).jpg	fold4	3	*Amblyomma*
673	Male	AlexNet	macho dubitatum (12).jpg	fold4	3	*Amblyomma*
674	Male	AlexNet	macho dubitatum (15).jpg	fold4	3	*Amblyomma*
675	Male	AlexNet	macho dubitatum (28).jpg	fold4	3	*Amblyomma*
676	Male	AlexNet	macho dubitatum (3).jpg	fold4	3	*Amblyomma*
677	Male	AlexNet	macho dubitatum (32).jpg	fold4	3	*Amblyomma*
678	Male	AlexNet	macho dubitatum (33).jpg	fold4	3	*Amblyomma*
679	Male	AlexNet	macho dubitatum (39).jpg	fold4	3	*Amblyomma*
680	Male	AlexNet	macho dubitatum (4).jpg	fold4	3	*Amblyomma*
681	Male	AlexNet	macho dubitatum (54).jpg	fold4	3	*Amblyomma*
682	Male	AlexNet	macho dubitatum (6).JPG	fold4	3	*Amblyomma*
683	Male	AlexNet	macho dubitatum (61).jpg	fold4	3	*Amblyomma*
684	Male	AlexNet	macho dubitatum (63).jpg	fold4	3	*Amblyomma*
685	Male	AlexNet	macho dubitatum (7).JPG	fold4	3	*Amblyomma*
686	Male	AlexNet	macho dubitatum (71).jpg	fold4	3	*Amblyomma*
687	Male	AlexNet	macho ovale (1).jpg	fold4	4	*Amblyomma*
688	Male	AlexNet	macho ovale (23).jpg	fold4	4	*Amblyomma*
689	Male	AlexNet	macho ovale (26).jpg	fold4	4	*Amblyomma*
690	Male	AlexNet	macho ovale (29).jpg	fold4	4	*Amblyomma*
691	Male	AlexNet	macho ovale (34).jpg	fold4	4	*Amblyomma*
692	Male	AlexNet	macho ovale (38).jpg	fold4	4	*Amblyomma*
693	Male	AlexNet	macho ovale (4).jpg	fold4	4	*Amblyomma*
694	Male	AlexNet	macho ovale (42).jpg	fold4	4	*Amblyomma*
695	Male	AlexNet	macho sculptum (10).JPG	fold4	5	*Amblyomma*
696	Male	AlexNet	macho sculptum (11).JPG	fold4	5	*Amblyomma*
697	Male	AlexNet	macho sculptum (15).JPG	fold4	5	*Amblyomma*
698	Male	AlexNet	macho sculptum (17).JPG	fold4	5	*Amblyomma*
699	Male	AlexNet	macho sculptum (33).JPG	fold4	5	*Amblyomma*
700	Male	AlexNet	macho sculptum (38).JPG	fold4	5	*Amblyomma*
701	Male	AlexNet	macho sculptum (47).JPG	fold4	5	*Amblyomma*
702	Male	AlexNet	macho sculptum (48).JPG	fold4	5	*Amblyomma*
703	Male	AlexNet	macho sculptum (51).JPG	fold4	5	*Amblyomma*
704	Male	AlexNet	macho sculptum (52).JPG	fold4	5	*Amblyomma*
705	Male	AlexNet	macho sculptum (61).JPG	fold4	5	*Amblyomma*
706	Male	AlexNet	macho sculptum (66).JPG	fold4	5	*Amblyomma*
707	Male	AlexNet	macho sculptum (68).JPG	fold4	5	*Amblyomma*
708	Male	AlexNet	macho sculptum (69).JPG	fold4	5	*Amblyomma*
709	Male	AlexNet	macho sculptum (72).JPG	fold4	5	*Amblyomma*
710	Male	AlexNet	macho sculptum (76).JPG	fold4	5	*Amblyomma*
711	Male	AlexNet	macho sculptum (77).JPG	fold4	5	*Amblyomma*
712	Male	AlexNet	macho sculptum (80).JPG	fold4	5	*Amblyomma*
713	Male	AlexNet	macho sculptum (9).JPG	fold4	5	*Amblyomma*
714	Male	AlexNet	macho triste (102).jpg	fold4	6	*Amblyomma*
715	Male	AlexNet	macho triste (2).jpg	fold4	6	*Amblyomma*
716	Male	AlexNet	macho triste (31).jpg	fold4	6	*Amblyomma*
717	Male	AlexNet	macho triste (37).jpg	fold4	6	*Amblyomma*
718	Male	AlexNet	macho triste (39).jpg	fold4	6	*Amblyomma*
719	Male	AlexNet	macho triste (41).jpg	fold4	6	*Amblyomma*
720	Male	AlexNet	macho triste (42).jpg	fold4	6	*Amblyomma*
721	Male	AlexNet	macho triste (46).jpg	fold4	6	*Amblyomma*
722	Male	AlexNet	macho triste (47).jpg	fold4	6	*Amblyomma*
723	Male	AlexNet	macho triste (54).jpg	fold4	6	*Amblyomma*
724	Male	AlexNet	macho triste (58).jpg	fold4	6	*Amblyomma*
725	Male	AlexNet	macho triste (65).jpg	fold4	6	*Amblyomma*
726	Male	AlexNet	macho triste (69).jpg	fold4	6	*Amblyomma*
727	Male	AlexNet	macho triste (70).jpg	fold4	6	*Amblyomma*
728	Male	AlexNet	macho triste (72).jpg	fold4	6	*Amblyomma*
729	Male	AlexNet	macho triste (79).jpg	fold4	6	*Amblyomma*
730	Male	AlexNet	macho triste (81).jpg	fold4	6	*Amblyomma*
731	Male	AlexNet	macho triste (84).jpg	fold4	6	*Amblyomma*
732	Male	AlexNet	macho triste (85).jpg	fold4	6	*Amblyomma*
733	Male	AlexNet	macho triste (98).jpg	fold4	6	*Amblyomma*
734	Male	AlexNet	macho triste (99).jpg	fold4	6	*Amblyomma*
735	Male	AlexNet	macho aureolatum (13).jpg	fold5	1	*Amblyomma*

736	Male	AlexNet	macho aureolatum (18).jpg	fold5	1	*Amblyomma*
737	Male	AlexNet	macho aureolatum (21).jpg	fold5	1	*Amblyomma*
738	Male	AlexNet	macho aureolatum (3).jpg	fold5	1	*Amblyomma*
739	Male	AlexNet	macho aureolatum (30).jpg	fold5	1	*Amblyomma*
740	Male	AlexNet	macho aureolatum (34).jpg	fold5	1	*Amblyomma*
741	Male	AlexNet	macho aureolatum (40).jpg	fold5	1	*Amblyomma*
742	Male	AlexNet	macho aureolatum (43).jpg	fold5	1	*Amblyomma*
743	Male	AlexNet	macho aureolatum (45).jpg	fold5	1	*Amblyomma*
744	Male	AlexNet	macho aureolatum (5).jpg	fold5	1	*Amblyomma*
745	Male	AlexNet	macho aureolatum (53).jpg	fold5	1	*Amblyomma*
746	Male	AlexNet	macho aureolatum (54).jpg	fold5	1	*Amblyomma*
747	Male	AlexNet	macho aureolatum (8).jpg	fold5	1	*Amblyomma*
748	Male	AlexNet	macho cajennense (18).jpg	fold5	2	*Amblyomma*
749	Male	AlexNet	macho cajennense (22).jpg	fold5	2	*Amblyomma*
750	Male	AlexNet	macho cajennense (25).jpg	fold5	2	*Amblyomma*
751	Male	AlexNet	macho cajennense (35).jpg	fold5	2	*Amblyomma*
752	Male	AlexNet	macho cajennense (4).jpg	fold5	2	*Amblyomma*
753	Male	AlexNet	macho cajennense (44).jpg	fold5	2	*Amblyomma*
754	Male	AlexNet	macho cajennense (48).jpg	fold5	2	*Amblyomma*
755	Male	AlexNet	macho cajennense (49).jpg	fold5	2	*Amblyomma*
756	Male	AlexNet	macho cajennense (50).jpg	fold5	2	*Amblyomma*
757	Male	AlexNet	macho cajennense (51).jpg	fold5	2	*Amblyomma*
758	Male	AlexNet	macho cajennense (57).jpg	fold5	2	*Amblyomma*
759	Male	AlexNet	macho cajennense (62).jpg	fold5	2	*Amblyomma*
760	Male	AlexNet	macho cajennense (69).jpg	fold5	2	*Amblyomma*
761	Male	AlexNet	macho cajennense (74).jpg	fold5	2	*Amblyomma*
762	Male	AlexNet	macho cajennense (75).jpg	fold5	2	*Amblyomma*
763	Male	AlexNet	macho cajennense (77).jpg	fold5	2	*Amblyomma*
764	Male	AlexNet	macho cajennense (78).jpg	fold5	2	*Amblyomma*
765	Male	AlexNet	macho dubitatum (17).jpg	fold5	3	*Amblyomma*
766	Male	AlexNet	macho dubitatum (19).jpg	fold5	3	*Amblyomma*
767	Male	AlexNet	macho dubitatum (24).jpg	fold5	3	*Amblyomma*
768	Male	AlexNet	macho dubitatum (27).jpg	fold5	3	*Amblyomma*
769	Male	AlexNet	macho dubitatum (34).jpg	fold5	3	*Amblyomma*
770	Male	AlexNet	macho dubitatum (44).jpg	fold5	3	*Amblyomma*
771	Male	AlexNet	macho dubitatum (45).jpg	fold5	3	*Amblyomma*
772	Male	AlexNet	macho dubitatum (49).jpg	fold5	3	*Amblyomma*
773	Male	AlexNet	macho dubitatum (51).jpg	fold5	3	*Amblyomma*
774	Male	AlexNet	macho dubitatum (58).jpg	fold5	3	*Amblyomma*
775	Male	AlexNet	macho dubitatum (62).jpg	fold5	3	*Amblyomma*
776	Male	AlexNet	macho dubitatum (64).jpg	fold5	3	*Amblyomma*
777	Male	AlexNet	macho dubitatum (68).jpg	fold5	3	*Amblyomma*
778	Male	AlexNet	macho dubitatum (70).jpg	fold5	3	*Amblyomma*
779	Male	AlexNet	macho dubitatum (8).JPG	fold5	3	*Amblyomma*
780	Male	AlexNet	macho ovale (13).jpg	fold5	4	*Amblyomma*
781	Male	AlexNet	macho ovale (16).jpg	fold5	4	*Amblyomma*
782	Male	AlexNet	macho ovale (17).jpg	fold5	4	*Amblyomma*
783	Male	AlexNet	macho ovale (21).jpg	fold5	4	*Amblyomma*
784	Male	AlexNet	macho ovale (24).jpg	fold5	4	*Amblyomma*
785	Male	AlexNet	macho ovale (25).jpg	fold5	4	*Amblyomma*
786	Male	AlexNet	macho ovale (3).jpg	fold5	4	*Amblyomma*
787	Male	AlexNet	macho ovale (32).jpg	fold5	4	*Amblyomma*
788	Male	AlexNet	macho sculptum (19).JPG	fold5	5	*Amblyomma*
789	Male	AlexNet	macho sculptum (25).JPG	fold5	5	*Amblyomma*
790	Male	AlexNet	macho sculptum (28).JPG	fold5	5	*Amblyomma*
791	Male	AlexNet	macho sculptum (29).JPG	fold5	5	*Amblyomma*
792	Male	AlexNet	macho sculptum (32).JPG	fold5	5	*Amblyomma*
793	Male	AlexNet	macho sculptum (41).JPG	fold5	5	*Amblyomma*
794	Male	AlexNet	macho sculptum (50).JPG	fold5	5	*Amblyomma*
795	Male	AlexNet	macho sculptum (54).JPG	fold5	5	*Amblyomma*
796	Male	AlexNet	macho sculptum (58).JPG	fold5	5	*Amblyomma*
797	Male	AlexNet	macho sculptum (6).jpg	fold5	5	*Amblyomma*
798	Male	AlexNet	macho sculptum (60).JPG	fold5	5	*Amblyomma*
799	Male	AlexNet	macho sculptum (64).JPG	fold5	5	*Amblyomma*
800	Male	AlexNet	macho sculptum (65).JPG	fold5	5	*Amblyomma*
801	Male	AlexNet	macho sculptum (74).JPG	fold5	5	*Amblyomma*
802	Male	AlexNet	macho sculptum (75).JPG	fold5	5	*Amblyomma*

803	Male	AlexNet	macho sculptum (79).JPG	fold5	5	*Amblyomma*
804	Male	AlexNet	macho sculptum (8).JPG	fold5	5	*Amblyomma*
805	Male	AlexNet	macho sculptum (88).jpg	fold5	5	*Amblyomma*
806	Male	AlexNet	macho triste (103).jpg	fold5	6	*Amblyomma*
807	Male	AlexNet	macho triste (11).jpg	fold5	6	*Amblyomma*
808	Male	AlexNet	macho triste (12).jpg	fold5	6	*Amblyomma*
809	Male	AlexNet	macho triste (22).jpg	fold5	6	*Amblyomma*
810	Male	AlexNet	macho triste (25).jpg	fold5	6	*Amblyomma*
811	Male	AlexNet	macho triste (27).jpg	fold5	6	*Amblyomma*
812	Male	AlexNet	macho triste (28).jpg	fold5	6	*Amblyomma*
813	Male	AlexNet	macho triste (30).jpg	fold5	6	*Amblyomma*
814	Male	AlexNet	macho triste (33).jpg	fold5	6	*Amblyomma*
815	Male	AlexNet	macho triste (36).jpg	fold5	6	*Amblyomma*
816	Male	AlexNet	macho triste (4).jpg	fold5	6	*Amblyomma*
817	Male	AlexNet	macho triste (53).jpg	fold5	6	*Amblyomma*
818	Male	AlexNet	macho triste (55).jpg	fold5	6	*Amblyomma*
819	Male	AlexNet	macho triste (57).jpg	fold5	6	*Amblyomma*
820	Male	AlexNet	macho triste (60).jpg	fold5	6	*Amblyomma*
821	Male	AlexNet	macho triste (74).jpg	fold5	6	*Amblyomma*
822	Male	AlexNet	macho triste (8).jpg	fold5	6	*Amblyomma*
823	Male	AlexNet	macho triste (89).jpg	fold5	6	*Amblyomma*
824	Male	AlexNet	macho triste (95).jpg	fold5	6	*Amblyomma*
825	Male	AlexNet	macho triste (96).jpg	fold5	6	*Amblyomma*
826	Male	AlexNet	macho triste (97).jpg	fold5	6	*Amblyomma*
827	Dorsal	AlexNet	dorsal aureolatum (11).jpg	fold1	1	*Amblyomma*
828	Dorsal	AlexNet	dorsal aureolatum (17).jpg	fold1	1	*Amblyomma*
829	Dorsal	AlexNet	dorsal aureolatum (23).jpg	fold1	1	*Amblyomma*
830	Dorsal	AlexNet	dorsal aureolatum (27).jpg	fold1	1	*Amblyomma*
831	Dorsal	AlexNet	dorsal aureolatum (30).jpg	fold1	1	*Amblyomma*
832	Dorsal	AlexNet	dorsal aureolatum (46).jpg	fold1	1	*Amblyomma*
833	Dorsal	AlexNet	dorsal aureolatum (48).jpg	fold1	1	*Amblyomma*
834	Dorsal	AlexNet	dorsal aureolatum (52).jpg	fold1	1	*Amblyomma*
835	Dorsal	AlexNet	dorsal aureolatum (55).jpg	fold1	1	*Amblyomma*
836	Dorsal	AlexNet	dorsal aureolatum (58).jpg	fold1	1	*Amblyomma*
837	Dorsal	AlexNet	dorsal aureolatum (61).jpg	fold1	1	*Amblyomma*
838	Dorsal	AlexNet	dorsal aureolatum (8).jpg	fold1	1	*Amblyomma*
839	Dorsal	AlexNet	dorsal cajennense (25).jpg	fold1	2	*Amblyomma*
840	Dorsal	AlexNet	dorsal cajennense (26).jpg	fold1	2	*Amblyomma*
841	Dorsal	AlexNet	dorsal cajennense (3).jpg	fold1	2	*Amblyomma*
842	Dorsal	AlexNet	dorsal cajennense (32).jpg	fold1	2	*Amblyomma*
843	Dorsal	AlexNet	dorsal cajennense (44).jpg	fold1	2	*Amblyomma*
844	Dorsal	AlexNet	dorsal cajennense (47).jpg	fold1	2	*Amblyomma*
845	Dorsal	AlexNet	dorsal cajennense (50).jpg	fold1	2	*Amblyomma*
846	Dorsal	AlexNet	dorsal cajennense (52).jpg	fold1	2	*Amblyomma*
847	Dorsal	AlexNet	dorsal cajennense (54).jpg	fold1	2	*Amblyomma*
848	Dorsal	AlexNet	dorsal cajennense (58).jpg	fold1	2	*Amblyomma*
849	Dorsal	AlexNet	dorsal cajennense (65).jpg	fold1	2	*Amblyomma*
850	Dorsal	AlexNet	dorsal cajennense (66).jpg	fold1	2	*Amblyomma*
851	Dorsal	AlexNet	dorsal cajennense (7).jpg	fold1	2	*Amblyomma*
852	Dorsal	AlexNet	dorsal cajennense (75).jpg	fold1	2	*Amblyomma*
853	Dorsal	AlexNet	dorsal cajennense (77).jpg	fold1	2	*Amblyomma*
854	Dorsal	AlexNet	dorsal cajennense (80).jpg	fold1	2	*Amblyomma*
855	Dorsal	AlexNet	dorsal dubitatum (10).jpg	fold1	3	*Amblyomma*
856	Dorsal	AlexNet	dorsal dubitatum (18).jpg	fold1	3	*Amblyomma*
857	Dorsal	AlexNet	dorsal dubitatum (19).jpg	fold1	3	*Amblyomma*
858	Dorsal	AlexNet	dorsal dubitatum (28).jpg	fold1	3	*Amblyomma*
859	Dorsal	AlexNet	dorsal dubitatum (32).jpg	fold1	3	*Amblyomma*
860	Dorsal	AlexNet	dorsal dubitatum (40).jpg	fold1	3	*Amblyomma*
861	Dorsal	AlexNet	dorsal dubitatum (46).jpg	fold1	3	*Amblyomma*
862	Dorsal	AlexNet	dorsal dubitatum (49).jpg	fold1	3	*Amblyomma*
863	Dorsal	AlexNet	dorsal dubitatum (55).jpg	fold1	3	*Amblyomma*
864	Dorsal	AlexNet	dorsal dubitatum (6).jpg	fold1	3	*Amblyomma*
865	Dorsal	AlexNet	dorsal dubitatum (9).JPG	fold1	3	*Amblyomma*
866	Dorsal	AlexNet	dorsal ovale (10).jpg	fold1	4	*Amblyomma*
867	Dorsal	AlexNet	dorsal ovale (14).jpg	fold1	4	*Amblyomma*
868	Dorsal	AlexNet	dorsal ovale (20).jpg	fold1	4	*Amblyomma*
869	Dorsal	AlexNet	dorsal ovale (21).jpg	fold1	4	*Amblyomma*

870	Dorsal	AlexNet	dorsal ovale (26).jpg	fold1	4	*Amblyomma*
871	Dorsal	AlexNet	dorsal ovale (29).jpg	fold1	4	*Amblyomma*
872	Dorsal	AlexNet	dorsal ovale (44).jpg	fold1	4	*Amblyomma*
873	Dorsal	AlexNet	dorsal ovale (48).jpg	fold1	4	*Amblyomma*
874	Dorsal	AlexNet	dorsal ovale (51).jpg	fold1	4	*Amblyomma*
875	Dorsal	AlexNet	dorsal ovale (53).jpg	fold1	4	*Amblyomma*
876	Dorsal	AlexNet	dorsal ovale (61).jpg	fold1	4	*Amblyomma*
877	Dorsal	AlexNet	dorsal ovale (7).jpg	fold1	4	*Amblyomma*
878	Dorsal	AlexNet	dorsal sculptum (14).JPG	fold1	5	*Amblyomma*
879	Dorsal	AlexNet	dorsal sculptum (20).JPG	fold1	5	*Amblyomma*
880	Dorsal	AlexNet	dorsal sculptum (22).JPG	fold1	5	*Amblyomma*
881	Dorsal	AlexNet	dorsal sculptum (23).JPG	fold1	5	*Amblyomma*
882	Dorsal	AlexNet	dorsal sculptum (25).JPG	fold1	5	*Amblyomma*
883	Dorsal	AlexNet	dorsal sculptum (29).jpg	fold1	5	*Amblyomma*
884	Dorsal	AlexNet	dorsal sculptum (37).JPG	fold1	5	*Amblyomma*
885	Dorsal	AlexNet	dorsal sculptum (38).JPG	fold1	5	*Amblyomma*
886	Dorsal	AlexNet	dorsal sculptum (42).JPG	fold1	5	*Amblyomma*
887	Dorsal	AlexNet	dorsal sculptum (5).JPG	fold1	5	*Amblyomma*
888	Dorsal	AlexNet	dorsal sculptum (51).JPG	fold1	5	*Amblyomma*
889	Dorsal	AlexNet	dorsal sculptum (52).JPG	fold1	5	*Amblyomma*
890	Dorsal	AlexNet	dorsal sculptum (59).JPG	fold1	5	*Amblyomma*
891	Dorsal	AlexNet	dorsal sculptum (72).JPG	fold1	5	*Amblyomma*
892	Dorsal	AlexNet	dorsal sculptum (75).JPG	fold1	5	*Amblyomma*
893	Dorsal	AlexNet	dorsal sculptum (9).JPG	fold1	5	*Amblyomma*
894	Dorsal	AlexNet	dorsal triste (19).jpg	fold1	6	*Amblyomma*
895	Dorsal	AlexNet	dorsal triste (2).jpg	fold1	6	*Amblyomma*
896	Dorsal	AlexNet	dorsal triste (20).jpg	fold1	6	*Amblyomma*
897	Dorsal	AlexNet	dorsal triste (24).jpg	fold1	6	*Amblyomma*
898	Dorsal	AlexNet	dorsal triste (25).jpg	fold1	6	*Amblyomma*
899	Dorsal	AlexNet	dorsal triste (26).jpg	fold1	6	*Amblyomma*
900	Dorsal	AlexNet	dorsal triste (45).jpg	fold1	6	*Amblyomma*
901	Dorsal	AlexNet	dorsal triste (46).jpg	fold1	6	*Amblyomma*
902	Dorsal	AlexNet	dorsal triste (47).jpg	fold1	6	*Amblyomma*
903	Dorsal	AlexNet	dorsal triste (51).jpg	fold1	6	*Amblyomma*
904	Dorsal	AlexNet	dorsal triste (6).jpg	fold1	6	*Amblyomma*
905	Dorsal	AlexNet	dorsal triste (61).jpg	fold1	6	*Amblyomma*
906	Dorsal	AlexNet	dorsal triste (63).jpg	fold1	6	*Amblyomma*
907	Dorsal	AlexNet	dorsal triste (72).jpg	fold1	6	*Amblyomma*
908	Dorsal	AlexNet	dorsal triste (74).jpg	fold1	6	*Amblyomma*
909	Dorsal	AlexNet	dorsal triste (79).jpg	fold1	6	*Amblyomma*
910	Dorsal	AlexNet	dorsal aureolatum (18).jpg	fold2	1	*Amblyomma*
911	Dorsal	AlexNet	dorsal aureolatum (21).jpg	fold2	1	*Amblyomma*
912	Dorsal	AlexNet	dorsal aureolatum (28).jpg	fold2	1	*Amblyomma*
913	Dorsal	AlexNet	dorsal aureolatum (29).jpg	fold2	1	*Amblyomma*
914	Dorsal	AlexNet	dorsal aureolatum (32).jpg	fold2	1	*Amblyomma*
915	Dorsal	AlexNet	dorsal aureolatum (38).jpg	fold2	1	*Amblyomma*
916	Dorsal	AlexNet	dorsal aureolatum (43).jpg	fold2	1	*Amblyomma*
917	Dorsal	AlexNet	dorsal aureolatum (44).jpg	fold2	1	*Amblyomma*
918	Dorsal	AlexNet	dorsal aureolatum (49).jpg	fold2	1	*Amblyomma*
919	Dorsal	AlexNet	dorsal aureolatum (5).jpg	fold2	1	*Amblyomma*
920	Dorsal	AlexNet	dorsal aureolatum (51).jpg	fold2	1	*Amblyomma*
921	Dorsal	AlexNet	dorsal aureolatum (6).jpg	fold2	1	*Amblyomma*
922	Dorsal	AlexNet	dorsal aureolatum (62).jpg	fold2	1	*Amblyomma*
923	Dorsal	AlexNet	dorsal cajennense (15).jpg	fold2	2	*Amblyomma*
924	Dorsal	AlexNet	dorsal cajennense (16).jpg	fold2	2	*Amblyomma*
925	Dorsal	AlexNet	dorsal cajennense (23).jpg	fold2	2	*Amblyomma*
926	Dorsal	AlexNet	dorsal cajennense (27).jpg	fold2	2	*Amblyomma*
927	Dorsal	AlexNet	dorsal cajennense (31).jpg	fold2	2	*Amblyomma*
928	Dorsal	AlexNet	dorsal cajennense (37).jpg	fold2	2	*Amblyomma*
929	Dorsal	AlexNet	dorsal cajennense (43).jpg	fold2	2	*Amblyomma*
930	Dorsal	AlexNet	dorsal cajennense (51).jpg	fold2	2	*Amblyomma*
931	Dorsal	AlexNet	dorsal cajennense (59).jpg	fold2	2	*Amblyomma*
932	Dorsal	AlexNet	dorsal cajennense (61).jpg	fold2	2	*Amblyomma*
933	Dorsal	AlexNet	dorsal cajennense (72).jpg	fold2	2	*Amblyomma*
934	Dorsal	AlexNet	dorsal cajennense (73).jpg	fold2	2	*Amblyomma*
935	Dorsal	AlexNet	dorsal cajennense (74).jpg	fold2	2	*Amblyomma*
936	Dorsal	AlexNet	dorsal cajennense (8).jpg	fold2	2	*Amblyomma*

937	Dorsal	AlexNet	dorsal cajennense (83).jpg	fold2	2	*Amblyomma*
938	Dorsal	AlexNet	dorsal cajennense (9).jpg	fold2	2	*Amblyomma*
939	Dorsal	AlexNet	dorsal dubitatum (14).jpg	fold2	3	*Amblyomma*
940	Dorsal	AlexNet	dorsal dubitatum (15).jpg	fold2	3	*Amblyomma*
941	Dorsal	AlexNet	dorsal dubitatum (2).jpg	fold2	3	*Amblyomma*
942	Dorsal	AlexNet	dorsal dubitatum (34).jpg	fold2	3	*Amblyomma*
943	Dorsal	AlexNet	dorsal dubitatum (36).jpg	fold2	3	*Amblyomma*
944	Dorsal	AlexNet	dorsal dubitatum (37).jpg	fold2	3	*Amblyomma*
945	Dorsal	AlexNet	dorsal dubitatum (43).jpg	fold2	3	*Amblyomma*
946	Dorsal	AlexNet	dorsal dubitatum (44).jpg	fold2	3	*Amblyomma*
947	Dorsal	AlexNet	dorsal dubitatum (47).jpg	fold2	3	*Amblyomma*
948	Dorsal	AlexNet	dorsal dubitatum (5).jpg	fold2	3	*Amblyomma*
949	Dorsal	AlexNet	dorsal dubitatum (50).jpg	fold2	3	*Amblyomma*
950	Dorsal	AlexNet	dorsal dubitatum (7).jpg	fold2	3	*Amblyomma*
951	Dorsal	AlexNet	dorsal ovale (15).jpg	fold2	4	*Amblyomma*
952	Dorsal	AlexNet	dorsal ovale (18).jpg	fold2	4	*Amblyomma*
953	Dorsal	AlexNet	dorsal ovale (22).jpg	fold2	4	*Amblyomma*
954	Dorsal	AlexNet	dorsal ovale (23).jpg	fold2	4	*Amblyomma*
955	Dorsal	AlexNet	dorsal ovale (27).jpg	fold2	4	*Amblyomma*
956	Dorsal	AlexNet	dorsal ovale (33).jpg	fold2	4	*Amblyomma*
957	Dorsal	AlexNet	dorsal ovale (38).JPG	fold2	4	*Amblyomma*
958	Dorsal	AlexNet	dorsal ovale (41).jpg	fold2	4	*Amblyomma*
959	Dorsal	AlexNet	dorsal ovale (42).jpg	fold2	4	*Amblyomma*
960	Dorsal	AlexNet	dorsal ovale (49).jpg	fold2	4	*Amblyomma*
961	Dorsal	AlexNet	dorsal ovale (5).jpg	fold2	4	*Amblyomma*
962	Dorsal	AlexNet	dorsal ovale (6).jpg	fold2	4	*Amblyomma*
963	Dorsal	AlexNet	dorsal sculptum (1).jpg	fold2	5	*Amblyomma*
964	Dorsal	AlexNet	dorsal sculptum (13).JPG	fold2	5	*Amblyomma*
965	Dorsal	AlexNet	dorsal sculptum (17).JPG	fold2	5	*Amblyomma*
966	Dorsal	AlexNet	dorsal sculptum (26).JPG	fold2	5	*Amblyomma*
967	Dorsal	AlexNet	dorsal sculptum (27).JPG	fold2	5	*Amblyomma*
968	Dorsal	AlexNet	dorsal sculptum (31).jpg	fold2	5	*Amblyomma*
969	Dorsal	AlexNet	dorsal sculptum (36).jpg	fold2	5	*Amblyomma*
970	Dorsal	AlexNet	dorsal sculptum (40).JPG	fold2	5	*Amblyomma*
971	Dorsal	AlexNet	dorsal sculptum (41).JPG	fold2	5	*Amblyomma*
972	Dorsal	AlexNet	dorsal sculptum (50).JPG	fold2	5	*Amblyomma*
973	Dorsal	AlexNet	dorsal sculptum (57).JPG	fold2	5	*Amblyomma*
974	Dorsal	AlexNet	dorsal sculptum (58).JPG	fold2	5	*Amblyomma*
975	Dorsal	AlexNet	dorsal sculptum (63).JPG	fold2	5	*Amblyomma*
976	Dorsal	AlexNet	dorsal sculptum (65).JPG	fold2	5	*Amblyomma*
977	Dorsal	AlexNet	dorsal sculptum (73).JPG	fold2	5	*Amblyomma*
978	Dorsal	AlexNet	dorsal sculptum (77).jpg	fold2	5	*Amblyomma*
979	Dorsal	AlexNet	dorsal sculptum (81).jpg	fold2	5	*Amblyomma*
980	Dorsal	AlexNet	dorsal triste (11).jpg	fold2	6	*Amblyomma*
981	Dorsal	AlexNet	dorsal triste (17).jpg	fold2	6	*Amblyomma*
982	Dorsal	AlexNet	dorsal triste (28).jpg	fold2	6	*Amblyomma*
983	Dorsal	AlexNet	dorsal triste (35).jpg	fold2	6	*Amblyomma*
984	Dorsal	AlexNet	dorsal triste (37).jpg	fold2	6	*Amblyomma*
985	Dorsal	AlexNet	dorsal triste (38).jpg	fold2	6	*Amblyomma*
986	Dorsal	AlexNet	dorsal triste (39).jpg	fold2	6	*Amblyomma*
987	Dorsal	AlexNet	dorsal triste (43).jpg	fold2	6	*Amblyomma*
988	Dorsal	AlexNet	dorsal triste (50).jpg	fold2	6	*Amblyomma*
989	Dorsal	AlexNet	dorsal triste (62).jpg	fold2	6	*Amblyomma*
990	Dorsal	AlexNet	dorsal triste (65).jpg	fold2	6	*Amblyomma*
991	Dorsal	AlexNet	dorsal triste (67).jpg	fold2	6	*Amblyomma*
992	Dorsal	AlexNet	dorsal triste (68).jpg	fold2	6	*Amblyomma*
993	Dorsal	AlexNet	dorsal triste (71).jpg	fold2	6	*Amblyomma*
994	Dorsal	AlexNet	dorsal triste (75).jpg	fold2	6	*Amblyomma*
995	Dorsal	AlexNet	dorsal triste (77).jpg	fold2	6	*Amblyomma*
996	Dorsal	AlexNet	dorsal aureolatum (1).jpg	fold3	1	*Amblyomma*
997	Dorsal	AlexNet	dorsal aureolatum (14).jpg	fold3	1	*Amblyomma*
998	Dorsal	AlexNet	dorsal aureolatum (19).jpg	fold3	1	*Amblyomma*
999	Dorsal	AlexNet	dorsal aureolatum (24).jpg	fold3	1	*Amblyomma*
1000	Dorsal	AlexNet	dorsal aureolatum (31).jpg	fold3	1	*Amblyomma*
1001	Dorsal	AlexNet	dorsal aureolatum (33).jpg	fold3	1	*Amblyomma*
1002	Dorsal	AlexNet	dorsal aureolatum (35).jpg	fold3	1	*Amblyomma*
1003	Dorsal	AlexNet	dorsal aureolatum (36).jpg	fold3	1	*Amblyomma*

1004	Dorsal	AlexNet	dorsal aureolatum (40).jpg	fold3	1	*Amblyomma*
1005	Dorsal	AlexNet	dorsal aureolatum (53).jpg	fold3	1	*Amblyomma*
1006	Dorsal	AlexNet	dorsal aureolatum (54).jpg	fold3	1	*Amblyomma*
1007	Dorsal	AlexNet	dorsal aureolatum (9).jpg	fold3	1	*Amblyomma*
1008	Dorsal	AlexNet	dorsal cajennense (10).jpg	fold3	2	*Amblyomma*
1009	Dorsal	AlexNet	dorsal cajennense (17).jpg	fold3	2	*Amblyomma*
1010	Dorsal	AlexNet	dorsal cajennense (2).jpg	fold3	2	*Amblyomma*
1011	Dorsal	AlexNet	dorsal cajennense (29).jpg	fold3	2	*Amblyomma*
1012	Dorsal	AlexNet	dorsal cajennense (35).jpg	fold3	2	*Amblyomma*
1013	Dorsal	AlexNet	dorsal cajennense (46).jpg	fold3	2	*Amblyomma*
1014	Dorsal	AlexNet	dorsal cajennense (48).jpg	fold3	2	*Amblyomma*
1015	Dorsal	AlexNet	dorsal cajennense (5).jpg	fold3	2	*Amblyomma*
1016	Dorsal	AlexNet	dorsal cajennense (53).jpg	fold3	2	*Amblyomma*
1017	Dorsal	AlexNet	dorsal cajennense (56).jpg	fold3	2	*Amblyomma*
1018	Dorsal	AlexNet	dorsal cajennense (57).jpg	fold3	2	*Amblyomma*
1019	Dorsal	AlexNet	dorsal cajennense (60).jpg	fold3	2	*Amblyomma*
1020	Dorsal	AlexNet	dorsal cajennense (62).jpg	fold3	2	*Amblyomma*
1021	Dorsal	AlexNet	dorsal cajennense (67).jpg	fold3	2	*Amblyomma*
1022	Dorsal	AlexNet	dorsal cajennense (70).jpg	fold3	2	*Amblyomma*
1023	Dorsal	AlexNet	dorsal cajennense (81).jpg	fold3	2	*Amblyomma*
1024	Dorsal	AlexNet	dorsal cajennense (82).jpg	fold3	2	*Amblyomma*
1025	Dorsal	AlexNet	dorsal dubitatum (1).jpg	fold3	3	*Amblyomma*
1026	Dorsal	AlexNet	dorsal dubitatum (11).jpg	fold3	3	*Amblyomma*
1027	Dorsal	AlexNet	dorsal dubitatum (26).jpg	fold3	3	*Amblyomma*
1028	Dorsal	AlexNet	dorsal dubitatum (27).jpg	fold3	3	*Amblyomma*
1029	Dorsal	AlexNet	dorsal dubitatum (38).jpg	fold3	3	*Amblyomma*
1030	Dorsal	AlexNet	dorsal dubitatum (39).jpg	fold3	3	*Amblyomma*
1031	Dorsal	AlexNet	dorsal dubitatum (4).jpg	fold3	3	*Amblyomma*
1032	Dorsal	AlexNet	dorsal dubitatum (41).jpg	fold3	3	*Amblyomma*
1033	Dorsal	AlexNet	dorsal dubitatum (42).jpg	fold3	3	*Amblyomma*
1034	Dorsal	AlexNet	dorsal dubitatum (48).jpg	fold3	3	*Amblyomma*
1035	Dorsal	AlexNet	dorsal dubitatum (56).jpg	fold3	3	*Amblyomma*
1036	Dorsal	AlexNet	dorsal ovale (17).jpg	fold3	4	*Amblyomma*
1037	Dorsal	AlexNet	dorsal ovale (19).jpg	fold3	4	*Amblyomma*
1038	Dorsal	AlexNet	dorsal ovale (2).jpg	fold3	4	*Amblyomma*
1039	Dorsal	AlexNet	dorsal ovale (30).jpg	fold3	4	*Amblyomma*
1040	Dorsal	AlexNet	dorsal ovale (32).jpg	fold3	4	*Amblyomma*
1041	Dorsal	AlexNet	dorsal ovale (4).jpg	fold3	4	*Amblyomma*
1042	Dorsal	AlexNet	dorsal ovale (40).jpg	fold3	4	*Amblyomma*
1043	Dorsal	AlexNet	dorsal ovale (50).jpg	fold3	4	*Amblyomma*
1044	Dorsal	AlexNet	dorsal ovale (52).jpg	fold3	4	*Amblyomma*
1045	Dorsal	AlexNet	dorsal ovale (54).JPG	fold3	4	*Amblyomma*
1046	Dorsal	AlexNet	dorsal ovale (57).jpg	fold3	4	*Amblyomma*
1047	Dorsal	AlexNet	dorsal ovale (60).jpg	fold3	4	*Amblyomma*
1048	Dorsal	AlexNet	dorsal sculptum (11).JPG	fold3	5	*Amblyomma*
1049	Dorsal	AlexNet	dorsal sculptum (12).JPG	fold3	5	*Amblyomma*
1050	Dorsal	AlexNet	dorsal sculptum (16).JPG	fold3	5	*Amblyomma*
1051	Dorsal	AlexNet	dorsal sculptum (39).JPG	fold3	5	*Amblyomma*
1052	Dorsal	AlexNet	dorsal sculptum (4).jpg	fold3	5	*Amblyomma*
1053	Dorsal	AlexNet	dorsal sculptum (43).JPG	fold3	5	*Amblyomma*
1054	Dorsal	AlexNet	dorsal sculptum (48).JPG	fold3	5	*Amblyomma*
1055	Dorsal	AlexNet	dorsal sculptum (53).JPG	fold3	5	*Amblyomma*
1056	Dorsal	AlexNet	dorsal sculptum (54).JPG	fold3	5	*Amblyomma*
1057	Dorsal	AlexNet	dorsal sculptum (56).JPG	fold3	5	*Amblyomma*
1058	Dorsal	AlexNet	dorsal sculptum (60).JPG	fold3	5	*Amblyomma*
1059	Dorsal	AlexNet	dorsal sculptum (66).JPG	fold3	5	*Amblyomma*
1060	Dorsal	AlexNet	dorsal sculptum (71).JPG	fold3	5	*Amblyomma*
1061	Dorsal	AlexNet	dorsal sculptum (74).JPG	fold3	5	*Amblyomma*
1062	Dorsal	AlexNet	dorsal sculptum (79).jpg	fold3	5	*Amblyomma*
1063	Dorsal	AlexNet	dorsal sculptum (8).JPG	fold3	5	*Amblyomma*
1064	Dorsal	AlexNet	dorsal triste (12).jpg	fold3	6	*Amblyomma*
1065	Dorsal	AlexNet	dorsal triste (13).jpg	fold3	6	*Amblyomma*
1066	Dorsal	AlexNet	dorsal triste (15).jpg	fold3	6	*Amblyomma*
1067	Dorsal	AlexNet	dorsal triste (16).jpg	fold3	6	*Amblyomma*
1068	Dorsal	AlexNet	dorsal triste (18).jpg	fold3	6	*Amblyomma*
1069	Dorsal	AlexNet	dorsal triste (21).jpg	fold3	6	*Amblyomma*
1070	Dorsal	AlexNet	dorsal triste (27).jpg	fold3	6	*Amblyomma*

1071	Dorsal	AlexNet	dorsal triste (30).jpg	fold3	6	*Amblyomma*
1072	Dorsal	AlexNet	dorsal triste (41).jpg	fold3	6	*Amblyomma*
1073	Dorsal	AlexNet	dorsal triste (49).jpg	fold3	6	*Amblyomma*
1074	Dorsal	AlexNet	dorsal triste (54).jpg	fold3	6	*Amblyomma*
1075	Dorsal	AlexNet	dorsal triste (56).jpg	fold3	6	*Amblyomma*
1076	Dorsal	AlexNet	dorsal triste (64).jpg	fold3	6	*Amblyomma*
1077	Dorsal	AlexNet	dorsal triste (66).jpg	fold3	6	*Amblyomma*
1078	Dorsal	AlexNet	dorsal triste (7).jpg	fold3	6	*Amblyomma*
1079	Dorsal	AlexNet	dorsal triste (73).jpg	fold3	6	*Amblyomma*
1080	Dorsal	AlexNet	dorsal aureolatum (12).jpg	fold4	1	*Amblyomma*
1081	Dorsal	AlexNet	dorsal aureolatum (13).jpg	fold4	1	*Amblyomma*
1082	Dorsal	AlexNet	dorsal aureolatum (15).jpg	fold4	1	*Amblyomma*
1083	Dorsal	AlexNet	dorsal aureolatum (16).jpg	fold4	1	*Amblyomma*
1084	Dorsal	AlexNet	dorsal aureolatum (20).jpg	fold4	1	*Amblyomma*
1085	Dorsal	AlexNet	dorsal aureolatum (22).jpg	fold4	1	*Amblyomma*
1086	Dorsal	AlexNet	dorsal aureolatum (25).jpg	fold4	1	*Amblyomma*
1087	Dorsal	AlexNet	dorsal aureolatum (26).jpg	fold4	1	*Amblyomma*
1088	Dorsal	AlexNet	dorsal aureolatum (37).jpg	fold4	1	*Amblyomma*
1089	Dorsal	AlexNet	dorsal aureolatum (4).jpg	fold4	1	*Amblyomma*
1090	Dorsal	AlexNet	dorsal aureolatum (56).jpg	fold4	1	*Amblyomma*
1091	Dorsal	AlexNet	dorsal aureolatum (63).jpg	fold4	1	*Amblyomma*
1092	Dorsal	AlexNet	dorsal aureolatum (7).jpg	fold4	1	*Amblyomma*
1093	Dorsal	AlexNet	dorsal cajennense (1).jpg	fold4	2	*Amblyomma*
1094	Dorsal	AlexNet	dorsal cajennense (11).jpg	fold4	2	*Amblyomma*
1095	Dorsal	AlexNet	dorsal cajennense (12).jpg	fold4	2	*Amblyomma*
1096	Dorsal	AlexNet	dorsal cajennense (13).jpg	fold4	2	*Amblyomma*
1097	Dorsal	AlexNet	dorsal cajennense (20).jpg	fold4	2	*Amblyomma*
1098	Dorsal	AlexNet	dorsal cajennense (33).jpg	fold4	2	*Amblyomma*
1099	Dorsal	AlexNet	dorsal cajennense (34).jpg	fold4	2	*Amblyomma*
1100	Dorsal	AlexNet	dorsal cajennense (36).jpg	fold4	2	*Amblyomma*
1101	Dorsal	AlexNet	dorsal cajennense (38).jpg	fold4	2	*Amblyomma*
1102	Dorsal	AlexNet	dorsal cajennense (4).jpg	fold4	2	*Amblyomma*
1103	Dorsal	AlexNet	dorsal cajennense (45).jpg	fold4	2	*Amblyomma*
1104	Dorsal	AlexNet	dorsal cajennense (6).jpg	fold4	2	*Amblyomma*
1105	Dorsal	AlexNet	dorsal cajennense (63).jpg	fold4	2	*Amblyomma*
1106	Dorsal	AlexNet	dorsal cajennense (64).jpg	fold4	2	*Amblyomma*
1107	Dorsal	AlexNet	dorsal cajennense (76).jpg	fold4	2	*Amblyomma*
1108	Dorsal	AlexNet	dorsal cajennense (78).jpg	fold4	2	*Amblyomma*
1109	Dorsal	AlexNet	dorsal cajennense (79).jpg	fold4	2	*Amblyomma*
1110	Dorsal	AlexNet	dorsal dubitatum (12).jpg	fold4	3	*Amblyomma*
1111	Dorsal	AlexNet	dorsal dubitatum (13).jpg	fold4	3	*Amblyomma*
1112	Dorsal	AlexNet	dorsal dubitatum (17).jpg	fold4	3	*Amblyomma*
1113	Dorsal	AlexNet	dorsal dubitatum (20).jpg	fold4	3	*Amblyomma*
1114	Dorsal	AlexNet	dorsal dubitatum (21).jpg	fold4	3	*Amblyomma*
1115	Dorsal	AlexNet	dorsal dubitatum (22).jpg	fold4	3	*Amblyomma*
1116	Dorsal	AlexNet	dorsal dubitatum (3).jpg	fold4	3	*Amblyomma*
1117	Dorsal	AlexNet	dorsal dubitatum (31).jpg	fold4	3	*Amblyomma*
1118	Dorsal	AlexNet	dorsal dubitatum (35).jpg	fold4	3	*Amblyomma*
1119	Dorsal	AlexNet	dorsal dubitatum (54).jpg	fold4	3	*Amblyomma*
1120	Dorsal	AlexNet	dorsal dubitatum (8).JPG	fold4	3	*Amblyomma*
1121	Dorsal	AlexNet	dorsal ovale (1).jpg	fold4	4	*Amblyomma*
1122	Dorsal	AlexNet	dorsal ovale (13).jpg	fold4	4	*Amblyomma*
1123	Dorsal	AlexNet	dorsal ovale (16).jpg	fold4	4	*Amblyomma*
1124	Dorsal	AlexNet	dorsal ovale (24).jpg	fold4	4	*Amblyomma*
1125	Dorsal	AlexNet	dorsal ovale (25).jpg	fold4	4	*Amblyomma*
1126	Dorsal	AlexNet	dorsal ovale (31).jpg	fold4	4	*Amblyomma*
1127	Dorsal	AlexNet	dorsal ovale (34).jpg	fold4	4	*Amblyomma*
1128	Dorsal	AlexNet	dorsal ovale (36).jpg	fold4	4	*Amblyomma*
1129	Dorsal	AlexNet	dorsal ovale (37).jpg	fold4	4	*Amblyomma*
1130	Dorsal	AlexNet	dorsal ovale (39).jpg	fold4	4	*Amblyomma*
1131	Dorsal	AlexNet	dorsal ovale (55).jpg	fold4	4	*Amblyomma*
1132	Dorsal	AlexNet	dorsal ovale (9).jpg	fold4	4	*Amblyomma*
1133	Dorsal	AlexNet	dorsal sculptum (15).JPG	fold4	5	*Amblyomma*
1134	Dorsal	AlexNet	dorsal sculptum (19).JPG	fold4	5	*Amblyomma*
1135	Dorsal	AlexNet	dorsal sculptum (2).jpg	fold4	5	*Amblyomma*
1136	Dorsal	AlexNet	dorsal sculptum (24).JPG	fold4	5	*Amblyomma*
1137	Dorsal	AlexNet	dorsal sculptum (30).jpg	fold4	5	*Amblyomma*

1138	Dorsal	AlexNet	dorsal sculptum (34).jpg	fold4	5	*Amblyomma*
1139	Dorsal	AlexNet	dorsal sculptum (35).jpg	fold4	5	*Amblyomma*
1140	Dorsal	AlexNet	dorsal sculptum (44).JPG	fold4	5	*Amblyomma*
1141	Dorsal	AlexNet	dorsal sculptum (45).JPG	fold4	5	*Amblyomma*
1142	Dorsal	AlexNet	dorsal sculptum (49).JPG	fold4	5	*Amblyomma*
1143	Dorsal	AlexNet	dorsal sculptum (55).JPG	fold4	5	*Amblyomma*
1144	Dorsal	AlexNet	dorsal sculptum (61).JPG	fold4	5	*Amblyomma*
1145	Dorsal	AlexNet	dorsal sculptum (62).JPG	fold4	5	*Amblyomma*
1146	Dorsal	AlexNet	dorsal sculptum (64).JPG	fold4	5	*Amblyomma*
1147	Dorsal	AlexNet	dorsal sculptum (69).JPG	fold4	5	*Amblyomma*
1148	Dorsal	AlexNet	dorsal sculptum (80).jpg	fold4	5	*Amblyomma*
1149	Dorsal	AlexNet	dorsal triste (10).jpg	fold4	6	*Amblyomma*
1150	Dorsal	AlexNet	dorsal triste (23).jpg	fold4	6	*Amblyomma*
1151	Dorsal	AlexNet	dorsal triste (29).jpg	fold4	6	*Amblyomma*
1152	Dorsal	AlexNet	dorsal triste (31).jpg	fold4	6	*Amblyomma*
1153	Dorsal	AlexNet	dorsal triste (32).jpg	fold4	6	*Amblyomma*
1154	Dorsal	AlexNet	dorsal triste (34).jpg	fold4	6	*Amblyomma*
1155	Dorsal	AlexNet	dorsal triste (4).jpg	fold4	6	*Amblyomma*
1156	Dorsal	AlexNet	dorsal triste (40).jpg	fold4	6	*Amblyomma*
1157	Dorsal	AlexNet	dorsal triste (5).jpg	fold4	6	*Amblyomma*
1158	Dorsal	AlexNet	dorsal triste (53).jpg	fold4	6	*Amblyomma*
1159	Dorsal	AlexNet	dorsal triste (55).jpg	fold4	6	*Amblyomma*
1160	Dorsal	AlexNet	dorsal triste (69).jpg	fold4	6	*Amblyomma*
1161	Dorsal	AlexNet	dorsal triste (70).jpg	fold4	6	*Amblyomma*
1162	Dorsal	AlexNet	dorsal triste (76).jpg	fold4	6	*Amblyomma*
1163	Dorsal	AlexNet	dorsal triste (8).jpg	fold4	6	*Amblyomma*
1164	Dorsal	AlexNet	dorsal aureolatum (10).jpg	fold5	1	*Amblyomma*
1165	Dorsal	AlexNet	dorsal aureolatum (2).jpg	fold5	1	*Amblyomma*
1166	Dorsal	AlexNet	dorsal aureolatum (3).jpg	fold5	1	*Amblyomma*
1167	Dorsal	AlexNet	dorsal aureolatum (34).jpg	fold5	1	*Amblyomma*
1168	Dorsal	AlexNet	dorsal aureolatum (39).jpg	fold5	1	*Amblyomma*
1169	Dorsal	AlexNet	dorsal aureolatum (41).jpg	fold5	1	*Amblyomma*
1170	Dorsal	AlexNet	dorsal aureolatum (42).jpg	fold5	1	*Amblyomma*
1171	Dorsal	AlexNet	dorsal aureolatum (45).jpg	fold5	1	*Amblyomma*
1172	Dorsal	AlexNet	dorsal aureolatum (47).jpg	fold5	1	*Amblyomma*
1173	Dorsal	AlexNet	dorsal aureolatum (50).jpg	fold5	1	*Amblyomma*
1174	Dorsal	AlexNet	dorsal aureolatum (57).jpg	fold5	1	*Amblyomma*
1175	Dorsal	AlexNet	dorsal aureolatum (59).jpg	fold5	1	*Amblyomma*
1176	Dorsal	AlexNet	dorsal aureolatum (60).jpg	fold5	1	*Amblyomma*
1177	Dorsal	AlexNet	dorsal cajennense (14).jpg	fold5	2	*Amblyomma*
1178	Dorsal	AlexNet	dorsal cajennense (18).jpg	fold5	2	*Amblyomma*
1179	Dorsal	AlexNet	dorsal cajennense (19).jpg	fold5	2	*Amblyomma*
1180	Dorsal	AlexNet	dorsal cajennense (21).jpg	fold5	2	*Amblyomma*
1181	Dorsal	AlexNet	dorsal cajennense (22).jpg	fold5	2	*Amblyomma*
1182	Dorsal	AlexNet	dorsal cajennense (24).jpg	fold5	2	*Amblyomma*
1183	Dorsal	AlexNet	dorsal cajennense (28).jpg	fold5	2	*Amblyomma*
1184	Dorsal	AlexNet	dorsal cajennense (30).jpg	fold5	2	*Amblyomma*
1185	Dorsal	AlexNet	dorsal cajennense (39).jpg	fold5	2	*Amblyomma*
1186	Dorsal	AlexNet	dorsal cajennense (40).jpg	fold5	2	*Amblyomma*
1187	Dorsal	AlexNet	dorsal cajennense (41).jpg	fold5	2	*Amblyomma*
1188	Dorsal	AlexNet	dorsal cajennense (42).jpg	fold5	2	*Amblyomma*
1189	Dorsal	AlexNet	dorsal cajennense (49).jpg	fold5	2	*Amblyomma*
1190	Dorsal	AlexNet	dorsal cajennense (55).jpg	fold5	2	*Amblyomma*
1191	Dorsal	AlexNet	dorsal cajennense (68).jpg	fold5	2	*Amblyomma*
1192	Dorsal	AlexNet	dorsal cajennense (69).jpg	fold5	2	*Amblyomma*
1193	Dorsal	AlexNet	dorsal cajennense (71).jpg	fold5	2	*Amblyomma*
1194	Dorsal	AlexNet	dorsal dubitatum (16).jpg	fold5	3	*Amblyomma*
1195	Dorsal	AlexNet	dorsal dubitatum (23).jpg	fold5	3	*Amblyomma*
1196	Dorsal	AlexNet	dorsal dubitatum (24).jpg	fold5	3	*Amblyomma*
1197	Dorsal	AlexNet	dorsal dubitatum (25).jpg	fold5	3	*Amblyomma*
1198	Dorsal	AlexNet	dorsal dubitatum (29).jpg	fold5	3	*Amblyomma*
1199	Dorsal	AlexNet	dorsal dubitatum (30).jpg	fold5	3	*Amblyomma*
1200	Dorsal	AlexNet	dorsal dubitatum (33).jpg	fold5	3	*Amblyomma*
1201	Dorsal	AlexNet	dorsal dubitatum (45).jpg	fold5	3	*Amblyomma*
1202	Dorsal	AlexNet	dorsal dubitatum (51).jpg	fold5	3	*Amblyomma*
1203	Dorsal	AlexNet	dorsal dubitatum (52).jpg	fold5	3	*Amblyomma*
1204	Dorsal	AlexNet	dorsal dubitatum (53).jpg	fold5	3	*Amblyomma*

1205	Dorsal	AlexNet	dorsal ovale (11).jpg	fold5	4	*Amblyomma*
1206	Dorsal	AlexNet	dorsal ovale (12).jpg	fold5	4	*Amblyomma*
1207	Dorsal	AlexNet	dorsal ovale (28).jpg	fold5	4	*Amblyomma*
1208	Dorsal	AlexNet	dorsal ovale (3).jpg	fold5	4	*Amblyomma*
1209	Dorsal	AlexNet	dorsal ovale (35).jpg	fold5	4	*Amblyomma*
1210	Dorsal	AlexNet	dorsal ovale (43).jpg	fold5	4	*Amblyomma*
1211	Dorsal	AlexNet	dorsal ovale (45).jpg	fold5	4	*Amblyomma*
1212	Dorsal	AlexNet	dorsal ovale (46).jpg	fold5	4	*Amblyomma*
1213	Dorsal	AlexNet	dorsal ovale (47).jpg	fold5	4	*Amblyomma*
1214	Dorsal	AlexNet	dorsal ovale (56).jpg	fold5	4	*Amblyomma*
1215	Dorsal	AlexNet	dorsal ovale (58).jpg	fold5	4	*Amblyomma*
1216	Dorsal	AlexNet	dorsal ovale (59).jpg	fold5	4	*Amblyomma*
1217	Dorsal	AlexNet	dorsal ovale (8).jpg	fold5	4	*Amblyomma*
1218	Dorsal	AlexNet	dorsal sculptum (10).JPG	fold5	5	*Amblyomma*
1219	Dorsal	AlexNet	dorsal sculptum (18).JPG	fold5	5	*Amblyomma*
1220	Dorsal	AlexNet	dorsal sculptum (21).JPG	fold5	5	*Amblyomma*
1221	Dorsal	AlexNet	dorsal sculptum (28).JPG	fold5	5	*Amblyomma*
1222	Dorsal	AlexNet	dorsal sculptum (3).jpg	fold5	5	*Amblyomma*
1223	Dorsal	AlexNet	dorsal sculptum (32).jpg	fold5	5	*Amblyomma*
1224	Dorsal	AlexNet	dorsal sculptum (33).jpg	fold5	5	*Amblyomma*
1225	Dorsal	AlexNet	dorsal sculptum (46).JPG	fold5	5	*Amblyomma*
1226	Dorsal	AlexNet	dorsal sculptum (47).JPG	fold5	5	*Amblyomma*
1227	Dorsal	AlexNet	dorsal sculptum (6).JPG	fold5	5	*Amblyomma*
1228	Dorsal	AlexNet	dorsal sculptum (67).JPG	fold5	5	*Amblyomma*
1229	Dorsal	AlexNet	dorsal sculptum (68).JPG	fold5	5	*Amblyomma*
1230	Dorsal	AlexNet	dorsal sculptum (7).JPG	fold5	5	*Amblyomma*
1231	Dorsal	AlexNet	dorsal sculptum (70).JPG	fold5	5	*Amblyomma*
1232	Dorsal	AlexNet	dorsal sculptum (76).jpg	fold5	5	*Amblyomma*
1233	Dorsal	AlexNet	dorsal sculptum (78).jpg	fold5	5	*Amblyomma*
1234	Dorsal	AlexNet	dorsal triste (1).jpg	fold5	6	*Amblyomma*
1235	Dorsal	AlexNet	dorsal triste (14).jpg	fold5	6	*Amblyomma*
1236	Dorsal	AlexNet	dorsal triste (22).jpg	fold5	6	*Amblyomma*
1237	Dorsal	AlexNet	dorsal triste (3).jpg	fold5	6	*Amblyomma*
1238	Dorsal	AlexNet	dorsal triste (33).jpg	fold5	6	*Amblyomma*
1239	Dorsal	AlexNet	dorsal triste (36).jpg	fold5	6	*Amblyomma*
1240	Dorsal	AlexNet	dorsal triste (42).jpg	fold5	6	*Amblyomma*
1241	Dorsal	AlexNet	dorsal triste (44).jpg	fold5	6	*Amblyomma*
1242	Dorsal	AlexNet	dorsal triste (48).jpg	fold5	6	*Amblyomma*
1243	Dorsal	AlexNet	dorsal triste (52).jpg	fold5	6	*Amblyomma*
1244	Dorsal	AlexNet	dorsal triste (57).jpg	fold5	6	*Amblyomma*
1245	Dorsal	AlexNet	dorsal triste (58).jpg	fold5	6	*Amblyomma*
1246	Dorsal	AlexNet	dorsal triste (59).jpg	fold5	6	*Amblyomma*
1247	Dorsal	AlexNet	dorsal triste (60).jpg	fold5	6	*Amblyomma*
1248	Dorsal	AlexNet	dorsal triste (78).jpg	fold5	6	*Amblyomma*
1249	Dorsal	AlexNet	dorsal triste (9).jpg	fold5	6	*Amblyomma*
1250	Ventral	AlexNet	ventral aureolatum (10).jpg	fold1	1	*Amblyomma*
1251	Ventral	AlexNet	ventral aureolatum (16).jpg	fold1	1	*Amblyomma*
1252	Ventral	AlexNet	ventral aureolatum (20).jpg	fold1	1	*Amblyomma*
1253	Ventral	AlexNet	ventral aureolatum (21).jpg	fold1	1	*Amblyomma*
1254	Ventral	AlexNet	ventral aureolatum (28).jpg	fold1	1	*Amblyomma*
1255	Ventral	AlexNet	ventral aureolatum (3).jpg	fold1	1	*Amblyomma*
1256	Ventral	AlexNet	ventral aureolatum (31).jpg	fold1	1	*Amblyomma*
1257	Ventral	AlexNet	ventral aureolatum (33).jpg	fold1	1	*Amblyomma*
1258	Ventral	AlexNet	ventral aureolatum (34).jpg	fold1	1	*Amblyomma*
1259	Ventral	AlexNet	ventral aureolatum (40).jpg	fold1	1	*Amblyomma*
1260	Ventral	AlexNet	ventral aureolatum (44).jpg	fold1	1	*Amblyomma*
1261	Ventral	AlexNet	ventral aureolatum (58).jpg	fold1	1	*Amblyomma*
1262	Ventral	AlexNet	ventral cajennense (14).jpg	fold1	2	*Amblyomma*
1263	Ventral	AlexNet	ventral cajennense (16).jpg	fold1	2	*Amblyomma*
1264	Ventral	AlexNet	ventral cajennense (28).jpg	fold1	2	*Amblyomma*
1265	Ventral	AlexNet	ventral cajennense (33).jpg	fold1	2	*Amblyomma*
1266	Ventral	AlexNet	ventral cajennense (4).jpg	fold1	2	*Amblyomma*
1267	Ventral	AlexNet	ventral cajennense (41).jpg	fold1	2	*Amblyomma*
1268	Ventral	AlexNet	ventral cajennense (5).jpg	fold1	2	*Amblyomma*
1269	Ventral	AlexNet	ventral cajennense (51).jpg	fold1	2	*Amblyomma*
1270	Ventral	AlexNet	ventral cajennense (57).jpg	fold1	2	*Amblyomma*
1271	Ventral	AlexNet	ventral cajennense (60).jpg	fold1	2	*Amblyomma*

1272	Ventral	AlexNet	ventral cajennense (64).jpg	fold1	2	*Amblyomma*
1273	Ventral	AlexNet	ventral cajennense (7).jpg	fold1	2	*Amblyomma*
1274	Ventral	AlexNet	ventral cajennense (72).jpg	fold1	2	*Amblyomma*
1275	Ventral	AlexNet	ventral cajennense (74).jpg	fold1	2	*Amblyomma*
1276	Ventral	AlexNet	ventral cajennense (75).jpg	fold1	2	*Amblyomma*
1277	Ventral	AlexNet	ventral cajennense (82).jpg	fold1	2	*Amblyomma*
1278	Ventral	AlexNet	ventral dubitatum (22).jpg	fold1	3	*Amblyomma*
1279	Ventral	AlexNet	ventral dubitatum (26).jpg	fold1	3	*Amblyomma*
1280	Ventral	AlexNet	ventral dubitatum (27).jpg	fold1	3	*Amblyomma*
1281	Ventral	AlexNet	ventral dubitatum (30).jpg	fold1	3	*Amblyomma*
1282	Ventral	AlexNet	ventral dubitatum (39).jpg	fold1	3	*Amblyomma*
1283	Ventral	AlexNet	ventral dubitatum (40).jpg	fold1	3	*Amblyomma*
1284	Ventral	AlexNet	ventral dubitatum (45).jpg	fold1	3	*Amblyomma*
1285	Ventral	AlexNet	ventral dubitatum (46).jpg	fold1	3	*Amblyomma*
1286	Ventral	AlexNet	ventral dubitatum (51).jpg	fold1	3	*Amblyomma*
1287	Ventral	AlexNet	ventral dubitatum (7).jpg	fold1	3	*Amblyomma*
1288	Ventral	AlexNet	ventral ovale (1).jpg	fold1	4	*Amblyomma*
1289	Ventral	AlexNet	ventral ovale (18).jpg	fold1	4	*Amblyomma*
1290	Ventral	AlexNet	ventral ovale (20).jpg	fold1	4	*Amblyomma*
1291	Ventral	AlexNet	ventral ovale (22).jpg	fold1	4	*Amblyomma*
1292	Ventral	AlexNet	ventral ovale (26).jpg	fold1	4	*Amblyomma*
1293	Ventral	AlexNet	ventral ovale (31).jpg	fold1	4	*Amblyomma*
1294	Ventral	AlexNet	ventral ovale (32).jpg	fold1	4	*Amblyomma*
1295	Ventral	AlexNet	ventral ovale (36).jpg	fold1	4	*Amblyomma*
1296	Ventral	AlexNet	ventral ovale (38).JPG	fold1	4	*Amblyomma*
1297	Ventral	AlexNet	ventral ovale (57).jpg	fold1	4	*Amblyomma*
1298	Ventral	AlexNet	ventral ovale (58).jpg	fold1	4	*Amblyomma*
1299	Ventral	AlexNet	ventral ovale (60).jpg	fold1	4	*Amblyomma*
1300	Ventral	AlexNet	ventral sculptum (10).JPG	fold1	5	*Amblyomma*
1301	Ventral	AlexNet	ventral sculptum (18).JPG	fold1	5	*Amblyomma*
1302	Ventral	AlexNet	ventral sculptum (2).jpg	fold1	5	*Amblyomma*
1303	Ventral	AlexNet	ventral sculptum (28).jpg	fold1	5	*Amblyomma*
1304	Ventral	AlexNet	ventral sculptum (29).jpg	fold1	5	*Amblyomma*
1305	Ventral	AlexNet	ventral sculptum (31).jpg	fold1	5	*Amblyomma*
1306	Ventral	AlexNet	ventral sculptum (32).jpg	fold1	5	*Amblyomma*
1307	Ventral	AlexNet	ventral sculptum (38).JPG	fold1	5	*Amblyomma*
1308	Ventral	AlexNet	ventral sculptum (40).JPG	fold1	5	*Amblyomma*
1309	Ventral	AlexNet	ventral sculptum (43).JPG	fold1	5	*Amblyomma*
1310	Ventral	AlexNet	ventral sculptum (45).JPG	fold1	5	*Amblyomma*
1311	Ventral	AlexNet	ventral sculptum (54).JPG	fold1	5	*Amblyomma*
1312	Ventral	AlexNet	ventral sculptum (6).JPG	fold1	5	*Amblyomma*
1313	Ventral	AlexNet	ventral sculptum (60).JPG	fold1	5	*Amblyomma*
1314	Ventral	AlexNet	ventral sculptum (67).JPG	fold1	5	*Amblyomma*
1315	Ventral	AlexNet	ventral triste (11).jpg	fold1	6	*Amblyomma*
1316	Ventral	AlexNet	ventral triste (16).jpg	fold1	6	*Amblyomma*
1317	Ventral	AlexNet	ventral triste (33).jpg	fold1	6	*Amblyomma*
1318	Ventral	AlexNet	ventral triste (37).jpg	fold1	6	*Amblyomma*
1319	Ventral	AlexNet	ventral triste (38).jpg	fold1	6	*Amblyomma*
1320	Ventral	AlexNet	ventral triste (39).jpg	fold1	6	*Amblyomma*
1321	Ventral	AlexNet	ventral triste (41).jpg	fold1	6	*Amblyomma*
1322	Ventral	AlexNet	ventral triste (42).jpg	fold1	6	*Amblyomma*
1323	Ventral	AlexNet	ventral triste (47).jpg	fold1	6	*Amblyomma*
1324	Ventral	AlexNet	ventral triste (66).jpg	fold1	6	*Amblyomma*
1325	Ventral	AlexNet	ventral triste (67).jpg	fold1	6	*Amblyomma*
1326	Ventral	AlexNet	ventral triste (68).jpg	fold1	6	*Amblyomma*
1327	Ventral	AlexNet	ventral triste (72).jpg	fold1	6	*Amblyomma*
1328	Ventral	AlexNet	ventral triste (75).jpg	fold1	6	*Amblyomma*
1329	Ventral	AlexNet	ventral triste (76).jpg	fold1	6	*Amblyomma*
1330	Ventral	AlexNet	ventral triste (77).jpg	fold1	6	*Amblyomma*
1331	Ventral	AlexNet	ventral aureolatum (12).jpg	fold2	1	*Amblyomma*
1332	Ventral	AlexNet	ventral aureolatum (18).jpg	fold2	1	*Amblyomma*
1333	Ventral	AlexNet	ventral aureolatum (22).jpg	fold2	1	*Amblyomma*
1334	Ventral	AlexNet	ventral aureolatum (26).jpg	fold2	1	*Amblyomma*
1335	Ventral	AlexNet	ventral aureolatum (27).jpg	fold2	1	*Amblyomma*
1336	Ventral	AlexNet	ventral aureolatum (4).jpg	fold2	1	*Amblyomma*
1337	Ventral	AlexNet	ventral aureolatum (47).jpg	fold2	1	*Amblyomma*
1338	Ventral	AlexNet	ventral aureolatum (48).jpg	fold2	1	*Amblyomma*

1339	Ventral	AlexNet	ventral aureolatum (49).jpg	fold2	1	*Amblyomma*
1340	Ventral	AlexNet	ventral aureolatum (50).jpg	fold2	1	*Amblyomma*
1341	Ventral	AlexNet	ventral aureolatum (51).jpg	fold2	1	*Amblyomma*
1342	Ventral	AlexNet	ventral aureolatum (56).jpg	fold2	1	*Amblyomma*
1343	Ventral	AlexNet	ventral cajennense (1).jpg	fold2	2	*Amblyomma*
1344	Ventral	AlexNet	ventral cajennense (15).jpg	fold2	2	*Amblyomma*
1345	Ventral	AlexNet	ventral cajennense (20).jpg	fold2	2	*Amblyomma*
1346	Ventral	AlexNet	ventral cajennense (23).jpg	fold2	2	*Amblyomma*
1347	Ventral	AlexNet	ventral cajennense (29).jpg	fold2	2	*Amblyomma*
1348	Ventral	AlexNet	ventral cajennense (30).jpg	fold2	2	*Amblyomma*
1349	Ventral	AlexNet	ventral cajennense (37).jpg	fold2	2	*Amblyomma*
1350	Ventral	AlexNet	ventral cajennense (49).jpg	fold2	2	*Amblyomma*
1351	Ventral	AlexNet	ventral cajennense (52).jpg	fold2	2	*Amblyomma*
1352	Ventral	AlexNet	ventral cajennense (56).jpg	fold2	2	*Amblyomma*
1353	Ventral	AlexNet	ventral cajennense (63).jpg	fold2	2	*Amblyomma*
1354	Ventral	AlexNet	ventral cajennense (65).jpg	fold2	2	*Amblyomma*
1355	Ventral	AlexNet	ventral cajennense (67).jpg	fold2	2	*Amblyomma*
1356	Ventral	AlexNet	ventral cajennense (70).jpg	fold2	2	*Amblyomma*
1357	Ventral	AlexNet	ventral cajennense (71).jpg	fold2	2	*Amblyomma*
1358	Ventral	AlexNet	ventral cajennense (9).jpg	fold2	2	*Amblyomma*
1359	Ventral	AlexNet	ventral dubitatum (19).jpg	fold2	3	*Amblyomma*
1360	Ventral	AlexNet	ventral dubitatum (23).jpg	fold2	3	*Amblyomma*
1361	Ventral	AlexNet	ventral dubitatum (24).jpg	fold2	3	*Amblyomma*
1362	Ventral	AlexNet	ventral dubitatum (25).jpg	fold2	3	*Amblyomma*
1363	Ventral	AlexNet	ventral dubitatum (28).jpg	fold2	3	*Amblyomma*
1364	Ventral	AlexNet	ventral dubitatum (36).jpg	fold2	3	*Amblyomma*
1365	Ventral	AlexNet	ventral dubitatum (38).jpg	fold2	3	*Amblyomma*
1366	Ventral	AlexNet	ventral dubitatum (41).jpg	fold2	3	*Amblyomma*
1367	Ventral	AlexNet	ventral dubitatum (42).jpg	fold2	3	*Amblyomma*
1368	Ventral	AlexNet	ventral dubitatum (48).jpg	fold2	3	*Amblyomma*
1369	Ventral	AlexNet	ventral dubitatum (49).jpg	fold2	3	*Amblyomma*
1370	Ventral	AlexNet	ventral ovale (10).jpg	fold2	4	*Amblyomma*
1371	Ventral	AlexNet	ventral ovale (13).jpg	fold2	4	*Amblyomma*
1372	Ventral	AlexNet	ventral ovale (14).jpg	fold2	4	*Amblyomma*
1373	Ventral	AlexNet	ventral ovale (28).jpg	fold2	4	*Amblyomma*
1374	Ventral	AlexNet	ventral ovale (29).jpg	fold2	4	*Amblyomma*
1375	Ventral	AlexNet	ventral ovale (3).jpg	fold2	4	*Amblyomma*
1376	Ventral	AlexNet	ventral ovale (42).jpg	fold2	4	*Amblyomma*
1377	Ventral	AlexNet	ventral ovale (44).jpg	fold2	4	*Amblyomma*
1378	Ventral	AlexNet	ventral ovale (49).jpg	fold2	4	*Amblyomma*
1379	Ventral	AlexNet	ventral ovale (51).jpg	fold2	4	*Amblyomma*
1380	Ventral	AlexNet	ventral ovale (8).jpg	fold2	4	*Amblyomma*
1381	Ventral	AlexNet	ventral ovale (9).jpg	fold2	4	*Amblyomma*
1382	Ventral	AlexNet	ventral sculptum (1).jpg	fold2	5	*Amblyomma*
1383	Ventral	AlexNet	ventral sculptum (12).JPG	fold2	5	*Amblyomma*
1384	Ventral	AlexNet	ventral sculptum (23).JPG	fold2	5	*Amblyomma*
1385	Ventral	AlexNet	ventral sculptum (30).jpg	fold2	5	*Amblyomma*
1386	Ventral	AlexNet	ventral sculptum (39).JPG	fold2	5	*Amblyomma*
1387	Ventral	AlexNet	ventral sculptum (4).jpg	fold2	5	*Amblyomma*
1388	Ventral	AlexNet	ventral sculptum (46).JPG	fold2	5	*Amblyomma*
1389	Ventral	AlexNet	ventral sculptum (48).JPG	fold2	5	*Amblyomma*
1390	Ventral	AlexNet	ventral sculptum (51).JPG	fold2	5	*Amblyomma*
1391	Ventral	AlexNet	ventral sculptum (53).JPG	fold2	5	*Amblyomma*
1392	Ventral	AlexNet	ventral sculptum (58).JPG	fold2	5	*Amblyomma*
1393	Ventral	AlexNet	ventral sculptum (63).JPG	fold2	5	*Amblyomma*
1394	Ventral	AlexNet	ventral sculptum (7).JPG	fold2	5	*Amblyomma*
1395	Ventral	AlexNet	ventral sculptum (9).JPG	fold2	5	*Amblyomma*
1396	Ventral	AlexNet	ventral triste (12).jpg	fold2	6	*Amblyomma*
1397	Ventral	AlexNet	ventral triste (2).jpg	fold2	6	*Amblyomma*
1398	Ventral	AlexNet	ventral triste (21).jpg	fold2	6	*Amblyomma*
1399	Ventral	AlexNet	ventral triste (30).jpg	fold2	6	*Amblyomma*
1400	Ventral	AlexNet	ventral triste (40).jpg	fold2	6	*Amblyomma*
1401	Ventral	AlexNet	ventral triste (43).jpg	fold2	6	*Amblyomma*
1402	Ventral	AlexNet	ventral triste (45).jpg	fold2	6	*Amblyomma*
1403	Ventral	AlexNet	ventral triste (48).jpg	fold2	6	*Amblyomma*
1404	Ventral	AlexNet	ventral triste (49).jpg	fold2	6	*Amblyomma*
1405	Ventral	AlexNet	ventral triste (52).jpg	fold2	6	*Amblyomma*

1406	Ventral	AlexNet	ventral triste (53).jpg	fold2	6	*Amblyomma*
1407	Ventral	AlexNet	ventral triste (54).jpg	fold2	6	*Amblyomma*
1408	Ventral	AlexNet	ventral triste (56).jpg	fold2	6	*Amblyomma*
1409	Ventral	AlexNet	ventral triste (57).jpg	fold2	6	*Amblyomma*
1410	Ventral	AlexNet	ventral triste (8).jpg	fold2	6	*Amblyomma*
1411	Ventral	AlexNet	ventral aureolatum (11).jpg	fold3	1	*Amblyomma*
1412	Ventral	AlexNet	ventral aureolatum (17).jpg	fold3	1	*Amblyomma*
1413	Ventral	AlexNet	ventral aureolatum (19).jpg	fold3	1	*Amblyomma*
1414	Ventral	AlexNet	ventral aureolatum (36).jpg	fold3	1	*Amblyomma*
1415	Ventral	AlexNet	ventral aureolatum (38).jpg	fold3	1	*Amblyomma*
1416	Ventral	AlexNet	ventral aureolatum (39).jpg	fold3	1	*Amblyomma*
1417	Ventral	AlexNet	ventral aureolatum (42).jpg	fold3	1	*Amblyomma*
1418	Ventral	AlexNet	ventral aureolatum (5).jpg	fold3	1	*Amblyomma*
1419	Ventral	AlexNet	ventral aureolatum (53).jpg	fold3	1	*Amblyomma*
1420	Ventral	AlexNet	ventral aureolatum (7).jpg	fold3	1	*Amblyomma*
1421	Ventral	AlexNet	ventral aureolatum (9).jpg	fold3	1	*Amblyomma*
1422	Ventral	AlexNet	ventral cajennense (11).jpg	fold3	2	*Amblyomma*
1423	Ventral	AlexNet	ventral cajennense (12).jpg	fold3	2	*Amblyomma*
1424	Ventral	AlexNet	ventral cajennense (22).jpg	fold3	2	*Amblyomma*
1425	Ventral	AlexNet	ventral cajennense (3).jpg	fold3	2	*Amblyomma*
1426	Ventral	AlexNet	ventral cajennense (31).jpg	fold3	2	*Amblyomma*
1427	Ventral	AlexNet	ventral cajennense (35).jpg	fold3	2	*Amblyomma*
1428	Ventral	AlexNet	ventral cajennense (36).jpg	fold3	2	*Amblyomma*
1429	Ventral	AlexNet	ventral cajennense (38).jpg	fold3	2	*Amblyomma*
1430	Ventral	AlexNet	ventral cajennense (39).jpg	fold3	2	*Amblyomma*
1431	Ventral	AlexNet	ventral cajennense (45).jpg	fold3	2	*Amblyomma*
1432	Ventral	AlexNet	ventral cajennense (47).jpg	fold3	2	*Amblyomma*
1433	Ventral	AlexNet	ventral cajennense (54).jpg	fold3	2	*Amblyomma*
1434	Ventral	AlexNet	ventral cajennense (61).jpg	fold3	2	*Amblyomma*
1435	Ventral	AlexNet	ventral cajennense (69).jpg	fold3	2	*Amblyomma*
1436	Ventral	AlexNet	ventral cajennense (78).jpg	fold3	2	*Amblyomma*
1437	Ventral	AlexNet	ventral cajennense (81).jpg	fold3	2	*Amblyomma*
1438	Ventral	AlexNet	ventral dubitatum (11).jpg	fold3	3	*Amblyomma*
1439	Ventral	AlexNet	ventral dubitatum (15).jpg	fold3	3	*Amblyomma*
1440	Ventral	AlexNet	ventral dubitatum (2).jpg	fold3	3	*Amblyomma*
1441	Ventral	AlexNet	ventral dubitatum (33).jpg	fold3	3	*Amblyomma*
1442	Ventral	AlexNet	ventral dubitatum (34).jpg	fold3	3	*Amblyomma*
1443	Ventral	AlexNet	ventral dubitatum (43).jpg	fold3	3	*Amblyomma*
1444	Ventral	AlexNet	ventral dubitatum (5).JPG	fold3	3	*Amblyomma*
1445	Ventral	AlexNet	ventral dubitatum (50).jpg	fold3	3	*Amblyomma*
1446	Ventral	AlexNet	ventral dubitatum (52).jpg	fold3	3	*Amblyomma*
1447	Ventral	AlexNet	ventral dubitatum (8).jpg	fold3	3	*Amblyomma*
1448	Ventral	AlexNet	ventral ovale (12).jpg	fold3	4	*Amblyomma*
1449	Ventral	AlexNet	ventral ovale (16).jpg	fold3	4	*Amblyomma*
1450	Ventral	AlexNet	ventral ovale (2).jpg	fold3	4	*Amblyomma*
1451	Ventral	AlexNet	ventral ovale (33).jpg	fold3	4	*Amblyomma*
1452	Ventral	AlexNet	ventral ovale (35).jpg	fold3	4	*Amblyomma*
1453	Ventral	AlexNet	ventral ovale (40).jpg	fold3	4	*Amblyomma*
1454	Ventral	AlexNet	ventral ovale (41).jpg	fold3	4	*Amblyomma*
1455	Ventral	AlexNet	ventral ovale (43).jpg	fold3	4	*Amblyomma*
1456	Ventral	AlexNet	ventral ovale (48).jpg	fold3	4	*Amblyomma*
1457	Ventral	AlexNet	ventral ovale (54).jpg	fold3	4	*Amblyomma*
1458	Ventral	AlexNet	ventral ovale (55).jpg	fold3	4	*Amblyomma*
1459	Ventral	AlexNet	ventral ovale (7).jpg	fold3	4	*Amblyomma*
1460	Ventral	AlexNet	ventral sculptum (13).JPG	fold3	5	*Amblyomma*
1461	Ventral	AlexNet	ventral sculptum (17).JPG	fold3	5	*Amblyomma*
1462	Ventral	AlexNet	ventral sculptum (25).JPG	fold3	5	*Amblyomma*
1463	Ventral	AlexNet	ventral sculptum (26).JPG	fold3	5	*Amblyomma*
1464	Ventral	AlexNet	ventral sculptum (44).JPG	fold3	5	*Amblyomma*
1465	Ventral	AlexNet	ventral sculptum (47).JPG	fold3	5	*Amblyomma*
1466	Ventral	AlexNet	ventral sculptum (49).JPG	fold3	5	*Amblyomma*
1467	Ventral	AlexNet	ventral sculptum (5).JPG	fold3	5	*Amblyomma*
1468	Ventral	AlexNet	ventral sculptum (50).JPG	fold3	5	*Amblyomma*
1469	Ventral	AlexNet	ventral sculptum (62).JPG	fold3	5	*Amblyomma*
1470	Ventral	AlexNet	ventral sculptum (64).JPG	fold3	5	*Amblyomma*
1471	Ventral	AlexNet	ventral sculptum (68).JPG	fold3	5	*Amblyomma*
1472	Ventral	AlexNet	ventral sculptum (69).JPG	fold3	5	*Amblyomma*

1473	Ventral	AlexNet	ventral sculptum (71).jpg	fold3	5	*Amblyomma*
1474	Ventral	AlexNet	ventral sculptum (74).jpg	fold3	5	*Amblyomma*
1475	Ventral	AlexNet	ventral triste (1).jpg	fold3	6	*Amblyomma*
1476	Ventral	AlexNet	ventral triste (13).jpg	fold3	6	*Amblyomma*
1477	Ventral	AlexNet	ventral triste (14).jpg	fold3	6	*Amblyomma*
1478	Ventral	AlexNet	ventral triste (20).jpg	fold3	6	*Amblyomma*
1479	Ventral	AlexNet	ventral triste (22).jpg	fold3	6	*Amblyomma*
1480	Ventral	AlexNet	ventral triste (23).jpg	fold3	6	*Amblyomma*
1481	Ventral	AlexNet	ventral triste (31).jpg	fold3	6	*Amblyomma*
1482	Ventral	AlexNet	ventral triste (32).jpg	fold3	6	*Amblyomma*
1483	Ventral	AlexNet	ventral triste (35).jpg	fold3	6	*Amblyomma*
1484	Ventral	AlexNet	ventral triste (46).jpg	fold3	6	*Amblyomma*
1485	Ventral	AlexNet	ventral triste (59).jpg	fold3	6	*Amblyomma*
1486	Ventral	AlexNet	ventral triste (63).jpg	fold3	6	*Amblyomma*
1487	Ventral	AlexNet	ventral triste (69).jpg	fold3	6	*Amblyomma*
1488	Ventral	AlexNet	ventral triste (70).jpg	fold3	6	*Amblyomma*
1489	Ventral	AlexNet	ventral triste (73).jpg	fold3	6	*Amblyomma*
1490	Ventral	AlexNet	ventral aureolatum (1).jpg	fold4	1	*Amblyomma*
1491	Ventral	AlexNet	ventral aureolatum (15).jpg	fold4	1	*Amblyomma*
1492	Ventral	AlexNet	ventral aureolatum (35).jpg	fold4	1	*Amblyomma*
1493	Ventral	AlexNet	ventral aureolatum (37).jpg	fold4	1	*Amblyomma*
1494	Ventral	AlexNet	ventral aureolatum (41).jpg	fold4	1	*Amblyomma*
1495	Ventral	AlexNet	ventral aureolatum (43).jpg	fold4	1	*Amblyomma*
1496	Ventral	AlexNet	ventral aureolatum (46).jpg	fold4	1	*Amblyomma*
1497	Ventral	AlexNet	ventral aureolatum (52).jpg	fold4	1	*Amblyomma*
1498	Ventral	AlexNet	ventral aureolatum (54).jpg	fold4	1	*Amblyomma*
1499	Ventral	AlexNet	ventral aureolatum (57).jpg	fold4	1	*Amblyomma*
1500	Ventral	AlexNet	ventral aureolatum (6).jpg	fold4	1	*Amblyomma*
1501	Ventral	AlexNet	ventral aureolatum (8).jpg	fold4	1	*Amblyomma*
1502	Ventral	AlexNet	ventral cajennense (10).jpg	fold4	2	*Amblyomma*
1503	Ventral	AlexNet	ventral cajennense (17).jpg	fold4	2	*Amblyomma*
1504	Ventral	AlexNet	ventral cajennense (18).jpg	fold4	2	*Amblyomma*
1505	Ventral	AlexNet	ventral cajennense (19).jpg	fold4	2	*Amblyomma*
1506	Ventral	AlexNet	ventral cajennense (21).jpg	fold4	2	*Amblyomma*
1507	Ventral	AlexNet	ventral cajennense (27).jpg	fold4	2	*Amblyomma*
1508	Ventral	AlexNet	ventral cajennense (34).jpg	fold4	2	*Amblyomma*
1509	Ventral	AlexNet	ventral cajennense (42).jpg	fold4	2	*Amblyomma*
1510	Ventral	AlexNet	ventral cajennense (44).jpg	fold4	2	*Amblyomma*
1511	Ventral	AlexNet	ventral cajennense (46).jpg	fold4	2	*Amblyomma*
1512	Ventral	AlexNet	ventral cajennense (48).jpg	fold4	2	*Amblyomma*
1513	Ventral	AlexNet	ventral cajennense (53).jpg	fold4	2	*Amblyomma*
1514	Ventral	AlexNet	ventral cajennense (55).jpg	fold4	2	*Amblyomma*
1515	Ventral	AlexNet	ventral cajennense (6).jpg	fold4	2	*Amblyomma*
1516	Ventral	AlexNet	ventral cajennense (62).jpg	fold4	2	*Amblyomma*
1517	Ventral	AlexNet	ventral cajennense (66).jpg	fold4	2	*Amblyomma*
1518	Ventral	AlexNet	ventral cajennense (73).jpg	fold4	2	*Amblyomma*
1519	Ventral	AlexNet	ventral dubitatum (1).jpg	fold4	3	*Amblyomma*
1520	Ventral	AlexNet	ventral dubitatum (12).jpg	fold4	3	*Amblyomma*
1521	Ventral	AlexNet	ventral dubitatum (13).jpg	fold4	3	*Amblyomma*
1522	Ventral	AlexNet	ventral dubitatum (14).jpg	fold4	3	*Amblyomma*
1523	Ventral	AlexNet	ventral dubitatum (20).jpg	fold4	3	*Amblyomma*
1524	Ventral	AlexNet	ventral dubitatum (3).jpg	fold4	3	*Amblyomma*
1525	Ventral	AlexNet	ventral dubitatum (32).jpg	fold4	3	*Amblyomma*
1526	Ventral	AlexNet	ventral dubitatum (35).jpg	fold4	3	*Amblyomma*
1527	Ventral	AlexNet	ventral dubitatum (37).jpg	fold4	3	*Amblyomma*
1528	Ventral	AlexNet	ventral dubitatum (4).JPG	fold4	3	*Amblyomma*
1529	Ventral	AlexNet	ventral dubitatum (44).jpg	fold4	3	*Amblyomma*
1530	Ventral	AlexNet	ventral ovale (19).jpg	fold4	4	*Amblyomma*
1531	Ventral	AlexNet	ventral ovale (23).jpg	fold4	4	*Amblyomma*
1532	Ventral	AlexNet	ventral ovale (24).jpg	fold4	4	*Amblyomma*
1533	Ventral	AlexNet	ventral ovale (30).jpg	fold4	4	*Amblyomma*
1534	Ventral	AlexNet	ventral ovale (34).jpg	fold4	4	*Amblyomma*
1535	Ventral	AlexNet	ventral ovale (37).jpg	fold4	4	*Amblyomma*
1536	Ventral	AlexNet	ventral ovale (4).jpg	fold4	4	*Amblyomma*
1537	Ventral	AlexNet	ventral ovale (45).jpg	fold4	4	*Amblyomma*
1538	Ventral	AlexNet	ventral ovale (5).jpg	fold4	4	*Amblyomma*
1539	Ventral	AlexNet	ventral ovale (50).jpg	fold4	4	*Amblyomma*

1540	Ventral	AlexNet	ventral ovale (53).JPG	fold4	4	*Amblyomma*
1541	Ventral	AlexNet	ventral ovale (6).jpg	fold4	4	*Amblyomma*
1542	Ventral	AlexNet	ventral sculptum (15).JPG	fold4	5	*Amblyomma*
1543	Ventral	AlexNet	ventral sculptum (16).JPG	fold4	5	*Amblyomma*
1544	Ventral	AlexNet	ventral sculptum (21).JPG	fold4	5	*Amblyomma*
1545	Ventral	AlexNet	ventral sculptum (22).JPG	fold4	5	*Amblyomma*
1546	Ventral	AlexNet	ventral sculptum (27).JPG	fold4	5	*Amblyomma*
1547	Ventral	AlexNet	ventral sculptum (3).jpg	fold4	5	*Amblyomma*
1548	Ventral	AlexNet	ventral sculptum (33).jpg	fold4	5	*Amblyomma*
1549	Ventral	AlexNet	ventral sculptum (37).JPG	fold4	5	*Amblyomma*
1550	Ventral	AlexNet	ventral sculptum (41).JPG	fold4	5	*Amblyomma*
1551	Ventral	AlexNet	ventral sculptum (42).JPG	fold4	5	*Amblyomma*
1552	Ventral	AlexNet	ventral sculptum (55).JPG	fold4	5	*Amblyomma*
1553	Ventral	AlexNet	ventral sculptum (59).JPG	fold4	5	*Amblyomma*
1554	Ventral	AlexNet	ventral sculptum (65).JPG	fold4	5	*Amblyomma*
1555	Ventral	AlexNet	ventral sculptum (66).JPG	fold4	5	*Amblyomma*
1556	Ventral	AlexNet	ventral sculptum (8).JPG	fold4	5	*Amblyomma*
1557	Ventral	AlexNet	ventral triste (10).jpg	fold4	6	*Amblyomma*
1558	Ventral	AlexNet	ventral triste (15).jpg	fold4	6	*Amblyomma*
1559	Ventral	AlexNet	ventral triste (18).jpg	fold4	6	*Amblyomma*
1560	Ventral	AlexNet	ventral triste (19).jpg	fold4	6	*Amblyomma*
1561	Ventral	AlexNet	ventral triste (25).jpg	fold4	6	*Amblyomma*
1562	Ventral	AlexNet	ventral triste (28).jpg	fold4	6	*Amblyomma*
1563	Ventral	AlexNet	ventral triste (29).jpg	fold4	6	*Amblyomma*
1564	Ventral	AlexNet	ventral triste (34).jpg	fold4	6	*Amblyomma*
1565	Ventral	AlexNet	ventral triste (36).jpg	fold4	6	*Amblyomma*
1566	Ventral	AlexNet	ventral triste (4).jpg	fold4	6	*Amblyomma*
1567	Ventral	AlexNet	ventral triste (44).jpg	fold4	6	*Amblyomma*
1568	Ventral	AlexNet	ventral triste (5).jpg	fold4	6	*Amblyomma*
1569	Ventral	AlexNet	ventral triste (6).jpg	fold4	6	*Amblyomma*
1570	Ventral	AlexNet	ventral triste (60).jpg	fold4	6	*Amblyomma*
1571	Ventral	AlexNet	ventral triste (71).jpg	fold4	6	*Amblyomma*
1572	Ventral	AlexNet	ventral triste (74).jpg	fold4	6	*Amblyomma*
1573	Ventral	AlexNet	ventral aureolatum (13).jpg	fold5	1	*Amblyomma*
1574	Ventral	AlexNet	ventral aureolatum (14).jpg	fold5	1	*Amblyomma*
1575	Ventral	AlexNet	ventral aureolatum (2).jpg	fold5	1	*Amblyomma*
1576	Ventral	AlexNet	ventral aureolatum (23).jpg	fold5	1	*Amblyomma*
1577	Ventral	AlexNet	ventral aureolatum (24).jpg	fold5	1	*Amblyomma*
1578	Ventral	AlexNet	ventral aureolatum (25).jpg	fold5	1	*Amblyomma*
1579	Ventral	AlexNet	ventral aureolatum (29).jpg	fold5	1	*Amblyomma*
1580	Ventral	AlexNet	ventral aureolatum (30).jpg	fold5	1	*Amblyomma*
1581	Ventral	AlexNet	ventral aureolatum (32).jpg	fold5	1	*Amblyomma*
1582	Ventral	AlexNet	ventral aureolatum (45).jpg	fold5	1	*Amblyomma*
1583	Ventral	AlexNet	ventral aureolatum (55).jpg	fold5	1	*Amblyomma*
1584	Ventral	AlexNet	ventral cajennense (13).jpg	fold5	2	*Amblyomma*
1585	Ventral	AlexNet	ventral cajennense (2).jpg	fold5	2	*Amblyomma*
1586	Ventral	AlexNet	ventral cajennense (24).jpg	fold5	2	*Amblyomma*
1587	Ventral	AlexNet	ventral cajennense (25).jpg	fold5	2	*Amblyomma*
1588	Ventral	AlexNet	ventral cajennense (26).jpg	fold5	2	*Amblyomma*
1589	Ventral	AlexNet	ventral cajennense (32).jpg	fold5	2	*Amblyomma*
1590	Ventral	AlexNet	ventral cajennense (40).jpg	fold5	2	*Amblyomma*
1591	Ventral	AlexNet	ventral cajennense (43).jpg	fold5	2	*Amblyomma*
1592	Ventral	AlexNet	ventral cajennense (50).jpg	fold5	2	*Amblyomma*
1593	Ventral	AlexNet	ventral cajennense (58).jpg	fold5	2	*Amblyomma*
1594	Ventral	AlexNet	ventral cajennense (59).jpg	fold5	2	*Amblyomma*
1595	Ventral	AlexNet	ventral cajennense (68).jpg	fold5	2	*Amblyomma*
1596	Ventral	AlexNet	ventral cajennense (76).jpg	fold5	2	*Amblyomma*
1597	Ventral	AlexNet	ventral cajennense (77).jpg	fold5	2	*Amblyomma*
1598	Ventral	AlexNet	ventral cajennense (79).jpg	fold5	2	*Amblyomma*
1599	Ventral	AlexNet	ventral cajennense (8).jpg	fold5	2	*Amblyomma*
1600	Ventral	AlexNet	ventral cajennense (80).jpg	fold5	2	*Amblyomma*
1601	Ventral	AlexNet	ventral dubitatum (10).jpg	fold5	3	*Amblyomma*
1602	Ventral	AlexNet	ventral dubitatum (16).jpg	fold5	3	*Amblyomma*
1603	Ventral	AlexNet	ventral dubitatum (17).jpg	fold5	3	*Amblyomma*
1604	Ventral	AlexNet	ventral dubitatum (18).jpg	fold5	3	*Amblyomma*
1605	Ventral	AlexNet	ventral dubitatum (21).jpg	fold5	3	*Amblyomma*
1606	Ventral	AlexNet	ventral dubitatum (29).jpg	fold5	3	*Amblyomma*

1607	Ventral	AlexNet	ventral dubitatum (31).jpg	fold5	3	*Amblyomma*
1608	Ventral	AlexNet	ventral dubitatum (47).jpg	fold5	3	*Amblyomma*
1609	Ventral	AlexNet	ventral dubitatum (6).jpg	fold5	3	*Amblyomma*
1610	Ventral	AlexNet	ventral dubitatum (9).jpg	fold5	3	*Amblyomma*
1611	Ventral	AlexNet	ventral ovale (11).jpg	fold5	4	*Amblyomma*
1612	Ventral	AlexNet	ventral ovale (15).jpg	fold5	4	*Amblyomma*
1613	Ventral	AlexNet	ventral ovale (17).jpg	fold5	4	*Amblyomma*
1614	Ventral	AlexNet	ventral ovale (21).jpg	fold5	4	*Amblyomma*
1615	Ventral	AlexNet	ventral ovale (25).jpg	fold5	4	*Amblyomma*
1616	Ventral	AlexNet	ventral ovale (27).jpg	fold5	4	*Amblyomma*
1617	Ventral	AlexNet	ventral ovale (39).jpg	fold5	4	*Amblyomma*
1618	Ventral	AlexNet	ventral ovale (46).jpg	fold5	4	*Amblyomma*
1619	Ventral	AlexNet	ventral ovale (47).jpg	fold5	4	*Amblyomma*
1620	Ventral	AlexNet	ventral ovale (52).jpg	fold5	4	*Amblyomma*
1621	Ventral	AlexNet	ventral ovale (56).jpg	fold5	4	*Amblyomma*
1622	Ventral	AlexNet	ventral ovale (59).jpg	fold5	4	*Amblyomma*
1623	Ventral	AlexNet	ventral sculptum (11).JPG	fold5	5	*Amblyomma*
1624	Ventral	AlexNet	ventral sculptum (14).JPG	fold5	5	*Amblyomma*
1625	Ventral	AlexNet	ventral sculptum (19).JPG	fold5	5	*Amblyomma*
1626	Ventral	AlexNet	ventral sculptum (20).JPG	fold5	5	*Amblyomma*
1627	Ventral	AlexNet	ventral sculptum (24).JPG	fold5	5	*Amblyomma*
1628	Ventral	AlexNet	ventral sculptum (34).jpg	fold5	5	*Amblyomma*
1629	Ventral	AlexNet	ventral sculptum (35).JPG	fold5	5	*Amblyomma*
1630	Ventral	AlexNet	ventral sculptum (36).JPG	fold5	5	*Amblyomma*
1631	Ventral	AlexNet	ventral sculptum (52).JPG	fold5	5	*Amblyomma*
1632	Ventral	AlexNet	ventral sculptum (56).JPG	fold5	5	*Amblyomma*
1633	Ventral	AlexNet	ventral sculptum (57).JPG	fold5	5	*Amblyomma*
1634	Ventral	AlexNet	ventral sculptum (61).JPG	fold5	5	*Amblyomma*
1635	Ventral	AlexNet	ventral sculptum (70).jpg	fold5	5	*Amblyomma*
1636	Ventral	AlexNet	ventral sculptum (72).jpg	fold5	5	*Amblyomma*
1637	Ventral	AlexNet	ventral sculptum (73).jpg	fold5	5	*Amblyomma*
1638	Ventral	AlexNet	ventral triste (17).jpg	fold5	6	*Amblyomma*
1639	Ventral	AlexNet	ventral triste (24).jpg	fold5	6	*Amblyomma*
1640	Ventral	AlexNet	ventral triste (26).jpg	fold5	6	*Amblyomma*
1641	Ventral	AlexNet	ventral triste (27).jpg	fold5	6	*Amblyomma*
1642	Ventral	AlexNet	ventral triste (3).jpg	fold5	6	*Amblyomma*
1643	Ventral	AlexNet	ventral triste (50).jpg	fold5	6	*Amblyomma*
1644	Ventral	AlexNet	ventral triste (51).jpg	fold5	6	*Amblyomma*
1645	Ventral	AlexNet	ventral triste (55).jpg	fold5	6	*Amblyomma*
1646	Ventral	AlexNet	ventral triste (58).jpg	fold5	6	*Amblyomma*
1647	Ventral	AlexNet	ventral triste (61).jpg	fold5	6	*Amblyomma*
1648	Ventral	AlexNet	ventral triste (62).jpg	fold5	6	*Amblyomma*
1649	Ventral	AlexNet	ventral triste (64).jpg	fold5	6	*Amblyomma*
1650	Ventral	AlexNet	ventral triste (65).jpg	fold5	6	*Amblyomma*
1651	Ventral	AlexNet	ventral triste (7).jpg	fold5	6	*Amblyomma*
1652	Ventral	AlexNet	ventral triste (9).jpg	fold5	6	*Amblyomma*
1653	Low resolution	AlexNet	baixa aureolatum (11).jpg	fold1	1	*Amblyomma*
1654	Low resolution	AlexNet	baixa aureolatum (17).jpg	fold1	1	*Amblyomma*
1655	Low resolution	AlexNet	baixa aureolatum (23).jpg	fold1	1	*Amblyomma*
1656	Low resolution	AlexNet	baixa aureolatum (27).jpg	fold1	1	*Amblyomma*
1657	Low resolution	AlexNet	baixa aureolatum (30).jpg	fold1	1	*Amblyomma*
1658	Low resolution	AlexNet	baixa aureolatum (46).jpg	fold1	1	*Amblyomma*
1659	Low resolution	AlexNet	baixa aureolatum (47).jpg	fold1	1	*Amblyomma*
1660	Low resolution	AlexNet	baixa aureolatum (48).jpg	fold1	1	*Amblyomma*
1661	Low resolution	AlexNet	baixa aureolatum (52).jpg	fold1	1	*Amblyomma*
1662	Low resolution	AlexNet	baixa aureolatum (7).jpg	fold1	1	*Amblyomma*
1663	Low resolution	AlexNet	baixa cajennense (1).jpg	fold1	2	*Amblyomma*
1664	Low resolution	AlexNet	baixa cajennense (23).jpg	fold1	2	*Amblyomma*
1665	Low resolution	AlexNet	baixa cajennense (27).jpg	fold1	2	*Amblyomma*
1666	Low resolution	AlexNet	baixa cajennense (30).jpg	fold1	2	*Amblyomma*
1667	Low resolution	AlexNet	baixa cajennense (31).jpg	fold1	2	*Amblyomma*
1668	Low resolution	AlexNet	baixa cajennense (37).jpg	fold1	2	*Amblyomma*
1669	Low resolution	AlexNet	baixa cajennense (4).jpg	fold1	2	*Amblyomma*
1670	Low resolution	AlexNet	baixa cajennense (44).jpg	fold1	2	*Amblyomma*
1671	Low resolution	AlexNet	baixa cajennense (48).jpg	fold1	2	*Amblyomma*
1672	Low resolution	AlexNet	baixa cajennense (5).jpg	fold1	2	*Amblyomma*
1673	Low resolution	AlexNet	baixa cajennense (50).jpg	fold1	2	*Amblyomma*

1674	Low resolution	AlexNet	baixa cajennense (51).jpg	fold1	2	*Amblyomma*
1675	Low resolution	AlexNet	baixa cajennense (64).jpg	fold1	2	*Amblyomma*
1676	Low resolution	AlexNet	baixa cajennense (7).jpg	fold1	2	*Amblyomma*
1677	Low resolution	AlexNet	baixa cajennense (77).jpg	fold1	2	*Amblyomma*
1678	Low resolution	AlexNet	baixa cajennense (80).jpg	fold1	2	*Amblyomma*
1679	Low resolution	AlexNet	baixa dubitatum (10).jpg	fold1	3	*Amblyomma*
1680	Low resolution	AlexNet	baixa dubitatum (18).jpg	fold1	3	*Amblyomma*
1681	Low resolution	AlexNet	baixa dubitatum (22).jpg	fold1	3	*Amblyomma*
1682	Low resolution	AlexNet	baixa dubitatum (23).jpg	fold1	3	*Amblyomma*
1683	Low resolution	AlexNet	baixa dubitatum (27).jpg	fold1	3	*Amblyomma*
1684	Low resolution	AlexNet	baixa dubitatum (30).jpg	fold1	3	*Amblyomma*
1685	Low resolution	AlexNet	baixa dubitatum (31).jpg	fold1	3	*Amblyomma*
1686	Low resolution	AlexNet	baixa dubitatum (5).jpg	fold1	3	*Amblyomma*
1687	Low resolution	AlexNet	baixa ovale (14).jpg	fold1	4	*Amblyomma*
1688	Low resolution	AlexNet	baixa ovale (20).jpg	fold1	4	*Amblyomma*
1689	Low resolution	AlexNet	baixa ovale (22).jpg	fold1	4	*Amblyomma*
1690	Low resolution	AlexNet	baixa ovale (29).jpg	fold1	4	*Amblyomma*
1691	Low resolution	AlexNet	baixa ovale (36).jpg	fold1	4	*Amblyomma*
1692	Low resolution	AlexNet	baixa ovale (39).jpg	fold1	4	*Amblyomma*
1693	Low resolution	AlexNet	baixa ovale (43).jpg	fold1	4	*Amblyomma*
1694	Low resolution	AlexNet	baixa ovale (44).jpg	fold1	4	*Amblyomma*
1695	Low resolution	AlexNet	baixa ovale (48).jpg	fold1	4	*Amblyomma*
1696	Low resolution	AlexNet	baixa ovale (54).jpg	fold1	4	*Amblyomma*
1697	Low resolution	AlexNet	baixa ovale (9).jpg	fold1	4	*Amblyomma*
1698	Low resolution	AlexNet	baixa sculptum (17).jpg	fold1	5	*Amblyomma*
1699	Low resolution	AlexNet	baixa sculptum (5).jpg	fold1	5	*Amblyomma*
1700	Low resolution	AlexNet	baixa sculptum (6).jpg	fold1	5	*Amblyomma*
1701	Low resolution	AlexNet	baixa sculptum (8).jpg	fold1	5	*Amblyomma*
1702	Low resolution	AlexNet	baixa triste (12).jpg	fold1	6	*Amblyomma*
1703	Low resolution	AlexNet	baixa triste (15).jpg	fold1	6	*Amblyomma*
1704	Low resolution	AlexNet	baixa triste (16).jpg	fold1	6	*Amblyomma*
1705	Low resolution	AlexNet	baixa triste (2).jpg	fold1	6	*Amblyomma*
1706	Low resolution	AlexNet	baixa triste (23).jpg	fold1	6	*Amblyomma*
1707	Low resolution	AlexNet	baixa triste (32).jpg	fold1	6	*Amblyomma*
1708	Low resolution	AlexNet	baixa triste (33).jpg	fold1	6	*Amblyomma*
1709	Low resolution	AlexNet	baixa triste (38).jpg	fold1	6	*Amblyomma*
1710	Low resolution	AlexNet	baixa triste (42).jpg	fold1	6	*Amblyomma*
1711	Low resolution	AlexNet	baixa triste (47).jpg	fold1	6	*Amblyomma*
1712	Low resolution	AlexNet	baixa triste (48).jpg	fold1	6	*Amblyomma*
1713	Low resolution	AlexNet	baixa triste (57).jpg	fold1	6	*Amblyomma*
1714	Low resolution	AlexNet	baixa triste (63).jpg	fold1	6	*Amblyomma*
1715	Low resolution	AlexNet	baixa triste (64).jpg	fold1	6	*Amblyomma*
1716	Low resolution	AlexNet	baixa triste (7).jpg	fold1	6	*Amblyomma*
1717	Low resolution	AlexNet	baixa triste (71).jpg	fold1	6	*Amblyomma*
1718	Low resolution	AlexNet	baixa aureolatum (18).jpg	fold2	1	*Amblyomma*
1719	Low resolution	AlexNet	baixa aureolatum (21).jpg	fold2	1	*Amblyomma*
1720	Low resolution	AlexNet	baixa aureolatum (28).jpg	fold2	1	*Amblyomma*
1721	Low resolution	AlexNet	baixa aureolatum (29).jpg	fold2	1	*Amblyomma*
1722	Low resolution	AlexNet	baixa aureolatum (32).jpg	fold2	1	*Amblyomma*
1723	Low resolution	AlexNet	baixa aureolatum (38).jpg	fold2	1	*Amblyomma*
1724	Low resolution	AlexNet	baixa aureolatum (43).jpg	fold2	1	*Amblyomma*
1725	Low resolution	AlexNet	baixa aureolatum (44).jpg	fold2	1	*Amblyomma*
1726	Low resolution	AlexNet	baixa aureolatum (49).jpg	fold2	1	*Amblyomma*
1727	Low resolution	AlexNet	baixa aureolatum (5).jpg	fold2	1	*Amblyomma*
1728	Low resolution	AlexNet	baixa aureolatum (51).jpg	fold2	1	*Amblyomma*
1729	Low resolution	AlexNet	baixa cajennense (13).jpg	fold2	2	*Amblyomma*
1730	Low resolution	AlexNet	baixa cajennense (14).jpg	fold2	2	*Amblyomma*
1731	Low resolution	AlexNet	baixa cajennense (34).jpg	fold2	2	*Amblyomma*
1732	Low resolution	AlexNet	baixa cajennense (35).jpg	fold2	2	*Amblyomma*
1733	Low resolution	AlexNet	baixa cajennense (39).jpg	fold2	2	*Amblyomma*
1734	Low resolution	AlexNet	baixa cajennense (41).jpg	fold2	2	*Amblyomma*
1735	Low resolution	AlexNet	baixa cajennense (43).jpg	fold2	2	*Amblyomma*
1736	Low resolution	AlexNet	baixa cajennense (53).jpg	fold2	2	*Amblyomma*
1737	Low resolution	AlexNet	baixa cajennense (56).jpg	fold2	2	*Amblyomma*
1738	Low resolution	AlexNet	baixa cajennense (6).jpg	fold2	2	*Amblyomma*
1739	Low resolution	AlexNet	baixa cajennense (61).jpg	fold2	2	*Amblyomma*
1740	Low resolution	AlexNet	baixa cajennense (63).jpg	fold2	2	*Amblyomma*

1741	Low resolution	AlexNet	baixa cajennense (67).jpg	fold2	2	*Amblyomma*
1742	Low resolution	AlexNet	baixa cajennense (74).jpg	fold2	2	*Amblyomma*
1743	Low resolution	AlexNet	baixa cajennense (75).jpg	fold2	2	*Amblyomma*
1744	Low resolution	AlexNet	baixa cajennense (79).jpg	fold2	2	*Amblyomma*
1745	Low resolution	AlexNet	baixa dubitatum (26).jpg	fold2	3	*Amblyomma*
1746	Low resolution	AlexNet	baixa dubitatum (33).jpg	fold2	3	*Amblyomma*
1747	Low resolution	AlexNet	baixa dubitatum (34).jpg	fold2	3	*Amblyomma*
1748	Low resolution	AlexNet	baixa dubitatum (35).jpg	fold2	3	*Amblyomma*
1749	Low resolution	AlexNet	baixa dubitatum (37).jpg	fold2	3	*Amblyomma*
1750	Low resolution	AlexNet	baixa dubitatum (39).jpg	fold2	3	*Amblyomma*
1751	Low resolution	AlexNet	baixa dubitatum (40).jpg	fold2	3	*Amblyomma*
1752	Low resolution	AlexNet	baixa dubitatum (43).jpg	fold2	3	*Amblyomma*
1753	Low resolution	AlexNet	baixa dubitatum (7).jpg	fold2	3	*Amblyomma*
1754	Low resolution	AlexNet	baixa ovale (2).jpg	fold2	4	*Amblyomma*
1755	Low resolution	AlexNet	baixa ovale (24).jpg	fold2	4	*Amblyomma*
1756	Low resolution	AlexNet	baixa ovale (25).jpg	fold2	4	*Amblyomma*
1757	Low resolution	AlexNet	baixa ovale (26).jpg	fold2	4	*Amblyomma*
1758	Low resolution	AlexNet	baixa ovale (27).jpg	fold2	4	*Amblyomma*
1759	Low resolution	AlexNet	baixa ovale (31).jpg	fold2	4	*Amblyomma*
1760	Low resolution	AlexNet	baixa ovale (35).jpg	fold2	4	*Amblyomma*
1761	Low resolution	AlexNet	baixa ovale (41).jpg	fold2	4	*Amblyomma*
1762	Low resolution	AlexNet	baixa ovale (42).jpg	fold2	4	*Amblyomma*
1763	Low resolution	AlexNet	baixa ovale (47).jpg	fold2	4	*Amblyomma*
1764	Low resolution	AlexNet	baixa ovale (5).jpg	fold2	4	*Amblyomma*
1765	Low resolution	AlexNet	baixa sculptum (13).jpg	fold2	5	*Amblyomma*
1766	Low resolution	AlexNet	baixa sculptum (3).jpg	fold2	5	*Amblyomma*
1767	Low resolution	AlexNet	baixa sculptum (7).jpg	fold2	5	*Amblyomma*
1768	Low resolution	AlexNet	baixa triste (10).jpg	fold2	6	*Amblyomma*
1769	Low resolution	AlexNet	baixa triste (11).jpg	fold2	6	*Amblyomma*
1770	Low resolution	AlexNet	baixa triste (13).jpg	fold2	6	*Amblyomma*
1771	Low resolution	AlexNet	baixa triste (17).jpg	fold2	6	*Amblyomma*
1772	Low resolution	AlexNet	baixa triste (21).jpg	fold2	6	*Amblyomma*
1773	Low resolution	AlexNet	baixa triste (25).jpg	fold2	6	*Amblyomma*
1774	Low resolution	AlexNet	baixa triste (26).jpg	fold2	6	*Amblyomma*
1775	Low resolution	AlexNet	baixa triste (30).jpg	fold2	6	*Amblyomma*
1776	Low resolution	AlexNet	baixa triste (37).jpg	fold2	6	*Amblyomma*
1777	Low resolution	AlexNet	baixa triste (40).jpg	fold2	6	*Amblyomma*
1778	Low resolution	AlexNet	baixa triste (41).jpg	fold2	6	*Amblyomma*
1779	Low resolution	AlexNet	baixa triste (50).jpg	fold2	6	*Amblyomma*
1780	Low resolution	AlexNet	baixa triste (55).jpg	fold2	6	*Amblyomma*
1781	Low resolution	AlexNet	baixa triste (61).jpg	fold2	6	*Amblyomma*
1782	Low resolution	AlexNet	baixa triste (69).jpg	fold2	6	*Amblyomma*
1783	Low resolution	AlexNet	baixa triste (75).jpg	fold2	6	*Amblyomma*
1784	Low resolution	AlexNet	baixa aureolatum (1).jpg	fold3	1	*Amblyomma*
1785	Low resolution	AlexNet	baixa aureolatum (14).jpg	fold3	1	*Amblyomma*
1786	Low resolution	AlexNet	baixa aureolatum (19).jpg	fold3	1	*Amblyomma*
1787	Low resolution	AlexNet	baixa aureolatum (24).jpg	fold3	1	*Amblyomma*
1788	Low resolution	AlexNet	baixa aureolatum (33).jpg	fold3	1	*Amblyomma*
1789	Low resolution	AlexNet	baixa aureolatum (35).jpg	fold3	1	*Amblyomma*
1790	Low resolution	AlexNet	baixa aureolatum (36).jpg	fold3	1	*Amblyomma*
1791	Low resolution	AlexNet	baixa aureolatum (40).jpg	fold3	1	*Amblyomma*
1792	Low resolution	AlexNet	baixa aureolatum (53).jpg	fold3	1	*Amblyomma*
1793	Low resolution	AlexNet	baixa aureolatum (6).jpg	fold3	1	*Amblyomma*
1794	Low resolution	AlexNet	baixa cajennense (10).jpg	fold3	2	*Amblyomma*
1795	Low resolution	AlexNet	baixa cajennense (11).jpg	fold3	2	*Amblyomma*
1796	Low resolution	AlexNet	baixa cajennense (16).jpg	fold3	2	*Amblyomma*
1797	Low resolution	AlexNet	baixa cajennense (18).jpg	fold3	2	*Amblyomma*
1798	Low resolution	AlexNet	baixa cajennense (2).jpg	fold3	2	*Amblyomma*
1799	Low resolution	AlexNet	baixa cajennense (26).jpg	fold3	2	*Amblyomma*
1800	Low resolution	AlexNet	baixa cajennense (29).jpg	fold3	2	*Amblyomma*
1801	Low resolution	AlexNet	baixa cajennense (38).jpg	fold3	2	*Amblyomma*
1802	Low resolution	AlexNet	baixa cajennense (55).jpg	fold3	2	*Amblyomma*
1803	Low resolution	AlexNet	baixa cajennense (57).jpg	fold3	2	*Amblyomma*
1804	Low resolution	AlexNet	baixa cajennense (59).jpg	fold3	2	*Amblyomma*
1805	Low resolution	AlexNet	baixa cajennense (62).jpg	fold3	2	*Amblyomma*
1806	Low resolution	AlexNet	baixa cajennense (65).jpg	fold3	2	*Amblyomma*
1807	Low resolution	AlexNet	baixa cajennense (66).jpg	fold3	2	*Amblyomma*

1808	Low resolution	AlexNet	baixa cajennense (71).jpg	fold3	2	*Amblyomma*
1809	Low resolution	AlexNet	baixa cajennense (76).jpg	fold3	2	*Amblyomma*
1810	Low resolution	AlexNet	baixa cajennense (8).jpg	fold3	2	*Amblyomma*
1811	Low resolution	AlexNet	baixa dubitatum (15).jpg	fold3	3	*Amblyomma*
1812	Low resolution	AlexNet	baixa dubitatum (20).jpg	fold3	3	*Amblyomma*
1813	Low resolution	AlexNet	baixa dubitatum (28).jpg	fold3	3	*Amblyomma*
1814	Low resolution	AlexNet	baixa dubitatum (29).jpg	fold3	3	*Amblyomma*
1815	Low resolution	AlexNet	baixa dubitatum (32).jpg	fold3	3	*Amblyomma*
1816	Low resolution	AlexNet	baixa dubitatum (38).jpg	fold3	3	*Amblyomma*
1817	Low resolution	AlexNet	baixa dubitatum (4).jpg	fold3	3	*Amblyomma*
1818	Low resolution	AlexNet	baixa dubitatum (41).jpg	fold3	3	*Amblyomma*
1819	Low resolution	AlexNet	baixa dubitatum (42).jpg	fold3	3	*Amblyomma*
1820	Low resolution	AlexNet	baixa ovale (11).jpg	fold3	4	*Amblyomma*
1821	Low resolution	AlexNet	baixa ovale (21).jpg	fold3	4	*Amblyomma*
1822	Low resolution	AlexNet	baixa ovale (28).jpg	fold3	4	*Amblyomma*
1823	Low resolution	AlexNet	baixa ovale (30).jpg	fold3	4	*Amblyomma*
1824	Low resolution	AlexNet	baixa ovale (34).jpg	fold3	4	*Amblyomma*
1825	Low resolution	AlexNet	baixa ovale (37).jpg	fold3	4	*Amblyomma*
1826	Low resolution	AlexNet	baixa ovale (45).jpg	fold3	4	*Amblyomma*
1827	Low resolution	AlexNet	baixa ovale (46).jpg	fold3	4	*Amblyomma*
1828	Low resolution	AlexNet	baixa ovale (52).jpg	fold3	4	*Amblyomma*
1829	Low resolution	AlexNet	baixa ovale (55).jpg	fold3	4	*Amblyomma*
1830	Low resolution	AlexNet	baixa ovale (8).jpg	fold3	4	*Amblyomma*
1831	Low resolution	AlexNet	baixa sculptum (1).jpg	fold3	5	*Amblyomma*
1832	Low resolution	AlexNet	baixa sculptum (11).jpg	fold3	5	*Amblyomma*
1833	Low resolution	AlexNet	baixa sculptum (12).jpg	fold3	5	*Amblyomma*
1834	Low resolution	AlexNet	baixa sculptum (9).jpg	fold3	5	*Amblyomma*
1835	Low resolution	AlexNet	baixa triste (1).jpg	fold3	6	*Amblyomma*
1836	Low resolution	AlexNet	baixa triste (14).jpg	fold3	6	*Amblyomma*
1837	Low resolution	AlexNet	baixa triste (24).jpg	fold3	6	*Amblyomma*
1838	Low resolution	AlexNet	baixa triste (28).jpg	fold3	6	*Amblyomma*
1839	Low resolution	AlexNet	baixa triste (34).jpg	fold3	6	*Amblyomma*
1840	Low resolution	AlexNet	baixa triste (36).jpg	fold3	6	*Amblyomma*
1841	Low resolution	AlexNet	baixa triste (39).jpg	fold3	6	*Amblyomma*
1842	Low resolution	AlexNet	baixa triste (4).jpg	fold3	6	*Amblyomma*
1843	Low resolution	AlexNet	baixa triste (52).jpg	fold3	6	*Amblyomma*
1844	Low resolution	AlexNet	baixa triste (53).jpg	fold3	6	*Amblyomma*
1845	Low resolution	AlexNet	baixa triste (66).jpg	fold3	6	*Amblyomma*
1846	Low resolution	AlexNet	baixa triste (73).jpg	fold3	6	*Amblyomma*
1847	Low resolution	AlexNet	baixa triste (74).jpg	fold3	6	*Amblyomma*
1848	Low resolution	AlexNet	baixa triste (76).jpg	fold3	6	*Amblyomma*
1849	Low resolution	AlexNet	baixa triste (77).jpg	fold3	6	*Amblyomma*
1850	Low resolution	AlexNet	baixa aureolatum (12).jpg	fold4	1	*Amblyomma*
1851	Low resolution	AlexNet	baixa aureolatum (13).jpg	fold4	1	*Amblyomma*
1852	Low resolution	AlexNet	baixa aureolatum (15).jpg	fold4	1	*Amblyomma*
1853	Low resolution	AlexNet	baixa aureolatum (16).jpg	fold4	1	*Amblyomma*
1854	Low resolution	AlexNet	baixa aureolatum (20).jpg	fold4	1	*Amblyomma*
1855	Low resolution	AlexNet	baixa aureolatum (22).jpg	fold4	1	*Amblyomma*
1856	Low resolution	AlexNet	baixa aureolatum (25).jpg	fold4	1	*Amblyomma*
1857	Low resolution	AlexNet	baixa aureolatum (26).jpg	fold4	1	*Amblyomma*
1858	Low resolution	AlexNet	baixa aureolatum (37).jpg	fold4	1	*Amblyomma*
1859	Low resolution	AlexNet	baixa aureolatum (4).jpg	fold4	1	*Amblyomma*
1860	Low resolution	AlexNet	baixa aureolatum (8).jpg	fold4	1	*Amblyomma*
1861	Low resolution	AlexNet	baixa cajennense (15).jpg	fold4	2	*Amblyomma*
1862	Low resolution	AlexNet	baixa cajennense (17).jpg	fold4	2	*Amblyomma*
1863	Low resolution	AlexNet	baixa cajennense (19).jpg	fold4	2	*Amblyomma*
1864	Low resolution	AlexNet	baixa cajennense (20).jpg	fold4	2	*Amblyomma*
1865	Low resolution	AlexNet	baixa cajennense (21).jpg	fold4	2	*Amblyomma*
1866	Low resolution	AlexNet	baixa cajennense (22).jpg	fold4	2	*Amblyomma*
1867	Low resolution	AlexNet	baixa cajennense (3).jpg	fold4	2	*Amblyomma*
1868	Low resolution	AlexNet	baixa cajennense (42).jpg	fold4	2	*Amblyomma*
1869	Low resolution	AlexNet	baixa cajennense (45).jpg	fold4	2	*Amblyomma*
1870	Low resolution	AlexNet	baixa cajennense (47).jpg	fold4	2	*Amblyomma*
1871	Low resolution	AlexNet	baixa cajennense (49).jpg	fold4	2	*Amblyomma*
1872	Low resolution	AlexNet	baixa cajennense (54).jpg	fold4	2	*Amblyomma*
1873	Low resolution	AlexNet	baixa cajennense (69).jpg	fold4	2	*Amblyomma*
1874	Low resolution	AlexNet	baixa cajennense (72).jpg	fold4	2	*Amblyomma*

1875	Low resolution	AlexNet	baixa cajennense (73).jpg	fold4	2	*Amblyomma*
1876	Low resolution	AlexNet	baixa cajennense (81).jpg	fold4	2	*Amblyomma*
1877	Low resolution	AlexNet	baixa dubitatum (1).jpg	fold4	3	*Amblyomma*
1878	Low resolution	AlexNet	baixa dubitatum (13).jpg	fold4	3	*Amblyomma*
1879	Low resolution	AlexNet	baixa dubitatum (14).jpg	fold4	3	*Amblyomma*
1880	Low resolution	AlexNet	baixa dubitatum (17).jpg	fold4	3	*Amblyomma*
1881	Low resolution	AlexNet	baixa dubitatum (2).jpg	fold4	3	*Amblyomma*
1882	Low resolution	AlexNet	baixa dubitatum (21).jpg	fold4	3	*Amblyomma*
1883	Low resolution	AlexNet	baixa dubitatum (36).jpg	fold4	3	*Amblyomma*
1884	Low resolution	AlexNet	baixa dubitatum (8).jpg	fold4	3	*Amblyomma*
1885	Low resolution	AlexNet	baixa dubitatum (9).jpg	fold4	3	*Amblyomma*
1886	Low resolution	AlexNet	baixa ovale (10).jpg	fold4	4	*Amblyomma*
1887	Low resolution	AlexNet	baixa ovale (13).jpg	fold4	4	*Amblyomma*
1888	Low resolution	AlexNet	baixa ovale (17).jpg	fold4	4	*Amblyomma*
1889	Low resolution	AlexNet	baixa ovale (18).jpg	fold4	4	*Amblyomma*
1890	Low resolution	AlexNet	baixa ovale (19).jpg	fold4	4	*Amblyomma*
1891	Low resolution	AlexNet	baixa ovale (38).jpg	fold4	4	*Amblyomma*
1892	Low resolution	AlexNet	baixa ovale (4).jpg	fold4	4	*Amblyomma*
1893	Low resolution	AlexNet	baixa ovale (40).jpg	fold4	4	*Amblyomma*
1894	Low resolution	AlexNet	baixa ovale (51).jpg	fold4	4	*Amblyomma*
1895	Low resolution	AlexNet	baixa ovale (53).jpg	fold4	4	*Amblyomma*
1896	Low resolution	AlexNet	baixa ovale (7).jpg	fold4	4	*Amblyomma*
1897	Low resolution	AlexNet	baixa sculptum (15).jpg	fold4	5	*Amblyomma*
1898	Low resolution	AlexNet	baixa sculptum (16).jpg	fold4	5	*Amblyomma*
1899	Low resolution	AlexNet	baixa sculptum (2).jpg	fold4	5	*Amblyomma*
1900	Low resolution	AlexNet	baixa triste (18).jpg	fold4	6	*Amblyomma*
1901	Low resolution	AlexNet	baixa triste (19).jpg	fold4	6	*Amblyomma*
1902	Low resolution	AlexNet	baixa triste (45).jpg	fold4	6	*Amblyomma*
1903	Low resolution	AlexNet	baixa triste (46).jpg	fold4	6	*Amblyomma*
1904	Low resolution	AlexNet	baixa triste (5).jpg	fold4	6	*Amblyomma*
1905	Low resolution	AlexNet	baixa triste (51).jpg	fold4	6	*Amblyomma*
1906	Low resolution	AlexNet	baixa triste (54).jpg	fold4	6	*Amblyomma*
1907	Low resolution	AlexNet	baixa triste (6).jpg	fold4	6	*Amblyomma*
1908	Low resolution	AlexNet	baixa triste (60).jpg	fold4	6	*Amblyomma*
1909	Low resolution	AlexNet	baixa triste (62).jpg	fold4	6	*Amblyomma*
1910	Low resolution	AlexNet	baixa triste (67).jpg	fold4	6	*Amblyomma*
1911	Low resolution	AlexNet	baixa triste (68).jpg	fold4	6	*Amblyomma*
1912	Low resolution	AlexNet	baixa triste (70).jpg	fold4	6	*Amblyomma*
1913	Low resolution	AlexNet	baixa triste (72).jpg	fold4	6	*Amblyomma*
1914	Low resolution	AlexNet	baixa triste (8).jpg	fold4	6	*Amblyomma*
1915	Low resolution	AlexNet	baixa aureolatum (10).jpg	fold5	1	*Amblyomma*
1916	Low resolution	AlexNet	baixa aureolatum (2).jpg	fold5	1	*Amblyomma*
1917	Low resolution	AlexNet	baixa aureolatum (3).jpg	fold5	1	*Amblyomma*
1918	Low resolution	AlexNet	baixa aureolatum (31).jpg	fold5	1	*Amblyomma*
1919	Low resolution	AlexNet	baixa aureolatum (34).jpg	fold5	1	*Amblyomma*
1920	Low resolution	AlexNet	baixa aureolatum (39).jpg	fold5	1	*Amblyomma*
1921	Low resolution	AlexNet	baixa aureolatum (41).jpg	fold5	1	*Amblyomma*
1922	Low resolution	AlexNet	baixa aureolatum (42).jpg	fold5	1	*Amblyomma*
1923	Low resolution	AlexNet	baixa aureolatum (45).jpg	fold5	1	*Amblyomma*
1924	Low resolution	AlexNet	baixa aureolatum (50).jpg	fold5	1	*Amblyomma*
1925	Low resolution	AlexNet	baixa aureolatum (9).jpg	fold5	1	*Amblyomma*
1926	Low resolution	AlexNet	baixa cajennense (12).jpg	fold5	2	*Amblyomma*
1927	Low resolution	AlexNet	baixa cajennense (24).jpg	fold5	2	*Amblyomma*
1928	Low resolution	AlexNet	baixa cajennense (25).jpg	fold5	2	*Amblyomma*
1929	Low resolution	AlexNet	baixa cajennense (28).jpg	fold5	2	*Amblyomma*
1930	Low resolution	AlexNet	baixa cajennense (32).jpg	fold5	2	*Amblyomma*
1931	Low resolution	AlexNet	baixa cajennense (33).jpg	fold5	2	*Amblyomma*
1932	Low resolution	AlexNet	baixa cajennense (36).jpg	fold5	2	*Amblyomma*
1933	Low resolution	AlexNet	baixa cajennense (40).jpg	fold5	2	*Amblyomma*
1934	Low resolution	AlexNet	baixa cajennense (46).jpg	fold5	2	*Amblyomma*
1935	Low resolution	AlexNet	baixa cajennense (52).jpg	fold5	2	*Amblyomma*
1936	Low resolution	AlexNet	baixa cajennense (58).jpg	fold5	2	*Amblyomma*
1937	Low resolution	AlexNet	baixa cajennense (60).jpg	fold5	2	*Amblyomma*
1938	Low resolution	AlexNet	baixa cajennense (68).jpg	fold5	2	*Amblyomma*
1939	Low resolution	AlexNet	baixa cajennense (70).jpg	fold5	2	*Amblyomma*
1940	Low resolution	AlexNet	baixa cajennense (78).jpg	fold5	2	*Amblyomma*
1941	Low resolution	AlexNet	baixa cajennense (82).jpg	fold5	2	*Amblyomma*

1942	Low resolution	AlexNet	baixa cajennense (9).jpg	fold5	2	*Amblyomma*
1943	Low resolution	AlexNet	baixa dubitatum (11).jpg	fold5	3	*Amblyomma*
1944	Low resolution	AlexNet	baixa dubitatum (12).jpg	fold5	3	*Amblyomma*
1945	Low resolution	AlexNet	baixa dubitatum (16).jpg	fold5	3	*Amblyomma*
1946	Low resolution	AlexNet	baixa dubitatum (19).jpg	fold5	3	*Amblyomma*
1947	Low resolution	AlexNet	baixa dubitatum (24).jpg	fold5	3	*Amblyomma*
1948	Low resolution	AlexNet	baixa dubitatum (25).jpg	fold5	3	*Amblyomma*
1949	Low resolution	AlexNet	baixa dubitatum (3).jpg	fold5	3	*Amblyomma*
1950	Low resolution	AlexNet	baixa dubitatum (44).jpg	fold5	3	*Amblyomma*
1951	Low resolution	AlexNet	baixa dubitatum (6).jpg	fold5	3	*Amblyomma*
1952	Low resolution	AlexNet	baixa ovale (1).jpg	fold5	4	*Amblyomma*
1953	Low resolution	AlexNet	baixa ovale (12).jpg	fold5	4	*Amblyomma*
1954	Low resolution	AlexNet	baixa ovale (15).jpg	fold5	4	*Amblyomma*
1955	Low resolution	AlexNet	baixa ovale (16).jpg	fold5	4	*Amblyomma*
1956	Low resolution	AlexNet	baixa ovale (23).jpg	fold5	4	*Amblyomma*
1957	Low resolution	AlexNet	baixa ovale (3).jpg	fold5	4	*Amblyomma*
1958	Low resolution	AlexNet	baixa ovale (32).jpg	fold5	4	*Amblyomma*
1959	Low resolution	AlexNet	baixa ovale (33).jpg	fold5	4	*Amblyomma*
1960	Low resolution	AlexNet	baixa ovale (49).jpg	fold5	4	*Amblyomma*
1961	Low resolution	AlexNet	baixa ovale (50).jpg	fold5	4	*Amblyomma*
1962	Low resolution	AlexNet	baixa ovale (6).jpg	fold5	4	*Amblyomma*
1963	Low resolution	AlexNet	baixa sculptum (10).jpg	fold5	5	*Amblyomma*
1964	Low resolution	AlexNet	baixa sculptum (14).jpg	fold5	5	*Amblyomma*
1965	Low resolution	AlexNet	baixa sculptum (4).jpg	fold5	5	*Amblyomma*
1966	Low resolution	AlexNet	baixa triste (20).jpg	fold5	6	*Amblyomma*
1967	Low resolution	AlexNet	baixa triste (22).jpg	fold5	6	*Amblyomma*
1968	Low resolution	AlexNet	baixa triste (27).jpg	fold5	6	*Amblyomma*
1969	Low resolution	AlexNet	baixa triste (29).jpg	fold5	6	*Amblyomma*
1970	Low resolution	AlexNet	baixa triste (3).jpg	fold5	6	*Amblyomma*
1971	Low resolution	AlexNet	baixa triste (31).jpg	fold5	6	*Amblyomma*
1972	Low resolution	AlexNet	baixa triste (35).jpg	fold5	6	*Amblyomma*
1973	Low resolution	AlexNet	baixa triste (43).jpg	fold5	6	*Amblyomma*
1974	Low resolution	AlexNet	baixa triste (44).jpg	fold5	6	*Amblyomma*
1975	Low resolution	AlexNet	baixa triste (49).jpg	fold5	6	*Amblyomma*
1976	Low resolution	AlexNet	baixa triste (56).jpg	fold5	6	*Amblyomma*
1977	Low resolution	AlexNet	baixa triste (58).jpg	fold5	6	*Amblyomma*
1978	Low resolution	AlexNet	baixa triste (59).jpg	fold5	6	*Amblyomma*
1979	Low resolution	AlexNet	baixa triste (65).jpg	fold5	6	*Amblyomma*
1980	Low resolution	AlexNet	baixa triste (9).jpg	fold5	6	*Amblyomma*
1981	High resolution	AlexNet	alta aureolatum (11).jpg	fold1	1	*Amblyomma*
1982	High resolution	AlexNet	alta aureolatum (17).jpg	fold1	1	*Amblyomma*
1983	High resolution	AlexNet	alta aureolatum (23).jpg	fold1	1	*Amblyomma*
1984	High resolution	AlexNet	alta aureolatum (27).jpg	fold1	1	*Amblyomma*
1985	High resolution	AlexNet	alta aureolatum (30).jpg	fold1	1	*Amblyomma*
1986	High resolution	AlexNet	alta aureolatum (46).jpg	fold1	1	*Amblyomma*
1987	High resolution	AlexNet	alta aureolatum (47).jpg	fold1	1	*Amblyomma*
1988	High resolution	AlexNet	alta aureolatum (48).jpg	fold1	1	*Amblyomma*
1989	High resolution	AlexNet	alta aureolatum (52).jpg	fold1	1	*Amblyomma*
1990	High resolution	AlexNet	alta aureolatum (55).jpg	fold1	1	*Amblyomma*
1991	High resolution	AlexNet	alta aureolatum (58).jpg	fold1	1	*Amblyomma*
1992	High resolution	AlexNet	alta aureolatum (61).jpg	fold1	1	*Amblyomma*
1993	High resolution	AlexNet	alta aureolatum (65).jpg	fold1	1	*Amblyomma*
1994	High resolution	AlexNet	alta cajennense (11).jpg	fold1	2	*Amblyomma*
1995	High resolution	AlexNet	alta cajennense (16).jpg	fold1	2	*Amblyomma*
1996	High resolution	AlexNet	alta cajennense (29).jpg	fold1	2	*Amblyomma*
1997	High resolution	AlexNet	alta cajennense (3).jpg	fold1	2	*Amblyomma*
1998	High resolution	AlexNet	alta cajennense (31).jpg	fold1	2	*Amblyomma*
1999	High resolution	AlexNet	alta cajennense (33).jpg	fold1	2	*Amblyomma*
2000	High resolution	AlexNet	alta cajennense (35).jpg	fold1	2	*Amblyomma*
2001	High resolution	AlexNet	alta cajennense (40).jpg	fold1	2	*Amblyomma*
2002	High resolution	AlexNet	alta cajennense (42).jpg	fold1	2	*Amblyomma*
2003	High resolution	AlexNet	alta cajennense (55).jpg	fold1	2	*Amblyomma*
2004	High resolution	AlexNet	alta cajennense (59).jpg	fold1	2	*Amblyomma*
2005	High resolution	AlexNet	alta cajennense (6).jpg	fold1	2	*Amblyomma*
2006	High resolution	AlexNet	alta cajennense (68).jpg	fold1	2	*Amblyomma*
2007	High resolution	AlexNet	alta cajennense (71).jpg	fold1	2	*Amblyomma*
2008	High resolution	AlexNet	alta cajennense (73).jpg	fold1	2	*Amblyomma*

2009	High resolution	AlexNet	alta cajennense (74).jpg	fold1	2	*Amblyomma*
2010	High resolution	AlexNet	alta cajennense (80).jpg	fold1	2	*Amblyomma*
2011	High resolution	AlexNet	alta dubitatum (11).jpg	fold1	3	*Amblyomma*
2012	High resolution	AlexNet	alta dubitatum (19).jpg	fold1	3	*Amblyomma*
2013	High resolution	AlexNet	alta dubitatum (2).jpg	fold1	3	*Amblyomma*
2014	High resolution	AlexNet	alta dubitatum (20).jpg	fold1	3	*Amblyomma*
2015	High resolution	AlexNet	alta dubitatum (24).jpg	fold1	3	*Amblyomma*
2016	High resolution	AlexNet	alta dubitatum (26).jpg	fold1	3	*Amblyomma*
2017	High resolution	AlexNet	alta dubitatum (29).jpg	fold1	3	*Amblyomma*
2018	High resolution	AlexNet	alta dubitatum (3).jpg	fold1	3	*Amblyomma*
2019	High resolution	AlexNet	alta dubitatum (32).jpg	fold1	3	*Amblyomma*
2020	High resolution	AlexNet	alta dubitatum (40).jpg	fold1	3	*Amblyomma*
2021	High resolution	AlexNet	alta dubitatum (48).jpg	fold1	3	*Amblyomma*
2022	High resolution	AlexNet	alta dubitatum (49).jpg	fold1	3	*Amblyomma*
2023	High resolution	AlexNet	alta dubitatum (9).JPG	fold1	3	*Amblyomma*
2024	High resolution	AlexNet	alta ovale (12).jpg	fold1	4	*Amblyomma*
2025	High resolution	AlexNet	alta ovale (19).jpg	fold1	4	*Amblyomma*
2026	High resolution	AlexNet	alta ovale (2).jpg	fold1	4	*Amblyomma*
2027	High resolution	AlexNet	alta ovale (23).jpg	fold1	4	*Amblyomma*
2028	High resolution	AlexNet	alta ovale (24).jpg	fold1	4	*Amblyomma*
2029	High resolution	AlexNet	alta ovale (26).jpg	fold1	4	*Amblyomma*
2030	High resolution	AlexNet	alta ovale (42).jpg	fold1	4	*Amblyomma*
2031	High resolution	AlexNet	alta ovale (51).jpg	fold1	4	*Amblyomma*
2032	High resolution	AlexNet	alta ovale (52).jpg	fold1	4	*Amblyomma*
2033	High resolution	AlexNet	alta ovale (54).jpg	fold1	4	*Amblyomma*
2034	High resolution	AlexNet	alta ovale (58).JPG	fold1	4	*Amblyomma*
2035	High resolution	AlexNet	alta ovale (62).jpg	fold1	4	*Amblyomma*
2036	High resolution	AlexNet	alta ovale (66).jpg	fold1	4	*Amblyomma*
2037	High resolution	AlexNet	alta sculptum (100).JPG	fold1	5	*Amblyomma*
2038	High resolution	AlexNet	alta sculptum (109).JPG	fold1	5	*Amblyomma*
2039	High resolution	AlexNet	alta sculptum (11).JPG	fold1	5	*Amblyomma*
2040	High resolution	AlexNet	alta sculptum (110).JPG	fold1	5	*Amblyomma*
2041	High resolution	AlexNet	alta sculptum (114).JPG	fold1	5	*Amblyomma*
2042	High resolution	AlexNet	alta sculptum (121).JPG	fold1	5	*Amblyomma*
2043	High resolution	AlexNet	alta sculptum (123).JPG	fold1	5	*Amblyomma*
2044	High resolution	AlexNet	alta sculptum (124).JPG	fold1	5	*Amblyomma*
2045	High resolution	AlexNet	alta sculptum (130).JPG	fold1	5	*Amblyomma*
2046	High resolution	AlexNet	alta sculptum (19).JPG	fold1	5	*Amblyomma*
2047	High resolution	AlexNet	alta sculptum (2).jpg	fold1	5	*Amblyomma*
2048	High resolution	AlexNet	alta sculptum (21).JPG	fold1	5	*Amblyomma*
2049	High resolution	AlexNet	alta sculptum (22).JPG	fold1	5	*Amblyomma*
2050	High resolution	AlexNet	alta sculptum (26).JPG	fold1	5	*Amblyomma*
2051	High resolution	AlexNet	alta sculptum (3).jpg	fold1	5	*Amblyomma*
2052	High resolution	AlexNet	alta sculptum (36).JPG	fold1	5	*Amblyomma*
2053	High resolution	AlexNet	alta sculptum (49).JPG	fold1	5	*Amblyomma*
2054	High resolution	AlexNet	alta sculptum (54).JPG	fold1	5	*Amblyomma*
2055	High resolution	AlexNet	alta sculptum (57).jpg	fold1	5	*Amblyomma*
2056	High resolution	AlexNet	alta sculptum (58).jpg	fold1	5	*Amblyomma*
2057	High resolution	AlexNet	alta sculptum (6).jpg	fold1	5	*Amblyomma*
2058	High resolution	AlexNet	alta sculptum (65).JPG	fold1	5	*Amblyomma*
2059	High resolution	AlexNet	alta sculptum (66).JPG	fold1	5	*Amblyomma*
2060	High resolution	AlexNet	alta sculptum (75).JPG	fold1	5	*Amblyomma*
2061	High resolution	AlexNet	alta sculptum (79).JPG	fold1	5	*Amblyomma*
2062	High resolution	AlexNet	alta sculptum (80).JPG	fold1	5	*Amblyomma*
2063	High resolution	AlexNet	alta sculptum (94).JPG	fold1	5	*Amblyomma*
2064	High resolution	AlexNet	alta triste (1).jpg	fold1	6	*Amblyomma*
2065	High resolution	AlexNet	alta triste (14).jpg	fold1	6	*Amblyomma*
2066	High resolution	AlexNet	alta triste (16).jpg	fold1	6	*Amblyomma*
2067	High resolution	AlexNet	alta triste (27).jpg	fold1	6	*Amblyomma*
2068	High resolution	AlexNet	alta triste (29).jpg	fold1	6	*Amblyomma*
2069	High resolution	AlexNet	alta triste (4).jpg	fold1	6	*Amblyomma*
2070	High resolution	AlexNet	alta triste (48).jpg	fold1	6	*Amblyomma*
2071	High resolution	AlexNet	alta triste (51).jpg	fold1	6	*Amblyomma*
2072	High resolution	AlexNet	alta triste (52).jpg	fold1	6	*Amblyomma*
2073	High resolution	AlexNet	alta triste (57).jpg	fold1	6	*Amblyomma*
2074	High resolution	AlexNet	alta triste (60).jpg	fold1	6	*Amblyomma*
2075	High resolution	AlexNet	alta triste (69).jpg	fold1	6	*Amblyomma*

2076	High resolution	AlexNet	alta triste (76).jpg	fold1	6	*Amblyomma*
2077	High resolution	AlexNet	alta triste (78).jpg	fold1	6	*Amblyomma*
2078	High resolution	AlexNet	alta triste (79).jpg	fold1	6	*Amblyomma*
2079	High resolution	AlexNet	alta triste (8).jpg	fold1	6	*Amblyomma*
2080	High resolution	AlexNet	alta aureolatum (18).jpg	fold2	1	*Amblyomma*
2081	High resolution	AlexNet	alta aureolatum (21).jpg	fold2	1	*Amblyomma*
2082	High resolution	AlexNet	alta aureolatum (28).jpg	fold2	1	*Amblyomma*
2083	High resolution	AlexNet	alta aureolatum (29).jpg	fold2	1	*Amblyomma*
2084	High resolution	AlexNet	alta aureolatum (32).jpg	fold2	1	*Amblyomma*
2085	High resolution	AlexNet	alta aureolatum (38).jpg	fold2	1	*Amblyomma*
2086	High resolution	AlexNet	alta aureolatum (43).jpg	fold2	1	*Amblyomma*
2087	High resolution	AlexNet	alta aureolatum (44).jpg	fold2	1	*Amblyomma*
2088	High resolution	AlexNet	alta aureolatum (49).jpg	fold2	1	*Amblyomma*
2089	High resolution	AlexNet	alta aureolatum (5).jpg	fold2	1	*Amblyomma*
2090	High resolution	AlexNet	alta aureolatum (51).jpg	fold2	1	*Amblyomma*
2091	High resolution	AlexNet	alta aureolatum (6).jpg	fold2	1	*Amblyomma*
2092	High resolution	AlexNet	alta aureolatum (62).jpg	fold2	1	*Amblyomma*
2093	High resolution	AlexNet	alta aureolatum (9).jpg	fold2	1	*Amblyomma*
2094	High resolution	AlexNet	alta cajennense (12).jpg	fold2	2	*Amblyomma*
2095	High resolution	AlexNet	alta cajennense (15).jpg	fold2	2	*Amblyomma*
2096	High resolution	AlexNet	alta cajennense (24).jpg	fold2	2	*Amblyomma*
2097	High resolution	AlexNet	alta cajennense (30).jpg	fold2	2	*Amblyomma*
2098	High resolution	AlexNet	alta cajennense (41).jpg	fold2	2	*Amblyomma*
2099	High resolution	AlexNet	alta cajennense (43).jpg	fold2	2	*Amblyomma*
2100	High resolution	AlexNet	alta cajennense (45).jpg	fold2	2	*Amblyomma*
2101	High resolution	AlexNet	alta cajennense (49).jpg	fold2	2	*Amblyomma*
2102	High resolution	AlexNet	alta cajennense (51).jpg	fold2	2	*Amblyomma*
2103	High resolution	AlexNet	alta cajennense (52).jpg	fold2	2	*Amblyomma*
2104	High resolution	AlexNet	alta cajennense (56).jpg	fold2	2	*Amblyomma*
2105	High resolution	AlexNet	alta cajennense (58).jpg	fold2	2	*Amblyomma*
2106	High resolution	AlexNet	alta cajennense (62).jpg	fold2	2	*Amblyomma*
2107	High resolution	AlexNet	alta cajennense (66).jpg	fold2	2	*Amblyomma*
2108	High resolution	AlexNet	alta cajennense (77).jpg	fold2	2	*Amblyomma*
2109	High resolution	AlexNet	alta cajennense (78).jpg	fold2	2	*Amblyomma*
2110	High resolution	AlexNet	alta cajennense (81).jpg	fold2	2	*Amblyomma*
2111	High resolution	AlexNet	alta dubitatum (21).jpg	fold2	3	*Amblyomma*
2112	High resolution	AlexNet	alta dubitatum (22).jpg	fold2	3	*Amblyomma*
2113	High resolution	AlexNet	alta dubitatum (33).jpg	fold2	3	*Amblyomma*
2114	High resolution	AlexNet	alta dubitatum (34).jpg	fold2	3	*Amblyomma*
2115	High resolution	AlexNet	alta dubitatum (35).jpg	fold2	3	*Amblyomma*
2116	High resolution	AlexNet	alta dubitatum (37).jpg	fold2	3	*Amblyomma*
2117	High resolution	AlexNet	alta dubitatum (38).jpg	fold2	3	*Amblyomma*
2118	High resolution	AlexNet	alta dubitatum (43).jpg	fold2	3	*Amblyomma*
2119	High resolution	AlexNet	alta dubitatum (47).jpg	fold2	3	*Amblyomma*
2120	High resolution	AlexNet	alta dubitatum (51).jpg	fold2	3	*Amblyomma*
2121	High resolution	AlexNet	alta dubitatum (58).jpg	fold2	3	*Amblyomma*
2122	High resolution	AlexNet	alta dubitatum (60).jpg	fold2	3	*Amblyomma*
2123	High resolution	AlexNet	alta dubitatum (64).jpg	fold2	3	*Amblyomma*
2124	High resolution	AlexNet	alta ovale (18).jpg	fold2	4	*Amblyomma*
2125	High resolution	AlexNet	alta ovale (27).jpg	fold2	4	*Amblyomma*
2126	High resolution	AlexNet	alta ovale (28).jpg	fold2	4	*Amblyomma*
2127	High resolution	AlexNet	alta ovale (38).jpg	fold2	4	*Amblyomma*
2128	High resolution	AlexNet	alta ovale (4).jpg	fold2	4	*Amblyomma*
2129	High resolution	AlexNet	alta ovale (41).jpg	fold2	4	*Amblyomma*
2130	High resolution	AlexNet	alta ovale (43).jpg	fold2	4	*Amblyomma*
2131	High resolution	AlexNet	alta ovale (44).jpg	fold2	4	*Amblyomma*
2132	High resolution	AlexNet	alta ovale (48).jpg	fold2	4	*Amblyomma*
2133	High resolution	AlexNet	alta ovale (50).jpg	fold2	4	*Amblyomma*
2134	High resolution	AlexNet	alta ovale (55).jpg	fold2	4	*Amblyomma*
2135	High resolution	AlexNet	alta ovale (65).jpg	fold2	4	*Amblyomma*
2136	High resolution	AlexNet	alta ovale (9).jpg	fold2	4	*Amblyomma*
2137	High resolution	AlexNet	alta sculptum (111).JPG	fold2	5	*Amblyomma*
2138	High resolution	AlexNet	alta sculptum (115).JPG	fold2	5	*Amblyomma*
2139	High resolution	AlexNet	alta sculptum (12).JPG	fold2	5	*Amblyomma*
2140	High resolution	AlexNet	alta sculptum (125).JPG	fold2	5	*Amblyomma*
2141	High resolution	AlexNet	alta sculptum (126).JPG	fold2	5	*Amblyomma*
2142	High resolution	AlexNet	alta sculptum (128).JPG	fold2	5	*Amblyomma*

2143	High resolution	AlexNet	alta sculptum (132).JPG	fold2	5	*Amblyomma*
2144	High resolution	AlexNet	alta sculptum (138).JPG	fold2	5	*Amblyomma*
2145	High resolution	AlexNet	alta sculptum (27).JPG	fold2	5	*Amblyomma*
2146	High resolution	AlexNet	alta sculptum (32).JPG	fold2	5	*Amblyomma*
2147	High resolution	AlexNet	alta sculptum (33).JPG	fold2	5	*Amblyomma*
2148	High resolution	AlexNet	alta sculptum (38).JPG	fold2	5	*Amblyomma*
2149	High resolution	AlexNet	alta sculptum (39).JPG	fold2	5	*Amblyomma*
2150	High resolution	AlexNet	alta sculptum (40).JPG	fold2	5	*Amblyomma*
2151	High resolution	AlexNet	alta sculptum (41).JPG	fold2	5	*Amblyomma*
2152	High resolution	AlexNet	alta sculptum (43).JPG	fold2	5	*Amblyomma*
2153	High resolution	AlexNet	alta sculptum (47).JPG	fold2	5	*Amblyomma*
2154	High resolution	AlexNet	alta sculptum (52).JPG	fold2	5	*Amblyomma*
2155	High resolution	AlexNet	alta sculptum (56).jpg	fold2	5	*Amblyomma*
2156	High resolution	AlexNet	alta sculptum (67).JPG	fold2	5	*Amblyomma*
2157	High resolution	AlexNet	alta sculptum (74).JPG	fold2	5	*Amblyomma*
2158	High resolution	AlexNet	alta sculptum (8).jpg	fold2	5	*Amblyomma*
2159	High resolution	AlexNet	alta sculptum (81).JPG	fold2	5	*Amblyomma*
2160	High resolution	AlexNet	alta sculptum (9).JPG	fold2	5	*Amblyomma*
2161	High resolution	AlexNet	alta sculptum (91).JPG	fold2	5	*Amblyomma*
2162	High resolution	AlexNet	alta sculptum (95).JPG	fold2	5	*Amblyomma*
2163	High resolution	AlexNet	alta sculptum (96).JPG	fold2	5	*Amblyomma*
2164	High resolution	AlexNet	alta sculptum (99).JPG	fold2	5	*Amblyomma*
2165	High resolution	AlexNet	alta triste (2).jpg	fold2	6	*Amblyomma*
2166	High resolution	AlexNet	alta triste (25).jpg	fold2	6	*Amblyomma*
2167	High resolution	AlexNet	alta triste (33).jpg	fold2	6	*Amblyomma*
2168	High resolution	AlexNet	alta triste (36).jpg	fold2	6	*Amblyomma*
2169	High resolution	AlexNet	alta triste (37).jpg	fold2	6	*Amblyomma*
2170	High resolution	AlexNet	alta triste (38).jpg	fold2	6	*Amblyomma*
2171	High resolution	AlexNet	alta triste (39).jpg	fold2	6	*Amblyomma*
2172	High resolution	AlexNet	alta triste (41).jpg	fold2	6	*Amblyomma*
2173	High resolution	AlexNet	alta triste (47).jpg	fold2	6	*Amblyomma*
2174	High resolution	AlexNet	alta triste (5).jpg	fold2	6	*Amblyomma*
2175	High resolution	AlexNet	alta triste (54).jpg	fold2	6	*Amblyomma*
2176	High resolution	AlexNet	alta triste (61).jpg	fold2	6	*Amblyomma*
2177	High resolution	AlexNet	alta triste (63).jpg	fold2	6	*Amblyomma*
2178	High resolution	AlexNet	alta triste (65).jpg	fold2	6	*Amblyomma*
2179	High resolution	AlexNet	alta triste (67).jpg	fold2	6	*Amblyomma*
2180	High resolution	AlexNet	alta triste (77).jpg	fold2	6	*Amblyomma*
2181	High resolution	AlexNet	alta aureolatum (1).jpg	fold3	1	*Amblyomma*
2182	High resolution	AlexNet	alta aureolatum (14).jpg	fold3	1	*Amblyomma*
2183	High resolution	AlexNet	alta aureolatum (19).jpg	fold3	1	*Amblyomma*
2184	High resolution	AlexNet	alta aureolatum (24).jpg	fold3	1	*Amblyomma*
2185	High resolution	AlexNet	alta aureolatum (31).jpg	fold3	1	*Amblyomma*
2186	High resolution	AlexNet	alta aureolatum (33).jpg	fold3	1	*Amblyomma*
2187	High resolution	AlexNet	alta aureolatum (35).jpg	fold3	1	*Amblyomma*
2188	High resolution	AlexNet	alta aureolatum (36).jpg	fold3	1	*Amblyomma*
2189	High resolution	AlexNet	alta aureolatum (40).jpg	fold3	1	*Amblyomma*
2190	High resolution	AlexNet	alta aureolatum (53).jpg	fold3	1	*Amblyomma*
2191	High resolution	AlexNet	alta aureolatum (54).jpg	fold3	1	*Amblyomma*
2192	High resolution	AlexNet	alta aureolatum (66).jpg	fold3	1	*Amblyomma*
2193	High resolution	AlexNet	alta aureolatum (67).jpg	fold3	1	*Amblyomma*
2194	High resolution	AlexNet	alta cajennense (1).jpg	fold3	2	*Amblyomma*
2195	High resolution	AlexNet	alta cajennense (13).jpg	fold3	2	*Amblyomma*
2196	High resolution	AlexNet	alta cajennense (14).jpg	fold3	2	*Amblyomma*
2197	High resolution	AlexNet	alta cajennense (17).jpg	fold3	2	*Amblyomma*
2198	High resolution	AlexNet	alta cajennense (18).jpg	fold3	2	*Amblyomma*
2199	High resolution	AlexNet	alta cajennense (2).jpg	fold3	2	*Amblyomma*
2200	High resolution	AlexNet	alta cajennense (23).jpg	fold3	2	*Amblyomma*
2201	High resolution	AlexNet	alta cajennense (26).jpg	fold3	2	*Amblyomma*
2202	High resolution	AlexNet	alta cajennense (34).jpg	fold3	2	*Amblyomma*
2203	High resolution	AlexNet	alta cajennense (36).jpg	fold3	2	*Amblyomma*
2204	High resolution	AlexNet	alta cajennense (37).jpg	fold3	2	*Amblyomma*
2205	High resolution	AlexNet	alta cajennense (38).jpg	fold3	2	*Amblyomma*
2206	High resolution	AlexNet	alta cajennense (50).jpg	fold3	2	*Amblyomma*
2207	High resolution	AlexNet	alta cajennense (63).jpg	fold3	2	*Amblyomma*
2208	High resolution	AlexNet	alta cajennense (64).jpg	fold3	2	*Amblyomma*
2209	High resolution	AlexNet	alta cajennense (67).jpg	fold3	2	*Amblyomma*

2210	High resolution	AlexNet	alta cajennense (9).jpg	fold3	2	*Amblyomma*
2211	High resolution	AlexNet	alta dubitatum (1).jpg	fold3	3	*Amblyomma*
2212	High resolution	AlexNet	alta dubitatum (10).JPG	fold3	3	*Amblyomma*
2213	High resolution	AlexNet	alta dubitatum (15).jpg	fold3	3	*Amblyomma*
2214	High resolution	AlexNet	alta dubitatum (31).jpg	fold3	3	*Amblyomma*
2215	High resolution	AlexNet	alta dubitatum (39).jpg	fold3	3	*Amblyomma*
2216	High resolution	AlexNet	alta dubitatum (4).jpg	fold3	3	*Amblyomma*
2217	High resolution	AlexNet	alta dubitatum (42).jpg	fold3	3	*Amblyomma*
2218	High resolution	AlexNet	alta dubitatum (45).jpg	fold3	3	*Amblyomma*
2219	High resolution	AlexNet	alta dubitatum (46).jpg	fold3	3	*Amblyomma*
2220	High resolution	AlexNet	alta dubitatum (53).jpg	fold3	3	*Amblyomma*
2221	High resolution	AlexNet	alta dubitatum (6).jpg	fold3	3	*Amblyomma*
2222	High resolution	AlexNet	alta dubitatum (62).jpg	fold3	3	*Amblyomma*
2223	High resolution	AlexNet	alta dubitatum (63).jpg	fold3	3	*Amblyomma*
2224	High resolution	AlexNet	alta ovale (15).jpg	fold3	4	*Amblyomma*
2225	High resolution	AlexNet	alta ovale (17).jpg	fold3	4	*Amblyomma*
2226	High resolution	AlexNet	alta ovale (22).jpg	fold3	4	*Amblyomma*
2227	High resolution	AlexNet	alta ovale (30).jpg	fold3	4	*Amblyomma*
2228	High resolution	AlexNet	alta ovale (32).jpg	fold3	4	*Amblyomma*
2229	High resolution	AlexNet	alta ovale (33).jpg	fold3	4	*Amblyomma*
2230	High resolution	AlexNet	alta ovale (34).jpg	fold3	4	*Amblyomma*
2231	High resolution	AlexNet	alta ovale (45).jpg	fold3	4	*Amblyomma*
2232	High resolution	AlexNet	alta ovale (46).jpg	fold3	4	*Amblyomma*
2233	High resolution	AlexNet	alta ovale (53).jpg	fold3	4	*Amblyomma*
2234	High resolution	AlexNet	alta ovale (56).jpg	fold3	4	*Amblyomma*
2235	High resolution	AlexNet	alta ovale (57).JPG	fold3	4	*Amblyomma*
2236	High resolution	AlexNet	alta ovale (60).jpg	fold3	4	*Amblyomma*
2237	High resolution	AlexNet	alta ovale (64).jpg	fold3	4	*Amblyomma*
2238	High resolution	AlexNet	alta sculptum (10).JPG	fold3	5	*Amblyomma*
2239	High resolution	AlexNet	alta sculptum (101).JPG	fold3	5	*Amblyomma*
2240	High resolution	AlexNet	alta sculptum (104).JPG	fold3	5	*Amblyomma*
2241	High resolution	AlexNet	alta sculptum (118).JPG	fold3	5	*Amblyomma*
2242	High resolution	AlexNet	alta sculptum (119).JPG	fold3	5	*Amblyomma*
2243	High resolution	AlexNet	alta sculptum (14).JPG	fold3	5	*Amblyomma*
2244	High resolution	AlexNet	alta sculptum (15).JPG	fold3	5	*Amblyomma*
2245	High resolution	AlexNet	alta sculptum (17).JPG	fold3	5	*Amblyomma*
2246	High resolution	AlexNet	alta sculptum (18).JPG	fold3	5	*Amblyomma*
2247	High resolution	AlexNet	alta sculptum (25).JPG	fold3	5	*Amblyomma*
2248	High resolution	AlexNet	alta sculptum (31).JPG	fold3	5	*Amblyomma*
2249	High resolution	AlexNet	alta sculptum (37).JPG	fold3	5	*Amblyomma*
2250	High resolution	AlexNet	alta sculptum (42).JPG	fold3	5	*Amblyomma*
2251	High resolution	AlexNet	alta sculptum (46).JPG	fold3	5	*Amblyomma*
2252	High resolution	AlexNet	alta sculptum (48).JPG	fold3	5	*Amblyomma*
2253	High resolution	AlexNet	alta sculptum (53).JPG	fold3	5	*Amblyomma*
2254	High resolution	AlexNet	alta sculptum (60).jpg	fold3	5	*Amblyomma*
2255	High resolution	AlexNet	alta sculptum (62).jpg	fold3	5	*Amblyomma*
2256	High resolution	AlexNet	alta sculptum (63).jpg	fold3	5	*Amblyomma*
2257	High resolution	AlexNet	alta sculptum (64).jpg	fold3	5	*Amblyomma*
2258	High resolution	AlexNet	alta sculptum (69).JPG	fold3	5	*Amblyomma*
2259	High resolution	AlexNet	alta sculptum (71).JPG	fold3	5	*Amblyomma*
2260	High resolution	AlexNet	alta sculptum (76).JPG	fold3	5	*Amblyomma*
2261	High resolution	AlexNet	alta sculptum (88).JPG	fold3	5	*Amblyomma*
2262	High resolution	AlexNet	alta sculptum (90).JPG	fold3	5	*Amblyomma*
2263	High resolution	AlexNet	alta sculptum (92).JPG	fold3	5	*Amblyomma*
2264	High resolution	AlexNet	alta sculptum (93).JPG	fold3	5	*Amblyomma*
2265	High resolution	AlexNet	alta sculptum (97).JPG	fold3	5	*Amblyomma*
2266	High resolution	AlexNet	alta triste (10).jpg	fold3	6	*Amblyomma*
2267	High resolution	AlexNet	alta triste (18).jpg	fold3	6	*Amblyomma*
2268	High resolution	AlexNet	alta triste (19).jpg	fold3	6	*Amblyomma*
2269	High resolution	AlexNet	alta triste (21).jpg	fold3	6	*Amblyomma*
2270	High resolution	AlexNet	alta triste (24).jpg	fold3	6	*Amblyomma*
2271	High resolution	AlexNet	alta triste (3).jpg	fold3	6	*Amblyomma*
2272	High resolution	AlexNet	alta triste (31).jpg	fold3	6	*Amblyomma*
2273	High resolution	AlexNet	alta triste (40).jpg	fold3	6	*Amblyomma*
2274	High resolution	AlexNet	alta triste (43).jpg	fold3	6	*Amblyomma*
2275	High resolution	AlexNet	alta triste (6).jpg	fold3	6	*Amblyomma*
2276	High resolution	AlexNet	alta triste (7).jpg	fold3	6	*Amblyomma*

2277	High resolution	AlexNet	alta triste (70).jpg	fold3	6	*Amblyomma*
2278	High resolution	AlexNet	alta triste (71).jpg	fold3	6	*Amblyomma*
2279	High resolution	AlexNet	alta triste (72).jpg	fold3	6	*Amblyomma*
2280	High resolution	AlexNet	alta triste (9).jpg	fold3	6	*Amblyomma*
2281	High resolution	AlexNet	alta aureolatum (12).jpg	fold4	1	*Amblyomma*
2282	High resolution	AlexNet	alta aureolatum (13).jpg	fold4	1	*Amblyomma*
2283	High resolution	AlexNet	alta aureolatum (15).jpg	fold4	1	*Amblyomma*
2284	High resolution	AlexNet	alta aureolatum (16).jpg	fold4	1	*Amblyomma*
2285	High resolution	AlexNet	alta aureolatum (20).jpg	fold4	1	*Amblyomma*
2286	High resolution	AlexNet	alta aureolatum (22).jpg	fold4	1	*Amblyomma*
2287	High resolution	AlexNet	alta aureolatum (25).jpg	fold4	1	*Amblyomma*
2288	High resolution	AlexNet	alta aureolatum (26).jpg	fold4	1	*Amblyomma*
2289	High resolution	AlexNet	alta aureolatum (37).jpg	fold4	1	*Amblyomma*
2290	High resolution	AlexNet	alta aureolatum (4).jpg	fold4	1	*Amblyomma*
2291	High resolution	AlexNet	alta aureolatum (56).jpg	fold4	1	*Amblyomma*
2292	High resolution	AlexNet	alta aureolatum (63).jpg	fold4	1	*Amblyomma*
2293	High resolution	AlexNet	alta aureolatum (64).jpg	fold4	1	*Amblyomma*
2294	High resolution	AlexNet	alta aureolatum (7).jpg	fold4	1	*Amblyomma*
2295	High resolution	AlexNet	alta cajennense (20).jpg	fold4	2	*Amblyomma*
2296	High resolution	AlexNet	alta cajennense (21).jpg	fold4	2	*Amblyomma*
2297	High resolution	AlexNet	alta cajennense (25).jpg	fold4	2	*Amblyomma*
2298	High resolution	AlexNet	alta cajennense (28).jpg	fold4	2	*Amblyomma*
2299	High resolution	AlexNet	alta cajennense (4).jpg	fold4	2	*Amblyomma*
2300	High resolution	AlexNet	alta cajennense (46).jpg	fold4	2	*Amblyomma*
2301	High resolution	AlexNet	alta cajennense (48).jpg	fold4	2	*Amblyomma*
2302	High resolution	AlexNet	alta cajennense (5).jpg	fold4	2	*Amblyomma*
2303	High resolution	AlexNet	alta cajennense (53).jpg	fold4	2	*Amblyomma*
2304	High resolution	AlexNet	alta cajennense (60).jpg	fold4	2	*Amblyomma*
2305	High resolution	AlexNet	alta cajennense (61).jpg	fold4	2	*Amblyomma*
2306	High resolution	AlexNet	alta cajennense (65).jpg	fold4	2	*Amblyomma*
2307	High resolution	AlexNet	alta cajennense (70).jpg	fold4	2	*Amblyomma*
2308	High resolution	AlexNet	alta cajennense (72).jpg	fold4	2	*Amblyomma*
2309	High resolution	AlexNet	alta cajennense (76).jpg	fold4	2	*Amblyomma*
2310	High resolution	AlexNet	alta cajennense (83).jpg	fold4	2	*Amblyomma*
2311	High resolution	AlexNet	alta dubitatum (12).jpg	fold4	3	*Amblyomma*
2312	High resolution	AlexNet	alta dubitatum (16).jpg	fold4	3	*Amblyomma*
2313	High resolution	AlexNet	alta dubitatum (17).jpg	fold4	3	*Amblyomma*
2314	High resolution	AlexNet	alta dubitatum (18).jpg	fold4	3	*Amblyomma*
2315	High resolution	AlexNet	alta dubitatum (25).jpg	fold4	3	*Amblyomma*
2316	High resolution	AlexNet	alta dubitatum (27).jpg	fold4	3	*Amblyomma*
2317	High resolution	AlexNet	alta dubitatum (30).jpg	fold4	3	*Amblyomma*
2318	High resolution	AlexNet	alta dubitatum (5).jpg	fold4	3	*Amblyomma*
2319	High resolution	AlexNet	alta dubitatum (54).jpg	fold4	3	*Amblyomma*
2320	High resolution	AlexNet	alta dubitatum (55).jpg	fold4	3	*Amblyomma*
2321	High resolution	AlexNet	alta dubitatum (56).jpg	fold4	3	*Amblyomma*
2322	High resolution	AlexNet	alta dubitatum (61).jpg	fold4	3	*Amblyomma*
2323	High resolution	AlexNet	alta dubitatum (65).jpg	fold4	3	*Amblyomma*
2324	High resolution	AlexNet	alta ovale (1).jpg	fold4	4	*Amblyomma*
2325	High resolution	AlexNet	alta ovale (10).jpg	fold4	4	*Amblyomma*
2326	High resolution	AlexNet	alta ovale (11).jpg	fold4	4	*Amblyomma*
2327	High resolution	AlexNet	alta ovale (14).jpg	fold4	4	*Amblyomma*
2328	High resolution	AlexNet	alta ovale (20).jpg	fold4	4	*Amblyomma*
2329	High resolution	AlexNet	alta ovale (21).jpg	fold4	4	*Amblyomma*
2330	High resolution	AlexNet	alta ovale (29).jpg	fold4	4	*Amblyomma*
2331	High resolution	AlexNet	alta ovale (3).jpg	fold4	4	*Amblyomma*
2332	High resolution	AlexNet	alta ovale (36).jpg	fold4	4	*Amblyomma*
2333	High resolution	AlexNet	alta ovale (37).jpg	fold4	4	*Amblyomma*
2334	High resolution	AlexNet	alta ovale (47).jpg	fold4	4	*Amblyomma*
2335	High resolution	AlexNet	alta ovale (6).jpg	fold4	4	*Amblyomma*
2336	High resolution	AlexNet	alta ovale (7).jpg	fold4	4	*Amblyomma*
2337	High resolution	AlexNet	alta sculptum (102).JPG	fold4	5	*Amblyomma*
2338	High resolution	AlexNet	alta sculptum (106).JPG	fold4	5	*Amblyomma*
2339	High resolution	AlexNet	alta sculptum (107).JPG	fold4	5	*Amblyomma*
2340	High resolution	AlexNet	alta sculptum (116).JPG	fold4	5	*Amblyomma*
2341	High resolution	AlexNet	alta sculptum (117).JPG	fold4	5	*Amblyomma*
2342	High resolution	AlexNet	alta sculptum (120).JPG	fold4	5	*Amblyomma*
2343	High resolution	AlexNet	alta sculptum (127).JPG	fold4	5	*Amblyomma*

2344	High resolution	AlexNet	alta sculptum (133).JPG	fold4	5	*Amblyomma*
2345	High resolution	AlexNet	alta sculptum (134).JPG	fold4	5	*Amblyomma*
2346	High resolution	AlexNet	alta sculptum (136).JPG	fold4	5	*Amblyomma*
2347	High resolution	AlexNet	alta sculptum (16).JPG	fold4	5	*Amblyomma*
2348	High resolution	AlexNet	alta sculptum (23).JPG	fold4	5	*Amblyomma*
2349	High resolution	AlexNet	alta sculptum (28).JPG	fold4	5	*Amblyomma*
2350	High resolution	AlexNet	alta sculptum (34).JPG	fold4	5	*Amblyomma*
2351	High resolution	AlexNet	alta sculptum (35).JPG	fold4	5	*Amblyomma*
2352	High resolution	AlexNet	alta sculptum (4).jpg	fold4	5	*Amblyomma*
2353	High resolution	AlexNet	alta sculptum (55).JPG	fold4	5	*Amblyomma*
2354	High resolution	AlexNet	alta sculptum (59).jpg	fold4	5	*Amblyomma*
2355	High resolution	AlexNet	alta sculptum (61).jpg	fold4	5	*Amblyomma*
2356	High resolution	AlexNet	alta sculptum (68).JPG	fold4	5	*Amblyomma*
2357	High resolution	AlexNet	alta sculptum (7).jpg	fold4	5	*Amblyomma*
2358	High resolution	AlexNet	alta sculptum (73).JPG	fold4	5	*Amblyomma*
2359	High resolution	AlexNet	alta sculptum (78).JPG	fold4	5	*Amblyomma*
2360	High resolution	AlexNet	alta sculptum (82).JPG	fold4	5	*Amblyomma*
2361	High resolution	AlexNet	alta sculptum (83).JPG	fold4	5	*Amblyomma*
2362	High resolution	AlexNet	alta sculptum (84).JPG	fold4	5	*Amblyomma*
2363	High resolution	AlexNet	alta sculptum (86).JPG	fold4	5	*Amblyomma*
2364	High resolution	AlexNet	alta triste (11).jpg	fold4	6	*Amblyomma*
2365	High resolution	AlexNet	alta triste (13).jpg	fold4	6	*Amblyomma*
2366	High resolution	AlexNet	alta triste (17).jpg	fold4	6	*Amblyomma*
2367	High resolution	AlexNet	alta triste (20).jpg	fold4	6	*Amblyomma*
2368	High resolution	AlexNet	alta triste (23).jpg	fold4	6	*Amblyomma*
2369	High resolution	AlexNet	alta triste (32).jpg	fold4	6	*Amblyomma*
2370	High resolution	AlexNet	alta triste (34).jpg	fold4	6	*Amblyomma*
2371	High resolution	AlexNet	alta triste (44).jpg	fold4	6	*Amblyomma*
2372	High resolution	AlexNet	alta triste (45).jpg	fold4	6	*Amblyomma*
2373	High resolution	AlexNet	alta triste (46).jpg	fold4	6	*Amblyomma*
2374	High resolution	AlexNet	alta triste (55).jpg	fold4	6	*Amblyomma*
2375	High resolution	AlexNet	alta triste (56).jpg	fold4	6	*Amblyomma*
2376	High resolution	AlexNet	alta triste (62).jpg	fold4	6	*Amblyomma*
2377	High resolution	AlexNet	alta triste (64).jpg	fold4	6	*Amblyomma*
2378	High resolution	AlexNet	alta triste (66).jpg	fold4	6	*Amblyomma*
2379	High resolution	AlexNet	alta triste (68).jpg	fold4	6	*Amblyomma*
2380	High resolution	AlexNet	alta aureolatum (10).jpg	fold5	1	*Amblyomma*
2381	High resolution	AlexNet	alta aureolatum (2).jpg	fold5	1	*Amblyomma*
2382	High resolution	AlexNet	alta aureolatum (3).jpg	fold5	1	*Amblyomma*
2383	High resolution	AlexNet	alta aureolatum (34).jpg	fold5	1	*Amblyomma*
2384	High resolution	AlexNet	alta aureolatum (39).jpg	fold5	1	*Amblyomma*
2385	High resolution	AlexNet	alta aureolatum (41).jpg	fold5	1	*Amblyomma*
2386	High resolution	AlexNet	alta aureolatum (42).jpg	fold5	1	*Amblyomma*
2387	High resolution	AlexNet	alta aureolatum (45).jpg	fold5	1	*Amblyomma*
2388	High resolution	AlexNet	alta aureolatum (50).jpg	fold5	1	*Amblyomma*
2389	High resolution	AlexNet	alta aureolatum (57).jpg	fold5	1	*Amblyomma*
2390	High resolution	AlexNet	alta aureolatum (59).jpg	fold5	1	*Amblyomma*
2391	High resolution	AlexNet	alta aureolatum (60).jpg	fold5	1	*Amblyomma*
2392	High resolution	AlexNet	alta aureolatum (68).jpg	fold5	1	*Amblyomma*
2393	High resolution	AlexNet	alta aureolatum (8).jpg	fold5	1	*Amblyomma*
2394	High resolution	AlexNet	alta cajennense (10).jpg	fold5	2	*Amblyomma*
2395	High resolution	AlexNet	alta cajennense (19).jpg	fold5	2	*Amblyomma*
2396	High resolution	AlexNet	alta cajennense (22).jpg	fold5	2	*Amblyomma*
2397	High resolution	AlexNet	alta cajennense (27).jpg	fold5	2	*Amblyomma*
2398	High resolution	AlexNet	alta cajennense (32).jpg	fold5	2	*Amblyomma*
2399	High resolution	AlexNet	alta cajennense (39).jpg	fold5	2	*Amblyomma*
2400	High resolution	AlexNet	alta cajennense (44).jpg	fold5	2	*Amblyomma*
2401	High resolution	AlexNet	alta cajennense (47).jpg	fold5	2	*Amblyomma*
2402	High resolution	AlexNet	alta cajennense (54).jpg	fold5	2	*Amblyomma*
2403	High resolution	AlexNet	alta cajennense (57).jpg	fold5	2	*Amblyomma*
2404	High resolution	AlexNet	alta cajennense (69).jpg	fold5	2	*Amblyomma*
2405	High resolution	AlexNet	alta cajennense (7).jpg	fold5	2	*Amblyomma*
2406	High resolution	AlexNet	alta cajennense (75).jpg	fold5	2	*Amblyomma*
2407	High resolution	AlexNet	alta cajennense (79).jpg	fold5	2	*Amblyomma*
2408	High resolution	AlexNet	alta cajennense (8).jpg	fold5	2	*Amblyomma*
2409	High resolution	AlexNet	alta cajennense (82).jpg	fold5	2	*Amblyomma*
2410	High resolution	AlexNet	alta dubitatum (13).jpg	fold5	3	*Amblyomma*

2411	High resolution	AlexNet	alta dubitatum (14).jpg	fold5	3	*Amblyomma*
2412	High resolution	AlexNet	alta dubitatum (23).jpg	fold5	3	*Amblyomma*
2413	High resolution	AlexNet	alta dubitatum (28).jpg	fold5	3	*Amblyomma*
2414	High resolution	AlexNet	alta dubitatum (36).jpg	fold5	3	*Amblyomma*
2415	High resolution	AlexNet	alta dubitatum (41).jpg	fold5	3	*Amblyomma*
2416	High resolution	AlexNet	alta dubitatum (44).jpg	fold5	3	*Amblyomma*
2417	High resolution	AlexNet	alta dubitatum (50).jpg	fold5	3	*Amblyomma*
2418	High resolution	AlexNet	alta dubitatum (52).jpg	fold5	3	*Amblyomma*
2419	High resolution	AlexNet	alta dubitatum (57).jpg	fold5	3	*Amblyomma*
2420	High resolution	AlexNet	alta dubitatum (59).jpg	fold5	3	*Amblyomma*
2421	High resolution	AlexNet	alta dubitatum (7).JPG	fold5	3	*Amblyomma*
2422	High resolution	AlexNet	alta dubitatum (8).JPG	fold5	3	*Amblyomma*
2423	High resolution	AlexNet	alta ovale (13).jpg	fold5	4	*Amblyomma*
2424	High resolution	AlexNet	alta ovale (16).jpg	fold5	4	*Amblyomma*
2425	High resolution	AlexNet	alta ovale (25).jpg	fold5	4	*Amblyomma*
2426	High resolution	AlexNet	alta ovale (31).jpg	fold5	4	*Amblyomma*
2427	High resolution	AlexNet	alta ovale (35).jpg	fold5	4	*Amblyomma*
2428	High resolution	AlexNet	alta ovale (39).JPG	fold5	4	*Amblyomma*
2429	High resolution	AlexNet	alta ovale (40).JPG	fold5	4	*Amblyomma*
2430	High resolution	AlexNet	alta ovale (49).jpg	fold5	4	*Amblyomma*
2431	High resolution	AlexNet	alta ovale (5).jpg	fold5	4	*Amblyomma*
2432	High resolution	AlexNet	alta ovale (59).jpg	fold5	4	*Amblyomma*
2433	High resolution	AlexNet	alta ovale (61).jpg	fold5	4	*Amblyomma*
2434	High resolution	AlexNet	alta ovale (63).jpg	fold5	4	*Amblyomma*
2435	High resolution	AlexNet	alta ovale (8).jpg	fold5	4	*Amblyomma*
2436	High resolution	AlexNet	alta sculptum (1).jpg	fold5	5	*Amblyomma*
2437	High resolution	AlexNet	alta sculptum (103).JPG	fold5	5	*Amblyomma*
2438	High resolution	AlexNet	alta sculptum (105).JPG	fold5	5	*Amblyomma*
2439	High resolution	AlexNet	alta sculptum (108).JPG	fold5	5	*Amblyomma*
2440	High resolution	AlexNet	alta sculptum (112).JPG	fold5	5	*Amblyomma*
2441	High resolution	AlexNet	alta sculptum (113).JPG	fold5	5	*Amblyomma*
2442	High resolution	AlexNet	alta sculptum (122).JPG	fold5	5	*Amblyomma*
2443	High resolution	AlexNet	alta sculptum (129).JPG	fold5	5	*Amblyomma*
2444	High resolution	AlexNet	alta sculptum (13).JPG	fold5	5	*Amblyomma*
2445	High resolution	AlexNet	alta sculptum (131).JPG	fold5	5	*Amblyomma*
2446	High resolution	AlexNet	alta sculptum (135).JPG	fold5	5	*Amblyomma*
2447	High resolution	AlexNet	alta sculptum (137).JPG	fold5	5	*Amblyomma*
2448	High resolution	AlexNet	alta sculptum (20).JPG	fold5	5	*Amblyomma*
2449	High resolution	AlexNet	alta sculptum (24).JPG	fold5	5	*Amblyomma*
2450	High resolution	AlexNet	alta sculptum (29).JPG	fold5	5	*Amblyomma*
2451	High resolution	AlexNet	alta sculptum (30).JPG	fold5	5	*Amblyomma*
2452	High resolution	AlexNet	alta sculptum (44).JPG	fold5	5	*Amblyomma*
2453	High resolution	AlexNet	alta sculptum (45).JPG	fold5	5	*Amblyomma*
2454	High resolution	AlexNet	alta sculptum (5).jpg	fold5	5	*Amblyomma*
2455	High resolution	AlexNet	alta sculptum (50).JPG	fold5	5	*Amblyomma*
2456	High resolution	AlexNet	alta sculptum (51).JPG	fold5	5	*Amblyomma*
2457	High resolution	AlexNet	alta sculptum (70).JPG	fold5	5	*Amblyomma*
2458	High resolution	AlexNet	alta sculptum (72).JPG	fold5	5	*Amblyomma*
2459	High resolution	AlexNet	alta sculptum (77).JPG	fold5	5	*Amblyomma*
2460	High resolution	AlexNet	alta sculptum (85).JPG	fold5	5	*Amblyomma*
2461	High resolution	AlexNet	alta sculptum (87).JPG	fold5	5	*Amblyomma*
2462	High resolution	AlexNet	alta sculptum (89).JPG	fold5	5	*Amblyomma*
2463	High resolution	AlexNet	alta sculptum (98).JPG	fold5	5	*Amblyomma*
2464	High resolution	AlexNet	alta triste (12).jpg	fold5	6	*Amblyomma*
2465	High resolution	AlexNet	alta triste (15).jpg	fold5	6	*Amblyomma*
2466	High resolution	AlexNet	alta triste (22).jpg	fold5	6	*Amblyomma*
2467	High resolution	AlexNet	alta triste (26).jpg	fold5	6	*Amblyomma*
2468	High resolution	AlexNet	alta triste (28).jpg	fold5	6	*Amblyomma*
2469	High resolution	AlexNet	alta triste (30).jpg	fold5	6	*Amblyomma*
2470	High resolution	AlexNet	alta triste (35).jpg	fold5	6	*Amblyomma*
2471	High resolution	AlexNet	alta triste (42).jpg	fold5	6	*Amblyomma*
2472	High resolution	AlexNet	alta triste (49).jpg	fold5	6	*Amblyomma*
2473	High resolution	AlexNet	alta triste (50).jpg	fold5	6	*Amblyomma*
2474	High resolution	AlexNet	alta triste (53).jpg	fold5	6	*Amblyomma*
2475	High resolution	AlexNet	alta triste (58).jpg	fold5	6	*Amblyomma*
2476	High resolution	AlexNet	alta triste (59).jpg	fold5	6	*Amblyomma*
2477	High resolution	AlexNet	alta triste (73).jpg	fold5	6	*Amblyomma*

2478	High resolution	AlexNet	alta triste (74).jpg	fold5	6 *Amblyomma*
2479	High resolution	AlexNet	alta triste (75).jpg	fold5	6 *Amblyomma*
2480	All together	AlexNet	todos aureolatum (100).jpg	fold1	1 *Amblyomma*
2481	All together	AlexNet	todos aureolatum (106).jpg	fold1	1 *Amblyomma*
2482	All together	AlexNet	todos aureolatum (21).jpg	fold1	1 *Amblyomma*
2483	All together	AlexNet	todos aureolatum (26).jpg	fold1	1 *Amblyomma*
2484	All together	AlexNet	todos aureolatum (27).jpg	fold1	1 *Amblyomma*
2485	All together	AlexNet	todos aureolatum (28).jpg	fold1	1 *Amblyomma*
2486	All together	AlexNet	todos aureolatum (35).jpg	fold1	1 *Amblyomma*
2487	All together	AlexNet	todos aureolatum (38).jpg	fold1	1 *Amblyomma*
2488	All together	AlexNet	todos aureolatum (41).jpg	fold1	1 *Amblyomma*
2489	All together	AlexNet	todos aureolatum (45).jpg	fold1	1 *Amblyomma*
2490	All together	AlexNet	todos aureolatum (56).jpg	fold1	1 *Amblyomma*
2491	All together	AlexNet	todos aureolatum (57).jpg	fold1	1 *Amblyomma*
2492	All together	AlexNet	todos aureolatum (6).jpg	fold1	1 *Amblyomma*
2493	All together	AlexNet	todos aureolatum (60).jpg	fold1	1 *Amblyomma*
2494	All together	AlexNet	todos aureolatum (66).jpg	fold1	1 *Amblyomma*
2495	All together	AlexNet	todos aureolatum (69).jpg	fold1	1 *Amblyomma*
2496	All together	AlexNet	todos aureolatum (72).jpg	fold1	1 *Amblyomma*
2497	All together	AlexNet	todos aureolatum (73).jpg	fold1	1 *Amblyomma*
2498	All together	AlexNet	todos aureolatum (79).jpg	fold1	1 *Amblyomma*
2499	All together	AlexNet	todos aureolatum (80).jpg	fold1	1 *Amblyomma*
2500	All together	AlexNet	todos aureolatum (82).jpg	fold1	1 *Amblyomma*
2501	All together	AlexNet	todos aureolatum (85).jpg	fold1	1 *Amblyomma*
2502	All together	AlexNet	todos aureolatum (90).jpg	fold1	1 *Amblyomma*
2503	All together	AlexNet	todos aureolatum (93).jpg	fold1	1 *Amblyomma*
2504	All together	AlexNet	todos cajennense (10).jpg	fold1	2 *Amblyomma*
2505	All together	AlexNet	todos cajennense (101).jpg	fold1	2 *Amblyomma*
2506	All together	AlexNet	todos cajennense (102).jpg	fold1	2 *Amblyomma*
2507	All together	AlexNet	todos cajennense (106).jpg	fold1	2 *Amblyomma*
2508	All together	AlexNet	todos cajennense (111).jpg	fold1	2 *Amblyomma*
2509	All together	AlexNet	todos cajennense (113).jpg	fold1	2 *Amblyomma*
2510	All together	AlexNet	todos cajennense (114).jpg	fold1	2 *Amblyomma*
2511	All together	AlexNet	todos cajennense (117).jpg	fold1	2 *Amblyomma*
2512	All together	AlexNet	todos cajennense (124).jpg	fold1	2 *Amblyomma*
2513	All together	AlexNet	todos cajennense (129).jpg	fold1	2 *Amblyomma*
2514	All together	AlexNet	todos cajennense (13).jpg	fold1	2 *Amblyomma*
2515	All together	AlexNet	todos cajennense (133).jpg	fold1	2 *Amblyomma*
2516	All together	AlexNet	todos cajennense (137).jpg	fold1	2 *Amblyomma*
2517	All together	AlexNet	todos cajennense (138).jpg	fold1	2 *Amblyomma*
2518	All together	AlexNet	todos cajennense (139).jpg	fold1	2 *Amblyomma*
2519	All together	AlexNet	todos cajennense (146).jpg	fold1	2 *Amblyomma*
2520	All together	AlexNet	todos cajennense (148).jpg	fold1	2 *Amblyomma*
2521	All together	AlexNet	todos cajennense (151).jpg	fold1	2 *Amblyomma*
2522	All together	AlexNet	todos cajennense (26).jpg	fold1	2 *Amblyomma*
2523	All together	AlexNet	todos cajennense (28).jpg	fold1	2 *Amblyomma*
2524	All together	AlexNet	todos cajennense (33).jpg	fold1	2 *Amblyomma*
2525	All together	AlexNet	todos cajennense (37).jpg	fold1	2 *Amblyomma*
2526	All together	AlexNet	todos cajennense (44).jpg	fold1	2 *Amblyomma*
2527	All together	AlexNet	todos cajennense (49).jpg	fold1	2 *Amblyomma*
2528	All together	AlexNet	todos cajennense (51).jpg	fold1	2 *Amblyomma*
2529	All together	AlexNet	todos cajennense (67).jpg	fold1	2 *Amblyomma*
2530	All together	AlexNet	todos cajennense (70).jpg	fold1	2 *Amblyomma*
2531	All together	AlexNet	todos cajennense (74).jpg	fold1	2 *Amblyomma*
2532	All together	AlexNet	todos cajennense (76).jpg	fold1	2 *Amblyomma*
2533	All together	AlexNet	todos cajennense (85).jpg	fold1	2 *Amblyomma*
2534	All together	AlexNet	todos cajennense (94).jpg	fold1	2 *Amblyomma*
2535	All together	AlexNet	todos cajennense (97).jpg	fold1	2 *Amblyomma*
2536	All together	AlexNet	todos cajennense (99).jpg	fold1	2 *Amblyomma*
2537	All together	AlexNet	todos dubitatum (103).jpg	fold1	3 *Amblyomma*
2538	All together	AlexNet	todos dubitatum (104).jpg	fold1	3 *Amblyomma*
2539	All together	AlexNet	todos dubitatum (109).jpg	fold1	3 *Amblyomma*
2540	All together	AlexNet	todos dubitatum (12).JPG	fold1	3 *Amblyomma*
2541	All together	AlexNet	todos dubitatum (15).jpg	fold1	3 *Amblyomma*
2542	All together	AlexNet	todos dubitatum (2).jpg	fold1	3 *Amblyomma*
2543	All together	AlexNet	todos dubitatum (20).jpg	fold1	3 *Amblyomma*
2544	All together	AlexNet	todos dubitatum (3).jpg	fold1	3 *Amblyomma*

2545	All together	AlexNet	todos dubitatum (36).jpg	fold1	3	*Amblyomma*
2546	All together	AlexNet	todos dubitatum (37).jpg	fold1	3	*Amblyomma*
2547	All together	AlexNet	todos dubitatum (39).jpg	fold1	3	*Amblyomma*
2548	All together	AlexNet	todos dubitatum (42).jpg	fold1	3	*Amblyomma*
2549	All together	AlexNet	todos dubitatum (44).jpg	fold1	3	*Amblyomma*
2550	All together	AlexNet	todos dubitatum (52).jpg	fold1	3	*Amblyomma*
2551	All together	AlexNet	todos dubitatum (56).jpg	fold1	3	*Amblyomma*
2552	All together	AlexNet	todos dubitatum (60).jpg	fold1	3	*Amblyomma*
2553	All together	AlexNet	todos dubitatum (62).jpg	fold1	3	*Amblyomma*
2554	All together	AlexNet	todos dubitatum (76).jpg	fold1	3	*Amblyomma*
2555	All together	AlexNet	todos dubitatum (77).jpg	fold1	3	*Amblyomma*
2556	All together	AlexNet	todos dubitatum (81).jpg	fold1	3	*Amblyomma*
2557	All together	AlexNet	todos dubitatum (82).jpg	fold1	3	*Amblyomma*
2558	All together	AlexNet	todos dubitatum (83).jpg	fold1	3	*Amblyomma*
2559	All together	AlexNet	todos ovale (100).jpg	fold1	4	*Amblyomma*
2560	All together	AlexNet	todos ovale (104).jpg	fold1	4	*Amblyomma*
2561	All together	AlexNet	todos ovale (106).JPG	fold1	4	*Amblyomma*
2562	All together	AlexNet	todos ovale (108).jpg	fold1	4	*Amblyomma*
2563	All together	AlexNet	todos ovale (109).jpg	fold1	4	*Amblyomma*
2564	All together	AlexNet	todos ovale (116).jpg	fold1	4	*Amblyomma*
2565	All together	AlexNet	todos ovale (118).jpg	fold1	4	*Amblyomma*
2566	All together	AlexNet	todos ovale (121).jpg	fold1	4	*Amblyomma*
2567	All together	AlexNet	todos ovale (16).jpg	fold1	4	*Amblyomma*
2568	All together	AlexNet	todos ovale (20).jpg	fold1	4	*Amblyomma*
2569	All together	AlexNet	todos ovale (24).jpg	fold1	4	*Amblyomma*
2570	All together	AlexNet	todos ovale (27).jpg	fold1	4	*Amblyomma*
2571	All together	AlexNet	todos ovale (34).jpg	fold1	4	*Amblyomma*
2572	All together	AlexNet	todos ovale (38).jpg	fold1	4	*Amblyomma*
2573	All together	AlexNet	todos ovale (4).jpg	fold1	4	*Amblyomma*
2574	All together	AlexNet	todos ovale (42).jpg	fold1	4	*Amblyomma*
2575	All together	AlexNet	todos ovale (47).jpg	fold1	4	*Amblyomma*
2576	All together	AlexNet	todos ovale (60).jpg	fold1	4	*Amblyomma*
2577	All together	AlexNet	todos ovale (62).jpg	fold1	4	*Amblyomma*
2578	All together	AlexNet	todos ovale (65).jpg	fold1	4	*Amblyomma*
2579	All together	AlexNet	todos ovale (7).jpg	fold1	4	*Amblyomma*
2580	All together	AlexNet	todos ovale (70).jpg	fold1	4	*Amblyomma*
2581	All together	AlexNet	todos ovale (85).jpg	fold1	4	*Amblyomma*
2582	All together	AlexNet	todos ovale (87).jpg	fold1	4	*Amblyomma*
2583	All together	AlexNet	todos sculptum (100).JPG	fold1	5	*Amblyomma*
2584	All together	AlexNet	todos sculptum (103).JPG	fold1	5	*Amblyomma*
2585	All together	AlexNet	todos sculptum (106).JPG	fold1	5	*Amblyomma*
2586	All together	AlexNet	todos sculptum (108).JPG	fold1	5	*Amblyomma*
2587	All together	AlexNet	todos sculptum (112).JPG	fold1	5	*Amblyomma*
2588	All together	AlexNet	todos sculptum (113).JPG	fold1	5	*Amblyomma*
2589	All together	AlexNet	todos sculptum (114).JPG	fold1	5	*Amblyomma*
2590	All together	AlexNet	todos sculptum (115).JPG	fold1	5	*Amblyomma*
2591	All together	AlexNet	todos sculptum (119).JPG	fold1	5	*Amblyomma*
2592	All together	AlexNet	todos sculptum (120).JPG	fold1	5	*Amblyomma*
2593	All together	AlexNet	todos sculptum (123).JPG	fold1	5	*Amblyomma*
2594	All together	AlexNet	todos sculptum (124).JPG	fold1	5	*Amblyomma*
2595	All together	AlexNet	todos sculptum (127).JPG	fold1	5	*Amblyomma*
2596	All together	AlexNet	todos sculptum (138).JPG	fold1	5	*Amblyomma*
2597	All together	AlexNet	todos sculptum (139).JPG	fold1	5	*Amblyomma*
2598	All together	AlexNet	todos sculptum (142).JPG	fold1	5	*Amblyomma*
2599	All together	AlexNet	todos sculptum (26).JPG	fold1	5	*Amblyomma*
2600	All together	AlexNet	todos sculptum (32).JPG	fold1	5	*Amblyomma*
2601	All together	AlexNet	todos sculptum (48).JPG	fold1	5	*Amblyomma*
2602	All together	AlexNet	todos sculptum (52).JPG	fold1	5	*Amblyomma*
2603	All together	AlexNet	todos sculptum (55).JPG	fold1	5	*Amblyomma*
2604	All together	AlexNet	todos sculptum (57).jpg	fold1	5	*Amblyomma*
2605	All together	AlexNet	todos sculptum (61).jpg	fold1	5	*Amblyomma*
2606	All together	AlexNet	todos sculptum (70).jpg	fold1	5	*Amblyomma*
2607	All together	AlexNet	todos sculptum (71).JPG	fold1	5	*Amblyomma*
2608	All together	AlexNet	todos sculptum (74).JPG	fold1	5	*Amblyomma*
2609	All together	AlexNet	todos sculptum (75).JPG	fold1	5	*Amblyomma*
2610	All together	AlexNet	todos sculptum (93).JPG	fold1	5	*Amblyomma*
2611	All together	AlexNet	todos sculptum (97).JPG	fold1	5	*Amblyomma*

2612	All together	AlexNet	todos sculptum (98).JPG	fold1	5	*Amblyomma*
2613	All together	AlexNet	todos sculptum (99).JPG	fold1	5	*Amblyomma*
2614	All together	AlexNet	todos triste (102).jpg	fold1	6	*Amblyomma*
2615	All together	AlexNet	todos triste (104).jpg	fold1	6	*Amblyomma*
2616	All together	AlexNet	todos triste (110).jpg	fold1	6	*Amblyomma*
2617	All together	AlexNet	todos triste (116).jpg	fold1	6	*Amblyomma*
2618	All together	AlexNet	todos triste (120).jpg	fold1	6	*Amblyomma*
2619	All together	AlexNet	todos triste (121).jpg	fold1	6	*Amblyomma*
2620	All together	AlexNet	todos triste (13).jpg	fold1	6	*Amblyomma*
2621	All together	AlexNet	todos triste (131).jpg	fold1	6	*Amblyomma*
2622	All together	AlexNet	todos triste (132).jpg	fold1	6	*Amblyomma*
2623	All together	AlexNet	todos triste (133).jpg	fold1	6	*Amblyomma*
2624	All together	AlexNet	todos triste (136).jpg	fold1	6	*Amblyomma*
2625	All together	AlexNet	todos triste (140).jpg	fold1	6	*Amblyomma*
2626	All together	AlexNet	todos triste (142).jpg	fold1	6	*Amblyomma*
2627	All together	AlexNet	todos triste (146).jpg	fold1	6	*Amblyomma*
2628	All together	AlexNet	todos triste (147).jpg	fold1	6	*Amblyomma*
2629	All together	AlexNet	todos triste (149).jpg	fold1	6	*Amblyomma*
2630	All together	AlexNet	todos triste (2).jpg	fold1	6	*Amblyomma*
2631	All together	AlexNet	todos triste (29).jpg	fold1	6	*Amblyomma*
2632	All together	AlexNet	todos triste (38).jpg	fold1	6	*Amblyomma*
2633	All together	AlexNet	todos triste (49).jpg	fold1	6	*Amblyomma*
2634	All together	AlexNet	todos triste (53).jpg	fold1	6	*Amblyomma*
2635	All together	AlexNet	todos triste (58).jpg	fold1	6	*Amblyomma*
2636	All together	AlexNet	todos triste (68).jpg	fold1	6	*Amblyomma*
2637	All together	AlexNet	todos triste (70).jpg	fold1	6	*Amblyomma*
2638	All together	AlexNet	todos triste (73).jpg	fold1	6	*Amblyomma*
2639	All together	AlexNet	todos triste (76).jpg	fold1	6	*Amblyomma*
2640	All together	AlexNet	todos triste (79).jpg	fold1	6	*Amblyomma*
2641	All together	AlexNet	todos triste (8).jpg	fold1	6	*Amblyomma*
2642	All together	AlexNet	todos triste (86).jpg	fold1	6	*Amblyomma*
2643	All together	AlexNet	todos triste (95).jpg	fold1	6	*Amblyomma*
2644	All together	AlexNet	todos triste (96).jpg	fold1	6	*Amblyomma*
2645	All together	AlexNet	todos aureolatum (107).jpg	fold2	1	*Amblyomma*
2646	All together	AlexNet	todos aureolatum (110).jpg	fold2	1	*Amblyomma*
2647	All together	AlexNet	todos aureolatum (112).jpg	fold2	1	*Amblyomma*
2648	All together	AlexNet	todos aureolatum (116).jpg	fold2	1	*Amblyomma*
2649	All together	AlexNet	todos aureolatum (117).jpg	fold2	1	*Amblyomma*
2650	All together	AlexNet	todos aureolatum (118).jpg	fold2	1	*Amblyomma*
2651	All together	AlexNet	todos aureolatum (12).jpg	fold2	1	*Amblyomma*
2652	All together	AlexNet	todos aureolatum (121).jpg	fold2	1	*Amblyomma*
2653	All together	AlexNet	todos aureolatum (18).jpg	fold2	1	*Amblyomma*
2654	All together	AlexNet	todos aureolatum (23).jpg	fold2	1	*Amblyomma*
2655	All together	AlexNet	todos aureolatum (24).jpg	fold2	1	*Amblyomma*
2656	All together	AlexNet	todos aureolatum (29).jpg	fold2	1	*Amblyomma*
2657	All together	AlexNet	todos aureolatum (3).jpg	fold2	1	*Amblyomma*
2658	All together	AlexNet	todos aureolatum (31).jpg	fold2	1	*Amblyomma*
2659	All together	AlexNet	todos aureolatum (32).jpg	fold2	1	*Amblyomma*
2660	All together	AlexNet	todos aureolatum (4).jpg	fold2	1	*Amblyomma*
2661	All together	AlexNet	todos aureolatum (42).jpg	fold2	1	*Amblyomma*
2662	All together	AlexNet	todos aureolatum (63).jpg	fold2	1	*Amblyomma*
2663	All together	AlexNet	todos aureolatum (64).jpg	fold2	1	*Amblyomma*
2664	All together	AlexNet	todos aureolatum (7).jpg	fold2	1	*Amblyomma*
2665	All together	AlexNet	todos aureolatum (77).jpg	fold2	1	*Amblyomma*
2666	All together	AlexNet	todos aureolatum (83).jpg	fold2	1	*Amblyomma*
2667	All together	AlexNet	todos aureolatum (84).jpg	fold2	1	*Amblyomma*
2668	All together	AlexNet	todos aureolatum (97).jpg	fold2	1	*Amblyomma*
2669	All together	AlexNet	todos cajennense (11).jpg	fold2	2	*Amblyomma*
2670	All together	AlexNet	todos cajennense (110).jpg	fold2	2	*Amblyomma*
2671	All together	AlexNet	todos cajennense (116).jpg	fold2	2	*Amblyomma*
2672	All together	AlexNet	todos cajennense (12).jpg	fold2	2	*Amblyomma*
2673	All together	AlexNet	todos cajennense (120).jpg	fold2	2	*Amblyomma*
2674	All together	AlexNet	todos cajennense (126).jpg	fold2	2	*Amblyomma*
2675	All together	AlexNet	todos cajennense (128).jpg	fold2	2	*Amblyomma*
2676	All together	AlexNet	todos cajennense (142).jpg	fold2	2	*Amblyomma*
2677	All together	AlexNet	todos cajennense (143).jpg	fold2	2	*Amblyomma*
2678	All together	AlexNet	todos cajennense (154).jpg	fold2	2	*Amblyomma*

2679	All together	AlexNet	todos cajennense (155).jpg	fold2	2	*Amblyomma*
2680	All together	AlexNet	todos cajennense (156).jpg	fold2	2	*Amblyomma*
2681	All together	AlexNet	todos cajennense (158).jpg	fold2	2	*Amblyomma*
2682	All together	AlexNet	todos cajennense (159).jpg	fold2	2	*Amblyomma*
2683	All together	AlexNet	todos cajennense (162).jpg	fold2	2	*Amblyomma*
2684	All together	AlexNet	todos cajennense (164).jpg	fold2	2	*Amblyomma*
2685	All together	AlexNet	todos cajennense (23).jpg	fold2	2	*Amblyomma*
2686	All together	AlexNet	todos cajennense (3).jpg	fold2	2	*Amblyomma*
2687	All together	AlexNet	todos cajennense (32).jpg	fold2	2	*Amblyomma*
2688	All together	AlexNet	todos cajennense (36).jpg	fold2	2	*Amblyomma*
2689	All together	AlexNet	todos cajennense (39).jpg	fold2	2	*Amblyomma*
2690	All together	AlexNet	todos cajennense (47).jpg	fold2	2	*Amblyomma*
2691	All together	AlexNet	todos cajennense (48).jpg	fold2	2	*Amblyomma*
2692	All together	AlexNet	todos cajennense (54).jpg	fold2	2	*Amblyomma*
2693	All together	AlexNet	todos cajennense (57).jpg	fold2	2	*Amblyomma*
2694	All together	AlexNet	todos cajennense (59).jpg	fold2	2	*Amblyomma*
2695	All together	AlexNet	todos cajennense (6).jpg	fold2	2	*Amblyomma*
2696	All together	AlexNet	todos cajennense (61).jpg	fold2	2	*Amblyomma*
2697	All together	AlexNet	todos cajennense (64).jpg	fold2	2	*Amblyomma*
2698	All together	AlexNet	todos cajennense (78).jpg	fold2	2	*Amblyomma*
2699	All together	AlexNet	todos cajennense (87).jpg	fold2	2	*Amblyomma*
2700	All together	AlexNet	todos cajennense (88).jpg	fold2	2	*Amblyomma*
2701	All together	AlexNet	todos cajennense (98).jpg	fold2	2	*Amblyomma*
2702	All together	AlexNet	todos dubitatum (1).jpg	fold2	3	*Amblyomma*
2703	All together	AlexNet	todos dubitatum (105).jpg	fold2	3	*Amblyomma*
2704	All together	AlexNet	todos dubitatum (107).jpg	fold2	3	*Amblyomma*
2705	All together	AlexNet	todos dubitatum (11).JPG	fold2	3	*Amblyomma*
2706	All together	AlexNet	todos dubitatum (25).jpg	fold2	3	*Amblyomma*
2707	All together	AlexNet	todos dubitatum (26).jpg	fold2	3	*Amblyomma*
2708	All together	AlexNet	todos dubitatum (46).jpg	fold2	3	*Amblyomma*
2709	All together	AlexNet	todos dubitatum (47).jpg	fold2	3	*Amblyomma*
2710	All together	AlexNet	todos dubitatum (49).jpg	fold2	3	*Amblyomma*
2711	All together	AlexNet	todos dubitatum (5).jpg	fold2	3	*Amblyomma*
2712	All together	AlexNet	todos dubitatum (57).jpg	fold2	3	*Amblyomma*
2713	All together	AlexNet	todos dubitatum (63).jpg	fold2	3	*Amblyomma*
2714	All together	AlexNet	todos dubitatum (69).jpg	fold2	3	*Amblyomma*
2715	All together	AlexNet	todos dubitatum (71).jpg	fold2	3	*Amblyomma*
2716	All together	AlexNet	todos dubitatum (74).jpg	fold2	3	*Amblyomma*
2717	All together	AlexNet	todos dubitatum (78).jpg	fold2	3	*Amblyomma*
2718	All together	AlexNet	todos dubitatum (8).jpg	fold2	3	*Amblyomma*
2719	All together	AlexNet	todos dubitatum (85).jpg	fold2	3	*Amblyomma*
2720	All together	AlexNet	todos dubitatum (92).jpg	fold2	3	*Amblyomma*
2721	All together	AlexNet	todos dubitatum (94).jpg	fold2	3	*Amblyomma*
2722	All together	AlexNet	todos dubitatum (95).jpg	fold2	3	*Amblyomma*
2723	All together	AlexNet	todos dubitatum (96).jpg	fold2	3	*Amblyomma*
2724	All together	AlexNet	todos ovale (10).jpg	fold2	4	*Amblyomma*
2725	All together	AlexNet	todos ovale (11).jpg	fold2	4	*Amblyomma*
2726	All together	AlexNet	todos ovale (110).jpg	fold2	4	*Amblyomma*
2727	All together	AlexNet	todos ovale (112).jpg	fold2	4	*Amblyomma*
2728	All together	AlexNet	todos ovale (2).jpg	fold2	4	*Amblyomma*
2729	All together	AlexNet	todos ovale (23).jpg	fold2	4	*Amblyomma*
2730	All together	AlexNet	todos ovale (25).jpg	fold2	4	*Amblyomma*
2731	All together	AlexNet	todos ovale (29).jpg	fold2	4	*Amblyomma*
2732	All together	AlexNet	todos ovale (32).jpg	fold2	4	*Amblyomma*
2733	All together	AlexNet	todos ovale (44).jpg	fold2	4	*Amblyomma*
2734	All together	AlexNet	todos ovale (46).jpg	fold2	4	*Amblyomma*
2735	All together	AlexNet	todos ovale (56).jpg	fold2	4	*Amblyomma*
2736	All together	AlexNet	todos ovale (57).jpg	fold2	4	*Amblyomma*
2737	All together	AlexNet	todos ovale (58).jpg	fold2	4	*Amblyomma*
2738	All together	AlexNet	todos ovale (67).jpg	fold2	4	*Amblyomma*
2739	All together	AlexNet	todos ovale (68).jpg	fold2	4	*Amblyomma*
2740	All together	AlexNet	todos ovale (74).jpg	fold2	4	*Amblyomma*
2741	All together	AlexNet	todos ovale (76).JPG	fold2	4	*Amblyomma*
2742	All together	AlexNet	todos ovale (78).jpg	fold2	4	*Amblyomma*
2743	All together	AlexNet	todos ovale (8).jpg	fold2	4	*Amblyomma*
2744	All together	AlexNet	todos ovale (86).jpg	fold2	4	*Amblyomma*
2745	All together	AlexNet	todos ovale (96).jpg	fold2	4	*Amblyomma*

2746	All together	AlexNet	todos ovale (97).jpg	fold2	4	*Amblyomma*
2747	All together	AlexNet	todos ovale (99).jpg	fold2	4	*Amblyomma*
2748	All together	AlexNet	todos sculptum (102).JPG	fold2	5	*Amblyomma*
2749	All together	AlexNet	todos sculptum (105).JPG	fold2	5	*Amblyomma*
2750	All together	AlexNet	todos sculptum (107).JPG	fold2	5	*Amblyomma*
2751	All together	AlexNet	todos sculptum (109).JPG	fold2	5	*Amblyomma*
2752	All together	AlexNet	todos sculptum (11).JPG	fold2	5	*Amblyomma*
2753	All together	AlexNet	todos sculptum (12).JPG	fold2	5	*Amblyomma*
2754	All together	AlexNet	todos sculptum (121).JPG	fold2	5	*Amblyomma*
2755	All together	AlexNet	todos sculptum (122).JPG	fold2	5	*Amblyomma*
2756	All together	AlexNet	todos sculptum (125).JPG	fold2	5	*Amblyomma*
2757	All together	AlexNet	todos sculptum (128).JPG	fold2	5	*Amblyomma*
2758	All together	AlexNet	todos sculptum (130).JPG	fold2	5	*Amblyomma*
2759	All together	AlexNet	todos sculptum (14).JPG	fold2	5	*Amblyomma*
2760	All together	AlexNet	todos sculptum (141).JPG	fold2	5	*Amblyomma*
2761	All together	AlexNet	todos sculptum (147).jpg	fold2	5	*Amblyomma*
2762	All together	AlexNet	todos sculptum (155).jpg	fold2	5	*Amblyomma*
2763	All together	AlexNet	todos sculptum (25).JPG	fold2	5	*Amblyomma*
2764	All together	AlexNet	todos sculptum (30).JPG	fold2	5	*Amblyomma*
2765	All together	AlexNet	todos sculptum (33).JPG	fold2	5	*Amblyomma*
2766	All together	AlexNet	todos sculptum (35).JPG	fold2	5	*Amblyomma*
2767	All together	AlexNet	todos sculptum (36).JPG	fold2	5	*Amblyomma*
2768	All together	AlexNet	todos sculptum (37).JPG	fold2	5	*Amblyomma*
2769	All together	AlexNet	todos sculptum (4).jpg	fold2	5	*Amblyomma*
2770	All together	AlexNet	todos sculptum (5).jpg	fold2	5	*Amblyomma*
2771	All together	AlexNet	todos sculptum (51).JPG	fold2	5	*Amblyomma*
2772	All together	AlexNet	todos sculptum (60).jpg	fold2	5	*Amblyomma*
2773	All together	AlexNet	todos sculptum (63).jpg	fold2	5	*Amblyomma*
2774	All together	AlexNet	todos sculptum (73).JPG	fold2	5	*Amblyomma*
2775	All together	AlexNet	todos sculptum (80).JPG	fold2	5	*Amblyomma*
2776	All together	AlexNet	todos sculptum (84).JPG	fold2	5	*Amblyomma*
2777	All together	AlexNet	todos sculptum (85).JPG	fold2	5	*Amblyomma*
2778	All together	AlexNet	todos sculptum (90).JPG	fold2	5	*Amblyomma*
2779	All together	AlexNet	todos triste (103).jpg	fold2	6	*Amblyomma*
2780	All together	AlexNet	todos triste (109).jpg	fold2	6	*Amblyomma*
2781	All together	AlexNet	todos triste (123).jpg	fold2	6	*Amblyomma*
2782	All together	AlexNet	todos triste (127).jpg	fold2	6	*Amblyomma*
2783	All together	AlexNet	todos triste (128).jpg	fold2	6	*Amblyomma*
2784	All together	AlexNet	todos triste (129).jpg	fold2	6	*Amblyomma*
2785	All together	AlexNet	todos triste (130).jpg	fold2	6	*Amblyomma*
2786	All together	AlexNet	todos triste (152).jpg	fold2	6	*Amblyomma*
2787	All together	AlexNet	todos triste (20).jpg	fold2	6	*Amblyomma*
2788	All together	AlexNet	todos triste (23).jpg	fold2	6	*Amblyomma*
2789	All together	AlexNet	todos triste (24).jpg	fold2	6	*Amblyomma*
2790	All together	AlexNet	todos triste (26).jpg	fold2	6	*Amblyomma*
2791	All together	AlexNet	todos triste (27).jpg	fold2	6	*Amblyomma*
2792	All together	AlexNet	todos triste (3).jpg	fold2	6	*Amblyomma*
2793	All together	AlexNet	todos triste (35).jpg	fold2	6	*Amblyomma*
2794	All together	AlexNet	todos triste (36).jpg	fold2	6	*Amblyomma*
2795	All together	AlexNet	todos triste (42).jpg	fold2	6	*Amblyomma*
2796	All together	AlexNet	todos triste (48).jpg	fold2	6	*Amblyomma*
2797	All together	AlexNet	todos triste (5).jpg	fold2	6	*Amblyomma*
2798	All together	AlexNet	todos triste (52).jpg	fold2	6	*Amblyomma*
2799	All together	AlexNet	todos triste (54).jpg	fold2	6	*Amblyomma*
2800	All together	AlexNet	todos triste (55).jpg	fold2	6	*Amblyomma*
2801	All together	AlexNet	todos triste (56).jpg	fold2	6	*Amblyomma*
2802	All together	AlexNet	todos triste (57).jpg	fold2	6	*Amblyomma*
2803	All together	AlexNet	todos triste (59).jpg	fold2	6	*Amblyomma*
2804	All together	AlexNet	todos triste (64).jpg	fold2	6	*Amblyomma*
2805	All together	AlexNet	todos triste (65).jpg	fold2	6	*Amblyomma*
2806	All together	AlexNet	todos triste (74).jpg	fold2	6	*Amblyomma*
2807	All together	AlexNet	todos triste (87).jpg	fold2	6	*Amblyomma*
2808	All together	AlexNet	todos triste (9).jpg	fold2	6	*Amblyomma*
2809	All together	AlexNet	todos triste (92).jpg	fold2	6	*Amblyomma*
2810	All together	AlexNet	todos aureolatum (1).jpg	fold3	1	*Amblyomma*
2811	All together	AlexNet	todos aureolatum (103).jpg	fold3	1	*Amblyomma*
2812	All together	AlexNet	todos aureolatum (108).jpg	fold3	1	*Amblyomma*

2813	All together	AlexNet	todos aureolatum (113).jpg	fold3	1	*Amblyomma*
2814	All together	AlexNet	todos aureolatum (120).jpg	fold3	1	*Amblyomma*
2815	All together	AlexNet	todos aureolatum (13).jpg	fold3	1	*Amblyomma*
2816	All together	AlexNet	todos aureolatum (15).jpg	fold3	1	*Amblyomma*
2817	All together	AlexNet	todos aureolatum (16).jpg	fold3	1	*Amblyomma*
2818	All together	AlexNet	todos aureolatum (20).jpg	fold3	1	*Amblyomma*
2819	All together	AlexNet	todos aureolatum (33).jpg	fold3	1	*Amblyomma*
2820	All together	AlexNet	todos aureolatum (34).jpg	fold3	1	*Amblyomma*
2821	All together	AlexNet	todos aureolatum (46).jpg	fold3	1	*Amblyomma*
2822	All together	AlexNet	todos aureolatum (47).jpg	fold3	1	*Amblyomma*
2823	All together	AlexNet	todos aureolatum (67).jpg	fold3	1	*Amblyomma*
2824	All together	AlexNet	todos aureolatum (68).jpg	fold3	1	*Amblyomma*
2825	All together	AlexNet	todos aureolatum (71).jpg	fold3	1	*Amblyomma*
2826	All together	AlexNet	todos aureolatum (74).jpg	fold3	1	*Amblyomma*
2827	All together	AlexNet	todos aureolatum (76).jpg	fold3	1	*Amblyomma*
2828	All together	AlexNet	todos aureolatum (8).jpg	fold3	1	*Amblyomma*
2829	All together	AlexNet	todos aureolatum (86).jpg	fold3	1	*Amblyomma*
2830	All together	AlexNet	todos aureolatum (89).jpg	fold3	1	*Amblyomma*
2831	All together	AlexNet	todos aureolatum (92).jpg	fold3	1	*Amblyomma*
2832	All together	AlexNet	todos aureolatum (94).jpg	fold3	1	*Amblyomma*
2833	All together	AlexNet	todos aureolatum (96).jpg	fold3	1	*Amblyomma*
2834	All together	AlexNet	todos cajennense (100).jpg	fold3	2	*Amblyomma*
2835	All together	AlexNet	todos cajennense (109).jpg	fold3	2	*Amblyomma*
2836	All together	AlexNet	todos cajennense (112).jpg	fold3	2	*Amblyomma*
2837	All together	AlexNet	todos cajennense (115).jpg	fold3	2	*Amblyomma*
2838	All together	AlexNet	todos cajennense (121).jpg	fold3	2	*Amblyomma*
2839	All together	AlexNet	todos cajennense (123).jpg	fold3	2	*Amblyomma*
2840	All together	AlexNet	todos cajennense (127).jpg	fold3	2	*Amblyomma*
2841	All together	AlexNet	todos cajennense (134).jpg	fold3	2	*Amblyomma*
2842	All together	AlexNet	todos cajennense (135).jpg	fold3	2	*Amblyomma*
2843	All together	AlexNet	todos cajennense (144).jpg	fold3	2	*Amblyomma*
2844	All together	AlexNet	todos cajennense (149).jpg	fold3	2	*Amblyomma*
2845	All together	AlexNet	todos cajennense (157).jpg	fold3	2	*Amblyomma*
2846	All together	AlexNet	todos cajennense (165).jpg	fold3	2	*Amblyomma*
2847	All together	AlexNet	todos cajennense (21).jpg	fold3	2	*Amblyomma*
2848	All together	AlexNet	todos cajennense (22).jpg	fold3	2	*Amblyomma*
2849	All together	AlexNet	todos cajennense (24).jpg	fold3	2	*Amblyomma*
2850	All together	AlexNet	todos cajennense (27).jpg	fold3	2	*Amblyomma*
2851	All together	AlexNet	todos cajennense (29).jpg	fold3	2	*Amblyomma*
2852	All together	AlexNet	todos cajennense (30).jpg	fold3	2	*Amblyomma*
2853	All together	AlexNet	todos cajennense (38).jpg	fold3	2	*Amblyomma*
2854	All together	AlexNet	todos cajennense (40).jpg	fold3	2	*Amblyomma*
2855	All together	AlexNet	todos cajennense (43).jpg	fold3	2	*Amblyomma*
2856	All together	AlexNet	todos cajennense (45).jpg	fold3	2	*Amblyomma*
2857	All together	AlexNet	todos cajennense (46).jpg	fold3	2	*Amblyomma*
2858	All together	AlexNet	todos cajennense (5).jpg	fold3	2	*Amblyomma*
2859	All together	AlexNet	todos cajennense (56).jpg	fold3	2	*Amblyomma*
2860	All together	AlexNet	todos cajennense (60).jpg	fold3	2	*Amblyomma*
2861	All together	AlexNet	todos cajennense (65).jpg	fold3	2	*Amblyomma*
2862	All together	AlexNet	todos cajennense (71).jpg	fold3	2	*Amblyomma*
2863	All together	AlexNet	todos cajennense (72).jpg	fold3	2	*Amblyomma*
2864	All together	AlexNet	todos cajennense (82).jpg	fold3	2	*Amblyomma*
2865	All together	AlexNet	todos cajennense (84).jpg	fold3	2	*Amblyomma*
2866	All together	AlexNet	todos cajennense (95).jpg	fold3	2	*Amblyomma*
2867	All together	AlexNet	todos dubitatum (101).jpg	fold3	3	*Amblyomma*
2868	All together	AlexNet	todos dubitatum (108).jpg	fold3	3	*Amblyomma*
2869	All together	AlexNet	todos dubitatum (13).JPG	fold3	3	*Amblyomma*
2870	All together	AlexNet	todos dubitatum (14).JPG	fold3	3	*Amblyomma*
2871	All together	AlexNet	todos dubitatum (19).jpg	fold3	3	*Amblyomma*
2872	All together	AlexNet	todos dubitatum (22).jpg	fold3	3	*Amblyomma*
2873	All together	AlexNet	todos dubitatum (23).jpg	fold3	3	*Amblyomma*
2874	All together	AlexNet	todos dubitatum (24).jpg	fold3	3	*Amblyomma*
2875	All together	AlexNet	todos dubitatum (28).jpg	fold3	3	*Amblyomma*
2876	All together	AlexNet	todos dubitatum (34).jpg	fold3	3	*Amblyomma*
2877	All together	AlexNet	todos dubitatum (40).jpg	fold3	3	*Amblyomma*
2878	All together	AlexNet	todos dubitatum (41).jpg	fold3	3	*Amblyomma*
2879	All together	AlexNet	todos dubitatum (43).jpg	fold3	3	*Amblyomma*

2880	All together	AlexNet	todos dubitatum (48).jpg	fold3	3	*Amblyomma*
2881	All together	AlexNet	todos dubitatum (55).jpg	fold3	3	*Amblyomma*
2882	All together	AlexNet	todos dubitatum (6).jpg	fold3	3	*Amblyomma*
2883	All together	AlexNet	todos dubitatum (66).jpg	fold3	3	*Amblyomma*
2884	All together	AlexNet	todos dubitatum (67).jpg	fold3	3	*Amblyomma*
2885	All together	AlexNet	todos dubitatum (70).jpg	fold3	3	*Amblyomma*
2886	All together	AlexNet	todos dubitatum (87).jpg	fold3	3	*Amblyomma*
2887	All together	AlexNet	todos dubitatum (90).jpg	fold3	3	*Amblyomma*
2888	All together	AlexNet	todos dubitatum (93).jpg	fold3	3	*Amblyomma*
2889	All together	AlexNet	todos ovale (101).jpg	fold3	4	*Amblyomma*
2890	All together	AlexNet	todos ovale (105).jpg	fold3	4	*Amblyomma*
2891	All together	AlexNet	todos ovale (107).JPG	fold3	4	*Amblyomma*
2892	All together	AlexNet	todos ovale (120).jpg	fold3	4	*Amblyomma*
2893	All together	AlexNet	todos ovale (13).jpg	fold3	4	*Amblyomma*
2894	All together	AlexNet	todos ovale (19).jpg	fold3	4	*Amblyomma*
2895	All together	AlexNet	todos ovale (22).jpg	fold3	4	*Amblyomma*
2896	All together	AlexNet	todos ovale (3).jpg	fold3	4	*Amblyomma*
2897	All together	AlexNet	todos ovale (30).jpg	fold3	4	*Amblyomma*
2898	All together	AlexNet	todos ovale (33).jpg	fold3	4	*Amblyomma*
2899	All together	AlexNet	todos ovale (36).jpg	fold3	4	*Amblyomma*
2900	All together	AlexNet	todos ovale (41).jpg	fold3	4	*Amblyomma*
2901	All together	AlexNet	todos ovale (43).jpg	fold3	4	*Amblyomma*
2902	All together	AlexNet	todos ovale (52).jpg	fold3	4	*Amblyomma*
2903	All together	AlexNet	todos ovale (54).jpg	fold3	4	*Amblyomma*
2904	All together	AlexNet	todos ovale (55).jpg	fold3	4	*Amblyomma*
2905	All together	AlexNet	todos ovale (71).jpg	fold3	4	*Amblyomma*
2906	All together	AlexNet	todos ovale (81).jpg	fold3	4	*Amblyomma*
2907	All together	AlexNet	todos ovale (82).jpg	fold3	4	*Amblyomma*
2908	All together	AlexNet	todos ovale (83).jpg	fold3	4	*Amblyomma*
2909	All together	AlexNet	todos ovale (84).jpg	fold3	4	*Amblyomma*
2910	All together	AlexNet	todos ovale (92).jpg	fold3	4	*Amblyomma*
2911	All together	AlexNet	todos ovale (93).jpg	fold3	4	*Amblyomma*
2912	All together	AlexNet	todos ovale (95).jpg	fold3	4	*Amblyomma*
2913	All together	AlexNet	todos sculptum (116).JPG	fold3	5	*Amblyomma*
2914	All together	AlexNet	todos sculptum (118).JPG	fold3	5	*Amblyomma*
2915	All together	AlexNet	todos sculptum (126).JPG	fold3	5	*Amblyomma*
2916	All together	AlexNet	todos sculptum (13).JPG	fold3	5	*Amblyomma*
2917	All together	AlexNet	todos sculptum (132).JPG	fold3	5	*Amblyomma*
2918	All together	AlexNet	todos sculptum (133).JPG	fold3	5	*Amblyomma*
2919	All together	AlexNet	todos sculptum (135).JPG	fold3	5	*Amblyomma*
2920	All together	AlexNet	todos sculptum (140).JPG	fold3	5	*Amblyomma*
2921	All together	AlexNet	todos sculptum (152).jpg	fold3	5	*Amblyomma*
2922	All together	AlexNet	todos sculptum (154).jpg	fold3	5	*Amblyomma*
2923	All together	AlexNet	todos sculptum (17).JPG	fold3	5	*Amblyomma*
2924	All together	AlexNet	todos sculptum (18).JPG	fold3	5	*Amblyomma*
2925	All together	AlexNet	todos sculptum (19).JPG	fold3	5	*Amblyomma*
2926	All together	AlexNet	todos sculptum (20).JPG	fold3	5	*Amblyomma*
2927	All together	AlexNet	todos sculptum (22).JPG	fold3	5	*Amblyomma*
2928	All together	AlexNet	todos sculptum (28).JPG	fold3	5	*Amblyomma*
2929	All together	AlexNet	todos sculptum (3).jpg	fold3	5	*Amblyomma*
2930	All together	AlexNet	todos sculptum (38).JPG	fold3	5	*Amblyomma*
2931	All together	AlexNet	todos sculptum (39).JPG	fold3	5	*Amblyomma*
2932	All together	AlexNet	todos sculptum (41).JPG	fold3	5	*Amblyomma*
2933	All together	AlexNet	todos sculptum (45).JPG	fold3	5	*Amblyomma*
2934	All together	AlexNet	todos sculptum (47).JPG	fold3	5	*Amblyomma*
2935	All together	AlexNet	todos sculptum (69).jpg	fold3	5	*Amblyomma*
2936	All together	AlexNet	todos sculptum (7).jpg	fold3	5	*Amblyomma*
2937	All together	AlexNet	todos sculptum (76).JPG	fold3	5	*Amblyomma*
2938	All together	AlexNet	todos sculptum (83).JPG	fold3	5	*Amblyomma*
2939	All together	AlexNet	todos sculptum (87).JPG	fold3	5	*Amblyomma*
2940	All together	AlexNet	todos sculptum (88).JPG	fold3	5	*Amblyomma*
2941	All together	AlexNet	todos sculptum (89).JPG	fold3	5	*Amblyomma*
2942	All together	AlexNet	todos sculptum (9).JPG	fold3	5	*Amblyomma*
2943	All together	AlexNet	todos sculptum (96).JPG	fold3	5	*Amblyomma*
2944	All together	AlexNet	todos triste (101).jpg	fold3	6	*Amblyomma*
2945	All together	AlexNet	todos triste (106).jpg	fold3	6	*Amblyomma*
2946	All together	AlexNet	todos triste (115).jpg	fold3	6	*Amblyomma*

2947	All together	AlexNet	todos triste (118).jpg	fold3	6	*Amblyomma*
2948	All together	AlexNet	todos triste (126).jpg	fold3	6	*Amblyomma*
2949	All together	AlexNet	todos triste (135).jpg	fold3	6	*Amblyomma*
2950	All together	AlexNet	todos triste (137).jpg	fold3	6	*Amblyomma*
2951	All together	AlexNet	todos triste (139).jpg	fold3	6	*Amblyomma*
2952	All together	AlexNet	todos triste (141).jpg	fold3	6	*Amblyomma*
2953	All together	AlexNet	todos triste (144).jpg	fold3	6	*Amblyomma*
2954	All together	AlexNet	todos triste (148).jpg	fold3	6	*Amblyomma*
2955	All together	AlexNet	todos triste (15).jpg	fold3	6	*Amblyomma*
2956	All together	AlexNet	todos triste (153).jpg	fold3	6	*Amblyomma*
2957	All together	AlexNet	todos triste (154).jpg	fold3	6	*Amblyomma*
2958	All together	AlexNet	todos triste (17).jpg	fold3	6	*Amblyomma*
2959	All together	AlexNet	todos triste (21).jpg	fold3	6	*Amblyomma*
2960	All together	AlexNet	todos triste (22).jpg	fold3	6	*Amblyomma*
2961	All together	AlexNet	todos triste (32).jpg	fold3	6	*Amblyomma*
2962	All together	AlexNet	todos triste (37).jpg	fold3	6	*Amblyomma*
2963	All together	AlexNet	todos triste (43).jpg	fold3	6	*Amblyomma*
2964	All together	AlexNet	todos triste (44).jpg	fold3	6	*Amblyomma*
2965	All together	AlexNet	todos triste (45).jpg	fold3	6	*Amblyomma*
2966	All together	AlexNet	todos triste (50).jpg	fold3	6	*Amblyomma*
2967	All together	AlexNet	todos triste (51).jpg	fold3	6	*Amblyomma*
2968	All together	AlexNet	todos triste (61).jpg	fold3	6	*Amblyomma*
2969	All together	AlexNet	todos triste (69).jpg	fold3	6	*Amblyomma*
2970	All together	AlexNet	todos triste (78).jpg	fold3	6	*Amblyomma*
2971	All together	AlexNet	todos triste (81).jpg	fold3	6	*Amblyomma*
2972	All together	AlexNet	todos triste (83).jpg	fold3	6	*Amblyomma*
2973	All together	AlexNet	todos triste (84).jpg	fold3	6	*Amblyomma*
2974	All together	AlexNet	todos triste (93).jpg	fold3	6	*Amblyomma*
2975	All together	AlexNet	todos aureolatum (101).jpg	fold4	1	*Amblyomma*
2976	All together	AlexNet	todos aureolatum (102).jpg	fold4	1	*Amblyomma*
2977	All together	AlexNet	todos aureolatum (104).jpg	fold4	1	*Amblyomma*
2978	All together	AlexNet	todos aureolatum (105).jpg	fold4	1	*Amblyomma*
2979	All together	AlexNet	todos aureolatum (11).jpg	fold4	1	*Amblyomma*
2980	All together	AlexNet	todos aureolatum (111).jpg	fold4	1	*Amblyomma*
2981	All together	AlexNet	todos aureolatum (114).jpg	fold4	1	*Amblyomma*
2982	All together	AlexNet	todos aureolatum (115).jpg	fold4	1	*Amblyomma*
2983	All together	AlexNet	todos aureolatum (17).jpg	fold4	1	*Amblyomma*
2984	All together	AlexNet	todos aureolatum (2).jpg	fold4	1	*Amblyomma*
2985	All together	AlexNet	todos aureolatum (36).jpg	fold4	1	*Amblyomma*
2986	All together	AlexNet	todos aureolatum (43).jpg	fold4	1	*Amblyomma*
2987	All together	AlexNet	todos aureolatum (44).jpg	fold4	1	*Amblyomma*
2988	All together	AlexNet	todos aureolatum (49).jpg	fold4	1	*Amblyomma*
2989	All together	AlexNet	todos aureolatum (50).jpg	fold4	1	*Amblyomma*
2990	All together	AlexNet	todos aureolatum (52).jpg	fold4	1	*Amblyomma*
2991	All together	AlexNet	todos aureolatum (59).jpg	fold4	1	*Amblyomma*
2992	All together	AlexNet	todos aureolatum (61).jpg	fold4	1	*Amblyomma*
2993	All together	AlexNet	todos aureolatum (70).jpg	fold4	1	*Amblyomma*
2994	All together	AlexNet	todos aureolatum (88).jpg	fold4	1	*Amblyomma*
2995	All together	AlexNet	todos aureolatum (9).jpg	fold4	1	*Amblyomma*
2996	All together	AlexNet	todos aureolatum (91).jpg	fold4	1	*Amblyomma*
2997	All together	AlexNet	todos aureolatum (95).jpg	fold4	1	*Amblyomma*
2998	All together	AlexNet	todos aureolatum (98).jpg	fold4	1	*Amblyomma*
2999	All together	AlexNet	todos aureolatum (99).jpg	fold4	1	*Amblyomma*
3000	All together	AlexNet	todos cajennense (1).jpg	fold4	2	*Amblyomma*
3001	All together	AlexNet	todos cajennense (103).jpg	fold4	2	*Amblyomma*
3002	All together	AlexNet	todos cajennense (107).jpg	fold4	2	*Amblyomma*
3003	All together	AlexNet	todos cajennense (118).jpg	fold4	2	*Amblyomma*
3004	All together	AlexNet	todos cajennense (119).jpg	fold4	2	*Amblyomma*
3005	All together	AlexNet	todos cajennense (122).jpg	fold4	2	*Amblyomma*
3006	All together	AlexNet	todos cajennense (125).jpg	fold4	2	*Amblyomma*
3007	All together	AlexNet	todos cajennense (130).jpg	fold4	2	*Amblyomma*
3008	All together	AlexNet	todos cajennense (131).jpg	fold4	2	*Amblyomma*
3009	All together	AlexNet	todos cajennense (136).jpg	fold4	2	*Amblyomma*
3010	All together	AlexNet	todos cajennense (152).jpg	fold4	2	*Amblyomma*
3011	All together	AlexNet	todos cajennense (16).jpg	fold4	2	*Amblyomma*
3012	All together	AlexNet	todos cajennense (160).jpg	fold4	2	*Amblyomma*
3013	All together	AlexNet	todos cajennense (163).jpg	fold4	2	*Amblyomma*

3014	All together	AlexNet	todos cajennense (17).jpg	fold4	2	*Amblyomma*
3015	All together	AlexNet	todos cajennense (18).jpg	fold4	2	*Amblyomma*
3016	All together	AlexNet	todos cajennense (25).jpg	fold4	2	*Amblyomma*
3017	All together	AlexNet	todos cajennense (31).jpg	fold4	2	*Amblyomma*
3018	All together	AlexNet	todos cajennense (34).jpg	fold4	2	*Amblyomma*
3019	All together	AlexNet	todos cajennense (35).jpg	fold4	2	*Amblyomma*
3020	All together	AlexNet	todos cajennense (50).jpg	fold4	2	*Amblyomma*
3021	All together	AlexNet	todos cajennense (52).jpg	fold4	2	*Amblyomma*
3022	All together	AlexNet	todos cajennense (58).jpg	fold4	2	*Amblyomma*
3023	All together	AlexNet	todos cajennense (66).jpg	fold4	2	*Amblyomma*
3024	All together	AlexNet	todos cajennense (68).jpg	fold4	2	*Amblyomma*
3025	All together	AlexNet	todos cajennense (69).jpg	fold4	2	*Amblyomma*
3026	All together	AlexNet	todos cajennense (7).jpg	fold4	2	*Amblyomma*
3027	All together	AlexNet	todos cajennense (80).jpg	fold4	2	*Amblyomma*
3028	All together	AlexNet	todos cajennense (81).jpg	fold4	2	*Amblyomma*
3029	All together	AlexNet	todos cajennense (89).jpg	fold4	2	*Amblyomma*
3030	All together	AlexNet	todos cajennense (91).jpg	fold4	2	*Amblyomma*
3031	All together	AlexNet	todos cajennense (92).jpg	fold4	2	*Amblyomma*
3032	All together	AlexNet	todos cajennense (96).jpg	fold4	2	*Amblyomma*
3033	All together	AlexNet	todos dubitatum (18).jpg	fold4	3	*Amblyomma*
3034	All together	AlexNet	todos dubitatum (27).jpg	fold4	3	*Amblyomma*
3035	All together	AlexNet	todos dubitatum (32).jpg	fold4	3	*Amblyomma*
3036	All together	AlexNet	todos dubitatum (33).jpg	fold4	3	*Amblyomma*
3037	All together	AlexNet	todos dubitatum (35).jpg	fold4	3	*Amblyomma*
3038	All together	AlexNet	todos dubitatum (4).jpg	fold4	3	*Amblyomma*
3039	All together	AlexNet	todos dubitatum (45).jpg	fold4	3	*Amblyomma*
3040	All together	AlexNet	todos dubitatum (50).jpg	fold4	3	*Amblyomma*
3041	All together	AlexNet	todos dubitatum (53).jpg	fold4	3	*Amblyomma*
3042	All together	AlexNet	todos dubitatum (58).jpg	fold4	3	*Amblyomma*
3043	All together	AlexNet	todos dubitatum (59).jpg	fold4	3	*Amblyomma*
3044	All together	AlexNet	todos dubitatum (64).jpg	fold4	3	*Amblyomma*
3045	All together	AlexNet	todos dubitatum (65).jpg	fold4	3	*Amblyomma*
3046	All together	AlexNet	todos dubitatum (7).jpg	fold4	3	*Amblyomma*
3047	All together	AlexNet	todos dubitatum (72).jpg	fold4	3	*Amblyomma*
3048	All together	AlexNet	todos dubitatum (73).jpg	fold4	3	*Amblyomma*
3049	All together	AlexNet	todos dubitatum (75).jpg	fold4	3	*Amblyomma*
3050	All together	AlexNet	todos dubitatum (79).jpg	fold4	3	*Amblyomma*
3051	All together	AlexNet	todos dubitatum (84).jpg	fold4	3	*Amblyomma*
3052	All together	AlexNet	todos dubitatum (88).jpg	fold4	3	*Amblyomma*
3053	All together	AlexNet	todos dubitatum (9).jpg	fold4	3	*Amblyomma*
3054	All together	AlexNet	todos dubitatum (99).jpg	fold4	3	*Amblyomma*
3055	All together	AlexNet	todos ovale (1).jpg	fold4	4	*Amblyomma*
3056	All together	AlexNet	todos ovale (103).jpg	fold4	4	*Amblyomma*
3057	All together	AlexNet	todos ovale (111).jpg	fold4	4	*Amblyomma*
3058	All together	AlexNet	todos ovale (113).jpg	fold4	4	*Amblyomma*
3059	All together	AlexNet	todos ovale (115).jpg	fold4	4	*Amblyomma*
3060	All together	AlexNet	todos ovale (15).jpg	fold4	4	*Amblyomma*
3061	All together	AlexNet	todos ovale (17).jpg	fold4	4	*Amblyomma*
3062	All together	AlexNet	todos ovale (21).jpg	fold4	4	*Amblyomma*
3063	All together	AlexNet	todos ovale (26).jpg	fold4	4	*Amblyomma*
3064	All together	AlexNet	todos ovale (28).jpg	fold4	4	*Amblyomma*
3065	All together	AlexNet	todos ovale (37).jpg	fold4	4	*Amblyomma*
3066	All together	AlexNet	todos ovale (39).jpg	fold4	4	*Amblyomma*
3067	All together	AlexNet	todos ovale (40).jpg	fold4	4	*Amblyomma*
3068	All together	AlexNet	todos ovale (51).jpg	fold4	4	*Amblyomma*
3069	All together	AlexNet	todos ovale (6).jpg	fold4	4	*Amblyomma*
3070	All together	AlexNet	todos ovale (63).jpg	fold4	4	*Amblyomma*
3071	All together	AlexNet	todos ovale (64).jpg	fold4	4	*Amblyomma*
3072	All together	AlexNet	todos ovale (69).jpg	fold4	4	*Amblyomma*
3073	All together	AlexNet	todos ovale (72).jpg	fold4	4	*Amblyomma*
3074	All together	AlexNet	todos ovale (80).jpg	fold4	4	*Amblyomma*
3075	All together	AlexNet	todos ovale (88).jpg	fold4	4	*Amblyomma*
3076	All together	AlexNet	todos ovale (9).jpg	fold4	4	*Amblyomma*
3077	All together	AlexNet	todos ovale (90).jpg	fold4	4	*Amblyomma*
3078	All together	AlexNet	todos ovale (91).jpg	fold4	4	*Amblyomma*
3079	All together	AlexNet	todos ovale (98).jpg	fold4	4	*Amblyomma*
3080	All together	AlexNet	todos sculptum (10).JPG	fold4	5	*Amblyomma*

3081	All together	AlexNet	todos sculptum (104).JPG	fold4	5	*Amblyomma*
3082	All together	AlexNet	todos sculptum (110).JPG	fold4	5	*Amblyomma*
3083	All together	AlexNet	todos sculptum (117).JPG	fold4	5	*Amblyomma*
3084	All together	AlexNet	todos sculptum (129).JPG	fold4	5	*Amblyomma*
3085	All together	AlexNet	todos sculptum (134).JPG	fold4	5	*Amblyomma*
3086	All together	AlexNet	todos sculptum (136).JPG	fold4	5	*Amblyomma*
3087	All together	AlexNet	todos sculptum (143).JPG	fold4	5	*Amblyomma*
3088	All together	AlexNet	todos sculptum (148).jpg	fold4	5	*Amblyomma*
3089	All together	AlexNet	todos sculptum (15).JPG	fold4	5	*Amblyomma*
3090	All together	AlexNet	todos sculptum (153).jpg	fold4	5	*Amblyomma*
3091	All together	AlexNet	todos sculptum (16).JPG	fold4	5	*Amblyomma*
3092	All together	AlexNet	todos sculptum (21).JPG	fold4	5	*Amblyomma*
3093	All together	AlexNet	todos sculptum (23).JPG	fold4	5	*Amblyomma*
3094	All together	AlexNet	todos sculptum (24).JPG	fold4	5	*Amblyomma*
3095	All together	AlexNet	todos sculptum (27).JPG	fold4	5	*Amblyomma*
3096	All together	AlexNet	todos sculptum (29).JPG	fold4	5	*Amblyomma*
3097	All together	AlexNet	todos sculptum (31).JPG	fold4	5	*Amblyomma*
3098	All together	AlexNet	todos sculptum (40).JPG	fold4	5	*Amblyomma*
3099	All together	AlexNet	todos sculptum (42).JPG	fold4	5	*Amblyomma*
3100	All together	AlexNet	todos sculptum (46).JPG	fold4	5	*Amblyomma*
3101	All together	AlexNet	todos sculptum (49).JPG	fold4	5	*Amblyomma*
3102	All together	AlexNet	todos sculptum (54).JPG	fold4	5	*Amblyomma*
3103	All together	AlexNet	todos sculptum (62).jpg	fold4	5	*Amblyomma*
3104	All together	AlexNet	todos sculptum (64).jpg	fold4	5	*Amblyomma*
3105	All together	AlexNet	todos sculptum (65).jpg	fold4	5	*Amblyomma*
3106	All together	AlexNet	todos sculptum (66).jpg	fold4	5	*Amblyomma*
3107	All together	AlexNet	todos sculptum (68).jpg	fold4	5	*Amblyomma*
3108	All together	AlexNet	todos sculptum (72).JPG	fold4	5	*Amblyomma*
3109	All together	AlexNet	todos sculptum (81).JPG	fold4	5	*Amblyomma*
3110	All together	AlexNet	todos sculptum (91).JPG	fold4	5	*Amblyomma*
3111	All together	AlexNet	todos triste (1).jpg	fold4	6	*Amblyomma*
3112	All together	AlexNet	todos triste (105).jpg	fold4	6	*Amblyomma*
3113	All together	AlexNet	todos triste (107).jpg	fold4	6	*Amblyomma*
3114	All together	AlexNet	todos triste (11).jpg	fold4	6	*Amblyomma*
3115	All together	AlexNet	todos triste (113).jpg	fold4	6	*Amblyomma*
3116	All together	AlexNet	todos triste (114).jpg	fold4	6	*Amblyomma*
3117	All together	AlexNet	todos triste (122).jpg	fold4	6	*Amblyomma*
3118	All together	AlexNet	todos triste (125).jpg	fold4	6	*Amblyomma*
3119	All together	AlexNet	todos triste (134).jpg	fold4	6	*Amblyomma*
3120	All together	AlexNet	todos triste (14).jpg	fold4	6	*Amblyomma*
3121	All together	AlexNet	todos triste (145).jpg	fold4	6	*Amblyomma*
3122	All together	AlexNet	todos triste (151).jpg	fold4	6	*Amblyomma*
3123	All together	AlexNet	todos triste (155).jpg	fold4	6	*Amblyomma*
3124	All together	AlexNet	todos triste (156).jpg	fold4	6	*Amblyomma*
3125	All together	AlexNet	todos triste (18).jpg	fold4	6	*Amblyomma*
3126	All together	AlexNet	todos triste (28).jpg	fold4	6	*Amblyomma*
3127	All together	AlexNet	todos triste (33).jpg	fold4	6	*Amblyomma*
3128	All together	AlexNet	todos triste (40).jpg	fold4	6	*Amblyomma*
3129	All together	AlexNet	todos triste (41).jpg	fold4	6	*Amblyomma*
3130	All together	AlexNet	todos triste (46).jpg	fold4	6	*Amblyomma*
3131	All together	AlexNet	todos triste (47).jpg	fold4	6	*Amblyomma*
3132	All together	AlexNet	todos triste (6).jpg	fold4	6	*Amblyomma*
3133	All together	AlexNet	todos triste (60).jpg	fold4	6	*Amblyomma*
3134	All together	AlexNet	todos triste (66).jpg	fold4	6	*Amblyomma*
3135	All together	AlexNet	todos triste (7).jpg	fold4	6	*Amblyomma*
3136	All together	AlexNet	todos triste (77).jpg	fold4	6	*Amblyomma*
3137	All together	AlexNet	todos triste (85).jpg	fold4	6	*Amblyomma*
3138	All together	AlexNet	todos triste (88).jpg	fold4	6	*Amblyomma*
3139	All together	AlexNet	todos triste (90).jpg	fold4	6	*Amblyomma*
3140	All together	AlexNet	todos triste (94).jpg	fold4	6	*Amblyomma*
3141	All together	AlexNet	todos triste (97).jpg	fold4	6	*Amblyomma*
3142	All together	AlexNet	todos triste (99).jpg	fold4	6	*Amblyomma*
3143	All together	AlexNet	todos aureolatum (10).jpg	fold5	1	*Amblyomma*
3144	All together	AlexNet	todos aureolatum (109).jpg	fold5	1	*Amblyomma*
3145	All together	AlexNet	todos aureolatum (119).jpg	fold5	1	*Amblyomma*
3146	All together	AlexNet	todos aureolatum (14).jpg	fold5	1	*Amblyomma*
3147	All together	AlexNet	todos aureolatum (19).jpg	fold5	1	*Amblyomma*

3148	All together	AlexNet	todos aureolatum (22).jpg	fold5	1	*Amblyomma*
3149	All together	AlexNet	todos aureolatum (25).jpg	fold5	1	*Amblyomma*
3150	All together	AlexNet	todos aureolatum (30).jpg	fold5	1	*Amblyomma*
3151	All together	AlexNet	todos aureolatum (37).jpg	fold5	1	*Amblyomma*
3152	All together	AlexNet	todos aureolatum (39).jpg	fold5	1	*Amblyomma*
3153	All together	AlexNet	todos aureolatum (40).jpg	fold5	1	*Amblyomma*
3154	All together	AlexNet	todos aureolatum (48).jpg	fold5	1	*Amblyomma*
3155	All together	AlexNet	todos aureolatum (5).jpg	fold5	1	*Amblyomma*
3156	All together	AlexNet	todos aureolatum (51).jpg	fold5	1	*Amblyomma*
3157	All together	AlexNet	todos aureolatum (53).jpg	fold5	1	*Amblyomma*
3158	All together	AlexNet	todos aureolatum (54).jpg	fold5	1	*Amblyomma*
3159	All together	AlexNet	todos aureolatum (55).jpg	fold5	1	*Amblyomma*
3160	All together	AlexNet	todos aureolatum (58).jpg	fold5	1	*Amblyomma*
3161	All together	AlexNet	todos aureolatum (62).jpg	fold5	1	*Amblyomma*
3162	All together	AlexNet	todos aureolatum (65).jpg	fold5	1	*Amblyomma*
3163	All together	AlexNet	todos aureolatum (75).jpg	fold5	1	*Amblyomma*
3164	All together	AlexNet	todos aureolatum (78).jpg	fold5	1	*Amblyomma*
3165	All together	AlexNet	todos aureolatum (81).jpg	fold5	1	*Amblyomma*
3166	All together	AlexNet	todos aureolatum (87).jpg	fold5	1	*Amblyomma*
3167	All together	AlexNet	todos cajennense (104).jpg	fold5	2	*Amblyomma*
3168	All together	AlexNet	todos cajennense (105).jpg	fold5	2	*Amblyomma*
3169	All together	AlexNet	todos cajennense (108).jpg	fold5	2	*Amblyomma*
3170	All together	AlexNet	todos cajennense (132).jpg	fold5	2	*Amblyomma*
3171	All together	AlexNet	todos cajennense (14).jpg	fold5	2	*Amblyomma*
3172	All together	AlexNet	todos cajennense (140).jpg	fold5	2	*Amblyomma*
3173	All together	AlexNet	todos cajennense (141).jpg	fold5	2	*Amblyomma*
3174	All together	AlexNet	todos cajennense (145).jpg	fold5	2	*Amblyomma*
3175	All together	AlexNet	todos cajennense (147).jpg	fold5	2	*Amblyomma*
3176	All together	AlexNet	todos cajennense (15).jpg	fold5	2	*Amblyomma*
3177	All together	AlexNet	todos cajennense (150).jpg	fold5	2	*Amblyomma*
3178	All together	AlexNet	todos cajennense (153).jpg	fold5	2	*Amblyomma*
3179	All together	AlexNet	todos cajennense (161).jpg	fold5	2	*Amblyomma*
3180	All together	AlexNet	todos cajennense (19).jpg	fold5	2	*Amblyomma*
3181	All together	AlexNet	todos cajennense (2).jpg	fold5	2	*Amblyomma*
3182	All together	AlexNet	todos cajennense (20).jpg	fold5	2	*Amblyomma*
3183	All together	AlexNet	todos cajennense (4).jpg	fold5	2	*Amblyomma*
3184	All together	AlexNet	todos cajennense (41).jpg	fold5	2	*Amblyomma*
3185	All together	AlexNet	todos cajennense (42).jpg	fold5	2	*Amblyomma*
3186	All together	AlexNet	todos cajennense (53).jpg	fold5	2	*Amblyomma*
3187	All together	AlexNet	todos cajennense (55).jpg	fold5	2	*Amblyomma*
3188	All together	AlexNet	todos cajennense (62).jpg	fold5	2	*Amblyomma*
3189	All together	AlexNet	todos cajennense (63).jpg	fold5	2	*Amblyomma*
3190	All together	AlexNet	todos cajennense (73).jpg	fold5	2	*Amblyomma*
3191	All together	AlexNet	todos cajennense (75).jpg	fold5	2	*Amblyomma*
3192	All together	AlexNet	todos cajennense (77).jpg	fold5	2	*Amblyomma*
3193	All together	AlexNet	todos cajennense (79).jpg	fold5	2	*Amblyomma*
3194	All together	AlexNet	todos cajennense (8).jpg	fold5	2	*Amblyomma*
3195	All together	AlexNet	todos cajennense (83).jpg	fold5	2	*Amblyomma*
3196	All together	AlexNet	todos cajennense (86).jpg	fold5	2	*Amblyomma*
3197	All together	AlexNet	todos cajennense (9).jpg	fold5	2	*Amblyomma*
3198	All together	AlexNet	todos cajennense (90).jpg	fold5	2	*Amblyomma*
3199	All together	AlexNet	todos cajennense (93).jpg	fold5	2	*Amblyomma*
3200	All together	AlexNet	todos dubitatum (10).jpg	fold5	3	*Amblyomma*
3201	All together	AlexNet	todos dubitatum (100).jpg	fold5	3	*Amblyomma*
3202	All together	AlexNet	todos dubitatum (102).jpg	fold5	3	*Amblyomma*
3203	All together	AlexNet	todos dubitatum (106).jpg	fold5	3	*Amblyomma*
3204	All together	AlexNet	todos dubitatum (16).jpg	fold5	3	*Amblyomma*
3205	All together	AlexNet	todos dubitatum (17).jpg	fold5	3	*Amblyomma*
3206	All together	AlexNet	todos dubitatum (21).jpg	fold5	3	*Amblyomma*
3207	All together	AlexNet	todos dubitatum (29).jpg	fold5	3	*Amblyomma*
3208	All together	AlexNet	todos dubitatum (30).jpg	fold5	3	*Amblyomma*
3209	All together	AlexNet	todos dubitatum (31).jpg	fold5	3	*Amblyomma*
3210	All together	AlexNet	todos dubitatum (38).jpg	fold5	3	*Amblyomma*
3211	All together	AlexNet	todos dubitatum (51).jpg	fold5	3	*Amblyomma*
3212	All together	AlexNet	todos dubitatum (54).jpg	fold5	3	*Amblyomma*
3213	All together	AlexNet	todos dubitatum (61).jpg	fold5	3	*Amblyomma*
3214	All together	AlexNet	todos dubitatum (68).jpg	fold5	3	*Amblyomma*

3215	All together	AlexNet	todos dubitatum (80).jpg	fold5	3	*Amblyomma*
3216	All together	AlexNet	todos dubitatum (86).jpg	fold5	3	*Amblyomma*
3217	All together	AlexNet	todos dubitatum (89).jpg	fold5	3	*Amblyomma*
3218	All together	AlexNet	todos dubitatum (91).jpg	fold5	3	*Amblyomma*
3219	All together	AlexNet	todos dubitatum (97).jpg	fold5	3	*Amblyomma*
3220	All together	AlexNet	todos dubitatum (98).jpg	fold5	3	*Amblyomma*
3221	All together	AlexNet	todos ovale (102).jpg	fold5	4	*Amblyomma*
3222	All together	AlexNet	todos ovale (114).jpg	fold5	4	*Amblyomma*
3223	All together	AlexNet	todos ovale (117).jpg	fold5	4	*Amblyomma*
3224	All together	AlexNet	todos ovale (119).jpg	fold5	4	*Amblyomma*
3225	All together	AlexNet	todos ovale (12).jpg	fold5	4	*Amblyomma*
3226	All together	AlexNet	todos ovale (14).jpg	fold5	4	*Amblyomma*
3227	All together	AlexNet	todos ovale (18).jpg	fold5	4	*Amblyomma*
3228	All together	AlexNet	todos ovale (31).jpg	fold5	4	*Amblyomma*
3229	All together	AlexNet	todos ovale (35).jpg	fold5	4	*Amblyomma*
3230	All together	AlexNet	todos ovale (45).jpg	fold5	4	*Amblyomma*
3231	All together	AlexNet	todos ovale (48).jpg	fold5	4	*Amblyomma*
3232	All together	AlexNet	todos ovale (49).jpg	fold5	4	*Amblyomma*
3233	All together	AlexNet	todos ovale (5).jpg	fold5	4	*Amblyomma*
3234	All together	AlexNet	todos ovale (50).jpg	fold5	4	*Amblyomma*
3235	All together	AlexNet	todos ovale (53).jpg	fold5	4	*Amblyomma*
3236	All together	AlexNet	todos ovale (59).jpg	fold5	4	*Amblyomma*
3237	All together	AlexNet	todos ovale (61).jpg	fold5	4	*Amblyomma*
3238	All together	AlexNet	todos ovale (66).jpg	fold5	4	*Amblyomma*
3239	All together	AlexNet	todos ovale (73).jpg	fold5	4	*Amblyomma*
3240	All together	AlexNet	todos ovale (75).JPG	fold5	4	*Amblyomma*
3241	All together	AlexNet	todos ovale (77).jpg	fold5	4	*Amblyomma*
3242	All together	AlexNet	todos ovale (79).jpg	fold5	4	*Amblyomma*
3243	All together	AlexNet	todos ovale (89).jpg	fold5	4	*Amblyomma*
3244	All together	AlexNet	todos ovale (94).jpg	fold5	4	*Amblyomma*
3245	All together	AlexNet	todos sculptum (1).jpg	fold5	5	*Amblyomma*
3246	All together	AlexNet	todos sculptum (101).JPG	fold5	5	*Amblyomma*
3247	All together	AlexNet	todos sculptum (111).JPG	fold5	5	*Amblyomma*
3248	All together	AlexNet	todos sculptum (131).JPG	fold5	5	*Amblyomma*
3249	All together	AlexNet	todos sculptum (137).JPG	fold5	5	*Amblyomma*
3250	All together	AlexNet	todos sculptum (144).JPG	fold5	5	*Amblyomma*
3251	All together	AlexNet	todos sculptum (145).jpg	fold5	5	*Amblyomma*
3252	All together	AlexNet	todos sculptum (146).jpg	fold5	5	*Amblyomma*
3253	All together	AlexNet	todos sculptum (149).jpg	fold5	5	*Amblyomma*
3254	All together	AlexNet	todos sculptum (150).jpg	fold5	5	*Amblyomma*
3255	All together	AlexNet	todos sculptum (151).jpg	fold5	5	*Amblyomma*
3256	All together	AlexNet	todos sculptum (2).jpg	fold5	5	*Amblyomma*
3257	All together	AlexNet	todos sculptum (34).JPG	fold5	5	*Amblyomma*
3258	All together	AlexNet	todos sculptum (43).JPG	fold5	5	*Amblyomma*
3259	All together	AlexNet	todos sculptum (44).JPG	fold5	5	*Amblyomma*
3260	All together	AlexNet	todos sculptum (50).JPG	fold5	5	*Amblyomma*
3261	All together	AlexNet	todos sculptum (53).JPG	fold5	5	*Amblyomma*
3262	All together	AlexNet	todos sculptum (56).jpg	fold5	5	*Amblyomma*
3263	All together	AlexNet	todos sculptum (58).jpg	fold5	5	*Amblyomma*
3264	All together	AlexNet	todos sculptum (59).jpg	fold5	5	*Amblyomma*
3265	All together	AlexNet	todos sculptum (6).jpg	fold5	5	*Amblyomma*
3266	All together	AlexNet	todos sculptum (67).jpg	fold5	5	*Amblyomma*
3267	All together	AlexNet	todos sculptum (77).JPG	fold5	5	*Amblyomma*
3268	All together	AlexNet	todos sculptum (78).JPG	fold5	5	*Amblyomma*
3269	All together	AlexNet	todos sculptum (79).JPG	fold5	5	*Amblyomma*
3270	All together	AlexNet	todos sculptum (8).jpg	fold5	5	*Amblyomma*
3271	All together	AlexNet	todos sculptum (82).JPG	fold5	5	*Amblyomma*
3272	All together	AlexNet	todos sculptum (86).JPG	fold5	5	*Amblyomma*
3273	All together	AlexNet	todos sculptum (92).JPG	fold5	5	*Amblyomma*
3274	All together	AlexNet	todos sculptum (94).JPG	fold5	5	*Amblyomma*
3275	All together	AlexNet	todos sculptum (95).JPG	fold5	5	*Amblyomma*
3276	All together	AlexNet	todos triste (10).jpg	fold5	6	*Amblyomma*
3277	All together	AlexNet	todos triste (100).jpg	fold5	6	*Amblyomma*
3278	All together	AlexNet	todos triste (108).jpg	fold5	6	*Amblyomma*
3279	All together	AlexNet	todos triste (111).jpg	fold5	6	*Amblyomma*
3280	All together	AlexNet	todos triste (112).jpg	fold5	6	*Amblyomma*
3281	All together	AlexNet	todos triste (117).jpg	fold5	6	*Amblyomma*

3282	All together	AlexNet	todos triste (119).jpg	fold5	6 *Amblyomma*
3283	All together	AlexNet	todos triste (12).jpg	fold5	6 *Amblyomma*
3284	All together	AlexNet	todos triste (124).jpg	fold5	6 *Amblyomma*
3285	All together	AlexNet	todos triste (138).jpg	fold5	6 *Amblyomma*
3286	All together	AlexNet	todos triste (143).jpg	fold5	6 *Amblyomma*
3287	All together	AlexNet	todos triste (150).jpg	fold5	6 *Amblyomma*
3288	All together	AlexNet	todos triste (16).jpg	fold5	6 *Amblyomma*
3289	All together	AlexNet	todos triste (19).jpg	fold5	6 *Amblyomma*
3290	All together	AlexNet	todos triste (25).jpg	fold5	6 *Amblyomma*
3291	All together	AlexNet	todos triste (30).jpg	fold5	6 *Amblyomma*
3292	All together	AlexNet	todos triste (31).jpg	fold5	6 *Amblyomma*
3293	All together	AlexNet	todos triste (34).jpg	fold5	6 *Amblyomma*
3294	All together	AlexNet	todos triste (39).jpg	fold5	6 *Amblyomma*
3295	All together	AlexNet	todos triste (4).jpg	fold5	6 *Amblyomma*
3296	All together	AlexNet	todos triste (62).jpg	fold5	6 *Amblyomma*
3297	All together	AlexNet	todos triste (63).jpg	fold5	6 *Amblyomma*
3298	All together	AlexNet	todos triste (67).jpg	fold5	6 *Amblyomma*
3299	All together	AlexNet	todos triste (71).jpg	fold5	6 *Amblyomma*
3300	All together	AlexNet	todos triste (72).jpg	fold5	6 *Amblyomma*
3301	All together	AlexNet	todos triste (75).jpg	fold5	6 *Amblyomma*
3302	All together	AlexNet	todos triste (80).jpg	fold5	6 *Amblyomma*
3303	All together	AlexNet	todos triste (82).jpg	fold5	6 *Amblyomma*
3304	All together	AlexNet	todos triste (89).jpg	fold5	6 *Amblyomma*
3305	All together	AlexNet	todos triste (91).jpg	fold5	6 *Amblyomma*
3306	All together	AlexNet	todos triste (98).jpg	fold5	6 *Amblyomma*
3307	Female	MobileNetV2	femea aureolatum (15).jpg	fold1	1 *Amblyomma*
3308	Female	MobileNetV2	femea aureolatum (27).jpg	fold1	1 *Amblyomma*
3309	Female	MobileNetV2	femea aureolatum (30).jpg	fold1	1 *Amblyomma*
3310	Female	MobileNetV2	femea aureolatum (35).jpg	fold1	1 *Amblyomma*
3311	Female	MobileNetV2	femea aureolatum (36).jpg	fold1	1 *Amblyomma*
3312	Female	MobileNetV2	femea aureolatum (43).jpg	fold1	1 *Amblyomma*
3313	Female	MobileNetV2	femea aureolatum (44).jpg	fold1	1 *Amblyomma*
3314	Female	MobileNetV2	femea aureolatum (46).jpg	fold1	1 *Amblyomma*
3315	Female	MobileNetV2	femea aureolatum (48).jpg	fold1	1 *Amblyomma*
3316	Female	MobileNetV2	femea aureolatum (5).jpg	fold1	1 *Amblyomma*
3317	Female	MobileNetV2	femea aureolatum (53).jpg	fold1	1 *Amblyomma*
3318	Female	MobileNetV2	femea cajennense (11).jpg	fold1	2 *Amblyomma*
3319	Female	MobileNetV2	femea cajennense (14).jpg	fold1	2 *Amblyomma*
3320	Female	MobileNetV2	femea cajennense (17).jpg	fold1	2 *Amblyomma*
3321	Female	MobileNetV2	femea cajennense (18).jpg	fold1	2 *Amblyomma*
3322	Female	MobileNetV2	femea cajennense (25).jpg	fold1	2 *Amblyomma*
3323	Female	MobileNetV2	femea cajennense (27).jpg	fold1	2 *Amblyomma*
3324	Female	MobileNetV2	femea cajennense (36).jpg	fold1	2 *Amblyomma*
3325	Female	MobileNetV2	femea cajennense (37).jpg	fold1	2 *Amblyomma*
3326	Female	MobileNetV2	femea cajennense (39).jpg	fold1	2 *Amblyomma*
3327	Female	MobileNetV2	femea cajennense (47).jpg	fold1	2 *Amblyomma*
3328	Female	MobileNetV2	femea cajennense (55).jpg	fold1	2 *Amblyomma*
3329	Female	MobileNetV2	femea cajennense (58).jpg	fold1	2 *Amblyomma*
3330	Female	MobileNetV2	femea cajennense (60).jpg	fold1	2 *Amblyomma*
3331	Female	MobileNetV2	femea cajennense (62).jpg	fold1	2 *Amblyomma*
3332	Female	MobileNetV2	femea cajennense (68).jpg	fold1	2 *Amblyomma*
3333	Female	MobileNetV2	femea cajennense (69).jpg	fold1	2 *Amblyomma*
3334	Female	MobileNetV2	femea cajennense (76).jpg	fold1	2 *Amblyomma*
3335	Female	MobileNetV2	femea dubitatum (11).jpg	fold1	3 *Amblyomma*
3336	Female	MobileNetV2	femea dubitatum (2).jpg	fold1	3 *Amblyomma*
3337	Female	MobileNetV2	femea dubitatum (23).jpg	fold1	3 *Amblyomma*
3338	Female	MobileNetV2	femea dubitatum (26).jpg	fold1	3 *Amblyomma*
3339	Female	MobileNetV2	femea dubitatum (28).jpg	fold1	3 *Amblyomma*
3340	Female	MobileNetV2	femea dubitatum (6).jpg	fold1	3 *Amblyomma*
3341	Female	MobileNetV2	femea dubitatum (8).jpg	fold1	3 *Amblyomma*
3342	Female	MobileNetV2	femea ovale (15).jpg	fold1	4 *Amblyomma*
3343	Female	MobileNetV2	femea ovale (17).jpg	fold1	4 *Amblyomma*
3344	Female	MobileNetV2	femea ovale (20).jpg	fold1	4 *Amblyomma*
3345	Female	MobileNetV2	femea ovale (22).jpg	fold1	4 *Amblyomma*
3346	Female	MobileNetV2	femea ovale (25).jpg	fold1	4 *Amblyomma*
3347	Female	MobileNetV2	femea ovale (28).jpg	fold1	4 *Amblyomma*
3348	Female	MobileNetV2	femea ovale (33).jpg	fold1	4 *Amblyomma*

3349	Female	MobileNetV2	femea ovale (35).jpg	fold1	4	*Amblyomma*
3350	Female	MobileNetV2	femea ovale (44).jpg	fold1	4	*Amblyomma*
3351	Female	MobileNetV2	femea ovale (51).jpg	fold1	4	*Amblyomma*
3352	Female	MobileNetV2	femea ovale (53).jpg	fold1	4	*Amblyomma*
3353	Female	MobileNetV2	femea ovale (59).jpg	fold1	4	*Amblyomma*
3354	Female	MobileNetV2	femea ovale (61).jpg	fold1	4	*Amblyomma*
3355	Female	MobileNetV2	femea ovale (62).jpg	fold1	4	*Amblyomma*
3356	Female	MobileNetV2	femea ovale (74).jpg	fold1	4	*Amblyomma*
3357	Female	MobileNetV2	femea ovale (75).jpg	fold1	4	*Amblyomma*
3358	Female	MobileNetV2	femea sculptum (1).jpg	fold1	5	*Amblyomma*
3359	Female	MobileNetV2	femea sculptum (11).JPG	fold1	5	*Amblyomma*
3360	Female	MobileNetV2	femea sculptum (13).JPG	fold1	5	*Amblyomma*
3361	Female	MobileNetV2	femea sculptum (14).JPG	fold1	5	*Amblyomma*
3362	Female	MobileNetV2	femea sculptum (22).JPG	fold1	5	*Amblyomma*
3363	Female	MobileNetV2	femea sculptum (39).JPG	fold1	5	*Amblyomma*
3364	Female	MobileNetV2	femea sculptum (58).jpg	fold1	5	*Amblyomma*
3365	Female	MobileNetV2	femea sculptum (6).jpg	fold1	5	*Amblyomma*
3366	Female	MobileNetV2	femea sculptum (60).jpg	fold1	5	*Amblyomma*
3367	Female	MobileNetV2	femea sculptum (61).jpg	fold1	5	*Amblyomma*
3368	Female	MobileNetV2	femea sculptum (62).jpg	fold1	5	*Amblyomma*
3369	Female	MobileNetV2	femea sculptum (8).jpg	fold1	5	*Amblyomma*
3370	Female	MobileNetV2	femea sculptum (9).JPG	fold1	5	*Amblyomma*
3371	Female	MobileNetV2	femea triste (12).jpg	fold1	6	*Amblyomma*
3372	Female	MobileNetV2	femea triste (13).jpg	fold1	6	*Amblyomma*
3373	Female	MobileNetV2	femea triste (14).jpg	fold1	6	*Amblyomma*
3374	Female	MobileNetV2	femea triste (23).jpg	fold1	6	*Amblyomma*
3375	Female	MobileNetV2	femea triste (26).jpg	fold1	6	*Amblyomma*
3376	Female	MobileNetV2	femea triste (32).jpg	fold1	6	*Amblyomma*
3377	Female	MobileNetV2	femea triste (35).jpg	fold1	6	*Amblyomma*
3378	Female	MobileNetV2	femea triste (38).jpg	fold1	6	*Amblyomma*
3379	Female	MobileNetV2	femea triste (45).jpg	fold1	6	*Amblyomma*
3380	Female	MobileNetV2	femea triste (46).jpg	fold1	6	*Amblyomma*
3381	Female	MobileNetV2	femea triste (47).jpg	fold1	6	*Amblyomma*
3382	Female	MobileNetV2	femea aureolatum (1).jpg	fold2	1	*Amblyomma*
3383	Female	MobileNetV2	femea aureolatum (10).jpg	fold2	1	*Amblyomma*
3384	Female	MobileNetV2	femea aureolatum (12).jpg	fold2	1	*Amblyomma*
3385	Female	MobileNetV2	femea aureolatum (16).jpg	fold2	1	*Amblyomma*
3386	Female	MobileNetV2	femea aureolatum (22).jpg	fold2	1	*Amblyomma*
3387	Female	MobileNetV2	femea aureolatum (28).jpg	fold2	1	*Amblyomma*
3388	Female	MobileNetV2	femea aureolatum (31).jpg	fold2	1	*Amblyomma*
3389	Female	MobileNetV2	femea aureolatum (37).jpg	fold2	1	*Amblyomma*
3390	Female	MobileNetV2	femea aureolatum (39).jpg	fold2	1	*Amblyomma*
3391	Female	MobileNetV2	femea aureolatum (50).jpg	fold2	1	*Amblyomma*
3392	Female	MobileNetV2	femea aureolatum (52).jpg	fold2	1	*Amblyomma*
3393	Female	MobileNetV2	femea aureolatum (8).jpg	fold2	1	*Amblyomma*
3394	Female	MobileNetV2	femea cajennense (10).jpg	fold2	2	*Amblyomma*
3395	Female	MobileNetV2	femea cajennense (12).jpg	fold2	2	*Amblyomma*
3396	Female	MobileNetV2	femea cajennense (19).jpg	fold2	2	*Amblyomma*
3397	Female	MobileNetV2	femea cajennense (23).jpg	fold2	2	*Amblyomma*
3398	Female	MobileNetV2	femea cajennense (3).jpg	fold2	2	*Amblyomma*
3399	Female	MobileNetV2	femea cajennense (34).jpg	fold2	2	*Amblyomma*
3400	Female	MobileNetV2	femea cajennense (35).jpg	fold2	2	*Amblyomma*
3401	Female	MobileNetV2	femea cajennense (53).jpg	fold2	2	*Amblyomma*
3402	Female	MobileNetV2	femea cajennense (54).jpg	fold2	2	*Amblyomma*
3403	Female	MobileNetV2	femea cajennense (57).jpg	fold2	2	*Amblyomma*
3404	Female	MobileNetV2	femea cajennense (6).jpg	fold2	2	*Amblyomma*
3405	Female	MobileNetV2	femea cajennense (63).jpg	fold2	2	*Amblyomma*
3406	Female	MobileNetV2	femea cajennense (66).jpg	fold2	2	*Amblyomma*
3407	Female	MobileNetV2	femea cajennense (67).jpg	fold2	2	*Amblyomma*
3408	Female	MobileNetV2	femea cajennense (7).jpg	fold2	2	*Amblyomma*
3409	Female	MobileNetV2	femea cajennense (77).jpg	fold2	2	*Amblyomma*
3410	Female	MobileNetV2	femea cajennense (9).jpg	fold2	2	*Amblyomma*
3411	Female	MobileNetV2	femea dubitatum (12).jpg	fold2	3	*Amblyomma*
3412	Female	MobileNetV2	femea dubitatum (13).jpg	fold2	3	*Amblyomma*
3413	Female	MobileNetV2	femea dubitatum (19).jpg	fold2	3	*Amblyomma*
3414	Female	MobileNetV2	femea dubitatum (21).jpg	fold2	3	*Amblyomma*
3415	Female	MobileNetV2	femea dubitatum (24).jpg	fold2	3	*Amblyomma*

3416	Female	MobileNetV2	femea dubitatum (3).jpg	fold2	3	*Amblyomma*
3417	Female	MobileNetV2	femea dubitatum (33).jpg	fold2	3	*Amblyomma*
3418	Female	MobileNetV2	femea ovale (1).jpg	fold2	4	*Amblyomma*
3419	Female	MobileNetV2	femea ovale (10).jpg	fold2	4	*Amblyomma*
3420	Female	MobileNetV2	femea ovale (14).jpg	fold2	4	*Amblyomma*
3421	Female	MobileNetV2	femea ovale (18).jpg	fold2	4	*Amblyomma*
3422	Female	MobileNetV2	femea ovale (29).jpg	fold2	4	*Amblyomma*
3423	Female	MobileNetV2	femea ovale (32).jpg	fold2	4	*Amblyomma*
3424	Female	MobileNetV2	femea ovale (34).jpg	fold2	4	*Amblyomma*
3425	Female	MobileNetV2	femea ovale (36).jpg	fold2	4	*Amblyomma*
3426	Female	MobileNetV2	femea ovale (39).jpg	fold2	4	*Amblyomma*
3427	Female	MobileNetV2	femea ovale (50).jpg	fold2	4	*Amblyomma*
3428	Female	MobileNetV2	femea ovale (55).jpg	fold2	4	*Amblyomma*
3429	Female	MobileNetV2	femea ovale (56).jpg	fold2	4	*Amblyomma*
3430	Female	MobileNetV2	femea ovale (6).jpg	fold2	4	*Amblyomma*
3431	Female	MobileNetV2	femea ovale (64).jpg	fold2	4	*Amblyomma*
3432	Female	MobileNetV2	femea ovale (65).jpg	fold2	4	*Amblyomma*
3433	Female	MobileNetV2	femea ovale (66).jpg	fold2	4	*Amblyomma*
3434	Female	MobileNetV2	femea sculptum (16).JPG	fold2	5	*Amblyomma*
3435	Female	MobileNetV2	femea sculptum (24).JPG	fold2	5	*Amblyomma*
3436	Female	MobileNetV2	femea sculptum (25).JPG	fold2	5	*Amblyomma*
3437	Female	MobileNetV2	femea sculptum (26).JPG	fold2	5	*Amblyomma*
3438	Female	MobileNetV2	femea sculptum (27).JPG	fold2	5	*Amblyomma*
3439	Female	MobileNetV2	femea sculptum (30).JPG	fold2	5	*Amblyomma*
3440	Female	MobileNetV2	femea sculptum (34).JPG	fold2	5	*Amblyomma*
3441	Female	MobileNetV2	femea sculptum (36).JPG	fold2	5	*Amblyomma*
3442	Female	MobileNetV2	femea sculptum (40).JPG	fold2	5	*Amblyomma*
3443	Female	MobileNetV2	femea sculptum (42).JPG	fold2	5	*Amblyomma*
3444	Female	MobileNetV2	femea sculptum (44).JPG	fold2	5	*Amblyomma*
3445	Female	MobileNetV2	femea sculptum (46).JPG	fold2	5	*Amblyomma*
3446	Female	MobileNetV2	femea triste (19).jpg	fold2	6	*Amblyomma*
3447	Female	MobileNetV2	femea triste (22).jpg	fold2	6	*Amblyomma*
3448	Female	MobileNetV2	femea triste (24).jpg	fold2	6	*Amblyomma*
3449	Female	MobileNetV2	femea triste (27).jpg	fold2	6	*Amblyomma*
3450	Female	MobileNetV2	femea triste (30).jpg	fold2	6	*Amblyomma*
3451	Female	MobileNetV2	femea triste (31).jpg	fold2	6	*Amblyomma*
3452	Female	MobileNetV2	femea triste (36).jpg	fold2	6	*Amblyomma*
3453	Female	MobileNetV2	femea triste (42).jpg	fold2	6	*Amblyomma*
3454	Female	MobileNetV2	femea triste (43).jpg	fold2	6	*Amblyomma*
3455	Female	MobileNetV2	femea triste (5).jpg	fold2	6	*Amblyomma*
3456	Female	MobileNetV2	femea aureolatum (11).jpg	fold3	1	*Amblyomma*
3457	Female	MobileNetV2	femea aureolatum (19).jpg	fold3	1	*Amblyomma*
3458	Female	MobileNetV2	femea aureolatum (2).jpg	fold3	1	*Amblyomma*
3459	Female	MobileNetV2	femea aureolatum (21).jpg	fold3	1	*Amblyomma*
3460	Female	MobileNetV2	femea aureolatum (32).jpg	fold3	1	*Amblyomma*
3461	Female	MobileNetV2	femea aureolatum (34).jpg	fold3	1	*Amblyomma*
3462	Female	MobileNetV2	femea aureolatum (38).jpg	fold3	1	*Amblyomma*
3463	Female	MobileNetV2	femea aureolatum (4).jpg	fold3	1	*Amblyomma*
3464	Female	MobileNetV2	femea aureolatum (47).jpg	fold3	1	*Amblyomma*
3465	Female	MobileNetV2	femea aureolatum (51).jpg	fold3	1	*Amblyomma*
3466	Female	MobileNetV2	femea aureolatum (55).jpg	fold3	1	*Amblyomma*
3467	Female	MobileNetV2	femea cajennense (1).jpg	fold3	2	*Amblyomma*
3468	Female	MobileNetV2	femea cajennense (22).jpg	fold3	2	*Amblyomma*
3469	Female	MobileNetV2	femea cajennense (26).jpg	fold3	2	*Amblyomma*
3470	Female	MobileNetV2	femea cajennense (28).jpg	fold3	2	*Amblyomma*
3471	Female	MobileNetV2	femea cajennense (32).jpg	fold3	2	*Amblyomma*
3472	Female	MobileNetV2	femea cajennense (38).jpg	fold3	2	*Amblyomma*
3473	Female	MobileNetV2	femea cajennense (41).jpg	fold3	2	*Amblyomma*
3474	Female	MobileNetV2	femea cajennense (50).jpg	fold3	2	*Amblyomma*
3475	Female	MobileNetV2	femea cajennense (51).jpg	fold3	2	*Amblyomma*
3476	Female	MobileNetV2	femea cajennense (71).jpg	fold3	2	*Amblyomma*
3477	Female	MobileNetV2	femea cajennense (72).jpg	fold3	2	*Amblyomma*
3478	Female	MobileNetV2	femea cajennense (73).jpg	fold3	2	*Amblyomma*
3479	Female	MobileNetV2	femea cajennense (78).jpg	fold3	2	*Amblyomma*
3480	Female	MobileNetV2	femea cajennense (79).jpg	fold3	2	*Amblyomma*
3481	Female	MobileNetV2	femea cajennense (8).jpg	fold3	2	*Amblyomma*
3482	Female	MobileNetV2	femea cajennense (80).jpg	fold3	2	*Amblyomma*

3483	Female	MobileNetV2	femea dubitatum (16).jpg	fold3	3	*Amblyomma*
3484	Female	MobileNetV2	femea dubitatum (18).jpg	fold3	3	*Amblyomma*
3485	Female	MobileNetV2	femea dubitatum (25).jpg	fold3	3	*Amblyomma*
3486	Female	MobileNetV2	femea dubitatum (27).jpg	fold3	3	*Amblyomma*
3487	Female	MobileNetV2	femea dubitatum (29).jpg	fold3	3	*Amblyomma*
3488	Female	MobileNetV2	femea dubitatum (32).jpg	fold3	3	*Amblyomma*
3489	Female	MobileNetV2	femea dubitatum (34).jpg	fold3	3	*Amblyomma*
3490	Female	MobileNetV2	femea ovale (11).jpg	fold3	4	*Amblyomma*
3491	Female	MobileNetV2	femea ovale (19).jpg	fold3	4	*Amblyomma*
3492	Female	MobileNetV2	femea ovale (26).jpg	fold3	4	*Amblyomma*
3493	Female	MobileNetV2	femea ovale (3).JPG	fold3	4	*Amblyomma*
3494	Female	MobileNetV2	femea ovale (30).jpg	fold3	4	*Amblyomma*
3495	Female	MobileNetV2	femea ovale (31).jpg	fold3	4	*Amblyomma*
3496	Female	MobileNetV2	femea ovale (37).jpg	fold3	4	*Amblyomma*
3497	Female	MobileNetV2	femea ovale (38).jpg	fold3	4	*Amblyomma*
3498	Female	MobileNetV2	femea ovale (45).jpg	fold3	4	*Amblyomma*
3499	Female	MobileNetV2	femea ovale (49).jpg	fold3	4	*Amblyomma*
3500	Female	MobileNetV2	femea ovale (5).jpg	fold3	4	*Amblyomma*
3501	Female	MobileNetV2	femea ovale (54).jpg	fold3	4	*Amblyomma*
3502	Female	MobileNetV2	femea ovale (67).jpg	fold3	4	*Amblyomma*
3503	Female	MobileNetV2	femea ovale (71).jpg	fold3	4	*Amblyomma*
3504	Female	MobileNetV2	femea ovale (78).jpg	fold3	4	*Amblyomma*
3505	Female	MobileNetV2	femea sculptum (10).JPG	fold3	5	*Amblyomma*
3506	Female	MobileNetV2	femea sculptum (17).JPG	fold3	5	*Amblyomma*
3507	Female	MobileNetV2	femea sculptum (18).JPG	fold3	5	*Amblyomma*
3508	Female	MobileNetV2	femea sculptum (21).JPG	fold3	5	*Amblyomma*
3509	Female	MobileNetV2	femea sculptum (28).JPG	fold3	5	*Amblyomma*
3510	Female	MobileNetV2	femea sculptum (43).JPG	fold3	5	*Amblyomma*
3511	Female	MobileNetV2	femea sculptum (45).JPG	fold3	5	*Amblyomma*
3512	Female	MobileNetV2	femea sculptum (47).JPG	fold3	5	*Amblyomma*
3513	Female	MobileNetV2	femea sculptum (49).JPG	fold3	5	*Amblyomma*
3514	Female	MobileNetV2	femea sculptum (53).JPG	fold3	5	*Amblyomma*
3515	Female	MobileNetV2	femea sculptum (54).JPG	fold3	5	*Amblyomma*
3516	Female	MobileNetV2	femea sculptum (59).jpg	fold3	5	*Amblyomma*
3517	Female	MobileNetV2	femea sculptum (7).jpg	fold3	5	*Amblyomma*
3518	Female	MobileNetV2	femea triste (1).jpg	fold3	6	*Amblyomma*
3519	Female	MobileNetV2	femea triste (10).jpg	fold3	6	*Amblyomma*
3520	Female	MobileNetV2	femea triste (11).jpg	fold3	6	*Amblyomma*
3521	Female	MobileNetV2	femea triste (2).jpg	fold3	6	*Amblyomma*
3522	Female	MobileNetV2	femea triste (37).jpg	fold3	6	*Amblyomma*
3523	Female	MobileNetV2	femea triste (4).jpg	fold3	6	*Amblyomma*
3524	Female	MobileNetV2	femea triste (44).jpg	fold3	6	*Amblyomma*
3525	Female	MobileNetV2	femea triste (50).jpg	fold3	6	*Amblyomma*
3526	Female	MobileNetV2	femea triste (7).jpg	fold3	6	*Amblyomma*
3527	Female	MobileNetV2	femea triste (9).jpg	fold3	6	*Amblyomma*
3528	Female	MobileNetV2	femea aureolatum (14).jpg	fold4	1	*Amblyomma*
3529	Female	MobileNetV2	femea aureolatum (17).jpg	fold4	1	*Amblyomma*
3530	Female	MobileNetV2	femea aureolatum (20).jpg	fold4	1	*Amblyomma*
3531	Female	MobileNetV2	femea aureolatum (29).jpg	fold4	1	*Amblyomma*
3532	Female	MobileNetV2	femea aureolatum (3).jpg	fold4	1	*Amblyomma*
3533	Female	MobileNetV2	femea aureolatum (40).jpg	fold4	1	*Amblyomma*
3534	Female	MobileNetV2	femea aureolatum (42).jpg	fold4	1	*Amblyomma*
3535	Female	MobileNetV2	femea aureolatum (49).jpg	fold4	1	*Amblyomma*
3536	Female	MobileNetV2	femea aureolatum (54).jpg	fold4	1	*Amblyomma*
3537	Female	MobileNetV2	femea aureolatum (6).jpg	fold4	1	*Amblyomma*
3538	Female	MobileNetV2	femea aureolatum (7).jpg	fold4	1	*Amblyomma*
3539	Female	MobileNetV2	femea aureolatum (9).jpg	fold4	1	*Amblyomma*
3540	Female	MobileNetV2	femea cajennense (16).jpg	fold4	2	*Amblyomma*
3541	Female	MobileNetV2	femea cajennense (20).jpg	fold4	2	*Amblyomma*
3542	Female	MobileNetV2	femea cajennense (24).jpg	fold4	2	*Amblyomma*
3543	Female	MobileNetV2	femea cajennense (30).jpg	fold4	2	*Amblyomma*
3544	Female	MobileNetV2	femea cajennense (4).jpg	fold4	2	*Amblyomma*
3545	Female	MobileNetV2	femea cajennense (43).jpg	fold4	2	*Amblyomma*
3546	Female	MobileNetV2	femea cajennense (44).jpg	fold4	2	*Amblyomma*
3547	Female	MobileNetV2	femea cajennense (45).jpg	fold4	2	*Amblyomma*
3548	Female	MobileNetV2	femea cajennense (46).jpg	fold4	2	*Amblyomma*
3549	Female	MobileNetV2	femea cajennense (52).jpg	fold4	2	*Amblyomma*

3550	Female	MobileNetV2	femea cajennense (56).jpg	fold4	2	*Amblyomma*
3551	Female	MobileNetV2	femea cajennense (61).jpg	fold4	2	*Amblyomma*
3552	Female	MobileNetV2	femea cajennense (70).jpg	fold4	2	*Amblyomma*
3553	Female	MobileNetV2	femea cajennense (74).jpg	fold4	2	*Amblyomma*
3554	Female	MobileNetV2	femea cajennense (75).jpg	fold4	2	*Amblyomma*
3555	Female	MobileNetV2	femea cajennense (81).jpg	fold4	2	*Amblyomma*
3556	Female	MobileNetV2	femea dubitatum (10).jpg	fold4	3	*Amblyomma*
3557	Female	MobileNetV2	femea dubitatum (15).jpg	fold4	3	*Amblyomma*
3558	Female	MobileNetV2	femea dubitatum (17).jpg	fold4	3	*Amblyomma*
3559	Female	MobileNetV2	femea dubitatum (22).jpg	fold4	3	*Amblyomma*
3560	Female	MobileNetV2	femea dubitatum (30).jpg	fold4	3	*Amblyomma*
3561	Female	MobileNetV2	femea dubitatum (7).jpg	fold4	3	*Amblyomma*
3562	Female	MobileNetV2	femea ovale (2).jpg	fold4	4	*Amblyomma*
3563	Female	MobileNetV2	femea ovale (23).jpg	fold4	4	*Amblyomma*
3564	Female	MobileNetV2	femea ovale (24).jpg	fold4	4	*Amblyomma*
3565	Female	MobileNetV2	femea ovale (4).JPG	fold4	4	*Amblyomma*
3566	Female	MobileNetV2	femea ovale (40).jpg	fold4	4	*Amblyomma*
3567	Female	MobileNetV2	femea ovale (46).jpg	fold4	4	*Amblyomma*
3568	Female	MobileNetV2	femea ovale (48).jpg	fold4	4	*Amblyomma*
3569	Female	MobileNetV2	femea ovale (52).jpg	fold4	4	*Amblyomma*
3570	Female	MobileNetV2	femea ovale (57).jpg	fold4	4	*Amblyomma*
3571	Female	MobileNetV2	femea ovale (58).jpg	fold4	4	*Amblyomma*
3572	Female	MobileNetV2	femea ovale (7).jpg	fold4	4	*Amblyomma*
3573	Female	MobileNetV2	femea ovale (70).jpg	fold4	4	*Amblyomma*
3574	Female	MobileNetV2	femea ovale (73).jpg	fold4	4	*Amblyomma*
3575	Female	MobileNetV2	femea ovale (76).jpg	fold4	4	*Amblyomma*
3576	Female	MobileNetV2	femea ovale (77).jpg	fold4	4	*Amblyomma*
3577	Female	MobileNetV2	femea ovale (9).jpg	fold4	4	*Amblyomma*
3578	Female	MobileNetV2	femea sculptum (15).JPG	fold4	5	*Amblyomma*
3579	Female	MobileNetV2	femea sculptum (19).JPG	fold4	5	*Amblyomma*
3580	Female	MobileNetV2	femea sculptum (2).jpg	fold4	5	*Amblyomma*
3581	Female	MobileNetV2	femea sculptum (23).JPG	fold4	5	*Amblyomma*
3582	Female	MobileNetV2	femea sculptum (29).JPG	fold4	5	*Amblyomma*
3583	Female	MobileNetV2	femea sculptum (3).jpg	fold4	5	*Amblyomma*
3584	Female	MobileNetV2	femea sculptum (37).JPG	fold4	5	*Amblyomma*
3585	Female	MobileNetV2	femea sculptum (38).JPG	fold4	5	*Amblyomma*
3586	Female	MobileNetV2	femea sculptum (41).JPG	fold4	5	*Amblyomma*
3587	Female	MobileNetV2	femea sculptum (5).jpg	fold4	5	*Amblyomma*
3588	Female	MobileNetV2	femea sculptum (52).JPG	fold4	5	*Amblyomma*
3589	Female	MobileNetV2	femea sculptum (56).jpg	fold4	5	*Amblyomma*
3590	Female	MobileNetV2	femea sculptum (57).jpg	fold4	5	*Amblyomma*
3591	Female	MobileNetV2	femea triste (15).jpg	fold4	6	*Amblyomma*
3592	Female	MobileNetV2	femea triste (18).jpg	fold4	6	*Amblyomma*
3593	Female	MobileNetV2	femea triste (28).jpg	fold4	6	*Amblyomma*
3594	Female	MobileNetV2	femea triste (3).jpg	fold4	6	*Amblyomma*
3595	Female	MobileNetV2	femea triste (33).jpg	fold4	6	*Amblyomma*
3596	Female	MobileNetV2	femea triste (34).jpg	fold4	6	*Amblyomma*
3597	Female	MobileNetV2	femea triste (40).jpg	fold4	6	*Amblyomma*
3598	Female	MobileNetV2	femea triste (41).jpg	fold4	6	*Amblyomma*
3599	Female	MobileNetV2	femea triste (51).jpg	fold4	6	*Amblyomma*
3600	Female	MobileNetV2	femea triste (52).jpg	fold4	6	*Amblyomma*
3601	Female	MobileNetV2	femea aureolatum (13).jpg	fold5	1	*Amblyomma*
3602	Female	MobileNetV2	femea aureolatum (18).jpg	fold5	1	*Amblyomma*
3603	Female	MobileNetV2	femea aureolatum (23).jpg	fold5	1	*Amblyomma*
3604	Female	MobileNetV2	femea aureolatum (24).jpg	fold5	1	*Amblyomma*
3605	Female	MobileNetV2	femea aureolatum (25).jpg	fold5	1	*Amblyomma*
3606	Female	MobileNetV2	femea aureolatum (26).jpg	fold5	1	*Amblyomma*
3607	Female	MobileNetV2	femea aureolatum (33).jpg	fold5	1	*Amblyomma*
3608	Female	MobileNetV2	femea aureolatum (41).jpg	fold5	1	*Amblyomma*
3609	Female	MobileNetV2	femea aureolatum (45).jpg	fold5	1	*Amblyomma*
3610	Female	MobileNetV2	femea aureolatum (56).jpg	fold5	1	*Amblyomma*
3611	Female	MobileNetV2	femea aureolatum (57).jpg	fold5	1	*Amblyomma*
3612	Female	MobileNetV2	femea aureolatum (58).jpg	fold5	1	*Amblyomma*
3613	Female	MobileNetV2	femea cajennense (13).jpg	fold5	2	*Amblyomma*
3614	Female	MobileNetV2	femea cajennense (15).jpg	fold5	2	*Amblyomma*
3615	Female	MobileNetV2	femea cajennense (2).jpg	fold5	2	*Amblyomma*
3616	Female	MobileNetV2	femea cajennense (21).jpg	fold5	2	*Amblyomma*

3617	Female	MobileNetV2	femea cajennense (29).jpg	fold5	2	*Amblyomma*
3618	Female	MobileNetV2	femea cajennense (31).jpg	fold5	2	*Amblyomma*
3619	Female	MobileNetV2	femea cajennense (33).jpg	fold5	2	*Amblyomma*
3620	Female	MobileNetV2	femea cajennense (40).jpg	fold5	2	*Amblyomma*
3621	Female	MobileNetV2	femea cajennense (42).jpg	fold5	2	*Amblyomma*
3622	Female	MobileNetV2	femea cajennense (48).jpg	fold5	2	*Amblyomma*
3623	Female	MobileNetV2	femea cajennense (49).jpg	fold5	2	*Amblyomma*
3624	Female	MobileNetV2	femea cajennense (5).jpg	fold5	2	*Amblyomma*
3625	Female	MobileNetV2	femea cajennense (59).jpg	fold5	2	*Amblyomma*
3626	Female	MobileNetV2	femea cajennense (64).jpg	fold5	2	*Amblyomma*
3627	Female	MobileNetV2	femea cajennense (65).jpg	fold5	2	*Amblyomma*
3628	Female	MobileNetV2	femea cajennense (82).jpg	fold5	2	*Amblyomma*
3629	Female	MobileNetV2	femea dubitatum (1).jpg	fold5	3	*Amblyomma*
3630	Female	MobileNetV2	femea dubitatum (14).jpg	fold5	3	*Amblyomma*
3631	Female	MobileNetV2	femea dubitatum (20).jpg	fold5	3	*Amblyomma*
3632	Female	MobileNetV2	femea dubitatum (31).jpg	fold5	3	*Amblyomma*
3633	Female	MobileNetV2	femea dubitatum (4).jpg	fold5	3	*Amblyomma*
3634	Female	MobileNetV2	femea dubitatum (5).jpg	fold5	3	*Amblyomma*
3635	Female	MobileNetV2	femea dubitatum (9).jpg	fold5	3	*Amblyomma*
3636	Female	MobileNetV2	femea ovale (12).jpg	fold5	4	*Amblyomma*
3637	Female	MobileNetV2	femea ovale (13).jpg	fold5	4	*Amblyomma*
3638	Female	MobileNetV2	femea ovale (16).jpg	fold5	4	*Amblyomma*
3639	Female	MobileNetV2	femea ovale (21).jpg	fold5	4	*Amblyomma*
3640	Female	MobileNetV2	femea ovale (27).jpg	fold5	4	*Amblyomma*
3641	Female	MobileNetV2	femea ovale (41).jpg	fold5	4	*Amblyomma*
3642	Female	MobileNetV2	femea ovale (42).jpg	fold5	4	*Amblyomma*
3643	Female	MobileNetV2	femea ovale (43).jpg	fold5	4	*Amblyomma*
3644	Female	MobileNetV2	femea ovale (47).jpg	fold5	4	*Amblyomma*
3645	Female	MobileNetV2	femea ovale (60).jpg	fold5	4	*Amblyomma*
3646	Female	MobileNetV2	femea ovale (63).jpg	fold5	4	*Amblyomma*
3647	Female	MobileNetV2	femea ovale (68).jpg	fold5	4	*Amblyomma*
3648	Female	MobileNetV2	femea ovale (69).jpg	fold5	4	*Amblyomma*
3649	Female	MobileNetV2	femea ovale (72).jpg	fold5	4	*Amblyomma*
3650	Female	MobileNetV2	femea ovale (79).jpg	fold5	4	*Amblyomma*
3651	Female	MobileNetV2	femea ovale (8).jpg	fold5	4	*Amblyomma*
3652	Female	MobileNetV2	femea sculptum (12).JPG	fold5	5	*Amblyomma*
3653	Female	MobileNetV2	femea sculptum (20).JPG	fold5	5	*Amblyomma*
3654	Female	MobileNetV2	femea sculptum (31).JPG	fold5	5	*Amblyomma*
3655	Female	MobileNetV2	femea sculptum (32).JPG	fold5	5	*Amblyomma*
3656	Female	MobileNetV2	femea sculptum (33).JPG	fold5	5	*Amblyomma*
3657	Female	MobileNetV2	femea sculptum (35).JPG	fold5	5	*Amblyomma*
3658	Female	MobileNetV2	femea sculptum (4).jpg	fold5	5	*Amblyomma*
3659	Female	MobileNetV2	femea sculptum (48).JPG	fold5	5	*Amblyomma*
3660	Female	MobileNetV2	femea sculptum (50).JPG	fold5	5	*Amblyomma*
3661	Female	MobileNetV2	femea sculptum (51).JPG	fold5	5	*Amblyomma*
3662	Female	MobileNetV2	femea sculptum (55).JPG	fold5	5	*Amblyomma*
3663	Female	MobileNetV2	femea sculptum (63).jpg	fold5	5	*Amblyomma*
3664	Female	MobileNetV2	femea triste (16).jpg	fold5	6	*Amblyomma*
3665	Female	MobileNetV2	femea triste (17).jpg	fold5	6	*Amblyomma*
3666	Female	MobileNetV2	femea triste (20).jpg	fold5	6	*Amblyomma*
3667	Female	MobileNetV2	femea triste (21).jpg	fold5	6	*Amblyomma*
3668	Female	MobileNetV2	femea triste (25).jpg	fold5	6	*Amblyomma*
3669	Female	MobileNetV2	femea triste (29).jpg	fold5	6	*Amblyomma*
3670	Female	MobileNetV2	femea triste (39).jpg	fold5	6	*Amblyomma*
3671	Female	MobileNetV2	femea triste (48).jpg	fold5	6	*Amblyomma*
3672	Female	MobileNetV2	femea triste (49).jpg	fold5	6	*Amblyomma*
3673	Female	MobileNetV2	femea triste (6).jpg	fold5	6	*Amblyomma*
3674	Female	MobileNetV2	femea triste (8).jpg	fold5	6	*Amblyomma*
3675	Male	MobileNetV2	macho aureolatum (11).jpg	fold1	1	*Amblyomma*
3676	Male	MobileNetV2	macho aureolatum (12).jpg	fold1	1	*Amblyomma*
3677	Male	MobileNetV2	macho aureolatum (16).jpg	fold1	1	*Amblyomma*
3678	Male	MobileNetV2	macho aureolatum (17).jpg	fold1	1	*Amblyomma*
3679	Male	MobileNetV2	macho aureolatum (35).jpg	fold1	1	*Amblyomma*
3680	Male	MobileNetV2	macho aureolatum (36).jpg	fold1	1	*Amblyomma*
3681	Male	MobileNetV2	macho aureolatum (42).jpg	fold1	1	*Amblyomma*
3682	Male	MobileNetV2	macho aureolatum (44).jpg	fold1	1	*Amblyomma*
3683	Male	MobileNetV2	macho aureolatum (51).jpg	fold1	1	*Amblyomma*

3684	Male	MobileNetV2	macho aureolatum (58).jpg	fold1	1	*Amblyomma*
3685	Male	MobileNetV2	macho aureolatum (61).jpg	fold1	1	*Amblyomma*
3686	Male	MobileNetV2	macho aureolatum (9).jpg	fold1	1	*Amblyomma*
3687	Male	MobileNetV2	macho cajennense (1).jpg	fold1	2	*Amblyomma*
3688	Male	MobileNetV2	macho cajennense (12).jpg	fold1	2	*Amblyomma*
3689	Male	MobileNetV2	macho cajennense (17).jpg	fold1	2	*Amblyomma*
3690	Male	MobileNetV2	macho cajennense (23).jpg	fold1	2	*Amblyomma*
3691	Male	MobileNetV2	macho cajennense (34).jpg	fold1	2	*Amblyomma*
3692	Male	MobileNetV2	macho cajennense (36).jpg	fold1	2	*Amblyomma*
3693	Male	MobileNetV2	macho cajennense (37).jpg	fold1	2	*Amblyomma*
3694	Male	MobileNetV2	macho cajennense (39).jpg	fold1	2	*Amblyomma*
3695	Male	MobileNetV2	macho cajennense (52).jpg	fold1	2	*Amblyomma*
3696	Male	MobileNetV2	macho cajennense (64).jpg	fold1	2	*Amblyomma*
3697	Male	MobileNetV2	macho cajennense (66).jpg	fold1	2	*Amblyomma*
3698	Male	MobileNetV2	macho cajennense (71).jpg	fold1	2	*Amblyomma*
3699	Male	MobileNetV2	macho cajennense (73).jpg	fold1	2	*Amblyomma*
3700	Male	MobileNetV2	macho cajennense (76).jpg	fold1	2	*Amblyomma*
3701	Male	MobileNetV2	macho cajennense (82).jpg	fold1	2	*Amblyomma*
3702	Male	MobileNetV2	macho cajennense (83).jpg	fold1	2	*Amblyomma*
3703	Male	MobileNetV2	macho dubitatum (11).jpg	fold1	3	*Amblyomma*
3704	Male	MobileNetV2	macho dubitatum (16).jpg	fold1	3	*Amblyomma*
3705	Male	MobileNetV2	macho dubitatum (18).jpg	fold1	3	*Amblyomma*
3706	Male	MobileNetV2	macho dubitatum (2).jpg	fold1	3	*Amblyomma*
3707	Male	MobileNetV2	macho dubitatum (21).jpg	fold1	3	*Amblyomma*
3708	Male	MobileNetV2	macho dubitatum (22).jpg	fold1	3	*Amblyomma*
3709	Male	MobileNetV2	macho dubitatum (31).jpg	fold1	3	*Amblyomma*
3710	Male	MobileNetV2	macho dubitatum (38).jpg	fold1	3	*Amblyomma*
3711	Male	MobileNetV2	macho dubitatum (40).jpg	fold1	3	*Amblyomma*
3712	Male	MobileNetV2	macho dubitatum (47).jpg	fold1	3	*Amblyomma*
3713	Male	MobileNetV2	macho dubitatum (50).jpg	fold1	3	*Amblyomma*
3714	Male	MobileNetV2	macho dubitatum (57).jpg	fold1	3	*Amblyomma*
3715	Male	MobileNetV2	macho dubitatum (69).jpg	fold1	3	*Amblyomma*
3716	Male	MobileNetV2	macho dubitatum (74).jpg	fold1	3	*Amblyomma*
3717	Male	MobileNetV2	macho dubitatum (9).jpg	fold1	3	*Amblyomma*
3718	Male	MobileNetV2	macho ovale (12).jpg	fold1	4	*Amblyomma*
3719	Male	MobileNetV2	macho ovale (14).jpg	fold1	4	*Amblyomma*
3720	Male	MobileNetV2	macho ovale (15).jpg	fold1	4	*Amblyomma*
3721	Male	MobileNetV2	macho ovale (2).jpg	fold1	4	*Amblyomma*
3722	Male	MobileNetV2	macho ovale (20).jpg	fold1	4	*Amblyomma*
3723	Male	MobileNetV2	macho ovale (30).jpg	fold1	4	*Amblyomma*
3724	Male	MobileNetV2	macho ovale (41).jpg	fold1	4	*Amblyomma*
3725	Male	MobileNetV2	macho ovale (7).jpg	fold1	4	*Amblyomma*
3726	Male	MobileNetV2	macho sculptum (16).JPG	fold1	5	*Amblyomma*
3727	Male	MobileNetV2	macho sculptum (20).JPG	fold1	5	*Amblyomma*
3728	Male	MobileNetV2	macho sculptum (23).JPG	fold1	5	*Amblyomma*
3729	Male	MobileNetV2	macho sculptum (3).jpg	fold1	5	*Amblyomma*
3730	Male	MobileNetV2	macho sculptum (30).JPG	fold1	5	*Amblyomma*
3731	Male	MobileNetV2	macho sculptum (4).jpg	fold1	5	*Amblyomma*
3732	Male	MobileNetV2	macho sculptum (40).JPG	fold1	5	*Amblyomma*
3733	Male	MobileNetV2	macho sculptum (43).JPG	fold1	5	*Amblyomma*
3734	Male	MobileNetV2	macho sculptum (45).JPG	fold1	5	*Amblyomma*
3735	Male	MobileNetV2	macho sculptum (46).JPG	fold1	5	*Amblyomma*
3736	Male	MobileNetV2	macho sculptum (5).jpg	fold1	5	*Amblyomma*
3737	Male	MobileNetV2	macho sculptum (53).JPG	fold1	5	*Amblyomma*
3738	Male	MobileNetV2	macho sculptum (56).JPG	fold1	5	*Amblyomma*
3739	Male	MobileNetV2	macho sculptum (62).JPG	fold1	5	*Amblyomma*
3740	Male	MobileNetV2	macho sculptum (63).JPG	fold1	5	*Amblyomma*
3741	Male	MobileNetV2	macho sculptum (71).JPG	fold1	5	*Amblyomma*
3742	Male	MobileNetV2	macho sculptum (81).JPG	fold1	5	*Amblyomma*
3743	Male	MobileNetV2	macho sculptum (89).jpg	fold1	5	*Amblyomma*
3744	Male	MobileNetV2	macho triste (10).jpg	fold1	6	*Amblyomma*
3745	Male	MobileNetV2	macho triste (13).jpg	fold1	6	*Amblyomma*
3746	Male	MobileNetV2	macho triste (18).jpg	fold1	6	*Amblyomma*
3747	Male	MobileNetV2	macho triste (19).jpg	fold1	6	*Amblyomma*
3748	Male	MobileNetV2	macho triste (23).jpg	fold1	6	*Amblyomma*
3749	Male	MobileNetV2	macho triste (29).jpg	fold1	6	*Amblyomma*
3750	Male	MobileNetV2	macho triste (3).jpg	fold1	6	*Amblyomma*

3751	Male	MobileNetV2	macho triste (32).jpg	fold1	6	*Amblyomma*
3752	Male	MobileNetV2	macho triste (38).jpg	fold1	6	*Amblyomma*
3753	Male	MobileNetV2	macho triste (43).jpg	fold1	6	*Amblyomma*
3754	Male	MobileNetV2	macho triste (48).jpg	fold1	6	*Amblyomma*
3755	Male	MobileNetV2	macho triste (5).jpg	fold1	6	*Amblyomma*
3756	Male	MobileNetV2	macho triste (52).jpg	fold1	6	*Amblyomma*
3757	Male	MobileNetV2	macho triste (56).jpg	fold1	6	*Amblyomma*
3758	Male	MobileNetV2	macho triste (7).jpg	fold1	6	*Amblyomma*
3759	Male	MobileNetV2	macho triste (75).jpg	fold1	6	*Amblyomma*
3760	Male	MobileNetV2	macho triste (83).jpg	fold1	6	*Amblyomma*
3761	Male	MobileNetV2	macho triste (88).jpg	fold1	6	*Amblyomma*
3762	Male	MobileNetV2	macho triste (92).jpg	fold1	6	*Amblyomma*
3763	Male	MobileNetV2	macho triste (93).jpg	fold1	6	*Amblyomma*
3764	Male	MobileNetV2	macho aureolatum (19).jpg	fold2	1	*Amblyomma*
3765	Male	MobileNetV2	macho aureolatum (22).jpg	fold2	1	*Amblyomma*
3766	Male	MobileNetV2	macho aureolatum (26).jpg	fold2	1	*Amblyomma*
3767	Male	MobileNetV2	macho aureolatum (28).jpg	fold2	1	*Amblyomma*
3768	Male	MobileNetV2	macho aureolatum (32).jpg	fold2	1	*Amblyomma*
3769	Male	MobileNetV2	macho aureolatum (46).jpg	fold2	1	*Amblyomma*
3770	Male	MobileNetV2	macho aureolatum (49).jpg	fold2	1	*Amblyomma*
3771	Male	MobileNetV2	macho aureolatum (50).jpg	fold2	1	*Amblyomma*
3772	Male	MobileNetV2	macho aureolatum (52).jpg	fold2	1	*Amblyomma*
3773	Male	MobileNetV2	macho aureolatum (56).jpg	fold2	1	*Amblyomma*
3774	Male	MobileNetV2	macho aureolatum (59).jpg	fold2	1	*Amblyomma*
3775	Male	MobileNetV2	macho aureolatum (6).jpg	fold2	1	*Amblyomma*
3776	Male	MobileNetV2	macho aureolatum (62).jpg	fold2	1	*Amblyomma*
3777	Male	MobileNetV2	macho cajennense (10).jpg	fold2	2	*Amblyomma*
3778	Male	MobileNetV2	macho cajennense (2).jpg	fold2	2	*Amblyomma*
3779	Male	MobileNetV2	macho cajennense (26).jpg	fold2	2	*Amblyomma*
3780	Male	MobileNetV2	macho cajennense (27).jpg	fold2	2	*Amblyomma*
3781	Male	MobileNetV2	macho cajennense (3).jpg	fold2	2	*Amblyomma*
3782	Male	MobileNetV2	macho cajennense (30).jpg	fold2	2	*Amblyomma*
3783	Male	MobileNetV2	macho cajennense (43).jpg	fold2	2	*Amblyomma*
3784	Male	MobileNetV2	macho cajennense (53).jpg	fold2	2	*Amblyomma*
3785	Male	MobileNetV2	macho cajennense (59).jpg	fold2	2	*Amblyomma*
3786	Male	MobileNetV2	macho cajennense (60).jpg	fold2	2	*Amblyomma*
3787	Male	MobileNetV2	macho cajennense (61).jpg	fold2	2	*Amblyomma*
3788	Male	MobileNetV2	macho cajennense (68).jpg	fold2	2	*Amblyomma*
3789	Male	MobileNetV2	macho cajennense (70).jpg	fold2	2	*Amblyomma*
3790	Male	MobileNetV2	macho cajennense (72).jpg	fold2	2	*Amblyomma*
3791	Male	MobileNetV2	macho cajennense (8).jpg	fold2	2	*Amblyomma*
3792	Male	MobileNetV2	macho cajennense (81).jpg	fold2	2	*Amblyomma*
3793	Male	MobileNetV2	macho cajennense (9).jpg	fold2	2	*Amblyomma*
3794	Male	MobileNetV2	macho dubitatum (1).jpg	fold2	3	*Amblyomma*
3795	Male	MobileNetV2	macho dubitatum (13).jpg	fold2	3	*Amblyomma*
3796	Male	MobileNetV2	macho dubitatum (29).jpg	fold2	3	*Amblyomma*
3797	Male	MobileNetV2	macho dubitatum (30).jpg	fold2	3	*Amblyomma*
3798	Male	MobileNetV2	macho dubitatum (35).jpg	fold2	3	*Amblyomma*
3799	Male	MobileNetV2	macho dubitatum (43).jpg	fold2	3	*Amblyomma*
3800	Male	MobileNetV2	macho dubitatum (46).jpg	fold2	3	*Amblyomma*
3801	Male	MobileNetV2	macho dubitatum (48).jpg	fold2	3	*Amblyomma*
3802	Male	MobileNetV2	macho dubitatum (5).JPG	fold2	3	*Amblyomma*
3803	Male	MobileNetV2	macho dubitatum (53).jpg	fold2	3	*Amblyomma*
3804	Male	MobileNetV2	macho dubitatum (55).jpg	fold2	3	*Amblyomma*
3805	Male	MobileNetV2	macho dubitatum (56).jpg	fold2	3	*Amblyomma*
3806	Male	MobileNetV2	macho dubitatum (60).jpg	fold2	3	*Amblyomma*
3807	Male	MobileNetV2	macho dubitatum (72).jpg	fold2	3	*Amblyomma*
3808	Male	MobileNetV2	macho dubitatum (75).jpg	fold2	3	*Amblyomma*
3809	Male	MobileNetV2	macho ovale (18).jpg	fold2	4	*Amblyomma*
3810	Male	MobileNetV2	macho ovale (27).JPG	fold2	4	*Amblyomma*
3811	Male	MobileNetV2	macho ovale (33).jpg	fold2	4	*Amblyomma*
3812	Male	MobileNetV2	macho ovale (35).jpg	fold2	4	*Amblyomma*
3813	Male	MobileNetV2	macho ovale (36).jpg	fold2	4	*Amblyomma*
3814	Male	MobileNetV2	macho ovale (37).jpg	fold2	4	*Amblyomma*
3815	Male	MobileNetV2	macho ovale (39).jpg	fold2	4	*Amblyomma*
3816	Male	MobileNetV2	macho ovale (40).jpg	fold2	4	*Amblyomma*
3817	Male	MobileNetV2	macho ovale (6).jpg	fold2	4	*Amblyomma*

3818	Male	MobileNetV2	macho sculptum (12).JPG	fold2	5	*Amblyomma*
3819	Male	MobileNetV2	macho sculptum (21).JPG	fold2	5	*Amblyomma*
3820	Male	MobileNetV2	macho sculptum (24).JPG	fold2	5	*Amblyomma*
3821	Male	MobileNetV2	macho sculptum (26).JPG	fold2	5	*Amblyomma*
3822	Male	MobileNetV2	macho sculptum (27).JPG	fold2	5	*Amblyomma*
3823	Male	MobileNetV2	macho sculptum (36).JPG	fold2	5	*Amblyomma*
3824	Male	MobileNetV2	macho sculptum (42).JPG	fold2	5	*Amblyomma*
3825	Male	MobileNetV2	macho sculptum (55).JPG	fold2	5	*Amblyomma*
3826	Male	MobileNetV2	macho sculptum (59).JPG	fold2	5	*Amblyomma*
3827	Male	MobileNetV2	macho sculptum (67).JPG	fold2	5	*Amblyomma*
3828	Male	MobileNetV2	macho sculptum (7).jpg	fold2	5	*Amblyomma*
3829	Male	MobileNetV2	macho sculptum (70).JPG	fold2	5	*Amblyomma*
3830	Male	MobileNetV2	macho sculptum (73).JPG	fold2	5	*Amblyomma*
3831	Male	MobileNetV2	macho sculptum (78).JPG	fold2	5	*Amblyomma*
3832	Male	MobileNetV2	macho sculptum (82).jpg	fold2	5	*Amblyomma*
3833	Male	MobileNetV2	macho sculptum (86).jpg	fold2	5	*Amblyomma*
3834	Male	MobileNetV2	macho sculptum (91).jpg	fold2	5	*Amblyomma*
3835	Male	MobileNetV2	macho sculptum (92).jpg	fold2	5	*Amblyomma*
3836	Male	MobileNetV2	macho triste (14).jpg	fold2	6	*Amblyomma*
3837	Male	MobileNetV2	macho triste (15).jpg	fold2	6	*Amblyomma*
3838	Male	MobileNetV2	macho triste (17).jpg	fold2	6	*Amblyomma*
3839	Male	MobileNetV2	macho triste (20).jpg	fold2	6	*Amblyomma*
3840	Male	MobileNetV2	macho triste (21).jpg	fold2	6	*Amblyomma*
3841	Male	MobileNetV2	macho triste (24).jpg	fold2	6	*Amblyomma*
3842	Male	MobileNetV2	macho triste (49).jpg	fold2	6	*Amblyomma*
3843	Male	MobileNetV2	macho triste (50).jpg	fold2	6	*Amblyomma*
3844	Male	MobileNetV2	macho triste (61).jpg	fold2	6	*Amblyomma*
3845	Male	MobileNetV2	macho triste (62).jpg	fold2	6	*Amblyomma*
3846	Male	MobileNetV2	macho triste (63).jpg	fold2	6	*Amblyomma*
3847	Male	MobileNetV2	macho triste (64).jpg	fold2	6	*Amblyomma*
3848	Male	MobileNetV2	macho triste (67).jpg	fold2	6	*Amblyomma*
3849	Male	MobileNetV2	macho triste (68).jpg	fold2	6	*Amblyomma*
3850	Male	MobileNetV2	macho triste (73).jpg	fold2	6	*Amblyomma*
3851	Male	MobileNetV2	macho triste (77).jpg	fold2	6	*Amblyomma*
3852	Male	MobileNetV2	macho triste (78).jpg	fold2	6	*Amblyomma*
3853	Male	MobileNetV2	macho triste (82).jpg	fold2	6	*Amblyomma*
3854	Male	MobileNetV2	macho triste (9).jpg	fold2	6	*Amblyomma*
3855	Male	MobileNetV2	macho triste (90).jpg	fold2	6	*Amblyomma*
3856	Male	MobileNetV2	macho triste (91).jpg	fold2	6	*Amblyomma*
3857	Male	MobileNetV2	macho aureolatum (14).jpg	fold3	1	*Amblyomma*
3858	Male	MobileNetV2	macho aureolatum (20).jpg	fold3	1	*Amblyomma*
3859	Male	MobileNetV2	macho aureolatum (27).jpg	fold3	1	*Amblyomma*
3860	Male	MobileNetV2	macho aureolatum (29).jpg	fold3	1	*Amblyomma*
3861	Male	MobileNetV2	macho aureolatum (31).jpg	fold3	1	*Amblyomma*
3862	Male	MobileNetV2	macho aureolatum (38).jpg	fold3	1	*Amblyomma*
3863	Male	MobileNetV2	macho aureolatum (41).jpg	fold3	1	*Amblyomma*
3864	Male	MobileNetV2	macho aureolatum (47).jpg	fold3	1	*Amblyomma*
3865	Male	MobileNetV2	macho aureolatum (55).jpg	fold3	1	*Amblyomma*
3866	Male	MobileNetV2	macho aureolatum (57).jpg	fold3	1	*Amblyomma*
3867	Male	MobileNetV2	macho aureolatum (63).jpg	fold3	1	*Amblyomma*
3868	Male	MobileNetV2	macho aureolatum (7).jpg	fold3	1	*Amblyomma*
3869	Male	MobileNetV2	macho cajennense (11).jpg	fold3	2	*Amblyomma*
3870	Male	MobileNetV2	macho cajennense (14).jpg	fold3	2	*Amblyomma*
3871	Male	MobileNetV2	macho cajennense (15).jpg	fold3	2	*Amblyomma*
3872	Male	MobileNetV2	macho cajennense (24).jpg	fold3	2	*Amblyomma*
3873	Male	MobileNetV2	macho cajennense (29).jpg	fold3	2	*Amblyomma*
3874	Male	MobileNetV2	macho cajennense (33).jpg	fold3	2	*Amblyomma*
3875	Male	MobileNetV2	macho cajennense (38).jpg	fold3	2	*Amblyomma*
3876	Male	MobileNetV2	macho cajennense (42).jpg	fold3	2	*Amblyomma*
3877	Male	MobileNetV2	macho cajennense (46).jpg	fold3	2	*Amblyomma*
3878	Male	MobileNetV2	macho cajennense (5).jpg	fold3	2	*Amblyomma*
3879	Male	MobileNetV2	macho cajennense (54).jpg	fold3	2	*Amblyomma*
3880	Male	MobileNetV2	macho cajennense (56).jpg	fold3	2	*Amblyomma*
3881	Male	MobileNetV2	macho cajennense (6).jpg	fold3	2	*Amblyomma*
3882	Male	MobileNetV2	macho cajennense (63).jpg	fold3	2	*Amblyomma*
3883	Male	MobileNetV2	macho cajennense (65).jpg	fold3	2	*Amblyomma*
3884	Male	MobileNetV2	macho cajennense (67).jpg	fold3	2	*Amblyomma*

3885	Male	MobileNetV2	macho cajennense (7).jpg	fold3	2	*Amblyomma*
3886	Male	MobileNetV2	macho dubitatum (14).jpg	fold3	3	*Amblyomma*
3887	Male	MobileNetV2	macho dubitatum (20).jpg	fold3	3	*Amblyomma*
3888	Male	MobileNetV2	macho dubitatum (23).jpg	fold3	3	*Amblyomma*
3889	Male	MobileNetV2	macho dubitatum (25).jpg	fold3	3	*Amblyomma*
3890	Male	MobileNetV2	macho dubitatum (26).jpg	fold3	3	*Amblyomma*
3891	Male	MobileNetV2	macho dubitatum (36).jpg	fold3	3	*Amblyomma*
3892	Male	MobileNetV2	macho dubitatum (37).jpg	fold3	3	*Amblyomma*
3893	Male	MobileNetV2	macho dubitatum (41).jpg	fold3	3	*Amblyomma*
3894	Male	MobileNetV2	macho dubitatum (42).jpg	fold3	3	*Amblyomma*
3895	Male	MobileNetV2	macho dubitatum (52).jpg	fold3	3	*Amblyomma*
3896	Male	MobileNetV2	macho dubitatum (59).jpg	fold3	3	*Amblyomma*
3897	Male	MobileNetV2	macho dubitatum (65).jpg	fold3	3	*Amblyomma*
3898	Male	MobileNetV2	macho dubitatum (66).jpg	fold3	3	*Amblyomma*
3899	Male	MobileNetV2	macho dubitatum (67).jpg	fold3	3	*Amblyomma*
3900	Male	MobileNetV2	macho dubitatum (73).jpg	fold3	3	*Amblyomma*
3901	Male	MobileNetV2	macho ovale (10).jpg	fold3	4	*Amblyomma*
3902	Male	MobileNetV2	macho ovale (11).jpg	fold3	4	*Amblyomma*
3903	Male	MobileNetV2	macho ovale (19).jpg	fold3	4	*Amblyomma*
3904	Male	MobileNetV2	macho ovale (22).jpg	fold3	4	*Amblyomma*
3905	Male	MobileNetV2	macho ovale (28).JPG	fold3	4	*Amblyomma*
3906	Male	MobileNetV2	macho ovale (31).jpg	fold3	4	*Amblyomma*
3907	Male	MobileNetV2	macho ovale (5).jpg	fold3	4	*Amblyomma*
3908	Male	MobileNetV2	macho ovale (8).jpg	fold3	4	*Amblyomma*
3909	Male	MobileNetV2	macho ovale (9).jpg	fold3	4	*Amblyomma*
3910	Male	MobileNetV2	macho sculptum (1).jpg	fold3	5	*Amblyomma*
3911	Male	MobileNetV2	macho sculptum (13).JPG	fold3	5	*Amblyomma*
3912	Male	MobileNetV2	macho sculptum (14).JPG	fold3	5	*Amblyomma*
3913	Male	MobileNetV2	macho sculptum (18).JPG	fold3	5	*Amblyomma*
3914	Male	MobileNetV2	macho sculptum (2).jpg	fold3	5	*Amblyomma*
3915	Male	MobileNetV2	macho sculptum (22).JPG	fold3	5	*Amblyomma*
3916	Male	MobileNetV2	macho sculptum (31).JPG	fold3	5	*Amblyomma*
3917	Male	MobileNetV2	macho sculptum (34).JPG	fold3	5	*Amblyomma*
3918	Male	MobileNetV2	macho sculptum (35).JPG	fold3	5	*Amblyomma*
3919	Male	MobileNetV2	macho sculptum (37).JPG	fold3	5	*Amblyomma*
3920	Male	MobileNetV2	macho sculptum (39).JPG	fold3	5	*Amblyomma*
3921	Male	MobileNetV2	macho sculptum (44).JPG	fold3	5	*Amblyomma*
3922	Male	MobileNetV2	macho sculptum (49).JPG	fold3	5	*Amblyomma*
3923	Male	MobileNetV2	macho sculptum (57).JPG	fold3	5	*Amblyomma*
3924	Male	MobileNetV2	macho sculptum (83).jpg	fold3	5	*Amblyomma*
3925	Male	MobileNetV2	macho sculptum (84).jpg	fold3	5	*Amblyomma*
3926	Male	MobileNetV2	macho sculptum (85).jpg	fold3	5	*Amblyomma*
3927	Male	MobileNetV2	macho sculptum (87).jpg	fold3	5	*Amblyomma*
3928	Male	MobileNetV2	macho sculptum (90).jpg	fold3	5	*Amblyomma*
3929	Male	MobileNetV2	macho triste (1).jpg	fold3	6	*Amblyomma*
3930	Male	MobileNetV2	macho triste (100).jpg	fold3	6	*Amblyomma*
3931	Male	MobileNetV2	macho triste (101).jpg	fold3	6	*Amblyomma*
3932	Male	MobileNetV2	macho triste (16).jpg	fold3	6	*Amblyomma*
3933	Male	MobileNetV2	macho triste (26).jpg	fold3	6	*Amblyomma*
3934	Male	MobileNetV2	macho triste (34).jpg	fold3	6	*Amblyomma*
3935	Male	MobileNetV2	macho triste (35).jpg	fold3	6	*Amblyomma*
3936	Male	MobileNetV2	macho triste (40).jpg	fold3	6	*Amblyomma*
3937	Male	MobileNetV2	macho triste (44).jpg	fold3	6	*Amblyomma*
3938	Male	MobileNetV2	macho triste (45).jpg	fold3	6	*Amblyomma*
3939	Male	MobileNetV2	macho triste (51).jpg	fold3	6	*Amblyomma*
3940	Male	MobileNetV2	macho triste (59).jpg	fold3	6	*Amblyomma*
3941	Male	MobileNetV2	macho triste (6).jpg	fold3	6	*Amblyomma*
3942	Male	MobileNetV2	macho triste (66).jpg	fold3	6	*Amblyomma*
3943	Male	MobileNetV2	macho triste (71).jpg	fold3	6	*Amblyomma*
3944	Male	MobileNetV2	macho triste (76).jpg	fold3	6	*Amblyomma*
3945	Male	MobileNetV2	macho triste (80).jpg	fold3	6	*Amblyomma*
3946	Male	MobileNetV2	macho triste (86).jpg	fold3	6	*Amblyomma*
3947	Male	MobileNetV2	macho triste (87).jpg	fold3	6	*Amblyomma*
3948	Male	MobileNetV2	macho triste (94).jpg	fold3	6	*Amblyomma*
3949	Male	MobileNetV2	macho aureolatum (1).jpg	fold4	1	*Amblyomma*
3950	Male	MobileNetV2	macho aureolatum (10).jpg	fold4	1	*Amblyomma*
3951	Male	MobileNetV2	macho aureolatum (15).jpg	fold4	1	*Amblyomma*

3952	Male	MobileNetV2	macho aureolatum (2).jpg	fold4	1	*Amblyomma*
3953	Male	MobileNetV2	macho aureolatum (23).jpg	fold4	1	*Amblyomma*
3954	Male	MobileNetV2	macho aureolatum (24).jpg	fold4	1	*Amblyomma*
3955	Male	MobileNetV2	macho aureolatum (25).jpg	fold4	1	*Amblyomma*
3956	Male	MobileNetV2	macho aureolatum (33).jpg	fold4	1	*Amblyomma*
3957	Male	MobileNetV2	macho aureolatum (37).jpg	fold4	1	*Amblyomma*
3958	Male	MobileNetV2	macho aureolatum (39).jpg	fold4	1	*Amblyomma*
3959	Male	MobileNetV2	macho aureolatum (4).jpg	fold4	1	*Amblyomma*
3960	Male	MobileNetV2	macho aureolatum (48).jpg	fold4	1	*Amblyomma*
3961	Male	MobileNetV2	macho aureolatum (60).jpg	fold4	1	*Amblyomma*
3962	Male	MobileNetV2	macho cajennense (13).jpg	fold4	2	*Amblyomma*
3963	Male	MobileNetV2	macho cajennense (16).jpg	fold4	2	*Amblyomma*
3964	Male	MobileNetV2	macho cajennense (19).jpg	fold4	2	*Amblyomma*
3965	Male	MobileNetV2	macho cajennense (20).jpg	fold4	2	*Amblyomma*
3966	Male	MobileNetV2	macho cajennense (21).jpg	fold4	2	*Amblyomma*
3967	Male	MobileNetV2	macho cajennense (28).jpg	fold4	2	*Amblyomma*
3968	Male	MobileNetV2	macho cajennense (31).jpg	fold4	2	*Amblyomma*
3969	Male	MobileNetV2	macho cajennense (32).jpg	fold4	2	*Amblyomma*
3970	Male	MobileNetV2	macho cajennense (40).jpg	fold4	2	*Amblyomma*
3971	Male	MobileNetV2	macho cajennense (41).jpg	fold4	2	*Amblyomma*
3972	Male	MobileNetV2	macho cajennense (45).jpg	fold4	2	*Amblyomma*
3973	Male	MobileNetV2	macho cajennense (47).jpg	fold4	2	*Amblyomma*
3974	Male	MobileNetV2	macho cajennense (55).jpg	fold4	2	*Amblyomma*
3975	Male	MobileNetV2	macho cajennense (58).jpg	fold4	2	*Amblyomma*
3976	Male	MobileNetV2	macho cajennense (79).jpg	fold4	2	*Amblyomma*
3977	Male	MobileNetV2	macho cajennense (80).jpg	fold4	2	*Amblyomma*
3978	Male	MobileNetV2	macho dubitatum (10).jpg	fold4	3	*Amblyomma*
3979	Male	MobileNetV2	macho dubitatum (12).jpg	fold4	3	*Amblyomma*
3980	Male	MobileNetV2	macho dubitatum (15).jpg	fold4	3	*Amblyomma*
3981	Male	MobileNetV2	macho dubitatum (28).jpg	fold4	3	*Amblyomma*
3982	Male	MobileNetV2	macho dubitatum (3).jpg	fold4	3	*Amblyomma*
3983	Male	MobileNetV2	macho dubitatum (32).jpg	fold4	3	*Amblyomma*
3984	Male	MobileNetV2	macho dubitatum (33).jpg	fold4	3	*Amblyomma*
3985	Male	MobileNetV2	macho dubitatum (39).jpg	fold4	3	*Amblyomma*
3986	Male	MobileNetV2	macho dubitatum (4).jpg	fold4	3	*Amblyomma*
3987	Male	MobileNetV2	macho dubitatum (54).jpg	fold4	3	*Amblyomma*
3988	Male	MobileNetV2	macho dubitatum (6).JPG	fold4	3	*Amblyomma*
3989	Male	MobileNetV2	macho dubitatum (61).jpg	fold4	3	*Amblyomma*
3990	Male	MobileNetV2	macho dubitatum (63).jpg	fold4	3	*Amblyomma*
3991	Male	MobileNetV2	macho dubitatum (7).JPG	fold4	3	*Amblyomma*
3992	Male	MobileNetV2	macho dubitatum (71).jpg	fold4	3	*Amblyomma*
3993	Male	MobileNetV2	macho ovale (1).jpg	fold4	4	*Amblyomma*
3994	Male	MobileNetV2	macho ovale (23).jpg	fold4	4	*Amblyomma*
3995	Male	MobileNetV2	macho ovale (26).jpg	fold4	4	*Amblyomma*
3996	Male	MobileNetV2	macho ovale (29).jpg	fold4	4	*Amblyomma*
3997	Male	MobileNetV2	macho ovale (34).jpg	fold4	4	*Amblyomma*
3998	Male	MobileNetV2	macho ovale (38).jpg	fold4	4	*Amblyomma*
3999	Male	MobileNetV2	macho ovale (4).jpg	fold4	4	*Amblyomma*
4000	Male	MobileNetV2	macho ovale (42).jpg	fold4	4	*Amblyomma*
4001	Male	MobileNetV2	macho sculptum (10).JPG	fold4	5	*Amblyomma*
4002	Male	MobileNetV2	macho sculptum (11).JPG	fold4	5	*Amblyomma*
4003	Male	MobileNetV2	macho sculptum (15).JPG	fold4	5	*Amblyomma*
4004	Male	MobileNetV2	macho sculptum (17).JPG	fold4	5	*Amblyomma*
4005	Male	MobileNetV2	macho sculptum (33).JPG	fold4	5	*Amblyomma*
4006	Male	MobileNetV2	macho sculptum (38).JPG	fold4	5	*Amblyomma*
4007	Male	MobileNetV2	macho sculptum (47).JPG	fold4	5	*Amblyomma*
4008	Male	MobileNetV2	macho sculptum (48).JPG	fold4	5	*Amblyomma*
4009	Male	MobileNetV2	macho sculptum (51).JPG	fold4	5	*Amblyomma*
4010	Male	MobileNetV2	macho sculptum (52).JPG	fold4	5	*Amblyomma*
4011	Male	MobileNetV2	macho sculptum (61).JPG	fold4	5	*Amblyomma*
4012	Male	MobileNetV2	macho sculptum (66).JPG	fold4	5	*Amblyomma*
4013	Male	MobileNetV2	macho sculptum (68).JPG	fold4	5	*Amblyomma*
4014	Male	MobileNetV2	macho sculptum (69).JPG	fold4	5	*Amblyomma*
4015	Male	MobileNetV2	macho sculptum (72).JPG	fold4	5	*Amblyomma*
4016	Male	MobileNetV2	macho sculptum (76).JPG	fold4	5	*Amblyomma*
4017	Male	MobileNetV2	macho sculptum (77).JPG	fold4	5	*Amblyomma*
4018	Male	MobileNetV2	macho sculptum (80).JPG	fold4	5	*Amblyomma*

4019	Male	MobileNetV2	macho sculptum (9).JPG	fold4	5	*Amblyomma*
4020	Male	MobileNetV2	macho triste (102).jpg	fold4	6	*Amblyomma*
4021	Male	MobileNetV2	macho triste (2).jpg	fold4	6	*Amblyomma*
4022	Male	MobileNetV2	macho triste (31).jpg	fold4	6	*Amblyomma*
4023	Male	MobileNetV2	macho triste (37).jpg	fold4	6	*Amblyomma*
4024	Male	MobileNetV2	macho triste (39).jpg	fold4	6	*Amblyomma*
4025	Male	MobileNetV2	macho triste (41).jpg	fold4	6	*Amblyomma*
4026	Male	MobileNetV2	macho triste (42).jpg	fold4	6	*Amblyomma*
4027	Male	MobileNetV2	macho triste (46).jpg	fold4	6	*Amblyomma*
4028	Male	MobileNetV2	macho triste (47).jpg	fold4	6	*Amblyomma*
4029	Male	MobileNetV2	macho triste (54).jpg	fold4	6	*Amblyomma*
4030	Male	MobileNetV2	macho triste (58).jpg	fold4	6	*Amblyomma*
4031	Male	MobileNetV2	macho triste (65).jpg	fold4	6	*Amblyomma*
4032	Male	MobileNetV2	macho triste (69).jpg	fold4	6	*Amblyomma*
4033	Male	MobileNetV2	macho triste (70).jpg	fold4	6	*Amblyomma*
4034	Male	MobileNetV2	macho triste (72).jpg	fold4	6	*Amblyomma*
4035	Male	MobileNetV2	macho triste (79).jpg	fold4	6	*Amblyomma*
4036	Male	MobileNetV2	macho triste (81).jpg	fold4	6	*Amblyomma*
4037	Male	MobileNetV2	macho triste (84).jpg	fold4	6	*Amblyomma*
4038	Male	MobileNetV2	macho triste (85).jpg	fold4	6	*Amblyomma*
4039	Male	MobileNetV2	macho triste (98).jpg	fold4	6	*Amblyomma*
4040	Male	MobileNetV2	macho triste (99).jpg	fold4	6	*Amblyomma*
4041	Male	MobileNetV2	macho aureolatum (13).jpg	fold5	1	*Amblyomma*
4042	Male	MobileNetV2	macho aureolatum (18).jpg	fold5	1	*Amblyomma*
4043	Male	MobileNetV2	macho aureolatum (21).jpg	fold5	1	*Amblyomma*
4044	Male	MobileNetV2	macho aureolatum (3).jpg	fold5	1	*Amblyomma*
4045	Male	MobileNetV2	macho aureolatum (30).jpg	fold5	1	*Amblyomma*
4046	Male	MobileNetV2	macho aureolatum (34).jpg	fold5	1	*Amblyomma*
4047	Male	MobileNetV2	macho aureolatum (40).jpg	fold5	1	*Amblyomma*
4048	Male	MobileNetV2	macho aureolatum (43).jpg	fold5	1	*Amblyomma*
4049	Male	MobileNetV2	macho aureolatum (45).jpg	fold5	1	*Amblyomma*
4050	Male	MobileNetV2	macho aureolatum (5).jpg	fold5	1	*Amblyomma*
4051	Male	MobileNetV2	macho aureolatum (53).jpg	fold5	1	*Amblyomma*
4052	Male	MobileNetV2	macho aureolatum (54).jpg	fold5	1	*Amblyomma*
4053	Male	MobileNetV2	macho aureolatum (8).jpg	fold5	1	*Amblyomma*
4054	Male	MobileNetV2	macho cajennense (18).jpg	fold5	2	*Amblyomma*
4055	Male	MobileNetV2	macho cajennense (22).jpg	fold5	2	*Amblyomma*
4056	Male	MobileNetV2	macho cajennense (25).jpg	fold5	2	*Amblyomma*
4057	Male	MobileNetV2	macho cajennense (35).jpg	fold5	2	*Amblyomma*
4058	Male	MobileNetV2	macho cajennense (4).jpg	fold5	2	*Amblyomma*
4059	Male	MobileNetV2	macho cajennense (44).jpg	fold5	2	*Amblyomma*
4060	Male	MobileNetV2	macho cajennense (48).jpg	fold5	2	*Amblyomma*
4061	Male	MobileNetV2	macho cajennense (49).jpg	fold5	2	*Amblyomma*
4062	Male	MobileNetV2	macho cajennense (50).jpg	fold5	2	*Amblyomma*
4063	Male	MobileNetV2	macho cajennense (51).jpg	fold5	2	*Amblyomma*
4064	Male	MobileNetV2	macho cajennense (57).jpg	fold5	2	*Amblyomma*
4065	Male	MobileNetV2	macho cajennense (62).jpg	fold5	2	*Amblyomma*
4066	Male	MobileNetV2	macho cajennense (69).jpg	fold5	2	*Amblyomma*
4067	Male	MobileNetV2	macho cajennense (74).jpg	fold5	2	*Amblyomma*
4068	Male	MobileNetV2	macho cajennense (75).jpg	fold5	2	*Amblyomma*
4069	Male	MobileNetV2	macho cajennense (77).jpg	fold5	2	*Amblyomma*
4070	Male	MobileNetV2	macho cajennense (78).jpg	fold5	2	*Amblyomma*
4071	Male	MobileNetV2	macho dubitatum (17).jpg	fold5	3	*Amblyomma*
4072	Male	MobileNetV2	macho dubitatum (19).jpg	fold5	3	*Amblyomma*
4073	Male	MobileNetV2	macho dubitatum (24).jpg	fold5	3	*Amblyomma*
4074	Male	MobileNetV2	macho dubitatum (27).jpg	fold5	3	*Amblyomma*
4075	Male	MobileNetV2	macho dubitatum (34).jpg	fold5	3	*Amblyomma*
4076	Male	MobileNetV2	macho dubitatum (44).jpg	fold5	3	*Amblyomma*
4077	Male	MobileNetV2	macho dubitatum (45).jpg	fold5	3	*Amblyomma*
4078	Male	MobileNetV2	macho dubitatum (49).jpg	fold5	3	*Amblyomma*
4079	Male	MobileNetV2	macho dubitatum (51).jpg	fold5	3	*Amblyomma*
4080	Male	MobileNetV2	macho dubitatum (58).jpg	fold5	3	*Amblyomma*
4081	Male	MobileNetV2	macho dubitatum (62).jpg	fold5	3	*Amblyomma*
4082	Male	MobileNetV2	macho dubitatum (64).jpg	fold5	3	*Amblyomma*
4083	Male	MobileNetV2	macho dubitatum (68).jpg	fold5	3	*Amblyomma*
4084	Male	MobileNetV2	macho dubitatum (70).jpg	fold5	3	*Amblyomma*
4085	Male	MobileNetV2	macho dubitatum (8).JPG	fold5	3	*Amblyomma*

4086	Male	MobileNetV2	macho ovale (13).jpg	fold5	4	*Amblyomma*
4087	Male	MobileNetV2	macho ovale (16).jpg	fold5	4	*Amblyomma*
4088	Male	MobileNetV2	macho ovale (17).jpg	fold5	4	*Amblyomma*
4089	Male	MobileNetV2	macho ovale (21).jpg	fold5	4	*Amblyomma*
4090	Male	MobileNetV2	macho ovale (24).jpg	fold5	4	*Amblyomma*
4091	Male	MobileNetV2	macho ovale (25).jpg	fold5	4	*Amblyomma*
4092	Male	MobileNetV2	macho ovale (3).jpg	fold5	4	*Amblyomma*
4093	Male	MobileNetV2	macho ovale (32).jpg	fold5	4	*Amblyomma*
4094	Male	MobileNetV2	macho sculptum (19).JPG	fold5	5	*Amblyomma*
4095	Male	MobileNetV2	macho sculptum (25).JPG	fold5	5	*Amblyomma*
4096	Male	MobileNetV2	macho sculptum (28).JPG	fold5	5	*Amblyomma*
4097	Male	MobileNetV2	macho sculptum (29).JPG	fold5	5	*Amblyomma*
4098	Male	MobileNetV2	macho sculptum (32).JPG	fold5	5	*Amblyomma*
4099	Male	MobileNetV2	macho sculptum (41).JPG	fold5	5	*Amblyomma*
4100	Male	MobileNetV2	macho sculptum (50).JPG	fold5	5	*Amblyomma*
4101	Male	MobileNetV2	macho sculptum (54).JPG	fold5	5	*Amblyomma*
4102	Male	MobileNetV2	macho sculptum (58).JPG	fold5	5	*Amblyomma*
4103	Male	MobileNetV2	macho sculptum (6).jpg	fold5	5	*Amblyomma*
4104	Male	MobileNetV2	macho sculptum (60).JPG	fold5	5	*Amblyomma*
4105	Male	MobileNetV2	macho sculptum (64).JPG	fold5	5	*Amblyomma*
4106	Male	MobileNetV2	macho sculptum (65).JPG	fold5	5	*Amblyomma*
4107	Male	MobileNetV2	macho sculptum (74).JPG	fold5	5	*Amblyomma*
4108	Male	MobileNetV2	macho sculptum (75).JPG	fold5	5	*Amblyomma*
4109	Male	MobileNetV2	macho sculptum (79).JPG	fold5	5	*Amblyomma*
4110	Male	MobileNetV2	macho sculptum (8).JPG	fold5	5	*Amblyomma*
4111	Male	MobileNetV2	macho sculptum (88).jpg	fold5	5	*Amblyomma*
4112	Male	MobileNetV2	macho triste (103).jpg	fold5	6	*Amblyomma*
4113	Male	MobileNetV2	macho triste (11).jpg	fold5	6	*Amblyomma*
4114	Male	MobileNetV2	macho triste (12).jpg	fold5	6	*Amblyomma*
4115	Male	MobileNetV2	macho triste (22).jpg	fold5	6	*Amblyomma*
4116	Male	MobileNetV2	macho triste (25).jpg	fold5	6	*Amblyomma*
4117	Male	MobileNetV2	macho triste (27).jpg	fold5	6	*Amblyomma*
4118	Male	MobileNetV2	macho triste (28).jpg	fold5	6	*Amblyomma*
4119	Male	MobileNetV2	macho triste (30).jpg	fold5	6	*Amblyomma*
4120	Male	MobileNetV2	macho triste (33).jpg	fold5	6	*Amblyomma*
4121	Male	MobileNetV2	macho triste (36).jpg	fold5	6	*Amblyomma*
4122	Male	MobileNetV2	macho triste (4).jpg	fold5	6	*Amblyomma*
4123	Male	MobileNetV2	macho triste (53).jpg	fold5	6	*Amblyomma*
4124	Male	MobileNetV2	macho triste (55).jpg	fold5	6	*Amblyomma*
4125	Male	MobileNetV2	macho triste (57).jpg	fold5	6	*Amblyomma*
4126	Male	MobileNetV2	macho triste (60).jpg	fold5	6	*Amblyomma*
4127	Male	MobileNetV2	macho triste (74).jpg	fold5	6	*Amblyomma*
4128	Male	MobileNetV2	macho triste (8).jpg	fold5	6	*Amblyomma*
4129	Male	MobileNetV2	macho triste (89).jpg	fold5	6	*Amblyomma*
4130	Male	MobileNetV2	macho triste (95).jpg	fold5	6	*Amblyomma*
4131	Male	MobileNetV2	macho triste (96).jpg	fold5	6	*Amblyomma*
4132	Male	MobileNetV2	macho triste (97).jpg	fold5	6	*Amblyomma*
4133	Dorsal	MobileNetV2	dorsal aureolatum (11).jpg	fold1	1	*Amblyomma*
4134	Dorsal	MobileNetV2	dorsal aureolatum (17).jpg	fold1	1	*Amblyomma*
4135	Dorsal	MobileNetV2	dorsal aureolatum (23).jpg	fold1	1	*Amblyomma*
4136	Dorsal	MobileNetV2	dorsal aureolatum (27).jpg	fold1	1	*Amblyomma*
4137	Dorsal	MobileNetV2	dorsal aureolatum (30).jpg	fold1	1	*Amblyomma*
4138	Dorsal	MobileNetV2	dorsal aureolatum (46).jpg	fold1	1	*Amblyomma*
4139	Dorsal	MobileNetV2	dorsal aureolatum (48).jpg	fold1	1	*Amblyomma*
4140	Dorsal	MobileNetV2	dorsal aureolatum (52).jpg	fold1	1	*Amblyomma*
4141	Dorsal	MobileNetV2	dorsal aureolatum (55).jpg	fold1	1	*Amblyomma*
4142	Dorsal	MobileNetV2	dorsal aureolatum (58).jpg	fold1	1	*Amblyomma*
4143	Dorsal	MobileNetV2	dorsal aureolatum (61).jpg	fold1	1	*Amblyomma*
4144	Dorsal	MobileNetV2	dorsal aureolatum (8).jpg	fold1	1	*Amblyomma*
4145	Dorsal	MobileNetV2	dorsal cajennense (25).jpg	fold1	2	*Amblyomma*
4146	Dorsal	MobileNetV2	dorsal cajennense (26).jpg	fold1	2	*Amblyomma*
4147	Dorsal	MobileNetV2	dorsal cajennense (3).jpg	fold1	2	*Amblyomma*
4148	Dorsal	MobileNetV2	dorsal cajennense (32).jpg	fold1	2	*Amblyomma*
4149	Dorsal	MobileNetV2	dorsal cajennense (44).jpg	fold1	2	*Amblyomma*
4150	Dorsal	MobileNetV2	dorsal cajennense (47).jpg	fold1	2	*Amblyomma*
4151	Dorsal	MobileNetV2	dorsal cajennense (50).jpg	fold1	2	*Amblyomma*
4152	Dorsal	MobileNetV2	dorsal cajennense (52).jpg	fold1	2	*Amblyomma*

4153	Dorsal	MobileNetV2	dorsal cajennense (54).jpg	fold1	2	*Amblyomma*
4154	Dorsal	MobileNetV2	dorsal cajennense (58).jpg	fold1	2	*Amblyomma*
4155	Dorsal	MobileNetV2	dorsal cajennense (65).jpg	fold1	2	*Amblyomma*
4156	Dorsal	MobileNetV2	dorsal cajennense (66).jpg	fold1	2	*Amblyomma*
4157	Dorsal	MobileNetV2	dorsal cajennense (7).jpg	fold1	2	*Amblyomma*
4158	Dorsal	MobileNetV2	dorsal cajennense (75).jpg	fold1	2	*Amblyomma*
4159	Dorsal	MobileNetV2	dorsal cajennense (77).jpg	fold1	2	*Amblyomma*
4160	Dorsal	MobileNetV2	dorsal cajennense (80).jpg	fold1	2	*Amblyomma*
4161	Dorsal	MobileNetV2	dorsal dubitatum (10).jpg	fold1	3	*Amblyomma*
4162	Dorsal	MobileNetV2	dorsal dubitatum (18).jpg	fold1	3	*Amblyomma*
4163	Dorsal	MobileNetV2	dorsal dubitatum (19).jpg	fold1	3	*Amblyomma*
4164	Dorsal	MobileNetV2	dorsal dubitatum (28).jpg	fold1	3	*Amblyomma*
4165	Dorsal	MobileNetV2	dorsal dubitatum (32).jpg	fold1	3	*Amblyomma*
4166	Dorsal	MobileNetV2	dorsal dubitatum (40).jpg	fold1	3	*Amblyomma*
4167	Dorsal	MobileNetV2	dorsal dubitatum (46).jpg	fold1	3	*Amblyomma*
4168	Dorsal	MobileNetV2	dorsal dubitatum (49).jpg	fold1	3	*Amblyomma*
4169	Dorsal	MobileNetV2	dorsal dubitatum (55).jpg	fold1	3	*Amblyomma*
4170	Dorsal	MobileNetV2	dorsal dubitatum (6).jpg	fold1	3	*Amblyomma*
4171	Dorsal	MobileNetV2	dorsal dubitatum (9).JPG	fold1	3	*Amblyomma*
4172	Dorsal	MobileNetV2	dorsal ovale (10).jpg	fold1	4	*Amblyomma*
4173	Dorsal	MobileNetV2	dorsal ovale (14).jpg	fold1	4	*Amblyomma*
4174	Dorsal	MobileNetV2	dorsal ovale (20).jpg	fold1	4	*Amblyomma*
4175	Dorsal	MobileNetV2	dorsal ovale (21).jpg	fold1	4	*Amblyomma*
4176	Dorsal	MobileNetV2	dorsal ovale (26).jpg	fold1	4	*Amblyomma*
4177	Dorsal	MobileNetV2	dorsal ovale (29).jpg	fold1	4	*Amblyomma*
4178	Dorsal	MobileNetV2	dorsal ovale (44).jpg	fold1	4	*Amblyomma*
4179	Dorsal	MobileNetV2	dorsal ovale (48).jpg	fold1	4	*Amblyomma*
4180	Dorsal	MobileNetV2	dorsal ovale (51).jpg	fold1	4	*Amblyomma*
4181	Dorsal	MobileNetV2	dorsal ovale (53).jpg	fold1	4	*Amblyomma*
4182	Dorsal	MobileNetV2	dorsal ovale (61).jpg	fold1	4	*Amblyomma*
4183	Dorsal	MobileNetV2	dorsal ovale (7).jpg	fold1	4	*Amblyomma*
4184	Dorsal	MobileNetV2	dorsal sculptum (14).JPG	fold1	5	*Amblyomma*
4185	Dorsal	MobileNetV2	dorsal sculptum (20).JPG	fold1	5	*Amblyomma*
4186	Dorsal	MobileNetV2	dorsal sculptum (22).JPG	fold1	5	*Amblyomma*
4187	Dorsal	MobileNetV2	dorsal sculptum (23).JPG	fold1	5	*Amblyomma*
4188	Dorsal	MobileNetV2	dorsal sculptum (25).JPG	fold1	5	*Amblyomma*
4189	Dorsal	MobileNetV2	dorsal sculptum (29).jpg	fold1	5	*Amblyomma*
4190	Dorsal	MobileNetV2	dorsal sculptum (37).JPG	fold1	5	*Amblyomma*
4191	Dorsal	MobileNetV2	dorsal sculptum (38).JPG	fold1	5	*Amblyomma*
4192	Dorsal	MobileNetV2	dorsal sculptum (42).JPG	fold1	5	*Amblyomma*
4193	Dorsal	MobileNetV2	dorsal sculptum (5).JPG	fold1	5	*Amblyomma*
4194	Dorsal	MobileNetV2	dorsal sculptum (51).JPG	fold1	5	*Amblyomma*
4195	Dorsal	MobileNetV2	dorsal sculptum (52).JPG	fold1	5	*Amblyomma*
4196	Dorsal	MobileNetV2	dorsal sculptum (59).JPG	fold1	5	*Amblyomma*
4197	Dorsal	MobileNetV2	dorsal sculptum (72).JPG	fold1	5	*Amblyomma*
4198	Dorsal	MobileNetV2	dorsal sculptum (75).JPG	fold1	5	*Amblyomma*
4199	Dorsal	MobileNetV2	dorsal sculptum (9).JPG	fold1	5	*Amblyomma*
4200	Dorsal	MobileNetV2	dorsal triste (19).jpg	fold1	6	*Amblyomma*
4201	Dorsal	MobileNetV2	dorsal triste (2).jpg	fold1	6	*Amblyomma*
4202	Dorsal	MobileNetV2	dorsal triste (20).jpg	fold1	6	*Amblyomma*
4203	Dorsal	MobileNetV2	dorsal triste (24).jpg	fold1	6	*Amblyomma*
4204	Dorsal	MobileNetV2	dorsal triste (25).jpg	fold1	6	*Amblyomma*
4205	Dorsal	MobileNetV2	dorsal triste (26).jpg	fold1	6	*Amblyomma*
4206	Dorsal	MobileNetV2	dorsal triste (45).jpg	fold1	6	*Amblyomma*
4207	Dorsal	MobileNetV2	dorsal triste (46).jpg	fold1	6	*Amblyomma*
4208	Dorsal	MobileNetV2	dorsal triste (47).jpg	fold1	6	*Amblyomma*
4209	Dorsal	MobileNetV2	dorsal triste (51).jpg	fold1	6	*Amblyomma*
4210	Dorsal	MobileNetV2	dorsal triste (6).jpg	fold1	6	*Amblyomma*
4211	Dorsal	MobileNetV2	dorsal triste (61).jpg	fold1	6	*Amblyomma*
4212	Dorsal	MobileNetV2	dorsal triste (63).jpg	fold1	6	*Amblyomma*
4213	Dorsal	MobileNetV2	dorsal triste (72).jpg	fold1	6	*Amblyomma*
4214	Dorsal	MobileNetV2	dorsal triste (74).jpg	fold1	6	*Amblyomma*
4215	Dorsal	MobileNetV2	dorsal triste (79).jpg	fold1	6	*Amblyomma*
4216	Dorsal	MobileNetV2	dorsal aureolatum (18).jpg	fold2	1	*Amblyomma*
4217	Dorsal	MobileNetV2	dorsal aureolatum (21).jpg	fold2	1	*Amblyomma*
4218	Dorsal	MobileNetV2	dorsal aureolatum (28).jpg	fold2	1	*Amblyomma*
4219	Dorsal	MobileNetV2	dorsal aureolatum (29).jpg	fold2	1	*Amblyomma*

4220	Dorsal	MobileNetV2	dorsal aureolatum (32).jpg	fold2	1	*Amblyomma*
4221	Dorsal	MobileNetV2	dorsal aureolatum (38).jpg	fold2	1	*Amblyomma*
4222	Dorsal	MobileNetV2	dorsal aureolatum (43).jpg	fold2	1	*Amblyomma*
4223	Dorsal	MobileNetV2	dorsal aureolatum (44).jpg	fold2	1	*Amblyomma*
4224	Dorsal	MobileNetV2	dorsal aureolatum (49).jpg	fold2	1	*Amblyomma*
4225	Dorsal	MobileNetV2	dorsal aureolatum (5).jpg	fold2	1	*Amblyomma*
4226	Dorsal	MobileNetV2	dorsal aureolatum (51).jpg	fold2	1	*Amblyomma*
4227	Dorsal	MobileNetV2	dorsal aureolatum (6).jpg	fold2	1	*Amblyomma*
4228	Dorsal	MobileNetV2	dorsal aureolatum (62).jpg	fold2	1	*Amblyomma*
4229	Dorsal	MobileNetV2	dorsal cajennense (15).jpg	fold2	2	*Amblyomma*
4230	Dorsal	MobileNetV2	dorsal cajennense (16).jpg	fold2	2	*Amblyomma*
4231	Dorsal	MobileNetV2	dorsal cajennense (23).jpg	fold2	2	*Amblyomma*
4232	Dorsal	MobileNetV2	dorsal cajennense (27).jpg	fold2	2	*Amblyomma*
4233	Dorsal	MobileNetV2	dorsal cajennense (31).jpg	fold2	2	*Amblyomma*
4234	Dorsal	MobileNetV2	dorsal cajennense (37).jpg	fold2	2	*Amblyomma*
4235	Dorsal	MobileNetV2	dorsal cajennense (43).jpg	fold2	2	*Amblyomma*
4236	Dorsal	MobileNetV2	dorsal cajennense (51).jpg	fold2	2	*Amblyomma*
4237	Dorsal	MobileNetV2	dorsal cajennense (59).jpg	fold2	2	*Amblyomma*
4238	Dorsal	MobileNetV2	dorsal cajennense (61).jpg	fold2	2	*Amblyomma*
4239	Dorsal	MobileNetV2	dorsal cajennense (72).jpg	fold2	2	*Amblyomma*
4240	Dorsal	MobileNetV2	dorsal cajennense (73).jpg	fold2	2	*Amblyomma*
4241	Dorsal	MobileNetV2	dorsal cajennense (74).jpg	fold2	2	*Amblyomma*
4242	Dorsal	MobileNetV2	dorsal cajennense (8).jpg	fold2	2	*Amblyomma*
4243	Dorsal	MobileNetV2	dorsal cajennense (83).jpg	fold2	2	*Amblyomma*
4244	Dorsal	MobileNetV2	dorsal cajennense (9).jpg	fold2	2	*Amblyomma*
4245	Dorsal	MobileNetV2	dorsal dubitatum (14).jpg	fold2	3	*Amblyomma*
4246	Dorsal	MobileNetV2	dorsal dubitatum (15).jpg	fold2	3	*Amblyomma*
4247	Dorsal	MobileNetV2	dorsal dubitatum (2).jpg	fold2	3	*Amblyomma*
4248	Dorsal	MobileNetV2	dorsal dubitatum (34).jpg	fold2	3	*Amblyomma*
4249	Dorsal	MobileNetV2	dorsal dubitatum (36).jpg	fold2	3	*Amblyomma*
4250	Dorsal	MobileNetV2	dorsal dubitatum (37).jpg	fold2	3	*Amblyomma*
4251	Dorsal	MobileNetV2	dorsal dubitatum (43).jpg	fold2	3	*Amblyomma*
4252	Dorsal	MobileNetV2	dorsal dubitatum (44).jpg	fold2	3	*Amblyomma*
4253	Dorsal	MobileNetV2	dorsal dubitatum (47).jpg	fold2	3	*Amblyomma*
4254	Dorsal	MobileNetV2	dorsal dubitatum (5).jpg	fold2	3	*Amblyomma*
4255	Dorsal	MobileNetV2	dorsal dubitatum (50).jpg	fold2	3	*Amblyomma*
4256	Dorsal	MobileNetV2	dorsal dubitatum (7).jpg	fold2	3	*Amblyomma*
4257	Dorsal	MobileNetV2	dorsal ovale (15).jpg	fold2	4	*Amblyomma*
4258	Dorsal	MobileNetV2	dorsal ovale (18).jpg	fold2	4	*Amblyomma*
4259	Dorsal	MobileNetV2	dorsal ovale (22).jpg	fold2	4	*Amblyomma*
4260	Dorsal	MobileNetV2	dorsal ovale (23).jpg	fold2	4	*Amblyomma*
4261	Dorsal	MobileNetV2	dorsal ovale (27).jpg	fold2	4	*Amblyomma*
4262	Dorsal	MobileNetV2	dorsal ovale (33).jpg	fold2	4	*Amblyomma*
4263	Dorsal	MobileNetV2	dorsal ovale (38).JPG	fold2	4	*Amblyomma*
4264	Dorsal	MobileNetV2	dorsal ovale (41).jpg	fold2	4	*Amblyomma*
4265	Dorsal	MobileNetV2	dorsal ovale (42).jpg	fold2	4	*Amblyomma*
4266	Dorsal	MobileNetV2	dorsal ovale (49).jpg	fold2	4	*Amblyomma*
4267	Dorsal	MobileNetV2	dorsal ovale (5).jpg	fold2	4	*Amblyomma*
4268	Dorsal	MobileNetV2	dorsal ovale (6).jpg	fold2	4	*Amblyomma*
4269	Dorsal	MobileNetV2	dorsal sculptum (1).jpg	fold2	5	*Amblyomma*
4270	Dorsal	MobileNetV2	dorsal sculptum (13).JPG	fold2	5	*Amblyomma*
4271	Dorsal	MobileNetV2	dorsal sculptum (17).JPG	fold2	5	*Amblyomma*
4272	Dorsal	MobileNetV2	dorsal sculptum (26).JPG	fold2	5	*Amblyomma*
4273	Dorsal	MobileNetV2	dorsal sculptum (27).JPG	fold2	5	*Amblyomma*
4274	Dorsal	MobileNetV2	dorsal sculptum (31).jpg	fold2	5	*Amblyomma*
4275	Dorsal	MobileNetV2	dorsal sculptum (36).jpg	fold2	5	*Amblyomma*
4276	Dorsal	MobileNetV2	dorsal sculptum (40).JPG	fold2	5	*Amblyomma*
4277	Dorsal	MobileNetV2	dorsal sculptum (41).JPG	fold2	5	*Amblyomma*
4278	Dorsal	MobileNetV2	dorsal sculptum (50).JPG	fold2	5	*Amblyomma*
4279	Dorsal	MobileNetV2	dorsal sculptum (57).JPG	fold2	5	*Amblyomma*
4280	Dorsal	MobileNetV2	dorsal sculptum (58).JPG	fold2	5	*Amblyomma*
4281	Dorsal	MobileNetV2	dorsal sculptum (63).JPG	fold2	5	*Amblyomma*
4282	Dorsal	MobileNetV2	dorsal sculptum (65).JPG	fold2	5	*Amblyomma*
4283	Dorsal	MobileNetV2	dorsal sculptum (73).JPG	fold2	5	*Amblyomma*
4284	Dorsal	MobileNetV2	dorsal sculptum (77).jpg	fold2	5	*Amblyomma*
4285	Dorsal	MobileNetV2	dorsal sculptum (81).jpg	fold2	5	*Amblyomma*
4286	Dorsal	MobileNetV2	dorsal triste (11).jpg	fold2	6	*Amblyomma*

4287	Dorsal	MobileNetV2	dorsal triste (17).jpg	fold2	6	*Amblyomma*
4288	Dorsal	MobileNetV2	dorsal triste (28).jpg	fold2	6	*Amblyomma*
4289	Dorsal	MobileNetV2	dorsal triste (35).jpg	fold2	6	*Amblyomma*
4290	Dorsal	MobileNetV2	dorsal triste (37).jpg	fold2	6	*Amblyomma*
4291	Dorsal	MobileNetV2	dorsal triste (38).jpg	fold2	6	*Amblyomma*
4292	Dorsal	MobileNetV2	dorsal triste (39).jpg	fold2	6	*Amblyomma*
4293	Dorsal	MobileNetV2	dorsal triste (43).jpg	fold2	6	*Amblyomma*
4294	Dorsal	MobileNetV2	dorsal triste (50).jpg	fold2	6	*Amblyomma*
4295	Dorsal	MobileNetV2	dorsal triste (62).jpg	fold2	6	*Amblyomma*
4296	Dorsal	MobileNetV2	dorsal triste (65).jpg	fold2	6	*Amblyomma*
4297	Dorsal	MobileNetV2	dorsal triste (67).jpg	fold2	6	*Amblyomma*
4298	Dorsal	MobileNetV2	dorsal triste (68).jpg	fold2	6	*Amblyomma*
4299	Dorsal	MobileNetV2	dorsal triste (71).jpg	fold2	6	*Amblyomma*
4300	Dorsal	MobileNetV2	dorsal triste (75).jpg	fold2	6	*Amblyomma*
4301	Dorsal	MobileNetV2	dorsal triste (77).jpg	fold2	6	*Amblyomma*
4302	Dorsal	MobileNetV2	dorsal aureolatum (1).jpg	fold3	1	*Amblyomma*
4303	Dorsal	MobileNetV2	dorsal aureolatum (14).jpg	fold3	1	*Amblyomma*
4304	Dorsal	MobileNetV2	dorsal aureolatum (19).jpg	fold3	1	*Amblyomma*
4305	Dorsal	MobileNetV2	dorsal aureolatum (24).jpg	fold3	1	*Amblyomma*
4306	Dorsal	MobileNetV2	dorsal aureolatum (31).jpg	fold3	1	*Amblyomma*
4307	Dorsal	MobileNetV2	dorsal aureolatum (33).jpg	fold3	1	*Amblyomma*
4308	Dorsal	MobileNetV2	dorsal aureolatum (35).jpg	fold3	1	*Amblyomma*
4309	Dorsal	MobileNetV2	dorsal aureolatum (36).jpg	fold3	1	*Amblyomma*
4310	Dorsal	MobileNetV2	dorsal aureolatum (40).jpg	fold3	1	*Amblyomma*
4311	Dorsal	MobileNetV2	dorsal aureolatum (53).jpg	fold3	1	*Amblyomma*
4312	Dorsal	MobileNetV2	dorsal aureolatum (54).jpg	fold3	1	*Amblyomma*
4313	Dorsal	MobileNetV2	dorsal aureolatum (9).jpg	fold3	1	*Amblyomma*
4314	Dorsal	MobileNetV2	dorsal cajennense (10).jpg	fold3	2	*Amblyomma*
4315	Dorsal	MobileNetV2	dorsal cajennense (17).jpg	fold3	2	*Amblyomma*
4316	Dorsal	MobileNetV2	dorsal cajennense (2).jpg	fold3	2	*Amblyomma*
4317	Dorsal	MobileNetV2	dorsal cajennense (29).jpg	fold3	2	*Amblyomma*
4318	Dorsal	MobileNetV2	dorsal cajennense (35).jpg	fold3	2	*Amblyomma*
4319	Dorsal	MobileNetV2	dorsal cajennense (46).jpg	fold3	2	*Amblyomma*
4320	Dorsal	MobileNetV2	dorsal cajennense (48).jpg	fold3	2	*Amblyomma*
4321	Dorsal	MobileNetV2	dorsal cajennense (5).jpg	fold3	2	*Amblyomma*
4322	Dorsal	MobileNetV2	dorsal cajennense (53).jpg	fold3	2	*Amblyomma*
4323	Dorsal	MobileNetV2	dorsal cajennense (56).jpg	fold3	2	*Amblyomma*
4324	Dorsal	MobileNetV2	dorsal cajennense (57).jpg	fold3	2	*Amblyomma*
4325	Dorsal	MobileNetV2	dorsal cajennense (60).jpg	fold3	2	*Amblyomma*
4326	Dorsal	MobileNetV2	dorsal cajennense (62).jpg	fold3	2	*Amblyomma*
4327	Dorsal	MobileNetV2	dorsal cajennense (67).jpg	fold3	2	*Amblyomma*
4328	Dorsal	MobileNetV2	dorsal cajennense (70).jpg	fold3	2	*Amblyomma*
4329	Dorsal	MobileNetV2	dorsal cajennense (81).jpg	fold3	2	*Amblyomma*
4330	Dorsal	MobileNetV2	dorsal cajennense (82).jpg	fold3	2	*Amblyomma*
4331	Dorsal	MobileNetV2	dorsal dubitatum (1).jpg	fold3	3	*Amblyomma*
4332	Dorsal	MobileNetV2	dorsal dubitatum (11).jpg	fold3	3	*Amblyomma*
4333	Dorsal	MobileNetV2	dorsal dubitatum (26).jpg	fold3	3	*Amblyomma*
4334	Dorsal	MobileNetV2	dorsal dubitatum (27).jpg	fold3	3	*Amblyomma*
4335	Dorsal	MobileNetV2	dorsal dubitatum (38).jpg	fold3	3	*Amblyomma*
4336	Dorsal	MobileNetV2	dorsal dubitatum (39).jpg	fold3	3	*Amblyomma*
4337	Dorsal	MobileNetV2	dorsal dubitatum (4).jpg	fold3	3	*Amblyomma*
4338	Dorsal	MobileNetV2	dorsal dubitatum (41).jpg	fold3	3	*Amblyomma*
4339	Dorsal	MobileNetV2	dorsal dubitatum (42).jpg	fold3	3	*Amblyomma*
4340	Dorsal	MobileNetV2	dorsal dubitatum (48).jpg	fold3	3	*Amblyomma*
4341	Dorsal	MobileNetV2	dorsal dubitatum (56).jpg	fold3	3	*Amblyomma*
4342	Dorsal	MobileNetV2	dorsal ovale (17).jpg	fold3	4	*Amblyomma*
4343	Dorsal	MobileNetV2	dorsal ovale (19).jpg	fold3	4	*Amblyomma*
4344	Dorsal	MobileNetV2	dorsal ovale (2).jpg	fold3	4	*Amblyomma*
4345	Dorsal	MobileNetV2	dorsal ovale (30).jpg	fold3	4	*Amblyomma*
4346	Dorsal	MobileNetV2	dorsal ovale (32).jpg	fold3	4	*Amblyomma*
4347	Dorsal	MobileNetV2	dorsal ovale (4).jpg	fold3	4	*Amblyomma*
4348	Dorsal	MobileNetV2	dorsal ovale (40).jpg	fold3	4	*Amblyomma*
4349	Dorsal	MobileNetV2	dorsal ovale (50).jpg	fold3	4	*Amblyomma*
4350	Dorsal	MobileNetV2	dorsal ovale (52).jpg	fold3	4	*Amblyomma*
4351	Dorsal	MobileNetV2	dorsal ovale (54).JPG	fold3	4	*Amblyomma*
4352	Dorsal	MobileNetV2	dorsal ovale (57).jpg	fold3	4	*Amblyomma*
4353	Dorsal	MobileNetV2	dorsal ovale (60).jpg	fold3	4	*Amblyomma*

4354	Dorsal	MobileNetV2	dorsal sculptum (11).JPG	fold3	5	*Amblyomma*
4355	Dorsal	MobileNetV2	dorsal sculptum (12).JPG	fold3	5	*Amblyomma*
4356	Dorsal	MobileNetV2	dorsal sculptum (16).JPG	fold3	5	*Amblyomma*
4357	Dorsal	MobileNetV2	dorsal sculptum (39).JPG	fold3	5	*Amblyomma*
4358	Dorsal	MobileNetV2	dorsal sculptum (4).jpg	fold3	5	*Amblyomma*
4359	Dorsal	MobileNetV2	dorsal sculptum (43).JPG	fold3	5	*Amblyomma*
4360	Dorsal	MobileNetV2	dorsal sculptum (48).JPG	fold3	5	*Amblyomma*
4361	Dorsal	MobileNetV2	dorsal sculptum (53).JPG	fold3	5	*Amblyomma*
4362	Dorsal	MobileNetV2	dorsal sculptum (54).JPG	fold3	5	*Amblyomma*
4363	Dorsal	MobileNetV2	dorsal sculptum (56).JPG	fold3	5	*Amblyomma*
4364	Dorsal	MobileNetV2	dorsal sculptum (60).JPG	fold3	5	*Amblyomma*
4365	Dorsal	MobileNetV2	dorsal sculptum (66).JPG	fold3	5	*Amblyomma*
4366	Dorsal	MobileNetV2	dorsal sculptum (71).JPG	fold3	5	*Amblyomma*
4367	Dorsal	MobileNetV2	dorsal sculptum (74).JPG	fold3	5	*Amblyomma*
4368	Dorsal	MobileNetV2	dorsal sculptum (79).jpg	fold3	5	*Amblyomma*
4369	Dorsal	MobileNetV2	dorsal sculptum (8).JPG	fold3	5	*Amblyomma*
4370	Dorsal	MobileNetV2	dorsal triste (12).jpg	fold3	6	*Amblyomma*
4371	Dorsal	MobileNetV2	dorsal triste (13).jpg	fold3	6	*Amblyomma*
4372	Dorsal	MobileNetV2	dorsal triste (15).jpg	fold3	6	*Amblyomma*
4373	Dorsal	MobileNetV2	dorsal triste (16).jpg	fold3	6	*Amblyomma*
4374	Dorsal	MobileNetV2	dorsal triste (18).jpg	fold3	6	*Amblyomma*
4375	Dorsal	MobileNetV2	dorsal triste (21).jpg	fold3	6	*Amblyomma*
4376	Dorsal	MobileNetV2	dorsal triste (27).jpg	fold3	6	*Amblyomma*
4377	Dorsal	MobileNetV2	dorsal triste (30).jpg	fold3	6	*Amblyomma*
4378	Dorsal	MobileNetV2	dorsal triste (41).jpg	fold3	6	*Amblyomma*
4379	Dorsal	MobileNetV2	dorsal triste (49).jpg	fold3	6	*Amblyomma*
4380	Dorsal	MobileNetV2	dorsal triste (54).jpg	fold3	6	*Amblyomma*
4381	Dorsal	MobileNetV2	dorsal triste (56).jpg	fold3	6	*Amblyomma*
4382	Dorsal	MobileNetV2	dorsal triste (64).jpg	fold3	6	*Amblyomma*
4383	Dorsal	MobileNetV2	dorsal triste (66).jpg	fold3	6	*Amblyomma*
4384	Dorsal	MobileNetV2	dorsal triste (7).jpg	fold3	6	*Amblyomma*
4385	Dorsal	MobileNetV2	dorsal triste (73).jpg	fold3	6	*Amblyomma*
4386	Dorsal	MobileNetV2	dorsal aureolatum (12).jpg	fold4	1	*Amblyomma*
4387	Dorsal	MobileNetV2	dorsal aureolatum (13).jpg	fold4	1	*Amblyomma*
4388	Dorsal	MobileNetV2	dorsal aureolatum (15).jpg	fold4	1	*Amblyomma*
4389	Dorsal	MobileNetV2	dorsal aureolatum (16).jpg	fold4	1	*Amblyomma*
4390	Dorsal	MobileNetV2	dorsal aureolatum (20).jpg	fold4	1	*Amblyomma*
4391	Dorsal	MobileNetV2	dorsal aureolatum (22).jpg	fold4	1	*Amblyomma*
4392	Dorsal	MobileNetV2	dorsal aureolatum (25).jpg	fold4	1	*Amblyomma*
4393	Dorsal	MobileNetV2	dorsal aureolatum (26).jpg	fold4	1	*Amblyomma*
4394	Dorsal	MobileNetV2	dorsal aureolatum (37).jpg	fold4	1	*Amblyomma*
4395	Dorsal	MobileNetV2	dorsal aureolatum (4).jpg	fold4	1	*Amblyomma*
4396	Dorsal	MobileNetV2	dorsal aureolatum (56).jpg	fold4	1	*Amblyomma*
4397	Dorsal	MobileNetV2	dorsal aureolatum (63).jpg	fold4	1	*Amblyomma*
4398	Dorsal	MobileNetV2	dorsal aureolatum (7).jpg	fold4	1	*Amblyomma*
4399	Dorsal	MobileNetV2	dorsal cajennense (1).jpg	fold4	2	*Amblyomma*
4400	Dorsal	MobileNetV2	dorsal cajennense (11).jpg	fold4	2	*Amblyomma*
4401	Dorsal	MobileNetV2	dorsal cajennense (12).jpg	fold4	2	*Amblyomma*
4402	Dorsal	MobileNetV2	dorsal cajennense (13).jpg	fold4	2	*Amblyomma*
4403	Dorsal	MobileNetV2	dorsal cajennense (20).jpg	fold4	2	*Amblyomma*
4404	Dorsal	MobileNetV2	dorsal cajennense (33).jpg	fold4	2	*Amblyomma*
4405	Dorsal	MobileNetV2	dorsal cajennense (34).jpg	fold4	2	*Amblyomma*
4406	Dorsal	MobileNetV2	dorsal cajennense (36).jpg	fold4	2	*Amblyomma*
4407	Dorsal	MobileNetV2	dorsal cajennense (38).jpg	fold4	2	*Amblyomma*
4408	Dorsal	MobileNetV2	dorsal cajennense (4).jpg	fold4	2	*Amblyomma*
4409	Dorsal	MobileNetV2	dorsal cajennense (45).jpg	fold4	2	*Amblyomma*
4410	Dorsal	MobileNetV2	dorsal cajennense (6).jpg	fold4	2	*Amblyomma*
4411	Dorsal	MobileNetV2	dorsal cajennense (63).jpg	fold4	2	*Amblyomma*
4412	Dorsal	MobileNetV2	dorsal cajennense (64).jpg	fold4	2	*Amblyomma*
4413	Dorsal	MobileNetV2	dorsal cajennense (76).jpg	fold4	2	*Amblyomma*
4414	Dorsal	MobileNetV2	dorsal cajennense (78).jpg	fold4	2	*Amblyomma*
4415	Dorsal	MobileNetV2	dorsal cajennense (79).jpg	fold4	2	*Amblyomma*
4416	Dorsal	MobileNetV2	dorsal dubitatum (12).jpg	fold4	3	*Amblyomma*
4417	Dorsal	MobileNetV2	dorsal dubitatum (13).jpg	fold4	3	*Amblyomma*
4418	Dorsal	MobileNetV2	dorsal dubitatum (17).jpg	fold4	3	*Amblyomma*
4419	Dorsal	MobileNetV2	dorsal dubitatum (20).jpg	fold4	3	*Amblyomma*
4420	Dorsal	MobileNetV2	dorsal dubitatum (21).jpg	fold4	3	*Amblyomma*

4421	Dorsal	MobileNetV2	dorsal dubitatum (22).jpg	fold4	3	*Amblyomma*
4422	Dorsal	MobileNetV2	dorsal dubitatum (3).jpg	fold4	3	*Amblyomma*
4423	Dorsal	MobileNetV2	dorsal dubitatum (31).jpg	fold4	3	*Amblyomma*
4424	Dorsal	MobileNetV2	dorsal dubitatum (35).jpg	fold4	3	*Amblyomma*
4425	Dorsal	MobileNetV2	dorsal dubitatum (54).jpg	fold4	3	*Amblyomma*
4426	Dorsal	MobileNetV2	dorsal dubitatum (8).JPG	fold4	3	*Amblyomma*
4427	Dorsal	MobileNetV2	dorsal ovale (1).jpg	fold4	4	*Amblyomma*
4428	Dorsal	MobileNetV2	dorsal ovale (13).jpg	fold4	4	*Amblyomma*
4429	Dorsal	MobileNetV2	dorsal ovale (16).jpg	fold4	4	*Amblyomma*
4430	Dorsal	MobileNetV2	dorsal ovale (24).jpg	fold4	4	*Amblyomma*
4431	Dorsal	MobileNetV2	dorsal ovale (25).jpg	fold4	4	*Amblyomma*
4432	Dorsal	MobileNetV2	dorsal ovale (31).jpg	fold4	4	*Amblyomma*
4433	Dorsal	MobileNetV2	dorsal ovale (34).jpg	fold4	4	*Amblyomma*
4434	Dorsal	MobileNetV2	dorsal ovale (36).jpg	fold4	4	*Amblyomma*
4435	Dorsal	MobileNetV2	dorsal ovale (37).jpg	fold4	4	*Amblyomma*
4436	Dorsal	MobileNetV2	dorsal ovale (39).jpg	fold4	4	*Amblyomma*
4437	Dorsal	MobileNetV2	dorsal ovale (55).jpg	fold4	4	*Amblyomma*
4438	Dorsal	MobileNetV2	dorsal ovale (9).jpg	fold4	4	*Amblyomma*
4439	Dorsal	MobileNetV2	dorsal sculptum (15).JPG	fold4	5	*Amblyomma*
4440	Dorsal	MobileNetV2	dorsal sculptum (19).JPG	fold4	5	*Amblyomma*
4441	Dorsal	MobileNetV2	dorsal sculptum (2).jpg	fold4	5	*Amblyomma*
4442	Dorsal	MobileNetV2	dorsal sculptum (24).JPG	fold4	5	*Amblyomma*
4443	Dorsal	MobileNetV2	dorsal sculptum (30).jpg	fold4	5	*Amblyomma*
4444	Dorsal	MobileNetV2	dorsal sculptum (34).jpg	fold4	5	*Amblyomma*
4445	Dorsal	MobileNetV2	dorsal sculptum (35).jpg	fold4	5	*Amblyomma*
4446	Dorsal	MobileNetV2	dorsal sculptum (44).JPG	fold4	5	*Amblyomma*
4447	Dorsal	MobileNetV2	dorsal sculptum (45).JPG	fold4	5	*Amblyomma*
4448	Dorsal	MobileNetV2	dorsal sculptum (49).JPG	fold4	5	*Amblyomma*
4449	Dorsal	MobileNetV2	dorsal sculptum (55).JPG	fold4	5	*Amblyomma*
4450	Dorsal	MobileNetV2	dorsal sculptum (61).JPG	fold4	5	*Amblyomma*
4451	Dorsal	MobileNetV2	dorsal sculptum (62).JPG	fold4	5	*Amblyomma*
4452	Dorsal	MobileNetV2	dorsal sculptum (64).JPG	fold4	5	*Amblyomma*
4453	Dorsal	MobileNetV2	dorsal sculptum (69).JPG	fold4	5	*Amblyomma*
4454	Dorsal	MobileNetV2	dorsal sculptum (80).jpg	fold4	5	*Amblyomma*
4455	Dorsal	MobileNetV2	dorsal triste (10).jpg	fold4	6	*Amblyomma*
4456	Dorsal	MobileNetV2	dorsal triste (23).jpg	fold4	6	*Amblyomma*
4457	Dorsal	MobileNetV2	dorsal triste (29).jpg	fold4	6	*Amblyomma*
4458	Dorsal	MobileNetV2	dorsal triste (31).jpg	fold4	6	*Amblyomma*
4459	Dorsal	MobileNetV2	dorsal triste (32).jpg	fold4	6	*Amblyomma*
4460	Dorsal	MobileNetV2	dorsal triste (34).jpg	fold4	6	*Amblyomma*
4461	Dorsal	MobileNetV2	dorsal triste (4).jpg	fold4	6	*Amblyomma*
4462	Dorsal	MobileNetV2	dorsal triste (40).jpg	fold4	6	*Amblyomma*
4463	Dorsal	MobileNetV2	dorsal triste (5).jpg	fold4	6	*Amblyomma*
4464	Dorsal	MobileNetV2	dorsal triste (53).jpg	fold4	6	*Amblyomma*
4465	Dorsal	MobileNetV2	dorsal triste (55).jpg	fold4	6	*Amblyomma*
4466	Dorsal	MobileNetV2	dorsal triste (69).jpg	fold4	6	*Amblyomma*
4467	Dorsal	MobileNetV2	dorsal triste (70).jpg	fold4	6	*Amblyomma*
4468	Dorsal	MobileNetV2	dorsal triste (76).jpg	fold4	6	*Amblyomma*
4469	Dorsal	MobileNetV2	dorsal triste (8).jpg	fold4	6	*Amblyomma*
4470	Dorsal	MobileNetV2	dorsal aureolatum (10).jpg	fold5	1	*Amblyomma*
4471	Dorsal	MobileNetV2	dorsal aureolatum (2).jpg	fold5	1	*Amblyomma*
4472	Dorsal	MobileNetV2	dorsal aureolatum (3).jpg	fold5	1	*Amblyomma*
4473	Dorsal	MobileNetV2	dorsal aureolatum (34).jpg	fold5	1	*Amblyomma*
4474	Dorsal	MobileNetV2	dorsal aureolatum (39).jpg	fold5	1	*Amblyomma*
4475	Dorsal	MobileNetV2	dorsal aureolatum (41).jpg	fold5	1	*Amblyomma*
4476	Dorsal	MobileNetV2	dorsal aureolatum (42).jpg	fold5	1	*Amblyomma*
4477	Dorsal	MobileNetV2	dorsal aureolatum (45).jpg	fold5	1	*Amblyomma*
4478	Dorsal	MobileNetV2	dorsal aureolatum (47).jpg	fold5	1	*Amblyomma*
4479	Dorsal	MobileNetV2	dorsal aureolatum (50).jpg	fold5	1	*Amblyomma*
4480	Dorsal	MobileNetV2	dorsal aureolatum (57).jpg	fold5	1	*Amblyomma*
4481	Dorsal	MobileNetV2	dorsal aureolatum (59).jpg	fold5	1	*Amblyomma*
4482	Dorsal	MobileNetV2	dorsal aureolatum (60).jpg	fold5	1	*Amblyomma*
4483	Dorsal	MobileNetV2	dorsal cajennense (14).jpg	fold5	2	*Amblyomma*
4484	Dorsal	MobileNetV2	dorsal cajennense (18).jpg	fold5	2	*Amblyomma*
4485	Dorsal	MobileNetV2	dorsal cajennense (19).jpg	fold5	2	*Amblyomma*
4486	Dorsal	MobileNetV2	dorsal cajennense (21).jpg	fold5	2	*Amblyomma*
4487	Dorsal	MobileNetV2	dorsal cajennense (22).jpg	fold5	2	*Amblyomma*

4488	Dorsal	MobileNetV2	dorsal cajennense (24).jpg	fold5	2	*Amblyomma*
4489	Dorsal	MobileNetV2	dorsal cajennense (28).jpg	fold5	2	*Amblyomma*
4490	Dorsal	MobileNetV2	dorsal cajennense (30).jpg	fold5	2	*Amblyomma*
4491	Dorsal	MobileNetV2	dorsal cajennense (39).jpg	fold5	2	*Amblyomma*
4492	Dorsal	MobileNetV2	dorsal cajennense (40).jpg	fold5	2	*Amblyomma*
4493	Dorsal	MobileNetV2	dorsal cajennense (41).jpg	fold5	2	*Amblyomma*
4494	Dorsal	MobileNetV2	dorsal cajennense (42).jpg	fold5	2	*Amblyomma*
4495	Dorsal	MobileNetV2	dorsal cajennense (49).jpg	fold5	2	*Amblyomma*
4496	Dorsal	MobileNetV2	dorsal cajennense (55).jpg	fold5	2	*Amblyomma*
4497	Dorsal	MobileNetV2	dorsal cajennense (68).jpg	fold5	2	*Amblyomma*
4498	Dorsal	MobileNetV2	dorsal cajennense (69).jpg	fold5	2	*Amblyomma*
4499	Dorsal	MobileNetV2	dorsal cajennense (71).jpg	fold5	2	*Amblyomma*
4500	Dorsal	MobileNetV2	dorsal dubitatum (16).jpg	fold5	3	*Amblyomma*
4501	Dorsal	MobileNetV2	dorsal dubitatum (23).jpg	fold5	3	*Amblyomma*
4502	Dorsal	MobileNetV2	dorsal dubitatum (24).jpg	fold5	3	*Amblyomma*
4503	Dorsal	MobileNetV2	dorsal dubitatum (25).jpg	fold5	3	*Amblyomma*
4504	Dorsal	MobileNetV2	dorsal dubitatum (29).jpg	fold5	3	*Amblyomma*
4505	Dorsal	MobileNetV2	dorsal dubitatum (30).jpg	fold5	3	*Amblyomma*
4506	Dorsal	MobileNetV2	dorsal dubitatum (33).jpg	fold5	3	*Amblyomma*
4507	Dorsal	MobileNetV2	dorsal dubitatum (45).jpg	fold5	3	*Amblyomma*
4508	Dorsal	MobileNetV2	dorsal dubitatum (51).jpg	fold5	3	*Amblyomma*
4509	Dorsal	MobileNetV2	dorsal dubitatum (52).jpg	fold5	3	*Amblyomma*
4510	Dorsal	MobileNetV2	dorsal dubitatum (53).jpg	fold5	3	*Amblyomma*
4511	Dorsal	MobileNetV2	dorsal ovale (11).jpg	fold5	4	*Amblyomma*
4512	Dorsal	MobileNetV2	dorsal ovale (12).jpg	fold5	4	*Amblyomma*
4513	Dorsal	MobileNetV2	dorsal ovale (28).jpg	fold5	4	*Amblyomma*
4514	Dorsal	MobileNetV2	dorsal ovale (3).jpg	fold5	4	*Amblyomma*
4515	Dorsal	MobileNetV2	dorsal ovale (35).jpg	fold5	4	*Amblyomma*
4516	Dorsal	MobileNetV2	dorsal ovale (43).jpg	fold5	4	*Amblyomma*
4517	Dorsal	MobileNetV2	dorsal ovale (45).jpg	fold5	4	*Amblyomma*
4518	Dorsal	MobileNetV2	dorsal ovale (46).jpg	fold5	4	*Amblyomma*
4519	Dorsal	MobileNetV2	dorsal ovale (47).jpg	fold5	4	*Amblyomma*
4520	Dorsal	MobileNetV2	dorsal ovale (56).jpg	fold5	4	*Amblyomma*
4521	Dorsal	MobileNetV2	dorsal ovale (58).jpg	fold5	4	*Amblyomma*
4522	Dorsal	MobileNetV2	dorsal ovale (59).jpg	fold5	4	*Amblyomma*
4523	Dorsal	MobileNetV2	dorsal ovale (8).jpg	fold5	4	*Amblyomma*
4524	Dorsal	MobileNetV2	dorsal sculptum (10).JPG	fold5	5	*Amblyomma*
4525	Dorsal	MobileNetV2	dorsal sculptum (18).JPG	fold5	5	*Amblyomma*
4526	Dorsal	MobileNetV2	dorsal sculptum (21).JPG	fold5	5	*Amblyomma*
4527	Dorsal	MobileNetV2	dorsal sculptum (28).JPG	fold5	5	*Amblyomma*
4528	Dorsal	MobileNetV2	dorsal sculptum (3).jpg	fold5	5	*Amblyomma*
4529	Dorsal	MobileNetV2	dorsal sculptum (32).jpg	fold5	5	*Amblyomma*
4530	Dorsal	MobileNetV2	dorsal sculptum (33).jpg	fold5	5	*Amblyomma*
4531	Dorsal	MobileNetV2	dorsal sculptum (46).JPG	fold5	5	*Amblyomma*
4532	Dorsal	MobileNetV2	dorsal sculptum (47).JPG	fold5	5	*Amblyomma*
4533	Dorsal	MobileNetV2	dorsal sculptum (6).JPG	fold5	5	*Amblyomma*
4534	Dorsal	MobileNetV2	dorsal sculptum (67).JPG	fold5	5	*Amblyomma*
4535	Dorsal	MobileNetV2	dorsal sculptum (68).JPG	fold5	5	*Amblyomma*
4536	Dorsal	MobileNetV2	dorsal sculptum (7).JPG	fold5	5	*Amblyomma*
4537	Dorsal	MobileNetV2	dorsal sculptum (70).JPG	fold5	5	*Amblyomma*
4538	Dorsal	MobileNetV2	dorsal sculptum (76).jpg	fold5	5	*Amblyomma*
4539	Dorsal	MobileNetV2	dorsal sculptum (78).jpg	fold5	5	*Amblyomma*
4540	Dorsal	MobileNetV2	dorsal triste (1).jpg	fold5	6	*Amblyomma*
4541	Dorsal	MobileNetV2	dorsal triste (14).jpg	fold5	6	*Amblyomma*
4542	Dorsal	MobileNetV2	dorsal triste (22).jpg	fold5	6	*Amblyomma*
4543	Dorsal	MobileNetV2	dorsal triste (3).jpg	fold5	6	*Amblyomma*
4544	Dorsal	MobileNetV2	dorsal triste (33).jpg	fold5	6	*Amblyomma*
4545	Dorsal	MobileNetV2	dorsal triste (36).jpg	fold5	6	*Amblyomma*
4546	Dorsal	MobileNetV2	dorsal triste (42).jpg	fold5	6	*Amblyomma*
4547	Dorsal	MobileNetV2	dorsal triste (44).jpg	fold5	6	*Amblyomma*
4548	Dorsal	MobileNetV2	dorsal triste (48).jpg	fold5	6	*Amblyomma*
4549	Dorsal	MobileNetV2	dorsal triste (52).jpg	fold5	6	*Amblyomma*
4550	Dorsal	MobileNetV2	dorsal triste (57).jpg	fold5	6	*Amblyomma*
4551	Dorsal	MobileNetV2	dorsal triste (58).jpg	fold5	6	*Amblyomma*
4552	Dorsal	MobileNetV2	dorsal triste (59).jpg	fold5	6	*Amblyomma*
4553	Dorsal	MobileNetV2	dorsal triste (60).jpg	fold5	6	*Amblyomma*
4554	Dorsal	MobileNetV2	dorsal triste (78).jpg	fold5	6	*Amblyomma*

4555	Dorsal	MobileNetV2	dorsal triste (9).jpg	fold5	6	*Amblyomma*
4556	Ventral	MobileNetV2	ventral aureolatum (10).jpg	fold1	1	*Amblyomma*
4557	Ventral	MobileNetV2	ventral aureolatum (16).jpg	fold1	1	*Amblyomma*
4558	Ventral	MobileNetV2	ventral aureolatum (20).jpg	fold1	1	*Amblyomma*
4559	Ventral	MobileNetV2	ventral aureolatum (21).jpg	fold1	1	*Amblyomma*
4560	Ventral	MobileNetV2	ventral aureolatum (28).jpg	fold1	1	*Amblyomma*
4561	Ventral	MobileNetV2	ventral aureolatum (3).jpg	fold1	1	*Amblyomma*
4562	Ventral	MobileNetV2	ventral aureolatum (31).jpg	fold1	1	*Amblyomma*
4563	Ventral	MobileNetV2	ventral aureolatum (33).jpg	fold1	1	*Amblyomma*
4564	Ventral	MobileNetV2	ventral aureolatum (34).jpg	fold1	1	*Amblyomma*
4565	Ventral	MobileNetV2	ventral aureolatum (40).jpg	fold1	1	*Amblyomma*
4566	Ventral	MobileNetV2	ventral aureolatum (44).jpg	fold1	1	*Amblyomma*
4567	Ventral	MobileNetV2	ventral aureolatum (58).jpg	fold1	1	*Amblyomma*
4568	Ventral	MobileNetV2	ventral cajennense (14).jpg	fold1	2	*Amblyomma*
4569	Ventral	MobileNetV2	ventral cajennense (16).jpg	fold1	2	*Amblyomma*
4570	Ventral	MobileNetV2	ventral cajennense (28).jpg	fold1	2	*Amblyomma*
4571	Ventral	MobileNetV2	ventral cajennense (33).jpg	fold1	2	*Amblyomma*
4572	Ventral	MobileNetV2	ventral cajennense (4).jpg	fold1	2	*Amblyomma*
4573	Ventral	MobileNetV2	ventral cajennense (41).jpg	fold1	2	*Amblyomma*
4574	Ventral	MobileNetV2	ventral cajennense (5).jpg	fold1	2	*Amblyomma*
4575	Ventral	MobileNetV2	ventral cajennense (51).jpg	fold1	2	*Amblyomma*
4576	Ventral	MobileNetV2	ventral cajennense (57).jpg	fold1	2	*Amblyomma*
4577	Ventral	MobileNetV2	ventral cajennense (60).jpg	fold1	2	*Amblyomma*
4578	Ventral	MobileNetV2	ventral cajennense (64).jpg	fold1	2	*Amblyomma*
4579	Ventral	MobileNetV2	ventral cajennense (7).jpg	fold1	2	*Amblyomma*
4580	Ventral	MobileNetV2	ventral cajennense (72).jpg	fold1	2	*Amblyomma*
4581	Ventral	MobileNetV2	ventral cajennense (74).jpg	fold1	2	*Amblyomma*
4582	Ventral	MobileNetV2	ventral cajennense (75).jpg	fold1	2	*Amblyomma*
4583	Ventral	MobileNetV2	ventral cajennense (82).jpg	fold1	2	*Amblyomma*
4584	Ventral	MobileNetV2	ventral dubitatum (22).jpg	fold1	3	*Amblyomma*
4585	Ventral	MobileNetV2	ventral dubitatum (26).jpg	fold1	3	*Amblyomma*
4586	Ventral	MobileNetV2	ventral dubitatum (27).jpg	fold1	3	*Amblyomma*
4587	Ventral	MobileNetV2	ventral dubitatum (30).jpg	fold1	3	*Amblyomma*
4588	Ventral	MobileNetV2	ventral dubitatum (39).jpg	fold1	3	*Amblyomma*
4589	Ventral	MobileNetV2	ventral dubitatum (40).jpg	fold1	3	*Amblyomma*
4590	Ventral	MobileNetV2	ventral dubitatum (45).jpg	fold1	3	*Amblyomma*
4591	Ventral	MobileNetV2	ventral dubitatum (46).jpg	fold1	3	*Amblyomma*
4592	Ventral	MobileNetV2	ventral dubitatum (51).jpg	fold1	3	*Amblyomma*
4593	Ventral	MobileNetV2	ventral dubitatum (7).jpg	fold1	3	*Amblyomma*
4594	Ventral	MobileNetV2	ventral ovale (1).jpg	fold1	4	*Amblyomma*
4595	Ventral	MobileNetV2	ventral ovale (18).jpg	fold1	4	*Amblyomma*
4596	Ventral	MobileNetV2	ventral ovale (20).jpg	fold1	4	*Amblyomma*
4597	Ventral	MobileNetV2	ventral ovale (22).jpg	fold1	4	*Amblyomma*
4598	Ventral	MobileNetV2	ventral ovale (26).jpg	fold1	4	*Amblyomma*
4599	Ventral	MobileNetV2	ventral ovale (31).jpg	fold1	4	*Amblyomma*
4600	Ventral	MobileNetV2	ventral ovale (32).jpg	fold1	4	*Amblyomma*
4601	Ventral	MobileNetV2	ventral ovale (36).jpg	fold1	4	*Amblyomma*
4602	Ventral	MobileNetV2	ventral ovale (38).JPG	fold1	4	*Amblyomma*
4603	Ventral	MobileNetV2	ventral ovale (57).jpg	fold1	4	*Amblyomma*
4604	Ventral	MobileNetV2	ventral ovale (58).jpg	fold1	4	*Amblyomma*
4605	Ventral	MobileNetV2	ventral ovale (60).jpg	fold1	4	*Amblyomma*
4606	Ventral	MobileNetV2	ventral sculptum (10).JPG	fold1	5	*Amblyomma*
4607	Ventral	MobileNetV2	ventral sculptum (18).JPG	fold1	5	*Amblyomma*
4608	Ventral	MobileNetV2	ventral sculptum (2).jpg	fold1	5	*Amblyomma*
4609	Ventral	MobileNetV2	ventral sculptum (28).jpg	fold1	5	*Amblyomma*
4610	Ventral	MobileNetV2	ventral sculptum (29).jpg	fold1	5	*Amblyomma*
4611	Ventral	MobileNetV2	ventral sculptum (31).jpg	fold1	5	*Amblyomma*
4612	Ventral	MobileNetV2	ventral sculptum (32).jpg	fold1	5	*Amblyomma*
4613	Ventral	MobileNetV2	ventral sculptum (38).JPG	fold1	5	*Amblyomma*
4614	Ventral	MobileNetV2	ventral sculptum (40).JPG	fold1	5	*Amblyomma*
4615	Ventral	MobileNetV2	ventral sculptum (43).JPG	fold1	5	*Amblyomma*
4616	Ventral	MobileNetV2	ventral sculptum (45).JPG	fold1	5	*Amblyomma*
4617	Ventral	MobileNetV2	ventral sculptum (54).JPG	fold1	5	*Amblyomma*
4618	Ventral	MobileNetV2	ventral sculptum (6).JPG	fold1	5	*Amblyomma*
4619	Ventral	MobileNetV2	ventral sculptum (60).JPG	fold1	5	*Amblyomma*
4620	Ventral	MobileNetV2	ventral sculptum (67).JPG	fold1	5	*Amblyomma*
4621	Ventral	MobileNetV2	ventral triste (11).jpg	fold1	6	*Amblyomma*

4622	Ventral	MobileNetV2	ventral triste (16).jpg	fold1	6	*Amblyomma*
4623	Ventral	MobileNetV2	ventral triste (33).jpg	fold1	6	*Amblyomma*
4624	Ventral	MobileNetV2	ventral triste (37).jpg	fold1	6	*Amblyomma*
4625	Ventral	MobileNetV2	ventral triste (38).jpg	fold1	6	*Amblyomma*
4626	Ventral	MobileNetV2	ventral triste (39).jpg	fold1	6	*Amblyomma*
4627	Ventral	MobileNetV2	ventral triste (41).jpg	fold1	6	*Amblyomma*
4628	Ventral	MobileNetV2	ventral triste (42).jpg	fold1	6	*Amblyomma*
4629	Ventral	MobileNetV2	ventral triste (47).jpg	fold1	6	*Amblyomma*
4630	Ventral	MobileNetV2	ventral triste (66).jpg	fold1	6	*Amblyomma*
4631	Ventral	MobileNetV2	ventral triste (67).jpg	fold1	6	*Amblyomma*
4632	Ventral	MobileNetV2	ventral triste (68).jpg	fold1	6	*Amblyomma*
4633	Ventral	MobileNetV2	ventral triste (72).jpg	fold1	6	*Amblyomma*
4634	Ventral	MobileNetV2	ventral triste (75).jpg	fold1	6	*Amblyomma*
4635	Ventral	MobileNetV2	ventral triste (76).jpg	fold1	6	*Amblyomma*
4636	Ventral	MobileNetV2	ventral triste (77).jpg	fold1	6	*Amblyomma*
4637	Ventral	MobileNetV2	ventral aureolatum (12).jpg	fold2	1	*Amblyomma*
4638	Ventral	MobileNetV2	ventral aureolatum (18).jpg	fold2	1	*Amblyomma*
4639	Ventral	MobileNetV2	ventral aureolatum (22).jpg	fold2	1	*Amblyomma*
4640	Ventral	MobileNetV2	ventral aureolatum (26).jpg	fold2	1	*Amblyomma*
4641	Ventral	MobileNetV2	ventral aureolatum (27).jpg	fold2	1	*Amblyomma*
4642	Ventral	MobileNetV2	ventral aureolatum (4).jpg	fold2	1	*Amblyomma*
4643	Ventral	MobileNetV2	ventral aureolatum (47).jpg	fold2	1	*Amblyomma*
4644	Ventral	MobileNetV2	ventral aureolatum (48).jpg	fold2	1	*Amblyomma*
4645	Ventral	MobileNetV2	ventral aureolatum (49).jpg	fold2	1	*Amblyomma*
4646	Ventral	MobileNetV2	ventral aureolatum (50).jpg	fold2	1	*Amblyomma*
4647	Ventral	MobileNetV2	ventral aureolatum (51).jpg	fold2	1	*Amblyomma*
4648	Ventral	MobileNetV2	ventral aureolatum (56).jpg	fold2	1	*Amblyomma*
4649	Ventral	MobileNetV2	ventral cajennense (1).jpg	fold2	2	*Amblyomma*
4650	Ventral	MobileNetV2	ventral cajennense (15).jpg	fold2	2	*Amblyomma*
4651	Ventral	MobileNetV2	ventral cajennense (20).jpg	fold2	2	*Amblyomma*
4652	Ventral	MobileNetV2	ventral cajennense (23).jpg	fold2	2	*Amblyomma*
4653	Ventral	MobileNetV2	ventral cajennense (29).jpg	fold2	2	*Amblyomma*
4654	Ventral	MobileNetV2	ventral cajennense (30).jpg	fold2	2	*Amblyomma*
4655	Ventral	MobileNetV2	ventral cajennense (37).jpg	fold2	2	*Amblyomma*
4656	Ventral	MobileNetV2	ventral cajennense (49).jpg	fold2	2	*Amblyomma*
4657	Ventral	MobileNetV2	ventral cajennense (52).jpg	fold2	2	*Amblyomma*
4658	Ventral	MobileNetV2	ventral cajennense (56).jpg	fold2	2	*Amblyomma*
4659	Ventral	MobileNetV2	ventral cajennense (63).jpg	fold2	2	*Amblyomma*
4660	Ventral	MobileNetV2	ventral cajennense (65).jpg	fold2	2	*Amblyomma*
4661	Ventral	MobileNetV2	ventral cajennense (67).jpg	fold2	2	*Amblyomma*
4662	Ventral	MobileNetV2	ventral cajennense (70).jpg	fold2	2	*Amblyomma*
4663	Ventral	MobileNetV2	ventral cajennense (71).jpg	fold2	2	*Amblyomma*
4664	Ventral	MobileNetV2	ventral cajennense (9).jpg	fold2	2	*Amblyomma*
4665	Ventral	MobileNetV2	ventral dubitatum (19).jpg	fold2	3	*Amblyomma*
4666	Ventral	MobileNetV2	ventral dubitatum (23).jpg	fold2	3	*Amblyomma*
4667	Ventral	MobileNetV2	ventral dubitatum (24).jpg	fold2	3	*Amblyomma*
4668	Ventral	MobileNetV2	ventral dubitatum (25).jpg	fold2	3	*Amblyomma*
4669	Ventral	MobileNetV2	ventral dubitatum (28).jpg	fold2	3	*Amblyomma*
4670	Ventral	MobileNetV2	ventral dubitatum (36).jpg	fold2	3	*Amblyomma*
4671	Ventral	MobileNetV2	ventral dubitatum (38).jpg	fold2	3	*Amblyomma*
4672	Ventral	MobileNetV2	ventral dubitatum (41).jpg	fold2	3	*Amblyomma*
4673	Ventral	MobileNetV2	ventral dubitatum (42).jpg	fold2	3	*Amblyomma*
4674	Ventral	MobileNetV2	ventral dubitatum (48).jpg	fold2	3	*Amblyomma*
4675	Ventral	MobileNetV2	ventral dubitatum (49).jpg	fold2	3	*Amblyomma*
4676	Ventral	MobileNetV2	ventral ovale (10).jpg	fold2	4	*Amblyomma*
4677	Ventral	MobileNetV2	ventral ovale (13).jpg	fold2	4	*Amblyomma*
4678	Ventral	MobileNetV2	ventral ovale (14).jpg	fold2	4	*Amblyomma*
4679	Ventral	MobileNetV2	ventral ovale (28).jpg	fold2	4	*Amblyomma*
4680	Ventral	MobileNetV2	ventral ovale (29).jpg	fold2	4	*Amblyomma*
4681	Ventral	MobileNetV2	ventral ovale (3).jpg	fold2	4	*Amblyomma*
4682	Ventral	MobileNetV2	ventral ovale (42).jpg	fold2	4	*Amblyomma*
4683	Ventral	MobileNetV2	ventral ovale (44).jpg	fold2	4	*Amblyomma*
4684	Ventral	MobileNetV2	ventral ovale (49).jpg	fold2	4	*Amblyomma*
4685	Ventral	MobileNetV2	ventral ovale (51).jpg	fold2	4	*Amblyomma*
4686	Ventral	MobileNetV2	ventral ovale (8).jpg	fold2	4	*Amblyomma*
4687	Ventral	MobileNetV2	ventral ovale (9).jpg	fold2	4	*Amblyomma*
4688	Ventral	MobileNetV2	ventral sculptum (1).jpg	fold2	5	*Amblyomma*

4689	Ventral	MobileNetV2	ventral sculptum (12).JPG	fold2	5	*Amblyomma*
4690	Ventral	MobileNetV2	ventral sculptum (23).JPG	fold2	5	*Amblyomma*
4691	Ventral	MobileNetV2	ventral sculptum (30).jpg	fold2	5	*Amblyomma*
4692	Ventral	MobileNetV2	ventral sculptum (39).JPG	fold2	5	*Amblyomma*
4693	Ventral	MobileNetV2	ventral sculptum (4).jpg	fold2	5	*Amblyomma*
4694	Ventral	MobileNetV2	ventral sculptum (46).JPG	fold2	5	*Amblyomma*
4695	Ventral	MobileNetV2	ventral sculptum (48).JPG	fold2	5	*Amblyomma*
4696	Ventral	MobileNetV2	ventral sculptum (51).JPG	fold2	5	*Amblyomma*
4697	Ventral	MobileNetV2	ventral sculptum (53).JPG	fold2	5	*Amblyomma*
4698	Ventral	MobileNetV2	ventral sculptum (58).JPG	fold2	5	*Amblyomma*
4699	Ventral	MobileNetV2	ventral sculptum (63).JPG	fold2	5	*Amblyomma*
4700	Ventral	MobileNetV2	ventral sculptum (7).JPG	fold2	5	*Amblyomma*
4701	Ventral	MobileNetV2	ventral sculptum (9).JPG	fold2	5	*Amblyomma*
4702	Ventral	MobileNetV2	ventral triste (12).jpg	fold2	6	*Amblyomma*
4703	Ventral	MobileNetV2	ventral triste (2).jpg	fold2	6	*Amblyomma*
4704	Ventral	MobileNetV2	ventral triste (21).jpg	fold2	6	*Amblyomma*
4705	Ventral	MobileNetV2	ventral triste (30).jpg	fold2	6	*Amblyomma*
4706	Ventral	MobileNetV2	ventral triste (40).jpg	fold2	6	*Amblyomma*
4707	Ventral	MobileNetV2	ventral triste (43).jpg	fold2	6	*Amblyomma*
4708	Ventral	MobileNetV2	ventral triste (45).jpg	fold2	6	*Amblyomma*
4709	Ventral	MobileNetV2	ventral triste (48).jpg	fold2	6	*Amblyomma*
4710	Ventral	MobileNetV2	ventral triste (49).jpg	fold2	6	*Amblyomma*
4711	Ventral	MobileNetV2	ventral triste (52).jpg	fold2	6	*Amblyomma*
4712	Ventral	MobileNetV2	ventral triste (53).jpg	fold2	6	*Amblyomma*
4713	Ventral	MobileNetV2	ventral triste (54).jpg	fold2	6	*Amblyomma*
4714	Ventral	MobileNetV2	ventral triste (56).jpg	fold2	6	*Amblyomma*
4715	Ventral	MobileNetV2	ventral triste (57).jpg	fold2	6	*Amblyomma*
4716	Ventral	MobileNetV2	ventral triste (8).jpg	fold2	6	*Amblyomma*
4717	Ventral	MobileNetV2	ventral aureolatum (11).jpg	fold3	1	*Amblyomma*
4718	Ventral	MobileNetV2	ventral aureolatum (17).jpg	fold3	1	*Amblyomma*
4719	Ventral	MobileNetV2	ventral aureolatum (19).jpg	fold3	1	*Amblyomma*
4720	Ventral	MobileNetV2	ventral aureolatum (36).jpg	fold3	1	*Amblyomma*
4721	Ventral	MobileNetV2	ventral aureolatum (38).jpg	fold3	1	*Amblyomma*
4722	Ventral	MobileNetV2	ventral aureolatum (39).jpg	fold3	1	*Amblyomma*
4723	Ventral	MobileNetV2	ventral aureolatum (42).jpg	fold3	1	*Amblyomma*
4724	Ventral	MobileNetV2	ventral aureolatum (5).jpg	fold3	1	*Amblyomma*
4725	Ventral	MobileNetV2	ventral aureolatum (53).jpg	fold3	1	*Amblyomma*
4726	Ventral	MobileNetV2	ventral aureolatum (7).jpg	fold3	1	*Amblyomma*
4727	Ventral	MobileNetV2	ventral aureolatum (9).jpg	fold3	1	*Amblyomma*
4728	Ventral	MobileNetV2	ventral cajennense (11).jpg	fold3	2	*Amblyomma*
4729	Ventral	MobileNetV2	ventral cajennense (12).jpg	fold3	2	*Amblyomma*
4730	Ventral	MobileNetV2	ventral cajennense (22).jpg	fold3	2	*Amblyomma*
4731	Ventral	MobileNetV2	ventral cajennense (3).jpg	fold3	2	*Amblyomma*
4732	Ventral	MobileNetV2	ventral cajennense (31).jpg	fold3	2	*Amblyomma*
4733	Ventral	MobileNetV2	ventral cajennense (35).jpg	fold3	2	*Amblyomma*
4734	Ventral	MobileNetV2	ventral cajennense (36).jpg	fold3	2	*Amblyomma*
4735	Ventral	MobileNetV2	ventral cajennense (38).jpg	fold3	2	*Amblyomma*
4736	Ventral	MobileNetV2	ventral cajennense (39).jpg	fold3	2	*Amblyomma*
4737	Ventral	MobileNetV2	ventral cajennense (45).jpg	fold3	2	*Amblyomma*
4738	Ventral	MobileNetV2	ventral cajennense (47).jpg	fold3	2	*Amblyomma*
4739	Ventral	MobileNetV2	ventral cajennense (54).jpg	fold3	2	*Amblyomma*
4740	Ventral	MobileNetV2	ventral cajennense (61).jpg	fold3	2	*Amblyomma*
4741	Ventral	MobileNetV2	ventral cajennense (69).jpg	fold3	2	*Amblyomma*
4742	Ventral	MobileNetV2	ventral cajennense (78).jpg	fold3	2	*Amblyomma*
4743	Ventral	MobileNetV2	ventral cajennense (81).jpg	fold3	2	*Amblyomma*
4744	Ventral	MobileNetV2	ventral dubitatum (11).jpg	fold3	3	*Amblyomma*
4745	Ventral	MobileNetV2	ventral dubitatum (15).jpg	fold3	3	*Amblyomma*
4746	Ventral	MobileNetV2	ventral dubitatum (2).jpg	fold3	3	*Amblyomma*
4747	Ventral	MobileNetV2	ventral dubitatum (33).jpg	fold3	3	*Amblyomma*
4748	Ventral	MobileNetV2	ventral dubitatum (34).jpg	fold3	3	*Amblyomma*
4749	Ventral	MobileNetV2	ventral dubitatum (43).jpg	fold3	3	*Amblyomma*
4750	Ventral	MobileNetV2	ventral dubitatum (5).JPG	fold3	3	*Amblyomma*
4751	Ventral	MobileNetV2	ventral dubitatum (50).jpg	fold3	3	*Amblyomma*
4752	Ventral	MobileNetV2	ventral dubitatum (52).jpg	fold3	3	*Amblyomma*
4753	Ventral	MobileNetV2	ventral dubitatum (8).jpg	fold3	3	*Amblyomma*
4754	Ventral	MobileNetV2	ventral ovale (12).jpg	fold3	4	*Amblyomma*
4755	Ventral	MobileNetV2	ventral ovale (16).jpg	fold3	4	*Amblyomma*

4756	Ventral	MobileNetV2	ventral ovale (2).jpg	fold3	4	*Amblyomma*
4757	Ventral	MobileNetV2	ventral ovale (33).jpg	fold3	4	*Amblyomma*
4758	Ventral	MobileNetV2	ventral ovale (35).jpg	fold3	4	*Amblyomma*
4759	Ventral	MobileNetV2	ventral ovale (40).jpg	fold3	4	*Amblyomma*
4760	Ventral	MobileNetV2	ventral ovale (41).jpg	fold3	4	*Amblyomma*
4761	Ventral	MobileNetV2	ventral ovale (43).jpg	fold3	4	*Amblyomma*
4762	Ventral	MobileNetV2	ventral ovale (48).jpg	fold3	4	*Amblyomma*
4763	Ventral	MobileNetV2	ventral ovale (54).jpg	fold3	4	*Amblyomma*
4764	Ventral	MobileNetV2	ventral ovale (55).jpg	fold3	4	*Amblyomma*
4765	Ventral	MobileNetV2	ventral ovale (7).jpg	fold3	4	*Amblyomma*
4766	Ventral	MobileNetV2	ventral sculptum (13).JPG	fold3	5	*Amblyomma*
4767	Ventral	MobileNetV2	ventral sculptum (17).JPG	fold3	5	*Amblyomma*
4768	Ventral	MobileNetV2	ventral sculptum (25).JPG	fold3	5	*Amblyomma*
4769	Ventral	MobileNetV2	ventral sculptum (26).JPG	fold3	5	*Amblyomma*
4770	Ventral	MobileNetV2	ventral sculptum (44).JPG	fold3	5	*Amblyomma*
4771	Ventral	MobileNetV2	ventral sculptum (47).JPG	fold3	5	*Amblyomma*
4772	Ventral	MobileNetV2	ventral sculptum (49).JPG	fold3	5	*Amblyomma*
4773	Ventral	MobileNetV2	ventral sculptum (5).JPG	fold3	5	*Amblyomma*
4774	Ventral	MobileNetV2	ventral sculptum (50).JPG	fold3	5	*Amblyomma*
4775	Ventral	MobileNetV2	ventral sculptum (62).JPG	fold3	5	*Amblyomma*
4776	Ventral	MobileNetV2	ventral sculptum (64).JPG	fold3	5	*Amblyomma*
4777	Ventral	MobileNetV2	ventral sculptum (68).JPG	fold3	5	*Amblyomma*
4778	Ventral	MobileNetV2	ventral sculptum (69).JPG	fold3	5	*Amblyomma*
4779	Ventral	MobileNetV2	ventral sculptum (71).jpg	fold3	5	*Amblyomma*
4780	Ventral	MobileNetV2	ventral sculptum (74).jpg	fold3	5	*Amblyomma*
4781	Ventral	MobileNetV2	ventral triste (1).jpg	fold3	6	*Amblyomma*
4782	Ventral	MobileNetV2	ventral triste (13).jpg	fold3	6	*Amblyomma*
4783	Ventral	MobileNetV2	ventral triste (14).jpg	fold3	6	*Amblyomma*
4784	Ventral	MobileNetV2	ventral triste (20).jpg	fold3	6	*Amblyomma*
4785	Ventral	MobileNetV2	ventral triste (22).jpg	fold3	6	*Amblyomma*
4786	Ventral	MobileNetV2	ventral triste (23).jpg	fold3	6	*Amblyomma*
4787	Ventral	MobileNetV2	ventral triste (31).jpg	fold3	6	*Amblyomma*
4788	Ventral	MobileNetV2	ventral triste (32).jpg	fold3	6	*Amblyomma*
4789	Ventral	MobileNetV2	ventral triste (35).jpg	fold3	6	*Amblyomma*
4790	Ventral	MobileNetV2	ventral triste (46).jpg	fold3	6	*Amblyomma*
4791	Ventral	MobileNetV2	ventral triste (59).jpg	fold3	6	*Amblyomma*
4792	Ventral	MobileNetV2	ventral triste (63).jpg	fold3	6	*Amblyomma*
4793	Ventral	MobileNetV2	ventral triste (69).jpg	fold3	6	*Amblyomma*
4794	Ventral	MobileNetV2	ventral triste (70).jpg	fold3	6	*Amblyomma*
4795	Ventral	MobileNetV2	ventral triste (73).jpg	fold3	6	*Amblyomma*
4796	Ventral	MobileNetV2	ventral aureolatum (1).jpg	fold4	1	*Amblyomma*
4797	Ventral	MobileNetV2	ventral aureolatum (15).jpg	fold4	1	*Amblyomma*
4798	Ventral	MobileNetV2	ventral aureolatum (35).jpg	fold4	1	*Amblyomma*
4799	Ventral	MobileNetV2	ventral aureolatum (37).jpg	fold4	1	*Amblyomma*
4800	Ventral	MobileNetV2	ventral aureolatum (41).jpg	fold4	1	*Amblyomma*
4801	Ventral	MobileNetV2	ventral aureolatum (43).jpg	fold4	1	*Amblyomma*
4802	Ventral	MobileNetV2	ventral aureolatum (46).jpg	fold4	1	*Amblyomma*
4803	Ventral	MobileNetV2	ventral aureolatum (52).jpg	fold4	1	*Amblyomma*
4804	Ventral	MobileNetV2	ventral aureolatum (54).jpg	fold4	1	*Amblyomma*
4805	Ventral	MobileNetV2	ventral aureolatum (57).jpg	fold4	1	*Amblyomma*
4806	Ventral	MobileNetV2	ventral aureolatum (6).jpg	fold4	1	*Amblyomma*
4807	Ventral	MobileNetV2	ventral aureolatum (8).jpg	fold4	1	*Amblyomma*
4808	Ventral	MobileNetV2	ventral cajennense (10).jpg	fold4	2	*Amblyomma*
4809	Ventral	MobileNetV2	ventral cajennense (17).jpg	fold4	2	*Amblyomma*
4810	Ventral	MobileNetV2	ventral cajennense (18).jpg	fold4	2	*Amblyomma*
4811	Ventral	MobileNetV2	ventral cajennense (19).jpg	fold4	2	*Amblyomma*
4812	Ventral	MobileNetV2	ventral cajennense (21).jpg	fold4	2	*Amblyomma*
4813	Ventral	MobileNetV2	ventral cajennense (27).jpg	fold4	2	*Amblyomma*
4814	Ventral	MobileNetV2	ventral cajennense (34).jpg	fold4	2	*Amblyomma*
4815	Ventral	MobileNetV2	ventral cajennense (42).jpg	fold4	2	*Amblyomma*
4816	Ventral	MobileNetV2	ventral cajennense (44).jpg	fold4	2	*Amblyomma*
4817	Ventral	MobileNetV2	ventral cajennense (46).jpg	fold4	2	*Amblyomma*
4818	Ventral	MobileNetV2	ventral cajennense (48).jpg	fold4	2	*Amblyomma*
4819	Ventral	MobileNetV2	ventral cajennense (53).jpg	fold4	2	*Amblyomma*
4820	Ventral	MobileNetV2	ventral cajennense (55).jpg	fold4	2	*Amblyomma*
4821	Ventral	MobileNetV2	ventral cajennense (6).jpg	fold4	2	*Amblyomma*
4822	Ventral	MobileNetV2	ventral cajennense (62).jpg	fold4	2	*Amblyomma*

4823	Ventral	MobileNetV2	ventral cajennense (66).jpg	fold4	2	*Amblyomma*
4824	Ventral	MobileNetV2	ventral cajennense (73).jpg	fold4	2	*Amblyomma*
4825	Ventral	MobileNetV2	ventral dubitatum (1).jpg	fold4	3	*Amblyomma*
4826	Ventral	MobileNetV2	ventral dubitatum (12).jpg	fold4	3	*Amblyomma*
4827	Ventral	MobileNetV2	ventral dubitatum (13).jpg	fold4	3	*Amblyomma*
4828	Ventral	MobileNetV2	ventral dubitatum (14).jpg	fold4	3	*Amblyomma*
4829	Ventral	MobileNetV2	ventral dubitatum (20).jpg	fold4	3	*Amblyomma*
4830	Ventral	MobileNetV2	ventral dubitatum (3).jpg	fold4	3	*Amblyomma*
4831	Ventral	MobileNetV2	ventral dubitatum (32).jpg	fold4	3	*Amblyomma*
4832	Ventral	MobileNetV2	ventral dubitatum (35).jpg	fold4	3	*Amblyomma*
4833	Ventral	MobileNetV2	ventral dubitatum (37).jpg	fold4	3	*Amblyomma*
4834	Ventral	MobileNetV2	ventral dubitatum (4).JPG	fold4	3	*Amblyomma*
4835	Ventral	MobileNetV2	ventral dubitatum (44).jpg	fold4	3	*Amblyomma*
4836	Ventral	MobileNetV2	ventral ovale (19).jpg	fold4	4	*Amblyomma*
4837	Ventral	MobileNetV2	ventral ovale (23).jpg	fold4	4	*Amblyomma*
4838	Ventral	MobileNetV2	ventral ovale (24).jpg	fold4	4	*Amblyomma*
4839	Ventral	MobileNetV2	ventral ovale (30).jpg	fold4	4	*Amblyomma*
4840	Ventral	MobileNetV2	ventral ovale (34).jpg	fold4	4	*Amblyomma*
4841	Ventral	MobileNetV2	ventral ovale (37).jpg	fold4	4	*Amblyomma*
4842	Ventral	MobileNetV2	ventral ovale (4).jpg	fold4	4	*Amblyomma*
4843	Ventral	MobileNetV2	ventral ovale (45).jpg	fold4	4	*Amblyomma*
4844	Ventral	MobileNetV2	ventral ovale (5).jpg	fold4	4	*Amblyomma*
4845	Ventral	MobileNetV2	ventral ovale (50).jpg	fold4	4	*Amblyomma*
4846	Ventral	MobileNetV2	ventral ovale (53).JPG	fold4	4	*Amblyomma*
4847	Ventral	MobileNetV2	ventral ovale (6).jpg	fold4	4	*Amblyomma*
4848	Ventral	MobileNetV2	ventral sculptum (15).JPG	fold4	5	*Amblyomma*
4849	Ventral	MobileNetV2	ventral sculptum (16).JPG	fold4	5	*Amblyomma*
4850	Ventral	MobileNetV2	ventral sculptum (21).JPG	fold4	5	*Amblyomma*
4851	Ventral	MobileNetV2	ventral sculptum (22).JPG	fold4	5	*Amblyomma*
4852	Ventral	MobileNetV2	ventral sculptum (27).JPG	fold4	5	*Amblyomma*
4853	Ventral	MobileNetV2	ventral sculptum (3).jpg	fold4	5	*Amblyomma*
4854	Ventral	MobileNetV2	ventral sculptum (33).jpg	fold4	5	*Amblyomma*
4855	Ventral	MobileNetV2	ventral sculptum (37).JPG	fold4	5	*Amblyomma*
4856	Ventral	MobileNetV2	ventral sculptum (41).JPG	fold4	5	*Amblyomma*
4857	Ventral	MobileNetV2	ventral sculptum (42).JPG	fold4	5	*Amblyomma*
4858	Ventral	MobileNetV2	ventral sculptum (55).JPG	fold4	5	*Amblyomma*
4859	Ventral	MobileNetV2	ventral sculptum (59).JPG	fold4	5	*Amblyomma*
4860	Ventral	MobileNetV2	ventral sculptum (65).JPG	fold4	5	*Amblyomma*
4861	Ventral	MobileNetV2	ventral sculptum (66).JPG	fold4	5	*Amblyomma*
4862	Ventral	MobileNetV2	ventral sculptum (8).JPG	fold4	5	*Amblyomma*
4863	Ventral	MobileNetV2	ventral triste (10).jpg	fold4	6	*Amblyomma*
4864	Ventral	MobileNetV2	ventral triste (15).jpg	fold4	6	*Amblyomma*
4865	Ventral	MobileNetV2	ventral triste (18).jpg	fold4	6	*Amblyomma*
4866	Ventral	MobileNetV2	ventral triste (19).jpg	fold4	6	*Amblyomma*
4867	Ventral	MobileNetV2	ventral triste (25).jpg	fold4	6	*Amblyomma*
4868	Ventral	MobileNetV2	ventral triste (28).jpg	fold4	6	*Amblyomma*
4869	Ventral	MobileNetV2	ventral triste (29).jpg	fold4	6	*Amblyomma*
4870	Ventral	MobileNetV2	ventral triste (34).jpg	fold4	6	*Amblyomma*
4871	Ventral	MobileNetV2	ventral triste (36).jpg	fold4	6	*Amblyomma*
4872	Ventral	MobileNetV2	ventral triste (4).jpg	fold4	6	*Amblyomma*
4873	Ventral	MobileNetV2	ventral triste (44).jpg	fold4	6	*Amblyomma*
4874	Ventral	MobileNetV2	ventral triste (5).jpg	fold4	6	*Amblyomma*
4875	Ventral	MobileNetV2	ventral triste (6).jpg	fold4	6	*Amblyomma*
4876	Ventral	MobileNetV2	ventral triste (60).jpg	fold4	6	*Amblyomma*
4877	Ventral	MobileNetV2	ventral triste (71).jpg	fold4	6	*Amblyomma*
4878	Ventral	MobileNetV2	ventral triste (74).jpg	fold4	6	*Amblyomma*
4879	Ventral	MobileNetV2	ventral aureolatum (13).jpg	fold5	1	*Amblyomma*
4880	Ventral	MobileNetV2	ventral aureolatum (14).jpg	fold5	1	*Amblyomma*
4881	Ventral	MobileNetV2	ventral aureolatum (2).jpg	fold5	1	*Amblyomma*
4882	Ventral	MobileNetV2	ventral aureolatum (23).jpg	fold5	1	*Amblyomma*
4883	Ventral	MobileNetV2	ventral aureolatum (24).jpg	fold5	1	*Amblyomma*
4884	Ventral	MobileNetV2	ventral aureolatum (25).jpg	fold5	1	*Amblyomma*
4885	Ventral	MobileNetV2	ventral aureolatum (29).jpg	fold5	1	*Amblyomma*
4886	Ventral	MobileNetV2	ventral aureolatum (30).jpg	fold5	1	*Amblyomma*
4887	Ventral	MobileNetV2	ventral aureolatum (32).jpg	fold5	1	*Amblyomma*
4888	Ventral	MobileNetV2	ventral aureolatum (45).jpg	fold5	1	*Amblyomma*
4889	Ventral	MobileNetV2	ventral aureolatum (55).jpg	fold5	1	*Amblyomma*

4890	Ventral	MobileNetV2	ventral cajennense (13).jpg	fold5	2	*Amblyomma*
4891	Ventral	MobileNetV2	ventral cajennense (2).jpg	fold5	2	*Amblyomma*
4892	Ventral	MobileNetV2	ventral cajennense (24).jpg	fold5	2	*Amblyomma*
4893	Ventral	MobileNetV2	ventral cajennense (25).jpg	fold5	2	*Amblyomma*
4894	Ventral	MobileNetV2	ventral cajennense (26).jpg	fold5	2	*Amblyomma*
4895	Ventral	MobileNetV2	ventral cajennense (32).jpg	fold5	2	*Amblyomma*
4896	Ventral	MobileNetV2	ventral cajennense (40).jpg	fold5	2	*Amblyomma*
4897	Ventral	MobileNetV2	ventral cajennense (43).jpg	fold5	2	*Amblyomma*
4898	Ventral	MobileNetV2	ventral cajennense (50).jpg	fold5	2	*Amblyomma*
4899	Ventral	MobileNetV2	ventral cajennense (58).jpg	fold5	2	*Amblyomma*
4900	Ventral	MobileNetV2	ventral cajennense (59).jpg	fold5	2	*Amblyomma*
4901	Ventral	MobileNetV2	ventral cajennense (68).jpg	fold5	2	*Amblyomma*
4902	Ventral	MobileNetV2	ventral cajennense (76).jpg	fold5	2	*Amblyomma*
4903	Ventral	MobileNetV2	ventral cajennense (77).jpg	fold5	2	*Amblyomma*
4904	Ventral	MobileNetV2	ventral cajennense (79).jpg	fold5	2	*Amblyomma*
4905	Ventral	MobileNetV2	ventral cajennense (8).jpg	fold5	2	*Amblyomma*
4906	Ventral	MobileNetV2	ventral cajennense (80).jpg	fold5	2	*Amblyomma*
4907	Ventral	MobileNetV2	ventral dubitatum (10).jpg	fold5	3	*Amblyomma*
4908	Ventral	MobileNetV2	ventral dubitatum (16).jpg	fold5	3	*Amblyomma*
4909	Ventral	MobileNetV2	ventral dubitatum (17).jpg	fold5	3	*Amblyomma*
4910	Ventral	MobileNetV2	ventral dubitatum (18).jpg	fold5	3	*Amblyomma*
4911	Ventral	MobileNetV2	ventral dubitatum (21).jpg	fold5	3	*Amblyomma*
4912	Ventral	MobileNetV2	ventral dubitatum (29).jpg	fold5	3	*Amblyomma*
4913	Ventral	MobileNetV2	ventral dubitatum (31).jpg	fold5	3	*Amblyomma*
4914	Ventral	MobileNetV2	ventral dubitatum (47).jpg	fold5	3	*Amblyomma*
4915	Ventral	MobileNetV2	ventral dubitatum (6).jpg	fold5	3	*Amblyomma*
4916	Ventral	MobileNetV2	ventral dubitatum (9).jpg	fold5	3	*Amblyomma*
4917	Ventral	MobileNetV2	ventral ovale (11).jpg	fold5	4	*Amblyomma*
4918	Ventral	MobileNetV2	ventral ovale (15).jpg	fold5	4	*Amblyomma*
4919	Ventral	MobileNetV2	ventral ovale (17).jpg	fold5	4	*Amblyomma*
4920	Ventral	MobileNetV2	ventral ovale (21).jpg	fold5	4	*Amblyomma*
4921	Ventral	MobileNetV2	ventral ovale (25).jpg	fold5	4	*Amblyomma*
4922	Ventral	MobileNetV2	ventral ovale (27).jpg	fold5	4	*Amblyomma*
4923	Ventral	MobileNetV2	ventral ovale (39).jpg	fold5	4	*Amblyomma*
4924	Ventral	MobileNetV2	ventral ovale (46).jpg	fold5	4	*Amblyomma*
4925	Ventral	MobileNetV2	ventral ovale (47).jpg	fold5	4	*Amblyomma*
4926	Ventral	MobileNetV2	ventral ovale (52).jpg	fold5	4	*Amblyomma*
4927	Ventral	MobileNetV2	ventral ovale (56).jpg	fold5	4	*Amblyomma*
4928	Ventral	MobileNetV2	ventral ovale (59).jpg	fold5	4	*Amblyomma*
4929	Ventral	MobileNetV2	ventral sculptum (11).JPG	fold5	5	*Amblyomma*
4930	Ventral	MobileNetV2	ventral sculptum (14).JPG	fold5	5	*Amblyomma*
4931	Ventral	MobileNetV2	ventral sculptum (19).JPG	fold5	5	*Amblyomma*
4932	Ventral	MobileNetV2	ventral sculptum (20).JPG	fold5	5	*Amblyomma*
4933	Ventral	MobileNetV2	ventral sculptum (24).JPG	fold5	5	*Amblyomma*
4934	Ventral	MobileNetV2	ventral sculptum (34).jpg	fold5	5	*Amblyomma*
4935	Ventral	MobileNetV2	ventral sculptum (35).JPG	fold5	5	*Amblyomma*
4936	Ventral	MobileNetV2	ventral sculptum (36).JPG	fold5	5	*Amblyomma*
4937	Ventral	MobileNetV2	ventral sculptum (52).JPG	fold5	5	*Amblyomma*
4938	Ventral	MobileNetV2	ventral sculptum (56).JPG	fold5	5	*Amblyomma*
4939	Ventral	MobileNetV2	ventral sculptum (57).JPG	fold5	5	*Amblyomma*
4940	Ventral	MobileNetV2	ventral sculptum (61).JPG	fold5	5	*Amblyomma*
4941	Ventral	MobileNetV2	ventral sculptum (70).jpg	fold5	5	*Amblyomma*
4942	Ventral	MobileNetV2	ventral sculptum (72).jpg	fold5	5	*Amblyomma*
4943	Ventral	MobileNetV2	ventral sculptum (73).jpg	fold5	5	*Amblyomma*
4944	Ventral	MobileNetV2	ventral triste (17).jpg	fold5	6	*Amblyomma*
4945	Ventral	MobileNetV2	ventral triste (24).jpg	fold5	6	*Amblyomma*
4946	Ventral	MobileNetV2	ventral triste (26).jpg	fold5	6	*Amblyomma*
4947	Ventral	MobileNetV2	ventral triste (27).jpg	fold5	6	*Amblyomma*
4948	Ventral	MobileNetV2	ventral triste (3).jpg	fold5	6	*Amblyomma*
4949	Ventral	MobileNetV2	ventral triste (50).jpg	fold5	6	*Amblyomma*
4950	Ventral	MobileNetV2	ventral triste (51).jpg	fold5	6	*Amblyomma*
4951	Ventral	MobileNetV2	ventral triste (55).jpg	fold5	6	*Amblyomma*
4952	Ventral	MobileNetV2	ventral triste (58).jpg	fold5	6	*Amblyomma*
4953	Ventral	MobileNetV2	ventral triste (61).jpg	fold5	6	*Amblyomma*
4954	Ventral	MobileNetV2	ventral triste (62).jpg	fold5	6	*Amblyomma*
4955	Ventral	MobileNetV2	ventral triste (64).jpg	fold5	6	*Amblyomma*
4956	Ventral	MobileNetV2	ventral triste (65).jpg	fold5	6	*Amblyomma*

4957	Ventral	MobileNetV2	ventral triste (7).jpg	fold5	6	*Amblyomma*
4958	Ventral	MobileNetV2	ventral triste (9).jpg	fold5	6	*Amblyomma*
4959	Low resolution	MobileNetV2	baixa aureolatum (11).jpg	fold1	1	*Amblyomma*
4960	Low resolution	MobileNetV2	baixa aureolatum (17).jpg	fold1	1	*Amblyomma*
4961	Low resolution	MobileNetV2	baixa aureolatum (23).jpg	fold1	1	*Amblyomma*
4962	Low resolution	MobileNetV2	baixa aureolatum (27).jpg	fold1	1	*Amblyomma*
4963	Low resolution	MobileNetV2	baixa aureolatum (30).jpg	fold1	1	*Amblyomma*
4964	Low resolution	MobileNetV2	baixa aureolatum (46).jpg	fold1	1	*Amblyomma*
4965	Low resolution	MobileNetV2	baixa aureolatum (47).jpg	fold1	1	*Amblyomma*
4966	Low resolution	MobileNetV2	baixa aureolatum (48).jpg	fold1	1	*Amblyomma*
4967	Low resolution	MobileNetV2	baixa aureolatum (52).jpg	fold1	1	*Amblyomma*
4968	Low resolution	MobileNetV2	baixa aureolatum (7).jpg	fold1	1	*Amblyomma*
4969	Low resolution	MobileNetV2	baixa cajennense (1).jpg	fold1	2	*Amblyomma*
4970	Low resolution	MobileNetV2	baixa cajennense (23).jpg	fold1	2	*Amblyomma*
4971	Low resolution	MobileNetV2	baixa cajennense (27).jpg	fold1	2	*Amblyomma*
4972	Low resolution	MobileNetV2	baixa cajennense (30).jpg	fold1	2	*Amblyomma*
4973	Low resolution	MobileNetV2	baixa cajennense (31).jpg	fold1	2	*Amblyomma*
4974	Low resolution	MobileNetV2	baixa cajennense (37).jpg	fold1	2	*Amblyomma*
4975	Low resolution	MobileNetV2	baixa cajennense (4).jpg	fold1	2	*Amblyomma*
4976	Low resolution	MobileNetV2	baixa cajennense (44).jpg	fold1	2	*Amblyomma*
4977	Low resolution	MobileNetV2	baixa cajennense (48).jpg	fold1	2	*Amblyomma*
4978	Low resolution	MobileNetV2	baixa cajennense (5).jpg	fold1	2	*Amblyomma*
4979	Low resolution	MobileNetV2	baixa cajennense (50).jpg	fold1	2	*Amblyomma*
4980	Low resolution	MobileNetV2	baixa cajennense (51).jpg	fold1	2	*Amblyomma*
4981	Low resolution	MobileNetV2	baixa cajennense (64).jpg	fold1	2	*Amblyomma*
4982	Low resolution	MobileNetV2	baixa cajennense (7).jpg	fold1	2	*Amblyomma*
4983	Low resolution	MobileNetV2	baixa cajennense (77).jpg	fold1	2	*Amblyomma*
4984	Low resolution	MobileNetV2	baixa cajennense (80).jpg	fold1	2	*Amblyomma*
4985	Low resolution	MobileNetV2	baixa dubitatum (10).jpg	fold1	3	*Amblyomma*
4986	Low resolution	MobileNetV2	baixa dubitatum (18).jpg	fold1	3	*Amblyomma*
4987	Low resolution	MobileNetV2	baixa dubitatum (22).jpg	fold1	3	*Amblyomma*
4988	Low resolution	MobileNetV2	baixa dubitatum (23).jpg	fold1	3	*Amblyomma*
4989	Low resolution	MobileNetV2	baixa dubitatum (27).jpg	fold1	3	*Amblyomma*
4990	Low resolution	MobileNetV2	baixa dubitatum (30).jpg	fold1	3	*Amblyomma*
4991	Low resolution	MobileNetV2	baixa dubitatum (31).jpg	fold1	3	*Amblyomma*
4992	Low resolution	MobileNetV2	baixa dubitatum (5).jpg	fold1	3	*Amblyomma*
4993	Low resolution	MobileNetV2	baixa ovale (14).jpg	fold1	4	*Amblyomma*
4994	Low resolution	MobileNetV2	baixa ovale (20).jpg	fold1	4	*Amblyomma*
4995	Low resolution	MobileNetV2	baixa ovale (22).jpg	fold1	4	*Amblyomma*
4996	Low resolution	MobileNetV2	baixa ovale (29).jpg	fold1	4	*Amblyomma*
4997	Low resolution	MobileNetV2	baixa ovale (36).jpg	fold1	4	*Amblyomma*
4998	Low resolution	MobileNetV2	baixa ovale (39).jpg	fold1	4	*Amblyomma*
4999	Low resolution	MobileNetV2	baixa ovale (43).jpg	fold1	4	*Amblyomma*
5000	Low resolution	MobileNetV2	baixa ovale (44).jpg	fold1	4	*Amblyomma*
5001	Low resolution	MobileNetV2	baixa ovale (48).jpg	fold1	4	*Amblyomma*
5002	Low resolution	MobileNetV2	baixa ovale (54).jpg	fold1	4	*Amblyomma*
5003	Low resolution	MobileNetV2	baixa ovale (9).jpg	fold1	4	*Amblyomma*
5004	Low resolution	MobileNetV2	baixa sculptum (17).jpg	fold1	5	*Amblyomma*
5005	Low resolution	MobileNetV2	baixa sculptum (5).jpg	fold1	5	*Amblyomma*
5006	Low resolution	MobileNetV2	baixa sculptum (6).jpg	fold1	5	*Amblyomma*
5007	Low resolution	MobileNetV2	baixa sculptum (8).jpg	fold1	5	*Amblyomma*
5008	Low resolution	MobileNetV2	baixa triste (12).jpg	fold1	6	*Amblyomma*
5009	Low resolution	MobileNetV2	baixa triste (15).jpg	fold1	6	*Amblyomma*
5010	Low resolution	MobileNetV2	baixa triste (16).jpg	fold1	6	*Amblyomma*
5011	Low resolution	MobileNetV2	baixa triste (2).jpg	fold1	6	*Amblyomma*
5012	Low resolution	MobileNetV2	baixa triste (23).jpg	fold1	6	*Amblyomma*
5013	Low resolution	MobileNetV2	baixa triste (32).jpg	fold1	6	*Amblyomma*
5014	Low resolution	MobileNetV2	baixa triste (33).jpg	fold1	6	*Amblyomma*
5015	Low resolution	MobileNetV2	baixa triste (38).jpg	fold1	6	*Amblyomma*
5016	Low resolution	MobileNetV2	baixa triste (42).jpg	fold1	6	*Amblyomma*
5017	Low resolution	MobileNetV2	baixa triste (47).jpg	fold1	6	*Amblyomma*
5018	Low resolution	MobileNetV2	baixa triste (48).jpg	fold1	6	*Amblyomma*
5019	Low resolution	MobileNetV2	baixa triste (57).jpg	fold1	6	*Amblyomma*
5020	Low resolution	MobileNetV2	baixa triste (63).jpg	fold1	6	*Amblyomma*
5021	Low resolution	MobileNetV2	baixa triste (64).jpg	fold1	6	*Amblyomma*
5022	Low resolution	MobileNetV2	baixa triste (7).jpg	fold1	6	*Amblyomma*
5023	Low resolution	MobileNetV2	baixa triste (71).jpg	fold1	6	*Amblyomma*

5024	Low resolution	MobileNetV2	baixa aureolatum (18).jpg	fold2	1	*Amblyomma*
5025	Low resolution	MobileNetV2	baixa aureolatum (21).jpg	fold2	1	*Amblyomma*
5026	Low resolution	MobileNetV2	baixa aureolatum (28).jpg	fold2	1	*Amblyomma*
5027	Low resolution	MobileNetV2	baixa aureolatum (29).jpg	fold2	1	*Amblyomma*
5028	Low resolution	MobileNetV2	baixa aureolatum (32).jpg	fold2	1	*Amblyomma*
5029	Low resolution	MobileNetV2	baixa aureolatum (38).jpg	fold2	1	*Amblyomma*
5030	Low resolution	MobileNetV2	baixa aureolatum (43).jpg	fold2	1	*Amblyomma*
5031	Low resolution	MobileNetV2	baixa aureolatum (44).jpg	fold2	1	*Amblyomma*
5032	Low resolution	MobileNetV2	baixa aureolatum (49).jpg	fold2	1	*Amblyomma*
5033	Low resolution	MobileNetV2	baixa aureolatum (5).jpg	fold2	1	*Amblyomma*
5034	Low resolution	MobileNetV2	baixa aureolatum (51).jpg	fold2	1	*Amblyomma*
5035	Low resolution	MobileNetV2	baixa cajennense (13).jpg	fold2	2	*Amblyomma*
5036	Low resolution	MobileNetV2	baixa cajennense (14).jpg	fold2	2	*Amblyomma*
5037	Low resolution	MobileNetV2	baixa cajennense (34).jpg	fold2	2	*Amblyomma*
5038	Low resolution	MobileNetV2	baixa cajennense (35).jpg	fold2	2	*Amblyomma*
5039	Low resolution	MobileNetV2	baixa cajennense (39).jpg	fold2	2	*Amblyomma*
5040	Low resolution	MobileNetV2	baixa cajennense (41).jpg	fold2	2	*Amblyomma*
5041	Low resolution	MobileNetV2	baixa cajennense (43).jpg	fold2	2	*Amblyomma*
5042	Low resolution	MobileNetV2	baixa cajennense (53).jpg	fold2	2	*Amblyomma*
5043	Low resolution	MobileNetV2	baixa cajennense (56).jpg	fold2	2	*Amblyomma*
5044	Low resolution	MobileNetV2	baixa cajennense (6).jpg	fold2	2	*Amblyomma*
5045	Low resolution	MobileNetV2	baixa cajennense (61).jpg	fold2	2	*Amblyomma*
5046	Low resolution	MobileNetV2	baixa cajennense (63).jpg	fold2	2	*Amblyomma*
5047	Low resolution	MobileNetV2	baixa cajennense (67).jpg	fold2	2	*Amblyomma*
5048	Low resolution	MobileNetV2	baixa cajennense (74).jpg	fold2	2	*Amblyomma*
5049	Low resolution	MobileNetV2	baixa cajennense (75).jpg	fold2	2	*Amblyomma*
5050	Low resolution	MobileNetV2	baixa cajennense (79).jpg	fold2	2	*Amblyomma*
5051	Low resolution	MobileNetV2	baixa dubitatum (26).jpg	fold2	3	*Amblyomma*
5052	Low resolution	MobileNetV2	baixa dubitatum (33).jpg	fold2	3	*Amblyomma*
5053	Low resolution	MobileNetV2	baixa dubitatum (34).jpg	fold2	3	*Amblyomma*
5054	Low resolution	MobileNetV2	baixa dubitatum (35).jpg	fold2	3	*Amblyomma*
5055	Low resolution	MobileNetV2	baixa dubitatum (37).jpg	fold2	3	*Amblyomma*
5056	Low resolution	MobileNetV2	baixa dubitatum (39).jpg	fold2	3	*Amblyomma*
5057	Low resolution	MobileNetV2	baixa dubitatum (40).jpg	fold2	3	*Amblyomma*
5058	Low resolution	MobileNetV2	baixa dubitatum (43).jpg	fold2	3	*Amblyomma*
5059	Low resolution	MobileNetV2	baixa dubitatum (7).jpg	fold2	3	*Amblyomma*
5060	Low resolution	MobileNetV2	baixa ovale (2).jpg	fold2	4	*Amblyomma*
5061	Low resolution	MobileNetV2	baixa ovale (24).jpg	fold2	4	*Amblyomma*
5062	Low resolution	MobileNetV2	baixa ovale (25).jpg	fold2	4	*Amblyomma*
5063	Low resolution	MobileNetV2	baixa ovale (26).jpg	fold2	4	*Amblyomma*
5064	Low resolution	MobileNetV2	baixa ovale (27).jpg	fold2	4	*Amblyomma*
5065	Low resolution	MobileNetV2	baixa ovale (31).jpg	fold2	4	*Amblyomma*
5066	Low resolution	MobileNetV2	baixa ovale (35).jpg	fold2	4	*Amblyomma*
5067	Low resolution	MobileNetV2	baixa ovale (41).jpg	fold2	4	*Amblyomma*
5068	Low resolution	MobileNetV2	baixa ovale (42).jpg	fold2	4	*Amblyomma*
5069	Low resolution	MobileNetV2	baixa ovale (47).jpg	fold2	4	*Amblyomma*
5070	Low resolution	MobileNetV2	baixa ovale (5).jpg	fold2	4	*Amblyomma*
5071	Low resolution	MobileNetV2	baixa sculptum (13).jpg	fold2	5	*Amblyomma*
5072	Low resolution	MobileNetV2	baixa sculptum (3).jpg	fold2	5	*Amblyomma*
5073	Low resolution	MobileNetV2	baixa sculptum (7).jpg	fold2	5	*Amblyomma*
5074	Low resolution	MobileNetV2	baixa triste (10).jpg	fold2	6	*Amblyomma*
5075	Low resolution	MobileNetV2	baixa triste (11).jpg	fold2	6	*Amblyomma*
5076	Low resolution	MobileNetV2	baixa triste (13).jpg	fold2	6	*Amblyomma*
5077	Low resolution	MobileNetV2	baixa triste (17).jpg	fold2	6	*Amblyomma*
5078	Low resolution	MobileNetV2	baixa triste (21).jpg	fold2	6	*Amblyomma*
5079	Low resolution	MobileNetV2	baixa triste (25).jpg	fold2	6	*Amblyomma*
5080	Low resolution	MobileNetV2	baixa triste (26).jpg	fold2	6	*Amblyomma*
5081	Low resolution	MobileNetV2	baixa triste (30).jpg	fold2	6	*Amblyomma*
5082	Low resolution	MobileNetV2	baixa triste (37).jpg	fold2	6	*Amblyomma*
5083	Low resolution	MobileNetV2	baixa triste (40).jpg	fold2	6	*Amblyomma*
5084	Low resolution	MobileNetV2	baixa triste (41).jpg	fold2	6	*Amblyomma*
5085	Low resolution	MobileNetV2	baixa triste (50).jpg	fold2	6	*Amblyomma*
5086	Low resolution	MobileNetV2	baixa triste (55).jpg	fold2	6	*Amblyomma*
5087	Low resolution	MobileNetV2	baixa triste (61).jpg	fold2	6	*Amblyomma*
5088	Low resolution	MobileNetV2	baixa triste (69).jpg	fold2	6	*Amblyomma*
5089	Low resolution	MobileNetV2	baixa triste (75).jpg	fold2	6	*Amblyomma*
5090	Low resolution	MobileNetV2	baixa aureolatum (1).jpg	fold3	1	*Amblyomma*

5091	Low resolution	MobileNetV2	baixa aureolatum (14).jpg	fold3	1	*Amblyomma*
5092	Low resolution	MobileNetV2	baixa aureolatum (19).jpg	fold3	1	*Amblyomma*
5093	Low resolution	MobileNetV2	baixa aureolatum (24).jpg	fold3	1	*Amblyomma*
5094	Low resolution	MobileNetV2	baixa aureolatum (33).jpg	fold3	1	*Amblyomma*
5095	Low resolution	MobileNetV2	baixa aureolatum (35).jpg	fold3	1	*Amblyomma*
5096	Low resolution	MobileNetV2	baixa aureolatum (36).jpg	fold3	1	*Amblyomma*
5097	Low resolution	MobileNetV2	baixa aureolatum (40).jpg	fold3	1	*Amblyomma*
5098	Low resolution	MobileNetV2	baixa aureolatum (53).jpg	fold3	1	*Amblyomma*
5099	Low resolution	MobileNetV2	baixa aureolatum (6).jpg	fold3	1	*Amblyomma*
5100	Low resolution	MobileNetV2	baixa cajennense (10).jpg	fold3	2	*Amblyomma*
5101	Low resolution	MobileNetV2	baixa cajennense (11).jpg	fold3	2	*Amblyomma*
5102	Low resolution	MobileNetV2	baixa cajennense (16).jpg	fold3	2	*Amblyomma*
5103	Low resolution	MobileNetV2	baixa cajennense (18).jpg	fold3	2	*Amblyomma*
5104	Low resolution	MobileNetV2	baixa cajennense (2).jpg	fold3	2	*Amblyomma*
5105	Low resolution	MobileNetV2	baixa cajennense (26).jpg	fold3	2	*Amblyomma*
5106	Low resolution	MobileNetV2	baixa cajennense (29).jpg	fold3	2	*Amblyomma*
5107	Low resolution	MobileNetV2	baixa cajennense (38).jpg	fold3	2	*Amblyomma*
5108	Low resolution	MobileNetV2	baixa cajennense (55).jpg	fold3	2	*Amblyomma*
5109	Low resolution	MobileNetV2	baixa cajennense (57).jpg	fold3	2	*Amblyomma*
5110	Low resolution	MobileNetV2	baixa cajennense (59).jpg	fold3	2	*Amblyomma*
5111	Low resolution	MobileNetV2	baixa cajennense (62).jpg	fold3	2	*Amblyomma*
5112	Low resolution	MobileNetV2	baixa cajennense (65).jpg	fold3	2	*Amblyomma*
5113	Low resolution	MobileNetV2	baixa cajennense (66).jpg	fold3	2	*Amblyomma*
5114	Low resolution	MobileNetV2	baixa cajennense (71).jpg	fold3	2	*Amblyomma*
5115	Low resolution	MobileNetV2	baixa cajennense (76).jpg	fold3	2	*Amblyomma*
5116	Low resolution	MobileNetV2	baixa cajennense (8).jpg	fold3	2	*Amblyomma*
5117	Low resolution	MobileNetV2	baixa dubitatum (15).jpg	fold3	3	*Amblyomma*
5118	Low resolution	MobileNetV2	baixa dubitatum (20).jpg	fold3	3	*Amblyomma*
5119	Low resolution	MobileNetV2	baixa dubitatum (28).jpg	fold3	3	*Amblyomma*
5120	Low resolution	MobileNetV2	baixa dubitatum (29).jpg	fold3	3	*Amblyomma*
5121	Low resolution	MobileNetV2	baixa dubitatum (32).jpg	fold3	3	*Amblyomma*
5122	Low resolution	MobileNetV2	baixa dubitatum (38).jpg	fold3	3	*Amblyomma*
5123	Low resolution	MobileNetV2	baixa dubitatum (4).jpg	fold3	3	*Amblyomma*
5124	Low resolution	MobileNetV2	baixa dubitatum (41).jpg	fold3	3	*Amblyomma*
5125	Low resolution	MobileNetV2	baixa dubitatum (42).jpg	fold3	3	*Amblyomma*
5126	Low resolution	MobileNetV2	baixa ovale (11).jpg	fold3	4	*Amblyomma*
5127	Low resolution	MobileNetV2	baixa ovale (21).jpg	fold3	4	*Amblyomma*
5128	Low resolution	MobileNetV2	baixa ovale (28).jpg	fold3	4	*Amblyomma*
5129	Low resolution	MobileNetV2	baixa ovale (30).jpg	fold3	4	*Amblyomma*
5130	Low resolution	MobileNetV2	baixa ovale (34).jpg	fold3	4	*Amblyomma*
5131	Low resolution	MobileNetV2	baixa ovale (37).jpg	fold3	4	*Amblyomma*
5132	Low resolution	MobileNetV2	baixa ovale (45).jpg	fold3	4	*Amblyomma*
5133	Low resolution	MobileNetV2	baixa ovale (46).jpg	fold3	4	*Amblyomma*
5134	Low resolution	MobileNetV2	baixa ovale (52).jpg	fold3	4	*Amblyomma*
5135	Low resolution	MobileNetV2	baixa ovale (55).jpg	fold3	4	*Amblyomma*
5136	Low resolution	MobileNetV2	baixa ovale (8).jpg	fold3	4	*Amblyomma*
5137	Low resolution	MobileNetV2	baixa sculptum (1).jpg	fold3	5	*Amblyomma*
5138	Low resolution	MobileNetV2	baixa sculptum (11).jpg	fold3	5	*Amblyomma*
5139	Low resolution	MobileNetV2	baixa sculptum (12).jpg	fold3	5	*Amblyomma*
5140	Low resolution	MobileNetV2	baixa sculptum (9).jpg	fold3	5	*Amblyomma*
5141	Low resolution	MobileNetV2	baixa triste (1).jpg	fold3	6	*Amblyomma*
5142	Low resolution	MobileNetV2	baixa triste (14).jpg	fold3	6	*Amblyomma*
5143	Low resolution	MobileNetV2	baixa triste (24).jpg	fold3	6	*Amblyomma*
5144	Low resolution	MobileNetV2	baixa triste (28).jpg	fold3	6	*Amblyomma*
5145	Low resolution	MobileNetV2	baixa triste (34).jpg	fold3	6	*Amblyomma*
5146	Low resolution	MobileNetV2	baixa triste (36).jpg	fold3	6	*Amblyomma*
5147	Low resolution	MobileNetV2	baixa triste (39).jpg	fold3	6	*Amblyomma*
5148	Low resolution	MobileNetV2	baixa triste (4).jpg	fold3	6	*Amblyomma*
5149	Low resolution	MobileNetV2	baixa triste (52).jpg	fold3	6	*Amblyomma*
5150	Low resolution	MobileNetV2	baixa triste (53).jpg	fold3	6	*Amblyomma*
5151	Low resolution	MobileNetV2	baixa triste (66).jpg	fold3	6	*Amblyomma*
5152	Low resolution	MobileNetV2	baixa triste (73).jpg	fold3	6	*Amblyomma*
5153	Low resolution	MobileNetV2	baixa triste (74).jpg	fold3	6	*Amblyomma*
5154	Low resolution	MobileNetV2	baixa triste (76).jpg	fold3	6	*Amblyomma*
5155	Low resolution	MobileNetV2	baixa triste (77).jpg	fold3	6	*Amblyomma*
5156	Low resolution	MobileNetV2	baixa aureolatum (12).jpg	fold4	1	*Amblyomma*
5157	Low resolution	MobileNetV2	baixa aureolatum (13).jpg	fold4	1	*Amblyomma*

5158	Low resolution	MobileNetV2	baixa aureolatum (15).jpg	fold4	1	*Amblyomma*
5159	Low resolution	MobileNetV2	baixa aureolatum (16).jpg	fold4	1	*Amblyomma*
5160	Low resolution	MobileNetV2	baixa aureolatum (20).jpg	fold4	1	*Amblyomma*
5161	Low resolution	MobileNetV2	baixa aureolatum (22).jpg	fold4	1	*Amblyomma*
5162	Low resolution	MobileNetV2	baixa aureolatum (25).jpg	fold4	1	*Amblyomma*
5163	Low resolution	MobileNetV2	baixa aureolatum (26).jpg	fold4	1	*Amblyomma*
5164	Low resolution	MobileNetV2	baixa aureolatum (37).jpg	fold4	1	*Amblyomma*
5165	Low resolution	MobileNetV2	baixa aureolatum (4).jpg	fold4	1	*Amblyomma*
5166	Low resolution	MobileNetV2	baixa aureolatum (8).jpg	fold4	1	*Amblyomma*
5167	Low resolution	MobileNetV2	baixa cajennense (15).jpg	fold4	2	*Amblyomma*
5168	Low resolution	MobileNetV2	baixa cajennense (17).jpg	fold4	2	*Amblyomma*
5169	Low resolution	MobileNetV2	baixa cajennense (19).jpg	fold4	2	*Amblyomma*
5170	Low resolution	MobileNetV2	baixa cajennense (20).jpg	fold4	2	*Amblyomma*
5171	Low resolution	MobileNetV2	baixa cajennense (21).jpg	fold4	2	*Amblyomma*
5172	Low resolution	MobileNetV2	baixa cajennense (22).jpg	fold4	2	*Amblyomma*
5173	Low resolution	MobileNetV2	baixa cajennense (3).jpg	fold4	2	*Amblyomma*
5174	Low resolution	MobileNetV2	baixa cajennense (42).jpg	fold4	2	*Amblyomma*
5175	Low resolution	MobileNetV2	baixa cajennense (45).jpg	fold4	2	*Amblyomma*
5176	Low resolution	MobileNetV2	baixa cajennense (47).jpg	fold4	2	*Amblyomma*
5177	Low resolution	MobileNetV2	baixa cajennense (49).jpg	fold4	2	*Amblyomma*
5178	Low resolution	MobileNetV2	baixa cajennense (54).jpg	fold4	2	*Amblyomma*
5179	Low resolution	MobileNetV2	baixa cajennense (69).jpg	fold4	2	*Amblyomma*
5180	Low resolution	MobileNetV2	baixa cajennense (72).jpg	fold4	2	*Amblyomma*
5181	Low resolution	MobileNetV2	baixa cajennense (73).jpg	fold4	2	*Amblyomma*
5182	Low resolution	MobileNetV2	baixa cajennense (81).jpg	fold4	2	*Amblyomma*
5183	Low resolution	MobileNetV2	baixa dubitatum (1).jpg	fold4	3	*Amblyomma*
5184	Low resolution	MobileNetV2	baixa dubitatum (13).jpg	fold4	3	*Amblyomma*
5185	Low resolution	MobileNetV2	baixa dubitatum (14).jpg	fold4	3	*Amblyomma*
5186	Low resolution	MobileNetV2	baixa dubitatum (17).jpg	fold4	3	*Amblyomma*
5187	Low resolution	MobileNetV2	baixa dubitatum (2).jpg	fold4	3	*Amblyomma*
5188	Low resolution	MobileNetV2	baixa dubitatum (21).jpg	fold4	3	*Amblyomma*
5189	Low resolution	MobileNetV2	baixa dubitatum (36).jpg	fold4	3	*Amblyomma*
5190	Low resolution	MobileNetV2	baixa dubitatum (8).jpg	fold4	3	*Amblyomma*
5191	Low resolution	MobileNetV2	baixa dubitatum (9).jpg	fold4	3	*Amblyomma*
5192	Low resolution	MobileNetV2	baixa ovale (10).jpg	fold4	4	*Amblyomma*
5193	Low resolution	MobileNetV2	baixa ovale (13).jpg	fold4	4	*Amblyomma*
5194	Low resolution	MobileNetV2	baixa ovale (17).jpg	fold4	4	*Amblyomma*
5195	Low resolution	MobileNetV2	baixa ovale (18).jpg	fold4	4	*Amblyomma*
5196	Low resolution	MobileNetV2	baixa ovale (19).jpg	fold4	4	*Amblyomma*
5197	Low resolution	MobileNetV2	baixa ovale (38).jpg	fold4	4	*Amblyomma*
5198	Low resolution	MobileNetV2	baixa ovale (4).jpg	fold4	4	*Amblyomma*
5199	Low resolution	MobileNetV2	baixa ovale (40).jpg	fold4	4	*Amblyomma*
5200	Low resolution	MobileNetV2	baixa ovale (51).jpg	fold4	4	*Amblyomma*
5201	Low resolution	MobileNetV2	baixa ovale (53).jpg	fold4	4	*Amblyomma*
5202	Low resolution	MobileNetV2	baixa ovale (7).jpg	fold4	4	*Amblyomma*
5203	Low resolution	MobileNetV2	baixa sculptum (15).jpg	fold4	5	*Amblyomma*
5204	Low resolution	MobileNetV2	baixa sculptum (16).jpg	fold4	5	*Amblyomma*
5205	Low resolution	MobileNetV2	baixa sculptum (2).jpg	fold4	5	*Amblyomma*
5206	Low resolution	MobileNetV2	baixa triste (18).jpg	fold4	6	*Amblyomma*
5207	Low resolution	MobileNetV2	baixa triste (19).jpg	fold4	6	*Amblyomma*
5208	Low resolution	MobileNetV2	baixa triste (45).jpg	fold4	6	*Amblyomma*
5209	Low resolution	MobileNetV2	baixa triste (46).jpg	fold4	6	*Amblyomma*
5210	Low resolution	MobileNetV2	baixa triste (5).jpg	fold4	6	*Amblyomma*
5211	Low resolution	MobileNetV2	baixa triste (51).jpg	fold4	6	*Amblyomma*
5212	Low resolution	MobileNetV2	baixa triste (54).jpg	fold4	6	*Amblyomma*
5213	Low resolution	MobileNetV2	baixa triste (6).jpg	fold4	6	*Amblyomma*
5214	Low resolution	MobileNetV2	baixa triste (60).jpg	fold4	6	*Amblyomma*
5215	Low resolution	MobileNetV2	baixa triste (62).jpg	fold4	6	*Amblyomma*
5216	Low resolution	MobileNetV2	baixa triste (67).jpg	fold4	6	*Amblyomma*
5217	Low resolution	MobileNetV2	baixa triste (68).jpg	fold4	6	*Amblyomma*
5218	Low resolution	MobileNetV2	baixa triste (70).jpg	fold4	6	*Amblyomma*
5219	Low resolution	MobileNetV2	baixa triste (72).jpg	fold4	6	*Amblyomma*
5220	Low resolution	MobileNetV2	baixa triste (8).jpg	fold4	6	*Amblyomma*
5221	Low resolution	MobileNetV2	baixa aureolatum (10).jpg	fold5	1	*Amblyomma*
5222	Low resolution	MobileNetV2	baixa aureolatum (2).jpg	fold5	1	*Amblyomma*
5223	Low resolution	MobileNetV2	baixa aureolatum (3).jpg	fold5	1	*Amblyomma*
5224	Low resolution	MobileNetV2	baixa aureolatum (31).jpg	fold5	1	*Amblyomma*

5225	Low resolution	MobileNetV2	baixa aureolatum (34).jpg	fold5	1	*Amblyomma*
5226	Low resolution	MobileNetV2	baixa aureolatum (39).jpg	fold5	1	*Amblyomma*
5227	Low resolution	MobileNetV2	baixa aureolatum (41).jpg	fold5	1	*Amblyomma*
5228	Low resolution	MobileNetV2	baixa aureolatum (42).jpg	fold5	1	*Amblyomma*
5229	Low resolution	MobileNetV2	baixa aureolatum (45).jpg	fold5	1	*Amblyomma*
5230	Low resolution	MobileNetV2	baixa aureolatum (50).jpg	fold5	1	*Amblyomma*
5231	Low resolution	MobileNetV2	baixa aureolatum (9).jpg	fold5	1	*Amblyomma*
5232	Low resolution	MobileNetV2	baixa cajennense (12).jpg	fold5	2	*Amblyomma*
5233	Low resolution	MobileNetV2	baixa cajennense (24).jpg	fold5	2	*Amblyomma*
5234	Low resolution	MobileNetV2	baixa cajennense (25).jpg	fold5	2	*Amblyomma*
5235	Low resolution	MobileNetV2	baixa cajennense (28).jpg	fold5	2	*Amblyomma*
5236	Low resolution	MobileNetV2	baixa cajennense (32).jpg	fold5	2	*Amblyomma*
5237	Low resolution	MobileNetV2	baixa cajennense (33).jpg	fold5	2	*Amblyomma*
5238	Low resolution	MobileNetV2	baixa cajennense (36).jpg	fold5	2	*Amblyomma*
5239	Low resolution	MobileNetV2	baixa cajennense (40).jpg	fold5	2	*Amblyomma*
5240	Low resolution	MobileNetV2	baixa cajennense (46).jpg	fold5	2	*Amblyomma*
5241	Low resolution	MobileNetV2	baixa cajennense (52).jpg	fold5	2	*Amblyomma*
5242	Low resolution	MobileNetV2	baixa cajennense (58).jpg	fold5	2	*Amblyomma*
5243	Low resolution	MobileNetV2	baixa cajennense (60).jpg	fold5	2	*Amblyomma*
5244	Low resolution	MobileNetV2	baixa cajennense (68).jpg	fold5	2	*Amblyomma*
5245	Low resolution	MobileNetV2	baixa cajennense (70).jpg	fold5	2	*Amblyomma*
5246	Low resolution	MobileNetV2	baixa cajennense (78).jpg	fold5	2	*Amblyomma*
5247	Low resolution	MobileNetV2	baixa cajennense (82).jpg	fold5	2	*Amblyomma*
5248	Low resolution	MobileNetV2	baixa cajennense (9).jpg	fold5	2	*Amblyomma*
5249	Low resolution	MobileNetV2	baixa dubitatum (11).jpg	fold5	3	*Amblyomma*
5250	Low resolution	MobileNetV2	baixa dubitatum (12).jpg	fold5	3	*Amblyomma*
5251	Low resolution	MobileNetV2	baixa dubitatum (16).jpg	fold5	3	*Amblyomma*
5252	Low resolution	MobileNetV2	baixa dubitatum (19).jpg	fold5	3	*Amblyomma*
5253	Low resolution	MobileNetV2	baixa dubitatum (24).jpg	fold5	3	*Amblyomma*
5254	Low resolution	MobileNetV2	baixa dubitatum (25).jpg	fold5	3	*Amblyomma*
5255	Low resolution	MobileNetV2	baixa dubitatum (3).jpg	fold5	3	*Amblyomma*
5256	Low resolution	MobileNetV2	baixa dubitatum (44).jpg	fold5	3	*Amblyomma*
5257	Low resolution	MobileNetV2	baixa dubitatum (6).jpg	fold5	3	*Amblyomma*
5258	Low resolution	MobileNetV2	baixa ovale (1).jpg	fold5	4	*Amblyomma*
5259	Low resolution	MobileNetV2	baixa ovale (12).jpg	fold5	4	*Amblyomma*
5260	Low resolution	MobileNetV2	baixa ovale (15).jpg	fold5	4	*Amblyomma*
5261	Low resolution	MobileNetV2	baixa ovale (16).jpg	fold5	4	*Amblyomma*
5262	Low resolution	MobileNetV2	baixa ovale (23).jpg	fold5	4	*Amblyomma*
5263	Low resolution	MobileNetV2	baixa ovale (3).jpg	fold5	4	*Amblyomma*
5264	Low resolution	MobileNetV2	baixa ovale (32).jpg	fold5	4	*Amblyomma*
5265	Low resolution	MobileNetV2	baixa ovale (33).jpg	fold5	4	*Amblyomma*
5266	Low resolution	MobileNetV2	baixa ovale (49).jpg	fold5	4	*Amblyomma*
5267	Low resolution	MobileNetV2	baixa ovale (50).jpg	fold5	4	*Amblyomma*
5268	Low resolution	MobileNetV2	baixa ovale (6).jpg	fold5	4	*Amblyomma*
5269	Low resolution	MobileNetV2	baixa sculptum (10).jpg	fold5	5	*Amblyomma*
5270	Low resolution	MobileNetV2	baixa sculptum (14).jpg	fold5	5	*Amblyomma*
5271	Low resolution	MobileNetV2	baixa sculptum (4).jpg	fold5	5	*Amblyomma*
5272	Low resolution	MobileNetV2	baixa triste (20).jpg	fold5	6	*Amblyomma*
5273	Low resolution	MobileNetV2	baixa triste (22).jpg	fold5	6	*Amblyomma*
5274	Low resolution	MobileNetV2	baixa triste (27).jpg	fold5	6	*Amblyomma*
5275	Low resolution	MobileNetV2	baixa triste (29).jpg	fold5	6	*Amblyomma*
5276	Low resolution	MobileNetV2	baixa triste (3).jpg	fold5	6	*Amblyomma*
5277	Low resolution	MobileNetV2	baixa triste (31).jpg	fold5	6	*Amblyomma*
5278	Low resolution	MobileNetV2	baixa triste (35).jpg	fold5	6	*Amblyomma*
5279	Low resolution	MobileNetV2	baixa triste (43).jpg	fold5	6	*Amblyomma*
5280	Low resolution	MobileNetV2	baixa triste (44).jpg	fold5	6	*Amblyomma*
5281	Low resolution	MobileNetV2	baixa triste (49).jpg	fold5	6	*Amblyomma*
5282	Low resolution	MobileNetV2	baixa triste (56).jpg	fold5	6	*Amblyomma*
5283	Low resolution	MobileNetV2	baixa triste (58).jpg	fold5	6	*Amblyomma*
5284	Low resolution	MobileNetV2	baixa triste (59).jpg	fold5	6	*Amblyomma*
5285	Low resolution	MobileNetV2	baixa triste (65).jpg	fold5	6	*Amblyomma*
5286	Low resolution	MobileNetV2	baixa triste (9).jpg	fold5	6	*Amblyomma*
5287	High resolution	MobileNetV2	alta aureolatum (11).jpg	fold1	1	*Amblyomma*
5288	High resolution	MobileNetV2	alta aureolatum (17).jpg	fold1	1	*Amblyomma*
5289	High resolution	MobileNetV2	alta aureolatum (23).jpg	fold1	1	*Amblyomma*
5290	High resolution	MobileNetV2	alta aureolatum (27).jpg	fold1	1	*Amblyomma*
5291	High resolution	MobileNetV2	alta aureolatum (30).jpg	fold1	1	*Amblyomma*

5292	High resolution	MobileNetV2	alta aureolatum (46).jpg	fold1	1	*Amblyomma*
5293	High resolution	MobileNetV2	alta aureolatum (47).jpg	fold1	1	*Amblyomma*
5294	High resolution	MobileNetV2	alta aureolatum (48).jpg	fold1	1	*Amblyomma*
5295	High resolution	MobileNetV2	alta aureolatum (52).jpg	fold1	1	*Amblyomma*
5296	High resolution	MobileNetV2	alta aureolatum (55).jpg	fold1	1	*Amblyomma*
5297	High resolution	MobileNetV2	alta aureolatum (58).jpg	fold1	1	*Amblyomma*
5298	High resolution	MobileNetV2	alta aureolatum (61).jpg	fold1	1	*Amblyomma*
5299	High resolution	MobileNetV2	alta aureolatum (65).jpg	fold1	1	*Amblyomma*
5300	High resolution	MobileNetV2	alta cajennense (11).jpg	fold1	2	*Amblyomma*
5301	High resolution	MobileNetV2	alta cajennense (16).jpg	fold1	2	*Amblyomma*
5302	High resolution	MobileNetV2	alta cajennense (29).jpg	fold1	2	*Amblyomma*
5303	High resolution	MobileNetV2	alta cajennense (3).jpg	fold1	2	*Amblyomma*
5304	High resolution	MobileNetV2	alta cajennense (31).jpg	fold1	2	*Amblyomma*
5305	High resolution	MobileNetV2	alta cajennense (33).jpg	fold1	2	*Amblyomma*
5306	High resolution	MobileNetV2	alta cajennense (35).jpg	fold1	2	*Amblyomma*
5307	High resolution	MobileNetV2	alta cajennense (40).jpg	fold1	2	*Amblyomma*
5308	High resolution	MobileNetV2	alta cajennense (42).jpg	fold1	2	*Amblyomma*
5309	High resolution	MobileNetV2	alta cajennense (55).jpg	fold1	2	*Amblyomma*
5310	High resolution	MobileNetV2	alta cajennense (59).jpg	fold1	2	*Amblyomma*
5311	High resolution	MobileNetV2	alta cajennense (6).jpg	fold1	2	*Amblyomma*
5312	High resolution	MobileNetV2	alta cajennense (68).jpg	fold1	2	*Amblyomma*
5313	High resolution	MobileNetV2	alta cajennense (71).jpg	fold1	2	*Amblyomma*
5314	High resolution	MobileNetV2	alta cajennense (73).jpg	fold1	2	*Amblyomma*
5315	High resolution	MobileNetV2	alta cajennense (74).jpg	fold1	2	*Amblyomma*
5316	High resolution	MobileNetV2	alta cajennense (80).jpg	fold1	2	*Amblyomma*
5317	High resolution	MobileNetV2	alta dubitatum (11).jpg	fold1	3	*Amblyomma*
5318	High resolution	MobileNetV2	alta dubitatum (19).jpg	fold1	3	*Amblyomma*
5319	High resolution	MobileNetV2	alta dubitatum (2).jpg	fold1	3	*Amblyomma*
5320	High resolution	MobileNetV2	alta dubitatum (20).jpg	fold1	3	*Amblyomma*
5321	High resolution	MobileNetV2	alta dubitatum (24).jpg	fold1	3	*Amblyomma*
5322	High resolution	MobileNetV2	alta dubitatum (26).jpg	fold1	3	*Amblyomma*
5323	High resolution	MobileNetV2	alta dubitatum (29).jpg	fold1	3	*Amblyomma*
5324	High resolution	MobileNetV2	alta dubitatum (3).jpg	fold1	3	*Amblyomma*
5325	High resolution	MobileNetV2	alta dubitatum (32).jpg	fold1	3	*Amblyomma*
5326	High resolution	MobileNetV2	alta dubitatum (40).jpg	fold1	3	*Amblyomma*
5327	High resolution	MobileNetV2	alta dubitatum (48).jpg	fold1	3	*Amblyomma*
5328	High resolution	MobileNetV2	alta dubitatum (49).jpg	fold1	3	*Amblyomma*
5329	High resolution	MobileNetV2	alta dubitatum (9).JPG	fold1	3	*Amblyomma*
5330	High resolution	MobileNetV2	alta ovale (12).jpg	fold1	4	*Amblyomma*
5331	High resolution	MobileNetV2	alta ovale (19).jpg	fold1	4	*Amblyomma*
5332	High resolution	MobileNetV2	alta ovale (2).jpg	fold1	4	*Amblyomma*
5333	High resolution	MobileNetV2	alta ovale (23).jpg	fold1	4	*Amblyomma*
5334	High resolution	MobileNetV2	alta ovale (24).jpg	fold1	4	*Amblyomma*
5335	High resolution	MobileNetV2	alta ovale (26).jpg	fold1	4	*Amblyomma*
5336	High resolution	MobileNetV2	alta ovale (42).jpg	fold1	4	*Amblyomma*
5337	High resolution	MobileNetV2	alta ovale (51).jpg	fold1	4	*Amblyomma*
5338	High resolution	MobileNetV2	alta ovale (52).jpg	fold1	4	*Amblyomma*
5339	High resolution	MobileNetV2	alta ovale (54).jpg	fold1	4	*Amblyomma*
5340	High resolution	MobileNetV2	alta ovale (58).JPG	fold1	4	*Amblyomma*
5341	High resolution	MobileNetV2	alta ovale (62).jpg	fold1	4	*Amblyomma*
5342	High resolution	MobileNetV2	alta ovale (66).jpg	fold1	4	*Amblyomma*
5343	High resolution	MobileNetV2	alta sculptum (100).JPG	fold1	5	*Amblyomma*
5344	High resolution	MobileNetV2	alta sculptum (109).JPG	fold1	5	*Amblyomma*
5345	High resolution	MobileNetV2	alta sculptum (11).JPG	fold1	5	*Amblyomma*
5346	High resolution	MobileNetV2	alta sculptum (110).JPG	fold1	5	*Amblyomma*
5347	High resolution	MobileNetV2	alta sculptum (114).JPG	fold1	5	*Amblyomma*
5348	High resolution	MobileNetV2	alta sculptum (121).JPG	fold1	5	*Amblyomma*
5349	High resolution	MobileNetV2	alta sculptum (123).JPG	fold1	5	*Amblyomma*
5350	High resolution	MobileNetV2	alta sculptum (124).JPG	fold1	5	*Amblyomma*
5351	High resolution	MobileNetV2	alta sculptum (130).JPG	fold1	5	*Amblyomma*
5352	High resolution	MobileNetV2	alta sculptum (19).JPG	fold1	5	*Amblyomma*
5353	High resolution	MobileNetV2	alta sculptum (2).jpg	fold1	5	*Amblyomma*
5354	High resolution	MobileNetV2	alta sculptum (21).JPG	fold1	5	*Amblyomma*
5355	High resolution	MobileNetV2	alta sculptum (22).JPG	fold1	5	*Amblyomma*
5356	High resolution	MobileNetV2	alta sculptum (26).JPG	fold1	5	*Amblyomma*
5357	High resolution	MobileNetV2	alta sculptum (3).jpg	fold1	5	*Amblyomma*
5358	High resolution	MobileNetV2	alta sculptum (36).JPG	fold1	5	*Amblyomma*

5359	High resolution	MobileNetV2	alta sculptum (49).JPG	fold1	5	*Amblyomma*
5360	High resolution	MobileNetV2	alta sculptum (54).JPG	fold1	5	*Amblyomma*
5361	High resolution	MobileNetV2	alta sculptum (57).jpg	fold1	5	*Amblyomma*
5362	High resolution	MobileNetV2	alta sculptum (58).jpg	fold1	5	*Amblyomma*
5363	High resolution	MobileNetV2	alta sculptum (6).jpg	fold1	5	*Amblyomma*
5364	High resolution	MobileNetV2	alta sculptum (65).JPG	fold1	5	*Amblyomma*
5365	High resolution	MobileNetV2	alta sculptum (66).JPG	fold1	5	*Amblyomma*
5366	High resolution	MobileNetV2	alta sculptum (75).JPG	fold1	5	*Amblyomma*
5367	High resolution	MobileNetV2	alta sculptum (79).JPG	fold1	5	*Amblyomma*
5368	High resolution	MobileNetV2	alta sculptum (80).JPG	fold1	5	*Amblyomma*
5369	High resolution	MobileNetV2	alta sculptum (94).JPG	fold1	5	*Amblyomma*
5370	High resolution	MobileNetV2	alta triste (1).jpg	fold1	6	*Amblyomma*
5371	High resolution	MobileNetV2	alta triste (14).jpg	fold1	6	*Amblyomma*
5372	High resolution	MobileNetV2	alta triste (16).jpg	fold1	6	*Amblyomma*
5373	High resolution	MobileNetV2	alta triste (27).jpg	fold1	6	*Amblyomma*
5374	High resolution	MobileNetV2	alta triste (29).jpg	fold1	6	*Amblyomma*
5375	High resolution	MobileNetV2	alta triste (4).jpg	fold1	6	*Amblyomma*
5376	High resolution	MobileNetV2	alta triste (48).jpg	fold1	6	*Amblyomma*
5377	High resolution	MobileNetV2	alta triste (51).jpg	fold1	6	*Amblyomma*
5378	High resolution	MobileNetV2	alta triste (52).jpg	fold1	6	*Amblyomma*
5379	High resolution	MobileNetV2	alta triste (57).jpg	fold1	6	*Amblyomma*
5380	High resolution	MobileNetV2	alta triste (60).jpg	fold1	6	*Amblyomma*
5381	High resolution	MobileNetV2	alta triste (69).jpg	fold1	6	*Amblyomma*
5382	High resolution	MobileNetV2	alta triste (76).jpg	fold1	6	*Amblyomma*
5383	High resolution	MobileNetV2	alta triste (78).jpg	fold1	6	*Amblyomma*
5384	High resolution	MobileNetV2	alta triste (79).jpg	fold1	6	*Amblyomma*
5385	High resolution	MobileNetV2	alta triste (8).jpg	fold1	6	*Amblyomma*
5386	High resolution	MobileNetV2	alta aureolatum (18).jpg	fold2	1	*Amblyomma*
5387	High resolution	MobileNetV2	alta aureolatum (21).jpg	fold2	1	*Amblyomma*
5388	High resolution	MobileNetV2	alta aureolatum (28).jpg	fold2	1	*Amblyomma*
5389	High resolution	MobileNetV2	alta aureolatum (29).jpg	fold2	1	*Amblyomma*
5390	High resolution	MobileNetV2	alta aureolatum (32).jpg	fold2	1	*Amblyomma*
5391	High resolution	MobileNetV2	alta aureolatum (38).jpg	fold2	1	*Amblyomma*
5392	High resolution	MobileNetV2	alta aureolatum (43).jpg	fold2	1	*Amblyomma*
5393	High resolution	MobileNetV2	alta aureolatum (44).jpg	fold2	1	*Amblyomma*
5394	High resolution	MobileNetV2	alta aureolatum (49).jpg	fold2	1	*Amblyomma*
5395	High resolution	MobileNetV2	alta aureolatum (5).jpg	fold2	1	*Amblyomma*
5396	High resolution	MobileNetV2	alta aureolatum (51).jpg	fold2	1	*Amblyomma*
5397	High resolution	MobileNetV2	alta aureolatum (6).jpg	fold2	1	*Amblyomma*
5398	High resolution	MobileNetV2	alta aureolatum (62).jpg	fold2	1	*Amblyomma*
5399	High resolution	MobileNetV2	alta aureolatum (9).jpg	fold2	1	*Amblyomma*
5400	High resolution	MobileNetV2	alta cajennense (12).jpg	fold2	2	*Amblyomma*
5401	High resolution	MobileNetV2	alta cajennense (15).jpg	fold2	2	*Amblyomma*
5402	High resolution	MobileNetV2	alta cajennense (24).jpg	fold2	2	*Amblyomma*
5403	High resolution	MobileNetV2	alta cajennense (30).jpg	fold2	2	*Amblyomma*
5404	High resolution	MobileNetV2	alta cajennense (41).jpg	fold2	2	*Amblyomma*
5405	High resolution	MobileNetV2	alta cajennense (43).jpg	fold2	2	*Amblyomma*
5406	High resolution	MobileNetV2	alta cajennense (45).jpg	fold2	2	*Amblyomma*
5407	High resolution	MobileNetV2	alta cajennense (49).jpg	fold2	2	*Amblyomma*
5408	High resolution	MobileNetV2	alta cajennense (51).jpg	fold2	2	*Amblyomma*
5409	High resolution	MobileNetV2	alta cajennense (52).jpg	fold2	2	*Amblyomma*
5410	High resolution	MobileNetV2	alta cajennense (56).jpg	fold2	2	*Amblyomma*
5411	High resolution	MobileNetV2	alta cajennense (58).jpg	fold2	2	*Amblyomma*
5412	High resolution	MobileNetV2	alta cajennense (62).jpg	fold2	2	*Amblyomma*
5413	High resolution	MobileNetV2	alta cajennense (66).jpg	fold2	2	*Amblyomma*
5414	High resolution	MobileNetV2	alta cajennense (77).jpg	fold2	2	*Amblyomma*
5415	High resolution	MobileNetV2	alta cajennense (78).jpg	fold2	2	*Amblyomma*
5416	High resolution	MobileNetV2	alta cajennense (81).jpg	fold2	2	*Amblyomma*
5417	High resolution	MobileNetV2	alta dubitatum (21).jpg	fold2	3	*Amblyomma*
5418	High resolution	MobileNetV2	alta dubitatum (22).jpg	fold2	3	*Amblyomma*
5419	High resolution	MobileNetV2	alta dubitatum (33).jpg	fold2	3	*Amblyomma*
5420	High resolution	MobileNetV2	alta dubitatum (34).jpg	fold2	3	*Amblyomma*
5421	High resolution	MobileNetV2	alta dubitatum (35).jpg	fold2	3	*Amblyomma*
5422	High resolution	MobileNetV2	alta dubitatum (37).jpg	fold2	3	*Amblyomma*
5423	High resolution	MobileNetV2	alta dubitatum (38).jpg	fold2	3	*Amblyomma*
5424	High resolution	MobileNetV2	alta dubitatum (43).jpg	fold2	3	*Amblyomma*
5425	High resolution	MobileNetV2	alta dubitatum (47).jpg	fold2	3	*Amblyomma*

5426	High resolution	MobileNetV2	alta dubitatum (51).jpg	fold2	3	*Amblyomma*
5427	High resolution	MobileNetV2	alta dubitatum (58).jpg	fold2	3	*Amblyomma*
5428	High resolution	MobileNetV2	alta dubitatum (60).jpg	fold2	3	*Amblyomma*
5429	High resolution	MobileNetV2	alta dubitatum (64).jpg	fold2	3	*Amblyomma*
5430	High resolution	MobileNetV2	alta ovale (18).jpg	fold2	4	*Amblyomma*
5431	High resolution	MobileNetV2	alta ovale (27).jpg	fold2	4	*Amblyomma*
5432	High resolution	MobileNetV2	alta ovale (28).jpg	fold2	4	*Amblyomma*
5433	High resolution	MobileNetV2	alta ovale (38).jpg	fold2	4	*Amblyomma*
5434	High resolution	MobileNetV2	alta ovale (4).jpg	fold2	4	*Amblyomma*
5435	High resolution	MobileNetV2	alta ovale (41).jpg	fold2	4	*Amblyomma*
5436	High resolution	MobileNetV2	alta ovale (43).jpg	fold2	4	*Amblyomma*
5437	High resolution	MobileNetV2	alta ovale (44).jpg	fold2	4	*Amblyomma*
5438	High resolution	MobileNetV2	alta ovale (48).jpg	fold2	4	*Amblyomma*
5439	High resolution	MobileNetV2	alta ovale (50).jpg	fold2	4	*Amblyomma*
5440	High resolution	MobileNetV2	alta ovale (55).jpg	fold2	4	*Amblyomma*
5441	High resolution	MobileNetV2	alta ovale (65).jpg	fold2	4	*Amblyomma*
5442	High resolution	MobileNetV2	alta ovale (9).jpg	fold2	4	*Amblyomma*
5443	High resolution	MobileNetV2	alta sculptum (111).JPG	fold2	5	*Amblyomma*
5444	High resolution	MobileNetV2	alta sculptum (115).JPG	fold2	5	*Amblyomma*
5445	High resolution	MobileNetV2	alta sculptum (12).JPG	fold2	5	*Amblyomma*
5446	High resolution	MobileNetV2	alta sculptum (125).JPG	fold2	5	*Amblyomma*
5447	High resolution	MobileNetV2	alta sculptum (126).JPG	fold2	5	*Amblyomma*
5448	High resolution	MobileNetV2	alta sculptum (128).JPG	fold2	5	*Amblyomma*
5449	High resolution	MobileNetV2	alta sculptum (132).JPG	fold2	5	*Amblyomma*
5450	High resolution	MobileNetV2	alta sculptum (138).JPG	fold2	5	*Amblyomma*
5451	High resolution	MobileNetV2	alta sculptum (27).JPG	fold2	5	*Amblyomma*
5452	High resolution	MobileNetV2	alta sculptum (32).JPG	fold2	5	*Amblyomma*
5453	High resolution	MobileNetV2	alta sculptum (33).JPG	fold2	5	*Amblyomma*
5454	High resolution	MobileNetV2	alta sculptum (38).JPG	fold2	5	*Amblyomma*
5455	High resolution	MobileNetV2	alta sculptum (39).JPG	fold2	5	*Amblyomma*
5456	High resolution	MobileNetV2	alta sculptum (40).JPG	fold2	5	*Amblyomma*
5457	High resolution	MobileNetV2	alta sculptum (41).JPG	fold2	5	*Amblyomma*
5458	High resolution	MobileNetV2	alta sculptum (43).JPG	fold2	5	*Amblyomma*
5459	High resolution	MobileNetV2	alta sculptum (47).JPG	fold2	5	*Amblyomma*
5460	High resolution	MobileNetV2	alta sculptum (52).JPG	fold2	5	*Amblyomma*
5461	High resolution	MobileNetV2	alta sculptum (56).jpg	fold2	5	*Amblyomma*
5462	High resolution	MobileNetV2	alta sculptum (67).JPG	fold2	5	*Amblyomma*
5463	High resolution	MobileNetV2	alta sculptum (74).JPG	fold2	5	*Amblyomma*
5464	High resolution	MobileNetV2	alta sculptum (8).jpg	fold2	5	*Amblyomma*
5465	High resolution	MobileNetV2	alta sculptum (81).JPG	fold2	5	*Amblyomma*
5466	High resolution	MobileNetV2	alta sculptum (9).JPG	fold2	5	*Amblyomma*
5467	High resolution	MobileNetV2	alta sculptum (91).JPG	fold2	5	*Amblyomma*
5468	High resolution	MobileNetV2	alta sculptum (95).JPG	fold2	5	*Amblyomma*
5469	High resolution	MobileNetV2	alta sculptum (96).JPG	fold2	5	*Amblyomma*
5470	High resolution	MobileNetV2	alta sculptum (99).JPG	fold2	5	*Amblyomma*
5471	High resolution	MobileNetV2	alta triste (2).jpg	fold2	6	*Amblyomma*
5472	High resolution	MobileNetV2	alta triste (25).jpg	fold2	6	*Amblyomma*
5473	High resolution	MobileNetV2	alta triste (33).jpg	fold2	6	*Amblyomma*
5474	High resolution	MobileNetV2	alta triste (36).jpg	fold2	6	*Amblyomma*
5475	High resolution	MobileNetV2	alta triste (37).jpg	fold2	6	*Amblyomma*
5476	High resolution	MobileNetV2	alta triste (38).jpg	fold2	6	*Amblyomma*
5477	High resolution	MobileNetV2	alta triste (39).jpg	fold2	6	*Amblyomma*
5478	High resolution	MobileNetV2	alta triste (41).jpg	fold2	6	*Amblyomma*
5479	High resolution	MobileNetV2	alta triste (47).jpg	fold2	6	*Amblyomma*
5480	High resolution	MobileNetV2	alta triste (5).jpg	fold2	6	*Amblyomma*
5481	High resolution	MobileNetV2	alta triste (54).jpg	fold2	6	*Amblyomma*
5482	High resolution	MobileNetV2	alta triste (61).jpg	fold2	6	*Amblyomma*
5483	High resolution	MobileNetV2	alta triste (63).jpg	fold2	6	*Amblyomma*
5484	High resolution	MobileNetV2	alta triste (65).jpg	fold2	6	*Amblyomma*
5485	High resolution	MobileNetV2	alta triste (67).jpg	fold2	6	*Amblyomma*
5486	High resolution	MobileNetV2	alta triste (77).jpg	fold2	6	*Amblyomma*
5487	High resolution	MobileNetV2	alta aureolatum (1).jpg	fold3	1	*Amblyomma*
5488	High resolution	MobileNetV2	alta aureolatum (14).jpg	fold3	1	*Amblyomma*
5489	High resolution	MobileNetV2	alta aureolatum (19).jpg	fold3	1	*Amblyomma*
5490	High resolution	MobileNetV2	alta aureolatum (24).jpg	fold3	1	*Amblyomma*
5491	High resolution	MobileNetV2	alta aureolatum (31).jpg	fold3	1	*Amblyomma*
5492	High resolution	MobileNetV2	alta aureolatum (33).jpg	fold3	1	*Amblyomma*

5493	High resolution	MobileNetV2	alta aureolatum (35).jpg	fold3	1	*Amblyomma*
5494	High resolution	MobileNetV2	alta aureolatum (36).jpg	fold3	1	*Amblyomma*
5495	High resolution	MobileNetV2	alta aureolatum (40).jpg	fold3	1	*Amblyomma*
5496	High resolution	MobileNetV2	alta aureolatum (53).jpg	fold3	1	*Amblyomma*
5497	High resolution	MobileNetV2	alta aureolatum (54).jpg	fold3	1	*Amblyomma*
5498	High resolution	MobileNetV2	alta aureolatum (66).jpg	fold3	1	*Amblyomma*
5499	High resolution	MobileNetV2	alta aureolatum (67).jpg	fold3	1	*Amblyomma*
5500	High resolution	MobileNetV2	alta cajennense (1).jpg	fold3	2	*Amblyomma*
5501	High resolution	MobileNetV2	alta cajennense (13).jpg	fold3	2	*Amblyomma*
5502	High resolution	MobileNetV2	alta cajennense (14).jpg	fold3	2	*Amblyomma*
5503	High resolution	MobileNetV2	alta cajennense (17).jpg	fold3	2	*Amblyomma*
5504	High resolution	MobileNetV2	alta cajennense (18).jpg	fold3	2	*Amblyomma*
5505	High resolution	MobileNetV2	alta cajennense (2).jpg	fold3	2	*Amblyomma*
5506	High resolution	MobileNetV2	alta cajennense (23).jpg	fold3	2	*Amblyomma*
5507	High resolution	MobileNetV2	alta cajennense (26).jpg	fold3	2	*Amblyomma*
5508	High resolution	MobileNetV2	alta cajennense (34).jpg	fold3	2	*Amblyomma*
5509	High resolution	MobileNetV2	alta cajennense (36).jpg	fold3	2	*Amblyomma*
5510	High resolution	MobileNetV2	alta cajennense (37).jpg	fold3	2	*Amblyomma*
5511	High resolution	MobileNetV2	alta cajennense (38).jpg	fold3	2	*Amblyomma*
5512	High resolution	MobileNetV2	alta cajennense (50).jpg	fold3	2	*Amblyomma*
5513	High resolution	MobileNetV2	alta cajennense (63).jpg	fold3	2	*Amblyomma*
5514	High resolution	MobileNetV2	alta cajennense (64).jpg	fold3	2	*Amblyomma*
5515	High resolution	MobileNetV2	alta cajennense (67).jpg	fold3	2	*Amblyomma*
5516	High resolution	MobileNetV2	alta cajennense (9).jpg	fold3	2	*Amblyomma*
5517	High resolution	MobileNetV2	alta dubitatum (1).jpg	fold3	3	*Amblyomma*
5518	High resolution	MobileNetV2	alta dubitatum (10).JPG	fold3	3	*Amblyomma*
5519	High resolution	MobileNetV2	alta dubitatum (15).jpg	fold3	3	*Amblyomma*
5520	High resolution	MobileNetV2	alta dubitatum (31).jpg	fold3	3	*Amblyomma*
5521	High resolution	MobileNetV2	alta dubitatum (39).jpg	fold3	3	*Amblyomma*
5522	High resolution	MobileNetV2	alta dubitatum (4).jpg	fold3	3	*Amblyomma*
5523	High resolution	MobileNetV2	alta dubitatum (42).jpg	fold3	3	*Amblyomma*
5524	High resolution	MobileNetV2	alta dubitatum (45).jpg	fold3	3	*Amblyomma*
5525	High resolution	MobileNetV2	alta dubitatum (46).jpg	fold3	3	*Amblyomma*
5526	High resolution	MobileNetV2	alta dubitatum (53).jpg	fold3	3	*Amblyomma*
5527	High resolution	MobileNetV2	alta dubitatum (6).jpg	fold3	3	*Amblyomma*
5528	High resolution	MobileNetV2	alta dubitatum (62).jpg	fold3	3	*Amblyomma*
5529	High resolution	MobileNetV2	alta dubitatum (63).jpg	fold3	3	*Amblyomma*
5530	High resolution	MobileNetV2	alta ovale (15).jpg	fold3	4	*Amblyomma*
5531	High resolution	MobileNetV2	alta ovale (17).jpg	fold3	4	*Amblyomma*
5532	High resolution	MobileNetV2	alta ovale (22).jpg	fold3	4	*Amblyomma*
5533	High resolution	MobileNetV2	alta ovale (30).jpg	fold3	4	*Amblyomma*
5534	High resolution	MobileNetV2	alta ovale (32).jpg	fold3	4	*Amblyomma*
5535	High resolution	MobileNetV2	alta ovale (33).jpg	fold3	4	*Amblyomma*
5536	High resolution	MobileNetV2	alta ovale (34).jpg	fold3	4	*Amblyomma*
5537	High resolution	MobileNetV2	alta ovale (45).jpg	fold3	4	*Amblyomma*
5538	High resolution	MobileNetV2	alta ovale (46).jpg	fold3	4	*Amblyomma*
5539	High resolution	MobileNetV2	alta ovale (53).jpg	fold3	4	*Amblyomma*
5540	High resolution	MobileNetV2	alta ovale (56).jpg	fold3	4	*Amblyomma*
5541	High resolution	MobileNetV2	alta ovale (57).JPG	fold3	4	*Amblyomma*
5542	High resolution	MobileNetV2	alta ovale (60).jpg	fold3	4	*Amblyomma*
5543	High resolution	MobileNetV2	alta ovale (64).jpg	fold3	4	*Amblyomma*
5544	High resolution	MobileNetV2	alta sculptum (10).JPG	fold3	5	*Amblyomma*
5545	High resolution	MobileNetV2	alta sculptum (101).JPG	fold3	5	*Amblyomma*
5546	High resolution	MobileNetV2	alta sculptum (104).JPG	fold3	5	*Amblyomma*
5547	High resolution	MobileNetV2	alta sculptum (118).JPG	fold3	5	*Amblyomma*
5548	High resolution	MobileNetV2	alta sculptum (119).JPG	fold3	5	*Amblyomma*
5549	High resolution	MobileNetV2	alta sculptum (14).JPG	fold3	5	*Amblyomma*
5550	High resolution	MobileNetV2	alta sculptum (15).JPG	fold3	5	*Amblyomma*
5551	High resolution	MobileNetV2	alta sculptum (17).JPG	fold3	5	*Amblyomma*
5552	High resolution	MobileNetV2	alta sculptum (18).JPG	fold3	5	*Amblyomma*
5553	High resolution	MobileNetV2	alta sculptum (25).JPG	fold3	5	*Amblyomma*
5554	High resolution	MobileNetV2	alta sculptum (31).JPG	fold3	5	*Amblyomma*
5555	High resolution	MobileNetV2	alta sculptum (37).JPG	fold3	5	*Amblyomma*
5556	High resolution	MobileNetV2	alta sculptum (42).JPG	fold3	5	*Amblyomma*
5557	High resolution	MobileNetV2	alta sculptum (46).JPG	fold3	5	*Amblyomma*
5558	High resolution	MobileNetV2	alta sculptum (48).JPG	fold3	5	*Amblyomma*
5559	High resolution	MobileNetV2	alta sculptum (53).JPG	fold3	5	*Amblyomma*

5560	High resolution	MobileNetV2	alta sculptum (60).jpg	fold3	5	*Amblyomma*
5561	High resolution	MobileNetV2	alta sculptum (62).jpg	fold3	5	*Amblyomma*
5562	High resolution	MobileNetV2	alta sculptum (63).jpg	fold3	5	*Amblyomma*
5563	High resolution	MobileNetV2	alta sculptum (64).jpg	fold3	5	*Amblyomma*
5564	High resolution	MobileNetV2	alta sculptum (69).JPG	fold3	5	*Amblyomma*
5565	High resolution	MobileNetV2	alta sculptum (71).JPG	fold3	5	*Amblyomma*
5566	High resolution	MobileNetV2	alta sculptum (76).JPG	fold3	5	*Amblyomma*
5567	High resolution	MobileNetV2	alta sculptum (88).JPG	fold3	5	*Amblyomma*
5568	High resolution	MobileNetV2	alta sculptum (90).JPG	fold3	5	*Amblyomma*
5569	High resolution	MobileNetV2	alta sculptum (92).JPG	fold3	5	*Amblyomma*
5570	High resolution	MobileNetV2	alta sculptum (93).JPG	fold3	5	*Amblyomma*
5571	High resolution	MobileNetV2	alta sculptum (97).JPG	fold3	5	*Amblyomma*
5572	High resolution	MobileNetV2	alta triste (10).jpg	fold3	6	*Amblyomma*
5573	High resolution	MobileNetV2	alta triste (18).jpg	fold3	6	*Amblyomma*
5574	High resolution	MobileNetV2	alta triste (19).jpg	fold3	6	*Amblyomma*
5575	High resolution	MobileNetV2	alta triste (21).jpg	fold3	6	*Amblyomma*
5576	High resolution	MobileNetV2	alta triste (24).jpg	fold3	6	*Amblyomma*
5577	High resolution	MobileNetV2	alta triste (3).jpg	fold3	6	*Amblyomma*
5578	High resolution	MobileNetV2	alta triste (31).jpg	fold3	6	*Amblyomma*
5579	High resolution	MobileNetV2	alta triste (40).jpg	fold3	6	*Amblyomma*
5580	High resolution	MobileNetV2	alta triste (43).jpg	fold3	6	*Amblyomma*
5581	High resolution	MobileNetV2	alta triste (6).jpg	fold3	6	*Amblyomma*
5582	High resolution	MobileNetV2	alta triste (7).jpg	fold3	6	*Amblyomma*
5583	High resolution	MobileNetV2	alta triste (70).jpg	fold3	6	*Amblyomma*
5584	High resolution	MobileNetV2	alta triste (71).jpg	fold3	6	*Amblyomma*
5585	High resolution	MobileNetV2	alta triste (72).jpg	fold3	6	*Amblyomma*
5586	High resolution	MobileNetV2	alta triste (9).jpg	fold3	6	*Amblyomma*
5587	High resolution	MobileNetV2	alta aureolatum (12).jpg	fold4	1	*Amblyomma*
5588	High resolution	MobileNetV2	alta aureolatum (13).jpg	fold4	1	*Amblyomma*
5589	High resolution	MobileNetV2	alta aureolatum (15).jpg	fold4	1	*Amblyomma*
5590	High resolution	MobileNetV2	alta aureolatum (16).jpg	fold4	1	*Amblyomma*
5591	High resolution	MobileNetV2	alta aureolatum (20).jpg	fold4	1	*Amblyomma*
5592	High resolution	MobileNetV2	alta aureolatum (22).jpg	fold4	1	*Amblyomma*
5593	High resolution	MobileNetV2	alta aureolatum (25).jpg	fold4	1	*Amblyomma*
5594	High resolution	MobileNetV2	alta aureolatum (26).jpg	fold4	1	*Amblyomma*
5595	High resolution	MobileNetV2	alta aureolatum (37).jpg	fold4	1	*Amblyomma*
5596	High resolution	MobileNetV2	alta aureolatum (4).jpg	fold4	1	*Amblyomma*
5597	High resolution	MobileNetV2	alta aureolatum (56).jpg	fold4	1	*Amblyomma*
5598	High resolution	MobileNetV2	alta aureolatum (63).jpg	fold4	1	*Amblyomma*
5599	High resolution	MobileNetV2	alta aureolatum (64).jpg	fold4	1	*Amblyomma*
5600	High resolution	MobileNetV2	alta aureolatum (7).jpg	fold4	1	*Amblyomma*
5601	High resolution	MobileNetV2	alta cajennense (20).jpg	fold4	2	*Amblyomma*
5602	High resolution	MobileNetV2	alta cajennense (21).jpg	fold4	2	*Amblyomma*
5603	High resolution	MobileNetV2	alta cajennense (25).jpg	fold4	2	*Amblyomma*
5604	High resolution	MobileNetV2	alta cajennense (28).jpg	fold4	2	*Amblyomma*
5605	High resolution	MobileNetV2	alta cajennense (4).jpg	fold4	2	*Amblyomma*
5606	High resolution	MobileNetV2	alta cajennense (46).jpg	fold4	2	*Amblyomma*
5607	High resolution	MobileNetV2	alta cajennense (48).jpg	fold4	2	*Amblyomma*
5608	High resolution	MobileNetV2	alta cajennense (5).jpg	fold4	2	*Amblyomma*
5609	High resolution	MobileNetV2	alta cajennense (53).jpg	fold4	2	*Amblyomma*
5610	High resolution	MobileNetV2	alta cajennense (60).jpg	fold4	2	*Amblyomma*
5611	High resolution	MobileNetV2	alta cajennense (61).jpg	fold4	2	*Amblyomma*
5612	High resolution	MobileNetV2	alta cajennense (65).jpg	fold4	2	*Amblyomma*
5613	High resolution	MobileNetV2	alta cajennense (70).jpg	fold4	2	*Amblyomma*
5614	High resolution	MobileNetV2	alta cajennense (72).jpg	fold4	2	*Amblyomma*
5615	High resolution	MobileNetV2	alta cajennense (76).jpg	fold4	2	*Amblyomma*
5616	High resolution	MobileNetV2	alta cajennense (83).jpg	fold4	2	*Amblyomma*
5617	High resolution	MobileNetV2	alta dubitatum (12).jpg	fold4	3	*Amblyomma*
5618	High resolution	MobileNetV2	alta dubitatum (16).jpg	fold4	3	*Amblyomma*
5619	High resolution	MobileNetV2	alta dubitatum (17).jpg	fold4	3	*Amblyomma*
5620	High resolution	MobileNetV2	alta dubitatum (18).jpg	fold4	3	*Amblyomma*
5621	High resolution	MobileNetV2	alta dubitatum (25).jpg	fold4	3	*Amblyomma*
5622	High resolution	MobileNetV2	alta dubitatum (27).jpg	fold4	3	*Amblyomma*
5623	High resolution	MobileNetV2	alta dubitatum (30).jpg	fold4	3	*Amblyomma*
5624	High resolution	MobileNetV2	alta dubitatum (5).jpg	fold4	3	*Amblyomma*
5625	High resolution	MobileNetV2	alta dubitatum (54).jpg	fold4	3	*Amblyomma*
5626	High resolution	MobileNetV2	alta dubitatum (55).jpg	fold4	3	*Amblyomma*

5627	High resolution	MobileNetV2	alta dubitatum (56).jpg	fold4	3	*Amblyomma*
5628	High resolution	MobileNetV2	alta dubitatum (61).jpg	fold4	3	*Amblyomma*
5629	High resolution	MobileNetV2	alta dubitatum (65).jpg	fold4	3	*Amblyomma*
5630	High resolution	MobileNetV2	alta ovale (1).jpg	fold4	4	*Amblyomma*
5631	High resolution	MobileNetV2	alta ovale (10).jpg	fold4	4	*Amblyomma*
5632	High resolution	MobileNetV2	alta ovale (11).jpg	fold4	4	*Amblyomma*
5633	High resolution	MobileNetV2	alta ovale (14).jpg	fold4	4	*Amblyomma*
5634	High resolution	MobileNetV2	alta ovale (20).jpg	fold4	4	*Amblyomma*
5635	High resolution	MobileNetV2	alta ovale (21).jpg	fold4	4	*Amblyomma*
5636	High resolution	MobileNetV2	alta ovale (29).jpg	fold4	4	*Amblyomma*
5637	High resolution	MobileNetV2	alta ovale (3).jpg	fold4	4	*Amblyomma*
5638	High resolution	MobileNetV2	alta ovale (36).jpg	fold4	4	*Amblyomma*
5639	High resolution	MobileNetV2	alta ovale (37).jpg	fold4	4	*Amblyomma*
5640	High resolution	MobileNetV2	alta ovale (47).jpg	fold4	4	*Amblyomma*
5641	High resolution	MobileNetV2	alta ovale (6).jpg	fold4	4	*Amblyomma*
5642	High resolution	MobileNetV2	alta ovale (7).jpg	fold4	4	*Amblyomma*
5643	High resolution	MobileNetV2	alta sculptum (102).JPG	fold4	5	*Amblyomma*
5644	High resolution	MobileNetV2	alta sculptum (106).JPG	fold4	5	*Amblyomma*
5645	High resolution	MobileNetV2	alta sculptum (107).JPG	fold4	5	*Amblyomma*
5646	High resolution	MobileNetV2	alta sculptum (116).JPG	fold4	5	*Amblyomma*
5647	High resolution	MobileNetV2	alta sculptum (117).JPG	fold4	5	*Amblyomma*
5648	High resolution	MobileNetV2	alta sculptum (120).JPG	fold4	5	*Amblyomma*
5649	High resolution	MobileNetV2	alta sculptum (127).JPG	fold4	5	*Amblyomma*
5650	High resolution	MobileNetV2	alta sculptum (133).JPG	fold4	5	*Amblyomma*
5651	High resolution	MobileNetV2	alta sculptum (134).JPG	fold4	5	*Amblyomma*
5652	High resolution	MobileNetV2	alta sculptum (136).JPG	fold4	5	*Amblyomma*
5653	High resolution	MobileNetV2	alta sculptum (16).JPG	fold4	5	*Amblyomma*
5654	High resolution	MobileNetV2	alta sculptum (23).JPG	fold4	5	*Amblyomma*
5655	High resolution	MobileNetV2	alta sculptum (28).JPG	fold4	5	*Amblyomma*
5656	High resolution	MobileNetV2	alta sculptum (34).JPG	fold4	5	*Amblyomma*
5657	High resolution	MobileNetV2	alta sculptum (35).JPG	fold4	5	*Amblyomma*
5658	High resolution	MobileNetV2	alta sculptum (4).jpg	fold4	5	*Amblyomma*
5659	High resolution	MobileNetV2	alta sculptum (55).JPG	fold4	5	*Amblyomma*
5660	High resolution	MobileNetV2	alta sculptum (59).jpg	fold4	5	*Amblyomma*
5661	High resolution	MobileNetV2	alta sculptum (61).jpg	fold4	5	*Amblyomma*
5662	High resolution	MobileNetV2	alta sculptum (68).JPG	fold4	5	*Amblyomma*
5663	High resolution	MobileNetV2	alta sculptum (7).jpg	fold4	5	*Amblyomma*
5664	High resolution	MobileNetV2	alta sculptum (73).JPG	fold4	5	*Amblyomma*
5665	High resolution	MobileNetV2	alta sculptum (78).JPG	fold4	5	*Amblyomma*
5666	High resolution	MobileNetV2	alta sculptum (82).JPG	fold4	5	*Amblyomma*
5667	High resolution	MobileNetV2	alta sculptum (83).JPG	fold4	5	*Amblyomma*
5668	High resolution	MobileNetV2	alta sculptum (84).JPG	fold4	5	*Amblyomma*
5669	High resolution	MobileNetV2	alta sculptum (86).JPG	fold4	5	*Amblyomma*
5670	High resolution	MobileNetV2	alta triste (11).jpg	fold4	6	*Amblyomma*
5671	High resolution	MobileNetV2	alta triste (13).jpg	fold4	6	*Amblyomma*
5672	High resolution	MobileNetV2	alta triste (17).jpg	fold4	6	*Amblyomma*
5673	High resolution	MobileNetV2	alta triste (20).jpg	fold4	6	*Amblyomma*
5674	High resolution	MobileNetV2	alta triste (23).jpg	fold4	6	*Amblyomma*
5675	High resolution	MobileNetV2	alta triste (32).jpg	fold4	6	*Amblyomma*
5676	High resolution	MobileNetV2	alta triste (34).jpg	fold4	6	*Amblyomma*
5677	High resolution	MobileNetV2	alta triste (44).jpg	fold4	6	*Amblyomma*
5678	High resolution	MobileNetV2	alta triste (45).jpg	fold4	6	*Amblyomma*
5679	High resolution	MobileNetV2	alta triste (46).jpg	fold4	6	*Amblyomma*
5680	High resolution	MobileNetV2	alta triste (55).jpg	fold4	6	*Amblyomma*
5681	High resolution	MobileNetV2	alta triste (56).jpg	fold4	6	*Amblyomma*
5682	High resolution	MobileNetV2	alta triste (62).jpg	fold4	6	*Amblyomma*
5683	High resolution	MobileNetV2	alta triste (64).jpg	fold4	6	*Amblyomma*
5684	High resolution	MobileNetV2	alta triste (66).jpg	fold4	6	*Amblyomma*
5685	High resolution	MobileNetV2	alta triste (68).jpg	fold4	6	*Amblyomma*
5686	High resolution	MobileNetV2	alta aureolatum (10).jpg	fold5	1	*Amblyomma*
5687	High resolution	MobileNetV2	alta aureolatum (2).jpg	fold5	1	*Amblyomma*
5688	High resolution	MobileNetV2	alta aureolatum (3).jpg	fold5	1	*Amblyomma*
5689	High resolution	MobileNetV2	alta aureolatum (34).jpg	fold5	1	*Amblyomma*
5690	High resolution	MobileNetV2	alta aureolatum (39).jpg	fold5	1	*Amblyomma*
5691	High resolution	MobileNetV2	alta aureolatum (41).jpg	fold5	1	*Amblyomma*
5692	High resolution	MobileNetV2	alta aureolatum (42).jpg	fold5	1	*Amblyomma*
5693	High resolution	MobileNetV2	alta aureolatum (45).jpg	fold5	1	*Amblyomma*

5694	High resolution	MobileNetV2	alta aureolatum (50).jpg	fold5	1	*Amblyomma*
5695	High resolution	MobileNetV2	alta aureolatum (57).jpg	fold5	1	*Amblyomma*
5696	High resolution	MobileNetV2	alta aureolatum (59).jpg	fold5	1	*Amblyomma*
5697	High resolution	MobileNetV2	alta aureolatum (60).jpg	fold5	1	*Amblyomma*
5698	High resolution	MobileNetV2	alta aureolatum (68).jpg	fold5	1	*Amblyomma*
5699	High resolution	MobileNetV2	alta aureolatum (8).jpg	fold5	1	*Amblyomma*
5700	High resolution	MobileNetV2	alta cajennense (10).jpg	fold5	2	*Amblyomma*
5701	High resolution	MobileNetV2	alta cajennense (19).jpg	fold5	2	*Amblyomma*
5702	High resolution	MobileNetV2	alta cajennense (22).jpg	fold5	2	*Amblyomma*
5703	High resolution	MobileNetV2	alta cajennense (27).jpg	fold5	2	*Amblyomma*
5704	High resolution	MobileNetV2	alta cajennense (32).jpg	fold5	2	*Amblyomma*
5705	High resolution	MobileNetV2	alta cajennense (39).jpg	fold5	2	*Amblyomma*
5706	High resolution	MobileNetV2	alta cajennense (44).jpg	fold5	2	*Amblyomma*
5707	High resolution	MobileNetV2	alta cajennense (47).jpg	fold5	2	*Amblyomma*
5708	High resolution	MobileNetV2	alta cajennense (54).jpg	fold5	2	*Amblyomma*
5709	High resolution	MobileNetV2	alta cajennense (57).jpg	fold5	2	*Amblyomma*
5710	High resolution	MobileNetV2	alta cajennense (69).jpg	fold5	2	*Amblyomma*
5711	High resolution	MobileNetV2	alta cajennense (7).jpg	fold5	2	*Amblyomma*
5712	High resolution	MobileNetV2	alta cajennense (75).jpg	fold5	2	*Amblyomma*
5713	High resolution	MobileNetV2	alta cajennense (79).jpg	fold5	2	*Amblyomma*
5714	High resolution	MobileNetV2	alta cajennense (8).jpg	fold5	2	*Amblyomma*
5715	High resolution	MobileNetV2	alta cajennense (82).jpg	fold5	2	*Amblyomma*
5716	High resolution	MobileNetV2	alta dubitatum (13).jpg	fold5	3	*Amblyomma*
5717	High resolution	MobileNetV2	alta dubitatum (14).jpg	fold5	3	*Amblyomma*
5718	High resolution	MobileNetV2	alta dubitatum (23).jpg	fold5	3	*Amblyomma*
5719	High resolution	MobileNetV2	alta dubitatum (28).jpg	fold5	3	*Amblyomma*
5720	High resolution	MobileNetV2	alta dubitatum (36).jpg	fold5	3	*Amblyomma*
5721	High resolution	MobileNetV2	alta dubitatum (41).jpg	fold5	3	*Amblyomma*
5722	High resolution	MobileNetV2	alta dubitatum (44).jpg	fold5	3	*Amblyomma*
5723	High resolution	MobileNetV2	alta dubitatum (50).jpg	fold5	3	*Amblyomma*
5724	High resolution	MobileNetV2	alta dubitatum (52).jpg	fold5	3	*Amblyomma*
5725	High resolution	MobileNetV2	alta dubitatum (57).jpg	fold5	3	*Amblyomma*
5726	High resolution	MobileNetV2	alta dubitatum (59).jpg	fold5	3	*Amblyomma*
5727	High resolution	MobileNetV2	alta dubitatum (7).JPG	fold5	3	*Amblyomma*
5728	High resolution	MobileNetV2	alta dubitatum (8).JPG	fold5	3	*Amblyomma*
5729	High resolution	MobileNetV2	alta ovale (13).jpg	fold5	4	*Amblyomma*
5730	High resolution	MobileNetV2	alta ovale (16).jpg	fold5	4	*Amblyomma*
5731	High resolution	MobileNetV2	alta ovale (25).jpg	fold5	4	*Amblyomma*
5732	High resolution	MobileNetV2	alta ovale (31).jpg	fold5	4	*Amblyomma*
5733	High resolution	MobileNetV2	alta ovale (35).jpg	fold5	4	*Amblyomma*
5734	High resolution	MobileNetV2	alta ovale (39).JPG	fold5	4	*Amblyomma*
5735	High resolution	MobileNetV2	alta ovale (40).JPG	fold5	4	*Amblyomma*
5736	High resolution	MobileNetV2	alta ovale (49).jpg	fold5	4	*Amblyomma*
5737	High resolution	MobileNetV2	alta ovale (5).jpg	fold5	4	*Amblyomma*
5738	High resolution	MobileNetV2	alta ovale (59).jpg	fold5	4	*Amblyomma*
5739	High resolution	MobileNetV2	alta ovale (61).jpg	fold5	4	*Amblyomma*
5740	High resolution	MobileNetV2	alta ovale (63).jpg	fold5	4	*Amblyomma*
5741	High resolution	MobileNetV2	alta ovale (8).jpg	fold5	4	*Amblyomma*
5742	High resolution	MobileNetV2	alta sculptum (1).jpg	fold5	5	*Amblyomma*
5743	High resolution	MobileNetV2	alta sculptum (103).JPG	fold5	5	*Amblyomma*
5744	High resolution	MobileNetV2	alta sculptum (105).JPG	fold5	5	*Amblyomma*
5745	High resolution	MobileNetV2	alta sculptum (108).JPG	fold5	5	*Amblyomma*
5746	High resolution	MobileNetV2	alta sculptum (112).JPG	fold5	5	*Amblyomma*
5747	High resolution	MobileNetV2	alta sculptum (113).JPG	fold5	5	*Amblyomma*
5748	High resolution	MobileNetV2	alta sculptum (122).JPG	fold5	5	*Amblyomma*
5749	High resolution	MobileNetV2	alta sculptum (129).JPG	fold5	5	*Amblyomma*
5750	High resolution	MobileNetV2	alta sculptum (13).JPG	fold5	5	*Amblyomma*
5751	High resolution	MobileNetV2	alta sculptum (131).JPG	fold5	5	*Amblyomma*
5752	High resolution	MobileNetV2	alta sculptum (135).JPG	fold5	5	*Amblyomma*
5753	High resolution	MobileNetV2	alta sculptum (137).JPG	fold5	5	*Amblyomma*
5754	High resolution	MobileNetV2	alta sculptum (20).JPG	fold5	5	*Amblyomma*
5755	High resolution	MobileNetV2	alta sculptum (24).JPG	fold5	5	*Amblyomma*
5756	High resolution	MobileNetV2	alta sculptum (29).JPG	fold5	5	*Amblyomma*
5757	High resolution	MobileNetV2	alta sculptum (30).JPG	fold5	5	*Amblyomma*
5758	High resolution	MobileNetV2	alta sculptum (44).JPG	fold5	5	*Amblyomma*
5759	High resolution	MobileNetV2	alta sculptum (45).JPG	fold5	5	*Amblyomma*
5760	High resolution	MobileNetV2	alta sculptum (5).jpg	fold5	5	*Amblyomma*

5761	High resolution	MobileNetV2	alta sculptum (50).JPG	fold5	5	*Amblyomma*
5762	High resolution	MobileNetV2	alta sculptum (51).JPG	fold5	5	*Amblyomma*
5763	High resolution	MobileNetV2	alta sculptum (70).JPG	fold5	5	*Amblyomma*
5764	High resolution	MobileNetV2	alta sculptum (72).JPG	fold5	5	*Amblyomma*
5765	High resolution	MobileNetV2	alta sculptum (77).JPG	fold5	5	*Amblyomma*
5766	High resolution	MobileNetV2	alta sculptum (85).JPG	fold5	5	*Amblyomma*
5767	High resolution	MobileNetV2	alta sculptum (87).JPG	fold5	5	*Amblyomma*
5768	High resolution	MobileNetV2	alta sculptum (89).JPG	fold5	5	*Amblyomma*
5769	High resolution	MobileNetV2	alta sculptum (98).JPG	fold5	5	*Amblyomma*
5770	High resolution	MobileNetV2	alta triste (12).jpg	fold5	6	*Amblyomma*
5771	High resolution	MobileNetV2	alta triste (15).jpg	fold5	6	*Amblyomma*
5772	High resolution	MobileNetV2	alta triste (22).jpg	fold5	6	*Amblyomma*
5773	High resolution	MobileNetV2	alta triste (26).jpg	fold5	6	*Amblyomma*
5774	High resolution	MobileNetV2	alta triste (28).jpg	fold5	6	*Amblyomma*
5775	High resolution	MobileNetV2	alta triste (30).jpg	fold5	6	*Amblyomma*
5776	High resolution	MobileNetV2	alta triste (35).jpg	fold5	6	*Amblyomma*
5777	High resolution	MobileNetV2	alta triste (42).jpg	fold5	6	*Amblyomma*
5778	High resolution	MobileNetV2	alta triste (49).jpg	fold5	6	*Amblyomma*
5779	High resolution	MobileNetV2	alta triste (50).jpg	fold5	6	*Amblyomma*
5780	High resolution	MobileNetV2	alta triste (53).jpg	fold5	6	*Amblyomma*
5781	High resolution	MobileNetV2	alta triste (58).jpg	fold5	6	*Amblyomma*
5782	High resolution	MobileNetV2	alta triste (59).jpg	fold5	6	*Amblyomma*
5783	High resolution	MobileNetV2	alta triste (73).jpg	fold5	6	*Amblyomma*
5784	High resolution	MobileNetV2	alta triste (74).jpg	fold5	6	*Amblyomma*
5785	High resolution	MobileNetV2	alta triste (75).jpg	fold5	6	*Amblyomma*
5786	All together	MobileNetV2	todos aureolatum (100).jpg	fold1	1	*Amblyomma*
5787	All together	MobileNetV2	todos aureolatum (106).jpg	fold1	1	*Amblyomma*
5788	All together	MobileNetV2	todos aureolatum (21).jpg	fold1	1	*Amblyomma*
5789	All together	MobileNetV2	todos aureolatum (26).jpg	fold1	1	*Amblyomma*
5790	All together	MobileNetV2	todos aureolatum (27).jpg	fold1	1	*Amblyomma*
5791	All together	MobileNetV2	todos aureolatum (28).jpg	fold1	1	*Amblyomma*
5792	All together	MobileNetV2	todos aureolatum (35).jpg	fold1	1	*Amblyomma*
5793	All together	MobileNetV2	todos aureolatum (38).jpg	fold1	1	*Amblyomma*
5794	All together	MobileNetV2	todos aureolatum (41).jpg	fold1	1	*Amblyomma*
5795	All together	MobileNetV2	todos aureolatum (45).jpg	fold1	1	*Amblyomma*
5796	All together	MobileNetV2	todos aureolatum (56).jpg	fold1	1	*Amblyomma*
5797	All together	MobileNetV2	todos aureolatum (57).jpg	fold1	1	*Amblyomma*
5798	All together	MobileNetV2	todos aureolatum (6).jpg	fold1	1	*Amblyomma*
5799	All together	MobileNetV2	todos aureolatum (60).jpg	fold1	1	*Amblyomma*
5800	All together	MobileNetV2	todos aureolatum (66).jpg	fold1	1	*Amblyomma*
5801	All together	MobileNetV2	todos aureolatum (69).jpg	fold1	1	*Amblyomma*
5802	All together	MobileNetV2	todos aureolatum (72).jpg	fold1	1	*Amblyomma*
5803	All together	MobileNetV2	todos aureolatum (73).jpg	fold1	1	*Amblyomma*
5804	All together	MobileNetV2	todos aureolatum (79).jpg	fold1	1	*Amblyomma*
5805	All together	MobileNetV2	todos aureolatum (80).jpg	fold1	1	*Amblyomma*
5806	All together	MobileNetV2	todos aureolatum (82).jpg	fold1	1	*Amblyomma*
5807	All together	MobileNetV2	todos aureolatum (85).jpg	fold1	1	*Amblyomma*
5808	All together	MobileNetV2	todos aureolatum (90).jpg	fold1	1	*Amblyomma*
5809	All together	MobileNetV2	todos aureolatum (93).jpg	fold1	1	*Amblyomma*
5810	All together	MobileNetV2	todos cajennense (10).jpg	fold1	2	*Amblyomma*
5811	All together	MobileNetV2	todos cajennense (101).jpg	fold1	2	*Amblyomma*
5812	All together	MobileNetV2	todos cajennense (102).jpg	fold1	2	*Amblyomma*
5813	All together	MobileNetV2	todos cajennense (106).jpg	fold1	2	*Amblyomma*
5814	All together	MobileNetV2	todos cajennense (111).jpg	fold1	2	*Amblyomma*
5815	All together	MobileNetV2	todos cajennense (113).jpg	fold1	2	*Amblyomma*
5816	All together	MobileNetV2	todos cajennense (114).jpg	fold1	2	*Amblyomma*
5817	All together	MobileNetV2	todos cajennense (117).jpg	fold1	2	*Amblyomma*
5818	All together	MobileNetV2	todos cajennense (124).jpg	fold1	2	*Amblyomma*
5819	All together	MobileNetV2	todos cajennense (129).jpg	fold1	2	*Amblyomma*
5820	All together	MobileNetV2	todos cajennense (13).jpg	fold1	2	*Amblyomma*
5821	All together	MobileNetV2	todos cajennense (133).jpg	fold1	2	*Amblyomma*
5822	All together	MobileNetV2	todos cajennense (137).jpg	fold1	2	*Amblyomma*
5823	All together	MobileNetV2	todos cajennense (138).jpg	fold1	2	*Amblyomma*
5824	All together	MobileNetV2	todos cajennense (139).jpg	fold1	2	*Amblyomma*
5825	All together	MobileNetV2	todos cajennense (146).jpg	fold1	2	*Amblyomma*
5826	All together	MobileNetV2	todos cajennense (148).jpg	fold1	2	*Amblyomma*
5827	All together	MobileNetV2	todos cajennense (151).jpg	fold1	2	*Amblyomma*

5828	All together	MobileNetV2	todos cajennense (26).jpg	fold1	2	*Amblyomma*
5829	All together	MobileNetV2	todos cajennense (28).jpg	fold1	2	*Amblyomma*
5830	All together	MobileNetV2	todos cajennense (33).jpg	fold1	2	*Amblyomma*
5831	All together	MobileNetV2	todos cajennense (37).jpg	fold1	2	*Amblyomma*
5832	All together	MobileNetV2	todos cajennense (44).jpg	fold1	2	*Amblyomma*
5833	All together	MobileNetV2	todos cajennense (49).jpg	fold1	2	*Amblyomma*
5834	All together	MobileNetV2	todos cajennense (51).jpg	fold1	2	*Amblyomma*
5835	All together	MobileNetV2	todos cajennense (67).jpg	fold1	2	*Amblyomma*
5836	All together	MobileNetV2	todos cajennense (70).jpg	fold1	2	*Amblyomma*
5837	All together	MobileNetV2	todos cajennense (74).jpg	fold1	2	*Amblyomma*
5838	All together	MobileNetV2	todos cajennense (76).jpg	fold1	2	*Amblyomma*
5839	All together	MobileNetV2	todos cajennense (85).jpg	fold1	2	*Amblyomma*
5840	All together	MobileNetV2	todos cajennense (94).jpg	fold1	2	*Amblyomma*
5841	All together	MobileNetV2	todos cajennense (97).jpg	fold1	2	*Amblyomma*
5842	All together	MobileNetV2	todos cajennense (99).jpg	fold1	2	*Amblyomma*
5843	All together	MobileNetV2	todos dubitatum (103).jpg	fold1	3	*Amblyomma*
5844	All together	MobileNetV2	todos dubitatum (104).jpg	fold1	3	*Amblyomma*
5845	All together	MobileNetV2	todos dubitatum (109).jpg	fold1	3	*Amblyomma*
5846	All together	MobileNetV2	todos dubitatum (12).JPG	fold1	3	*Amblyomma*
5847	All together	MobileNetV2	todos dubitatum (15).jpg	fold1	3	*Amblyomma*
5848	All together	MobileNetV2	todos dubitatum (2).jpg	fold1	3	*Amblyomma*
5849	All together	MobileNetV2	todos dubitatum (20).jpg	fold1	3	*Amblyomma*
5850	All together	MobileNetV2	todos dubitatum (3).jpg	fold1	3	*Amblyomma*
5851	All together	MobileNetV2	todos dubitatum (36).jpg	fold1	3	*Amblyomma*
5852	All together	MobileNetV2	todos dubitatum (37).jpg	fold1	3	*Amblyomma*
5853	All together	MobileNetV2	todos dubitatum (39).jpg	fold1	3	*Amblyomma*
5854	All together	MobileNetV2	todos dubitatum (42).jpg	fold1	3	*Amblyomma*
5855	All together	MobileNetV2	todos dubitatum (44).jpg	fold1	3	*Amblyomma*
5856	All together	MobileNetV2	todos dubitatum (52).jpg	fold1	3	*Amblyomma*
5857	All together	MobileNetV2	todos dubitatum (56).jpg	fold1	3	*Amblyomma*
5858	All together	MobileNetV2	todos dubitatum (60).jpg	fold1	3	*Amblyomma*
5859	All together	MobileNetV2	todos dubitatum (62).jpg	fold1	3	*Amblyomma*
5860	All together	MobileNetV2	todos dubitatum (76).jpg	fold1	3	*Amblyomma*
5861	All together	MobileNetV2	todos dubitatum (77).jpg	fold1	3	*Amblyomma*
5862	All together	MobileNetV2	todos dubitatum (81).jpg	fold1	3	*Amblyomma*
5863	All together	MobileNetV2	todos dubitatum (82).jpg	fold1	3	*Amblyomma*
5864	All together	MobileNetV2	todos dubitatum (83).jpg	fold1	3	*Amblyomma*
5865	All together	MobileNetV2	todos ovale (100).jpg	fold1	4	*Amblyomma*
5866	All together	MobileNetV2	todos ovale (104).jpg	fold1	4	*Amblyomma*
5867	All together	MobileNetV2	todos ovale (106).JPG	fold1	4	*Amblyomma*
5868	All together	MobileNetV2	todos ovale (108).jpg	fold1	4	*Amblyomma*
5869	All together	MobileNetV2	todos ovale (109).jpg	fold1	4	*Amblyomma*
5870	All together	MobileNetV2	todos ovale (116).jpg	fold1	4	*Amblyomma*
5871	All together	MobileNetV2	todos ovale (118).jpg	fold1	4	*Amblyomma*
5872	All together	MobileNetV2	todos ovale (121).jpg	fold1	4	*Amblyomma*
5873	All together	MobileNetV2	todos ovale (16).jpg	fold1	4	*Amblyomma*
5874	All together	MobileNetV2	todos ovale (20).jpg	fold1	4	*Amblyomma*
5875	All together	MobileNetV2	todos ovale (24).jpg	fold1	4	*Amblyomma*
5876	All together	MobileNetV2	todos ovale (27).jpg	fold1	4	*Amblyomma*
5877	All together	MobileNetV2	todos ovale (34).jpg	fold1	4	*Amblyomma*
5878	All together	MobileNetV2	todos ovale (38).jpg	fold1	4	*Amblyomma*
5879	All together	MobileNetV2	todos ovale (4).jpg	fold1	4	*Amblyomma*
5880	All together	MobileNetV2	todos ovale (42).jpg	fold1	4	*Amblyomma*
5881	All together	MobileNetV2	todos ovale (47).jpg	fold1	4	*Amblyomma*
5882	All together	MobileNetV2	todos ovale (60).jpg	fold1	4	*Amblyomma*
5883	All together	MobileNetV2	todos ovale (62).jpg	fold1	4	*Amblyomma*
5884	All together	MobileNetV2	todos ovale (65).jpg	fold1	4	*Amblyomma*
5885	All together	MobileNetV2	todos ovale (7).jpg	fold1	4	*Amblyomma*
5886	All together	MobileNetV2	todos ovale (70).jpg	fold1	4	*Amblyomma*
5887	All together	MobileNetV2	todos ovale (85).jpg	fold1	4	*Amblyomma*
5888	All together	MobileNetV2	todos ovale (87).jpg	fold1	4	*Amblyomma*
5889	All together	MobileNetV2	todos sculptum (100).JPG	fold1	5	*Amblyomma*
5890	All together	MobileNetV2	todos sculptum (103).JPG	fold1	5	*Amblyomma*
5891	All together	MobileNetV2	todos sculptum (106).JPG	fold1	5	*Amblyomma*
5892	All together	MobileNetV2	todos sculptum (108).JPG	fold1	5	*Amblyomma*
5893	All together	MobileNetV2	todos sculptum (112).JPG	fold1	5	*Amblyomma*
5894	All together	MobileNetV2	todos sculptum (113).JPG	fold1	5	*Amblyomma*

5895	All together	MobileNetV2	todos sculptum (114).JPG	fold1	5	*Amblyomma*
5896	All together	MobileNetV2	todos sculptum (115).JPG	fold1	5	*Amblyomma*
5897	All together	MobileNetV2	todos sculptum (119).JPG	fold1	5	*Amblyomma*
5898	All together	MobileNetV2	todos sculptum (120).JPG	fold1	5	*Amblyomma*
5899	All together	MobileNetV2	todos sculptum (123).JPG	fold1	5	*Amblyomma*
5900	All together	MobileNetV2	todos sculptum (124).JPG	fold1	5	*Amblyomma*
5901	All together	MobileNetV2	todos sculptum (127).JPG	fold1	5	*Amblyomma*
5902	All together	MobileNetV2	todos sculptum (138).JPG	fold1	5	*Amblyomma*
5903	All together	MobileNetV2	todos sculptum (139).JPG	fold1	5	*Amblyomma*
5904	All together	MobileNetV2	todos sculptum (142).JPG	fold1	5	*Amblyomma*
5905	All together	MobileNetV2	todos sculptum (26).JPG	fold1	5	*Amblyomma*
5906	All together	MobileNetV2	todos sculptum (32).JPG	fold1	5	*Amblyomma*
5907	All together	MobileNetV2	todos sculptum (48).JPG	fold1	5	*Amblyomma*
5908	All together	MobileNetV2	todos sculptum (52).JPG	fold1	5	*Amblyomma*
5909	All together	MobileNetV2	todos sculptum (55).JPG	fold1	5	*Amblyomma*
5910	All together	MobileNetV2	todos sculptum (57).jpg	fold1	5	*Amblyomma*
5911	All together	MobileNetV2	todos sculptum (61).jpg	fold1	5	*Amblyomma*
5912	All together	MobileNetV2	todos sculptum (70).jpg	fold1	5	*Amblyomma*
5913	All together	MobileNetV2	todos sculptum (71).JPG	fold1	5	*Amblyomma*
5914	All together	MobileNetV2	todos sculptum (74).JPG	fold1	5	*Amblyomma*
5915	All together	MobileNetV2	todos sculptum (75).JPG	fold1	5	*Amblyomma*
5916	All together	MobileNetV2	todos sculptum (93).JPG	fold1	5	*Amblyomma*
5917	All together	MobileNetV2	todos sculptum (97).JPG	fold1	5	*Amblyomma*
5918	All together	MobileNetV2	todos sculptum (98).JPG	fold1	5	*Amblyomma*
5919	All together	MobileNetV2	todos sculptum (99).JPG	fold1	5	*Amblyomma*
5920	All together	MobileNetV2	todos triste (102).jpg	fold1	6	*Amblyomma*
5921	All together	MobileNetV2	todos triste (104).jpg	fold1	6	*Amblyomma*
5922	All together	MobileNetV2	todos triste (110).jpg	fold1	6	*Amblyomma*
5923	All together	MobileNetV2	todos triste (116).jpg	fold1	6	*Amblyomma*
5924	All together	MobileNetV2	todos triste (120).jpg	fold1	6	*Amblyomma*
5925	All together	MobileNetV2	todos triste (121).jpg	fold1	6	*Amblyomma*
5926	All together	MobileNetV2	todos triste (13).jpg	fold1	6	*Amblyomma*
5927	All together	MobileNetV2	todos triste (131).jpg	fold1	6	*Amblyomma*
5928	All together	MobileNetV2	todos triste (132).jpg	fold1	6	*Amblyomma*
5929	All together	MobileNetV2	todos triste (133).jpg	fold1	6	*Amblyomma*
5930	All together	MobileNetV2	todos triste (136).jpg	fold1	6	*Amblyomma*
5931	All together	MobileNetV2	todos triste (140).jpg	fold1	6	*Amblyomma*
5932	All together	MobileNetV2	todos triste (142).jpg	fold1	6	*Amblyomma*
5933	All together	MobileNetV2	todos triste (146).jpg	fold1	6	*Amblyomma*
5934	All together	MobileNetV2	todos triste (147).jpg	fold1	6	*Amblyomma*
5935	All together	MobileNetV2	todos triste (149).jpg	fold1	6	*Amblyomma*
5936	All together	MobileNetV2	todos triste (2).jpg	fold1	6	*Amblyomma*
5937	All together	MobileNetV2	todos triste (29).jpg	fold1	6	*Amblyomma*
5938	All together	MobileNetV2	todos triste (38).jpg	fold1	6	*Amblyomma*
5939	All together	MobileNetV2	todos triste (49).jpg	fold1	6	*Amblyomma*
5940	All together	MobileNetV2	todos triste (53).jpg	fold1	6	*Amblyomma*
5941	All together	MobileNetV2	todos triste (58).jpg	fold1	6	*Amblyomma*
5942	All together	MobileNetV2	todos triste (68).jpg	fold1	6	*Amblyomma*
5943	All together	MobileNetV2	todos triste (70).jpg	fold1	6	*Amblyomma*
5944	All together	MobileNetV2	todos triste (73).jpg	fold1	6	*Amblyomma*
5945	All together	MobileNetV2	todos triste (76).jpg	fold1	6	*Amblyomma*
5946	All together	MobileNetV2	todos triste (79).jpg	fold1	6	*Amblyomma*
5947	All together	MobileNetV2	todos triste (8).jpg	fold1	6	*Amblyomma*
5948	All together	MobileNetV2	todos triste (86).jpg	fold1	6	*Amblyomma*
5949	All together	MobileNetV2	todos triste (95).jpg	fold1	6	*Amblyomma*
5950	All together	MobileNetV2	todos triste (96).jpg	fold1	6	*Amblyomma*
5951	All together	MobileNetV2	todos aureolatum (107).jpg	fold2	1	*Amblyomma*
5952	All together	MobileNetV2	todos aureolatum (110).jpg	fold2	1	*Amblyomma*
5953	All together	MobileNetV2	todos aureolatum (112).jpg	fold2	1	*Amblyomma*
5954	All together	MobileNetV2	todos aureolatum (116).jpg	fold2	1	*Amblyomma*
5955	All together	MobileNetV2	todos aureolatum (117).jpg	fold2	1	*Amblyomma*
5956	All together	MobileNetV2	todos aureolatum (118).jpg	fold2	1	*Amblyomma*
5957	All together	MobileNetV2	todos aureolatum (12).jpg	fold2	1	*Amblyomma*
5958	All together	MobileNetV2	todos aureolatum (121).jpg	fold2	1	*Amblyomma*
5959	All together	MobileNetV2	todos aureolatum (18).jpg	fold2	1	*Amblyomma*
5960	All together	MobileNetV2	todos aureolatum (23).jpg	fold2	1	*Amblyomma*
5961	All together	MobileNetV2	todos aureolatum (24).jpg	fold2	1	*Amblyomma*

5962	All together	MobileNetV2	todos aureolatum (29).jpg	fold2	1	*Amblyomma*
5963	All together	MobileNetV2	todos aureolatum (3).jpg	fold2	1	*Amblyomma*
5964	All together	MobileNetV2	todos aureolatum (31).jpg	fold2	1	*Amblyomma*
5965	All together	MobileNetV2	todos aureolatum (32).jpg	fold2	1	*Amblyomma*
5966	All together	MobileNetV2	todos aureolatum (4).jpg	fold2	1	*Amblyomma*
5967	All together	MobileNetV2	todos aureolatum (42).jpg	fold2	1	*Amblyomma*
5968	All together	MobileNetV2	todos aureolatum (63).jpg	fold2	1	*Amblyomma*
5969	All together	MobileNetV2	todos aureolatum (64).jpg	fold2	1	*Amblyomma*
5970	All together	MobileNetV2	todos aureolatum (7).jpg	fold2	1	*Amblyomma*
5971	All together	MobileNetV2	todos aureolatum (77).jpg	fold2	1	*Amblyomma*
5972	All together	MobileNetV2	todos aureolatum (83).jpg	fold2	1	*Amblyomma*
5973	All together	MobileNetV2	todos aureolatum (84).jpg	fold2	1	*Amblyomma*
5974	All together	MobileNetV2	todos aureolatum (97).jpg	fold2	1	*Amblyomma*
5975	All together	MobileNetV2	todos cajennense (11).jpg	fold2	2	*Amblyomma*
5976	All together	MobileNetV2	todos cajennense (110).jpg	fold2	2	*Amblyomma*
5977	All together	MobileNetV2	todos cajennense (116).jpg	fold2	2	*Amblyomma*
5978	All together	MobileNetV2	todos cajennense (12).jpg	fold2	2	*Amblyomma*
5979	All together	MobileNetV2	todos cajennense (120).jpg	fold2	2	*Amblyomma*
5980	All together	MobileNetV2	todos cajennense (126).jpg	fold2	2	*Amblyomma*
5981	All together	MobileNetV2	todos cajennense (128).jpg	fold2	2	*Amblyomma*
5982	All together	MobileNetV2	todos cajennense (142).jpg	fold2	2	*Amblyomma*
5983	All together	MobileNetV2	todos cajennense (143).jpg	fold2	2	*Amblyomma*
5984	All together	MobileNetV2	todos cajennense (154).jpg	fold2	2	*Amblyomma*
5985	All together	MobileNetV2	todos cajennense (155).jpg	fold2	2	*Amblyomma*
5986	All together	MobileNetV2	todos cajennense (156).jpg	fold2	2	*Amblyomma*
5987	All together	MobileNetV2	todos cajennense (158).jpg	fold2	2	*Amblyomma*
5988	All together	MobileNetV2	todos cajennense (159).jpg	fold2	2	*Amblyomma*
5989	All together	MobileNetV2	todos cajennense (162).jpg	fold2	2	*Amblyomma*
5990	All together	MobileNetV2	todos cajennense (164).jpg	fold2	2	*Amblyomma*
5991	All together	MobileNetV2	todos cajennense (23).jpg	fold2	2	*Amblyomma*
5992	All together	MobileNetV2	todos cajennense (3).jpg	fold2	2	*Amblyomma*
5993	All together	MobileNetV2	todos cajennense (32).jpg	fold2	2	*Amblyomma*
5994	All together	MobileNetV2	todos cajennense (36).jpg	fold2	2	*Amblyomma*
5995	All together	MobileNetV2	todos cajennense (39).jpg	fold2	2	*Amblyomma*
5996	All together	MobileNetV2	todos cajennense (47).jpg	fold2	2	*Amblyomma*
5997	All together	MobileNetV2	todos cajennense (48).jpg	fold2	2	*Amblyomma*
5998	All together	MobileNetV2	todos cajennense (54).jpg	fold2	2	*Amblyomma*
5999	All together	MobileNetV2	todos cajennense (57).jpg	fold2	2	*Amblyomma*
6000	All together	MobileNetV2	todos cajennense (59).jpg	fold2	2	*Amblyomma*
6001	All together	MobileNetV2	todos cajennense (6).jpg	fold2	2	*Amblyomma*
6002	All together	MobileNetV2	todos cajennense (61).jpg	fold2	2	*Amblyomma*
6003	All together	MobileNetV2	todos cajennense (64).jpg	fold2	2	*Amblyomma*
6004	All together	MobileNetV2	todos cajennense (78).jpg	fold2	2	*Amblyomma*
6005	All together	MobileNetV2	todos cajennense (87).jpg	fold2	2	*Amblyomma*
6006	All together	MobileNetV2	todos cajennense (88).jpg	fold2	2	*Amblyomma*
6007	All together	MobileNetV2	todos cajennense (98).jpg	fold2	2	*Amblyomma*
6008	All together	MobileNetV2	todos dubitatum (1).jpg	fold2	3	*Amblyomma*
6009	All together	MobileNetV2	todos dubitatum (105).jpg	fold2	3	*Amblyomma*
6010	All together	MobileNetV2	todos dubitatum (107).jpg	fold2	3	*Amblyomma*
6011	All together	MobileNetV2	todos dubitatum (11).JPG	fold2	3	*Amblyomma*
6012	All together	MobileNetV2	todos dubitatum (25).jpg	fold2	3	*Amblyomma*
6013	All together	MobileNetV2	todos dubitatum (26).jpg	fold2	3	*Amblyomma*
6014	All together	MobileNetV2	todos dubitatum (46).jpg	fold2	3	*Amblyomma*
6015	All together	MobileNetV2	todos dubitatum (47).jpg	fold2	3	*Amblyomma*
6016	All together	MobileNetV2	todos dubitatum (49).jpg	fold2	3	*Amblyomma*
6017	All together	MobileNetV2	todos dubitatum (5).jpg	fold2	3	*Amblyomma*
6018	All together	MobileNetV2	todos dubitatum (57).jpg	fold2	3	*Amblyomma*
6019	All together	MobileNetV2	todos dubitatum (63).jpg	fold2	3	*Amblyomma*
6020	All together	MobileNetV2	todos dubitatum (69).jpg	fold2	3	*Amblyomma*
6021	All together	MobileNetV2	todos dubitatum (71).jpg	fold2	3	*Amblyomma*
6022	All together	MobileNetV2	todos dubitatum (74).jpg	fold2	3	*Amblyomma*
6023	All together	MobileNetV2	todos dubitatum (78).jpg	fold2	3	*Amblyomma*
6024	All together	MobileNetV2	todos dubitatum (8).jpg	fold2	3	*Amblyomma*
6025	All together	MobileNetV2	todos dubitatum (85).jpg	fold2	3	*Amblyomma*
6026	All together	MobileNetV2	todos dubitatum (92).jpg	fold2	3	*Amblyomma*
6027	All together	MobileNetV2	todos dubitatum (94).jpg	fold2	3	*Amblyomma*
6028	All together	MobileNetV2	todos dubitatum (95).jpg	fold2	3	*Amblyomma*

6029	All together	MobileNetV2	todos dubitatum (96).jpg	fold2	3	*Amblyomma*
6030	All together	MobileNetV2	todos ovale (10).jpg	fold2	4	*Amblyomma*
6031	All together	MobileNetV2	todos ovale (11).jpg	fold2	4	*Amblyomma*
6032	All together	MobileNetV2	todos ovale (110).jpg	fold2	4	*Amblyomma*
6033	All together	MobileNetV2	todos ovale (112).jpg	fold2	4	*Amblyomma*
6034	All together	MobileNetV2	todos ovale (2).jpg	fold2	4	*Amblyomma*
6035	All together	MobileNetV2	todos ovale (23).jpg	fold2	4	*Amblyomma*
6036	All together	MobileNetV2	todos ovale (25).jpg	fold2	4	*Amblyomma*
6037	All together	MobileNetV2	todos ovale (29).jpg	fold2	4	*Amblyomma*
6038	All together	MobileNetV2	todos ovale (32).jpg	fold2	4	*Amblyomma*
6039	All together	MobileNetV2	todos ovale (44).jpg	fold2	4	*Amblyomma*
6040	All together	MobileNetV2	todos ovale (46).jpg	fold2	4	*Amblyomma*
6041	All together	MobileNetV2	todos ovale (56).jpg	fold2	4	*Amblyomma*
6042	All together	MobileNetV2	todos ovale (57).jpg	fold2	4	*Amblyomma*
6043	All together	MobileNetV2	todos ovale (58).jpg	fold2	4	*Amblyomma*
6044	All together	MobileNetV2	todos ovale (67).jpg	fold2	4	*Amblyomma*
6045	All together	MobileNetV2	todos ovale (68).jpg	fold2	4	*Amblyomma*
6046	All together	MobileNetV2	todos ovale (74).jpg	fold2	4	*Amblyomma*
6047	All together	MobileNetV2	todos ovale (76).JPG	fold2	4	*Amblyomma*
6048	All together	MobileNetV2	todos ovale (78).jpg	fold2	4	*Amblyomma*
6049	All together	MobileNetV2	todos ovale (8).jpg	fold2	4	*Amblyomma*
6050	All together	MobileNetV2	todos ovale (86).jpg	fold2	4	*Amblyomma*
6051	All together	MobileNetV2	todos ovale (96).jpg	fold2	4	*Amblyomma*
6052	All together	MobileNetV2	todos ovale (97).jpg	fold2	4	*Amblyomma*
6053	All together	MobileNetV2	todos ovale (99).jpg	fold2	4	*Amblyomma*
6054	All together	MobileNetV2	todos sculptum (102).JPG	fold2	5	*Amblyomma*
6055	All together	MobileNetV2	todos sculptum (105).JPG	fold2	5	*Amblyomma*
6056	All together	MobileNetV2	todos sculptum (107).JPG	fold2	5	*Amblyomma*
6057	All together	MobileNetV2	todos sculptum (109).JPG	fold2	5	*Amblyomma*
6058	All together	MobileNetV2	todos sculptum (11).JPG	fold2	5	*Amblyomma*
6059	All together	MobileNetV2	todos sculptum (12).JPG	fold2	5	*Amblyomma*
6060	All together	MobileNetV2	todos sculptum (121).JPG	fold2	5	*Amblyomma*
6061	All together	MobileNetV2	todos sculptum (122).JPG	fold2	5	*Amblyomma*
6062	All together	MobileNetV2	todos sculptum (125).JPG	fold2	5	*Amblyomma*
6063	All together	MobileNetV2	todos sculptum (128).JPG	fold2	5	*Amblyomma*
6064	All together	MobileNetV2	todos sculptum (130).JPG	fold2	5	*Amblyomma*
6065	All together	MobileNetV2	todos sculptum (14).JPG	fold2	5	*Amblyomma*
6066	All together	MobileNetV2	todos sculptum (141).JPG	fold2	5	*Amblyomma*
6067	All together	MobileNetV2	todos sculptum (147).jpg	fold2	5	*Amblyomma*
6068	All together	MobileNetV2	todos sculptum (155).jpg	fold2	5	*Amblyomma*
6069	All together	MobileNetV2	todos sculptum (25).JPG	fold2	5	*Amblyomma*
6070	All together	MobileNetV2	todos sculptum (30).JPG	fold2	5	*Amblyomma*
6071	All together	MobileNetV2	todos sculptum (33).JPG	fold2	5	*Amblyomma*
6072	All together	MobileNetV2	todos sculptum (35).JPG	fold2	5	*Amblyomma*
6073	All together	MobileNetV2	todos sculptum (36).JPG	fold2	5	*Amblyomma*
6074	All together	MobileNetV2	todos sculptum (37).JPG	fold2	5	*Amblyomma*
6075	All together	MobileNetV2	todos sculptum (4).jpg	fold2	5	*Amblyomma*
6076	All together	MobileNetV2	todos sculptum (5).jpg	fold2	5	*Amblyomma*
6077	All together	MobileNetV2	todos sculptum (51).JPG	fold2	5	*Amblyomma*
6078	All together	MobileNetV2	todos sculptum (60).jpg	fold2	5	*Amblyomma*
6079	All together	MobileNetV2	todos sculptum (63).jpg	fold2	5	*Amblyomma*
6080	All together	MobileNetV2	todos sculptum (73).JPG	fold2	5	*Amblyomma*
6081	All together	MobileNetV2	todos sculptum (80).JPG	fold2	5	*Amblyomma*
6082	All together	MobileNetV2	todos sculptum (84).JPG	fold2	5	*Amblyomma*
6083	All together	MobileNetV2	todos sculptum (85).JPG	fold2	5	*Amblyomma*
6084	All together	MobileNetV2	todos sculptum (90).JPG	fold2	5	*Amblyomma*
6085	All together	MobileNetV2	todos triste (103).jpg	fold2	6	*Amblyomma*
6086	All together	MobileNetV2	todos triste (109).jpg	fold2	6	*Amblyomma*
6087	All together	MobileNetV2	todos triste (123).jpg	fold2	6	*Amblyomma*
6088	All together	MobileNetV2	todos triste (127).jpg	fold2	6	*Amblyomma*
6089	All together	MobileNetV2	todos triste (128).jpg	fold2	6	*Amblyomma*
6090	All together	MobileNetV2	todos triste (129).jpg	fold2	6	*Amblyomma*
6091	All together	MobileNetV2	todos triste (130).jpg	fold2	6	*Amblyomma*
6092	All together	MobileNetV2	todos triste (152).jpg	fold2	6	*Amblyomma*
6093	All together	MobileNetV2	todos triste (20).jpg	fold2	6	*Amblyomma*
6094	All together	MobileNetV2	todos triste (23).jpg	fold2	6	*Amblyomma*
6095	All together	MobileNetV2	todos triste (24).jpg	fold2	6	*Amblyomma*

6096	All together	MobileNetV2	todos triste (26).jpg	fold2	6	*Amblyomma*
6097	All together	MobileNetV2	todos triste (27).jpg	fold2	6	*Amblyomma*
6098	All together	MobileNetV2	todos triste (3).jpg	fold2	6	*Amblyomma*
6099	All together	MobileNetV2	todos triste (35).jpg	fold2	6	*Amblyomma*
6100	All together	MobileNetV2	todos triste (36).jpg	fold2	6	*Amblyomma*
6101	All together	MobileNetV2	todos triste (42).jpg	fold2	6	*Amblyomma*
6102	All together	MobileNetV2	todos triste (48).jpg	fold2	6	*Amblyomma*
6103	All together	MobileNetV2	todos triste (5).jpg	fold2	6	*Amblyomma*
6104	All together	MobileNetV2	todos triste (52).jpg	fold2	6	*Amblyomma*
6105	All together	MobileNetV2	todos triste (54).jpg	fold2	6	*Amblyomma*
6106	All together	MobileNetV2	todos triste (55).jpg	fold2	6	*Amblyomma*
6107	All together	MobileNetV2	todos triste (56).jpg	fold2	6	*Amblyomma*
6108	All together	MobileNetV2	todos triste (57).jpg	fold2	6	*Amblyomma*
6109	All together	MobileNetV2	todos triste (59).jpg	fold2	6	*Amblyomma*
6110	All together	MobileNetV2	todos triste (64).jpg	fold2	6	*Amblyomma*
6111	All together	MobileNetV2	todos triste (65).jpg	fold2	6	*Amblyomma*
6112	All together	MobileNetV2	todos triste (74).jpg	fold2	6	*Amblyomma*
6113	All together	MobileNetV2	todos triste (87).jpg	fold2	6	*Amblyomma*
6114	All together	MobileNetV2	todos triste (9).jpg	fold2	6	*Amblyomma*
6115	All together	MobileNetV2	todos triste (92).jpg	fold2	6	*Amblyomma*
6116	All together	MobileNetV2	todos aureolatum (1).jpg	fold3	1	*Amblyomma*
6117	All together	MobileNetV2	todos aureolatum (103).jpg	fold3	1	*Amblyomma*
6118	All together	MobileNetV2	todos aureolatum (108).jpg	fold3	1	*Amblyomma*
6119	All together	MobileNetV2	todos aureolatum (113).jpg	fold3	1	*Amblyomma*
6120	All together	MobileNetV2	todos aureolatum (120).jpg	fold3	1	*Amblyomma*
6121	All together	MobileNetV2	todos aureolatum (13).jpg	fold3	1	*Amblyomma*
6122	All together	MobileNetV2	todos aureolatum (15).jpg	fold3	1	*Amblyomma*
6123	All together	MobileNetV2	todos aureolatum (16).jpg	fold3	1	*Amblyomma*
6124	All together	MobileNetV2	todos aureolatum (20).jpg	fold3	1	*Amblyomma*
6125	All together	MobileNetV2	todos aureolatum (33).jpg	fold3	1	*Amblyomma*
6126	All together	MobileNetV2	todos aureolatum (34).jpg	fold3	1	*Amblyomma*
6127	All together	MobileNetV2	todos aureolatum (46).jpg	fold3	1	*Amblyomma*
6128	All together	MobileNetV2	todos aureolatum (47).jpg	fold3	1	*Amblyomma*
6129	All together	MobileNetV2	todos aureolatum (67).jpg	fold3	1	*Amblyomma*
6130	All together	MobileNetV2	todos aureolatum (68).jpg	fold3	1	*Amblyomma*
6131	All together	MobileNetV2	todos aureolatum (71).jpg	fold3	1	*Amblyomma*
6132	All together	MobileNetV2	todos aureolatum (74).jpg	fold3	1	*Amblyomma*
6133	All together	MobileNetV2	todos aureolatum (76).jpg	fold3	1	*Amblyomma*
6134	All together	MobileNetV2	todos aureolatum (8).jpg	fold3	1	*Amblyomma*
6135	All together	MobileNetV2	todos aureolatum (86).jpg	fold3	1	*Amblyomma*
6136	All together	MobileNetV2	todos aureolatum (89).jpg	fold3	1	*Amblyomma*
6137	All together	MobileNetV2	todos aureolatum (92).jpg	fold3	1	*Amblyomma*
6138	All together	MobileNetV2	todos aureolatum (94).jpg	fold3	1	*Amblyomma*
6139	All together	MobileNetV2	todos aureolatum (96).jpg	fold3	1	*Amblyomma*
6140	All together	MobileNetV2	todos cajennense (100).jpg	fold3	2	*Amblyomma*
6141	All together	MobileNetV2	todos cajennense (109).jpg	fold3	2	*Amblyomma*
6142	All together	MobileNetV2	todos cajennense (112).jpg	fold3	2	*Amblyomma*
6143	All together	MobileNetV2	todos cajennense (115).jpg	fold3	2	*Amblyomma*
6144	All together	MobileNetV2	todos cajennense (121).jpg	fold3	2	*Amblyomma*
6145	All together	MobileNetV2	todos cajennense (123).jpg	fold3	2	*Amblyomma*
6146	All together	MobileNetV2	todos cajennense (127).jpg	fold3	2	*Amblyomma*
6147	All together	MobileNetV2	todos cajennense (134).jpg	fold3	2	*Amblyomma*
6148	All together	MobileNetV2	todos cajennense (135).jpg	fold3	2	*Amblyomma*
6149	All together	MobileNetV2	todos cajennense (144).jpg	fold3	2	*Amblyomma*
6150	All together	MobileNetV2	todos cajennense (149).jpg	fold3	2	*Amblyomma*
6151	All together	MobileNetV2	todos cajennense (157).jpg	fold3	2	*Amblyomma*
6152	All together	MobileNetV2	todos cajennense (165).jpg	fold3	2	*Amblyomma*
6153	All together	MobileNetV2	todos cajennense (21).jpg	fold3	2	*Amblyomma*
6154	All together	MobileNetV2	todos cajennense (22).jpg	fold3	2	*Amblyomma*
6155	All together	MobileNetV2	todos cajennense (24).jpg	fold3	2	*Amblyomma*
6156	All together	MobileNetV2	todos cajennense (27).jpg	fold3	2	*Amblyomma*
6157	All together	MobileNetV2	todos cajennense (29).jpg	fold3	2	*Amblyomma*
6158	All together	MobileNetV2	todos cajennense (30).jpg	fold3	2	*Amblyomma*
6159	All together	MobileNetV2	todos cajennense (38).jpg	fold3	2	*Amblyomma*
6160	All together	MobileNetV2	todos cajennense (40).jpg	fold3	2	*Amblyomma*
6161	All together	MobileNetV2	todos cajennense (43).jpg	fold3	2	*Amblyomma*
6162	All together	MobileNetV2	todos cajennense (45).jpg	fold3	2	*Amblyomma*

6163	All together	MobileNetV2	todos cajennense (46).jpg	fold3	2	*Amblyomma*
6164	All together	MobileNetV2	todos cajennense (5).jpg	fold3	2	*Amblyomma*
6165	All together	MobileNetV2	todos cajennense (56).jpg	fold3	2	*Amblyomma*
6166	All together	MobileNetV2	todos cajennense (60).jpg	fold3	2	*Amblyomma*
6167	All together	MobileNetV2	todos cajennense (65).jpg	fold3	2	*Amblyomma*
6168	All together	MobileNetV2	todos cajennense (71).jpg	fold3	2	*Amblyomma*
6169	All together	MobileNetV2	todos cajennense (72).jpg	fold3	2	*Amblyomma*
6170	All together	MobileNetV2	todos cajennense (82).jpg	fold3	2	*Amblyomma*
6171	All together	MobileNetV2	todos cajennense (84).jpg	fold3	2	*Amblyomma*
6172	All together	MobileNetV2	todos cajennense (95).jpg	fold3	2	*Amblyomma*
6173	All together	MobileNetV2	todos dubitatum (101).jpg	fold3	3	*Amblyomma*
6174	All together	MobileNetV2	todos dubitatum (108).jpg	fold3	3	*Amblyomma*
6175	All together	MobileNetV2	todos dubitatum (13).JPG	fold3	3	*Amblyomma*
6176	All together	MobileNetV2	todos dubitatum (14).JPG	fold3	3	*Amblyomma*
6177	All together	MobileNetV2	todos dubitatum (19).jpg	fold3	3	*Amblyomma*
6178	All together	MobileNetV2	todos dubitatum (22).jpg	fold3	3	*Amblyomma*
6179	All together	MobileNetV2	todos dubitatum (23).jpg	fold3	3	*Amblyomma*
6180	All together	MobileNetV2	todos dubitatum (24).jpg	fold3	3	*Amblyomma*
6181	All together	MobileNetV2	todos dubitatum (28).jpg	fold3	3	*Amblyomma*
6182	All together	MobileNetV2	todos dubitatum (34).jpg	fold3	3	*Amblyomma*
6183	All together	MobileNetV2	todos dubitatum (40).jpg	fold3	3	*Amblyomma*
6184	All together	MobileNetV2	todos dubitatum (41).jpg	fold3	3	*Amblyomma*
6185	All together	MobileNetV2	todos dubitatum (43).jpg	fold3	3	*Amblyomma*
6186	All together	MobileNetV2	todos dubitatum (48).jpg	fold3	3	*Amblyomma*
6187	All together	MobileNetV2	todos dubitatum (55).jpg	fold3	3	*Amblyomma*
6188	All together	MobileNetV2	todos dubitatum (6).jpg	fold3	3	*Amblyomma*
6189	All together	MobileNetV2	todos dubitatum (66).jpg	fold3	3	*Amblyomma*
6190	All together	MobileNetV2	todos dubitatum (67).jpg	fold3	3	*Amblyomma*
6191	All together	MobileNetV2	todos dubitatum (70).jpg	fold3	3	*Amblyomma*
6192	All together	MobileNetV2	todos dubitatum (87).jpg	fold3	3	*Amblyomma*
6193	All together	MobileNetV2	todos dubitatum (90).jpg	fold3	3	*Amblyomma*
6194	All together	MobileNetV2	todos dubitatum (93).jpg	fold3	3	*Amblyomma*
6195	All together	MobileNetV2	todos ovale (101).jpg	fold3	4	*Amblyomma*
6196	All together	MobileNetV2	todos ovale (105).jpg	fold3	4	*Amblyomma*
6197	All together	MobileNetV2	todos ovale (107).JPG	fold3	4	*Amblyomma*
6198	All together	MobileNetV2	todos ovale (120).jpg	fold3	4	*Amblyomma*
6199	All together	MobileNetV2	todos ovale (13).jpg	fold3	4	*Amblyomma*
6200	All together	MobileNetV2	todos ovale (19).jpg	fold3	4	*Amblyomma*
6201	All together	MobileNetV2	todos ovale (22).jpg	fold3	4	*Amblyomma*
6202	All together	MobileNetV2	todos ovale (3).jpg	fold3	4	*Amblyomma*
6203	All together	MobileNetV2	todos ovale (30).jpg	fold3	4	*Amblyomma*
6204	All together	MobileNetV2	todos ovale (33).jpg	fold3	4	*Amblyomma*
6205	All together	MobileNetV2	todos ovale (36).jpg	fold3	4	*Amblyomma*
6206	All together	MobileNetV2	todos ovale (41).jpg	fold3	4	*Amblyomma*
6207	All together	MobileNetV2	todos ovale (43).jpg	fold3	4	*Amblyomma*
6208	All together	MobileNetV2	todos ovale (52).jpg	fold3	4	*Amblyomma*
6209	All together	MobileNetV2	todos ovale (54).jpg	fold3	4	*Amblyomma*
6210	All together	MobileNetV2	todos ovale (55).jpg	fold3	4	*Amblyomma*
6211	All together	MobileNetV2	todos ovale (71).jpg	fold3	4	*Amblyomma*
6212	All together	MobileNetV2	todos ovale (81).jpg	fold3	4	*Amblyomma*
6213	All together	MobileNetV2	todos ovale (82).jpg	fold3	4	*Amblyomma*
6214	All together	MobileNetV2	todos ovale (83).jpg	fold3	4	*Amblyomma*
6215	All together	MobileNetV2	todos ovale (84).jpg	fold3	4	*Amblyomma*
6216	All together	MobileNetV2	todos ovale (92).jpg	fold3	4	*Amblyomma*
6217	All together	MobileNetV2	todos ovale (93).jpg	fold3	4	*Amblyomma*
6218	All together	MobileNetV2	todos ovale (95).jpg	fold3	4	*Amblyomma*
6219	All together	MobileNetV2	todos sculptum (116).JPG	fold3	5	*Amblyomma*
6220	All together	MobileNetV2	todos sculptum (118).JPG	fold3	5	*Amblyomma*
6221	All together	MobileNetV2	todos sculptum (126).JPG	fold3	5	*Amblyomma*
6222	All together	MobileNetV2	todos sculptum (13).JPG	fold3	5	*Amblyomma*
6223	All together	MobileNetV2	todos sculptum (132).JPG	fold3	5	*Amblyomma*
6224	All together	MobileNetV2	todos sculptum (133).JPG	fold3	5	*Amblyomma*
6225	All together	MobileNetV2	todos sculptum (135).JPG	fold3	5	*Amblyomma*
6226	All together	MobileNetV2	todos sculptum (140).JPG	fold3	5	*Amblyomma*
6227	All together	MobileNetV2	todos sculptum (152).jpg	fold3	5	*Amblyomma*
6228	All together	MobileNetV2	todos sculptum (154).jpg	fold3	5	*Amblyomma*
6229	All together	MobileNetV2	todos sculptum (17).JPG	fold3	5	*Amblyomma*

6230	All together	MobileNetV2	todos sculptum (18).JPG	fold3	5	*Amblyomma*
6231	All together	MobileNetV2	todos sculptum (19).JPG	fold3	5	*Amblyomma*
6232	All together	MobileNetV2	todos sculptum (20).JPG	fold3	5	*Amblyomma*
6233	All together	MobileNetV2	todos sculptum (22).JPG	fold3	5	*Amblyomma*
6234	All together	MobileNetV2	todos sculptum (28).JPG	fold3	5	*Amblyomma*
6235	All together	MobileNetV2	todos sculptum (3).jpg	fold3	5	*Amblyomma*
6236	All together	MobileNetV2	todos sculptum (38).JPG	fold3	5	*Amblyomma*
6237	All together	MobileNetV2	todos sculptum (39).JPG	fold3	5	*Amblyomma*
6238	All together	MobileNetV2	todos sculptum (41).JPG	fold3	5	*Amblyomma*
6239	All together	MobileNetV2	todos sculptum (45).JPG	fold3	5	*Amblyomma*
6240	All together	MobileNetV2	todos sculptum (47).JPG	fold3	5	*Amblyomma*
6241	All together	MobileNetV2	todos sculptum (69).jpg	fold3	5	*Amblyomma*
6242	All together	MobileNetV2	todos sculptum (7).jpg	fold3	5	*Amblyomma*
6243	All together	MobileNetV2	todos sculptum (76).JPG	fold3	5	*Amblyomma*
6244	All together	MobileNetV2	todos sculptum (83).JPG	fold3	5	*Amblyomma*
6245	All together	MobileNetV2	todos sculptum (87).JPG	fold3	5	*Amblyomma*
6246	All together	MobileNetV2	todos sculptum (88).JPG	fold3	5	*Amblyomma*
6247	All together	MobileNetV2	todos sculptum (89).JPG	fold3	5	*Amblyomma*
6248	All together	MobileNetV2	todos sculptum (9).JPG	fold3	5	*Amblyomma*
6249	All together	MobileNetV2	todos sculptum (96).JPG	fold3	5	*Amblyomma*
6250	All together	MobileNetV2	todos triste (101).jpg	fold3	6	*Amblyomma*
6251	All together	MobileNetV2	todos triste (106).jpg	fold3	6	*Amblyomma*
6252	All together	MobileNetV2	todos triste (115).jpg	fold3	6	*Amblyomma*
6253	All together	MobileNetV2	todos triste (118).jpg	fold3	6	*Amblyomma*
6254	All together	MobileNetV2	todos triste (126).jpg	fold3	6	*Amblyomma*
6255	All together	MobileNetV2	todos triste (135).jpg	fold3	6	*Amblyomma*
6256	All together	MobileNetV2	todos triste (137).jpg	fold3	6	*Amblyomma*
6257	All together	MobileNetV2	todos triste (139).jpg	fold3	6	*Amblyomma*
6258	All together	MobileNetV2	todos triste (141).jpg	fold3	6	*Amblyomma*
6259	All together	MobileNetV2	todos triste (144).jpg	fold3	6	*Amblyomma*
6260	All together	MobileNetV2	todos triste (148).jpg	fold3	6	*Amblyomma*
6261	All together	MobileNetV2	todos triste (15).jpg	fold3	6	*Amblyomma*
6262	All together	MobileNetV2	todos triste (153).jpg	fold3	6	*Amblyomma*
6263	All together	MobileNetV2	todos triste (154).jpg	fold3	6	*Amblyomma*
6264	All together	MobileNetV2	todos triste (17).jpg	fold3	6	*Amblyomma*
6265	All together	MobileNetV2	todos triste (21).jpg	fold3	6	*Amblyomma*
6266	All together	MobileNetV2	todos triste (22).jpg	fold3	6	*Amblyomma*
6267	All together	MobileNetV2	todos triste (32).jpg	fold3	6	*Amblyomma*
6268	All together	MobileNetV2	todos triste (37).jpg	fold3	6	*Amblyomma*
6269	All together	MobileNetV2	todos triste (43).jpg	fold3	6	*Amblyomma*
6270	All together	MobileNetV2	todos triste (44).jpg	fold3	6	*Amblyomma*
6271	All together	MobileNetV2	todos triste (45).jpg	fold3	6	*Amblyomma*
6272	All together	MobileNetV2	todos triste (50).jpg	fold3	6	*Amblyomma*
6273	All together	MobileNetV2	todos triste (51).jpg	fold3	6	*Amblyomma*
6274	All together	MobileNetV2	todos triste (61).jpg	fold3	6	*Amblyomma*
6275	All together	MobileNetV2	todos triste (69).jpg	fold3	6	*Amblyomma*
6276	All together	MobileNetV2	todos triste (78).jpg	fold3	6	*Amblyomma*
6277	All together	MobileNetV2	todos triste (81).jpg	fold3	6	*Amblyomma*
6278	All together	MobileNetV2	todos triste (83).jpg	fold3	6	*Amblyomma*
6279	All together	MobileNetV2	todos triste (84).jpg	fold3	6	*Amblyomma*
6280	All together	MobileNetV2	todos triste (93).jpg	fold3	6	*Amblyomma*
6281	All together	MobileNetV2	todos aureolatum (101).jpg	fold4	1	*Amblyomma*
6282	All together	MobileNetV2	todos aureolatum (102).jpg	fold4	1	*Amblyomma*
6283	All together	MobileNetV2	todos aureolatum (104).jpg	fold4	1	*Amblyomma*
6284	All together	MobileNetV2	todos aureolatum (105).jpg	fold4	1	*Amblyomma*
6285	All together	MobileNetV2	todos aureolatum (11).jpg	fold4	1	*Amblyomma*
6286	All together	MobileNetV2	todos aureolatum (111).jpg	fold4	1	*Amblyomma*
6287	All together	MobileNetV2	todos aureolatum (114).jpg	fold4	1	*Amblyomma*
6288	All together	MobileNetV2	todos aureolatum (115).jpg	fold4	1	*Amblyomma*
6289	All together	MobileNetV2	todos aureolatum (17).jpg	fold4	1	*Amblyomma*
6290	All together	MobileNetV2	todos aureolatum (2).jpg	fold4	1	*Amblyomma*
6291	All together	MobileNetV2	todos aureolatum (36).jpg	fold4	1	*Amblyomma*
6292	All together	MobileNetV2	todos aureolatum (43).jpg	fold4	1	*Amblyomma*
6293	All together	MobileNetV2	todos aureolatum (44).jpg	fold4	1	*Amblyomma*
6294	All together	MobileNetV2	todos aureolatum (49).jpg	fold4	1	*Amblyomma*
6295	All together	MobileNetV2	todos aureolatum (50).jpg	fold4	1	*Amblyomma*
6296	All together	MobileNetV2	todos aureolatum (52).jpg	fold4	1	*Amblyomma*

6297	All together	MobileNetV2	todos aureolatum (59).jpg	fold4	1	*Amblyomma*
6298	All together	MobileNetV2	todos aureolatum (61).jpg	fold4	1	*Amblyomma*
6299	All together	MobileNetV2	todos aureolatum (70).jpg	fold4	1	*Amblyomma*
6300	All together	MobileNetV2	todos aureolatum (88).jpg	fold4	1	*Amblyomma*
6301	All together	MobileNetV2	todos aureolatum (9).jpg	fold4	1	*Amblyomma*
6302	All together	MobileNetV2	todos aureolatum (91).jpg	fold4	1	*Amblyomma*
6303	All together	MobileNetV2	todos aureolatum (95).jpg	fold4	1	*Amblyomma*
6304	All together	MobileNetV2	todos aureolatum (98).jpg	fold4	1	*Amblyomma*
6305	All together	MobileNetV2	todos aureolatum (99).jpg	fold4	1	*Amblyomma*
6306	All together	MobileNetV2	todos cajennense (1).jpg	fold4	2	*Amblyomma*
6307	All together	MobileNetV2	todos cajennense (103).jpg	fold4	2	*Amblyomma*
6308	All together	MobileNetV2	todos cajennense (107).jpg	fold4	2	*Amblyomma*
6309	All together	MobileNetV2	todos cajennense (118).jpg	fold4	2	*Amblyomma*
6310	All together	MobileNetV2	todos cajennense (119).jpg	fold4	2	*Amblyomma*
6311	All together	MobileNetV2	todos cajennense (122).jpg	fold4	2	*Amblyomma*
6312	All together	MobileNetV2	todos cajennense (125).jpg	fold4	2	*Amblyomma*
6313	All together	MobileNetV2	todos cajennense (130).jpg	fold4	2	*Amblyomma*
6314	All together	MobileNetV2	todos cajennense (131).jpg	fold4	2	*Amblyomma*
6315	All together	MobileNetV2	todos cajennense (136).jpg	fold4	2	*Amblyomma*
6316	All together	MobileNetV2	todos cajennense (152).jpg	fold4	2	*Amblyomma*
6317	All together	MobileNetV2	todos cajennense (16).jpg	fold4	2	*Amblyomma*
6318	All together	MobileNetV2	todos cajennense (160).jpg	fold4	2	*Amblyomma*
6319	All together	MobileNetV2	todos cajennense (163).jpg	fold4	2	*Amblyomma*
6320	All together	MobileNetV2	todos cajennense (17).jpg	fold4	2	*Amblyomma*
6321	All together	MobileNetV2	todos cajennense (18).jpg	fold4	2	*Amblyomma*
6322	All together	MobileNetV2	todos cajennense (25).jpg	fold4	2	*Amblyomma*
6323	All together	MobileNetV2	todos cajennense (31).jpg	fold4	2	*Amblyomma*
6324	All together	MobileNetV2	todos cajennense (34).jpg	fold4	2	*Amblyomma*
6325	All together	MobileNetV2	todos cajennense (35).jpg	fold4	2	*Amblyomma*
6326	All together	MobileNetV2	todos cajennense (50).jpg	fold4	2	*Amblyomma*
6327	All together	MobileNetV2	todos cajennense (52).jpg	fold4	2	*Amblyomma*
6328	All together	MobileNetV2	todos cajennense (58).jpg	fold4	2	*Amblyomma*
6329	All together	MobileNetV2	todos cajennense (66).jpg	fold4	2	*Amblyomma*
6330	All together	MobileNetV2	todos cajennense (68).jpg	fold4	2	*Amblyomma*
6331	All together	MobileNetV2	todos cajennense (69).jpg	fold4	2	*Amblyomma*
6332	All together	MobileNetV2	todos cajennense (7).jpg	fold4	2	*Amblyomma*
6333	All together	MobileNetV2	todos cajennense (80).jpg	fold4	2	*Amblyomma*
6334	All together	MobileNetV2	todos cajennense (81).jpg	fold4	2	*Amblyomma*
6335	All together	MobileNetV2	todos cajennense (89).jpg	fold4	2	*Amblyomma*
6336	All together	MobileNetV2	todos cajennense (91).jpg	fold4	2	*Amblyomma*
6337	All together	MobileNetV2	todos cajennense (92).jpg	fold4	2	*Amblyomma*
6338	All together	MobileNetV2	todos cajennense (96).jpg	fold4	2	*Amblyomma*
6339	All together	MobileNetV2	todos dubitatum (18).jpg	fold4	3	*Amblyomma*
6340	All together	MobileNetV2	todos dubitatum (27).jpg	fold4	3	*Amblyomma*
6341	All together	MobileNetV2	todos dubitatum (32).jpg	fold4	3	*Amblyomma*
6342	All together	MobileNetV2	todos dubitatum (33).jpg	fold4	3	*Amblyomma*
6343	All together	MobileNetV2	todos dubitatum (35).jpg	fold4	3	*Amblyomma*
6344	All together	MobileNetV2	todos dubitatum (4).jpg	fold4	3	*Amblyomma*
6345	All together	MobileNetV2	todos dubitatum (45).jpg	fold4	3	*Amblyomma*
6346	All together	MobileNetV2	todos dubitatum (50).jpg	fold4	3	*Amblyomma*
6347	All together	MobileNetV2	todos dubitatum (53).jpg	fold4	3	*Amblyomma*
6348	All together	MobileNetV2	todos dubitatum (58).jpg	fold4	3	*Amblyomma*
6349	All together	MobileNetV2	todos dubitatum (59).jpg	fold4	3	*Amblyomma*
6350	All together	MobileNetV2	todos dubitatum (64).jpg	fold4	3	*Amblyomma*
6351	All together	MobileNetV2	todos dubitatum (65).jpg	fold4	3	*Amblyomma*
6352	All together	MobileNetV2	todos dubitatum (7).jpg	fold4	3	*Amblyomma*
6353	All together	MobileNetV2	todos dubitatum (72).jpg	fold4	3	*Amblyomma*
6354	All together	MobileNetV2	todos dubitatum (73).jpg	fold4	3	*Amblyomma*
6355	All together	MobileNetV2	todos dubitatum (75).jpg	fold4	3	*Amblyomma*
6356	All together	MobileNetV2	todos dubitatum (79).jpg	fold4	3	*Amblyomma*
6357	All together	MobileNetV2	todos dubitatum (84).jpg	fold4	3	*Amblyomma*
6358	All together	MobileNetV2	todos dubitatum (88).jpg	fold4	3	*Amblyomma*
6359	All together	MobileNetV2	todos dubitatum (9).jpg	fold4	3	*Amblyomma*
6360	All together	MobileNetV2	todos dubitatum (99).jpg	fold4	3	*Amblyomma*
6361	All together	MobileNetV2	todos ovale (1).jpg	fold4	4	*Amblyomma*
6362	All together	MobileNetV2	todos ovale (103).jpg	fold4	4	*Amblyomma*
6363	All together	MobileNetV2	todos ovale (111).jpg	fold4	4	*Amblyomma*

6364	All together	MobileNetV2	todos ovale (113).jpg	fold4	4	*Amblyomma*
6365	All together	MobileNetV2	todos ovale (115).jpg	fold4	4	*Amblyomma*
6366	All together	MobileNetV2	todos ovale (15).jpg	fold4	4	*Amblyomma*
6367	All together	MobileNetV2	todos ovale (17).jpg	fold4	4	*Amblyomma*
6368	All together	MobileNetV2	todos ovale (21).jpg	fold4	4	*Amblyomma*
6369	All together	MobileNetV2	todos ovale (26).jpg	fold4	4	*Amblyomma*
6370	All together	MobileNetV2	todos ovale (28).jpg	fold4	4	*Amblyomma*
6371	All together	MobileNetV2	todos ovale (37).jpg	fold4	4	*Amblyomma*
6372	All together	MobileNetV2	todos ovale (39).jpg	fold4	4	*Amblyomma*
6373	All together	MobileNetV2	todos ovale (40).jpg	fold4	4	*Amblyomma*
6374	All together	MobileNetV2	todos ovale (51).jpg	fold4	4	*Amblyomma*
6375	All together	MobileNetV2	todos ovale (6).jpg	fold4	4	*Amblyomma*
6376	All together	MobileNetV2	todos ovale (63).jpg	fold4	4	*Amblyomma*
6377	All together	MobileNetV2	todos ovale (64).jpg	fold4	4	*Amblyomma*
6378	All together	MobileNetV2	todos ovale (69).jpg	fold4	4	*Amblyomma*
6379	All together	MobileNetV2	todos ovale (72).jpg	fold4	4	*Amblyomma*
6380	All together	MobileNetV2	todos ovale (80).jpg	fold4	4	*Amblyomma*
6381	All together	MobileNetV2	todos ovale (88).jpg	fold4	4	*Amblyomma*
6382	All together	MobileNetV2	todos ovale (9).jpg	fold4	4	*Amblyomma*
6383	All together	MobileNetV2	todos ovale (90).jpg	fold4	4	*Amblyomma*
6384	All together	MobileNetV2	todos ovale (91).jpg	fold4	4	*Amblyomma*
6385	All together	MobileNetV2	todos ovale (98).jpg	fold4	4	*Amblyomma*
6386	All together	MobileNetV2	todos sculptum (10).JPG	fold4	5	*Amblyomma*
6387	All together	MobileNetV2	todos sculptum (104).JPG	fold4	5	*Amblyomma*
6388	All together	MobileNetV2	todos sculptum (110).JPG	fold4	5	*Amblyomma*
6389	All together	MobileNetV2	todos sculptum (117).JPG	fold4	5	*Amblyomma*
6390	All together	MobileNetV2	todos sculptum (129).JPG	fold4	5	*Amblyomma*
6391	All together	MobileNetV2	todos sculptum (134).JPG	fold4	5	*Amblyomma*
6392	All together	MobileNetV2	todos sculptum (136).JPG	fold4	5	*Amblyomma*
6393	All together	MobileNetV2	todos sculptum (143).JPG	fold4	5	*Amblyomma*
6394	All together	MobileNetV2	todos sculptum (148).jpg	fold4	5	*Amblyomma*
6395	All together	MobileNetV2	todos sculptum (15).JPG	fold4	5	*Amblyomma*
6396	All together	MobileNetV2	todos sculptum (153).jpg	fold4	5	*Amblyomma*
6397	All together	MobileNetV2	todos sculptum (16).JPG	fold4	5	*Amblyomma*
6398	All together	MobileNetV2	todos sculptum (21).JPG	fold4	5	*Amblyomma*
6399	All together	MobileNetV2	todos sculptum (23).JPG	fold4	5	*Amblyomma*
6400	All together	MobileNetV2	todos sculptum (24).JPG	fold4	5	*Amblyomma*
6401	All together	MobileNetV2	todos sculptum (27).JPG	fold4	5	*Amblyomma*
6402	All together	MobileNetV2	todos sculptum (29).JPG	fold4	5	*Amblyomma*
6403	All together	MobileNetV2	todos sculptum (31).JPG	fold4	5	*Amblyomma*
6404	All together	MobileNetV2	todos sculptum (40).JPG	fold4	5	*Amblyomma*
6405	All together	MobileNetV2	todos sculptum (42).JPG	fold4	5	*Amblyomma*
6406	All together	MobileNetV2	todos sculptum (46).JPG	fold4	5	*Amblyomma*
6407	All together	MobileNetV2	todos sculptum (49).JPG	fold4	5	*Amblyomma*
6408	All together	MobileNetV2	todos sculptum (54).JPG	fold4	5	*Amblyomma*
6409	All together	MobileNetV2	todos sculptum (62).jpg	fold4	5	*Amblyomma*
6410	All together	MobileNetV2	todos sculptum (64).jpg	fold4	5	*Amblyomma*
6411	All together	MobileNetV2	todos sculptum (65).jpg	fold4	5	*Amblyomma*
6412	All together	MobileNetV2	todos sculptum (66).jpg	fold4	5	*Amblyomma*
6413	All together	MobileNetV2	todos sculptum (68).jpg	fold4	5	*Amblyomma*
6414	All together	MobileNetV2	todos sculptum (72).JPG	fold4	5	*Amblyomma*
6415	All together	MobileNetV2	todos sculptum (81).JPG	fold4	5	*Amblyomma*
6416	All together	MobileNetV2	todos sculptum (91).JPG	fold4	5	*Amblyomma*
6417	All together	MobileNetV2	todos triste (1).jpg	fold4	6	*Amblyomma*
6418	All together	MobileNetV2	todos triste (105).jpg	fold4	6	*Amblyomma*
6419	All together	MobileNetV2	todos triste (107).jpg	fold4	6	*Amblyomma*
6420	All together	MobileNetV2	todos triste (11).jpg	fold4	6	*Amblyomma*
6421	All together	MobileNetV2	todos triste (113).jpg	fold4	6	*Amblyomma*
6422	All together	MobileNetV2	todos triste (114).jpg	fold4	6	*Amblyomma*
6423	All together	MobileNetV2	todos triste (122).jpg	fold4	6	*Amblyomma*
6424	All together	MobileNetV2	todos triste (125).jpg	fold4	6	*Amblyomma*
6425	All together	MobileNetV2	todos triste (134).jpg	fold4	6	*Amblyomma*
6426	All together	MobileNetV2	todos triste (14).jpg	fold4	6	*Amblyomma*
6427	All together	MobileNetV2	todos triste (145).jpg	fold4	6	*Amblyomma*
6428	All together	MobileNetV2	todos triste (151).jpg	fold4	6	*Amblyomma*
6429	All together	MobileNetV2	todos triste (155).jpg	fold4	6	*Amblyomma*
6430	All together	MobileNetV2	todos triste (156).jpg	fold4	6	*Amblyomma*

6431	All together	MobileNetV2	todos triste (18).jpg	fold4	6	*Amblyomma*
6432	All together	MobileNetV2	todos triste (28).jpg	fold4	6	*Amblyomma*
6433	All together	MobileNetV2	todos triste (33).jpg	fold4	6	*Amblyomma*
6434	All together	MobileNetV2	todos triste (40).jpg	fold4	6	*Amblyomma*
6435	All together	MobileNetV2	todos triste (41).jpg	fold4	6	*Amblyomma*
6436	All together	MobileNetV2	todos triste (46).jpg	fold4	6	*Amblyomma*
6437	All together	MobileNetV2	todos triste (47).jpg	fold4	6	*Amblyomma*
6438	All together	MobileNetV2	todos triste (6).jpg	fold4	6	*Amblyomma*
6439	All together	MobileNetV2	todos triste (60).jpg	fold4	6	*Amblyomma*
6440	All together	MobileNetV2	todos triste (66).jpg	fold4	6	*Amblyomma*
6441	All together	MobileNetV2	todos triste (7).jpg	fold4	6	*Amblyomma*
6442	All together	MobileNetV2	todos triste (77).jpg	fold4	6	*Amblyomma*
6443	All together	MobileNetV2	todos triste (85).jpg	fold4	6	*Amblyomma*
6444	All together	MobileNetV2	todos triste (88).jpg	fold4	6	*Amblyomma*
6445	All together	MobileNetV2	todos triste (90).jpg	fold4	6	*Amblyomma*
6446	All together	MobileNetV2	todos triste (94).jpg	fold4	6	*Amblyomma*
6447	All together	MobileNetV2	todos triste (97).jpg	fold4	6	*Amblyomma*
6448	All together	MobileNetV2	todos triste (99).jpg	fold4	6	*Amblyomma*
6449	All together	MobileNetV2	todos aureolatum (10).jpg	fold5	1	*Amblyomma*
6450	All together	MobileNetV2	todos aureolatum (109).jpg	fold5	1	*Amblyomma*
6451	All together	MobileNetV2	todos aureolatum (119).jpg	fold5	1	*Amblyomma*
6452	All together	MobileNetV2	todos aureolatum (14).jpg	fold5	1	*Amblyomma*
6453	All together	MobileNetV2	todos aureolatum (19).jpg	fold5	1	*Amblyomma*
6454	All together	MobileNetV2	todos aureolatum (22).jpg	fold5	1	*Amblyomma*
6455	All together	MobileNetV2	todos aureolatum (25).jpg	fold5	1	*Amblyomma*
6456	All together	MobileNetV2	todos aureolatum (30).jpg	fold5	1	*Amblyomma*
6457	All together	MobileNetV2	todos aureolatum (37).jpg	fold5	1	*Amblyomma*
6458	All together	MobileNetV2	todos aureolatum (39).jpg	fold5	1	*Amblyomma*
6459	All together	MobileNetV2	todos aureolatum (40).jpg	fold5	1	*Amblyomma*
6460	All together	MobileNetV2	todos aureolatum (48).jpg	fold5	1	*Amblyomma*
6461	All together	MobileNetV2	todos aureolatum (5).jpg	fold5	1	*Amblyomma*
6462	All together	MobileNetV2	todos aureolatum (51).jpg	fold5	1	*Amblyomma*
6463	All together	MobileNetV2	todos aureolatum (53).jpg	fold5	1	*Amblyomma*
6464	All together	MobileNetV2	todos aureolatum (54).jpg	fold5	1	*Amblyomma*
6465	All together	MobileNetV2	todos aureolatum (55).jpg	fold5	1	*Amblyomma*
6466	All together	MobileNetV2	todos aureolatum (58).jpg	fold5	1	*Amblyomma*
6467	All together	MobileNetV2	todos aureolatum (62).jpg	fold5	1	*Amblyomma*
6468	All together	MobileNetV2	todos aureolatum (65).jpg	fold5	1	*Amblyomma*
6469	All together	MobileNetV2	todos aureolatum (75).jpg	fold5	1	*Amblyomma*
6470	All together	MobileNetV2	todos aureolatum (78).jpg	fold5	1	*Amblyomma*
6471	All together	MobileNetV2	todos aureolatum (81).jpg	fold5	1	*Amblyomma*
6472	All together	MobileNetV2	todos aureolatum (87).jpg	fold5	1	*Amblyomma*
6473	All together	MobileNetV2	todos cajennense (104).jpg	fold5	2	*Amblyomma*
6474	All together	MobileNetV2	todos cajennense (105).jpg	fold5	2	*Amblyomma*
6475	All together	MobileNetV2	todos cajennense (108).jpg	fold5	2	*Amblyomma*
6476	All together	MobileNetV2	todos cajennense (132).jpg	fold5	2	*Amblyomma*
6477	All together	MobileNetV2	todos cajennense (14).jpg	fold5	2	*Amblyomma*
6478	All together	MobileNetV2	todos cajennense (140).jpg	fold5	2	*Amblyomma*
6479	All together	MobileNetV2	todos cajennense (141).jpg	fold5	2	*Amblyomma*
6480	All together	MobileNetV2	todos cajennense (145).jpg	fold5	2	*Amblyomma*
6481	All together	MobileNetV2	todos cajennense (147).jpg	fold5	2	*Amblyomma*
6482	All together	MobileNetV2	todos cajennense (15).jpg	fold5	2	*Amblyomma*
6483	All together	MobileNetV2	todos cajennense (150).jpg	fold5	2	*Amblyomma*
6484	All together	MobileNetV2	todos cajennense (153).jpg	fold5	2	*Amblyomma*
6485	All together	MobileNetV2	todos cajennense (161).jpg	fold5	2	*Amblyomma*
6486	All together	MobileNetV2	todos cajennense (19).jpg	fold5	2	*Amblyomma*
6487	All together	MobileNetV2	todos cajennense (2).jpg	fold5	2	*Amblyomma*
6488	All together	MobileNetV2	todos cajennense (20).jpg	fold5	2	*Amblyomma*
6489	All together	MobileNetV2	todos cajennense (4).jpg	fold5	2	*Amblyomma*
6490	All together	MobileNetV2	todos cajennense (41).jpg	fold5	2	*Amblyomma*
6491	All together	MobileNetV2	todos cajennense (42).jpg	fold5	2	*Amblyomma*
6492	All together	MobileNetV2	todos cajennense (53).jpg	fold5	2	*Amblyomma*
6493	All together	MobileNetV2	todos cajennense (55).jpg	fold5	2	*Amblyomma*
6494	All together	MobileNetV2	todos cajennense (62).jpg	fold5	2	*Amblyomma*
6495	All together	MobileNetV2	todos cajennense (63).jpg	fold5	2	*Amblyomma*
6496	All together	MobileNetV2	todos cajennense (73).jpg	fold5	2	*Amblyomma*
6497	All together	MobileNetV2	todos cajennense (75).jpg	fold5	2	*Amblyomma*

6498	All together	MobileNetV2	todos cajennense (77).jpg	fold5	2	*Amblyomma*
6499	All together	MobileNetV2	todos cajennense (79).jpg	fold5	2	*Amblyomma*
6500	All together	MobileNetV2	todos cajennense (8).jpg	fold5	2	*Amblyomma*
6501	All together	MobileNetV2	todos cajennense (83).jpg	fold5	2	*Amblyomma*
6502	All together	MobileNetV2	todos cajennense (86).jpg	fold5	2	*Amblyomma*
6503	All together	MobileNetV2	todos cajennense (9).jpg	fold5	2	*Amblyomma*
6504	All together	MobileNetV2	todos cajennense (90).jpg	fold5	2	*Amblyomma*
6505	All together	MobileNetV2	todos cajennense (93).jpg	fold5	2	*Amblyomma*
6506	All together	MobileNetV2	todos dubitatum (10).jpg	fold5	3	*Amblyomma*
6507	All together	MobileNetV2	todos dubitatum (100).jpg	fold5	3	*Amblyomma*
6508	All together	MobileNetV2	todos dubitatum (102).jpg	fold5	3	*Amblyomma*
6509	All together	MobileNetV2	todos dubitatum (106).jpg	fold5	3	*Amblyomma*
6510	All together	MobileNetV2	todos dubitatum (16).jpg	fold5	3	*Amblyomma*
6511	All together	MobileNetV2	todos dubitatum (17).jpg	fold5	3	*Amblyomma*
6512	All together	MobileNetV2	todos dubitatum (21).jpg	fold5	3	*Amblyomma*
6513	All together	MobileNetV2	todos dubitatum (29).jpg	fold5	3	*Amblyomma*
6514	All together	MobileNetV2	todos dubitatum (30).jpg	fold5	3	*Amblyomma*
6515	All together	MobileNetV2	todos dubitatum (31).jpg	fold5	3	*Amblyomma*
6516	All together	MobileNetV2	todos dubitatum (38).jpg	fold5	3	*Amblyomma*
6517	All together	MobileNetV2	todos dubitatum (51).jpg	fold5	3	*Amblyomma*
6518	All together	MobileNetV2	todos dubitatum (54).jpg	fold5	3	*Amblyomma*
6519	All together	MobileNetV2	todos dubitatum (61).jpg	fold5	3	*Amblyomma*
6520	All together	MobileNetV2	todos dubitatum (68).jpg	fold5	3	*Amblyomma*
6521	All together	MobileNetV2	todos dubitatum (80).jpg	fold5	3	*Amblyomma*
6522	All together	MobileNetV2	todos dubitatum (86).jpg	fold5	3	*Amblyomma*
6523	All together	MobileNetV2	todos dubitatum (89).jpg	fold5	3	*Amblyomma*
6524	All together	MobileNetV2	todos dubitatum (91).jpg	fold5	3	*Amblyomma*
6525	All together	MobileNetV2	todos dubitatum (97).jpg	fold5	3	*Amblyomma*
6526	All together	MobileNetV2	todos dubitatum (98).jpg	fold5	3	*Amblyomma*
6527	All together	MobileNetV2	todos ovale (102).jpg	fold5	4	*Amblyomma*
6528	All together	MobileNetV2	todos ovale (114).jpg	fold5	4	*Amblyomma*
6529	All together	MobileNetV2	todos ovale (117).jpg	fold5	4	*Amblyomma*
6530	All together	MobileNetV2	todos ovale (119).jpg	fold5	4	*Amblyomma*
6531	All together	MobileNetV2	todos ovale (12).jpg	fold5	4	*Amblyomma*
6532	All together	MobileNetV2	todos ovale (14).jpg	fold5	4	*Amblyomma*
6533	All together	MobileNetV2	todos ovale (18).jpg	fold5	4	*Amblyomma*
6534	All together	MobileNetV2	todos ovale (31).jpg	fold5	4	*Amblyomma*
6535	All together	MobileNetV2	todos ovale (35).jpg	fold5	4	*Amblyomma*
6536	All together	MobileNetV2	todos ovale (45).jpg	fold5	4	*Amblyomma*
6537	All together	MobileNetV2	todos ovale (48).jpg	fold5	4	*Amblyomma*
6538	All together	MobileNetV2	todos ovale (49).jpg	fold5	4	*Amblyomma*
6539	All together	MobileNetV2	todos ovale (5).jpg	fold5	4	*Amblyomma*
6540	All together	MobileNetV2	todos ovale (50).jpg	fold5	4	*Amblyomma*
6541	All together	MobileNetV2	todos ovale (53).jpg	fold5	4	*Amblyomma*
6542	All together	MobileNetV2	todos ovale (59).jpg	fold5	4	*Amblyomma*
6543	All together	MobileNetV2	todos ovale (61).jpg	fold5	4	*Amblyomma*
6544	All together	MobileNetV2	todos ovale (66).jpg	fold5	4	*Amblyomma*
6545	All together	MobileNetV2	todos ovale (73).jpg	fold5	4	*Amblyomma*
6546	All together	MobileNetV2	todos ovale (75).JPG	fold5	4	*Amblyomma*
6547	All together	MobileNetV2	todos ovale (77).jpg	fold5	4	*Amblyomma*
6548	All together	MobileNetV2	todos ovale (79).jpg	fold5	4	*Amblyomma*
6549	All together	MobileNetV2	todos ovale (89).jpg	fold5	4	*Amblyomma*
6550	All together	MobileNetV2	todos ovale (94).jpg	fold5	4	*Amblyomma*
6551	All together	MobileNetV2	todos sculptum (1).jpg	fold5	5	*Amblyomma*
6552	All together	MobileNetV2	todos sculptum (101).JPG	fold5	5	*Amblyomma*
6553	All together	MobileNetV2	todos sculptum (111).JPG	fold5	5	*Amblyomma*
6554	All together	MobileNetV2	todos sculptum (131).JPG	fold5	5	*Amblyomma*
6555	All together	MobileNetV2	todos sculptum (137).JPG	fold5	5	*Amblyomma*
6556	All together	MobileNetV2	todos sculptum (144).JPG	fold5	5	*Amblyomma*
6557	All together	MobileNetV2	todos sculptum (145).jpg	fold5	5	*Amblyomma*
6558	All together	MobileNetV2	todos sculptum (146).jpg	fold5	5	*Amblyomma*
6559	All together	MobileNetV2	todos sculptum (149).jpg	fold5	5	*Amblyomma*
6560	All together	MobileNetV2	todos sculptum (150).jpg	fold5	5	*Amblyomma*
6561	All together	MobileNetV2	todos sculptum (151).jpg	fold5	5	*Amblyomma*
6562	All together	MobileNetV2	todos sculptum (2).jpg	fold5	5	*Amblyomma*
6563	All together	MobileNetV2	todos sculptum (34).JPG	fold5	5	*Amblyomma*
6564	All together	MobileNetV2	todos sculptum (43).JPG	fold5	5	*Amblyomma*

6565	All together	MobileNetV2	todos sculptum (44).JPG	fold5	5	*Amblyomma*
6566	All together	MobileNetV2	todos sculptum (50).JPG	fold5	5	*Amblyomma*
6567	All together	MobileNetV2	todos sculptum (53).JPG	fold5	5	*Amblyomma*
6568	All together	MobileNetV2	todos sculptum (56).jpg	fold5	5	*Amblyomma*
6569	All together	MobileNetV2	todos sculptum (58).jpg	fold5	5	*Amblyomma*
6570	All together	MobileNetV2	todos sculptum (59).jpg	fold5	5	*Amblyomma*
6571	All together	MobileNetV2	todos sculptum (6).jpg	fold5	5	*Amblyomma*
6572	All together	MobileNetV2	todos sculptum (67).jpg	fold5	5	*Amblyomma*
6573	All together	MobileNetV2	todos sculptum (77).JPG	fold5	5	*Amblyomma*
6574	All together	MobileNetV2	todos sculptum (78).JPG	fold5	5	*Amblyomma*
6575	All together	MobileNetV2	todos sculptum (79).JPG	fold5	5	*Amblyomma*
6576	All together	MobileNetV2	todos sculptum (8).jpg	fold5	5	*Amblyomma*
6577	All together	MobileNetV2	todos sculptum (82).JPG	fold5	5	*Amblyomma*
6578	All together	MobileNetV2	todos sculptum (86).JPG	fold5	5	*Amblyomma*
6579	All together	MobileNetV2	todos sculptum (92).JPG	fold5	5	*Amblyomma*
6580	All together	MobileNetV2	todos sculptum (94).JPG	fold5	5	*Amblyomma*
6581	All together	MobileNetV2	todos sculptum (95).JPG	fold5	5	*Amblyomma*
6582	All together	MobileNetV2	todos triste (10).jpg	fold5	6	*Amblyomma*
6583	All together	MobileNetV2	todos triste (100).jpg	fold5	6	*Amblyomma*
6584	All together	MobileNetV2	todos triste (108).jpg	fold5	6	*Amblyomma*
6585	All together	MobileNetV2	todos triste (111).jpg	fold5	6	*Amblyomma*
6586	All together	MobileNetV2	todos triste (112).jpg	fold5	6	*Amblyomma*
6587	All together	MobileNetV2	todos triste (117).jpg	fold5	6	*Amblyomma*
6588	All together	MobileNetV2	todos triste (119).jpg	fold5	6	*Amblyomma*
6589	All together	MobileNetV2	todos triste (12).jpg	fold5	6	*Amblyomma*
6590	All together	MobileNetV2	todos triste (124).jpg	fold5	6	*Amblyomma*
6591	All together	MobileNetV2	todos triste (138).jpg	fold5	6	*Amblyomma*
6592	All together	MobileNetV2	todos triste (143).jpg	fold5	6	*Amblyomma*
6593	All together	MobileNetV2	todos triste (150).jpg	fold5	6	*Amblyomma*
6594	All together	MobileNetV2	todos triste (16).jpg	fold5	6	*Amblyomma*
6595	All together	MobileNetV2	todos triste (19).jpg	fold5	6	*Amblyomma*
6596	All together	MobileNetV2	todos triste (25).jpg	fold5	6	*Amblyomma*
6597	All together	MobileNetV2	todos triste (30).jpg	fold5	6	*Amblyomma*
6598	All together	MobileNetV2	todos triste (31).jpg	fold5	6	*Amblyomma*
6599	All together	MobileNetV2	todos triste (34).jpg	fold5	6	*Amblyomma*
6600	All together	MobileNetV2	todos triste (39).jpg	fold5	6	*Amblyomma*
6601	All together	MobileNetV2	todos triste (4).jpg	fold5	6	*Amblyomma*
6602	All together	MobileNetV2	todos triste (62).jpg	fold5	6	*Amblyomma*
6603	All together	MobileNetV2	todos triste (63).jpg	fold5	6	*Amblyomma*
6604	All together	MobileNetV2	todos triste (67).jpg	fold5	6	*Amblyomma*
6605	All together	MobileNetV2	todos triste (71).jpg	fold5	6	*Amblyomma*
6606	All together	MobileNetV2	todos triste (72).jpg	fold5	6	*Amblyomma*
6607	All together	MobileNetV2	todos triste (75).jpg	fold5	6	*Amblyomma*
6608	All together	MobileNetV2	todos triste (80).jpg	fold5	6	*Amblyomma*
6609	All together	MobileNetV2	todos triste (82).jpg	fold5	6	*Amblyomma*
6610	All together	MobileNetV2	todos triste (89).jpg	fold5	6	*Amblyomma*
6611	All together	MobileNetV2	todos triste (91).jpg	fold5	6	*Amblyomma*
6612	All together	MobileNetV2	todos triste (98).jpg	fold5	6	*Amblyomma*
6613	Female	ResNet-50	femea aureolatum (15).jpg	fold1	1	*Amblyomma*
6614	Female	ResNet-50	femea aureolatum (27).jpg	fold1	1	*Amblyomma*
6615	Female	ResNet-50	femea aureolatum (30).jpg	fold1	1	*Amblyomma*
6616	Female	ResNet-50	femea aureolatum (35).jpg	fold1	1	*Amblyomma*
6617	Female	ResNet-50	femea aureolatum (36).jpg	fold1	1	*Amblyomma*
6618	Female	ResNet-50	femea aureolatum (43).jpg	fold1	1	*Amblyomma*
6619	Female	ResNet-50	femea aureolatum (44).jpg	fold1	1	*Amblyomma*
6620	Female	ResNet-50	femea aureolatum (46).jpg	fold1	1	*Amblyomma*
6621	Female	ResNet-50	femea aureolatum (48).jpg	fold1	1	*Amblyomma*
6622	Female	ResNet-50	femea aureolatum (5).jpg	fold1	1	*Amblyomma*
6623	Female	ResNet-50	femea aureolatum (53).jpg	fold1	1	*Amblyomma*
6624	Female	ResNet-50	femea cajennense (11).jpg	fold1	2	*Amblyomma*
6625	Female	ResNet-50	femea cajennense (14).jpg	fold1	2	*Amblyomma*
6626	Female	ResNet-50	femea cajennense (17).jpg	fold1	2	*Amblyomma*
6627	Female	ResNet-50	femea cajennense (18).jpg	fold1	2	*Amblyomma*
6628	Female	ResNet-50	femea cajennense (25).jpg	fold1	2	*Amblyomma*
6629	Female	ResNet-50	femea cajennense (27).jpg	fold1	2	*Amblyomma*
6630	Female	ResNet-50	femea cajennense (36).jpg	fold1	2	*Amblyomma*
6631	Female	ResNet-50	femea cajennense (37).jpg	fold1	2	*Amblyomma*

6632	Female	ResNet-50	femea cajennense (39).jpg	fold1	2	*Amblyomma*
6633	Female	ResNet-50	femea cajennense (47).jpg	fold1	2	*Amblyomma*
6634	Female	ResNet-50	femea cajennense (55).jpg	fold1	2	*Amblyomma*
6635	Female	ResNet-50	femea cajennense (58).jpg	fold1	2	*Amblyomma*
6636	Female	ResNet-50	femea cajennense (60).jpg	fold1	2	*Amblyomma*
6637	Female	ResNet-50	femea cajennense (62).jpg	fold1	2	*Amblyomma*
6638	Female	ResNet-50	femea cajennense (68).jpg	fold1	2	*Amblyomma*
6639	Female	ResNet-50	femea cajennense (69).jpg	fold1	2	*Amblyomma*
6640	Female	ResNet-50	femea cajennense (76).jpg	fold1	2	*Amblyomma*
6641	Female	ResNet-50	femea dubitatum (11).jpg	fold1	3	*Amblyomma*
6642	Female	ResNet-50	femea dubitatum (2).jpg	fold1	3	*Amblyomma*
6643	Female	ResNet-50	femea dubitatum (23).jpg	fold1	3	*Amblyomma*
6644	Female	ResNet-50	femea dubitatum (26).jpg	fold1	3	*Amblyomma*
6645	Female	ResNet-50	femea dubitatum (28).jpg	fold1	3	*Amblyomma*
6646	Female	ResNet-50	femea dubitatum (6).jpg	fold1	3	*Amblyomma*
6647	Female	ResNet-50	femea dubitatum (8).jpg	fold1	3	*Amblyomma*
6648	Female	ResNet-50	femea ovale (15).jpg	fold1	4	*Amblyomma*
6649	Female	ResNet-50	femea ovale (17).jpg	fold1	4	*Amblyomma*
6650	Female	ResNet-50	femea ovale (20).jpg	fold1	4	*Amblyomma*
6651	Female	ResNet-50	femea ovale (22).jpg	fold1	4	*Amblyomma*
6652	Female	ResNet-50	femea ovale (25).jpg	fold1	4	*Amblyomma*
6653	Female	ResNet-50	femea ovale (28).jpg	fold1	4	*Amblyomma*
6654	Female	ResNet-50	femea ovale (33).jpg	fold1	4	*Amblyomma*
6655	Female	ResNet-50	femea ovale (35).jpg	fold1	4	*Amblyomma*
6656	Female	ResNet-50	femea ovale (44).jpg	fold1	4	*Amblyomma*
6657	Female	ResNet-50	femea ovale (51).jpg	fold1	4	*Amblyomma*
6658	Female	ResNet-50	femea ovale (53).jpg	fold1	4	*Amblyomma*
6659	Female	ResNet-50	femea ovale (59).jpg	fold1	4	*Amblyomma*
6660	Female	ResNet-50	femea ovale (61).jpg	fold1	4	*Amblyomma*
6661	Female	ResNet-50	femea ovale (62).jpg	fold1	4	*Amblyomma*
6662	Female	ResNet-50	femea ovale (74).jpg	fold1	4	*Amblyomma*
6663	Female	ResNet-50	femea ovale (75).jpg	fold1	4	*Amblyomma*
6664	Female	ResNet-50	femea sculptum (1).jpg	fold1	5	*Amblyomma*
6665	Female	ResNet-50	femea sculptum (11).JPG	fold1	5	*Amblyomma*
6666	Female	ResNet-50	femea sculptum (13).JPG	fold1	5	*Amblyomma*
6667	Female	ResNet-50	femea sculptum (14).JPG	fold1	5	*Amblyomma*
6668	Female	ResNet-50	femea sculptum (22).JPG	fold1	5	*Amblyomma*
6669	Female	ResNet-50	femea sculptum (39).JPG	fold1	5	*Amblyomma*
6670	Female	ResNet-50	femea sculptum (58).jpg	fold1	5	*Amblyomma*
6671	Female	ResNet-50	femea sculptum (6).jpg	fold1	5	*Amblyomma*
6672	Female	ResNet-50	femea sculptum (60).jpg	fold1	5	*Amblyomma*
6673	Female	ResNet-50	femea sculptum (61).jpg	fold1	5	*Amblyomma*
6674	Female	ResNet-50	femea sculptum (62).jpg	fold1	5	*Amblyomma*
6675	Female	ResNet-50	femea sculptum (8).jpg	fold1	5	*Amblyomma*
6676	Female	ResNet-50	femea sculptum (9).JPG	fold1	5	*Amblyomma*
6677	Female	ResNet-50	femea triste (12).jpg	fold1	6	*Amblyomma*
6678	Female	ResNet-50	femea triste (13).jpg	fold1	6	*Amblyomma*
6679	Female	ResNet-50	femea triste (14).jpg	fold1	6	*Amblyomma*
6680	Female	ResNet-50	femea triste (23).jpg	fold1	6	*Amblyomma*
6681	Female	ResNet-50	femea triste (26).jpg	fold1	6	*Amblyomma*
6682	Female	ResNet-50	femea triste (32).jpg	fold1	6	*Amblyomma*
6683	Female	ResNet-50	femea triste (35).jpg	fold1	6	*Amblyomma*
6684	Female	ResNet-50	femea triste (38).jpg	fold1	6	*Amblyomma*
6685	Female	ResNet-50	femea triste (45).jpg	fold1	6	*Amblyomma*
6686	Female	ResNet-50	femea triste (46).jpg	fold1	6	*Amblyomma*
6687	Female	ResNet-50	femea triste (47).jpg	fold1	6	*Amblyomma*
6688	Female	ResNet-50	femea aureolatum (1).jpg	fold2	1	*Amblyomma*
6689	Female	ResNet-50	femea aureolatum (10).jpg	fold2	1	*Amblyomma*
6690	Female	ResNet-50	femea aureolatum (12).jpg	fold2	1	*Amblyomma*
6691	Female	ResNet-50	femea aureolatum (16).jpg	fold2	1	*Amblyomma*
6692	Female	ResNet-50	femea aureolatum (22).jpg	fold2	1	*Amblyomma*
6693	Female	ResNet-50	femea aureolatum (28).jpg	fold2	1	*Amblyomma*
6694	Female	ResNet-50	femea aureolatum (31).jpg	fold2	1	*Amblyomma*
6695	Female	ResNet-50	femea aureolatum (37).jpg	fold2	1	*Amblyomma*
6696	Female	ResNet-50	femea aureolatum (39).jpg	fold2	1	*Amblyomma*
6697	Female	ResNet-50	femea aureolatum (50).jpg	fold2	1	*Amblyomma*
6698	Female	ResNet-50	femea aureolatum (52).jpg	fold2	1	*Amblyomma*

6699	Female	ResNet-50	femea aureolatum (8).jpg	fold2	1	*Amblyomma*
6700	Female	ResNet-50	femea cajennense (10).jpg	fold2	2	*Amblyomma*
6701	Female	ResNet-50	femea cajennense (12).jpg	fold2	2	*Amblyomma*
6702	Female	ResNet-50	femea cajennense (19).jpg	fold2	2	*Amblyomma*
6703	Female	ResNet-50	femea cajennense (23).jpg	fold2	2	*Amblyomma*
6704	Female	ResNet-50	femea cajennense (3).jpg	fold2	2	*Amblyomma*
6705	Female	ResNet-50	femea cajennense (34).jpg	fold2	2	*Amblyomma*
6706	Female	ResNet-50	femea cajennense (35).jpg	fold2	2	*Amblyomma*
6707	Female	ResNet-50	femea cajennense (53).jpg	fold2	2	*Amblyomma*
6708	Female	ResNet-50	femea cajennense (54).jpg	fold2	2	*Amblyomma*
6709	Female	ResNet-50	femea cajennense (57).jpg	fold2	2	*Amblyomma*
6710	Female	ResNet-50	femea cajennense (6).jpg	fold2	2	*Amblyomma*
6711	Female	ResNet-50	femea cajennense (63).jpg	fold2	2	*Amblyomma*
6712	Female	ResNet-50	femea cajennense (66).jpg	fold2	2	*Amblyomma*
6713	Female	ResNet-50	femea cajennense (67).jpg	fold2	2	*Amblyomma*
6714	Female	ResNet-50	femea cajennense (7).jpg	fold2	2	*Amblyomma*
6715	Female	ResNet-50	femea cajennense (77).jpg	fold2	2	*Amblyomma*
6716	Female	ResNet-50	femea cajennense (9).jpg	fold2	2	*Amblyomma*
6717	Female	ResNet-50	femea dubitatum (12).jpg	fold2	3	*Amblyomma*
6718	Female	ResNet-50	femea dubitatum (13).jpg	fold2	3	*Amblyomma*
6719	Female	ResNet-50	femea dubitatum (19).jpg	fold2	3	*Amblyomma*
6720	Female	ResNet-50	femea dubitatum (21).jpg	fold2	3	*Amblyomma*
6721	Female	ResNet-50	femea dubitatum (24).jpg	fold2	3	*Amblyomma*
6722	Female	ResNet-50	femea dubitatum (3).jpg	fold2	3	*Amblyomma*
6723	Female	ResNet-50	femea dubitatum (33).jpg	fold2	3	*Amblyomma*
6724	Female	ResNet-50	femea ovale (1).jpg	fold2	4	*Amblyomma*
6725	Female	ResNet-50	femea ovale (10).jpg	fold2	4	*Amblyomma*
6726	Female	ResNet-50	femea ovale (14).jpg	fold2	4	*Amblyomma*
6727	Female	ResNet-50	femea ovale (18).jpg	fold2	4	*Amblyomma*
6728	Female	ResNet-50	femea ovale (29).jpg	fold2	4	*Amblyomma*
6729	Female	ResNet-50	femea ovale (32).jpg	fold2	4	*Amblyomma*
6730	Female	ResNet-50	femea ovale (34).jpg	fold2	4	*Amblyomma*
6731	Female	ResNet-50	femea ovale (36).jpg	fold2	4	*Amblyomma*
6732	Female	ResNet-50	femea ovale (39).jpg	fold2	4	*Amblyomma*
6733	Female	ResNet-50	femea ovale (50).jpg	fold2	4	*Amblyomma*
6734	Female	ResNet-50	femea ovale (55).jpg	fold2	4	*Amblyomma*
6735	Female	ResNet-50	femea ovale (56).jpg	fold2	4	*Amblyomma*
6736	Female	ResNet-50	femea ovale (6).jpg	fold2	4	*Amblyomma*
6737	Female	ResNet-50	femea ovale (64).jpg	fold2	4	*Amblyomma*
6738	Female	ResNet-50	femea ovale (65).jpg	fold2	4	*Amblyomma*
6739	Female	ResNet-50	femea ovale (66).jpg	fold2	4	*Amblyomma*
6740	Female	ResNet-50	femea sculptum (16).JPG	fold2	5	*Amblyomma*
6741	Female	ResNet-50	femea sculptum (24).JPG	fold2	5	*Amblyomma*
6742	Female	ResNet-50	femea sculptum (25).JPG	fold2	5	*Amblyomma*
6743	Female	ResNet-50	femea sculptum (26).JPG	fold2	5	*Amblyomma*
6744	Female	ResNet-50	femea sculptum (27).JPG	fold2	5	*Amblyomma*
6745	Female	ResNet-50	femea sculptum (30).JPG	fold2	5	*Amblyomma*
6746	Female	ResNet-50	femea sculptum (34).JPG	fold2	5	*Amblyomma*
6747	Female	ResNet-50	femea sculptum (36).JPG	fold2	5	*Amblyomma*
6748	Female	ResNet-50	femea sculptum (40).JPG	fold2	5	*Amblyomma*
6749	Female	ResNet-50	femea sculptum (42).JPG	fold2	5	*Amblyomma*
6750	Female	ResNet-50	femea sculptum (44).JPG	fold2	5	*Amblyomma*
6751	Female	ResNet-50	femea sculptum (46).JPG	fold2	5	*Amblyomma*
6752	Female	ResNet-50	femea triste (19).jpg	fold2	6	*Amblyomma*
6753	Female	ResNet-50	femea triste (22).jpg	fold2	6	*Amblyomma*
6754	Female	ResNet-50	femea triste (24).jpg	fold2	6	*Amblyomma*
6755	Female	ResNet-50	femea triste (27).jpg	fold2	6	*Amblyomma*
6756	Female	ResNet-50	femea triste (30).jpg	fold2	6	*Amblyomma*
6757	Female	ResNet-50	femea triste (31).jpg	fold2	6	*Amblyomma*
6758	Female	ResNet-50	femea triste (36).jpg	fold2	6	*Amblyomma*
6759	Female	ResNet-50	femea triste (42).jpg	fold2	6	*Amblyomma*
6760	Female	ResNet-50	femea triste (43).jpg	fold2	6	*Amblyomma*
6761	Female	ResNet-50	femea triste (5).jpg	fold2	6	*Amblyomma*
6762	Female	ResNet-50	femea aureolatum (11).jpg	fold3	1	*Amblyomma*
6763	Female	ResNet-50	femea aureolatum (19).jpg	fold3	1	*Amblyomma*
6764	Female	ResNet-50	femea aureolatum (2).jpg	fold3	1	*Amblyomma*
6765	Female	ResNet-50	femea aureolatum (21).jpg	fold3	1	*Amblyomma*

6766	Female	ResNet-50	femea aureolatum (32).jpg	fold3	1	*Amblyomma*
6767	Female	ResNet-50	femea aureolatum (34).jpg	fold3	1	*Amblyomma*
6768	Female	ResNet-50	femea aureolatum (38).jpg	fold3	1	*Amblyomma*
6769	Female	ResNet-50	femea aureolatum (4).jpg	fold3	1	*Amblyomma*
6770	Female	ResNet-50	femea aureolatum (47).jpg	fold3	1	*Amblyomma*
6771	Female	ResNet-50	femea aureolatum (51).jpg	fold3	1	*Amblyomma*
6772	Female	ResNet-50	femea aureolatum (55).jpg	fold3	1	*Amblyomma*
6773	Female	ResNet-50	femea cajennense (1).jpg	fold3	2	*Amblyomma*
6774	Female	ResNet-50	femea cajennense (22).jpg	fold3	2	*Amblyomma*
6775	Female	ResNet-50	femea cajennense (26).jpg	fold3	2	*Amblyomma*
6776	Female	ResNet-50	femea cajennense (28).jpg	fold3	2	*Amblyomma*
6777	Female	ResNet-50	femea cajennense (32).jpg	fold3	2	*Amblyomma*
6778	Female	ResNet-50	femea cajennense (38).jpg	fold3	2	*Amblyomma*
6779	Female	ResNet-50	femea cajennense (41).jpg	fold3	2	*Amblyomma*
6780	Female	ResNet-50	femea cajennense (50).jpg	fold3	2	*Amblyomma*
6781	Female	ResNet-50	femea cajennense (51).jpg	fold3	2	*Amblyomma*
6782	Female	ResNet-50	femea cajennense (71).jpg	fold3	2	*Amblyomma*
6783	Female	ResNet-50	femea cajennense (72).jpg	fold3	2	*Amblyomma*
6784	Female	ResNet-50	femea cajennense (73).jpg	fold3	2	*Amblyomma*
6785	Female	ResNet-50	femea cajennense (78).jpg	fold3	2	*Amblyomma*
6786	Female	ResNet-50	femea cajennense (79).jpg	fold3	2	*Amblyomma*
6787	Female	ResNet-50	femea cajennense (8).jpg	fold3	2	*Amblyomma*
6788	Female	ResNet-50	femea cajennense (80).jpg	fold3	2	*Amblyomma*
6789	Female	ResNet-50	femea dubitatum (16).jpg	fold3	3	*Amblyomma*
6790	Female	ResNet-50	femea dubitatum (18).jpg	fold3	3	*Amblyomma*
6791	Female	ResNet-50	femea dubitatum (25).jpg	fold3	3	*Amblyomma*
6792	Female	ResNet-50	femea dubitatum (27).jpg	fold3	3	*Amblyomma*
6793	Female	ResNet-50	femea dubitatum (29).jpg	fold3	3	*Amblyomma*
6794	Female	ResNet-50	femea dubitatum (32).jpg	fold3	3	*Amblyomma*
6795	Female	ResNet-50	femea dubitatum (34).jpg	fold3	3	*Amblyomma*
6796	Female	ResNet-50	femea ovale (11).jpg	fold3	4	*Amblyomma*
6797	Female	ResNet-50	femea ovale (19).jpg	fold3	4	*Amblyomma*
6798	Female	ResNet-50	femea ovale (26).jpg	fold3	4	*Amblyomma*
6799	Female	ResNet-50	femea ovale (3).JPG	fold3	4	*Amblyomma*
6800	Female	ResNet-50	femea ovale (30).jpg	fold3	4	*Amblyomma*
6801	Female	ResNet-50	femea ovale (31).jpg	fold3	4	*Amblyomma*
6802	Female	ResNet-50	femea ovale (37).jpg	fold3	4	*Amblyomma*
6803	Female	ResNet-50	femea ovale (38).jpg	fold3	4	*Amblyomma*
6804	Female	ResNet-50	femea ovale (45).jpg	fold3	4	*Amblyomma*
6805	Female	ResNet-50	femea ovale (49).jpg	fold3	4	*Amblyomma*
6806	Female	ResNet-50	femea ovale (5).jpg	fold3	4	*Amblyomma*
6807	Female	ResNet-50	femea ovale (54).jpg	fold3	4	*Amblyomma*
6808	Female	ResNet-50	femea ovale (67).jpg	fold3	4	*Amblyomma*
6809	Female	ResNet-50	femea ovale (71).jpg	fold3	4	*Amblyomma*
6810	Female	ResNet-50	femea ovale (78).jpg	fold3	4	*Amblyomma*
6811	Female	ResNet-50	femea sculptum (10).JPG	fold3	5	*Amblyomma*
6812	Female	ResNet-50	femea sculptum (17).JPG	fold3	5	*Amblyomma*
6813	Female	ResNet-50	femea sculptum (18).JPG	fold3	5	*Amblyomma*
6814	Female	ResNet-50	femea sculptum (21).JPG	fold3	5	*Amblyomma*
6815	Female	ResNet-50	femea sculptum (28).JPG	fold3	5	*Amblyomma*
6816	Female	ResNet-50	femea sculptum (43).JPG	fold3	5	*Amblyomma*
6817	Female	ResNet-50	femea sculptum (45).JPG	fold3	5	*Amblyomma*
6818	Female	ResNet-50	femea sculptum (47).JPG	fold3	5	*Amblyomma*
6819	Female	ResNet-50	femea sculptum (49).JPG	fold3	5	*Amblyomma*
6820	Female	ResNet-50	femea sculptum (53).JPG	fold3	5	*Amblyomma*
6821	Female	ResNet-50	femea sculptum (54).JPG	fold3	5	*Amblyomma*
6822	Female	ResNet-50	femea sculptum (59).jpg	fold3	5	*Amblyomma*
6823	Female	ResNet-50	femea sculptum (7).jpg	fold3	5	*Amblyomma*
6824	Female	ResNet-50	femea triste (1).jpg	fold3	6	*Amblyomma*
6825	Female	ResNet-50	femea triste (10).jpg	fold3	6	*Amblyomma*
6826	Female	ResNet-50	femea triste (11).jpg	fold3	6	*Amblyomma*
6827	Female	ResNet-50	femea triste (2).jpg	fold3	6	*Amblyomma*
6828	Female	ResNet-50	femea triste (37).jpg	fold3	6	*Amblyomma*
6829	Female	ResNet-50	femea triste (4).jpg	fold3	6	*Amblyomma*
6830	Female	ResNet-50	femea triste (44).jpg	fold3	6	*Amblyomma*
6831	Female	ResNet-50	femea triste (50).jpg	fold3	6	*Amblyomma*
6832	Female	ResNet-50	femea triste (7).jpg	fold3	6	*Amblyomma*

6833	Female	ResNet-50	femea triste (9).jpg	fold3	6	*Amblyomma*
6834	Female	ResNet-50	femea aureolatum (14).jpg	fold4	1	*Amblyomma*
6835	Female	ResNet-50	femea aureolatum (17).jpg	fold4	1	*Amblyomma*
6836	Female	ResNet-50	femea aureolatum (20).jpg	fold4	1	*Amblyomma*
6837	Female	ResNet-50	femea aureolatum (29).jpg	fold4	1	*Amblyomma*
6838	Female	ResNet-50	femea aureolatum (3).jpg	fold4	1	*Amblyomma*
6839	Female	ResNet-50	femea aureolatum (40).jpg	fold4	1	*Amblyomma*
6840	Female	ResNet-50	femea aureolatum (42).jpg	fold4	1	*Amblyomma*
6841	Female	ResNet-50	femea aureolatum (49).jpg	fold4	1	*Amblyomma*
6842	Female	ResNet-50	femea aureolatum (54).jpg	fold4	1	*Amblyomma*
6843	Female	ResNet-50	femea aureolatum (6).jpg	fold4	1	*Amblyomma*
6844	Female	ResNet-50	femea aureolatum (7).jpg	fold4	1	*Amblyomma*
6845	Female	ResNet-50	femea aureolatum (9).jpg	fold4	1	*Amblyomma*
6846	Female	ResNet-50	femea cajennense (16).jpg	fold4	2	*Amblyomma*
6847	Female	ResNet-50	femea cajennense (20).jpg	fold4	2	*Amblyomma*
6848	Female	ResNet-50	femea cajennense (24).jpg	fold4	2	*Amblyomma*
6849	Female	ResNet-50	femea cajennense (30).jpg	fold4	2	*Amblyomma*
6850	Female	ResNet-50	femea cajennense (4).jpg	fold4	2	*Amblyomma*
6851	Female	ResNet-50	femea cajennense (43).jpg	fold4	2	*Amblyomma*
6852	Female	ResNet-50	femea cajennense (44).jpg	fold4	2	*Amblyomma*
6853	Female	ResNet-50	femea cajennense (45).jpg	fold4	2	*Amblyomma*
6854	Female	ResNet-50	femea cajennense (46).jpg	fold4	2	*Amblyomma*
6855	Female	ResNet-50	femea cajennense (52).jpg	fold4	2	*Amblyomma*
6856	Female	ResNet-50	femea cajennense (56).jpg	fold4	2	*Amblyomma*
6857	Female	ResNet-50	femea cajennense (61).jpg	fold4	2	*Amblyomma*
6858	Female	ResNet-50	femea cajennense (70).jpg	fold4	2	*Amblyomma*
6859	Female	ResNet-50	femea cajennense (74).jpg	fold4	2	*Amblyomma*
6860	Female	ResNet-50	femea cajennense (75).jpg	fold4	2	*Amblyomma*
6861	Female	ResNet-50	femea cajennense (81).jpg	fold4	2	*Amblyomma*
6862	Female	ResNet-50	femea dubitatum (10).jpg	fold4	3	*Amblyomma*
6863	Female	ResNet-50	femea dubitatum (15).jpg	fold4	3	*Amblyomma*
6864	Female	ResNet-50	femea dubitatum (17).jpg	fold4	3	*Amblyomma*
6865	Female	ResNet-50	femea dubitatum (22).jpg	fold4	3	*Amblyomma*
6866	Female	ResNet-50	femea dubitatum (30).jpg	fold4	3	*Amblyomma*
6867	Female	ResNet-50	femea dubitatum (7).jpg	fold4	3	*Amblyomma*
6868	Female	ResNet-50	femea ovale (2).jpg	fold4	4	*Amblyomma*
6869	Female	ResNet-50	femea ovale (23).jpg	fold4	4	*Amblyomma*
6870	Female	ResNet-50	femea ovale (24).jpg	fold4	4	*Amblyomma*
6871	Female	ResNet-50	femea ovale (4).JPG	fold4	4	*Amblyomma*
6872	Female	ResNet-50	femea ovale (40).jpg	fold4	4	*Amblyomma*
6873	Female	ResNet-50	femea ovale (46).jpg	fold4	4	*Amblyomma*
6874	Female	ResNet-50	femea ovale (48).jpg	fold4	4	*Amblyomma*
6875	Female	ResNet-50	femea ovale (52).jpg	fold4	4	*Amblyomma*
6876	Female	ResNet-50	femea ovale (57).jpg	fold4	4	*Amblyomma*
6877	Female	ResNet-50	femea ovale (58).jpg	fold4	4	*Amblyomma*
6878	Female	ResNet-50	femea ovale (7).jpg	fold4	4	*Amblyomma*
6879	Female	ResNet-50	femea ovale (70).jpg	fold4	4	*Amblyomma*
6880	Female	ResNet-50	femea ovale (73).jpg	fold4	4	*Amblyomma*
6881	Female	ResNet-50	femea ovale (76).jpg	fold4	4	*Amblyomma*
6882	Female	ResNet-50	femea ovale (77).jpg	fold4	4	*Amblyomma*
6883	Female	ResNet-50	femea ovale (9).jpg	fold4	4	*Amblyomma*
6884	Female	ResNet-50	femea sculptum (15).JPG	fold4	5	*Amblyomma*
6885	Female	ResNet-50	femea sculptum (19).JPG	fold4	5	*Amblyomma*
6886	Female	ResNet-50	femea sculptum (2).jpg	fold4	5	*Amblyomma*
6887	Female	ResNet-50	femea sculptum (23).JPG	fold4	5	*Amblyomma*
6888	Female	ResNet-50	femea sculptum (29).JPG	fold4	5	*Amblyomma*
6889	Female	ResNet-50	femea sculptum (3).jpg	fold4	5	*Amblyomma*
6890	Female	ResNet-50	femea sculptum (37).JPG	fold4	5	*Amblyomma*
6891	Female	ResNet-50	femea sculptum (38).JPG	fold4	5	*Amblyomma*
6892	Female	ResNet-50	femea sculptum (41).JPG	fold4	5	*Amblyomma*
6893	Female	ResNet-50	femea sculptum (5).jpg	fold4	5	*Amblyomma*
6894	Female	ResNet-50	femea sculptum (52).JPG	fold4	5	*Amblyomma*
6895	Female	ResNet-50	femea sculptum (56).jpg	fold4	5	*Amblyomma*
6896	Female	ResNet-50	femea sculptum (57).jpg	fold4	5	*Amblyomma*
6897	Female	ResNet-50	femea triste (15).jpg	fold4	6	*Amblyomma*
6898	Female	ResNet-50	femea triste (18).jpg	fold4	6	*Amblyomma*
6899	Female	ResNet-50	femea triste (28).jpg	fold4	6	*Amblyomma*

6900	Female	ResNet-50	femea triste (3).jpg	fold4	6	*Amblyomma*
6901	Female	ResNet-50	femea triste (33).jpg	fold4	6	*Amblyomma*
6902	Female	ResNet-50	femea triste (34).jpg	fold4	6	*Amblyomma*
6903	Female	ResNet-50	femea triste (40).jpg	fold4	6	*Amblyomma*
6904	Female	ResNet-50	femea triste (41).jpg	fold4	6	*Amblyomma*
6905	Female	ResNet-50	femea triste (51).jpg	fold4	6	*Amblyomma*
6906	Female	ResNet-50	femea triste (52).jpg	fold4	6	*Amblyomma*
6907	Female	ResNet-50	femea aureolatum (13).jpg	fold5	1	*Amblyomma*
6908	Female	ResNet-50	femea aureolatum (18).jpg	fold5	1	*Amblyomma*
6909	Female	ResNet-50	femea aureolatum (23).jpg	fold5	1	*Amblyomma*
6910	Female	ResNet-50	femea aureolatum (24).jpg	fold5	1	*Amblyomma*
6911	Female	ResNet-50	femea aureolatum (25).jpg	fold5	1	*Amblyomma*
6912	Female	ResNet-50	femea aureolatum (26).jpg	fold5	1	*Amblyomma*
6913	Female	ResNet-50	femea aureolatum (33).jpg	fold5	1	*Amblyomma*
6914	Female	ResNet-50	femea aureolatum (41).jpg	fold5	1	*Amblyomma*
6915	Female	ResNet-50	femea aureolatum (45).jpg	fold5	1	*Amblyomma*
6916	Female	ResNet-50	femea aureolatum (56).jpg	fold5	1	*Amblyomma*
6917	Female	ResNet-50	femea aureolatum (57).jpg	fold5	1	*Amblyomma*
6918	Female	ResNet-50	femea aureolatum (58).jpg	fold5	1	*Amblyomma*
6919	Female	ResNet-50	femea cajennense (13).jpg	fold5	2	*Amblyomma*
6920	Female	ResNet-50	femea cajennense (15).jpg	fold5	2	*Amblyomma*
6921	Female	ResNet-50	femea cajennense (2).jpg	fold5	2	*Amblyomma*
6922	Female	ResNet-50	femea cajennense (21).jpg	fold5	2	*Amblyomma*
6923	Female	ResNet-50	femea cajennense (29).jpg	fold5	2	*Amblyomma*
6924	Female	ResNet-50	femea cajennense (31).jpg	fold5	2	*Amblyomma*
6925	Female	ResNet-50	femea cajennense (33).jpg	fold5	2	*Amblyomma*
6926	Female	ResNet-50	femea cajennense (40).jpg	fold5	2	*Amblyomma*
6927	Female	ResNet-50	femea cajennense (42).jpg	fold5	2	*Amblyomma*
6928	Female	ResNet-50	femea cajennense (48).jpg	fold5	2	*Amblyomma*
6929	Female	ResNet-50	femea cajennense (49).jpg	fold5	2	*Amblyomma*
6930	Female	ResNet-50	femea cajennense (5).jpg	fold5	2	*Amblyomma*
6931	Female	ResNet-50	femea cajennense (59).jpg	fold5	2	*Amblyomma*
6932	Female	ResNet-50	femea cajennense (64).jpg	fold5	2	*Amblyomma*
6933	Female	ResNet-50	femea cajennense (65).jpg	fold5	2	*Amblyomma*
6934	Female	ResNet-50	femea cajennense (82).jpg	fold5	2	*Amblyomma*
6935	Female	ResNet-50	femea dubitatum (1).jpg	fold5	3	*Amblyomma*
6936	Female	ResNet-50	femea dubitatum (14).jpg	fold5	3	*Amblyomma*
6937	Female	ResNet-50	femea dubitatum (20).jpg	fold5	3	*Amblyomma*
6938	Female	ResNet-50	femea dubitatum (31).jpg	fold5	3	*Amblyomma*
6939	Female	ResNet-50	femea dubitatum (4).jpg	fold5	3	*Amblyomma*
6940	Female	ResNet-50	femea dubitatum (5).jpg	fold5	3	*Amblyomma*
6941	Female	ResNet-50	femea dubitatum (9).jpg	fold5	3	*Amblyomma*
6942	Female	ResNet-50	femea ovale (12).jpg	fold5	4	*Amblyomma*
6943	Female	ResNet-50	femea ovale (13).jpg	fold5	4	*Amblyomma*
6944	Female	ResNet-50	femea ovale (16).jpg	fold5	4	*Amblyomma*
6945	Female	ResNet-50	femea ovale (21).jpg	fold5	4	*Amblyomma*
6946	Female	ResNet-50	femea ovale (27).jpg	fold5	4	*Amblyomma*
6947	Female	ResNet-50	femea ovale (41).jpg	fold5	4	*Amblyomma*
6948	Female	ResNet-50	femea ovale (42).jpg	fold5	4	*Amblyomma*
6949	Female	ResNet-50	femea ovale (43).jpg	fold5	4	*Amblyomma*
6950	Female	ResNet-50	femea ovale (47).jpg	fold5	4	*Amblyomma*
6951	Female	ResNet-50	femea ovale (60).jpg	fold5	4	*Amblyomma*
6952	Female	ResNet-50	femea ovale (63).jpg	fold5	4	*Amblyomma*
6953	Female	ResNet-50	femea ovale (68).jpg	fold5	4	*Amblyomma*
6954	Female	ResNet-50	femea ovale (69).jpg	fold5	4	*Amblyomma*
6955	Female	ResNet-50	femea ovale (72).jpg	fold5	4	*Amblyomma*
6956	Female	ResNet-50	femea ovale (79).jpg	fold5	4	*Amblyomma*
6957	Female	ResNet-50	femea ovale (8).jpg	fold5	4	*Amblyomma*
6958	Female	ResNet-50	femea sculptum (12).JPG	fold5	5	*Amblyomma*
6959	Female	ResNet-50	femea sculptum (20).JPG	fold5	5	*Amblyomma*
6960	Female	ResNet-50	femea sculptum (31).JPG	fold5	5	*Amblyomma*
6961	Female	ResNet-50	femea sculptum (32).JPG	fold5	5	*Amblyomma*
6962	Female	ResNet-50	femea sculptum (33).JPG	fold5	5	*Amblyomma*
6963	Female	ResNet-50	femea sculptum (35).JPG	fold5	5	*Amblyomma*
6964	Female	ResNet-50	femea sculptum (4).jpg	fold5	5	*Amblyomma*
6965	Female	ResNet-50	femea sculptum (48).JPG	fold5	5	*Amblyomma*
6966	Female	ResNet-50	femea sculptum (50).JPG	fold5	5	*Amblyomma*

6967	Female	ResNet-50	femea sculptum (51).JPG	fold5	5	*Amblyomma*
6968	Female	ResNet-50	femea sculptum (55).JPG	fold5	5	*Amblyomma*
6969	Female	ResNet-50	femea sculptum (63).jpg	fold5	5	*Amblyomma*
6970	Female	ResNet-50	femea triste (16).jpg	fold5	6	*Amblyomma*
6971	Female	ResNet-50	femea triste (17).jpg	fold5	6	*Amblyomma*
6972	Female	ResNet-50	femea triste (20).jpg	fold5	6	*Amblyomma*
6973	Female	ResNet-50	femea triste (21).jpg	fold5	6	*Amblyomma*
6974	Female	ResNet-50	femea triste (25).jpg	fold5	6	*Amblyomma*
6975	Female	ResNet-50	femea triste (29).jpg	fold5	6	*Amblyomma*
6976	Female	ResNet-50	femea triste (39).jpg	fold5	6	*Amblyomma*
6977	Female	ResNet-50	femea triste (48).jpg	fold5	6	*Amblyomma*
6978	Female	ResNet-50	femea triste (49).jpg	fold5	6	*Amblyomma*
6979	Female	ResNet-50	femea triste (6).jpg	fold5	6	*Amblyomma*
6980	Female	ResNet-50	femea triste (8).jpg	fold5	6	*Amblyomma*
6981	Male	ResNet-50	macho aureolatum (11).jpg	fold1	1	*Amblyomma*
6982	Male	ResNet-50	macho aureolatum (12).jpg	fold1	1	*Amblyomma*
6983	Male	ResNet-50	macho aureolatum (16).jpg	fold1	1	*Amblyomma*
6984	Male	ResNet-50	macho aureolatum (17).jpg	fold1	1	*Amblyomma*
6985	Male	ResNet-50	macho aureolatum (35).jpg	fold1	1	*Amblyomma*
6986	Male	ResNet-50	macho aureolatum (36).jpg	fold1	1	*Amblyomma*
6987	Male	ResNet-50	macho aureolatum (42).jpg	fold1	1	*Amblyomma*
6988	Male	ResNet-50	macho aureolatum (44).jpg	fold1	1	*Amblyomma*
6989	Male	ResNet-50	macho aureolatum (51).jpg	fold1	1	*Amblyomma*
6990	Male	ResNet-50	macho aureolatum (58).jpg	fold1	1	*Amblyomma*
6991	Male	ResNet-50	macho aureolatum (61).jpg	fold1	1	*Amblyomma*
6992	Male	ResNet-50	macho aureolatum (9).jpg	fold1	1	*Amblyomma*
6993	Male	ResNet-50	macho cajennense (1).jpg	fold1	2	*Amblyomma*
6994	Male	ResNet-50	macho cajennense (12).jpg	fold1	2	*Amblyomma*
6995	Male	ResNet-50	macho cajennense (17).jpg	fold1	2	*Amblyomma*
6996	Male	ResNet-50	macho cajennense (23).jpg	fold1	2	*Amblyomma*
6997	Male	ResNet-50	macho cajennense (34).jpg	fold1	2	*Amblyomma*
6998	Male	ResNet-50	macho cajennense (36).jpg	fold1	2	*Amblyomma*
6999	Male	ResNet-50	macho cajennense (37).jpg	fold1	2	*Amblyomma*
7000	Male	ResNet-50	macho cajennense (39).jpg	fold1	2	*Amblyomma*
7001	Male	ResNet-50	macho cajennense (52).jpg	fold1	2	*Amblyomma*
7002	Male	ResNet-50	macho cajennense (64).jpg	fold1	2	*Amblyomma*
7003	Male	ResNet-50	macho cajennense (66).jpg	fold1	2	*Amblyomma*
7004	Male	ResNet-50	macho cajennense (71).jpg	fold1	2	*Amblyomma*
7005	Male	ResNet-50	macho cajennense (73).jpg	fold1	2	*Amblyomma*
7006	Male	ResNet-50	macho cajennense (76).jpg	fold1	2	*Amblyomma*
7007	Male	ResNet-50	macho cajennense (82).jpg	fold1	2	*Amblyomma*
7008	Male	ResNet-50	macho cajennense (83).jpg	fold1	2	*Amblyomma*
7009	Male	ResNet-50	macho dubitatum (11).jpg	fold1	3	*Amblyomma*
7010	Male	ResNet-50	macho dubitatum (16).jpg	fold1	3	*Amblyomma*
7011	Male	ResNet-50	macho dubitatum (18).jpg	fold1	3	*Amblyomma*
7012	Male	ResNet-50	macho dubitatum (2).jpg	fold1	3	*Amblyomma*
7013	Male	ResNet-50	macho dubitatum (21).jpg	fold1	3	*Amblyomma*
7014	Male	ResNet-50	macho dubitatum (22).jpg	fold1	3	*Amblyomma*
7015	Male	ResNet-50	macho dubitatum (31).jpg	fold1	3	*Amblyomma*
7016	Male	ResNet-50	macho dubitatum (38).jpg	fold1	3	*Amblyomma*
7017	Male	ResNet-50	macho dubitatum (40).jpg	fold1	3	*Amblyomma*
7018	Male	ResNet-50	macho dubitatum (47).jpg	fold1	3	*Amblyomma*
7019	Male	ResNet-50	macho dubitatum (50).jpg	fold1	3	*Amblyomma*
7020	Male	ResNet-50	macho dubitatum (57).jpg	fold1	3	*Amblyomma*
7021	Male	ResNet-50	macho dubitatum (69).jpg	fold1	3	*Amblyomma*
7022	Male	ResNet-50	macho dubitatum (74).jpg	fold1	3	*Amblyomma*
7023	Male	ResNet-50	macho dubitatum (9).jpg	fold1	3	*Amblyomma*
7024	Male	ResNet-50	macho ovale (12).jpg	fold1	4	*Amblyomma*
7025	Male	ResNet-50	macho ovale (14).jpg	fold1	4	*Amblyomma*
7026	Male	ResNet-50	macho ovale (15).jpg	fold1	4	*Amblyomma*
7027	Male	ResNet-50	macho ovale (2).jpg	fold1	4	*Amblyomma*
7028	Male	ResNet-50	macho ovale (20).jpg	fold1	4	*Amblyomma*
7029	Male	ResNet-50	macho ovale (30).jpg	fold1	4	*Amblyomma*
7030	Male	ResNet-50	macho ovale (41).jpg	fold1	4	*Amblyomma*
7031	Male	ResNet-50	macho ovale (7).jpg	fold1	4	*Amblyomma*
7032	Male	ResNet-50	macho sculptum (16).JPG	fold1	5	*Amblyomma*
7033	Male	ResNet-50	macho sculptum (20).JPG	fold1	5	*Amblyomma*

7034	Male	ResNet-50	macho sculptum (23).JPG	fold1	5	*Amblyomma*
7035	Male	ResNet-50	macho sculptum (3).jpg	fold1	5	*Amblyomma*
7036	Male	ResNet-50	macho sculptum (30).JPG	fold1	5	*Amblyomma*
7037	Male	ResNet-50	macho sculptum (4).jpg	fold1	5	*Amblyomma*
7038	Male	ResNet-50	macho sculptum (40).JPG	fold1	5	*Amblyomma*
7039	Male	ResNet-50	macho sculptum (43).JPG	fold1	5	*Amblyomma*
7040	Male	ResNet-50	macho sculptum (45).JPG	fold1	5	*Amblyomma*
7041	Male	ResNet-50	macho sculptum (46).JPG	fold1	5	*Amblyomma*
7042	Male	ResNet-50	macho sculptum (5).jpg	fold1	5	*Amblyomma*
7043	Male	ResNet-50	macho sculptum (53).JPG	fold1	5	*Amblyomma*
7044	Male	ResNet-50	macho sculptum (56).JPG	fold1	5	*Amblyomma*
7045	Male	ResNet-50	macho sculptum (62).JPG	fold1	5	*Amblyomma*
7046	Male	ResNet-50	macho sculptum (63).JPG	fold1	5	*Amblyomma*
7047	Male	ResNet-50	macho sculptum (71).JPG	fold1	5	*Amblyomma*
7048	Male	ResNet-50	macho sculptum (81).JPG	fold1	5	*Amblyomma*
7049	Male	ResNet-50	macho sculptum (89).jpg	fold1	5	*Amblyomma*
7050	Male	ResNet-50	macho triste (10).jpg	fold1	6	*Amblyomma*
7051	Male	ResNet-50	macho triste (13).jpg	fold1	6	*Amblyomma*
7052	Male	ResNet-50	macho triste (18).jpg	fold1	6	*Amblyomma*
7053	Male	ResNet-50	macho triste (19).jpg	fold1	6	*Amblyomma*
7054	Male	ResNet-50	macho triste (23).jpg	fold1	6	*Amblyomma*
7055	Male	ResNet-50	macho triste (29).jpg	fold1	6	*Amblyomma*
7056	Male	ResNet-50	macho triste (3).jpg	fold1	6	*Amblyomma*
7057	Male	ResNet-50	macho triste (32).jpg	fold1	6	*Amblyomma*
7058	Male	ResNet-50	macho triste (38).jpg	fold1	6	*Amblyomma*
7059	Male	ResNet-50	macho triste (43).jpg	fold1	6	*Amblyomma*
7060	Male	ResNet-50	macho triste (48).jpg	fold1	6	*Amblyomma*
7061	Male	ResNet-50	macho triste (5).jpg	fold1	6	*Amblyomma*
7062	Male	ResNet-50	macho triste (52).jpg	fold1	6	*Amblyomma*
7063	Male	ResNet-50	macho triste (56).jpg	fold1	6	*Amblyomma*
7064	Male	ResNet-50	macho triste (7).jpg	fold1	6	*Amblyomma*
7065	Male	ResNet-50	macho triste (75).jpg	fold1	6	*Amblyomma*
7066	Male	ResNet-50	macho triste (83).jpg	fold1	6	*Amblyomma*
7067	Male	ResNet-50	macho triste (88).jpg	fold1	6	*Amblyomma*
7068	Male	ResNet-50	macho triste (92).jpg	fold1	6	*Amblyomma*
7069	Male	ResNet-50	macho triste (93).jpg	fold1	6	*Amblyomma*
7070	Male	ResNet-50	macho aureolatum (19).jpg	fold2	1	*Amblyomma*
7071	Male	ResNet-50	macho aureolatum (22).jpg	fold2	1	*Amblyomma*
7072	Male	ResNet-50	macho aureolatum (26).jpg	fold2	1	*Amblyomma*
7073	Male	ResNet-50	macho aureolatum (28).jpg	fold2	1	*Amblyomma*
7074	Male	ResNet-50	macho aureolatum (32).jpg	fold2	1	*Amblyomma*
7075	Male	ResNet-50	macho aureolatum (46).jpg	fold2	1	*Amblyomma*
7076	Male	ResNet-50	macho aureolatum (49).jpg	fold2	1	*Amblyomma*
7077	Male	ResNet-50	macho aureolatum (50).jpg	fold2	1	*Amblyomma*
7078	Male	ResNet-50	macho aureolatum (52).jpg	fold2	1	*Amblyomma*
7079	Male	ResNet-50	macho aureolatum (56).jpg	fold2	1	*Amblyomma*
7080	Male	ResNet-50	macho aureolatum (59).jpg	fold2	1	*Amblyomma*
7081	Male	ResNet-50	macho aureolatum (6).jpg	fold2	1	*Amblyomma*
7082	Male	ResNet-50	macho aureolatum (62).jpg	fold2	1	*Amblyomma*
7083	Male	ResNet-50	macho cajennense (10).jpg	fold2	2	*Amblyomma*
7084	Male	ResNet-50	macho cajennense (2).jpg	fold2	2	*Amblyomma*
7085	Male	ResNet-50	macho cajennense (26).jpg	fold2	2	*Amblyomma*
7086	Male	ResNet-50	macho cajennense (27).jpg	fold2	2	*Amblyomma*
7087	Male	ResNet-50	macho cajennense (3).jpg	fold2	2	*Amblyomma*
7088	Male	ResNet-50	macho cajennense (30).jpg	fold2	2	*Amblyomma*
7089	Male	ResNet-50	macho cajennense (43).jpg	fold2	2	*Amblyomma*
7090	Male	ResNet-50	macho cajennense (53).jpg	fold2	2	*Amblyomma*
7091	Male	ResNet-50	macho cajennense (59).jpg	fold2	2	*Amblyomma*
7092	Male	ResNet-50	macho cajennense (60).jpg	fold2	2	*Amblyomma*
7093	Male	ResNet-50	macho cajennense (61).jpg	fold2	2	*Amblyomma*
7094	Male	ResNet-50	macho cajennense (68).jpg	fold2	2	*Amblyomma*
7095	Male	ResNet-50	macho cajennense (70).jpg	fold2	2	*Amblyomma*
7096	Male	ResNet-50	macho cajennense (72).jpg	fold2	2	*Amblyomma*
7097	Male	ResNet-50	macho cajennense (8).jpg	fold2	2	*Amblyomma*
7098	Male	ResNet-50	macho cajennense (81).jpg	fold2	2	*Amblyomma*
7099	Male	ResNet-50	macho cajennense (9).jpg	fold2	2	*Amblyomma*
7100	Male	ResNet-50	macho dubitatum (1).jpg	fold2	3	*Amblyomma*

7101	Male	ResNet-50	macho dubitatum (13).jpg	fold2	3	*Amblyomma*
7102	Male	ResNet-50	macho dubitatum (29).jpg	fold2	3	*Amblyomma*
7103	Male	ResNet-50	macho dubitatum (30).jpg	fold2	3	*Amblyomma*
7104	Male	ResNet-50	macho dubitatum (35).jpg	fold2	3	*Amblyomma*
7105	Male	ResNet-50	macho dubitatum (43).jpg	fold2	3	*Amblyomma*
7106	Male	ResNet-50	macho dubitatum (46).jpg	fold2	3	*Amblyomma*
7107	Male	ResNet-50	macho dubitatum (48).jpg	fold2	3	*Amblyomma*
7108	Male	ResNet-50	macho dubitatum (5).JPG	fold2	3	*Amblyomma*
7109	Male	ResNet-50	macho dubitatum (53).jpg	fold2	3	*Amblyomma*
7110	Male	ResNet-50	macho dubitatum (55).jpg	fold2	3	*Amblyomma*
7111	Male	ResNet-50	macho dubitatum (56).jpg	fold2	3	*Amblyomma*
7112	Male	ResNet-50	macho dubitatum (60).jpg	fold2	3	*Amblyomma*
7113	Male	ResNet-50	macho dubitatum (72).jpg	fold2	3	*Amblyomma*
7114	Male	ResNet-50	macho dubitatum (75).jpg	fold2	3	*Amblyomma*
7115	Male	ResNet-50	macho ovale (18).jpg	fold2	4	*Amblyomma*
7116	Male	ResNet-50	macho ovale (27).JPG	fold2	4	*Amblyomma*
7117	Male	ResNet-50	macho ovale (33).jpg	fold2	4	*Amblyomma*
7118	Male	ResNet-50	macho ovale (35).jpg	fold2	4	*Amblyomma*
7119	Male	ResNet-50	macho ovale (36).jpg	fold2	4	*Amblyomma*
7120	Male	ResNet-50	macho ovale (37).jpg	fold2	4	*Amblyomma*
7121	Male	ResNet-50	macho ovale (39).jpg	fold2	4	*Amblyomma*
7122	Male	ResNet-50	macho ovale (40).jpg	fold2	4	*Amblyomma*
7123	Male	ResNet-50	macho ovale (6).jpg	fold2	4	*Amblyomma*
7124	Male	ResNet-50	macho sculptum (12).JPG	fold2	5	*Amblyomma*
7125	Male	ResNet-50	macho sculptum (21).JPG	fold2	5	*Amblyomma*
7126	Male	ResNet-50	macho sculptum (24).JPG	fold2	5	*Amblyomma*
7127	Male	ResNet-50	macho sculptum (26).JPG	fold2	5	*Amblyomma*
7128	Male	ResNet-50	macho sculptum (27).JPG	fold2	5	*Amblyomma*
7129	Male	ResNet-50	macho sculptum (36).JPG	fold2	5	*Amblyomma*
7130	Male	ResNet-50	macho sculptum (42).JPG	fold2	5	*Amblyomma*
7131	Male	ResNet-50	macho sculptum (55).JPG	fold2	5	*Amblyomma*
7132	Male	ResNet-50	macho sculptum (59).JPG	fold2	5	*Amblyomma*
7133	Male	ResNet-50	macho sculptum (67).JPG	fold2	5	*Amblyomma*
7134	Male	ResNet-50	macho sculptum (7).jpg	fold2	5	*Amblyomma*
7135	Male	ResNet-50	macho sculptum (70).JPG	fold2	5	*Amblyomma*
7136	Male	ResNet-50	macho sculptum (73).JPG	fold2	5	*Amblyomma*
7137	Male	ResNet-50	macho sculptum (78).JPG	fold2	5	*Amblyomma*
7138	Male	ResNet-50	macho sculptum (82).jpg	fold2	5	*Amblyomma*
7139	Male	ResNet-50	macho sculptum (86).jpg	fold2	5	*Amblyomma*
7140	Male	ResNet-50	macho sculptum (91).jpg	fold2	5	*Amblyomma*
7141	Male	ResNet-50	macho sculptum (92).jpg	fold2	5	*Amblyomma*
7142	Male	ResNet-50	macho triste (14).jpg	fold2	6	*Amblyomma*
7143	Male	ResNet-50	macho triste (15).jpg	fold2	6	*Amblyomma*
7144	Male	ResNet-50	macho triste (17).jpg	fold2	6	*Amblyomma*
7145	Male	ResNet-50	macho triste (20).jpg	fold2	6	*Amblyomma*
7146	Male	ResNet-50	macho triste (21).jpg	fold2	6	*Amblyomma*
7147	Male	ResNet-50	macho triste (24).jpg	fold2	6	*Amblyomma*
7148	Male	ResNet-50	macho triste (49).jpg	fold2	6	*Amblyomma*
7149	Male	ResNet-50	macho triste (50).jpg	fold2	6	*Amblyomma*
7150	Male	ResNet-50	macho triste (61).jpg	fold2	6	*Amblyomma*
7151	Male	ResNet-50	macho triste (62).jpg	fold2	6	*Amblyomma*
7152	Male	ResNet-50	macho triste (63).jpg	fold2	6	*Amblyomma*
7153	Male	ResNet-50	macho triste (64).jpg	fold2	6	*Amblyomma*
7154	Male	ResNet-50	macho triste (67).jpg	fold2	6	*Amblyomma*
7155	Male	ResNet-50	macho triste (68).jpg	fold2	6	*Amblyomma*
7156	Male	ResNet-50	macho triste (73).jpg	fold2	6	*Amblyomma*
7157	Male	ResNet-50	macho triste (77).jpg	fold2	6	*Amblyomma*
7158	Male	ResNet-50	macho triste (78).jpg	fold2	6	*Amblyomma*
7159	Male	ResNet-50	macho triste (82).jpg	fold2	6	*Amblyomma*
7160	Male	ResNet-50	macho triste (9).jpg	fold2	6	*Amblyomma*
7161	Male	ResNet-50	macho triste (90).jpg	fold2	6	*Amblyomma*
7162	Male	ResNet-50	macho triste (91).jpg	fold2	6	*Amblyomma*
7163	Male	ResNet-50	macho aureolatum (14).jpg	fold3	1	*Amblyomma*
7164	Male	ResNet-50	macho aureolatum (20).jpg	fold3	1	*Amblyomma*
7165	Male	ResNet-50	macho aureolatum (27).jpg	fold3	1	*Amblyomma*
7166	Male	ResNet-50	macho aureolatum (29).jpg	fold3	1	*Amblyomma*
7167	Male	ResNet-50	macho aureolatum (31).jpg	fold3	1	*Amblyomma*

7168	Male	ResNet-50	macho aureolatum (38).jpg	fold3	1	*Amblyomma*
7169	Male	ResNet-50	macho aureolatum (41).jpg	fold3	1	*Amblyomma*
7170	Male	ResNet-50	macho aureolatum (47).jpg	fold3	1	*Amblyomma*
7171	Male	ResNet-50	macho aureolatum (55).jpg	fold3	1	*Amblyomma*
7172	Male	ResNet-50	macho aureolatum (57).jpg	fold3	1	*Amblyomma*
7173	Male	ResNet-50	macho aureolatum (63).jpg	fold3	1	*Amblyomma*
7174	Male	ResNet-50	macho aureolatum (7).jpg	fold3	1	*Amblyomma*
7175	Male	ResNet-50	macho cajennense (11).jpg	fold3	2	*Amblyomma*
7176	Male	ResNet-50	macho cajennense (14).jpg	fold3	2	*Amblyomma*
7177	Male	ResNet-50	macho cajennense (15).jpg	fold3	2	*Amblyomma*
7178	Male	ResNet-50	macho cajennense (24).jpg	fold3	2	*Amblyomma*
7179	Male	ResNet-50	macho cajennense (29).jpg	fold3	2	*Amblyomma*
7180	Male	ResNet-50	macho cajennense (33).jpg	fold3	2	*Amblyomma*
7181	Male	ResNet-50	macho cajennense (38).jpg	fold3	2	*Amblyomma*
7182	Male	ResNet-50	macho cajennense (42).jpg	fold3	2	*Amblyomma*
7183	Male	ResNet-50	macho cajennense (46).jpg	fold3	2	*Amblyomma*
7184	Male	ResNet-50	macho cajennense (5).jpg	fold3	2	*Amblyomma*
7185	Male	ResNet-50	macho cajennense (54).jpg	fold3	2	*Amblyomma*
7186	Male	ResNet-50	macho cajennense (56).jpg	fold3	2	*Amblyomma*
7187	Male	ResNet-50	macho cajennense (6).jpg	fold3	2	*Amblyomma*
7188	Male	ResNet-50	macho cajennense (63).jpg	fold3	2	*Amblyomma*
7189	Male	ResNet-50	macho cajennense (65).jpg	fold3	2	*Amblyomma*
7190	Male	ResNet-50	macho cajennense (67).jpg	fold3	2	*Amblyomma*
7191	Male	ResNet-50	macho cajennense (7).jpg	fold3	2	*Amblyomma*
7192	Male	ResNet-50	macho dubitatum (14).jpg	fold3	3	*Amblyomma*
7193	Male	ResNet-50	macho dubitatum (20).jpg	fold3	3	*Amblyomma*
7194	Male	ResNet-50	macho dubitatum (23).jpg	fold3	3	*Amblyomma*
7195	Male	ResNet-50	macho dubitatum (25).jpg	fold3	3	*Amblyomma*
7196	Male	ResNet-50	macho dubitatum (26).jpg	fold3	3	*Amblyomma*
7197	Male	ResNet-50	macho dubitatum (36).jpg	fold3	3	*Amblyomma*
7198	Male	ResNet-50	macho dubitatum (37).jpg	fold3	3	*Amblyomma*
7199	Male	ResNet-50	macho dubitatum (41).jpg	fold3	3	*Amblyomma*
7200	Male	ResNet-50	macho dubitatum (42).jpg	fold3	3	*Amblyomma*
7201	Male	ResNet-50	macho dubitatum (52).jpg	fold3	3	*Amblyomma*
7202	Male	ResNet-50	macho dubitatum (59).jpg	fold3	3	*Amblyomma*
7203	Male	ResNet-50	macho dubitatum (65).jpg	fold3	3	*Amblyomma*
7204	Male	ResNet-50	macho dubitatum (66).jpg	fold3	3	*Amblyomma*
7205	Male	ResNet-50	macho dubitatum (67).jpg	fold3	3	*Amblyomma*
7206	Male	ResNet-50	macho dubitatum (73).jpg	fold3	3	*Amblyomma*
7207	Male	ResNet-50	macho ovale (10).jpg	fold3	4	*Amblyomma*
7208	Male	ResNet-50	macho ovale (11).jpg	fold3	4	*Amblyomma*
7209	Male	ResNet-50	macho ovale (19).jpg	fold3	4	*Amblyomma*
7210	Male	ResNet-50	macho ovale (22).jpg	fold3	4	*Amblyomma*
7211	Male	ResNet-50	macho ovale (28).JPG	fold3	4	*Amblyomma*
7212	Male	ResNet-50	macho ovale (31).jpg	fold3	4	*Amblyomma*
7213	Male	ResNet-50	macho ovale (5).jpg	fold3	4	*Amblyomma*
7214	Male	ResNet-50	macho ovale (8).jpg	fold3	4	*Amblyomma*
7215	Male	ResNet-50	macho ovale (9).jpg	fold3	4	*Amblyomma*
7216	Male	ResNet-50	macho sculptum (1).jpg	fold3	5	*Amblyomma*
7217	Male	ResNet-50	macho sculptum (13).JPG	fold3	5	*Amblyomma*
7218	Male	ResNet-50	macho sculptum (14).JPG	fold3	5	*Amblyomma*
7219	Male	ResNet-50	macho sculptum (18).JPG	fold3	5	*Amblyomma*
7220	Male	ResNet-50	macho sculptum (2).jpg	fold3	5	*Amblyomma*
7221	Male	ResNet-50	macho sculptum (22).JPG	fold3	5	*Amblyomma*
7222	Male	ResNet-50	macho sculptum (31).JPG	fold3	5	*Amblyomma*
7223	Male	ResNet-50	macho sculptum (34).JPG	fold3	5	*Amblyomma*
7224	Male	ResNet-50	macho sculptum (35).JPG	fold3	5	*Amblyomma*
7225	Male	ResNet-50	macho sculptum (37).JPG	fold3	5	*Amblyomma*
7226	Male	ResNet-50	macho sculptum (39).JPG	fold3	5	*Amblyomma*
7227	Male	ResNet-50	macho sculptum (44).JPG	fold3	5	*Amblyomma*
7228	Male	ResNet-50	macho sculptum (49).JPG	fold3	5	*Amblyomma*
7229	Male	ResNet-50	macho sculptum (57).JPG	fold3	5	*Amblyomma*
7230	Male	ResNet-50	macho sculptum (83).jpg	fold3	5	*Amblyomma*
7231	Male	ResNet-50	macho sculptum (84).jpg	fold3	5	*Amblyomma*
7232	Male	ResNet-50	macho sculptum (85).jpg	fold3	5	*Amblyomma*
7233	Male	ResNet-50	macho sculptum (87).jpg	fold3	5	*Amblyomma*
7234	Male	ResNet-50	macho sculptum (90).jpg	fold3	5	*Amblyomma*

7235	Male	ResNet-50	macho triste (1).jpg	fold3	6	*Amblyomma*
7236	Male	ResNet-50	macho triste (100).jpg	fold3	6	*Amblyomma*
7237	Male	ResNet-50	macho triste (101).jpg	fold3	6	*Amblyomma*
7238	Male	ResNet-50	macho triste (16).jpg	fold3	6	*Amblyomma*
7239	Male	ResNet-50	macho triste (26).jpg	fold3	6	*Amblyomma*
7240	Male	ResNet-50	macho triste (34).jpg	fold3	6	*Amblyomma*
7241	Male	ResNet-50	macho triste (35).jpg	fold3	6	*Amblyomma*
7242	Male	ResNet-50	macho triste (40).jpg	fold3	6	*Amblyomma*
7243	Male	ResNet-50	macho triste (44).jpg	fold3	6	*Amblyomma*
7244	Male	ResNet-50	macho triste (45).jpg	fold3	6	*Amblyomma*
7245	Male	ResNet-50	macho triste (51).jpg	fold3	6	*Amblyomma*
7246	Male	ResNet-50	macho triste (59).jpg	fold3	6	*Amblyomma*
7247	Male	ResNet-50	macho triste (6).jpg	fold3	6	*Amblyomma*
7248	Male	ResNet-50	macho triste (66).jpg	fold3	6	*Amblyomma*
7249	Male	ResNet-50	macho triste (71).jpg	fold3	6	*Amblyomma*
7250	Male	ResNet-50	macho triste (76).jpg	fold3	6	*Amblyomma*
7251	Male	ResNet-50	macho triste (80).jpg	fold3	6	*Amblyomma*
7252	Male	ResNet-50	macho triste (86).jpg	fold3	6	*Amblyomma*
7253	Male	ResNet-50	macho triste (87).jpg	fold3	6	*Amblyomma*
7254	Male	ResNet-50	macho triste (94).jpg	fold3	6	*Amblyomma*
7255	Male	ResNet-50	macho aureolatum (1).jpg	fold4	1	*Amblyomma*
7256	Male	ResNet-50	macho aureolatum (10).jpg	fold4	1	*Amblyomma*
7257	Male	ResNet-50	macho aureolatum (15).jpg	fold4	1	*Amblyomma*
7258	Male	ResNet-50	macho aureolatum (2).jpg	fold4	1	*Amblyomma*
7259	Male	ResNet-50	macho aureolatum (23).jpg	fold4	1	*Amblyomma*
7260	Male	ResNet-50	macho aureolatum (24).jpg	fold4	1	*Amblyomma*
7261	Male	ResNet-50	macho aureolatum (25).jpg	fold4	1	*Amblyomma*
7262	Male	ResNet-50	macho aureolatum (33).jpg	fold4	1	*Amblyomma*
7263	Male	ResNet-50	macho aureolatum (37).jpg	fold4	1	*Amblyomma*
7264	Male	ResNet-50	macho aureolatum (39).jpg	fold4	1	*Amblyomma*
7265	Male	ResNet-50	macho aureolatum (4).jpg	fold4	1	*Amblyomma*
7266	Male	ResNet-50	macho aureolatum (48).jpg	fold4	1	*Amblyomma*
7267	Male	ResNet-50	macho aureolatum (60).jpg	fold4	1	*Amblyomma*
7268	Male	ResNet-50	macho cajennense (13).jpg	fold4	2	*Amblyomma*
7269	Male	ResNet-50	macho cajennense (16).jpg	fold4	2	*Amblyomma*
7270	Male	ResNet-50	macho cajennense (19).jpg	fold4	2	*Amblyomma*
7271	Male	ResNet-50	macho cajennense (20).jpg	fold4	2	*Amblyomma*
7272	Male	ResNet-50	macho cajennense (21).jpg	fold4	2	*Amblyomma*
7273	Male	ResNet-50	macho cajennense (28).jpg	fold4	2	*Amblyomma*
7274	Male	ResNet-50	macho cajennense (31).jpg	fold4	2	*Amblyomma*
7275	Male	ResNet-50	macho cajennense (32).jpg	fold4	2	*Amblyomma*
7276	Male	ResNet-50	macho cajennense (40).jpg	fold4	2	*Amblyomma*
7277	Male	ResNet-50	macho cajennense (41).jpg	fold4	2	*Amblyomma*
7278	Male	ResNet-50	macho cajennense (45).jpg	fold4	2	*Amblyomma*
7279	Male	ResNet-50	macho cajennense (47).jpg	fold4	2	*Amblyomma*
7280	Male	ResNet-50	macho cajennense (55).jpg	fold4	2	*Amblyomma*
7281	Male	ResNet-50	macho cajennense (58).jpg	fold4	2	*Amblyomma*
7282	Male	ResNet-50	macho cajennense (79).jpg	fold4	2	*Amblyomma*
7283	Male	ResNet-50	macho cajennense (80).jpg	fold4	2	*Amblyomma*
7284	Male	ResNet-50	macho dubitatum (10).jpg	fold4	3	*Amblyomma*
7285	Male	ResNet-50	macho dubitatum (12).jpg	fold4	3	*Amblyomma*
7286	Male	ResNet-50	macho dubitatum (15).jpg	fold4	3	*Amblyomma*
7287	Male	ResNet-50	macho dubitatum (28).jpg	fold4	3	*Amblyomma*
7288	Male	ResNet-50	macho dubitatum (3).jpg	fold4	3	*Amblyomma*
7289	Male	ResNet-50	macho dubitatum (32).jpg	fold4	3	*Amblyomma*
7290	Male	ResNet-50	macho dubitatum (33).jpg	fold4	3	*Amblyomma*
7291	Male	ResNet-50	macho dubitatum (39).jpg	fold4	3	*Amblyomma*
7292	Male	ResNet-50	macho dubitatum (4).jpg	fold4	3	*Amblyomma*
7293	Male	ResNet-50	macho dubitatum (54).jpg	fold4	3	*Amblyomma*
7294	Male	ResNet-50	macho dubitatum (6).JPG	fold4	3	*Amblyomma*
7295	Male	ResNet-50	macho dubitatum (61).jpg	fold4	3	*Amblyomma*
7296	Male	ResNet-50	macho dubitatum (63).jpg	fold4	3	*Amblyomma*
7297	Male	ResNet-50	macho dubitatum (7).JPG	fold4	3	*Amblyomma*
7298	Male	ResNet-50	macho dubitatum (71).jpg	fold4	3	*Amblyomma*
7299	Male	ResNet-50	macho ovale (1).jpg	fold4	4	*Amblyomma*
7300	Male	ResNet-50	macho ovale (23).jpg	fold4	4	*Amblyomma*
7301	Male	ResNet-50	macho ovale (26).jpg	fold4	4	*Amblyomma*

7302	Male	ResNet-50	macho ovale (29).jpg	fold4	4	*Amblyomma*
7303	Male	ResNet-50	macho ovale (34).jpg	fold4	4	*Amblyomma*
7304	Male	ResNet-50	macho ovale (38).jpg	fold4	4	*Amblyomma*
7305	Male	ResNet-50	macho ovale (4).jpg	fold4	4	*Amblyomma*
7306	Male	ResNet-50	macho ovale (42).jpg	fold4	4	*Amblyomma*
7307	Male	ResNet-50	macho sculptum (10).JPG	fold4	5	*Amblyomma*
7308	Male	ResNet-50	macho sculptum (11).JPG	fold4	5	*Amblyomma*
7309	Male	ResNet-50	macho sculptum (15).JPG	fold4	5	*Amblyomma*
7310	Male	ResNet-50	macho sculptum (17).JPG	fold4	5	*Amblyomma*
7311	Male	ResNet-50	macho sculptum (33).JPG	fold4	5	*Amblyomma*
7312	Male	ResNet-50	macho sculptum (38).JPG	fold4	5	*Amblyomma*
7313	Male	ResNet-50	macho sculptum (47).JPG	fold4	5	*Amblyomma*
7314	Male	ResNet-50	macho sculptum (48).JPG	fold4	5	*Amblyomma*
7315	Male	ResNet-50	macho sculptum (51).JPG	fold4	5	*Amblyomma*
7316	Male	ResNet-50	macho sculptum (52).JPG	fold4	5	*Amblyomma*
7317	Male	ResNet-50	macho sculptum (61).JPG	fold4	5	*Amblyomma*
7318	Male	ResNet-50	macho sculptum (66).JPG	fold4	5	*Amblyomma*
7319	Male	ResNet-50	macho sculptum (68).JPG	fold4	5	*Amblyomma*
7320	Male	ResNet-50	macho sculptum (69).JPG	fold4	5	*Amblyomma*
7321	Male	ResNet-50	macho sculptum (72).JPG	fold4	5	*Amblyomma*
7322	Male	ResNet-50	macho sculptum (76).JPG	fold4	5	*Amblyomma*
7323	Male	ResNet-50	macho sculptum (77).JPG	fold4	5	*Amblyomma*
7324	Male	ResNet-50	macho sculptum (80).JPG	fold4	5	*Amblyomma*
7325	Male	ResNet-50	macho sculptum (9).JPG	fold4	5	*Amblyomma*
7326	Male	ResNet-50	macho triste (102).jpg	fold4	6	*Amblyomma*
7327	Male	ResNet-50	macho triste (2).jpg	fold4	6	*Amblyomma*
7328	Male	ResNet-50	macho triste (31).jpg	fold4	6	*Amblyomma*
7329	Male	ResNet-50	macho triste (37).jpg	fold4	6	*Amblyomma*
7330	Male	ResNet-50	macho triste (39).jpg	fold4	6	*Amblyomma*
7331	Male	ResNet-50	macho triste (41).jpg	fold4	6	*Amblyomma*
7332	Male	ResNet-50	macho triste (42).jpg	fold4	6	*Amblyomma*
7333	Male	ResNet-50	macho triste (46).jpg	fold4	6	*Amblyomma*
7334	Male	ResNet-50	macho triste (47).jpg	fold4	6	*Amblyomma*
7335	Male	ResNet-50	macho triste (54).jpg	fold4	6	*Amblyomma*
7336	Male	ResNet-50	macho triste (58).jpg	fold4	6	*Amblyomma*
7337	Male	ResNet-50	macho triste (65).jpg	fold4	6	*Amblyomma*
7338	Male	ResNet-50	macho triste (69).jpg	fold4	6	*Amblyomma*
7339	Male	ResNet-50	macho triste (70).jpg	fold4	6	*Amblyomma*
7340	Male	ResNet-50	macho triste (72).jpg	fold4	6	*Amblyomma*
7341	Male	ResNet-50	macho triste (79).jpg	fold4	6	*Amblyomma*
7342	Male	ResNet-50	macho triste (81).jpg	fold4	6	*Amblyomma*
7343	Male	ResNet-50	macho triste (84).jpg	fold4	6	*Amblyomma*
7344	Male	ResNet-50	macho triste (85).jpg	fold4	6	*Amblyomma*
7345	Male	ResNet-50	macho triste (98).jpg	fold4	6	*Amblyomma*
7346	Male	ResNet-50	macho triste (99).jpg	fold4	6	*Amblyomma*
7347	Male	ResNet-50	macho aureolatum (13).jpg	fold5	1	*Amblyomma*
7348	Male	ResNet-50	macho aureolatum (18).jpg	fold5	1	*Amblyomma*
7349	Male	ResNet-50	macho aureolatum (21).jpg	fold5	1	*Amblyomma*
7350	Male	ResNet-50	macho aureolatum (3).jpg	fold5	1	*Amblyomma*
7351	Male	ResNet-50	macho aureolatum (30).jpg	fold5	1	*Amblyomma*
7352	Male	ResNet-50	macho aureolatum (34).jpg	fold5	1	*Amblyomma*
7353	Male	ResNet-50	macho aureolatum (40).jpg	fold5	1	*Amblyomma*
7354	Male	ResNet-50	macho aureolatum (43).jpg	fold5	1	*Amblyomma*
7355	Male	ResNet-50	macho aureolatum (45).jpg	fold5	1	*Amblyomma*
7356	Male	ResNet-50	macho aureolatum (5).jpg	fold5	1	*Amblyomma*
7357	Male	ResNet-50	macho aureolatum (53).jpg	fold5	1	*Amblyomma*
7358	Male	ResNet-50	macho aureolatum (54).jpg	fold5	1	*Amblyomma*
7359	Male	ResNet-50	macho aureolatum (8).jpg	fold5	1	*Amblyomma*
7360	Male	ResNet-50	macho cajennense (18).jpg	fold5	2	*Amblyomma*
7361	Male	ResNet-50	macho cajennense (22).jpg	fold5	2	*Amblyomma*
7362	Male	ResNet-50	macho cajennense (25).jpg	fold5	2	*Amblyomma*
7363	Male	ResNet-50	macho cajennense (35).jpg	fold5	2	*Amblyomma*
7364	Male	ResNet-50	macho cajennense (4).jpg	fold5	2	*Amblyomma*
7365	Male	ResNet-50	macho cajennense (44).jpg	fold5	2	*Amblyomma*
7366	Male	ResNet-50	macho cajennense (48).jpg	fold5	2	*Amblyomma*
7367	Male	ResNet-50	macho cajennense (49).jpg	fold5	2	*Amblyomma*
7368	Male	ResNet-50	macho cajennense (50).jpg	fold5	2	*Amblyomma*

7369	Male	ResNet-50	macho cajennense (51).jpg	fold5	2	*Amblyomma*
7370	Male	ResNet-50	macho cajennense (57).jpg	fold5	2	*Amblyomma*
7371	Male	ResNet-50	macho cajennense (62).jpg	fold5	2	*Amblyomma*
7372	Male	ResNet-50	macho cajennense (69).jpg	fold5	2	*Amblyomma*
7373	Male	ResNet-50	macho cajennense (74).jpg	fold5	2	*Amblyomma*
7374	Male	ResNet-50	macho cajennense (75).jpg	fold5	2	*Amblyomma*
7375	Male	ResNet-50	macho cajennense (77).jpg	fold5	2	*Amblyomma*
7376	Male	ResNet-50	macho cajennense (78).jpg	fold5	2	*Amblyomma*
7377	Male	ResNet-50	macho dubitatum (17).jpg	fold5	3	*Amblyomma*
7378	Male	ResNet-50	macho dubitatum (19).jpg	fold5	3	*Amblyomma*
7379	Male	ResNet-50	macho dubitatum (24).jpg	fold5	3	*Amblyomma*
7380	Male	ResNet-50	macho dubitatum (27).jpg	fold5	3	*Amblyomma*
7381	Male	ResNet-50	macho dubitatum (34).jpg	fold5	3	*Amblyomma*
7382	Male	ResNet-50	macho dubitatum (44).jpg	fold5	3	*Amblyomma*
7383	Male	ResNet-50	macho dubitatum (45).jpg	fold5	3	*Amblyomma*
7384	Male	ResNet-50	macho dubitatum (49).jpg	fold5	3	*Amblyomma*
7385	Male	ResNet-50	macho dubitatum (51).jpg	fold5	3	*Amblyomma*
7386	Male	ResNet-50	macho dubitatum (58).jpg	fold5	3	*Amblyomma*
7387	Male	ResNet-50	macho dubitatum (62).jpg	fold5	3	*Amblyomma*
7388	Male	ResNet-50	macho dubitatum (64).jpg	fold5	3	*Amblyomma*
7389	Male	ResNet-50	macho dubitatum (68).jpg	fold5	3	*Amblyomma*
7390	Male	ResNet-50	macho dubitatum (70).jpg	fold5	3	*Amblyomma*
7391	Male	ResNet-50	macho dubitatum (8).JPG	fold5	3	*Amblyomma*
7392	Male	ResNet-50	macho ovale (13).jpg	fold5	4	*Amblyomma*
7393	Male	ResNet-50	macho ovale (16).jpg	fold5	4	*Amblyomma*
7394	Male	ResNet-50	macho ovale (17).jpg	fold5	4	*Amblyomma*
7395	Male	ResNet-50	macho ovale (21).jpg	fold5	4	*Amblyomma*
7396	Male	ResNet-50	macho ovale (24).jpg	fold5	4	*Amblyomma*
7397	Male	ResNet-50	macho ovale (25).jpg	fold5	4	*Amblyomma*
7398	Male	ResNet-50	macho ovale (3).jpg	fold5	4	*Amblyomma*
7399	Male	ResNet-50	macho ovale (32).jpg	fold5	4	*Amblyomma*
7400	Male	ResNet-50	macho sculptum (19).JPG	fold5	5	*Amblyomma*
7401	Male	ResNet-50	macho sculptum (25).JPG	fold5	5	*Amblyomma*
7402	Male	ResNet-50	macho sculptum (28).JPG	fold5	5	*Amblyomma*
7403	Male	ResNet-50	macho sculptum (29).JPG	fold5	5	*Amblyomma*
7404	Male	ResNet-50	macho sculptum (32).JPG	fold5	5	*Amblyomma*
7405	Male	ResNet-50	macho sculptum (41).JPG	fold5	5	*Amblyomma*
7406	Male	ResNet-50	macho sculptum (50).JPG	fold5	5	*Amblyomma*
7407	Male	ResNet-50	macho sculptum (54).JPG	fold5	5	*Amblyomma*
7408	Male	ResNet-50	macho sculptum (58).JPG	fold5	5	*Amblyomma*
7409	Male	ResNet-50	macho sculptum (6).jpg	fold5	5	*Amblyomma*
7410	Male	ResNet-50	macho sculptum (60).JPG	fold5	5	*Amblyomma*
7411	Male	ResNet-50	macho sculptum (64).JPG	fold5	5	*Amblyomma*
7412	Male	ResNet-50	macho sculptum (65).JPG	fold5	5	*Amblyomma*
7413	Male	ResNet-50	macho sculptum (74).JPG	fold5	5	*Amblyomma*
7414	Male	ResNet-50	macho sculptum (75).JPG	fold5	5	*Amblyomma*
7415	Male	ResNet-50	macho sculptum (79).JPG	fold5	5	*Amblyomma*
7416	Male	ResNet-50	macho sculptum (8).JPG	fold5	5	*Amblyomma*
7417	Male	ResNet-50	macho sculptum (88).jpg	fold5	5	*Amblyomma*
7418	Male	ResNet-50	macho triste (103).jpg	fold5	6	*Amblyomma*
7419	Male	ResNet-50	macho triste (11).jpg	fold5	6	*Amblyomma*
7420	Male	ResNet-50	macho triste (12).jpg	fold5	6	*Amblyomma*
7421	Male	ResNet-50	macho triste (22).jpg	fold5	6	*Amblyomma*
7422	Male	ResNet-50	macho triste (25).jpg	fold5	6	*Amblyomma*
7423	Male	ResNet-50	macho triste (27).jpg	fold5	6	*Amblyomma*
7424	Male	ResNet-50	macho triste (28).jpg	fold5	6	*Amblyomma*
7425	Male	ResNet-50	macho triste (30).jpg	fold5	6	*Amblyomma*
7426	Male	ResNet-50	macho triste (33).jpg	fold5	6	*Amblyomma*
7427	Male	ResNet-50	macho triste (36).jpg	fold5	6	*Amblyomma*
7428	Male	ResNet-50	macho triste (4).jpg	fold5	6	*Amblyomma*
7429	Male	ResNet-50	macho triste (53).jpg	fold5	6	*Amblyomma*
7430	Male	ResNet-50	macho triste (55).jpg	fold5	6	*Amblyomma*
7431	Male	ResNet-50	macho triste (57).jpg	fold5	6	*Amblyomma*
7432	Male	ResNet-50	macho triste (60).jpg	fold5	6	*Amblyomma*
7433	Male	ResNet-50	macho triste (74).jpg	fold5	6	*Amblyomma*
7434	Male	ResNet-50	macho triste (8).jpg	fold5	6	*Amblyomma*
7435	Male	ResNet-50	macho triste (89).jpg	fold5	6	*Amblyomma*

7436	Male	ResNet-50	macho triste (95).jpg	fold5	6	*Amblyomma*
7437	Male	ResNet-50	macho triste (96).jpg	fold5	6	*Amblyomma*
7438	Male	ResNet-50	macho triste (97).jpg	fold5	6	*Amblyomma*
7439	Dorsal	ResNet-50	dorsal aureolatum (11).jpg	fold1	1	*Amblyomma*
7440	Dorsal	ResNet-50	dorsal aureolatum (17).jpg	fold1	1	*Amblyomma*
7441	Dorsal	ResNet-50	dorsal aureolatum (23).jpg	fold1	1	*Amblyomma*
7442	Dorsal	ResNet-50	dorsal aureolatum (27).jpg	fold1	1	*Amblyomma*
7443	Dorsal	ResNet-50	dorsal aureolatum (30).jpg	fold1	1	*Amblyomma*
7444	Dorsal	ResNet-50	dorsal aureolatum (46).jpg	fold1	1	*Amblyomma*
7445	Dorsal	ResNet-50	dorsal aureolatum (48).jpg	fold1	1	*Amblyomma*
7446	Dorsal	ResNet-50	dorsal aureolatum (52).jpg	fold1	1	*Amblyomma*
7447	Dorsal	ResNet-50	dorsal aureolatum (55).jpg	fold1	1	*Amblyomma*
7448	Dorsal	ResNet-50	dorsal aureolatum (58).jpg	fold1	1	*Amblyomma*
7449	Dorsal	ResNet-50	dorsal aureolatum (61).jpg	fold1	1	*Amblyomma*
7450	Dorsal	ResNet-50	dorsal aureolatum (8).jpg	fold1	1	*Amblyomma*
7451	Dorsal	ResNet-50	dorsal cajennense (25).jpg	fold1	2	*Amblyomma*
7452	Dorsal	ResNet-50	dorsal cajennense (26).jpg	fold1	2	*Amblyomma*
7453	Dorsal	ResNet-50	dorsal cajennense (3).jpg	fold1	2	*Amblyomma*
7454	Dorsal	ResNet-50	dorsal cajennense (32).jpg	fold1	2	*Amblyomma*
7455	Dorsal	ResNet-50	dorsal cajennense (44).jpg	fold1	2	*Amblyomma*
7456	Dorsal	ResNet-50	dorsal cajennense (47).jpg	fold1	2	*Amblyomma*
7457	Dorsal	ResNet-50	dorsal cajennense (50).jpg	fold1	2	*Amblyomma*
7458	Dorsal	ResNet-50	dorsal cajennense (52).jpg	fold1	2	*Amblyomma*
7459	Dorsal	ResNet-50	dorsal cajennense (54).jpg	fold1	2	*Amblyomma*
7460	Dorsal	ResNet-50	dorsal cajennense (58).jpg	fold1	2	*Amblyomma*
7461	Dorsal	ResNet-50	dorsal cajennense (65).jpg	fold1	2	*Amblyomma*
7462	Dorsal	ResNet-50	dorsal cajennense (66).jpg	fold1	2	*Amblyomma*
7463	Dorsal	ResNet-50	dorsal cajennense (7).jpg	fold1	2	*Amblyomma*
7464	Dorsal	ResNet-50	dorsal cajennense (75).jpg	fold1	2	*Amblyomma*
7465	Dorsal	ResNet-50	dorsal cajennense (77).jpg	fold1	2	*Amblyomma*
7466	Dorsal	ResNet-50	dorsal cajennense (80).jpg	fold1	2	*Amblyomma*
7467	Dorsal	ResNet-50	dorsal dubitatum (10).jpg	fold1	3	*Amblyomma*
7468	Dorsal	ResNet-50	dorsal dubitatum (18).jpg	fold1	3	*Amblyomma*
7469	Dorsal	ResNet-50	dorsal dubitatum (19).jpg	fold1	3	*Amblyomma*
7470	Dorsal	ResNet-50	dorsal dubitatum (28).jpg	fold1	3	*Amblyomma*
7471	Dorsal	ResNet-50	dorsal dubitatum (32).jpg	fold1	3	*Amblyomma*
7472	Dorsal	ResNet-50	dorsal dubitatum (40).jpg	fold1	3	*Amblyomma*
7473	Dorsal	ResNet-50	dorsal dubitatum (46).jpg	fold1	3	*Amblyomma*
7474	Dorsal	ResNet-50	dorsal dubitatum (49).jpg	fold1	3	*Amblyomma*
7475	Dorsal	ResNet-50	dorsal dubitatum (55).jpg	fold1	3	*Amblyomma*
7476	Dorsal	ResNet-50	dorsal dubitatum (6).jpg	fold1	3	*Amblyomma*
7477	Dorsal	ResNet-50	dorsal dubitatum (9).JPG	fold1	3	*Amblyomma*
7478	Dorsal	ResNet-50	dorsal ovale (10).jpg	fold1	4	*Amblyomma*
7479	Dorsal	ResNet-50	dorsal ovale (14).jpg	fold1	4	*Amblyomma*
7480	Dorsal	ResNet-50	dorsal ovale (20).jpg	fold1	4	*Amblyomma*
7481	Dorsal	ResNet-50	dorsal ovale (21).jpg	fold1	4	*Amblyomma*
7482	Dorsal	ResNet-50	dorsal ovale (26).jpg	fold1	4	*Amblyomma*
7483	Dorsal	ResNet-50	dorsal ovale (29).jpg	fold1	4	*Amblyomma*
7484	Dorsal	ResNet-50	dorsal ovale (44).jpg	fold1	4	*Amblyomma*
7485	Dorsal	ResNet-50	dorsal ovale (48).jpg	fold1	4	*Amblyomma*
7486	Dorsal	ResNet-50	dorsal ovale (51).jpg	fold1	4	*Amblyomma*
7487	Dorsal	ResNet-50	dorsal ovale (53).jpg	fold1	4	*Amblyomma*
7488	Dorsal	ResNet-50	dorsal ovale (61).jpg	fold1	4	*Amblyomma*
7489	Dorsal	ResNet-50	dorsal ovale (7).jpg	fold1	4	*Amblyomma*
7490	Dorsal	ResNet-50	dorsal sculptum (14).JPG	fold1	5	*Amblyomma*
7491	Dorsal	ResNet-50	dorsal sculptum (20).JPG	fold1	5	*Amblyomma*
7492	Dorsal	ResNet-50	dorsal sculptum (22).JPG	fold1	5	*Amblyomma*
7493	Dorsal	ResNet-50	dorsal sculptum (23).JPG	fold1	5	*Amblyomma*
7494	Dorsal	ResNet-50	dorsal sculptum (25).JPG	fold1	5	*Amblyomma*
7495	Dorsal	ResNet-50	dorsal sculptum (29).jpg	fold1	5	*Amblyomma*
7496	Dorsal	ResNet-50	dorsal sculptum (37).JPG	fold1	5	*Amblyomma*
7497	Dorsal	ResNet-50	dorsal sculptum (38).JPG	fold1	5	*Amblyomma*
7498	Dorsal	ResNet-50	dorsal sculptum (42).JPG	fold1	5	*Amblyomma*
7499	Dorsal	ResNet-50	dorsal sculptum (5).JPG	fold1	5	*Amblyomma*
7500	Dorsal	ResNet-50	dorsal sculptum (51).JPG	fold1	5	*Amblyomma*
7501	Dorsal	ResNet-50	dorsal sculptum (52).JPG	fold1	5	*Amblyomma*
7502	Dorsal	ResNet-50	dorsal sculptum (59).JPG	fold1	5	*Amblyomma*

7503	Dorsal	ResNet-50	dorsal sculptum (72).JPG	fold1	5	*Amblyomma*
7504	Dorsal	ResNet-50	dorsal sculptum (75).JPG	fold1	5	*Amblyomma*
7505	Dorsal	ResNet-50	dorsal sculptum (9).JPG	fold1	5	*Amblyomma*
7506	Dorsal	ResNet-50	dorsal triste (19).jpg	fold1	6	*Amblyomma*
7507	Dorsal	ResNet-50	dorsal triste (2).jpg	fold1	6	*Amblyomma*
7508	Dorsal	ResNet-50	dorsal triste (20).jpg	fold1	6	*Amblyomma*
7509	Dorsal	ResNet-50	dorsal triste (24).jpg	fold1	6	*Amblyomma*
7510	Dorsal	ResNet-50	dorsal triste (25).jpg	fold1	6	*Amblyomma*
7511	Dorsal	ResNet-50	dorsal triste (26).jpg	fold1	6	*Amblyomma*
7512	Dorsal	ResNet-50	dorsal triste (45).jpg	fold1	6	*Amblyomma*
7513	Dorsal	ResNet-50	dorsal triste (46).jpg	fold1	6	*Amblyomma*
7514	Dorsal	ResNet-50	dorsal triste (47).jpg	fold1	6	*Amblyomma*
7515	Dorsal	ResNet-50	dorsal triste (51).jpg	fold1	6	*Amblyomma*
7516	Dorsal	ResNet-50	dorsal triste (6).jpg	fold1	6	*Amblyomma*
7517	Dorsal	ResNet-50	dorsal triste (61).jpg	fold1	6	*Amblyomma*
7518	Dorsal	ResNet-50	dorsal triste (63).jpg	fold1	6	*Amblyomma*
7519	Dorsal	ResNet-50	dorsal triste (72).jpg	fold1	6	*Amblyomma*
7520	Dorsal	ResNet-50	dorsal triste (74).jpg	fold1	6	*Amblyomma*
7521	Dorsal	ResNet-50	dorsal triste (79).jpg	fold1	6	*Amblyomma*
7522	Dorsal	ResNet-50	dorsal aureolatum (18).jpg	fold2	1	*Amblyomma*
7523	Dorsal	ResNet-50	dorsal aureolatum (21).jpg	fold2	1	*Amblyomma*
7524	Dorsal	ResNet-50	dorsal aureolatum (28).jpg	fold2	1	*Amblyomma*
7525	Dorsal	ResNet-50	dorsal aureolatum (29).jpg	fold2	1	*Amblyomma*
7526	Dorsal	ResNet-50	dorsal aureolatum (32).jpg	fold2	1	*Amblyomma*
7527	Dorsal	ResNet-50	dorsal aureolatum (38).jpg	fold2	1	*Amblyomma*
7528	Dorsal	ResNet-50	dorsal aureolatum (43).jpg	fold2	1	*Amblyomma*
7529	Dorsal	ResNet-50	dorsal aureolatum (44).jpg	fold2	1	*Amblyomma*
7530	Dorsal	ResNet-50	dorsal aureolatum (49).jpg	fold2	1	*Amblyomma*
7531	Dorsal	ResNet-50	dorsal aureolatum (5).jpg	fold2	1	*Amblyomma*
7532	Dorsal	ResNet-50	dorsal aureolatum (51).jpg	fold2	1	*Amblyomma*
7533	Dorsal	ResNet-50	dorsal aureolatum (6).jpg	fold2	1	*Amblyomma*
7534	Dorsal	ResNet-50	dorsal aureolatum (62).jpg	fold2	1	*Amblyomma*
7535	Dorsal	ResNet-50	dorsal cajennense (15).jpg	fold2	2	*Amblyomma*
7536	Dorsal	ResNet-50	dorsal cajennense (16).jpg	fold2	2	*Amblyomma*
7537	Dorsal	ResNet-50	dorsal cajennense (23).jpg	fold2	2	*Amblyomma*
7538	Dorsal	ResNet-50	dorsal cajennense (27).jpg	fold2	2	*Amblyomma*
7539	Dorsal	ResNet-50	dorsal cajennense (31).jpg	fold2	2	*Amblyomma*
7540	Dorsal	ResNet-50	dorsal cajennense (37).jpg	fold2	2	*Amblyomma*
7541	Dorsal	ResNet-50	dorsal cajennense (43).jpg	fold2	2	*Amblyomma*
7542	Dorsal	ResNet-50	dorsal cajennense (51).jpg	fold2	2	*Amblyomma*
7543	Dorsal	ResNet-50	dorsal cajennense (59).jpg	fold2	2	*Amblyomma*
7544	Dorsal	ResNet-50	dorsal cajennense (61).jpg	fold2	2	*Amblyomma*
7545	Dorsal	ResNet-50	dorsal cajennense (72).jpg	fold2	2	*Amblyomma*
7546	Dorsal	ResNet-50	dorsal cajennense (73).jpg	fold2	2	*Amblyomma*
7547	Dorsal	ResNet-50	dorsal cajennense (74).jpg	fold2	2	*Amblyomma*
7548	Dorsal	ResNet-50	dorsal cajennense (8).jpg	fold2	2	*Amblyomma*
7549	Dorsal	ResNet-50	dorsal cajennense (83).jpg	fold2	2	*Amblyomma*
7550	Dorsal	ResNet-50	dorsal cajennense (9).jpg	fold2	2	*Amblyomma*
7551	Dorsal	ResNet-50	dorsal dubitatum (14).jpg	fold2	3	*Amblyomma*
7552	Dorsal	ResNet-50	dorsal dubitatum (15).jpg	fold2	3	*Amblyomma*
7553	Dorsal	ResNet-50	dorsal dubitatum (2).jpg	fold2	3	*Amblyomma*
7554	Dorsal	ResNet-50	dorsal dubitatum (34).jpg	fold2	3	*Amblyomma*
7555	Dorsal	ResNet-50	dorsal dubitatum (36).jpg	fold2	3	*Amblyomma*
7556	Dorsal	ResNet-50	dorsal dubitatum (37).jpg	fold2	3	*Amblyomma*
7557	Dorsal	ResNet-50	dorsal dubitatum (43).jpg	fold2	3	*Amblyomma*
7558	Dorsal	ResNet-50	dorsal dubitatum (44).jpg	fold2	3	*Amblyomma*
7559	Dorsal	ResNet-50	dorsal dubitatum (47).jpg	fold2	3	*Amblyomma*
7560	Dorsal	ResNet-50	dorsal dubitatum (5).jpg	fold2	3	*Amblyomma*
7561	Dorsal	ResNet-50	dorsal dubitatum (50).jpg	fold2	3	*Amblyomma*
7562	Dorsal	ResNet-50	dorsal dubitatum (7).jpg	fold2	3	*Amblyomma*
7563	Dorsal	ResNet-50	dorsal ovale (15).jpg	fold2	4	*Amblyomma*
7564	Dorsal	ResNet-50	dorsal ovale (18).jpg	fold2	4	*Amblyomma*
7565	Dorsal	ResNet-50	dorsal ovale (22).jpg	fold2	4	*Amblyomma*
7566	Dorsal	ResNet-50	dorsal ovale (23).jpg	fold2	4	*Amblyomma*
7567	Dorsal	ResNet-50	dorsal ovale (27).jpg	fold2	4	*Amblyomma*
7568	Dorsal	ResNet-50	dorsal ovale (33).jpg	fold2	4	*Amblyomma*
7569	Dorsal	ResNet-50	dorsal ovale (38).JPG	fold2	4	*Amblyomma*

7570	Dorsal	ResNet-50	dorsal ovale (41).jpg	fold2	4 *Amblyomma*
7571	Dorsal	ResNet-50	dorsal ovale (42).jpg	fold2	4 *Amblyomma*
7572	Dorsal	ResNet-50	dorsal ovale (49).jpg	fold2	4 *Amblyomma*
7573	Dorsal	ResNet-50	dorsal ovale (5).jpg	fold2	4 *Amblyomma*
7574	Dorsal	ResNet-50	dorsal ovale (6).jpg	fold2	4 *Amblyomma*
7575	Dorsal	ResNet-50	dorsal sculptum (1).jpg	fold2	5 *Amblyomma*
7576	Dorsal	ResNet-50	dorsal sculptum (13).JPG	fold2	5 *Amblyomma*
7577	Dorsal	ResNet-50	dorsal sculptum (17).JPG	fold2	5 *Amblyomma*
7578	Dorsal	ResNet-50	dorsal sculptum (26).JPG	fold2	5 *Amblyomma*
7579	Dorsal	ResNet-50	dorsal sculptum (27).JPG	fold2	5 *Amblyomma*
7580	Dorsal	ResNet-50	dorsal sculptum (31).jpg	fold2	5 *Amblyomma*
7581	Dorsal	ResNet-50	dorsal sculptum (36).jpg	fold2	5 *Amblyomma*
7582	Dorsal	ResNet-50	dorsal sculptum (40).JPG	fold2	5 *Amblyomma*
7583	Dorsal	ResNet-50	dorsal sculptum (41).JPG	fold2	5 *Amblyomma*
7584	Dorsal	ResNet-50	dorsal sculptum (50).JPG	fold2	5 *Amblyomma*
7585	Dorsal	ResNet-50	dorsal sculptum (57).JPG	fold2	5 *Amblyomma*
7586	Dorsal	ResNet-50	dorsal sculptum (58).JPG	fold2	5 *Amblyomma*
7587	Dorsal	ResNet-50	dorsal sculptum (63).JPG	fold2	5 *Amblyomma*
7588	Dorsal	ResNet-50	dorsal sculptum (65).JPG	fold2	5 *Amblyomma*
7589	Dorsal	ResNet-50	dorsal sculptum (73).JPG	fold2	5 *Amblyomma*
7590	Dorsal	ResNet-50	dorsal sculptum (77).jpg	fold2	5 *Amblyomma*
7591	Dorsal	ResNet-50	dorsal sculptum (81).jpg	fold2	5 *Amblyomma*
7592	Dorsal	ResNet-50	dorsal triste (11).jpg	fold2	6 *Amblyomma*
7593	Dorsal	ResNet-50	dorsal triste (17).jpg	fold2	6 *Amblyomma*
7594	Dorsal	ResNet-50	dorsal triste (28).jpg	fold2	6 *Amblyomma*
7595	Dorsal	ResNet-50	dorsal triste (35).jpg	fold2	6 *Amblyomma*
7596	Dorsal	ResNet-50	dorsal triste (37).jpg	fold2	6 *Amblyomma*
7597	Dorsal	ResNet-50	dorsal triste (38).jpg	fold2	6 *Amblyomma*
7598	Dorsal	ResNet-50	dorsal triste (39).jpg	fold2	6 *Amblyomma*
7599	Dorsal	ResNet-50	dorsal triste (43).jpg	fold2	6 *Amblyomma*
7600	Dorsal	ResNet-50	dorsal triste (50).jpg	fold2	6 *Amblyomma*
7601	Dorsal	ResNet-50	dorsal triste (62).jpg	fold2	6 *Amblyomma*
7602	Dorsal	ResNet-50	dorsal triste (65).jpg	fold2	6 *Amblyomma*
7603	Dorsal	ResNet-50	dorsal triste (67).jpg	fold2	6 *Amblyomma*
7604	Dorsal	ResNet-50	dorsal triste (68).jpg	fold2	6 *Amblyomma*
7605	Dorsal	ResNet-50	dorsal triste (71).jpg	fold2	6 *Amblyomma*
7606	Dorsal	ResNet-50	dorsal triste (75).jpg	fold2	6 *Amblyomma*
7607	Dorsal	ResNet-50	dorsal triste (77).jpg	fold2	6 *Amblyomma*
7608	Dorsal	ResNet-50	dorsal aureolatum (1).jpg	fold3	1 *Amblyomma*
7609	Dorsal	ResNet-50	dorsal aureolatum (14).jpg	fold3	1 *Amblyomma*
7610	Dorsal	ResNet-50	dorsal aureolatum (19).jpg	fold3	1 *Amblyomma*
7611	Dorsal	ResNet-50	dorsal aureolatum (24).jpg	fold3	1 *Amblyomma*
7612	Dorsal	ResNet-50	dorsal aureolatum (31).jpg	fold3	1 *Amblyomma*
7613	Dorsal	ResNet-50	dorsal aureolatum (33).jpg	fold3	1 *Amblyomma*
7614	Dorsal	ResNet-50	dorsal aureolatum (35).jpg	fold3	1 *Amblyomma*
7615	Dorsal	ResNet-50	dorsal aureolatum (36).jpg	fold3	1 *Amblyomma*
7616	Dorsal	ResNet-50	dorsal aureolatum (40).jpg	fold3	1 *Amblyomma*
7617	Dorsal	ResNet-50	dorsal aureolatum (53).jpg	fold3	1 *Amblyomma*
7618	Dorsal	ResNet-50	dorsal aureolatum (54).jpg	fold3	1 *Amblyomma*
7619	Dorsal	ResNet-50	dorsal aureolatum (9).jpg	fold3	1 *Amblyomma*
7620	Dorsal	ResNet-50	dorsal cajennense (10).jpg	fold3	2 *Amblyomma*
7621	Dorsal	ResNet-50	dorsal cajennense (17).jpg	fold3	2 *Amblyomma*
7622	Dorsal	ResNet-50	dorsal cajennense (2).jpg	fold3	2 *Amblyomma*
7623	Dorsal	ResNet-50	dorsal cajennense (29).jpg	fold3	2 *Amblyomma*
7624	Dorsal	ResNet-50	dorsal cajennense (35).jpg	fold3	2 *Amblyomma*
7625	Dorsal	ResNet-50	dorsal cajennense (46).jpg	fold3	2 *Amblyomma*
7626	Dorsal	ResNet-50	dorsal cajennense (48).jpg	fold3	2 *Amblyomma*
7627	Dorsal	ResNet-50	dorsal cajennense (5).jpg	fold3	2 *Amblyomma*
7628	Dorsal	ResNet-50	dorsal cajennense (53).jpg	fold3	2 *Amblyomma*
7629	Dorsal	ResNet-50	dorsal cajennense (56).jpg	fold3	2 *Amblyomma*
7630	Dorsal	ResNet-50	dorsal cajennense (57).jpg	fold3	2 *Amblyomma*
7631	Dorsal	ResNet-50	dorsal cajennense (60).jpg	fold3	2 *Amblyomma*
7632	Dorsal	ResNet-50	dorsal cajennense (62).jpg	fold3	2 *Amblyomma*
7633	Dorsal	ResNet-50	dorsal cajennense (67).jpg	fold3	2 *Amblyomma*
7634	Dorsal	ResNet-50	dorsal cajennense (70).jpg	fold3	2 *Amblyomma*
7635	Dorsal	ResNet-50	dorsal cajennense (81).jpg	fold3	2 *Amblyomma*
7636	Dorsal	ResNet-50	dorsal cajennense (82).jpg	fold3	2 *Amblyomma*

7637	Dorsal	ResNet-50	dorsal dubitatum (1).jpg	fold3	3	*Amblyomma*
7638	Dorsal	ResNet-50	dorsal dubitatum (11).jpg	fold3	3	*Amblyomma*
7639	Dorsal	ResNet-50	dorsal dubitatum (26).jpg	fold3	3	*Amblyomma*
7640	Dorsal	ResNet-50	dorsal dubitatum (27).jpg	fold3	3	*Amblyomma*
7641	Dorsal	ResNet-50	dorsal dubitatum (38).jpg	fold3	3	*Amblyomma*
7642	Dorsal	ResNet-50	dorsal dubitatum (39).jpg	fold3	3	*Amblyomma*
7643	Dorsal	ResNet-50	dorsal dubitatum (4).jpg	fold3	3	*Amblyomma*
7644	Dorsal	ResNet-50	dorsal dubitatum (41).jpg	fold3	3	*Amblyomma*
7645	Dorsal	ResNet-50	dorsal dubitatum (42).jpg	fold3	3	*Amblyomma*
7646	Dorsal	ResNet-50	dorsal dubitatum (48).jpg	fold3	3	*Amblyomma*
7647	Dorsal	ResNet-50	dorsal dubitatum (56).jpg	fold3	3	*Amblyomma*
7648	Dorsal	ResNet-50	dorsal ovale (17).jpg	fold3	4	*Amblyomma*
7649	Dorsal	ResNet-50	dorsal ovale (19).jpg	fold3	4	*Amblyomma*
7650	Dorsal	ResNet-50	dorsal ovale (2).jpg	fold3	4	*Amblyomma*
7651	Dorsal	ResNet-50	dorsal ovale (30).jpg	fold3	4	*Amblyomma*
7652	Dorsal	ResNet-50	dorsal ovale (32).jpg	fold3	4	*Amblyomma*
7653	Dorsal	ResNet-50	dorsal ovale (4).jpg	fold3	4	*Amblyomma*
7654	Dorsal	ResNet-50	dorsal ovale (40).jpg	fold3	4	*Amblyomma*
7655	Dorsal	ResNet-50	dorsal ovale (50).jpg	fold3	4	*Amblyomma*
7656	Dorsal	ResNet-50	dorsal ovale (52).jpg	fold3	4	*Amblyomma*
7657	Dorsal	ResNet-50	dorsal ovale (54).JPG	fold3	4	*Amblyomma*
7658	Dorsal	ResNet-50	dorsal ovale (57).jpg	fold3	4	*Amblyomma*
7659	Dorsal	ResNet-50	dorsal ovale (60).jpg	fold3	4	*Amblyomma*
7660	Dorsal	ResNet-50	dorsal sculptum (11).JPG	fold3	5	*Amblyomma*
7661	Dorsal	ResNet-50	dorsal sculptum (12).JPG	fold3	5	*Amblyomma*
7662	Dorsal	ResNet-50	dorsal sculptum (16).JPG	fold3	5	*Amblyomma*
7663	Dorsal	ResNet-50	dorsal sculptum (39).JPG	fold3	5	*Amblyomma*
7664	Dorsal	ResNet-50	dorsal sculptum (4).jpg	fold3	5	*Amblyomma*
7665	Dorsal	ResNet-50	dorsal sculptum (43).JPG	fold3	5	*Amblyomma*
7666	Dorsal	ResNet-50	dorsal sculptum (48).JPG	fold3	5	*Amblyomma*
7667	Dorsal	ResNet-50	dorsal sculptum (53).JPG	fold3	5	*Amblyomma*
7668	Dorsal	ResNet-50	dorsal sculptum (54).JPG	fold3	5	*Amblyomma*
7669	Dorsal	ResNet-50	dorsal sculptum (56).JPG	fold3	5	*Amblyomma*
7670	Dorsal	ResNet-50	dorsal sculptum (60).JPG	fold3	5	*Amblyomma*
7671	Dorsal	ResNet-50	dorsal sculptum (66).JPG	fold3	5	*Amblyomma*
7672	Dorsal	ResNet-50	dorsal sculptum (71).JPG	fold3	5	*Amblyomma*
7673	Dorsal	ResNet-50	dorsal sculptum (74).JPG	fold3	5	*Amblyomma*
7674	Dorsal	ResNet-50	dorsal sculptum (79).jpg	fold3	5	*Amblyomma*
7675	Dorsal	ResNet-50	dorsal sculptum (8).JPG	fold3	5	*Amblyomma*
7676	Dorsal	ResNet-50	dorsal triste (12).jpg	fold3	6	*Amblyomma*
7677	Dorsal	ResNet-50	dorsal triste (13).jpg	fold3	6	*Amblyomma*
7678	Dorsal	ResNet-50	dorsal triste (15).jpg	fold3	6	*Amblyomma*
7679	Dorsal	ResNet-50	dorsal triste (16).jpg	fold3	6	*Amblyomma*
7680	Dorsal	ResNet-50	dorsal triste (18).jpg	fold3	6	*Amblyomma*
7681	Dorsal	ResNet-50	dorsal triste (21).jpg	fold3	6	*Amblyomma*
7682	Dorsal	ResNet-50	dorsal triste (27).jpg	fold3	6	*Amblyomma*
7683	Dorsal	ResNet-50	dorsal triste (30).jpg	fold3	6	*Amblyomma*
7684	Dorsal	ResNet-50	dorsal triste (41).jpg	fold3	6	*Amblyomma*
7685	Dorsal	ResNet-50	dorsal triste (49).jpg	fold3	6	*Amblyomma*
7686	Dorsal	ResNet-50	dorsal triste (54).jpg	fold3	6	*Amblyomma*
7687	Dorsal	ResNet-50	dorsal triste (56).jpg	fold3	6	*Amblyomma*
7688	Dorsal	ResNet-50	dorsal triste (64).jpg	fold3	6	*Amblyomma*
7689	Dorsal	ResNet-50	dorsal triste (66).jpg	fold3	6	*Amblyomma*
7690	Dorsal	ResNet-50	dorsal triste (7).jpg	fold3	6	*Amblyomma*
7691	Dorsal	ResNet-50	dorsal triste (73).jpg	fold3	6	*Amblyomma*
7692	Dorsal	ResNet-50	dorsal aureolatum (12).jpg	fold4	1	*Amblyomma*
7693	Dorsal	ResNet-50	dorsal aureolatum (13).jpg	fold4	1	*Amblyomma*
7694	Dorsal	ResNet-50	dorsal aureolatum (15).jpg	fold4	1	*Amblyomma*
7695	Dorsal	ResNet-50	dorsal aureolatum (16).jpg	fold4	1	*Amblyomma*
7696	Dorsal	ResNet-50	dorsal aureolatum (20).jpg	fold4	1	*Amblyomma*
7697	Dorsal	ResNet-50	dorsal aureolatum (22).jpg	fold4	1	*Amblyomma*
7698	Dorsal	ResNet-50	dorsal aureolatum (25).jpg	fold4	1	*Amblyomma*
7699	Dorsal	ResNet-50	dorsal aureolatum (26).jpg	fold4	1	*Amblyomma*
7700	Dorsal	ResNet-50	dorsal aureolatum (37).jpg	fold4	1	*Amblyomma*
7701	Dorsal	ResNet-50	dorsal aureolatum (4).jpg	fold4	1	*Amblyomma*
7702	Dorsal	ResNet-50	dorsal aureolatum (56).jpg	fold4	1	*Amblyomma*
7703	Dorsal	ResNet-50	dorsal aureolatum (63).jpg	fold4	1	*Amblyomma*

7704	Dorsal	ResNet-50	dorsal aureolatum (7).jpg	fold4	1	*Amblyomma*
7705	Dorsal	ResNet-50	dorsal cajennense (1).jpg	fold4	2	*Amblyomma*
7706	Dorsal	ResNet-50	dorsal cajennense (11).jpg	fold4	2	*Amblyomma*
7707	Dorsal	ResNet-50	dorsal cajennense (12).jpg	fold4	2	*Amblyomma*
7708	Dorsal	ResNet-50	dorsal cajennense (13).jpg	fold4	2	*Amblyomma*
7709	Dorsal	ResNet-50	dorsal cajennense (20).jpg	fold4	2	*Amblyomma*
7710	Dorsal	ResNet-50	dorsal cajennense (33).jpg	fold4	2	*Amblyomma*
7711	Dorsal	ResNet-50	dorsal cajennense (34).jpg	fold4	2	*Amblyomma*
7712	Dorsal	ResNet-50	dorsal cajennense (36).jpg	fold4	2	*Amblyomma*
7713	Dorsal	ResNet-50	dorsal cajennense (38).jpg	fold4	2	*Amblyomma*
7714	Dorsal	ResNet-50	dorsal cajennense (4).jpg	fold4	2	*Amblyomma*
7715	Dorsal	ResNet-50	dorsal cajennense (45).jpg	fold4	2	*Amblyomma*
7716	Dorsal	ResNet-50	dorsal cajennense (6).jpg	fold4	2	*Amblyomma*
7717	Dorsal	ResNet-50	dorsal cajennense (63).jpg	fold4	2	*Amblyomma*
7718	Dorsal	ResNet-50	dorsal cajennense (64).jpg	fold4	2	*Amblyomma*
7719	Dorsal	ResNet-50	dorsal cajennense (76).jpg	fold4	2	*Amblyomma*
7720	Dorsal	ResNet-50	dorsal cajennense (78).jpg	fold4	2	*Amblyomma*
7721	Dorsal	ResNet-50	dorsal cajennense (79).jpg	fold4	2	*Amblyomma*
7722	Dorsal	ResNet-50	dorsal dubitatum (12).jpg	fold4	3	*Amblyomma*
7723	Dorsal	ResNet-50	dorsal dubitatum (13).jpg	fold4	3	*Amblyomma*
7724	Dorsal	ResNet-50	dorsal dubitatum (17).jpg	fold4	3	*Amblyomma*
7725	Dorsal	ResNet-50	dorsal dubitatum (20).jpg	fold4	3	*Amblyomma*
7726	Dorsal	ResNet-50	dorsal dubitatum (21).jpg	fold4	3	*Amblyomma*
7727	Dorsal	ResNet-50	dorsal dubitatum (22).jpg	fold4	3	*Amblyomma*
7728	Dorsal	ResNet-50	dorsal dubitatum (3).jpg	fold4	3	*Amblyomma*
7729	Dorsal	ResNet-50	dorsal dubitatum (31).jpg	fold4	3	*Amblyomma*
7730	Dorsal	ResNet-50	dorsal dubitatum (35).jpg	fold4	3	*Amblyomma*
7731	Dorsal	ResNet-50	dorsal dubitatum (54).jpg	fold4	3	*Amblyomma*
7732	Dorsal	ResNet-50	dorsal dubitatum (8).JPG	fold4	3	*Amblyomma*
7733	Dorsal	ResNet-50	dorsal ovale (1).jpg	fold4	4	*Amblyomma*
7734	Dorsal	ResNet-50	dorsal ovale (13).jpg	fold4	4	*Amblyomma*
7735	Dorsal	ResNet-50	dorsal ovale (16).jpg	fold4	4	*Amblyomma*
7736	Dorsal	ResNet-50	dorsal ovale (24).jpg	fold4	4	*Amblyomma*
7737	Dorsal	ResNet-50	dorsal ovale (25).jpg	fold4	4	*Amblyomma*
7738	Dorsal	ResNet-50	dorsal ovale (31).jpg	fold4	4	*Amblyomma*
7739	Dorsal	ResNet-50	dorsal ovale (34).jpg	fold4	4	*Amblyomma*
7740	Dorsal	ResNet-50	dorsal ovale (36).jpg	fold4	4	*Amblyomma*
7741	Dorsal	ResNet-50	dorsal ovale (37).jpg	fold4	4	*Amblyomma*
7742	Dorsal	ResNet-50	dorsal ovale (39).jpg	fold4	4	*Amblyomma*
7743	Dorsal	ResNet-50	dorsal ovale (55).jpg	fold4	4	*Amblyomma*
7744	Dorsal	ResNet-50	dorsal ovale (9).jpg	fold4	4	*Amblyomma*
7745	Dorsal	ResNet-50	dorsal sculptum (15).JPG	fold4	5	*Amblyomma*
7746	Dorsal	ResNet-50	dorsal sculptum (19).JPG	fold4	5	*Amblyomma*
7747	Dorsal	ResNet-50	dorsal sculptum (2).jpg	fold4	5	*Amblyomma*
7748	Dorsal	ResNet-50	dorsal sculptum (24).JPG	fold4	5	*Amblyomma*
7749	Dorsal	ResNet-50	dorsal sculptum (30).jpg	fold4	5	*Amblyomma*
7750	Dorsal	ResNet-50	dorsal sculptum (34).jpg	fold4	5	*Amblyomma*
7751	Dorsal	ResNet-50	dorsal sculptum (35).jpg	fold4	5	*Amblyomma*
7752	Dorsal	ResNet-50	dorsal sculptum (44).JPG	fold4	5	*Amblyomma*
7753	Dorsal	ResNet-50	dorsal sculptum (45).JPG	fold4	5	*Amblyomma*
7754	Dorsal	ResNet-50	dorsal sculptum (49).JPG	fold4	5	*Amblyomma*
7755	Dorsal	ResNet-50	dorsal sculptum (55).JPG	fold4	5	*Amblyomma*
7756	Dorsal	ResNet-50	dorsal sculptum (61).JPG	fold4	5	*Amblyomma*
7757	Dorsal	ResNet-50	dorsal sculptum (62).JPG	fold4	5	*Amblyomma*
7758	Dorsal	ResNet-50	dorsal sculptum (64).JPG	fold4	5	*Amblyomma*
7759	Dorsal	ResNet-50	dorsal sculptum (69).JPG	fold4	5	*Amblyomma*
7760	Dorsal	ResNet-50	dorsal sculptum (80).jpg	fold4	5	*Amblyomma*
7761	Dorsal	ResNet-50	dorsal triste (10).jpg	fold4	6	*Amblyomma*
7762	Dorsal	ResNet-50	dorsal triste (23).jpg	fold4	6	*Amblyomma*
7763	Dorsal	ResNet-50	dorsal triste (29).jpg	fold4	6	*Amblyomma*
7764	Dorsal	ResNet-50	dorsal triste (31).jpg	fold4	6	*Amblyomma*
7765	Dorsal	ResNet-50	dorsal triste (32).jpg	fold4	6	*Amblyomma*
7766	Dorsal	ResNet-50	dorsal triste (34).jpg	fold4	6	*Amblyomma*
7767	Dorsal	ResNet-50	dorsal triste (4).jpg	fold4	6	*Amblyomma*
7768	Dorsal	ResNet-50	dorsal triste (40).jpg	fold4	6	*Amblyomma*
7769	Dorsal	ResNet-50	dorsal triste (5).jpg	fold4	6	*Amblyomma*
7770	Dorsal	ResNet-50	dorsal triste (53).jpg	fold4	6	*Amblyomma*

7771	Dorsal	ResNet-50	dorsal triste (55).jpg	fold4	6	*Amblyomma*
7772	Dorsal	ResNet-50	dorsal triste (69).jpg	fold4	6	*Amblyomma*
7773	Dorsal	ResNet-50	dorsal triste (70).jpg	fold4	6	*Amblyomma*
7774	Dorsal	ResNet-50	dorsal triste (76).jpg	fold4	6	*Amblyomma*
7775	Dorsal	ResNet-50	dorsal triste (8).jpg	fold4	6	*Amblyomma*
7776	Dorsal	ResNet-50	dorsal aureolatum (10).jpg	fold5	1	*Amblyomma*
7777	Dorsal	ResNet-50	dorsal aureolatum (2).jpg	fold5	1	*Amblyomma*
7778	Dorsal	ResNet-50	dorsal aureolatum (3).jpg	fold5	1	*Amblyomma*
7779	Dorsal	ResNet-50	dorsal aureolatum (34).jpg	fold5	1	*Amblyomma*
7780	Dorsal	ResNet-50	dorsal aureolatum (39).jpg	fold5	1	*Amblyomma*
7781	Dorsal	ResNet-50	dorsal aureolatum (41).jpg	fold5	1	*Amblyomma*
7782	Dorsal	ResNet-50	dorsal aureolatum (42).jpg	fold5	1	*Amblyomma*
7783	Dorsal	ResNet-50	dorsal aureolatum (45).jpg	fold5	1	*Amblyomma*
7784	Dorsal	ResNet-50	dorsal aureolatum (47).jpg	fold5	1	*Amblyomma*
7785	Dorsal	ResNet-50	dorsal aureolatum (50).jpg	fold5	1	*Amblyomma*
7786	Dorsal	ResNet-50	dorsal aureolatum (57).jpg	fold5	1	*Amblyomma*
7787	Dorsal	ResNet-50	dorsal aureolatum (59).jpg	fold5	1	*Amblyomma*
7788	Dorsal	ResNet-50	dorsal aureolatum (60).jpg	fold5	1	*Amblyomma*
7789	Dorsal	ResNet-50	dorsal cajennense (14).jpg	fold5	2	*Amblyomma*
7790	Dorsal	ResNet-50	dorsal cajennense (18).jpg	fold5	2	*Amblyomma*
7791	Dorsal	ResNet-50	dorsal cajennense (19).jpg	fold5	2	*Amblyomma*
7792	Dorsal	ResNet-50	dorsal cajennense (21).jpg	fold5	2	*Amblyomma*
7793	Dorsal	ResNet-50	dorsal cajennense (22).jpg	fold5	2	*Amblyomma*
7794	Dorsal	ResNet-50	dorsal cajennense (24).jpg	fold5	2	*Amblyomma*
7795	Dorsal	ResNet-50	dorsal cajennense (28).jpg	fold5	2	*Amblyomma*
7796	Dorsal	ResNet-50	dorsal cajennense (30).jpg	fold5	2	*Amblyomma*
7797	Dorsal	ResNet-50	dorsal cajennense (39).jpg	fold5	2	*Amblyomma*
7798	Dorsal	ResNet-50	dorsal cajennense (40).jpg	fold5	2	*Amblyomma*
7799	Dorsal	ResNet-50	dorsal cajennense (41).jpg	fold5	2	*Amblyomma*
7800	Dorsal	ResNet-50	dorsal cajennense (42).jpg	fold5	2	*Amblyomma*
7801	Dorsal	ResNet-50	dorsal cajennense (49).jpg	fold5	2	*Amblyomma*
7802	Dorsal	ResNet-50	dorsal cajennense (55).jpg	fold5	2	*Amblyomma*
7803	Dorsal	ResNet-50	dorsal cajennense (68).jpg	fold5	2	*Amblyomma*
7804	Dorsal	ResNet-50	dorsal cajennense (69).jpg	fold5	2	*Amblyomma*
7805	Dorsal	ResNet-50	dorsal cajennense (71).jpg	fold5	2	*Amblyomma*
7806	Dorsal	ResNet-50	dorsal dubitatum (16).jpg	fold5	3	*Amblyomma*
7807	Dorsal	ResNet-50	dorsal dubitatum (23).jpg	fold5	3	*Amblyomma*
7808	Dorsal	ResNet-50	dorsal dubitatum (24).jpg	fold5	3	*Amblyomma*
7809	Dorsal	ResNet-50	dorsal dubitatum (25).jpg	fold5	3	*Amblyomma*
7810	Dorsal	ResNet-50	dorsal dubitatum (29).jpg	fold5	3	*Amblyomma*
7811	Dorsal	ResNet-50	dorsal dubitatum (30).jpg	fold5	3	*Amblyomma*
7812	Dorsal	ResNet-50	dorsal dubitatum (33).jpg	fold5	3	*Amblyomma*
7813	Dorsal	ResNet-50	dorsal dubitatum (45).jpg	fold5	3	*Amblyomma*
7814	Dorsal	ResNet-50	dorsal dubitatum (51).jpg	fold5	3	*Amblyomma*
7815	Dorsal	ResNet-50	dorsal dubitatum (52).jpg	fold5	3	*Amblyomma*
7816	Dorsal	ResNet-50	dorsal dubitatum (53).jpg	fold5	3	*Amblyomma*
7817	Dorsal	ResNet-50	dorsal ovale (11).jpg	fold5	4	*Amblyomma*
7818	Dorsal	ResNet-50	dorsal ovale (12).jpg	fold5	4	*Amblyomma*
7819	Dorsal	ResNet-50	dorsal ovale (28).jpg	fold5	4	*Amblyomma*
7820	Dorsal	ResNet-50	dorsal ovale (3).jpg	fold5	4	*Amblyomma*
7821	Dorsal	ResNet-50	dorsal ovale (35).jpg	fold5	4	*Amblyomma*
7822	Dorsal	ResNet-50	dorsal ovale (43).jpg	fold5	4	*Amblyomma*
7823	Dorsal	ResNet-50	dorsal ovale (45).jpg	fold5	4	*Amblyomma*
7824	Dorsal	ResNet-50	dorsal ovale (46).jpg	fold5	4	*Amblyomma*
7825	Dorsal	ResNet-50	dorsal ovale (47).jpg	fold5	4	*Amblyomma*
7826	Dorsal	ResNet-50	dorsal ovale (56).jpg	fold5	4	*Amblyomma*
7827	Dorsal	ResNet-50	dorsal ovale (58).jpg	fold5	4	*Amblyomma*
7828	Dorsal	ResNet-50	dorsal ovale (59).jpg	fold5	4	*Amblyomma*
7829	Dorsal	ResNet-50	dorsal ovale (8).jpg	fold5	4	*Amblyomma*
7830	Dorsal	ResNet-50	dorsal sculptum (10).JPG	fold5	5	*Amblyomma*
7831	Dorsal	ResNet-50	dorsal sculptum (18).JPG	fold5	5	*Amblyomma*
7832	Dorsal	ResNet-50	dorsal sculptum (21).JPG	fold5	5	*Amblyomma*
7833	Dorsal	ResNet-50	dorsal sculptum (28).JPG	fold5	5	*Amblyomma*
7834	Dorsal	ResNet-50	dorsal sculptum (3).jpg	fold5	5	*Amblyomma*
7835	Dorsal	ResNet-50	dorsal sculptum (32).jpg	fold5	5	*Amblyomma*
7836	Dorsal	ResNet-50	dorsal sculptum (33).jpg	fold5	5	*Amblyomma*
7837	Dorsal	ResNet-50	dorsal sculptum (46).JPG	fold5	5	*Amblyomma*

7838	Dorsal	ResNet-50	dorsal sculptum (47).JPG	fold5	5	*Amblyomma*
7839	Dorsal	ResNet-50	dorsal sculptum (6).JPG	fold5	5	*Amblyomma*
7840	Dorsal	ResNet-50	dorsal sculptum (67).JPG	fold5	5	*Amblyomma*
7841	Dorsal	ResNet-50	dorsal sculptum (68).JPG	fold5	5	*Amblyomma*
7842	Dorsal	ResNet-50	dorsal sculptum (7).JPG	fold5	5	*Amblyomma*
7843	Dorsal	ResNet-50	dorsal sculptum (70).JPG	fold5	5	*Amblyomma*
7844	Dorsal	ResNet-50	dorsal sculptum (76).jpg	fold5	5	*Amblyomma*
7845	Dorsal	ResNet-50	dorsal sculptum (78).jpg	fold5	5	*Amblyomma*
7846	Dorsal	ResNet-50	dorsal triste (1).jpg	fold5	6	*Amblyomma*
7847	Dorsal	ResNet-50	dorsal triste (14).jpg	fold5	6	*Amblyomma*
7848	Dorsal	ResNet-50	dorsal triste (22).jpg	fold5	6	*Amblyomma*
7849	Dorsal	ResNet-50	dorsal triste (3).jpg	fold5	6	*Amblyomma*
7850	Dorsal	ResNet-50	dorsal triste (33).jpg	fold5	6	*Amblyomma*
7851	Dorsal	ResNet-50	dorsal triste (36).jpg	fold5	6	*Amblyomma*
7852	Dorsal	ResNet-50	dorsal triste (42).jpg	fold5	6	*Amblyomma*
7853	Dorsal	ResNet-50	dorsal triste (44).jpg	fold5	6	*Amblyomma*
7854	Dorsal	ResNet-50	dorsal triste (48).jpg	fold5	6	*Amblyomma*
7855	Dorsal	ResNet-50	dorsal triste (52).jpg	fold5	6	*Amblyomma*
7856	Dorsal	ResNet-50	dorsal triste (57).jpg	fold5	6	*Amblyomma*
7857	Dorsal	ResNet-50	dorsal triste (58).jpg	fold5	6	*Amblyomma*
7858	Dorsal	ResNet-50	dorsal triste (59).jpg	fold5	6	*Amblyomma*
7859	Dorsal	ResNet-50	dorsal triste (60).jpg	fold5	6	*Amblyomma*
7860	Dorsal	ResNet-50	dorsal triste (78).jpg	fold5	6	*Amblyomma*
7861	Dorsal	ResNet-50	dorsal triste (9).jpg	fold5	6	*Amblyomma*
7862	Ventral	ResNet-50	ventral aureolatum (10).jpg	fold1	1	*Amblyomma*
7863	Ventral	ResNet-50	ventral aureolatum (16).jpg	fold1	1	*Amblyomma*
7864	Ventral	ResNet-50	ventral aureolatum (20).jpg	fold1	1	*Amblyomma*
7865	Ventral	ResNet-50	ventral aureolatum (21).jpg	fold1	1	*Amblyomma*
7866	Ventral	ResNet-50	ventral aureolatum (28).jpg	fold1	1	*Amblyomma*
7867	Ventral	ResNet-50	ventral aureolatum (3).jpg	fold1	1	*Amblyomma*
7868	Ventral	ResNet-50	ventral aureolatum (31).jpg	fold1	1	*Amblyomma*
7869	Ventral	ResNet-50	ventral aureolatum (33).jpg	fold1	1	*Amblyomma*
7870	Ventral	ResNet-50	ventral aureolatum (34).jpg	fold1	1	*Amblyomma*
7871	Ventral	ResNet-50	ventral aureolatum (40).jpg	fold1	1	*Amblyomma*
7872	Ventral	ResNet-50	ventral aureolatum (44).jpg	fold1	1	*Amblyomma*
7873	Ventral	ResNet-50	ventral aureolatum (58).jpg	fold1	1	*Amblyomma*
7874	Ventral	ResNet-50	ventral cajennense (14).jpg	fold1	2	*Amblyomma*
7875	Ventral	ResNet-50	ventral cajennense (16).jpg	fold1	2	*Amblyomma*
7876	Ventral	ResNet-50	ventral cajennense (28).jpg	fold1	2	*Amblyomma*
7877	Ventral	ResNet-50	ventral cajennense (33).jpg	fold1	2	*Amblyomma*
7878	Ventral	ResNet-50	ventral cajennense (4).jpg	fold1	2	*Amblyomma*
7879	Ventral	ResNet-50	ventral cajennense (41).jpg	fold1	2	*Amblyomma*
7880	Ventral	ResNet-50	ventral cajennense (5).jpg	fold1	2	*Amblyomma*
7881	Ventral	ResNet-50	ventral cajennense (51).jpg	fold1	2	*Amblyomma*
7882	Ventral	ResNet-50	ventral cajennense (57).jpg	fold1	2	*Amblyomma*
7883	Ventral	ResNet-50	ventral cajennense (60).jpg	fold1	2	*Amblyomma*
7884	Ventral	ResNet-50	ventral cajennense (64).jpg	fold1	2	*Amblyomma*
7885	Ventral	ResNet-50	ventral cajennense (7).jpg	fold1	2	*Amblyomma*
7886	Ventral	ResNet-50	ventral cajennense (72).jpg	fold1	2	*Amblyomma*
7887	Ventral	ResNet-50	ventral cajennense (74).jpg	fold1	2	*Amblyomma*
7888	Ventral	ResNet-50	ventral cajennense (75).jpg	fold1	2	*Amblyomma*
7889	Ventral	ResNet-50	ventral cajennense (82).jpg	fold1	2	*Amblyomma*
7890	Ventral	ResNet-50	ventral dubitatum (22).jpg	fold1	3	*Amblyomma*
7891	Ventral	ResNet-50	ventral dubitatum (26).jpg	fold1	3	*Amblyomma*
7892	Ventral	ResNet-50	ventral dubitatum (27).jpg	fold1	3	*Amblyomma*
7893	Ventral	ResNet-50	ventral dubitatum (30).jpg	fold1	3	*Amblyomma*
7894	Ventral	ResNet-50	ventral dubitatum (39).jpg	fold1	3	*Amblyomma*
7895	Ventral	ResNet-50	ventral dubitatum (40).jpg	fold1	3	*Amblyomma*
7896	Ventral	ResNet-50	ventral dubitatum (45).jpg	fold1	3	*Amblyomma*
7897	Ventral	ResNet-50	ventral dubitatum (46).jpg	fold1	3	*Amblyomma*
7898	Ventral	ResNet-50	ventral dubitatum (51).jpg	fold1	3	*Amblyomma*
7899	Ventral	ResNet-50	ventral dubitatum (7).jpg	fold1	3	*Amblyomma*
7900	Ventral	ResNet-50	ventral ovale (1).jpg	fold1	4	*Amblyomma*
7901	Ventral	ResNet-50	ventral ovale (18).jpg	fold1	4	*Amblyomma*
7902	Ventral	ResNet-50	ventral ovale (20).jpg	fold1	4	*Amblyomma*
7903	Ventral	ResNet-50	ventral ovale (22).jpg	fold1	4	*Amblyomma*
7904	Ventral	ResNet-50	ventral ovale (26).jpg	fold1	4	*Amblyomma*

7905	Ventral	ResNet-50	ventral ovale (31).jpg	fold1	4	*Amblyomma*
7906	Ventral	ResNet-50	ventral ovale (32).jpg	fold1	4	*Amblyomma*
7907	Ventral	ResNet-50	ventral ovale (36).jpg	fold1	4	*Amblyomma*
7908	Ventral	ResNet-50	ventral ovale (38).JPG	fold1	4	*Amblyomma*
7909	Ventral	ResNet-50	ventral ovale (57).jpg	fold1	4	*Amblyomma*
7910	Ventral	ResNet-50	ventral ovale (58).jpg	fold1	4	*Amblyomma*
7911	Ventral	ResNet-50	ventral ovale (60).jpg	fold1	4	*Amblyomma*
7912	Ventral	ResNet-50	ventral sculptum (10).JPG	fold1	5	*Amblyomma*
7913	Ventral	ResNet-50	ventral sculptum (18).JPG	fold1	5	*Amblyomma*
7914	Ventral	ResNet-50	ventral sculptum (2).jpg	fold1	5	*Amblyomma*
7915	Ventral	ResNet-50	ventral sculptum (28).jpg	fold1	5	*Amblyomma*
7916	Ventral	ResNet-50	ventral sculptum (29).jpg	fold1	5	*Amblyomma*
7917	Ventral	ResNet-50	ventral sculptum (31).jpg	fold1	5	*Amblyomma*
7918	Ventral	ResNet-50	ventral sculptum (32).jpg	fold1	5	*Amblyomma*
7919	Ventral	ResNet-50	ventral sculptum (38).JPG	fold1	5	*Amblyomma*
7920	Ventral	ResNet-50	ventral sculptum (40).JPG	fold1	5	*Amblyomma*
7921	Ventral	ResNet-50	ventral sculptum (43).JPG	fold1	5	*Amblyomma*
7922	Ventral	ResNet-50	ventral sculptum (45).JPG	fold1	5	*Amblyomma*
7923	Ventral	ResNet-50	ventral sculptum (54).JPG	fold1	5	*Amblyomma*
7924	Ventral	ResNet-50	ventral sculptum (6).JPG	fold1	5	*Amblyomma*
7925	Ventral	ResNet-50	ventral sculptum (60).JPG	fold1	5	*Amblyomma*
7926	Ventral	ResNet-50	ventral sculptum (67).JPG	fold1	5	*Amblyomma*
7927	Ventral	ResNet-50	ventral triste (11).jpg	fold1	6	*Amblyomma*
7928	Ventral	ResNet-50	ventral triste (16).jpg	fold1	6	*Amblyomma*
7929	Ventral	ResNet-50	ventral triste (33).jpg	fold1	6	*Amblyomma*
7930	Ventral	ResNet-50	ventral triste (37).jpg	fold1	6	*Amblyomma*
7931	Ventral	ResNet-50	ventral triste (38).jpg	fold1	6	*Amblyomma*
7932	Ventral	ResNet-50	ventral triste (39).jpg	fold1	6	*Amblyomma*
7933	Ventral	ResNet-50	ventral triste (41).jpg	fold1	6	*Amblyomma*
7934	Ventral	ResNet-50	ventral triste (42).jpg	fold1	6	*Amblyomma*
7935	Ventral	ResNet-50	ventral triste (47).jpg	fold1	6	*Amblyomma*
7936	Ventral	ResNet-50	ventral triste (66).jpg	fold1	6	*Amblyomma*
7937	Ventral	ResNet-50	ventral triste (67).jpg	fold1	6	*Amblyomma*
7938	Ventral	ResNet-50	ventral triste (68).jpg	fold1	6	*Amblyomma*
7939	Ventral	ResNet-50	ventral triste (72).jpg	fold1	6	*Amblyomma*
7940	Ventral	ResNet-50	ventral triste (75).jpg	fold1	6	*Amblyomma*
7941	Ventral	ResNet-50	ventral triste (76).jpg	fold1	6	*Amblyomma*
7942	Ventral	ResNet-50	ventral triste (77).jpg	fold1	6	*Amblyomma*
7943	Ventral	ResNet-50	ventral aureolatum (12).jpg	fold2	1	*Amblyomma*
7944	Ventral	ResNet-50	ventral aureolatum (18).jpg	fold2	1	*Amblyomma*
7945	Ventral	ResNet-50	ventral aureolatum (22).jpg	fold2	1	*Amblyomma*
7946	Ventral	ResNet-50	ventral aureolatum (26).jpg	fold2	1	*Amblyomma*
7947	Ventral	ResNet-50	ventral aureolatum (27).jpg	fold2	1	*Amblyomma*
7948	Ventral	ResNet-50	ventral aureolatum (4).jpg	fold2	1	*Amblyomma*
7949	Ventral	ResNet-50	ventral aureolatum (47).jpg	fold2	1	*Amblyomma*
7950	Ventral	ResNet-50	ventral aureolatum (48).jpg	fold2	1	*Amblyomma*
7951	Ventral	ResNet-50	ventral aureolatum (49).jpg	fold2	1	*Amblyomma*
7952	Ventral	ResNet-50	ventral aureolatum (50).jpg	fold2	1	*Amblyomma*
7953	Ventral	ResNet-50	ventral aureolatum (51).jpg	fold2	1	*Amblyomma*
7954	Ventral	ResNet-50	ventral aureolatum (56).jpg	fold2	1	*Amblyomma*
7955	Ventral	ResNet-50	ventral cajennense (1).jpg	fold2	2	*Amblyomma*
7956	Ventral	ResNet-50	ventral cajennense (15).jpg	fold2	2	*Amblyomma*
7957	Ventral	ResNet-50	ventral cajennense (20).jpg	fold2	2	*Amblyomma*
7958	Ventral	ResNet-50	ventral cajennense (23).jpg	fold2	2	*Amblyomma*
7959	Ventral	ResNet-50	ventral cajennense (29).jpg	fold2	2	*Amblyomma*
7960	Ventral	ResNet-50	ventral cajennense (30).jpg	fold2	2	*Amblyomma*
7961	Ventral	ResNet-50	ventral cajennense (37).jpg	fold2	2	*Amblyomma*
7962	Ventral	ResNet-50	ventral cajennense (49).jpg	fold2	2	*Amblyomma*
7963	Ventral	ResNet-50	ventral cajennense (52).jpg	fold2	2	*Amblyomma*
7964	Ventral	ResNet-50	ventral cajennense (56).jpg	fold2	2	*Amblyomma*
7965	Ventral	ResNet-50	ventral cajennense (63).jpg	fold2	2	*Amblyomma*
7966	Ventral	ResNet-50	ventral cajennense (65).jpg	fold2	2	*Amblyomma*
7967	Ventral	ResNet-50	ventral cajennense (67).jpg	fold2	2	*Amblyomma*
7968	Ventral	ResNet-50	ventral cajennense (70).jpg	fold2	2	*Amblyomma*
7969	Ventral	ResNet-50	ventral cajennense (71).jpg	fold2	2	*Amblyomma*
7970	Ventral	ResNet-50	ventral cajennense (9).jpg	fold2	2	*Amblyomma*
7971	Ventral	ResNet-50	ventral dubitatum (19).jpg	fold2	3	*Amblyomma*

7972	Ventral	ResNet-50	ventral dubitatum (23).jpg	fold2	3	*Amblyomma*
7973	Ventral	ResNet-50	ventral dubitatum (24).jpg	fold2	3	*Amblyomma*
7974	Ventral	ResNet-50	ventral dubitatum (25).jpg	fold2	3	*Amblyomma*
7975	Ventral	ResNet-50	ventral dubitatum (28).jpg	fold2	3	*Amblyomma*
7976	Ventral	ResNet-50	ventral dubitatum (36).jpg	fold2	3	*Amblyomma*
7977	Ventral	ResNet-50	ventral dubitatum (38).jpg	fold2	3	*Amblyomma*
7978	Ventral	ResNet-50	ventral dubitatum (41).jpg	fold2	3	*Amblyomma*
7979	Ventral	ResNet-50	ventral dubitatum (42).jpg	fold2	3	*Amblyomma*
7980	Ventral	ResNet-50	ventral dubitatum (48).jpg	fold2	3	*Amblyomma*
7981	Ventral	ResNet-50	ventral dubitatum (49).jpg	fold2	3	*Amblyomma*
7982	Ventral	ResNet-50	ventral ovale (10).jpg	fold2	4	*Amblyomma*
7983	Ventral	ResNet-50	ventral ovale (13).jpg	fold2	4	*Amblyomma*
7984	Ventral	ResNet-50	ventral ovale (14).jpg	fold2	4	*Amblyomma*
7985	Ventral	ResNet-50	ventral ovale (28).jpg	fold2	4	*Amblyomma*
7986	Ventral	ResNet-50	ventral ovale (29).jpg	fold2	4	*Amblyomma*
7987	Ventral	ResNet-50	ventral ovale (3).jpg	fold2	4	*Amblyomma*
7988	Ventral	ResNet-50	ventral ovale (42).jpg	fold2	4	*Amblyomma*
7989	Ventral	ResNet-50	ventral ovale (44).jpg	fold2	4	*Amblyomma*
7990	Ventral	ResNet-50	ventral ovale (49).jpg	fold2	4	*Amblyomma*
7991	Ventral	ResNet-50	ventral ovale (51).jpg	fold2	4	*Amblyomma*
7992	Ventral	ResNet-50	ventral ovale (8).jpg	fold2	4	*Amblyomma*
7993	Ventral	ResNet-50	ventral ovale (9).jpg	fold2	4	*Amblyomma*
7994	Ventral	ResNet-50	ventral sculptum (1).jpg	fold2	5	*Amblyomma*
7995	Ventral	ResNet-50	ventral sculptum (12).JPG	fold2	5	*Amblyomma*
7996	Ventral	ResNet-50	ventral sculptum (23).JPG	fold2	5	*Amblyomma*
7997	Ventral	ResNet-50	ventral sculptum (30).jpg	fold2	5	*Amblyomma*
7998	Ventral	ResNet-50	ventral sculptum (39).JPG	fold2	5	*Amblyomma*
7999	Ventral	ResNet-50	ventral sculptum (4).jpg	fold2	5	*Amblyomma*
8000	Ventral	ResNet-50	ventral sculptum (46).JPG	fold2	5	*Amblyomma*
8001	Ventral	ResNet-50	ventral sculptum (48).JPG	fold2	5	*Amblyomma*
8002	Ventral	ResNet-50	ventral sculptum (51).JPG	fold2	5	*Amblyomma*
8003	Ventral	ResNet-50	ventral sculptum (53).JPG	fold2	5	*Amblyomma*
8004	Ventral	ResNet-50	ventral sculptum (58).JPG	fold2	5	*Amblyomma*
8005	Ventral	ResNet-50	ventral sculptum (63).JPG	fold2	5	*Amblyomma*
8006	Ventral	ResNet-50	ventral sculptum (7).JPG	fold2	5	*Amblyomma*
8007	Ventral	ResNet-50	ventral sculptum (9).JPG	fold2	5	*Amblyomma*
8008	Ventral	ResNet-50	ventral triste (12).jpg	fold2	6	*Amblyomma*
8009	Ventral	ResNet-50	ventral triste (2).jpg	fold2	6	*Amblyomma*
8010	Ventral	ResNet-50	ventral triste (21).jpg	fold2	6	*Amblyomma*
8011	Ventral	ResNet-50	ventral triste (30).jpg	fold2	6	*Amblyomma*
8012	Ventral	ResNet-50	ventral triste (40).jpg	fold2	6	*Amblyomma*
8013	Ventral	ResNet-50	ventral triste (43).jpg	fold2	6	*Amblyomma*
8014	Ventral	ResNet-50	ventral triste (45).jpg	fold2	6	*Amblyomma*
8015	Ventral	ResNet-50	ventral triste (48).jpg	fold2	6	*Amblyomma*
8016	Ventral	ResNet-50	ventral triste (49).jpg	fold2	6	*Amblyomma*
8017	Ventral	ResNet-50	ventral triste (52).jpg	fold2	6	*Amblyomma*
8018	Ventral	ResNet-50	ventral triste (53).jpg	fold2	6	*Amblyomma*
8019	Ventral	ResNet-50	ventral triste (54).jpg	fold2	6	*Amblyomma*
8020	Ventral	ResNet-50	ventral triste (56).jpg	fold2	6	*Amblyomma*
8021	Ventral	ResNet-50	ventral triste (57).jpg	fold2	6	*Amblyomma*
8022	Ventral	ResNet-50	ventral triste (8).jpg	fold2	6	*Amblyomma*
8023	Ventral	ResNet-50	ventral aureolatum (11).jpg	fold3	1	*Amblyomma*
8024	Ventral	ResNet-50	ventral aureolatum (17).jpg	fold3	1	*Amblyomma*
8025	Ventral	ResNet-50	ventral aureolatum (19).jpg	fold3	1	*Amblyomma*
8026	Ventral	ResNet-50	ventral aureolatum (36).jpg	fold3	1	*Amblyomma*
8027	Ventral	ResNet-50	ventral aureolatum (38).jpg	fold3	1	*Amblyomma*
8028	Ventral	ResNet-50	ventral aureolatum (39).jpg	fold3	1	*Amblyomma*
8029	Ventral	ResNet-50	ventral aureolatum (42).jpg	fold3	1	*Amblyomma*
8030	Ventral	ResNet-50	ventral aureolatum (5).jpg	fold3	1	*Amblyomma*
8031	Ventral	ResNet-50	ventral aureolatum (53).jpg	fold3	1	*Amblyomma*
8032	Ventral	ResNet-50	ventral aureolatum (7).jpg	fold3	1	*Amblyomma*
8033	Ventral	ResNet-50	ventral aureolatum (9).jpg	fold3	1	*Amblyomma*
8034	Ventral	ResNet-50	ventral cajennense (11).jpg	fold3	2	*Amblyomma*
8035	Ventral	ResNet-50	ventral cajennense (12).jpg	fold3	2	*Amblyomma*
8036	Ventral	ResNet-50	ventral cajennense (22).jpg	fold3	2	*Amblyomma*
8037	Ventral	ResNet-50	ventral cajennense (3).jpg	fold3	2	*Amblyomma*
8038	Ventral	ResNet-50	ventral cajennense (31).jpg	fold3	2	*Amblyomma*

8039	Ventral	ResNet-50	ventral cajennense (35).jpg	fold3	2	*Amblyomma*
8040	Ventral	ResNet-50	ventral cajennense (36).jpg	fold3	2	*Amblyomma*
8041	Ventral	ResNet-50	ventral cajennense (38).jpg	fold3	2	*Amblyomma*
8042	Ventral	ResNet-50	ventral cajennense (39).jpg	fold3	2	*Amblyomma*
8043	Ventral	ResNet-50	ventral cajennense (45).jpg	fold3	2	*Amblyomma*
8044	Ventral	ResNet-50	ventral cajennense (47).jpg	fold3	2	*Amblyomma*
8045	Ventral	ResNet-50	ventral cajennense (54).jpg	fold3	2	*Amblyomma*
8046	Ventral	ResNet-50	ventral cajennense (61).jpg	fold3	2	*Amblyomma*
8047	Ventral	ResNet-50	ventral cajennense (69).jpg	fold3	2	*Amblyomma*
8048	Ventral	ResNet-50	ventral cajennense (78).jpg	fold3	2	*Amblyomma*
8049	Ventral	ResNet-50	ventral cajennense (81).jpg	fold3	2	*Amblyomma*
8050	Ventral	ResNet-50	ventral dubitatum (11).jpg	fold3	3	*Amblyomma*
8051	Ventral	ResNet-50	ventral dubitatum (15).jpg	fold3	3	*Amblyomma*
8052	Ventral	ResNet-50	ventral dubitatum (2).jpg	fold3	3	*Amblyomma*
8053	Ventral	ResNet-50	ventral dubitatum (33).jpg	fold3	3	*Amblyomma*
8054	Ventral	ResNet-50	ventral dubitatum (34).jpg	fold3	3	*Amblyomma*
8055	Ventral	ResNet-50	ventral dubitatum (43).jpg	fold3	3	*Amblyomma*
8056	Ventral	ResNet-50	ventral dubitatum (5).JPG	fold3	3	*Amblyomma*
8057	Ventral	ResNet-50	ventral dubitatum (50).jpg	fold3	3	*Amblyomma*
8058	Ventral	ResNet-50	ventral dubitatum (52).jpg	fold3	3	*Amblyomma*
8059	Ventral	ResNet-50	ventral dubitatum (8).jpg	fold3	3	*Amblyomma*
8060	Ventral	ResNet-50	ventral ovale (12).jpg	fold3	4	*Amblyomma*
8061	Ventral	ResNet-50	ventral ovale (16).jpg	fold3	4	*Amblyomma*
8062	Ventral	ResNet-50	ventral ovale (2).jpg	fold3	4	*Amblyomma*
8063	Ventral	ResNet-50	ventral ovale (33).jpg	fold3	4	*Amblyomma*
8064	Ventral	ResNet-50	ventral ovale (35).jpg	fold3	4	*Amblyomma*
8065	Ventral	ResNet-50	ventral ovale (40).jpg	fold3	4	*Amblyomma*
8066	Ventral	ResNet-50	ventral ovale (41).jpg	fold3	4	*Amblyomma*
8067	Ventral	ResNet-50	ventral ovale (43).jpg	fold3	4	*Amblyomma*
8068	Ventral	ResNet-50	ventral ovale (48).jpg	fold3	4	*Amblyomma*
8069	Ventral	ResNet-50	ventral ovale (54).jpg	fold3	4	*Amblyomma*
8070	Ventral	ResNet-50	ventral ovale (55).jpg	fold3	4	*Amblyomma*
8071	Ventral	ResNet-50	ventral ovale (7).jpg	fold3	4	*Amblyomma*
8072	Ventral	ResNet-50	ventral sculptum (13).JPG	fold3	5	*Amblyomma*
8073	Ventral	ResNet-50	ventral sculptum (17).JPG	fold3	5	*Amblyomma*
8074	Ventral	ResNet-50	ventral sculptum (25).JPG	fold3	5	*Amblyomma*
8075	Ventral	ResNet-50	ventral sculptum (26).JPG	fold3	5	*Amblyomma*
8076	Ventral	ResNet-50	ventral sculptum (44).JPG	fold3	5	*Amblyomma*
8077	Ventral	ResNet-50	ventral sculptum (47).JPG	fold3	5	*Amblyomma*
8078	Ventral	ResNet-50	ventral sculptum (49).JPG	fold3	5	*Amblyomma*
8079	Ventral	ResNet-50	ventral sculptum (5).JPG	fold3	5	*Amblyomma*
8080	Ventral	ResNet-50	ventral sculptum (50).JPG	fold3	5	*Amblyomma*
8081	Ventral	ResNet-50	ventral sculptum (62).JPG	fold3	5	*Amblyomma*
8082	Ventral	ResNet-50	ventral sculptum (64).JPG	fold3	5	*Amblyomma*
8083	Ventral	ResNet-50	ventral sculptum (68).JPG	fold3	5	*Amblyomma*
8084	Ventral	ResNet-50	ventral sculptum (69).JPG	fold3	5	*Amblyomma*
8085	Ventral	ResNet-50	ventral sculptum (71).jpg	fold3	5	*Amblyomma*
8086	Ventral	ResNet-50	ventral sculptum (74).jpg	fold3	5	*Amblyomma*
8087	Ventral	ResNet-50	ventral triste (1).jpg	fold3	6	*Amblyomma*
8088	Ventral	ResNet-50	ventral triste (13).jpg	fold3	6	*Amblyomma*
8089	Ventral	ResNet-50	ventral triste (14).jpg	fold3	6	*Amblyomma*
8090	Ventral	ResNet-50	ventral triste (20).jpg	fold3	6	*Amblyomma*
8091	Ventral	ResNet-50	ventral triste (22).jpg	fold3	6	*Amblyomma*
8092	Ventral	ResNet-50	ventral triste (23).jpg	fold3	6	*Amblyomma*
8093	Ventral	ResNet-50	ventral triste (31).jpg	fold3	6	*Amblyomma*
8094	Ventral	ResNet-50	ventral triste (32).jpg	fold3	6	*Amblyomma*
8095	Ventral	ResNet-50	ventral triste (35).jpg	fold3	6	*Amblyomma*
8096	Ventral	ResNet-50	ventral triste (46).jpg	fold3	6	*Amblyomma*
8097	Ventral	ResNet-50	ventral triste (59).jpg	fold3	6	*Amblyomma*
8098	Ventral	ResNet-50	ventral triste (63).jpg	fold3	6	*Amblyomma*
8099	Ventral	ResNet-50	ventral triste (69).jpg	fold3	6	*Amblyomma*
8100	Ventral	ResNet-50	ventral triste (70).jpg	fold3	6	*Amblyomma*
8101	Ventral	ResNet-50	ventral triste (73).jpg	fold3	6	*Amblyomma*
8102	Ventral	ResNet-50	ventral aureolatum (1).jpg	fold4	1	*Amblyomma*
8103	Ventral	ResNet-50	ventral aureolatum (15).jpg	fold4	1	*Amblyomma*
8104	Ventral	ResNet-50	ventral aureolatum (35).jpg	fold4	1	*Amblyomma*
8105	Ventral	ResNet-50	ventral aureolatum (37).jpg	fold4	1	*Amblyomma*

8106	Ventral	ResNet-50	ventral aureolatum (41).jpg	fold4	1	*Amblyomma*
8107	Ventral	ResNet-50	ventral aureolatum (43).jpg	fold4	1	*Amblyomma*
8108	Ventral	ResNet-50	ventral aureolatum (46).jpg	fold4	1	*Amblyomma*
8109	Ventral	ResNet-50	ventral aureolatum (52).jpg	fold4	1	*Amblyomma*
8110	Ventral	ResNet-50	ventral aureolatum (54).jpg	fold4	1	*Amblyomma*
8111	Ventral	ResNet-50	ventral aureolatum (57).jpg	fold4	1	*Amblyomma*
8112	Ventral	ResNet-50	ventral aureolatum (6).jpg	fold4	1	*Amblyomma*
8113	Ventral	ResNet-50	ventral aureolatum (8).jpg	fold4	1	*Amblyomma*
8114	Ventral	ResNet-50	ventral cajennense (10).jpg	fold4	2	*Amblyomma*
8115	Ventral	ResNet-50	ventral cajennense (17).jpg	fold4	2	*Amblyomma*
8116	Ventral	ResNet-50	ventral cajennense (18).jpg	fold4	2	*Amblyomma*
8117	Ventral	ResNet-50	ventral cajennense (19).jpg	fold4	2	*Amblyomma*
8118	Ventral	ResNet-50	ventral cajennense (21).jpg	fold4	2	*Amblyomma*
8119	Ventral	ResNet-50	ventral cajennense (27).jpg	fold4	2	*Amblyomma*
8120	Ventral	ResNet-50	ventral cajennense (34).jpg	fold4	2	*Amblyomma*
8121	Ventral	ResNet-50	ventral cajennense (42).jpg	fold4	2	*Amblyomma*
8122	Ventral	ResNet-50	ventral cajennense (44).jpg	fold4	2	*Amblyomma*
8123	Ventral	ResNet-50	ventral cajennense (46).jpg	fold4	2	*Amblyomma*
8124	Ventral	ResNet-50	ventral cajennense (48).jpg	fold4	2	*Amblyomma*
8125	Ventral	ResNet-50	ventral cajennense (53).jpg	fold4	2	*Amblyomma*
8126	Ventral	ResNet-50	ventral cajennense (55).jpg	fold4	2	*Amblyomma*
8127	Ventral	ResNet-50	ventral cajennense (6).jpg	fold4	2	*Amblyomma*
8128	Ventral	ResNet-50	ventral cajennense (62).jpg	fold4	2	*Amblyomma*
8129	Ventral	ResNet-50	ventral cajennense (66).jpg	fold4	2	*Amblyomma*
8130	Ventral	ResNet-50	ventral cajennense (73).jpg	fold4	2	*Amblyomma*
8131	Ventral	ResNet-50	ventral dubitatum (1).jpg	fold4	3	*Amblyomma*
8132	Ventral	ResNet-50	ventral dubitatum (12).jpg	fold4	3	*Amblyomma*
8133	Ventral	ResNet-50	ventral dubitatum (13).jpg	fold4	3	*Amblyomma*
8134	Ventral	ResNet-50	ventral dubitatum (14).jpg	fold4	3	*Amblyomma*
8135	Ventral	ResNet-50	ventral dubitatum (20).jpg	fold4	3	*Amblyomma*
8136	Ventral	ResNet-50	ventral dubitatum (3).jpg	fold4	3	*Amblyomma*
8137	Ventral	ResNet-50	ventral dubitatum (32).jpg	fold4	3	*Amblyomma*
8138	Ventral	ResNet-50	ventral dubitatum (35).jpg	fold4	3	*Amblyomma*
8139	Ventral	ResNet-50	ventral dubitatum (37).jpg	fold4	3	*Amblyomma*
8140	Ventral	ResNet-50	ventral dubitatum (4).JPG	fold4	3	*Amblyomma*
8141	Ventral	ResNet-50	ventral dubitatum (44).jpg	fold4	3	*Amblyomma*
8142	Ventral	ResNet-50	ventral ovale (19).jpg	fold4	4	*Amblyomma*
8143	Ventral	ResNet-50	ventral ovale (23).jpg	fold4	4	*Amblyomma*
8144	Ventral	ResNet-50	ventral ovale (24).jpg	fold4	4	*Amblyomma*
8145	Ventral	ResNet-50	ventral ovale (30).jpg	fold4	4	*Amblyomma*
8146	Ventral	ResNet-50	ventral ovale (34).jpg	fold4	4	*Amblyomma*
8147	Ventral	ResNet-50	ventral ovale (37).jpg	fold4	4	*Amblyomma*
8148	Ventral	ResNet-50	ventral ovale (4).jpg	fold4	4	*Amblyomma*
8149	Ventral	ResNet-50	ventral ovale (45).jpg	fold4	4	*Amblyomma*
8150	Ventral	ResNet-50	ventral ovale (5).jpg	fold4	4	*Amblyomma*
8151	Ventral	ResNet-50	ventral ovale (50).jpg	fold4	4	*Amblyomma*
8152	Ventral	ResNet-50	ventral ovale (53).JPG	fold4	4	*Amblyomma*
8153	Ventral	ResNet-50	ventral ovale (6).jpg	fold4	4	*Amblyomma*
8154	Ventral	ResNet-50	ventral sculptum (15).JPG	fold4	5	*Amblyomma*
8155	Ventral	ResNet-50	ventral sculptum (16).JPG	fold4	5	*Amblyomma*
8156	Ventral	ResNet-50	ventral sculptum (21).JPG	fold4	5	*Amblyomma*
8157	Ventral	ResNet-50	ventral sculptum (22).JPG	fold4	5	*Amblyomma*
8158	Ventral	ResNet-50	ventral sculptum (27).JPG	fold4	5	*Amblyomma*
8159	Ventral	ResNet-50	ventral sculptum (3).jpg	fold4	5	*Amblyomma*
8160	Ventral	ResNet-50	ventral sculptum (33).jpg	fold4	5	*Amblyomma*
8161	Ventral	ResNet-50	ventral sculptum (37).JPG	fold4	5	*Amblyomma*
8162	Ventral	ResNet-50	ventral sculptum (41).JPG	fold4	5	*Amblyomma*
8163	Ventral	ResNet-50	ventral sculptum (42).JPG	fold4	5	*Amblyomma*
8164	Ventral	ResNet-50	ventral sculptum (55).JPG	fold4	5	*Amblyomma*
8165	Ventral	ResNet-50	ventral sculptum (59).JPG	fold4	5	*Amblyomma*
8166	Ventral	ResNet-50	ventral sculptum (65).JPG	fold4	5	*Amblyomma*
8167	Ventral	ResNet-50	ventral sculptum (66).JPG	fold4	5	*Amblyomma*
8168	Ventral	ResNet-50	ventral sculptum (8).JPG	fold4	5	*Amblyomma*
8169	Ventral	ResNet-50	ventral triste (10).jpg	fold4	6	*Amblyomma*
8170	Ventral	ResNet-50	ventral triste (15).jpg	fold4	6	*Amblyomma*
8171	Ventral	ResNet-50	ventral triste (18).jpg	fold4	6	*Amblyomma*
8172	Ventral	ResNet-50	ventral triste (19).jpg	fold4	6	*Amblyomma*

8173	Ventral	ResNet-50	ventral triste (25).jpg	fold4	6	*Amblyomma*
8174	Ventral	ResNet-50	ventral triste (28).jpg	fold4	6	*Amblyomma*
8175	Ventral	ResNet-50	ventral triste (29).jpg	fold4	6	*Amblyomma*
8176	Ventral	ResNet-50	ventral triste (34).jpg	fold4	6	*Amblyomma*
8177	Ventral	ResNet-50	ventral triste (36).jpg	fold4	6	*Amblyomma*
8178	Ventral	ResNet-50	ventral triste (4).jpg	fold4	6	*Amblyomma*
8179	Ventral	ResNet-50	ventral triste (44).jpg	fold4	6	*Amblyomma*
8180	Ventral	ResNet-50	ventral triste (5).jpg	fold4	6	*Amblyomma*
8181	Ventral	ResNet-50	ventral triste (6).jpg	fold4	6	*Amblyomma*
8182	Ventral	ResNet-50	ventral triste (60).jpg	fold4	6	*Amblyomma*
8183	Ventral	ResNet-50	ventral triste (71).jpg	fold4	6	*Amblyomma*
8184	Ventral	ResNet-50	ventral triste (74).jpg	fold4	6	*Amblyomma*
8185	Ventral	ResNet-50	ventral aureolatum (13).jpg	fold5	1	*Amblyomma*
8186	Ventral	ResNet-50	ventral aureolatum (14).jpg	fold5	1	*Amblyomma*
8187	Ventral	ResNet-50	ventral aureolatum (2).jpg	fold5	1	*Amblyomma*
8188	Ventral	ResNet-50	ventral aureolatum (23).jpg	fold5	1	*Amblyomma*
8189	Ventral	ResNet-50	ventral aureolatum (24).jpg	fold5	1	*Amblyomma*
8190	Ventral	ResNet-50	ventral aureolatum (25).jpg	fold5	1	*Amblyomma*
8191	Ventral	ResNet-50	ventral aureolatum (29).jpg	fold5	1	*Amblyomma*
8192	Ventral	ResNet-50	ventral aureolatum (30).jpg	fold5	1	*Amblyomma*
8193	Ventral	ResNet-50	ventral aureolatum (32).jpg	fold5	1	*Amblyomma*
8194	Ventral	ResNet-50	ventral aureolatum (45).jpg	fold5	1	*Amblyomma*
8195	Ventral	ResNet-50	ventral aureolatum (55).jpg	fold5	1	*Amblyomma*
8196	Ventral	ResNet-50	ventral cajennense (13).jpg	fold5	2	*Amblyomma*
8197	Ventral	ResNet-50	ventral cajennense (2).jpg	fold5	2	*Amblyomma*
8198	Ventral	ResNet-50	ventral cajennense (24).jpg	fold5	2	*Amblyomma*
8199	Ventral	ResNet-50	ventral cajennense (25).jpg	fold5	2	*Amblyomma*
8200	Ventral	ResNet-50	ventral cajennense (26).jpg	fold5	2	*Amblyomma*
8201	Ventral	ResNet-50	ventral cajennense (32).jpg	fold5	2	*Amblyomma*
8202	Ventral	ResNet-50	ventral cajennense (40).jpg	fold5	2	*Amblyomma*
8203	Ventral	ResNet-50	ventral cajennense (43).jpg	fold5	2	*Amblyomma*
8204	Ventral	ResNet-50	ventral cajennense (50).jpg	fold5	2	*Amblyomma*
8205	Ventral	ResNet-50	ventral cajennense (58).jpg	fold5	2	*Amblyomma*
8206	Ventral	ResNet-50	ventral cajennense (59).jpg	fold5	2	*Amblyomma*
8207	Ventral	ResNet-50	ventral cajennense (68).jpg	fold5	2	*Amblyomma*
8208	Ventral	ResNet-50	ventral cajennense (76).jpg	fold5	2	*Amblyomma*
8209	Ventral	ResNet-50	ventral cajennense (77).jpg	fold5	2	*Amblyomma*
8210	Ventral	ResNet-50	ventral cajennense (79).jpg	fold5	2	*Amblyomma*
8211	Ventral	ResNet-50	ventral cajennense (8).jpg	fold5	2	*Amblyomma*
8212	Ventral	ResNet-50	ventral cajennense (80).jpg	fold5	2	*Amblyomma*
8213	Ventral	ResNet-50	ventral dubitatum (10).jpg	fold5	3	*Amblyomma*
8214	Ventral	ResNet-50	ventral dubitatum (16).jpg	fold5	3	*Amblyomma*
8215	Ventral	ResNet-50	ventral dubitatum (17).jpg	fold5	3	*Amblyomma*
8216	Ventral	ResNet-50	ventral dubitatum (18).jpg	fold5	3	*Amblyomma*
8217	Ventral	ResNet-50	ventral dubitatum (21).jpg	fold5	3	*Amblyomma*
8218	Ventral	ResNet-50	ventral dubitatum (29).jpg	fold5	3	*Amblyomma*
8219	Ventral	ResNet-50	ventral dubitatum (31).jpg	fold5	3	*Amblyomma*
8220	Ventral	ResNet-50	ventral dubitatum (47).jpg	fold5	3	*Amblyomma*
8221	Ventral	ResNet-50	ventral dubitatum (6).jpg	fold5	3	*Amblyomma*
8222	Ventral	ResNet-50	ventral dubitatum (9).jpg	fold5	3	*Amblyomma*
8223	Ventral	ResNet-50	ventral ovale (11).jpg	fold5	4	*Amblyomma*
8224	Ventral	ResNet-50	ventral ovale (15).jpg	fold5	4	*Amblyomma*
8225	Ventral	ResNet-50	ventral ovale (17).jpg	fold5	4	*Amblyomma*
8226	Ventral	ResNet-50	ventral ovale (21).jpg	fold5	4	*Amblyomma*
8227	Ventral	ResNet-50	ventral ovale (25).jpg	fold5	4	*Amblyomma*
8228	Ventral	ResNet-50	ventral ovale (27).jpg	fold5	4	*Amblyomma*
8229	Ventral	ResNet-50	ventral ovale (39).jpg	fold5	4	*Amblyomma*
8230	Ventral	ResNet-50	ventral ovale (46).jpg	fold5	4	*Amblyomma*
8231	Ventral	ResNet-50	ventral ovale (47).jpg	fold5	4	*Amblyomma*
8232	Ventral	ResNet-50	ventral ovale (52).jpg	fold5	4	*Amblyomma*
8233	Ventral	ResNet-50	ventral ovale (56).jpg	fold5	4	*Amblyomma*
8234	Ventral	ResNet-50	ventral ovale (59).jpg	fold5	4	*Amblyomma*
8235	Ventral	ResNet-50	ventral sculptum (11).JPG	fold5	5	*Amblyomma*
8236	Ventral	ResNet-50	ventral sculptum (14).JPG	fold5	5	*Amblyomma*
8237	Ventral	ResNet-50	ventral sculptum (19).JPG	fold5	5	*Amblyomma*
8238	Ventral	ResNet-50	ventral sculptum (20).JPG	fold5	5	*Amblyomma*
8239	Ventral	ResNet-50	ventral sculptum (24).JPG	fold5	5	*Amblyomma*

8240	Ventral	ResNet-50	ventral sculptum (34).jpg	fold5	5	*Amblyomma*
8241	Ventral	ResNet-50	ventral sculptum (35).JPG	fold5	5	*Amblyomma*
8242	Ventral	ResNet-50	ventral sculptum (36).JPG	fold5	5	*Amblyomma*
8243	Ventral	ResNet-50	ventral sculptum (52).JPG	fold5	5	*Amblyomma*
8244	Ventral	ResNet-50	ventral sculptum (56).JPG	fold5	5	*Amblyomma*
8245	Ventral	ResNet-50	ventral sculptum (57).JPG	fold5	5	*Amblyomma*
8246	Ventral	ResNet-50	ventral sculptum (61).JPG	fold5	5	*Amblyomma*
8247	Ventral	ResNet-50	ventral sculptum (70).jpg	fold5	5	*Amblyomma*
8248	Ventral	ResNet-50	ventral sculptum (72).jpg	fold5	5	*Amblyomma*
8249	Ventral	ResNet-50	ventral sculptum (73).jpg	fold5	5	*Amblyomma*
8250	Ventral	ResNet-50	ventral triste (17).jpg	fold5	6	*Amblyomma*
8251	Ventral	ResNet-50	ventral triste (24).jpg	fold5	6	*Amblyomma*
8252	Ventral	ResNet-50	ventral triste (26).jpg	fold5	6	*Amblyomma*
8253	Ventral	ResNet-50	ventral triste (27).jpg	fold5	6	*Amblyomma*
8254	Ventral	ResNet-50	ventral triste (3).jpg	fold5	6	*Amblyomma*
8255	Ventral	ResNet-50	ventral triste (50).jpg	fold5	6	*Amblyomma*
8256	Ventral	ResNet-50	ventral triste (51).jpg	fold5	6	*Amblyomma*
8257	Ventral	ResNet-50	ventral triste (55).jpg	fold5	6	*Amblyomma*
8258	Ventral	ResNet-50	ventral triste (58).jpg	fold5	6	*Amblyomma*
8259	Ventral	ResNet-50	ventral triste (61).jpg	fold5	6	*Amblyomma*
8260	Ventral	ResNet-50	ventral triste (62).jpg	fold5	6	*Amblyomma*
8261	Ventral	ResNet-50	ventral triste (64).jpg	fold5	6	*Amblyomma*
8262	Ventral	ResNet-50	ventral triste (65).jpg	fold5	6	*Amblyomma*
8263	Ventral	ResNet-50	ventral triste (7).jpg	fold5	6	*Amblyomma*
8264	Ventral	ResNet-50	ventral triste (9).jpg	fold5	6	*Amblyomma*
8265	Low resolution	ResNet-50	baixa aureolatum (11).jpg	fold1	1	*Amblyomma*
8266	Low resolution	ResNet-50	baixa aureolatum (17).jpg	fold1	1	*Amblyomma*
8267	Low resolution	ResNet-50	baixa aureolatum (23).jpg	fold1	1	*Amblyomma*
8268	Low resolution	ResNet-50	baixa aureolatum (27).jpg	fold1	1	*Amblyomma*
8269	Low resolution	ResNet-50	baixa aureolatum (30).jpg	fold1	1	*Amblyomma*
8270	Low resolution	ResNet-50	baixa aureolatum (46).jpg	fold1	1	*Amblyomma*
8271	Low resolution	ResNet-50	baixa aureolatum (47).jpg	fold1	1	*Amblyomma*
8272	Low resolution	ResNet-50	baixa aureolatum (48).jpg	fold1	1	*Amblyomma*
8273	Low resolution	ResNet-50	baixa aureolatum (52).jpg	fold1	1	*Amblyomma*
8274	Low resolution	ResNet-50	baixa aureolatum (7).jpg	fold1	1	*Amblyomma*
8275	Low resolution	ResNet-50	baixa cajennense (1).jpg	fold1	2	*Amblyomma*
8276	Low resolution	ResNet-50	baixa cajennense (23).jpg	fold1	2	*Amblyomma*
8277	Low resolution	ResNet-50	baixa cajennense (27).jpg	fold1	2	*Amblyomma*
8278	Low resolution	ResNet-50	baixa cajennense (30).jpg	fold1	2	*Amblyomma*
8279	Low resolution	ResNet-50	baixa cajennense (31).jpg	fold1	2	*Amblyomma*
8280	Low resolution	ResNet-50	baixa cajennense (37).jpg	fold1	2	*Amblyomma*
8281	Low resolution	ResNet-50	baixa cajennense (4).jpg	fold1	2	*Amblyomma*
8282	Low resolution	ResNet-50	baixa cajennense (44).jpg	fold1	2	*Amblyomma*
8283	Low resolution	ResNet-50	baixa cajennense (48).jpg	fold1	2	*Amblyomma*
8284	Low resolution	ResNet-50	baixa cajennense (5).jpg	fold1	2	*Amblyomma*
8285	Low resolution	ResNet-50	baixa cajennense (50).jpg	fold1	2	*Amblyomma*
8286	Low resolution	ResNet-50	baixa cajennense (51).jpg	fold1	2	*Amblyomma*
8287	Low resolution	ResNet-50	baixa cajennense (64).jpg	fold1	2	*Amblyomma*
8288	Low resolution	ResNet-50	baixa cajennense (7).jpg	fold1	2	*Amblyomma*
8289	Low resolution	ResNet-50	baixa cajennense (77).jpg	fold1	2	*Amblyomma*
8290	Low resolution	ResNet-50	baixa cajennense (80).jpg	fold1	2	*Amblyomma*
8291	Low resolution	ResNet-50	baixa dubitatum (10).jpg	fold1	3	*Amblyomma*
8292	Low resolution	ResNet-50	baixa dubitatum (18).jpg	fold1	3	*Amblyomma*
8293	Low resolution	ResNet-50	baixa dubitatum (22).jpg	fold1	3	*Amblyomma*
8294	Low resolution	ResNet-50	baixa dubitatum (23).jpg	fold1	3	*Amblyomma*
8295	Low resolution	ResNet-50	baixa dubitatum (27).jpg	fold1	3	*Amblyomma*
8296	Low resolution	ResNet-50	baixa dubitatum (30).jpg	fold1	3	*Amblyomma*
8297	Low resolution	ResNet-50	baixa dubitatum (31).jpg	fold1	3	*Amblyomma*
8298	Low resolution	ResNet-50	baixa dubitatum (5).jpg	fold1	3	*Amblyomma*
8299	Low resolution	ResNet-50	baixa ovale (14).jpg	fold1	4	*Amblyomma*
8300	Low resolution	ResNet-50	baixa ovale (20).jpg	fold1	4	*Amblyomma*
8301	Low resolution	ResNet-50	baixa ovale (22).jpg	fold1	4	*Amblyomma*
8302	Low resolution	ResNet-50	baixa ovale (29).jpg	fold1	4	*Amblyomma*
8303	Low resolution	ResNet-50	baixa ovale (36).jpg	fold1	4	*Amblyomma*
8304	Low resolution	ResNet-50	baixa ovale (39).jpg	fold1	4	*Amblyomma*
8305	Low resolution	ResNet-50	baixa ovale (43).jpg	fold1	4	*Amblyomma*
8306	Low resolution	ResNet-50	baixa ovale (44).jpg	fold1	4	*Amblyomma*

8307	Low resolution	ResNet-50	baixa ovale (48).jpg	fold1	4	*Amblyomma*
8308	Low resolution	ResNet-50	baixa ovale (54).jpg	fold1	4	*Amblyomma*
8309	Low resolution	ResNet-50	baixa ovale (9).jpg	fold1	4	*Amblyomma*
8310	Low resolution	ResNet-50	baixa sculptum (17).jpg	fold1	5	*Amblyomma*
8311	Low resolution	ResNet-50	baixa sculptum (5).jpg	fold1	5	*Amblyomma*
8312	Low resolution	ResNet-50	baixa sculptum (6).jpg	fold1	5	*Amblyomma*
8313	Low resolution	ResNet-50	baixa sculptum (8).jpg	fold1	5	*Amblyomma*
8314	Low resolution	ResNet-50	baixa triste (12).jpg	fold1	6	*Amblyomma*
8315	Low resolution	ResNet-50	baixa triste (15).jpg	fold1	6	*Amblyomma*
8316	Low resolution	ResNet-50	baixa triste (16).jpg	fold1	6	*Amblyomma*
8317	Low resolution	ResNet-50	baixa triste (2).jpg	fold1	6	*Amblyomma*
8318	Low resolution	ResNet-50	baixa triste (23).jpg	fold1	6	*Amblyomma*
8319	Low resolution	ResNet-50	baixa triste (32).jpg	fold1	6	*Amblyomma*
8320	Low resolution	ResNet-50	baixa triste (33).jpg	fold1	6	*Amblyomma*
8321	Low resolution	ResNet-50	baixa triste (38).jpg	fold1	6	*Amblyomma*
8322	Low resolution	ResNet-50	baixa triste (42).jpg	fold1	6	*Amblyomma*
8323	Low resolution	ResNet-50	baixa triste (47).jpg	fold1	6	*Amblyomma*
8324	Low resolution	ResNet-50	baixa triste (48).jpg	fold1	6	*Amblyomma*
8325	Low resolution	ResNet-50	baixa triste (57).jpg	fold1	6	*Amblyomma*
8326	Low resolution	ResNet-50	baixa triste (63).jpg	fold1	6	*Amblyomma*
8327	Low resolution	ResNet-50	baixa triste (64).jpg	fold1	6	*Amblyomma*
8328	Low resolution	ResNet-50	baixa triste (7).jpg	fold1	6	*Amblyomma*
8329	Low resolution	ResNet-50	baixa triste (71).jpg	fold1	6	*Amblyomma*
8330	Low resolution	ResNet-50	baixa aureolatum (18).jpg	fold2	1	*Amblyomma*
8331	Low resolution	ResNet-50	baixa aureolatum (21).jpg	fold2	1	*Amblyomma*
8332	Low resolution	ResNet-50	baixa aureolatum (28).jpg	fold2	1	*Amblyomma*
8333	Low resolution	ResNet-50	baixa aureolatum (29).jpg	fold2	1	*Amblyomma*
8334	Low resolution	ResNet-50	baixa aureolatum (32).jpg	fold2	1	*Amblyomma*
8335	Low resolution	ResNet-50	baixa aureolatum (38).jpg	fold2	1	*Amblyomma*
8336	Low resolution	ResNet-50	baixa aureolatum (43).jpg	fold2	1	*Amblyomma*
8337	Low resolution	ResNet-50	baixa aureolatum (44).jpg	fold2	1	*Amblyomma*
8338	Low resolution	ResNet-50	baixa aureolatum (49).jpg	fold2	1	*Amblyomma*
8339	Low resolution	ResNet-50	baixa aureolatum (5).jpg	fold2	1	*Amblyomma*
8340	Low resolution	ResNet-50	baixa aureolatum (51).jpg	fold2	1	*Amblyomma*
8341	Low resolution	ResNet-50	baixa cajennense (13).jpg	fold2	2	*Amblyomma*
8342	Low resolution	ResNet-50	baixa cajennense (14).jpg	fold2	2	*Amblyomma*
8343	Low resolution	ResNet-50	baixa cajennense (34).jpg	fold2	2	*Amblyomma*
8344	Low resolution	ResNet-50	baixa cajennense (35).jpg	fold2	2	*Amblyomma*
8345	Low resolution	ResNet-50	baixa cajennense (39).jpg	fold2	2	*Amblyomma*
8346	Low resolution	ResNet-50	baixa cajennense (41).jpg	fold2	2	*Amblyomma*
8347	Low resolution	ResNet-50	baixa cajennense (43).jpg	fold2	2	*Amblyomma*
8348	Low resolution	ResNet-50	baixa cajennense (53).jpg	fold2	2	*Amblyomma*
8349	Low resolution	ResNet-50	baixa cajennense (56).jpg	fold2	2	*Amblyomma*
8350	Low resolution	ResNet-50	baixa cajennense (6).jpg	fold2	2	*Amblyomma*
8351	Low resolution	ResNet-50	baixa cajennense (61).jpg	fold2	2	*Amblyomma*
8352	Low resolution	ResNet-50	baixa cajennense (63).jpg	fold2	2	*Amblyomma*
8353	Low resolution	ResNet-50	baixa cajennense (67).jpg	fold2	2	*Amblyomma*
8354	Low resolution	ResNet-50	baixa cajennense (74).jpg	fold2	2	*Amblyomma*
8355	Low resolution	ResNet-50	baixa cajennense (75).jpg	fold2	2	*Amblyomma*
8356	Low resolution	ResNet-50	baixa cajennense (79).jpg	fold2	2	*Amblyomma*
8357	Low resolution	ResNet-50	baixa dubitatum (26).jpg	fold2	3	*Amblyomma*
8358	Low resolution	ResNet-50	baixa dubitatum (33).jpg	fold2	3	*Amblyomma*
8359	Low resolution	ResNet-50	baixa dubitatum (34).jpg	fold2	3	*Amblyomma*
8360	Low resolution	ResNet-50	baixa dubitatum (35).jpg	fold2	3	*Amblyomma*
8361	Low resolution	ResNet-50	baixa dubitatum (37).jpg	fold2	3	*Amblyomma*
8362	Low resolution	ResNet-50	baixa dubitatum (39).jpg	fold2	3	*Amblyomma*
8363	Low resolution	ResNet-50	baixa dubitatum (40).jpg	fold2	3	*Amblyomma*
8364	Low resolution	ResNet-50	baixa dubitatum (43).jpg	fold2	3	*Amblyomma*
8365	Low resolution	ResNet-50	baixa dubitatum (7).jpg	fold2	3	*Amblyomma*
8366	Low resolution	ResNet-50	baixa ovale (2).jpg	fold2	4	*Amblyomma*
8367	Low resolution	ResNet-50	baixa ovale (24).jpg	fold2	4	*Amblyomma*
8368	Low resolution	ResNet-50	baixa ovale (25).jpg	fold2	4	*Amblyomma*
8369	Low resolution	ResNet-50	baixa ovale (26).jpg	fold2	4	*Amblyomma*
8370	Low resolution	ResNet-50	baixa ovale (27).jpg	fold2	4	*Amblyomma*
8371	Low resolution	ResNet-50	baixa ovale (31).jpg	fold2	4	*Amblyomma*
8372	Low resolution	ResNet-50	baixa ovale (35).jpg	fold2	4	*Amblyomma*
8373	Low resolution	ResNet-50	baixa ovale (41).jpg	fold2	4	*Amblyomma*

8374	Low resolution	ResNet-50	baixa ovale (42).jpg	fold2	4	*Amblyomma*
8375	Low resolution	ResNet-50	baixa ovale (47).jpg	fold2	4	*Amblyomma*
8376	Low resolution	ResNet-50	baixa ovale (5).jpg	fold2	4	*Amblyomma*
8377	Low resolution	ResNet-50	baixa sculptum (13).jpg	fold2	5	*Amblyomma*
8378	Low resolution	ResNet-50	baixa sculptum (3).jpg	fold2	5	*Amblyomma*
8379	Low resolution	ResNet-50	baixa sculptum (7).jpg	fold2	5	*Amblyomma*
8380	Low resolution	ResNet-50	baixa triste (10).jpg	fold2	6	*Amblyomma*
8381	Low resolution	ResNet-50	baixa triste (11).jpg	fold2	6	*Amblyomma*
8382	Low resolution	ResNet-50	baixa triste (13).jpg	fold2	6	*Amblyomma*
8383	Low resolution	ResNet-50	baixa triste (17).jpg	fold2	6	*Amblyomma*
8384	Low resolution	ResNet-50	baixa triste (21).jpg	fold2	6	*Amblyomma*
8385	Low resolution	ResNet-50	baixa triste (25).jpg	fold2	6	*Amblyomma*
8386	Low resolution	ResNet-50	baixa triste (26).jpg	fold2	6	*Amblyomma*
8387	Low resolution	ResNet-50	baixa triste (30).jpg	fold2	6	*Amblyomma*
8388	Low resolution	ResNet-50	baixa triste (37).jpg	fold2	6	*Amblyomma*
8389	Low resolution	ResNet-50	baixa triste (40).jpg	fold2	6	*Amblyomma*
8390	Low resolution	ResNet-50	baixa triste (41).jpg	fold2	6	*Amblyomma*
8391	Low resolution	ResNet-50	baixa triste (50).jpg	fold2	6	*Amblyomma*
8392	Low resolution	ResNet-50	baixa triste (55).jpg	fold2	6	*Amblyomma*
8393	Low resolution	ResNet-50	baixa triste (61).jpg	fold2	6	*Amblyomma*
8394	Low resolution	ResNet-50	baixa triste (69).jpg	fold2	6	*Amblyomma*
8395	Low resolution	ResNet-50	baixa triste (75).jpg	fold2	6	*Amblyomma*
8396	Low resolution	ResNet-50	baixa aureolatum (1).jpg	fold3	1	*Amblyomma*
8397	Low resolution	ResNet-50	baixa aureolatum (14).jpg	fold3	1	*Amblyomma*
8398	Low resolution	ResNet-50	baixa aureolatum (19).jpg	fold3	1	*Amblyomma*
8399	Low resolution	ResNet-50	baixa aureolatum (24).jpg	fold3	1	*Amblyomma*
8400	Low resolution	ResNet-50	baixa aureolatum (33).jpg	fold3	1	*Amblyomma*
8401	Low resolution	ResNet-50	baixa aureolatum (35).jpg	fold3	1	*Amblyomma*
8402	Low resolution	ResNet-50	baixa aureolatum (36).jpg	fold3	1	*Amblyomma*
8403	Low resolution	ResNet-50	baixa aureolatum (40).jpg	fold3	1	*Amblyomma*
8404	Low resolution	ResNet-50	baixa aureolatum (53).jpg	fold3	1	*Amblyomma*
8405	Low resolution	ResNet-50	baixa aureolatum (6).jpg	fold3	1	*Amblyomma*
8406	Low resolution	ResNet-50	baixa cajennense (10).jpg	fold3	2	*Amblyomma*
8407	Low resolution	ResNet-50	baixa cajennense (11).jpg	fold3	2	*Amblyomma*
8408	Low resolution	ResNet-50	baixa cajennense (16).jpg	fold3	2	*Amblyomma*
8409	Low resolution	ResNet-50	baixa cajennense (18).jpg	fold3	2	*Amblyomma*
8410	Low resolution	ResNet-50	baixa cajennense (2).jpg	fold3	2	*Amblyomma*
8411	Low resolution	ResNet-50	baixa cajennense (26).jpg	fold3	2	*Amblyomma*
8412	Low resolution	ResNet-50	baixa cajennense (29).jpg	fold3	2	*Amblyomma*
8413	Low resolution	ResNet-50	baixa cajennense (38).jpg	fold3	2	*Amblyomma*
8414	Low resolution	ResNet-50	baixa cajennense (55).jpg	fold3	2	*Amblyomma*
8415	Low resolution	ResNet-50	baixa cajennense (57).jpg	fold3	2	*Amblyomma*
8416	Low resolution	ResNet-50	baixa cajennense (59).jpg	fold3	2	*Amblyomma*
8417	Low resolution	ResNet-50	baixa cajennense (62).jpg	fold3	2	*Amblyomma*
8418	Low resolution	ResNet-50	baixa cajennense (65).jpg	fold3	2	*Amblyomma*
8419	Low resolution	ResNet-50	baixa cajennense (66).jpg	fold3	2	*Amblyomma*
8420	Low resolution	ResNet-50	baixa cajennense (71).jpg	fold3	2	*Amblyomma*
8421	Low resolution	ResNet-50	baixa cajennense (76).jpg	fold3	2	*Amblyomma*
8422	Low resolution	ResNet-50	baixa cajennense (8).jpg	fold3	2	*Amblyomma*
8423	Low resolution	ResNet-50	baixa dubitatum (15).jpg	fold3	3	*Amblyomma*
8424	Low resolution	ResNet-50	baixa dubitatum (20).jpg	fold3	3	*Amblyomma*
8425	Low resolution	ResNet-50	baixa dubitatum (28).jpg	fold3	3	*Amblyomma*
8426	Low resolution	ResNet-50	baixa dubitatum (29).jpg	fold3	3	*Amblyomma*
8427	Low resolution	ResNet-50	baixa dubitatum (32).jpg	fold3	3	*Amblyomma*
8428	Low resolution	ResNet-50	baixa dubitatum (38).jpg	fold3	3	*Amblyomma*
8429	Low resolution	ResNet-50	baixa dubitatum (4).jpg	fold3	3	*Amblyomma*
8430	Low resolution	ResNet-50	baixa dubitatum (41).jpg	fold3	3	*Amblyomma*
8431	Low resolution	ResNet-50	baixa dubitatum (42).jpg	fold3	3	*Amblyomma*
8432	Low resolution	ResNet-50	baixa ovale (11).jpg	fold3	4	*Amblyomma*
8433	Low resolution	ResNet-50	baixa ovale (21).jpg	fold3	4	*Amblyomma*
8434	Low resolution	ResNet-50	baixa ovale (28).jpg	fold3	4	*Amblyomma*
8435	Low resolution	ResNet-50	baixa ovale (30).jpg	fold3	4	*Amblyomma*
8436	Low resolution	ResNet-50	baixa ovale (34).jpg	fold3	4	*Amblyomma*
8437	Low resolution	ResNet-50	baixa ovale (37).jpg	fold3	4	*Amblyomma*
8438	Low resolution	ResNet-50	baixa ovale (45).jpg	fold3	4	*Amblyomma*
8439	Low resolution	ResNet-50	baixa ovale (46).jpg	fold3	4	*Amblyomma*
8440	Low resolution	ResNet-50	baixa ovale (52).jpg	fold3	4	*Amblyomma*

8441	Low resolution	ResNet-50	baixa ovale (55).jpg	fold3	4	*Amblyomma*
8442	Low resolution	ResNet-50	baixa ovale (8).jpg	fold3	4	*Amblyomma*
8443	Low resolution	ResNet-50	baixa sculptum (1).jpg	fold3	5	*Amblyomma*
8444	Low resolution	ResNet-50	baixa sculptum (11).jpg	fold3	5	*Amblyomma*
8445	Low resolution	ResNet-50	baixa sculptum (12).jpg	fold3	5	*Amblyomma*
8446	Low resolution	ResNet-50	baixa sculptum (9).jpg	fold3	5	*Amblyomma*
8447	Low resolution	ResNet-50	baixa triste (1).jpg	fold3	6	*Amblyomma*
8448	Low resolution	ResNet-50	baixa triste (14).jpg	fold3	6	*Amblyomma*
8449	Low resolution	ResNet-50	baixa triste (24).jpg	fold3	6	*Amblyomma*
8450	Low resolution	ResNet-50	baixa triste (28).jpg	fold3	6	*Amblyomma*
8451	Low resolution	ResNet-50	baixa triste (34).jpg	fold3	6	*Amblyomma*
8452	Low resolution	ResNet-50	baixa triste (36).jpg	fold3	6	*Amblyomma*
8453	Low resolution	ResNet-50	baixa triste (39).jpg	fold3	6	*Amblyomma*
8454	Low resolution	ResNet-50	baixa triste (4).jpg	fold3	6	*Amblyomma*
8455	Low resolution	ResNet-50	baixa triste (52).jpg	fold3	6	*Amblyomma*
8456	Low resolution	ResNet-50	baixa triste (53).jpg	fold3	6	*Amblyomma*
8457	Low resolution	ResNet-50	baixa triste (66).jpg	fold3	6	*Amblyomma*
8458	Low resolution	ResNet-50	baixa triste (73).jpg	fold3	6	*Amblyomma*
8459	Low resolution	ResNet-50	baixa triste (74).jpg	fold3	6	*Amblyomma*
8460	Low resolution	ResNet-50	baixa triste (76).jpg	fold3	6	*Amblyomma*
8461	Low resolution	ResNet-50	baixa triste (77).jpg	fold3	6	*Amblyomma*
8462	Low resolution	ResNet-50	baixa aureolatum (12).jpg	fold4	1	*Amblyomma*
8463	Low resolution	ResNet-50	baixa aureolatum (13).jpg	fold4	1	*Amblyomma*
8464	Low resolution	ResNet-50	baixa aureolatum (15).jpg	fold4	1	*Amblyomma*
8465	Low resolution	ResNet-50	baixa aureolatum (16).jpg	fold4	1	*Amblyomma*
8466	Low resolution	ResNet-50	baixa aureolatum (20).jpg	fold4	1	*Amblyomma*
8467	Low resolution	ResNet-50	baixa aureolatum (22).jpg	fold4	1	*Amblyomma*
8468	Low resolution	ResNet-50	baixa aureolatum (25).jpg	fold4	1	*Amblyomma*
8469	Low resolution	ResNet-50	baixa aureolatum (26).jpg	fold4	1	*Amblyomma*
8470	Low resolution	ResNet-50	baixa aureolatum (37).jpg	fold4	1	*Amblyomma*
8471	Low resolution	ResNet-50	baixa aureolatum (4).jpg	fold4	1	*Amblyomma*
8472	Low resolution	ResNet-50	baixa aureolatum (8).jpg	fold4	1	*Amblyomma*
8473	Low resolution	ResNet-50	baixa cajennense (15).jpg	fold4	2	*Amblyomma*
8474	Low resolution	ResNet-50	baixa cajennense (17).jpg	fold4	2	*Amblyomma*
8475	Low resolution	ResNet-50	baixa cajennense (19).jpg	fold4	2	*Amblyomma*
8476	Low resolution	ResNet-50	baixa cajennense (20).jpg	fold4	2	*Amblyomma*
8477	Low resolution	ResNet-50	baixa cajennense (21).jpg	fold4	2	*Amblyomma*
8478	Low resolution	ResNet-50	baixa cajennense (22).jpg	fold4	2	*Amblyomma*
8479	Low resolution	ResNet-50	baixa cajennense (3).jpg	fold4	2	*Amblyomma*
8480	Low resolution	ResNet-50	baixa cajennense (42).jpg	fold4	2	*Amblyomma*
8481	Low resolution	ResNet-50	baixa cajennense (45).jpg	fold4	2	*Amblyomma*
8482	Low resolution	ResNet-50	baixa cajennense (47).jpg	fold4	2	*Amblyomma*
8483	Low resolution	ResNet-50	baixa cajennense (49).jpg	fold4	2	*Amblyomma*
8484	Low resolution	ResNet-50	baixa cajennense (54).jpg	fold4	2	*Amblyomma*
8485	Low resolution	ResNet-50	baixa cajennense (69).jpg	fold4	2	*Amblyomma*
8486	Low resolution	ResNet-50	baixa cajennense (72).jpg	fold4	2	*Amblyomma*
8487	Low resolution	ResNet-50	baixa cajennense (73).jpg	fold4	2	*Amblyomma*
8488	Low resolution	ResNet-50	baixa cajennense (81).jpg	fold4	2	*Amblyomma*
8489	Low resolution	ResNet-50	baixa dubitatum (1).jpg	fold4	3	*Amblyomma*
8490	Low resolution	ResNet-50	baixa dubitatum (13).jpg	fold4	3	*Amblyomma*
8491	Low resolution	ResNet-50	baixa dubitatum (14).jpg	fold4	3	*Amblyomma*
8492	Low resolution	ResNet-50	baixa dubitatum (17).jpg	fold4	3	*Amblyomma*
8493	Low resolution	ResNet-50	baixa dubitatum (2).jpg	fold4	3	*Amblyomma*
8494	Low resolution	ResNet-50	baixa dubitatum (21).jpg	fold4	3	*Amblyomma*
8495	Low resolution	ResNet-50	baixa dubitatum (36).jpg	fold4	3	*Amblyomma*
8496	Low resolution	ResNet-50	baixa dubitatum (8).jpg	fold4	3	*Amblyomma*
8497	Low resolution	ResNet-50	baixa dubitatum (9).jpg	fold4	3	*Amblyomma*
8498	Low resolution	ResNet-50	baixa ovale (10).jpg	fold4	4	*Amblyomma*
8499	Low resolution	ResNet-50	baixa ovale (13).jpg	fold4	4	*Amblyomma*
8500	Low resolution	ResNet-50	baixa ovale (17).jpg	fold4	4	*Amblyomma*
8501	Low resolution	ResNet-50	baixa ovale (18).jpg	fold4	4	*Amblyomma*
8502	Low resolution	ResNet-50	baixa ovale (19).jpg	fold4	4	*Amblyomma*
8503	Low resolution	ResNet-50	baixa ovale (38).jpg	fold4	4	*Amblyomma*
8504	Low resolution	ResNet-50	baixa ovale (4).jpg	fold4	4	*Amblyomma*
8505	Low resolution	ResNet-50	baixa ovale (40).jpg	fold4	4	*Amblyomma*
8506	Low resolution	ResNet-50	baixa ovale (51).jpg	fold4	4	*Amblyomma*
8507	Low resolution	ResNet-50	baixa ovale (53).jpg	fold4	4	*Amblyomma*

8508	Low resolution	ResNet-50	baixa ovale (7).jpg	fold4	4	*Amblyomma*
8509	Low resolution	ResNet-50	baixa sculptum (15).jpg	fold4	5	*Amblyomma*
8510	Low resolution	ResNet-50	baixa sculptum (16).jpg	fold4	5	*Amblyomma*
8511	Low resolution	ResNet-50	baixa sculptum (2).jpg	fold4	5	*Amblyomma*
8512	Low resolution	ResNet-50	baixa triste (18).jpg	fold4	6	*Amblyomma*
8513	Low resolution	ResNet-50	baixa triste (19).jpg	fold4	6	*Amblyomma*
8514	Low resolution	ResNet-50	baixa triste (45).jpg	fold4	6	*Amblyomma*
8515	Low resolution	ResNet-50	baixa triste (46).jpg	fold4	6	*Amblyomma*
8516	Low resolution	ResNet-50	baixa triste (5).jpg	fold4	6	*Amblyomma*
8517	Low resolution	ResNet-50	baixa triste (51).jpg	fold4	6	*Amblyomma*
8518	Low resolution	ResNet-50	baixa triste (54).jpg	fold4	6	*Amblyomma*
8519	Low resolution	ResNet-50	baixa triste (6).jpg	fold4	6	*Amblyomma*
8520	Low resolution	ResNet-50	baixa triste (60).jpg	fold4	6	*Amblyomma*
8521	Low resolution	ResNet-50	baixa triste (62).jpg	fold4	6	*Amblyomma*
8522	Low resolution	ResNet-50	baixa triste (67).jpg	fold4	6	*Amblyomma*
8523	Low resolution	ResNet-50	baixa triste (68).jpg	fold4	6	*Amblyomma*
8524	Low resolution	ResNet-50	baixa triste (70).jpg	fold4	6	*Amblyomma*
8525	Low resolution	ResNet-50	baixa triste (72).jpg	fold4	6	*Amblyomma*
8526	Low resolution	ResNet-50	baixa triste (8).jpg	fold4	6	*Amblyomma*
8527	Low resolution	ResNet-50	baixa aureolatum (10).jpg	fold5	1	*Amblyomma*
8528	Low resolution	ResNet-50	baixa aureolatum (2).jpg	fold5	1	*Amblyomma*
8529	Low resolution	ResNet-50	baixa aureolatum (3).jpg	fold5	1	*Amblyomma*
8530	Low resolution	ResNet-50	baixa aureolatum (31).jpg	fold5	1	*Amblyomma*
8531	Low resolution	ResNet-50	baixa aureolatum (34).jpg	fold5	1	*Amblyomma*
8532	Low resolution	ResNet-50	baixa aureolatum (39).jpg	fold5	1	*Amblyomma*
8533	Low resolution	ResNet-50	baixa aureolatum (41).jpg	fold5	1	*Amblyomma*
8534	Low resolution	ResNet-50	baixa aureolatum (42).jpg	fold5	1	*Amblyomma*
8535	Low resolution	ResNet-50	baixa aureolatum (45).jpg	fold5	1	*Amblyomma*
8536	Low resolution	ResNet-50	baixa aureolatum (50).jpg	fold5	1	*Amblyomma*
8537	Low resolution	ResNet-50	baixa aureolatum (9).jpg	fold5	1	*Amblyomma*
8538	Low resolution	ResNet-50	baixa cajennense (12).jpg	fold5	2	*Amblyomma*
8539	Low resolution	ResNet-50	baixa cajennense (24).jpg	fold5	2	*Amblyomma*
8540	Low resolution	ResNet-50	baixa cajennense (25).jpg	fold5	2	*Amblyomma*
8541	Low resolution	ResNet-50	baixa cajennense (28).jpg	fold5	2	*Amblyomma*
8542	Low resolution	ResNet-50	baixa cajennense (32).jpg	fold5	2	*Amblyomma*
8543	Low resolution	ResNet-50	baixa cajennense (33).jpg	fold5	2	*Amblyomma*
8544	Low resolution	ResNet-50	baixa cajennense (36).jpg	fold5	2	*Amblyomma*
8545	Low resolution	ResNet-50	baixa cajennense (40).jpg	fold5	2	*Amblyomma*
8546	Low resolution	ResNet-50	baixa cajennense (46).jpg	fold5	2	*Amblyomma*
8547	Low resolution	ResNet-50	baixa cajennense (52).jpg	fold5	2	*Amblyomma*
8548	Low resolution	ResNet-50	baixa cajennense (58).jpg	fold5	2	*Amblyomma*
8549	Low resolution	ResNet-50	baixa cajennense (60).jpg	fold5	2	*Amblyomma*
8550	Low resolution	ResNet-50	baixa cajennense (68).jpg	fold5	2	*Amblyomma*
8551	Low resolution	ResNet-50	baixa cajennense (70).jpg	fold5	2	*Amblyomma*
8552	Low resolution	ResNet-50	baixa cajennense (78).jpg	fold5	2	*Amblyomma*
8553	Low resolution	ResNet-50	baixa cajennense (82).jpg	fold5	2	*Amblyomma*
8554	Low resolution	ResNet-50	baixa cajennense (9).jpg	fold5	2	*Amblyomma*
8555	Low resolution	ResNet-50	baixa dubitatum (11).jpg	fold5	3	*Amblyomma*
8556	Low resolution	ResNet-50	baixa dubitatum (12).jpg	fold5	3	*Amblyomma*
8557	Low resolution	ResNet-50	baixa dubitatum (16).jpg	fold5	3	*Amblyomma*
8558	Low resolution	ResNet-50	baixa dubitatum (19).jpg	fold5	3	*Amblyomma*
8559	Low resolution	ResNet-50	baixa dubitatum (24).jpg	fold5	3	*Amblyomma*
8560	Low resolution	ResNet-50	baixa dubitatum (25).jpg	fold5	3	*Amblyomma*
8561	Low resolution	ResNet-50	baixa dubitatum (3).jpg	fold5	3	*Amblyomma*
8562	Low resolution	ResNet-50	baixa dubitatum (44).jpg	fold5	3	*Amblyomma*
8563	Low resolution	ResNet-50	baixa dubitatum (6).jpg	fold5	3	*Amblyomma*
8564	Low resolution	ResNet-50	baixa ovale (1).jpg	fold5	4	*Amblyomma*
8565	Low resolution	ResNet-50	baixa ovale (12).jpg	fold5	4	*Amblyomma*
8566	Low resolution	ResNet-50	baixa ovale (15).jpg	fold5	4	*Amblyomma*
8567	Low resolution	ResNet-50	baixa ovale (16).jpg	fold5	4	*Amblyomma*
8568	Low resolution	ResNet-50	baixa ovale (23).jpg	fold5	4	*Amblyomma*
8569	Low resolution	ResNet-50	baixa ovale (3).jpg	fold5	4	*Amblyomma*
8570	Low resolution	ResNet-50	baixa ovale (32).jpg	fold5	4	*Amblyomma*
8571	Low resolution	ResNet-50	baixa ovale (33).jpg	fold5	4	*Amblyomma*
8572	Low resolution	ResNet-50	baixa ovale (49).jpg	fold5	4	*Amblyomma*
8573	Low resolution	ResNet-50	baixa ovale (50).jpg	fold5	4	*Amblyomma*
8574	Low resolution	ResNet-50	baixa ovale (6).jpg	fold5	4	*Amblyomma*

8575	Low resolution	ResNet-50	baixa sculptum (10).jpg	fold5	5	*Amblyomma*
8576	Low resolution	ResNet-50	baixa sculptum (14).jpg	fold5	5	*Amblyomma*
8577	Low resolution	ResNet-50	baixa sculptum (4).jpg	fold5	5	*Amblyomma*
8578	Low resolution	ResNet-50	baixa triste (20).jpg	fold5	6	*Amblyomma*
8579	Low resolution	ResNet-50	baixa triste (22).jpg	fold5	6	*Amblyomma*
8580	Low resolution	ResNet-50	baixa triste (27).jpg	fold5	6	*Amblyomma*
8581	Low resolution	ResNet-50	baixa triste (29).jpg	fold5	6	*Amblyomma*
8582	Low resolution	ResNet-50	baixa triste (3).jpg	fold5	6	*Amblyomma*
8583	Low resolution	ResNet-50	baixa triste (31).jpg	fold5	6	*Amblyomma*
8584	Low resolution	ResNet-50	baixa triste (35).jpg	fold5	6	*Amblyomma*
8585	Low resolution	ResNet-50	baixa triste (43).jpg	fold5	6	*Amblyomma*
8586	Low resolution	ResNet-50	baixa triste (44).jpg	fold5	6	*Amblyomma*
8587	Low resolution	ResNet-50	baixa triste (49).jpg	fold5	6	*Amblyomma*
8588	Low resolution	ResNet-50	baixa triste (56).jpg	fold5	6	*Amblyomma*
8589	Low resolution	ResNet-50	baixa triste (58).jpg	fold5	6	*Amblyomma*
8590	Low resolution	ResNet-50	baixa triste (59).jpg	fold5	6	*Amblyomma*
8591	Low resolution	ResNet-50	baixa triste (65).jpg	fold5	6	*Amblyomma*
8592	Low resolution	ResNet-50	baixa triste (9).jpg	fold5	6	*Amblyomma*
8593	High resolution	ResNet-50	alta aureolatum (11).jpg	fold1	1	*Amblyomma*
8594	High resolution	ResNet-50	alta aureolatum (17).jpg	fold1	1	*Amblyomma*
8595	High resolution	ResNet-50	alta aureolatum (23).jpg	fold1	1	*Amblyomma*
8596	High resolution	ResNet-50	alta aureolatum (27).jpg	fold1	1	*Amblyomma*
8597	High resolution	ResNet-50	alta aureolatum (30).jpg	fold1	1	*Amblyomma*
8598	High resolution	ResNet-50	alta aureolatum (46).jpg	fold1	1	*Amblyomma*
8599	High resolution	ResNet-50	alta aureolatum (47).jpg	fold1	1	*Amblyomma*
8600	High resolution	ResNet-50	alta aureolatum (48).jpg	fold1	1	*Amblyomma*
8601	High resolution	ResNet-50	alta aureolatum (52).jpg	fold1	1	*Amblyomma*
8602	High resolution	ResNet-50	alta aureolatum (55).jpg	fold1	1	*Amblyomma*
8603	High resolution	ResNet-50	alta aureolatum (58).jpg	fold1	1	*Amblyomma*
8604	High resolution	ResNet-50	alta aureolatum (61).jpg	fold1	1	*Amblyomma*
8605	High resolution	ResNet-50	alta aureolatum (65).jpg	fold1	1	*Amblyomma*
8606	High resolution	ResNet-50	alta cajennense (11).jpg	fold1	2	*Amblyomma*
8607	High resolution	ResNet-50	alta cajennense (16).jpg	fold1	2	*Amblyomma*
8608	High resolution	ResNet-50	alta cajennense (29).jpg	fold1	2	*Amblyomma*
8609	High resolution	ResNet-50	alta cajennense (3).jpg	fold1	2	*Amblyomma*
8610	High resolution	ResNet-50	alta cajennense (31).jpg	fold1	2	*Amblyomma*
8611	High resolution	ResNet-50	alta cajennense (33).jpg	fold1	2	*Amblyomma*
8612	High resolution	ResNet-50	alta cajennense (35).jpg	fold1	2	*Amblyomma*
8613	High resolution	ResNet-50	alta cajennense (40).jpg	fold1	2	*Amblyomma*
8614	High resolution	ResNet-50	alta cajennense (42).jpg	fold1	2	*Amblyomma*
8615	High resolution	ResNet-50	alta cajennense (55).jpg	fold1	2	*Amblyomma*
8616	High resolution	ResNet-50	alta cajennense (59).jpg	fold1	2	*Amblyomma*
8617	High resolution	ResNet-50	alta cajennense (6).jpg	fold1	2	*Amblyomma*
8618	High resolution	ResNet-50	alta cajennense (68).jpg	fold1	2	*Amblyomma*
8619	High resolution	ResNet-50	alta cajennense (71).jpg	fold1	2	*Amblyomma*
8620	High resolution	ResNet-50	alta cajennense (73).jpg	fold1	2	*Amblyomma*
8621	High resolution	ResNet-50	alta cajennense (74).jpg	fold1	2	*Amblyomma*
8622	High resolution	ResNet-50	alta cajennense (80).jpg	fold1	2	*Amblyomma*
8623	High resolution	ResNet-50	alta dubitatum (11).jpg	fold1	3	*Amblyomma*
8624	High resolution	ResNet-50	alta dubitatum (19).jpg	fold1	3	*Amblyomma*
8625	High resolution	ResNet-50	alta dubitatum (2).jpg	fold1	3	*Amblyomma*
8626	High resolution	ResNet-50	alta dubitatum (20).jpg	fold1	3	*Amblyomma*
8627	High resolution	ResNet-50	alta dubitatum (24).jpg	fold1	3	*Amblyomma*
8628	High resolution	ResNet-50	alta dubitatum (26).jpg	fold1	3	*Amblyomma*
8629	High resolution	ResNet-50	alta dubitatum (29).jpg	fold1	3	*Amblyomma*
8630	High resolution	ResNet-50	alta dubitatum (3).jpg	fold1	3	*Amblyomma*
8631	High resolution	ResNet-50	alta dubitatum (32).jpg	fold1	3	*Amblyomma*
8632	High resolution	ResNet-50	alta dubitatum (40).jpg	fold1	3	*Amblyomma*
8633	High resolution	ResNet-50	alta dubitatum (48).jpg	fold1	3	*Amblyomma*
8634	High resolution	ResNet-50	alta dubitatum (49).jpg	fold1	3	*Amblyomma*
8635	High resolution	ResNet-50	alta dubitatum (9).JPG	fold1	3	*Amblyomma*
8636	High resolution	ResNet-50	alta ovale (12).jpg	fold1	4	*Amblyomma*
8637	High resolution	ResNet-50	alta ovale (19).jpg	fold1	4	*Amblyomma*
8638	High resolution	ResNet-50	alta ovale (2).jpg	fold1	4	*Amblyomma*
8639	High resolution	ResNet-50	alta ovale (23).jpg	fold1	4	*Amblyomma*
8640	High resolution	ResNet-50	alta ovale (24).jpg	fold1	4	*Amblyomma*
8641	High resolution	ResNet-50	alta ovale (26).jpg	fold1	4	*Amblyomma*

8642	High resolution	ResNet-50	alta ovale (42).jpg	fold1	4	*Amblyomma*
8643	High resolution	ResNet-50	alta ovale (51).jpg	fold1	4	*Amblyomma*
8644	High resolution	ResNet-50	alta ovale (52).jpg	fold1	4	*Amblyomma*
8645	High resolution	ResNet-50	alta ovale (54).jpg	fold1	4	*Amblyomma*
8646	High resolution	ResNet-50	alta ovale (58).JPG	fold1	4	*Amblyomma*
8647	High resolution	ResNet-50	alta ovale (62).jpg	fold1	4	*Amblyomma*
8648	High resolution	ResNet-50	alta ovale (66).jpg	fold1	4	*Amblyomma*
8649	High resolution	ResNet-50	alta sculptum (100).JPG	fold1	5	*Amblyomma*
8650	High resolution	ResNet-50	alta sculptum (109).JPG	fold1	5	*Amblyomma*
8651	High resolution	ResNet-50	alta sculptum (11).JPG	fold1	5	*Amblyomma*
8652	High resolution	ResNet-50	alta sculptum (110).JPG	fold1	5	*Amblyomma*
8653	High resolution	ResNet-50	alta sculptum (114).JPG	fold1	5	*Amblyomma*
8654	High resolution	ResNet-50	alta sculptum (121).JPG	fold1	5	*Amblyomma*
8655	High resolution	ResNet-50	alta sculptum (123).JPG	fold1	5	*Amblyomma*
8656	High resolution	ResNet-50	alta sculptum (124).JPG	fold1	5	*Amblyomma*
8657	High resolution	ResNet-50	alta sculptum (130).JPG	fold1	5	*Amblyomma*
8658	High resolution	ResNet-50	alta sculptum (19).JPG	fold1	5	*Amblyomma*
8659	High resolution	ResNet-50	alta sculptum (2).jpg	fold1	5	*Amblyomma*
8660	High resolution	ResNet-50	alta sculptum (21).JPG	fold1	5	*Amblyomma*
8661	High resolution	ResNet-50	alta sculptum (22).JPG	fold1	5	*Amblyomma*
8662	High resolution	ResNet-50	alta sculptum (26).JPG	fold1	5	*Amblyomma*
8663	High resolution	ResNet-50	alta sculptum (3).jpg	fold1	5	*Amblyomma*
8664	High resolution	ResNet-50	alta sculptum (36).JPG	fold1	5	*Amblyomma*
8665	High resolution	ResNet-50	alta sculptum (49).JPG	fold1	5	*Amblyomma*
8666	High resolution	ResNet-50	alta sculptum (54).JPG	fold1	5	*Amblyomma*
8667	High resolution	ResNet-50	alta sculptum (57).jpg	fold1	5	*Amblyomma*
8668	High resolution	ResNet-50	alta sculptum (58).jpg	fold1	5	*Amblyomma*
8669	High resolution	ResNet-50	alta sculptum (6).jpg	fold1	5	*Amblyomma*
8670	High resolution	ResNet-50	alta sculptum (65).JPG	fold1	5	*Amblyomma*
8671	High resolution	ResNet-50	alta sculptum (66).JPG	fold1	5	*Amblyomma*
8672	High resolution	ResNet-50	alta sculptum (75).JPG	fold1	5	*Amblyomma*
8673	High resolution	ResNet-50	alta sculptum (79).JPG	fold1	5	*Amblyomma*
8674	High resolution	ResNet-50	alta sculptum (80).JPG	fold1	5	*Amblyomma*
8675	High resolution	ResNet-50	alta sculptum (94).JPG	fold1	5	*Amblyomma*
8676	High resolution	ResNet-50	alta triste (1).jpg	fold1	6	*Amblyomma*
8677	High resolution	ResNet-50	alta triste (14).jpg	fold1	6	*Amblyomma*
8678	High resolution	ResNet-50	alta triste (16).jpg	fold1	6	*Amblyomma*
8679	High resolution	ResNet-50	alta triste (27).jpg	fold1	6	*Amblyomma*
8680	High resolution	ResNet-50	alta triste (29).jpg	fold1	6	*Amblyomma*
8681	High resolution	ResNet-50	alta triste (4).jpg	fold1	6	*Amblyomma*
8682	High resolution	ResNet-50	alta triste (48).jpg	fold1	6	*Amblyomma*
8683	High resolution	ResNet-50	alta triste (51).jpg	fold1	6	*Amblyomma*
8684	High resolution	ResNet-50	alta triste (52).jpg	fold1	6	*Amblyomma*
8685	High resolution	ResNet-50	alta triste (57).jpg	fold1	6	*Amblyomma*
8686	High resolution	ResNet-50	alta triste (60).jpg	fold1	6	*Amblyomma*
8687	High resolution	ResNet-50	alta triste (69).jpg	fold1	6	*Amblyomma*
8688	High resolution	ResNet-50	alta triste (76).jpg	fold1	6	*Amblyomma*
8689	High resolution	ResNet-50	alta triste (78).jpg	fold1	6	*Amblyomma*
8690	High resolution	ResNet-50	alta triste (79).jpg	fold1	6	*Amblyomma*
8691	High resolution	ResNet-50	alta triste (8).jpg	fold1	6	*Amblyomma*
8692	High resolution	ResNet-50	alta aureolatum (18).jpg	fold2	1	*Amblyomma*
8693	High resolution	ResNet-50	alta aureolatum (21).jpg	fold2	1	*Amblyomma*
8694	High resolution	ResNet-50	alta aureolatum (28).jpg	fold2	1	*Amblyomma*
8695	High resolution	ResNet-50	alta aureolatum (29).jpg	fold2	1	*Amblyomma*
8696	High resolution	ResNet-50	alta aureolatum (32).jpg	fold2	1	*Amblyomma*
8697	High resolution	ResNet-50	alta aureolatum (38).jpg	fold2	1	*Amblyomma*
8698	High resolution	ResNet-50	alta aureolatum (43).jpg	fold2	1	*Amblyomma*
8699	High resolution	ResNet-50	alta aureolatum (44).jpg	fold2	1	*Amblyomma*
8700	High resolution	ResNet-50	alta aureolatum (49).jpg	fold2	1	*Amblyomma*
8701	High resolution	ResNet-50	alta aureolatum (5).jpg	fold2	1	*Amblyomma*
8702	High resolution	ResNet-50	alta aureolatum (51).jpg	fold2	1	*Amblyomma*
8703	High resolution	ResNet-50	alta aureolatum (6).jpg	fold2	1	*Amblyomma*
8704	High resolution	ResNet-50	alta aureolatum (62).jpg	fold2	1	*Amblyomma*
8705	High resolution	ResNet-50	alta aureolatum (9).jpg	fold2	1	*Amblyomma*
8706	High resolution	ResNet-50	alta cajennense (12).jpg	fold2	2	*Amblyomma*
8707	High resolution	ResNet-50	alta cajennense (15).jpg	fold2	2	*Amblyomma*
8708	High resolution	ResNet-50	alta cajennense (24).jpg	fold2	2	*Amblyomma*

8709	High resolution	ResNet-50	alta cajennense (30).jpg	fold2	2	*Amblyomma*
8710	High resolution	ResNet-50	alta cajennense (41).jpg	fold2	2	*Amblyomma*
8711	High resolution	ResNet-50	alta cajennense (43).jpg	fold2	2	*Amblyomma*
8712	High resolution	ResNet-50	alta cajennense (45).jpg	fold2	2	*Amblyomma*
8713	High resolution	ResNet-50	alta cajennense (49).jpg	fold2	2	*Amblyomma*
8714	High resolution	ResNet-50	alta cajennense (51).jpg	fold2	2	*Amblyomma*
8715	High resolution	ResNet-50	alta cajennense (52).jpg	fold2	2	*Amblyomma*
8716	High resolution	ResNet-50	alta cajennense (56).jpg	fold2	2	*Amblyomma*
8717	High resolution	ResNet-50	alta cajennense (58).jpg	fold2	2	*Amblyomma*
8718	High resolution	ResNet-50	alta cajennense (62).jpg	fold2	2	*Amblyomma*
8719	High resolution	ResNet-50	alta cajennense (66).jpg	fold2	2	*Amblyomma*
8720	High resolution	ResNet-50	alta cajennense (77).jpg	fold2	2	*Amblyomma*
8721	High resolution	ResNet-50	alta cajennense (78).jpg	fold2	2	*Amblyomma*
8722	High resolution	ResNet-50	alta cajennense (81).jpg	fold2	2	*Amblyomma*
8723	High resolution	ResNet-50	alta dubitatum (21).jpg	fold2	3	*Amblyomma*
8724	High resolution	ResNet-50	alta dubitatum (22).jpg	fold2	3	*Amblyomma*
8725	High resolution	ResNet-50	alta dubitatum (33).jpg	fold2	3	*Amblyomma*
8726	High resolution	ResNet-50	alta dubitatum (34).jpg	fold2	3	*Amblyomma*
8727	High resolution	ResNet-50	alta dubitatum (35).jpg	fold2	3	*Amblyomma*
8728	High resolution	ResNet-50	alta dubitatum (37).jpg	fold2	3	*Amblyomma*
8729	High resolution	ResNet-50	alta dubitatum (38).jpg	fold2	3	*Amblyomma*
8730	High resolution	ResNet-50	alta dubitatum (43).jpg	fold2	3	*Amblyomma*
8731	High resolution	ResNet-50	alta dubitatum (47).jpg	fold2	3	*Amblyomma*
8732	High resolution	ResNet-50	alta dubitatum (51).jpg	fold2	3	*Amblyomma*
8733	High resolution	ResNet-50	alta dubitatum (58).jpg	fold2	3	*Amblyomma*
8734	High resolution	ResNet-50	alta dubitatum (60).jpg	fold2	3	*Amblyomma*
8735	High resolution	ResNet-50	alta dubitatum (64).jpg	fold2	3	*Amblyomma*
8736	High resolution	ResNet-50	alta ovale (18).jpg	fold2	4	*Amblyomma*
8737	High resolution	ResNet-50	alta ovale (27).jpg	fold2	4	*Amblyomma*
8738	High resolution	ResNet-50	alta ovale (28).jpg	fold2	4	*Amblyomma*
8739	High resolution	ResNet-50	alta ovale (38).jpg	fold2	4	*Amblyomma*
8740	High resolution	ResNet-50	alta ovale (4).jpg	fold2	4	*Amblyomma*
8741	High resolution	ResNet-50	alta ovale (41).jpg	fold2	4	*Amblyomma*
8742	High resolution	ResNet-50	alta ovale (43).jpg	fold2	4	*Amblyomma*
8743	High resolution	ResNet-50	alta ovale (44).jpg	fold2	4	*Amblyomma*
8744	High resolution	ResNet-50	alta ovale (48).jpg	fold2	4	*Amblyomma*
8745	High resolution	ResNet-50	alta ovale (50).jpg	fold2	4	*Amblyomma*
8746	High resolution	ResNet-50	alta ovale (55).jpg	fold2	4	*Amblyomma*
8747	High resolution	ResNet-50	alta ovale (65).jpg	fold2	4	*Amblyomma*
8748	High resolution	ResNet-50	alta ovale (9).jpg	fold2	4	*Amblyomma*
8749	High resolution	ResNet-50	alta sculptum (111).JPG	fold2	5	*Amblyomma*
8750	High resolution	ResNet-50	alta sculptum (115).JPG	fold2	5	*Amblyomma*
8751	High resolution	ResNet-50	alta sculptum (12).JPG	fold2	5	*Amblyomma*
8752	High resolution	ResNet-50	alta sculptum (125).JPG	fold2	5	*Amblyomma*
8753	High resolution	ResNet-50	alta sculptum (126).JPG	fold2	5	*Amblyomma*
8754	High resolution	ResNet-50	alta sculptum (128).JPG	fold2	5	*Amblyomma*
8755	High resolution	ResNet-50	alta sculptum (132).JPG	fold2	5	*Amblyomma*
8756	High resolution	ResNet-50	alta sculptum (138).JPG	fold2	5	*Amblyomma*
8757	High resolution	ResNet-50	alta sculptum (27).JPG	fold2	5	*Amblyomma*
8758	High resolution	ResNet-50	alta sculptum (32).JPG	fold2	5	*Amblyomma*
8759	High resolution	ResNet-50	alta sculptum (33).JPG	fold2	5	*Amblyomma*
8760	High resolution	ResNet-50	alta sculptum (38).JPG	fold2	5	*Amblyomma*
8761	High resolution	ResNet-50	alta sculptum (39).JPG	fold2	5	*Amblyomma*
8762	High resolution	ResNet-50	alta sculptum (40).JPG	fold2	5	*Amblyomma*
8763	High resolution	ResNet-50	alta sculptum (41).JPG	fold2	5	*Amblyomma*
8764	High resolution	ResNet-50	alta sculptum (43).JPG	fold2	5	*Amblyomma*
8765	High resolution	ResNet-50	alta sculptum (47).JPG	fold2	5	*Amblyomma*
8766	High resolution	ResNet-50	alta sculptum (52).JPG	fold2	5	*Amblyomma*
8767	High resolution	ResNet-50	alta sculptum (56).jpg	fold2	5	*Amblyomma*
8768	High resolution	ResNet-50	alta sculptum (67).JPG	fold2	5	*Amblyomma*
8769	High resolution	ResNet-50	alta sculptum (74).JPG	fold2	5	*Amblyomma*
8770	High resolution	ResNet-50	alta sculptum (8).jpg	fold2	5	*Amblyomma*
8771	High resolution	ResNet-50	alta sculptum (81).JPG	fold2	5	*Amblyomma*
8772	High resolution	ResNet-50	alta sculptum (9).JPG	fold2	5	*Amblyomma*
8773	High resolution	ResNet-50	alta sculptum (91).JPG	fold2	5	*Amblyomma*
8774	High resolution	ResNet-50	alta sculptum (95).JPG	fold2	5	*Amblyomma*
8775	High resolution	ResNet-50	alta sculptum (96).JPG	fold2	5	*Amblyomma*

8776	High resolution	ResNet-50	alta sculptum (99).JPG	fold2	5	*Amblyomma*
8777	High resolution	ResNet-50	alta triste (2).jpg	fold2	6	*Amblyomma*
8778	High resolution	ResNet-50	alta triste (25).jpg	fold2	6	*Amblyomma*
8779	High resolution	ResNet-50	alta triste (33).jpg	fold2	6	*Amblyomma*
8780	High resolution	ResNet-50	alta triste (36).jpg	fold2	6	*Amblyomma*
8781	High resolution	ResNet-50	alta triste (37).jpg	fold2	6	*Amblyomma*
8782	High resolution	ResNet-50	alta triste (38).jpg	fold2	6	*Amblyomma*
8783	High resolution	ResNet-50	alta triste (39).jpg	fold2	6	*Amblyomma*
8784	High resolution	ResNet-50	alta triste (41).jpg	fold2	6	*Amblyomma*
8785	High resolution	ResNet-50	alta triste (47).jpg	fold2	6	*Amblyomma*
8786	High resolution	ResNet-50	alta triste (5).jpg	fold2	6	*Amblyomma*
8787	High resolution	ResNet-50	alta triste (54).jpg	fold2	6	*Amblyomma*
8788	High resolution	ResNet-50	alta triste (61).jpg	fold2	6	*Amblyomma*
8789	High resolution	ResNet-50	alta triste (63).jpg	fold2	6	*Amblyomma*
8790	High resolution	ResNet-50	alta triste (65).jpg	fold2	6	*Amblyomma*
8791	High resolution	ResNet-50	alta triste (67).jpg	fold2	6	*Amblyomma*
8792	High resolution	ResNet-50	alta triste (77).jpg	fold2	6	*Amblyomma*
8793	High resolution	ResNet-50	alta aureolatum (1).jpg	fold3	1	*Amblyomma*
8794	High resolution	ResNet-50	alta aureolatum (14).jpg	fold3	1	*Amblyomma*
8795	High resolution	ResNet-50	alta aureolatum (19).jpg	fold3	1	*Amblyomma*
8796	High resolution	ResNet-50	alta aureolatum (24).jpg	fold3	1	*Amblyomma*
8797	High resolution	ResNet-50	alta aureolatum (31).jpg	fold3	1	*Amblyomma*
8798	High resolution	ResNet-50	alta aureolatum (33).jpg	fold3	1	*Amblyomma*
8799	High resolution	ResNet-50	alta aureolatum (35).jpg	fold3	1	*Amblyomma*
8800	High resolution	ResNet-50	alta aureolatum (36).jpg	fold3	1	*Amblyomma*
8801	High resolution	ResNet-50	alta aureolatum (40).jpg	fold3	1	*Amblyomma*
8802	High resolution	ResNet-50	alta aureolatum (53).jpg	fold3	1	*Amblyomma*
8803	High resolution	ResNet-50	alta aureolatum (54).jpg	fold3	1	*Amblyomma*
8804	High resolution	ResNet-50	alta aureolatum (66).jpg	fold3	1	*Amblyomma*
8805	High resolution	ResNet-50	alta aureolatum (67).jpg	fold3	1	*Amblyomma*
8806	High resolution	ResNet-50	alta cajennense (1).jpg	fold3	2	*Amblyomma*
8807	High resolution	ResNet-50	alta cajennense (13).jpg	fold3	2	*Amblyomma*
8808	High resolution	ResNet-50	alta cajennense (14).jpg	fold3	2	*Amblyomma*
8809	High resolution	ResNet-50	alta cajennense (17).jpg	fold3	2	*Amblyomma*
8810	High resolution	ResNet-50	alta cajennense (18).jpg	fold3	2	*Amblyomma*
8811	High resolution	ResNet-50	alta cajennense (2).jpg	fold3	2	*Amblyomma*
8812	High resolution	ResNet-50	alta cajennense (23).jpg	fold3	2	*Amblyomma*
8813	High resolution	ResNet-50	alta cajennense (26).jpg	fold3	2	*Amblyomma*
8814	High resolution	ResNet-50	alta cajennense (34).jpg	fold3	2	*Amblyomma*
8815	High resolution	ResNet-50	alta cajennense (36).jpg	fold3	2	*Amblyomma*
8816	High resolution	ResNet-50	alta cajennense (37).jpg	fold3	2	*Amblyomma*
8817	High resolution	ResNet-50	alta cajennense (38).jpg	fold3	2	*Amblyomma*
8818	High resolution	ResNet-50	alta cajennense (50).jpg	fold3	2	*Amblyomma*
8819	High resolution	ResNet-50	alta cajennense (63).jpg	fold3	2	*Amblyomma*
8820	High resolution	ResNet-50	alta cajennense (64).jpg	fold3	2	*Amblyomma*
8821	High resolution	ResNet-50	alta cajennense (67).jpg	fold3	2	*Amblyomma*
8822	High resolution	ResNet-50	alta cajennense (9).jpg	fold3	2	*Amblyomma*
8823	High resolution	ResNet-50	alta dubitatum (1).jpg	fold3	3	*Amblyomma*
8824	High resolution	ResNet-50	alta dubitatum (10).JPG	fold3	3	*Amblyomma*
8825	High resolution	ResNet-50	alta dubitatum (15).jpg	fold3	3	*Amblyomma*
8826	High resolution	ResNet-50	alta dubitatum (31).jpg	fold3	3	*Amblyomma*
8827	High resolution	ResNet-50	alta dubitatum (39).jpg	fold3	3	*Amblyomma*
8828	High resolution	ResNet-50	alta dubitatum (4).jpg	fold3	3	*Amblyomma*
8829	High resolution	ResNet-50	alta dubitatum (42).jpg	fold3	3	*Amblyomma*
8830	High resolution	ResNet-50	alta dubitatum (45).jpg	fold3	3	*Amblyomma*
8831	High resolution	ResNet-50	alta dubitatum (46).jpg	fold3	3	*Amblyomma*
8832	High resolution	ResNet-50	alta dubitatum (53).jpg	fold3	3	*Amblyomma*
8833	High resolution	ResNet-50	alta dubitatum (6).jpg	fold3	3	*Amblyomma*
8834	High resolution	ResNet-50	alta dubitatum (62).jpg	fold3	3	*Amblyomma*
8835	High resolution	ResNet-50	alta dubitatum (63).jpg	fold3	3	*Amblyomma*
8836	High resolution	ResNet-50	alta ovale (15).jpg	fold3	4	*Amblyomma*
8837	High resolution	ResNet-50	alta ovale (17).jpg	fold3	4	*Amblyomma*
8838	High resolution	ResNet-50	alta ovale (22).jpg	fold3	4	*Amblyomma*
8839	High resolution	ResNet-50	alta ovale (30).jpg	fold3	4	*Amblyomma*
8840	High resolution	ResNet-50	alta ovale (32).jpg	fold3	4	*Amblyomma*
8841	High resolution	ResNet-50	alta ovale (33).jpg	fold3	4	*Amblyomma*
8842	High resolution	ResNet-50	alta ovale (34).jpg	fold3	4	*Amblyomma*

8843	High resolution	ResNet-50	alta ovale (45).jpg	fold3	4	*Amblyomma*
8844	High resolution	ResNet-50	alta ovale (46).jpg	fold3	4	*Amblyomma*
8845	High resolution	ResNet-50	alta ovale (53).jpg	fold3	4	*Amblyomma*
8846	High resolution	ResNet-50	alta ovale (56).jpg	fold3	4	*Amblyomma*
8847	High resolution	ResNet-50	alta ovale (57).JPG	fold3	4	*Amblyomma*
8848	High resolution	ResNet-50	alta ovale (60).jpg	fold3	4	*Amblyomma*
8849	High resolution	ResNet-50	alta ovale (64).jpg	fold3	4	*Amblyomma*
8850	High resolution	ResNet-50	alta sculptum (10).JPG	fold3	5	*Amblyomma*
8851	High resolution	ResNet-50	alta sculptum (101).JPG	fold3	5	*Amblyomma*
8852	High resolution	ResNet-50	alta sculptum (104).JPG	fold3	5	*Amblyomma*
8853	High resolution	ResNet-50	alta sculptum (118).JPG	fold3	5	*Amblyomma*
8854	High resolution	ResNet-50	alta sculptum (119).JPG	fold3	5	*Amblyomma*
8855	High resolution	ResNet-50	alta sculptum (14).JPG	fold3	5	*Amblyomma*
8856	High resolution	ResNet-50	alta sculptum (15).JPG	fold3	5	*Amblyomma*
8857	High resolution	ResNet-50	alta sculptum (17).JPG	fold3	5	*Amblyomma*
8858	High resolution	ResNet-50	alta sculptum (18).JPG	fold3	5	*Amblyomma*
8859	High resolution	ResNet-50	alta sculptum (25).JPG	fold3	5	*Amblyomma*
8860	High resolution	ResNet-50	alta sculptum (31).JPG	fold3	5	*Amblyomma*
8861	High resolution	ResNet-50	alta sculptum (37).JPG	fold3	5	*Amblyomma*
8862	High resolution	ResNet-50	alta sculptum (42).JPG	fold3	5	*Amblyomma*
8863	High resolution	ResNet-50	alta sculptum (46).JPG	fold3	5	*Amblyomma*
8864	High resolution	ResNet-50	alta sculptum (48).JPG	fold3	5	*Amblyomma*
8865	High resolution	ResNet-50	alta sculptum (53).JPG	fold3	5	*Amblyomma*
8866	High resolution	ResNet-50	alta sculptum (60).jpg	fold3	5	*Amblyomma*
8867	High resolution	ResNet-50	alta sculptum (62).jpg	fold3	5	*Amblyomma*
8868	High resolution	ResNet-50	alta sculptum (63).jpg	fold3	5	*Amblyomma*
8869	High resolution	ResNet-50	alta sculptum (64).jpg	fold3	5	*Amblyomma*
8870	High resolution	ResNet-50	alta sculptum (69).JPG	fold3	5	*Amblyomma*
8871	High resolution	ResNet-50	alta sculptum (71).JPG	fold3	5	*Amblyomma*
8872	High resolution	ResNet-50	alta sculptum (76).JPG	fold3	5	*Amblyomma*
8873	High resolution	ResNet-50	alta sculptum (88).JPG	fold3	5	*Amblyomma*
8874	High resolution	ResNet-50	alta sculptum (90).JPG	fold3	5	*Amblyomma*
8875	High resolution	ResNet-50	alta sculptum (92).JPG	fold3	5	*Amblyomma*
8876	High resolution	ResNet-50	alta sculptum (93).JPG	fold3	5	*Amblyomma*
8877	High resolution	ResNet-50	alta sculptum (97).JPG	fold3	5	*Amblyomma*
8878	High resolution	ResNet-50	alta triste (10).jpg	fold3	6	*Amblyomma*
8879	High resolution	ResNet-50	alta triste (18).jpg	fold3	6	*Amblyomma*
8880	High resolution	ResNet-50	alta triste (19).jpg	fold3	6	*Amblyomma*
8881	High resolution	ResNet-50	alta triste (21).jpg	fold3	6	*Amblyomma*
8882	High resolution	ResNet-50	alta triste (24).jpg	fold3	6	*Amblyomma*
8883	High resolution	ResNet-50	alta triste (3).jpg	fold3	6	*Amblyomma*
8884	High resolution	ResNet-50	alta triste (31).jpg	fold3	6	*Amblyomma*
8885	High resolution	ResNet-50	alta triste (40).jpg	fold3	6	*Amblyomma*
8886	High resolution	ResNet-50	alta triste (43).jpg	fold3	6	*Amblyomma*
8887	High resolution	ResNet-50	alta triste (6).jpg	fold3	6	*Amblyomma*
8888	High resolution	ResNet-50	alta triste (7).jpg	fold3	6	*Amblyomma*
8889	High resolution	ResNet-50	alta triste (70).jpg	fold3	6	*Amblyomma*
8890	High resolution	ResNet-50	alta triste (71).jpg	fold3	6	*Amblyomma*
8891	High resolution	ResNet-50	alta triste (72).jpg	fold3	6	*Amblyomma*
8892	High resolution	ResNet-50	alta triste (9).jpg	fold3	6	*Amblyomma*
8893	High resolution	ResNet-50	alta aureolatum (12).jpg	fold4	1	*Amblyomma*
8894	High resolution	ResNet-50	alta aureolatum (13).jpg	fold4	1	*Amblyomma*
8895	High resolution	ResNet-50	alta aureolatum (15).jpg	fold4	1	*Amblyomma*
8896	High resolution	ResNet-50	alta aureolatum (16).jpg	fold4	1	*Amblyomma*
8897	High resolution	ResNet-50	alta aureolatum (20).jpg	fold4	1	*Amblyomma*
8898	High resolution	ResNet-50	alta aureolatum (22).jpg	fold4	1	*Amblyomma*
8899	High resolution	ResNet-50	alta aureolatum (25).jpg	fold4	1	*Amblyomma*
8900	High resolution	ResNet-50	alta aureolatum (26).jpg	fold4	1	*Amblyomma*
8901	High resolution	ResNet-50	alta aureolatum (37).jpg	fold4	1	*Amblyomma*
8902	High resolution	ResNet-50	alta aureolatum (4).jpg	fold4	1	*Amblyomma*
8903	High resolution	ResNet-50	alta aureolatum (56).jpg	fold4	1	*Amblyomma*
8904	High resolution	ResNet-50	alta aureolatum (63).jpg	fold4	1	*Amblyomma*
8905	High resolution	ResNet-50	alta aureolatum (64).jpg	fold4	1	*Amblyomma*
8906	High resolution	ResNet-50	alta aureolatum (7).jpg	fold4	1	*Amblyomma*
8907	High resolution	ResNet-50	alta cajennense (20).jpg	fold4	2	*Amblyomma*
8908	High resolution	ResNet-50	alta cajennense (21).jpg	fold4	2	*Amblyomma*
8909	High resolution	ResNet-50	alta cajennense (25).jpg	fold4	2	*Amblyomma*

8910	High resolution	ResNet-50	alta cajennense (28).jpg	fold4	2	*Amblyomma*
8911	High resolution	ResNet-50	alta cajennense (4).jpg	fold4	2	*Amblyomma*
8912	High resolution	ResNet-50	alta cajennense (46).jpg	fold4	2	*Amblyomma*
8913	High resolution	ResNet-50	alta cajennense (48).jpg	fold4	2	*Amblyomma*
8914	High resolution	ResNet-50	alta cajennense (5).jpg	fold4	2	*Amblyomma*
8915	High resolution	ResNet-50	alta cajennense (53).jpg	fold4	2	*Amblyomma*
8916	High resolution	ResNet-50	alta cajennense (60).jpg	fold4	2	*Amblyomma*
8917	High resolution	ResNet-50	alta cajennense (61).jpg	fold4	2	*Amblyomma*
8918	High resolution	ResNet-50	alta cajennense (65).jpg	fold4	2	*Amblyomma*
8919	High resolution	ResNet-50	alta cajennense (70).jpg	fold4	2	*Amblyomma*
8920	High resolution	ResNet-50	alta cajennense (72).jpg	fold4	2	*Amblyomma*
8921	High resolution	ResNet-50	alta cajennense (76).jpg	fold4	2	*Amblyomma*
8922	High resolution	ResNet-50	alta cajennense (83).jpg	fold4	2	*Amblyomma*
8923	High resolution	ResNet-50	alta dubitatum (12).jpg	fold4	3	*Amblyomma*
8924	High resolution	ResNet-50	alta dubitatum (16).jpg	fold4	3	*Amblyomma*
8925	High resolution	ResNet-50	alta dubitatum (17).jpg	fold4	3	*Amblyomma*
8926	High resolution	ResNet-50	alta dubitatum (18).jpg	fold4	3	*Amblyomma*
8927	High resolution	ResNet-50	alta dubitatum (25).jpg	fold4	3	*Amblyomma*
8928	High resolution	ResNet-50	alta dubitatum (27).jpg	fold4	3	*Amblyomma*
8929	High resolution	ResNet-50	alta dubitatum (30).jpg	fold4	3	*Amblyomma*
8930	High resolution	ResNet-50	alta dubitatum (5).jpg	fold4	3	*Amblyomma*
8931	High resolution	ResNet-50	alta dubitatum (54).jpg	fold4	3	*Amblyomma*
8932	High resolution	ResNet-50	alta dubitatum (55).jpg	fold4	3	*Amblyomma*
8933	High resolution	ResNet-50	alta dubitatum (56).jpg	fold4	3	*Amblyomma*
8934	High resolution	ResNet-50	alta dubitatum (61).jpg	fold4	3	*Amblyomma*
8935	High resolution	ResNet-50	alta dubitatum (65).jpg	fold4	3	*Amblyomma*
8936	High resolution	ResNet-50	alta ovale (1).jpg	fold4	4	*Amblyomma*
8937	High resolution	ResNet-50	alta ovale (10).jpg	fold4	4	*Amblyomma*
8938	High resolution	ResNet-50	alta ovale (11).jpg	fold4	4	*Amblyomma*
8939	High resolution	ResNet-50	alta ovale (14).jpg	fold4	4	*Amblyomma*
8940	High resolution	ResNet-50	alta ovale (20).jpg	fold4	4	*Amblyomma*
8941	High resolution	ResNet-50	alta ovale (21).jpg	fold4	4	*Amblyomma*
8942	High resolution	ResNet-50	alta ovale (29).jpg	fold4	4	*Amblyomma*
8943	High resolution	ResNet-50	alta ovale (3).jpg	fold4	4	*Amblyomma*
8944	High resolution	ResNet-50	alta ovale (36).jpg	fold4	4	*Amblyomma*
8945	High resolution	ResNet-50	alta ovale (37).jpg	fold4	4	*Amblyomma*
8946	High resolution	ResNet-50	alta ovale (47).jpg	fold4	4	*Amblyomma*
8947	High resolution	ResNet-50	alta ovale (6).jpg	fold4	4	*Amblyomma*
8948	High resolution	ResNet-50	alta ovale (7).jpg	fold4	4	*Amblyomma*
8949	High resolution	ResNet-50	alta sculptum (102).JPG	fold4	5	*Amblyomma*
8950	High resolution	ResNet-50	alta sculptum (106).JPG	fold4	5	*Amblyomma*
8951	High resolution	ResNet-50	alta sculptum (107).JPG	fold4	5	*Amblyomma*
8952	High resolution	ResNet-50	alta sculptum (116).JPG	fold4	5	*Amblyomma*
8953	High resolution	ResNet-50	alta sculptum (117).JPG	fold4	5	*Amblyomma*
8954	High resolution	ResNet-50	alta sculptum (120).JPG	fold4	5	*Amblyomma*
8955	High resolution	ResNet-50	alta sculptum (127).JPG	fold4	5	*Amblyomma*
8956	High resolution	ResNet-50	alta sculptum (133).JPG	fold4	5	*Amblyomma*
8957	High resolution	ResNet-50	alta sculptum (134).JPG	fold4	5	*Amblyomma*
8958	High resolution	ResNet-50	alta sculptum (136).JPG	fold4	5	*Amblyomma*
8959	High resolution	ResNet-50	alta sculptum (16).JPG	fold4	5	*Amblyomma*
8960	High resolution	ResNet-50	alta sculptum (23).JPG	fold4	5	*Amblyomma*
8961	High resolution	ResNet-50	alta sculptum (28).JPG	fold4	5	*Amblyomma*
8962	High resolution	ResNet-50	alta sculptum (34).JPG	fold4	5	*Amblyomma*
8963	High resolution	ResNet-50	alta sculptum (35).JPG	fold4	5	*Amblyomma*
8964	High resolution	ResNet-50	alta sculptum (4).jpg	fold4	5	*Amblyomma*
8965	High resolution	ResNet-50	alta sculptum (55).JPG	fold4	5	*Amblyomma*
8966	High resolution	ResNet-50	alta sculptum (59).jpg	fold4	5	*Amblyomma*
8967	High resolution	ResNet-50	alta sculptum (61).jpg	fold4	5	*Amblyomma*
8968	High resolution	ResNet-50	alta sculptum (68).JPG	fold4	5	*Amblyomma*
8969	High resolution	ResNet-50	alta sculptum (7).jpg	fold4	5	*Amblyomma*
8970	High resolution	ResNet-50	alta sculptum (73).JPG	fold4	5	*Amblyomma*
8971	High resolution	ResNet-50	alta sculptum (78).JPG	fold4	5	*Amblyomma*
8972	High resolution	ResNet-50	alta sculptum (82).JPG	fold4	5	*Amblyomma*
8973	High resolution	ResNet-50	alta sculptum (83).JPG	fold4	5	*Amblyomma*
8974	High resolution	ResNet-50	alta sculptum (84).JPG	fold4	5	*Amblyomma*
8975	High resolution	ResNet-50	alta sculptum (86).JPG	fold4	5	*Amblyomma*
8976	High resolution	ResNet-50	alta triste (11).jpg	fold4	6	*Amblyomma*

8977	High resolution	ResNet-50	alta triste (13).jpg	fold4	6	*Amblyomma*
8978	High resolution	ResNet-50	alta triste (17).jpg	fold4	6	*Amblyomma*
8979	High resolution	ResNet-50	alta triste (20).jpg	fold4	6	*Amblyomma*
8980	High resolution	ResNet-50	alta triste (23).jpg	fold4	6	*Amblyomma*
8981	High resolution	ResNet-50	alta triste (32).jpg	fold4	6	*Amblyomma*
8982	High resolution	ResNet-50	alta triste (34).jpg	fold4	6	*Amblyomma*
8983	High resolution	ResNet-50	alta triste (44).jpg	fold4	6	*Amblyomma*
8984	High resolution	ResNet-50	alta triste (45).jpg	fold4	6	*Amblyomma*
8985	High resolution	ResNet-50	alta triste (46).jpg	fold4	6	*Amblyomma*
8986	High resolution	ResNet-50	alta triste (55).jpg	fold4	6	*Amblyomma*
8987	High resolution	ResNet-50	alta triste (56).jpg	fold4	6	*Amblyomma*
8988	High resolution	ResNet-50	alta triste (62).jpg	fold4	6	*Amblyomma*
8989	High resolution	ResNet-50	alta triste (64).jpg	fold4	6	*Amblyomma*
8990	High resolution	ResNet-50	alta triste (66).jpg	fold4	6	*Amblyomma*
8991	High resolution	ResNet-50	alta triste (68).jpg	fold4	6	*Amblyomma*
8992	High resolution	ResNet-50	alta aureolatum (10).jpg	fold5	1	*Amblyomma*
8993	High resolution	ResNet-50	alta aureolatum (2).jpg	fold5	1	*Amblyomma*
8994	High resolution	ResNet-50	alta aureolatum (3).jpg	fold5	1	*Amblyomma*
8995	High resolution	ResNet-50	alta aureolatum (34).jpg	fold5	1	*Amblyomma*
8996	High resolution	ResNet-50	alta aureolatum (39).jpg	fold5	1	*Amblyomma*
8997	High resolution	ResNet-50	alta aureolatum (41).jpg	fold5	1	*Amblyomma*
8998	High resolution	ResNet-50	alta aureolatum (42).jpg	fold5	1	*Amblyomma*
8999	High resolution	ResNet-50	alta aureolatum (45).jpg	fold5	1	*Amblyomma*
9000	High resolution	ResNet-50	alta aureolatum (50).jpg	fold5	1	*Amblyomma*
9001	High resolution	ResNet-50	alta aureolatum (57).jpg	fold5	1	*Amblyomma*
9002	High resolution	ResNet-50	alta aureolatum (59).jpg	fold5	1	*Amblyomma*
9003	High resolution	ResNet-50	alta aureolatum (60).jpg	fold5	1	*Amblyomma*
9004	High resolution	ResNet-50	alta aureolatum (68).jpg	fold5	1	*Amblyomma*
9005	High resolution	ResNet-50	alta aureolatum (8).jpg	fold5	1	*Amblyomma*
9006	High resolution	ResNet-50	alta cajennense (10).jpg	fold5	2	*Amblyomma*
9007	High resolution	ResNet-50	alta cajennense (19).jpg	fold5	2	*Amblyomma*
9008	High resolution	ResNet-50	alta cajennense (22).jpg	fold5	2	*Amblyomma*
9009	High resolution	ResNet-50	alta cajennense (27).jpg	fold5	2	*Amblyomma*
9010	High resolution	ResNet-50	alta cajennense (32).jpg	fold5	2	*Amblyomma*
9011	High resolution	ResNet-50	alta cajennense (39).jpg	fold5	2	*Amblyomma*
9012	High resolution	ResNet-50	alta cajennense (44).jpg	fold5	2	*Amblyomma*
9013	High resolution	ResNet-50	alta cajennense (47).jpg	fold5	2	*Amblyomma*
9014	High resolution	ResNet-50	alta cajennense (54).jpg	fold5	2	*Amblyomma*
9015	High resolution	ResNet-50	alta cajennense (57).jpg	fold5	2	*Amblyomma*
9016	High resolution	ResNet-50	alta cajennense (69).jpg	fold5	2	*Amblyomma*
9017	High resolution	ResNet-50	alta cajennense (7).jpg	fold5	2	*Amblyomma*
9018	High resolution	ResNet-50	alta cajennense (75).jpg	fold5	2	*Amblyomma*
9019	High resolution	ResNet-50	alta cajennense (79).jpg	fold5	2	*Amblyomma*
9020	High resolution	ResNet-50	alta cajennense (8).jpg	fold5	2	*Amblyomma*
9021	High resolution	ResNet-50	alta cajennense (82).jpg	fold5	2	*Amblyomma*
9022	High resolution	ResNet-50	alta dubitatum (13).jpg	fold5	3	*Amblyomma*
9023	High resolution	ResNet-50	alta dubitatum (14).jpg	fold5	3	*Amblyomma*
9024	High resolution	ResNet-50	alta dubitatum (23).jpg	fold5	3	*Amblyomma*
9025	High resolution	ResNet-50	alta dubitatum (28).jpg	fold5	3	*Amblyomma*
9026	High resolution	ResNet-50	alta dubitatum (36).jpg	fold5	3	*Amblyomma*
9027	High resolution	ResNet-50	alta dubitatum (41).jpg	fold5	3	*Amblyomma*
9028	High resolution	ResNet-50	alta dubitatum (44).jpg	fold5	3	*Amblyomma*
9029	High resolution	ResNet-50	alta dubitatum (50).jpg	fold5	3	*Amblyomma*
9030	High resolution	ResNet-50	alta dubitatum (52).jpg	fold5	3	*Amblyomma*
9031	High resolution	ResNet-50	alta dubitatum (57).jpg	fold5	3	*Amblyomma*
9032	High resolution	ResNet-50	alta dubitatum (59).jpg	fold5	3	*Amblyomma*
9033	High resolution	ResNet-50	alta dubitatum (7).JPG	fold5	3	*Amblyomma*
9034	High resolution	ResNet-50	alta dubitatum (8).JPG	fold5	3	*Amblyomma*
9035	High resolution	ResNet-50	alta ovale (13).jpg	fold5	4	*Amblyomma*
9036	High resolution	ResNet-50	alta ovale (16).jpg	fold5	4	*Amblyomma*
9037	High resolution	ResNet-50	alta ovale (25).jpg	fold5	4	*Amblyomma*
9038	High resolution	ResNet-50	alta ovale (31).jpg	fold5	4	*Amblyomma*
9039	High resolution	ResNet-50	alta ovale (35).jpg	fold5	4	*Amblyomma*
9040	High resolution	ResNet-50	alta ovale (39).JPG	fold5	4	*Amblyomma*
9041	High resolution	ResNet-50	alta ovale (40).JPG	fold5	4	*Amblyomma*
9042	High resolution	ResNet-50	alta ovale (49).jpg	fold5	4	*Amblyomma*
9043	High resolution	ResNet-50	alta ovale (5).jpg	fold5	4	*Amblyomma*

9044	High resolution	ResNet-50	alta ovale (59).jpg	fold5	4	*Amblyomma*
9045	High resolution	ResNet-50	alta ovale (61).jpg	fold5	4	*Amblyomma*
9046	High resolution	ResNet-50	alta ovale (63).jpg	fold5	4	*Amblyomma*
9047	High resolution	ResNet-50	alta ovale (8).jpg	fold5	4	*Amblyomma*
9048	High resolution	ResNet-50	alta sculptum (1).jpg	fold5	5	*Amblyomma*
9049	High resolution	ResNet-50	alta sculptum (103).JPG	fold5	5	*Amblyomma*
9050	High resolution	ResNet-50	alta sculptum (105).JPG	fold5	5	*Amblyomma*
9051	High resolution	ResNet-50	alta sculptum (108).JPG	fold5	5	*Amblyomma*
9052	High resolution	ResNet-50	alta sculptum (112).JPG	fold5	5	*Amblyomma*
9053	High resolution	ResNet-50	alta sculptum (113).JPG	fold5	5	*Amblyomma*
9054	High resolution	ResNet-50	alta sculptum (122).JPG	fold5	5	*Amblyomma*
9055	High resolution	ResNet-50	alta sculptum (129).JPG	fold5	5	*Amblyomma*
9056	High resolution	ResNet-50	alta sculptum (13).JPG	fold5	5	*Amblyomma*
9057	High resolution	ResNet-50	alta sculptum (131).JPG	fold5	5	*Amblyomma*
9058	High resolution	ResNet-50	alta sculptum (135).JPG	fold5	5	*Amblyomma*
9059	High resolution	ResNet-50	alta sculptum (137).JPG	fold5	5	*Amblyomma*
9060	High resolution	ResNet-50	alta sculptum (20).JPG	fold5	5	*Amblyomma*
9061	High resolution	ResNet-50	alta sculptum (24).JPG	fold5	5	*Amblyomma*
9062	High resolution	ResNet-50	alta sculptum (29).JPG	fold5	5	*Amblyomma*
9063	High resolution	ResNet-50	alta sculptum (30).JPG	fold5	5	*Amblyomma*
9064	High resolution	ResNet-50	alta sculptum (44).JPG	fold5	5	*Amblyomma*
9065	High resolution	ResNet-50	alta sculptum (45).JPG	fold5	5	*Amblyomma*
9066	High resolution	ResNet-50	alta sculptum (5).jpg	fold5	5	*Amblyomma*
9067	High resolution	ResNet-50	alta sculptum (50).JPG	fold5	5	*Amblyomma*
9068	High resolution	ResNet-50	alta sculptum (51).JPG	fold5	5	*Amblyomma*
9069	High resolution	ResNet-50	alta sculptum (70).JPG	fold5	5	*Amblyomma*
9070	High resolution	ResNet-50	alta sculptum (72).JPG	fold5	5	*Amblyomma*
9071	High resolution	ResNet-50	alta sculptum (77).JPG	fold5	5	*Amblyomma*
9072	High resolution	ResNet-50	alta sculptum (85).JPG	fold5	5	*Amblyomma*
9073	High resolution	ResNet-50	alta sculptum (87).JPG	fold5	5	*Amblyomma*
9074	High resolution	ResNet-50	alta sculptum (89).JPG	fold5	5	*Amblyomma*
9075	High resolution	ResNet-50	alta sculptum (98).JPG	fold5	5	*Amblyomma*
9076	High resolution	ResNet-50	alta triste (12).jpg	fold5	6	*Amblyomma*
9077	High resolution	ResNet-50	alta triste (15).jpg	fold5	6	*Amblyomma*
9078	High resolution	ResNet-50	alta triste (22).jpg	fold5	6	*Amblyomma*
9079	High resolution	ResNet-50	alta triste (26).jpg	fold5	6	*Amblyomma*
9080	High resolution	ResNet-50	alta triste (28).jpg	fold5	6	*Amblyomma*
9081	High resolution	ResNet-50	alta triste (30).jpg	fold5	6	*Amblyomma*
9082	High resolution	ResNet-50	alta triste (35).jpg	fold5	6	*Amblyomma*
9083	High resolution	ResNet-50	alta triste (42).jpg	fold5	6	*Amblyomma*
9084	High resolution	ResNet-50	alta triste (49).jpg	fold5	6	*Amblyomma*
9085	High resolution	ResNet-50	alta triste (50).jpg	fold5	6	*Amblyomma*
9086	High resolution	ResNet-50	alta triste (53).jpg	fold5	6	*Amblyomma*
9087	High resolution	ResNet-50	alta triste (58).jpg	fold5	6	*Amblyomma*
9088	High resolution	ResNet-50	alta triste (59).jpg	fold5	6	*Amblyomma*
9089	High resolution	ResNet-50	alta triste (73).jpg	fold5	6	*Amblyomma*
9090	High resolution	ResNet-50	alta triste (74).jpg	fold5	6	*Amblyomma*
9091	High resolution	ResNet-50	alta triste (75).jpg	fold5	6	*Amblyomma*
9092	All together	ResNet-50	todos aureolatum (100).jpg	fold1	1	*Amblyomma*
9093	All together	ResNet-50	todos aureolatum (106).jpg	fold1	1	*Amblyomma*
9094	All together	ResNet-50	todos aureolatum (21).jpg	fold1	1	*Amblyomma*
9095	All together	ResNet-50	todos aureolatum (26).jpg	fold1	1	*Amblyomma*
9096	All together	ResNet-50	todos aureolatum (27).jpg	fold1	1	*Amblyomma*
9097	All together	ResNet-50	todos aureolatum (28).jpg	fold1	1	*Amblyomma*
9098	All together	ResNet-50	todos aureolatum (35).jpg	fold1	1	*Amblyomma*
9099	All together	ResNet-50	todos aureolatum (38).jpg	fold1	1	*Amblyomma*
9100	All together	ResNet-50	todos aureolatum (41).jpg	fold1	1	*Amblyomma*
9101	All together	ResNet-50	todos aureolatum (45).jpg	fold1	1	*Amblyomma*
9102	All together	ResNet-50	todos aureolatum (56).jpg	fold1	1	*Amblyomma*
9103	All together	ResNet-50	todos aureolatum (57).jpg	fold1	1	*Amblyomma*
9104	All together	ResNet-50	todos aureolatum (6).jpg	fold1	1	*Amblyomma*
9105	All together	ResNet-50	todos aureolatum (60).jpg	fold1	1	*Amblyomma*
9106	All together	ResNet-50	todos aureolatum (66).jpg	fold1	1	*Amblyomma*
9107	All together	ResNet-50	todos aureolatum (69).jpg	fold1	1	*Amblyomma*
9108	All together	ResNet-50	todos aureolatum (72).jpg	fold1	1	*Amblyomma*
9109	All together	ResNet-50	todos aureolatum (73).jpg	fold1	1	*Amblyomma*
9110	All together	ResNet-50	todos aureolatum (79).jpg	fold1	1	*Amblyomma*

9111	All together	ResNet-50	todos aureolatum (80).jpg	fold1	1	*Amblyomma*
9112	All together	ResNet-50	todos aureolatum (82).jpg	fold1	1	*Amblyomma*
9113	All together	ResNet-50	todos aureolatum (85).jpg	fold1	1	*Amblyomma*
9114	All together	ResNet-50	todos aureolatum (90).jpg	fold1	1	*Amblyomma*
9115	All together	ResNet-50	todos aureolatum (93).jpg	fold1	1	*Amblyomma*
9116	All together	ResNet-50	todos cajennense (10).jpg	fold1	2	*Amblyomma*
9117	All together	ResNet-50	todos cajennense (101).jpg	fold1	2	*Amblyomma*
9118	All together	ResNet-50	todos cajennense (102).jpg	fold1	2	*Amblyomma*
9119	All together	ResNet-50	todos cajennense (106).jpg	fold1	2	*Amblyomma*
9120	All together	ResNet-50	todos cajennense (111).jpg	fold1	2	*Amblyomma*
9121	All together	ResNet-50	todos cajennense (113).jpg	fold1	2	*Amblyomma*
9122	All together	ResNet-50	todos cajennense (114).jpg	fold1	2	*Amblyomma*
9123	All together	ResNet-50	todos cajennense (117).jpg	fold1	2	*Amblyomma*
9124	All together	ResNet-50	todos cajennense (124).jpg	fold1	2	*Amblyomma*
9125	All together	ResNet-50	todos cajennense (129).jpg	fold1	2	*Amblyomma*
9126	All together	ResNet-50	todos cajennense (13).jpg	fold1	2	*Amblyomma*
9127	All together	ResNet-50	todos cajennense (133).jpg	fold1	2	*Amblyomma*
9128	All together	ResNet-50	todos cajennense (137).jpg	fold1	2	*Amblyomma*
9129	All together	ResNet-50	todos cajennense (138).jpg	fold1	2	*Amblyomma*
9130	All together	ResNet-50	todos cajennense (139).jpg	fold1	2	*Amblyomma*
9131	All together	ResNet-50	todos cajennense (146).jpg	fold1	2	*Amblyomma*
9132	All together	ResNet-50	todos cajennense (148).jpg	fold1	2	*Amblyomma*
9133	All together	ResNet-50	todos cajennense (151).jpg	fold1	2	*Amblyomma*
9134	All together	ResNet-50	todos cajennense (26).jpg	fold1	2	*Amblyomma*
9135	All together	ResNet-50	todos cajennense (28).jpg	fold1	2	*Amblyomma*
9136	All together	ResNet-50	todos cajennense (33).jpg	fold1	2	*Amblyomma*
9137	All together	ResNet-50	todos cajennense (37).jpg	fold1	2	*Amblyomma*
9138	All together	ResNet-50	todos cajennense (44).jpg	fold1	2	*Amblyomma*
9139	All together	ResNet-50	todos cajennense (49).jpg	fold1	2	*Amblyomma*
9140	All together	ResNet-50	todos cajennense (51).jpg	fold1	2	*Amblyomma*
9141	All together	ResNet-50	todos cajennense (67).jpg	fold1	2	*Amblyomma*
9142	All together	ResNet-50	todos cajennense (70).jpg	fold1	2	*Amblyomma*
9143	All together	ResNet-50	todos cajennense (74).jpg	fold1	2	*Amblyomma*
9144	All together	ResNet-50	todos cajennense (76).jpg	fold1	2	*Amblyomma*
9145	All together	ResNet-50	todos cajennense (85).jpg	fold1	2	*Amblyomma*
9146	All together	ResNet-50	todos cajennense (94).jpg	fold1	2	*Amblyomma*
9147	All together	ResNet-50	todos cajennense (97).jpg	fold1	2	*Amblyomma*
9148	All together	ResNet-50	todos cajennense (99).jpg	fold1	2	*Amblyomma*
9149	All together	ResNet-50	todos dubitatum (103).jpg	fold1	3	*Amblyomma*
9150	All together	ResNet-50	todos dubitatum (104).jpg	fold1	3	*Amblyomma*
9151	All together	ResNet-50	todos dubitatum (109).jpg	fold1	3	*Amblyomma*
9152	All together	ResNet-50	todos dubitatum (12).JPG	fold1	3	*Amblyomma*
9153	All together	ResNet-50	todos dubitatum (15).jpg	fold1	3	*Amblyomma*
9154	All together	ResNet-50	todos dubitatum (2).jpg	fold1	3	*Amblyomma*
9155	All together	ResNet-50	todos dubitatum (20).jpg	fold1	3	*Amblyomma*
9156	All together	ResNet-50	todos dubitatum (3).jpg	fold1	3	*Amblyomma*
9157	All together	ResNet-50	todos dubitatum (36).jpg	fold1	3	*Amblyomma*
9158	All together	ResNet-50	todos dubitatum (37).jpg	fold1	3	*Amblyomma*
9159	All together	ResNet-50	todos dubitatum (39).jpg	fold1	3	*Amblyomma*
9160	All together	ResNet-50	todos dubitatum (42).jpg	fold1	3	*Amblyomma*
9161	All together	ResNet-50	todos dubitatum (44).jpg	fold1	3	*Amblyomma*
9162	All together	ResNet-50	todos dubitatum (52).jpg	fold1	3	*Amblyomma*
9163	All together	ResNet-50	todos dubitatum (56).jpg	fold1	3	*Amblyomma*
9164	All together	ResNet-50	todos dubitatum (60).jpg	fold1	3	*Amblyomma*
9165	All together	ResNet-50	todos dubitatum (62).jpg	fold1	3	*Amblyomma*
9166	All together	ResNet-50	todos dubitatum (76).jpg	fold1	3	*Amblyomma*
9167	All together	ResNet-50	todos dubitatum (77).jpg	fold1	3	*Amblyomma*
9168	All together	ResNet-50	todos dubitatum (81).jpg	fold1	3	*Amblyomma*
9169	All together	ResNet-50	todos dubitatum (82).jpg	fold1	3	*Amblyomma*
9170	All together	ResNet-50	todos dubitatum (83).jpg	fold1	3	*Amblyomma*
9171	All together	ResNet-50	todos ovale (100).jpg	fold1	4	*Amblyomma*
9172	All together	ResNet-50	todos ovale (104).jpg	fold1	4	*Amblyomma*
9173	All together	ResNet-50	todos ovale (106).JPG	fold1	4	*Amblyomma*
9174	All together	ResNet-50	todos ovale (108).jpg	fold1	4	*Amblyomma*
9175	All together	ResNet-50	todos ovale (109).jpg	fold1	4	*Amblyomma*
9176	All together	ResNet-50	todos ovale (116).jpg	fold1	4	*Amblyomma*
9177	All together	ResNet-50	todos ovale (118).jpg	fold1	4	*Amblyomma*

9178	All together	ResNet-50	todos ovale (121).jpg	fold1	4	*Amblyomma*
9179	All together	ResNet-50	todos ovale (16).jpg	fold1	4	*Amblyomma*
9180	All together	ResNet-50	todos ovale (20).jpg	fold1	4	*Amblyomma*
9181	All together	ResNet-50	todos ovale (24).jpg	fold1	4	*Amblyomma*
9182	All together	ResNet-50	todos ovale (27).jpg	fold1	4	*Amblyomma*
9183	All together	ResNet-50	todos ovale (34).jpg	fold1	4	*Amblyomma*
9184	All together	ResNet-50	todos ovale (38).jpg	fold1	4	*Amblyomma*
9185	All together	ResNet-50	todos ovale (4).jpg	fold1	4	*Amblyomma*
9186	All together	ResNet-50	todos ovale (42).jpg	fold1	4	*Amblyomma*
9187	All together	ResNet-50	todos ovale (47).jpg	fold1	4	*Amblyomma*
9188	All together	ResNet-50	todos ovale (60).jpg	fold1	4	*Amblyomma*
9189	All together	ResNet-50	todos ovale (62).jpg	fold1	4	*Amblyomma*
9190	All together	ResNet-50	todos ovale (65).jpg	fold1	4	*Amblyomma*
9191	All together	ResNet-50	todos ovale (7).jpg	fold1	4	*Amblyomma*
9192	All together	ResNet-50	todos ovale (70).jpg	fold1	4	*Amblyomma*
9193	All together	ResNet-50	todos ovale (85).jpg	fold1	4	*Amblyomma*
9194	All together	ResNet-50	todos ovale (87).jpg	fold1	4	*Amblyomma*
9195	All together	ResNet-50	todos sculptum (100).JPG	fold1	5	*Amblyomma*
9196	All together	ResNet-50	todos sculptum (103).JPG	fold1	5	*Amblyomma*
9197	All together	ResNet-50	todos sculptum (106).JPG	fold1	5	*Amblyomma*
9198	All together	ResNet-50	todos sculptum (108).JPG	fold1	5	*Amblyomma*
9199	All together	ResNet-50	todos sculptum (112).JPG	fold1	5	*Amblyomma*
9200	All together	ResNet-50	todos sculptum (113).JPG	fold1	5	*Amblyomma*
9201	All together	ResNet-50	todos sculptum (114).JPG	fold1	5	*Amblyomma*
9202	All together	ResNet-50	todos sculptum (115).JPG	fold1	5	*Amblyomma*
9203	All together	ResNet-50	todos sculptum (119).JPG	fold1	5	*Amblyomma*
9204	All together	ResNet-50	todos sculptum (120).JPG	fold1	5	*Amblyomma*
9205	All together	ResNet-50	todos sculptum (123).JPG	fold1	5	*Amblyomma*
9206	All together	ResNet-50	todos sculptum (124).JPG	fold1	5	*Amblyomma*
9207	All together	ResNet-50	todos sculptum (127).JPG	fold1	5	*Amblyomma*
9208	All together	ResNet-50	todos sculptum (138).JPG	fold1	5	*Amblyomma*
9209	All together	ResNet-50	todos sculptum (139).JPG	fold1	5	*Amblyomma*
9210	All together	ResNet-50	todos sculptum (142).JPG	fold1	5	*Amblyomma*
9211	All together	ResNet-50	todos sculptum (26).JPG	fold1	5	*Amblyomma*
9212	All together	ResNet-50	todos sculptum (32).JPG	fold1	5	*Amblyomma*
9213	All together	ResNet-50	todos sculptum (48).JPG	fold1	5	*Amblyomma*
9214	All together	ResNet-50	todos sculptum (52).JPG	fold1	5	*Amblyomma*
9215	All together	ResNet-50	todos sculptum (55).JPG	fold1	5	*Amblyomma*
9216	All together	ResNet-50	todos sculptum (57).jpg	fold1	5	*Amblyomma*
9217	All together	ResNet-50	todos sculptum (61).jpg	fold1	5	*Amblyomma*
9218	All together	ResNet-50	todos sculptum (70).jpg	fold1	5	*Amblyomma*
9219	All together	ResNet-50	todos sculptum (71).JPG	fold1	5	*Amblyomma*
9220	All together	ResNet-50	todos sculptum (74).JPG	fold1	5	*Amblyomma*
9221	All together	ResNet-50	todos sculptum (75).JPG	fold1	5	*Amblyomma*
9222	All together	ResNet-50	todos sculptum (93).JPG	fold1	5	*Amblyomma*
9223	All together	ResNet-50	todos sculptum (97).JPG	fold1	5	*Amblyomma*
9224	All together	ResNet-50	todos sculptum (98).JPG	fold1	5	*Amblyomma*
9225	All together	ResNet-50	todos sculptum (99).JPG	fold1	5	*Amblyomma*
9226	All together	ResNet-50	todos triste (102).jpg	fold1	6	*Amblyomma*
9227	All together	ResNet-50	todos triste (104).jpg	fold1	6	*Amblyomma*
9228	All together	ResNet-50	todos triste (110).jpg	fold1	6	*Amblyomma*
9229	All together	ResNet-50	todos triste (116).jpg	fold1	6	*Amblyomma*
9230	All together	ResNet-50	todos triste (120).jpg	fold1	6	*Amblyomma*
9231	All together	ResNet-50	todos triste (121).jpg	fold1	6	*Amblyomma*
9232	All together	ResNet-50	todos triste (13).jpg	fold1	6	*Amblyomma*
9233	All together	ResNet-50	todos triste (131).jpg	fold1	6	*Amblyomma*
9234	All together	ResNet-50	todos triste (132).jpg	fold1	6	*Amblyomma*
9235	All together	ResNet-50	todos triste (133).jpg	fold1	6	*Amblyomma*
9236	All together	ResNet-50	todos triste (136).jpg	fold1	6	*Amblyomma*
9237	All together	ResNet-50	todos triste (140).jpg	fold1	6	*Amblyomma*
9238	All together	ResNet-50	todos triste (142).jpg	fold1	6	*Amblyomma*
9239	All together	ResNet-50	todos triste (146).jpg	fold1	6	*Amblyomma*
9240	All together	ResNet-50	todos triste (147).jpg	fold1	6	*Amblyomma*
9241	All together	ResNet-50	todos triste (149).jpg	fold1	6	*Amblyomma*
9242	All together	ResNet-50	todos triste (2).jpg	fold1	6	*Amblyomma*
9243	All together	ResNet-50	todos triste (29).jpg	fold1	6	*Amblyomma*
9244	All together	ResNet-50	todos triste (38).jpg	fold1	6	*Amblyomma*

9245	All together	ResNet-50	todos triste (49).jpg	fold1	6	*Amblyomma*
9246	All together	ResNet-50	todos triste (53).jpg	fold1	6	*Amblyomma*
9247	All together	ResNet-50	todos triste (58).jpg	fold1	6	*Amblyomma*
9248	All together	ResNet-50	todos triste (68).jpg	fold1	6	*Amblyomma*
9249	All together	ResNet-50	todos triste (70).jpg	fold1	6	*Amblyomma*
9250	All together	ResNet-50	todos triste (73).jpg	fold1	6	*Amblyomma*
9251	All together	ResNet-50	todos triste (76).jpg	fold1	6	*Amblyomma*
9252	All together	ResNet-50	todos triste (79).jpg	fold1	6	*Amblyomma*
9253	All together	ResNet-50	todos triste (8).jpg	fold1	6	*Amblyomma*
9254	All together	ResNet-50	todos triste (86).jpg	fold1	6	*Amblyomma*
9255	All together	ResNet-50	todos triste (95).jpg	fold1	6	*Amblyomma*
9256	All together	ResNet-50	todos triste (96).jpg	fold1	6	*Amblyomma*
9257	All together	ResNet-50	todos aureolatum (107).jpg	fold2	1	*Amblyomma*
9258	All together	ResNet-50	todos aureolatum (110).jpg	fold2	1	*Amblyomma*
9259	All together	ResNet-50	todos aureolatum (112).jpg	fold2	1	*Amblyomma*
9260	All together	ResNet-50	todos aureolatum (116).jpg	fold2	1	*Amblyomma*
9261	All together	ResNet-50	todos aureolatum (117).jpg	fold2	1	*Amblyomma*
9262	All together	ResNet-50	todos aureolatum (118).jpg	fold2	1	*Amblyomma*
9263	All together	ResNet-50	todos aureolatum (12).jpg	fold2	1	*Amblyomma*
9264	All together	ResNet-50	todos aureolatum (121).jpg	fold2	1	*Amblyomma*
9265	All together	ResNet-50	todos aureolatum (18).jpg	fold2	1	*Amblyomma*
9266	All together	ResNet-50	todos aureolatum (23).jpg	fold2	1	*Amblyomma*
9267	All together	ResNet-50	todos aureolatum (24).jpg	fold2	1	*Amblyomma*
9268	All together	ResNet-50	todos aureolatum (29).jpg	fold2	1	*Amblyomma*
9269	All together	ResNet-50	todos aureolatum (3).jpg	fold2	1	*Amblyomma*
9270	All together	ResNet-50	todos aureolatum (31).jpg	fold2	1	*Amblyomma*
9271	All together	ResNet-50	todos aureolatum (32).jpg	fold2	1	*Amblyomma*
9272	All together	ResNet-50	todos aureolatum (4).jpg	fold2	1	*Amblyomma*
9273	All together	ResNet-50	todos aureolatum (42).jpg	fold2	1	*Amblyomma*
9274	All together	ResNet-50	todos aureolatum (63).jpg	fold2	1	*Amblyomma*
9275	All together	ResNet-50	todos aureolatum (64).jpg	fold2	1	*Amblyomma*
9276	All together	ResNet-50	todos aureolatum (7).jpg	fold2	1	*Amblyomma*
9277	All together	ResNet-50	todos aureolatum (77).jpg	fold2	1	*Amblyomma*
9278	All together	ResNet-50	todos aureolatum (83).jpg	fold2	1	*Amblyomma*
9279	All together	ResNet-50	todos aureolatum (84).jpg	fold2	1	*Amblyomma*
9280	All together	ResNet-50	todos aureolatum (97).jpg	fold2	1	*Amblyomma*
9281	All together	ResNet-50	todos cajennense (11).jpg	fold2	2	*Amblyomma*
9282	All together	ResNet-50	todos cajennense (110).jpg	fold2	2	*Amblyomma*
9283	All together	ResNet-50	todos cajennense (116).jpg	fold2	2	*Amblyomma*
9284	All together	ResNet-50	todos cajennense (12).jpg	fold2	2	*Amblyomma*
9285	All together	ResNet-50	todos cajennense (120).jpg	fold2	2	*Amblyomma*
9286	All together	ResNet-50	todos cajennense (126).jpg	fold2	2	*Amblyomma*
9287	All together	ResNet-50	todos cajennense (128).jpg	fold2	2	*Amblyomma*
9288	All together	ResNet-50	todos cajennense (142).jpg	fold2	2	*Amblyomma*
9289	All together	ResNet-50	todos cajennense (143).jpg	fold2	2	*Amblyomma*
9290	All together	ResNet-50	todos cajennense (154).jpg	fold2	2	*Amblyomma*
9291	All together	ResNet-50	todos cajennense (155).jpg	fold2	2	*Amblyomma*
9292	All together	ResNet-50	todos cajennense (156).jpg	fold2	2	*Amblyomma*
9293	All together	ResNet-50	todos cajennense (158).jpg	fold2	2	*Amblyomma*
9294	All together	ResNet-50	todos cajennense (159).jpg	fold2	2	*Amblyomma*
9295	All together	ResNet-50	todos cajennense (162).jpg	fold2	2	*Amblyomma*
9296	All together	ResNet-50	todos cajennense (164).jpg	fold2	2	*Amblyomma*
9297	All together	ResNet-50	todos cajennense (23).jpg	fold2	2	*Amblyomma*
9298	All together	ResNet-50	todos cajennense (3).jpg	fold2	2	*Amblyomma*
9299	All together	ResNet-50	todos cajennense (32).jpg	fold2	2	*Amblyomma*
9300	All together	ResNet-50	todos cajennense (36).jpg	fold2	2	*Amblyomma*
9301	All together	ResNet-50	todos cajennense (39).jpg	fold2	2	*Amblyomma*
9302	All together	ResNet-50	todos cajennense (47).jpg	fold2	2	*Amblyomma*
9303	All together	ResNet-50	todos cajennense (48).jpg	fold2	2	*Amblyomma*
9304	All together	ResNet-50	todos cajennense (54).jpg	fold2	2	*Amblyomma*
9305	All together	ResNet-50	todos cajennense (57).jpg	fold2	2	*Amblyomma*
9306	All together	ResNet-50	todos cajennense (59).jpg	fold2	2	*Amblyomma*
9307	All together	ResNet-50	todos cajennense (6).jpg	fold2	2	*Amblyomma*
9308	All together	ResNet-50	todos cajennense (61).jpg	fold2	2	*Amblyomma*
9309	All together	ResNet-50	todos cajennense (64).jpg	fold2	2	*Amblyomma*
9310	All together	ResNet-50	todos cajennense (78).jpg	fold2	2	*Amblyomma*
9311	All together	ResNet-50	todos cajennense (87).jpg	fold2	2	*Amblyomma*

9312	All together	ResNet-50	todos cajennense (88).jpg	fold2	2	*Amblyomma*
9313	All together	ResNet-50	todos cajennense (98).jpg	fold2	2	*Amblyomma*
9314	All together	ResNet-50	todos dubitatum (1).jpg	fold2	3	*Amblyomma*
9315	All together	ResNet-50	todos dubitatum (105).jpg	fold2	3	*Amblyomma*
9316	All together	ResNet-50	todos dubitatum (107).jpg	fold2	3	*Amblyomma*
9317	All together	ResNet-50	todos dubitatum (11).JPG	fold2	3	*Amblyomma*
9318	All together	ResNet-50	todos dubitatum (25).jpg	fold2	3	*Amblyomma*
9319	All together	ResNet-50	todos dubitatum (26).jpg	fold2	3	*Amblyomma*
9320	All together	ResNet-50	todos dubitatum (46).jpg	fold2	3	*Amblyomma*
9321	All together	ResNet-50	todos dubitatum (47).jpg	fold2	3	*Amblyomma*
9322	All together	ResNet-50	todos dubitatum (49).jpg	fold2	3	*Amblyomma*
9323	All together	ResNet-50	todos dubitatum (5).jpg	fold2	3	*Amblyomma*
9324	All together	ResNet-50	todos dubitatum (57).jpg	fold2	3	*Amblyomma*
9325	All together	ResNet-50	todos dubitatum (63).jpg	fold2	3	*Amblyomma*
9326	All together	ResNet-50	todos dubitatum (69).jpg	fold2	3	*Amblyomma*
9327	All together	ResNet-50	todos dubitatum (71).jpg	fold2	3	*Amblyomma*
9328	All together	ResNet-50	todos dubitatum (74).jpg	fold2	3	*Amblyomma*
9329	All together	ResNet-50	todos dubitatum (78).jpg	fold2	3	*Amblyomma*
9330	All together	ResNet-50	todos dubitatum (8).jpg	fold2	3	*Amblyomma*
9331	All together	ResNet-50	todos dubitatum (85).jpg	fold2	3	*Amblyomma*
9332	All together	ResNet-50	todos dubitatum (92).jpg	fold2	3	*Amblyomma*
9333	All together	ResNet-50	todos dubitatum (94).jpg	fold2	3	*Amblyomma*
9334	All together	ResNet-50	todos dubitatum (95).jpg	fold2	3	*Amblyomma*
9335	All together	ResNet-50	todos dubitatum (96).jpg	fold2	3	*Amblyomma*
9336	All together	ResNet-50	todos ovale (10).jpg	fold2	4	*Amblyomma*
9337	All together	ResNet-50	todos ovale (11).jpg	fold2	4	*Amblyomma*
9338	All together	ResNet-50	todos ovale (110).jpg	fold2	4	*Amblyomma*
9339	All together	ResNet-50	todos ovale (112).jpg	fold2	4	*Amblyomma*
9340	All together	ResNet-50	todos ovale (2).jpg	fold2	4	*Amblyomma*
9341	All together	ResNet-50	todos ovale (23).jpg	fold2	4	*Amblyomma*
9342	All together	ResNet-50	todos ovale (25).jpg	fold2	4	*Amblyomma*
9343	All together	ResNet-50	todos ovale (29).jpg	fold2	4	*Amblyomma*
9344	All together	ResNet-50	todos ovale (32).jpg	fold2	4	*Amblyomma*
9345	All together	ResNet-50	todos ovale (44).jpg	fold2	4	*Amblyomma*
9346	All together	ResNet-50	todos ovale (46).jpg	fold2	4	*Amblyomma*
9347	All together	ResNet-50	todos ovale (56).jpg	fold2	4	*Amblyomma*
9348	All together	ResNet-50	todos ovale (57).jpg	fold2	4	*Amblyomma*
9349	All together	ResNet-50	todos ovale (58).jpg	fold2	4	*Amblyomma*
9350	All together	ResNet-50	todos ovale (67).jpg	fold2	4	*Amblyomma*
9351	All together	ResNet-50	todos ovale (68).jpg	fold2	4	*Amblyomma*
9352	All together	ResNet-50	todos ovale (74).jpg	fold2	4	*Amblyomma*
9353	All together	ResNet-50	todos ovale (76).JPG	fold2	4	*Amblyomma*
9354	All together	ResNet-50	todos ovale (78).jpg	fold2	4	*Amblyomma*
9355	All together	ResNet-50	todos ovale (8).jpg	fold2	4	*Amblyomma*
9356	All together	ResNet-50	todos ovale (86).jpg	fold2	4	*Amblyomma*
9357	All together	ResNet-50	todos ovale (96).jpg	fold2	4	*Amblyomma*
9358	All together	ResNet-50	todos ovale (97).jpg	fold2	4	*Amblyomma*
9359	All together	ResNet-50	todos ovale (99).jpg	fold2	4	*Amblyomma*
9360	All together	ResNet-50	todos sculptum (102).JPG	fold2	5	*Amblyomma*
9361	All together	ResNet-50	todos sculptum (105).JPG	fold2	5	*Amblyomma*
9362	All together	ResNet-50	todos sculptum (107).JPG	fold2	5	*Amblyomma*
9363	All together	ResNet-50	todos sculptum (109).JPG	fold2	5	*Amblyomma*
9364	All together	ResNet-50	todos sculptum (11).JPG	fold2	5	*Amblyomma*
9365	All together	ResNet-50	todos sculptum (12).JPG	fold2	5	*Amblyomma*
9366	All together	ResNet-50	todos sculptum (121).JPG	fold2	5	*Amblyomma*
9367	All together	ResNet-50	todos sculptum (122).JPG	fold2	5	*Amblyomma*
9368	All together	ResNet-50	todos sculptum (125).JPG	fold2	5	*Amblyomma*
9369	All together	ResNet-50	todos sculptum (128).JPG	fold2	5	*Amblyomma*
9370	All together	ResNet-50	todos sculptum (130).JPG	fold2	5	*Amblyomma*
9371	All together	ResNet-50	todos sculptum (14).JPG	fold2	5	*Amblyomma*
9372	All together	ResNet-50	todos sculptum (141).JPG	fold2	5	*Amblyomma*
9373	All together	ResNet-50	todos sculptum (147).jpg	fold2	5	*Amblyomma*
9374	All together	ResNet-50	todos sculptum (155).jpg	fold2	5	*Amblyomma*
9375	All together	ResNet-50	todos sculptum (25).JPG	fold2	5	*Amblyomma*
9376	All together	ResNet-50	todos sculptum (30).JPG	fold2	5	*Amblyomma*
9377	All together	ResNet-50	todos sculptum (33).JPG	fold2	5	*Amblyomma*
9378	All together	ResNet-50	todos sculptum (35).JPG	fold2	5	*Amblyomma*

9379	All together	ResNet-50	todos sculptum (36).JPG	fold2	5	*Amblyomma*
9380	All together	ResNet-50	todos sculptum (37).JPG	fold2	5	*Amblyomma*
9381	All together	ResNet-50	todos sculptum (4).jpg	fold2	5	*Amblyomma*
9382	All together	ResNet-50	todos sculptum (5).jpg	fold2	5	*Amblyomma*
9383	All together	ResNet-50	todos sculptum (51).JPG	fold2	5	*Amblyomma*
9384	All together	ResNet-50	todos sculptum (60).jpg	fold2	5	*Amblyomma*
9385	All together	ResNet-50	todos sculptum (63).jpg	fold2	5	*Amblyomma*
9386	All together	ResNet-50	todos sculptum (73).JPG	fold2	5	*Amblyomma*
9387	All together	ResNet-50	todos sculptum (80).JPG	fold2	5	*Amblyomma*
9388	All together	ResNet-50	todos sculptum (84).JPG	fold2	5	*Amblyomma*
9389	All together	ResNet-50	todos sculptum (85).JPG	fold2	5	*Amblyomma*
9390	All together	ResNet-50	todos sculptum (90).JPG	fold2	5	*Amblyomma*
9391	All together	ResNet-50	todos triste (103).jpg	fold2	6	*Amblyomma*
9392	All together	ResNet-50	todos triste (109).jpg	fold2	6	*Amblyomma*
9393	All together	ResNet-50	todos triste (123).jpg	fold2	6	*Amblyomma*
9394	All together	ResNet-50	todos triste (127).jpg	fold2	6	*Amblyomma*
9395	All together	ResNet-50	todos triste (128).jpg	fold2	6	*Amblyomma*
9396	All together	ResNet-50	todos triste (129).jpg	fold2	6	*Amblyomma*
9397	All together	ResNet-50	todos triste (130).jpg	fold2	6	*Amblyomma*
9398	All together	ResNet-50	todos triste (152).jpg	fold2	6	*Amblyomma*
9399	All together	ResNet-50	todos triste (20).jpg	fold2	6	*Amblyomma*
9400	All together	ResNet-50	todos triste (23).jpg	fold2	6	*Amblyomma*
9401	All together	ResNet-50	todos triste (24).jpg	fold2	6	*Amblyomma*
9402	All together	ResNet-50	todos triste (26).jpg	fold2	6	*Amblyomma*
9403	All together	ResNet-50	todos triste (27).jpg	fold2	6	*Amblyomma*
9404	All together	ResNet-50	todos triste (3).jpg	fold2	6	*Amblyomma*
9405	All together	ResNet-50	todos triste (35).jpg	fold2	6	*Amblyomma*
9406	All together	ResNet-50	todos triste (36).jpg	fold2	6	*Amblyomma*
9407	All together	ResNet-50	todos triste (42).jpg	fold2	6	*Amblyomma*
9408	All together	ResNet-50	todos triste (48).jpg	fold2	6	*Amblyomma*
9409	All together	ResNet-50	todos triste (5).jpg	fold2	6	*Amblyomma*
9410	All together	ResNet-50	todos triste (52).jpg	fold2	6	*Amblyomma*
9411	All together	ResNet-50	todos triste (54).jpg	fold2	6	*Amblyomma*
9412	All together	ResNet-50	todos triste (55).jpg	fold2	6	*Amblyomma*
9413	All together	ResNet-50	todos triste (56).jpg	fold2	6	*Amblyomma*
9414	All together	ResNet-50	todos triste (57).jpg	fold2	6	*Amblyomma*
9415	All together	ResNet-50	todos triste (59).jpg	fold2	6	*Amblyomma*
9416	All together	ResNet-50	todos triste (64).jpg	fold2	6	*Amblyomma*
9417	All together	ResNet-50	todos triste (65).jpg	fold2	6	*Amblyomma*
9418	All together	ResNet-50	todos triste (74).jpg	fold2	6	*Amblyomma*
9419	All together	ResNet-50	todos triste (87).jpg	fold2	6	*Amblyomma*
9420	All together	ResNet-50	todos triste (9).jpg	fold2	6	*Amblyomma*
9421	All together	ResNet-50	todos triste (92).jpg	fold2	6	*Amblyomma*
9422	All together	ResNet-50	todos aureolatum (1).jpg	fold3	1	*Amblyomma*
9423	All together	ResNet-50	todos aureolatum (103).jpg	fold3	1	*Amblyomma*
9424	All together	ResNet-50	todos aureolatum (108).jpg	fold3	1	*Amblyomma*
9425	All together	ResNet-50	todos aureolatum (113).jpg	fold3	1	*Amblyomma*
9426	All together	ResNet-50	todos aureolatum (120).jpg	fold3	1	*Amblyomma*
9427	All together	ResNet-50	todos aureolatum (13).jpg	fold3	1	*Amblyomma*
9428	All together	ResNet-50	todos aureolatum (15).jpg	fold3	1	*Amblyomma*
9429	All together	ResNet-50	todos aureolatum (16).jpg	fold3	1	*Amblyomma*
9430	All together	ResNet-50	todos aureolatum (20).jpg	fold3	1	*Amblyomma*
9431	All together	ResNet-50	todos aureolatum (33).jpg	fold3	1	*Amblyomma*
9432	All together	ResNet-50	todos aureolatum (34).jpg	fold3	1	*Amblyomma*
9433	All together	ResNet-50	todos aureolatum (46).jpg	fold3	1	*Amblyomma*
9434	All together	ResNet-50	todos aureolatum (47).jpg	fold3	1	*Amblyomma*
9435	All together	ResNet-50	todos aureolatum (67).jpg	fold3	1	*Amblyomma*
9436	All together	ResNet-50	todos aureolatum (68).jpg	fold3	1	*Amblyomma*
9437	All together	ResNet-50	todos aureolatum (71).jpg	fold3	1	*Amblyomma*
9438	All together	ResNet-50	todos aureolatum (74).jpg	fold3	1	*Amblyomma*
9439	All together	ResNet-50	todos aureolatum (76).jpg	fold3	1	*Amblyomma*
9440	All together	ResNet-50	todos aureolatum (8).jpg	fold3	1	*Amblyomma*
9441	All together	ResNet-50	todos aureolatum (86).jpg	fold3	1	*Amblyomma*
9442	All together	ResNet-50	todos aureolatum (89).jpg	fold3	1	*Amblyomma*
9443	All together	ResNet-50	todos aureolatum (92).jpg	fold3	1	*Amblyomma*
9444	All together	ResNet-50	todos aureolatum (94).jpg	fold3	1	*Amblyomma*
9445	All together	ResNet-50	todos aureolatum (96).jpg	fold3	1	*Amblyomma*

9446	All together	ResNet-50	todos cajennense (100).jpg	fold3	2 *Amblyomma*
9447	All together	ResNet-50	todos cajennense (109).jpg	fold3	2 *Amblyomma*
9448	All together	ResNet-50	todos cajennense (112).jpg	fold3	2 *Amblyomma*
9449	All together	ResNet-50	todos cajennense (115).jpg	fold3	2 *Amblyomma*
9450	All together	ResNet-50	todos cajennense (121).jpg	fold3	2 *Amblyomma*
9451	All together	ResNet-50	todos cajennense (123).jpg	fold3	2 *Amblyomma*
9452	All together	ResNet-50	todos cajennense (127).jpg	fold3	2 *Amblyomma*
9453	All together	ResNet-50	todos cajennense (134).jpg	fold3	2 *Amblyomma*
9454	All together	ResNet-50	todos cajennense (135).jpg	fold3	2 *Amblyomma*
9455	All together	ResNet-50	todos cajennense (144).jpg	fold3	2 *Amblyomma*
9456	All together	ResNet-50	todos cajennense (149).jpg	fold3	2 *Amblyomma*
9457	All together	ResNet-50	todos cajennense (157).jpg	fold3	2 *Amblyomma*
9458	All together	ResNet-50	todos cajennense (165).jpg	fold3	2 *Amblyomma*
9459	All together	ResNet-50	todos cajennense (21).jpg	fold3	2 *Amblyomma*
9460	All together	ResNet-50	todos cajennense (22).jpg	fold3	2 *Amblyomma*
9461	All together	ResNet-50	todos cajennense (24).jpg	fold3	2 *Amblyomma*
9462	All together	ResNet-50	todos cajennense (27).jpg	fold3	2 *Amblyomma*
9463	All together	ResNet-50	todos cajennense (29).jpg	fold3	2 *Amblyomma*
9464	All together	ResNet-50	todos cajennense (30).jpg	fold3	2 *Amblyomma*
9465	All together	ResNet-50	todos cajennense (38).jpg	fold3	2 *Amblyomma*
9466	All together	ResNet-50	todos cajennense (40).jpg	fold3	2 *Amblyomma*
9467	All together	ResNet-50	todos cajennense (43).jpg	fold3	2 *Amblyomma*
9468	All together	ResNet-50	todos cajennense (45).jpg	fold3	2 *Amblyomma*
9469	All together	ResNet-50	todos cajennense (46).jpg	fold3	2 *Amblyomma*
9470	All together	ResNet-50	todos cajennense (5).jpg	fold3	2 *Amblyomma*
9471	All together	ResNet-50	todos cajennense (56).jpg	fold3	2 *Amblyomma*
9472	All together	ResNet-50	todos cajennense (60).jpg	fold3	2 *Amblyomma*
9473	All together	ResNet-50	todos cajennense (65).jpg	fold3	2 *Amblyomma*
9474	All together	ResNet-50	todos cajennense (71).jpg	fold3	2 *Amblyomma*
9475	All together	ResNet-50	todos cajennense (72).jpg	fold3	2 *Amblyomma*
9476	All together	ResNet-50	todos cajennense (82).jpg	fold3	2 *Amblyomma*
9477	All together	ResNet-50	todos cajennense (84).jpg	fold3	2 *Amblyomma*
9478	All together	ResNet-50	todos cajennense (95).jpg	fold3	2 *Amblyomma*
9479	All together	ResNet-50	todos dubitatum (101).jpg	fold3	3 *Amblyomma*
9480	All together	ResNet-50	todos dubitatum (108).jpg	fold3	3 *Amblyomma*
9481	All together	ResNet-50	todos dubitatum (13).JPG	fold3	3 *Amblyomma*
9482	All together	ResNet-50	todos dubitatum (14).JPG	fold3	3 *Amblyomma*
9483	All together	ResNet-50	todos dubitatum (19).jpg	fold3	3 *Amblyomma*
9484	All together	ResNet-50	todos dubitatum (22).jpg	fold3	3 *Amblyomma*
9485	All together	ResNet-50	todos dubitatum (23).jpg	fold3	3 *Amblyomma*
9486	All together	ResNet-50	todos dubitatum (24).jpg	fold3	3 *Amblyomma*
9487	All together	ResNet-50	todos dubitatum (28).jpg	fold3	3 *Amblyomma*
9488	All together	ResNet-50	todos dubitatum (34).jpg	fold3	3 *Amblyomma*
9489	All together	ResNet-50	todos dubitatum (40).jpg	fold3	3 *Amblyomma*
9490	All together	ResNet-50	todos dubitatum (41).jpg	fold3	3 *Amblyomma*
9491	All together	ResNet-50	todos dubitatum (43).jpg	fold3	3 *Amblyomma*
9492	All together	ResNet-50	todos dubitatum (48).jpg	fold3	3 *Amblyomma*
9493	All together	ResNet-50	todos dubitatum (55).jpg	fold3	3 *Amblyomma*
9494	All together	ResNet-50	todos dubitatum (6).jpg	fold3	3 *Amblyomma*
9495	All together	ResNet-50	todos dubitatum (66).jpg	fold3	3 *Amblyomma*
9496	All together	ResNet-50	todos dubitatum (67).jpg	fold3	3 *Amblyomma*
9497	All together	ResNet-50	todos dubitatum (70).jpg	fold3	3 *Amblyomma*
9498	All together	ResNet-50	todos dubitatum (87).jpg	fold3	3 *Amblyomma*
9499	All together	ResNet-50	todos dubitatum (90).jpg	fold3	3 *Amblyomma*
9500	All together	ResNet-50	todos dubitatum (93).jpg	fold3	3 *Amblyomma*
9501	All together	ResNet-50	todos ovale (101).jpg	fold3	4 *Amblyomma*
9502	All together	ResNet-50	todos ovale (105).jpg	fold3	4 *Amblyomma*
9503	All together	ResNet-50	todos ovale (107).JPG	fold3	4 *Amblyomma*
9504	All together	ResNet-50	todos ovale (120).jpg	fold3	4 *Amblyomma*
9505	All together	ResNet-50	todos ovale (13).jpg	fold3	4 *Amblyomma*
9506	All together	ResNet-50	todos ovale (19).jpg	fold3	4 *Amblyomma*
9507	All together	ResNet-50	todos ovale (22).jpg	fold3	4 *Amblyomma*
9508	All together	ResNet-50	todos ovale (3).jpg	fold3	4 *Amblyomma*
9509	All together	ResNet-50	todos ovale (30).jpg	fold3	4 *Amblyomma*
9510	All together	ResNet-50	todos ovale (33).jpg	fold3	4 *Amblyomma*
9511	All together	ResNet-50	todos ovale (36).jpg	fold3	4 *Amblyomma*
9512	All together	ResNet-50	todos ovale (41).jpg	fold3	4 *Amblyomma*

9513	All together	ResNet-50	todos ovale (43).jpg	fold3	4	*Amblyomma*
9514	All together	ResNet-50	todos ovale (52).jpg	fold3	4	*Amblyomma*
9515	All together	ResNet-50	todos ovale (54).jpg	fold3	4	*Amblyomma*
9516	All together	ResNet-50	todos ovale (55).jpg	fold3	4	*Amblyomma*
9517	All together	ResNet-50	todos ovale (71).jpg	fold3	4	*Amblyomma*
9518	All together	ResNet-50	todos ovale (81).jpg	fold3	4	*Amblyomma*
9519	All together	ResNet-50	todos ovale (82).jpg	fold3	4	*Amblyomma*
9520	All together	ResNet-50	todos ovale (83).jpg	fold3	4	*Amblyomma*
9521	All together	ResNet-50	todos ovale (84).jpg	fold3	4	*Amblyomma*
9522	All together	ResNet-50	todos ovale (92).jpg	fold3	4	*Amblyomma*
9523	All together	ResNet-50	todos ovale (93).jpg	fold3	4	*Amblyomma*
9524	All together	ResNet-50	todos ovale (95).jpg	fold3	4	*Amblyomma*
9525	All together	ResNet-50	todos sculptum (116).JPG	fold3	5	*Amblyomma*
9526	All together	ResNet-50	todos sculptum (118).JPG	fold3	5	*Amblyomma*
9527	All together	ResNet-50	todos sculptum (126).JPG	fold3	5	*Amblyomma*
9528	All together	ResNet-50	todos sculptum (13).JPG	fold3	5	*Amblyomma*
9529	All together	ResNet-50	todos sculptum (132).JPG	fold3	5	*Amblyomma*
9530	All together	ResNet-50	todos sculptum (133).JPG	fold3	5	*Amblyomma*
9531	All together	ResNet-50	todos sculptum (135).JPG	fold3	5	*Amblyomma*
9532	All together	ResNet-50	todos sculptum (140).JPG	fold3	5	*Amblyomma*
9533	All together	ResNet-50	todos sculptum (152).jpg	fold3	5	*Amblyomma*
9534	All together	ResNet-50	todos sculptum (154).jpg	fold3	5	*Amblyomma*
9535	All together	ResNet-50	todos sculptum (17).JPG	fold3	5	*Amblyomma*
9536	All together	ResNet-50	todos sculptum (18).JPG	fold3	5	*Amblyomma*
9537	All together	ResNet-50	todos sculptum (19).JPG	fold3	5	*Amblyomma*
9538	All together	ResNet-50	todos sculptum (20).JPG	fold3	5	*Amblyomma*
9539	All together	ResNet-50	todos sculptum (22).JPG	fold3	5	*Amblyomma*
9540	All together	ResNet-50	todos sculptum (28).JPG	fold3	5	*Amblyomma*
9541	All together	ResNet-50	todos sculptum (3).jpg	fold3	5	*Amblyomma*
9542	All together	ResNet-50	todos sculptum (38).JPG	fold3	5	*Amblyomma*
9543	All together	ResNet-50	todos sculptum (39).JPG	fold3	5	*Amblyomma*
9544	All together	ResNet-50	todos sculptum (41).JPG	fold3	5	*Amblyomma*
9545	All together	ResNet-50	todos sculptum (45).JPG	fold3	5	*Amblyomma*
9546	All together	ResNet-50	todos sculptum (47).JPG	fold3	5	*Amblyomma*
9547	All together	ResNet-50	todos sculptum (69).jpg	fold3	5	*Amblyomma*
9548	All together	ResNet-50	todos sculptum (7).jpg	fold3	5	*Amblyomma*
9549	All together	ResNet-50	todos sculptum (76).JPG	fold3	5	*Amblyomma*
9550	All together	ResNet-50	todos sculptum (83).JPG	fold3	5	*Amblyomma*
9551	All together	ResNet-50	todos sculptum (87).JPG	fold3	5	*Amblyomma*
9552	All together	ResNet-50	todos sculptum (88).JPG	fold3	5	*Amblyomma*
9553	All together	ResNet-50	todos sculptum (89).JPG	fold3	5	*Amblyomma*
9554	All together	ResNet-50	todos sculptum (9).JPG	fold3	5	*Amblyomma*
9555	All together	ResNet-50	todos sculptum (96).JPG	fold3	5	*Amblyomma*
9556	All together	ResNet-50	todos triste (101).jpg	fold3	6	*Amblyomma*
9557	All together	ResNet-50	todos triste (106).jpg	fold3	6	*Amblyomma*
9558	All together	ResNet-50	todos triste (115).jpg	fold3	6	*Amblyomma*
9559	All together	ResNet-50	todos triste (118).jpg	fold3	6	*Amblyomma*
9560	All together	ResNet-50	todos triste (126).jpg	fold3	6	*Amblyomma*
9561	All together	ResNet-50	todos triste (135).jpg	fold3	6	*Amblyomma*
9562	All together	ResNet-50	todos triste (137).jpg	fold3	6	*Amblyomma*
9563	All together	ResNet-50	todos triste (139).jpg	fold3	6	*Amblyomma*
9564	All together	ResNet-50	todos triste (141).jpg	fold3	6	*Amblyomma*
9565	All together	ResNet-50	todos triste (144).jpg	fold3	6	*Amblyomma*
9566	All together	ResNet-50	todos triste (148).jpg	fold3	6	*Amblyomma*
9567	All together	ResNet-50	todos triste (15).jpg	fold3	6	*Amblyomma*
9568	All together	ResNet-50	todos triste (153).jpg	fold3	6	*Amblyomma*
9569	All together	ResNet-50	todos triste (154).jpg	fold3	6	*Amblyomma*
9570	All together	ResNet-50	todos triste (17).jpg	fold3	6	*Amblyomma*
9571	All together	ResNet-50	todos triste (21).jpg	fold3	6	*Amblyomma*
9572	All together	ResNet-50	todos triste (22).jpg	fold3	6	*Amblyomma*
9573	All together	ResNet-50	todos triste (32).jpg	fold3	6	*Amblyomma*
9574	All together	ResNet-50	todos triste (37).jpg	fold3	6	*Amblyomma*
9575	All together	ResNet-50	todos triste (43).jpg	fold3	6	*Amblyomma*
9576	All together	ResNet-50	todos triste (44).jpg	fold3	6	*Amblyomma*
9577	All together	ResNet-50	todos triste (45).jpg	fold3	6	*Amblyomma*
9578	All together	ResNet-50	todos triste (50).jpg	fold3	6	*Amblyomma*
9579	All together	ResNet-50	todos triste (51).jpg	fold3	6	*Amblyomma*

9580	All together	ResNet-50	todos triste (61).jpg	fold3	6	*Amblyomma*
9581	All together	ResNet-50	todos triste (69).jpg	fold3	6	*Amblyomma*
9582	All together	ResNet-50	todos triste (78).jpg	fold3	6	*Amblyomma*
9583	All together	ResNet-50	todos triste (81).jpg	fold3	6	*Amblyomma*
9584	All together	ResNet-50	todos triste (83).jpg	fold3	6	*Amblyomma*
9585	All together	ResNet-50	todos triste (84).jpg	fold3	6	*Amblyomma*
9586	All together	ResNet-50	todos triste (93).jpg	fold3	6	*Amblyomma*
9587	All together	ResNet-50	todos aureolatum (101).jpg	fold4	1	*Amblyomma*
9588	All together	ResNet-50	todos aureolatum (102).jpg	fold4	1	*Amblyomma*
9589	All together	ResNet-50	todos aureolatum (104).jpg	fold4	1	*Amblyomma*
9590	All together	ResNet-50	todos aureolatum (105).jpg	fold4	1	*Amblyomma*
9591	All together	ResNet-50	todos aureolatum (11).jpg	fold4	1	*Amblyomma*
9592	All together	ResNet-50	todos aureolatum (111).jpg	fold4	1	*Amblyomma*
9593	All together	ResNet-50	todos aureolatum (114).jpg	fold4	1	*Amblyomma*
9594	All together	ResNet-50	todos aureolatum (115).jpg	fold4	1	*Amblyomma*
9595	All together	ResNet-50	todos aureolatum (17).jpg	fold4	1	*Amblyomma*
9596	All together	ResNet-50	todos aureolatum (2).jpg	fold4	1	*Amblyomma*
9597	All together	ResNet-50	todos aureolatum (36).jpg	fold4	1	*Amblyomma*
9598	All together	ResNet-50	todos aureolatum (43).jpg	fold4	1	*Amblyomma*
9599	All together	ResNet-50	todos aureolatum (44).jpg	fold4	1	*Amblyomma*
9600	All together	ResNet-50	todos aureolatum (49).jpg	fold4	1	*Amblyomma*
9601	All together	ResNet-50	todos aureolatum (50).jpg	fold4	1	*Amblyomma*
9602	All together	ResNet-50	todos aureolatum (52).jpg	fold4	1	*Amblyomma*
9603	All together	ResNet-50	todos aureolatum (59).jpg	fold4	1	*Amblyomma*
9604	All together	ResNet-50	todos aureolatum (61).jpg	fold4	1	*Amblyomma*
9605	All together	ResNet-50	todos aureolatum (70).jpg	fold4	1	*Amblyomma*
9606	All together	ResNet-50	todos aureolatum (88).jpg	fold4	1	*Amblyomma*
9607	All together	ResNet-50	todos aureolatum (9).jpg	fold4	1	*Amblyomma*
9608	All together	ResNet-50	todos aureolatum (91).jpg	fold4	1	*Amblyomma*
9609	All together	ResNet-50	todos aureolatum (95).jpg	fold4	1	*Amblyomma*
9610	All together	ResNet-50	todos aureolatum (98).jpg	fold4	1	*Amblyomma*
9611	All together	ResNet-50	todos aureolatum (99).jpg	fold4	1	*Amblyomma*
9612	All together	ResNet-50	todos cajennense (1).jpg	fold4	2	*Amblyomma*
9613	All together	ResNet-50	todos cajennense (103).jpg	fold4	2	*Amblyomma*
9614	All together	ResNet-50	todos cajennense (107).jpg	fold4	2	*Amblyomma*
9615	All together	ResNet-50	todos cajennense (118).jpg	fold4	2	*Amblyomma*
9616	All together	ResNet-50	todos cajennense (119).jpg	fold4	2	*Amblyomma*
9617	All together	ResNet-50	todos cajennense (122).jpg	fold4	2	*Amblyomma*
9618	All together	ResNet-50	todos cajennense (125).jpg	fold4	2	*Amblyomma*
9619	All together	ResNet-50	todos cajennense (130).jpg	fold4	2	*Amblyomma*
9620	All together	ResNet-50	todos cajennense (131).jpg	fold4	2	*Amblyomma*
9621	All together	ResNet-50	todos cajennense (136).jpg	fold4	2	*Amblyomma*
9622	All together	ResNet-50	todos cajennense (152).jpg	fold4	2	*Amblyomma*
9623	All together	ResNet-50	todos cajennense (16).jpg	fold4	2	*Amblyomma*
9624	All together	ResNet-50	todos cajennense (160).jpg	fold4	2	*Amblyomma*
9625	All together	ResNet-50	todos cajennense (163).jpg	fold4	2	*Amblyomma*
9626	All together	ResNet-50	todos cajennense (17).jpg	fold4	2	*Amblyomma*
9627	All together	ResNet-50	todos cajennense (18).jpg	fold4	2	*Amblyomma*
9628	All together	ResNet-50	todos cajennense (25).jpg	fold4	2	*Amblyomma*
9629	All together	ResNet-50	todos cajennense (31).jpg	fold4	2	*Amblyomma*
9630	All together	ResNet-50	todos cajennense (34).jpg	fold4	2	*Amblyomma*
9631	All together	ResNet-50	todos cajennense (35).jpg	fold4	2	*Amblyomma*
9632	All together	ResNet-50	todos cajennense (50).jpg	fold4	2	*Amblyomma*
9633	All together	ResNet-50	todos cajennense (52).jpg	fold4	2	*Amblyomma*
9634	All together	ResNet-50	todos cajennense (58).jpg	fold4	2	*Amblyomma*
9635	All together	ResNet-50	todos cajennense (66).jpg	fold4	2	*Amblyomma*
9636	All together	ResNet-50	todos cajennense (68).jpg	fold4	2	*Amblyomma*
9637	All together	ResNet-50	todos cajennense (69).jpg	fold4	2	*Amblyomma*
9638	All together	ResNet-50	todos cajennense (7).jpg	fold4	2	*Amblyomma*
9639	All together	ResNet-50	todos cajennense (80).jpg	fold4	2	*Amblyomma*
9640	All together	ResNet-50	todos cajennense (81).jpg	fold4	2	*Amblyomma*
9641	All together	ResNet-50	todos cajennense (89).jpg	fold4	2	*Amblyomma*
9642	All together	ResNet-50	todos cajennense (91).jpg	fold4	2	*Amblyomma*
9643	All together	ResNet-50	todos cajennense (92).jpg	fold4	2	*Amblyomma*
9644	All together	ResNet-50	todos cajennense (96).jpg	fold4	2	*Amblyomma*
9645	All together	ResNet-50	todos dubitatum (18).jpg	fold4	3	*Amblyomma*
9646	All together	ResNet-50	todos dubitatum (27).jpg	fold4	3	*Amblyomma*

9647	All together	ResNet-50	todos dubitatum (32).jpg	fold4	3	*Amblyomma*
9648	All together	ResNet-50	todos dubitatum (33).jpg	fold4	3	*Amblyomma*
9649	All together	ResNet-50	todos dubitatum (35).jpg	fold4	3	*Amblyomma*
9650	All together	ResNet-50	todos dubitatum (4).jpg	fold4	3	*Amblyomma*
9651	All together	ResNet-50	todos dubitatum (45).jpg	fold4	3	*Amblyomma*
9652	All together	ResNet-50	todos dubitatum (50).jpg	fold4	3	*Amblyomma*
9653	All together	ResNet-50	todos dubitatum (53).jpg	fold4	3	*Amblyomma*
9654	All together	ResNet-50	todos dubitatum (58).jpg	fold4	3	*Amblyomma*
9655	All together	ResNet-50	todos dubitatum (59).jpg	fold4	3	*Amblyomma*
9656	All together	ResNet-50	todos dubitatum (64).jpg	fold4	3	*Amblyomma*
9657	All together	ResNet-50	todos dubitatum (65).jpg	fold4	3	*Amblyomma*
9658	All together	ResNet-50	todos dubitatum (7).jpg	fold4	3	*Amblyomma*
9659	All together	ResNet-50	todos dubitatum (72).jpg	fold4	3	*Amblyomma*
9660	All together	ResNet-50	todos dubitatum (73).jpg	fold4	3	*Amblyomma*
9661	All together	ResNet-50	todos dubitatum (75).jpg	fold4	3	*Amblyomma*
9662	All together	ResNet-50	todos dubitatum (79).jpg	fold4	3	*Amblyomma*
9663	All together	ResNet-50	todos dubitatum (84).jpg	fold4	3	*Amblyomma*
9664	All together	ResNet-50	todos dubitatum (88).jpg	fold4	3	*Amblyomma*
9665	All together	ResNet-50	todos dubitatum (9).jpg	fold4	3	*Amblyomma*
9666	All together	ResNet-50	todos dubitatum (99).jpg	fold4	3	*Amblyomma*
9667	All together	ResNet-50	todos ovale (1).jpg	fold4	4	*Amblyomma*
9668	All together	ResNet-50	todos ovale (103).jpg	fold4	4	*Amblyomma*
9669	All together	ResNet-50	todos ovale (111).jpg	fold4	4	*Amblyomma*
9670	All together	ResNet-50	todos ovale (113).jpg	fold4	4	*Amblyomma*
9671	All together	ResNet-50	todos ovale (115).jpg	fold4	4	*Amblyomma*
9672	All together	ResNet-50	todos ovale (15).jpg	fold4	4	*Amblyomma*
9673	All together	ResNet-50	todos ovale (17).jpg	fold4	4	*Amblyomma*
9674	All together	ResNet-50	todos ovale (21).jpg	fold4	4	*Amblyomma*
9675	All together	ResNet-50	todos ovale (26).jpg	fold4	4	*Amblyomma*
9676	All together	ResNet-50	todos ovale (28).jpg	fold4	4	*Amblyomma*
9677	All together	ResNet-50	todos ovale (37).jpg	fold4	4	*Amblyomma*
9678	All together	ResNet-50	todos ovale (39).jpg	fold4	4	*Amblyomma*
9679	All together	ResNet-50	todos ovale (40).jpg	fold4	4	*Amblyomma*
9680	All together	ResNet-50	todos ovale (51).jpg	fold4	4	*Amblyomma*
9681	All together	ResNet-50	todos ovale (6).jpg	fold4	4	*Amblyomma*
9682	All together	ResNet-50	todos ovale (63).jpg	fold4	4	*Amblyomma*
9683	All together	ResNet-50	todos ovale (64).jpg	fold4	4	*Amblyomma*
9684	All together	ResNet-50	todos ovale (69).jpg	fold4	4	*Amblyomma*
9685	All together	ResNet-50	todos ovale (72).jpg	fold4	4	*Amblyomma*
9686	All together	ResNet-50	todos ovale (80).jpg	fold4	4	*Amblyomma*
9687	All together	ResNet-50	todos ovale (88).jpg	fold4	4	*Amblyomma*
9688	All together	ResNet-50	todos ovale (9).jpg	fold4	4	*Amblyomma*
9689	All together	ResNet-50	todos ovale (90).jpg	fold4	4	*Amblyomma*
9690	All together	ResNet-50	todos ovale (91).jpg	fold4	4	*Amblyomma*
9691	All together	ResNet-50	todos ovale (98).jpg	fold4	4	*Amblyomma*
9692	All together	ResNet-50	todos sculptum (10).JPG	fold4	5	*Amblyomma*
9693	All together	ResNet-50	todos sculptum (104).JPG	fold4	5	*Amblyomma*
9694	All together	ResNet-50	todos sculptum (110).JPG	fold4	5	*Amblyomma*
9695	All together	ResNet-50	todos sculptum (117).JPG	fold4	5	*Amblyomma*
9696	All together	ResNet-50	todos sculptum (129).JPG	fold4	5	*Amblyomma*
9697	All together	ResNet-50	todos sculptum (134).JPG	fold4	5	*Amblyomma*
9698	All together	ResNet-50	todos sculptum (136).JPG	fold4	5	*Amblyomma*
9699	All together	ResNet-50	todos sculptum (143).JPG	fold4	5	*Amblyomma*
9700	All together	ResNet-50	todos sculptum (148).jpg	fold4	5	*Amblyomma*
9701	All together	ResNet-50	todos sculptum (15).JPG	fold4	5	*Amblyomma*
9702	All together	ResNet-50	todos sculptum (153).jpg	fold4	5	*Amblyomma*
9703	All together	ResNet-50	todos sculptum (16).JPG	fold4	5	*Amblyomma*
9704	All together	ResNet-50	todos sculptum (21).JPG	fold4	5	*Amblyomma*
9705	All together	ResNet-50	todos sculptum (23).JPG	fold4	5	*Amblyomma*
9706	All together	ResNet-50	todos sculptum (24).JPG	fold4	5	*Amblyomma*
9707	All together	ResNet-50	todos sculptum (27).JPG	fold4	5	*Amblyomma*
9708	All together	ResNet-50	todos sculptum (29).JPG	fold4	5	*Amblyomma*
9709	All together	ResNet-50	todos sculptum (31).JPG	fold4	5	*Amblyomma*
9710	All together	ResNet-50	todos sculptum (40).JPG	fold4	5	*Amblyomma*
9711	All together	ResNet-50	todos sculptum (42).JPG	fold4	5	*Amblyomma*
9712	All together	ResNet-50	todos sculptum (46).JPG	fold4	5	*Amblyomma*
9713	All together	ResNet-50	todos sculptum (49).JPG	fold4	5	*Amblyomma*

9714	All together	ResNet-50	todos sculptum (54).JPG	fold4	5	*Amblyomma*
9715	All together	ResNet-50	todos sculptum (62).jpg	fold4	5	*Amblyomma*
9716	All together	ResNet-50	todos sculptum (64).jpg	fold4	5	*Amblyomma*
9717	All together	ResNet-50	todos sculptum (65).jpg	fold4	5	*Amblyomma*
9718	All together	ResNet-50	todos sculptum (66).jpg	fold4	5	*Amblyomma*
9719	All together	ResNet-50	todos sculptum (68).jpg	fold4	5	*Amblyomma*
9720	All together	ResNet-50	todos sculptum (72).JPG	fold4	5	*Amblyomma*
9721	All together	ResNet-50	todos sculptum (81).JPG	fold4	5	*Amblyomma*
9722	All together	ResNet-50	todos sculptum (91).JPG	fold4	5	*Amblyomma*
9723	All together	ResNet-50	todos triste (1).jpg	fold4	6	*Amblyomma*
9724	All together	ResNet-50	todos triste (105).jpg	fold4	6	*Amblyomma*
9725	All together	ResNet-50	todos triste (107).jpg	fold4	6	*Amblyomma*
9726	All together	ResNet-50	todos triste (11).jpg	fold4	6	*Amblyomma*
9727	All together	ResNet-50	todos triste (113).jpg	fold4	6	*Amblyomma*
9728	All together	ResNet-50	todos triste (114).jpg	fold4	6	*Amblyomma*
9729	All together	ResNet-50	todos triste (122).jpg	fold4	6	*Amblyomma*
9730	All together	ResNet-50	todos triste (125).jpg	fold4	6	*Amblyomma*
9731	All together	ResNet-50	todos triste (134).jpg	fold4	6	*Amblyomma*
9732	All together	ResNet-50	todos triste (14).jpg	fold4	6	*Amblyomma*
9733	All together	ResNet-50	todos triste (145).jpg	fold4	6	*Amblyomma*
9734	All together	ResNet-50	todos triste (151).jpg	fold4	6	*Amblyomma*
9735	All together	ResNet-50	todos triste (155).jpg	fold4	6	*Amblyomma*
9736	All together	ResNet-50	todos triste (156).jpg	fold4	6	*Amblyomma*
9737	All together	ResNet-50	todos triste (18).jpg	fold4	6	*Amblyomma*
9738	All together	ResNet-50	todos triste (28).jpg	fold4	6	*Amblyomma*
9739	All together	ResNet-50	todos triste (33).jpg	fold4	6	*Amblyomma*
9740	All together	ResNet-50	todos triste (40).jpg	fold4	6	*Amblyomma*
9741	All together	ResNet-50	todos triste (41).jpg	fold4	6	*Amblyomma*
9742	All together	ResNet-50	todos triste (46).jpg	fold4	6	*Amblyomma*
9743	All together	ResNet-50	todos triste (47).jpg	fold4	6	*Amblyomma*
9744	All together	ResNet-50	todos triste (6).jpg	fold4	6	*Amblyomma*
9745	All together	ResNet-50	todos triste (60).jpg	fold4	6	*Amblyomma*
9746	All together	ResNet-50	todos triste (66).jpg	fold4	6	*Amblyomma*
9747	All together	ResNet-50	todos triste (7).jpg	fold4	6	*Amblyomma*
9748	All together	ResNet-50	todos triste (77).jpg	fold4	6	*Amblyomma*
9749	All together	ResNet-50	todos triste (85).jpg	fold4	6	*Amblyomma*
9750	All together	ResNet-50	todos triste (88).jpg	fold4	6	*Amblyomma*
9751	All together	ResNet-50	todos triste (90).jpg	fold4	6	*Amblyomma*
9752	All together	ResNet-50	todos triste (94).jpg	fold4	6	*Amblyomma*
9753	All together	ResNet-50	todos triste (97).jpg	fold4	6	*Amblyomma*
9754	All together	ResNet-50	todos triste (99).jpg	fold4	6	*Amblyomma*
9755	All together	ResNet-50	todos aureolatum (10).jpg	fold5	1	*Amblyomma*
9756	All together	ResNet-50	todos aureolatum (109).jpg	fold5	1	*Amblyomma*
9757	All together	ResNet-50	todos aureolatum (119).jpg	fold5	1	*Amblyomma*
9758	All together	ResNet-50	todos aureolatum (14).jpg	fold5	1	*Amblyomma*
9759	All together	ResNet-50	todos aureolatum (19).jpg	fold5	1	*Amblyomma*
9760	All together	ResNet-50	todos aureolatum (22).jpg	fold5	1	*Amblyomma*
9761	All together	ResNet-50	todos aureolatum (25).jpg	fold5	1	*Amblyomma*
9762	All together	ResNet-50	todos aureolatum (30).jpg	fold5	1	*Amblyomma*
9763	All together	ResNet-50	todos aureolatum (37).jpg	fold5	1	*Amblyomma*
9764	All together	ResNet-50	todos aureolatum (39).jpg	fold5	1	*Amblyomma*
9765	All together	ResNet-50	todos aureolatum (40).jpg	fold5	1	*Amblyomma*
9766	All together	ResNet-50	todos aureolatum (48).jpg	fold5	1	*Amblyomma*
9767	All together	ResNet-50	todos aureolatum (5).jpg	fold5	1	*Amblyomma*
9768	All together	ResNet-50	todos aureolatum (51).jpg	fold5	1	*Amblyomma*
9769	All together	ResNet-50	todos aureolatum (53).jpg	fold5	1	*Amblyomma*
9770	All together	ResNet-50	todos aureolatum (54).jpg	fold5	1	*Amblyomma*
9771	All together	ResNet-50	todos aureolatum (55).jpg	fold5	1	*Amblyomma*
9772	All together	ResNet-50	todos aureolatum (58).jpg	fold5	1	*Amblyomma*
9773	All together	ResNet-50	todos aureolatum (62).jpg	fold5	1	*Amblyomma*
9774	All together	ResNet-50	todos aureolatum (65).jpg	fold5	1	*Amblyomma*
9775	All together	ResNet-50	todos aureolatum (75).jpg	fold5	1	*Amblyomma*
9776	All together	ResNet-50	todos aureolatum (78).jpg	fold5	1	*Amblyomma*
9777	All together	ResNet-50	todos aureolatum (81).jpg	fold5	1	*Amblyomma*
9778	All together	ResNet-50	todos aureolatum (87).jpg	fold5	1	*Amblyomma*
9779	All together	ResNet-50	todos cajennense (104).jpg	fold5	2	*Amblyomma*
9780	All together	ResNet-50	todos cajennense (105).jpg	fold5	2	*Amblyomma*

9781	All together	ResNet-50	todos cajennense (108).jpg	fold5	2	*Amblyomma*
9782	All together	ResNet-50	todos cajennense (132).jpg	fold5	2	*Amblyomma*
9783	All together	ResNet-50	todos cajennense (14).jpg	fold5	2	*Amblyomma*
9784	All together	ResNet-50	todos cajennense (140).jpg	fold5	2	*Amblyomma*
9785	All together	ResNet-50	todos cajennense (141).jpg	fold5	2	*Amblyomma*
9786	All together	ResNet-50	todos cajennense (145).jpg	fold5	2	*Amblyomma*
9787	All together	ResNet-50	todos cajennense (147).jpg	fold5	2	*Amblyomma*
9788	All together	ResNet-50	todos cajennense (15).jpg	fold5	2	*Amblyomma*
9789	All together	ResNet-50	todos cajennense (150).jpg	fold5	2	*Amblyomma*
9790	All together	ResNet-50	todos cajennense (153).jpg	fold5	2	*Amblyomma*
9791	All together	ResNet-50	todos cajennense (161).jpg	fold5	2	*Amblyomma*
9792	All together	ResNet-50	todos cajennense (19).jpg	fold5	2	*Amblyomma*
9793	All together	ResNet-50	todos cajennense (2).jpg	fold5	2	*Amblyomma*
9794	All together	ResNet-50	todos cajennense (20).jpg	fold5	2	*Amblyomma*
9795	All together	ResNet-50	todos cajennense (4).jpg	fold5	2	*Amblyomma*
9796	All together	ResNet-50	todos cajennense (41).jpg	fold5	2	*Amblyomma*
9797	All together	ResNet-50	todos cajennense (42).jpg	fold5	2	*Amblyomma*
9798	All together	ResNet-50	todos cajennense (53).jpg	fold5	2	*Amblyomma*
9799	All together	ResNet-50	todos cajennense (55).jpg	fold5	2	*Amblyomma*
9800	All together	ResNet-50	todos cajennense (62).jpg	fold5	2	*Amblyomma*
9801	All together	ResNet-50	todos cajennense (63).jpg	fold5	2	*Amblyomma*
9802	All together	ResNet-50	todos cajennense (73).jpg	fold5	2	*Amblyomma*
9803	All together	ResNet-50	todos cajennense (75).jpg	fold5	2	*Amblyomma*
9804	All together	ResNet-50	todos cajennense (77).jpg	fold5	2	*Amblyomma*
9805	All together	ResNet-50	todos cajennense (79).jpg	fold5	2	*Amblyomma*
9806	All together	ResNet-50	todos cajennense (8).jpg	fold5	2	*Amblyomma*
9807	All together	ResNet-50	todos cajennense (83).jpg	fold5	2	*Amblyomma*
9808	All together	ResNet-50	todos cajennense (86).jpg	fold5	2	*Amblyomma*
9809	All together	ResNet-50	todos cajennense (9).jpg	fold5	2	*Amblyomma*
9810	All together	ResNet-50	todos cajennense (90).jpg	fold5	2	*Amblyomma*
9811	All together	ResNet-50	todos cajennense (93).jpg	fold5	2	*Amblyomma*
9812	All together	ResNet-50	todos dubitatum (10).jpg	fold5	3	*Amblyomma*
9813	All together	ResNet-50	todos dubitatum (100).jpg	fold5	3	*Amblyomma*
9814	All together	ResNet-50	todos dubitatum (102).jpg	fold5	3	*Amblyomma*
9815	All together	ResNet-50	todos dubitatum (106).jpg	fold5	3	*Amblyomma*
9816	All together	ResNet-50	todos dubitatum (16).jpg	fold5	3	*Amblyomma*
9817	All together	ResNet-50	todos dubitatum (17).jpg	fold5	3	*Amblyomma*
9818	All together	ResNet-50	todos dubitatum (21).jpg	fold5	3	*Amblyomma*
9819	All together	ResNet-50	todos dubitatum (29).jpg	fold5	3	*Amblyomma*
9820	All together	ResNet-50	todos dubitatum (30).jpg	fold5	3	*Amblyomma*
9821	All together	ResNet-50	todos dubitatum (31).jpg	fold5	3	*Amblyomma*
9822	All together	ResNet-50	todos dubitatum (38).jpg	fold5	3	*Amblyomma*
9823	All together	ResNet-50	todos dubitatum (51).jpg	fold5	3	*Amblyomma*
9824	All together	ResNet-50	todos dubitatum (54).jpg	fold5	3	*Amblyomma*
9825	All together	ResNet-50	todos dubitatum (61).jpg	fold5	3	*Amblyomma*
9826	All together	ResNet-50	todos dubitatum (68).jpg	fold5	3	*Amblyomma*
9827	All together	ResNet-50	todos dubitatum (80).jpg	fold5	3	*Amblyomma*
9828	All together	ResNet-50	todos dubitatum (86).jpg	fold5	3	*Amblyomma*
9829	All together	ResNet-50	todos dubitatum (89).jpg	fold5	3	*Amblyomma*
9830	All together	ResNet-50	todos dubitatum (91).jpg	fold5	3	*Amblyomma*
9831	All together	ResNet-50	todos dubitatum (97).jpg	fold5	3	*Amblyomma*
9832	All together	ResNet-50	todos dubitatum (98).jpg	fold5	3	*Amblyomma*
9833	All together	ResNet-50	todos ovale (102).jpg	fold5	4	*Amblyomma*
9834	All together	ResNet-50	todos ovale (114).jpg	fold5	4	*Amblyomma*
9835	All together	ResNet-50	todos ovale (117).jpg	fold5	4	*Amblyomma*
9836	All together	ResNet-50	todos ovale (119).jpg	fold5	4	*Amblyomma*
9837	All together	ResNet-50	todos ovale (12).jpg	fold5	4	*Amblyomma*
9838	All together	ResNet-50	todos ovale (14).jpg	fold5	4	*Amblyomma*
9839	All together	ResNet-50	todos ovale (18).jpg	fold5	4	*Amblyomma*
9840	All together	ResNet-50	todos ovale (31).jpg	fold5	4	*Amblyomma*
9841	All together	ResNet-50	todos ovale (35).jpg	fold5	4	*Amblyomma*
9842	All together	ResNet-50	todos ovale (45).jpg	fold5	4	*Amblyomma*
9843	All together	ResNet-50	todos ovale (48).jpg	fold5	4	*Amblyomma*
9844	All together	ResNet-50	todos ovale (49).jpg	fold5	4	*Amblyomma*
9845	All together	ResNet-50	todos ovale (5).jpg	fold5	4	*Amblyomma*
9846	All together	ResNet-50	todos ovale (50).jpg	fold5	4	*Amblyomma*
9847	All together	ResNet-50	todos ovale (53).jpg	fold5	4	*Amblyomma*

9848	All together	ResNet-50	todos ovale (59).jpg	fold5	4	*Amblyomma*
9849	All together	ResNet-50	todos ovale (61).jpg	fold5	4	*Amblyomma*
9850	All together	ResNet-50	todos ovale (66).jpg	fold5	4	*Amblyomma*
9851	All together	ResNet-50	todos ovale (73).jpg	fold5	4	*Amblyomma*
9852	All together	ResNet-50	todos ovale (75).JPG	fold5	4	*Amblyomma*
9853	All together	ResNet-50	todos ovale (77).jpg	fold5	4	*Amblyomma*
9854	All together	ResNet-50	todos ovale (79).jpg	fold5	4	*Amblyomma*
9855	All together	ResNet-50	todos ovale (89).jpg	fold5	4	*Amblyomma*
9856	All together	ResNet-50	todos ovale (94).jpg	fold5	4	*Amblyomma*
9857	All together	ResNet-50	todos sculptum (1).jpg	fold5	5	*Amblyomma*
9858	All together	ResNet-50	todos sculptum (101).JPG	fold5	5	*Amblyomma*
9859	All together	ResNet-50	todos sculptum (111).JPG	fold5	5	*Amblyomma*
9860	All together	ResNet-50	todos sculptum (131).JPG	fold5	5	*Amblyomma*
9861	All together	ResNet-50	todos sculptum (137).JPG	fold5	5	*Amblyomma*
9862	All together	ResNet-50	todos sculptum (144).JPG	fold5	5	*Amblyomma*
9863	All together	ResNet-50	todos sculptum (145).jpg	fold5	5	*Amblyomma*
9864	All together	ResNet-50	todos sculptum (146).jpg	fold5	5	*Amblyomma*
9865	All together	ResNet-50	todos sculptum (149).jpg	fold5	5	*Amblyomma*
9866	All together	ResNet-50	todos sculptum (150).jpg	fold5	5	*Amblyomma*
9867	All together	ResNet-50	todos sculptum (151).jpg	fold5	5	*Amblyomma*
9868	All together	ResNet-50	todos sculptum (2).jpg	fold5	5	*Amblyomma*
9869	All together	ResNet-50	todos sculptum (34).JPG	fold5	5	*Amblyomma*
9870	All together	ResNet-50	todos sculptum (43).JPG	fold5	5	*Amblyomma*
9871	All together	ResNet-50	todos sculptum (44).JPG	fold5	5	*Amblyomma*
9872	All together	ResNet-50	todos sculptum (50).JPG	fold5	5	*Amblyomma*
9873	All together	ResNet-50	todos sculptum (53).JPG	fold5	5	*Amblyomma*
9874	All together	ResNet-50	todos sculptum (56).jpg	fold5	5	*Amblyomma*
9875	All together	ResNet-50	todos sculptum (58).jpg	fold5	5	*Amblyomma*
9876	All together	ResNet-50	todos sculptum (59).jpg	fold5	5	*Amblyomma*
9877	All together	ResNet-50	todos sculptum (6).jpg	fold5	5	*Amblyomma*
9878	All together	ResNet-50	todos sculptum (67).jpg	fold5	5	*Amblyomma*
9879	All together	ResNet-50	todos sculptum (77).JPG	fold5	5	*Amblyomma*
9880	All together	ResNet-50	todos sculptum (78).JPG	fold5	5	*Amblyomma*
9881	All together	ResNet-50	todos sculptum (79).JPG	fold5	5	*Amblyomma*
9882	All together	ResNet-50	todos sculptum (8).jpg	fold5	5	*Amblyomma*
9883	All together	ResNet-50	todos sculptum (82).JPG	fold5	5	*Amblyomma*
9884	All together	ResNet-50	todos sculptum (86).JPG	fold5	5	*Amblyomma*
9885	All together	ResNet-50	todos sculptum (92).JPG	fold5	5	*Amblyomma*
9886	All together	ResNet-50	todos sculptum (94).JPG	fold5	5	*Amblyomma*
9887	All together	ResNet-50	todos sculptum (95).JPG	fold5	5	*Amblyomma*
9888	All together	ResNet-50	todos triste (10).jpg	fold5	6	*Amblyomma*
9889	All together	ResNet-50	todos triste (100).jpg	fold5	6	*Amblyomma*
9890	All together	ResNet-50	todos triste (108).jpg	fold5	6	*Amblyomma*
9891	All together	ResNet-50	todos triste (111).jpg	fold5	6	*Amblyomma*
9892	All together	ResNet-50	todos triste (112).jpg	fold5	6	*Amblyomma*
9893	All together	ResNet-50	todos triste (117).jpg	fold5	6	*Amblyomma*
9894	All together	ResNet-50	todos triste (119).jpg	fold5	6	*Amblyomma*
9895	All together	ResNet-50	todos triste (12).jpg	fold5	6	*Amblyomma*
9896	All together	ResNet-50	todos triste (124).jpg	fold5	6	*Amblyomma*
9897	All together	ResNet-50	todos triste (138).jpg	fold5	6	*Amblyomma*
9898	All together	ResNet-50	todos triste (143).jpg	fold5	6	*Amblyomma*
9899	All together	ResNet-50	todos triste (150).jpg	fold5	6	*Amblyomma*
9900	All together	ResNet-50	todos triste (16).jpg	fold5	6	*Amblyomma*
9901	All together	ResNet-50	todos triste (19).jpg	fold5	6	*Amblyomma*
9902	All together	ResNet-50	todos triste (25).jpg	fold5	6	*Amblyomma*
9903	All together	ResNet-50	todos triste (30).jpg	fold5	6	*Amblyomma*
9904	All together	ResNet-50	todos triste (31).jpg	fold5	6	*Amblyomma*
9905	All together	ResNet-50	todos triste (34).jpg	fold5	6	*Amblyomma*
9906	All together	ResNet-50	todos triste (39).jpg	fold5	6	*Amblyomma*
9907	All together	ResNet-50	todos triste (4).jpg	fold5	6	*Amblyomma*
9908	All together	ResNet-50	todos triste (62).jpg	fold5	6	*Amblyomma*
9909	All together	ResNet-50	todos triste (63).jpg	fold5	6	*Amblyomma*
9910	All together	ResNet-50	todos triste (67).jpg	fold5	6	*Amblyomma*
9911	All together	ResNet-50	todos triste (71).jpg	fold5	6	*Amblyomma*
9912	All together	ResNet-50	todos triste (72).jpg	fold5	6	*Amblyomma*
9913	All together	ResNet-50	todos triste (75).jpg	fold5	6	*Amblyomma*
9914	All together	ResNet-50	todos triste (80).jpg	fold5	6	*Amblyomma*

9915	All together	ResNet-50	todos triste (82).jpg	fold5	6 *Amblyomma*
9916	All together	ResNet-50	todos triste (89).jpg	fold5	6 *Amblyomma*
9917	All together	ResNet-50	todos triste (91).jpg	fold5	6 *Amblyomma*
9918	All together	ResNet-50	todos triste (98).jpg	fold5	6 *Amblyomma*

/ three algorithms using cross-validation.

Species	Prediction	Out	TRUTH	Algo
Amblyomma aureolatum	*Amblyomma aureolatum*	1	1	1
Amblyomma aureolatum	*Amblyomma aureolatum*	1	1	1
Amblyomma aureolatum	*Amblyomma aureolatum*	1	1	1
Amblyomma aureolatum	*Amblyomma aureolatum*	1	1	1
Amblyomma aureolatum	*Amblyomma aureolatum*	1	1	1
Amblyomma aureolatum	*Amblyomma aureolatum*	1	1	1
Amblyomma aureolatum	*Amblyomma aureolatum*	1	1	1
Amblyomma aureolatum	*Amblyomma aureolatum*	1	1	1
Amblyomma aureolatum	*Amblyomma aureolatum*	1	1	1
Amblyomma aureolatum	*Amblyomma sculptum*	0	1	5
Amblyomma aureolatum	*Amblyomma aureolatum*	1	1	1
Amblyomma cajennense	*Amblyomma cajennense*	1	2	2
Amblyomma cajennense	*Amblyomma sculptum*	0	2	5
Amblyomma cajennense	*Amblyomma cajennense*	1	2	2
Amblyomma cajennense	*Amblyomma cajennense*	1	2	2
Amblyomma cajennense	*Amblyomma cajennense*	1	2	2
Amblyomma cajennense	*Amblyomma cajennense*	1	2	2
Amblyomma cajennense	*Amblyomma cajennense*	1	2	2
Amblyomma cajennense	*Amblyomma cajennense*	1	2	2
Amblyomma cajennense	*Amblyomma cajennense*	1	2	2
Amblyomma cajennense	*Amblyomma cajennense*	1	2	2
Amblyomma cajennense	*Amblyomma cajennense*	1	2	2
Amblyomma cajennense	*Amblyomma cajennense*	1	2	2
Amblyomma cajennense	*Amblyomma cajennense*	1	2	2
Amblyomma cajennense	*Amblyomma aureolatum*	0	2	1
Amblyomma cajennense	*Amblyomma cajennense*	1	2	2
Amblyomma cajennense	*Amblyomma cajennense*	1	2	2
Amblyomma cajennense	*Amblyomma cajennense*	1	2	2
Amblyomma dubitatum	*Amblyomma dubitatum*	1	3	3
Amblyomma dubitatum	*Amblyomma ovale*	0	3	4
Amblyomma dubitatum	*Amblyomma dubitatum*	1	3	3
Amblyomma dubitatum	*Amblyomma aureolatum*	0	3	1
Amblyomma dubitatum	*Amblyomma dubitatum*	1	3	3
Amblyomma dubitatum	*Amblyomma dubitatum*	1	3	3
Amblyomma dubitatum	*Amblyomma dubitatum*	1	3	3
Amblyomma ovale	*Amblyomma ovale*	1	4	4
Amblyomma ovale	*Amblyomma ovale*	1	4	4
Amblyomma ovale	*Amblyomma ovale*	1	4	4
Amblyomma ovale	*Amblyomma ovale*	1	4	4
Amblyomma ovale	*Amblyomma ovale*	1	4	4
Amblyomma ovale	*Amblyomma ovale*	1	4	4
Amblyomma ovale	*Amblyomma ovale*	1	4	4
Amblyomma ovale	*Amblyomma ovale*	1	4	4
Amblyomma ovale	*Amblyomma ovale*	1	4	4
Amblyomma ovale	*Amblyomma ovale*	1	4	4
Amblyomma ovale	*Amblyomma ovale*	1	4	4
Amblyomma ovale	*Amblyomma ovale*	1	4	4
Amblyomma ovale	*Amblyomma ovale*	1	4	4
Amblyomma ovale	*Amblyomma ovale*	1	4	4
Amblyomma ovale	*Amblyomma ovale*	1	4	4
Amblyomma ovale	*Amblyomma ovale*	1	4	4
Amblyomma sculptum	*Amblyomma sculptum*	1	5	5
Amblyomma sculptum	*Amblyomma sculptum*	1	5	5
Amblyomma sculptum	*Amblyomma sculptum*	1	5	5
Amblyomma sculptum	*Amblyomma sculptum*	1	5	5
Amblyomma sculptum	*Amblyomma sculptum*	1	5	5
Amblyomma sculptum	*Amblyomma sculptum*	1	5	5
Amblyomma sculptum	*Amblyomma dubitatum*	0	5	3
Amblyomma sculptum	*Amblyomma sculptum*	1	5	5
Amblyomma sculptum	*Amblyomma dubitatum*	0	5	3
Amblyomma sculptum	*Amblyomma ovale*	0	5	4
Amblyomma sculptum	*Amblyomma sculptum*	1	5	5
Amblyomma sculptum	*Amblyomma dubitatum*	0	5	3
Amblyomma sculptum	*Amblyomma sculptum*	1	5	5
Amblyomma triste	*Amblyomma aureolatum*	0	6	1

Amblyomma triste	*Amblyomma triste*	1	6	6
Amblyomma triste	*Amblyomma aureolatum*	0	6	1
Amblyomma triste	*Amblyomma triste*	1	6	6
Amblyomma triste	*Amblyomma triste*	1	6	6
Amblyomma triste	*Amblyomma triste*	1	6	6
Amblyomma triste	*Amblyomma triste*	1	6	6
Amblyomma triste	*Amblyomma triste*	1	6	6
Amblyomma triste	*Amblyomma dubitatum*	0	6	3
Amblyomma triste	*Amblyomma dubitatum*	0	6	3
Amblyomma triste	*Amblyomma triste*	1	6	6
Amblyomma aureolatum	*Amblyomma cajennense*	0	1	2
Amblyomma aureolatum	*Amblyomma aureolatum*	1	1	1
Amblyomma aureolatum	*Amblyomma aureolatum*	1	1	1
Amblyomma aureolatum	*Amblyomma aureolatum*	1	1	1
Amblyomma aureolatum	*Amblyomma aureolatum*	1	1	1
Amblyomma aureolatum	*Amblyomma aureolatum*	1	1	1
Amblyomma aureolatum	*Amblyomma aureolatum*	1	1	1
Amblyomma aureolatum	*Amblyomma aureolatum*	1	1	1
Amblyomma aureolatum	*Amblyomma aureolatum*	1	1	1
Amblyomma aureolatum	*Amblyomma aureolatum*	1	1	1
Amblyomma aureolatum	*Amblyomma aureolatum*	1	1	1
Amblyomma aureolatum	*Amblyomma aureolatum*	1	1	1
Amblyomma cajennense	*Amblyomma cajennense*	1	2	2
Amblyomma cajennense	*Amblyomma cajennense*	1	2	2
Amblyomma cajennense	*Amblyomma cajennense*	1	2	2
Amblyomma cajennense	*Amblyomma cajennense*	1	2	2
Amblyomma cajennense	*Amblyomma cajennense*	1	2	2
Amblyomma cajennense	*Amblyomma cajennense*	1	2	2
Amblyomma cajennense	*Amblyomma cajennense*	1	2	2
Amblyomma cajennense	*Amblyomma cajennense*	1	2	2
Amblyomma cajennense	*Amblyomma cajennense*	1	2	2
Amblyomma cajennense	*Amblyomma cajennense*	1	2	2
Amblyomma cajennense	*Amblyomma cajennense*	1	2	2
Amblyomma cajennense	*Amblyomma cajennense*	1	2	2
Amblyomma cajennense	*Amblyomma cajennense*	1	2	2
Amblyomma cajennense	*Amblyomma cajennense*	1	2	2
Amblyomma cajennense	*Amblyomma cajennense*	1	2	2
Amblyomma cajennense	*Amblyomma cajennense*	1	2	2
Amblyomma cajennense	*Amblyomma cajennense*	1	2	2
Amblyomma dubitatum	*Amblyomma dubitatum*	1	3	3
Amblyomma dubitatum	*Amblyomma dubitatum*	1	3	3
Amblyomma dubitatum	*Amblyomma dubitatum*	1	3	3
Amblyomma dubitatum	*Amblyomma dubitatum*	1	3	3
Amblyomma dubitatum	*Amblyomma dubitatum*	1	3	3
Amblyomma dubitatum	*Amblyomma triste*	0	3	6
Amblyomma dubitatum	*Amblyomma dubitatum*	1	3	3
Amblyomma ovale	*Amblyomma ovale*	1	4	4
Amblyomma ovale	*Amblyomma ovale*	1	4	4
Amblyomma ovale	*Amblyomma ovale*	1	4	4
Amblyomma ovale	*Amblyomma ovale*	1	4	4
Amblyomma ovale	*Amblyomma ovale*	1	4	4
Amblyomma ovale	*Amblyomma ovale*	1	4	4
Amblyomma ovale	*Amblyomma ovale*	1	4	4
Amblyomma ovale	*Amblyomma ovale*	1	4	4
Amblyomma ovale	*Amblyomma ovale*	1	4	4
Amblyomma ovale	*Amblyomma ovale*	1	4	4
Amblyomma ovale	*Amblyomma ovale*	1	4	4
Amblyomma ovale	*Amblyomma ovale*	1	4	4
Amblyomma ovale	*Amblyomma ovale*	1	4	4
Amblyomma ovale	*Amblyomma ovale*	1	4	4
Amblyomma ovale	*Amblyomma ovale*	1	4	4
Amblyomma ovale	*Amblyomma ovale*	1	4	4
Amblyomma sculptum	*Amblyomma sculptum*	1	5	5
Amblyomma sculptum	*Amblyomma sculptum*	1	5	5
Amblyomma sculptum	*Amblyomma sculptum*	1	5	5
Amblyomma sculptum	*Amblyomma sculptum*	1	5	5
Amblyomma sculptum	*Amblyomma sculptum*	1	5	5

Amblyomma sculptum	*Amblyomma sculptum*	1	5	5
Amblyomma sculptum	*Amblyomma sculptum*	1	5	5
Amblyomma sculptum	*Amblyomma sculptum*	1	5	5
Amblyomma sculptum	*Amblyomma sculptum*	1	5	5
Amblyomma sculptum	*Amblyomma sculptum*	1	5	5
Amblyomma sculptum	*Amblyomma sculptum*	1	5	5
Amblyomma sculptum	*Amblyomma ovale*	0	5	4
Amblyomma triste	*Amblyomma triste*	1	6	6
Amblyomma triste	*Amblyomma triste*	1	6	6
Amblyomma triste	*Amblyomma triste*	1	6	6
Amblyomma triste	*Amblyomma triste*	1	6	6
Amblyomma triste	*Amblyomma triste*	1	6	6
Amblyomma triste	*Amblyomma triste*	1	6	6
Amblyomma triste	*Amblyomma triste*	1	6	6
Amblyomma triste	*Amblyomma triste*	1	6	6
Amblyomma triste	*Amblyomma triste*	1	6	6
Amblyomma triste	*Amblyomma triste*	1	6	6
Amblyomma aureolatum	*Amblyomma aureolatum*	1	1	1
Amblyomma aureolatum	*Amblyomma ovale*	0	1	4
Amblyomma aureolatum	*Amblyomma ovale*	0	1	4
Amblyomma aureolatum	*Amblyomma aureolatum*	1	1	1
Amblyomma aureolatum	*Amblyomma aureolatum*	1	1	1
Amblyomma aureolatum	*Amblyomma aureolatum*	1	1	1
Amblyomma aureolatum	*Amblyomma aureolatum*	1	1	1
Amblyomma aureolatum	*Amblyomma aureolatum*	1	1	1
Amblyomma aureolatum	*Amblyomma aureolatum*	1	1	1
Amblyomma aureolatum	*Amblyomma aureolatum*	1	1	1
Amblyomma aureolatum	*Amblyomma aureolatum*	1	1	1
Amblyomma cajennense	*Amblyomma cajennense*	1	2	2
Amblyomma cajennense	*Amblyomma cajennense*	1	2	2
Amblyomma cajennense	*Amblyomma cajennense*	1	2	2
Amblyomma cajennense	*Amblyomma ovale*	0	2	4
Amblyomma cajennense	*Amblyomma cajennense*	1	2	2
Amblyomma cajennense	*Amblyomma cajennense*	1	2	2
Amblyomma cajennense	*Amblyomma cajennense*	1	2	2
Amblyomma cajennense	*Amblyomma cajennense*	1	2	2
Amblyomma cajennense	*Amblyomma cajennense*	1	2	2
Amblyomma cajennense	*Amblyomma cajennense*	1	2	2
Amblyomma cajennense	*Amblyomma cajennense*	1	2	2
Amblyomma cajennense	*Amblyomma cajennense*	1	2	2
Amblyomma cajennense	*Amblyomma cajennense*	1	2	2
Amblyomma cajennense	*Amblyomma cajennense*	1	2	2
Amblyomma cajennense	*Amblyomma cajennense*	1	2	2
Amblyomma cajennense	*Amblyomma cajennense*	1	2	2
Amblyomma dubitatum	*Amblyomma dubitatum*	1	3	3
Amblyomma dubitatum	*Amblyomma dubitatum*	1	3	3
Amblyomma dubitatum	*Amblyomma dubitatum*	1	3	3
Amblyomma dubitatum	*Amblyomma dubitatum*	1	3	3
Amblyomma dubitatum	*Amblyomma cajennense*	0	3	2
Amblyomma dubitatum	*Amblyomma ovale*	0	3	4
Amblyomma dubitatum	*Amblyomma dubitatum*	1	3	3
Amblyomma ovale	*Amblyomma ovale*	1	4	4
Amblyomma ovale	*Amblyomma ovale*	1	4	4
Amblyomma ovale	*Amblyomma ovale*	1	4	4
Amblyomma ovale	*Amblyomma ovale*	1	4	4
Amblyomma ovale	*Amblyomma ovale*	1	4	4
Amblyomma ovale	*Amblyomma ovale*	1	4	4
Amblyomma ovale	*Amblyomma ovale*	1	4	4
Amblyomma ovale	*Amblyomma ovale*	1	4	4
Amblyomma ovale	*Amblyomma ovale*	1	4	4
Amblyomma ovale	*Amblyomma ovale*	1	4	4
Amblyomma ovale	*Amblyomma aureolatum*	0	4	1
Amblyomma ovale	*Amblyomma ovale*	1	4	4
Amblyomma ovale	*Amblyomma ovale*	1	4	4
Amblyomma ovale	*Amblyomma ovale*	1	4	4
Amblyomma ovale	*Amblyomma ovale*	1	4	4
Amblyomma sculptum	*Amblyomma sculptum*	1	5	5

Amblyomma sculptum	*Amblyomma sculptum*	1	5	5
Amblyomma sculptum	*Amblyomma sculptum*	1	5	5
Amblyomma sculptum	*Amblyomma sculptum*	1	5	5
Amblyomma sculptum	*Amblyomma sculptum*	1	5	5
Amblyomma sculptum	*Amblyomma sculptum*	1	5	5
Amblyomma sculptum	*Amblyomma sculptum*	1	5	5
Amblyomma sculptum	*Amblyomma sculptum*	1	5	5
Amblyomma sculptum	*Amblyomma sculptum*	1	5	5
Amblyomma sculptum	*Amblyomma sculptum*	1	5	5
Amblyomma sculptum	*Amblyomma sculptum*	1	5	5
Amblyomma sculptum	*Amblyomma sculptum*	1	5	5
Amblyomma sculptum	*Amblyomma sculptum*	1	5	5
Amblyomma triste	*Amblyomma sculptum*	0	6	5
Amblyomma triste	*Amblyomma ovale*	0	6	4
Amblyomma triste	*Amblyomma triste*	1	6	6
Amblyomma triste	*Amblyomma sculptum*	0	6	5
Amblyomma triste	*Amblyomma triste*	1	6	6
Amblyomma triste	*Amblyomma triste*	1	6	6
Amblyomma triste	*Amblyomma triste*	1	6	6
Amblyomma triste	*Amblyomma triste*	1	6	6
Amblyomma triste	*Amblyomma triste*	1	6	6
Amblyomma triste	*Amblyomma triste*	1	6	6
Amblyomma aureolatum	*Amblyomma aureolatum*	1	1	1
Amblyomma aureolatum	*Amblyomma aureolatum*	1	1	1
Amblyomma aureolatum	*Amblyomma aureolatum*	1	1	1
Amblyomma aureolatum	*Amblyomma aureolatum*	1	1	1
Amblyomma aureolatum	*Amblyomma aureolatum*	1	1	1
Amblyomma aureolatum	*Amblyomma aureolatum*	1	1	1
Amblyomma aureolatum	*Amblyomma aureolatum*	1	1	1
Amblyomma aureolatum	*Amblyomma aureolatum*	1	1	1
Amblyomma aureolatum	*Amblyomma aureolatum*	1	1	1
Amblyomma aureolatum	*Amblyomma aureolatum*	1	1	1
Amblyomma aureolatum	*Amblyomma aureolatum*	1	1	1
Amblyomma aureolatum	*Amblyomma aureolatum*	1	1	1
Amblyomma cajennense	*Amblyomma cajennense*	1	2	2
Amblyomma cajennense	*Amblyomma cajennense*	1	2	2
Amblyomma cajennense	*Amblyomma cajennense*	1	2	2
Amblyomma cajennense	*Amblyomma cajennense*	1	2	2
Amblyomma cajennense	*Amblyomma cajennense*	1	2	2
Amblyomma cajennense	*Amblyomma cajennense*	1	2	2
Amblyomma cajennense	*Amblyomma cajennense*	1	2	2
Amblyomma cajennense	*Amblyomma cajennense*	1	2	2
Amblyomma cajennense	*Amblyomma cajennense*	1	2	2
Amblyomma cajennense	*Amblyomma cajennense*	1	2	2
Amblyomma cajennense	*Amblyomma cajennense*	1	2	2
Amblyomma cajennense	*Amblyomma cajennense*	1	2	2
Amblyomma cajennense	*Amblyomma cajennense*	1	2	2
Amblyomma cajennense	*Amblyomma cajennense*	1	2	2
Amblyomma cajennense	*Amblyomma cajennense*	1	2	2
Amblyomma cajennense	*Amblyomma cajennense*	1	2	2
Amblyomma dubitatum	*Amblyomma dubitatum*	1	3	3
Amblyomma dubitatum	*Amblyomma aureolatum*	0	3	1
Amblyomma dubitatum	*Amblyomma triste*	0	3	6
Amblyomma dubitatum	*Amblyomma dubitatum*	1	3	3
Amblyomma dubitatum	*Amblyomma sculptum*	0	3	5
Amblyomma dubitatum	*Amblyomma dubitatum*	1	3	3
Amblyomma ovale	*Amblyomma ovale*	1	4	4
Amblyomma ovale	*Amblyomma ovale*	1	4	4
Amblyomma ovale	*Amblyomma ovale*	1	4	4
Amblyomma ovale	*Amblyomma cajennense*	0	4	2
Amblyomma ovale	*Amblyomma ovale*	1	4	4
Amblyomma ovale	*Amblyomma ovale*	1	4	4
Amblyomma ovale	*Amblyomma ovale*	1	4	4
Amblyomma ovale	*Amblyomma ovale*	1	4	4
Amblyomma ovale	*Amblyomma ovale*	1	4	4
Amblyomma ovale	*Amblyomma ovale*	1	4	4
Amblyomma ovale	*Amblyomma aureolatum*	0	4	1

Amblyomma ovale	*Amblyomma ovale*	1	4	4
Amblyomma ovale	*Amblyomma ovale*	1	4	4
Amblyomma ovale	*Amblyomma ovale*	1	4	4
Amblyomma ovale	*Amblyomma ovale*	1	4	4
Amblyomma ovale	*Amblyomma ovale*	1	4	4
Amblyomma sculptum	*Amblyomma sculptum*	1	5	5
Amblyomma sculptum	*Amblyomma sculptum*	1	5	5
Amblyomma sculptum	*Amblyomma sculptum*	1	5	5
Amblyomma sculptum	*Amblyomma sculptum*	1	5	5
Amblyomma sculptum	*Amblyomma sculptum*	1	5	5
Amblyomma sculptum	*Amblyomma sculptum*	1	5	5
Amblyomma sculptum	*Amblyomma sculptum*	1	5	5
Amblyomma sculptum	*Amblyomma sculptum*	1	5	5
Amblyomma sculptum	*Amblyomma sculptum*	1	5	5
Amblyomma sculptum	*Amblyomma sculptum*	1	5	5
Amblyomma sculptum	*Amblyomma sculptum*	1	5	5
Amblyomma sculptum	*Amblyomma aureolatum*	0	5	1
Amblyomma sculptum	*Amblyomma dubitatum*	0	5	3
Amblyomma triste	*Amblyomma triste*	1	6	6
Amblyomma triste	*Amblyomma triste*	1	6	6
Amblyomma triste	*Amblyomma triste*	1	6	6
Amblyomma triste	*Amblyomma triste*	1	6	6
Amblyomma triste	*Amblyomma triste*	1	6	6
Amblyomma triste	*Amblyomma triste*	1	6	6
Amblyomma triste	*Amblyomma triste*	1	6	6
Amblyomma triste	*Amblyomma triste*	1	6	6
Amblyomma triste	*Amblyomma triste*	1	6	6
Amblyomma triste	*Amblyomma triste*	1	6	6
Amblyomma aureolatum	*Amblyomma aureolatum*	1	1	1
Amblyomma aureolatum	*Amblyomma aureolatum*	1	1	1
Amblyomma aureolatum	*Amblyomma aureolatum*	1	1	1
Amblyomma aureolatum	*Amblyomma aureolatum*	1	1	1
Amblyomma aureolatum	*Amblyomma ovale*	0	1	4
Amblyomma aureolatum	*Amblyomma aureolatum*	1	1	1
Amblyomma aureolatum	*Amblyomma aureolatum*	1	1	1
Amblyomma aureolatum	*Amblyomma aureolatum*	1	1	1
Amblyomma aureolatum	*Amblyomma aureolatum*	1	1	1
Amblyomma aureolatum	*Amblyomma aureolatum*	1	1	1
Amblyomma aureolatum	*Amblyomma triste*	0	1	6
Amblyomma aureolatum	*Amblyomma aureolatum*	1	1	1
Amblyomma cajennense	*Amblyomma cajennense*	1	2	2
Amblyomma cajennense	*Amblyomma cajennense*	1	2	2
Amblyomma cajennense	*Amblyomma cajennense*	1	2	2
Amblyomma cajennense	*Amblyomma cajennense*	1	2	2
Amblyomma cajennense	*Amblyomma cajennense*	1	2	2
Amblyomma cajennense	*Amblyomma cajennense*	1	2	2
Amblyomma cajennense	*Amblyomma cajennense*	1	2	2
Amblyomma cajennense	*Amblyomma cajennense*	1	2	2
Amblyomma cajennense	*Amblyomma cajennense*	1	2	2
Amblyomma cajennense	*Amblyomma cajennense*	1	2	2
Amblyomma cajennense	*Amblyomma cajennense*	1	2	2
Amblyomma cajennense	*Amblyomma cajennense*	1	2	2
Amblyomma cajennense	*Amblyomma cajennense*	1	2	2
Amblyomma cajennense	*Amblyomma cajennense*	1	2	2
Amblyomma cajennense	*Amblyomma cajennense*	1	2	2
Amblyomma cajennense	*Amblyomma cajennense*	1	2	2
Amblyomma dubitatum	*Amblyomma aureolatum*	0	3	1
Amblyomma dubitatum	*Amblyomma aureolatum*	0	3	1
Amblyomma dubitatum	*Amblyomma dubitatum*	1	3	3
Amblyomma dubitatum	*Amblyomma dubitatum*	1	3	3
Amblyomma dubitatum	*Amblyomma ovale*	0	3	4
Amblyomma dubitatum	*Amblyomma dubitatum*	1	3	3
Amblyomma dubitatum	*Amblyomma dubitatum*	1	3	3
Amblyomma ovale	*Amblyomma ovale*	1	4	4
Amblyomma ovale	*Amblyomma ovale*	1	4	4
Amblyomma ovale	*Amblyomma ovale*	1	4	4
Amblyomma ovale	*Amblyomma ovale*	1	4	4

Amblyomma ovale	*Amblyomma ovale*	1	4	4
Amblyomma ovale	*Amblyomma ovale*	1	4	4
Amblyomma ovale	*Amblyomma ovale*	1	4	4
Amblyomma ovale	*Amblyomma ovale*	1	4	4
Amblyomma ovale	*Amblyomma ovale*	1	4	4
Amblyomma ovale	*Amblyomma ovale*	1	4	4
Amblyomma ovale	*Amblyomma ovale*	1	4	4
Amblyomma ovale	*Amblyomma ovale*	1	4	4
Amblyomma ovale	*Amblyomma ovale*	1	4	4
Amblyomma ovale	*Amblyomma ovale*	1	4	4
Amblyomma ovale	*Amblyomma ovale*	1	4	4
Amblyomma ovale	*Amblyomma ovale*	1	4	4
Amblyomma sculptum	*Amblyomma sculptum*	1	5	5
Amblyomma sculptum	*Amblyomma sculptum*	1	5	5
Amblyomma sculptum	*Amblyomma sculptum*	1	5	5
Amblyomma sculptum	*Amblyomma triste*	0	5	6
Amblyomma sculptum	*Amblyomma sculptum*	1	5	5
Amblyomma sculptum	*Amblyomma sculptum*	1	5	5
Amblyomma sculptum	*Amblyomma triste*	0	5	6
Amblyomma sculptum	*Amblyomma sculptum*	1	5	5
Amblyomma sculptum	*Amblyomma sculptum*	1	5	5
Amblyomma sculptum	*Amblyomma sculptum*	1	5	5
Amblyomma sculptum	*Amblyomma sculptum*	1	5	5
Amblyomma sculptum	*Amblyomma aureolatum*	0	5	1
Amblyomma triste	*Amblyomma triste*	1	6	6
Amblyomma triste	*Amblyomma triste*	1	6	6
Amblyomma triste	*Amblyomma triste*	1	6	6
Amblyomma triste	*Amblyomma triste*	1	6	6
Amblyomma triste	*Amblyomma triste*	1	6	6
Amblyomma triste	*Amblyomma triste*	1	6	6
Amblyomma triste	*Amblyomma triste*	1	6	6
Amblyomma triste	*Amblyomma ovale*	0	6	4
Amblyomma triste	*Amblyomma triste*	1	6	6
Amblyomma triste	*Amblyomma triste*	1	6	6
Amblyomma triste	*Amblyomma triste*	1	6	6
Amblyomma aureolatum	*Amblyomma aureolatum*	1	1	1
Amblyomma aureolatum	*Amblyomma aureolatum*	1	1	1
Amblyomma aureolatum	*Amblyomma aureolatum*	1	1	1
Amblyomma aureolatum	*Amblyomma aureolatum*	1	1	1
Amblyomma aureolatum	*Amblyomma aureolatum*	1	1	1
Amblyomma aureolatum	*Amblyomma aureolatum*	1	1	1
Amblyomma aureolatum	*Amblyomma aureolatum*	1	1	1
Amblyomma aureolatum	*Amblyomma aureolatum*	1	1	1
Amblyomma aureolatum	*Amblyomma aureolatum*	1	1	1
Amblyomma aureolatum	*Amblyomma aureolatum*	1	1	1
Amblyomma aureolatum	*Amblyomma aureolatum*	1	1	1
Amblyomma aureolatum	*Amblyomma aureolatum*	1	1	1
Amblyomma cajennense	*Amblyomma cajennense*	1	2	2
Amblyomma cajennense	*Amblyomma cajennense*	1	2	2
Amblyomma cajennense	*Amblyomma cajennense*	1	2	2
Amblyomma cajennense	*Amblyomma cajennense*	1	2	2
Amblyomma cajennense	*Amblyomma cajennense*	1	2	2
Amblyomma cajennense	*Amblyomma cajennense*	1	2	2
Amblyomma cajennense	*Amblyomma cajennense*	1	2	2
Amblyomma cajennense	*Amblyomma cajennense*	1	2	2
Amblyomma cajennense	*Amblyomma cajennense*	1	2	2
Amblyomma cajennense	*Amblyomma cajennense*	1	2	2
Amblyomma cajennense	*Amblyomma dubitatum*	0	2	3
Amblyomma cajennense	*Amblyomma cajennense*	1	2	2
Amblyomma cajennense	*Amblyomma cajennense*	1	2	2
Amblyomma cajennense	*Amblyomma cajennense*	1	2	2
Amblyomma cajennense	*Amblyomma cajennense*	1	2	2
Amblyomma cajennense	*Amblyomma cajennense*	1	2	2
Amblyomma dubitatum	*Amblyomma dubitatum*	1	3	3
Amblyomma dubitatum	*Amblyomma dubitatum*	1	3	3
Amblyomma dubitatum	*Amblyomma dubitatum*	1	3	3
Amblyomma dubitatum	*Amblyomma dubitatum*	1	3	3

Amblyomma dubitatum	*Amblyomma dubitatum*	1	3	3
Amblyomma dubitatum	*Amblyomma dubitatum*	1	3	3
Amblyomma dubitatum	*Amblyomma dubitatum*	1	3	3
Amblyomma dubitatum	*Amblyomma dubitatum*	1	3	3
Amblyomma dubitatum	*Amblyomma dubitatum*	1	3	3
Amblyomma dubitatum	*Amblyomma dubitatum*	1	3	3
Amblyomma dubitatum	*Amblyomma dubitatum*	1	3	3
Amblyomma dubitatum	*Amblyomma dubitatum*	1	3	3
Amblyomma dubitatum	*Amblyomma dubitatum*	1	3	3
Amblyomma dubitatum	*Amblyomma dubitatum*	1	3	3
Amblyomma dubitatum	*Amblyomma dubitatum*	1	3	3
Amblyomma ovale	*Amblyomma ovale*	1	4	4
Amblyomma ovale	*Amblyomma ovale*	1	4	4
Amblyomma ovale	*Amblyomma ovale*	1	4	4
Amblyomma ovale	*Amblyomma ovale*	1	4	4
Amblyomma ovale	*Amblyomma ovale*	1	4	4
Amblyomma ovale	*Amblyomma ovale*	1	4	4
Amblyomma ovale	*Amblyomma ovale*	1	4	4
Amblyomma ovale	*Amblyomma ovale*	1	4	4
Amblyomma sculptum	*Amblyomma sculptum*	1	5	5
Amblyomma sculptum	*Amblyomma sculptum*	1	5	5
Amblyomma sculptum	*Amblyomma sculptum*	1	5	5
Amblyomma sculptum	*Amblyomma sculptum*	1	5	5
Amblyomma sculptum	*Amblyomma sculptum*	1	5	5
Amblyomma sculptum	*Amblyomma sculptum*	1	5	5
Amblyomma sculptum	*Amblyomma sculptum*	1	5	5
Amblyomma sculptum	*Amblyomma sculptum*	1	5	5
Amblyomma sculptum	*Amblyomma sculptum*	1	5	5
Amblyomma sculptum	*Amblyomma sculptum*	1	5	5
Amblyomma sculptum	*Amblyomma sculptum*	1	5	5
Amblyomma sculptum	*Amblyomma sculptum*	1	5	5
Amblyomma sculptum	*Amblyomma sculptum*	1	5	5
Amblyomma sculptum	*Amblyomma sculptum*	1	5	5
Amblyomma sculptum	*Amblyomma sculptum*	1	5	5
Amblyomma sculptum	*Amblyomma sculptum*	1	5	5
Amblyomma sculptum	*Amblyomma dubitatum*	0	5	3
Amblyomma sculptum	*Amblyomma sculptum*	1	5	5
Amblyomma triste	*Amblyomma triste*	1	6	6
Amblyomma triste	*Amblyomma triste*	1	6	6
Amblyomma triste	*Amblyomma triste*	1	6	6
Amblyomma triste	*Amblyomma triste*	1	6	6
Amblyomma triste	*Amblyomma triste*	1	6	6
Amblyomma triste	*Amblyomma triste*	1	6	6
Amblyomma triste	*Amblyomma triste*	1	6	6
Amblyomma triste	*Amblyomma triste*	1	6	6
Amblyomma triste	*Amblyomma triste*	1	6	6
Amblyomma triste	*Amblyomma triste*	1	6	6
Amblyomma triste	*Amblyomma triste*	1	6	6
Amblyomma triste	*Amblyomma triste*	1	6	6
Amblyomma triste	*Amblyomma triste*	1	6	6
Amblyomma triste	*Amblyomma triste*	1	6	6
Amblyomma triste	*Amblyomma triste*	1	6	6
Amblyomma triste	*Amblyomma triste*	1	6	6
Amblyomma triste	*Amblyomma triste*	1	6	6
Amblyomma triste	*Amblyomma triste*	1	6	6
Amblyomma triste	*Amblyomma triste*	1	6	6
Amblyomma triste	*Amblyomma triste*	1	6	6
Amblyomma aureolatum	*Amblyomma aureolatum*	1	1	1
Amblyomma aureolatum	*Amblyomma aureolatum*	1	1	1
Amblyomma aureolatum	*Amblyomma aureolatum*	1	1	1
Amblyomma aureolatum	*Amblyomma aureolatum*	1	1	1
Amblyomma aureolatum	*Amblyomma aureolatum*	1	1	1
Amblyomma aureolatum	*Amblyomma aureolatum*	1	1	1
Amblyomma aureolatum	*Amblyomma aureolatum*	1	1	1
Amblyomma aureolatum	*Amblyomma aureolatum*	1	1	1
Amblyomma aureolatum	*Amblyomma aureolatum*	1	1	1
Amblyomma aureolatum	*Amblyomma aureolatum*	1	1	1

Amblyomma aureolatum	*Amblyomma aureolatum*	1	1	1
Amblyomma aureolatum	*Amblyomma aureolatum*	1	1	1
Amblyomma aureolatum	*Amblyomma aureolatum*	1	1	1
Amblyomma cajennense	*Amblyomma cajennense*	1	2	2
Amblyomma cajennense	*Amblyomma cajennense*	1	2	2
Amblyomma cajennense	*Amblyomma cajennense*	1	2	2
Amblyomma cajennense	*Amblyomma cajennense*	1	2	2
Amblyomma cajennense	*Amblyomma cajennense*	1	2	2
Amblyomma cajennense	*Amblyomma cajennense*	1	2	2
Amblyomma cajennense	*Amblyomma cajennense*	1	2	2
Amblyomma cajennense	*Amblyomma cajennense*	1	2	2
Amblyomma cajennense	*Amblyomma cajennense*	1	2	2
Amblyomma cajennense	*Amblyomma triste*	0	2	6
Amblyomma cajennense	*Amblyomma cajennense*	1	2	2
Amblyomma cajennense	*Amblyomma cajennense*	1	2	2
Amblyomma cajennense	*Amblyomma cajennense*	1	2	2
Amblyomma cajennense	*Amblyomma cajennense*	1	2	2
Amblyomma cajennense	*Amblyomma cajennense*	1	2	2
Amblyomma cajennense	*Amblyomma cajennense*	1	2	2
Amblyomma cajennense	*Amblyomma cajennense*	1	2	2
Amblyomma dubitatum	*Amblyomma cajennense*	0	3	2
Amblyomma dubitatum	*Amblyomma dubitatum*	1	3	3
Amblyomma dubitatum	*Amblyomma dubitatum*	1	3	3
Amblyomma dubitatum	*Amblyomma dubitatum*	1	3	3
Amblyomma dubitatum	*Amblyomma dubitatum*	1	3	3
Amblyomma dubitatum	*Amblyomma dubitatum*	1	3	3
Amblyomma dubitatum	*Amblyomma dubitatum*	1	3	3
Amblyomma dubitatum	*Amblyomma dubitatum*	1	3	3
Amblyomma dubitatum	*Amblyomma sculptum*	0	3	5
Amblyomma dubitatum	*Amblyomma dubitatum*	1	3	3
Amblyomma dubitatum	*Amblyomma dubitatum*	1	3	3
Amblyomma dubitatum	*Amblyomma dubitatum*	1	3	3
Amblyomma dubitatum	*Amblyomma dubitatum*	1	3	3
Amblyomma dubitatum	*Amblyomma dubitatum*	1	3	3
Amblyomma dubitatum	*Amblyomma dubitatum*	1	3	3
Amblyomma ovale	*Amblyomma ovale*	1	4	4
Amblyomma ovale	*Amblyomma cajennense*	0	4	2
Amblyomma ovale	*Amblyomma ovale*	1	4	4
Amblyomma ovale	*Amblyomma triste*	0	4	6
Amblyomma ovale	*Amblyomma ovale*	1	4	4
Amblyomma ovale	*Amblyomma ovale*	1	4	4
Amblyomma ovale	*Amblyomma aureolatum*	0	4	1
Amblyomma ovale	*Amblyomma aureolatum*	0	4	1
Amblyomma ovale	*Amblyomma ovale*	1	4	4
Amblyomma sculptum	*Amblyomma sculptum*	1	5	5
Amblyomma sculptum	*Amblyomma sculptum*	1	5	5
Amblyomma sculptum	*Amblyomma sculptum*	1	5	5
Amblyomma sculptum	*Amblyomma sculptum*	1	5	5
Amblyomma sculptum	*Amblyomma sculptum*	1	5	5
Amblyomma sculptum	*Amblyomma sculptum*	1	5	5
Amblyomma sculptum	*Amblyomma sculptum*	1	5	5
Amblyomma sculptum	*Amblyomma sculptum*	1	5	5
Amblyomma sculptum	*Amblyomma sculptum*	1	5	5
Amblyomma sculptum	*Amblyomma sculptum*	1	5	5
Amblyomma sculptum	*Amblyomma sculptum*	1	5	5
Amblyomma sculptum	*Amblyomma sculptum*	1	5	5
Amblyomma sculptum	*Amblyomma sculptum*	1	5	5
Amblyomma sculptum	*Amblyomma sculptum*	1	5	5
Amblyomma sculptum	*Amblyomma sculptum*	1	5	5
Amblyomma sculptum	*Amblyomma sculptum*	1	5	5
Amblyomma sculptum	*Amblyomma sculptum*	1	5	5
Amblyomma sculptum	*Amblyomma sculptum*	1	5	5
Amblyomma triste	*Amblyomma triste*	1	6	6
Amblyomma triste	*Amblyomma triste*	1	6	6
Amblyomma triste	*Amblyomma triste*	1	6	6
Amblyomma triste	*Amblyomma triste*	1	6	6
Amblyomma triste	*Amblyomma triste*	1	6	6

Amblyomma triste	*Amblyomma triste*	1	6	6
Amblyomma triste	*Amblyomma triste*	1	6	6
Amblyomma triste	*Amblyomma triste*	1	6	6
Amblyomma triste	*Amblyomma triste*	1	6	6
Amblyomma triste	*Amblyomma triste*	1	6	6
Amblyomma triste	*Amblyomma triste*	1	6	6
Amblyomma triste	*Amblyomma triste*	1	6	6
Amblyomma triste	*Amblyomma triste*	1	6	6
Amblyomma triste	*Amblyomma triste*	1	6	6
Amblyomma triste	*Amblyomma triste*	1	6	6
Amblyomma triste	*Amblyomma triste*	1	6	6
Amblyomma triste	*Amblyomma triste*	1	6	6
Amblyomma triste	*Amblyomma triste*	1	6	6
Amblyomma triste	*Amblyomma triste*	1	6	6
Amblyomma triste	*Amblyomma triste*	1	6	6
Amblyomma triste	*Amblyomma triste*	1	6	6
Amblyomma aureolatum	*Amblyomma aureolatum*	1	1	1
Amblyomma aureolatum	*Amblyomma aureolatum*	1	1	1
Amblyomma aureolatum	*Amblyomma aureolatum*	1	1	1
Amblyomma aureolatum	*Amblyomma aureolatum*	1	1	1
Amblyomma aureolatum	*Amblyomma aureolatum*	1	1	1
Amblyomma aureolatum	*Amblyomma aureolatum*	1	1	1
Amblyomma aureolatum	*Amblyomma aureolatum*	1	1	1
Amblyomma aureolatum	*Amblyomma aureolatum*	1	1	1
Amblyomma aureolatum	*Amblyomma aureolatum*	1	1	1
Amblyomma aureolatum	*Amblyomma aureolatum*	1	1	1
Amblyomma aureolatum	*Amblyomma aureolatum*	1	1	1
Amblyomma aureolatum	*Amblyomma aureolatum*	1	1	1
Amblyomma cajennense	*Amblyomma cajennense*	1	2	2
Amblyomma cajennense	*Amblyomma cajennense*	1	2	2
Amblyomma cajennense	*Amblyomma cajennense*	1	2	2
Amblyomma cajennense	*Amblyomma cajennense*	1	2	2
Amblyomma cajennense	*Amblyomma cajennense*	1	2	2
Amblyomma cajennense	*Amblyomma cajennense*	1	2	2
Amblyomma cajennense	*Amblyomma cajennense*	1	2	2
Amblyomma cajennense	*Amblyomma cajennense*	1	2	2
Amblyomma cajennense	*Amblyomma cajennense*	1	2	2
Amblyomma cajennense	*Amblyomma cajennense*	1	2	2
Amblyomma cajennense	*Amblyomma cajennense*	1	2	2
Amblyomma cajennense	*Amblyomma cajennense*	1	2	2
Amblyomma cajennense	*Amblyomma cajennense*	1	2	2
Amblyomma cajennense	*Amblyomma cajennense*	1	2	2
Amblyomma cajennense	*Amblyomma cajennense*	1	2	2
Amblyomma cajennense	*Amblyomma cajennense*	1	2	2
Amblyomma cajennense	*Amblyomma cajennense*	1	2	2
Amblyomma dubitatum	*Amblyomma dubitatum*	1	3	3
Amblyomma dubitatum	*Amblyomma dubitatum*	1	3	3
Amblyomma dubitatum	*Amblyomma dubitatum*	1	3	3
Amblyomma dubitatum	*Amblyomma dubitatum*	1	3	3
Amblyomma dubitatum	*Amblyomma dubitatum*	1	3	3
Amblyomma dubitatum	*Amblyomma dubitatum*	1	3	3
Amblyomma dubitatum	*Amblyomma dubitatum*	1	3	3
Amblyomma dubitatum	*Amblyomma dubitatum*	1	3	3
Amblyomma dubitatum	*Amblyomma dubitatum*	1	3	3
Amblyomma dubitatum	*Amblyomma dubitatum*	1	3	3
Amblyomma dubitatum	*Amblyomma dubitatum*	1	3	3
Amblyomma dubitatum	*Amblyomma dubitatum*	1	3	3
Amblyomma dubitatum	*Amblyomma dubitatum*	1	3	3
Amblyomma dubitatum	*Amblyomma dubitatum*	1	3	3
Amblyomma dubitatum	*Amblyomma dubitatum*	1	3	3
Amblyomma ovale	*Amblyomma ovale*	1	4	4
Amblyomma ovale	*Amblyomma ovale*	1	4	4
Amblyomma ovale	*Amblyomma ovale*	1	4	4
Amblyomma ovale	*Amblyomma ovale*	1	4	4
Amblyomma ovale	*Amblyomma ovale*	1	4	4
Amblyomma ovale	*Amblyomma ovale*	1	4	4
Amblyomma ovale	*Amblyomma ovale*	1	4	4

Amblyomma ovale	*Amblyomma ovale*	1	4	4
Amblyomma ovale	*Amblyomma ovale*	1	4	4
Amblyomma sculptum	*Amblyomma sculptum*	1	5	5
Amblyomma sculptum	*Amblyomma sculptum*	1	5	5
Amblyomma sculptum	*Amblyomma sculptum*	1	5	5
Amblyomma sculptum	*Amblyomma sculptum*	1	5	5
Amblyomma sculptum	*Amblyomma sculptum*	1	5	5
Amblyomma sculptum	*Amblyomma sculptum*	1	5	5
Amblyomma sculptum	*Amblyomma sculptum*	1	5	5
Amblyomma sculptum	*Amblyomma sculptum*	1	5	5
Amblyomma sculptum	*Amblyomma sculptum*	1	5	5
Amblyomma sculptum	*Amblyomma sculptum*	1	5	5
Amblyomma sculptum	*Amblyomma sculptum*	1	5	5
Amblyomma sculptum	*Amblyomma sculptum*	1	5	5
Amblyomma sculptum	*Amblyomma sculptum*	1	5	5
Amblyomma sculptum	*Amblyomma sculptum*	1	5	5
Amblyomma sculptum	*Amblyomma sculptum*	1	5	5
Amblyomma sculptum	*Amblyomma sculptum*	1	5	5
Amblyomma sculptum	*Amblyomma sculptum*	1	5	5
Amblyomma sculptum	*Amblyomma sculptum*	1	5	5
Amblyomma sculptum	*Amblyomma sculptum*	1	5	5
Amblyomma triste	*Amblyomma dubitatum*	0	6	3
Amblyomma triste	*Amblyomma triste*	1	6	6
Amblyomma triste	*Amblyomma triste*	1	6	6
Amblyomma triste	*Amblyomma triste*	1	6	6
Amblyomma triste	*Amblyomma triste*	1	6	6
Amblyomma triste	*Amblyomma triste*	1	6	6
Amblyomma triste	*Amblyomma ovale*	0	6	4
Amblyomma triste	*Amblyomma triste*	1	6	6
Amblyomma triste	*Amblyomma triste*	1	6	6
Amblyomma triste	*Amblyomma triste*	1	6	6
Amblyomma triste	*Amblyomma triste*	1	6	6
Amblyomma triste	*Amblyomma triste*	1	6	6
Amblyomma triste	*Amblyomma cajennense*	0	6	2
Amblyomma triste	*Amblyomma triste*	1	6	6
Amblyomma triste	*Amblyomma triste*	1	6	6
Amblyomma triste	*Amblyomma triste*	1	6	6
Amblyomma triste	*Amblyomma triste*	1	6	6
Amblyomma triste	*Amblyomma triste*	1	6	6
Amblyomma triste	*Amblyomma triste*	1	6	6
Amblyomma triste	*Amblyomma triste*	1	6	6
Amblyomma aureolatum	*Amblyomma aureolatum*	1	1	1
Amblyomma aureolatum	*Amblyomma aureolatum*	1	1	1
Amblyomma aureolatum	*Amblyomma aureolatum*	1	1	1
Amblyomma aureolatum	*Amblyomma aureolatum*	1	1	1
Amblyomma aureolatum	*Amblyomma aureolatum*	1	1	1
Amblyomma aureolatum	*Amblyomma aureolatum*	1	1	1
Amblyomma aureolatum	*Amblyomma aureolatum*	1	1	1
Amblyomma aureolatum	*Amblyomma aureolatum*	1	1	1
Amblyomma aureolatum	*Amblyomma aureolatum*	1	1	1
Amblyomma aureolatum	*Amblyomma aureolatum*	1	1	1
Amblyomma aureolatum	*Amblyomma aureolatum*	1	1	1
Amblyomma aureolatum	*Amblyomma aureolatum*	1	1	1
Amblyomma aureolatum	*Amblyomma aureolatum*	1	1	1
Amblyomma cajennense	*Amblyomma cajennense*	1	2	2
Amblyomma cajennense	*Amblyomma cajennense*	1	2	2
Amblyomma cajennense	*Amblyomma cajennense*	1	2	2
Amblyomma cajennense	*Amblyomma cajennense*	1	2	2
Amblyomma cajennense	*Amblyomma cajennense*	1	2	2
Amblyomma cajennense	*Amblyomma cajennense*	1	2	2
Amblyomma cajennense	*Amblyomma cajennense*	1	2	2
Amblyomma cajennense	*Amblyomma cajennense*	1	2	2
Amblyomma cajennense	*Amblyomma cajennense*	1	2	2
Amblyomma cajennense	*Amblyomma cajennense*	1	2	2
Amblyomma cajennense	*Amblyomma cajennense*	1	2	2
Amblyomma cajennense	*Amblyomma cajennense*	1	2	2
Amblyomma cajennense	*Amblyomma cajennense*	1	2	2

Amblyomma cajennense	*Amblyomma cajennense*	1	2	2
Amblyomma cajennense	*Amblyomma cajennense*	1	2	2
Amblyomma cajennense	*Amblyomma cajennense*	1	2	2
Amblyomma dubitatum	*Amblyomma dubitatum*	1	3	3
Amblyomma dubitatum	*Amblyomma dubitatum*	1	3	3
Amblyomma dubitatum	*Amblyomma dubitatum*	1	3	3
Amblyomma dubitatum	*Amblyomma dubitatum*	1	3	3
Amblyomma dubitatum	*Amblyomma dubitatum*	1	3	3
Amblyomma dubitatum	*Amblyomma dubitatum*	1	3	3
Amblyomma dubitatum	*Amblyomma dubitatum*	1	3	3
Amblyomma dubitatum	*Amblyomma dubitatum*	1	3	3
Amblyomma dubitatum	*Amblyomma ovale*	0	3	4
Amblyomma dubitatum	*Amblyomma dubitatum*	1	3	3
Amblyomma dubitatum	*Amblyomma sculptum*	0	3	5
Amblyomma dubitatum	*Amblyomma dubitatum*	1	3	3
Amblyomma dubitatum	*Amblyomma dubitatum*	1	3	3
Amblyomma dubitatum	*Amblyomma ovale*	0	3	4
Amblyomma dubitatum	*Amblyomma dubitatum*	1	3	3
Amblyomma ovale	*Amblyomma ovale*	1	4	4
Amblyomma ovale	*Amblyomma ovale*	1	4	4
Amblyomma ovale	*Amblyomma triste*	0	4	6
Amblyomma ovale	*Amblyomma aureolatum*	0	4	1
Amblyomma ovale	*Amblyomma triste*	0	4	6
Amblyomma ovale	*Amblyomma ovale*	1	4	4
Amblyomma ovale	*Amblyomma ovale*	1	4	4
Amblyomma ovale	*Amblyomma dubitatum*	0	4	3
Amblyomma sculptum	*Amblyomma sculptum*	1	5	5
Amblyomma sculptum	*Amblyomma sculptum*	1	5	5
Amblyomma sculptum	*Amblyomma sculptum*	1	5	5
Amblyomma sculptum	*Amblyomma sculptum*	1	5	5
Amblyomma sculptum	*Amblyomma sculptum*	1	5	5
Amblyomma sculptum	*Amblyomma sculptum*	1	5	5
Amblyomma sculptum	*Amblyomma sculptum*	1	5	5
Amblyomma sculptum	*Amblyomma sculptum*	1	5	5
Amblyomma sculptum	*Amblyomma sculptum*	1	5	5
Amblyomma sculptum	*Amblyomma sculptum*	1	5	5
Amblyomma sculptum	*Amblyomma sculptum*	1	5	5
Amblyomma sculptum	*Amblyomma sculptum*	1	5	5
Amblyomma sculptum	*Amblyomma sculptum*	1	5	5
Amblyomma sculptum	*Amblyomma sculptum*	1	5	5
Amblyomma sculptum	*Amblyomma sculptum*	1	5	5
Amblyomma sculptum	*Amblyomma sculptum*	1	5	5
Amblyomma sculptum	*Amblyomma sculptum*	1	5	5
Amblyomma sculptum	*Amblyomma sculptum*	1	5	5
Amblyomma sculptum	*Amblyomma sculptum*	1	5	5
Amblyomma triste	*Amblyomma triste*	1	6	6
Amblyomma triste	*Amblyomma triste*	1	6	6
Amblyomma triste	*Amblyomma triste*	1	6	6
Amblyomma triste	*Amblyomma triste*	1	6	6
Amblyomma triste	*Amblyomma triste*	1	6	6
Amblyomma triste	*Amblyomma triste*	1	6	6
Amblyomma triste	*Amblyomma triste*	1	6	6
Amblyomma triste	*Amblyomma triste*	1	6	6
Amblyomma triste	*Amblyomma triste*	1	6	6
Amblyomma triste	*Amblyomma triste*	1	6	6
Amblyomma triste	*Amblyomma triste*	1	6	6
Amblyomma triste	*Amblyomma triste*	1	6	6
Amblyomma triste	*Amblyomma triste*	1	6	6
Amblyomma triste	*Amblyomma triste*	1	6	6
Amblyomma triste	*Amblyomma triste*	1	6	6
Amblyomma triste	*Amblyomma triste*	1	6	6
Amblyomma triste	*Amblyomma triste*	1	6	6
Amblyomma triste	*Amblyomma triste*	1	6	6
Amblyomma triste	*Amblyomma triste*	1	6	6
Amblyomma triste	*Amblyomma triste*	1	6	6
Amblyomma triste	*Amblyomma triste*	1	6	6
Amblyomma triste	*Amblyomma triste*	1	6	6
Amblyomma aureolatum	*Amblyomma aureolatum*	1	1	1

Amblyomma aureolatum	*Amblyomma aureolatum*	1	1	1
Amblyomma aureolatum	*Amblyomma aureolatum*	1	1	1
Amblyomma aureolatum	*Amblyomma aureolatum*	1	1	1
Amblyomma aureolatum	*Amblyomma aureolatum*	1	1	1
Amblyomma aureolatum	*Amblyomma aureolatum*	1	1	1
Amblyomma aureolatum	*Amblyomma aureolatum*	1	1	1
Amblyomma aureolatum	*Amblyomma aureolatum*	1	1	1
Amblyomma aureolatum	*Amblyomma aureolatum*	1	1	1
Amblyomma aureolatum	*Amblyomma aureolatum*	1	1	1
Amblyomma aureolatum	*Amblyomma aureolatum*	1	1	1
Amblyomma aureolatum	*Amblyomma aureolatum*	1	1	1
Amblyomma aureolatum	*Amblyomma aureolatum*	1	1	1
Amblyomma cajennense	*Amblyomma cajennense*	1	2	2
Amblyomma cajennense	*Amblyomma cajennense*	1	2	2
Amblyomma cajennense	*Amblyomma cajennense*	1	2	2
Amblyomma cajennense	*Amblyomma cajennense*	1	2	2
Amblyomma cajennense	*Amblyomma cajennense*	1	2	2
Amblyomma cajennense	*Amblyomma cajennense*	1	2	2
Amblyomma cajennense	*Amblyomma cajennense*	1	2	2
Amblyomma cajennense	*Amblyomma cajennense*	1	2	2
Amblyomma cajennense	*Amblyomma cajennense*	1	2	2
Amblyomma cajennense	*Amblyomma cajennense*	1	2	2
Amblyomma cajennense	*Amblyomma cajennense*	1	2	2
Amblyomma cajennense	*Amblyomma cajennense*	1	2	2
Amblyomma cajennense	*Amblyomma cajennense*	1	2	2
Amblyomma cajennense	*Amblyomma cajennense*	1	2	2
Amblyomma cajennense	*Amblyomma cajennense*	1	2	2
Amblyomma cajennense	*Amblyomma cajennense*	1	2	2
Amblyomma cajennense	*Amblyomma cajennense*	1	2	2
Amblyomma dubitatum	*Amblyomma dubitatum*	1	3	3
Amblyomma dubitatum	*Amblyomma dubitatum*	1	3	3
Amblyomma dubitatum	*Amblyomma dubitatum*	1	3	3
Amblyomma dubitatum	*Amblyomma dubitatum*	1	3	3
Amblyomma dubitatum	*Amblyomma dubitatum*	1	3	3
Amblyomma dubitatum	*Amblyomma dubitatum*	1	3	3
Amblyomma dubitatum	*Amblyomma dubitatum*	1	3	3
Amblyomma dubitatum	*Amblyomma dubitatum*	1	3	3
Amblyomma dubitatum	*Amblyomma dubitatum*	1	3	3
Amblyomma dubitatum	*Amblyomma dubitatum*	1	3	3
Amblyomma dubitatum	*Amblyomma dubitatum*	1	3	3
Amblyomma dubitatum	*Amblyomma dubitatum*	1	3	3
Amblyomma dubitatum	*Amblyomma dubitatum*	1	3	3
Amblyomma dubitatum	*Amblyomma dubitatum*	1	3	3
Amblyomma dubitatum	*Amblyomma ovale*	0	3	4
Amblyomma ovale	*Amblyomma ovale*	1	4	4
Amblyomma ovale	*Amblyomma ovale*	1	4	4
Amblyomma ovale	*Amblyomma ovale*	1	4	4
Amblyomma ovale	*Amblyomma ovale*	1	4	4
Amblyomma ovale	*Amblyomma ovale*	1	4	4
Amblyomma ovale	*Amblyomma ovale*	1	4	4
Amblyomma ovale	*Amblyomma ovale*	1	4	4
Amblyomma ovale	*Amblyomma ovale*	1	4	4
Amblyomma sculptum	*Amblyomma sculptum*	1	5	5
Amblyomma sculptum	*Amblyomma sculptum*	1	5	5
Amblyomma sculptum	*Amblyomma sculptum*	1	5	5
Amblyomma sculptum	*Amblyomma sculptum*	1	5	5
Amblyomma sculptum	*Amblyomma sculptum*	1	5	5
Amblyomma sculptum	*Amblyomma sculptum*	1	5	5
Amblyomma sculptum	*Amblyomma sculptum*	1	5	5
Amblyomma sculptum	*Amblyomma sculptum*	1	5	5
Amblyomma sculptum	*Amblyomma sculptum*	1	5	5
Amblyomma sculptum	*Amblyomma cajennense*	0	5	2
Amblyomma sculptum	*Amblyomma sculptum*	1	5	5
Amblyomma sculptum	*Amblyomma sculptum*	1	5	5
Amblyomma sculptum	*Amblyomma sculptum*	1	5	5
Amblyomma sculptum	*Amblyomma sculptum*	1	5	5
Amblyomma sculptum	*Amblyomma sculptum*	1	5	5

Amblyomma sculptum	*Amblyomma sculptum*	1	5	5
Amblyomma sculptum	*Amblyomma sculptum*	1	5	5
Amblyomma sculptum	*Amblyomma sculptum*	1	5	5
Amblyomma triste	*Amblyomma triste*	1	6	6
Amblyomma triste	*Amblyomma triste*	1	6	6
Amblyomma triste	*Amblyomma triste*	1	6	6
Amblyomma triste	*Amblyomma triste*	1	6	6
Amblyomma triste	*Amblyomma triste*	1	6	6
Amblyomma triste	*Amblyomma triste*	1	6	6
Amblyomma triste	*Amblyomma triste*	1	6	6
Amblyomma triste	*Amblyomma triste*	1	6	6
Amblyomma triste	*Amblyomma triste*	1	6	6
Amblyomma triste	*Amblyomma triste*	1	6	6
Amblyomma triste	*Amblyomma triste*	1	6	6
Amblyomma triste	*Amblyomma triste*	1	6	6
Amblyomma triste	*Amblyomma triste*	1	6	6
Amblyomma triste	*Amblyomma triste*	1	6	6
Amblyomma triste	*Amblyomma triste*	1	6	6
Amblyomma triste	*Amblyomma triste*	1	6	6
Amblyomma triste	*Amblyomma ovale*	0	6	4
Amblyomma triste	*Amblyomma triste*	1	6	6
Amblyomma triste	*Amblyomma aureolatum*	0	6	1
Amblyomma triste	*Amblyomma triste*	1	6	6
Amblyomma triste	*Amblyomma triste*	1	6	6
Amblyomma aureolatum	*Amblyomma triste*	0	1	6
Amblyomma aureolatum	*Amblyomma triste*	0	1	6
Amblyomma aureolatum	*Amblyomma aureolatum*	1	1	1
Amblyomma aureolatum	*Amblyomma aureolatum*	1	1	1
Amblyomma aureolatum	*Amblyomma ovale*	0	1	4
Amblyomma aureolatum	*Amblyomma aureolatum*	1	1	1
Amblyomma aureolatum	*Amblyomma aureolatum*	1	1	1
Amblyomma aureolatum	*Amblyomma aureolatum*	1	1	1
Amblyomma aureolatum	*Amblyomma aureolatum*	1	1	1
Amblyomma aureolatum	*Amblyomma aureolatum*	1	1	1
Amblyomma aureolatum	*Amblyomma aureolatum*	1	1	1
Amblyomma aureolatum	*Amblyomma triste*	0	1	6
Amblyomma cajennense	*Amblyomma cajennense*	1	2	2
Amblyomma cajennense	*Amblyomma cajennense*	1	2	2
Amblyomma cajennense	*Amblyomma cajennense*	1	2	2
Amblyomma cajennense	*Amblyomma cajennense*	1	2	2
Amblyomma cajennense	*Amblyomma cajennense*	1	2	2
Amblyomma cajennense	*Amblyomma cajennense*	1	2	2
Amblyomma cajennense	*Amblyomma cajennense*	1	2	2
Amblyomma cajennense	*Amblyomma cajennense*	1	2	2
Amblyomma cajennense	*Amblyomma cajennense*	1	2	2
Amblyomma cajennense	*Amblyomma cajennense*	1	2	2
Amblyomma cajennense	*Amblyomma cajennense*	1	2	2
Amblyomma cajennense	*Amblyomma triste*	0	2	6
Amblyomma cajennense	*Amblyomma cajennense*	1	2	2
Amblyomma cajennense	*Amblyomma cajennense*	1	2	2
Amblyomma cajennense	*Amblyomma cajennense*	1	2	2
Amblyomma cajennense	*Amblyomma cajennense*	1	2	2
Amblyomma dubitatum	*Amblyomma dubitatum*	1	3	3
Amblyomma dubitatum	*Amblyomma dubitatum*	1	3	3
Amblyomma dubitatum	*Amblyomma dubitatum*	1	3	3
Amblyomma dubitatum	*Amblyomma dubitatum*	1	3	3
Amblyomma dubitatum	*Amblyomma dubitatum*	1	3	3
Amblyomma dubitatum	*Amblyomma dubitatum*	1	3	3
Amblyomma dubitatum	*Amblyomma dubitatum*	1	3	3
Amblyomma dubitatum	*Amblyomma dubitatum*	1	3	3
Amblyomma dubitatum	*Amblyomma dubitatum*	1	3	3
Amblyomma dubitatum	*Amblyomma triste*	0	3	6
Amblyomma dubitatum	*Amblyomma triste*	0	3	6
Amblyomma ovale	*Amblyomma ovale*	1	4	4
Amblyomma ovale	*Amblyomma ovale*	1	4	4
Amblyomma ovale	*Amblyomma ovale*	1	4	4
Amblyomma ovale	*Amblyomma ovale*	1	4	4

Amblyomma ovale	*Amblyomma ovale*	1	4	4
Amblyomma ovale	*Amblyomma ovale*	1	4	4
Amblyomma ovale	*Amblyomma ovale*	1	4	4
Amblyomma ovale	*Amblyomma ovale*	1	4	4
Amblyomma ovale	*Amblyomma ovale*	1	4	4
Amblyomma ovale	*Amblyomma triste*	0	4	6
Amblyomma ovale	*Amblyomma ovale*	1	4	4
Amblyomma ovale	*Amblyomma ovale*	1	4	4
Amblyomma sculptum	*Amblyomma sculptum*	1	5	5
Amblyomma sculptum	*Amblyomma sculptum*	1	5	5
Amblyomma sculptum	*Amblyomma sculptum*	1	5	5
Amblyomma sculptum	*Amblyomma sculptum*	1	5	5
Amblyomma sculptum	*Amblyomma sculptum*	1	5	5
Amblyomma sculptum	*Amblyomma dubitatum*	0	5	3
Amblyomma sculptum	*Amblyomma sculptum*	1	5	5
Amblyomma sculptum	*Amblyomma sculptum*	1	5	5
Amblyomma sculptum	*Amblyomma sculptum*	1	5	5
Amblyomma sculptum	*Amblyomma sculptum*	1	5	5
Amblyomma sculptum	*Amblyomma sculptum*	1	5	5
Amblyomma sculptum	*Amblyomma sculptum*	1	5	5
Amblyomma sculptum	*Amblyomma sculptum*	1	5	5
Amblyomma sculptum	*Amblyomma triste*	0	5	6
Amblyomma sculptum	*Amblyomma sculptum*	1	5	5
Amblyomma sculptum	*Amblyomma sculptum*	1	5	5
Amblyomma triste	*Amblyomma triste*	1	6	6
Amblyomma triste	*Amblyomma triste*	1	6	6
Amblyomma triste	*Amblyomma triste*	1	6	6
Amblyomma triste	*Amblyomma triste*	1	6	6
Amblyomma triste	*Amblyomma triste*	1	6	6
Amblyomma triste	*Amblyomma triste*	1	6	6
Amblyomma triste	*Amblyomma triste*	1	6	6
Amblyomma triste	*Amblyomma triste*	1	6	6
Amblyomma triste	*Amblyomma triste*	1	6	6
Amblyomma triste	*Amblyomma triste*	1	6	6
Amblyomma triste	*Amblyomma triste*	1	6	6
Amblyomma triste	*Amblyomma triste*	1	6	6
Amblyomma triste	*Amblyomma triste*	1	6	6
Amblyomma triste	*Amblyomma triste*	1	6	6
Amblyomma triste	*Amblyomma triste*	1	6	6
Amblyomma triste	*Amblyomma triste*	1	6	6
Amblyomma aureolatum	*Amblyomma ovale*	0	1	4
Amblyomma aureolatum	*Amblyomma aureolatum*	1	1	1
Amblyomma aureolatum	*Amblyomma aureolatum*	1	1	1
Amblyomma aureolatum	*Amblyomma aureolatum*	1	1	1
Amblyomma aureolatum	*Amblyomma triste*	0	1	6
Amblyomma aureolatum	*Amblyomma aureolatum*	1	1	1
Amblyomma aureolatum	*Amblyomma triste*	0	1	6
Amblyomma aureolatum	*Amblyomma aureolatum*	1	1	1
Amblyomma aureolatum	*Amblyomma aureolatum*	1	1	1
Amblyomma aureolatum	*Amblyomma aureolatum*	1	1	1
Amblyomma aureolatum	*Amblyomma aureolatum*	1	1	1
Amblyomma aureolatum	*Amblyomma aureolatum*	1	1	1
Amblyomma aureolatum	*Amblyomma aureolatum*	1	1	1
Amblyomma cajennense	*Amblyomma triste*	0	2	6
Amblyomma cajennense	*Amblyomma cajennense*	1	2	2
Amblyomma cajennense	*Amblyomma cajennense*	1	2	2
Amblyomma cajennense	*Amblyomma triste*	0	2	6
Amblyomma cajennense	*Amblyomma cajennense*	1	2	2
Amblyomma cajennense	*Amblyomma cajennense*	1	2	2
Amblyomma cajennense	*Amblyomma cajennense*	1	2	2
Amblyomma cajennense	*Amblyomma cajennense*	1	2	2
Amblyomma cajennense	*Amblyomma cajennense*	1	2	2
Amblyomma cajennense	*Amblyomma cajennense*	1	2	2
Amblyomma cajennense	*Amblyomma cajennense*	1	2	2
Amblyomma cajennense	*Amblyomma cajennense*	1	2	2
Amblyomma cajennense	*Amblyomma cajennense*	1	2	2
Amblyomma cajennense	*Amblyomma cajennense*	1	2	2

Amblyomma cajennense	*Amblyomma cajennense*	1	2	2
Amblyomma cajennense	*Amblyomma triste*	0	2	6
Amblyomma dubitatum	*Amblyomma dubitatum*	1	3	3
Amblyomma dubitatum	*Amblyomma triste*	0	3	6
Amblyomma dubitatum	*Amblyomma dubitatum*	1	3	3
Amblyomma dubitatum	*Amblyomma dubitatum*	1	3	3
Amblyomma dubitatum	*Amblyomma dubitatum*	1	3	3
Amblyomma dubitatum	*Amblyomma dubitatum*	1	3	3
Amblyomma dubitatum	*Amblyomma triste*	0	3	6
Amblyomma dubitatum	*Amblyomma dubitatum*	1	3	3
Amblyomma dubitatum	*Amblyomma dubitatum*	1	3	3
Amblyomma dubitatum	*Amblyomma aureolatum*	0	3	1
Amblyomma dubitatum	*Amblyomma triste*	0	3	6
Amblyomma dubitatum	*Amblyomma triste*	0	3	6
Amblyomma ovale	*Amblyomma ovale*	1	4	4
Amblyomma ovale	*Amblyomma ovale*	1	4	4
Amblyomma ovale	*Amblyomma ovale*	1	4	4
Amblyomma ovale	*Amblyomma ovale*	1	4	4
Amblyomma ovale	*Amblyomma ovale*	1	4	4
Amblyomma ovale	*Amblyomma ovale*	1	4	4
Amblyomma ovale	*Amblyomma triste*	0	4	6
Amblyomma ovale	*Amblyomma ovale*	1	4	4
Amblyomma ovale	*Amblyomma ovale*	1	4	4
Amblyomma ovale	*Amblyomma ovale*	1	4	4
Amblyomma ovale	*Amblyomma ovale*	1	4	4
Amblyomma ovale	*Amblyomma ovale*	1	4	4
Amblyomma sculptum	*Amblyomma triste*	0	5	6
Amblyomma sculptum	*Amblyomma sculptum*	1	5	5
Amblyomma sculptum	*Amblyomma sculptum*	1	5	5
Amblyomma sculptum	*Amblyomma sculptum*	1	5	5
Amblyomma sculptum	*Amblyomma sculptum*	1	5	5
Amblyomma sculptum	*Amblyomma sculptum*	1	5	5
Amblyomma sculptum	*Amblyomma cajennense*	0	5	2
Amblyomma sculptum	*Amblyomma sculptum*	1	5	5
Amblyomma sculptum	*Amblyomma sculptum*	1	5	5
Amblyomma sculptum	*Amblyomma sculptum*	1	5	5
Amblyomma sculptum	*Amblyomma sculptum*	1	5	5
Amblyomma sculptum	*Amblyomma sculptum*	1	5	5
Amblyomma sculptum	*Amblyomma sculptum*	1	5	5
Amblyomma sculptum	*Amblyomma sculptum*	1	5	5
Amblyomma sculptum	*Amblyomma sculptum*	1	5	5
Amblyomma sculptum	*Amblyomma sculptum*	1	5	5
Amblyomma sculptum	*Amblyomma sculptum*	1	5	5
Amblyomma triste	*Amblyomma triste*	1	6	6
Amblyomma triste	*Amblyomma triste*	1	6	6
Amblyomma triste	*Amblyomma triste*	1	6	6
Amblyomma triste	*Amblyomma triste*	1	6	6
Amblyomma triste	*Amblyomma triste*	1	6	6
Amblyomma triste	*Amblyomma triste*	1	6	6
Amblyomma triste	*Amblyomma triste*	1	6	6
Amblyomma triste	*Amblyomma triste*	1	6	6
Amblyomma triste	*Amblyomma triste*	1	6	6
Amblyomma triste	*Amblyomma triste*	1	6	6
Amblyomma triste	*Amblyomma triste*	1	6	6
Amblyomma triste	*Amblyomma triste*	1	6	6
Amblyomma triste	*Amblyomma triste*	1	6	6
Amblyomma triste	*Amblyomma triste*	1	6	6
Amblyomma triste	*Amblyomma triste*	1	6	6
Amblyomma triste	*Amblyomma triste*	1	6	6
Amblyomma aureolatum	*Amblyomma aureolatum*	1	1	1
Amblyomma aureolatum	*Amblyomma aureolatum*	1	1	1
Amblyomma aureolatum	*Amblyomma aureolatum*	1	1	1
Amblyomma aureolatum	*Amblyomma aureolatum*	1	1	1
Amblyomma aureolatum	*Amblyomma aureolatum*	1	1	1
Amblyomma aureolatum	*Amblyomma aureolatum*	1	1	1
Amblyomma aureolatum	*Amblyomma aureolatum*	1	1	1
Amblyomma aureolatum	*Amblyomma aureolatum*	1	1	1

Amblyomma aureolatum	*Amblyomma aureolatum*	1	1	1
Amblyomma aureolatum	*Amblyomma aureolatum*	1	1	1
Amblyomma aureolatum	*Amblyomma aureolatum*	1	1	1
Amblyomma aureolatum	*Amblyomma aureolatum*	1	1	1
Amblyomma cajennense	*Amblyomma cajennense*	1	2	2
Amblyomma cajennense	*Amblyomma cajennense*	1	2	2
Amblyomma cajennense	*Amblyomma cajennense*	1	2	2
Amblyomma cajennense	*Amblyomma cajennense*	1	2	2
Amblyomma cajennense	*Amblyomma cajennense*	1	2	2
Amblyomma cajennense	*Amblyomma cajennense*	1	2	2
Amblyomma cajennense	*Amblyomma cajennense*	1	2	2
Amblyomma cajennense	*Amblyomma cajennense*	1	2	2
Amblyomma cajennense	*Amblyomma cajennense*	1	2	2
Amblyomma cajennense	*Amblyomma cajennense*	1	2	2
Amblyomma cajennense	*Amblyomma cajennense*	1	2	2
Amblyomma cajennense	*Amblyomma cajennense*	1	2	2
Amblyomma cajennense	*Amblyomma cajennense*	1	2	2
Amblyomma cajennense	*Amblyomma cajennense*	1	2	2
Amblyomma cajennense	*Amblyomma cajennense*	1	2	2
Amblyomma cajennense	*Amblyomma cajennense*	1	2	2
Amblyomma cajennense	*Amblyomma cajennense*	1	2	2
Amblyomma dubitatum	*Amblyomma cajennense*	0	3	2
Amblyomma dubitatum	*Amblyomma dubitatum*	1	3	3
Amblyomma dubitatum	*Amblyomma dubitatum*	1	3	3
Amblyomma dubitatum	*Amblyomma dubitatum*	1	3	3
Amblyomma dubitatum	*Amblyomma dubitatum*	1	3	3
Amblyomma dubitatum	*Amblyomma ovale*	0	3	4
Amblyomma dubitatum	*Amblyomma sculptum*	0	3	5
Amblyomma dubitatum	*Amblyomma aureolatum*	0	3	1
Amblyomma dubitatum	*Amblyomma cajennense*	0	3	2
Amblyomma dubitatum	*Amblyomma dubitatum*	1	3	3
Amblyomma dubitatum	*Amblyomma dubitatum*	1	3	3
Amblyomma ovale	*Amblyomma ovale*	1	4	4
Amblyomma ovale	*Amblyomma ovale*	1	4	4
Amblyomma ovale	*Amblyomma ovale*	1	4	4
Amblyomma ovale	*Amblyomma ovale*	1	4	4
Amblyomma ovale	*Amblyomma aureolatum*	0	4	1
Amblyomma ovale	*Amblyomma ovale*	1	4	4
Amblyomma ovale	*Amblyomma aureolatum*	0	4	1
Amblyomma ovale	*Amblyomma ovale*	1	4	4
Amblyomma ovale	*Amblyomma ovale*	1	4	4
Amblyomma ovale	*Amblyomma cajennense*	0	4	2
Amblyomma ovale	*Amblyomma ovale*	1	4	4
Amblyomma ovale	*Amblyomma ovale*	1	4	4
Amblyomma sculptum	*Amblyomma sculptum*	1	5	5
Amblyomma sculptum	*Amblyomma sculptum*	1	5	5
Amblyomma sculptum	*Amblyomma sculptum*	1	5	5
Amblyomma sculptum	*Amblyomma sculptum*	1	5	5
Amblyomma sculptum	*Amblyomma dubitatum*	0	5	3
Amblyomma sculptum	*Amblyomma sculptum*	1	5	5
Amblyomma sculptum	*Amblyomma sculptum*	1	5	5
Amblyomma sculptum	*Amblyomma sculptum*	1	5	5
Amblyomma sculptum	*Amblyomma sculptum*	1	5	5
Amblyomma sculptum	*Amblyomma sculptum*	1	5	5
Amblyomma sculptum	*Amblyomma sculptum*	1	5	5
Amblyomma sculptum	*Amblyomma sculptum*	1	5	5
Amblyomma sculptum	*Amblyomma sculptum*	1	5	5
Amblyomma sculptum	*Amblyomma sculptum*	1	5	5
Amblyomma sculptum	*Amblyomma sculptum*	1	5	5
Amblyomma sculptum	*Amblyomma sculptum*	1	5	5
Amblyomma triste	*Amblyomma cajennense*	0	6	2
Amblyomma triste	*Amblyomma triste*	1	6	6
Amblyomma triste	*Amblyomma triste*	1	6	6
Amblyomma triste	*Amblyomma triste*	1	6	6
Amblyomma triste	*Amblyomma aureolatum*	0	6	1
Amblyomma triste	*Amblyomma triste*	1	6	6
Amblyomma triste	*Amblyomma triste*	1	6	6

Amblyomma triste	*Amblyomma triste*	1	6	6
Amblyomma triste	*Amblyomma triste*	1	6	6
Amblyomma triste	*Amblyomma triste*	1	6	6
Amblyomma triste	*Amblyomma triste*	1	6	6
Amblyomma triste	*Amblyomma triste*	1	6	6
Amblyomma triste	*Amblyomma triste*	1	6	6
Amblyomma triste	*Amblyomma triste*	1	6	6
Amblyomma triste	*Amblyomma triste*	1	6	6
Amblyomma triste	*Amblyomma triste*	1	6	6
Amblyomma aureolatum	*Amblyomma aureolatum*	1	1	1
Amblyomma aureolatum	*Amblyomma aureolatum*	1	1	1
Amblyomma aureolatum	*Amblyomma aureolatum*	1	1	1
Amblyomma aureolatum	*Amblyomma aureolatum*	1	1	1
Amblyomma aureolatum	*Amblyomma dubitatum*	0	1	3
Amblyomma aureolatum	*Amblyomma aureolatum*	1	1	1
Amblyomma aureolatum	*Amblyomma aureolatum*	1	1	1
Amblyomma aureolatum	*Amblyomma aureolatum*	1	1	1
Amblyomma aureolatum	*Amblyomma aureolatum*	1	1	1
Amblyomma aureolatum	*Amblyomma aureolatum*	1	1	1
Amblyomma aureolatum	*Amblyomma aureolatum*	1	1	1
Amblyomma aureolatum	*Amblyomma aureolatum*	1	1	1
Amblyomma aureolatum	*Amblyomma aureolatum*	1	1	1
Amblyomma cajennense	*Amblyomma cajennense*	1	2	2
Amblyomma cajennense	*Amblyomma cajennense*	1	2	2
Amblyomma cajennense	*Amblyomma cajennense*	1	2	2
Amblyomma cajennense	*Amblyomma cajennense*	1	2	2
Amblyomma cajennense	*Amblyomma cajennense*	1	2	2
Amblyomma cajennense	*Amblyomma cajennense*	1	2	2
Amblyomma cajennense	*Amblyomma cajennense*	1	2	2
Amblyomma cajennense	*Amblyomma cajennense*	1	2	2
Amblyomma cajennense	*Amblyomma cajennense*	1	2	2
Amblyomma cajennense	*Amblyomma cajennense*	1	2	2
Amblyomma cajennense	*Amblyomma cajennense*	1	2	2
Amblyomma cajennense	*Amblyomma cajennense*	1	2	2
Amblyomma cajennense	*Amblyomma cajennense*	1	2	2
Amblyomma cajennense	*Amblyomma cajennense*	1	2	2
Amblyomma cajennense	*Amblyomma cajennense*	1	2	2
Amblyomma cajennense	*Amblyomma cajennense*	1	2	2
Amblyomma cajennense	*Amblyomma cajennense*	1	2	2
Amblyomma dubitatum	*Amblyomma triste*	0	3	6
Amblyomma dubitatum	*Amblyomma dubitatum*	1	3	3
Amblyomma dubitatum	*Amblyomma dubitatum*	1	3	3
Amblyomma dubitatum	*Amblyomma dubitatum*	1	3	3
Amblyomma dubitatum	*Amblyomma dubitatum*	1	3	3
Amblyomma dubitatum	*Amblyomma dubitatum*	1	3	3
Amblyomma dubitatum	*Amblyomma sculptum*	0	3	5
Amblyomma dubitatum	*Amblyomma dubitatum*	1	3	3
Amblyomma dubitatum	*Amblyomma aureolatum*	0	3	1
Amblyomma dubitatum	*Amblyomma dubitatum*	1	3	3
Amblyomma dubitatum	*Amblyomma sculptum*	0	3	5
Amblyomma ovale	*Amblyomma ovale*	1	4	4
Amblyomma ovale	*Amblyomma ovale*	1	4	4
Amblyomma ovale	*Amblyomma ovale*	1	4	4
Amblyomma ovale	*Amblyomma ovale*	1	4	4
Amblyomma ovale	*Amblyomma ovale*	1	4	4
Amblyomma ovale	*Amblyomma ovale*	1	4	4
Amblyomma ovale	*Amblyomma triste*	0	4	6
Amblyomma ovale	*Amblyomma triste*	0	4	6
Amblyomma ovale	*Amblyomma sculptum*	0	4	5
Amblyomma ovale	*Amblyomma ovale*	1	4	4
Amblyomma ovale	*Amblyomma ovale*	1	4	4
Amblyomma ovale	*Amblyomma ovale*	1	4	4
Amblyomma sculptum	*Amblyomma sculptum*	1	5	5
Amblyomma sculptum	*Amblyomma sculptum*	1	5	5
Amblyomma sculptum	*Amblyomma sculptum*	1	5	5
Amblyomma sculptum	*Amblyomma sculptum*	1	5	5
Amblyomma sculptum	*Amblyomma sculptum*	1	5	5

Amblyomma sculptum	*Amblyomma sculptum*	1	5	5
Amblyomma sculptum	*Amblyomma sculptum*	1	5	5
Amblyomma sculptum	*Amblyomma sculptum*	1	5	5
Amblyomma sculptum	*Amblyomma sculptum*	1	5	5
Amblyomma sculptum	*Amblyomma sculptum*	1	5	5
Amblyomma sculptum	*Amblyomma sculptum*	1	5	5
Amblyomma sculptum	*Amblyomma sculptum*	1	5	5
Amblyomma sculptum	*Amblyomma sculptum*	1	5	5
Amblyomma sculptum	*Amblyomma sculptum*	1	5	5
Amblyomma sculptum	*Amblyomma sculptum*	1	5	5
Amblyomma sculptum	*Amblyomma sculptum*	1	5	5
Amblyomma triste	*Amblyomma triste*	1	6	6
Amblyomma triste	*Amblyomma triste*	1	6	6
Amblyomma triste	*Amblyomma triste*	1	6	6
Amblyomma triste	*Amblyomma triste*	1	6	6
Amblyomma triste	*Amblyomma triste*	1	6	6
Amblyomma triste	*Amblyomma triste*	1	6	6
Amblyomma triste	*Amblyomma triste*	1	6	6
Amblyomma triste	*Amblyomma triste*	1	6	6
Amblyomma triste	*Amblyomma triste*	1	6	6
Amblyomma triste	*Amblyomma triste*	1	6	6
Amblyomma triste	*Amblyomma triste*	1	6	6
Amblyomma triste	*Amblyomma triste*	1	6	6
Amblyomma triste	*Amblyomma triste*	1	6	6
Amblyomma triste	*Amblyomma triste*	1	6	6
Amblyomma triste	*Amblyomma triste*	1	6	6
Amblyomma aureolatum	*Amblyomma aureolatum*	1	1	1
Amblyomma aureolatum	*Amblyomma aureolatum*	1	1	1
Amblyomma aureolatum	*Amblyomma aureolatum*	1	1	1
Amblyomma aureolatum	*Amblyomma aureolatum*	1	1	1
Amblyomma aureolatum	*Amblyomma aureolatum*	1	1	1
Amblyomma aureolatum	*Amblyomma aureolatum*	1	1	1
Amblyomma aureolatum	*Amblyomma aureolatum*	1	1	1
Amblyomma aureolatum	*Amblyomma aureolatum*	1	1	1
Amblyomma aureolatum	*Amblyomma aureolatum*	1	1	1
Amblyomma aureolatum	*Amblyomma aureolatum*	1	1	1
Amblyomma aureolatum	*Amblyomma aureolatum*	1	1	1
Amblyomma aureolatum	*Amblyomma aureolatum*	1	1	1
Amblyomma aureolatum	*Amblyomma aureolatum*	1	1	1
Amblyomma cajennense	*Amblyomma cajennense*	1	2	2
Amblyomma cajennense	*Amblyomma cajennense*	1	2	2
Amblyomma cajennense	*Amblyomma cajennense*	1	2	2
Amblyomma cajennense	*Amblyomma cajennense*	1	2	2
Amblyomma cajennense	*Amblyomma cajennense*	1	2	2
Amblyomma cajennense	*Amblyomma cajennense*	1	2	2
Amblyomma cajennense	*Amblyomma cajennense*	1	2	2
Amblyomma cajennense	*Amblyomma cajennense*	1	2	2
Amblyomma cajennense	*Amblyomma cajennense*	1	2	2
Amblyomma cajennense	*Amblyomma cajennense*	1	2	2
Amblyomma cajennense	*Amblyomma cajennense*	1	2	2
Amblyomma cajennense	*Amblyomma cajennense*	1	2	2
Amblyomma cajennense	*Amblyomma cajennense*	1	2	2
Amblyomma cajennense	*Amblyomma cajennense*	1	2	2
Amblyomma cajennense	*Amblyomma cajennense*	1	2	2
Amblyomma cajennense	*Amblyomma cajennense*	1	2	2
Amblyomma cajennense	*Amblyomma cajennense*	1	2	2
Amblyomma dubitatum	*Amblyomma dubitatum*	1	3	3
Amblyomma dubitatum	*Amblyomma dubitatum*	1	3	3
Amblyomma dubitatum	*Amblyomma dubitatum*	1	3	3
Amblyomma dubitatum	*Amblyomma dubitatum*	1	3	3
Amblyomma dubitatum	*Amblyomma dubitatum*	1	3	3
Amblyomma dubitatum	*Amblyomma dubitatum*	1	3	3
Amblyomma dubitatum	*Amblyomma dubitatum*	1	3	3
Amblyomma dubitatum	*Amblyomma dubitatum*	1	3	3
Amblyomma dubitatum	*Amblyomma aureolatum*	0	3	1
Amblyomma dubitatum	*Amblyomma dubitatum*	1	3	3
Amblyomma dubitatum	*Amblyomma dubitatum*	1	3	3

Amblyomma ovale	*Amblyomma ovale*	1	4	4
Amblyomma ovale	*Amblyomma ovale*	1	4	4
Amblyomma ovale	*Amblyomma ovale*	1	4	4
Amblyomma ovale	*Amblyomma ovale*	1	4	4
Amblyomma ovale	*Amblyomma aureolatum*	0	4	1
Amblyomma ovale	*Amblyomma ovale*	1	4	4
Amblyomma ovale	*Amblyomma ovale*	1	4	4
Amblyomma ovale	*Amblyomma dubitatum*	0	4	3
Amblyomma ovale	*Amblyomma ovale*	1	4	4
Amblyomma ovale	*Amblyomma ovale*	1	4	4
Amblyomma ovale	*Amblyomma ovale*	1	4	4
Amblyomma ovale	*Amblyomma ovale*	1	4	4
Amblyomma ovale	*Amblyomma ovale*	1	4	4
Amblyomma sculptum	*Amblyomma sculptum*	1	5	5
Amblyomma sculptum	*Amblyomma sculptum*	1	5	5
Amblyomma sculptum	*Amblyomma sculptum*	1	5	5
Amblyomma sculptum	*Amblyomma sculptum*	1	5	5
Amblyomma sculptum	*Amblyomma dubitatum*	0	5	3
Amblyomma sculptum	*Amblyomma sculptum*	1	5	5
Amblyomma sculptum	*Amblyomma sculptum*	1	5	5
Amblyomma sculptum	*Amblyomma sculptum*	1	5	5
Amblyomma sculptum	*Amblyomma sculptum*	1	5	5
Amblyomma sculptum	*Amblyomma sculptum*	1	5	5
Amblyomma sculptum	*Amblyomma sculptum*	1	5	5
Amblyomma sculptum	*Amblyomma sculptum*	1	5	5
Amblyomma sculptum	*Amblyomma sculptum*	1	5	5
Amblyomma sculptum	*Amblyomma sculptum*	1	5	5
Amblyomma sculptum	*Amblyomma sculptum*	1	5	5
Amblyomma sculptum	*Amblyomma sculptum*	1	5	5
Amblyomma triste	*Amblyomma triste*	1	6	6
Amblyomma triste	*Amblyomma triste*	1	6	6
Amblyomma triste	*Amblyomma triste*	1	6	6
Amblyomma triste	*Amblyomma triste*	1	6	6
Amblyomma triste	*Amblyomma triste*	1	6	6
Amblyomma triste	*Amblyomma triste*	1	6	6
Amblyomma triste	*Amblyomma triste*	1	6	6
Amblyomma triste	*Amblyomma triste*	1	6	6
Amblyomma triste	*Amblyomma triste*	1	6	6
Amblyomma triste	*Amblyomma triste*	1	6	6
Amblyomma triste	*Amblyomma triste*	1	6	6
Amblyomma triste	*Amblyomma aureolatum*	0	6	1
Amblyomma triste	*Amblyomma triste*	1	6	6
Amblyomma triste	*Amblyomma triste*	1	6	6
Amblyomma triste	*Amblyomma triste*	1	6	6
Amblyomma triste	*Amblyomma triste*	1	6	6
Amblyomma aureolatum	*Amblyomma aureolatum*	1	1	1
Amblyomma aureolatum	*Amblyomma aureolatum*	1	1	1
Amblyomma aureolatum	*Amblyomma aureolatum*	1	1	1
Amblyomma aureolatum	*Amblyomma aureolatum*	1	1	1
Amblyomma aureolatum	*Amblyomma aureolatum*	1	1	1
Amblyomma aureolatum	*Amblyomma aureolatum*	1	1	1
Amblyomma aureolatum	*Amblyomma aureolatum*	1	1	1
Amblyomma aureolatum	*Amblyomma aureolatum*	1	1	1
Amblyomma aureolatum	*Amblyomma aureolatum*	1	1	1
Amblyomma aureolatum	*Amblyomma aureolatum*	1	1	1
Amblyomma aureolatum	*Amblyomma aureolatum*	1	1	1
Amblyomma aureolatum	*Amblyomma aureolatum*	1	1	1
Amblyomma cajennense	*Amblyomma cajennense*	1	2	2
Amblyomma cajennense	*Amblyomma cajennense*	1	2	2
Amblyomma cajennense	*Amblyomma cajennense*	1	2	2
Amblyomma cajennense	*Amblyomma cajennense*	1	2	2
Amblyomma cajennense	*Amblyomma cajennense*	1	2	2
Amblyomma cajennense	*Amblyomma cajennense*	1	2	2
Amblyomma cajennense	*Amblyomma cajennense*	1	2	2
Amblyomma cajennense	*Amblyomma cajennense*	1	2	2
Amblyomma cajennense	*Amblyomma cajennense*	1	2	2
Amblyomma cajennense	*Amblyomma cajennense*	1	2	2

Amblyomma cajennense	*Amblyomma cajennense*	1	2	2
Amblyomma cajennense	*Amblyomma triste*	0	2	6
Amblyomma cajennense	*Amblyomma cajennense*	1	2	2
Amblyomma cajennense	*Amblyomma cajennense*	1	2	2
Amblyomma cajennense	*Amblyomma cajennense*	1	2	2
Amblyomma cajennense	*Amblyomma cajennense*	1	2	2
Amblyomma dubitatum	*Amblyomma dubitatum*	1	3	3
Amblyomma dubitatum	*Amblyomma dubitatum*	1	3	3
Amblyomma dubitatum	*Amblyomma dubitatum*	1	3	3
Amblyomma dubitatum	*Amblyomma dubitatum*	1	3	3
Amblyomma dubitatum	*Amblyomma dubitatum*	1	3	3
Amblyomma dubitatum	*Amblyomma dubitatum*	1	3	3
Amblyomma dubitatum	*Amblyomma dubitatum*	1	3	3
Amblyomma dubitatum	*Amblyomma dubitatum*	1	3	3
Amblyomma dubitatum	*Amblyomma triste*	0	3	6
Amblyomma dubitatum	*Amblyomma dubitatum*	1	3	3
Amblyomma ovale	*Amblyomma ovale*	1	4	4
Amblyomma ovale	*Amblyomma dubitatum*	0	4	3
Amblyomma ovale	*Amblyomma ovale*	1	4	4
Amblyomma ovale	*Amblyomma ovale*	1	4	4
Amblyomma ovale	*Amblyomma ovale*	1	4	4
Amblyomma ovale	*Amblyomma ovale*	1	4	4
Amblyomma ovale	*Amblyomma ovale*	1	4	4
Amblyomma ovale	*Amblyomma ovale*	1	4	4
Amblyomma ovale	*Amblyomma triste*	0	4	6
Amblyomma ovale	*Amblyomma ovale*	1	4	4
Amblyomma ovale	*Amblyomma ovale*	1	4	4
Amblyomma ovale	*Amblyomma ovale*	1	4	4
Amblyomma sculptum	*Amblyomma sculptum*	1	5	5
Amblyomma sculptum	*Amblyomma sculptum*	1	5	5
Amblyomma sculptum	*Amblyomma ovale*	0	5	4
Amblyomma sculptum	*Amblyomma dubitatum*	0	5	3
Amblyomma sculptum	*Amblyomma dubitatum*	0	5	3
Amblyomma sculptum	*Amblyomma aureolatum*	0	5	1
Amblyomma sculptum	*Amblyomma sculptum*	1	5	5
Amblyomma sculptum	*Amblyomma sculptum*	1	5	5
Amblyomma sculptum	*Amblyomma sculptum*	1	5	5
Amblyomma sculptum	*Amblyomma sculptum*	1	5	5
Amblyomma sculptum	*Amblyomma sculptum*	1	5	5
Amblyomma sculptum	*Amblyomma sculptum*	1	5	5
Amblyomma sculptum	*Amblyomma sculptum*	1	5	5
Amblyomma sculptum	*Amblyomma sculptum*	1	5	5
Amblyomma sculptum	*Amblyomma sculptum*	1	5	5
Amblyomma triste	*Amblyomma triste*	1	6	6
Amblyomma triste	*Amblyomma triste*	1	6	6
Amblyomma triste	*Amblyomma ovale*	0	6	4
Amblyomma triste	*Amblyomma triste*	1	6	6
Amblyomma triste	*Amblyomma triste*	1	6	6
Amblyomma triste	*Amblyomma triste*	1	6	6
Amblyomma triste	*Amblyomma triste*	1	6	6
Amblyomma triste	*Amblyomma triste*	1	6	6
Amblyomma triste	*Amblyomma triste*	1	6	6
Amblyomma triste	*Amblyomma triste*	1	6	6
Amblyomma triste	*Amblyomma triste*	1	6	6
Amblyomma triste	*Amblyomma triste*	1	6	6
Amblyomma triste	*Amblyomma triste*	1	6	6
Amblyomma triste	*Amblyomma aureolatum*	0	6	1
Amblyomma triste	*Amblyomma triste*	1	6	6
Amblyomma triste	*Amblyomma triste*	1	6	6
Amblyomma aureolatum	*Amblyomma aureolatum*	1	1	1
Amblyomma aureolatum	*Amblyomma aureolatum*	1	1	1
Amblyomma aureolatum	*Amblyomma aureolatum*	1	1	1
Amblyomma aureolatum	*Amblyomma aureolatum*	1	1	1
Amblyomma aureolatum	*Amblyomma aureolatum*	1	1	1
Amblyomma aureolatum	*Amblyomma aureolatum*	1	1	1
Amblyomma aureolatum	*Amblyomma aureolatum*	1	1	1
Amblyomma aureolatum	*Amblyomma aureolatum*	1	1	1

Amblyomma aureolatum	*Amblyomma aureolatum*	1	1	1
Amblyomma aureolatum	*Amblyomma aureolatum*	1	1	1
Amblyomma aureolatum	*Amblyomma aureolatum*	1	1	1
Amblyomma aureolatum	*Amblyomma aureolatum*	1	1	1
Amblyomma cajennense	*Amblyomma cajennense*	1	2	2
Amblyomma cajennense	*Amblyomma cajennense*	1	2	2
Amblyomma cajennense	*Amblyomma cajennense*	1	2	2
Amblyomma cajennense	*Amblyomma cajennense*	1	2	2
Amblyomma cajennense	*Amblyomma cajennense*	1	2	2
Amblyomma cajennense	*Amblyomma cajennense*	1	2	2
Amblyomma cajennense	*Amblyomma cajennense*	1	2	2
Amblyomma cajennense	*Amblyomma cajennense*	1	2	2
Amblyomma cajennense	*Amblyomma cajennense*	1	2	2
Amblyomma cajennense	*Amblyomma cajennense*	1	2	2
Amblyomma cajennense	*Amblyomma cajennense*	1	2	2
Amblyomma cajennense	*Amblyomma cajennense*	1	2	2
Amblyomma cajennense	*Amblyomma cajennense*	1	2	2
Amblyomma cajennense	*Amblyomma cajennense*	1	2	2
Amblyomma cajennense	*Amblyomma cajennense*	1	2	2
Amblyomma cajennense	*Amblyomma cajennense*	1	2	2
Amblyomma dubitatum	*Amblyomma dubitatum*	1	3	3
Amblyomma dubitatum	*Amblyomma dubitatum*	1	3	3
Amblyomma dubitatum	*Amblyomma dubitatum*	1	3	3
Amblyomma dubitatum	*Amblyomma dubitatum*	1	3	3
Amblyomma dubitatum	*Amblyomma dubitatum*	1	3	3
Amblyomma dubitatum	*Amblyomma dubitatum*	1	3	3
Amblyomma dubitatum	*Amblyomma cajennense*	0	3	2
Amblyomma dubitatum	*Amblyomma dubitatum*	1	3	3
Amblyomma dubitatum	*Amblyomma dubitatum*	1	3	3
Amblyomma dubitatum	*Amblyomma dubitatum*	1	3	3
Amblyomma dubitatum	*Amblyomma dubitatum*	1	3	3
Amblyomma ovale	*Amblyomma aureolatum*	0	4	1
Amblyomma ovale	*Amblyomma ovale*	1	4	4
Amblyomma ovale	*Amblyomma ovale*	1	4	4
Amblyomma ovale	*Amblyomma ovale*	1	4	4
Amblyomma ovale	*Amblyomma ovale*	1	4	4
Amblyomma ovale	*Amblyomma ovale*	1	4	4
Amblyomma ovale	*Amblyomma sculptum*	0	4	5
Amblyomma ovale	*Amblyomma ovale*	1	4	4
Amblyomma ovale	*Amblyomma ovale*	1	4	4
Amblyomma ovale	*Amblyomma ovale*	1	4	4
Amblyomma ovale	*Amblyomma ovale*	1	4	4
Amblyomma ovale	*Amblyomma ovale*	1	4	4
Amblyomma sculptum	*Amblyomma ovale*	0	5	4
Amblyomma sculptum	*Amblyomma sculptum*	1	5	5
Amblyomma sculptum	*Amblyomma ovale*	0	5	4
Amblyomma sculptum	*Amblyomma sculptum*	1	5	5
Amblyomma sculptum	*Amblyomma sculptum*	1	5	5
Amblyomma sculptum	*Amblyomma dubitatum*	0	5	3
Amblyomma sculptum	*Amblyomma sculptum*	1	5	5
Amblyomma sculptum	*Amblyomma sculptum*	1	5	5
Amblyomma sculptum	*Amblyomma sculptum*	1	5	5
Amblyomma sculptum	*Amblyomma sculptum*	1	5	5
Amblyomma sculptum	*Amblyomma sculptum*	1	5	5
Amblyomma sculptum	*Amblyomma sculptum*	1	5	5
Amblyomma sculptum	*Amblyomma sculptum*	1	5	5
Amblyomma sculptum	*Amblyomma sculptum*	1	5	5
Amblyomma triste	*Amblyomma triste*	1	6	6
Amblyomma triste	*Amblyomma triste*	1	6	6
Amblyomma triste	*Amblyomma triste*	1	6	6
Amblyomma triste	*Amblyomma triste*	1	6	6
Amblyomma triste	*Amblyomma aureolatum*	0	6	1
Amblyomma triste	*Amblyomma triste*	1	6	6
Amblyomma triste	*Amblyomma triste*	1	6	6
Amblyomma triste	*Amblyomma aureolatum*	0	6	1
Amblyomma triste	*Amblyomma triste*	1	6	6
Amblyomma triste	*Amblyomma triste*	1	6	6

Amblyomma triste	*Amblyomma triste*	1	6	6
Amblyomma triste	*Amblyomma triste*	1	6	6
Amblyomma triste	*Amblyomma triste*	1	6	6
Amblyomma triste	*Amblyomma triste*	1	6	6
Amblyomma triste	*Amblyomma triste*	1	6	6
Amblyomma aureolatum	*Amblyomma aureolatum*	1	1	1
Amblyomma aureolatum	*Amblyomma aureolatum*	1	1	1
Amblyomma aureolatum	*Amblyomma aureolatum*	1	1	1
Amblyomma aureolatum	*Amblyomma aureolatum*	1	1	1
Amblyomma aureolatum	*Amblyomma ovale*	0	1	4
Amblyomma aureolatum	*Amblyomma aureolatum*	1	1	1
Amblyomma aureolatum	*Amblyomma aureolatum*	1	1	1
Amblyomma aureolatum	*Amblyomma aureolatum*	1	1	1
Amblyomma aureolatum	*Amblyomma aureolatum*	1	1	1
Amblyomma aureolatum	*Amblyomma aureolatum*	1	1	1
Amblyomma aureolatum	*Amblyomma aureolatum*	1	1	1
Amblyomma cajennense	*Amblyomma cajennense*	1	2	2
Amblyomma cajennense	*Amblyomma cajennense*	1	2	2
Amblyomma cajennense	*Amblyomma cajennense*	1	2	2
Amblyomma cajennense	*Amblyomma cajennense*	1	2	2
Amblyomma cajennense	*Amblyomma cajennense*	1	2	2
Amblyomma cajennense	*Amblyomma cajennense*	1	2	2
Amblyomma cajennense	*Amblyomma triste*	0	2	6
Amblyomma cajennense	*Amblyomma triste*	0	2	6
Amblyomma cajennense	*Amblyomma cajennense*	1	2	2
Amblyomma cajennense	*Amblyomma cajennense*	1	2	2
Amblyomma cajennense	*Amblyomma cajennense*	1	2	2
Amblyomma cajennense	*Amblyomma cajennense*	1	2	2
Amblyomma cajennense	*Amblyomma cajennense*	1	2	2
Amblyomma cajennense	*Amblyomma cajennense*	1	2	2
Amblyomma cajennense	*Amblyomma cajennense*	1	2	2
Amblyomma cajennense	*Amblyomma cajennense*	1	2	2
Amblyomma dubitatum	*Amblyomma dubitatum*	1	3	3
Amblyomma dubitatum	*Amblyomma dubitatum*	1	3	3
Amblyomma dubitatum	*Amblyomma ovale*	0	3	4
Amblyomma dubitatum	*Amblyomma dubitatum*	1	3	3
Amblyomma dubitatum	*Amblyomma dubitatum*	1	3	3
Amblyomma dubitatum	*Amblyomma dubitatum*	1	3	3
Amblyomma dubitatum	*Amblyomma sculptum*	0	3	5
Amblyomma dubitatum	*Amblyomma dubitatum*	1	3	3
Amblyomma dubitatum	*Amblyomma triste*	0	3	6
Amblyomma dubitatum	*Amblyomma dubitatum*	1	3	3
Amblyomma ovale	*Amblyomma ovale*	1	4	4
Amblyomma ovale	*Amblyomma ovale*	1	4	4
Amblyomma ovale	*Amblyomma ovale*	1	4	4
Amblyomma ovale	*Amblyomma ovale*	1	4	4
Amblyomma ovale	*Amblyomma ovale*	1	4	4
Amblyomma ovale	*Amblyomma triste*	0	4	6
Amblyomma ovale	*Amblyomma ovale*	1	4	4
Amblyomma ovale	*Amblyomma ovale*	1	4	4
Amblyomma ovale	*Amblyomma ovale*	1	4	4
Amblyomma ovale	*Amblyomma aureolatum*	0	4	1
Amblyomma ovale	*Amblyomma ovale*	1	4	4
Amblyomma ovale	*Amblyomma ovale*	1	4	4
Amblyomma sculptum	*Amblyomma sculptum*	1	5	5
Amblyomma sculptum	*Amblyomma sculptum*	1	5	5
Amblyomma sculptum	*Amblyomma sculptum*	1	5	5
Amblyomma sculptum	*Amblyomma sculptum*	1	5	5
Amblyomma sculptum	*Amblyomma dubitatum*	0	5	3
Amblyomma sculptum	*Amblyomma sculptum*	1	5	5
Amblyomma sculptum	*Amblyomma sculptum*	1	5	5
Amblyomma sculptum	*Amblyomma sculptum*	1	5	5
Amblyomma sculptum	*Amblyomma sculptum*	1	5	5
Amblyomma sculptum	*Amblyomma sculptum*	1	5	5
Amblyomma sculptum	*Amblyomma sculptum*	1	5	5
Amblyomma sculptum	*Amblyomma sculptum*	1	5	5
Amblyomma sculptum	*Amblyomma sculptum*	1	5	5

Amblyomma sculptum	*Amblyomma sculptum*	1	5	5
Amblyomma sculptum	*Amblyomma sculptum*	1	5	5
Amblyomma triste	*Amblyomma triste*	1	6	6
Amblyomma triste	*Amblyomma ovale*	0	6	4
Amblyomma triste	*Amblyomma triste*	1	6	6
Amblyomma triste	*Amblyomma triste*	1	6	6
Amblyomma triste	*Amblyomma triste*	1	6	6
Amblyomma triste	*Amblyomma triste*	1	6	6
Amblyomma triste	*Amblyomma triste*	1	6	6
Amblyomma triste	*Amblyomma triste*	1	6	6
Amblyomma triste	*Amblyomma triste*	1	6	6
Amblyomma triste	*Amblyomma triste*	1	6	6
Amblyomma triste	*Amblyomma triste*	1	6	6
Amblyomma triste	*Amblyomma triste*	1	6	6
Amblyomma triste	*Amblyomma triste*	1	6	6
Amblyomma triste	*Amblyomma triste*	1	6	6
Amblyomma triste	*Amblyomma triste*	1	6	6
Amblyomma aureolatum	*Amblyomma aureolatum*	1	1	1
Amblyomma aureolatum	*Amblyomma aureolatum*	1	1	1
Amblyomma aureolatum	*Amblyomma aureolatum*	1	1	1
Amblyomma aureolatum	*Amblyomma triste*	0	1	6
Amblyomma aureolatum	*Amblyomma aureolatum*	1	1	1
Amblyomma aureolatum	*Amblyomma aureolatum*	1	1	1
Amblyomma aureolatum	*Amblyomma aureolatum*	1	1	1
Amblyomma aureolatum	*Amblyomma aureolatum*	1	1	1
Amblyomma aureolatum	*Amblyomma aureolatum*	1	1	1
Amblyomma aureolatum	*Amblyomma aureolatum*	1	1	1
Amblyomma aureolatum	*Amblyomma aureolatum*	1	1	1
Amblyomma aureolatum	*Amblyomma cajennense*	0	1	2
Amblyomma cajennense	*Amblyomma cajennense*	1	2	2
Amblyomma cajennense	*Amblyomma cajennense*	1	2	2
Amblyomma cajennense	*Amblyomma cajennense*	1	2	2
Amblyomma cajennense	*Amblyomma cajennense*	1	2	2
Amblyomma cajennense	*Amblyomma cajennense*	1	2	2
Amblyomma cajennense	*Amblyomma triste*	0	2	6
Amblyomma cajennense	*Amblyomma cajennense*	1	2	2
Amblyomma cajennense	*Amblyomma cajennense*	1	2	2
Amblyomma cajennense	*Amblyomma cajennense*	1	2	2
Amblyomma cajennense	*Amblyomma cajennense*	1	2	2
Amblyomma cajennense	*Amblyomma cajennense*	1	2	2
Amblyomma cajennense	*Amblyomma cajennense*	1	2	2
Amblyomma cajennense	*Amblyomma cajennense*	1	2	2
Amblyomma cajennense	*Amblyomma cajennense*	1	2	2
Amblyomma cajennense	*Amblyomma cajennense*	1	2	2
Amblyomma cajennense	*Amblyomma cajennense*	1	2	2
Amblyomma cajennense	*Amblyomma cajennense*	1	2	2
Amblyomma dubitatum	*Amblyomma ovale*	0	3	4
Amblyomma dubitatum	*Amblyomma dubitatum*	1	3	3
Amblyomma dubitatum	*Amblyomma dubitatum*	1	3	3
Amblyomma dubitatum	*Amblyomma dubitatum*	1	3	3
Amblyomma dubitatum	*Amblyomma dubitatum*	1	3	3
Amblyomma dubitatum	*Amblyomma dubitatum*	1	3	3
Amblyomma dubitatum	*Amblyomma aureolatum*	0	3	1
Amblyomma dubitatum	*Amblyomma dubitatum*	1	3	3
Amblyomma dubitatum	*Amblyomma dubitatum*	1	3	3
Amblyomma dubitatum	*Amblyomma sculptum*	0	3	5
Amblyomma dubitatum	*Amblyomma dubitatum*	1	3	3
Amblyomma ovale	*Amblyomma ovale*	1	4	4
Amblyomma ovale	*Amblyomma ovale*	1	4	4
Amblyomma ovale	*Amblyomma ovale*	1	4	4
Amblyomma ovale	*Amblyomma triste*	0	4	6
Amblyomma ovale	*Amblyomma ovale*	1	4	4
Amblyomma ovale	*Amblyomma ovale*	1	4	4
Amblyomma ovale	*Amblyomma ovale*	1	4	4
Amblyomma ovale	*Amblyomma ovale*	1	4	4
Amblyomma ovale	*Amblyomma ovale*	1	4	4
Amblyomma ovale	*Amblyomma ovale*	1	4	4

Amblyomma ovale	*Amblyomma sculptum*	0	4	5
Amblyomma ovale	*Amblyomma ovale*	1	4	4
Amblyomma sculptum	*Amblyomma sculptum*	1	5	5
Amblyomma sculptum	*Amblyomma triste*	0	5	6
Amblyomma sculptum	*Amblyomma sculptum*	1	5	5
Amblyomma sculptum	*Amblyomma sculptum*	1	5	5
Amblyomma sculptum	*Amblyomma sculptum*	1	5	5
Amblyomma sculptum	*Amblyomma sculptum*	1	5	5
Amblyomma sculptum	*Amblyomma sculptum*	1	5	5
Amblyomma sculptum	*Amblyomma sculptum*	1	5	5
Amblyomma sculptum	*Amblyomma sculptum*	1	5	5
Amblyomma sculptum	*Amblyomma sculptum*	1	5	5
Amblyomma sculptum	*Amblyomma sculptum*	1	5	5
Amblyomma sculptum	*Amblyomma sculptum*	1	5	5
Amblyomma sculptum	*Amblyomma sculptum*	1	5	5
Amblyomma sculptum	*Amblyomma sculptum*	1	5	5
Amblyomma sculptum	*Amblyomma sculptum*	1	5	5
Amblyomma triste	*Amblyomma triste*	1	6	6
Amblyomma triste	*Amblyomma triste*	1	6	6
Amblyomma triste	*Amblyomma cajennense*	0	6	2
Amblyomma triste	*Amblyomma triste*	1	6	6
Amblyomma triste	*Amblyomma triste*	1	6	6
Amblyomma triste	*Amblyomma triste*	1	6	6
Amblyomma triste	*Amblyomma triste*	1	6	6
Amblyomma triste	*Amblyomma triste*	1	6	6
Amblyomma triste	*Amblyomma triste*	1	6	6
Amblyomma triste	*Amblyomma cajennense*	0	6	2
Amblyomma triste	*Amblyomma triste*	1	6	6
Amblyomma triste	*Amblyomma triste*	1	6	6
Amblyomma triste	*Amblyomma triste*	1	6	6
Amblyomma triste	*Amblyomma triste*	1	6	6
Amblyomma triste	*Amblyomma triste*	1	6	6
Amblyomma triste	*Amblyomma triste*	1	6	6
Amblyomma aureolatum	*Amblyomma aureolatum*	1	1	1
Amblyomma aureolatum	*Amblyomma aureolatum*	1	1	1
Amblyomma aureolatum	*Amblyomma aureolatum*	1	1	1
Amblyomma aureolatum	*Amblyomma aureolatum*	1	1	1
Amblyomma aureolatum	*Amblyomma dubitatum*	0	1	3
Amblyomma aureolatum	*Amblyomma aureolatum*	1	1	1
Amblyomma aureolatum	*Amblyomma aureolatum*	1	1	1
Amblyomma aureolatum	*Amblyomma aureolatum*	1	1	1
Amblyomma aureolatum	*Amblyomma aureolatum*	1	1	1
Amblyomma aureolatum	*Amblyomma aureolatum*	1	1	1
Amblyomma aureolatum	*Amblyomma aureolatum*	1	1	1
Amblyomma cajennense	*Amblyomma cajennense*	1	2	2
Amblyomma cajennense	*Amblyomma cajennense*	1	2	2
Amblyomma cajennense	*Amblyomma cajennense*	1	2	2
Amblyomma cajennense	*Amblyomma cajennense*	1	2	2
Amblyomma cajennense	*Amblyomma cajennense*	1	2	2
Amblyomma cajennense	*Amblyomma cajennense*	1	2	2
Amblyomma cajennense	*Amblyomma cajennense*	1	2	2
Amblyomma cajennense	*Amblyomma cajennense*	1	2	2
Amblyomma cajennense	*Amblyomma cajennense*	1	2	2
Amblyomma cajennense	*Amblyomma cajennense*	1	2	2
Amblyomma cajennense	*Amblyomma cajennense*	1	2	2
Amblyomma cajennense	*Amblyomma cajennense*	1	2	2
Amblyomma cajennense	*Amblyomma cajennense*	1	2	2
Amblyomma cajennense	*Amblyomma cajennense*	1	2	2
Amblyomma cajennense	*Amblyomma cajennense*	1	2	2
Amblyomma cajennense	*Amblyomma cajennense*	1	2	2
Amblyomma cajennense	*Amblyomma cajennense*	1	2	2
Amblyomma dubitatum	*Amblyomma dubitatum*	1	3	3
Amblyomma dubitatum	*Amblyomma dubitatum*	1	3	3
Amblyomma dubitatum	*Amblyomma dubitatum*	1	3	3
Amblyomma dubitatum	*Amblyomma dubitatum*	1	3	3
Amblyomma dubitatum	*Amblyomma dubitatum*	1	3	3
Amblyomma dubitatum	*Amblyomma dubitatum*	1	3	3

Amblyomma dubitatum	*Amblyomma dubitatum*	1	3	3
Amblyomma dubitatum	*Amblyomma dubitatum*	1	3	3
Amblyomma dubitatum	*Amblyomma dubitatum*	1	3	3
Amblyomma dubitatum	*Amblyomma dubitatum*	1	3	3
Amblyomma ovale	*Amblyomma ovale*	1	4	4
Amblyomma ovale	*Amblyomma sculptum*	0	4	5
Amblyomma ovale	*Amblyomma ovale*	1	4	4
Amblyomma ovale	*Amblyomma ovale*	1	4	4
Amblyomma ovale	*Amblyomma ovale*	1	4	4
Amblyomma ovale	*Amblyomma ovale*	1	4	4
Amblyomma ovale	*Amblyomma aureolatum*	0	4	1
Amblyomma ovale	*Amblyomma ovale*	1	4	4
Amblyomma ovale	*Amblyomma ovale*	1	4	4
Amblyomma ovale	*Amblyomma sculptum*	0	4	5
Amblyomma ovale	*Amblyomma ovale*	1	4	4
Amblyomma ovale	*Amblyomma ovale*	1	4	4
Amblyomma sculptum	*Amblyomma sculptum*	1	5	5
Amblyomma sculptum	*Amblyomma sculptum*	1	5	5
Amblyomma sculptum	*Amblyomma sculptum*	1	5	5
Amblyomma sculptum	*Amblyomma sculptum*	1	5	5
Amblyomma sculptum	*Amblyomma sculptum*	1	5	5
Amblyomma sculptum	*Amblyomma sculptum*	1	5	5
Amblyomma sculptum	*Amblyomma sculptum*	1	5	5
Amblyomma sculptum	*Amblyomma sculptum*	1	5	5
Amblyomma sculptum	*Amblyomma sculptum*	1	5	5
Amblyomma sculptum	*Amblyomma sculptum*	1	5	5
Amblyomma sculptum	*Amblyomma sculptum*	1	5	5
Amblyomma sculptum	*Amblyomma sculptum*	1	5	5
Amblyomma sculptum	*Amblyomma sculptum*	1	5	5
Amblyomma sculptum	*Amblyomma sculptum*	1	5	5
Amblyomma sculptum	*Amblyomma sculptum*	1	5	5
Amblyomma triste	*Amblyomma triste*	1	6	6
Amblyomma triste	*Amblyomma cajennense*	0	6	2
Amblyomma triste	*Amblyomma triste*	1	6	6
Amblyomma triste	*Amblyomma triste*	1	6	6
Amblyomma triste	*Amblyomma triste*	1	6	6
Amblyomma triste	*Amblyomma triste*	1	6	6
Amblyomma triste	*Amblyomma triste*	1	6	6
Amblyomma triste	*Amblyomma triste*	1	6	6
Amblyomma triste	*Amblyomma triste*	1	6	6
Amblyomma triste	*Amblyomma triste*	1	6	6
Amblyomma triste	*Amblyomma triste*	1	6	6
Amblyomma triste	*Amblyomma triste*	1	6	6
Amblyomma triste	*Amblyomma triste*	1	6	6
Amblyomma triste	*Amblyomma triste*	1	6	6
Amblyomma triste	*Amblyomma triste*	1	6	6
Amblyomma aureolatum	*Amblyomma aureolatum*	1	1	1
Amblyomma aureolatum	*Amblyomma triste*	0	1	6
Amblyomma aureolatum	*Amblyomma aureolatum*	1	1	1
Amblyomma aureolatum	*Amblyomma aureolatum*	1	1	1
Amblyomma aureolatum	*Amblyomma aureolatum*	1	1	1
Amblyomma aureolatum	*Amblyomma aureolatum*	1	1	1
Amblyomma aureolatum	*Amblyomma aureolatum*	1	1	1
Amblyomma aureolatum	*Amblyomma aureolatum*	1	1	1
Amblyomma aureolatum	*Amblyomma aureolatum*	1	1	1
Amblyomma aureolatum	*Amblyomma aureolatum*	1	1	1
Amblyomma cajennense	*Amblyomma cajennense*	1	2	2
Amblyomma cajennense	*Amblyomma cajennense*	1	2	2
Amblyomma cajennense	*Amblyomma cajennense*	1	2	2
Amblyomma cajennense	*Amblyomma cajennense*	1	2	2
Amblyomma cajennense	*Amblyomma cajennense*	1	2	2
Amblyomma cajennense	*Amblyomma cajennense*	1	2	2
Amblyomma cajennense	*Amblyomma cajennense*	1	2	2
Amblyomma cajennense	*Amblyomma cajennense*	1	2	2
Amblyomma cajennense	*Amblyomma triste*	0	2	6
Amblyomma cajennense	*Amblyomma cajennense*	1	2	2
Amblyomma cajennense	*Amblyomma cajennense*	1	2	2

Amblyomma cajennense	*Amblyomma cajennense*	1	2	2
Amblyomma cajennense	*Amblyomma cajennense*	1	2	2
Amblyomma cajennense	*Amblyomma cajennense*	1	2	2
Amblyomma cajennense	*Amblyomma cajennense*	1	2	2
Amblyomma cajennense	*Amblyomma triste*	0	2	6
Amblyomma dubitatum	*Amblyomma dubitatum*	1	3	3
Amblyomma dubitatum	*Amblyomma dubitatum*	1	3	3
Amblyomma dubitatum	*Amblyomma dubitatum*	1	3	3
Amblyomma dubitatum	*Amblyomma dubitatum*	1	3	3
Amblyomma dubitatum	*Amblyomma dubitatum*	1	3	3
Amblyomma dubitatum	*Amblyomma dubitatum*	1	3	3
Amblyomma dubitatum	*Amblyomma dubitatum*	1	3	3
Amblyomma dubitatum	*Amblyomma ovale*	0	3	4
Amblyomma ovale	*Amblyomma ovale*	1	4	4
Amblyomma ovale	*Amblyomma ovale*	1	4	4
Amblyomma ovale	*Amblyomma ovale*	1	4	4
Amblyomma ovale	*Amblyomma ovale*	1	4	4
Amblyomma ovale	*Amblyomma ovale*	1	4	4
Amblyomma ovale	*Amblyomma ovale*	1	4	4
Amblyomma ovale	*Amblyomma ovale*	1	4	4
Amblyomma ovale	*Amblyomma ovale*	1	4	4
Amblyomma ovale	*Amblyomma ovale*	1	4	4
Amblyomma ovale	*Amblyomma ovale*	1	4	4
Amblyomma ovale	*Amblyomma ovale*	1	4	4
Amblyomma sculptum	*Amblyomma sculptum*	1	5	5
Amblyomma sculptum	*Amblyomma sculptum*	1	5	5
Amblyomma sculptum	*Amblyomma sculptum*	1	5	5
Amblyomma sculptum	*Amblyomma sculptum*	1	5	5
Amblyomma triste	*Amblyomma triste*	1	6	6
Amblyomma triste	*Amblyomma triste*	1	6	6
Amblyomma triste	*Amblyomma triste*	1	6	6
Amblyomma triste	*Amblyomma triste*	1	6	6
Amblyomma triste	*Amblyomma triste*	1	6	6
Amblyomma triste	*Amblyomma triste*	1	6	6
Amblyomma triste	*Amblyomma triste*	1	6	6
Amblyomma triste	*Amblyomma triste*	1	6	6
Amblyomma triste	*Amblyomma triste*	1	6	6
Amblyomma triste	*Amblyomma triste*	1	6	6
Amblyomma triste	*Amblyomma triste*	1	6	6
Amblyomma triste	*Amblyomma triste*	1	6	6
Amblyomma triste	*Amblyomma triste*	1	6	6
Amblyomma triste	*Amblyomma triste*	1	6	6
Amblyomma triste	*Amblyomma triste*	1	6	6
Amblyomma triste	*Amblyomma triste*	1	6	6
Amblyomma aureolatum	*Amblyomma ovale*	0	1	4
Amblyomma aureolatum	*Amblyomma aureolatum*	1	1	1
Amblyomma aureolatum	*Amblyomma sculptum*	0	1	5
Amblyomma aureolatum	*Amblyomma aureolatum*	1	1	1
Amblyomma aureolatum	*Amblyomma aureolatum*	1	1	1
Amblyomma aureolatum	*Amblyomma aureolatum*	1	1	1
Amblyomma aureolatum	*Amblyomma aureolatum*	1	1	1
Amblyomma aureolatum	*Amblyomma dubitatum*	0	1	3
Amblyomma aureolatum	*Amblyomma aureolatum*	1	1	1
Amblyomma aureolatum	*Amblyomma ovale*	0	1	4
Amblyomma aureolatum	*Amblyomma aureolatum*	1	1	1
Amblyomma cajennense	*Amblyomma dubitatum*	0	2	3
Amblyomma cajennense	*Amblyomma cajennense*	1	2	2
Amblyomma cajennense	*Amblyomma cajennense*	1	2	2
Amblyomma cajennense	*Amblyomma cajennense*	1	2	2
Amblyomma cajennense	*Amblyomma cajennense*	1	2	2
Amblyomma cajennense	*Amblyomma cajennense*	1	2	2
Amblyomma cajennense	*Amblyomma cajennense*	1	2	2
Amblyomma cajennense	*Amblyomma cajennense*	1	2	2
Amblyomma cajennense	*Amblyomma cajennense*	1	2	2
Amblyomma cajennense	*Amblyomma cajennense*	1	2	2
Amblyomma cajennense	*Amblyomma cajennense*	1	2	2
Amblyomma cajennense	*Amblyomma cajennense*	1	2	2

Amblyomma cajennense	*Amblyomma cajennense*	1	2	2
Amblyomma cajennense	*Amblyomma cajennense*	1	2	2
Amblyomma cajennense	*Amblyomma cajennense*	1	2	2
Amblyomma cajennense	*Amblyomma cajennense*	1	2	2
Amblyomma dubitatum	*Amblyomma dubitatum*	1	3	3
Amblyomma dubitatum	*Amblyomma dubitatum*	1	3	3
Amblyomma dubitatum	*Amblyomma dubitatum*	1	3	3
Amblyomma dubitatum	*Amblyomma dubitatum*	1	3	3
Amblyomma dubitatum	*Amblyomma dubitatum*	1	3	3
Amblyomma dubitatum	*Amblyomma dubitatum*	1	3	3
Amblyomma dubitatum	*Amblyomma dubitatum*	1	3	3
Amblyomma dubitatum	*Amblyomma dubitatum*	1	3	3
Amblyomma dubitatum	*Amblyomma dubitatum*	1	3	3
Amblyomma ovale	*Amblyomma ovale*	1	4	4
Amblyomma ovale	*Amblyomma ovale*	1	4	4
Amblyomma ovale	*Amblyomma ovale*	1	4	4
Amblyomma ovale	*Amblyomma ovale*	1	4	4
Amblyomma ovale	*Amblyomma ovale*	1	4	4
Amblyomma ovale	*Amblyomma ovale*	1	4	4
Amblyomma ovale	*Amblyomma ovale*	1	4	4
Amblyomma ovale	*Amblyomma ovale*	1	4	4
Amblyomma ovale	*Amblyomma ovale*	1	4	4
Amblyomma ovale	*Amblyomma ovale*	1	4	4
Amblyomma ovale	*Amblyomma ovale*	1	4	4
Amblyomma sculptum	*Amblyomma sculptum*	1	5	5
Amblyomma sculptum	*Amblyomma sculptum*	1	5	5
Amblyomma sculptum	*Amblyomma sculptum*	1	5	5
Amblyomma triste	*Amblyomma triste*	1	6	6
Amblyomma triste	*Amblyomma triste*	1	6	6
Amblyomma triste	*Amblyomma triste*	1	6	6
Amblyomma triste	*Amblyomma triste*	1	6	6
Amblyomma triste	*Amblyomma triste*	1	6	6
Amblyomma triste	*Amblyomma triste*	1	6	6
Amblyomma triste	*Amblyomma triste*	1	6	6
Amblyomma triste	*Amblyomma triste*	1	6	6
Amblyomma triste	*Amblyomma triste*	1	6	6
Amblyomma triste	*Amblyomma triste*	1	6	6
Amblyomma triste	*Amblyomma triste*	1	6	6
Amblyomma triste	*Amblyomma triste*	1	6	6
Amblyomma triste	*Amblyomma triste*	1	6	6
Amblyomma triste	*Amblyomma triste*	1	6	6
Amblyomma triste	*Amblyomma triste*	1	6	6
Amblyomma triste	*Amblyomma triste*	1	6	6
Amblyomma aureolatum	*Amblyomma aureolatum*	1	1	1
Amblyomma aureolatum	*Amblyomma aureolatum*	1	1	1
Amblyomma aureolatum	*Amblyomma dubitatum*	0	1	3
Amblyomma aureolatum	*Amblyomma aureolatum*	1	1	1
Amblyomma aureolatum	*Amblyomma aureolatum*	1	1	1
Amblyomma aureolatum	*Amblyomma aureolatum*	1	1	1
Amblyomma aureolatum	*Amblyomma aureolatum*	1	1	1
Amblyomma aureolatum	*Amblyomma aureolatum*	1	1	1
Amblyomma aureolatum	*Amblyomma aureolatum*	1	1	1
Amblyomma aureolatum	*Amblyomma aureolatum*	1	1	1
Amblyomma cajennense	*Amblyomma cajennense*	1	2	2
Amblyomma cajennense	*Amblyomma cajennense*	1	2	2
Amblyomma cajennense	*Amblyomma cajennense*	1	2	2
Amblyomma cajennense	*Amblyomma cajennense*	1	2	2
Amblyomma cajennense	*Amblyomma cajennense*	1	2	2
Amblyomma cajennense	*Amblyomma cajennense*	1	2	2
Amblyomma cajennense	*Amblyomma cajennense*	1	2	2
Amblyomma cajennense	*Amblyomma cajennense*	1	2	2
Amblyomma cajennense	*Amblyomma cajennense*	1	2	2
Amblyomma cajennense	*Amblyomma cajennense*	1	2	2
Amblyomma cajennense	*Amblyomma cajennense*	1	2	2
Amblyomma cajennense	*Amblyomma cajennense*	1	2	2
Amblyomma cajennense	*Amblyomma cajennense*	1	2	2
Amblyomma cajennense	*Amblyomma cajennense*	1	2	2

Amblyomma cajennense	*Amblyomma cajennense*	1	2	2
Amblyomma cajennense	*Amblyomma cajennense*	1	2	2
Amblyomma cajennense	*Amblyomma cajennense*	1	2	2
Amblyomma dubitatum	*Amblyomma dubitatum*	1	3	3
Amblyomma dubitatum	*Amblyomma dubitatum*	1	3	3
Amblyomma dubitatum	*Amblyomma dubitatum*	1	3	3
Amblyomma dubitatum	*Amblyomma dubitatum*	1	3	3
Amblyomma dubitatum	*Amblyomma dubitatum*	1	3	3
Amblyomma dubitatum	*Amblyomma dubitatum*	1	3	3
Amblyomma dubitatum	*Amblyomma ovale*	0	3	4
Amblyomma dubitatum	*Amblyomma dubitatum*	1	3	3
Amblyomma dubitatum	*Amblyomma dubitatum*	1	3	3
Amblyomma ovale	*Amblyomma ovale*	1	4	4
Amblyomma ovale	*Amblyomma ovale*	1	4	4
Amblyomma ovale	*Amblyomma ovale*	1	4	4
Amblyomma ovale	*Amblyomma ovale*	1	4	4
Amblyomma ovale	*Amblyomma ovale*	1	4	4
Amblyomma ovale	*Amblyomma aureolatum*	0	4	1
Amblyomma ovale	*Amblyomma ovale*	1	4	4
Amblyomma ovale	*Amblyomma ovale*	1	4	4
Amblyomma ovale	*Amblyomma ovale*	1	4	4
Amblyomma ovale	*Amblyomma ovale*	1	4	4
Amblyomma ovale	*Amblyomma ovale*	1	4	4
Amblyomma sculptum	*Amblyomma sculptum*	1	5	5
Amblyomma sculptum	*Amblyomma sculptum*	1	5	5
Amblyomma sculptum	*Amblyomma sculptum*	1	5	5
Amblyomma sculptum	*Amblyomma sculptum*	1	5	5
Amblyomma triste	*Amblyomma triste*	1	6	6
Amblyomma triste	*Amblyomma triste*	1	6	6
Amblyomma triste	*Amblyomma triste*	1	6	6
Amblyomma triste	*Amblyomma aureolatum*	0	6	1
Amblyomma triste	*Amblyomma triste*	1	6	6
Amblyomma triste	*Amblyomma triste*	1	6	6
Amblyomma triste	*Amblyomma triste*	1	6	6
Amblyomma triste	*Amblyomma triste*	1	6	6
Amblyomma triste	*Amblyomma triste*	1	6	6
Amblyomma triste	*Amblyomma triste*	1	6	6
Amblyomma triste	*Amblyomma triste*	1	6	6
Amblyomma triste	*Amblyomma triste*	1	6	6
Amblyomma triste	*Amblyomma triste*	1	6	6
Amblyomma triste	*Amblyomma triste*	1	6	6
Amblyomma triste	*Amblyomma triste*	1	6	6
Amblyomma aureolatum	*Amblyomma aureolatum*	1	1	1
Amblyomma aureolatum	*Amblyomma aureolatum*	1	1	1
Amblyomma aureolatum	*Amblyomma aureolatum*	1	1	1
Amblyomma aureolatum	*Amblyomma aureolatum*	1	1	1
Amblyomma aureolatum	*Amblyomma aureolatum*	1	1	1
Amblyomma aureolatum	*Amblyomma ovale*	0	1	4
Amblyomma aureolatum	*Amblyomma aureolatum*	1	1	1
Amblyomma aureolatum	*Amblyomma aureolatum*	1	1	1
Amblyomma aureolatum	*Amblyomma ovale*	0	1	4
Amblyomma aureolatum	*Amblyomma aureolatum*	1	1	1
Amblyomma aureolatum	*Amblyomma aureolatum*	1	1	1
Amblyomma cajennense	*Amblyomma cajennense*	1	2	2
Amblyomma cajennense	*Amblyomma cajennense*	1	2	2
Amblyomma cajennense	*Amblyomma cajennense*	1	2	2
Amblyomma cajennense	*Amblyomma cajennense*	1	2	2
Amblyomma cajennense	*Amblyomma cajennense*	1	2	2
Amblyomma cajennense	*Amblyomma cajennense*	1	2	2
Amblyomma cajennense	*Amblyomma cajennense*	1	2	2
Amblyomma cajennense	*Amblyomma cajennense*	1	2	2
Amblyomma cajennense	*Amblyomma cajennense*	1	2	2
Amblyomma cajennense	*Amblyomma triste*	0	2	6
Amblyomma cajennense	*Amblyomma cajennense*	1	2	2
Amblyomma cajennense	*Amblyomma cajennense*	1	2	2
Amblyomma cajennense	*Amblyomma cajennense*	1	2	2
Amblyomma cajennense	*Amblyomma cajennense*	1	2	2

Amblyomma cajennense	*Amblyomma cajennense*	1	2	2
Amblyomma cajennense	*Amblyomma cajennense*	1	2	2
Amblyomma dubitatum	*Amblyomma aureolatum*	0	3	1
Amblyomma dubitatum	*Amblyomma dubitatum*	1	3	3
Amblyomma dubitatum	*Amblyomma ovale*	0	3	4
Amblyomma dubitatum	*Amblyomma dubitatum*	1	3	3
Amblyomma dubitatum	*Amblyomma ovale*	0	3	4
Amblyomma dubitatum	*Amblyomma dubitatum*	1	3	3
Amblyomma dubitatum	*Amblyomma dubitatum*	1	3	3
Amblyomma dubitatum	*Amblyomma dubitatum*	1	3	3
Amblyomma dubitatum	*Amblyomma dubitatum*	1	3	3
Amblyomma ovale	*Amblyomma ovale*	1	4	4
Amblyomma ovale	*Amblyomma ovale*	1	4	4
Amblyomma ovale	*Amblyomma ovale*	1	4	4
Amblyomma ovale	*Amblyomma ovale*	1	4	4
Amblyomma ovale	*Amblyomma ovale*	1	4	4
Amblyomma ovale	*Amblyomma ovale*	1	4	4
Amblyomma ovale	*Amblyomma ovale*	1	4	4
Amblyomma ovale	*Amblyomma ovale*	1	4	4
Amblyomma ovale	*Amblyomma ovale*	1	4	4
Amblyomma ovale	*Amblyomma ovale*	1	4	4
Amblyomma ovale	*Amblyomma ovale*	1	4	4
Amblyomma sculptum	*Amblyomma sculptum*	1	5	5
Amblyomma sculptum	*Amblyomma sculptum*	1	5	5
Amblyomma sculptum	*Amblyomma dubitatum*	0	5	3
Amblyomma triste	*Amblyomma triste*	1	6	6
Amblyomma triste	*Amblyomma triste*	1	6	6
Amblyomma triste	*Amblyomma triste*	1	6	6
Amblyomma triste	*Amblyomma triste*	1	6	6
Amblyomma triste	*Amblyomma triste*	1	6	6
Amblyomma triste	*Amblyomma triste*	1	6	6
Amblyomma triste	*Amblyomma triste*	1	6	6
Amblyomma triste	*Amblyomma triste*	1	6	6
Amblyomma triste	*Amblyomma triste*	1	6	6
Amblyomma triste	*Amblyomma triste*	1	6	6
Amblyomma triste	*Amblyomma triste*	1	6	6
Amblyomma triste	*Amblyomma triste*	1	6	6
Amblyomma triste	*Amblyomma triste*	1	6	6
Amblyomma triste	*Amblyomma triste*	1	6	6
Amblyomma triste	*Amblyomma triste*	1	6	6
Amblyomma aureolatum	*Amblyomma aureolatum*	1	1	1
Amblyomma aureolatum	*Amblyomma aureolatum*	1	1	1
Amblyomma aureolatum	*Amblyomma aureolatum*	1	1	1
Amblyomma aureolatum	*Amblyomma aureolatum*	1	1	1
Amblyomma aureolatum	*Amblyomma aureolatum*	1	1	1
Amblyomma aureolatum	*Amblyomma aureolatum*	1	1	1
Amblyomma aureolatum	*Amblyomma aureolatum*	1	1	1
Amblyomma aureolatum	*Amblyomma aureolatum*	1	1	1
Amblyomma aureolatum	*Amblyomma aureolatum*	1	1	1
Amblyomma aureolatum	*Amblyomma aureolatum*	1	1	1
Amblyomma aureolatum	*Amblyomma aureolatum*	1	1	1
Amblyomma cajennense	*Amblyomma cajennense*	1	2	2
Amblyomma cajennense	*Amblyomma cajennense*	1	2	2
Amblyomma cajennense	*Amblyomma cajennense*	1	2	2
Amblyomma cajennense	*Amblyomma triste*	0	2	6
Amblyomma cajennense	*Amblyomma cajennense*	1	2	2
Amblyomma cajennense	*Amblyomma cajennense*	1	2	2
Amblyomma cajennense	*Amblyomma cajennense*	1	2	2
Amblyomma cajennense	*Amblyomma cajennense*	1	2	2
Amblyomma cajennense	*Amblyomma cajennense*	1	2	2
Amblyomma cajennense	*Amblyomma cajennense*	1	2	2
Amblyomma cajennense	*Amblyomma cajennense*	1	2	2
Amblyomma cajennense	*Amblyomma cajennense*	1	2	2
Amblyomma cajennense	*Amblyomma aureolatum*	0	2	1
Amblyomma cajennense	*Amblyomma cajennense*	1	2	2
Amblyomma cajennense	*Amblyomma cajennense*	1	2	2
Amblyomma cajennense	*Amblyomma cajennense*	1	2	2

Amblyomma cajennense	*Amblyomma cajennense*	1	2	2
Amblyomma dubitatum	*Amblyomma ovale*	0	3	4
Amblyomma dubitatum	*Amblyomma dubitatum*	1	3	3
Amblyomma dubitatum	*Amblyomma dubitatum*	1	3	3
Amblyomma dubitatum	*Amblyomma dubitatum*	1	3	3
Amblyomma dubitatum	*Amblyomma dubitatum*	1	3	3
Amblyomma dubitatum	*Amblyomma dubitatum*	1	3	3
Amblyomma dubitatum	*Amblyomma aureolatum*	0	3	1
Amblyomma dubitatum	*Amblyomma dubitatum*	1	3	3
Amblyomma dubitatum	*Amblyomma dubitatum*	1	3	3
Amblyomma ovale	*Amblyomma ovale*	1	4	4
Amblyomma ovale	*Amblyomma ovale*	1	4	4
Amblyomma ovale	*Amblyomma ovale*	1	4	4
Amblyomma ovale	*Amblyomma ovale*	1	4	4
Amblyomma ovale	*Amblyomma ovale*	1	4	4
Amblyomma ovale	*Amblyomma ovale*	1	4	4
Amblyomma ovale	*Amblyomma ovale*	1	4	4
Amblyomma ovale	*Amblyomma ovale*	1	4	4
Amblyomma ovale	*Amblyomma ovale*	1	4	4
Amblyomma ovale	*Amblyomma ovale*	1	4	4
Amblyomma ovale	*Amblyomma ovale*	1	4	4
Amblyomma sculptum	*Amblyomma sculptum*	1	5	5
Amblyomma sculptum	*Amblyomma sculptum*	1	5	5
Amblyomma sculptum	*Amblyomma sculptum*	1	5	5
Amblyomma triste	*Amblyomma ovale*	0	6	4
Amblyomma triste	*Amblyomma triste*	1	6	6
Amblyomma triste	*Amblyomma triste*	1	6	6
Amblyomma triste	*Amblyomma triste*	1	6	6
Amblyomma triste	*Amblyomma triste*	1	6	6
Amblyomma triste	*Amblyomma triste*	1	6	6
Amblyomma triste	*Amblyomma triste*	1	6	6
Amblyomma triste	*Amblyomma triste*	1	6	6
Amblyomma triste	*Amblyomma triste*	1	6	6
Amblyomma triste	*Amblyomma dubitatum*	0	6	3
Amblyomma triste	*Amblyomma triste*	1	6	6
Amblyomma triste	*Amblyomma triste*	1	6	6
Amblyomma triste	*Amblyomma triste*	1	6	6
Amblyomma triste	*Amblyomma triste*	1	6	6
Amblyomma triste	*Amblyomma triste*	1	6	6
Amblyomma aureolatum	*Amblyomma aureolatum*	1	1	1
Amblyomma aureolatum	*Amblyomma aureolatum*	1	1	1
Amblyomma aureolatum	*Amblyomma aureolatum*	1	1	1
Amblyomma aureolatum	*Amblyomma aureolatum*	1	1	1
Amblyomma aureolatum	*Amblyomma aureolatum*	1	1	1
Amblyomma aureolatum	*Amblyomma aureolatum*	1	1	1
Amblyomma aureolatum	*Amblyomma aureolatum*	1	1	1
Amblyomma aureolatum	*Amblyomma aureolatum*	1	1	1
Amblyomma aureolatum	*Amblyomma aureolatum*	1	1	1
Amblyomma aureolatum	*Amblyomma aureolatum*	1	1	1
Amblyomma aureolatum	*Amblyomma aureolatum*	1	1	1
Amblyomma aureolatum	*Amblyomma aureolatum*	1	1	1
Amblyomma aureolatum	*Amblyomma aureolatum*	1	1	1
Amblyomma cajennense	*Amblyomma cajennense*	1	2	2
Amblyomma cajennense	*Amblyomma cajennense*	1	2	2
Amblyomma cajennense	*Amblyomma cajennense*	1	2	2
Amblyomma cajennense	*Amblyomma cajennense*	1	2	2
Amblyomma cajennense	*Amblyomma cajennense*	1	2	2
Amblyomma cajennense	*Amblyomma cajennense*	1	2	2
Amblyomma cajennense	*Amblyomma cajennense*	1	2	2
Amblyomma cajennense	*Amblyomma cajennense*	1	2	2
Amblyomma cajennense	*Amblyomma cajennense*	1	2	2
Amblyomma cajennense	*Amblyomma cajennense*	1	2	2
Amblyomma cajennense	*Amblyomma cajennense*	1	2	2
Amblyomma cajennense	*Amblyomma cajennense*	1	2	2
Amblyomma cajennense	*Amblyomma cajennense*	1	2	2
Amblyomma cajennense	*Amblyomma cajennense*	1	2	2
Amblyomma cajennense	*Amblyomma cajennense*	1	2	2

Amblyomma cajennense	*Amblyomma cajennense*	1	2	2
Amblyomma cajennense	*Amblyomma cajennense*	1	2	2
Amblyomma dubitatum	*Amblyomma dubitatum*	1	3	3
Amblyomma dubitatum	*Amblyomma dubitatum*	1	3	3
Amblyomma dubitatum	*Amblyomma ovale*	0	3	4
Amblyomma dubitatum	*Amblyomma dubitatum*	1	3	3
Amblyomma dubitatum	*Amblyomma dubitatum*	1	3	3
Amblyomma dubitatum	*Amblyomma dubitatum*	1	3	3
Amblyomma dubitatum	*Amblyomma dubitatum*	1	3	3
Amblyomma dubitatum	*Amblyomma dubitatum*	1	3	3
Amblyomma dubitatum	*Amblyomma dubitatum*	1	3	3
Amblyomma dubitatum	*Amblyomma dubitatum*	1	3	3
Amblyomma dubitatum	*Amblyomma dubitatum*	1	3	3
Amblyomma dubitatum	*Amblyomma dubitatum*	1	3	3
Amblyomma dubitatum	*Amblyomma dubitatum*	1	3	3
Amblyomma ovale	*Amblyomma ovale*	1	4	4
Amblyomma ovale	*Amblyomma ovale*	1	4	4
Amblyomma ovale	*Amblyomma ovale*	1	4	4
Amblyomma ovale	*Amblyomma ovale*	1	4	4
Amblyomma ovale	*Amblyomma ovale*	1	4	4
Amblyomma ovale	*Amblyomma ovale*	1	4	4
Amblyomma ovale	*Amblyomma ovale*	1	4	4
Amblyomma ovale	*Amblyomma ovale*	1	4	4
Amblyomma ovale	*Amblyomma ovale*	1	4	4
Amblyomma ovale	*Amblyomma ovale*	1	4	4
Amblyomma ovale	*Amblyomma ovale*	1	4	4
Amblyomma ovale	*Amblyomma ovale*	1	4	4
Amblyomma ovale	*Amblyomma ovale*	1	4	4
Amblyomma sculptum	*Amblyomma sculptum*	1	5	5
Amblyomma sculptum	*Amblyomma sculptum*	1	5	5
Amblyomma sculptum	*Amblyomma sculptum*	1	5	5
Amblyomma sculptum	*Amblyomma sculptum*	1	5	5
Amblyomma sculptum	*Amblyomma sculptum*	1	5	5
Amblyomma sculptum	*Amblyomma sculptum*	1	5	5
Amblyomma sculptum	*Amblyomma sculptum*	1	5	5
Amblyomma sculptum	*Amblyomma sculptum*	1	5	5
Amblyomma sculptum	*Amblyomma sculptum*	1	5	5
Amblyomma sculptum	*Amblyomma sculptum*	1	5	5
Amblyomma sculptum	*Amblyomma sculptum*	1	5	5
Amblyomma sculptum	*Amblyomma sculptum*	1	5	5
Amblyomma sculptum	*Amblyomma sculptum*	1	5	5
Amblyomma sculptum	*Amblyomma sculptum*	1	5	5
Amblyomma sculptum	*Amblyomma sculptum*	1	5	5
Amblyomma sculptum	*Amblyomma sculptum*	1	5	5
Amblyomma sculptum	*Amblyomma sculptum*	1	5	5
Amblyomma sculptum	*Amblyomma sculptum*	1	5	5
Amblyomma sculptum	*Amblyomma dubitatum*	0	5	3
Amblyomma sculptum	*Amblyomma sculptum*	1	5	5
Amblyomma sculptum	*Amblyomma sculptum*	1	5	5
Amblyomma sculptum	*Amblyomma sculptum*	1	5	5
Amblyomma sculptum	*Amblyomma sculptum*	1	5	5
Amblyomma sculptum	*Amblyomma sculptum*	1	5	5
Amblyomma sculptum	*Amblyomma sculptum*	1	5	5
Amblyomma sculptum	*Amblyomma sculptum*	1	5	5
Amblyomma sculptum	*Amblyomma sculptum*	1	5	5
Amblyomma triste	*Amblyomma triste*	1	6	6
Amblyomma triste	*Amblyomma triste*	1	6	6
Amblyomma triste	*Amblyomma triste*	1	6	6
Amblyomma triste	*Amblyomma triste*	1	6	6
Amblyomma triste	*Amblyomma triste*	1	6	6
Amblyomma triste	*Amblyomma triste*	1	6	6
Amblyomma triste	*Amblyomma triste*	1	6	6
Amblyomma triste	*Amblyomma triste*	1	6	6
Amblyomma triste	*Amblyomma triste*	1	6	6
Amblyomma triste	*Amblyomma triste*	1	6	6
Amblyomma triste	*Amblyomma triste*	1	6	6
Amblyomma triste	*Amblyomma triste*	1	6	6

Amblyomma triste	*Amblyomma triste*	1	6	6
Amblyomma triste	*Amblyomma ovale*	0	6	4
Amblyomma triste	*Amblyomma ovale*	0	6	4
Amblyomma triste	*Amblyomma triste*	1	6	6
Amblyomma aureolatum	*Amblyomma aureolatum*	1	1	1
Amblyomma aureolatum	*Amblyomma aureolatum*	1	1	1
Amblyomma aureolatum	*Amblyomma aureolatum*	1	1	1
Amblyomma aureolatum	*Amblyomma aureolatum*	1	1	1
Amblyomma aureolatum	*Amblyomma aureolatum*	1	1	1
Amblyomma aureolatum	*Amblyomma aureolatum*	1	1	1
Amblyomma aureolatum	*Amblyomma dubitatum*	0	1	3
Amblyomma aureolatum	*Amblyomma ovale*	0	1	4
Amblyomma aureolatum	*Amblyomma aureolatum*	1	1	1
Amblyomma aureolatum	*Amblyomma aureolatum*	1	1	1
Amblyomma aureolatum	*Amblyomma aureolatum*	1	1	1
Amblyomma aureolatum	*Amblyomma aureolatum*	1	1	1
Amblyomma aureolatum	*Amblyomma aureolatum*	1	1	1
Amblyomma aureolatum	*Amblyomma aureolatum*	1	1	1
Amblyomma cajennense	*Amblyomma triste*	0	2	6
Amblyomma cajennense	*Amblyomma cajennense*	1	2	2
Amblyomma cajennense	*Amblyomma cajennense*	1	2	2
Amblyomma cajennense	*Amblyomma cajennense*	1	2	2
Amblyomma cajennense	*Amblyomma cajennense*	1	2	2
Amblyomma cajennense	*Amblyomma cajennense*	1	2	2
Amblyomma cajennense	*Amblyomma cajennense*	1	2	2
Amblyomma cajennense	*Amblyomma cajennense*	1	2	2
Amblyomma cajennense	*Amblyomma cajennense*	1	2	2
Amblyomma cajennense	*Amblyomma cajennense*	1	2	2
Amblyomma cajennense	*Amblyomma cajennense*	1	2	2
Amblyomma cajennense	*Amblyomma cajennense*	1	2	2
Amblyomma cajennense	*Amblyomma cajennense*	1	2	2
Amblyomma cajennense	*Amblyomma cajennense*	1	2	2
Amblyomma cajennense	*Amblyomma cajennense*	1	2	2
Amblyomma cajennense	*Amblyomma cajennense*	1	2	2
Amblyomma cajennense	*Amblyomma cajennense*	1	2	2
Amblyomma dubitatum	*Amblyomma ovale*	0	3	4
Amblyomma dubitatum	*Amblyomma ovale*	0	3	4
Amblyomma dubitatum	*Amblyomma dubitatum*	1	3	3
Amblyomma dubitatum	*Amblyomma dubitatum*	1	3	3
Amblyomma dubitatum	*Amblyomma dubitatum*	1	3	3
Amblyomma dubitatum	*Amblyomma dubitatum*	1	3	3
Amblyomma dubitatum	*Amblyomma aureolatum*	0	3	1
Amblyomma dubitatum	*Amblyomma dubitatum*	1	3	3
Amblyomma dubitatum	*Amblyomma dubitatum*	1	3	3
Amblyomma dubitatum	*Amblyomma dubitatum*	1	3	3
Amblyomma dubitatum	*Amblyomma dubitatum*	1	3	3
Amblyomma dubitatum	*Amblyomma dubitatum*	1	3	3
Amblyomma dubitatum	*Amblyomma dubitatum*	1	3	3
Amblyomma ovale	*Amblyomma ovale*	1	4	4
Amblyomma ovale	*Amblyomma ovale*	1	4	4
Amblyomma ovale	*Amblyomma ovale*	1	4	4
Amblyomma ovale	*Amblyomma ovale*	1	4	4
Amblyomma ovale	*Amblyomma ovale*	1	4	4
Amblyomma ovale	*Amblyomma aureolatum*	0	4	1
Amblyomma ovale	*Amblyomma aureolatum*	0	4	1
Amblyomma ovale	*Amblyomma ovale*	1	4	4
Amblyomma ovale	*Amblyomma ovale*	1	4	4
Amblyomma ovale	*Amblyomma ovale*	1	4	4
Amblyomma ovale	*Amblyomma ovale*	1	4	4
Amblyomma ovale	*Amblyomma ovale*	1	4	4
Amblyomma ovale	*Amblyomma ovale*	1	4	4
Amblyomma sculptum	*Amblyomma sculptum*	1	5	5
Amblyomma sculptum	*Amblyomma sculptum*	1	5	5
Amblyomma sculptum	*Amblyomma sculptum*	1	5	5
Amblyomma sculptum	*Amblyomma sculptum*	1	5	5
Amblyomma sculptum	*Amblyomma sculptum*	1	5	5
Amblyomma sculptum	*Amblyomma sculptum*	1	5	5

Amblyomma sculptum	*Amblyomma sculptum*	1	5	5
Amblyomma sculptum	*Amblyomma sculptum*	1	5	5
Amblyomma sculptum	*Amblyomma sculptum*	1	5	5
Amblyomma sculptum	*Amblyomma triste*	0	5	6
Amblyomma sculptum	*Amblyomma sculptum*	1	5	5
Amblyomma sculptum	*Amblyomma sculptum*	1	5	5
Amblyomma sculptum	*Amblyomma sculptum*	1	5	5
Amblyomma sculptum	*Amblyomma sculptum*	1	5	5
Amblyomma sculptum	*Amblyomma sculptum*	1	5	5
Amblyomma sculptum	*Amblyomma sculptum*	1	5	5
Amblyomma sculptum	*Amblyomma sculptum*	1	5	5
Amblyomma sculptum	*Amblyomma sculptum*	1	5	5
Amblyomma sculptum	*Amblyomma dubitatum*	0	5	3
Amblyomma sculptum	*Amblyomma sculptum*	1	5	5
Amblyomma sculptum	*Amblyomma sculptum*	1	5	5
Amblyomma sculptum	*Amblyomma sculptum*	1	5	5
Amblyomma sculptum	*Amblyomma sculptum*	1	5	5
Amblyomma sculptum	*Amblyomma sculptum*	1	5	5
Amblyomma sculptum	*Amblyomma sculptum*	1	5	5
Amblyomma sculptum	*Amblyomma sculptum*	1	5	5
Amblyomma sculptum	*Amblyomma sculptum*	1	5	5
Amblyomma sculptum	*Amblyomma sculptum*	1	5	5
Amblyomma triste	*Amblyomma triste*	1	6	6
Amblyomma triste	*Amblyomma triste*	1	6	6
Amblyomma triste	*Amblyomma triste*	1	6	6
Amblyomma triste	*Amblyomma triste*	1	6	6
Amblyomma triste	*Amblyomma triste*	1	6	6
Amblyomma triste	*Amblyomma triste*	1	6	6
Amblyomma triste	*Amblyomma triste*	1	6	6
Amblyomma triste	*Amblyomma triste*	1	6	6
Amblyomma triste	*Amblyomma triste*	1	6	6
Amblyomma triste	*Amblyomma triste*	1	6	6
Amblyomma triste	*Amblyomma triste*	1	6	6
Amblyomma triste	*Amblyomma triste*	1	6	6
Amblyomma triste	*Amblyomma triste*	1	6	6
Amblyomma triste	*Amblyomma triste*	1	6	6
Amblyomma triste	*Amblyomma triste*	1	6	6
Amblyomma triste	*Amblyomma aureolatum*	0	6	1
Amblyomma aureolatum	*Amblyomma aureolatum*	1	1	1
Amblyomma aureolatum	*Amblyomma aureolatum*	1	1	1
Amblyomma aureolatum	*Amblyomma aureolatum*	1	1	1
Amblyomma aureolatum	*Amblyomma aureolatum*	1	1	1
Amblyomma aureolatum	*Amblyomma aureolatum*	1	1	1
Amblyomma aureolatum	*Amblyomma aureolatum*	1	1	1
Amblyomma aureolatum	*Amblyomma aureolatum*	1	1	1
Amblyomma aureolatum	*Amblyomma aureolatum*	1	1	1
Amblyomma aureolatum	*Amblyomma aureolatum*	1	1	1
Amblyomma aureolatum	*Amblyomma aureolatum*	1	1	1
Amblyomma aureolatum	*Amblyomma aureolatum*	1	1	1
Amblyomma aureolatum	*Amblyomma aureolatum*	1	1	1
Amblyomma aureolatum	*Amblyomma aureolatum*	1	1	1
Amblyomma cajennense	*Amblyomma cajennense*	1	2	2
Amblyomma cajennense	*Amblyomma cajennense*	1	2	2
Amblyomma cajennense	*Amblyomma cajennense*	1	2	2
Amblyomma cajennense	*Amblyomma cajennense*	1	2	2
Amblyomma cajennense	*Amblyomma cajennense*	1	2	2
Amblyomma cajennense	*Amblyomma cajennense*	1	2	2
Amblyomma cajennense	*Amblyomma cajennense*	1	2	2
Amblyomma cajennense	*Amblyomma cajennense*	1	2	2
Amblyomma cajennense	*Amblyomma cajennense*	1	2	2
Amblyomma cajennense	*Amblyomma cajennense*	1	2	2
Amblyomma cajennense	*Amblyomma cajennense*	1	2	2
Amblyomma cajennense	*Amblyomma cajennense*	1	2	2
Amblyomma cajennense	*Amblyomma cajennense*	1	2	2
Amblyomma cajennense	*Amblyomma cajennense*	1	2	2
Amblyomma cajennense	*Amblyomma cajennense*	1	2	2
Amblyomma cajennense	*Amblyomma cajennense*	1	2	2

Amblyomma cajennense	*Amblyomma cajennense*	1	2	2
Amblyomma dubitatum	*Amblyomma sculptum*	0	3	5
Amblyomma dubitatum	*Amblyomma dubitatum*	1	3	3
Amblyomma dubitatum	*Amblyomma dubitatum*	1	3	3
Amblyomma dubitatum	*Amblyomma dubitatum*	1	3	3
Amblyomma dubitatum	*Amblyomma dubitatum*	1	3	3
Amblyomma dubitatum	*Amblyomma triste*	0	3	6
Amblyomma dubitatum	*Amblyomma dubitatum*	1	3	3
Amblyomma dubitatum	*Amblyomma dubitatum*	1	3	3
Amblyomma dubitatum	*Amblyomma dubitatum*	1	3	3
Amblyomma dubitatum	*Amblyomma dubitatum*	1	3	3
Amblyomma dubitatum	*Amblyomma dubitatum*	1	3	3
Amblyomma dubitatum	*Amblyomma dubitatum*	1	3	3
Amblyomma dubitatum	*Amblyomma dubitatum*	1	3	3
Amblyomma ovale	*Amblyomma ovale*	1	4	4
Amblyomma ovale	*Amblyomma ovale*	1	4	4
Amblyomma ovale	*Amblyomma ovale*	1	4	4
Amblyomma ovale	*Amblyomma ovale*	1	4	4
Amblyomma ovale	*Amblyomma ovale*	1	4	4
Amblyomma ovale	*Amblyomma ovale*	1	4	4
Amblyomma ovale	*Amblyomma ovale*	1	4	4
Amblyomma ovale	*Amblyomma ovale*	1	4	4
Amblyomma ovale	*Amblyomma ovale*	1	4	4
Amblyomma ovale	*Amblyomma ovale*	1	4	4
Amblyomma ovale	*Amblyomma triste*	0	4	6
Amblyomma ovale	*Amblyomma cajennense*	0	4	2
Amblyomma ovale	*Amblyomma ovale*	1	4	4
Amblyomma ovale	*Amblyomma ovale*	1	4	4
Amblyomma sculptum	*Amblyomma sculptum*	1	5	5
Amblyomma sculptum	*Amblyomma sculptum*	1	5	5
Amblyomma sculptum	*Amblyomma sculptum*	1	5	5
Amblyomma sculptum	*Amblyomma sculptum*	1	5	5
Amblyomma sculptum	*Amblyomma sculptum*	1	5	5
Amblyomma sculptum	*Amblyomma sculptum*	1	5	5
Amblyomma sculptum	*Amblyomma sculptum*	1	5	5
Amblyomma sculptum	*Amblyomma sculptum*	1	5	5
Amblyomma sculptum	*Amblyomma sculptum*	1	5	5
Amblyomma sculptum	*Amblyomma sculptum*	1	5	5
Amblyomma sculptum	*Amblyomma sculptum*	1	5	5
Amblyomma sculptum	*Amblyomma sculptum*	1	5	5
Amblyomma sculptum	*Amblyomma sculptum*	1	5	5
Amblyomma sculptum	*Amblyomma sculptum*	1	5	5
Amblyomma sculptum	*Amblyomma sculptum*	1	5	5
Amblyomma sculptum	*Amblyomma sculptum*	1	5	5
Amblyomma sculptum	*Amblyomma sculptum*	1	5	5
Amblyomma sculptum	*Amblyomma sculptum*	1	5	5
Amblyomma sculptum	*Amblyomma dubitatum*	0	5	3
Amblyomma sculptum	*Amblyomma dubitatum*	0	5	3
Amblyomma sculptum	*Amblyomma sculptum*	1	5	5
Amblyomma sculptum	*Amblyomma sculptum*	1	5	5
Amblyomma sculptum	*Amblyomma sculptum*	1	5	5
Amblyomma sculptum	*Amblyomma sculptum*	1	5	5
Amblyomma sculptum	*Amblyomma sculptum*	1	5	5
Amblyomma sculptum	*Amblyomma sculptum*	1	5	5
Amblyomma sculptum	*Amblyomma sculptum*	1	5	5
Amblyomma sculptum	*Amblyomma sculptum*	1	5	5
Amblyomma triste	*Amblyomma triste*	1	6	6
Amblyomma triste	*Amblyomma triste*	1	6	6
Amblyomma triste	*Amblyomma triste*	1	6	6
Amblyomma triste	*Amblyomma triste*	1	6	6
Amblyomma triste	*Amblyomma triste*	1	6	6
Amblyomma triste	*Amblyomma triste*	1	6	6
Amblyomma triste	*Amblyomma triste*	1	6	6
Amblyomma triste	*Amblyomma triste*	1	6	6
Amblyomma triste	*Amblyomma triste*	1	6	6
Amblyomma triste	*Amblyomma triste*	1	6	6
Amblyomma triste	*Amblyomma cajennense*	0	6	2

Amblyomma triste	*Amblyomma triste*	1	6	6
Amblyomma triste	*Amblyomma triste*	1	6	6
Amblyomma triste	*Amblyomma triste*	1	6	6
Amblyomma triste	*Amblyomma triste*	1	6	6
Amblyomma aureolatum	*Amblyomma aureolatum*	1	1	1
Amblyomma aureolatum	*Amblyomma aureolatum*	1	1	1
Amblyomma aureolatum	*Amblyomma aureolatum*	1	1	1
Amblyomma aureolatum	*Amblyomma aureolatum*	1	1	1
Amblyomma aureolatum	*Amblyomma aureolatum*	1	1	1
Amblyomma aureolatum	*Amblyomma aureolatum*	1	1	1
Amblyomma aureolatum	*Amblyomma aureolatum*	1	1	1
Amblyomma aureolatum	*Amblyomma aureolatum*	1	1	1
Amblyomma aureolatum	*Amblyomma aureolatum*	1	1	1
Amblyomma aureolatum	*Amblyomma aureolatum*	1	1	1
Amblyomma aureolatum	*Amblyomma aureolatum*	1	1	1
Amblyomma aureolatum	*Amblyomma aureolatum*	1	1	1
Amblyomma aureolatum	*Amblyomma aureolatum*	1	1	1
Amblyomma aureolatum	*Amblyomma aureolatum*	1	1	1
Amblyomma cajennense	*Amblyomma cajennense*	1	2	2
Amblyomma cajennense	*Amblyomma cajennense*	1	2	2
Amblyomma cajennense	*Amblyomma cajennense*	1	2	2
Amblyomma cajennense	*Amblyomma cajennense*	1	2	2
Amblyomma cajennense	*Amblyomma cajennense*	1	2	2
Amblyomma cajennense	*Amblyomma cajennense*	1	2	2
Amblyomma cajennense	*Amblyomma cajennense*	1	2	2
Amblyomma cajennense	*Amblyomma cajennense*	1	2	2
Amblyomma cajennense	*Amblyomma cajennense*	1	2	2
Amblyomma cajennense	*Amblyomma cajennense*	1	2	2
Amblyomma cajennense	*Amblyomma cajennense*	1	2	2
Amblyomma cajennense	*Amblyomma cajennense*	1	2	2
Amblyomma cajennense	*Amblyomma cajennense*	1	2	2
Amblyomma cajennense	*Amblyomma cajennense*	1	2	2
Amblyomma cajennense	*Amblyomma cajennense*	1	2	2
Amblyomma cajennense	*Amblyomma cajennense*	1	2	2
Amblyomma dubitatum	*Amblyomma dubitatum*	1	3	3
Amblyomma dubitatum	*Amblyomma dubitatum*	1	3	3
Amblyomma dubitatum	*Amblyomma dubitatum*	1	3	3
Amblyomma dubitatum	*Amblyomma dubitatum*	1	3	3
Amblyomma dubitatum	*Amblyomma dubitatum*	1	3	3
Amblyomma dubitatum	*Amblyomma dubitatum*	1	3	3
Amblyomma dubitatum	*Amblyomma dubitatum*	1	3	3
Amblyomma dubitatum	*Amblyomma dubitatum*	1	3	3
Amblyomma dubitatum	*Amblyomma dubitatum*	1	3	3
Amblyomma dubitatum	*Amblyomma dubitatum*	1	3	3
Amblyomma dubitatum	*Amblyomma aureolatum*	0	3	1
Amblyomma dubitatum	*Amblyomma dubitatum*	1	3	3
Amblyomma dubitatum	*Amblyomma dubitatum*	1	3	3
Amblyomma ovale	*Amblyomma ovale*	1	4	4
Amblyomma ovale	*Amblyomma ovale*	1	4	4
Amblyomma ovale	*Amblyomma ovale*	1	4	4
Amblyomma ovale	*Amblyomma ovale*	1	4	4
Amblyomma ovale	*Amblyomma ovale*	1	4	4
Amblyomma ovale	*Amblyomma ovale*	1	4	4
Amblyomma ovale	*Amblyomma ovale*	1	4	4
Amblyomma ovale	*Amblyomma ovale*	1	4	4
Amblyomma ovale	*Amblyomma ovale*	1	4	4
Amblyomma ovale	*Amblyomma ovale*	1	4	4
Amblyomma ovale	*Amblyomma ovale*	1	4	4
Amblyomma ovale	*Amblyomma ovale*	1	4	4
Amblyomma ovale	*Amblyomma ovale*	1	4	4
Amblyomma sculptum	*Amblyomma sculptum*	1	5	5
Amblyomma sculptum	*Amblyomma sculptum*	1	5	5
Amblyomma sculptum	*Amblyomma sculptum*	1	5	5
Amblyomma sculptum	*Amblyomma sculptum*	1	5	5
Amblyomma sculptum	*Amblyomma sculptum*	1	5	5
Amblyomma sculptum	*Amblyomma sculptum*	1	5	5
Amblyomma sculptum	*Amblyomma sculptum*	1	5	5

Amblyomma sculptum	*Amblyomma sculptum*	1	5	5
Amblyomma sculptum	*Amblyomma sculptum*	1	5	5
Amblyomma sculptum	*Amblyomma sculptum*	1	5	5
Amblyomma sculptum	*Amblyomma sculptum*	1	5	5
Amblyomma sculptum	*Amblyomma sculptum*	1	5	5
Amblyomma sculptum	*Amblyomma sculptum*	1	5	5
Amblyomma sculptum	*Amblyomma sculptum*	1	5	5
Amblyomma sculptum	*Amblyomma sculptum*	1	5	5
Amblyomma sculptum	*Amblyomma sculptum*	1	5	5
Amblyomma sculptum	*Amblyomma sculptum*	1	5	5
Amblyomma sculptum	*Amblyomma sculptum*	1	5	5
Amblyomma sculptum	*Amblyomma sculptum*	1	5	5
Amblyomma sculptum	*Amblyomma sculptum*	1	5	5
Amblyomma sculptum	*Amblyomma sculptum*	1	5	5
Amblyomma sculptum	*Amblyomma sculptum*	1	5	5
Amblyomma sculptum	*Amblyomma sculptum*	1	5	5
Amblyomma sculptum	*Amblyomma sculptum*	1	5	5
Amblyomma sculptum	*Amblyomma sculptum*	1	5	5
Amblyomma sculptum	*Amblyomma sculptum*	1	5	5
Amblyomma sculptum	*Amblyomma sculptum*	1	5	5
Amblyomma triste	*Amblyomma triste*	1	6	6
Amblyomma triste	*Amblyomma triste*	1	6	6
Amblyomma triste	*Amblyomma aureolatum*	0	6	1
Amblyomma triste	*Amblyomma triste*	1	6	6
Amblyomma triste	*Amblyomma triste*	1	6	6
Amblyomma triste	*Amblyomma triste*	1	6	6
Amblyomma triste	*Amblyomma triste*	1	6	6
Amblyomma triste	*Amblyomma aureolatum*	0	6	1
Amblyomma triste	*Amblyomma aureolatum*	0	6	1
Amblyomma triste	*Amblyomma triste*	1	6	6
Amblyomma triste	*Amblyomma triste*	1	6	6
Amblyomma triste	*Amblyomma triste*	1	6	6
Amblyomma triste	*Amblyomma triste*	1	6	6
Amblyomma triste	*Amblyomma triste*	1	6	6
Amblyomma triste	*Amblyomma triste*	1	6	6
Amblyomma triste	*Amblyomma triste*	1	6	6
Amblyomma aureolatum	*Amblyomma aureolatum*	1	1	1
Amblyomma aureolatum	*Amblyomma aureolatum*	1	1	1
Amblyomma aureolatum	*Amblyomma aureolatum*	1	1	1
Amblyomma aureolatum	*Amblyomma aureolatum*	1	1	1
Amblyomma aureolatum	*Amblyomma aureolatum*	1	1	1
Amblyomma aureolatum	*Amblyomma aureolatum*	1	1	1
Amblyomma aureolatum	*Amblyomma aureolatum*	1	1	1
Amblyomma aureolatum	*Amblyomma aureolatum*	1	1	1
Amblyomma aureolatum	*Amblyomma aureolatum*	1	1	1
Amblyomma aureolatum	*Amblyomma aureolatum*	1	1	1
Amblyomma aureolatum	*Amblyomma aureolatum*	1	1	1
Amblyomma aureolatum	*Amblyomma aureolatum*	1	1	1
Amblyomma aureolatum	*Amblyomma aureolatum*	1	1	1
Amblyomma aureolatum	*Amblyomma aureolatum*	1	1	1
Amblyomma cajennense	*Amblyomma cajennense*	1	2	2
Amblyomma cajennense	*Amblyomma cajennense*	1	2	2
Amblyomma cajennense	*Amblyomma cajennense*	1	2	2
Amblyomma cajennense	*Amblyomma cajennense*	1	2	2
Amblyomma cajennense	*Amblyomma cajennense*	1	2	2
Amblyomma cajennense	*Amblyomma cajennense*	1	2	2
Amblyomma cajennense	*Amblyomma cajennense*	1	2	2
Amblyomma cajennense	*Amblyomma cajennense*	1	2	2
Amblyomma cajennense	*Amblyomma cajennense*	1	2	2
Amblyomma cajennense	*Amblyomma cajennense*	1	2	2
Amblyomma cajennense	*Amblyomma cajennense*	1	2	2
Amblyomma cajennense	*Amblyomma cajennense*	1	2	2
Amblyomma cajennense	*Amblyomma cajennense*	1	2	2
Amblyomma cajennense	*Amblyomma cajennense*	1	2	2
Amblyomma cajennense	*Amblyomma cajennense*	1	2	2
Amblyomma cajennense	*Amblyomma cajennense*	1	2	2
Amblyomma dubitatum	*Amblyomma dubitatum*	1	3	3

Amblyomma dubitatum	*Amblyomma dubitatum*	1	3	3
Amblyomma dubitatum	*Amblyomma dubitatum*	1	3	3
Amblyomma dubitatum	*Amblyomma dubitatum*	1	3	3
Amblyomma dubitatum	*Amblyomma aureolatum*	0	3	1
Amblyomma dubitatum	*Amblyomma dubitatum*	1	3	3
Amblyomma dubitatum	*Amblyomma aureolatum*	0	3	1
Amblyomma dubitatum	*Amblyomma dubitatum*	1	3	3
Amblyomma dubitatum	*Amblyomma aureolatum*	0	3	1
Amblyomma dubitatum	*Amblyomma dubitatum*	1	3	3
Amblyomma dubitatum	*Amblyomma aureolatum*	0	3	1
Amblyomma dubitatum	*Amblyomma sculptum*	0	3	5
Amblyomma dubitatum	*Amblyomma sculptum*	0	3	5
Amblyomma ovale	*Amblyomma ovale*	1	4	4
Amblyomma ovale	*Amblyomma ovale*	1	4	4
Amblyomma ovale	*Amblyomma ovale*	1	4	4
Amblyomma ovale	*Amblyomma ovale*	1	4	4
Amblyomma ovale	*Amblyomma ovale*	1	4	4
Amblyomma ovale	*Amblyomma ovale*	1	4	4
Amblyomma ovale	*Amblyomma cajennense*	0	4	2
Amblyomma ovale	*Amblyomma ovale*	1	4	4
Amblyomma ovale	*Amblyomma ovale*	1	4	4
Amblyomma ovale	*Amblyomma aureolatum*	0	4	1
Amblyomma ovale	*Amblyomma ovale*	1	4	4
Amblyomma ovale	*Amblyomma ovale*	1	4	4
Amblyomma ovale	*Amblyomma ovale*	1	4	4
Amblyomma sculptum	*Amblyomma sculptum*	1	5	5
Amblyomma sculptum	*Amblyomma sculptum*	1	5	5
Amblyomma sculptum	*Amblyomma sculptum*	1	5	5
Amblyomma sculptum	*Amblyomma sculptum*	1	5	5
Amblyomma sculptum	*Amblyomma sculptum*	1	5	5
Amblyomma sculptum	*Amblyomma sculptum*	1	5	5
Amblyomma sculptum	*Amblyomma sculptum*	1	5	5
Amblyomma sculptum	*Amblyomma sculptum*	1	5	5
Amblyomma sculptum	*Amblyomma sculptum*	1	5	5
Amblyomma sculptum	*Amblyomma sculptum*	1	5	5
Amblyomma sculptum	*Amblyomma sculptum*	1	5	5
Amblyomma sculptum	*Amblyomma sculptum*	1	5	5
Amblyomma sculptum	*Amblyomma sculptum*	1	5	5
Amblyomma sculptum	*Amblyomma sculptum*	1	5	5
Amblyomma sculptum	*Amblyomma sculptum*	1	5	5
Amblyomma sculptum	*Amblyomma sculptum*	1	5	5
Amblyomma sculptum	*Amblyomma sculptum*	1	5	5
Amblyomma sculptum	*Amblyomma sculptum*	1	5	5
Amblyomma sculptum	*Amblyomma sculptum*	1	5	5
Amblyomma sculptum	*Amblyomma sculptum*	1	5	5
Amblyomma sculptum	*Amblyomma sculptum*	1	5	5
Amblyomma sculptum	*Amblyomma sculptum*	1	5	5
Amblyomma sculptum	*Amblyomma sculptum*	1	5	5
Amblyomma sculptum	*Amblyomma sculptum*	1	5	5
Amblyomma sculptum	*Amblyomma sculptum*	1	5	5
Amblyomma sculptum	*Amblyomma sculptum*	1	5	5
Amblyomma sculptum	*Amblyomma sculptum*	1	5	5
Amblyomma sculptum	*Amblyomma sculptum*	1	5	5
Amblyomma sculptum	*Amblyomma sculptum*	1	5	5
Amblyomma sculptum	*Amblyomma sculptum*	1	5	5
Amblyomma sculptum	*Amblyomma sculptum*	1	5	5
Amblyomma triste	*Amblyomma triste*	1	6	6
Amblyomma triste	*Amblyomma triste*	1	6	6
Amblyomma triste	*Amblyomma triste*	1	6	6
Amblyomma triste	*Amblyomma triste*	1	6	6
Amblyomma triste	*Amblyomma aureolatum*	0	6	1
Amblyomma triste	*Amblyomma triste*	1	6	6
Amblyomma triste	*Amblyomma triste*	1	6	6
Amblyomma triste	*Amblyomma triste*	1	6	6
Amblyomma triste	*Amblyomma triste*	1	6	6
Amblyomma triste	*Amblyomma triste*	1	6	6
Amblyomma triste	*Amblyomma triste*	1	6	6
Amblyomma triste	*Amblyomma triste*	1	6	6
Amblyomma triste	*Amblyomma triste*	1	6	6
Amblyomma triste	*Amblyomma triste*	1	6	6

Amblyomma triste	*Amblyomma triste*	1	6	6
Amblyomma triste	*Amblyomma triste*	1	6	6
Amblyomma aureolatum	*Amblyomma aureolatum*	1	1	1
Amblyomma aureolatum	*Amblyomma aureolatum*	1	1	1
Amblyomma aureolatum	*Amblyomma triste*	0	1	6
Amblyomma aureolatum	*Amblyomma aureolatum*	1	1	1
Amblyomma aureolatum	*Amblyomma aureolatum*	1	1	1
Amblyomma aureolatum	*Amblyomma aureolatum*	1	1	1
Amblyomma aureolatum	*Amblyomma aureolatum*	1	1	1
Amblyomma aureolatum	*Amblyomma aureolatum*	1	1	1
Amblyomma aureolatum	*Amblyomma aureolatum*	1	1	1
Amblyomma aureolatum	*Amblyomma aureolatum*	1	1	1
Amblyomma aureolatum	*Amblyomma aureolatum*	1	1	1
Amblyomma aureolatum	*Amblyomma aureolatum*	1	1	1
Amblyomma aureolatum	*Amblyomma aureolatum*	1	1	1
Amblyomma aureolatum	*Amblyomma aureolatum*	1	1	1
Amblyomma aureolatum	*Amblyomma aureolatum*	1	1	1
Amblyomma aureolatum	*Amblyomma aureolatum*	1	1	1
Amblyomma aureolatum	*Amblyomma aureolatum*	1	1	1
Amblyomma aureolatum	*Amblyomma aureolatum*	1	1	1
Amblyomma aureolatum	*Amblyomma aureolatum*	1	1	1
Amblyomma aureolatum	*Amblyomma aureolatum*	1	1	1
Amblyomma aureolatum	*Amblyomma aureolatum*	1	1	1
Amblyomma aureolatum	*Amblyomma aureolatum*	1	1	1
Amblyomma aureolatum	*Amblyomma aureolatum*	1	1	1
Amblyomma aureolatum	*Amblyomma aureolatum*	1	1	1
Amblyomma cajennense	*Amblyomma cajennense*	1	2	2
Amblyomma cajennense	*Amblyomma cajennense*	1	2	2
Amblyomma cajennense	*Amblyomma cajennense*	1	2	2
Amblyomma cajennense	*Amblyomma cajennense*	1	2	2
Amblyomma cajennense	*Amblyomma cajennense*	1	2	2
Amblyomma cajennense	*Amblyomma cajennense*	1	2	2
Amblyomma cajennense	*Amblyomma cajennense*	1	2	2
Amblyomma cajennense	*Amblyomma cajennense*	1	2	2
Amblyomma cajennense	*Amblyomma cajennense*	1	2	2
Amblyomma cajennense	*Amblyomma cajennense*	1	2	2
Amblyomma cajennense	*Amblyomma cajennense*	1	2	2
Amblyomma cajennense	*Amblyomma cajennense*	1	2	2
Amblyomma cajennense	*Amblyomma cajennense*	1	2	2
Amblyomma cajennense	*Amblyomma cajennense*	1	2	2
Amblyomma cajennense	*Amblyomma cajennense*	1	2	2
Amblyomma cajennense	*Amblyomma cajennense*	1	2	2
Amblyomma cajennense	*Amblyomma cajennense*	1	2	2
Amblyomma cajennense	*Amblyomma cajennense*	1	2	2
Amblyomma cajennense	*Amblyomma cajennense*	1	2	2
Amblyomma cajennense	*Amblyomma cajennense*	1	2	2
Amblyomma cajennense	*Amblyomma cajennense*	1	2	2
Amblyomma cajennense	*Amblyomma cajennense*	1	2	2
Amblyomma cajennense	*Amblyomma cajennense*	1	2	2
Amblyomma cajennense	*Amblyomma cajennense*	1	2	2
Amblyomma cajennense	*Amblyomma cajennense*	1	2	2
Amblyomma cajennense	*Amblyomma cajennense*	1	2	2
Amblyomma cajennense	*Amblyomma cajennense*	1	2	2
Amblyomma cajennense	*Amblyomma cajennense*	1	2	2
Amblyomma cajennense	*Amblyomma cajennense*	1	2	2
Amblyomma cajennense	*Amblyomma cajennense*	1	2	2
Amblyomma cajennense	*Amblyomma cajennense*	1	2	2
Amblyomma cajennense	*Amblyomma cajennense*	1	2	2
Amblyomma cajennense	*Amblyomma cajennense*	1	2	2
Amblyomma dubitatum	*Amblyomma dubitatum*	1	3	3
Amblyomma dubitatum	*Amblyomma dubitatum*	1	3	3
Amblyomma dubitatum	*Amblyomma dubitatum*	1	3	3
Amblyomma dubitatum	*Amblyomma sculptum*	0	3	5
Amblyomma dubitatum	*Amblyomma dubitatum*	1	3	3
Amblyomma dubitatum	*Amblyomma sculptum*	0	3	5
Amblyomma dubitatum	*Amblyomma dubitatum*	1	3	3
Amblyomma dubitatum	*Amblyomma sculptum*	0	3	5

Amblyomma dubitatum	*Amblyomma dubitatum*	1	3	3
Amblyomma dubitatum	*Amblyomma dubitatum*	1	3	3
Amblyomma dubitatum	*Amblyomma dubitatum*	1	3	3
Amblyomma dubitatum	*Amblyomma dubitatum*	1	3	3
Amblyomma dubitatum	*Amblyomma dubitatum*	1	3	3
Amblyomma dubitatum	*Amblyomma dubitatum*	1	3	3
Amblyomma dubitatum	*Amblyomma dubitatum*	1	3	3
Amblyomma dubitatum	*Amblyomma dubitatum*	1	3	3
Amblyomma dubitatum	*Amblyomma dubitatum*	1	3	3
Amblyomma dubitatum	*Amblyomma dubitatum*	1	3	3
Amblyomma dubitatum	*Amblyomma dubitatum*	1	3	3
Amblyomma dubitatum	*Amblyomma cajennense*	0	3	2
Amblyomma dubitatum	*Amblyomma dubitatum*	1	3	3
Amblyomma dubitatum	*Amblyomma dubitatum*	1	3	3
Amblyomma ovale	*Amblyomma ovale*	1	4	4
Amblyomma ovale	*Amblyomma triste*	0	4	6
Amblyomma ovale	*Amblyomma ovale*	1	4	4
Amblyomma ovale	*Amblyomma aureolatum*	0	4	1
Amblyomma ovale	*Amblyomma ovale*	1	4	4
Amblyomma ovale	*Amblyomma ovale*	1	4	4
Amblyomma ovale	*Amblyomma ovale*	1	4	4
Amblyomma ovale	*Amblyomma ovale*	1	4	4
Amblyomma ovale	*Amblyomma ovale*	1	4	4
Amblyomma ovale	*Amblyomma ovale*	1	4	4
Amblyomma ovale	*Amblyomma ovale*	1	4	4
Amblyomma ovale	*Amblyomma ovale*	1	4	4
Amblyomma ovale	*Amblyomma ovale*	1	4	4
Amblyomma ovale	*Amblyomma ovale*	1	4	4
Amblyomma ovale	*Amblyomma ovale*	1	4	4
Amblyomma ovale	*Amblyomma ovale*	1	4	4
Amblyomma ovale	*Amblyomma ovale*	1	4	4
Amblyomma ovale	*Amblyomma ovale*	1	4	4
Amblyomma ovale	*Amblyomma ovale*	1	4	4
Amblyomma ovale	*Amblyomma ovale*	1	4	4
Amblyomma ovale	*Amblyomma ovale*	1	4	4
Amblyomma ovale	*Amblyomma ovale*	1	4	4
Amblyomma ovale	*Amblyomma ovale*	1	4	4
Amblyomma ovale	*Amblyomma ovale*	1	4	4
Amblyomma sculptum	*Amblyomma sculptum*	1	5	5
Amblyomma sculptum	*Amblyomma sculptum*	1	5	5
Amblyomma sculptum	*Amblyomma sculptum*	1	5	5
Amblyomma sculptum	*Amblyomma sculptum*	1	5	5
Amblyomma sculptum	*Amblyomma sculptum*	1	5	5
Amblyomma sculptum	*Amblyomma sculptum*	1	5	5
Amblyomma sculptum	*Amblyomma sculptum*	1	5	5
Amblyomma sculptum	*Amblyomma sculptum*	1	5	5
Amblyomma sculptum	*Amblyomma sculptum*	1	5	5
Amblyomma sculptum	*Amblyomma sculptum*	1	5	5
Amblyomma sculptum	*Amblyomma sculptum*	1	5	5
Amblyomma sculptum	*Amblyomma sculptum*	1	5	5
Amblyomma sculptum	*Amblyomma sculptum*	1	5	5
Amblyomma sculptum	*Amblyomma sculptum*	1	5	5
Amblyomma sculptum	*Amblyomma sculptum*	1	5	5
Amblyomma sculptum	*Amblyomma sculptum*	1	5	5
Amblyomma sculptum	*Amblyomma sculptum*	1	5	5
Amblyomma sculptum	*Amblyomma triste*	0	5	6
Amblyomma sculptum	*Amblyomma sculptum*	1	5	5
Amblyomma sculptum	*Amblyomma sculptum*	1	5	5
Amblyomma sculptum	*Amblyomma sculptum*	1	5	5
Amblyomma sculptum	*Amblyomma sculptum*	1	5	5
Amblyomma sculptum	*Amblyomma sculptum*	1	5	5
Amblyomma sculptum	*Amblyomma sculptum*	1	5	5
Amblyomma sculptum	*Amblyomma sculptum*	1	5	5
Amblyomma sculptum	*Amblyomma sculptum*	1	5	5
Amblyomma sculptum	*Amblyomma sculptum*	1	5	5
Amblyomma sculptum	*Amblyomma sculptum*	1	5	5
Amblyomma sculptum	*Amblyomma sculptum*	1	5	5

Amblyomma sculptum	*Amblyomma sculptum*	1	5	5
Amblyomma sculptum	*Amblyomma sculptum*	1	5	5
Amblyomma triste	*Amblyomma triste*	1	6	6
Amblyomma triste	*Amblyomma triste*	1	6	6
Amblyomma triste	*Amblyomma triste*	1	6	6
Amblyomma triste	*Amblyomma triste*	1	6	6
Amblyomma triste	*Amblyomma triste*	1	6	6
Amblyomma triste	*Amblyomma triste*	1	6	6
Amblyomma triste	*Amblyomma triste*	1	6	6
Amblyomma triste	*Amblyomma triste*	1	6	6
Amblyomma triste	*Amblyomma triste*	1	6	6
Amblyomma triste	*Amblyomma triste*	1	6	6
Amblyomma triste	*Amblyomma triste*	1	6	6
Amblyomma triste	*Amblyomma triste*	1	6	6
Amblyomma triste	*Amblyomma triste*	1	6	6
Amblyomma triste	*Amblyomma triste*	1	6	6
Amblyomma triste	*Amblyomma triste*	1	6	6
Amblyomma triste	*Amblyomma triste*	1	6	6
Amblyomma triste	*Amblyomma triste*	1	6	6
Amblyomma triste	*Amblyomma triste*	1	6	6
Amblyomma triste	*Amblyomma triste*	1	6	6
Amblyomma triste	*Amblyomma triste*	1	6	6
Amblyomma triste	*Amblyomma triste*	1	6	6
Amblyomma triste	*Amblyomma triste*	1	6	6
Amblyomma triste	*Amblyomma triste*	1	6	6
Amblyomma triste	*Amblyomma triste*	1	6	6
Amblyomma triste	*Amblyomma triste*	1	6	6
Amblyomma triste	*Amblyomma triste*	1	6	6
Amblyomma triste	*Amblyomma triste*	1	6	6
Amblyomma triste	*Amblyomma triste*	1	6	6
Amblyomma triste	*Amblyomma triste*	1	6	6
Amblyomma triste	*Amblyomma triste*	1	6	6
Amblyomma triste	*Amblyomma triste*	1	6	6
Amblyomma aureolatum	*Amblyomma aureolatum*	1	1	1
Amblyomma aureolatum	*Amblyomma aureolatum*	1	1	1
Amblyomma aureolatum	*Amblyomma aureolatum*	1	1	1
Amblyomma aureolatum	*Amblyomma aureolatum*	1	1	1
Amblyomma aureolatum	*Amblyomma aureolatum*	1	1	1
Amblyomma aureolatum	*Amblyomma aureolatum*	1	1	1
Amblyomma aureolatum	*Amblyomma aureolatum*	1	1	1
Amblyomma aureolatum	*Amblyomma aureolatum*	1	1	1
Amblyomma aureolatum	*Amblyomma aureolatum*	1	1	1
Amblyomma aureolatum	*Amblyomma aureolatum*	1	1	1
Amblyomma aureolatum	*Amblyomma aureolatum*	1	1	1
Amblyomma aureolatum	*Amblyomma aureolatum*	1	1	1
Amblyomma aureolatum	*Amblyomma aureolatum*	1	1	1
Amblyomma aureolatum	*Amblyomma aureolatum*	1	1	1
Amblyomma aureolatum	*Amblyomma aureolatum*	1	1	1
Amblyomma aureolatum	*Amblyomma aureolatum*	1	1	1
Amblyomma aureolatum	*Amblyomma aureolatum*	1	1	1
Amblyomma aureolatum	*Amblyomma aureolatum*	1	1	1
Amblyomma aureolatum	*Amblyomma aureolatum*	1	1	1
Amblyomma aureolatum	*Amblyomma aureolatum*	1	1	1
Amblyomma aureolatum	*Amblyomma aureolatum*	1	1	1
Amblyomma aureolatum	*Amblyomma aureolatum*	1	1	1
Amblyomma aureolatum	*Amblyomma aureolatum*	1	1	1
Amblyomma aureolatum	*Amblyomma aureolatum*	1	1	1
Amblyomma cajennense	*Amblyomma cajennense*	1	2	2
Amblyomma cajennense	*Amblyomma cajennense*	1	2	2
Amblyomma cajennense	*Amblyomma cajennense*	1	2	2
Amblyomma cajennense	*Amblyomma cajennense*	1	2	2
Amblyomma cajennense	*Amblyomma cajennense*	1	2	2
Amblyomma cajennense	*Amblyomma cajennense*	1	2	2
Amblyomma cajennense	*Amblyomma cajennense*	1	2	2
Amblyomma cajennense	*Amblyomma cajennense*	1	2	2
Amblyomma cajennense	*Amblyomma cajennense*	1	2	2
Amblyomma cajennense	*Amblyomma cajennense*	1	2	2

Amblyomma cajennense	*Amblyomma cajennense*	1	2	2
Amblyomma cajennense	*Amblyomma cajennense*	1	2	2
Amblyomma cajennense	*Amblyomma cajennense*	1	2	2
Amblyomma cajennense	*Amblyomma cajennense*	1	2	2
Amblyomma cajennense	*Amblyomma cajennense*	1	2	2
Amblyomma cajennense	*Amblyomma cajennense*	1	2	2
Amblyomma cajennense	*Amblyomma cajennense*	1	2	2
Amblyomma cajennense	*Amblyomma cajennense*	1	2	2
Amblyomma cajennense	*Amblyomma cajennense*	1	2	2
Amblyomma cajennense	*Amblyomma cajennense*	1	2	2
Amblyomma cajennense	*Amblyomma cajennense*	1	2	2
Amblyomma cajennense	*Amblyomma cajennense*	1	2	2
Amblyomma cajennense	*Amblyomma cajennense*	1	2	2
Amblyomma cajennense	*Amblyomma cajennense*	1	2	2
Amblyomma cajennense	*Amblyomma cajennense*	1	2	2
Amblyomma cajennense	*Amblyomma cajennense*	1	2	2
Amblyomma cajennense	*Amblyomma cajennense*	1	2	2
Amblyomma cajennense	*Amblyomma cajennense*	1	2	2
Amblyomma cajennense	*Amblyomma cajennense*	1	2	2
Amblyomma cajennense	*Amblyomma cajennense*	1	2	2
Amblyomma cajennense	*Amblyomma cajennense*	1	2	2
Amblyomma cajennense	*Amblyomma cajennense*	1	2	2
Amblyomma cajennense	*Amblyomma cajennense*	1	2	2
Amblyomma dubitatum	*Amblyomma ovale*	0	3	4
Amblyomma dubitatum	*Amblyomma dubitatum*	1	3	3
Amblyomma dubitatum	*Amblyomma dubitatum*	1	3	3
Amblyomma dubitatum	*Amblyomma sculptum*	0	3	5
Amblyomma dubitatum	*Amblyomma dubitatum*	1	3	3
Amblyomma dubitatum	*Amblyomma cajennense*	0	3	2
Amblyomma dubitatum	*Amblyomma dubitatum*	1	3	3
Amblyomma dubitatum	*Amblyomma dubitatum*	1	3	3
Amblyomma dubitatum	*Amblyomma dubitatum*	1	3	3
Amblyomma dubitatum	*Amblyomma dubitatum*	1	3	3
Amblyomma dubitatum	*Amblyomma dubitatum*	1	3	3
Amblyomma dubitatum	*Amblyomma dubitatum*	1	3	3
Amblyomma dubitatum	*Amblyomma dubitatum*	1	3	3
Amblyomma dubitatum	*Amblyomma dubitatum*	1	3	3
Amblyomma dubitatum	*Amblyomma dubitatum*	1	3	3
Amblyomma dubitatum	*Amblyomma dubitatum*	1	3	3
Amblyomma dubitatum	*Amblyomma dubitatum*	1	3	3
Amblyomma dubitatum	*Amblyomma dubitatum*	1	3	3
Amblyomma dubitatum	*Amblyomma dubitatum*	1	3	3
Amblyomma dubitatum	*Amblyomma dubitatum*	1	3	3
Amblyomma dubitatum	*Amblyomma dubitatum*	1	3	3
Amblyomma dubitatum	*Amblyomma dubitatum*	1	3	3
Amblyomma ovale	*Amblyomma ovale*	1	4	4
Amblyomma ovale	*Amblyomma ovale*	1	4	4
Amblyomma ovale	*Amblyomma ovale*	1	4	4
Amblyomma ovale	*Amblyomma ovale*	1	4	4
Amblyomma ovale	*Amblyomma ovale*	1	4	4
Amblyomma ovale	*Amblyomma ovale*	1	4	4
Amblyomma ovale	*Amblyomma ovale*	1	4	4
Amblyomma ovale	*Amblyomma ovale*	1	4	4
Amblyomma ovale	*Amblyomma ovale*	1	4	4
Amblyomma ovale	*Amblyomma ovale*	1	4	4
Amblyomma ovale	*Amblyomma ovale*	1	4	4
Amblyomma ovale	*Amblyomma ovale*	1	4	4
Amblyomma ovale	*Amblyomma ovale*	1	4	4
Amblyomma ovale	*Amblyomma ovale*	1	4	4
Amblyomma ovale	*Amblyomma ovale*	1	4	4
Amblyomma ovale	*Amblyomma ovale*	1	4	4
Amblyomma ovale	*Amblyomma ovale*	1	4	4
Amblyomma ovale	*Amblyomma ovale*	1	4	4
Amblyomma ovale	*Amblyomma ovale*	1	4	4
Amblyomma ovale	*Amblyomma ovale*	1	4	4
Amblyomma ovale	*Amblyomma ovale*	1	4	4
Amblyomma ovale	*Amblyomma ovale*	1	4	4

Amblyomma ovale	*Amblyomma ovale*	1	4	4
Amblyomma ovale	*Amblyomma ovale*	1	4	4
Amblyomma sculptum	*Amblyomma sculptum*	1	5	5
Amblyomma sculptum	*Amblyomma sculptum*	1	5	5
Amblyomma sculptum	*Amblyomma sculptum*	1	5	5
Amblyomma sculptum	*Amblyomma sculptum*	1	5	5
Amblyomma sculptum	*Amblyomma sculptum*	1	5	5
Amblyomma sculptum	*Amblyomma sculptum*	1	5	5
Amblyomma sculptum	*Amblyomma sculptum*	1	5	5
Amblyomma sculptum	*Amblyomma sculptum*	1	5	5
Amblyomma sculptum	*Amblyomma sculptum*	1	5	5
Amblyomma sculptum	*Amblyomma sculptum*	1	5	5
Amblyomma sculptum	*Amblyomma sculptum*	1	5	5
Amblyomma sculptum	*Amblyomma sculptum*	1	5	5
Amblyomma sculptum	*Amblyomma sculptum*	1	5	5
Amblyomma sculptum	*Amblyomma sculptum*	1	5	5
Amblyomma sculptum	*Amblyomma sculptum*	1	5	5
Amblyomma sculptum	*Amblyomma sculptum*	1	5	5
Amblyomma sculptum	*Amblyomma sculptum*	1	5	5
Amblyomma sculptum	*Amblyomma sculptum*	1	5	5
Amblyomma sculptum	*Amblyomma sculptum*	1	5	5
Amblyomma sculptum	*Amblyomma sculptum*	1	5	5
Amblyomma sculptum	*Amblyomma sculptum*	1	5	5
Amblyomma sculptum	*Amblyomma ovale*	0	5	4
Amblyomma sculptum	*Amblyomma sculptum*	1	5	5
Amblyomma sculptum	*Amblyomma sculptum*	1	5	5
Amblyomma sculptum	*Amblyomma sculptum*	1	5	5
Amblyomma sculptum	*Amblyomma sculptum*	1	5	5
Amblyomma sculptum	*Amblyomma sculptum*	1	5	5
Amblyomma sculptum	*Amblyomma sculptum*	1	5	5
Amblyomma sculptum	*Amblyomma sculptum*	1	5	5
Amblyomma sculptum	*Amblyomma sculptum*	1	5	5
Amblyomma sculptum	*Amblyomma sculptum*	1	5	5
Amblyomma triste	*Amblyomma triste*	1	6	6
Amblyomma triste	*Amblyomma triste*	1	6	6
Amblyomma triste	*Amblyomma triste*	1	6	6
Amblyomma triste	*Amblyomma triste*	1	6	6
Amblyomma triste	*Amblyomma triste*	1	6	6
Amblyomma triste	*Amblyomma triste*	1	6	6
Amblyomma triste	*Amblyomma triste*	1	6	6
Amblyomma triste	*Amblyomma triste*	1	6	6
Amblyomma triste	*Amblyomma triste*	1	6	6
Amblyomma triste	*Amblyomma cajennense*	0	6	2
Amblyomma triste	*Amblyomma triste*	1	6	6
Amblyomma triste	*Amblyomma triste*	1	6	6
Amblyomma triste	*Amblyomma triste*	1	6	6
Amblyomma triste	*Amblyomma triste*	1	6	6
Amblyomma triste	*Amblyomma triste*	1	6	6
Amblyomma triste	*Amblyomma triste*	1	6	6
Amblyomma triste	*Amblyomma triste*	1	6	6
Amblyomma triste	*Amblyomma triste*	1	6	6
Amblyomma triste	*Amblyomma triste*	1	6	6
Amblyomma triste	*Amblyomma triste*	1	6	6
Amblyomma triste	*Amblyomma triste*	1	6	6
Amblyomma triste	*Amblyomma triste*	1	6	6
Amblyomma triste	*Amblyomma triste*	1	6	6
Amblyomma triste	*Amblyomma triste*	1	6	6
Amblyomma triste	*Amblyomma triste*	1	6	6
Amblyomma triste	*Amblyomma triste*	1	6	6
Amblyomma triste	*Amblyomma triste*	1	6	6
Amblyomma triste	*Amblyomma triste*	1	6	6
Amblyomma triste	*Amblyomma triste*	1	6	6
Amblyomma triste	*Amblyomma triste*	1	6	6
Amblyomma triste	*Amblyomma triste*	1	6	6
Amblyomma aureolatum	*Amblyomma aureolatum*	1	1	1
Amblyomma aureolatum	*Amblyomma aureolatum*	1	1	1
Amblyomma aureolatum	*Amblyomma aureolatum*	1	1	1

Amblyomma aureolatum	*Amblyomma aureolatum*	1	1	1
Amblyomma aureolatum	*Amblyomma aureolatum*	1	1	1
Amblyomma aureolatum	*Amblyomma aureolatum*	1	1	1
Amblyomma aureolatum	*Amblyomma aureolatum*	1	1	1
Amblyomma aureolatum	*Amblyomma aureolatum*	1	1	1
Amblyomma aureolatum	*Amblyomma aureolatum*	1	1	1
Amblyomma aureolatum	*Amblyomma cajennense*	0	1	2
Amblyomma aureolatum	*Amblyomma aureolatum*	1	1	1
Amblyomma aureolatum	*Amblyomma aureolatum*	1	1	1
Amblyomma aureolatum	*Amblyomma aureolatum*	1	1	1
Amblyomma aureolatum	*Amblyomma aureolatum*	1	1	1
Amblyomma aureolatum	*Amblyomma aureolatum*	1	1	1
Amblyomma aureolatum	*Amblyomma aureolatum*	1	1	1
Amblyomma aureolatum	*Amblyomma aureolatum*	1	1	1
Amblyomma aureolatum	*Amblyomma aureolatum*	1	1	1
Amblyomma aureolatum	*Amblyomma aureolatum*	1	1	1
Amblyomma aureolatum	*Amblyomma aureolatum*	1	1	1
Amblyomma aureolatum	*Amblyomma aureolatum*	1	1	1
Amblyomma aureolatum	*Amblyomma aureolatum*	1	1	1
Amblyomma aureolatum	*Amblyomma aureolatum*	1	1	1
Amblyomma aureolatum	*Amblyomma aureolatum*	1	1	1
Amblyomma cajennense	*Amblyomma cajennense*	1	2	2
Amblyomma cajennense	*Amblyomma cajennense*	1	2	2
Amblyomma cajennense	*Amblyomma cajennense*	1	2	2
Amblyomma cajennense	*Amblyomma cajennense*	1	2	2
Amblyomma cajennense	*Amblyomma cajennense*	1	2	2
Amblyomma cajennense	*Amblyomma cajennense*	1	2	2
Amblyomma cajennense	*Amblyomma cajennense*	1	2	2
Amblyomma cajennense	*Amblyomma cajennense*	1	2	2
Amblyomma cajennense	*Amblyomma cajennense*	1	2	2
Amblyomma cajennense	*Amblyomma cajennense*	1	2	2
Amblyomma cajennense	*Amblyomma cajennense*	1	2	2
Amblyomma cajennense	*Amblyomma cajennense*	1	2	2
Amblyomma cajennense	*Amblyomma cajennense*	1	2	2
Amblyomma cajennense	*Amblyomma cajennense*	1	2	2
Amblyomma cajennense	*Amblyomma cajennense*	1	2	2
Amblyomma cajennense	*Amblyomma cajennense*	1	2	2
Amblyomma cajennense	*Amblyomma cajennense*	1	2	2
Amblyomma cajennense	*Amblyomma cajennense*	1	2	2
Amblyomma cajennense	*Amblyomma cajennense*	1	2	2
Amblyomma cajennense	*Amblyomma cajennense*	1	2	2
Amblyomma cajennense	*Amblyomma cajennense*	1	2	2
Amblyomma cajennense	*Amblyomma cajennense*	1	2	2
Amblyomma cajennense	*Amblyomma cajennense*	1	2	2
Amblyomma cajennense	*Amblyomma cajennense*	1	2	2
Amblyomma cajennense	*Amblyomma cajennense*	1	2	2
Amblyomma cajennense	*Amblyomma cajennense*	1	2	2
Amblyomma cajennense	*Amblyomma cajennense*	1	2	2
Amblyomma cajennense	*Amblyomma cajennense*	1	2	2
Amblyomma cajennense	*Amblyomma cajennense*	1	2	2
Amblyomma cajennense	*Amblyomma cajennense*	1	2	2
Amblyomma cajennense	*Amblyomma cajennense*	1	2	2
Amblyomma cajennense	*Amblyomma cajennense*	1	2	2
Amblyomma cajennense	*Amblyomma cajennense*	1	2	2
Amblyomma cajennense	*Amblyomma cajennense*	1	2	2
Amblyomma cajennense	*Amblyomma cajennense*	1	2	2
Amblyomma dubitatum	*Amblyomma dubitatum*	1	3	3
Amblyomma dubitatum	*Amblyomma dubitatum*	1	3	3
Amblyomma dubitatum	*Amblyomma dubitatum*	1	3	3
Amblyomma dubitatum	*Amblyomma dubitatum*	1	3	3
Amblyomma dubitatum	*Amblyomma dubitatum*	1	3	3
Amblyomma dubitatum	*Amblyomma dubitatum*	1	3	3
Amblyomma dubitatum	*Amblyomma dubitatum*	1	3	3
Amblyomma dubitatum	*Amblyomma dubitatum*	1	3	3
Amblyomma dubitatum	*Amblyomma dubitatum*	1	3	3
Amblyomma dubitatum	*Amblyomma dubitatum*	1	3	3
Amblyomma dubitatum	*Amblyomma dubitatum*	1	3	3
Amblyomma dubitatum	*Amblyomma dubitatum*	1	3	3
Amblyomma dubitatum	*Amblyomma dubitatum*	1	3	3

Amblyomma dubitatum	*Amblyomma dubitatum*	1	3	3
Amblyomma dubitatum	*Amblyomma dubitatum*	1	3	3
Amblyomma dubitatum	*Amblyomma dubitatum*	1	3	3
Amblyomma dubitatum	*Amblyomma aureolatum*	0	3	1
Amblyomma dubitatum	*Amblyomma dubitatum*	1	3	3
Amblyomma dubitatum	*Amblyomma dubitatum*	1	3	3
Amblyomma dubitatum	*Amblyomma dubitatum*	1	3	3
Amblyomma dubitatum	*Amblyomma dubitatum*	1	3	3
Amblyomma dubitatum	*Amblyomma dubitatum*	1	3	3
Amblyomma ovale	*Amblyomma ovale*	1	4	4
Amblyomma ovale	*Amblyomma aureolatum*	0	4	1
Amblyomma ovale	*Amblyomma cajennense*	0	4	2
Amblyomma ovale	*Amblyomma ovale*	1	4	4
Amblyomma ovale	*Amblyomma ovale*	1	4	4
Amblyomma ovale	*Amblyomma ovale*	1	4	4
Amblyomma ovale	*Amblyomma ovale*	1	4	4
Amblyomma ovale	*Amblyomma ovale*	1	4	4
Amblyomma ovale	*Amblyomma ovale*	1	4	4
Amblyomma ovale	*Amblyomma ovale*	1	4	4
Amblyomma ovale	*Amblyomma ovale*	1	4	4
Amblyomma ovale	*Amblyomma ovale*	1	4	4
Amblyomma ovale	*Amblyomma ovale*	1	4	4
Amblyomma ovale	*Amblyomma ovale*	1	4	4
Amblyomma ovale	*Amblyomma ovale*	1	4	4
Amblyomma ovale	*Amblyomma ovale*	1	4	4
Amblyomma ovale	*Amblyomma ovale*	1	4	4
Amblyomma ovale	*Amblyomma aureolatum*	0	4	1
Amblyomma ovale	*Amblyomma aureolatum*	0	4	1
Amblyomma ovale	*Amblyomma sculptum*	0	4	5
Amblyomma ovale	*Amblyomma ovale*	1	4	4
Amblyomma ovale	*Amblyomma ovale*	1	4	4
Amblyomma ovale	*Amblyomma ovale*	1	4	4
Amblyomma ovale	*Amblyomma ovale*	1	4	4
Amblyomma sculptum	*Amblyomma sculptum*	1	5	5
Amblyomma sculptum	*Amblyomma sculptum*	1	5	5
Amblyomma sculptum	*Amblyomma sculptum*	1	5	5
Amblyomma sculptum	*Amblyomma sculptum*	1	5	5
Amblyomma sculptum	*Amblyomma sculptum*	1	5	5
Amblyomma sculptum	*Amblyomma sculptum*	1	5	5
Amblyomma sculptum	*Amblyomma sculptum*	1	5	5
Amblyomma sculptum	*Amblyomma sculptum*	1	5	5
Amblyomma sculptum	*Amblyomma sculptum*	1	5	5
Amblyomma sculptum	*Amblyomma sculptum*	1	5	5
Amblyomma sculptum	*Amblyomma sculptum*	1	5	5
Amblyomma sculptum	*Amblyomma sculptum*	1	5	5
Amblyomma sculptum	*Amblyomma sculptum*	1	5	5
Amblyomma sculptum	*Amblyomma sculptum*	1	5	5
Amblyomma sculptum	*Amblyomma sculptum*	1	5	5
Amblyomma sculptum	*Amblyomma sculptum*	1	5	5
Amblyomma sculptum	*Amblyomma sculptum*	1	5	5
Amblyomma sculptum	*Amblyomma sculptum*	1	5	5
Amblyomma sculptum	*Amblyomma sculptum*	1	5	5
Amblyomma sculptum	*Amblyomma sculptum*	1	5	5
Amblyomma sculptum	*Amblyomma sculptum*	1	5	5
Amblyomma sculptum	*Amblyomma sculptum*	1	5	5
Amblyomma sculptum	*Amblyomma sculptum*	1	5	5
Amblyomma sculptum	*Amblyomma cajennense*	0	5	2
Amblyomma sculptum	*Amblyomma dubitatum*	0	5	3
Amblyomma sculptum	*Amblyomma sculptum*	1	5	5
Amblyomma sculptum	*Amblyomma sculptum*	1	5	5
Amblyomma sculptum	*Amblyomma sculptum*	1	5	5
Amblyomma sculptum	*Amblyomma sculptum*	1	5	5
Amblyomma sculptum	*Amblyomma sculptum*	1	5	5
Amblyomma sculptum	*Amblyomma sculptum*	1	5	5
Amblyomma sculptum	*Amblyomma sculptum*	1	5	5
Amblyomma triste	*Amblyomma triste*	1	6	6
Amblyomma triste	*Amblyomma triste*	1	6	6
Amblyomma triste	*Amblyomma triste*	1	6	6

Amblyomma triste	*Amblyomma triste*	1	6	6
Amblyomma triste	*Amblyomma triste*	1	6	6
Amblyomma triste	*Amblyomma triste*	1	6	6
Amblyomma triste	*Amblyomma triste*	1	6	6
Amblyomma triste	*Amblyomma triste*	1	6	6
Amblyomma triste	*Amblyomma triste*	1	6	6
Amblyomma triste	*Amblyomma triste*	1	6	6
Amblyomma triste	*Amblyomma triste*	1	6	6
Amblyomma triste	*Amblyomma triste*	1	6	6
Amblyomma triste	*Amblyomma triste*	1	6	6
Amblyomma triste	*Amblyomma triste*	1	6	6
Amblyomma triste	*Amblyomma triste*	1	6	6
Amblyomma triste	*Amblyomma triste*	1	6	6
Amblyomma triste	*Amblyomma triste*	1	6	6
Amblyomma triste	*Amblyomma triste*	1	6	6
Amblyomma triste	*Amblyomma triste*	1	6	6
Amblyomma triste	*Amblyomma triste*	1	6	6
Amblyomma triste	*Amblyomma triste*	1	6	6
Amblyomma triste	*Amblyomma triste*	1	6	6
Amblyomma triste	*Amblyomma triste*	1	6	6
Amblyomma triste	*Amblyomma triste*	1	6	6
Amblyomma triste	*Amblyomma triste*	1	6	6
Amblyomma triste	*Amblyomma triste*	1	6	6
Amblyomma triste	*Amblyomma triste*	1	6	6
Amblyomma triste	*Amblyomma triste*	1	6	6
Amblyomma triste	*Amblyomma triste*	1	6	6
Amblyomma triste	*Amblyomma triste*	1	6	6
Amblyomma triste	*Amblyomma triste*	1	6	6
Amblyomma aureolatum	*Amblyomma aureolatum*	1	1	1
Amblyomma aureolatum	*Amblyomma aureolatum*	1	1	1
Amblyomma aureolatum	*Amblyomma aureolatum*	1	1	1
Amblyomma aureolatum	*Amblyomma aureolatum*	1	1	1
Amblyomma aureolatum	*Amblyomma aureolatum*	1	1	1
Amblyomma aureolatum	*Amblyomma aureolatum*	1	1	1
Amblyomma aureolatum	*Amblyomma aureolatum*	1	1	1
Amblyomma aureolatum	*Amblyomma aureolatum*	1	1	1
Amblyomma aureolatum	*Amblyomma aureolatum*	1	1	1
Amblyomma aureolatum	*Amblyomma aureolatum*	1	1	1
Amblyomma aureolatum	*Amblyomma aureolatum*	1	1	1
Amblyomma aureolatum	*Amblyomma aureolatum*	1	1	1
Amblyomma aureolatum	*Amblyomma aureolatum*	1	1	1
Amblyomma aureolatum	*Amblyomma aureolatum*	1	1	1
Amblyomma aureolatum	*Amblyomma aureolatum*	1	1	1
Amblyomma aureolatum	*Amblyomma aureolatum*	1	1	1
Amblyomma aureolatum	*Amblyomma triste*	0	1	6
Amblyomma aureolatum	*Amblyomma aureolatum*	1	1	1
Amblyomma aureolatum	*Amblyomma aureolatum*	1	1	1
Amblyomma aureolatum	*Amblyomma aureolatum*	1	1	1
Amblyomma aureolatum	*Amblyomma aureolatum*	1	1	1
Amblyomma aureolatum	*Amblyomma aureolatum*	1	1	1
Amblyomma aureolatum	*Amblyomma aureolatum*	1	1	1
Amblyomma aureolatum	*Amblyomma aureolatum*	1	1	1
Amblyomma aureolatum	*Amblyomma aureolatum*	1	1	1
Amblyomma cajennense	*Amblyomma cajennense*	1	2	2
Amblyomma cajennense	*Amblyomma cajennense*	1	2	2
Amblyomma cajennense	*Amblyomma cajennense*	1	2	2
Amblyomma cajennense	*Amblyomma cajennense*	1	2	2
Amblyomma cajennense	*Amblyomma cajennense*	1	2	2
Amblyomma cajennense	*Amblyomma cajennense*	1	2	2
Amblyomma cajennense	*Amblyomma cajennense*	1	2	2
Amblyomma cajennense	*Amblyomma triste*	0	2	6
Amblyomma cajennense	*Amblyomma cajennense*	1	2	2
Amblyomma cajennense	*Amblyomma cajennense*	1	2	2
Amblyomma cajennense	*Amblyomma cajennense*	1	2	2
Amblyomma cajennense	*Amblyomma cajennense*	1	2	2
Amblyomma cajennense	*Amblyomma cajennense*	1	2	2
Amblyomma cajennense	*Amblyomma cajennense*	1	2	2

Amblyomma cajennense	*Amblyomma cajennense*	1	2	2
Amblyomma cajennense	*Amblyomma cajennense*	1	2	2
Amblyomma cajennense	*Amblyomma cajennense*	1	2	2
Amblyomma cajennense	*Amblyomma cajennense*	1	2	2
Amblyomma cajennense	*Amblyomma cajennense*	1	2	2
Amblyomma cajennense	*Amblyomma cajennense*	1	2	2
Amblyomma cajennense	*Amblyomma cajennense*	1	2	2
Amblyomma cajennense	*Amblyomma cajennense*	1	2	2
Amblyomma cajennense	*Amblyomma cajennense*	1	2	2
Amblyomma cajennense	*Amblyomma cajennense*	1	2	2
Amblyomma cajennense	*Amblyomma cajennense*	1	2	2
Amblyomma cajennense	*Amblyomma cajennense*	1	2	2
Amblyomma cajennense	*Amblyomma cajennense*	1	2	2
Amblyomma cajennense	*Amblyomma cajennense*	1	2	2
Amblyomma cajennense	*Amblyomma cajennense*	1	2	2
Amblyomma cajennense	*Amblyomma cajennense*	1	2	2
Amblyomma cajennense	*Amblyomma cajennense*	1	2	2
Amblyomma cajennense	*Amblyomma cajennense*	1	2	2
Amblyomma cajennense	*Amblyomma cajennense*	1	2	2
Amblyomma dubitatum	*Amblyomma dubitatum*	1	3	3
Amblyomma dubitatum	*Amblyomma dubitatum*	1	3	3
Amblyomma dubitatum	*Amblyomma dubitatum*	1	3	3
Amblyomma dubitatum	*Amblyomma dubitatum*	1	3	3
Amblyomma dubitatum	*Amblyomma dubitatum*	1	3	3
Amblyomma dubitatum	*Amblyomma ovale*	0	3	4
Amblyomma dubitatum	*Amblyomma dubitatum*	1	3	3
Amblyomma dubitatum	*Amblyomma dubitatum*	1	3	3
Amblyomma dubitatum	*Amblyomma dubitatum*	1	3	3
Amblyomma dubitatum	*Amblyomma dubitatum*	1	3	3
Amblyomma dubitatum	*Amblyomma dubitatum*	1	3	3
Amblyomma dubitatum	*Amblyomma dubitatum*	1	3	3
Amblyomma dubitatum	*Amblyomma dubitatum*	1	3	3
Amblyomma dubitatum	*Amblyomma aureolatum*	0	3	1
Amblyomma dubitatum	*Amblyomma dubitatum*	1	3	3
Amblyomma dubitatum	*Amblyomma sculptum*	0	3	5
Amblyomma dubitatum	*Amblyomma dubitatum*	1	3	3
Amblyomma dubitatum	*Amblyomma dubitatum*	1	3	3
Amblyomma dubitatum	*Amblyomma dubitatum*	1	3	3
Amblyomma dubitatum	*Amblyomma dubitatum*	1	3	3
Amblyomma dubitatum	*Amblyomma sculptum*	0	3	5
Amblyomma dubitatum	*Amblyomma aureolatum*	0	3	1
Amblyomma ovale	*Amblyomma ovale*	1	4	4
Amblyomma ovale	*Amblyomma ovale*	1	4	4
Amblyomma ovale	*Amblyomma ovale*	1	4	4
Amblyomma ovale	*Amblyomma triste*	0	4	6
Amblyomma ovale	*Amblyomma ovale*	1	4	4
Amblyomma ovale	*Amblyomma ovale*	1	4	4
Amblyomma ovale	*Amblyomma ovale*	1	4	4
Amblyomma ovale	*Amblyomma ovale*	1	4	4
Amblyomma ovale	*Amblyomma ovale*	1	4	4
Amblyomma ovale	*Amblyomma ovale*	1	4	4
Amblyomma ovale	*Amblyomma aureolatum*	0	4	1
Amblyomma ovale	*Amblyomma ovale*	1	4	4
Amblyomma ovale	*Amblyomma ovale*	1	4	4
Amblyomma ovale	*Amblyomma ovale*	1	4	4
Amblyomma ovale	*Amblyomma ovale*	1	4	4
Amblyomma ovale	*Amblyomma aureolatum*	0	4	1
Amblyomma ovale	*Amblyomma ovale*	1	4	4
Amblyomma ovale	*Amblyomma ovale*	1	4	4
Amblyomma ovale	*Amblyomma ovale*	1	4	4
Amblyomma ovale	*Amblyomma ovale*	1	4	4
Amblyomma ovale	*Amblyomma ovale*	1	4	4
Amblyomma ovale	*Amblyomma ovale*	1	4	4
Amblyomma ovale	*Amblyomma ovale*	1	4	4
Amblyomma ovale	*Amblyomma ovale*	1	4	4
Amblyomma ovale	*Amblyomma ovale*	1	4	4
Amblyomma sculptum	*Amblyomma sculptum*	1	5	5

Amblyomma sculptum	*Amblyomma sculptum*	1	5	5
Amblyomma sculptum	*Amblyomma sculptum*	1	5	5
Amblyomma sculptum	*Amblyomma sculptum*	1	5	5
Amblyomma sculptum	*Amblyomma sculptum*	1	5	5
Amblyomma sculptum	*Amblyomma sculptum*	1	5	5
Amblyomma sculptum	*Amblyomma sculptum*	1	5	5
Amblyomma sculptum	*Amblyomma sculptum*	1	5	5
Amblyomma sculptum	*Amblyomma sculptum*	1	5	5
Amblyomma sculptum	*Amblyomma sculptum*	1	5	5
Amblyomma sculptum	*Amblyomma sculptum*	1	5	5
Amblyomma sculptum	*Amblyomma sculptum*	1	5	5
Amblyomma sculptum	*Amblyomma sculptum*	1	5	5
Amblyomma sculptum	*Amblyomma sculptum*	1	5	5
Amblyomma sculptum	*Amblyomma sculptum*	1	5	5
Amblyomma sculptum	*Amblyomma sculptum*	1	5	5
Amblyomma sculptum	*Amblyomma sculptum*	1	5	5
Amblyomma sculptum	*Amblyomma sculptum*	1	5	5
Amblyomma sculptum	*Amblyomma sculptum*	1	5	5
Amblyomma sculptum	*Amblyomma sculptum*	1	5	5
Amblyomma sculptum	*Amblyomma ovale*	0	5	4
Amblyomma sculptum	*Amblyomma sculptum*	1	5	5
Amblyomma sculptum	*Amblyomma sculptum*	1	5	5
Amblyomma sculptum	*Amblyomma sculptum*	1	5	5
Amblyomma sculptum	*Amblyomma sculptum*	1	5	5
Amblyomma sculptum	*Amblyomma sculptum*	1	5	5
Amblyomma sculptum	*Amblyomma sculptum*	1	5	5
Amblyomma sculptum	*Amblyomma sculptum*	1	5	5
Amblyomma sculptum	*Amblyomma sculptum*	1	5	5
Amblyomma sculptum	*Amblyomma sculptum*	1	5	5
Amblyomma sculptum	*Amblyomma sculptum*	1	5	5
Amblyomma triste	*Amblyomma triste*	1	6	6
Amblyomma triste	*Amblyomma triste*	1	6	6
Amblyomma triste	*Amblyomma triste*	1	6	6
Amblyomma triste	*Amblyomma triste*	1	6	6
Amblyomma triste	*Amblyomma triste*	1	6	6
Amblyomma triste	*Amblyomma triste*	1	6	6
Amblyomma triste	*Amblyomma triste*	1	6	6
Amblyomma triste	*Amblyomma triste*	1	6	6
Amblyomma triste	*Amblyomma triste*	1	6	6
Amblyomma triste	*Amblyomma triste*	1	6	6
Amblyomma triste	*Amblyomma triste*	1	6	6
Amblyomma triste	*Amblyomma triste*	1	6	6
Amblyomma triste	*Amblyomma triste*	1	6	6
Amblyomma triste	*Amblyomma ovale*	0	6	4
Amblyomma triste	*Amblyomma triste*	1	6	6
Amblyomma triste	*Amblyomma triste*	1	6	6
Amblyomma triste	*Amblyomma triste*	1	6	6
Amblyomma triste	*Amblyomma triste*	1	6	6
Amblyomma triste	*Amblyomma triste*	1	6	6
Amblyomma triste	*Amblyomma triste*	1	6	6
Amblyomma triste	*Amblyomma triste*	1	6	6
Amblyomma triste	*Amblyomma triste*	1	6	6
Amblyomma triste	*Amblyomma triste*	1	6	6
Amblyomma triste	*Amblyomma triste*	1	6	6
Amblyomma triste	*Amblyomma cajennense*	0	6	2
Amblyomma triste	*Amblyomma triste*	1	6	6
Amblyomma triste	*Amblyomma triste*	1	6	6
Amblyomma triste	*Amblyomma triste*	1	6	6
Amblyomma triste	*Amblyomma triste*	1	6	6
Amblyomma triste	*Amblyomma triste*	1	6	6
Amblyomma triste	*Amblyomma triste*	1	6	6
Amblyomma triste	*Amblyomma triste*	1	6	6
Amblyomma aureolatum	*Amblyomma aureolatum*	1	1	1
Amblyomma aureolatum	*Amblyomma aureolatum*	1	1	1
Amblyomma aureolatum	*Amblyomma aureolatum*	1	1	1
Amblyomma aureolatum	*Amblyomma aureolatum*	1	1	1
Amblyomma aureolatum	*Amblyomma aureolatum*	1	1	1

Amblyomma aureolatum	*Amblyomma aureolatum*	1	1	1
Amblyomma aureolatum	*Amblyomma aureolatum*	1	1	1
Amblyomma aureolatum	*Amblyomma aureolatum*	1	1	1
Amblyomma aureolatum	*Amblyomma dubitatum*	0	1	3
Amblyomma aureolatum	*Amblyomma aureolatum*	1	1	1
Amblyomma aureolatum	*Amblyomma aureolatum*	1	1	1
Amblyomma aureolatum	*Amblyomma aureolatum*	1	1	1
Amblyomma aureolatum	*Amblyomma aureolatum*	1	1	1
Amblyomma aureolatum	*Amblyomma aureolatum*	1	1	1
Amblyomma aureolatum	*Amblyomma aureolatum*	1	1	1
Amblyomma aureolatum	*Amblyomma aureolatum*	1	1	1
Amblyomma aureolatum	*Amblyomma aureolatum*	1	1	1
Amblyomma aureolatum	*Amblyomma aureolatum*	1	1	1
Amblyomma aureolatum	*Amblyomma aureolatum*	1	1	1
Amblyomma aureolatum	*Amblyomma aureolatum*	1	1	1
Amblyomma aureolatum	*Amblyomma aureolatum*	1	1	1
Amblyomma aureolatum	*Amblyomma aureolatum*	1	1	1
Amblyomma aureolatum	*Amblyomma aureolatum*	1	1	1
Amblyomma aureolatum	*Amblyomma aureolatum*	1	1	1
Amblyomma cajennense	*Amblyomma cajennense*	1	2	2
Amblyomma cajennense	*Amblyomma cajennense*	1	2	2
Amblyomma cajennense	*Amblyomma cajennense*	1	2	2
Amblyomma cajennense	*Amblyomma cajennense*	1	2	2
Amblyomma cajennense	*Amblyomma cajennense*	1	2	2
Amblyomma cajennense	*Amblyomma cajennense*	1	2	2
Amblyomma cajennense	*Amblyomma cajennense*	1	2	2
Amblyomma cajennense	*Amblyomma cajennense*	1	2	2
Amblyomma cajennense	*Amblyomma cajennense*	1	2	2
Amblyomma cajennense	*Amblyomma cajennense*	1	2	2
Amblyomma cajennense	*Amblyomma cajennense*	1	2	2
Amblyomma cajennense	*Amblyomma cajennense*	1	2	2
Amblyomma cajennense	*Amblyomma cajennense*	1	2	2
Amblyomma cajennense	*Amblyomma cajennense*	1	2	2
Amblyomma cajennense	*Amblyomma cajennense*	1	2	2
Amblyomma cajennense	*Amblyomma aureolatum*	0	2	1
Amblyomma cajennense	*Amblyomma cajennense*	1	2	2
Amblyomma cajennense	*Amblyomma cajennense*	1	2	2
Amblyomma cajennense	*Amblyomma cajennense*	1	2	2
Amblyomma cajennense	*Amblyomma cajennense*	1	2	2
Amblyomma cajennense	*Amblyomma cajennense*	1	2	2
Amblyomma cajennense	*Amblyomma cajennense*	1	2	2
Amblyomma cajennense	*Amblyomma cajennense*	1	2	2
Amblyomma cajennense	*Amblyomma cajennense*	1	2	2
Amblyomma cajennense	*Amblyomma cajennense*	1	2	2
Amblyomma cajennense	*Amblyomma cajennense*	1	2	2
Amblyomma cajennense	*Amblyomma cajennense*	1	2	2
Amblyomma cajennense	*Amblyomma cajennense*	1	2	2
Amblyomma cajennense	*Amblyomma cajennense*	1	2	2
Amblyomma cajennense	*Amblyomma cajennense*	1	2	2
Amblyomma cajennense	*Amblyomma cajennense*	1	2	2
Amblyomma cajennense	*Amblyomma cajennense*	1	2	2
Amblyomma cajennense	*Amblyomma cajennense*	1	2	2
Amblyomma dubitatum	*Amblyomma dubitatum*	1	3	3
Amblyomma dubitatum	*Amblyomma dubitatum*	1	3	3
Amblyomma dubitatum	*Amblyomma dubitatum*	1	3	3
Amblyomma dubitatum	*Amblyomma dubitatum*	1	3	3
Amblyomma dubitatum	*Amblyomma dubitatum*	1	3	3
Amblyomma dubitatum	*Amblyomma dubitatum*	1	3	3
Amblyomma dubitatum	*Amblyomma dubitatum*	1	3	3
Amblyomma dubitatum	*Amblyomma dubitatum*	1	3	3
Amblyomma dubitatum	*Amblyomma dubitatum*	1	3	3
Amblyomma dubitatum	*Amblyomma dubitatum*	1	3	3
Amblyomma dubitatum	*Amblyomma dubitatum*	1	3	3
Amblyomma dubitatum	*Amblyomma dubitatum*	1	3	3
Amblyomma dubitatum	*Amblyomma dubitatum*	1	3	3
Amblyomma dubitatum	*Amblyomma dubitatum*	1	3	3
Amblyomma dubitatum	*Amblyomma dubitatum*	1	3	3

Amblyomma dubitatum	*Amblyomma dubitatum*	1	3	3
Amblyomma dubitatum	*Amblyomma dubitatum*	1	3	3
Amblyomma dubitatum	*Amblyomma dubitatum*	1	3	3
Amblyomma dubitatum	*Amblyomma dubitatum*	1	3	3
Amblyomma dubitatum	*Amblyomma dubitatum*	1	3	3
Amblyomma dubitatum	*Amblyomma dubitatum*	1	3	3
Amblyomma ovale	*Amblyomma ovale*	1	4	4
Amblyomma ovale	*Amblyomma ovale*	1	4	4
Amblyomma ovale	*Amblyomma ovale*	1	4	4
Amblyomma ovale	*Amblyomma ovale*	1	4	4
Amblyomma ovale	*Amblyomma ovale*	1	4	4
Amblyomma ovale	*Amblyomma ovale*	1	4	4
Amblyomma ovale	*Amblyomma ovale*	1	4	4
Amblyomma ovale	*Amblyomma ovale*	1	4	4
Amblyomma ovale	*Amblyomma ovale*	1	4	4
Amblyomma ovale	*Amblyomma ovale*	1	4	4
Amblyomma ovale	*Amblyomma ovale*	1	4	4
Amblyomma ovale	*Amblyomma ovale*	1	4	4
Amblyomma ovale	*Amblyomma ovale*	1	4	4
Amblyomma ovale	*Amblyomma ovale*	1	4	4
Amblyomma ovale	*Amblyomma ovale*	1	4	4
Amblyomma ovale	*Amblyomma ovale*	1	4	4
Amblyomma ovale	*Amblyomma ovale*	1	4	4
Amblyomma ovale	*Amblyomma ovale*	1	4	4
Amblyomma ovale	*Amblyomma ovale*	1	4	4
Amblyomma ovale	*Amblyomma aureolatum*	0	4	1
Amblyomma ovale	*Amblyomma aureolatum*	0	4	1
Amblyomma ovale	*Amblyomma triste*	0	4	6
Amblyomma ovale	*Amblyomma ovale*	1	4	4
Amblyomma ovale	*Amblyomma ovale*	1	4	4
Amblyomma sculptum	*Amblyomma ovale*	0	5	4
Amblyomma sculptum	*Amblyomma sculptum*	1	5	5
Amblyomma sculptum	*Amblyomma sculptum*	1	5	5
Amblyomma sculptum	*Amblyomma sculptum*	1	5	5
Amblyomma sculptum	*Amblyomma sculptum*	1	5	5
Amblyomma sculptum	*Amblyomma sculptum*	1	5	5
Amblyomma sculptum	*Amblyomma sculptum*	1	5	5
Amblyomma sculptum	*Amblyomma sculptum*	1	5	5
Amblyomma sculptum	*Amblyomma sculptum*	1	5	5
Amblyomma sculptum	*Amblyomma sculptum*	1	5	5
Amblyomma sculptum	*Amblyomma sculptum*	1	5	5
Amblyomma sculptum	*Amblyomma ovale*	0	5	4
Amblyomma sculptum	*Amblyomma sculptum*	1	5	5
Amblyomma sculptum	*Amblyomma sculptum*	1	5	5
Amblyomma sculptum	*Amblyomma sculptum*	1	5	5
Amblyomma sculptum	*Amblyomma sculptum*	1	5	5
Amblyomma sculptum	*Amblyomma sculptum*	1	5	5
Amblyomma sculptum	*Amblyomma dubitatum*	0	5	3
Amblyomma sculptum	*Amblyomma dubitatum*	0	5	3
Amblyomma sculptum	*Amblyomma dubitatum*	0	5	3
Amblyomma sculptum	*Amblyomma cajennense*	0	5	2
Amblyomma sculptum	*Amblyomma ovale*	0	5	4
Amblyomma sculptum	*Amblyomma sculptum*	1	5	5
Amblyomma sculptum	*Amblyomma sculptum*	1	5	5
Amblyomma sculptum	*Amblyomma sculptum*	1	5	5
Amblyomma sculptum	*Amblyomma sculptum*	1	5	5
Amblyomma sculptum	*Amblyomma sculptum*	1	5	5
Amblyomma sculptum	*Amblyomma sculptum*	1	5	5
Amblyomma sculptum	*Amblyomma sculptum*	1	5	5
Amblyomma sculptum	*Amblyomma sculptum*	1	5	5
Amblyomma sculptum	*Amblyomma sculptum*	1	5	5
Amblyomma triste	*Amblyomma triste*	1	6	6
Amblyomma triste	*Amblyomma triste*	1	6	6
Amblyomma triste	*Amblyomma triste*	1	6	6
Amblyomma triste	*Amblyomma triste*	1	6	6
Amblyomma triste	*Amblyomma triste*	1	6	6
Amblyomma triste	*Amblyomma triste*	1	6	6

Amblyomma triste	*Amblyomma triste*	1	6	6
Amblyomma triste	*Amblyomma triste*	1	6	6
Amblyomma triste	*Amblyomma triste*	1	6	6
Amblyomma triste	*Amblyomma triste*	1	6	6
Amblyomma triste	*Amblyomma triste*	1	6	6
Amblyomma triste	*Amblyomma triste*	1	6	6
Amblyomma triste	*Amblyomma triste*	1	6	6
Amblyomma triste	*Amblyomma triste*	1	6	6
Amblyomma triste	*Amblyomma triste*	1	6	6
Amblyomma triste	*Amblyomma triste*	1	6	6
Amblyomma triste	*Amblyomma triste*	1	6	6
Amblyomma triste	*Amblyomma triste*	1	6	6
Amblyomma triste	*Amblyomma triste*	1	6	6
Amblyomma triste	*Amblyomma triste*	1	6	6
Amblyomma triste	*Amblyomma triste*	1	6	6
Amblyomma triste	*Amblyomma triste*	1	6	6
Amblyomma triste	*Amblyomma triste*	1	6	6
Amblyomma triste	*Amblyomma triste*	1	6	6
Amblyomma triste	*Amblyomma triste*	1	6	6
Amblyomma triste	*Amblyomma triste*	1	6	6
Amblyomma triste	*Amblyomma triste*	1	6	6
Amblyomma triste	*Amblyomma aureolatum*	0	6	1
Amblyomma triste	*Amblyomma triste*	1	6	6
Amblyomma triste	*Amblyomma triste*	1	6	6
Amblyomma triste	*Amblyomma aureolatum*	0	6	1
Amblyomma aureolatum	*Amblyomma aureolatum*	1	1	1
Amblyomma aureolatum	*Amblyomma aureolatum*	1	1	1
Amblyomma aureolatum	*Amblyomma aureolatum*	1	1	1
Amblyomma aureolatum	*Amblyomma aureolatum*	1	1	1
Amblyomma aureolatum	*Amblyomma aureolatum*	1	1	1
Amblyomma aureolatum	*Amblyomma aureolatum*	1	1	1
Amblyomma aureolatum	*Amblyomma aureolatum*	1	1	1
Amblyomma aureolatum	*Amblyomma aureolatum*	1	1	1
Amblyomma aureolatum	*Amblyomma aureolatum*	1	1	1
Amblyomma aureolatum	*Amblyomma sculptum*	0	1	5
Amblyomma aureolatum	*Amblyomma aureolatum*	1	1	1
Amblyomma cajennense	*Amblyomma cajennense*	1	2	2
Amblyomma cajennense	*Amblyomma cajennense*	1	2	2
Amblyomma cajennense	*Amblyomma cajennense*	1	2	2
Amblyomma cajennense	*Amblyomma cajennense*	1	2	2
Amblyomma cajennense	*Amblyomma cajennense*	1	2	2
Amblyomma cajennense	*Amblyomma aureolatum*	0	2	1
Amblyomma cajennense	*Amblyomma cajennense*	1	2	2
Amblyomma cajennense	*Amblyomma cajennense*	1	2	2
Amblyomma cajennense	*Amblyomma cajennense*	1	2	2
Amblyomma cajennense	*Amblyomma cajennense*	1	2	2
Amblyomma cajennense	*Amblyomma cajennense*	1	2	2
Amblyomma cajennense	*Amblyomma cajennense*	1	2	2
Amblyomma cajennense	*Amblyomma cajennense*	1	2	2
Amblyomma cajennense	*Amblyomma aureolatum*	0	2	1
Amblyomma cajennense	*Amblyomma cajennense*	1	2	2
Amblyomma cajennense	*Amblyomma cajennense*	1	2	2
Amblyomma cajennense	*Amblyomma cajennense*	1	2	2
Amblyomma dubitatum	*Amblyomma ovale*	0	3	4
Amblyomma dubitatum	*Amblyomma cajennense*	0	3	2
Amblyomma dubitatum	*Amblyomma dubitatum*	1	3	3
Amblyomma dubitatum	*Amblyomma dubitatum*	1	3	3
Amblyomma dubitatum	*Amblyomma dubitatum*	1	3	3
Amblyomma dubitatum	*Amblyomma cajennense*	0	3	2
Amblyomma dubitatum	*Amblyomma aureolatum*	0	3	1
Amblyomma ovale	*Amblyomma ovale*	1	4	4
Amblyomma ovale	*Amblyomma ovale*	1	4	4
Amblyomma ovale	*Amblyomma ovale*	1	4	4
Amblyomma ovale	*Amblyomma ovale*	1	4	4
Amblyomma ovale	*Amblyomma ovale*	1	4	4
Amblyomma ovale	*Amblyomma dubitatum*	0	4	3
Amblyomma ovale	*Amblyomma ovale*	1	4	4

Amblyomma ovale	*Amblyomma ovale*	1	4	4
Amblyomma ovale	*Amblyomma ovale*	1	4	4
Amblyomma ovale	*Amblyomma ovale*	1	4	4
Amblyomma ovale	*Amblyomma ovale*	1	4	4
Amblyomma ovale	*Amblyomma ovale*	1	4	4
Amblyomma ovale	*Amblyomma ovale*	1	4	4
Amblyomma ovale	*Amblyomma ovale*	1	4	4
Amblyomma ovale	*Amblyomma ovale*	1	4	4
Amblyomma ovale	*Amblyomma ovale*	1	4	4
Amblyomma sculptum	*Amblyomma sculptum*	1	5	5
Amblyomma sculptum	*Amblyomma sculptum*	1	5	5
Amblyomma sculptum	*Amblyomma sculptum*	1	5	5
Amblyomma sculptum	*Amblyomma sculptum*	1	5	5
Amblyomma sculptum	*Amblyomma sculptum*	1	5	5
Amblyomma sculptum	*Amblyomma sculptum*	1	5	5
Amblyomma sculptum	*Amblyomma dubitatum*	0	5	3
Amblyomma sculptum	*Amblyomma sculptum*	1	5	5
Amblyomma sculptum	*Amblyomma dubitatum*	0	5	3
Amblyomma sculptum	*Amblyomma dubitatum*	0	5	3
Amblyomma sculptum	*Amblyomma ovale*	0	5	4
Amblyomma sculptum	*Amblyomma sculptum*	1	5	5
Amblyomma sculptum	*Amblyomma sculptum*	1	5	5
Amblyomma triste	*Amblyomma ovale*	0	6	4
Amblyomma triste	*Amblyomma triste*	1	6	6
Amblyomma triste	*Amblyomma triste*	1	6	6
Amblyomma triste	*Amblyomma triste*	1	6	6
Amblyomma triste	*Amblyomma triste*	1	6	6
Amblyomma triste	*Amblyomma triste*	1	6	6
Amblyomma triste	*Amblyomma triste*	1	6	6
Amblyomma triste	*Amblyomma triste*	1	6	6
Amblyomma triste	*Amblyomma triste*	1	6	6
Amblyomma triste	*Amblyomma ovale*	0	6	4
Amblyomma triste	*Amblyomma triste*	1	6	6
Amblyomma aureolatum	*Amblyomma aureolatum*	1	1	1
Amblyomma aureolatum	*Amblyomma aureolatum*	1	1	1
Amblyomma aureolatum	*Amblyomma aureolatum*	1	1	1
Amblyomma aureolatum	*Amblyomma aureolatum*	1	1	1
Amblyomma aureolatum	*Amblyomma aureolatum*	1	1	1
Amblyomma aureolatum	*Amblyomma aureolatum*	1	1	1
Amblyomma aureolatum	*Amblyomma aureolatum*	1	1	1
Amblyomma aureolatum	*Amblyomma aureolatum*	1	1	1
Amblyomma aureolatum	*Amblyomma aureolatum*	1	1	1
Amblyomma aureolatum	*Amblyomma aureolatum*	1	1	1
Amblyomma aureolatum	*Amblyomma aureolatum*	1	1	1
Amblyomma aureolatum	*Amblyomma aureolatum*	1	1	1
Amblyomma cajennense	*Amblyomma cajennense*	1	2	2
Amblyomma cajennense	*Amblyomma cajennense*	1	2	2
Amblyomma cajennense	*Amblyomma cajennense*	1	2	2
Amblyomma cajennense	*Amblyomma triste*	0	2	6
Amblyomma cajennense	*Amblyomma cajennense*	1	2	2
Amblyomma cajennense	*Amblyomma cajennense*	1	2	2
Amblyomma cajennense	*Amblyomma cajennense*	1	2	2
Amblyomma cajennense	*Amblyomma cajennense*	1	2	2
Amblyomma cajennense	*Amblyomma cajennense*	1	2	2
Amblyomma cajennense	*Amblyomma cajennense*	1	2	2
Amblyomma cajennense	*Amblyomma cajennense*	1	2	2
Amblyomma cajennense	*Amblyomma cajennense*	1	2	2
Amblyomma cajennense	*Amblyomma cajennense*	1	2	2
Amblyomma cajennense	*Amblyomma cajennense*	1	2	2
Amblyomma cajennense	*Amblyomma cajennense*	1	2	2
Amblyomma cajennense	*Amblyomma cajennense*	1	2	2
Amblyomma cajennense	*Amblyomma cajennense*	1	2	2
Amblyomma dubitatum	*Amblyomma dubitatum*	1	3	3
Amblyomma dubitatum	*Amblyomma cajennense*	0	3	2
Amblyomma dubitatum	*Amblyomma dubitatum*	1	3	3
Amblyomma dubitatum	*Amblyomma dubitatum*	1	3	3
Amblyomma dubitatum	*Amblyomma aureolatum*	0	3	1

Amblyomma dubitatum	*Amblyomma sculptum*	0	3	5
Amblyomma dubitatum	*Amblyomma dubitatum*	1	3	3
Amblyomma ovale	*Amblyomma cajennense*	0	4	2
Amblyomma ovale	*Amblyomma ovale*	1	4	4
Amblyomma ovale	*Amblyomma ovale*	1	4	4
Amblyomma ovale	*Amblyomma ovale*	1	4	4
Amblyomma ovale	*Amblyomma ovale*	1	4	4
Amblyomma ovale	*Amblyomma ovale*	1	4	4
Amblyomma ovale	*Amblyomma ovale*	1	4	4
Amblyomma ovale	*Amblyomma ovale*	1	4	4
Amblyomma ovale	*Amblyomma ovale*	1	4	4
Amblyomma ovale	*Amblyomma ovale*	1	4	4
Amblyomma ovale	*Amblyomma dubitatum*	0	4	3
Amblyomma ovale	*Amblyomma cajennense*	0	4	2
Amblyomma ovale	*Amblyomma ovale*	1	4	4
Amblyomma ovale	*Amblyomma ovale*	1	4	4
Amblyomma ovale	*Amblyomma ovale*	1	4	4
Amblyomma ovale	*Amblyomma ovale*	1	4	4
Amblyomma sculptum	*Amblyomma sculptum*	1	5	5
Amblyomma sculptum	*Amblyomma sculptum*	1	5	5
Amblyomma sculptum	*Amblyomma sculptum*	1	5	5
Amblyomma sculptum	*Amblyomma sculptum*	1	5	5
Amblyomma sculptum	*Amblyomma sculptum*	1	5	5
Amblyomma sculptum	*Amblyomma cajennense*	0	5	2
Amblyomma sculptum	*Amblyomma sculptum*	1	5	5
Amblyomma sculptum	*Amblyomma sculptum*	1	5	5
Amblyomma sculptum	*Amblyomma sculptum*	1	5	5
Amblyomma sculptum	*Amblyomma sculptum*	1	5	5
Amblyomma sculptum	*Amblyomma sculptum*	1	5	5
Amblyomma sculptum	*Amblyomma sculptum*	1	5	5
Amblyomma triste	*Amblyomma triste*	1	6	6
Amblyomma triste	*Amblyomma triste*	1	6	6
Amblyomma triste	*Amblyomma triste*	1	6	6
Amblyomma triste	*Amblyomma triste*	1	6	6
Amblyomma triste	*Amblyomma triste*	1	6	6
Amblyomma triste	*Amblyomma triste*	1	6	6
Amblyomma triste	*Amblyomma triste*	1	6	6
Amblyomma triste	*Amblyomma triste*	1	6	6
Amblyomma triste	*Amblyomma triste*	1	6	6
Amblyomma triste	*Amblyomma triste*	1	6	6
Amblyomma aureolatum	*Amblyomma aureolatum*	1	1	1
Amblyomma aureolatum	*Amblyomma aureolatum*	1	1	1
Amblyomma aureolatum	*Amblyomma cajennense*	0	1	2
Amblyomma aureolatum	*Amblyomma aureolatum*	1	1	1
Amblyomma aureolatum	*Amblyomma aureolatum*	1	1	1
Amblyomma aureolatum	*Amblyomma aureolatum*	1	1	1
Amblyomma aureolatum	*Amblyomma aureolatum*	1	1	1
Amblyomma aureolatum	*Amblyomma aureolatum*	1	1	1
Amblyomma aureolatum	*Amblyomma aureolatum*	1	1	1
Amblyomma aureolatum	*Amblyomma triste*	0	1	6
Amblyomma aureolatum	*Amblyomma triste*	0	1	6
Amblyomma cajennense	*Amblyomma cajennense*	1	2	2
Amblyomma cajennense	*Amblyomma cajennense*	1	2	2
Amblyomma cajennense	*Amblyomma cajennense*	1	2	2
Amblyomma cajennense	*Amblyomma cajennense*	1	2	2
Amblyomma cajennense	*Amblyomma cajennense*	1	2	2
Amblyomma cajennense	*Amblyomma cajennense*	1	2	2
Amblyomma cajennense	*Amblyomma cajennense*	1	2	2
Amblyomma cajennense	*Amblyomma cajennense*	1	2	2
Amblyomma cajennense	*Amblyomma cajennense*	1	2	2
Amblyomma cajennense	*Amblyomma cajennense*	1	2	2
Amblyomma cajennense	*Amblyomma cajennense*	1	2	2
Amblyomma cajennense	*Amblyomma cajennense*	1	2	2
Amblyomma cajennense	*Amblyomma cajennense*	1	2	2
Amblyomma cajennense	*Amblyomma cajennense*	1	2	2
Amblyomma cajennense	*Amblyomma cajennense*	1	2	2
Amblyomma cajennense	*Amblyomma cajennense*	1	2	2

Amblyomma dubitatum	*Amblyomma cajennense*	0	3	2
Amblyomma dubitatum	*Amblyomma sculptum*	0	3	5
Amblyomma dubitatum	*Amblyomma dubitatum*	1	3	3
Amblyomma dubitatum	*Amblyomma dubitatum*	1	3	3
Amblyomma dubitatum	*Amblyomma cajennense*	0	3	2
Amblyomma dubitatum	*Amblyomma dubitatum*	1	3	3
Amblyomma dubitatum	*Amblyomma dubitatum*	1	3	3
Amblyomma ovale	*Amblyomma ovale*	1	4	4
Amblyomma ovale	*Amblyomma ovale*	1	4	4
Amblyomma ovale	*Amblyomma ovale*	1	4	4
Amblyomma ovale	*Amblyomma ovale*	1	4	4
Amblyomma ovale	*Amblyomma ovale*	1	4	4
Amblyomma ovale	*Amblyomma ovale*	1	4	4
Amblyomma ovale	*Amblyomma ovale*	1	4	4
Amblyomma ovale	*Amblyomma ovale*	1	4	4
Amblyomma ovale	*Amblyomma ovale*	1	4	4
Amblyomma ovale	*Amblyomma ovale*	1	4	4
Amblyomma ovale	*Amblyomma ovale*	1	4	4
Amblyomma ovale	*Amblyomma ovale*	1	4	4
Amblyomma ovale	*Amblyomma ovale*	1	4	4
Amblyomma ovale	*Amblyomma ovale*	1	4	4
Amblyomma ovale	*Amblyomma ovale*	1	4	4
Amblyomma sculptum	*Amblyomma sculptum*	1	5	5
Amblyomma sculptum	*Amblyomma sculptum*	1	5	5
Amblyomma sculptum	*Amblyomma sculptum*	1	5	5
Amblyomma sculptum	*Amblyomma sculptum*	1	5	5
Amblyomma sculptum	*Amblyomma sculptum*	1	5	5
Amblyomma sculptum	*Amblyomma sculptum*	1	5	5
Amblyomma sculptum	*Amblyomma sculptum*	1	5	5
Amblyomma sculptum	*Amblyomma sculptum*	1	5	5
Amblyomma sculptum	*Amblyomma sculptum*	1	5	5
Amblyomma sculptum	*Amblyomma sculptum*	1	5	5
Amblyomma sculptum	*Amblyomma sculptum*	1	5	5
Amblyomma sculptum	*Amblyomma sculptum*	1	5	5
Amblyomma sculptum	*Amblyomma cajennense*	0	5	2
Amblyomma triste	*Amblyomma sculptum*	0	6	5
Amblyomma triste	*Amblyomma cajennense*	0	6	2
Amblyomma triste	*Amblyomma triste*	1	6	6
Amblyomma triste	*Amblyomma sculptum*	0	6	5
Amblyomma triste	*Amblyomma triste*	1	6	6
Amblyomma triste	*Amblyomma ovale*	0	6	4
Amblyomma triste	*Amblyomma triste*	1	6	6
Amblyomma triste	*Amblyomma triste*	1	6	6
Amblyomma triste	*Amblyomma triste*	1	6	6
Amblyomma triste	*Amblyomma triste*	1	6	6
Amblyomma aureolatum	*Amblyomma aureolatum*	1	1	1
Amblyomma aureolatum	*Amblyomma aureolatum*	1	1	1
Amblyomma aureolatum	*Amblyomma aureolatum*	1	1	1
Amblyomma aureolatum	*Amblyomma aureolatum*	1	1	1
Amblyomma aureolatum	*Amblyomma dubitatum*	0	1	3
Amblyomma aureolatum	*Amblyomma aureolatum*	1	1	1
Amblyomma aureolatum	*Amblyomma aureolatum*	1	1	1
Amblyomma aureolatum	*Amblyomma aureolatum*	1	1	1
Amblyomma aureolatum	*Amblyomma aureolatum*	1	1	1
Amblyomma aureolatum	*Amblyomma aureolatum*	1	1	1
Amblyomma aureolatum	*Amblyomma aureolatum*	1	1	1
Amblyomma aureolatum	*Amblyomma aureolatum*	1	1	1
Amblyomma cajennense	*Amblyomma cajennense*	1	2	2
Amblyomma cajennense	*Amblyomma cajennense*	1	2	2
Amblyomma cajennense	*Amblyomma cajennense*	1	2	2
Amblyomma cajennense	*Amblyomma cajennense*	1	2	2
Amblyomma cajennense	*Amblyomma cajennense*	1	2	2
Amblyomma cajennense	*Amblyomma cajennense*	1	2	2
Amblyomma cajennense	*Amblyomma cajennense*	1	2	2
Amblyomma cajennense	*Amblyomma cajennense*	1	2	2
Amblyomma cajennense	*Amblyomma cajennense*	1	2	2
Amblyomma cajennense	*Amblyomma cajennense*	1	2	2

Amblyomma cajennense	*Amblyomma cajennense*	1	2	2
Amblyomma cajennense	*Amblyomma cajennense*	1	2	2
Amblyomma cajennense	*Amblyomma cajennense*	1	2	2
Amblyomma cajennense	*Amblyomma cajennense*	1	2	2
Amblyomma cajennense	*Amblyomma cajennense*	1	2	2
Amblyomma cajennense	*Amblyomma cajennense*	1	2	2
Amblyomma dubitatum	*Amblyomma dubitatum*	1	3	3
Amblyomma dubitatum	*Amblyomma dubitatum*	1	3	3
Amblyomma dubitatum	*Amblyomma dubitatum*	1	3	3
Amblyomma dubitatum	*Amblyomma dubitatum*	1	3	3
Amblyomma dubitatum	*Amblyomma sculptum*	0	3	5
Amblyomma dubitatum	*Amblyomma dubitatum*	1	3	3
Amblyomma ovale	*Amblyomma dubitatum*	0	4	3
Amblyomma ovale	*Amblyomma ovale*	1	4	4
Amblyomma ovale	*Amblyomma ovale*	1	4	4
Amblyomma ovale	*Amblyomma ovale*	1	4	4
Amblyomma ovale	*Amblyomma ovale*	1	4	4
Amblyomma ovale	*Amblyomma ovale*	1	4	4
Amblyomma ovale	*Amblyomma ovale*	1	4	4
Amblyomma ovale	*Amblyomma ovale*	1	4	4
Amblyomma ovale	*Amblyomma ovale*	1	4	4
Amblyomma ovale	*Amblyomma ovale*	1	4	4
Amblyomma ovale	*Amblyomma aureolatum*	0	4	1
Amblyomma ovale	*Amblyomma ovale*	1	4	4
Amblyomma ovale	*Amblyomma ovale*	1	4	4
Amblyomma ovale	*Amblyomma ovale*	1	4	4
Amblyomma ovale	*Amblyomma ovale*	1	4	4
Amblyomma ovale	*Amblyomma dubitatum*	0	4	3
Amblyomma sculptum	*Amblyomma sculptum*	1	5	5
Amblyomma sculptum	*Amblyomma sculptum*	1	5	5
Amblyomma sculptum	*Amblyomma triste*	0	5	6
Amblyomma sculptum	*Amblyomma sculptum*	1	5	5
Amblyomma sculptum	*Amblyomma sculptum*	1	5	5
Amblyomma sculptum	*Amblyomma triste*	0	5	6
Amblyomma sculptum	*Amblyomma sculptum*	1	5	5
Amblyomma sculptum	*Amblyomma sculptum*	1	5	5
Amblyomma sculptum	*Amblyomma sculptum*	1	5	5
Amblyomma sculptum	*Amblyomma dubitatum*	0	5	3
Amblyomma sculptum	*Amblyomma sculptum*	1	5	5
Amblyomma sculptum	*Amblyomma dubitatum*	0	5	3
Amblyomma sculptum	*Amblyomma sculptum*	1	5	5
Amblyomma triste	*Amblyomma triste*	1	6	6
Amblyomma triste	*Amblyomma triste*	1	6	6
Amblyomma triste	*Amblyomma triste*	1	6	6
Amblyomma triste	*Amblyomma triste*	1	6	6
Amblyomma triste	*Amblyomma triste*	1	6	6
Amblyomma triste	*Amblyomma triste*	1	6	6
Amblyomma triste	*Amblyomma triste*	1	6	6
Amblyomma triste	*Amblyomma triste*	1	6	6
Amblyomma triste	*Amblyomma triste*	1	6	6
Amblyomma triste	*Amblyomma triste*	1	6	6
Amblyomma aureolatum	*Amblyomma aureolatum*	1	1	1
Amblyomma aureolatum	*Amblyomma aureolatum*	1	1	1
Amblyomma aureolatum	*Amblyomma aureolatum*	1	1	1
Amblyomma aureolatum	*Amblyomma aureolatum*	1	1	1
Amblyomma aureolatum	*Amblyomma aureolatum*	1	1	1
Amblyomma aureolatum	*Amblyomma aureolatum*	1	1	1
Amblyomma aureolatum	*Amblyomma aureolatum*	1	1	1
Amblyomma aureolatum	*Amblyomma aureolatum*	1	1	1
Amblyomma aureolatum	*Amblyomma aureolatum*	1	1	1
Amblyomma aureolatum	*Amblyomma aureolatum*	1	1	1
Amblyomma aureolatum	*Amblyomma triste*	0	1	6
Amblyomma aureolatum	*Amblyomma aureolatum*	1	1	1
Amblyomma cajennense	*Amblyomma cajennense*	1	2	2
Amblyomma cajennense	*Amblyomma cajennense*	1	2	2
Amblyomma cajennense	*Amblyomma cajennense*	1	2	2
Amblyomma cajennense	*Amblyomma cajennense*	1	2	2

Amblyomma cajennense	*Amblyomma cajennense*	1	2	2
Amblyomma cajennense	*Amblyomma cajennense*	1	2	2
Amblyomma cajennense	*Amblyomma cajennense*	1	2	2
Amblyomma cajennense	*Amblyomma cajennense*	1	2	2
Amblyomma cajennense	*Amblyomma cajennense*	1	2	2
Amblyomma cajennense	*Amblyomma cajennense*	1	2	2
Amblyomma cajennense	*Amblyomma cajennense*	1	2	2
Amblyomma cajennense	*Amblyomma cajennense*	1	2	2
Amblyomma cajennense	*Amblyomma cajennense*	1	2	2
Amblyomma cajennense	*Amblyomma cajennense*	1	2	2
Amblyomma cajennense	*Amblyomma cajennense*	1	2	2
Amblyomma cajennense	*Amblyomma cajennense*	1	2	2
Amblyomma dubitatum	*Amblyomma triste*	0	3	6
Amblyomma dubitatum	*Amblyomma dubitatum*	1	3	3
Amblyomma dubitatum	*Amblyomma aureolatum*	0	3	1
Amblyomma dubitatum	*Amblyomma dubitatum*	1	3	3
Amblyomma dubitatum	*Amblyomma triste*	0	3	6
Amblyomma dubitatum	*Amblyomma dubitatum*	1	3	3
Amblyomma dubitatum	*Amblyomma dubitatum*	1	3	3
Amblyomma ovale	*Amblyomma ovale*	1	4	4
Amblyomma ovale	*Amblyomma ovale*	1	4	4
Amblyomma ovale	*Amblyomma ovale*	1	4	4
Amblyomma ovale	*Amblyomma ovale*	1	4	4
Amblyomma ovale	*Amblyomma ovale*	1	4	4
Amblyomma ovale	*Amblyomma ovale*	1	4	4
Amblyomma ovale	*Amblyomma ovale*	1	4	4
Amblyomma ovale	*Amblyomma ovale*	1	4	4
Amblyomma ovale	*Amblyomma ovale*	1	4	4
Amblyomma ovale	*Amblyomma ovale*	1	4	4
Amblyomma ovale	*Amblyomma ovale*	1	4	4
Amblyomma ovale	*Amblyomma ovale*	1	4	4
Amblyomma ovale	*Amblyomma ovale*	1	4	4
Amblyomma ovale	*Amblyomma ovale*	1	4	4
Amblyomma ovale	*Amblyomma ovale*	1	4	4
Amblyomma ovale	*Amblyomma ovale*	1	4	4
Amblyomma sculptum	*Amblyomma sculptum*	1	5	5
Amblyomma sculptum	*Amblyomma sculptum*	1	5	5
Amblyomma sculptum	*Amblyomma sculptum*	1	5	5
Amblyomma sculptum	*Amblyomma sculptum*	1	5	5
Amblyomma sculptum	*Amblyomma sculptum*	1	5	5
Amblyomma sculptum	*Amblyomma sculptum*	1	5	5
Amblyomma sculptum	*Amblyomma triste*	0	5	6
Amblyomma sculptum	*Amblyomma sculptum*	1	5	5
Amblyomma sculptum	*Amblyomma sculptum*	1	5	5
Amblyomma sculptum	*Amblyomma sculptum*	1	5	5
Amblyomma sculptum	*Amblyomma sculptum*	1	5	5
Amblyomma sculptum	*Amblyomma sculptum*	1	5	5
Amblyomma triste	*Amblyomma triste*	1	6	6
Amblyomma triste	*Amblyomma triste*	1	6	6
Amblyomma triste	*Amblyomma triste*	1	6	6
Amblyomma triste	*Amblyomma triste*	1	6	6
Amblyomma triste	*Amblyomma triste*	1	6	6
Amblyomma triste	*Amblyomma triste*	1	6	6
Amblyomma triste	*Amblyomma triste*	1	6	6
Amblyomma triste	*Amblyomma triste*	1	6	6
Amblyomma triste	*Amblyomma triste*	1	6	6
Amblyomma triste	*Amblyomma triste*	1	6	6
Amblyomma triste	*Amblyomma triste*	1	6	6
Amblyomma aureolatum	*Amblyomma aureolatum*	1	1	1
Amblyomma aureolatum	*Amblyomma aureolatum*	1	1	1
Amblyomma aureolatum	*Amblyomma aureolatum*	1	1	1
Amblyomma aureolatum	*Amblyomma aureolatum*	1	1	1
Amblyomma aureolatum	*Amblyomma aureolatum*	1	1	1
Amblyomma aureolatum	*Amblyomma aureolatum*	1	1	1
Amblyomma aureolatum	*Amblyomma dubitatum*	0	1	3
Amblyomma aureolatum	*Amblyomma aureolatum*	1	1	1
Amblyomma aureolatum	*Amblyomma aureolatum*	1	1	1

Amblyomma aureolatum	*Amblyomma aureolatum*	1	1	1
Amblyomma aureolatum	*Amblyomma aureolatum*	1	1	1
Amblyomma aureolatum	*Amblyomma aureolatum*	1	1	1
Amblyomma cajennense	*Amblyomma cajennense*	1	2	2
Amblyomma cajennense	*Amblyomma cajennense*	1	2	2
Amblyomma cajennense	*Amblyomma cajennense*	1	2	2
Amblyomma cajennense	*Amblyomma cajennense*	1	2	2
Amblyomma cajennense	*Amblyomma cajennense*	1	2	2
Amblyomma cajennense	*Amblyomma cajennense*	1	2	2
Amblyomma cajennense	*Amblyomma cajennense*	1	2	2
Amblyomma cajennense	*Amblyomma cajennense*	1	2	2
Amblyomma cajennense	*Amblyomma cajennense*	1	2	2
Amblyomma cajennense	*Amblyomma cajennense*	1	2	2
Amblyomma cajennense	*Amblyomma cajennense*	1	2	2
Amblyomma cajennense	*Amblyomma cajennense*	1	2	2
Amblyomma cajennense	*Amblyomma cajennense*	1	2	2
Amblyomma cajennense	*Amblyomma cajennense*	1	2	2
Amblyomma cajennense	*Amblyomma cajennense*	1	2	2
Amblyomma cajennense	*Amblyomma sculptum*	0	2	5
Amblyomma dubitatum	*Amblyomma dubitatum*	1	3	3
Amblyomma dubitatum	*Amblyomma dubitatum*	1	3	3
Amblyomma dubitatum	*Amblyomma dubitatum*	1	3	3
Amblyomma dubitatum	*Amblyomma dubitatum*	1	3	3
Amblyomma dubitatum	*Amblyomma dubitatum*	1	3	3
Amblyomma dubitatum	*Amblyomma dubitatum*	1	3	3
Amblyomma dubitatum	*Amblyomma dubitatum*	1	3	3
Amblyomma dubitatum	*Amblyomma dubitatum*	1	3	3
Amblyomma dubitatum	*Amblyomma dubitatum*	1	3	3
Amblyomma dubitatum	*Amblyomma dubitatum*	1	3	3
Amblyomma dubitatum	*Amblyomma dubitatum*	1	3	3
Amblyomma dubitatum	*Amblyomma dubitatum*	1	3	3
Amblyomma dubitatum	*Amblyomma dubitatum*	1	3	3
Amblyomma dubitatum	*Amblyomma dubitatum*	1	3	3
Amblyomma dubitatum	*Amblyomma dubitatum*	1	3	3
Amblyomma ovale	*Amblyomma ovale*	1	4	4
Amblyomma ovale	*Amblyomma ovale*	1	4	4
Amblyomma ovale	*Amblyomma aureolatum*	0	4	1
Amblyomma ovale	*Amblyomma ovale*	1	4	4
Amblyomma ovale	*Amblyomma ovale*	1	4	4
Amblyomma ovale	*Amblyomma ovale*	1	4	4
Amblyomma ovale	*Amblyomma ovale*	1	4	4
Amblyomma ovale	*Amblyomma ovale*	1	4	4
Amblyomma sculptum	*Amblyomma sculptum*	1	5	5
Amblyomma sculptum	*Amblyomma sculptum*	1	5	5
Amblyomma sculptum	*Amblyomma sculptum*	1	5	5
Amblyomma sculptum	*Amblyomma sculptum*	1	5	5
Amblyomma sculptum	*Amblyomma sculptum*	1	5	5
Amblyomma sculptum	*Amblyomma sculptum*	1	5	5
Amblyomma sculptum	*Amblyomma sculptum*	1	5	5
Amblyomma sculptum	*Amblyomma sculptum*	1	5	5
Amblyomma sculptum	*Amblyomma sculptum*	1	5	5
Amblyomma sculptum	*Amblyomma sculptum*	1	5	5
Amblyomma sculptum	*Amblyomma sculptum*	1	5	5
Amblyomma sculptum	*Amblyomma sculptum*	1	5	5
Amblyomma sculptum	*Amblyomma sculptum*	1	5	5
Amblyomma sculptum	*Amblyomma sculptum*	1	5	5
Amblyomma sculptum	*Amblyomma sculptum*	1	5	5
Amblyomma sculptum	*Amblyomma sculptum*	1	5	5
Amblyomma sculptum	*Amblyomma sculptum*	1	5	5
Amblyomma sculptum	*Amblyomma sculptum*	1	5	5
Amblyomma triste	*Amblyomma cajennense*	0	6	2
Amblyomma triste	*Amblyomma triste*	1	6	6
Amblyomma triste	*Amblyomma triste*	1	6	6
Amblyomma triste	*Amblyomma triste*	1	6	6
Amblyomma triste	*Amblyomma triste*	1	6	6
Amblyomma triste	*Amblyomma triste*	1	6	6
Amblyomma triste	*Amblyomma triste*	1	6	6

Amblyomma triste	*Amblyomma triste*	1	6	6
Amblyomma triste	*Amblyomma triste*	1	6	6
Amblyomma triste	*Amblyomma triste*	1	6	6
Amblyomma triste	*Amblyomma triste*	1	6	6
Amblyomma triste	*Amblyomma triste*	1	6	6
Amblyomma triste	*Amblyomma triste*	1	6	6
Amblyomma triste	*Amblyomma triste*	1	6	6
Amblyomma triste	*Amblyomma triste*	1	6	6
Amblyomma triste	*Amblyomma triste*	1	6	6
Amblyomma triste	*Amblyomma triste*	1	6	6
Amblyomma triste	*Amblyomma triste*	1	6	6
Amblyomma triste	*Amblyomma triste*	1	6	6
Amblyomma triste	*Amblyomma triste*	1	6	6
Amblyomma aureolatum	*Amblyomma aureolatum*	1	1	1
Amblyomma aureolatum	*Amblyomma ovale*	0	1	4
Amblyomma aureolatum	*Amblyomma aureolatum*	1	1	1
Amblyomma aureolatum	*Amblyomma aureolatum*	1	1	1
Amblyomma aureolatum	*Amblyomma aureolatum*	1	1	1
Amblyomma aureolatum	*Amblyomma aureolatum*	1	1	1
Amblyomma aureolatum	*Amblyomma aureolatum*	1	1	1
Amblyomma aureolatum	*Amblyomma aureolatum*	1	1	1
Amblyomma aureolatum	*Amblyomma aureolatum*	1	1	1
Amblyomma aureolatum	*Amblyomma aureolatum*	1	1	1
Amblyomma aureolatum	*Amblyomma aureolatum*	1	1	1
Amblyomma aureolatum	*Amblyomma ovale*	0	1	4
Amblyomma aureolatum	*Amblyomma aureolatum*	1	1	1
Amblyomma cajennense	*Amblyomma cajennense*	1	2	2
Amblyomma cajennense	*Amblyomma cajennense*	1	2	2
Amblyomma cajennense	*Amblyomma cajennense*	1	2	2
Amblyomma cajennense	*Amblyomma cajennense*	1	2	2
Amblyomma cajennense	*Amblyomma cajennense*	1	2	2
Amblyomma cajennense	*Amblyomma cajennense*	1	2	2
Amblyomma cajennense	*Amblyomma cajennense*	1	2	2
Amblyomma cajennense	*Amblyomma cajennense*	1	2	2
Amblyomma cajennense	*Amblyomma cajennense*	1	2	2
Amblyomma cajennense	*Amblyomma cajennense*	1	2	2
Amblyomma cajennense	*Amblyomma cajennense*	1	2	2
Amblyomma cajennense	*Amblyomma cajennense*	1	2	2
Amblyomma cajennense	*Amblyomma cajennense*	1	2	2
Amblyomma cajennense	*Amblyomma cajennense*	1	2	2
Amblyomma cajennense	*Amblyomma cajennense*	1	2	2
Amblyomma cajennense	*Amblyomma cajennense*	1	2	2
Amblyomma cajennense	*Amblyomma cajennense*	1	2	2
Amblyomma dubitatum	*Amblyomma cajennense*	0	3	2
Amblyomma dubitatum	*Amblyomma dubitatum*	1	3	3
Amblyomma dubitatum	*Amblyomma dubitatum*	1	3	3
Amblyomma dubitatum	*Amblyomma dubitatum*	1	3	3
Amblyomma dubitatum	*Amblyomma dubitatum*	1	3	3
Amblyomma dubitatum	*Amblyomma dubitatum*	1	3	3
Amblyomma dubitatum	*Amblyomma dubitatum*	1	3	3
Amblyomma dubitatum	*Amblyomma dubitatum*	1	3	3
Amblyomma dubitatum	*Amblyomma ovale*	0	3	4
Amblyomma dubitatum	*Amblyomma dubitatum*	1	3	3
Amblyomma dubitatum	*Amblyomma dubitatum*	1	3	3
Amblyomma dubitatum	*Amblyomma dubitatum*	1	3	3
Amblyomma dubitatum	*Amblyomma dubitatum*	1	3	3
Amblyomma dubitatum	*Amblyomma dubitatum*	1	3	3
Amblyomma dubitatum	*Amblyomma dubitatum*	1	3	3
Amblyomma ovale	*Amblyomma ovale*	1	4	4
Amblyomma ovale	*Amblyomma ovale*	1	4	4
Amblyomma ovale	*Amblyomma ovale*	1	4	4
Amblyomma ovale	*Amblyomma ovale*	1	4	4
Amblyomma ovale	*Amblyomma ovale*	1	4	4
Amblyomma ovale	*Amblyomma ovale*	1	4	4
Amblyomma ovale	*Amblyomma aureolatum*	0	4	1
Amblyomma ovale	*Amblyomma ovale*	1	4	4
Amblyomma ovale	*Amblyomma ovale*	1	4	4

Amblyomma sculptum	*Amblyomma sculptum*	1	5	5
Amblyomma sculptum	*Amblyomma sculptum*	1	5	5
Amblyomma sculptum	*Amblyomma sculptum*	1	5	5
Amblyomma sculptum	*Amblyomma sculptum*	1	5	5
Amblyomma sculptum	*Amblyomma sculptum*	1	5	5
Amblyomma sculptum	*Amblyomma sculptum*	1	5	5
Amblyomma sculptum	*Amblyomma sculptum*	1	5	5
Amblyomma sculptum	*Amblyomma sculptum*	1	5	5
Amblyomma sculptum	*Amblyomma sculptum*	1	5	5
Amblyomma sculptum	*Amblyomma sculptum*	1	5	5
Amblyomma sculptum	*Amblyomma sculptum*	1	5	5
Amblyomma sculptum	*Amblyomma sculptum*	1	5	5
Amblyomma sculptum	*Amblyomma sculptum*	1	5	5
Amblyomma sculptum	*Amblyomma sculptum*	1	5	5
Amblyomma sculptum	*Amblyomma sculptum*	1	5	5
Amblyomma sculptum	*Amblyomma sculptum*	1	5	5
Amblyomma sculptum	*Amblyomma sculptum*	1	5	5
Amblyomma sculptum	*Amblyomma sculptum*	1	5	5
Amblyomma triste	*Amblyomma triste*	1	6	6
Amblyomma triste	*Amblyomma triste*	1	6	6
Amblyomma triste	*Amblyomma ovale*	0	6	4
Amblyomma triste	*Amblyomma triste*	1	6	6
Amblyomma triste	*Amblyomma triste*	1	6	6
Amblyomma triste	*Amblyomma triste*	1	6	6
Amblyomma triste	*Amblyomma triste*	1	6	6
Amblyomma triste	*Amblyomma triste*	1	6	6
Amblyomma triste	*Amblyomma triste*	1	6	6
Amblyomma triste	*Amblyomma triste*	1	6	6
Amblyomma triste	*Amblyomma triste*	1	6	6
Amblyomma triste	*Amblyomma triste*	1	6	6
Amblyomma triste	*Amblyomma triste*	1	6	6
Amblyomma triste	*Amblyomma triste*	1	6	6
Amblyomma triste	*Amblyomma triste*	1	6	6
Amblyomma triste	*Amblyomma triste*	1	6	6
Amblyomma triste	*Amblyomma dubitatum*	0	6	3
Amblyomma triste	*Amblyomma triste*	1	6	6
Amblyomma triste	*Amblyomma triste*	1	6	6
Amblyomma triste	*Amblyomma triste*	1	6	6
Amblyomma triste	*Amblyomma dubitatum*	0	6	3
Amblyomma aureolatum	*Amblyomma aureolatum*	1	1	1
Amblyomma aureolatum	*Amblyomma aureolatum*	1	1	1
Amblyomma aureolatum	*Amblyomma aureolatum*	1	1	1
Amblyomma aureolatum	*Amblyomma aureolatum*	1	1	1
Amblyomma aureolatum	*Amblyomma aureolatum*	1	1	1
Amblyomma aureolatum	*Amblyomma aureolatum*	1	1	1
Amblyomma aureolatum	*Amblyomma aureolatum*	1	1	1
Amblyomma aureolatum	*Amblyomma aureolatum*	1	1	1
Amblyomma aureolatum	*Amblyomma aureolatum*	1	1	1
Amblyomma aureolatum	*Amblyomma aureolatum*	1	1	1
Amblyomma aureolatum	*Amblyomma aureolatum*	1	1	1
Amblyomma aureolatum	*Amblyomma aureolatum*	1	1	1
Amblyomma cajennense	*Amblyomma cajennense*	1	2	2
Amblyomma cajennense	*Amblyomma cajennense*	1	2	2
Amblyomma cajennense	*Amblyomma cajennense*	1	2	2
Amblyomma cajennense	*Amblyomma cajennense*	1	2	2
Amblyomma cajennense	*Amblyomma cajennense*	1	2	2
Amblyomma cajennense	*Amblyomma cajennense*	1	2	2
Amblyomma cajennense	*Amblyomma cajennense*	1	2	2
Amblyomma cajennense	*Amblyomma cajennense*	1	2	2
Amblyomma cajennense	*Amblyomma cajennense*	1	2	2
Amblyomma cajennense	*Amblyomma cajennense*	1	2	2
Amblyomma cajennense	*Amblyomma cajennense*	1	2	2
Amblyomma cajennense	*Amblyomma cajennense*	1	2	2
Amblyomma cajennense	*Amblyomma cajennense*	1	2	2
Amblyomma cajennense	*Amblyomma cajennense*	1	2	2
Amblyomma cajennense	*Amblyomma cajennense*	1	2	2
Amblyomma cajennense	*Amblyomma triste*	0	2	6

Amblyomma cajennense	*Amblyomma cajennense*	1	2	2
Amblyomma dubitatum	*Amblyomma dubitatum*	1	3	3
Amblyomma dubitatum	*Amblyomma dubitatum*	1	3	3
Amblyomma dubitatum	*Amblyomma dubitatum*	1	3	3
Amblyomma dubitatum	*Amblyomma dubitatum*	1	3	3
Amblyomma dubitatum	*Amblyomma dubitatum*	1	3	3
Amblyomma dubitatum	*Amblyomma dubitatum*	1	3	3
Amblyomma dubitatum	*Amblyomma dubitatum*	1	3	3
Amblyomma dubitatum	*Amblyomma dubitatum*	1	3	3
Amblyomma dubitatum	*Amblyomma dubitatum*	1	3	3
Amblyomma dubitatum	*Amblyomma dubitatum*	1	3	3
Amblyomma dubitatum	*Amblyomma dubitatum*	1	3	3
Amblyomma dubitatum	*Amblyomma dubitatum*	1	3	3
Amblyomma dubitatum	*Amblyomma dubitatum*	1	3	3
Amblyomma dubitatum	*Amblyomma dubitatum*	1	3	3
Amblyomma dubitatum	*Amblyomma dubitatum*	1	3	3
Amblyomma ovale	*Amblyomma ovale*	1	4	4
Amblyomma ovale	*Amblyomma ovale*	1	4	4
Amblyomma ovale	*Amblyomma ovale*	1	4	4
Amblyomma ovale	*Amblyomma ovale*	1	4	4
Amblyomma ovale	*Amblyomma sculptum*	0	4	5
Amblyomma ovale	*Amblyomma triste*	0	4	6
Amblyomma ovale	*Amblyomma ovale*	1	4	4
Amblyomma ovale	*Amblyomma ovale*	1	4	4
Amblyomma ovale	*Amblyomma ovale*	1	4	4
Amblyomma sculptum	*Amblyomma sculptum*	1	5	5
Amblyomma sculptum	*Amblyomma sculptum*	1	5	5
Amblyomma sculptum	*Amblyomma sculptum*	1	5	5
Amblyomma sculptum	*Amblyomma sculptum*	1	5	5
Amblyomma sculptum	*Amblyomma cajennense*	0	5	2
Amblyomma sculptum	*Amblyomma sculptum*	1	5	5
Amblyomma sculptum	*Amblyomma sculptum*	1	5	5
Amblyomma sculptum	*Amblyomma sculptum*	1	5	5
Amblyomma sculptum	*Amblyomma sculptum*	1	5	5
Amblyomma sculptum	*Amblyomma sculptum*	1	5	5
Amblyomma sculptum	*Amblyomma sculptum*	1	5	5
Amblyomma sculptum	*Amblyomma sculptum*	1	5	5
Amblyomma sculptum	*Amblyomma sculptum*	1	5	5
Amblyomma sculptum	*Amblyomma sculptum*	1	5	5
Amblyomma sculptum	*Amblyomma sculptum*	1	5	5
Amblyomma sculptum	*Amblyomma sculptum*	1	5	5
Amblyomma sculptum	*Amblyomma sculptum*	1	5	5
Amblyomma sculptum	*Amblyomma sculptum*	1	5	5
Amblyomma sculptum	*Amblyomma sculptum*	1	5	5
Amblyomma triste	*Amblyomma dubitatum*	0	6	3
Amblyomma triste	*Amblyomma triste*	1	6	6
Amblyomma triste	*Amblyomma triste*	1	6	6
Amblyomma triste	*Amblyomma triste*	1	6	6
Amblyomma triste	*Amblyomma triste*	1	6	6
Amblyomma triste	*Amblyomma triste*	1	6	6
Amblyomma triste	*Amblyomma dubitatum*	0	6	3
Amblyomma triste	*Amblyomma triste*	1	6	6
Amblyomma triste	*Amblyomma triste*	1	6	6
Amblyomma triste	*Amblyomma triste*	1	6	6
Amblyomma triste	*Amblyomma triste*	1	6	6
Amblyomma triste	*Amblyomma triste*	1	6	6
Amblyomma triste	*Amblyomma cajennense*	0	6	2
Amblyomma triste	*Amblyomma triste*	1	6	6
Amblyomma triste	*Amblyomma triste*	1	6	6
Amblyomma triste	*Amblyomma triste*	1	6	6
Amblyomma triste	*Amblyomma triste*	1	6	6
Amblyomma triste	*Amblyomma triste*	1	6	6
Amblyomma triste	*Amblyomma triste*	1	6	6
Amblyomma triste	*Amblyomma triste*	1	6	6
Amblyomma aureolatum	*Amblyomma aureolatum*	1	1	1
Amblyomma aureolatum	*Amblyomma aureolatum*	1	1	1
Amblyomma aureolatum	*Amblyomma aureolatum*	1	1	1

Amblyomma aureolatum	*Amblyomma aureolatum*	1	1	1
Amblyomma aureolatum	*Amblyomma sculptum*	0	1	5
Amblyomma aureolatum	*Amblyomma aureolatum*	1	1	1
Amblyomma aureolatum	*Amblyomma aureolatum*	1	1	1
Amblyomma aureolatum	*Amblyomma aureolatum*	1	1	1
Amblyomma aureolatum	*Amblyomma aureolatum*	1	1	1
Amblyomma aureolatum	*Amblyomma aureolatum*	1	1	1
Amblyomma aureolatum	*Amblyomma aureolatum*	1	1	1
Amblyomma aureolatum	*Amblyomma aureolatum*	1	1	1
Amblyomma aureolatum	*Amblyomma aureolatum*	1	1	1
Amblyomma cajennense	*Amblyomma cajennense*	1	2	2
Amblyomma cajennense	*Amblyomma cajennense*	1	2	2
Amblyomma cajennense	*Amblyomma cajennense*	1	2	2
Amblyomma cajennense	*Amblyomma cajennense*	1	2	2
Amblyomma cajennense	*Amblyomma cajennense*	1	2	2
Amblyomma cajennense	*Amblyomma cajennense*	1	2	2
Amblyomma cajennense	*Amblyomma cajennense*	1	2	2
Amblyomma cajennense	*Amblyomma cajennense*	1	2	2
Amblyomma cajennense	*Amblyomma cajennense*	1	2	2
Amblyomma cajennense	*Amblyomma cajennense*	1	2	2
Amblyomma cajennense	*Amblyomma cajennense*	1	2	2
Amblyomma cajennense	*Amblyomma cajennense*	1	2	2
Amblyomma cajennense	*Amblyomma cajennense*	1	2	2
Amblyomma cajennense	*Amblyomma cajennense*	1	2	2
Amblyomma cajennense	*Amblyomma cajennense*	1	2	2
Amblyomma cajennense	*Amblyomma cajennense*	1	2	2
Amblyomma dubitatum	*Amblyomma dubitatum*	1	3	3
Amblyomma dubitatum	*Amblyomma cajennense*	0	3	2
Amblyomma dubitatum	*Amblyomma dubitatum*	1	3	3
Amblyomma dubitatum	*Amblyomma dubitatum*	1	3	3
Amblyomma dubitatum	*Amblyomma dubitatum*	1	3	3
Amblyomma dubitatum	*Amblyomma dubitatum*	1	3	3
Amblyomma dubitatum	*Amblyomma dubitatum*	1	3	3
Amblyomma dubitatum	*Amblyomma dubitatum*	1	3	3
Amblyomma dubitatum	*Amblyomma dubitatum*	1	3	3
Amblyomma dubitatum	*Amblyomma dubitatum*	1	3	3
Amblyomma dubitatum	*Amblyomma dubitatum*	1	3	3
Amblyomma dubitatum	*Amblyomma dubitatum*	1	3	3
Amblyomma dubitatum	*Amblyomma dubitatum*	1	3	3
Amblyomma dubitatum	*Amblyomma dubitatum*	1	3	3
Amblyomma dubitatum	*Amblyomma dubitatum*	1	3	3
Amblyomma ovale	*Amblyomma ovale*	1	4	4
Amblyomma ovale	*Amblyomma ovale*	1	4	4
Amblyomma ovale	*Amblyomma ovale*	1	4	4
Amblyomma ovale	*Amblyomma ovale*	1	4	4
Amblyomma ovale	*Amblyomma ovale*	1	4	4
Amblyomma ovale	*Amblyomma ovale*	1	4	4
Amblyomma ovale	*Amblyomma ovale*	1	4	4
Amblyomma ovale	*Amblyomma sculptum*	0	4	5
Amblyomma sculptum	*Amblyomma sculptum*	1	5	5
Amblyomma sculptum	*Amblyomma sculptum*	1	5	5
Amblyomma sculptum	*Amblyomma sculptum*	1	5	5
Amblyomma sculptum	*Amblyomma sculptum*	1	5	5
Amblyomma sculptum	*Amblyomma sculptum*	1	5	5
Amblyomma sculptum	*Amblyomma sculptum*	1	5	5
Amblyomma sculptum	*Amblyomma sculptum*	1	5	5
Amblyomma sculptum	*Amblyomma sculptum*	1	5	5
Amblyomma sculptum	*Amblyomma sculptum*	1	5	5
Amblyomma sculptum	*Amblyomma sculptum*	1	5	5
Amblyomma sculptum	*Amblyomma sculptum*	1	5	5
Amblyomma sculptum	*Amblyomma sculptum*	1	5	5
Amblyomma sculptum	*Amblyomma sculptum*	1	5	5
Amblyomma sculptum	*Amblyomma sculptum*	1	5	5
Amblyomma sculptum	*Amblyomma sculptum*	1	5	5
Amblyomma sculptum	*Amblyomma sculptum*	1	5	5
Amblyomma sculptum	*Amblyomma sculptum*	1	5	5
Amblyomma sculptum	*Amblyomma sculptum*	1	5	5

Amblyomma sculptum	*Amblyomma sculptum*	1	5	5
Amblyomma triste	*Amblyomma triste*	1	6	6
Amblyomma triste	*Amblyomma triste*	1	6	6
Amblyomma triste	*Amblyomma triste*	1	6	6
Amblyomma triste	*Amblyomma triste*	1	6	6
Amblyomma triste	*Amblyomma triste*	1	6	6
Amblyomma triste	*Amblyomma triste*	1	6	6
Amblyomma triste	*Amblyomma triste*	1	6	6
Amblyomma triste	*Amblyomma triste*	1	6	6
Amblyomma triste	*Amblyomma triste*	1	6	6
Amblyomma triste	*Amblyomma triste*	1	6	6
Amblyomma triste	*Amblyomma triste*	1	6	6
Amblyomma triste	*Amblyomma triste*	1	6	6
Amblyomma triste	*Amblyomma triste*	1	6	6
Amblyomma triste	*Amblyomma triste*	1	6	6
Amblyomma triste	*Amblyomma triste*	1	6	6
Amblyomma triste	*Amblyomma triste*	1	6	6
Amblyomma triste	*Amblyomma triste*	1	6	6
Amblyomma triste	*Amblyomma triste*	1	6	6
Amblyomma triste	*Amblyomma triste*	1	6	6
Amblyomma triste	*Amblyomma triste*	1	6	6
Amblyomma triste	*Amblyomma triste*	1	6	6
Amblyomma aureolatum	*Amblyomma aureolatum*	1	1	1
Amblyomma aureolatum	*Amblyomma aureolatum*	1	1	1
Amblyomma aureolatum	*Amblyomma aureolatum*	1	1	1
Amblyomma aureolatum	*Amblyomma aureolatum*	1	1	1
Amblyomma aureolatum	*Amblyomma aureolatum*	1	1	1
Amblyomma aureolatum	*Amblyomma aureolatum*	1	1	1
Amblyomma aureolatum	*Amblyomma aureolatum*	1	1	1
Amblyomma aureolatum	*Amblyomma aureolatum*	1	1	1
Amblyomma aureolatum	*Amblyomma aureolatum*	1	1	1
Amblyomma aureolatum	*Amblyomma aureolatum*	1	1	1
Amblyomma aureolatum	*Amblyomma aureolatum*	1	1	1
Amblyomma aureolatum	*Amblyomma aureolatum*	1	1	1
Amblyomma aureolatum	*Amblyomma aureolatum*	1	1	1
Amblyomma cajennense	*Amblyomma cajennense*	1	2	2
Amblyomma cajennense	*Amblyomma cajennense*	1	2	2
Amblyomma cajennense	*Amblyomma cajennense*	1	2	2
Amblyomma cajennense	*Amblyomma cajennense*	1	2	2
Amblyomma cajennense	*Amblyomma cajennense*	1	2	2
Amblyomma cajennense	*Amblyomma cajennense*	1	2	2
Amblyomma cajennense	*Amblyomma cajennense*	1	2	2
Amblyomma cajennense	*Amblyomma cajennense*	1	2	2
Amblyomma cajennense	*Amblyomma cajennense*	1	2	2
Amblyomma cajennense	*Amblyomma cajennense*	1	2	2
Amblyomma cajennense	*Amblyomma cajennense*	1	2	2
Amblyomma cajennense	*Amblyomma cajennense*	1	2	2
Amblyomma cajennense	*Amblyomma cajennense*	1	2	2
Amblyomma cajennense	*Amblyomma cajennense*	1	2	2
Amblyomma cajennense	*Amblyomma cajennense*	1	2	2
Amblyomma cajennense	*Amblyomma cajennense*	1	2	2
Amblyomma cajennense	*Amblyomma cajennense*	1	2	2
Amblyomma dubitatum	*Amblyomma ovale*	0	3	4
Amblyomma dubitatum	*Amblyomma dubitatum*	1	3	3
Amblyomma dubitatum	*Amblyomma dubitatum*	1	3	3
Amblyomma dubitatum	*Amblyomma dubitatum*	1	3	3
Amblyomma dubitatum	*Amblyomma dubitatum*	1	3	3
Amblyomma dubitatum	*Amblyomma dubitatum*	1	3	3
Amblyomma dubitatum	*Amblyomma dubitatum*	1	3	3
Amblyomma dubitatum	*Amblyomma dubitatum*	1	3	3
Amblyomma dubitatum	*Amblyomma dubitatum*	1	3	3
Amblyomma dubitatum	*Amblyomma dubitatum*	1	3	3
Amblyomma dubitatum	*Amblyomma dubitatum*	1	3	3
Amblyomma dubitatum	*Amblyomma dubitatum*	1	3	3
Amblyomma dubitatum	*Amblyomma dubitatum*	1	3	3
Amblyomma dubitatum	*Amblyomma dubitatum*	1	3	3
Amblyomma dubitatum	*Amblyomma dubitatum*	1	3	3

Amblyomma ovale	*Amblyomma ovale*	1	4	4
Amblyomma ovale	*Amblyomma ovale*	1	4	4
Amblyomma ovale	*Amblyomma ovale*	1	4	4
Amblyomma ovale	*Amblyomma cajennense*	0	4	2
Amblyomma ovale	*Amblyomma ovale*	1	4	4
Amblyomma ovale	*Amblyomma aureolatum*	0	4	1
Amblyomma ovale	*Amblyomma ovale*	1	4	4
Amblyomma ovale	*Amblyomma ovale*	1	4	4
Amblyomma sculptum	*Amblyomma sculptum*	1	5	5
Amblyomma sculptum	*Amblyomma sculptum*	1	5	5
Amblyomma sculptum	*Amblyomma sculptum*	1	5	5
Amblyomma sculptum	*Amblyomma sculptum*	1	5	5
Amblyomma sculptum	*Amblyomma sculptum*	1	5	5
Amblyomma sculptum	*Amblyomma sculptum*	1	5	5
Amblyomma sculptum	*Amblyomma sculptum*	1	5	5
Amblyomma sculptum	*Amblyomma sculptum*	1	5	5
Amblyomma sculptum	*Amblyomma sculptum*	1	5	5
Amblyomma sculptum	*Amblyomma sculptum*	1	5	5
Amblyomma sculptum	*Amblyomma sculptum*	1	5	5
Amblyomma sculptum	*Amblyomma sculptum*	1	5	5
Amblyomma sculptum	*Amblyomma sculptum*	1	5	5
Amblyomma sculptum	*Amblyomma sculptum*	1	5	5
Amblyomma sculptum	*Amblyomma aureolatum*	0	5	1
Amblyomma sculptum	*Amblyomma sculptum*	1	5	5
Amblyomma sculptum	*Amblyomma sculptum*	1	5	5
Amblyomma sculptum	*Amblyomma sculptum*	1	5	5
Amblyomma triste	*Amblyomma triste*	1	6	6
Amblyomma triste	*Amblyomma triste*	1	6	6
Amblyomma triste	*Amblyomma triste*	1	6	6
Amblyomma triste	*Amblyomma triste*	1	6	6
Amblyomma triste	*Amblyomma triste*	1	6	6
Amblyomma triste	*Amblyomma triste*	1	6	6
Amblyomma triste	*Amblyomma triste*	1	6	6
Amblyomma triste	*Amblyomma triste*	1	6	6
Amblyomma triste	*Amblyomma triste*	1	6	6
Amblyomma triste	*Amblyomma triste*	1	6	6
Amblyomma triste	*Amblyomma ovale*	0	6	4
Amblyomma triste	*Amblyomma triste*	1	6	6
Amblyomma triste	*Amblyomma triste*	1	6	6
Amblyomma triste	*Amblyomma triste*	1	6	6
Amblyomma triste	*Amblyomma triste*	1	6	6
Amblyomma triste	*Amblyomma triste*	1	6	6
Amblyomma triste	*Amblyomma triste*	1	6	6
Amblyomma triste	*Amblyomma triste*	1	6	6
Amblyomma triste	*Amblyomma triste*	1	6	6
Amblyomma triste	*Amblyomma triste*	1	6	6
Amblyomma triste	*Amblyomma triste*	1	6	6
Amblyomma aureolatum	*Amblyomma cajennense*	0	1	2
Amblyomma aureolatum	*Amblyomma dubitatum*	0	1	3
Amblyomma aureolatum	*Amblyomma aureolatum*	1	1	1
Amblyomma aureolatum	*Amblyomma aureolatum*	1	1	1
Amblyomma aureolatum	*Amblyomma aureolatum*	1	1	1
Amblyomma aureolatum	*Amblyomma aureolatum*	1	1	1
Amblyomma aureolatum	*Amblyomma aureolatum*	1	1	1
Amblyomma aureolatum	*Amblyomma aureolatum*	1	1	1
Amblyomma aureolatum	*Amblyomma aureolatum*	1	1	1
Amblyomma aureolatum	*Amblyomma aureolatum*	1	1	1
Amblyomma aureolatum	*Amblyomma aureolatum*	1	1	1
Amblyomma aureolatum	*Amblyomma triste*	0	1	6
Amblyomma cajennense	*Amblyomma cajennense*	1	2	2
Amblyomma cajennense	*Amblyomma cajennense*	1	2	2
Amblyomma cajennense	*Amblyomma cajennense*	1	2	2
Amblyomma cajennense	*Amblyomma cajennense*	1	2	2
Amblyomma cajennense	*Amblyomma cajennense*	1	2	2
Amblyomma cajennense	*Amblyomma cajennense*	1	2	2
Amblyomma cajennense	*Amblyomma cajennense*	1	2	2
Amblyomma cajennense	*Amblyomma cajennense*	1	2	2

Amblyomma cajennense	*Amblyomma cajennense*	1	2	2
Amblyomma cajennense	*Amblyomma cajennense*	1	2	2
Amblyomma cajennense	*Amblyomma cajennense*	1	2	2
Amblyomma cajennense	*Amblyomma aureolatum*	0	2	1
Amblyomma cajennense	*Amblyomma cajennense*	1	2	2
Amblyomma cajennense	*Amblyomma cajennense*	1	2	2
Amblyomma cajennense	*Amblyomma cajennense*	1	2	2
Amblyomma cajennense	*Amblyomma cajennense*	1	2	2
Amblyomma dubitatum	*Amblyomma dubitatum*	1	3	3
Amblyomma dubitatum	*Amblyomma dubitatum*	1	3	3
Amblyomma dubitatum	*Amblyomma dubitatum*	1	3	3
Amblyomma dubitatum	*Amblyomma dubitatum*	1	3	3
Amblyomma dubitatum	*Amblyomma dubitatum*	1	3	3
Amblyomma dubitatum	*Amblyomma dubitatum*	1	3	3
Amblyomma dubitatum	*Amblyomma dubitatum*	1	3	3
Amblyomma dubitatum	*Amblyomma dubitatum*	1	3	3
Amblyomma dubitatum	*Amblyomma dubitatum*	1	3	3
Amblyomma dubitatum	*Amblyomma dubitatum*	1	3	3
Amblyomma dubitatum	*Amblyomma dubitatum*	1	3	3
Amblyomma ovale	*Amblyomma ovale*	1	4	4
Amblyomma ovale	*Amblyomma ovale*	1	4	4
Amblyomma ovale	*Amblyomma ovale*	1	4	4
Amblyomma ovale	*Amblyomma ovale*	1	4	4
Amblyomma ovale	*Amblyomma ovale*	1	4	4
Amblyomma ovale	*Amblyomma ovale*	1	4	4
Amblyomma ovale	*Amblyomma triste*	0	4	6
Amblyomma ovale	*Amblyomma ovale*	1	4	4
Amblyomma ovale	*Amblyomma ovale*	1	4	4
Amblyomma ovale	*Amblyomma aureolatum*	0	4	1
Amblyomma ovale	*Amblyomma ovale*	1	4	4
Amblyomma ovale	*Amblyomma cajennense*	0	4	2
Amblyomma sculptum	*Amblyomma sculptum*	1	5	5
Amblyomma sculptum	*Amblyomma sculptum*	1	5	5
Amblyomma sculptum	*Amblyomma dubitatum*	0	5	3
Amblyomma sculptum	*Amblyomma sculptum*	1	5	5
Amblyomma sculptum	*Amblyomma sculptum*	1	5	5
Amblyomma sculptum	*Amblyomma dubitatum*	0	5	3
Amblyomma sculptum	*Amblyomma sculptum*	1	5	5
Amblyomma sculptum	*Amblyomma sculptum*	1	5	5
Amblyomma sculptum	*Amblyomma sculptum*	1	5	5
Amblyomma sculptum	*Amblyomma sculptum*	1	5	5
Amblyomma sculptum	*Amblyomma sculptum*	1	5	5
Amblyomma sculptum	*Amblyomma sculptum*	1	5	5
Amblyomma sculptum	*Amblyomma sculptum*	1	5	5
Amblyomma sculptum	*Amblyomma sculptum*	1	5	5
Amblyomma sculptum	*Amblyomma sculptum*	1	5	5
Amblyomma sculptum	*Amblyomma sculptum*	1	5	5
Amblyomma triste	*Amblyomma triste*	1	6	6
Amblyomma triste	*Amblyomma triste*	1	6	6
Amblyomma triste	*Amblyomma triste*	1	6	6
Amblyomma triste	*Amblyomma triste*	1	6	6
Amblyomma triste	*Amblyomma triste*	1	6	6
Amblyomma triste	*Amblyomma triste*	1	6	6
Amblyomma triste	*Amblyomma triste*	1	6	6
Amblyomma triste	*Amblyomma triste*	1	6	6
Amblyomma triste	*Amblyomma triste*	1	6	6
Amblyomma triste	*Amblyomma triste*	1	6	6
Amblyomma triste	*Amblyomma triste*	1	6	6
Amblyomma triste	*Amblyomma triste*	1	6	6
Amblyomma triste	*Amblyomma triste*	1	6	6
Amblyomma triste	*Amblyomma triste*	1	6	6
Amblyomma triste	*Amblyomma triste*	1	6	6
Amblyomma triste	*Amblyomma triste*	1	6	6
Amblyomma aureolatum	*Amblyomma cajennense*	0	1	2
Amblyomma aureolatum	*Amblyomma aureolatum*	1	1	1
Amblyomma aureolatum	*Amblyomma aureolatum*	1	1	1
Amblyomma aureolatum	*Amblyomma aureolatum*	1	1	1

Amblyomma aureolatum	*Amblyomma triste*	0	1	6
Amblyomma aureolatum	*Amblyomma aureolatum*	1	1	1
Amblyomma aureolatum	*Amblyomma dubitatum*	0	1	3
Amblyomma aureolatum	*Amblyomma aureolatum*	1	1	1
Amblyomma aureolatum	*Amblyomma aureolatum*	1	1	1
Amblyomma aureolatum	*Amblyomma aureolatum*	1	1	1
Amblyomma aureolatum	*Amblyomma aureolatum*	1	1	1
Amblyomma aureolatum	*Amblyomma aureolatum*	1	1	1
Amblyomma aureolatum	*Amblyomma aureolatum*	1	1	1
Amblyomma cajennense	*Amblyomma ovale*	0	2	4
Amblyomma cajennense	*Amblyomma cajennense*	1	2	2
Amblyomma cajennense	*Amblyomma cajennense*	1	2	2
Amblyomma cajennense	*Amblyomma cajennense*	1	2	2
Amblyomma cajennense	*Amblyomma cajennense*	1	2	2
Amblyomma cajennense	*Amblyomma cajennense*	1	2	2
Amblyomma cajennense	*Amblyomma cajennense*	1	2	2
Amblyomma cajennense	*Amblyomma cajennense*	1	2	2
Amblyomma cajennense	*Amblyomma cajennense*	1	2	2
Amblyomma cajennense	*Amblyomma cajennense*	1	2	2
Amblyomma cajennense	*Amblyomma cajennense*	1	2	2
Amblyomma cajennense	*Amblyomma cajennense*	1	2	2
Amblyomma cajennense	*Amblyomma cajennense*	1	2	2
Amblyomma cajennense	*Amblyomma cajennense*	1	2	2
Amblyomma cajennense	*Amblyomma cajennense*	1	2	2
Amblyomma cajennense	*Amblyomma cajennense*	1	2	2
Amblyomma dubitatum	*Amblyomma cajennense*	0	3	2
Amblyomma dubitatum	*Amblyomma dubitatum*	1	3	3
Amblyomma dubitatum	*Amblyomma cajennense*	0	3	2
Amblyomma dubitatum	*Amblyomma ovale*	0	3	4
Amblyomma dubitatum	*Amblyomma dubitatum*	1	3	3
Amblyomma dubitatum	*Amblyomma dubitatum*	1	3	3
Amblyomma dubitatum	*Amblyomma sculptum*	0	3	5
Amblyomma dubitatum	*Amblyomma dubitatum*	1	3	3
Amblyomma dubitatum	*Amblyomma dubitatum*	1	3	3
Amblyomma dubitatum	*Amblyomma dubitatum*	1	3	3
Amblyomma dubitatum	*Amblyomma dubitatum*	1	3	3
Amblyomma dubitatum	*Amblyomma dubitatum*	1	3	3
Amblyomma ovale	*Amblyomma ovale*	1	4	4
Amblyomma ovale	*Amblyomma ovale*	1	4	4
Amblyomma ovale	*Amblyomma ovale*	1	4	4
Amblyomma ovale	*Amblyomma ovale*	1	4	4
Amblyomma ovale	*Amblyomma ovale*	1	4	4
Amblyomma ovale	*Amblyomma ovale*	1	4	4
Amblyomma ovale	*Amblyomma aureolatum*	0	4	1
Amblyomma ovale	*Amblyomma ovale*	1	4	4
Amblyomma ovale	*Amblyomma ovale*	1	4	4
Amblyomma ovale	*Amblyomma ovale*	1	4	4
Amblyomma ovale	*Amblyomma ovale*	1	4	4
Amblyomma ovale	*Amblyomma ovale*	1	4	4
Amblyomma sculptum	*Amblyomma sculptum*	1	5	5
Amblyomma sculptum	*Amblyomma sculptum*	1	5	5
Amblyomma sculptum	*Amblyomma sculptum*	1	5	5
Amblyomma sculptum	*Amblyomma sculptum*	1	5	5
Amblyomma sculptum	*Amblyomma sculptum*	1	5	5
Amblyomma sculptum	*Amblyomma sculptum*	1	5	5
Amblyomma sculptum	*Amblyomma sculptum*	1	5	5
Amblyomma sculptum	*Amblyomma sculptum*	1	5	5
Amblyomma sculptum	*Amblyomma sculptum*	1	5	5
Amblyomma sculptum	*Amblyomma sculptum*	1	5	5
Amblyomma sculptum	*Amblyomma sculptum*	1	5	5
Amblyomma sculptum	*Amblyomma sculptum*	1	5	5
Amblyomma sculptum	*Amblyomma sculptum*	1	5	5
Amblyomma sculptum	*Amblyomma sculptum*	1	5	5
Amblyomma sculptum	*Amblyomma sculptum*	1	5	5
Amblyomma sculptum	*Amblyomma sculptum*	1	5	5
Amblyomma sculptum	*Amblyomma sculptum*	1	5	5
Amblyomma triste	*Amblyomma triste*	1	6	6

Amblyomma triste	*Amblyomma triste*	1	6	6
Amblyomma triste	*Amblyomma triste*	1	6	6
Amblyomma triste	*Amblyomma triste*	1	6	6
Amblyomma triste	*Amblyomma triste*	1	6	6
Amblyomma triste	*Amblyomma triste*	1	6	6
Amblyomma triste	*Amblyomma triste*	1	6	6
Amblyomma triste	*Amblyomma triste*	1	6	6
Amblyomma triste	*Amblyomma dubitatum*	0	6	3
Amblyomma triste	*Amblyomma triste*	1	6	6
Amblyomma triste	*Amblyomma triste*	1	6	6
Amblyomma triste	*Amblyomma triste*	1	6	6
Amblyomma triste	*Amblyomma triste*	1	6	6
Amblyomma triste	*Amblyomma triste*	1	6	6
Amblyomma triste	*Amblyomma triste*	1	6	6
Amblyomma triste	*Amblyomma aureolatum*	0	6	1
Amblyomma aureolatum	*Amblyomma aureolatum*	1	1	1
Amblyomma aureolatum	*Amblyomma aureolatum*	1	1	1
Amblyomma aureolatum	*Amblyomma aureolatum*	1	1	1
Amblyomma aureolatum	*Amblyomma aureolatum*	1	1	1
Amblyomma aureolatum	*Amblyomma aureolatum*	1	1	1
Amblyomma aureolatum	*Amblyomma aureolatum*	1	1	1
Amblyomma aureolatum	*Amblyomma aureolatum*	1	1	1
Amblyomma aureolatum	*Amblyomma aureolatum*	1	1	1
Amblyomma aureolatum	*Amblyomma aureolatum*	1	1	1
Amblyomma aureolatum	*Amblyomma aureolatum*	1	1	1
Amblyomma aureolatum	*Amblyomma aureolatum*	1	1	1
Amblyomma aureolatum	*Amblyomma aureolatum*	1	1	1
Amblyomma cajennense	*Amblyomma cajennense*	1	2	2
Amblyomma cajennense	*Amblyomma cajennense*	1	2	2
Amblyomma cajennense	*Amblyomma cajennense*	1	2	2
Amblyomma cajennense	*Amblyomma cajennense*	1	2	2
Amblyomma cajennense	*Amblyomma cajennense*	1	2	2
Amblyomma cajennense	*Amblyomma cajennense*	1	2	2
Amblyomma cajennense	*Amblyomma cajennense*	1	2	2
Amblyomma cajennense	*Amblyomma cajennense*	1	2	2
Amblyomma cajennense	*Amblyomma cajennense*	1	2	2
Amblyomma cajennense	*Amblyomma cajennense*	1	2	2
Amblyomma cajennense	*Amblyomma cajennense*	1	2	2
Amblyomma cajennense	*Amblyomma cajennense*	1	2	2
Amblyomma cajennense	*Amblyomma triste*	0	2	6
Amblyomma cajennense	*Amblyomma cajennense*	1	2	2
Amblyomma cajennense	*Amblyomma cajennense*	1	2	2
Amblyomma cajennense	*Amblyomma cajennense*	1	2	2
Amblyomma cajennense	*Amblyomma cajennense*	1	2	2
Amblyomma dubitatum	*Amblyomma ovale*	0	3	4
Amblyomma dubitatum	*Amblyomma dubitatum*	1	3	3
Amblyomma dubitatum	*Amblyomma aureolatum*	0	3	1
Amblyomma dubitatum	*Amblyomma dubitatum*	1	3	3
Amblyomma dubitatum	*Amblyomma aureolatum*	0	3	1
Amblyomma dubitatum	*Amblyomma dubitatum*	1	3	3
Amblyomma dubitatum	*Amblyomma dubitatum*	1	3	3
Amblyomma dubitatum	*Amblyomma aureolatum*	0	3	1
Amblyomma dubitatum	*Amblyomma sculptum*	0	3	5
Amblyomma dubitatum	*Amblyomma dubitatum*	1	3	3
Amblyomma dubitatum	*Amblyomma dubitatum*	1	3	3
Amblyomma ovale	*Amblyomma ovale*	1	4	4
Amblyomma ovale	*Amblyomma ovale*	1	4	4
Amblyomma ovale	*Amblyomma ovale*	1	4	4
Amblyomma ovale	*Amblyomma ovale*	1	4	4
Amblyomma ovale	*Amblyomma ovale*	1	4	4
Amblyomma ovale	*Amblyomma ovale*	1	4	4
Amblyomma ovale	*Amblyomma dubitatum*	0	4	3
Amblyomma ovale	*Amblyomma ovale*	1	4	4
Amblyomma ovale	*Amblyomma ovale*	1	4	4
Amblyomma ovale	*Amblyomma sculptum*	0	4	5
Amblyomma ovale	*Amblyomma ovale*	1	4	4
Amblyomma ovale	*Amblyomma ovale*	1	4	4

Amblyomma sculptum	*Amblyomma sculptum*	1	5	5
Amblyomma sculptum	*Amblyomma sculptum*	1	5	5
Amblyomma sculptum	*Amblyomma sculptum*	1	5	5
Amblyomma sculptum	*Amblyomma sculptum*	1	5	5
Amblyomma sculptum	*Amblyomma sculptum*	1	5	5
Amblyomma sculptum	*Amblyomma sculptum*	1	5	5
Amblyomma sculptum	*Amblyomma sculptum*	1	5	5
Amblyomma sculptum	*Amblyomma sculptum*	1	5	5
Amblyomma sculptum	*Amblyomma sculptum*	1	5	5
Amblyomma sculptum	*Amblyomma sculptum*	1	5	5
Amblyomma sculptum	*Amblyomma sculptum*	1	5	5
Amblyomma sculptum	*Amblyomma sculptum*	1	5	5
Amblyomma sculptum	*Amblyomma sculptum*	1	5	5
Amblyomma sculptum	*Amblyomma sculptum*	1	5	5
Amblyomma sculptum	*Amblyomma sculptum*	1	5	5
Amblyomma sculptum	*Amblyomma sculptum*	1	5	5
Amblyomma triste	*Amblyomma triste*	1	6	6
Amblyomma triste	*Amblyomma triste*	1	6	6
Amblyomma triste	*Amblyomma triste*	1	6	6
Amblyomma triste	*Amblyomma triste*	1	6	6
Amblyomma triste	*Amblyomma triste*	1	6	6
Amblyomma triste	*Amblyomma triste*	1	6	6
Amblyomma triste	*Amblyomma triste*	1	6	6
Amblyomma triste	*Amblyomma triste*	1	6	6
Amblyomma triste	*Amblyomma triste*	1	6	6
Amblyomma triste	*Amblyomma triste*	1	6	6
Amblyomma triste	*Amblyomma triste*	1	6	6
Amblyomma triste	*Amblyomma triste*	1	6	6
Amblyomma triste	*Amblyomma triste*	1	6	6
Amblyomma triste	*Amblyomma triste*	1	6	6
Amblyomma triste	*Amblyomma triste*	1	6	6
Amblyomma triste	*Amblyomma triste*	1	6	6
Amblyomma aureolatum	*Amblyomma aureolatum*	1	1	1
Amblyomma aureolatum	*Amblyomma aureolatum*	1	1	1
Amblyomma aureolatum	*Amblyomma aureolatum*	1	1	1
Amblyomma aureolatum	*Amblyomma aureolatum*	1	1	1
Amblyomma aureolatum	*Amblyomma ovale*	0	1	4
Amblyomma aureolatum	*Amblyomma aureolatum*	1	1	1
Amblyomma aureolatum	*Amblyomma aureolatum*	1	1	1
Amblyomma aureolatum	*Amblyomma aureolatum*	1	1	1
Amblyomma aureolatum	*Amblyomma aureolatum*	1	1	1
Amblyomma aureolatum	*Amblyomma aureolatum*	1	1	1
Amblyomma aureolatum	*Amblyomma aureolatum*	1	1	1
Amblyomma aureolatum	*Amblyomma aureolatum*	1	1	1
Amblyomma aureolatum	*Amblyomma aureolatum*	1	1	1
Amblyomma cajennense	*Amblyomma cajennense*	1	2	2
Amblyomma cajennense	*Amblyomma cajennense*	1	2	2
Amblyomma cajennense	*Amblyomma cajennense*	1	2	2
Amblyomma cajennense	*Amblyomma cajennense*	1	2	2
Amblyomma cajennense	*Amblyomma cajennense*	1	2	2
Amblyomma cajennense	*Amblyomma cajennense*	1	2	2
Amblyomma cajennense	*Amblyomma cajennense*	1	2	2
Amblyomma cajennense	*Amblyomma cajennense*	1	2	2
Amblyomma cajennense	*Amblyomma cajennense*	1	2	2
Amblyomma cajennense	*Amblyomma cajennense*	1	2	2
Amblyomma cajennense	*Amblyomma cajennense*	1	2	2
Amblyomma cajennense	*Amblyomma cajennense*	1	2	2
Amblyomma cajennense	*Amblyomma cajennense*	1	2	2
Amblyomma cajennense	*Amblyomma cajennense*	1	2	2
Amblyomma cajennense	*Amblyomma cajennense*	1	2	2
Amblyomma cajennense	*Amblyomma cajennense*	1	2	2
Amblyomma cajennense	*Amblyomma cajennense*	1	2	2
Amblyomma dubitatum	*Amblyomma dubitatum*	1	3	3
Amblyomma dubitatum	*Amblyomma dubitatum*	1	3	3
Amblyomma dubitatum	*Amblyomma dubitatum*	1	3	3
Amblyomma dubitatum	*Amblyomma dubitatum*	1	3	3
Amblyomma dubitatum	*Amblyomma dubitatum*	1	3	3

Amblyomma dubitatum	*Amblyomma dubitatum*	1	3	3
Amblyomma dubitatum	*Amblyomma sculptum*	0	3	5
Amblyomma dubitatum	*Amblyomma dubitatum*	1	3	3
Amblyomma dubitatum	*Amblyomma aureolatum*	0	3	1
Amblyomma dubitatum	*Amblyomma dubitatum*	1	3	3
Amblyomma dubitatum	*Amblyomma sculptum*	0	3	5
Amblyomma ovale	*Amblyomma ovale*	1	4	4
Amblyomma ovale	*Amblyomma ovale*	1	4	4
Amblyomma ovale	*Amblyomma ovale*	1	4	4
Amblyomma ovale	*Amblyomma ovale*	1	4	4
Amblyomma ovale	*Amblyomma ovale*	1	4	4
Amblyomma ovale	*Amblyomma ovale*	1	4	4
Amblyomma ovale	*Amblyomma triste*	0	4	6
Amblyomma ovale	*Amblyomma aureolatum*	0	4	1
Amblyomma ovale	*Amblyomma cajennense*	0	4	2
Amblyomma ovale	*Amblyomma ovale*	1	4	4
Amblyomma ovale	*Amblyomma ovale*	1	4	4
Amblyomma ovale	*Amblyomma aureolatum*	0	4	1
Amblyomma sculptum	*Amblyomma sculptum*	1	5	5
Amblyomma sculptum	*Amblyomma sculptum*	1	5	5
Amblyomma sculptum	*Amblyomma sculptum*	1	5	5
Amblyomma sculptum	*Amblyomma sculptum*	1	5	5
Amblyomma sculptum	*Amblyomma cajennense*	0	5	2
Amblyomma sculptum	*Amblyomma sculptum*	1	5	5
Amblyomma sculptum	*Amblyomma sculptum*	1	5	5
Amblyomma sculptum	*Amblyomma sculptum*	1	5	5
Amblyomma sculptum	*Amblyomma sculptum*	1	5	5
Amblyomma sculptum	*Amblyomma sculptum*	1	5	5
Amblyomma sculptum	*Amblyomma sculptum*	1	5	5
Amblyomma sculptum	*Amblyomma sculptum*	1	5	5
Amblyomma sculptum	*Amblyomma sculptum*	1	5	5
Amblyomma sculptum	*Amblyomma sculptum*	1	5	5
Amblyomma sculptum	*Amblyomma sculptum*	1	5	5
Amblyomma sculptum	*Amblyomma sculptum*	1	5	5
Amblyomma triste	*Amblyomma triste*	1	6	6
Amblyomma triste	*Amblyomma triste*	1	6	6
Amblyomma triste	*Amblyomma triste*	1	6	6
Amblyomma triste	*Amblyomma triste*	1	6	6
Amblyomma triste	*Amblyomma triste*	1	6	6
Amblyomma triste	*Amblyomma triste*	1	6	6
Amblyomma triste	*Amblyomma triste*	1	6	6
Amblyomma triste	*Amblyomma triste*	1	6	6
Amblyomma triste	*Amblyomma triste*	1	6	6
Amblyomma triste	*Amblyomma triste*	1	6	6
Amblyomma triste	*Amblyomma triste*	1	6	6
Amblyomma triste	*Amblyomma triste*	1	6	6
Amblyomma triste	*Amblyomma triste*	1	6	6
Amblyomma triste	*Amblyomma triste*	1	6	6
Amblyomma triste	*Amblyomma triste*	1	6	6
Amblyomma aureolatum	*Amblyomma cajennense*	0	1	2
Amblyomma aureolatum	*Amblyomma aureolatum*	1	1	1
Amblyomma aureolatum	*Amblyomma aureolatum*	1	1	1
Amblyomma aureolatum	*Amblyomma aureolatum*	1	1	1
Amblyomma aureolatum	*Amblyomma aureolatum*	1	1	1
Amblyomma aureolatum	*Amblyomma aureolatum*	1	1	1
Amblyomma aureolatum	*Amblyomma cajennense*	0	1	2
Amblyomma aureolatum	*Amblyomma aureolatum*	1	1	1
Amblyomma aureolatum	*Amblyomma aureolatum*	1	1	1
Amblyomma aureolatum	*Amblyomma aureolatum*	1	1	1
Amblyomma aureolatum	*Amblyomma aureolatum*	1	1	1
Amblyomma aureolatum	*Amblyomma aureolatum*	1	1	1
Amblyomma aureolatum	*Amblyomma aureolatum*	1	1	1
Amblyomma cajennense	*Amblyomma cajennense*	1	2	2
Amblyomma cajennense	*Amblyomma cajennense*	1	2	2
Amblyomma cajennense	*Amblyomma cajennense*	1	2	2
Amblyomma cajennense	*Amblyomma cajennense*	1	2	2
Amblyomma cajennense	*Amblyomma cajennense*	1	2	2

Amblyomma cajennense	*Amblyomma cajennense*	1	2	2
Amblyomma cajennense	*Amblyomma cajennense*	1	2	2
Amblyomma cajennense	*Amblyomma cajennense*	1	2	2
Amblyomma cajennense	*Amblyomma cajennense*	1	2	2
Amblyomma cajennense	*Amblyomma cajennense*	1	2	2
Amblyomma cajennense	*Amblyomma cajennense*	1	2	2
Amblyomma cajennense	*Amblyomma cajennense*	1	2	2
Amblyomma cajennense	*Amblyomma cajennense*	1	2	2
Amblyomma cajennense	*Amblyomma cajennense*	1	2	2
Amblyomma cajennense	*Amblyomma cajennense*	1	2	2
Amblyomma cajennense	*Amblyomma cajennense*	1	2	2
Amblyomma cajennense	*Amblyomma cajennense*	1	2	2
Amblyomma dubitatum	*Amblyomma dubitatum*	1	3	3
Amblyomma dubitatum	*Amblyomma dubitatum*	1	3	3
Amblyomma dubitatum	*Amblyomma aureolatum*	0	3	1
Amblyomma dubitatum	*Amblyomma aureolatum*	0	3	1
Amblyomma dubitatum	*Amblyomma dubitatum*	1	3	3
Amblyomma dubitatum	*Amblyomma dubitatum*	1	3	3
Amblyomma dubitatum	*Amblyomma dubitatum*	1	3	3
Amblyomma dubitatum	*Amblyomma dubitatum*	1	3	3
Amblyomma dubitatum	*Amblyomma dubitatum*	1	3	3
Amblyomma dubitatum	*Amblyomma dubitatum*	1	3	3
Amblyomma dubitatum	*Amblyomma dubitatum*	1	3	3
Amblyomma ovale	*Amblyomma ovale*	1	4	4
Amblyomma ovale	*Amblyomma ovale*	1	4	4
Amblyomma ovale	*Amblyomma ovale*	1	4	4
Amblyomma ovale	*Amblyomma ovale*	1	4	4
Amblyomma ovale	*Amblyomma ovale*	1	4	4
Amblyomma ovale	*Amblyomma ovale*	1	4	4
Amblyomma ovale	*Amblyomma ovale*	1	4	4
Amblyomma ovale	*Amblyomma ovale*	1	4	4
Amblyomma ovale	*Amblyomma ovale*	1	4	4
Amblyomma ovale	*Amblyomma ovale*	1	4	4
Amblyomma ovale	*Amblyomma ovale*	1	4	4
Amblyomma ovale	*Amblyomma ovale*	1	4	4
Amblyomma ovale	*Amblyomma ovale*	1	4	4
Amblyomma sculptum	*Amblyomma sculptum*	1	5	5
Amblyomma sculptum	*Amblyomma sculptum*	1	5	5
Amblyomma sculptum	*Amblyomma sculptum*	1	5	5
Amblyomma sculptum	*Amblyomma sculptum*	1	5	5
Amblyomma sculptum	*Amblyomma sculptum*	1	5	5
Amblyomma sculptum	*Amblyomma sculptum*	1	5	5
Amblyomma sculptum	*Amblyomma cajennense*	0	5	2
Amblyomma sculptum	*Amblyomma sculptum*	1	5	5
Amblyomma sculptum	*Amblyomma sculptum*	1	5	5
Amblyomma sculptum	*Amblyomma sculptum*	1	5	5
Amblyomma sculptum	*Amblyomma sculptum*	1	5	5
Amblyomma sculptum	*Amblyomma sculptum*	1	5	5
Amblyomma sculptum	*Amblyomma sculptum*	1	5	5
Amblyomma sculptum	*Amblyomma sculptum*	1	5	5
Amblyomma sculptum	*Amblyomma sculptum*	1	5	5
Amblyomma sculptum	*Amblyomma cajennense*	0	5	2
Amblyomma triste	*Amblyomma triste*	1	6	6
Amblyomma triste	*Amblyomma cajennense*	0	6	2
Amblyomma triste	*Amblyomma triste*	1	6	6
Amblyomma triste	*Amblyomma triste*	1	6	6
Amblyomma triste	*Amblyomma triste*	1	6	6
Amblyomma triste	*Amblyomma triste*	1	6	6
Amblyomma triste	*Amblyomma triste*	1	6	6
Amblyomma triste	*Amblyomma triste*	1	6	6
Amblyomma triste	*Amblyomma triste*	1	6	6
Amblyomma triste	*Amblyomma triste*	1	6	6
Amblyomma triste	*Amblyomma triste*	1	6	6
Amblyomma triste	*Amblyomma dubitatum*	0	6	3
Amblyomma triste	*Amblyomma triste*	1	6	6
Amblyomma triste	*Amblyomma triste*	1	6	6
Amblyomma triste	*Amblyomma cajennense*	0	6	2

Amblyomma triste	*Amblyomma triste*	1	6	6
Amblyomma aureolatum	*Amblyomma aureolatum*	1	1	1
Amblyomma aureolatum	*Amblyomma aureolatum*	1	1	1
Amblyomma aureolatum	*Amblyomma aureolatum*	1	1	1
Amblyomma aureolatum	*Amblyomma aureolatum*	1	1	1
Amblyomma aureolatum	*Amblyomma aureolatum*	1	1	1
Amblyomma aureolatum	*Amblyomma aureolatum*	1	1	1
Amblyomma aureolatum	*Amblyomma aureolatum*	1	1	1
Amblyomma aureolatum	*Amblyomma aureolatum*	1	1	1
Amblyomma aureolatum	*Amblyomma aureolatum*	1	1	1
Amblyomma aureolatum	*Amblyomma aureolatum*	1	1	1
Amblyomma aureolatum	*Amblyomma aureolatum*	1	1	1
Amblyomma aureolatum	*Amblyomma aureolatum*	1	1	1
Amblyomma cajennense	*Amblyomma cajennense*	1	2	2
Amblyomma cajennense	*Amblyomma cajennense*	1	2	2
Amblyomma cajennense	*Amblyomma cajennense*	1	2	2
Amblyomma cajennense	*Amblyomma cajennense*	1	2	2
Amblyomma cajennense	*Amblyomma cajennense*	1	2	2
Amblyomma cajennense	*Amblyomma cajennense*	1	2	2
Amblyomma cajennense	*Amblyomma cajennense*	1	2	2
Amblyomma cajennense	*Amblyomma cajennense*	1	2	2
Amblyomma cajennense	*Amblyomma cajennense*	1	2	2
Amblyomma cajennense	*Amblyomma cajennense*	1	2	2
Amblyomma cajennense	*Amblyomma triste*	0	2	6
Amblyomma cajennense	*Amblyomma cajennense*	1	2	2
Amblyomma cajennense	*Amblyomma cajennense*	1	2	2
Amblyomma cajennense	*Amblyomma cajennense*	1	2	2
Amblyomma cajennense	*Amblyomma cajennense*	1	2	2
Amblyomma cajennense	*Amblyomma cajennense*	1	2	2
Amblyomma dubitatum	*Amblyomma dubitatum*	1	3	3
Amblyomma dubitatum	*Amblyomma dubitatum*	1	3	3
Amblyomma dubitatum	*Amblyomma dubitatum*	1	3	3
Amblyomma dubitatum	*Amblyomma dubitatum*	1	3	3
Amblyomma dubitatum	*Amblyomma dubitatum*	1	3	3
Amblyomma dubitatum	*Amblyomma dubitatum*	1	3	3
Amblyomma dubitatum	*Amblyomma dubitatum*	1	3	3
Amblyomma dubitatum	*Amblyomma dubitatum*	1	3	3
Amblyomma dubitatum	*Amblyomma dubitatum*	1	3	3
Amblyomma dubitatum	*Amblyomma dubitatum*	1	3	3
Amblyomma ovale	*Amblyomma ovale*	1	4	4
Amblyomma ovale	*Amblyomma ovale*	1	4	4
Amblyomma ovale	*Amblyomma aureolatum*	0	4	1
Amblyomma ovale	*Amblyomma ovale*	1	4	4
Amblyomma ovale	*Amblyomma ovale*	1	4	4
Amblyomma ovale	*Amblyomma ovale*	1	4	4
Amblyomma ovale	*Amblyomma ovale*	1	4	4
Amblyomma ovale	*Amblyomma ovale*	1	4	4
Amblyomma ovale	*Amblyomma cajennense*	0	4	2
Amblyomma ovale	*Amblyomma ovale*	1	4	4
Amblyomma ovale	*Amblyomma dubitatum*	0	4	3
Amblyomma ovale	*Amblyomma dubitatum*	0	4	3
Amblyomma sculptum	*Amblyomma sculptum*	1	5	5
Amblyomma sculptum	*Amblyomma sculptum*	1	5	5
Amblyomma sculptum	*Amblyomma ovale*	0	5	4
Amblyomma sculptum	*Amblyomma dubitatum*	0	5	3
Amblyomma sculptum	*Amblyomma dubitatum*	0	5	3
Amblyomma sculptum	*Amblyomma sculptum*	1	5	5
Amblyomma sculptum	*Amblyomma cajennense*	0	5	2
Amblyomma sculptum	*Amblyomma sculptum*	1	5	5
Amblyomma sculptum	*Amblyomma sculptum*	1	5	5
Amblyomma sculptum	*Amblyomma sculptum*	1	5	5
Amblyomma sculptum	*Amblyomma sculptum*	1	5	5
Amblyomma sculptum	*Amblyomma sculptum*	1	5	5
Amblyomma sculptum	*Amblyomma sculptum*	1	5	5
Amblyomma sculptum	*Amblyomma sculptum*	1	5	5
Amblyomma sculptum	*Amblyomma sculptum*	1	5	5
Amblyomma triste	*Amblyomma triste*	1	6	6

Amblyomma triste	*Amblyomma triste*	1	6	6
Amblyomma triste	*Amblyomma triste*	1	6	6
Amblyomma triste	*Amblyomma triste*	1	6	6
Amblyomma triste	*Amblyomma triste*	1	6	6
Amblyomma triste	*Amblyomma triste*	1	6	6
Amblyomma triste	*Amblyomma triste*	1	6	6
Amblyomma triste	*Amblyomma triste*	1	6	6
Amblyomma triste	*Amblyomma triste*	1	6	6
Amblyomma triste	*Amblyomma triste*	1	6	6
Amblyomma triste	*Amblyomma triste*	1	6	6
Amblyomma triste	*Amblyomma triste*	1	6	6
Amblyomma triste	*Amblyomma triste*	1	6	6
Amblyomma triste	*Amblyomma ovale*	0	6	4
Amblyomma triste	*Amblyomma aureolatum*	0	6	1
Amblyomma triste	*Amblyomma ovale*	0	6	4
Amblyomma aureolatum	*Amblyomma aureolatum*	1	1	1
Amblyomma aureolatum	*Amblyomma aureolatum*	1	1	1
Amblyomma aureolatum	*Amblyomma aureolatum*	1	1	1
Amblyomma aureolatum	*Amblyomma ovale*	0	1	4
Amblyomma aureolatum	*Amblyomma aureolatum*	1	1	1
Amblyomma aureolatum	*Amblyomma aureolatum*	1	1	1
Amblyomma aureolatum	*Amblyomma aureolatum*	1	1	1
Amblyomma aureolatum	*Amblyomma aureolatum*	1	1	1
Amblyomma aureolatum	*Amblyomma aureolatum*	1	1	1
Amblyomma aureolatum	*Amblyomma aureolatum*	1	1	1
Amblyomma aureolatum	*Amblyomma aureolatum*	1	1	1
Amblyomma aureolatum	*Amblyomma aureolatum*	1	1	1
Amblyomma cajennense	*Amblyomma cajennense*	1	2	2
Amblyomma cajennense	*Amblyomma cajennense*	1	2	2
Amblyomma cajennense	*Amblyomma cajennense*	1	2	2
Amblyomma cajennense	*Amblyomma cajennense*	1	2	2
Amblyomma cajennense	*Amblyomma cajennense*	1	2	2
Amblyomma cajennense	*Amblyomma cajennense*	1	2	2
Amblyomma cajennense	*Amblyomma cajennense*	1	2	2
Amblyomma cajennense	*Amblyomma cajennense*	1	2	2
Amblyomma cajennense	*Amblyomma cajennense*	1	2	2
Amblyomma cajennense	*Amblyomma triste*	0	2	6
Amblyomma cajennense	*Amblyomma cajennense*	1	2	2
Amblyomma cajennense	*Amblyomma cajennense*	1	2	2
Amblyomma cajennense	*Amblyomma cajennense*	1	2	2
Amblyomma cajennense	*Amblyomma cajennense*	1	2	2
Amblyomma cajennense	*Amblyomma cajennense*	1	2	2
Amblyomma cajennense	*Amblyomma cajennense*	1	2	2
Amblyomma dubitatum	*Amblyomma dubitatum*	1	3	3
Amblyomma dubitatum	*Amblyomma dubitatum*	1	3	3
Amblyomma dubitatum	*Amblyomma dubitatum*	1	3	3
Amblyomma dubitatum	*Amblyomma ovale*	0	3	4
Amblyomma dubitatum	*Amblyomma dubitatum*	1	3	3
Amblyomma dubitatum	*Amblyomma cajennense*	0	3	2
Amblyomma dubitatum	*Amblyomma cajennense*	0	3	2
Amblyomma dubitatum	*Amblyomma dubitatum*	1	3	3
Amblyomma dubitatum	*Amblyomma dubitatum*	1	3	3
Amblyomma dubitatum	*Amblyomma triste*	0	3	6
Amblyomma dubitatum	*Amblyomma sculptum*	0	3	5
Amblyomma ovale	*Amblyomma ovale*	1	4	4
Amblyomma ovale	*Amblyomma ovale*	1	4	4
Amblyomma ovale	*Amblyomma ovale*	1	4	4
Amblyomma ovale	*Amblyomma ovale*	1	4	4
Amblyomma ovale	*Amblyomma ovale*	1	4	4
Amblyomma ovale	*Amblyomma ovale*	1	4	4
Amblyomma ovale	*Amblyomma ovale*	1	4	4
Amblyomma ovale	*Amblyomma ovale*	1	4	4
Amblyomma ovale	*Amblyomma ovale*	1	4	4
Amblyomma ovale	*Amblyomma ovale*	1	4	4
Amblyomma ovale	*Amblyomma ovale*	1	4	4
Amblyomma ovale	*Amblyomma ovale*	1	4	4
Amblyomma sculptum	*Amblyomma cajennense*	0	5	2

Amblyomma sculptum	*Amblyomma ovale*	0	5	4
Amblyomma sculptum	*Amblyomma aureolatum*	0	5	1
Amblyomma sculptum	*Amblyomma sculptum*	1	5	5
Amblyomma sculptum	*Amblyomma sculptum*	1	5	5
Amblyomma sculptum	*Amblyomma cajennense*	0	5	2
Amblyomma sculptum	*Amblyomma sculptum*	1	5	5
Amblyomma sculptum	*Amblyomma sculptum*	1	5	5
Amblyomma sculptum	*Amblyomma sculptum*	1	5	5
Amblyomma sculptum	*Amblyomma sculptum*	1	5	5
Amblyomma sculptum	*Amblyomma sculptum*	1	5	5
Amblyomma sculptum	*Amblyomma sculptum*	1	5	5
Amblyomma sculptum	*Amblyomma sculptum*	1	5	5
Amblyomma sculptum	*Amblyomma sculptum*	1	5	5
Amblyomma triste	*Amblyomma triste*	1	6	6
Amblyomma triste	*Amblyomma triste*	1	6	6
Amblyomma triste	*Amblyomma triste*	1	6	6
Amblyomma triste	*Amblyomma triste*	1	6	6
Amblyomma triste	*Amblyomma triste*	1	6	6
Amblyomma triste	*Amblyomma triste*	1	6	6
Amblyomma triste	*Amblyomma triste*	1	6	6
Amblyomma triste	*Amblyomma triste*	1	6	6
Amblyomma triste	*Amblyomma triste*	1	6	6
Amblyomma triste	*Amblyomma triste*	1	6	6
Amblyomma triste	*Amblyomma triste*	1	6	6
Amblyomma triste	*Amblyomma triste*	1	6	6
Amblyomma triste	*Amblyomma triste*	1	6	6
Amblyomma triste	*Amblyomma triste*	1	6	6
Amblyomma triste	*Amblyomma cajennense*	0	6	2
Amblyomma aureolatum	*Amblyomma aureolatum*	1	1	1
Amblyomma aureolatum	*Amblyomma aureolatum*	1	1	1
Amblyomma aureolatum	*Amblyomma aureolatum*	1	1	1
Amblyomma aureolatum	*Amblyomma aureolatum*	1	1	1
Amblyomma aureolatum	*Amblyomma dubitatum*	0	1	3
Amblyomma aureolatum	*Amblyomma aureolatum*	1	1	1
Amblyomma aureolatum	*Amblyomma aureolatum*	1	1	1
Amblyomma aureolatum	*Amblyomma aureolatum*	1	1	1
Amblyomma aureolatum	*Amblyomma aureolatum*	1	1	1
Amblyomma aureolatum	*Amblyomma aureolatum*	1	1	1
Amblyomma aureolatum	*Amblyomma aureolatum*	1	1	1
Amblyomma cajennense	*Amblyomma cajennense*	1	2	2
Amblyomma cajennense	*Amblyomma cajennense*	1	2	2
Amblyomma cajennense	*Amblyomma cajennense*	1	2	2
Amblyomma cajennense	*Amblyomma cajennense*	1	2	2
Amblyomma cajennense	*Amblyomma cajennense*	1	2	2
Amblyomma cajennense	*Amblyomma cajennense*	1	2	2
Amblyomma cajennense	*Amblyomma cajennense*	1	2	2
Amblyomma cajennense	*Amblyomma cajennense*	1	2	2
Amblyomma cajennense	*Amblyomma cajennense*	1	2	2
Amblyomma cajennense	*Amblyomma cajennense*	1	2	2
Amblyomma cajennense	*Amblyomma cajennense*	1	2	2
Amblyomma cajennense	*Amblyomma cajennense*	1	2	2
Amblyomma cajennense	*Amblyomma cajennense*	1	2	2
Amblyomma cajennense	*Amblyomma cajennense*	1	2	2
Amblyomma cajennense	*Amblyomma cajennense*	1	2	2
Amblyomma cajennense	*Amblyomma cajennense*	1	2	2
Amblyomma dubitatum	*Amblyomma dubitatum*	1	3	3
Amblyomma dubitatum	*Amblyomma dubitatum*	1	3	3
Amblyomma dubitatum	*Amblyomma triste*	0	3	6
Amblyomma dubitatum	*Amblyomma dubitatum*	1	3	3
Amblyomma dubitatum	*Amblyomma aureolatum*	0	3	1
Amblyomma dubitatum	*Amblyomma dubitatum*	1	3	3
Amblyomma dubitatum	*Amblyomma ovale*	0	3	4
Amblyomma dubitatum	*Amblyomma dubitatum*	1	3	3
Amblyomma dubitatum	*Amblyomma dubitatum*	1	3	3
Amblyomma dubitatum	*Amblyomma dubitatum*	1	3	3
Amblyomma ovale	*Amblyomma ovale*	1	4	4
Amblyomma ovale	*Amblyomma ovale*	1	4	4

Amblyomma ovale	*Amblyomma ovale*	1	4	4
Amblyomma ovale	*Amblyomma aureolatum*	0	4	1
Amblyomma ovale	*Amblyomma ovale*	1	4	4
Amblyomma ovale	*Amblyomma triste*	0	4	6
Amblyomma ovale	*Amblyomma aureolatum*	0	4	1
Amblyomma ovale	*Amblyomma ovale*	1	4	4
Amblyomma ovale	*Amblyomma ovale*	1	4	4
Amblyomma ovale	*Amblyomma ovale*	1	4	4
Amblyomma ovale	*Amblyomma ovale*	1	4	4
Amblyomma ovale	*Amblyomma ovale*	1	4	4
Amblyomma sculptum	*Amblyomma sculptum*	1	5	5
Amblyomma sculptum	*Amblyomma sculptum*	1	5	5
Amblyomma sculptum	*Amblyomma sculptum*	1	5	5
Amblyomma sculptum	*Amblyomma sculptum*	1	5	5
Amblyomma sculptum	*Amblyomma sculptum*	1	5	5
Amblyomma sculptum	*Amblyomma sculptum*	1	5	5
Amblyomma sculptum	*Amblyomma sculptum*	1	5	5
Amblyomma sculptum	*Amblyomma sculptum*	1	5	5
Amblyomma sculptum	*Amblyomma sculptum*	1	5	5
Amblyomma sculptum	*Amblyomma sculptum*	1	5	5
Amblyomma sculptum	*Amblyomma sculptum*	1	5	5
Amblyomma sculptum	*Amblyomma sculptum*	1	5	5
Amblyomma sculptum	*Amblyomma sculptum*	1	5	5
Amblyomma sculptum	*Amblyomma sculptum*	1	5	5
Amblyomma sculptum	*Amblyomma sculptum*	1	5	5
Amblyomma triste	*Amblyomma triste*	1	6	6
Amblyomma triste	*Amblyomma ovale*	0	6	4
Amblyomma triste	*Amblyomma triste*	1	6	6
Amblyomma triste	*Amblyomma triste*	1	6	6
Amblyomma triste	*Amblyomma triste*	1	6	6
Amblyomma triste	*Amblyomma triste*	1	6	6
Amblyomma triste	*Amblyomma triste*	1	6	6
Amblyomma triste	*Amblyomma triste*	1	6	6
Amblyomma triste	*Amblyomma triste*	1	6	6
Amblyomma triste	*Amblyomma dubitatum*	0	6	3
Amblyomma triste	*Amblyomma triste*	1	6	6
Amblyomma triste	*Amblyomma triste*	1	6	6
Amblyomma triste	*Amblyomma aureolatum*	0	6	1
Amblyomma triste	*Amblyomma triste*	1	6	6
Amblyomma triste	*Amblyomma triste*	1	6	6
Amblyomma aureolatum	*Amblyomma aureolatum*	1	1	1
Amblyomma aureolatum	*Amblyomma aureolatum*	1	1	1
Amblyomma aureolatum	*Amblyomma aureolatum*	1	1	1
Amblyomma aureolatum	*Amblyomma aureolatum*	1	1	1
Amblyomma aureolatum	*Amblyomma aureolatum*	1	1	1
Amblyomma aureolatum	*Amblyomma aureolatum*	1	1	1
Amblyomma aureolatum	*Amblyomma aureolatum*	1	1	1
Amblyomma aureolatum	*Amblyomma aureolatum*	1	1	1
Amblyomma aureolatum	*Amblyomma aureolatum*	1	1	1
Amblyomma aureolatum	*Amblyomma aureolatum*	1	1	1
Amblyomma aureolatum	*Amblyomma aureolatum*	1	1	1
Amblyomma aureolatum	*Amblyomma aureolatum*	1	1	1
Amblyomma cajennense	*Amblyomma cajennense*	1	2	2
Amblyomma cajennense	*Amblyomma cajennense*	1	2	2
Amblyomma cajennense	*Amblyomma cajennense*	1	2	2
Amblyomma cajennense	*Amblyomma cajennense*	1	2	2
Amblyomma cajennense	*Amblyomma cajennense*	1	2	2
Amblyomma cajennense	*Amblyomma cajennense*	1	2	2
Amblyomma cajennense	*Amblyomma cajennense*	1	2	2
Amblyomma cajennense	*Amblyomma cajennense*	1	2	2
Amblyomma cajennense	*Amblyomma cajennense*	1	2	2
Amblyomma cajennense	*Amblyomma cajennense*	1	2	2
Amblyomma cajennense	*Amblyomma cajennense*	1	2	2
Amblyomma cajennense	*Amblyomma cajennense*	1	2	2
Amblyomma cajennense	*Amblyomma cajennense*	1	2	2
Amblyomma cajennense	*Amblyomma cajennense*	1	2	2
Amblyomma cajennense	*Amblyomma cajennense*	1	2	2

Amblyomma cajennense	*Amblyomma cajennense*	1	2	2
Amblyomma cajennense	*Amblyomma cajennense*	1	2	2
Amblyomma dubitatum	*Amblyomma dubitatum*	1	3	3
Amblyomma dubitatum	*Amblyomma dubitatum*	1	3	3
Amblyomma dubitatum	*Amblyomma dubitatum*	1	3	3
Amblyomma dubitatum	*Amblyomma dubitatum*	1	3	3
Amblyomma dubitatum	*Amblyomma dubitatum*	1	3	3
Amblyomma dubitatum	*Amblyomma dubitatum*	1	3	3
Amblyomma dubitatum	*Amblyomma aureolatum*	0	3	1
Amblyomma dubitatum	*Amblyomma dubitatum*	1	3	3
Amblyomma dubitatum	*Amblyomma dubitatum*	1	3	3
Amblyomma dubitatum	*Amblyomma ovale*	0	3	4
Amblyomma dubitatum	*Amblyomma dubitatum*	1	3	3
Amblyomma ovale	*Amblyomma ovale*	1	4	4
Amblyomma ovale	*Amblyomma ovale*	1	4	4
Amblyomma ovale	*Amblyomma ovale*	1	4	4
Amblyomma ovale	*Amblyomma ovale*	1	4	4
Amblyomma ovale	*Amblyomma ovale*	1	4	4
Amblyomma ovale	*Amblyomma ovale*	1	4	4
Amblyomma ovale	*Amblyomma ovale*	1	4	4
Amblyomma ovale	*Amblyomma ovale*	1	4	4
Amblyomma ovale	*Amblyomma ovale*	1	4	4
Amblyomma ovale	*Amblyomma ovale*	1	4	4
Amblyomma ovale	*Amblyomma cajennense*	0	4	2
Amblyomma ovale	*Amblyomma ovale*	1	4	4
Amblyomma sculptum	*Amblyomma sculptum*	1	5	5
Amblyomma sculptum	*Amblyomma sculptum*	1	5	5
Amblyomma sculptum	*Amblyomma sculptum*	1	5	5
Amblyomma sculptum	*Amblyomma sculptum*	1	5	5
Amblyomma sculptum	*Amblyomma aureolatum*	0	5	1
Amblyomma sculptum	*Amblyomma cajennense*	0	5	2
Amblyomma sculptum	*Amblyomma dubitatum*	0	5	3
Amblyomma sculptum	*Amblyomma sculptum*	1	5	5
Amblyomma sculptum	*Amblyomma sculptum*	1	5	5
Amblyomma sculptum	*Amblyomma sculptum*	1	5	5
Amblyomma sculptum	*Amblyomma sculptum*	1	5	5
Amblyomma sculptum	*Amblyomma sculptum*	1	5	5
Amblyomma sculptum	*Amblyomma sculptum*	1	5	5
Amblyomma sculptum	*Amblyomma sculptum*	1	5	5
Amblyomma sculptum	*Amblyomma sculptum*	1	5	5
Amblyomma triste	*Amblyomma triste*	1	6	6
Amblyomma triste	*Amblyomma triste*	1	6	6
Amblyomma triste	*Amblyomma cajennense*	0	6	2
Amblyomma triste	*Amblyomma ovale*	0	6	4
Amblyomma triste	*Amblyomma triste*	1	6	6
Amblyomma triste	*Amblyomma triste*	1	6	6
Amblyomma triste	*Amblyomma triste*	1	6	6
Amblyomma triste	*Amblyomma triste*	1	6	6
Amblyomma triste	*Amblyomma triste*	1	6	6
Amblyomma triste	*Amblyomma cajennense*	0	6	2
Amblyomma triste	*Amblyomma triste*	1	6	6
Amblyomma triste	*Amblyomma triste*	1	6	6
Amblyomma triste	*Amblyomma triste*	1	6	6
Amblyomma triste	*Amblyomma triste*	1	6	6
Amblyomma triste	*Amblyomma triste*	1	6	6
Amblyomma triste	*Amblyomma triste*	1	6	6
Amblyomma aureolatum	*Amblyomma aureolatum*	1	1	1
Amblyomma aureolatum	*Amblyomma aureolatum*	1	1	1
Amblyomma aureolatum	*Amblyomma aureolatum*	1	1	1
Amblyomma aureolatum	*Amblyomma aureolatum*	1	1	1
Amblyomma aureolatum	*Amblyomma aureolatum*	1	1	1
Amblyomma aureolatum	*Amblyomma aureolatum*	1	1	1
Amblyomma aureolatum	*Amblyomma aureolatum*	1	1	1
Amblyomma aureolatum	*Amblyomma aureolatum*	1	1	1
Amblyomma aureolatum	*Amblyomma aureolatum*	1	1	1
Amblyomma aureolatum	*Amblyomma aureolatum*	1	1	1
Amblyomma aureolatum	*Amblyomma aureolatum*	1	1	1

Amblyomma cajennense	*Amblyomma cajennense*	1	2	2
Amblyomma cajennense	*Amblyomma cajennense*	1	2	2
Amblyomma cajennense	*Amblyomma cajennense*	1	2	2
Amblyomma cajennense	*Amblyomma cajennense*	1	2	2
Amblyomma cajennense	*Amblyomma cajennense*	1	2	2
Amblyomma cajennense	*Amblyomma cajennense*	1	2	2
Amblyomma cajennense	*Amblyomma cajennense*	1	2	2
Amblyomma cajennense	*Amblyomma cajennense*	1	2	2
Amblyomma cajennense	*Amblyomma cajennense*	1	2	2
Amblyomma cajennense	*Amblyomma cajennense*	1	2	2
Amblyomma cajennense	*Amblyomma cajennense*	1	2	2
Amblyomma cajennense	*Amblyomma cajennense*	1	2	2
Amblyomma cajennense	*Amblyomma cajennense*	1	2	2
Amblyomma cajennense	*Amblyomma cajennense*	1	2	2
Amblyomma cajennense	*Amblyomma cajennense*	1	2	2
Amblyomma cajennense	*Amblyomma cajennense*	1	2	2
Amblyomma cajennense	*Amblyomma cajennense*	1	2	2
Amblyomma dubitatum	*Amblyomma dubitatum*	1	3	3
Amblyomma dubitatum	*Amblyomma sculptum*	0	3	5
Amblyomma dubitatum	*Amblyomma dubitatum*	1	3	3
Amblyomma dubitatum	*Amblyomma dubitatum*	1	3	3
Amblyomma dubitatum	*Amblyomma dubitatum*	1	3	3
Amblyomma dubitatum	*Amblyomma cajennense*	0	3	2
Amblyomma dubitatum	*Amblyomma dubitatum*	1	3	3
Amblyomma dubitatum	*Amblyomma dubitatum*	1	3	3
Amblyomma dubitatum	*Amblyomma dubitatum*	1	3	3
Amblyomma dubitatum	*Amblyomma dubitatum*	1	3	3
Amblyomma ovale	*Amblyomma ovale*	1	4	4
Amblyomma ovale	*Amblyomma ovale*	1	4	4
Amblyomma ovale	*Amblyomma ovale*	1	4	4
Amblyomma ovale	*Amblyomma ovale*	1	4	4
Amblyomma ovale	*Amblyomma ovale*	1	4	4
Amblyomma ovale	*Amblyomma ovale*	1	4	4
Amblyomma ovale	*Amblyomma ovale*	1	4	4
Amblyomma ovale	*Amblyomma ovale*	1	4	4
Amblyomma ovale	*Amblyomma ovale*	1	4	4
Amblyomma ovale	*Amblyomma sculptum*	0	4	5
Amblyomma ovale	*Amblyomma ovale*	1	4	4
Amblyomma ovale	*Amblyomma ovale*	1	4	4
Amblyomma sculptum	*Amblyomma sculptum*	1	5	5
Amblyomma sculptum	*Amblyomma sculptum*	1	5	5
Amblyomma sculptum	*Amblyomma sculptum*	1	5	5
Amblyomma sculptum	*Amblyomma sculptum*	1	5	5
Amblyomma sculptum	*Amblyomma sculptum*	1	5	5
Amblyomma sculptum	*Amblyomma aureolatum*	0	5	1
Amblyomma sculptum	*Amblyomma sculptum*	1	5	5
Amblyomma sculptum	*Amblyomma sculptum*	1	5	5
Amblyomma sculptum	*Amblyomma sculptum*	1	5	5
Amblyomma sculptum	*Amblyomma sculptum*	1	5	5
Amblyomma sculptum	*Amblyomma sculptum*	1	5	5
Amblyomma sculptum	*Amblyomma sculptum*	1	5	5
Amblyomma sculptum	*Amblyomma sculptum*	1	5	5
Amblyomma sculptum	*Amblyomma sculptum*	1	5	5
Amblyomma sculptum	*Amblyomma sculptum*	1	5	5
Amblyomma triste	*Amblyomma triste*	1	6	6
Amblyomma triste	*Amblyomma triste*	1	6	6
Amblyomma triste	*Amblyomma triste*	1	6	6
Amblyomma triste	*Amblyomma triste*	1	6	6
Amblyomma triste	*Amblyomma triste*	1	6	6
Amblyomma triste	*Amblyomma triste*	1	6	6
Amblyomma triste	*Amblyomma triste*	1	6	6
Amblyomma triste	*Amblyomma triste*	1	6	6
Amblyomma triste	*Amblyomma triste*	1	6	6
Amblyomma triste	*Amblyomma triste*	1	6	6
Amblyomma triste	*Amblyomma triste*	1	6	6
Amblyomma triste	*Amblyomma triste*	1	6	6
Amblyomma triste	*Amblyomma triste*	1	6	6

Amblyomma triste	*Amblyomma triste*	1	6	6
Amblyomma triste	*Amblyomma cajennense*	0	6	2
Amblyomma aureolatum	*Amblyomma aureolatum*	1	1	1
Amblyomma aureolatum	*Amblyomma aureolatum*	1	1	1
Amblyomma aureolatum	*Amblyomma aureolatum*	1	1	1
Amblyomma aureolatum	*Amblyomma aureolatum*	1	1	1
Amblyomma aureolatum	*Amblyomma aureolatum*	1	1	1
Amblyomma aureolatum	*Amblyomma aureolatum*	1	1	1
Amblyomma aureolatum	*Amblyomma aureolatum*	1	1	1
Amblyomma aureolatum	*Amblyomma aureolatum*	1	1	1
Amblyomma aureolatum	*Amblyomma aureolatum*	1	1	1
Amblyomma aureolatum	*Amblyomma aureolatum*	1	1	1
Amblyomma cajennense	*Amblyomma cajennense*	1	2	2
Amblyomma cajennense	*Amblyomma cajennense*	1	2	2
Amblyomma cajennense	*Amblyomma cajennense*	1	2	2
Amblyomma cajennense	*Amblyomma dubitatum*	0	2	3
Amblyomma cajennense	*Amblyomma cajennense*	1	2	2
Amblyomma cajennense	*Amblyomma cajennense*	1	2	2
Amblyomma cajennense	*Amblyomma cajennense*	1	2	2
Amblyomma cajennense	*Amblyomma cajennense*	1	2	2
Amblyomma cajennense	*Amblyomma triste*	0	2	6
Amblyomma cajennense	*Amblyomma cajennense*	1	2	2
Amblyomma cajennense	*Amblyomma sculptum*	0	2	5
Amblyomma cajennense	*Amblyomma cajennense*	1	2	2
Amblyomma cajennense	*Amblyomma cajennense*	1	2	2
Amblyomma cajennense	*Amblyomma cajennense*	1	2	2
Amblyomma cajennense	*Amblyomma cajennense*	1	2	2
Amblyomma cajennense	*Amblyomma dubitatum*	0	2	3
Amblyomma dubitatum	*Amblyomma dubitatum*	1	3	3
Amblyomma dubitatum	*Amblyomma dubitatum*	1	3	3
Amblyomma dubitatum	*Amblyomma dubitatum*	1	3	3
Amblyomma dubitatum	*Amblyomma dubitatum*	1	3	3
Amblyomma dubitatum	*Amblyomma dubitatum*	1	3	3
Amblyomma dubitatum	*Amblyomma dubitatum*	1	3	3
Amblyomma dubitatum	*Amblyomma dubitatum*	1	3	3
Amblyomma dubitatum	*Amblyomma dubitatum*	1	3	3
Amblyomma ovale	*Amblyomma ovale*	1	4	4
Amblyomma ovale	*Amblyomma ovale*	1	4	4
Amblyomma ovale	*Amblyomma ovale*	1	4	4
Amblyomma ovale	*Amblyomma triste*	0	4	6
Amblyomma ovale	*Amblyomma ovale*	1	4	4
Amblyomma ovale	*Amblyomma triste*	0	4	6
Amblyomma ovale	*Amblyomma ovale*	1	4	4
Amblyomma ovale	*Amblyomma ovale*	1	4	4
Amblyomma ovale	*Amblyomma ovale*	1	4	4
Amblyomma ovale	*Amblyomma dubitatum*	0	4	3
Amblyomma ovale	*Amblyomma ovale*	1	4	4
Amblyomma sculptum	*Amblyomma sculptum*	1	5	5
Amblyomma sculptum	*Amblyomma sculptum*	1	5	5
Amblyomma sculptum	*Amblyomma sculptum*	1	5	5
Amblyomma sculptum	*Amblyomma sculptum*	1	5	5
Amblyomma triste	*Amblyomma triste*	1	6	6
Amblyomma triste	*Amblyomma triste*	1	6	6
Amblyomma triste	*Amblyomma triste*	1	6	6
Amblyomma triste	*Amblyomma triste*	1	6	6
Amblyomma triste	*Amblyomma triste*	1	6	6
Amblyomma triste	*Amblyomma triste*	1	6	6
Amblyomma triste	*Amblyomma triste*	1	6	6
Amblyomma triste	*Amblyomma triste*	1	6	6
Amblyomma triste	*Amblyomma triste*	1	6	6
Amblyomma triste	*Amblyomma triste*	1	6	6
Amblyomma triste	*Amblyomma triste*	1	6	6
Amblyomma triste	*Amblyomma triste*	1	6	6
Amblyomma triste	*Amblyomma triste*	1	6	6
Amblyomma triste	*Amblyomma triste*	1	6	6
Amblyomma triste	*Amblyomma triste*	1	6	6
Amblyomma triste	*Amblyomma triste*	1	6	6

Amblyomma aureolatum	*Amblyomma cajennense*	0	1	2
Amblyomma aureolatum	*Amblyomma aureolatum*	1	1	1
Amblyomma aureolatum	*Amblyomma aureolatum*	1	1	1
Amblyomma aureolatum	*Amblyomma aureolatum*	1	1	1
Amblyomma aureolatum	*Amblyomma aureolatum*	1	1	1
Amblyomma aureolatum	*Amblyomma aureolatum*	1	1	1
Amblyomma aureolatum	*Amblyomma aureolatum*	1	1	1
Amblyomma aureolatum	*Amblyomma aureolatum*	1	1	1
Amblyomma aureolatum	*Amblyomma aureolatum*	1	1	1
Amblyomma aureolatum	*Amblyomma aureolatum*	1	1	1
Amblyomma aureolatum	*Amblyomma aureolatum*	1	1	1
Amblyomma cajennense	*Amblyomma cajennense*	1	2	2
Amblyomma cajennense	*Amblyomma cajennense*	1	2	2
Amblyomma cajennense	*Amblyomma cajennense*	1	2	2
Amblyomma cajennense	*Amblyomma cajennense*	1	2	2
Amblyomma cajennense	*Amblyomma cajennense*	1	2	2
Amblyomma cajennense	*Amblyomma cajennense*	1	2	2
Amblyomma cajennense	*Amblyomma cajennense*	1	2	2
Amblyomma cajennense	*Amblyomma cajennense*	1	2	2
Amblyomma cajennense	*Amblyomma cajennense*	1	2	2
Amblyomma cajennense	*Amblyomma cajennense*	1	2	2
Amblyomma cajennense	*Amblyomma cajennense*	1	2	2
Amblyomma cajennense	*Amblyomma cajennense*	1	2	2
Amblyomma cajennense	*Amblyomma cajennense*	1	2	2
Amblyomma cajennense	*Amblyomma cajennense*	1	2	2
Amblyomma cajennense	*Amblyomma cajennense*	1	2	2
Amblyomma cajennense	*Amblyomma cajennense*	1	2	2
Amblyomma dubitatum	*Amblyomma dubitatum*	1	3	3
Amblyomma dubitatum	*Amblyomma dubitatum*	1	3	3
Amblyomma dubitatum	*Amblyomma dubitatum*	1	3	3
Amblyomma dubitatum	*Amblyomma dubitatum*	1	3	3
Amblyomma dubitatum	*Amblyomma dubitatum*	1	3	3
Amblyomma dubitatum	*Amblyomma dubitatum*	1	3	3
Amblyomma dubitatum	*Amblyomma dubitatum*	1	3	3
Amblyomma dubitatum	*Amblyomma dubitatum*	1	3	3
Amblyomma dubitatum	*Amblyomma dubitatum*	1	3	3
Amblyomma ovale	*Amblyomma ovale*	1	4	4
Amblyomma ovale	*Amblyomma triste*	0	4	6
Amblyomma ovale	*Amblyomma ovale*	1	4	4
Amblyomma ovale	*Amblyomma ovale*	1	4	4
Amblyomma ovale	*Amblyomma ovale*	1	4	4
Amblyomma ovale	*Amblyomma ovale*	1	4	4
Amblyomma ovale	*Amblyomma ovale*	1	4	4
Amblyomma ovale	*Amblyomma ovale*	1	4	4
Amblyomma ovale	*Amblyomma ovale*	1	4	4
Amblyomma ovale	*Amblyomma ovale*	1	4	4
Amblyomma ovale	*Amblyomma ovale*	1	4	4
Amblyomma sculptum	*Amblyomma sculptum*	1	5	5
Amblyomma sculptum	*Amblyomma sculptum*	1	5	5
Amblyomma sculptum	*Amblyomma sculptum*	1	5	5
Amblyomma triste	*Amblyomma triste*	1	6	6
Amblyomma triste	*Amblyomma triste*	1	6	6
Amblyomma triste	*Amblyomma triste*	1	6	6
Amblyomma triste	*Amblyomma triste*	1	6	6
Amblyomma triste	*Amblyomma triste*	1	6	6
Amblyomma triste	*Amblyomma triste*	1	6	6
Amblyomma triste	*Amblyomma triste*	1	6	6
Amblyomma triste	*Amblyomma triste*	1	6	6
Amblyomma triste	*Amblyomma dubitatum*	0	6	3
Amblyomma triste	*Amblyomma triste*	1	6	6
Amblyomma triste	*Amblyomma triste*	1	6	6
Amblyomma triste	*Amblyomma triste*	1	6	6
Amblyomma triste	*Amblyomma triste*	1	6	6
Amblyomma triste	*Amblyomma triste*	1	6	6
Amblyomma triste	*Amblyomma triste*	1	6	6
Amblyomma triste	*Amblyomma triste*	1	6	6
Amblyomma aureolatum	*Amblyomma aureolatum*	1	1	1

Amblyomma aureolatum	*Amblyomma aureolatum*	1	1	1
Amblyomma aureolatum	*Amblyomma triste*	0	1	6
Amblyomma aureolatum	*Amblyomma aureolatum*	1	1	1
Amblyomma aureolatum	*Amblyomma aureolatum*	1	1	1
Amblyomma aureolatum	*Amblyomma aureolatum*	1	1	1
Amblyomma aureolatum	*Amblyomma aureolatum*	1	1	1
Amblyomma aureolatum	*Amblyomma aureolatum*	1	1	1
Amblyomma aureolatum	*Amblyomma aureolatum*	1	1	1
Amblyomma aureolatum	*Amblyomma aureolatum*	1	1	1
Amblyomma cajennense	*Amblyomma cajennense*	1	2	2
Amblyomma cajennense	*Amblyomma cajennense*	1	2	2
Amblyomma cajennense	*Amblyomma cajennense*	1	2	2
Amblyomma cajennense	*Amblyomma cajennense*	1	2	2
Amblyomma cajennense	*Amblyomma cajennense*	1	2	2
Amblyomma cajennense	*Amblyomma cajennense*	1	2	2
Amblyomma cajennense	*Amblyomma cajennense*	1	2	2
Amblyomma cajennense	*Amblyomma cajennense*	1	2	2
Amblyomma cajennense	*Amblyomma cajennense*	1	2	2
Amblyomma cajennense	*Amblyomma cajennense*	1	2	2
Amblyomma cajennense	*Amblyomma cajennense*	1	2	2
Amblyomma cajennense	*Amblyomma cajennense*	1	2	2
Amblyomma cajennense	*Amblyomma cajennense*	1	2	2
Amblyomma cajennense	*Amblyomma cajennense*	1	2	2
Amblyomma cajennense	*Amblyomma cajennense*	1	2	2
Amblyomma cajennense	*Amblyomma cajennense*	1	2	2
Amblyomma cajennense	*Amblyomma cajennense*	1	2	2
Amblyomma dubitatum	*Amblyomma dubitatum*	1	3	3
Amblyomma dubitatum	*Amblyomma dubitatum*	1	3	3
Amblyomma dubitatum	*Amblyomma dubitatum*	1	3	3
Amblyomma dubitatum	*Amblyomma dubitatum*	1	3	3
Amblyomma dubitatum	*Amblyomma dubitatum*	1	3	3
Amblyomma dubitatum	*Amblyomma dubitatum*	1	3	3
Amblyomma dubitatum	*Amblyomma dubitatum*	1	3	3
Amblyomma dubitatum	*Amblyomma dubitatum*	1	3	3
Amblyomma dubitatum	*Amblyomma dubitatum*	1	3	3
Amblyomma ovale	*Amblyomma ovale*	1	4	4
Amblyomma ovale	*Amblyomma ovale*	1	4	4
Amblyomma ovale	*Amblyomma ovale*	1	4	4
Amblyomma ovale	*Amblyomma ovale*	1	4	4
Amblyomma ovale	*Amblyomma ovale*	1	4	4
Amblyomma ovale	*Amblyomma aureolatum*	0	4	1
Amblyomma ovale	*Amblyomma ovale*	1	4	4
Amblyomma ovale	*Amblyomma ovale*	1	4	4
Amblyomma ovale	*Amblyomma aureolatum*	0	4	1
Amblyomma ovale	*Amblyomma dubitatum*	0	4	3
Amblyomma ovale	*Amblyomma ovale*	1	4	4
Amblyomma sculptum	*Amblyomma dubitatum*	0	5	3
Amblyomma sculptum	*Amblyomma sculptum*	1	5	5
Amblyomma sculptum	*Amblyomma sculptum*	1	5	5
Amblyomma sculptum	*Amblyomma sculptum*	1	5	5
Amblyomma triste	*Amblyomma triste*	1	6	6
Amblyomma triste	*Amblyomma triste*	1	6	6
Amblyomma triste	*Amblyomma triste*	1	6	6
Amblyomma triste	*Amblyomma triste*	1	6	6
Amblyomma triste	*Amblyomma triste*	1	6	6
Amblyomma triste	*Amblyomma triste*	1	6	6
Amblyomma triste	*Amblyomma triste*	1	6	6
Amblyomma triste	*Amblyomma triste*	1	6	6
Amblyomma triste	*Amblyomma triste*	1	6	6
Amblyomma triste	*Amblyomma triste*	1	6	6
Amblyomma triste	*Amblyomma triste*	1	6	6
Amblyomma triste	*Amblyomma triste*	1	6	6
Amblyomma triste	*Amblyomma triste*	1	6	6
Amblyomma triste	*Amblyomma triste*	1	6	6
Amblyomma triste	*Amblyomma triste*	1	6	6
Amblyomma aureolatum	*Amblyomma aureolatum*	1	1	1
Amblyomma aureolatum	*Amblyomma aureolatum*	1	1	1

Amblyomma aureolatum	*Amblyomma aureolatum*	1	1	1
Amblyomma aureolatum	*Amblyomma aureolatum*	1	1	1
Amblyomma aureolatum	*Amblyomma aureolatum*	1	1	1
Amblyomma aureolatum	*Amblyomma aureolatum*	1	1	1
Amblyomma aureolatum	*Amblyomma aureolatum*	1	1	1
Amblyomma aureolatum	*Amblyomma aureolatum*	1	1	1
Amblyomma aureolatum	*Amblyomma aureolatum*	1	1	1
Amblyomma aureolatum	*Amblyomma aureolatum*	1	1	1
Amblyomma aureolatum	*Amblyomma aureolatum*	1	1	1
Amblyomma cajennense	*Amblyomma cajennense*	1	2	2
Amblyomma cajennense	*Amblyomma cajennense*	1	2	2
Amblyomma cajennense	*Amblyomma cajennense*	1	2	2
Amblyomma cajennense	*Amblyomma cajennense*	1	2	2
Amblyomma cajennense	*Amblyomma cajennense*	1	2	2
Amblyomma cajennense	*Amblyomma cajennense*	1	2	2
Amblyomma cajennense	*Amblyomma cajennense*	1	2	2
Amblyomma cajennense	*Amblyomma cajennense*	1	2	2
Amblyomma cajennense	*Amblyomma cajennense*	1	2	2
Amblyomma cajennense	*Amblyomma triste*	0	2	6
Amblyomma cajennense	*Amblyomma cajennense*	1	2	2
Amblyomma cajennense	*Amblyomma cajennense*	1	2	2
Amblyomma cajennense	*Amblyomma cajennense*	1	2	2
Amblyomma cajennense	*Amblyomma cajennense*	1	2	2
Amblyomma cajennense	*Amblyomma cajennense*	1	2	2
Amblyomma cajennense	*Amblyomma cajennense*	1	2	2
Amblyomma dubitatum	*Amblyomma aureolatum*	0	3	1
Amblyomma dubitatum	*Amblyomma dubitatum*	1	3	3
Amblyomma dubitatum	*Amblyomma dubitatum*	1	3	3
Amblyomma dubitatum	*Amblyomma dubitatum*	1	3	3
Amblyomma dubitatum	*Amblyomma ovale*	0	3	4
Amblyomma dubitatum	*Amblyomma dubitatum*	1	3	3
Amblyomma dubitatum	*Amblyomma dubitatum*	1	3	3
Amblyomma dubitatum	*Amblyomma dubitatum*	1	3	3
Amblyomma dubitatum	*Amblyomma dubitatum*	1	3	3
Amblyomma ovale	*Amblyomma ovale*	1	4	4
Amblyomma ovale	*Amblyomma ovale*	1	4	4
Amblyomma ovale	*Amblyomma ovale*	1	4	4
Amblyomma ovale	*Amblyomma triste*	0	4	6
Amblyomma ovale	*Amblyomma ovale*	1	4	4
Amblyomma ovale	*Amblyomma ovale*	1	4	4
Amblyomma ovale	*Amblyomma ovale*	1	4	4
Amblyomma ovale	*Amblyomma ovale*	1	4	4
Amblyomma ovale	*Amblyomma triste*	0	4	6
Amblyomma ovale	*Amblyomma aureolatum*	0	4	1
Amblyomma ovale	*Amblyomma ovale*	1	4	4
Amblyomma sculptum	*Amblyomma sculptum*	1	5	5
Amblyomma sculptum	*Amblyomma sculptum*	1	5	5
Amblyomma sculptum	*Amblyomma dubitatum*	0	5	3
Amblyomma triste	*Amblyomma triste*	1	6	6
Amblyomma triste	*Amblyomma triste*	1	6	6
Amblyomma triste	*Amblyomma triste*	1	6	6
Amblyomma triste	*Amblyomma triste*	1	6	6
Amblyomma triste	*Amblyomma triste*	1	6	6
Amblyomma triste	*Amblyomma triste*	1	6	6
Amblyomma triste	*Amblyomma triste*	1	6	6
Amblyomma triste	*Amblyomma triste*	1	6	6
Amblyomma triste	*Amblyomma triste*	1	6	6
Amblyomma triste	*Amblyomma triste*	1	6	6
Amblyomma triste	*Amblyomma triste*	1	6	6
Amblyomma triste	*Amblyomma triste*	1	6	6
Amblyomma triste	*Amblyomma triste*	1	6	6
Amblyomma triste	*Amblyomma triste*	1	6	6
Amblyomma triste	*Amblyomma triste*	1	6	6
Amblyomma aureolatum	*Amblyomma aureolatum*	1	1	1
Amblyomma aureolatum	*Amblyomma aureolatum*	1	1	1
Amblyomma aureolatum	*Amblyomma aureolatum*	1	1	1
Amblyomma aureolatum	*Amblyomma aureolatum*	1	1	1

Amblyomma aureolatum	*Amblyomma aureolatum*	1	1	1
Amblyomma aureolatum	*Amblyomma aureolatum*	1	1	1
Amblyomma aureolatum	*Amblyomma aureolatum*	1	1	1
Amblyomma aureolatum	*Amblyomma ovale*	0	1	4
Amblyomma aureolatum	*Amblyomma aureolatum*	1	1	1
Amblyomma aureolatum	*Amblyomma aureolatum*	1	1	1
Amblyomma aureolatum	*Amblyomma aureolatum*	1	1	1
Amblyomma cajennense	*Amblyomma cajennense*	1	2	2
Amblyomma cajennense	*Amblyomma cajennense*	1	2	2
Amblyomma cajennense	*Amblyomma cajennense*	1	2	2
Amblyomma cajennense	*Amblyomma cajennense*	1	2	2
Amblyomma cajennense	*Amblyomma cajennense*	1	2	2
Amblyomma cajennense	*Amblyomma cajennense*	1	2	2
Amblyomma cajennense	*Amblyomma cajennense*	1	2	2
Amblyomma cajennense	*Amblyomma cajennense*	1	2	2
Amblyomma cajennense	*Amblyomma cajennense*	1	2	2
Amblyomma cajennense	*Amblyomma cajennense*	1	2	2
Amblyomma cajennense	*Amblyomma cajennense*	1	2	2
Amblyomma cajennense	*Amblyomma cajennense*	1	2	2
Amblyomma cajennense	*Amblyomma cajennense*	1	2	2
Amblyomma cajennense	*Amblyomma cajennense*	1	2	2
Amblyomma cajennense	*Amblyomma cajennense*	1	2	2
Amblyomma cajennense	*Amblyomma cajennense*	1	2	2
Amblyomma cajennense	*Amblyomma cajennense*	1	2	2
Amblyomma dubitatum	*Amblyomma ovale*	0	3	4
Amblyomma dubitatum	*Amblyomma dubitatum*	1	3	3
Amblyomma dubitatum	*Amblyomma dubitatum*	1	3	3
Amblyomma dubitatum	*Amblyomma dubitatum*	1	3	3
Amblyomma dubitatum	*Amblyomma dubitatum*	1	3	3
Amblyomma dubitatum	*Amblyomma dubitatum*	1	3	3
Amblyomma dubitatum	*Amblyomma aureolatum*	0	3	1
Amblyomma dubitatum	*Amblyomma dubitatum*	1	3	3
Amblyomma dubitatum	*Amblyomma dubitatum*	1	3	3
Amblyomma ovale	*Amblyomma aureolatum*	0	4	1
Amblyomma ovale	*Amblyomma ovale*	1	4	4
Amblyomma ovale	*Amblyomma ovale*	1	4	4
Amblyomma ovale	*Amblyomma ovale*	1	4	4
Amblyomma ovale	*Amblyomma ovale*	1	4	4
Amblyomma ovale	*Amblyomma ovale*	1	4	4
Amblyomma ovale	*Amblyomma ovale*	1	4	4
Amblyomma ovale	*Amblyomma cajennense*	0	4	2
Amblyomma ovale	*Amblyomma ovale*	1	4	4
Amblyomma ovale	*Amblyomma ovale*	1	4	4
Amblyomma ovale	*Amblyomma ovale*	1	4	4
Amblyomma sculptum	*Amblyomma sculptum*	1	5	5
Amblyomma sculptum	*Amblyomma sculptum*	1	5	5
Amblyomma sculptum	*Amblyomma sculptum*	1	5	5
Amblyomma triste	*Amblyomma triste*	1	6	6
Amblyomma triste	*Amblyomma triste*	1	6	6
Amblyomma triste	*Amblyomma triste*	1	6	6
Amblyomma triste	*Amblyomma triste*	1	6	6
Amblyomma triste	*Amblyomma triste*	1	6	6
Amblyomma triste	*Amblyomma triste*	1	6	6
Amblyomma triste	*Amblyomma triste*	1	6	6
Amblyomma triste	*Amblyomma triste*	1	6	6
Amblyomma triste	*Amblyomma triste*	1	6	6
Amblyomma triste	*Amblyomma triste*	1	6	6
Amblyomma triste	*Amblyomma triste*	1	6	6
Amblyomma triste	*Amblyomma triste*	1	6	6
Amblyomma triste	*Amblyomma triste*	1	6	6
Amblyomma triste	*Amblyomma triste*	1	6	6
Amblyomma triste	*Amblyomma triste*	1	6	6
Amblyomma aureolatum	*Amblyomma aureolatum*	1	1	1
Amblyomma aureolatum	*Amblyomma aureolatum*	1	1	1
Amblyomma aureolatum	*Amblyomma aureolatum*	1	1	1
Amblyomma aureolatum	*Amblyomma aureolatum*	1	1	1
Amblyomma aureolatum	*Amblyomma aureolatum*	1	1	1

Amblyomma aureolatum	*Amblyomma aureolatum*	1	1	1
Amblyomma aureolatum	*Amblyomma aureolatum*	1	1	1
Amblyomma aureolatum	*Amblyomma aureolatum*	1	1	1
Amblyomma aureolatum	*Amblyomma aureolatum*	1	1	1
Amblyomma aureolatum	*Amblyomma aureolatum*	1	1	1
Amblyomma aureolatum	*Amblyomma aureolatum*	1	1	1
Amblyomma aureolatum	*Amblyomma aureolatum*	1	1	1
Amblyomma aureolatum	*Amblyomma aureolatum*	1	1	1
Amblyomma cajennense	*Amblyomma cajennense*	1	2	2
Amblyomma cajennense	*Amblyomma cajennense*	1	2	2
Amblyomma cajennense	*Amblyomma cajennense*	1	2	2
Amblyomma cajennense	*Amblyomma cajennense*	1	2	2
Amblyomma cajennense	*Amblyomma cajennense*	1	2	2
Amblyomma cajennense	*Amblyomma cajennense*	1	2	2
Amblyomma cajennense	*Amblyomma cajennense*	1	2	2
Amblyomma cajennense	*Amblyomma cajennense*	1	2	2
Amblyomma cajennense	*Amblyomma cajennense*	1	2	2
Amblyomma cajennense	*Amblyomma cajennense*	1	2	2
Amblyomma cajennense	*Amblyomma cajennense*	1	2	2
Amblyomma cajennense	*Amblyomma triste*	0	2	6
Amblyomma cajennense	*Amblyomma cajennense*	1	2	2
Amblyomma cajennense	*Amblyomma cajennense*	1	2	2
Amblyomma cajennense	*Amblyomma cajennense*	1	2	2
Amblyomma cajennense	*Amblyomma cajennense*	1	2	2
Amblyomma cajennense	*Amblyomma cajennense*	1	2	2
Amblyomma dubitatum	*Amblyomma dubitatum*	1	3	3
Amblyomma dubitatum	*Amblyomma dubitatum*	1	3	3
Amblyomma dubitatum	*Amblyomma ovale*	0	3	4
Amblyomma dubitatum	*Amblyomma dubitatum*	1	3	3
Amblyomma dubitatum	*Amblyomma dubitatum*	1	3	3
Amblyomma dubitatum	*Amblyomma dubitatum*	1	3	3
Amblyomma dubitatum	*Amblyomma dubitatum*	1	3	3
Amblyomma dubitatum	*Amblyomma cajennense*	0	3	2
Amblyomma dubitatum	*Amblyomma dubitatum*	1	3	3
Amblyomma dubitatum	*Amblyomma dubitatum*	1	3	3
Amblyomma dubitatum	*Amblyomma dubitatum*	1	3	3
Amblyomma dubitatum	*Amblyomma cajennense*	0	3	2
Amblyomma dubitatum	*Amblyomma sculptum*	0	3	5
Amblyomma ovale	*Amblyomma ovale*	1	4	4
Amblyomma ovale	*Amblyomma ovale*	1	4	4
Amblyomma ovale	*Amblyomma aureolatum*	0	4	1
Amblyomma ovale	*Amblyomma ovale*	1	4	4
Amblyomma ovale	*Amblyomma ovale*	1	4	4
Amblyomma ovale	*Amblyomma ovale*	1	4	4
Amblyomma ovale	*Amblyomma triste*	0	4	6
Amblyomma ovale	*Amblyomma ovale*	1	4	4
Amblyomma ovale	*Amblyomma ovale*	1	4	4
Amblyomma ovale	*Amblyomma ovale*	1	4	4
Amblyomma ovale	*Amblyomma sculptum*	0	4	5
Amblyomma ovale	*Amblyomma ovale*	1	4	4
Amblyomma ovale	*Amblyomma dubitatum*	0	4	3
Amblyomma sculptum	*Amblyomma sculptum*	1	5	5
Amblyomma sculptum	*Amblyomma sculptum*	1	5	5
Amblyomma sculptum	*Amblyomma sculptum*	1	5	5
Amblyomma sculptum	*Amblyomma sculptum*	1	5	5
Amblyomma sculptum	*Amblyomma sculptum*	1	5	5
Amblyomma sculptum	*Amblyomma sculptum*	1	5	5
Amblyomma sculptum	*Amblyomma cajennense*	0	5	2
Amblyomma sculptum	*Amblyomma cajennense*	0	5	2
Amblyomma sculptum	*Amblyomma sculptum*	1	5	5
Amblyomma sculptum	*Amblyomma sculptum*	1	5	5
Amblyomma sculptum	*Amblyomma sculptum*	1	5	5
Amblyomma sculptum	*Amblyomma sculptum*	1	5	5
Amblyomma sculptum	*Amblyomma sculptum*	1	5	5
Amblyomma sculptum	*Amblyomma sculptum*	1	5	5
Amblyomma sculptum	*Amblyomma sculptum*	1	5	5
Amblyomma sculptum	*Amblyomma sculptum*	1	5	5

Amblyomma sculptum	*Amblyomma sculptum*	1	5	5
Amblyomma sculptum	*Amblyomma sculptum*	1	5	5
Amblyomma sculptum	*Amblyomma dubitatum*	0	5	3
Amblyomma sculptum	*Amblyomma sculptum*	1	5	5
Amblyomma sculptum	*Amblyomma sculptum*	1	5	5
Amblyomma sculptum	*Amblyomma sculptum*	1	5	5
Amblyomma sculptum	*Amblyomma sculptum*	1	5	5
Amblyomma sculptum	*Amblyomma sculptum*	1	5	5
Amblyomma sculptum	*Amblyomma sculptum*	1	5	5
Amblyomma sculptum	*Amblyomma sculptum*	1	5	5
Amblyomma sculptum	*Amblyomma sculptum*	1	5	5
Amblyomma triste	*Amblyomma triste*	1	6	6
Amblyomma triste	*Amblyomma triste*	1	6	6
Amblyomma triste	*Amblyomma triste*	1	6	6
Amblyomma triste	*Amblyomma triste*	1	6	6
Amblyomma triste	*Amblyomma triste*	1	6	6
Amblyomma triste	*Amblyomma triste*	1	6	6
Amblyomma triste	*Amblyomma triste*	1	6	6
Amblyomma triste	*Amblyomma triste*	1	6	6
Amblyomma triste	*Amblyomma triste*	1	6	6
Amblyomma triste	*Amblyomma triste*	1	6	6
Amblyomma triste	*Amblyomma triste*	1	6	6
Amblyomma triste	*Amblyomma triste*	1	6	6
Amblyomma triste	*Amblyomma triste*	1	6	6
Amblyomma triste	*Amblyomma ovale*	0	6	4
Amblyomma triste	*Amblyomma ovale*	0	6	4
Amblyomma triste	*Amblyomma triste*	1	6	6
Amblyomma aureolatum	*Amblyomma aureolatum*	1	1	1
Amblyomma aureolatum	*Amblyomma aureolatum*	1	1	1
Amblyomma aureolatum	*Amblyomma aureolatum*	1	1	1
Amblyomma aureolatum	*Amblyomma aureolatum*	1	1	1
Amblyomma aureolatum	*Amblyomma aureolatum*	1	1	1
Amblyomma aureolatum	*Amblyomma aureolatum*	1	1	1
Amblyomma aureolatum	*Amblyomma ovale*	0	1	4
Amblyomma aureolatum	*Amblyomma triste*	0	1	6
Amblyomma aureolatum	*Amblyomma aureolatum*	1	1	1
Amblyomma aureolatum	*Amblyomma aureolatum*	1	1	1
Amblyomma aureolatum	*Amblyomma aureolatum*	1	1	1
Amblyomma aureolatum	*Amblyomma aureolatum*	1	1	1
Amblyomma aureolatum	*Amblyomma aureolatum*	1	1	1
Amblyomma aureolatum	*Amblyomma aureolatum*	1	1	1
Amblyomma cajennense	*Amblyomma cajennense*	1	2	2
Amblyomma cajennense	*Amblyomma cajennense*	1	2	2
Amblyomma cajennense	*Amblyomma cajennense*	1	2	2
Amblyomma cajennense	*Amblyomma cajennense*	1	2	2
Amblyomma cajennense	*Amblyomma cajennense*	1	2	2
Amblyomma cajennense	*Amblyomma cajennense*	1	2	2
Amblyomma cajennense	*Amblyomma cajennense*	1	2	2
Amblyomma cajennense	*Amblyomma cajennense*	1	2	2
Amblyomma cajennense	*Amblyomma cajennense*	1	2	2
Amblyomma cajennense	*Amblyomma cajennense*	1	2	2
Amblyomma cajennense	*Amblyomma cajennense*	1	2	2
Amblyomma cajennense	*Amblyomma cajennense*	1	2	2
Amblyomma cajennense	*Amblyomma cajennense*	1	2	2
Amblyomma cajennense	*Amblyomma cajennense*	1	2	2
Amblyomma cajennense	*Amblyomma cajennense*	1	2	2
Amblyomma cajennense	*Amblyomma cajennense*	1	2	2
Amblyomma cajennense	*Amblyomma cajennense*	1	2	2
Amblyomma dubitatum	*Amblyomma triste*	0	3	6
Amblyomma dubitatum	*Amblyomma dubitatum*	1	3	3
Amblyomma dubitatum	*Amblyomma dubitatum*	1	3	3
Amblyomma dubitatum	*Amblyomma dubitatum*	1	3	3
Amblyomma dubitatum	*Amblyomma dubitatum*	1	3	3
Amblyomma dubitatum	*Amblyomma dubitatum*	1	3	3
Amblyomma dubitatum	*Amblyomma dubitatum*	1	3	3
Amblyomma dubitatum	*Amblyomma dubitatum*	1	3	3
Amblyomma dubitatum	*Amblyomma dubitatum*	1	3	3

Amblyomma dubitatum	*Amblyomma dubitatum*	1	3	3
Amblyomma dubitatum	*Amblyomma aureolatum*	0	3	1
Amblyomma dubitatum	*Amblyomma dubitatum*	1	3	3
Amblyomma dubitatum	*Amblyomma dubitatum*	1	3	3
Amblyomma ovale	*Amblyomma ovale*	1	4	4
Amblyomma ovale	*Amblyomma ovale*	1	4	4
Amblyomma ovale	*Amblyomma ovale*	1	4	4
Amblyomma ovale	*Amblyomma ovale*	1	4	4
Amblyomma ovale	*Amblyomma ovale*	1	4	4
Amblyomma ovale	*Amblyomma aureolatum*	0	4	1
Amblyomma ovale	*Amblyomma sculptum*	0	4	5
Amblyomma ovale	*Amblyomma aureolatum*	0	4	1
Amblyomma ovale	*Amblyomma ovale*	1	4	4
Amblyomma ovale	*Amblyomma ovale*	1	4	4
Amblyomma ovale	*Amblyomma ovale*	1	4	4
Amblyomma ovale	*Amblyomma ovale*	1	4	4
Amblyomma ovale	*Amblyomma ovale*	1	4	4
Amblyomma sculptum	*Amblyomma sculptum*	1	5	5
Amblyomma sculptum	*Amblyomma sculptum*	1	5	5
Amblyomma sculptum	*Amblyomma sculptum*	1	5	5
Amblyomma sculptum	*Amblyomma sculptum*	1	5	5
Amblyomma sculptum	*Amblyomma sculptum*	1	5	5
Amblyomma sculptum	*Amblyomma sculptum*	1	5	5
Amblyomma sculptum	*Amblyomma sculptum*	1	5	5
Amblyomma sculptum	*Amblyomma sculptum*	1	5	5
Amblyomma sculptum	*Amblyomma sculptum*	1	5	5
Amblyomma sculptum	*Amblyomma dubitatum*	0	5	3
Amblyomma sculptum	*Amblyomma sculptum*	1	5	5
Amblyomma sculptum	*Amblyomma sculptum*	1	5	5
Amblyomma sculptum	*Amblyomma sculptum*	1	5	5
Amblyomma sculptum	*Amblyomma sculptum*	1	5	5
Amblyomma sculptum	*Amblyomma sculptum*	1	5	5
Amblyomma sculptum	*Amblyomma sculptum*	1	5	5
Amblyomma sculptum	*Amblyomma sculptum*	1	5	5
Amblyomma sculptum	*Amblyomma sculptum*	1	5	5
Amblyomma sculptum	*Amblyomma dubitatum*	0	5	3
Amblyomma sculptum	*Amblyomma sculptum*	1	5	5
Amblyomma sculptum	*Amblyomma sculptum*	1	5	5
Amblyomma sculptum	*Amblyomma sculptum*	1	5	5
Amblyomma sculptum	*Amblyomma sculptum*	1	5	5
Amblyomma sculptum	*Amblyomma sculptum*	1	5	5
Amblyomma sculptum	*Amblyomma sculptum*	1	5	5
Amblyomma sculptum	*Amblyomma sculptum*	1	5	5
Amblyomma sculptum	*Amblyomma sculptum*	1	5	5
Amblyomma sculptum	*Amblyomma sculptum*	1	5	5
Amblyomma triste	*Amblyomma triste*	1	6	6
Amblyomma triste	*Amblyomma triste*	1	6	6
Amblyomma triste	*Amblyomma triste*	1	6	6
Amblyomma triste	*Amblyomma triste*	1	6	6
Amblyomma triste	*Amblyomma triste*	1	6	6
Amblyomma triste	*Amblyomma triste*	1	6	6
Amblyomma triste	*Amblyomma triste*	1	6	6
Amblyomma triste	*Amblyomma triste*	1	6	6
Amblyomma triste	*Amblyomma triste*	1	6	6
Amblyomma triste	*Amblyomma triste*	1	6	6
Amblyomma triste	*Amblyomma triste*	1	6	6
Amblyomma triste	*Amblyomma triste*	1	6	6
Amblyomma triste	*Amblyomma triste*	1	6	6
Amblyomma triste	*Amblyomma triste*	1	6	6
Amblyomma triste	*Amblyomma triste*	1	6	6
Amblyomma triste	*Amblyomma triste*	1	6	6
Amblyomma aureolatum	*Amblyomma aureolatum*	1	1	1
Amblyomma aureolatum	*Amblyomma aureolatum*	1	1	1
Amblyomma aureolatum	*Amblyomma aureolatum*	1	1	1
Amblyomma aureolatum	*Amblyomma aureolatum*	1	1	1
Amblyomma aureolatum	*Amblyomma aureolatum*	1	1	1
Amblyomma aureolatum	*Amblyomma aureolatum*	1	1	1

Amblyomma aureolatum	*Amblyomma aureolatum*	1	1	1
Amblyomma aureolatum	*Amblyomma aureolatum*	1	1	1
Amblyomma aureolatum	*Amblyomma aureolatum*	1	1	1
Amblyomma aureolatum	*Amblyomma aureolatum*	1	1	1
Amblyomma aureolatum	*Amblyomma aureolatum*	1	1	1
Amblyomma aureolatum	*Amblyomma aureolatum*	1	1	1
Amblyomma aureolatum	*Amblyomma aureolatum*	1	1	1
Amblyomma cajennense	*Amblyomma cajennense*	1	2	2
Amblyomma cajennense	*Amblyomma cajennense*	1	2	2
Amblyomma cajennense	*Amblyomma cajennense*	1	2	2
Amblyomma cajennense	*Amblyomma cajennense*	1	2	2
Amblyomma cajennense	*Amblyomma cajennense*	1	2	2
Amblyomma cajennense	*Amblyomma cajennense*	1	2	2
Amblyomma cajennense	*Amblyomma cajennense*	1	2	2
Amblyomma cajennense	*Amblyomma cajennense*	1	2	2
Amblyomma cajennense	*Amblyomma cajennense*	1	2	2
Amblyomma cajennense	*Amblyomma cajennense*	1	2	2
Amblyomma cajennense	*Amblyomma cajennense*	1	2	2
Amblyomma cajennense	*Amblyomma cajennense*	1	2	2
Amblyomma cajennense	*Amblyomma cajennense*	1	2	2
Amblyomma cajennense	*Amblyomma cajennense*	1	2	2
Amblyomma cajennense	*Amblyomma cajennense*	1	2	2
Amblyomma cajennense	*Amblyomma cajennense*	1	2	2
Amblyomma cajennense	*Amblyomma cajennense*	1	2	2
Amblyomma dubitatum	*Amblyomma sculptum*	0	3	5
Amblyomma dubitatum	*Amblyomma ovale*	0	3	4
Amblyomma dubitatum	*Amblyomma dubitatum*	1	3	3
Amblyomma dubitatum	*Amblyomma dubitatum*	1	3	3
Amblyomma dubitatum	*Amblyomma dubitatum*	1	3	3
Amblyomma dubitatum	*Amblyomma triste*	0	3	6
Amblyomma dubitatum	*Amblyomma dubitatum*	1	3	3
Amblyomma dubitatum	*Amblyomma dubitatum*	1	3	3
Amblyomma dubitatum	*Amblyomma dubitatum*	1	3	3
Amblyomma dubitatum	*Amblyomma cajennense*	0	3	2
Amblyomma dubitatum	*Amblyomma dubitatum*	1	3	3
Amblyomma dubitatum	*Amblyomma dubitatum*	1	3	3
Amblyomma dubitatum	*Amblyomma aureolatum*	0	3	1
Amblyomma ovale	*Amblyomma ovale*	1	4	4
Amblyomma ovale	*Amblyomma ovale*	1	4	4
Amblyomma ovale	*Amblyomma ovale*	1	4	4
Amblyomma ovale	*Amblyomma cajennense*	0	4	2
Amblyomma ovale	*Amblyomma ovale*	1	4	4
Amblyomma ovale	*Amblyomma ovale*	1	4	4
Amblyomma ovale	*Amblyomma ovale*	1	4	4
Amblyomma ovale	*Amblyomma ovale*	1	4	4
Amblyomma ovale	*Amblyomma aureolatum*	0	4	1
Amblyomma ovale	*Amblyomma ovale*	1	4	4
Amblyomma ovale	*Amblyomma ovale*	1	4	4
Amblyomma ovale	*Amblyomma sculptum*	0	4	5
Amblyomma ovale	*Amblyomma ovale*	1	4	4
Amblyomma ovale	*Amblyomma ovale*	1	4	4
Amblyomma sculptum	*Amblyomma sculptum*	1	5	5
Amblyomma sculptum	*Amblyomma sculptum*	1	5	5
Amblyomma sculptum	*Amblyomma sculptum*	1	5	5
Amblyomma sculptum	*Amblyomma sculptum*	1	5	5
Amblyomma sculptum	*Amblyomma sculptum*	1	5	5
Amblyomma sculptum	*Amblyomma sculptum*	1	5	5
Amblyomma sculptum	*Amblyomma sculptum*	1	5	5
Amblyomma sculptum	*Amblyomma sculptum*	1	5	5
Amblyomma sculptum	*Amblyomma sculptum*	1	5	5
Amblyomma sculptum	*Amblyomma sculptum*	1	5	5
Amblyomma sculptum	*Amblyomma sculptum*	1	5	5
Amblyomma sculptum	*Amblyomma sculptum*	1	5	5
Amblyomma sculptum	*Amblyomma sculptum*	1	5	5
Amblyomma sculptum	*Amblyomma aureolatum*	0	5	1
Amblyomma sculptum	*Amblyomma sculptum*	1	5	5
Amblyomma sculptum	*Amblyomma sculptum*	1	5	5

Amblyomma sculptum	*Amblyomma sculptum*	1	5	5
Amblyomma sculptum	*Amblyomma sculptum*	1	5	5
Amblyomma sculptum	*Amblyomma aureolatum*	0	5	1
Amblyomma sculptum	*Amblyomma cajennense*	0	5	2
Amblyomma sculptum	*Amblyomma sculptum*	1	5	5
Amblyomma sculptum	*Amblyomma sculptum*	1	5	5
Amblyomma sculptum	*Amblyomma sculptum*	1	5	5
Amblyomma sculptum	*Amblyomma sculptum*	1	5	5
Amblyomma sculptum	*Amblyomma sculptum*	1	5	5
Amblyomma sculptum	*Amblyomma sculptum*	1	5	5
Amblyomma sculptum	*Amblyomma sculptum*	1	5	5
Amblyomma sculptum	*Amblyomma sculptum*	1	5	5
Amblyomma triste	*Amblyomma triste*	1	6	6
Amblyomma triste	*Amblyomma triste*	1	6	6
Amblyomma triste	*Amblyomma triste*	1	6	6
Amblyomma triste	*Amblyomma triste*	1	6	6
Amblyomma triste	*Amblyomma triste*	1	6	6
Amblyomma triste	*Amblyomma triste*	1	6	6
Amblyomma triste	*Amblyomma triste*	1	6	6
Amblyomma triste	*Amblyomma triste*	1	6	6
Amblyomma triste	*Amblyomma triste*	1	6	6
Amblyomma triste	*Amblyomma triste*	1	6	6
Amblyomma triste	*Amblyomma cajennense*	0	6	2
Amblyomma triste	*Amblyomma triste*	1	6	6
Amblyomma triste	*Amblyomma triste*	1	6	6
Amblyomma triste	*Amblyomma triste*	1	6	6
Amblyomma triste	*Amblyomma triste*	1	6	6
Amblyomma aureolatum	*Amblyomma aureolatum*	1	1	1
Amblyomma aureolatum	*Amblyomma aureolatum*	1	1	1
Amblyomma aureolatum	*Amblyomma aureolatum*	1	1	1
Amblyomma aureolatum	*Amblyomma aureolatum*	1	1	1
Amblyomma aureolatum	*Amblyomma aureolatum*	1	1	1
Amblyomma aureolatum	*Amblyomma aureolatum*	1	1	1
Amblyomma aureolatum	*Amblyomma aureolatum*	1	1	1
Amblyomma aureolatum	*Amblyomma aureolatum*	1	1	1
Amblyomma aureolatum	*Amblyomma aureolatum*	1	1	1
Amblyomma aureolatum	*Amblyomma aureolatum*	1	1	1
Amblyomma aureolatum	*Amblyomma aureolatum*	1	1	1
Amblyomma aureolatum	*Amblyomma aureolatum*	1	1	1
Amblyomma aureolatum	*Amblyomma aureolatum*	1	1	1
Amblyomma aureolatum	*Amblyomma aureolatum*	1	1	1
Amblyomma cajennense	*Amblyomma cajennense*	1	2	2
Amblyomma cajennense	*Amblyomma cajennense*	1	2	2
Amblyomma cajennense	*Amblyomma cajennense*	1	2	2
Amblyomma cajennense	*Amblyomma cajennense*	1	2	2
Amblyomma cajennense	*Amblyomma cajennense*	1	2	2
Amblyomma cajennense	*Amblyomma cajennense*	1	2	2
Amblyomma cajennense	*Amblyomma cajennense*	1	2	2
Amblyomma cajennense	*Amblyomma cajennense*	1	2	2
Amblyomma cajennense	*Amblyomma cajennense*	1	2	2
Amblyomma cajennense	*Amblyomma cajennense*	1	2	2
Amblyomma cajennense	*Amblyomma cajennense*	1	2	2
Amblyomma cajennense	*Amblyomma cajennense*	1	2	2
Amblyomma cajennense	*Amblyomma cajennense*	1	2	2
Amblyomma cajennense	*Amblyomma cajennense*	1	2	2
Amblyomma cajennense	*Amblyomma cajennense*	1	2	2
Amblyomma cajennense	*Amblyomma cajennense*	1	2	2
Amblyomma dubitatum	*Amblyomma dubitatum*	1	3	3
Amblyomma dubitatum	*Amblyomma dubitatum*	1	3	3
Amblyomma dubitatum	*Amblyomma dubitatum*	1	3	3
Amblyomma dubitatum	*Amblyomma dubitatum*	1	3	3
Amblyomma dubitatum	*Amblyomma dubitatum*	1	3	3
Amblyomma dubitatum	*Amblyomma dubitatum*	1	3	3
Amblyomma dubitatum	*Amblyomma dubitatum*	1	3	3
Amblyomma dubitatum	*Amblyomma aureolatum*	0	3	1
Amblyomma dubitatum	*Amblyomma dubitatum*	1	3	3
Amblyomma dubitatum	*Amblyomma dubitatum*	1	3	3

Amblyomma dubitatum	*Amblyomma aureolatum*	0	3	1
Amblyomma dubitatum	*Amblyomma dubitatum*	1	3	3
Amblyomma dubitatum	*Amblyomma dubitatum*	1	3	3
Amblyomma ovale	*Amblyomma ovale*	1	4	4
Amblyomma ovale	*Amblyomma ovale*	1	4	4
Amblyomma ovale	*Amblyomma ovale*	1	4	4
Amblyomma ovale	*Amblyomma ovale*	1	4	4
Amblyomma ovale	*Amblyomma ovale*	1	4	4
Amblyomma ovale	*Amblyomma ovale*	1	4	4
Amblyomma ovale	*Amblyomma ovale*	1	4	4
Amblyomma ovale	*Amblyomma ovale*	1	4	4
Amblyomma ovale	*Amblyomma ovale*	1	4	4
Amblyomma ovale	*Amblyomma dubitatum*	0	4	3
Amblyomma ovale	*Amblyomma ovale*	1	4	4
Amblyomma ovale	*Amblyomma ovale*	1	4	4
Amblyomma ovale	*Amblyomma ovale*	1	4	4
Amblyomma sculptum	*Amblyomma sculptum*	1	5	5
Amblyomma sculptum	*Amblyomma sculptum*	1	5	5
Amblyomma sculptum	*Amblyomma sculptum*	1	5	5
Amblyomma sculptum	*Amblyomma sculptum*	1	5	5
Amblyomma sculptum	*Amblyomma sculptum*	1	5	5
Amblyomma sculptum	*Amblyomma sculptum*	1	5	5
Amblyomma sculptum	*Amblyomma sculptum*	1	5	5
Amblyomma sculptum	*Amblyomma sculptum*	1	5	5
Amblyomma sculptum	*Amblyomma sculptum*	1	5	5
Amblyomma sculptum	*Amblyomma sculptum*	1	5	5
Amblyomma sculptum	*Amblyomma sculptum*	1	5	5
Amblyomma sculptum	*Amblyomma sculptum*	1	5	5
Amblyomma sculptum	*Amblyomma sculptum*	1	5	5
Amblyomma sculptum	*Amblyomma sculptum*	1	5	5
Amblyomma sculptum	*Amblyomma sculptum*	1	5	5
Amblyomma sculptum	*Amblyomma ovale*	0	5	4
Amblyomma sculptum	*Amblyomma sculptum*	1	5	5
Amblyomma sculptum	*Amblyomma sculptum*	1	5	5
Amblyomma sculptum	*Amblyomma sculptum*	1	5	5
Amblyomma sculptum	*Amblyomma sculptum*	1	5	5
Amblyomma sculptum	*Amblyomma sculptum*	1	5	5
Amblyomma sculptum	*Amblyomma sculptum*	1	5	5
Amblyomma sculptum	*Amblyomma sculptum*	1	5	5
Amblyomma sculptum	*Amblyomma sculptum*	1	5	5
Amblyomma sculptum	*Amblyomma sculptum*	1	5	5
Amblyomma sculptum	*Amblyomma sculptum*	1	5	5
Amblyomma sculptum	*Amblyomma sculptum*	1	5	5
Amblyomma triste	*Amblyomma triste*	1	6	6
Amblyomma triste	*Amblyomma triste*	1	6	6
Amblyomma triste	*Amblyomma triste*	1	6	6
Amblyomma triste	*Amblyomma triste*	1	6	6
Amblyomma triste	*Amblyomma triste*	1	6	6
Amblyomma triste	*Amblyomma triste*	1	6	6
Amblyomma triste	*Amblyomma triste*	1	6	6
Amblyomma triste	*Amblyomma triste*	1	6	6
Amblyomma triste	*Amblyomma triste*	1	6	6
Amblyomma triste	*Amblyomma triste*	1	6	6
Amblyomma triste	*Amblyomma triste*	1	6	6
Amblyomma triste	*Amblyomma triste*	1	6	6
Amblyomma triste	*Amblyomma triste*	1	6	6
Amblyomma triste	*Amblyomma triste*	1	6	6
Amblyomma triste	*Amblyomma triste*	1	6	6
Amblyomma triste	*Amblyomma triste*	1	6	6
Amblyomma aureolatum	*Amblyomma cajennense*	0	1	2
Amblyomma aureolatum	*Amblyomma ovale*	0	1	4
Amblyomma aureolatum	*Amblyomma aureolatum*	1	1	1
Amblyomma aureolatum	*Amblyomma aureolatum*	1	1	1
Amblyomma aureolatum	*Amblyomma aureolatum*	1	1	1
Amblyomma aureolatum	*Amblyomma aureolatum*	1	1	1
Amblyomma aureolatum	*Amblyomma aureolatum*	1	1	1
Amblyomma aureolatum	*Amblyomma aureolatum*	1	1	1

Amblyomma aureolatum	*Amblyomma aureolatum*	1	1	1
Amblyomma aureolatum	*Amblyomma aureolatum*	1	1	1
Amblyomma aureolatum	*Amblyomma aureolatum*	1	1	1
Amblyomma aureolatum	*Amblyomma aureolatum*	1	1	1
Amblyomma aureolatum	*Amblyomma aureolatum*	1	1	1
Amblyomma aureolatum	*Amblyomma ovale*	0	1	4
Amblyomma cajennense	*Amblyomma cajennense*	1	2	2
Amblyomma cajennense	*Amblyomma cajennense*	1	2	2
Amblyomma cajennense	*Amblyomma cajennense*	1	2	2
Amblyomma cajennense	*Amblyomma cajennense*	1	2	2
Amblyomma cajennense	*Amblyomma cajennense*	1	2	2
Amblyomma cajennense	*Amblyomma cajennense*	1	2	2
Amblyomma cajennense	*Amblyomma cajennense*	1	2	2
Amblyomma cajennense	*Amblyomma cajennense*	1	2	2
Amblyomma cajennense	*Amblyomma cajennense*	1	2	2
Amblyomma cajennense	*Amblyomma cajennense*	1	2	2
Amblyomma cajennense	*Amblyomma cajennense*	1	2	2
Amblyomma cajennense	*Amblyomma cajennense*	1	2	2
Amblyomma cajennense	*Amblyomma cajennense*	1	2	2
Amblyomma cajennense	*Amblyomma cajennense*	1	2	2
Amblyomma cajennense	*Amblyomma cajennense*	1	2	2
Amblyomma cajennense	*Amblyomma cajennense*	1	2	2
Amblyomma dubitatum	*Amblyomma aureolatum*	0	3	1
Amblyomma dubitatum	*Amblyomma dubitatum*	1	3	3
Amblyomma dubitatum	*Amblyomma dubitatum*	1	3	3
Amblyomma dubitatum	*Amblyomma dubitatum*	1	3	3
Amblyomma dubitatum	*Amblyomma dubitatum*	1	3	3
Amblyomma dubitatum	*Amblyomma dubitatum*	1	3	3
Amblyomma dubitatum	*Amblyomma aureolatum*	0	3	1
Amblyomma dubitatum	*Amblyomma dubitatum*	1	3	3
Amblyomma dubitatum	*Amblyomma dubitatum*	1	3	3
Amblyomma dubitatum	*Amblyomma dubitatum*	1	3	3
Amblyomma dubitatum	*Amblyomma aureolatum*	0	3	1
Amblyomma dubitatum	*Amblyomma sculptum*	0	3	5
Amblyomma dubitatum	*Amblyomma sculptum*	0	3	5
Amblyomma ovale	*Amblyomma ovale*	1	4	4
Amblyomma ovale	*Amblyomma ovale*	1	4	4
Amblyomma ovale	*Amblyomma ovale*	1	4	4
Amblyomma ovale	*Amblyomma ovale*	1	4	4
Amblyomma ovale	*Amblyomma ovale*	1	4	4
Amblyomma ovale	*Amblyomma ovale*	1	4	4
Amblyomma ovale	*Amblyomma sculptum*	0	4	5
Amblyomma ovale	*Amblyomma aureolatum*	0	4	1
Amblyomma ovale	*Amblyomma ovale*	1	4	4
Amblyomma ovale	*Amblyomma ovale*	1	4	4
Amblyomma ovale	*Amblyomma ovale*	1	4	4
Amblyomma ovale	*Amblyomma ovale*	1	4	4
Amblyomma ovale	*Amblyomma ovale*	1	4	4
Amblyomma sculptum	*Amblyomma sculptum*	1	5	5
Amblyomma sculptum	*Amblyomma sculptum*	1	5	5
Amblyomma sculptum	*Amblyomma sculptum*	1	5	5
Amblyomma sculptum	*Amblyomma sculptum*	1	5	5
Amblyomma sculptum	*Amblyomma sculptum*	1	5	5
Amblyomma sculptum	*Amblyomma sculptum*	1	5	5
Amblyomma sculptum	*Amblyomma sculptum*	1	5	5
Amblyomma sculptum	*Amblyomma sculptum*	1	5	5
Amblyomma sculptum	*Amblyomma sculptum*	1	5	5
Amblyomma sculptum	*Amblyomma sculptum*	1	5	5
Amblyomma sculptum	*Amblyomma sculptum*	1	5	5
Amblyomma sculptum	*Amblyomma sculptum*	1	5	5
Amblyomma sculptum	*Amblyomma sculptum*	1	5	5
Amblyomma sculptum	*Amblyomma sculptum*	1	5	5
Amblyomma sculptum	*Amblyomma sculptum*	1	5	5
Amblyomma sculptum	*Amblyomma sculptum*	1	5	5
Amblyomma sculptum	*Amblyomma sculptum*	1	5	5
Amblyomma sculptum	*Amblyomma sculptum*	1	5	5
Amblyomma sculptum	*Amblyomma sculptum*	1	5	5

Amblyomma sculptum	*Amblyomma sculptum*	1	5	5
Amblyomma sculptum	*Amblyomma sculptum*	1	5	5
Amblyomma sculptum	*Amblyomma sculptum*	1	5	5
Amblyomma sculptum	*Amblyomma sculptum*	1	5	5
Amblyomma sculptum	*Amblyomma sculptum*	1	5	5
Amblyomma sculptum	*Amblyomma sculptum*	1	5	5
Amblyomma sculptum	*Amblyomma sculptum*	1	5	5
Amblyomma sculptum	*Amblyomma sculptum*	1	5	5
Amblyomma sculptum	*Amblyomma sculptum*	1	5	5
Amblyomma triste	*Amblyomma triste*	1	6	6
Amblyomma triste	*Amblyomma triste*	1	6	6
Amblyomma triste	*Amblyomma triste*	1	6	6
Amblyomma triste	*Amblyomma triste*	1	6	6
Amblyomma triste	*Amblyomma aureolatum*	0	6	1
Amblyomma triste	*Amblyomma triste*	1	6	6
Amblyomma triste	*Amblyomma triste*	1	6	6
Amblyomma triste	*Amblyomma triste*	1	6	6
Amblyomma triste	*Amblyomma triste*	1	6	6
Amblyomma triste	*Amblyomma triste*	1	6	6
Amblyomma triste	*Amblyomma triste*	1	6	6
Amblyomma triste	*Amblyomma triste*	1	6	6
Amblyomma triste	*Amblyomma triste*	1	6	6
Amblyomma triste	*Amblyomma triste*	1	6	6
Amblyomma triste	*Amblyomma triste*	1	6	6
Amblyomma triste	*Amblyomma triste*	1	6	6
Amblyomma aureolatum	*Amblyomma aureolatum*	1	1	1
Amblyomma aureolatum	*Amblyomma aureolatum*	1	1	1
Amblyomma aureolatum	*Amblyomma triste*	0	1	6
Amblyomma aureolatum	*Amblyomma aureolatum*	1	1	1
Amblyomma aureolatum	*Amblyomma aureolatum*	1	1	1
Amblyomma aureolatum	*Amblyomma aureolatum*	1	1	1
Amblyomma aureolatum	*Amblyomma aureolatum*	1	1	1
Amblyomma aureolatum	*Amblyomma aureolatum*	1	1	1
Amblyomma aureolatum	*Amblyomma aureolatum*	1	1	1
Amblyomma aureolatum	*Amblyomma aureolatum*	1	1	1
Amblyomma aureolatum	*Amblyomma aureolatum*	1	1	1
Amblyomma aureolatum	*Amblyomma aureolatum*	1	1	1
Amblyomma aureolatum	*Amblyomma aureolatum*	1	1	1
Amblyomma aureolatum	*Amblyomma aureolatum*	1	1	1
Amblyomma aureolatum	*Amblyomma aureolatum*	1	1	1
Amblyomma aureolatum	*Amblyomma aureolatum*	1	1	1
Amblyomma aureolatum	*Amblyomma aureolatum*	1	1	1
Amblyomma aureolatum	*Amblyomma aureolatum*	1	1	1
Amblyomma aureolatum	*Amblyomma cajennense*	0	1	2
Amblyomma aureolatum	*Amblyomma aureolatum*	1	1	1
Amblyomma aureolatum	*Amblyomma aureolatum*	1	1	1
Amblyomma aureolatum	*Amblyomma aureolatum*	1	1	1
Amblyomma aureolatum	*Amblyomma aureolatum*	1	1	1
Amblyomma aureolatum	*Amblyomma aureolatum*	1	1	1
Amblyomma cajennense	*Amblyomma cajennense*	1	2	2
Amblyomma cajennense	*Amblyomma cajennense*	1	2	2
Amblyomma cajennense	*Amblyomma cajennense*	1	2	2
Amblyomma cajennense	*Amblyomma cajennense*	1	2	2
Amblyomma cajennense	*Amblyomma cajennense*	1	2	2
Amblyomma cajennense	*Amblyomma cajennense*	1	2	2
Amblyomma cajennense	*Amblyomma cajennense*	1	2	2
Amblyomma cajennense	*Amblyomma cajennense*	1	2	2
Amblyomma cajennense	*Amblyomma cajennense*	1	2	2
Amblyomma cajennense	*Amblyomma triste*	0	2	6
Amblyomma cajennense	*Amblyomma cajennense*	1	2	2
Amblyomma cajennense	*Amblyomma cajennense*	1	2	2
Amblyomma cajennense	*Amblyomma cajennense*	1	2	2
Amblyomma cajennense	*Amblyomma cajennense*	1	2	2
Amblyomma cajennense	*Amblyomma cajennense*	1	2	2
Amblyomma cajennense	*Amblyomma cajennense*	1	2	2
Amblyomma cajennense	*Amblyomma cajennense*	1	2	2
Amblyomma cajennense	*Amblyomma cajennense*	1	2	2

Amblyomma cajennense	*Amblyomma cajennense*	1	2	2
Amblyomma cajennense	*Amblyomma cajennense*	1	2	2
Amblyomma cajennense	*Amblyomma cajennense*	1	2	2
Amblyomma cajennense	*Amblyomma cajennense*	1	2	2
Amblyomma cajennense	*Amblyomma cajennense*	1	2	2
Amblyomma cajennense	*Amblyomma cajennense*	1	2	2
Amblyomma cajennense	*Amblyomma cajennense*	1	2	2
Amblyomma cajennense	*Amblyomma cajennense*	1	2	2
Amblyomma cajennense	*Amblyomma cajennense*	1	2	2
Amblyomma cajennense	*Amblyomma cajennense*	1	2	2
Amblyomma cajennense	*Amblyomma cajennense*	1	2	2
Amblyomma cajennense	*Amblyomma cajennense*	1	2	2
Amblyomma cajennense	*Amblyomma cajennense*	1	2	2
Amblyomma cajennense	*Amblyomma cajennense*	1	2	2
Amblyomma cajennense	*Amblyomma cajennense*	1	2	2
Amblyomma dubitatum	*Amblyomma dubitatum*	1	3	3
Amblyomma dubitatum	*Amblyomma dubitatum*	1	3	3
Amblyomma dubitatum	*Amblyomma dubitatum*	1	3	3
Amblyomma dubitatum	*Amblyomma aureolatum*	0	3	1
Amblyomma dubitatum	*Amblyomma dubitatum*	1	3	3
Amblyomma dubitatum	*Amblyomma dubitatum*	1	3	3
Amblyomma dubitatum	*Amblyomma dubitatum*	1	3	3
Amblyomma dubitatum	*Amblyomma sculptum*	0	3	5
Amblyomma dubitatum	*Amblyomma dubitatum*	1	3	3
Amblyomma dubitatum	*Amblyomma dubitatum*	1	3	3
Amblyomma dubitatum	*Amblyomma dubitatum*	1	3	3
Amblyomma dubitatum	*Amblyomma dubitatum*	1	3	3
Amblyomma dubitatum	*Amblyomma dubitatum*	1	3	3
Amblyomma dubitatum	*Amblyomma dubitatum*	1	3	3
Amblyomma dubitatum	*Amblyomma dubitatum*	1	3	3
Amblyomma dubitatum	*Amblyomma dubitatum*	1	3	3
Amblyomma dubitatum	*Amblyomma sculptum*	0	3	5
Amblyomma dubitatum	*Amblyomma dubitatum*	1	3	3
Amblyomma dubitatum	*Amblyomma dubitatum*	1	3	3
Amblyomma dubitatum	*Amblyomma dubitatum*	1	3	3
Amblyomma dubitatum	*Amblyomma dubitatum*	1	3	3
Amblyomma dubitatum	*Amblyomma dubitatum*	1	3	3
Amblyomma ovale	*Amblyomma ovale*	1	4	4
Amblyomma ovale	*Amblyomma triste*	0	4	6
Amblyomma ovale	*Amblyomma sculptum*	0	4	5
Amblyomma ovale	*Amblyomma aureolatum*	0	4	1
Amblyomma ovale	*Amblyomma aureolatum*	0	4	1
Amblyomma ovale	*Amblyomma ovale*	1	4	4
Amblyomma ovale	*Amblyomma aureolatum*	0	4	1
Amblyomma ovale	*Amblyomma dubitatum*	0	4	3
Amblyomma ovale	*Amblyomma ovale*	1	4	4
Amblyomma ovale	*Amblyomma ovale*	1	4	4
Amblyomma ovale	*Amblyomma ovale*	1	4	4
Amblyomma ovale	*Amblyomma ovale*	1	4	4
Amblyomma ovale	*Amblyomma ovale*	1	4	4
Amblyomma ovale	*Amblyomma ovale*	1	4	4
Amblyomma ovale	*Amblyomma ovale*	1	4	4
Amblyomma ovale	*Amblyomma ovale*	1	4	4
Amblyomma ovale	*Amblyomma ovale*	1	4	4
Amblyomma ovale	*Amblyomma ovale*	1	4	4
Amblyomma ovale	*Amblyomma ovale*	1	4	4
Amblyomma ovale	*Amblyomma ovale*	1	4	4
Amblyomma ovale	*Amblyomma ovale*	1	4	4
Amblyomma ovale	*Amblyomma ovale*	1	4	4
Amblyomma ovale	*Amblyomma ovale*	1	4	4
Amblyomma ovale	*Amblyomma ovale*	1	4	4
Amblyomma sculptum	*Amblyomma sculptum*	1	5	5
Amblyomma sculptum	*Amblyomma sculptum*	1	5	5
Amblyomma sculptum	*Amblyomma sculptum*	1	5	5
Amblyomma sculptum	*Amblyomma sculptum*	1	5	5
Amblyomma sculptum	*Amblyomma sculptum*	1	5	5
Amblyomma sculptum	*Amblyomma sculptum*	1	5	5

Amblyomma sculptum	*Amblyomma sculptum*	1	5	5
Amblyomma sculptum	*Amblyomma sculptum*	1	5	5
Amblyomma sculptum	*Amblyomma sculptum*	1	5	5
Amblyomma sculptum	*Amblyomma sculptum*	1	5	5
Amblyomma sculptum	*Amblyomma sculptum*	1	5	5
Amblyomma sculptum	*Amblyomma sculptum*	1	5	5
Amblyomma sculptum	*Amblyomma sculptum*	1	5	5
Amblyomma sculptum	*Amblyomma sculptum*	1	5	5
Amblyomma sculptum	*Amblyomma sculptum*	1	5	5
Amblyomma sculptum	*Amblyomma sculptum*	1	5	5
Amblyomma sculptum	*Amblyomma sculptum*	1	5	5
Amblyomma sculptum	*Amblyomma dubitatum*	0	5	3
Amblyomma sculptum	*Amblyomma sculptum*	1	5	5
Amblyomma sculptum	*Amblyomma sculptum*	1	5	5
Amblyomma sculptum	*Amblyomma sculptum*	1	5	5
Amblyomma sculptum	*Amblyomma sculptum*	1	5	5
Amblyomma sculptum	*Amblyomma sculptum*	1	5	5
Amblyomma sculptum	*Amblyomma sculptum*	1	5	5
Amblyomma sculptum	*Amblyomma sculptum*	1	5	5
Amblyomma sculptum	*Amblyomma sculptum*	1	5	5
Amblyomma sculptum	*Amblyomma sculptum*	1	5	5
Amblyomma sculptum	*Amblyomma sculptum*	1	5	5
Amblyomma sculptum	*Amblyomma sculptum*	1	5	5
Amblyomma sculptum	*Amblyomma sculptum*	1	5	5
Amblyomma sculptum	*Amblyomma sculptum*	1	5	5
Amblyomma triste	*Amblyomma triste*	1	6	6
Amblyomma triste	*Amblyomma triste*	1	6	6
Amblyomma triste	*Amblyomma triste*	1	6	6
Amblyomma triste	*Amblyomma triste*	1	6	6
Amblyomma triste	*Amblyomma triste*	1	6	6
Amblyomma triste	*Amblyomma triste*	1	6	6
Amblyomma triste	*Amblyomma triste*	1	6	6
Amblyomma triste	*Amblyomma triste*	1	6	6
Amblyomma triste	*Amblyomma triste*	1	6	6
Amblyomma triste	*Amblyomma triste*	1	6	6
Amblyomma triste	*Amblyomma triste*	1	6	6
Amblyomma triste	*Amblyomma triste*	1	6	6
Amblyomma triste	*Amblyomma triste*	1	6	6
Amblyomma triste	*Amblyomma triste*	1	6	6
Amblyomma triste	*Amblyomma triste*	1	6	6
Amblyomma triste	*Amblyomma triste*	1	6	6
Amblyomma triste	*Amblyomma triste*	1	6	6
Amblyomma triste	*Amblyomma triste*	1	6	6
Amblyomma triste	*Amblyomma triste*	1	6	6
Amblyomma triste	*Amblyomma triste*	1	6	6
Amblyomma triste	*Amblyomma triste*	1	6	6
Amblyomma triste	*Amblyomma triste*	1	6	6
Amblyomma triste	*Amblyomma triste*	1	6	6
Amblyomma triste	*Amblyomma triste*	1	6	6
Amblyomma triste	*Amblyomma triste*	1	6	6
Amblyomma triste	*Amblyomma triste*	1	6	6
Amblyomma triste	*Amblyomma triste*	1	6	6
Amblyomma triste	*Amblyomma triste*	1	6	6
Amblyomma triste	*Amblyomma triste*	1	6	6
Amblyomma triste	*Amblyomma triste*	1	6	6
Amblyomma triste	*Amblyomma triste*	1	6	6
Amblyomma aureolatum	*Amblyomma aureolatum*	1	1	1
Amblyomma aureolatum	*Amblyomma aureolatum*	1	1	1
Amblyomma aureolatum	*Amblyomma aureolatum*	1	1	1
Amblyomma aureolatum	*Amblyomma aureolatum*	1	1	1
Amblyomma aureolatum	*Amblyomma aureolatum*	1	1	1
Amblyomma aureolatum	*Amblyomma aureolatum*	1	1	1
Amblyomma aureolatum	*Amblyomma aureolatum*	1	1	1
Amblyomma aureolatum	*Amblyomma aureolatum*	1	1	1
Amblyomma aureolatum	*Amblyomma aureolatum*	1	1	1
Amblyomma aureolatum	*Amblyomma aureolatum*	1	1	1
Amblyomma aureolatum	*Amblyomma aureolatum*	1	1	1

Amblyomma aureolatum	*Amblyomma aureolatum*	1	1	1
Amblyomma aureolatum	*Amblyomma aureolatum*	1	1	1
Amblyomma aureolatum	*Amblyomma aureolatum*	1	1	1
Amblyomma aureolatum	*Amblyomma aureolatum*	1	1	1
Amblyomma aureolatum	*Amblyomma aureolatum*	1	1	1
Amblyomma aureolatum	*Amblyomma aureolatum*	1	1	1
Amblyomma aureolatum	*Amblyomma aureolatum*	1	1	1
Amblyomma aureolatum	*Amblyomma aureolatum*	1	1	1
Amblyomma aureolatum	*Amblyomma aureolatum*	1	1	1
Amblyomma aureolatum	*Amblyomma aureolatum*	1	1	1
Amblyomma aureolatum	*Amblyomma aureolatum*	1	1	1
Amblyomma aureolatum	*Amblyomma aureolatum*	1	1	1
Amblyomma aureolatum	*Amblyomma aureolatum*	1	1	1
Amblyomma cajennense	*Amblyomma cajennense*	1	2	2
Amblyomma cajennense	*Amblyomma cajennense*	1	2	2
Amblyomma cajennense	*Amblyomma cajennense*	1	2	2
Amblyomma cajennense	*Amblyomma cajennense*	1	2	2
Amblyomma cajennense	*Amblyomma cajennense*	1	2	2
Amblyomma cajennense	*Amblyomma cajennense*	1	2	2
Amblyomma cajennense	*Amblyomma cajennense*	1	2	2
Amblyomma cajennense	*Amblyomma cajennense*	1	2	2
Amblyomma cajennense	*Amblyomma cajennense*	1	2	2
Amblyomma cajennense	*Amblyomma cajennense*	1	2	2
Amblyomma cajennense	*Amblyomma cajennense*	1	2	2
Amblyomma cajennense	*Amblyomma cajennense*	1	2	2
Amblyomma cajennense	*Amblyomma cajennense*	1	2	2
Amblyomma cajennense	*Amblyomma cajennense*	1	2	2
Amblyomma cajennense	*Amblyomma cajennense*	1	2	2
Amblyomma cajennense	*Amblyomma cajennense*	1	2	2
Amblyomma cajennense	*Amblyomma cajennense*	1	2	2
Amblyomma cajennense	*Amblyomma cajennense*	1	2	2
Amblyomma cajennense	*Amblyomma cajennense*	1	2	2
Amblyomma cajennense	*Amblyomma cajennense*	1	2	2
Amblyomma cajennense	*Amblyomma cajennense*	1	2	2
Amblyomma cajennense	*Amblyomma cajennense*	1	2	2
Amblyomma cajennense	*Amblyomma cajennense*	1	2	2
Amblyomma cajennense	*Amblyomma cajennense*	1	2	2
Amblyomma cajennense	*Amblyomma cajennense*	1	2	2
Amblyomma cajennense	*Amblyomma cajennense*	1	2	2
Amblyomma cajennense	*Amblyomma cajennense*	1	2	2
Amblyomma cajennense	*Amblyomma cajennense*	1	2	2
Amblyomma cajennense	*Amblyomma cajennense*	1	2	2
Amblyomma cajennense	*Amblyomma cajennense*	1	2	2
Amblyomma cajennense	*Amblyomma cajennense*	1	2	2
Amblyomma cajennense	*Amblyomma cajennense*	1	2	2
Amblyomma cajennense	*Amblyomma cajennense*	1	2	2
Amblyomma dubitatum	*Amblyomma triste*	0	3	6
Amblyomma dubitatum	*Amblyomma dubitatum*	1	3	3
Amblyomma dubitatum	*Amblyomma dubitatum*	1	3	3
Amblyomma dubitatum	*Amblyomma aureolatum*	0	3	1
Amblyomma dubitatum	*Amblyomma dubitatum*	1	3	3
Amblyomma dubitatum	*Amblyomma dubitatum*	1	3	3
Amblyomma dubitatum	*Amblyomma dubitatum*	1	3	3
Amblyomma dubitatum	*Amblyomma dubitatum*	1	3	3
Amblyomma dubitatum	*Amblyomma dubitatum*	1	3	3
Amblyomma dubitatum	*Amblyomma dubitatum*	1	3	3
Amblyomma dubitatum	*Amblyomma dubitatum*	1	3	3
Amblyomma dubitatum	*Amblyomma dubitatum*	1	3	3
Amblyomma dubitatum	*Amblyomma dubitatum*	1	3	3
Amblyomma dubitatum	*Amblyomma dubitatum*	1	3	3
Amblyomma dubitatum	*Amblyomma dubitatum*	1	3	3
Amblyomma dubitatum	*Amblyomma dubitatum*	1	3	3
Amblyomma dubitatum	*Amblyomma sculptum*	0	3	5
Amblyomma dubitatum	*Amblyomma dubitatum*	1	3	3
Amblyomma dubitatum	*Amblyomma dubitatum*	1	3	3
Amblyomma dubitatum	*Amblyomma dubitatum*	1	3	3
Amblyomma dubitatum	*Amblyomma dubitatum*	1	3	3

Amblyomma dubitatum	*Amblyomma dubitatum*	1	3	3
Amblyomma ovale	*Amblyomma ovale*	1	4	4
Amblyomma ovale	*Amblyomma ovale*	1	4	4
Amblyomma ovale	*Amblyomma dubitatum*	0	4	3
Amblyomma ovale	*Amblyomma ovale*	1	4	4
Amblyomma ovale	*Amblyomma aureolatum*	0	4	1
Amblyomma ovale	*Amblyomma ovale*	1	4	4
Amblyomma ovale	*Amblyomma ovale*	1	4	4
Amblyomma ovale	*Amblyomma ovale*	1	4	4
Amblyomma ovale	*Amblyomma ovale*	1	4	4
Amblyomma ovale	*Amblyomma ovale*	1	4	4
Amblyomma ovale	*Amblyomma ovale*	1	4	4
Amblyomma ovale	*Amblyomma ovale*	1	4	4
Amblyomma ovale	*Amblyomma ovale*	1	4	4
Amblyomma ovale	*Amblyomma ovale*	1	4	4
Amblyomma ovale	*Amblyomma ovale*	1	4	4
Amblyomma ovale	*Amblyomma ovale*	1	4	4
Amblyomma ovale	*Amblyomma ovale*	1	4	4
Amblyomma ovale	*Amblyomma cajennense*	0	4	2
Amblyomma ovale	*Amblyomma ovale*	1	4	4
Amblyomma ovale	*Amblyomma aureolatum*	0	4	1
Amblyomma ovale	*Amblyomma ovale*	1	4	4
Amblyomma ovale	*Amblyomma ovale*	1	4	4
Amblyomma ovale	*Amblyomma ovale*	1	4	4
Amblyomma ovale	*Amblyomma ovale*	1	4	4
Amblyomma sculptum	*Amblyomma sculptum*	1	5	5
Amblyomma sculptum	*Amblyomma sculptum*	1	5	5
Amblyomma sculptum	*Amblyomma sculptum*	1	5	5
Amblyomma sculptum	*Amblyomma sculptum*	1	5	5
Amblyomma sculptum	*Amblyomma sculptum*	1	5	5
Amblyomma sculptum	*Amblyomma sculptum*	1	5	5
Amblyomma sculptum	*Amblyomma sculptum*	1	5	5
Amblyomma sculptum	*Amblyomma sculptum*	1	5	5
Amblyomma sculptum	*Amblyomma sculptum*	1	5	5
Amblyomma sculptum	*Amblyomma sculptum*	1	5	5
Amblyomma sculptum	*Amblyomma sculptum*	1	5	5
Amblyomma sculptum	*Amblyomma sculptum*	1	5	5
Amblyomma sculptum	*Amblyomma sculptum*	1	5	5
Amblyomma sculptum	*Amblyomma sculptum*	1	5	5
Amblyomma sculptum	*Amblyomma sculptum*	1	5	5
Amblyomma sculptum	*Amblyomma sculptum*	1	5	5
Amblyomma sculptum	*Amblyomma sculptum*	1	5	5
Amblyomma sculptum	*Amblyomma sculptum*	1	5	5
Amblyomma sculptum	*Amblyomma sculptum*	1	5	5
Amblyomma sculptum	*Amblyomma sculptum*	1	5	5
Amblyomma sculptum	*Amblyomma sculptum*	1	5	5
Amblyomma sculptum	*Amblyomma triste*	0	5	6
Amblyomma sculptum	*Amblyomma sculptum*	1	5	5
Amblyomma sculptum	*Amblyomma sculptum*	1	5	5
Amblyomma sculptum	*Amblyomma sculptum*	1	5	5
Amblyomma sculptum	*Amblyomma sculptum*	1	5	5
Amblyomma sculptum	*Amblyomma sculptum*	1	5	5
Amblyomma sculptum	*Amblyomma sculptum*	1	5	5
Amblyomma sculptum	*Amblyomma sculptum*	1	5	5
Amblyomma sculptum	*Amblyomma sculptum*	1	5	5
Amblyomma sculptum	*Amblyomma sculptum*	1	5	5
Amblyomma triste	*Amblyomma triste*	1	6	6
Amblyomma triste	*Amblyomma triste*	1	6	6
Amblyomma triste	*Amblyomma triste*	1	6	6
Amblyomma triste	*Amblyomma triste*	1	6	6
Amblyomma triste	*Amblyomma triste*	1	6	6
Amblyomma triste	*Amblyomma triste*	1	6	6
Amblyomma triste	*Amblyomma triste*	1	6	6
Amblyomma triste	*Amblyomma triste*	1	6	6
Amblyomma triste	*Amblyomma triste*	1	6	6
Amblyomma triste	*Amblyomma triste*	1	6	6
Amblyomma triste	*Amblyomma triste*	1	6	6

Amblyomma triste	*Amblyomma triste*	1	6	6
Amblyomma triste	*Amblyomma triste*	1	6	6
Amblyomma triste	*Amblyomma triste*	1	6	6
Amblyomma triste	*Amblyomma triste*	1	6	6
Amblyomma triste	*Amblyomma triste*	1	6	6
Amblyomma triste	*Amblyomma triste*	1	6	6
Amblyomma triste	*Amblyomma triste*	1	6	6
Amblyomma triste	*Amblyomma triste*	1	6	6
Amblyomma triste	*Amblyomma triste*	1	6	6
Amblyomma triste	*Amblyomma aureolatum*	0	6	1
Amblyomma triste	*Amblyomma triste*	1	6	6
Amblyomma triste	*Amblyomma cajennense*	0	6	2
Amblyomma triste	*Amblyomma triste*	1	6	6
Amblyomma triste	*Amblyomma triste*	1	6	6
Amblyomma triste	*Amblyomma triste*	1	6	6
Amblyomma triste	*Amblyomma triste*	1	6	6
Amblyomma triste	*Amblyomma dubitatum*	0	6	3
Amblyomma triste	*Amblyomma triste*	1	6	6
Amblyomma triste	*Amblyomma triste*	1	6	6
Amblyomma triste	*Amblyomma triste*	1	6	6
Amblyomma aureolatum	*Amblyomma aureolatum*	1	1	1
Amblyomma aureolatum	*Amblyomma aureolatum*	1	1	1
Amblyomma aureolatum	*Amblyomma aureolatum*	1	1	1
Amblyomma aureolatum	*Amblyomma aureolatum*	1	1	1
Amblyomma aureolatum	*Amblyomma aureolatum*	1	1	1
Amblyomma aureolatum	*Amblyomma aureolatum*	1	1	1
Amblyomma aureolatum	*Amblyomma aureolatum*	1	1	1
Amblyomma aureolatum	*Amblyomma aureolatum*	1	1	1
Amblyomma aureolatum	*Amblyomma aureolatum*	1	1	1
Amblyomma aureolatum	*Amblyomma aureolatum*	1	1	1
Amblyomma aureolatum	*Amblyomma cajennense*	0	1	2
Amblyomma aureolatum	*Amblyomma aureolatum*	1	1	1
Amblyomma aureolatum	*Amblyomma aureolatum*	1	1	1
Amblyomma aureolatum	*Amblyomma aureolatum*	1	1	1
Amblyomma aureolatum	*Amblyomma aureolatum*	1	1	1
Amblyomma aureolatum	*Amblyomma aureolatum*	1	1	1
Amblyomma aureolatum	*Amblyomma aureolatum*	1	1	1
Amblyomma aureolatum	*Amblyomma aureolatum*	1	1	1
Amblyomma aureolatum	*Amblyomma aureolatum*	1	1	1
Amblyomma aureolatum	*Amblyomma aureolatum*	1	1	1
Amblyomma aureolatum	*Amblyomma aureolatum*	1	1	1
Amblyomma aureolatum	*Amblyomma aureolatum*	1	1	1
Amblyomma aureolatum	*Amblyomma aureolatum*	1	1	1
Amblyomma aureolatum	*Amblyomma aureolatum*	1	1	1
Amblyomma cajennense	*Amblyomma cajennense*	1	2	2
Amblyomma cajennense	*Amblyomma cajennense*	1	2	2
Amblyomma cajennense	*Amblyomma triste*	0	2	6
Amblyomma cajennense	*Amblyomma cajennense*	1	2	2
Amblyomma cajennense	*Amblyomma cajennense*	1	2	2
Amblyomma cajennense	*Amblyomma cajennense*	1	2	2
Amblyomma cajennense	*Amblyomma cajennense*	1	2	2
Amblyomma cajennense	*Amblyomma cajennense*	1	2	2
Amblyomma cajennense	*Amblyomma cajennense*	1	2	2
Amblyomma cajennense	*Amblyomma cajennense*	1	2	2
Amblyomma cajennense	*Amblyomma cajennense*	1	2	2
Amblyomma cajennense	*Amblyomma cajennense*	1	2	2
Amblyomma cajennense	*Amblyomma cajennense*	1	2	2
Amblyomma cajennense	*Amblyomma cajennense*	1	2	2
Amblyomma cajennense	*Amblyomma cajennense*	1	2	2
Amblyomma cajennense	*Amblyomma cajennense*	1	2	2
Amblyomma cajennense	*Amblyomma cajennense*	1	2	2
Amblyomma cajennense	*Amblyomma cajennense*	1	2	2
Amblyomma cajennense	*Amblyomma cajennense*	1	2	2
Amblyomma cajennense	*Amblyomma cajennense*	1	2	2
Amblyomma cajennense	*Amblyomma cajennense*	1	2	2
Amblyomma cajennense	*Amblyomma cajennense*	1	2	2
Amblyomma cajennense	*Amblyomma cajennense*	1	2	2

Amblyomma cajennense	*Amblyomma cajennense*	1	2	2
Amblyomma cajennense	*Amblyomma cajennense*	1	2	2
Amblyomma cajennense	*Amblyomma cajennense*	1	2	2
Amblyomma cajennense	*Amblyomma cajennense*	1	2	2
Amblyomma cajennense	*Amblyomma cajennense*	1	2	2
Amblyomma cajennense	*Amblyomma cajennense*	1	2	2
Amblyomma cajennense	*Amblyomma cajennense*	1	2	2
Amblyomma cajennense	*Amblyomma cajennense*	1	2	2
Amblyomma cajennense	*Amblyomma cajennense*	1	2	2
Amblyomma cajennense	*Amblyomma cajennense*	1	2	2
Amblyomma dubitatum	*Amblyomma dubitatum*	1	3	3
Amblyomma dubitatum	*Amblyomma dubitatum*	1	3	3
Amblyomma dubitatum	*Amblyomma sculptum*	0	3	5
Amblyomma dubitatum	*Amblyomma dubitatum*	1	3	3
Amblyomma dubitatum	*Amblyomma dubitatum*	1	3	3
Amblyomma dubitatum	*Amblyomma dubitatum*	1	3	3
Amblyomma dubitatum	*Amblyomma aureolatum*	0	3	1
Amblyomma dubitatum	*Amblyomma aureolatum*	0	3	1
Amblyomma dubitatum	*Amblyomma dubitatum*	1	3	3
Amblyomma dubitatum	*Amblyomma dubitatum*	1	3	3
Amblyomma dubitatum	*Amblyomma dubitatum*	1	3	3
Amblyomma dubitatum	*Amblyomma dubitatum*	1	3	3
Amblyomma dubitatum	*Amblyomma dubitatum*	1	3	3
Amblyomma dubitatum	*Amblyomma dubitatum*	1	3	3
Amblyomma dubitatum	*Amblyomma dubitatum*	1	3	3
Amblyomma dubitatum	*Amblyomma dubitatum*	1	3	3
Amblyomma dubitatum	*Amblyomma aureolatum*	0	3	1
Amblyomma dubitatum	*Amblyomma dubitatum*	1	3	3
Amblyomma dubitatum	*Amblyomma dubitatum*	1	3	3
Amblyomma dubitatum	*Amblyomma dubitatum*	1	3	3
Amblyomma dubitatum	*Amblyomma dubitatum*	1	3	3
Amblyomma dubitatum	*Amblyomma dubitatum*	1	3	3
Amblyomma ovale	*Amblyomma ovale*	1	4	4
Amblyomma ovale	*Amblyomma aureolatum*	0	4	1
Amblyomma ovale	*Amblyomma dubitatum*	0	4	3
Amblyomma ovale	*Amblyomma ovale*	1	4	4
Amblyomma ovale	*Amblyomma ovale*	1	4	4
Amblyomma ovale	*Amblyomma ovale*	1	4	4
Amblyomma ovale	*Amblyomma ovale*	1	4	4
Amblyomma ovale	*Amblyomma ovale*	1	4	4
Amblyomma ovale	*Amblyomma ovale*	1	4	4
Amblyomma ovale	*Amblyomma ovale*	1	4	4
Amblyomma ovale	*Amblyomma ovale*	1	4	4
Amblyomma ovale	*Amblyomma ovale*	1	4	4
Amblyomma ovale	*Amblyomma ovale*	1	4	4
Amblyomma ovale	*Amblyomma ovale*	1	4	4
Amblyomma ovale	*Amblyomma ovale*	1	4	4
Amblyomma ovale	*Amblyomma ovale*	1	4	4
Amblyomma ovale	*Amblyomma ovale*	1	4	4
Amblyomma ovale	*Amblyomma aureolatum*	0	4	1
Amblyomma ovale	*Amblyomma aureolatum*	0	4	1
Amblyomma ovale	*Amblyomma ovale*	1	4	4
Amblyomma ovale	*Amblyomma aureolatum*	0	4	1
Amblyomma ovale	*Amblyomma ovale*	1	4	4
Amblyomma ovale	*Amblyomma ovale*	1	4	4
Amblyomma ovale	*Amblyomma ovale*	1	4	4
Amblyomma sculptum	*Amblyomma sculptum*	1	5	5
Amblyomma sculptum	*Amblyomma sculptum*	1	5	5
Amblyomma sculptum	*Amblyomma sculptum*	1	5	5
Amblyomma sculptum	*Amblyomma sculptum*	1	5	5
Amblyomma sculptum	*Amblyomma sculptum*	1	5	5
Amblyomma sculptum	*Amblyomma sculptum*	1	5	5
Amblyomma sculptum	*Amblyomma sculptum*	1	5	5
Amblyomma sculptum	*Amblyomma sculptum*	1	5	5
Amblyomma sculptum	*Amblyomma sculptum*	1	5	5
Amblyomma sculptum	*Amblyomma sculptum*	1	5	5
Amblyomma sculptum	*Amblyomma sculptum*	1	5	5

Amblyomma sculptum	*Amblyomma sculptum*	1	5	5
Amblyomma sculptum	*Amblyomma sculptum*	1	5	5
Amblyomma sculptum	*Amblyomma sculptum*	1	5	5
Amblyomma sculptum	*Amblyomma sculptum*	1	5	5
Amblyomma sculptum	*Amblyomma sculptum*	1	5	5
Amblyomma sculptum	*Amblyomma sculptum*	1	5	5
Amblyomma sculptum	*Amblyomma sculptum*	1	5	5
Amblyomma sculptum	*Amblyomma sculptum*	1	5	5
Amblyomma sculptum	*Amblyomma sculptum*	1	5	5
Amblyomma sculptum	*Amblyomma sculptum*	1	5	5
Amblyomma sculptum	*Amblyomma sculptum*	1	5	5
Amblyomma sculptum	*Amblyomma aureolatum*	0	5	1
Amblyomma sculptum	*Amblyomma dubitatum*	0	5	3
Amblyomma sculptum	*Amblyomma sculptum*	1	5	5
Amblyomma sculptum	*Amblyomma sculptum*	1	5	5
Amblyomma sculptum	*Amblyomma sculptum*	1	5	5
Amblyomma sculptum	*Amblyomma sculptum*	1	5	5
Amblyomma sculptum	*Amblyomma sculptum*	1	5	5
Amblyomma sculptum	*Amblyomma sculptum*	1	5	5
Amblyomma sculptum	*Amblyomma sculptum*	1	5	5
Amblyomma triste	*Amblyomma triste*	1	6	6
Amblyomma triste	*Amblyomma triste*	1	6	6
Amblyomma triste	*Amblyomma triste*	1	6	6
Amblyomma triste	*Amblyomma triste*	1	6	6
Amblyomma triste	*Amblyomma triste*	1	6	6
Amblyomma triste	*Amblyomma triste*	1	6	6
Amblyomma triste	*Amblyomma triste*	1	6	6
Amblyomma triste	*Amblyomma triste*	1	6	6
Amblyomma triste	*Amblyomma triste*	1	6	6
Amblyomma triste	*Amblyomma triste*	1	6	6
Amblyomma triste	*Amblyomma triste*	1	6	6
Amblyomma triste	*Amblyomma triste*	1	6	6
Amblyomma triste	*Amblyomma triste*	1	6	6
Amblyomma triste	*Amblyomma triste*	1	6	6
Amblyomma triste	*Amblyomma triste*	1	6	6
Amblyomma triste	*Amblyomma triste*	1	6	6
Amblyomma triste	*Amblyomma triste*	1	6	6
Amblyomma triste	*Amblyomma triste*	1	6	6
Amblyomma triste	*Amblyomma triste*	1	6	6
Amblyomma triste	*Amblyomma triste*	1	6	6
Amblyomma triste	*Amblyomma triste*	1	6	6
Amblyomma triste	*Amblyomma triste*	1	6	6
Amblyomma triste	*Amblyomma triste*	1	6	6
Amblyomma triste	*Amblyomma triste*	1	6	6
Amblyomma triste	*Amblyomma triste*	1	6	6
Amblyomma triste	*Amblyomma triste*	1	6	6
Amblyomma triste	*Amblyomma triste*	1	6	6
Amblyomma triste	*Amblyomma triste*	1	6	6
Amblyomma triste	*Amblyomma triste*	1	6	6
Amblyomma triste	*Amblyomma triste*	1	6	6
Amblyomma triste	*Amblyomma triste*	1	6	6
Amblyomma aureolatum	*Amblyomma aureolatum*	1	1	1
Amblyomma aureolatum	*Amblyomma aureolatum*	1	1	1
Amblyomma aureolatum	*Amblyomma aureolatum*	1	1	1
Amblyomma aureolatum	*Amblyomma aureolatum*	1	1	1
Amblyomma aureolatum	*Amblyomma aureolatum*	1	1	1
Amblyomma aureolatum	*Amblyomma aureolatum*	1	1	1
Amblyomma aureolatum	*Amblyomma aureolatum*	1	1	1
Amblyomma aureolatum	*Amblyomma aureolatum*	1	1	1
Amblyomma aureolatum	*Amblyomma aureolatum*	1	1	1
Amblyomma aureolatum	*Amblyomma aureolatum*	1	1	1
Amblyomma aureolatum	*Amblyomma aureolatum*	1	1	1
Amblyomma aureolatum	*Amblyomma aureolatum*	1	1	1
Amblyomma aureolatum	*Amblyomma aureolatum*	1	1	1
Amblyomma aureolatum	*Amblyomma aureolatum*	1	1	1
Amblyomma aureolatum	*Amblyomma aureolatum*	1	1	1
Amblyomma aureolatum	*Amblyomma aureolatum*	1	1	1

Amblyomma aureolatum	*Amblyomma aureolatum*	1	1	1
Amblyomma aureolatum	*Amblyomma aureolatum*	1	1	1
Amblyomma aureolatum	*Amblyomma aureolatum*	1	1	1
Amblyomma aureolatum	*Amblyomma aureolatum*	1	1	1
Amblyomma aureolatum	*Amblyomma aureolatum*	1	1	1
Amblyomma aureolatum	*Amblyomma aureolatum*	1	1	1
Amblyomma aureolatum	*Amblyomma aureolatum*	1	1	1
Amblyomma aureolatum	*Amblyomma aureolatum*	1	1	1
Amblyomma aureolatum	*Amblyomma aureolatum*	1	1	1
Amblyomma cajennense	*Amblyomma cajennense*	1	2	2
Amblyomma cajennense	*Amblyomma cajennense*	1	2	2
Amblyomma cajennense	*Amblyomma cajennense*	1	2	2
Amblyomma cajennense	*Amblyomma cajennense*	1	2	2
Amblyomma cajennense	*Amblyomma cajennense*	1	2	2
Amblyomma cajennense	*Amblyomma cajennense*	1	2	2
Amblyomma cajennense	*Amblyomma cajennense*	1	2	2
Amblyomma cajennense	*Amblyomma dubitatum*	0	2	3
Amblyomma cajennense	*Amblyomma cajennense*	1	2	2
Amblyomma cajennense	*Amblyomma cajennense*	1	2	2
Amblyomma cajennense	*Amblyomma cajennense*	1	2	2
Amblyomma cajennense	*Amblyomma cajennense*	1	2	2
Amblyomma cajennense	*Amblyomma cajennense*	1	2	2
Amblyomma cajennense	*Amblyomma cajennense*	1	2	2
Amblyomma cajennense	*Amblyomma cajennense*	1	2	2
Amblyomma cajennense	*Amblyomma cajennense*	1	2	2
Amblyomma cajennense	*Amblyomma cajennense*	1	2	2
Amblyomma cajennense	*Amblyomma cajennense*	1	2	2
Amblyomma cajennense	*Amblyomma cajennense*	1	2	2
Amblyomma cajennense	*Amblyomma cajennense*	1	2	2
Amblyomma cajennense	*Amblyomma cajennense*	1	2	2
Amblyomma cajennense	*Amblyomma cajennense*	1	2	2
Amblyomma cajennense	*Amblyomma cajennense*	1	2	2
Amblyomma cajennense	*Amblyomma cajennense*	1	2	2
Amblyomma cajennense	*Amblyomma cajennense*	1	2	2
Amblyomma cajennense	*Amblyomma cajennense*	1	2	2
Amblyomma cajennense	*Amblyomma cajennense*	1	2	2
Amblyomma cajennense	*Amblyomma cajennense*	1	2	2
Amblyomma cajennense	*Amblyomma cajennense*	1	2	2
Amblyomma cajennense	*Amblyomma cajennense*	1	2	2
Amblyomma cajennense	*Amblyomma cajennense*	1	2	2
Amblyomma cajennense	*Amblyomma cajennense*	1	2	2
Amblyomma cajennense	*Amblyomma cajennense*	1	2	2
Amblyomma cajennense	*Amblyomma cajennense*	1	2	2
Amblyomma dubitatum	*Amblyomma triste*	0	3	6
Amblyomma dubitatum	*Amblyomma dubitatum*	1	3	3
Amblyomma dubitatum	*Amblyomma dubitatum*	1	3	3
Amblyomma dubitatum	*Amblyomma dubitatum*	1	3	3
Amblyomma dubitatum	*Amblyomma dubitatum*	1	3	3
Amblyomma dubitatum	*Amblyomma aureolatum*	0	3	1
Amblyomma dubitatum	*Amblyomma dubitatum*	1	3	3
Amblyomma dubitatum	*Amblyomma dubitatum*	1	3	3
Amblyomma dubitatum	*Amblyomma dubitatum*	1	3	3
Amblyomma dubitatum	*Amblyomma dubitatum*	1	3	3
Amblyomma dubitatum	*Amblyomma dubitatum*	1	3	3
Amblyomma dubitatum	*Amblyomma dubitatum*	1	3	3
Amblyomma dubitatum	*Amblyomma dubitatum*	1	3	3
Amblyomma dubitatum	*Amblyomma dubitatum*	1	3	3
Amblyomma dubitatum	*Amblyomma aureolatum*	0	3	1
Amblyomma dubitatum	*Amblyomma aureolatum*	0	3	1
Amblyomma dubitatum	*Amblyomma dubitatum*	1	3	3
Amblyomma dubitatum	*Amblyomma dubitatum*	1	3	3
Amblyomma dubitatum	*Amblyomma dubitatum*	1	3	3
Amblyomma dubitatum	*Amblyomma dubitatum*	1	3	3
Amblyomma dubitatum	*Amblyomma dubitatum*	1	3	3
Amblyomma dubitatum	*Amblyomma dubitatum*	1	3	3
Amblyomma ovale	*Amblyomma ovale*	1	4	4
Amblyomma ovale	*Amblyomma ovale*	1	4	4
Amblyomma ovale	*Amblyomma ovale*	1	4	4

Amblyomma ovale	*Amblyomma ovale*	1	4	4
Amblyomma ovale	*Amblyomma ovale*	1	4	4
Amblyomma ovale	*Amblyomma ovale*	1	4	4
Amblyomma ovale	*Amblyomma ovale*	1	4	4
Amblyomma ovale	*Amblyomma ovale*	1	4	4
Amblyomma ovale	*Amblyomma ovale*	1	4	4
Amblyomma ovale	*Amblyomma ovale*	1	4	4
Amblyomma ovale	*Amblyomma aureolatum*	0	4	1
Amblyomma ovale	*Amblyomma ovale*	1	4	4
Amblyomma ovale	*Amblyomma aureolatum*	0	4	1
Amblyomma ovale	*Amblyomma ovale*	1	4	4
Amblyomma ovale	*Amblyomma ovale*	1	4	4
Amblyomma ovale	*Amblyomma ovale*	1	4	4
Amblyomma ovale	*Amblyomma triste*	0	4	6
Amblyomma ovale	*Amblyomma ovale*	1	4	4
Amblyomma ovale	*Amblyomma ovale*	1	4	4
Amblyomma ovale	*Amblyomma ovale*	1	4	4
Amblyomma ovale	*Amblyomma ovale*	1	4	4
Amblyomma ovale	*Amblyomma ovale*	1	4	4
Amblyomma ovale	*Amblyomma ovale*	1	4	4
Amblyomma ovale	*Amblyomma ovale*	1	4	4
Amblyomma ovale	*Amblyomma ovale*	1	4	4
Amblyomma sculptum	*Amblyomma sculptum*	1	5	5
Amblyomma sculptum	*Amblyomma sculptum*	1	5	5
Amblyomma sculptum	*Amblyomma sculptum*	1	5	5
Amblyomma sculptum	*Amblyomma sculptum*	1	5	5
Amblyomma sculptum	*Amblyomma sculptum*	1	5	5
Amblyomma sculptum	*Amblyomma sculptum*	1	5	5
Amblyomma sculptum	*Amblyomma sculptum*	1	5	5
Amblyomma sculptum	*Amblyomma sculptum*	1	5	5
Amblyomma sculptum	*Amblyomma sculptum*	1	5	5
Amblyomma sculptum	*Amblyomma sculptum*	1	5	5
Amblyomma sculptum	*Amblyomma sculptum*	1	5	5
Amblyomma sculptum	*Amblyomma sculptum*	1	5	5
Amblyomma sculptum	*Amblyomma sculptum*	1	5	5
Amblyomma sculptum	*Amblyomma sculptum*	1	5	5
Amblyomma sculptum	*Amblyomma sculptum*	1	5	5
Amblyomma sculptum	*Amblyomma sculptum*	1	5	5
Amblyomma sculptum	*Amblyomma sculptum*	1	5	5
Amblyomma sculptum	*Amblyomma sculptum*	1	5	5
Amblyomma sculptum	*Amblyomma sculptum*	1	5	5
Amblyomma sculptum	*Amblyomma sculptum*	1	5	5
Amblyomma sculptum	*Amblyomma sculptum*	1	5	5
Amblyomma sculptum	*Amblyomma sculptum*	1	5	5
Amblyomma sculptum	*Amblyomma sculptum*	1	5	5
Amblyomma sculptum	*Amblyomma sculptum*	1	5	5
Amblyomma sculptum	*Amblyomma sculptum*	1	5	5
Amblyomma sculptum	*Amblyomma sculptum*	1	5	5
Amblyomma sculptum	*Amblyomma sculptum*	1	5	5
Amblyomma sculptum	*Amblyomma sculptum*	1	5	5
Amblyomma sculptum	*Amblyomma sculptum*	1	5	5
Amblyomma sculptum	*Amblyomma sculptum*	1	5	5
Amblyomma sculptum	*Amblyomma sculptum*	1	5	5
Amblyomma triste	*Amblyomma triste*	1	6	6
Amblyomma triste	*Amblyomma triste*	1	6	6
Amblyomma triste	*Amblyomma triste*	1	6	6
Amblyomma triste	*Amblyomma dubitatum*	0	6	3
Amblyomma triste	*Amblyomma triste*	1	6	6
Amblyomma triste	*Amblyomma triste*	1	6	6
Amblyomma triste	*Amblyomma triste*	1	6	6
Amblyomma triste	*Amblyomma triste*	1	6	6
Amblyomma triste	*Amblyomma triste*	1	6	6
Amblyomma triste	*Amblyomma triste*	1	6	6
Amblyomma triste	*Amblyomma triste*	1	6	6
Amblyomma triste	*Amblyomma triste*	1	6	6
Amblyomma triste	*Amblyomma triste*	1	6	6
Amblyomma triste	*Amblyomma sculptum*	0	6	5

Amblyomma triste	*Amblyomma triste*	1	6	6
Amblyomma triste	*Amblyomma triste*	1	6	6
Amblyomma triste	*Amblyomma triste*	1	6	6
Amblyomma triste	*Amblyomma triste*	1	6	6
Amblyomma triste	*Amblyomma triste*	1	6	6
Amblyomma triste	*Amblyomma triste*	1	6	6
Amblyomma triste	*Amblyomma triste*	1	6	6
Amblyomma triste	*Amblyomma triste*	1	6	6
Amblyomma triste	*Amblyomma triste*	1	6	6
Amblyomma triste	*Amblyomma triste*	1	6	6
Amblyomma triste	*Amblyomma triste*	1	6	6
Amblyomma triste	*Amblyomma triste*	1	6	6
Amblyomma triste	*Amblyomma triste*	1	6	6
Amblyomma triste	*Amblyomma triste*	1	6	6
Amblyomma triste	*Amblyomma triste*	1	6	6
Amblyomma triste	*Amblyomma triste*	1	6	6
Amblyomma triste	*Amblyomma triste*	1	6	6
Amblyomma triste	*Amblyomma triste*	1	6	6
Amblyomma aureolatum	*Amblyomma aureolatum*	1	1	1
Amblyomma aureolatum	*Amblyomma aureolatum*	1	1	1
Amblyomma aureolatum	*Amblyomma aureolatum*	1	1	1
Amblyomma aureolatum	*Amblyomma aureolatum*	1	1	1
Amblyomma aureolatum	*Amblyomma aureolatum*	1	1	1
Amblyomma aureolatum	*Amblyomma aureolatum*	1	1	1
Amblyomma aureolatum	*Amblyomma aureolatum*	1	1	1
Amblyomma aureolatum	*Amblyomma aureolatum*	1	1	1
Amblyomma aureolatum	*Amblyomma dubitatum*	0	1	3
Amblyomma aureolatum	*Amblyomma aureolatum*	1	1	1
Amblyomma aureolatum	*Amblyomma aureolatum*	1	1	1
Amblyomma aureolatum	*Amblyomma aureolatum*	1	1	1
Amblyomma aureolatum	*Amblyomma aureolatum*	1	1	1
Amblyomma aureolatum	*Amblyomma aureolatum*	1	1	1
Amblyomma aureolatum	*Amblyomma aureolatum*	1	1	1
Amblyomma aureolatum	*Amblyomma aureolatum*	1	1	1
Amblyomma aureolatum	*Amblyomma aureolatum*	1	1	1
Amblyomma aureolatum	*Amblyomma aureolatum*	1	1	1
Amblyomma aureolatum	*Amblyomma aureolatum*	1	1	1
Amblyomma aureolatum	*Amblyomma aureolatum*	1	1	1
Amblyomma aureolatum	*Amblyomma aureolatum*	1	1	1
Amblyomma aureolatum	*Amblyomma aureolatum*	1	1	1
Amblyomma aureolatum	*Amblyomma ovale*	0	1	4
Amblyomma aureolatum	*Amblyomma aureolatum*	1	1	1
Amblyomma cajennense	*Amblyomma cajennense*	1	2	2
Amblyomma cajennense	*Amblyomma cajennense*	1	2	2
Amblyomma cajennense	*Amblyomma cajennense*	1	2	2
Amblyomma cajennense	*Amblyomma cajennense*	1	2	2
Amblyomma cajennense	*Amblyomma cajennense*	1	2	2
Amblyomma cajennense	*Amblyomma cajennense*	1	2	2
Amblyomma cajennense	*Amblyomma cajennense*	1	2	2
Amblyomma cajennense	*Amblyomma cajennense*	1	2	2
Amblyomma cajennense	*Amblyomma cajennense*	1	2	2
Amblyomma cajennense	*Amblyomma cajennense*	1	2	2
Amblyomma cajennense	*Amblyomma cajennense*	1	2	2
Amblyomma cajennense	*Amblyomma cajennense*	1	2	2
Amblyomma cajennense	*Amblyomma cajennense*	1	2	2
Amblyomma cajennense	*Amblyomma cajennense*	1	2	2
Amblyomma cajennense	*Amblyomma cajennense*	1	2	2
Amblyomma cajennense	*Amblyomma cajennense*	1	2	2
Amblyomma cajennense	*Amblyomma cajennense*	1	2	2
Amblyomma cajennense	*Amblyomma cajennense*	1	2	2
Amblyomma cajennense	*Amblyomma cajennense*	1	2	2
Amblyomma cajennense	*Amblyomma cajennense*	1	2	2
Amblyomma cajennense	*Amblyomma cajennense*	1	2	2
Amblyomma cajennense	*Amblyomma cajennense*	1	2	2
Amblyomma cajennense	*Amblyomma cajennense*	1	2	2
Amblyomma cajennense	*Amblyomma cajennense*	1	2	2

Amblyomma cajennense	*Amblyomma cajennense*	1	2	2
Amblyomma cajennense	*Amblyomma cajennense*	1	2	2
Amblyomma cajennense	*Amblyomma cajennense*	1	2	2
Amblyomma cajennense	*Amblyomma cajennense*	1	2	2
Amblyomma cajennense	*Amblyomma cajennense*	1	2	2
Amblyomma cajennense	*Amblyomma cajennense*	1	2	2
Amblyomma cajennense	*Amblyomma cajennense*	1	2	2
Amblyomma cajennense	*Amblyomma cajennense*	1	2	2
Amblyomma dubitatum	*Amblyomma dubitatum*	1	3	3
Amblyomma dubitatum	*Amblyomma dubitatum*	1	3	3
Amblyomma dubitatum	*Amblyomma dubitatum*	1	3	3
Amblyomma dubitatum	*Amblyomma dubitatum*	1	3	3
Amblyomma dubitatum	*Amblyomma dubitatum*	1	3	3
Amblyomma dubitatum	*Amblyomma dubitatum*	1	3	3
Amblyomma dubitatum	*Amblyomma dubitatum*	1	3	3
Amblyomma dubitatum	*Amblyomma dubitatum*	1	3	3
Amblyomma dubitatum	*Amblyomma dubitatum*	1	3	3
Amblyomma dubitatum	*Amblyomma dubitatum*	1	3	3
Amblyomma dubitatum	*Amblyomma dubitatum*	1	3	3
Amblyomma dubitatum	*Amblyomma dubitatum*	1	3	3
Amblyomma dubitatum	*Amblyomma dubitatum*	1	3	3
Amblyomma dubitatum	*Amblyomma dubitatum*	1	3	3
Amblyomma dubitatum	*Amblyomma dubitatum*	1	3	3
Amblyomma dubitatum	*Amblyomma dubitatum*	1	3	3
Amblyomma dubitatum	*Amblyomma dubitatum*	1	3	3
Amblyomma dubitatum	*Amblyomma dubitatum*	1	3	3
Amblyomma dubitatum	*Amblyomma dubitatum*	1	3	3
Amblyomma dubitatum	*Amblyomma dubitatum*	1	3	3
Amblyomma dubitatum	*Amblyomma dubitatum*	1	3	3
Amblyomma ovale	*Amblyomma ovale*	1	4	4
Amblyomma ovale	*Amblyomma ovale*	1	4	4
Amblyomma ovale	*Amblyomma ovale*	1	4	4
Amblyomma ovale	*Amblyomma ovale*	1	4	4
Amblyomma ovale	*Amblyomma ovale*	1	4	4
Amblyomma ovale	*Amblyomma ovale*	1	4	4
Amblyomma ovale	*Amblyomma ovale*	1	4	4
Amblyomma ovale	*Amblyomma ovale*	1	4	4
Amblyomma ovale	*Amblyomma ovale*	1	4	4
Amblyomma ovale	*Amblyomma ovale*	1	4	4
Amblyomma ovale	*Amblyomma ovale*	1	4	4
Amblyomma ovale	*Amblyomma ovale*	1	4	4
Amblyomma ovale	*Amblyomma ovale*	1	4	4
Amblyomma ovale	*Amblyomma ovale*	1	4	4
Amblyomma ovale	*Amblyomma ovale*	1	4	4
Amblyomma ovale	*Amblyomma ovale*	1	4	4
Amblyomma ovale	*Amblyomma ovale*	1	4	4
Amblyomma ovale	*Amblyomma ovale*	1	4	4
Amblyomma ovale	*Amblyomma ovale*	1	4	4
Amblyomma ovale	*Amblyomma ovale*	1	4	4
Amblyomma ovale	*Amblyomma ovale*	1	4	4
Amblyomma ovale	*Amblyomma aureolatum*	0	4	1
Amblyomma ovale	*Amblyomma ovale*	1	4	4
Amblyomma ovale	*Amblyomma ovale*	1	4	4
Amblyomma sculptum	*Amblyomma sculptum*	1	5	5
Amblyomma sculptum	*Amblyomma sculptum*	1	5	5
Amblyomma sculptum	*Amblyomma sculptum*	1	5	5
Amblyomma sculptum	*Amblyomma sculptum*	1	5	5
Amblyomma sculptum	*Amblyomma sculptum*	1	5	5
Amblyomma sculptum	*Amblyomma sculptum*	1	5	5
Amblyomma sculptum	*Amblyomma sculptum*	1	5	5
Amblyomma sculptum	*Amblyomma sculptum*	1	5	5
Amblyomma sculptum	*Amblyomma sculptum*	1	5	5
Amblyomma sculptum	*Amblyomma sculptum*	1	5	5
Amblyomma sculptum	*Amblyomma sculptum*	1	5	5
Amblyomma sculptum	*Amblyomma sculptum*	1	5	5
Amblyomma sculptum	*Amblyomma sculptum*	1	5	5
Amblyomma sculptum	*Amblyomma sculptum*	1	5	5

Amblyomma sculptum	*Amblyomma sculptum*	1	5	5
Amblyomma sculptum	*Amblyomma sculptum*	1	5	5
Amblyomma sculptum	*Amblyomma sculptum*	1	5	5
Amblyomma sculptum	*Amblyomma dubitatum*	0	5	3
Amblyomma sculptum	*Amblyomma sculptum*	1	5	5
Amblyomma sculptum	*Amblyomma sculptum*	1	5	5
Amblyomma sculptum	*Amblyomma sculptum*	1	5	5
Amblyomma sculptum	*Amblyomma sculptum*	1	5	5
Amblyomma sculptum	*Amblyomma sculptum*	1	5	5
Amblyomma sculptum	*Amblyomma sculptum*	1	5	5
Amblyomma sculptum	*Amblyomma sculptum*	1	5	5
Amblyomma sculptum	*Amblyomma sculptum*	1	5	5
Amblyomma sculptum	*Amblyomma sculptum*	1	5	5
Amblyomma sculptum	*Amblyomma sculptum*	1	5	5
Amblyomma sculptum	*Amblyomma sculptum*	1	5	5
Amblyomma sculptum	*Amblyomma sculptum*	1	5	5
Amblyomma sculptum	*Amblyomma sculptum*	1	5	5
Amblyomma triste	*Amblyomma triste*	1	6	6
Amblyomma triste	*Amblyomma triste*	1	6	6
Amblyomma triste	*Amblyomma triste*	1	6	6
Amblyomma triste	*Amblyomma triste*	1	6	6
Amblyomma triste	*Amblyomma triste*	1	6	6
Amblyomma triste	*Amblyomma triste*	1	6	6
Amblyomma triste	*Amblyomma triste*	1	6	6
Amblyomma triste	*Amblyomma triste*	1	6	6
Amblyomma triste	*Amblyomma triste*	1	6	6
Amblyomma triste	*Amblyomma triste*	1	6	6
Amblyomma triste	*Amblyomma triste*	1	6	6
Amblyomma triste	*Amblyomma triste*	1	6	6
Amblyomma triste	*Amblyomma triste*	1	6	6
Amblyomma triste	*Amblyomma cajennense*	0	6	2
Amblyomma triste	*Amblyomma triste*	1	6	6
Amblyomma triste	*Amblyomma triste*	1	6	6
Amblyomma triste	*Amblyomma triste*	1	6	6
Amblyomma triste	*Amblyomma triste*	1	6	6
Amblyomma triste	*Amblyomma triste*	1	6	6
Amblyomma triste	*Amblyomma triste*	1	6	6
Amblyomma triste	*Amblyomma triste*	1	6	6
Amblyomma triste	*Amblyomma triste*	1	6	6
Amblyomma triste	*Amblyomma triste*	1	6	6
Amblyomma triste	*Amblyomma triste*	1	6	6
Amblyomma triste	*Amblyomma triste*	1	6	6
Amblyomma triste	*Amblyomma triste*	1	6	6
Amblyomma triste	*Amblyomma triste*	1	6	6
Amblyomma triste	*Amblyomma aureolatum*	0	6	1
Amblyomma triste	*Amblyomma triste*	1	6	6
Amblyomma triste	*Amblyomma triste*	1	6	6
Amblyomma triste	*Amblyomma triste*	1	6	6
Amblyomma aureolatum	*Amblyomma aureolatum*	1	1	1
Amblyomma aureolatum	*Amblyomma aureolatum*	1	1	1
Amblyomma aureolatum	*Amblyomma aureolatum*	1	1	1
Amblyomma aureolatum	*Amblyomma aureolatum*	1	1	1
Amblyomma aureolatum	*Amblyomma aureolatum*	1	1	1
Amblyomma aureolatum	*Amblyomma aureolatum*	1	1	1
Amblyomma aureolatum	*Amblyomma aureolatum*	1	1	1
Amblyomma aureolatum	*Amblyomma aureolatum*	1	1	1
Amblyomma aureolatum	*Amblyomma aureolatum*	1	1	1
Amblyomma aureolatum	*Amblyomma sculptum*	0	1	5
Amblyomma aureolatum	*Amblyomma aureolatum*	1	1	1
Amblyomma cajennense	*Amblyomma cajennense*	1	2	2
Amblyomma cajennense	*Amblyomma triste*	0	2	6
Amblyomma cajennense	*Amblyomma cajennense*	1	2	2
Amblyomma cajennense	*Amblyomma cajennense*	1	2	2
Amblyomma cajennense	*Amblyomma cajennense*	1	2	2
Amblyomma cajennense	*Amblyomma cajennense*	1	2	2
Amblyomma cajennense	*Amblyomma cajennense*	1	2	2
Amblyomma cajennense	*Amblyomma cajennense*	1	2	2

Amblyomma cajennense	*Amblyomma cajennense*	1	2	2
Amblyomma cajennense	*Amblyomma cajennense*	1	2	2
Amblyomma cajennense	*Amblyomma cajennense*	1	2	2
Amblyomma cajennense	*Amblyomma cajennense*	1	2	2
Amblyomma cajennense	*Amblyomma cajennense*	1	2	2
Amblyomma cajennense	*Amblyomma cajennense*	1	2	2
Amblyomma cajennense	*Amblyomma cajennense*	1	2	2
Amblyomma cajennense	*Amblyomma cajennense*	1	2	2
Amblyomma cajennense	*Amblyomma cajennense*	1	2	2
Amblyomma dubitatum	*Amblyomma dubitatum*	1	3	3
Amblyomma dubitatum	*Amblyomma sculptum*	0	3	5
Amblyomma dubitatum	*Amblyomma dubitatum*	1	3	3
Amblyomma dubitatum	*Amblyomma aureolatum*	0	3	1
Amblyomma dubitatum	*Amblyomma dubitatum*	1	3	3
Amblyomma dubitatum	*Amblyomma dubitatum*	1	3	3
Amblyomma dubitatum	*Amblyomma dubitatum*	1	3	3
Amblyomma ovale	*Amblyomma ovale*	1	4	4
Amblyomma ovale	*Amblyomma ovale*	1	4	4
Amblyomma ovale	*Amblyomma ovale*	1	4	4
Amblyomma ovale	*Amblyomma ovale*	1	4	4
Amblyomma ovale	*Amblyomma ovale*	1	4	4
Amblyomma ovale	*Amblyomma ovale*	1	4	4
Amblyomma ovale	*Amblyomma ovale*	1	4	4
Amblyomma ovale	*Amblyomma ovale*	1	4	4
Amblyomma ovale	*Amblyomma ovale*	1	4	4
Amblyomma ovale	*Amblyomma ovale*	1	4	4
Amblyomma ovale	*Amblyomma ovale*	1	4	4
Amblyomma ovale	*Amblyomma ovale*	1	4	4
Amblyomma ovale	*Amblyomma ovale*	1	4	4
Amblyomma ovale	*Amblyomma ovale*	1	4	4
Amblyomma ovale	*Amblyomma ovale*	1	4	4
Amblyomma ovale	*Amblyomma ovale*	1	4	4
Amblyomma sculptum	*Amblyomma sculptum*	1	5	5
Amblyomma sculptum	*Amblyomma sculptum*	1	5	5
Amblyomma sculptum	*Amblyomma sculptum*	1	5	5
Amblyomma sculptum	*Amblyomma sculptum*	1	5	5
Amblyomma sculptum	*Amblyomma sculptum*	1	5	5
Amblyomma sculptum	*Amblyomma sculptum*	1	5	5
Amblyomma sculptum	*Amblyomma sculptum*	1	5	5
Amblyomma sculptum	*Amblyomma sculptum*	1	5	5
Amblyomma sculptum	*Amblyomma dubitatum*	0	5	3
Amblyomma sculptum	*Amblyomma dubitatum*	0	5	3
Amblyomma sculptum	*Amblyomma sculptum*	1	5	5
Amblyomma sculptum	*Amblyomma dubitatum*	0	5	3
Amblyomma sculptum	*Amblyomma sculptum*	1	5	5
Amblyomma triste	*Amblyomma triste*	1	6	6
Amblyomma triste	*Amblyomma aureolatum*	0	6	1
Amblyomma triste	*Amblyomma aureolatum*	0	6	1
Amblyomma triste	*Amblyomma triste*	1	6	6
Amblyomma triste	*Amblyomma triste*	1	6	6
Amblyomma triste	*Amblyomma triste*	1	6	6
Amblyomma triste	*Amblyomma triste*	1	6	6
Amblyomma triste	*Amblyomma triste*	1	6	6
Amblyomma triste	*Amblyomma ovale*	0	6	4
Amblyomma triste	*Amblyomma ovale*	0	6	4
Amblyomma triste	*Amblyomma ovale*	0	6	4
Amblyomma aureolatum	*Amblyomma cajennense*	0	1	2
Amblyomma aureolatum	*Amblyomma aureolatum*	1	1	1
Amblyomma aureolatum	*Amblyomma aureolatum*	1	1	1
Amblyomma aureolatum	*Amblyomma aureolatum*	1	1	1
Amblyomma aureolatum	*Amblyomma aureolatum*	1	1	1
Amblyomma aureolatum	*Amblyomma aureolatum*	1	1	1
Amblyomma aureolatum	*Amblyomma aureolatum*	1	1	1
Amblyomma aureolatum	*Amblyomma aureolatum*	1	1	1
Amblyomma aureolatum	*Amblyomma aureolatum*	1	1	1
Amblyomma aureolatum	*Amblyomma aureolatum*	1	1	1
Amblyomma aureolatum	*Amblyomma aureolatum*	1	1	1

Amblyomma aureolatum	*Amblyomma aureolatum*	1	1	1
Amblyomma cajennense	*Amblyomma cajennense*	1	2	2
Amblyomma cajennense	*Amblyomma aureolatum*	0	2	1
Amblyomma cajennense	*Amblyomma cajennense*	1	2	2
Amblyomma cajennense	*Amblyomma triste*	0	2	6
Amblyomma cajennense	*Amblyomma cajennense*	1	2	2
Amblyomma cajennense	*Amblyomma cajennense*	1	2	2
Amblyomma cajennense	*Amblyomma cajennense*	1	2	2
Amblyomma cajennense	*Amblyomma cajennense*	1	2	2
Amblyomma cajennense	*Amblyomma cajennense*	1	2	2
Amblyomma cajennense	*Amblyomma cajennense*	1	2	2
Amblyomma cajennense	*Amblyomma cajennense*	1	2	2
Amblyomma cajennense	*Amblyomma cajennense*	1	2	2
Amblyomma cajennense	*Amblyomma cajennense*	1	2	2
Amblyomma cajennense	*Amblyomma cajennense*	1	2	2
Amblyomma cajennense	*Amblyomma cajennense*	1	2	2
Amblyomma cajennense	*Amblyomma cajennense*	1	2	2
Amblyomma cajennense	*Amblyomma cajennense*	1	2	2
Amblyomma dubitatum	*Amblyomma dubitatum*	1	3	3
Amblyomma dubitatum	*Amblyomma dubitatum*	1	3	3
Amblyomma dubitatum	*Amblyomma dubitatum*	1	3	3
Amblyomma dubitatum	*Amblyomma dubitatum*	1	3	3
Amblyomma dubitatum	*Amblyomma aureolatum*	0	3	1
Amblyomma dubitatum	*Amblyomma sculptum*	0	3	5
Amblyomma dubitatum	*Amblyomma dubitatum*	1	3	3
Amblyomma ovale	*Amblyomma dubitatum*	0	4	3
Amblyomma ovale	*Amblyomma aureolatum*	0	4	1
Amblyomma ovale	*Amblyomma ovale*	1	4	4
Amblyomma ovale	*Amblyomma ovale*	1	4	4
Amblyomma ovale	*Amblyomma ovale*	1	4	4
Amblyomma ovale	*Amblyomma ovale*	1	4	4
Amblyomma ovale	*Amblyomma ovale*	1	4	4
Amblyomma ovale	*Amblyomma ovale*	1	4	4
Amblyomma ovale	*Amblyomma ovale*	1	4	4
Amblyomma ovale	*Amblyomma ovale*	1	4	4
Amblyomma ovale	*Amblyomma ovale*	1	4	4
Amblyomma ovale	*Amblyomma triste*	0	4	6
Amblyomma ovale	*Amblyomma ovale*	1	4	4
Amblyomma ovale	*Amblyomma ovale*	1	4	4
Amblyomma ovale	*Amblyomma ovale*	1	4	4
Amblyomma ovale	*Amblyomma ovale*	1	4	4
Amblyomma sculptum	*Amblyomma sculptum*	1	5	5
Amblyomma sculptum	*Amblyomma sculptum*	1	5	5
Amblyomma sculptum	*Amblyomma sculptum*	1	5	5
Amblyomma sculptum	*Amblyomma sculptum*	1	5	5
Amblyomma sculptum	*Amblyomma sculptum*	1	5	5
Amblyomma sculptum	*Amblyomma sculptum*	1	5	5
Amblyomma sculptum	*Amblyomma sculptum*	1	5	5
Amblyomma sculptum	*Amblyomma sculptum*	1	5	5
Amblyomma sculptum	*Amblyomma dubitatum*	0	5	3
Amblyomma sculptum	*Amblyomma sculptum*	1	5	5
Amblyomma sculptum	*Amblyomma sculptum*	1	5	5
Amblyomma sculptum	*Amblyomma ovale*	0	5	4
Amblyomma triste	*Amblyomma aureolatum*	0	6	1
Amblyomma triste	*Amblyomma triste*	1	6	6
Amblyomma triste	*Amblyomma triste*	1	6	6
Amblyomma triste	*Amblyomma triste*	1	6	6
Amblyomma triste	*Amblyomma triste*	1	6	6
Amblyomma triste	*Amblyomma triste*	1	6	6
Amblyomma triste	*Amblyomma triste*	1	6	6
Amblyomma triste	*Amblyomma triste*	1	6	6
Amblyomma triste	*Amblyomma triste*	1	6	6
Amblyomma triste	*Amblyomma triste*	1	6	6
Amblyomma aureolatum	*Amblyomma aureolatum*	1	1	1
Amblyomma aureolatum	*Amblyomma aureolatum*	1	1	1
Amblyomma aureolatum	*Amblyomma cajennense*	0	1	2
Amblyomma aureolatum	*Amblyomma aureolatum*	1	1	1

Amblyomma aureolatum	*Amblyomma aureolatum*	1	1	1
Amblyomma aureolatum	*Amblyomma aureolatum*	1	1	1
Amblyomma aureolatum	*Amblyomma aureolatum*	1	1	1
Amblyomma aureolatum	*Amblyomma aureolatum*	1	1	1
Amblyomma aureolatum	*Amblyomma dubitatum*	0	1	3
Amblyomma aureolatum	*Amblyomma cajennense*	0	1	2
Amblyomma aureolatum	*Amblyomma aureolatum*	1	1	1
Amblyomma cajennense	*Amblyomma cajennense*	1	2	2
Amblyomma cajennense	*Amblyomma cajennense*	1	2	2
Amblyomma cajennense	*Amblyomma cajennense*	1	2	2
Amblyomma cajennense	*Amblyomma cajennense*	1	2	2
Amblyomma cajennense	*Amblyomma cajennense*	1	2	2
Amblyomma cajennense	*Amblyomma cajennense*	1	2	2
Amblyomma cajennense	*Amblyomma cajennense*	1	2	2
Amblyomma cajennense	*Amblyomma cajennense*	1	2	2
Amblyomma cajennense	*Amblyomma cajennense*	1	2	2
Amblyomma cajennense	*Amblyomma cajennense*	1	2	2
Amblyomma cajennense	*Amblyomma cajennense*	1	2	2
Amblyomma cajennense	*Amblyomma cajennense*	1	2	2
Amblyomma cajennense	*Amblyomma cajennense*	1	2	2
Amblyomma cajennense	*Amblyomma cajennense*	1	2	2
Amblyomma cajennense	*Amblyomma cajennense*	1	2	2
Amblyomma cajennense	*Amblyomma cajennense*	1	2	2
Amblyomma dubitatum	*Amblyomma dubitatum*	1	3	3
Amblyomma dubitatum	*Amblyomma dubitatum*	1	3	3
Amblyomma dubitatum	*Amblyomma dubitatum*	1	3	3
Amblyomma dubitatum	*Amblyomma dubitatum*	1	3	3
Amblyomma dubitatum	*Amblyomma cajennense*	0	3	2
Amblyomma dubitatum	*Amblyomma ovale*	0	3	4
Amblyomma dubitatum	*Amblyomma dubitatum*	1	3	3
Amblyomma ovale	*Amblyomma ovale*	1	4	4
Amblyomma ovale	*Amblyomma ovale*	1	4	4
Amblyomma ovale	*Amblyomma ovale*	1	4	4
Amblyomma ovale	*Amblyomma ovale*	1	4	4
Amblyomma ovale	*Amblyomma ovale*	1	4	4
Amblyomma ovale	*Amblyomma ovale*	1	4	4
Amblyomma ovale	*Amblyomma ovale*	1	4	4
Amblyomma ovale	*Amblyomma ovale*	1	4	4
Amblyomma ovale	*Amblyomma ovale*	1	4	4
Amblyomma ovale	*Amblyomma ovale*	1	4	4
Amblyomma ovale	*Amblyomma aureolatum*	0	4	1
Amblyomma ovale	*Amblyomma triste*	0	4	6
Amblyomma ovale	*Amblyomma ovale*	1	4	4
Amblyomma ovale	*Amblyomma ovale*	1	4	4
Amblyomma ovale	*Amblyomma ovale*	1	4	4
Amblyomma sculptum	*Amblyomma cajennense*	0	5	2
Amblyomma sculptum	*Amblyomma sculptum*	1	5	5
Amblyomma sculptum	*Amblyomma sculptum*	1	5	5
Amblyomma sculptum	*Amblyomma sculptum*	1	5	5
Amblyomma sculptum	*Amblyomma sculptum*	1	5	5
Amblyomma sculptum	*Amblyomma cajennense*	0	5	2
Amblyomma sculptum	*Amblyomma sculptum*	1	5	5
Amblyomma sculptum	*Amblyomma sculptum*	1	5	5
Amblyomma sculptum	*Amblyomma sculptum*	1	5	5
Amblyomma sculptum	*Amblyomma sculptum*	1	5	5
Amblyomma sculptum	*Amblyomma aureolatum*	0	5	1
Amblyomma sculptum	*Amblyomma sculptum*	1	5	5
Amblyomma sculptum	*Amblyomma sculptum*	1	5	5
Amblyomma triste	*Amblyomma sculptum*	0	6	5
Amblyomma triste	*Amblyomma triste*	1	6	6
Amblyomma triste	*Amblyomma triste*	1	6	6
Amblyomma triste	*Amblyomma sculptum*	0	6	5
Amblyomma triste	*Amblyomma triste*	1	6	6
Amblyomma triste	*Amblyomma aureolatum*	0	6	1
Amblyomma triste	*Amblyomma triste*	1	6	6
Amblyomma triste	*Amblyomma triste*	1	6	6
Amblyomma triste	*Amblyomma triste*	1	6	6

Amblyomma triste	*Amblyomma triste*	1	6	6
Amblyomma aureolatum	*Amblyomma aureolatum*	1	1	1
Amblyomma aureolatum	*Amblyomma aureolatum*	1	1	1
Amblyomma aureolatum	*Amblyomma aureolatum*	1	1	1
Amblyomma aureolatum	*Amblyomma aureolatum*	1	1	1
Amblyomma aureolatum	*Amblyomma ovale*	0	1	4
Amblyomma aureolatum	*Amblyomma aureolatum*	1	1	1
Amblyomma aureolatum	*Amblyomma aureolatum*	1	1	1
Amblyomma aureolatum	*Amblyomma aureolatum*	1	1	1
Amblyomma aureolatum	*Amblyomma aureolatum*	1	1	1
Amblyomma aureolatum	*Amblyomma aureolatum*	1	1	1
Amblyomma aureolatum	*Amblyomma aureolatum*	1	1	1
Amblyomma aureolatum	*Amblyomma aureolatum*	1	1	1
Amblyomma cajennense	*Amblyomma cajennense*	1	2	2
Amblyomma cajennense	*Amblyomma cajennense*	1	2	2
Amblyomma cajennense	*Amblyomma cajennense*	1	2	2
Amblyomma cajennense	*Amblyomma cajennense*	1	2	2
Amblyomma cajennense	*Amblyomma cajennense*	1	2	2
Amblyomma cajennense	*Amblyomma cajennense*	1	2	2
Amblyomma cajennense	*Amblyomma cajennense*	1	2	2
Amblyomma cajennense	*Amblyomma cajennense*	1	2	2
Amblyomma cajennense	*Amblyomma triste*	0	2	6
Amblyomma cajennense	*Amblyomma cajennense*	1	2	2
Amblyomma cajennense	*Amblyomma cajennense*	1	2	2
Amblyomma cajennense	*Amblyomma cajennense*	1	2	2
Amblyomma cajennense	*Amblyomma cajennense*	1	2	2
Amblyomma cajennense	*Amblyomma cajennense*	1	2	2
Amblyomma cajennense	*Amblyomma cajennense*	1	2	2
Amblyomma cajennense	*Amblyomma cajennense*	1	2	2
Amblyomma dubitatum	*Amblyomma dubitatum*	1	3	3
Amblyomma dubitatum	*Amblyomma aureolatum*	0	3	1
Amblyomma dubitatum	*Amblyomma dubitatum*	1	3	3
Amblyomma dubitatum	*Amblyomma dubitatum*	1	3	3
Amblyomma dubitatum	*Amblyomma dubitatum*	1	3	3
Amblyomma dubitatum	*Amblyomma dubitatum*	1	3	3
Amblyomma ovale	*Amblyomma ovale*	1	4	4
Amblyomma ovale	*Amblyomma ovale*	1	4	4
Amblyomma ovale	*Amblyomma ovale*	1	4	4
Amblyomma ovale	*Amblyomma ovale*	1	4	4
Amblyomma ovale	*Amblyomma ovale*	1	4	4
Amblyomma ovale	*Amblyomma ovale*	1	4	4
Amblyomma ovale	*Amblyomma ovale*	1	4	4
Amblyomma ovale	*Amblyomma ovale*	1	4	4
Amblyomma ovale	*Amblyomma ovale*	1	4	4
Amblyomma ovale	*Amblyomma ovale*	1	4	4
Amblyomma ovale	*Amblyomma aureolatum*	0	4	1
Amblyomma ovale	*Amblyomma ovale*	1	4	4
Amblyomma ovale	*Amblyomma ovale*	1	4	4
Amblyomma ovale	*Amblyomma ovale*	1	4	4
Amblyomma ovale	*Amblyomma ovale*	1	4	4
Amblyomma ovale	*Amblyomma ovale*	1	4	4
Amblyomma sculptum	*Amblyomma sculptum*	1	5	5
Amblyomma sculptum	*Amblyomma sculptum*	1	5	5
Amblyomma sculptum	*Amblyomma sculptum*	1	5	5
Amblyomma sculptum	*Amblyomma sculptum*	1	5	5
Amblyomma sculptum	*Amblyomma sculptum*	1	5	5
Amblyomma sculptum	*Amblyomma sculptum*	1	5	5
Amblyomma sculptum	*Amblyomma sculptum*	1	5	5
Amblyomma sculptum	*Amblyomma sculptum*	1	5	5
Amblyomma sculptum	*Amblyomma sculptum*	1	5	5
Amblyomma sculptum	*Amblyomma dubitatum*	0	5	3
Amblyomma sculptum	*Amblyomma sculptum*	1	5	5
Amblyomma sculptum	*Amblyomma dubitatum*	0	5	3
Amblyomma sculptum	*Amblyomma dubitatum*	0	5	3
Amblyomma triste	*Amblyomma triste*	1	6	6
Amblyomma triste	*Amblyomma triste*	1	6	6
Amblyomma triste	*Amblyomma triste*	1	6	6

Amblyomma triste	*Amblyomma aureolatum*	0	6	1
Amblyomma triste	*Amblyomma triste*	1	6	6
Amblyomma triste	*Amblyomma triste*	1	6	6
Amblyomma triste	*Amblyomma triste*	1	6	6
Amblyomma triste	*Amblyomma triste*	1	6	6
Amblyomma triste	*Amblyomma triste*	1	6	6
Amblyomma triste	*Amblyomma triste*	1	6	6
Amblyomma aureolatum	*Amblyomma aureolatum*	1	1	1
Amblyomma aureolatum	*Amblyomma aureolatum*	1	1	1
Amblyomma aureolatum	*Amblyomma aureolatum*	1	1	1
Amblyomma aureolatum	*Amblyomma aureolatum*	1	1	1
Amblyomma aureolatum	*Amblyomma aureolatum*	1	1	1
Amblyomma aureolatum	*Amblyomma aureolatum*	1	1	1
Amblyomma aureolatum	*Amblyomma ovale*	0	1	4
Amblyomma aureolatum	*Amblyomma aureolatum*	1	1	1
Amblyomma aureolatum	*Amblyomma aureolatum*	1	1	1
Amblyomma aureolatum	*Amblyomma aureolatum*	1	1	1
Amblyomma aureolatum	*Amblyomma aureolatum*	1	1	1
Amblyomma aureolatum	*Amblyomma aureolatum*	1	1	1
Amblyomma cajennense	*Amblyomma cajennense*	1	2	2
Amblyomma cajennense	*Amblyomma cajennense*	1	2	2
Amblyomma cajennense	*Amblyomma cajennense*	1	2	2
Amblyomma cajennense	*Amblyomma cajennense*	1	2	2
Amblyomma cajennense	*Amblyomma cajennense*	1	2	2
Amblyomma cajennense	*Amblyomma cajennense*	1	2	2
Amblyomma cajennense	*Amblyomma cajennense*	1	2	2
Amblyomma cajennense	*Amblyomma cajennense*	1	2	2
Amblyomma cajennense	*Amblyomma cajennense*	1	2	2
Amblyomma cajennense	*Amblyomma cajennense*	1	2	2
Amblyomma cajennense	*Amblyomma cajennense*	1	2	2
Amblyomma cajennense	*Amblyomma cajennense*	1	2	2
Amblyomma cajennense	*Amblyomma cajennense*	1	2	2
Amblyomma cajennense	*Amblyomma cajennense*	1	2	2
Amblyomma cajennense	*Amblyomma cajennense*	1	2	2
Amblyomma cajennense	*Amblyomma cajennense*	1	2	2
Amblyomma dubitatum	*Amblyomma sculptum*	0	3	5
Amblyomma dubitatum	*Amblyomma triste*	0	3	6
Amblyomma dubitatum	*Amblyomma aureolatum*	0	3	1
Amblyomma dubitatum	*Amblyomma sculptum*	0	3	5
Amblyomma dubitatum	*Amblyomma ovale*	0	3	4
Amblyomma dubitatum	*Amblyomma sculptum*	0	3	5
Amblyomma dubitatum	*Amblyomma dubitatum*	1	3	3
Amblyomma ovale	*Amblyomma dubitatum*	0	4	3
Amblyomma ovale	*Amblyomma ovale*	1	4	4
Amblyomma ovale	*Amblyomma ovale*	1	4	4
Amblyomma ovale	*Amblyomma ovale*	1	4	4
Amblyomma ovale	*Amblyomma ovale*	1	4	4
Amblyomma ovale	*Amblyomma ovale*	1	4	4
Amblyomma ovale	*Amblyomma ovale*	1	4	4
Amblyomma ovale	*Amblyomma ovale*	1	4	4
Amblyomma ovale	*Amblyomma ovale*	1	4	4
Amblyomma ovale	*Amblyomma ovale*	1	4	4
Amblyomma ovale	*Amblyomma ovale*	1	4	4
Amblyomma ovale	*Amblyomma ovale*	1	4	4
Amblyomma ovale	*Amblyomma ovale*	1	4	4
Amblyomma ovale	*Amblyomma ovale*	1	4	4
Amblyomma ovale	*Amblyomma ovale*	1	4	4
Amblyomma ovale	*Amblyomma ovale*	1	4	4
Amblyomma sculptum	*Amblyomma sculptum*	1	5	5
Amblyomma sculptum	*Amblyomma sculptum*	1	5	5
Amblyomma sculptum	*Amblyomma sculptum*	1	5	5
Amblyomma sculptum	*Amblyomma dubitatum*	0	5	3
Amblyomma sculptum	*Amblyomma sculptum*	1	5	5
Amblyomma sculptum	*Amblyomma sculptum*	1	5	5
Amblyomma sculptum	*Amblyomma sculptum*	1	5	5
Amblyomma sculptum	*Amblyomma sculptum*	1	5	5
Amblyomma sculptum	*Amblyomma sculptum*	1	5	5

Amblyomma sculptum	*Amblyomma sculptum*	1	5	5
Amblyomma sculptum	*Amblyomma cajennense*	0	5	2
Amblyomma sculptum	*Amblyomma aureolatum*	0	5	1
Amblyomma triste	*Amblyomma triste*	1	6	6
Amblyomma triste	*Amblyomma triste*	1	6	6
Amblyomma triste	*Amblyomma triste*	1	6	6
Amblyomma triste	*Amblyomma cajennense*	0	6	2
Amblyomma triste	*Amblyomma triste*	1	6	6
Amblyomma triste	*Amblyomma triste*	1	6	6
Amblyomma triste	*Amblyomma cajennense*	0	6	2
Amblyomma triste	*Amblyomma triste*	1	6	6
Amblyomma triste	*Amblyomma triste*	1	6	6
Amblyomma triste	*Amblyomma triste*	1	6	6
Amblyomma triste	*Amblyomma triste*	1	6	6
Amblyomma aureolatum	*Amblyomma aureolatum*	1	1	1
Amblyomma aureolatum	*Amblyomma aureolatum*	1	1	1
Amblyomma aureolatum	*Amblyomma aureolatum*	1	1	1
Amblyomma aureolatum	*Amblyomma cajennense*	0	1	2
Amblyomma aureolatum	*Amblyomma aureolatum*	1	1	1
Amblyomma aureolatum	*Amblyomma aureolatum*	1	1	1
Amblyomma aureolatum	*Amblyomma aureolatum*	1	1	1
Amblyomma aureolatum	*Amblyomma aureolatum*	1	1	1
Amblyomma aureolatum	*Amblyomma aureolatum*	1	1	1
Amblyomma aureolatum	*Amblyomma aureolatum*	1	1	1
Amblyomma aureolatum	*Amblyomma aureolatum*	1	1	1
Amblyomma aureolatum	*Amblyomma aureolatum*	1	1	1
Amblyomma cajennense	*Amblyomma cajennense*	1	2	2
Amblyomma cajennense	*Amblyomma cajennense*	1	2	2
Amblyomma cajennense	*Amblyomma cajennense*	1	2	2
Amblyomma cajennense	*Amblyomma cajennense*	1	2	2
Amblyomma cajennense	*Amblyomma cajennense*	1	2	2
Amblyomma cajennense	*Amblyomma cajennense*	1	2	2
Amblyomma cajennense	*Amblyomma cajennense*	1	2	2
Amblyomma cajennense	*Amblyomma cajennense*	1	2	2
Amblyomma cajennense	*Amblyomma aureolatum*	0	2	1
Amblyomma cajennense	*Amblyomma cajennense*	1	2	2
Amblyomma cajennense	*Amblyomma cajennense*	1	2	2
Amblyomma cajennense	*Amblyomma cajennense*	1	2	2
Amblyomma cajennense	*Amblyomma cajennense*	1	2	2
Amblyomma cajennense	*Amblyomma cajennense*	1	2	2
Amblyomma cajennense	*Amblyomma cajennense*	1	2	2
Amblyomma cajennense	*Amblyomma triste*	0	2	6
Amblyomma dubitatum	*Amblyomma dubitatum*	1	3	3
Amblyomma dubitatum	*Amblyomma dubitatum*	1	3	3
Amblyomma dubitatum	*Amblyomma dubitatum*	1	3	3
Amblyomma dubitatum	*Amblyomma sculptum*	0	3	5
Amblyomma dubitatum	*Amblyomma dubitatum*	1	3	3
Amblyomma dubitatum	*Amblyomma dubitatum*	1	3	3
Amblyomma dubitatum	*Amblyomma dubitatum*	1	3	3
Amblyomma dubitatum	*Amblyomma dubitatum*	1	3	3
Amblyomma dubitatum	*Amblyomma dubitatum*	1	3	3
Amblyomma dubitatum	*Amblyomma dubitatum*	1	3	3
Amblyomma dubitatum	*Amblyomma dubitatum*	1	3	3
Amblyomma dubitatum	*Amblyomma dubitatum*	1	3	3
Amblyomma dubitatum	*Amblyomma dubitatum*	1	3	3
Amblyomma dubitatum	*Amblyomma dubitatum*	1	3	3
Amblyomma dubitatum	*Amblyomma dubitatum*	1	3	3
Amblyomma ovale	*Amblyomma ovale*	1	4	4
Amblyomma ovale	*Amblyomma ovale*	1	4	4
Amblyomma ovale	*Amblyomma aureolatum*	0	4	1
Amblyomma ovale	*Amblyomma ovale*	1	4	4
Amblyomma ovale	*Amblyomma ovale*	1	4	4
Amblyomma ovale	*Amblyomma ovale*	1	4	4
Amblyomma ovale	*Amblyomma ovale*	1	4	4
Amblyomma ovale	*Amblyomma ovale*	1	4	4
Amblyomma sculptum	*Amblyomma sculptum*	1	5	5
Amblyomma sculptum	*Amblyomma sculptum*	1	5	5

Amblyomma sculptum	*Amblyomma sculptum*	1	5	5
Amblyomma sculptum	*Amblyomma sculptum*	1	5	5
Amblyomma sculptum	*Amblyomma sculptum*	1	5	5
Amblyomma sculptum	*Amblyomma ovale*	0	5	4
Amblyomma sculptum	*Amblyomma sculptum*	1	5	5
Amblyomma sculptum	*Amblyomma sculptum*	1	5	5
Amblyomma sculptum	*Amblyomma sculptum*	1	5	5
Amblyomma sculptum	*Amblyomma sculptum*	1	5	5
Amblyomma sculptum	*Amblyomma sculptum*	1	5	5
Amblyomma sculptum	*Amblyomma sculptum*	1	5	5
Amblyomma sculptum	*Amblyomma sculptum*	1	5	5
Amblyomma sculptum	*Amblyomma sculptum*	1	5	5
Amblyomma sculptum	*Amblyomma sculptum*	1	5	5
Amblyomma sculptum	*Amblyomma sculptum*	1	5	5
Amblyomma sculptum	*Amblyomma sculptum*	1	5	5
Amblyomma sculptum	*Amblyomma sculptum*	1	5	5
Amblyomma triste	*Amblyomma triste*	1	6	6
Amblyomma triste	*Amblyomma triste*	1	6	6
Amblyomma triste	*Amblyomma triste*	1	6	6
Amblyomma triste	*Amblyomma triste*	1	6	6
Amblyomma triste	*Amblyomma triste*	1	6	6
Amblyomma triste	*Amblyomma triste*	1	6	6
Amblyomma triste	*Amblyomma triste*	1	6	6
Amblyomma triste	*Amblyomma triste*	1	6	6
Amblyomma triste	*Amblyomma triste*	1	6	6
Amblyomma triste	*Amblyomma aureolatum*	0	6	1
Amblyomma triste	*Amblyomma triste*	1	6	6
Amblyomma triste	*Amblyomma triste*	1	6	6
Amblyomma triste	*Amblyomma triste*	1	6	6
Amblyomma triste	*Amblyomma triste*	1	6	6
Amblyomma triste	*Amblyomma triste*	1	6	6
Amblyomma triste	*Amblyomma triste*	1	6	6
Amblyomma triste	*Amblyomma triste*	1	6	6
Amblyomma triste	*Amblyomma triste*	1	6	6
Amblyomma triste	*Amblyomma triste*	1	6	6
Amblyomma triste	*Amblyomma triste*	1	6	6
Amblyomma aureolatum	*Amblyomma aureolatum*	1	1	1
Amblyomma aureolatum	*Amblyomma sculptum*	0	1	5
Amblyomma aureolatum	*Amblyomma aureolatum*	1	1	1
Amblyomma aureolatum	*Amblyomma aureolatum*	1	1	1
Amblyomma aureolatum	*Amblyomma aureolatum*	1	1	1
Amblyomma aureolatum	*Amblyomma aureolatum*	1	1	1
Amblyomma aureolatum	*Amblyomma aureolatum*	1	1	1
Amblyomma aureolatum	*Amblyomma aureolatum*	1	1	1
Amblyomma aureolatum	*Amblyomma aureolatum*	1	1	1
Amblyomma aureolatum	*Amblyomma aureolatum*	1	1	1
Amblyomma aureolatum	*Amblyomma aureolatum*	1	1	1
Amblyomma aureolatum	*Amblyomma aureolatum*	1	1	1
Amblyomma aureolatum	*Amblyomma aureolatum*	1	1	1
Amblyomma cajennense	*Amblyomma cajennense*	1	2	2
Amblyomma cajennense	*Amblyomma cajennense*	1	2	2
Amblyomma cajennense	*Amblyomma cajennense*	1	2	2
Amblyomma cajennense	*Amblyomma cajennense*	1	2	2
Amblyomma cajennense	*Amblyomma cajennense*	1	2	2
Amblyomma cajennense	*Amblyomma cajennense*	1	2	2
Amblyomma cajennense	*Amblyomma cajennense*	1	2	2
Amblyomma cajennense	*Amblyomma cajennense*	1	2	2
Amblyomma cajennense	*Amblyomma cajennense*	1	2	2
Amblyomma cajennense	*Amblyomma cajennense*	1	2	2
Amblyomma cajennense	*Amblyomma cajennense*	1	2	2
Amblyomma cajennense	*Amblyomma cajennense*	1	2	2
Amblyomma cajennense	*Amblyomma cajennense*	1	2	2
Amblyomma cajennense	*Amblyomma cajennense*	1	2	2
Amblyomma cajennense	*Amblyomma cajennense*	1	2	2
Amblyomma cajennense	*Amblyomma cajennense*	1	2	2
Amblyomma cajennense	*Amblyomma cajennense*	1	2	2
Amblyomma dubitatum	*Amblyomma triste*	0	3	6

Amblyomma dubitatum	*Amblyomma dubitatum*	1	3	3
Amblyomma dubitatum	*Amblyomma dubitatum*	1	3	3
Amblyomma dubitatum	*Amblyomma aureolatum*	0	3	1
Amblyomma dubitatum	*Amblyomma dubitatum*	1	3	3
Amblyomma dubitatum	*Amblyomma dubitatum*	1	3	3
Amblyomma dubitatum	*Amblyomma dubitatum*	1	3	3
Amblyomma dubitatum	*Amblyomma dubitatum*	1	3	3
Amblyomma dubitatum	*Amblyomma dubitatum*	1	3	3
Amblyomma dubitatum	*Amblyomma dubitatum*	1	3	3
Amblyomma dubitatum	*Amblyomma dubitatum*	1	3	3
Amblyomma dubitatum	*Amblyomma dubitatum*	1	3	3
Amblyomma dubitatum	*Amblyomma dubitatum*	1	3	3
Amblyomma dubitatum	*Amblyomma dubitatum*	1	3	3
Amblyomma dubitatum	*Amblyomma dubitatum*	1	3	3
Amblyomma ovale	*Amblyomma ovale*	1	4	4
Amblyomma ovale	*Amblyomma aureolatum*	0	4	1
Amblyomma ovale	*Amblyomma ovale*	1	4	4
Amblyomma ovale	*Amblyomma dubitatum*	0	4	3
Amblyomma ovale	*Amblyomma ovale*	1	4	4
Amblyomma ovale	*Amblyomma ovale*	1	4	4
Amblyomma ovale	*Amblyomma ovale*	1	4	4
Amblyomma ovale	*Amblyomma ovale*	1	4	4
Amblyomma ovale	*Amblyomma ovale*	1	4	4
Amblyomma sculptum	*Amblyomma sculptum*	1	5	5
Amblyomma sculptum	*Amblyomma sculptum*	1	5	5
Amblyomma sculptum	*Amblyomma sculptum*	1	5	5
Amblyomma sculptum	*Amblyomma sculptum*	1	5	5
Amblyomma sculptum	*Amblyomma sculptum*	1	5	5
Amblyomma sculptum	*Amblyomma sculptum*	1	5	5
Amblyomma sculptum	*Amblyomma sculptum*	1	5	5
Amblyomma sculptum	*Amblyomma sculptum*	1	5	5
Amblyomma sculptum	*Amblyomma sculptum*	1	5	5
Amblyomma sculptum	*Amblyomma sculptum*	1	5	5
Amblyomma sculptum	*Amblyomma dubitatum*	0	5	3
Amblyomma sculptum	*Amblyomma sculptum*	1	5	5
Amblyomma sculptum	*Amblyomma sculptum*	1	5	5
Amblyomma sculptum	*Amblyomma sculptum*	1	5	5
Amblyomma sculptum	*Amblyomma sculptum*	1	5	5
Amblyomma sculptum	*Amblyomma sculptum*	1	5	5
Amblyomma sculptum	*Amblyomma sculptum*	1	5	5
Amblyomma sculptum	*Amblyomma sculptum*	1	5	5
Amblyomma triste	*Amblyomma triste*	1	6	6
Amblyomma triste	*Amblyomma triste*	1	6	6
Amblyomma triste	*Amblyomma triste*	1	6	6
Amblyomma triste	*Amblyomma triste*	1	6	6
Amblyomma triste	*Amblyomma triste*	1	6	6
Amblyomma triste	*Amblyomma triste*	1	6	6
Amblyomma triste	*Amblyomma triste*	1	6	6
Amblyomma triste	*Amblyomma triste*	1	6	6
Amblyomma triste	*Amblyomma triste*	1	6	6
Amblyomma triste	*Amblyomma triste*	1	6	6
Amblyomma triste	*Amblyomma triste*	1	6	6
Amblyomma triste	*Amblyomma triste*	1	6	6
Amblyomma triste	*Amblyomma triste*	1	6	6
Amblyomma triste	*Amblyomma triste*	1	6	6
Amblyomma triste	*Amblyomma triste*	1	6	6
Amblyomma triste	*Amblyomma triste*	1	6	6
Amblyomma triste	*Amblyomma triste*	1	6	6
Amblyomma triste	*Amblyomma triste*	1	6	6
Amblyomma triste	*Amblyomma triste*	1	6	6
Amblyomma triste	*Amblyomma triste*	1	6	6
Amblyomma triste	*Amblyomma triste*	1	6	6
Amblyomma aureolatum	*Amblyomma aureolatum*	1	1	1
Amblyomma aureolatum	*Amblyomma aureolatum*	1	1	1
Amblyomma aureolatum	*Amblyomma aureolatum*	1	1	1
Amblyomma aureolatum	*Amblyomma aureolatum*	1	1	1
Amblyomma aureolatum	*Amblyomma aureolatum*	1	1	1

Amblyomma aureolatum	*Amblyomma aureolatum*	1	1	1
Amblyomma aureolatum	*Amblyomma aureolatum*	1	1	1
Amblyomma aureolatum	*Amblyomma aureolatum*	1	1	1
Amblyomma aureolatum	*Amblyomma aureolatum*	1	1	1
Amblyomma aureolatum	*Amblyomma aureolatum*	1	1	1
Amblyomma aureolatum	*Amblyomma aureolatum*	1	1	1
Amblyomma aureolatum	*Amblyomma aureolatum*	1	1	1
Amblyomma cajennense	*Amblyomma cajennense*	1	2	2
Amblyomma cajennense	*Amblyomma cajennense*	1	2	2
Amblyomma cajennense	*Amblyomma cajennense*	1	2	2
Amblyomma cajennense	*Amblyomma cajennense*	1	2	2
Amblyomma cajennense	*Amblyomma cajennense*	1	2	2
Amblyomma cajennense	*Amblyomma cajennense*	1	2	2
Amblyomma cajennense	*Amblyomma cajennense*	1	2	2
Amblyomma cajennense	*Amblyomma cajennense*	1	2	2
Amblyomma cajennense	*Amblyomma cajennense*	1	2	2
Amblyomma cajennense	*Amblyomma cajennense*	1	2	2
Amblyomma cajennense	*Amblyomma cajennense*	1	2	2
Amblyomma cajennense	*Amblyomma cajennense*	1	2	2
Amblyomma cajennense	*Amblyomma cajennense*	1	2	2
Amblyomma cajennense	*Amblyomma cajennense*	1	2	2
Amblyomma cajennense	*Amblyomma cajennense*	1	2	2
Amblyomma cajennense	*Amblyomma triste*	0	2	6
Amblyomma cajennense	*Amblyomma cajennense*	1	2	2
Amblyomma dubitatum	*Amblyomma dubitatum*	1	3	3
Amblyomma dubitatum	*Amblyomma dubitatum*	1	3	3
Amblyomma dubitatum	*Amblyomma dubitatum*	1	3	3
Amblyomma dubitatum	*Amblyomma dubitatum*	1	3	3
Amblyomma dubitatum	*Amblyomma dubitatum*	1	3	3
Amblyomma dubitatum	*Amblyomma dubitatum*	1	3	3
Amblyomma dubitatum	*Amblyomma dubitatum*	1	3	3
Amblyomma dubitatum	*Amblyomma dubitatum*	1	3	3
Amblyomma dubitatum	*Amblyomma dubitatum*	1	3	3
Amblyomma dubitatum	*Amblyomma dubitatum*	1	3	3
Amblyomma dubitatum	*Amblyomma dubitatum*	1	3	3
Amblyomma dubitatum	*Amblyomma dubitatum*	1	3	3
Amblyomma dubitatum	*Amblyomma dubitatum*	1	3	3
Amblyomma dubitatum	*Amblyomma dubitatum*	1	3	3
Amblyomma dubitatum	*Amblyomma dubitatum*	1	3	3
Amblyomma ovale	*Amblyomma ovale*	1	4	4
Amblyomma ovale	*Amblyomma ovale*	1	4	4
Amblyomma ovale	*Amblyomma ovale*	1	4	4
Amblyomma ovale	*Amblyomma ovale*	1	4	4
Amblyomma ovale	*Amblyomma ovale*	1	4	4
Amblyomma ovale	*Amblyomma ovale*	1	4	4
Amblyomma ovale	*Amblyomma ovale*	1	4	4
Amblyomma ovale	*Amblyomma ovale*	1	4	4
Amblyomma ovale	*Amblyomma ovale*	1	4	4
Amblyomma sculptum	*Amblyomma ovale*	0	5	4
Amblyomma sculptum	*Amblyomma sculptum*	1	5	5
Amblyomma sculptum	*Amblyomma sculptum*	1	5	5
Amblyomma sculptum	*Amblyomma sculptum*	1	5	5
Amblyomma sculptum	*Amblyomma ovale*	0	5	4
Amblyomma sculptum	*Amblyomma sculptum*	1	5	5
Amblyomma sculptum	*Amblyomma sculptum*	1	5	5
Amblyomma sculptum	*Amblyomma sculptum*	1	5	5
Amblyomma sculptum	*Amblyomma sculptum*	1	5	5
Amblyomma sculptum	*Amblyomma sculptum*	1	5	5
Amblyomma sculptum	*Amblyomma sculptum*	1	5	5
Amblyomma sculptum	*Amblyomma sculptum*	1	5	5
Amblyomma sculptum	*Amblyomma sculptum*	1	5	5
Amblyomma sculptum	*Amblyomma sculptum*	1	5	5
Amblyomma sculptum	*Amblyomma sculptum*	1	5	5
Amblyomma sculptum	*Amblyomma sculptum*	1	5	5
Amblyomma sculptum	*Amblyomma sculptum*	1	5	5
Amblyomma sculptum	*Amblyomma sculptum*	1	5	5
Amblyomma sculptum	*Amblyomma sculptum*	1	5	5

Amblyomma triste	*Amblyomma triste*	1	6	6
Amblyomma triste	*Amblyomma triste*	1	6	6
Amblyomma triste	*Amblyomma triste*	1	6	6
Amblyomma triste	*Amblyomma triste*	1	6	6
Amblyomma triste	*Amblyomma triste*	1	6	6
Amblyomma triste	*Amblyomma triste*	1	6	6
Amblyomma triste	*Amblyomma dubitatum*	0	6	3
Amblyomma triste	*Amblyomma triste*	1	6	6
Amblyomma triste	*Amblyomma triste*	1	6	6
Amblyomma triste	*Amblyomma triste*	1	6	6
Amblyomma triste	*Amblyomma triste*	1	6	6
Amblyomma triste	*Amblyomma triste*	1	6	6
Amblyomma triste	*Amblyomma triste*	1	6	6
Amblyomma triste	*Amblyomma triste*	1	6	6
Amblyomma triste	*Amblyomma triste*	1	6	6
Amblyomma triste	*Amblyomma triste*	1	6	6
Amblyomma triste	*Amblyomma triste*	1	6	6
Amblyomma triste	*Amblyomma triste*	1	6	6
Amblyomma triste	*Amblyomma triste*	1	6	6
Amblyomma triste	*Amblyomma triste*	1	6	6
Amblyomma aureolatum	*Amblyomma aureolatum*	1	1	1
Amblyomma aureolatum	*Amblyomma aureolatum*	1	1	1
Amblyomma aureolatum	*Amblyomma aureolatum*	1	1	1
Amblyomma aureolatum	*Amblyomma aureolatum*	1	1	1
Amblyomma aureolatum	*Amblyomma sculptum*	0	1	5
Amblyomma aureolatum	*Amblyomma aureolatum*	1	1	1
Amblyomma aureolatum	*Amblyomma aureolatum*	1	1	1
Amblyomma aureolatum	*Amblyomma aureolatum*	1	1	1
Amblyomma aureolatum	*Amblyomma aureolatum*	1	1	1
Amblyomma aureolatum	*Amblyomma aureolatum*	1	1	1
Amblyomma aureolatum	*Amblyomma aureolatum*	1	1	1
Amblyomma aureolatum	*Amblyomma aureolatum*	1	1	1
Amblyomma aureolatum	*Amblyomma aureolatum*	1	1	1
Amblyomma cajennense	*Amblyomma cajennense*	1	2	2
Amblyomma cajennense	*Amblyomma cajennense*	1	2	2
Amblyomma cajennense	*Amblyomma cajennense*	1	2	2
Amblyomma cajennense	*Amblyomma cajennense*	1	2	2
Amblyomma cajennense	*Amblyomma cajennense*	1	2	2
Amblyomma cajennense	*Amblyomma cajennense*	1	2	2
Amblyomma cajennense	*Amblyomma cajennense*	1	2	2
Amblyomma cajennense	*Amblyomma cajennense*	1	2	2
Amblyomma cajennense	*Amblyomma cajennense*	1	2	2
Amblyomma cajennense	*Amblyomma cajennense*	1	2	2
Amblyomma cajennense	*Amblyomma cajennense*	1	2	2
Amblyomma cajennense	*Amblyomma cajennense*	1	2	2
Amblyomma cajennense	*Amblyomma cajennense*	1	2	2
Amblyomma cajennense	*Amblyomma cajennense*	1	2	2
Amblyomma cajennense	*Amblyomma cajennense*	1	2	2
Amblyomma cajennense	*Amblyomma cajennense*	1	2	2
Amblyomma dubitatum	*Amblyomma dubitatum*	1	3	3
Amblyomma dubitatum	*Amblyomma cajennense*	0	3	2
Amblyomma dubitatum	*Amblyomma dubitatum*	1	3	3
Amblyomma dubitatum	*Amblyomma dubitatum*	1	3	3
Amblyomma dubitatum	*Amblyomma sculptum*	0	3	5
Amblyomma dubitatum	*Amblyomma dubitatum*	1	3	3
Amblyomma dubitatum	*Amblyomma dubitatum*	1	3	3
Amblyomma dubitatum	*Amblyomma dubitatum*	1	3	3
Amblyomma dubitatum	*Amblyomma ovale*	0	3	4
Amblyomma dubitatum	*Amblyomma dubitatum*	1	3	3
Amblyomma dubitatum	*Amblyomma sculptum*	0	3	5
Amblyomma dubitatum	*Amblyomma dubitatum*	1	3	3
Amblyomma dubitatum	*Amblyomma dubitatum*	1	3	3
Amblyomma dubitatum	*Amblyomma sculptum*	0	3	5
Amblyomma dubitatum	*Amblyomma dubitatum*	1	3	3
Amblyomma ovale	*Amblyomma ovale*	1	4	4
Amblyomma ovale	*Amblyomma triste*	0	4	6
Amblyomma ovale	*Amblyomma ovale*	1	4	4

Amblyomma ovale	*Amblyomma aureolatum*	0	4	1
Amblyomma ovale	*Amblyomma ovale*	1	4	4
Amblyomma ovale	*Amblyomma ovale*	1	4	4
Amblyomma ovale	*Amblyomma ovale*	1	4	4
Amblyomma ovale	*Amblyomma sculptum*	0	4	5
Amblyomma sculptum	*Amblyomma sculptum*	1	5	5
Amblyomma sculptum	*Amblyomma sculptum*	1	5	5
Amblyomma sculptum	*Amblyomma sculptum*	1	5	5
Amblyomma sculptum	*Amblyomma sculptum*	1	5	5
Amblyomma sculptum	*Amblyomma sculptum*	1	5	5
Amblyomma sculptum	*Amblyomma sculptum*	1	5	5
Amblyomma sculptum	*Amblyomma sculptum*	1	5	5
Amblyomma sculptum	*Amblyomma sculptum*	1	5	5
Amblyomma sculptum	*Amblyomma sculptum*	1	5	5
Amblyomma sculptum	*Amblyomma sculptum*	1	5	5
Amblyomma sculptum	*Amblyomma sculptum*	1	5	5
Amblyomma sculptum	*Amblyomma sculptum*	1	5	5
Amblyomma sculptum	*Amblyomma sculptum*	1	5	5
Amblyomma sculptum	*Amblyomma sculptum*	1	5	5
Amblyomma sculptum	*Amblyomma sculptum*	1	5	5
Amblyomma sculptum	*Amblyomma sculptum*	1	5	5
Amblyomma sculptum	*Amblyomma sculptum*	1	5	5
Amblyomma sculptum	*Amblyomma sculptum*	1	5	5
Amblyomma sculptum	*Amblyomma sculptum*	1	5	5
Amblyomma triste	*Amblyomma triste*	1	6	6
Amblyomma triste	*Amblyomma triste*	1	6	6
Amblyomma triste	*Amblyomma triste*	1	6	6
Amblyomma triste	*Amblyomma triste*	1	6	6
Amblyomma triste	*Amblyomma triste*	1	6	6
Amblyomma triste	*Amblyomma triste*	1	6	6
Amblyomma triste	*Amblyomma triste*	1	6	6
Amblyomma triste	*Amblyomma triste*	1	6	6
Amblyomma triste	*Amblyomma triste*	1	6	6
Amblyomma triste	*Amblyomma triste*	1	6	6
Amblyomma triste	*Amblyomma triste*	1	6	6
Amblyomma triste	*Amblyomma triste*	1	6	6
Amblyomma triste	*Amblyomma triste*	1	6	6
Amblyomma triste	*Amblyomma triste*	1	6	6
Amblyomma triste	*Amblyomma triste*	1	6	6
Amblyomma triste	*Amblyomma triste*	1	6	6
Amblyomma triste	*Amblyomma triste*	1	6	6
Amblyomma triste	*Amblyomma triste*	1	6	6
Amblyomma triste	*Amblyomma triste*	1	6	6
Amblyomma triste	*Amblyomma triste*	1	6	6
Amblyomma triste	*Amblyomma triste*	1	6	6
Amblyomma aureolatum	*Amblyomma aureolatum*	1	1	1
Amblyomma aureolatum	*Amblyomma aureolatum*	1	1	1
Amblyomma aureolatum	*Amblyomma ovale*	0	1	4
Amblyomma aureolatum	*Amblyomma aureolatum*	1	1	1
Amblyomma aureolatum	*Amblyomma aureolatum*	1	1	1
Amblyomma aureolatum	*Amblyomma aureolatum*	1	1	1
Amblyomma aureolatum	*Amblyomma aureolatum*	1	1	1
Amblyomma aureolatum	*Amblyomma aureolatum*	1	1	1
Amblyomma aureolatum	*Amblyomma aureolatum*	1	1	1
Amblyomma aureolatum	*Amblyomma aureolatum*	1	1	1
Amblyomma aureolatum	*Amblyomma aureolatum*	1	1	1
Amblyomma aureolatum	*Amblyomma aureolatum*	1	1	1
Amblyomma aureolatum	*Amblyomma aureolatum*	1	1	1
Amblyomma cajennense	*Amblyomma cajennense*	1	2	2
Amblyomma cajennense	*Amblyomma cajennense*	1	2	2
Amblyomma cajennense	*Amblyomma cajennense*	1	2	2
Amblyomma cajennense	*Amblyomma cajennense*	1	2	2
Amblyomma cajennense	*Amblyomma cajennense*	1	2	2
Amblyomma cajennense	*Amblyomma cajennense*	1	2	2
Amblyomma cajennense	*Amblyomma cajennense*	1	2	2
Amblyomma cajennense	*Amblyomma cajennense*	1	2	2
Amblyomma cajennense	*Amblyomma cajennense*	1	2	2

Amblyomma cajennense	*Amblyomma cajennense*	1	2	2
Amblyomma cajennense	*Amblyomma cajennense*	1	2	2
Amblyomma cajennense	*Amblyomma cajennense*	1	2	2
Amblyomma cajennense	*Amblyomma cajennense*	1	2	2
Amblyomma cajennense	*Amblyomma cajennense*	1	2	2
Amblyomma cajennense	*Amblyomma cajennense*	1	2	2
Amblyomma cajennense	*Amblyomma cajennense*	1	2	2
Amblyomma cajennense	*Amblyomma cajennense*	1	2	2
Amblyomma dubitatum	*Amblyomma dubitatum*	1	3	3
Amblyomma dubitatum	*Amblyomma dubitatum*	1	3	3
Amblyomma dubitatum	*Amblyomma dubitatum*	1	3	3
Amblyomma dubitatum	*Amblyomma dubitatum*	1	3	3
Amblyomma dubitatum	*Amblyomma dubitatum*	1	3	3
Amblyomma dubitatum	*Amblyomma dubitatum*	1	3	3
Amblyomma dubitatum	*Amblyomma dubitatum*	1	3	3
Amblyomma dubitatum	*Amblyomma dubitatum*	1	3	3
Amblyomma dubitatum	*Amblyomma dubitatum*	1	3	3
Amblyomma dubitatum	*Amblyomma dubitatum*	1	3	3
Amblyomma dubitatum	*Amblyomma dubitatum*	1	3	3
Amblyomma dubitatum	*Amblyomma dubitatum*	1	3	3
Amblyomma dubitatum	*Amblyomma dubitatum*	1	3	3
Amblyomma dubitatum	*Amblyomma dubitatum*	1	3	3
Amblyomma dubitatum	*Amblyomma dubitatum*	1	3	3
Amblyomma ovale	*Amblyomma ovale*	1	4	4
Amblyomma ovale	*Amblyomma ovale*	1	4	4
Amblyomma ovale	*Amblyomma ovale*	1	4	4
Amblyomma ovale	*Amblyomma ovale*	1	4	4
Amblyomma ovale	*Amblyomma ovale*	1	4	4
Amblyomma ovale	*Amblyomma ovale*	1	4	4
Amblyomma ovale	*Amblyomma ovale*	1	4	4
Amblyomma ovale	*Amblyomma ovale*	1	4	4
Amblyomma sculptum	*Amblyomma sculptum*	1	5	5
Amblyomma sculptum	*Amblyomma sculptum*	1	5	5
Amblyomma sculptum	*Amblyomma sculptum*	1	5	5
Amblyomma sculptum	*Amblyomma sculptum*	1	5	5
Amblyomma sculptum	*Amblyomma sculptum*	1	5	5
Amblyomma sculptum	*Amblyomma sculptum*	1	5	5
Amblyomma sculptum	*Amblyomma sculptum*	1	5	5
Amblyomma sculptum	*Amblyomma sculptum*	1	5	5
Amblyomma sculptum	*Amblyomma sculptum*	1	5	5
Amblyomma sculptum	*Amblyomma sculptum*	1	5	5
Amblyomma sculptum	*Amblyomma sculptum*	1	5	5
Amblyomma sculptum	*Amblyomma sculptum*	1	5	5
Amblyomma sculptum	*Amblyomma sculptum*	1	5	5
Amblyomma sculptum	*Amblyomma sculptum*	1	5	5
Amblyomma sculptum	*Amblyomma sculptum*	1	5	5
Amblyomma sculptum	*Amblyomma sculptum*	1	5	5
Amblyomma sculptum	*Amblyomma sculptum*	1	5	5
Amblyomma sculptum	*Amblyomma sculptum*	1	5	5
Amblyomma triste	*Amblyomma triste*	1	6	6
Amblyomma triste	*Amblyomma triste*	1	6	6
Amblyomma triste	*Amblyomma triste*	1	6	6
Amblyomma triste	*Amblyomma triste*	1	6	6
Amblyomma triste	*Amblyomma triste*	1	6	6
Amblyomma triste	*Amblyomma triste*	1	6	6
Amblyomma triste	*Amblyomma triste*	1	6	6
Amblyomma triste	*Amblyomma sculptum*	0	6	5
Amblyomma triste	*Amblyomma triste*	1	6	6
Amblyomma triste	*Amblyomma triste*	1	6	6
Amblyomma triste	*Amblyomma triste*	1	6	6
Amblyomma triste	*Amblyomma triste*	1	6	6
Amblyomma triste	*Amblyomma triste*	1	6	6
Amblyomma triste	*Amblyomma triste*	1	6	6
Amblyomma triste	*Amblyomma triste*	1	6	6
Amblyomma triste	*Amblyomma triste*	1	6	6
Amblyomma triste	*Amblyomma triste*	1	6	6
Amblyomma triste	*Amblyomma triste*	1	6	6

Amblyomma triste	*Amblyomma dubitatum*	0	6	3
Amblyomma triste	*Amblyomma triste*	1	6	6
Amblyomma triste	*Amblyomma triste*	1	6	6
Amblyomma aureolatum	*Amblyomma triste*	0	1	6
Amblyomma aureolatum	*Amblyomma cajennense*	0	1	2
Amblyomma aureolatum	*Amblyomma aureolatum*	1	1	1
Amblyomma aureolatum	*Amblyomma aureolatum*	1	1	1
Amblyomma aureolatum	*Amblyomma aureolatum*	1	1	1
Amblyomma aureolatum	*Amblyomma aureolatum*	1	1	1
Amblyomma aureolatum	*Amblyomma aureolatum*	1	1	1
Amblyomma aureolatum	*Amblyomma aureolatum*	1	1	1
Amblyomma aureolatum	*Amblyomma aureolatum*	1	1	1
Amblyomma aureolatum	*Amblyomma aureolatum*	1	1	1
Amblyomma aureolatum	*Amblyomma aureolatum*	1	1	1
Amblyomma aureolatum	*Amblyomma dubitatum*	0	1	3
Amblyomma cajennense	*Amblyomma cajennense*	1	2	2
Amblyomma cajennense	*Amblyomma cajennense*	1	2	2
Amblyomma cajennense	*Amblyomma cajennense*	1	2	2
Amblyomma cajennense	*Amblyomma cajennense*	1	2	2
Amblyomma cajennense	*Amblyomma cajennense*	1	2	2
Amblyomma cajennense	*Amblyomma cajennense*	1	2	2
Amblyomma cajennense	*Amblyomma cajennense*	1	2	2
Amblyomma cajennense	*Amblyomma cajennense*	1	2	2
Amblyomma cajennense	*Amblyomma cajennense*	1	2	2
Amblyomma cajennense	*Amblyomma cajennense*	1	2	2
Amblyomma cajennense	*Amblyomma cajennense*	1	2	2
Amblyomma cajennense	*Amblyomma triste*	0	2	6
Amblyomma cajennense	*Amblyomma cajennense*	1	2	2
Amblyomma cajennense	*Amblyomma cajennense*	1	2	2
Amblyomma cajennense	*Amblyomma cajennense*	1	2	2
Amblyomma cajennense	*Amblyomma cajennense*	1	2	2
Amblyomma dubitatum	*Amblyomma dubitatum*	1	3	3
Amblyomma dubitatum	*Amblyomma dubitatum*	1	3	3
Amblyomma dubitatum	*Amblyomma dubitatum*	1	3	3
Amblyomma dubitatum	*Amblyomma dubitatum*	1	3	3
Amblyomma dubitatum	*Amblyomma dubitatum*	1	3	3
Amblyomma dubitatum	*Amblyomma dubitatum*	1	3	3
Amblyomma dubitatum	*Amblyomma dubitatum*	1	3	3
Amblyomma dubitatum	*Amblyomma dubitatum*	1	3	3
Amblyomma dubitatum	*Amblyomma dubitatum*	1	3	3
Amblyomma dubitatum	*Amblyomma sculptum*	0	3	5
Amblyomma dubitatum	*Amblyomma dubitatum*	1	3	3
Amblyomma ovale	*Amblyomma ovale*	1	4	4
Amblyomma ovale	*Amblyomma ovale*	1	4	4
Amblyomma ovale	*Amblyomma ovale*	1	4	4
Amblyomma ovale	*Amblyomma ovale*	1	4	4
Amblyomma ovale	*Amblyomma ovale*	1	4	4
Amblyomma ovale	*Amblyomma ovale*	1	4	4
Amblyomma ovale	*Amblyomma ovale*	1	4	4
Amblyomma ovale	*Amblyomma ovale*	1	4	4
Amblyomma ovale	*Amblyomma ovale*	1	4	4
Amblyomma ovale	*Amblyomma triste*	0	4	6
Amblyomma ovale	*Amblyomma ovale*	1	4	4
Amblyomma ovale	*Amblyomma ovale*	1	4	4
Amblyomma sculptum	*Amblyomma sculptum*	1	5	5
Amblyomma sculptum	*Amblyomma sculptum*	1	5	5
Amblyomma sculptum	*Amblyomma sculptum*	1	5	5
Amblyomma sculptum	*Amblyomma sculptum*	1	5	5
Amblyomma sculptum	*Amblyomma sculptum*	1	5	5
Amblyomma sculptum	*Amblyomma sculptum*	1	5	5
Amblyomma sculptum	*Amblyomma sculptum*	1	5	5
Amblyomma sculptum	*Amblyomma cajennense*	0	5	2
Amblyomma sculptum	*Amblyomma sculptum*	1	5	5
Amblyomma sculptum	*Amblyomma sculptum*	1	5	5
Amblyomma sculptum	*Amblyomma sculptum*	1	5	5
Amblyomma sculptum	*Amblyomma sculptum*	1	5	5
Amblyomma sculptum	*Amblyomma sculptum*	1	5	5

Amblyomma sculptum	*Amblyomma sculptum*	1	5	5
Amblyomma sculptum	*Amblyomma sculptum*	1	5	5
Amblyomma sculptum	*Amblyomma sculptum*	1	5	5
Amblyomma triste	*Amblyomma triste*	1	6	6
Amblyomma triste	*Amblyomma triste*	1	6	6
Amblyomma triste	*Amblyomma triste*	1	6	6
Amblyomma triste	*Amblyomma triste*	1	6	6
Amblyomma triste	*Amblyomma triste*	1	6	6
Amblyomma triste	*Amblyomma triste*	1	6	6
Amblyomma triste	*Amblyomma triste*	1	6	6
Amblyomma triste	*Amblyomma triste*	1	6	6
Amblyomma triste	*Amblyomma triste*	1	6	6
Amblyomma triste	*Amblyomma triste*	1	6	6
Amblyomma triste	*Amblyomma triste*	1	6	6
Amblyomma triste	*Amblyomma triste*	1	6	6
Amblyomma triste	*Amblyomma triste*	1	6	6
Amblyomma triste	*Amblyomma triste*	1	6	6
Amblyomma triste	*Amblyomma triste*	1	6	6
Amblyomma triste	*Amblyomma triste*	1	6	6
Amblyomma aureolatum	*Amblyomma aureolatum*	1	1	1
Amblyomma aureolatum	*Amblyomma aureolatum*	1	1	1
Amblyomma aureolatum	*Amblyomma aureolatum*	1	1	1
Amblyomma aureolatum	*Amblyomma aureolatum*	1	1	1
Amblyomma aureolatum	*Amblyomma aureolatum*	1	1	1
Amblyomma aureolatum	*Amblyomma aureolatum*	1	1	1
Amblyomma aureolatum	*Amblyomma dubitatum*	0	1	3
Amblyomma aureolatum	*Amblyomma aureolatum*	1	1	1
Amblyomma aureolatum	*Amblyomma aureolatum*	1	1	1
Amblyomma aureolatum	*Amblyomma aureolatum*	1	1	1
Amblyomma aureolatum	*Amblyomma aureolatum*	1	1	1
Amblyomma aureolatum	*Amblyomma aureolatum*	1	1	1
Amblyomma aureolatum	*Amblyomma aureolatum*	1	1	1
Amblyomma cajennense	*Amblyomma cajennense*	1	2	2
Amblyomma cajennense	*Amblyomma cajennense*	1	2	2
Amblyomma cajennense	*Amblyomma cajennense*	1	2	2
Amblyomma cajennense	*Amblyomma cajennense*	1	2	2
Amblyomma cajennense	*Amblyomma cajennense*	1	2	2
Amblyomma cajennense	*Amblyomma cajennense*	1	2	2
Amblyomma cajennense	*Amblyomma cajennense*	1	2	2
Amblyomma cajennense	*Amblyomma cajennense*	1	2	2
Amblyomma cajennense	*Amblyomma cajennense*	1	2	2
Amblyomma cajennense	*Amblyomma cajennense*	1	2	2
Amblyomma cajennense	*Amblyomma cajennense*	1	2	2
Amblyomma cajennense	*Amblyomma cajennense*	1	2	2
Amblyomma cajennense	*Amblyomma cajennense*	1	2	2
Amblyomma cajennense	*Amblyomma cajennense*	1	2	2
Amblyomma cajennense	*Amblyomma cajennense*	1	2	2
Amblyomma cajennense	*Amblyomma cajennense*	1	2	2
Amblyomma dubitatum	*Amblyomma aureolatum*	0	3	1
Amblyomma dubitatum	*Amblyomma cajennense*	0	3	2
Amblyomma dubitatum	*Amblyomma ovale*	0	3	4
Amblyomma dubitatum	*Amblyomma dubitatum*	1	3	3
Amblyomma dubitatum	*Amblyomma aureolatum*	0	3	1
Amblyomma dubitatum	*Amblyomma dubitatum*	1	3	3
Amblyomma dubitatum	*Amblyomma dubitatum*	1	3	3
Amblyomma dubitatum	*Amblyomma cajennense*	0	3	2
Amblyomma dubitatum	*Amblyomma dubitatum*	1	3	3
Amblyomma dubitatum	*Amblyomma aureolatum*	0	3	1
Amblyomma dubitatum	*Amblyomma dubitatum*	1	3	3
Amblyomma dubitatum	*Amblyomma cajennense*	0	3	2
Amblyomma ovale	*Amblyomma ovale*	1	4	4
Amblyomma ovale	*Amblyomma ovale*	1	4	4
Amblyomma ovale	*Amblyomma ovale*	1	4	4
Amblyomma ovale	*Amblyomma ovale*	1	4	4
Amblyomma ovale	*Amblyomma ovale*	1	4	4
Amblyomma ovale	*Amblyomma ovale*	1	4	4
Amblyomma ovale	*Amblyomma aureolatum*	0	4	1

Amblyomma ovale	*Amblyomma ovale*	1	4	4
Amblyomma ovale	*Amblyomma ovale*	1	4	4
Amblyomma ovale	*Amblyomma ovale*	1	4	4
Amblyomma ovale	*Amblyomma ovale*	1	4	4
Amblyomma ovale	*Amblyomma ovale*	1	4	4
Amblyomma sculptum	*Amblyomma sculptum*	1	5	5
Amblyomma sculptum	*Amblyomma sculptum*	1	5	5
Amblyomma sculptum	*Amblyomma sculptum*	1	5	5
Amblyomma sculptum	*Amblyomma sculptum*	1	5	5
Amblyomma sculptum	*Amblyomma dubitatum*	0	5	3
Amblyomma sculptum	*Amblyomma dubitatum*	0	5	3
Amblyomma sculptum	*Amblyomma dubitatum*	0	5	3
Amblyomma sculptum	*Amblyomma sculptum*	1	5	5
Amblyomma sculptum	*Amblyomma sculptum*	1	5	5
Amblyomma sculptum	*Amblyomma sculptum*	1	5	5
Amblyomma sculptum	*Amblyomma sculptum*	1	5	5
Amblyomma sculptum	*Amblyomma sculptum*	1	5	5
Amblyomma sculptum	*Amblyomma sculptum*	1	5	5
Amblyomma sculptum	*Amblyomma sculptum*	1	5	5
Amblyomma sculptum	*Amblyomma sculptum*	1	5	5
Amblyomma sculptum	*Amblyomma sculptum*	1	5	5
Amblyomma sculptum	*Amblyomma sculptum*	1	5	5
Amblyomma triste	*Amblyomma triste*	1	6	6
Amblyomma triste	*Amblyomma triste*	1	6	6
Amblyomma triste	*Amblyomma triste*	1	6	6
Amblyomma triste	*Amblyomma triste*	1	6	6
Amblyomma triste	*Amblyomma triste*	1	6	6
Amblyomma triste	*Amblyomma aureolatum*	0	6	1
Amblyomma triste	*Amblyomma triste*	1	6	6
Amblyomma triste	*Amblyomma triste*	1	6	6
Amblyomma triste	*Amblyomma dubitatum*	0	6	3
Amblyomma triste	*Amblyomma triste*	1	6	6
Amblyomma triste	*Amblyomma triste*	1	6	6
Amblyomma triste	*Amblyomma triste*	1	6	6
Amblyomma triste	*Amblyomma triste*	1	6	6
Amblyomma triste	*Amblyomma triste*	1	6	6
Amblyomma triste	*Amblyomma triste*	1	6	6
Amblyomma triste	*Amblyomma ovale*	0	6	4
Amblyomma aureolatum	*Amblyomma aureolatum*	1	1	1
Amblyomma aureolatum	*Amblyomma aureolatum*	1	1	1
Amblyomma aureolatum	*Amblyomma aureolatum*	1	1	1
Amblyomma aureolatum	*Amblyomma aureolatum*	1	1	1
Amblyomma aureolatum	*Amblyomma aureolatum*	1	1	1
Amblyomma aureolatum	*Amblyomma aureolatum*	1	1	1
Amblyomma aureolatum	*Amblyomma aureolatum*	1	1	1
Amblyomma aureolatum	*Amblyomma aureolatum*	1	1	1
Amblyomma aureolatum	*Amblyomma aureolatum*	1	1	1
Amblyomma aureolatum	*Amblyomma dubitatum*	0	1	3
Amblyomma aureolatum	*Amblyomma aureolatum*	1	1	1
Amblyomma aureolatum	*Amblyomma aureolatum*	1	1	1
Amblyomma cajennense	*Amblyomma cajennense*	1	2	2
Amblyomma cajennense	*Amblyomma cajennense*	1	2	2
Amblyomma cajennense	*Amblyomma triste*	0	2	6
Amblyomma cajennense	*Amblyomma cajennense*	1	2	2
Amblyomma cajennense	*Amblyomma cajennense*	1	2	2
Amblyomma cajennense	*Amblyomma cajennense*	1	2	2
Amblyomma cajennense	*Amblyomma dubitatum*	0	2	3
Amblyomma cajennense	*Amblyomma cajennense*	1	2	2
Amblyomma cajennense	*Amblyomma cajennense*	1	2	2
Amblyomma cajennense	*Amblyomma cajennense*	1	2	2
Amblyomma cajennense	*Amblyomma cajennense*	1	2	2
Amblyomma cajennense	*Amblyomma cajennense*	1	2	2
Amblyomma cajennense	*Amblyomma cajennense*	1	2	2
Amblyomma cajennense	*Amblyomma cajennense*	1	2	2
Amblyomma cajennense	*Amblyomma cajennense*	1	2	2
Amblyomma cajennense	*Amblyomma cajennense*	1	2	2
Amblyomma cajennense	*Amblyomma aureolatum*	0	2	1

Amblyomma dubitatum	*Amblyomma triste*	0	3	6
Amblyomma dubitatum	*Amblyomma sculptum*	0	3	5
Amblyomma dubitatum	*Amblyomma dubitatum*	1	3	3
Amblyomma dubitatum	*Amblyomma dubitatum*	1	3	3
Amblyomma dubitatum	*Amblyomma sculptum*	0	3	5
Amblyomma dubitatum	*Amblyomma aureolatum*	0	3	1
Amblyomma dubitatum	*Amblyomma sculptum*	0	3	5
Amblyomma dubitatum	*Amblyomma dubitatum*	1	3	3
Amblyomma dubitatum	*Amblyomma sculptum*	0	3	5
Amblyomma dubitatum	*Amblyomma dubitatum*	1	3	3
Amblyomma dubitatum	*Amblyomma aureolatum*	0	3	1
Amblyomma ovale	*Amblyomma ovale*	1	4	4
Amblyomma ovale	*Amblyomma ovale*	1	4	4
Amblyomma ovale	*Amblyomma ovale*	1	4	4
Amblyomma ovale	*Amblyomma ovale*	1	4	4
Amblyomma ovale	*Amblyomma aureolatum*	0	4	1
Amblyomma ovale	*Amblyomma ovale*	1	4	4
Amblyomma ovale	*Amblyomma aureolatum*	0	4	1
Amblyomma ovale	*Amblyomma ovale*	1	4	4
Amblyomma ovale	*Amblyomma ovale*	1	4	4
Amblyomma ovale	*Amblyomma ovale*	1	4	4
Amblyomma ovale	*Amblyomma ovale*	1	4	4
Amblyomma ovale	*Amblyomma ovale*	1	4	4
Amblyomma sculptum	*Amblyomma sculptum*	1	5	5
Amblyomma sculptum	*Amblyomma sculptum*	1	5	5
Amblyomma sculptum	*Amblyomma sculptum*	1	5	5
Amblyomma sculptum	*Amblyomma sculptum*	1	5	5
Amblyomma sculptum	*Amblyomma sculptum*	1	5	5
Amblyomma sculptum	*Amblyomma sculptum*	1	5	5
Amblyomma sculptum	*Amblyomma sculptum*	1	5	5
Amblyomma sculptum	*Amblyomma sculptum*	1	5	5
Amblyomma sculptum	*Amblyomma sculptum*	1	5	5
Amblyomma sculptum	*Amblyomma sculptum*	1	5	5
Amblyomma sculptum	*Amblyomma sculptum*	1	5	5
Amblyomma sculptum	*Amblyomma sculptum*	1	5	5
Amblyomma sculptum	*Amblyomma cajennense*	0	5	2
Amblyomma sculptum	*Amblyomma sculptum*	1	5	5
Amblyomma sculptum	*Amblyomma sculptum*	1	5	5
Amblyomma sculptum	*Amblyomma sculptum*	1	5	5
Amblyomma triste	*Amblyomma triste*	1	6	6
Amblyomma triste	*Amblyomma triste*	1	6	6
Amblyomma triste	*Amblyomma ovale*	0	6	4
Amblyomma triste	*Amblyomma triste*	1	6	6
Amblyomma triste	*Amblyomma triste*	1	6	6
Amblyomma triste	*Amblyomma triste*	1	6	6
Amblyomma triste	*Amblyomma triste*	1	6	6
Amblyomma triste	*Amblyomma triste*	1	6	6
Amblyomma triste	*Amblyomma triste*	1	6	6
Amblyomma triste	*Amblyomma triste*	1	6	6
Amblyomma triste	*Amblyomma triste*	1	6	6
Amblyomma triste	*Amblyomma triste*	1	6	6
Amblyomma triste	*Amblyomma triste*	1	6	6
Amblyomma triste	*Amblyomma triste*	1	6	6
Amblyomma triste	*Amblyomma triste*	1	6	6
Amblyomma triste	*Amblyomma triste*	1	6	6
Amblyomma aureolatum	*Amblyomma aureolatum*	1	1	1
Amblyomma aureolatum	*Amblyomma aureolatum*	1	1	1
Amblyomma aureolatum	*Amblyomma aureolatum*	1	1	1
Amblyomma aureolatum	*Amblyomma aureolatum*	1	1	1
Amblyomma aureolatum	*Amblyomma dubitatum*	0	1	3
Amblyomma aureolatum	*Amblyomma aureolatum*	1	1	1
Amblyomma aureolatum	*Amblyomma aureolatum*	1	1	1
Amblyomma aureolatum	*Amblyomma aureolatum*	1	1	1
Amblyomma aureolatum	*Amblyomma aureolatum*	1	1	1
Amblyomma aureolatum	*Amblyomma aureolatum*	1	1	1
Amblyomma aureolatum	*Amblyomma aureolatum*	1	1	1
Amblyomma aureolatum	*Amblyomma aureolatum*	1	1	1

Amblyomma aureolatum	*Amblyomma aureolatum*	1	1	1
Amblyomma cajennense	*Amblyomma cajennense*	1	2	2
Amblyomma cajennense	*Amblyomma cajennense*	1	2	2
Amblyomma cajennense	*Amblyomma cajennense*	1	2	2
Amblyomma cajennense	*Amblyomma cajennense*	1	2	2
Amblyomma cajennense	*Amblyomma cajennense*	1	2	2
Amblyomma cajennense	*Amblyomma cajennense*	1	2	2
Amblyomma cajennense	*Amblyomma cajennense*	1	2	2
Amblyomma cajennense	*Amblyomma cajennense*	1	2	2
Amblyomma cajennense	*Amblyomma cajennense*	1	2	2
Amblyomma cajennense	*Amblyomma cajennense*	1	2	2
Amblyomma cajennense	*Amblyomma cajennense*	1	2	2
Amblyomma cajennense	*Amblyomma triste*	0	2	6
Amblyomma cajennense	*Amblyomma cajennense*	1	2	2
Amblyomma cajennense	*Amblyomma cajennense*	1	2	2
Amblyomma cajennense	*Amblyomma cajennense*	1	2	2
Amblyomma cajennense	*Amblyomma cajennense*	1	2	2
Amblyomma cajennense	*Amblyomma cajennense*	1	2	2
Amblyomma dubitatum	*Amblyomma dubitatum*	1	3	3
Amblyomma dubitatum	*Amblyomma dubitatum*	1	3	3
Amblyomma dubitatum	*Amblyomma dubitatum*	1	3	3
Amblyomma dubitatum	*Amblyomma sculptum*	0	3	5
Amblyomma dubitatum	*Amblyomma dubitatum*	1	3	3
Amblyomma dubitatum	*Amblyomma dubitatum*	1	3	3
Amblyomma dubitatum	*Amblyomma cajennense*	0	3	2
Amblyomma dubitatum	*Amblyomma dubitatum*	1	3	3
Amblyomma dubitatum	*Amblyomma dubitatum*	1	3	3
Amblyomma dubitatum	*Amblyomma dubitatum*	1	3	3
Amblyomma dubitatum	*Amblyomma dubitatum*	1	3	3
Amblyomma ovale	*Amblyomma ovale*	1	4	4
Amblyomma ovale	*Amblyomma ovale*	1	4	4
Amblyomma ovale	*Amblyomma ovale*	1	4	4
Amblyomma ovale	*Amblyomma ovale*	1	4	4
Amblyomma ovale	*Amblyomma aureolatum*	0	4	1
Amblyomma ovale	*Amblyomma ovale*	1	4	4
Amblyomma ovale	*Amblyomma triste*	0	4	6
Amblyomma ovale	*Amblyomma aureolatum*	0	4	1
Amblyomma ovale	*Amblyomma dubitatum*	0	4	3
Amblyomma ovale	*Amblyomma ovale*	1	4	4
Amblyomma ovale	*Amblyomma ovale*	1	4	4
Amblyomma ovale	*Amblyomma ovale*	1	4	4
Amblyomma sculptum	*Amblyomma sculptum*	1	5	5
Amblyomma sculptum	*Amblyomma sculptum*	1	5	5
Amblyomma sculptum	*Amblyomma sculptum*	1	5	5
Amblyomma sculptum	*Amblyomma sculptum*	1	5	5
Amblyomma sculptum	*Amblyomma dubitatum*	0	5	3
Amblyomma sculptum	*Amblyomma sculptum*	1	5	5
Amblyomma sculptum	*Amblyomma sculptum*	1	5	5
Amblyomma sculptum	*Amblyomma sculptum*	1	5	5
Amblyomma sculptum	*Amblyomma sculptum*	1	5	5
Amblyomma sculptum	*Amblyomma sculptum*	1	5	5
Amblyomma sculptum	*Amblyomma sculptum*	1	5	5
Amblyomma sculptum	*Amblyomma sculptum*	1	5	5
Amblyomma sculptum	*Amblyomma sculptum*	1	5	5
Amblyomma sculptum	*Amblyomma sculptum*	1	5	5
Amblyomma sculptum	*Amblyomma sculptum*	1	5	5
Amblyomma sculptum	*Amblyomma sculptum*	1	5	5
Amblyomma triste	*Amblyomma triste*	1	6	6
Amblyomma triste	*Amblyomma triste*	1	6	6
Amblyomma triste	*Amblyomma triste*	1	6	6
Amblyomma triste	*Amblyomma triste*	1	6	6
Amblyomma triste	*Amblyomma triste*	1	6	6
Amblyomma triste	*Amblyomma triste*	1	6	6
Amblyomma triste	*Amblyomma triste*	1	6	6
Amblyomma triste	*Amblyomma triste*	1	6	6
Amblyomma triste	*Amblyomma triste*	1	6	6
Amblyomma triste	*Amblyomma triste*	1	6	6

Amblyomma triste	*Amblyomma triste*	1	6	6
Amblyomma triste	*Amblyomma triste*	1	6	6
Amblyomma triste	*Amblyomma triste*	1	6	6
Amblyomma triste	*Amblyomma triste*	1	6	6
Amblyomma triste	*Amblyomma triste*	1	6	6
Amblyomma aureolatum	*Amblyomma aureolatum*	1	1	1
Amblyomma aureolatum	*Amblyomma aureolatum*	1	1	1
Amblyomma aureolatum	*Amblyomma aureolatum*	1	1	1
Amblyomma aureolatum	*Amblyomma aureolatum*	1	1	1
Amblyomma aureolatum	*Amblyomma aureolatum*	1	1	1
Amblyomma aureolatum	*Amblyomma aureolatum*	1	1	1
Amblyomma aureolatum	*Amblyomma cajennense*	0	1	2
Amblyomma aureolatum	*Amblyomma aureolatum*	1	1	1
Amblyomma aureolatum	*Amblyomma aureolatum*	1	1	1
Amblyomma aureolatum	*Amblyomma aureolatum*	1	1	1
Amblyomma aureolatum	*Amblyomma aureolatum*	1	1	1
Amblyomma aureolatum	*Amblyomma aureolatum*	1	1	1
Amblyomma aureolatum	*Amblyomma aureolatum*	1	1	1
Amblyomma cajennense	*Amblyomma cajennense*	1	2	2
Amblyomma cajennense	*Amblyomma cajennense*	1	2	2
Amblyomma cajennense	*Amblyomma cajennense*	1	2	2
Amblyomma cajennense	*Amblyomma cajennense*	1	2	2
Amblyomma cajennense	*Amblyomma cajennense*	1	2	2
Amblyomma cajennense	*Amblyomma cajennense*	1	2	2
Amblyomma cajennense	*Amblyomma cajennense*	1	2	2
Amblyomma cajennense	*Amblyomma cajennense*	1	2	2
Amblyomma cajennense	*Amblyomma cajennense*	1	2	2
Amblyomma cajennense	*Amblyomma cajennense*	1	2	2
Amblyomma cajennense	*Amblyomma cajennense*	1	2	2
Amblyomma cajennense	*Amblyomma cajennense*	1	2	2
Amblyomma cajennense	*Amblyomma cajennense*	1	2	2
Amblyomma cajennense	*Amblyomma cajennense*	1	2	2
Amblyomma cajennense	*Amblyomma cajennense*	1	2	2
Amblyomma cajennense	*Amblyomma cajennense*	1	2	2
Amblyomma cajennense	*Amblyomma cajennense*	1	2	2
Amblyomma dubitatum	*Amblyomma dubitatum*	1	3	3
Amblyomma dubitatum	*Amblyomma dubitatum*	1	3	3
Amblyomma dubitatum	*Amblyomma dubitatum*	1	3	3
Amblyomma dubitatum	*Amblyomma dubitatum*	1	3	3
Amblyomma dubitatum	*Amblyomma dubitatum*	1	3	3
Amblyomma dubitatum	*Amblyomma dubitatum*	1	3	3
Amblyomma dubitatum	*Amblyomma dubitatum*	1	3	3
Amblyomma dubitatum	*Amblyomma dubitatum*	1	3	3
Amblyomma dubitatum	*Amblyomma dubitatum*	1	3	3
Amblyomma dubitatum	*Amblyomma dubitatum*	1	3	3
Amblyomma dubitatum	*Amblyomma dubitatum*	1	3	3
Amblyomma ovale	*Amblyomma ovale*	1	4	4
Amblyomma ovale	*Amblyomma ovale*	1	4	4
Amblyomma ovale	*Amblyomma ovale*	1	4	4
Amblyomma ovale	*Amblyomma ovale*	1	4	4
Amblyomma ovale	*Amblyomma aureolatum*	0	4	1
Amblyomma ovale	*Amblyomma aureolatum*	0	4	1
Amblyomma ovale	*Amblyomma aureolatum*	0	4	1
Amblyomma ovale	*Amblyomma aureolatum*	0	4	1
Amblyomma ovale	*Amblyomma aureolatum*	0	4	1
Amblyomma ovale	*Amblyomma ovale*	1	4	4
Amblyomma ovale	*Amblyomma ovale*	1	4	4
Amblyomma ovale	*Amblyomma dubitatum*	0	4	3
Amblyomma ovale	*Amblyomma ovale*	1	4	4
Amblyomma sculptum	*Amblyomma sculptum*	1	5	5
Amblyomma sculptum	*Amblyomma sculptum*	1	5	5
Amblyomma sculptum	*Amblyomma dubitatum*	0	5	3
Amblyomma sculptum	*Amblyomma cajennense*	0	5	2
Amblyomma sculptum	*Amblyomma dubitatum*	0	5	3
Amblyomma sculptum	*Amblyomma sculptum*	1	5	5
Amblyomma sculptum	*Amblyomma sculptum*	1	5	5
Amblyomma sculptum	*Amblyomma sculptum*	1	5	5

Amblyomma sculptum	*Amblyomma sculptum*	1	5	5
Amblyomma sculptum	*Amblyomma sculptum*	1	5	5
Amblyomma sculptum	*Amblyomma sculptum*	1	5	5
Amblyomma sculptum	*Amblyomma sculptum*	1	5	5
Amblyomma sculptum	*Amblyomma sculptum*	1	5	5
Amblyomma sculptum	*Amblyomma sculptum*	1	5	5
Amblyomma sculptum	*Amblyomma sculptum*	1	5	5
Amblyomma sculptum	*Amblyomma sculptum*	1	5	5
Amblyomma triste	*Amblyomma triste*	1	6	6
Amblyomma triste	*Amblyomma triste*	1	6	6
Amblyomma triste	*Amblyomma triste*	1	6	6
Amblyomma triste	*Amblyomma triste*	1	6	6
Amblyomma triste	*Amblyomma triste*	1	6	6
Amblyomma triste	*Amblyomma triste*	1	6	6
Amblyomma triste	*Amblyomma triste*	1	6	6
Amblyomma triste	*Amblyomma triste*	1	6	6
Amblyomma triste	*Amblyomma triste*	1	6	6
Amblyomma triste	*Amblyomma triste*	1	6	6
Amblyomma triste	*Amblyomma triste*	1	6	6
Amblyomma triste	*Amblyomma aureolatum*	0	6	1
Amblyomma triste	*Amblyomma triste*	1	6	6
Amblyomma triste	*Amblyomma triste*	1	6	6
Amblyomma triste	*Amblyomma triste*	1	6	6
Amblyomma triste	*Amblyomma triste*	1	6	6
Amblyomma aureolatum	*Amblyomma aureolatum*	1	1	1
Amblyomma aureolatum	*Amblyomma aureolatum*	1	1	1
Amblyomma aureolatum	*Amblyomma aureolatum*	1	1	1
Amblyomma aureolatum	*Amblyomma aureolatum*	1	1	1
Amblyomma aureolatum	*Amblyomma aureolatum*	1	1	1
Amblyomma aureolatum	*Amblyomma aureolatum*	1	1	1
Amblyomma aureolatum	*Amblyomma aureolatum*	1	1	1
Amblyomma aureolatum	*Amblyomma aureolatum*	1	1	1
Amblyomma aureolatum	*Amblyomma aureolatum*	1	1	1
Amblyomma aureolatum	*Amblyomma aureolatum*	1	1	1
Amblyomma aureolatum	*Amblyomma aureolatum*	1	1	1
Amblyomma aureolatum	*Amblyomma aureolatum*	1	1	1
Amblyomma cajennense	*Amblyomma cajennense*	1	2	2
Amblyomma cajennense	*Amblyomma cajennense*	1	2	2
Amblyomma cajennense	*Amblyomma cajennense*	1	2	2
Amblyomma cajennense	*Amblyomma cajennense*	1	2	2
Amblyomma cajennense	*Amblyomma cajennense*	1	2	2
Amblyomma cajennense	*Amblyomma cajennense*	1	2	2
Amblyomma cajennense	*Amblyomma cajennense*	1	2	2
Amblyomma cajennense	*Amblyomma cajennense*	1	2	2
Amblyomma cajennense	*Amblyomma cajennense*	1	2	2
Amblyomma cajennense	*Amblyomma cajennense*	1	2	2
Amblyomma cajennense	*Amblyomma cajennense*	1	2	2
Amblyomma cajennense	*Amblyomma cajennense*	1	2	2
Amblyomma cajennense	*Amblyomma cajennense*	1	2	2
Amblyomma cajennense	*Amblyomma cajennense*	1	2	2
Amblyomma cajennense	*Amblyomma cajennense*	1	2	2
Amblyomma cajennense	*Amblyomma cajennense*	1	2	2
Amblyomma dubitatum	*Amblyomma dubitatum*	1	3	3
Amblyomma dubitatum	*Amblyomma dubitatum*	1	3	3
Amblyomma dubitatum	*Amblyomma dubitatum*	1	3	3
Amblyomma dubitatum	*Amblyomma dubitatum*	1	3	3
Amblyomma dubitatum	*Amblyomma dubitatum*	1	3	3
Amblyomma dubitatum	*Amblyomma dubitatum*	1	3	3
Amblyomma dubitatum	*Amblyomma dubitatum*	1	3	3
Amblyomma dubitatum	*Amblyomma dubitatum*	1	3	3
Amblyomma dubitatum	*Amblyomma dubitatum*	1	3	3
Amblyomma dubitatum	*Amblyomma triste*	0	3	6
Amblyomma ovale	*Amblyomma ovale*	1	4	4
Amblyomma ovale	*Amblyomma ovale*	1	4	4
Amblyomma ovale	*Amblyomma aureolatum*	0	4	1
Amblyomma ovale	*Amblyomma ovale*	1	4	4
Amblyomma ovale	*Amblyomma ovale*	1	4	4

Amblyomma ovale	*Amblyomma ovale*	1	4	4
Amblyomma ovale	*Amblyomma ovale*	1	4	4
Amblyomma ovale	*Amblyomma ovale*	1	4	4
Amblyomma ovale	*Amblyomma cajennense*	0	4	2
Amblyomma ovale	*Amblyomma aureolatum*	0	4	1
Amblyomma ovale	*Amblyomma triste*	0	4	6
Amblyomma ovale	*Amblyomma ovale*	1	4	4
Amblyomma sculptum	*Amblyomma sculptum*	1	5	5
Amblyomma sculptum	*Amblyomma sculptum*	1	5	5
Amblyomma sculptum	*Amblyomma ovale*	0	5	4
Amblyomma sculptum	*Amblyomma dubitatum*	0	5	3
Amblyomma sculptum	*Amblyomma dubitatum*	0	5	3
Amblyomma sculptum	*Amblyomma aureolatum*	0	5	1
Amblyomma sculptum	*Amblyomma sculptum*	1	5	5
Amblyomma sculptum	*Amblyomma sculptum*	1	5	5
Amblyomma sculptum	*Amblyomma sculptum*	1	5	5
Amblyomma sculptum	*Amblyomma sculptum*	1	5	5
Amblyomma sculptum	*Amblyomma sculptum*	1	5	5
Amblyomma sculptum	*Amblyomma sculptum*	1	5	5
Amblyomma sculptum	*Amblyomma sculptum*	1	5	5
Amblyomma sculptum	*Amblyomma sculptum*	1	5	5
Amblyomma sculptum	*Amblyomma sculptum*	1	5	5
Amblyomma triste	*Amblyomma triste*	1	6	6
Amblyomma triste	*Amblyomma triste*	1	6	6
Amblyomma triste	*Amblyomma triste*	1	6	6
Amblyomma triste	*Amblyomma triste*	1	6	6
Amblyomma triste	*Amblyomma triste*	1	6	6
Amblyomma triste	*Amblyomma triste*	1	6	6
Amblyomma triste	*Amblyomma triste*	1	6	6
Amblyomma triste	*Amblyomma triste*	1	6	6
Amblyomma triste	*Amblyomma triste*	1	6	6
Amblyomma triste	*Amblyomma triste*	1	6	6
Amblyomma triste	*Amblyomma triste*	1	6	6
Amblyomma triste	*Amblyomma triste*	1	6	6
Amblyomma triste	*Amblyomma triste*	1	6	6
Amblyomma triste	*Amblyomma triste*	1	6	6
Amblyomma triste	*Amblyomma aureolatum*	0	6	1
Amblyomma triste	*Amblyomma triste*	1	6	6
Amblyomma aureolatum	*Amblyomma aureolatum*	1	1	1
Amblyomma aureolatum	*Amblyomma aureolatum*	1	1	1
Amblyomma aureolatum	*Amblyomma aureolatum*	1	1	1
Amblyomma aureolatum	*Amblyomma ovale*	0	1	4
Amblyomma aureolatum	*Amblyomma aureolatum*	1	1	1
Amblyomma aureolatum	*Amblyomma aureolatum*	1	1	1
Amblyomma aureolatum	*Amblyomma aureolatum*	1	1	1
Amblyomma aureolatum	*Amblyomma aureolatum*	1	1	1
Amblyomma aureolatum	*Amblyomma aureolatum*	1	1	1
Amblyomma aureolatum	*Amblyomma aureolatum*	1	1	1
Amblyomma aureolatum	*Amblyomma aureolatum*	1	1	1
Amblyomma aureolatum	*Amblyomma aureolatum*	1	1	1
Amblyomma cajennense	*Amblyomma cajennense*	1	2	2
Amblyomma cajennense	*Amblyomma cajennense*	1	2	2
Amblyomma cajennense	*Amblyomma cajennense*	1	2	2
Amblyomma cajennense	*Amblyomma cajennense*	1	2	2
Amblyomma cajennense	*Amblyomma cajennense*	1	2	2
Amblyomma cajennense	*Amblyomma cajennense*	1	2	2
Amblyomma cajennense	*Amblyomma cajennense*	1	2	2
Amblyomma cajennense	*Amblyomma cajennense*	1	2	2
Amblyomma cajennense	*Amblyomma cajennense*	1	2	2
Amblyomma cajennense	*Amblyomma dubitatum*	0	2	3
Amblyomma cajennense	*Amblyomma cajennense*	1	2	2
Amblyomma cajennense	*Amblyomma ovale*	0	2	4
Amblyomma cajennense	*Amblyomma cajennense*	1	2	2
Amblyomma cajennense	*Amblyomma cajennense*	1	2	2
Amblyomma cajennense	*Amblyomma cajennense*	1	2	2
Amblyomma cajennense	*Amblyomma cajennense*	1	2	2
Amblyomma dubitatum	*Amblyomma dubitatum*	1	3	3

Amblyomma dubitatum	*Amblyomma dubitatum*	1	3	3
Amblyomma dubitatum	*Amblyomma dubitatum*	1	3	3
Amblyomma dubitatum	*Amblyomma dubitatum*	1	3	3
Amblyomma dubitatum	*Amblyomma dubitatum*	1	3	3
Amblyomma dubitatum	*Amblyomma dubitatum*	1	3	3
Amblyomma dubitatum	*Amblyomma dubitatum*	1	3	3
Amblyomma dubitatum	*Amblyomma dubitatum*	1	3	3
Amblyomma dubitatum	*Amblyomma dubitatum*	1	3	3
Amblyomma dubitatum	*Amblyomma dubitatum*	1	3	3
Amblyomma dubitatum	*Amblyomma dubitatum*	1	3	3
Amblyomma ovale	*Amblyomma ovale*	1	4	4
Amblyomma ovale	*Amblyomma ovale*	1	4	4
Amblyomma ovale	*Amblyomma ovale*	1	4	4
Amblyomma ovale	*Amblyomma ovale*	1	4	4
Amblyomma ovale	*Amblyomma ovale*	1	4	4
Amblyomma ovale	*Amblyomma ovale*	1	4	4
Amblyomma ovale	*Amblyomma ovale*	1	4	4
Amblyomma ovale	*Amblyomma ovale*	1	4	4
Amblyomma ovale	*Amblyomma ovale*	1	4	4
Amblyomma ovale	*Amblyomma ovale*	1	4	4
Amblyomma ovale	*Amblyomma ovale*	1	4	4
Amblyomma ovale	*Amblyomma ovale*	1	4	4
Amblyomma sculptum	*Amblyomma ovale*	0	5	4
Amblyomma sculptum	*Amblyomma sculptum*	1	5	5
Amblyomma sculptum	*Amblyomma sculptum*	1	5	5
Amblyomma sculptum	*Amblyomma sculptum*	1	5	5
Amblyomma sculptum	*Amblyomma sculptum*	1	5	5
Amblyomma sculptum	*Amblyomma dubitatum*	0	5	3
Amblyomma sculptum	*Amblyomma sculptum*	1	5	5
Amblyomma sculptum	*Amblyomma sculptum*	1	5	5
Amblyomma sculptum	*Amblyomma sculptum*	1	5	5
Amblyomma sculptum	*Amblyomma sculptum*	1	5	5
Amblyomma sculptum	*Amblyomma sculptum*	1	5	5
Amblyomma sculptum	*Amblyomma sculptum*	1	5	5
Amblyomma sculptum	*Amblyomma sculptum*	1	5	5
Amblyomma sculptum	*Amblyomma sculptum*	1	5	5
Amblyomma triste	*Amblyomma triste*	1	6	6
Amblyomma triste	*Amblyomma triste*	1	6	6
Amblyomma triste	*Amblyomma triste*	1	6	6
Amblyomma triste	*Amblyomma triste*	1	6	6
Amblyomma triste	*Amblyomma triste*	1	6	6
Amblyomma triste	*Amblyomma triste*	1	6	6
Amblyomma triste	*Amblyomma triste*	1	6	6
Amblyomma triste	*Amblyomma aureolatum*	0	6	1
Amblyomma triste	*Amblyomma triste*	1	6	6
Amblyomma triste	*Amblyomma triste*	1	6	6
Amblyomma triste	*Amblyomma triste*	1	6	6
Amblyomma triste	*Amblyomma triste*	1	6	6
Amblyomma triste	*Amblyomma triste*	1	6	6
Amblyomma triste	*Amblyomma triste*	1	6	6
Amblyomma triste	*Amblyomma triste*	1	6	6
Amblyomma aureolatum	*Amblyomma aureolatum*	1	1	1
Amblyomma aureolatum	*Amblyomma triste*	0	1	6
Amblyomma aureolatum	*Amblyomma aureolatum*	1	1	1
Amblyomma aureolatum	*Amblyomma aureolatum*	1	1	1
Amblyomma aureolatum	*Amblyomma ovale*	0	1	4
Amblyomma aureolatum	*Amblyomma aureolatum*	1	1	1
Amblyomma aureolatum	*Amblyomma aureolatum*	1	1	1
Amblyomma aureolatum	*Amblyomma aureolatum*	1	1	1
Amblyomma aureolatum	*Amblyomma aureolatum*	1	1	1
Amblyomma aureolatum	*Amblyomma aureolatum*	1	1	1
Amblyomma aureolatum	*Amblyomma aureolatum*	1	1	1
Amblyomma cajennense	*Amblyomma cajennense*	1	2	2
Amblyomma cajennense	*Amblyomma cajennense*	1	2	2
Amblyomma cajennense	*Amblyomma cajennense*	1	2	2
Amblyomma cajennense	*Amblyomma cajennense*	1	2	2
Amblyomma cajennense	*Amblyomma cajennense*	1	2	2

Amblyomma cajennense	*Amblyomma cajennense*	1	2	2
Amblyomma cajennense	*Amblyomma cajennense*	1	2	2
Amblyomma cajennense	*Amblyomma cajennense*	1	2	2
Amblyomma cajennense	*Amblyomma cajennense*	1	2	2
Amblyomma cajennense	*Amblyomma cajennense*	1	2	2
Amblyomma cajennense	*Amblyomma cajennense*	1	2	2
Amblyomma cajennense	*Amblyomma cajennense*	1	2	2
Amblyomma cajennense	*Amblyomma cajennense*	1	2	2
Amblyomma cajennense	*Amblyomma cajennense*	1	2	2
Amblyomma cajennense	*Amblyomma cajennense*	1	2	2
Amblyomma cajennense	*Amblyomma cajennense*	1	2	2
Amblyomma dubitatum	*Amblyomma dubitatum*	1	3	3
Amblyomma dubitatum	*Amblyomma dubitatum*	1	3	3
Amblyomma dubitatum	*Amblyomma dubitatum*	1	3	3
Amblyomma dubitatum	*Amblyomma dubitatum*	1	3	3
Amblyomma dubitatum	*Amblyomma aureolatum*	0	3	1
Amblyomma dubitatum	*Amblyomma dubitatum*	1	3	3
Amblyomma dubitatum	*Amblyomma sculptum*	0	3	5
Amblyomma dubitatum	*Amblyomma dubitatum*	1	3	3
Amblyomma dubitatum	*Amblyomma dubitatum*	1	3	3
Amblyomma dubitatum	*Amblyomma dubitatum*	1	3	3
Amblyomma ovale	*Amblyomma ovale*	1	4	4
Amblyomma ovale	*Amblyomma ovale*	1	4	4
Amblyomma ovale	*Amblyomma ovale*	1	4	4
Amblyomma ovale	*Amblyomma ovale*	1	4	4
Amblyomma ovale	*Amblyomma ovale*	1	4	4
Amblyomma ovale	*Amblyomma ovale*	1	4	4
Amblyomma ovale	*Amblyomma ovale*	1	4	4
Amblyomma ovale	*Amblyomma ovale*	1	4	4
Amblyomma ovale	*Amblyomma ovale*	1	4	4
Amblyomma ovale	*Amblyomma ovale*	1	4	4
Amblyomma ovale	*Amblyomma ovale*	1	4	4
Amblyomma ovale	*Amblyomma ovale*	1	4	4
Amblyomma sculptum	*Amblyomma sculptum*	1	5	5
Amblyomma sculptum	*Amblyomma sculptum*	1	5	5
Amblyomma sculptum	*Amblyomma sculptum*	1	5	5
Amblyomma sculptum	*Amblyomma sculptum*	1	5	5
Amblyomma sculptum	*Amblyomma sculptum*	1	5	5
Amblyomma sculptum	*Amblyomma sculptum*	1	5	5
Amblyomma sculptum	*Amblyomma sculptum*	1	5	5
Amblyomma sculptum	*Amblyomma sculptum*	1	5	5
Amblyomma sculptum	*Amblyomma sculptum*	1	5	5
Amblyomma sculptum	*Amblyomma sculptum*	1	5	5
Amblyomma sculptum	*Amblyomma sculptum*	1	5	5
Amblyomma sculptum	*Amblyomma sculptum*	1	5	5
Amblyomma sculptum	*Amblyomma sculptum*	1	5	5
Amblyomma sculptum	*Amblyomma sculptum*	1	5	5
Amblyomma sculptum	*Amblyomma sculptum*	1	5	5
Amblyomma triste	*Amblyomma triste*	1	6	6
Amblyomma triste	*Amblyomma triste*	1	6	6
Amblyomma triste	*Amblyomma triste*	1	6	6
Amblyomma triste	*Amblyomma triste*	1	6	6
Amblyomma triste	*Amblyomma triste*	1	6	6
Amblyomma triste	*Amblyomma triste*	1	6	6
Amblyomma triste	*Amblyomma cajennense*	0	6	2
Amblyomma triste	*Amblyomma triste*	1	6	6
Amblyomma triste	*Amblyomma triste*	1	6	6
Amblyomma triste	*Amblyomma triste*	1	6	6
Amblyomma triste	*Amblyomma triste*	1	6	6
Amblyomma triste	*Amblyomma triste*	1	6	6
Amblyomma triste	*Amblyomma triste*	1	6	6
Amblyomma triste	*Amblyomma triste*	1	6	6
Amblyomma triste	*Amblyomma triste*	1	6	6
Amblyomma aureolatum	*Amblyomma aureolatum*	1	1	1
Amblyomma aureolatum	*Amblyomma aureolatum*	1	1	1
Amblyomma aureolatum	*Amblyomma aureolatum*	1	1	1
Amblyomma aureolatum	*Amblyomma triste*	0	1	6

Amblyomma aureolatum	*Amblyomma aureolatum*	1	1	1
Amblyomma aureolatum	*Amblyomma aureolatum*	1	1	1
Amblyomma aureolatum	*Amblyomma aureolatum*	1	1	1
Amblyomma aureolatum	*Amblyomma aureolatum*	1	1	1
Amblyomma aureolatum	*Amblyomma aureolatum*	1	1	1
Amblyomma aureolatum	*Amblyomma aureolatum*	1	1	1
Amblyomma aureolatum	*Amblyomma aureolatum*	1	1	1
Amblyomma aureolatum	*Amblyomma aureolatum*	1	1	1
Amblyomma cajennense	*Amblyomma cajennense*	1	2	2
Amblyomma cajennense	*Amblyomma cajennense*	1	2	2
Amblyomma cajennense	*Amblyomma cajennense*	1	2	2
Amblyomma cajennense	*Amblyomma cajennense*	1	2	2
Amblyomma cajennense	*Amblyomma cajennense*	1	2	2
Amblyomma cajennense	*Amblyomma cajennense*	1	2	2
Amblyomma cajennense	*Amblyomma cajennense*	1	2	2
Amblyomma cajennense	*Amblyomma cajennense*	1	2	2
Amblyomma cajennense	*Amblyomma cajennense*	1	2	2
Amblyomma cajennense	*Amblyomma cajennense*	1	2	2
Amblyomma cajennense	*Amblyomma cajennense*	1	2	2
Amblyomma cajennense	*Amblyomma cajennense*	1	2	2
Amblyomma cajennense	*Amblyomma triste*	0	2	6
Amblyomma cajennense	*Amblyomma cajennense*	1	2	2
Amblyomma cajennense	*Amblyomma cajennense*	1	2	2
Amblyomma cajennense	*Amblyomma sculptum*	0	2	5
Amblyomma cajennense	*Amblyomma cajennense*	1	2	2
Amblyomma dubitatum	*Amblyomma ovale*	0	3	4
Amblyomma dubitatum	*Amblyomma dubitatum*	1	3	3
Amblyomma dubitatum	*Amblyomma dubitatum*	1	3	3
Amblyomma dubitatum	*Amblyomma dubitatum*	1	3	3
Amblyomma dubitatum	*Amblyomma dubitatum*	1	3	3
Amblyomma dubitatum	*Amblyomma sculptum*	0	3	5
Amblyomma dubitatum	*Amblyomma dubitatum*	1	3	3
Amblyomma dubitatum	*Amblyomma dubitatum*	1	3	3
Amblyomma dubitatum	*Amblyomma dubitatum*	1	3	3
Amblyomma dubitatum	*Amblyomma sculptum*	0	3	5
Amblyomma dubitatum	*Amblyomma dubitatum*	1	3	3
Amblyomma ovale	*Amblyomma ovale*	1	4	4
Amblyomma ovale	*Amblyomma ovale*	1	4	4
Amblyomma ovale	*Amblyomma ovale*	1	4	4
Amblyomma ovale	*Amblyomma ovale*	1	4	4
Amblyomma ovale	*Amblyomma ovale*	1	4	4
Amblyomma ovale	*Amblyomma sculptum*	0	4	5
Amblyomma ovale	*Amblyomma ovale*	1	4	4
Amblyomma ovale	*Amblyomma ovale*	1	4	4
Amblyomma ovale	*Amblyomma ovale*	1	4	4
Amblyomma ovale	*Amblyomma dubitatum*	0	4	3
Amblyomma ovale	*Amblyomma dubitatum*	0	4	3
Amblyomma ovale	*Amblyomma ovale*	1	4	4
Amblyomma sculptum	*Amblyomma sculptum*	1	5	5
Amblyomma sculptum	*Amblyomma triste*	0	5	6
Amblyomma sculptum	*Amblyomma sculptum*	1	5	5
Amblyomma sculptum	*Amblyomma sculptum*	1	5	5
Amblyomma sculptum	*Amblyomma sculptum*	1	5	5
Amblyomma sculptum	*Amblyomma sculptum*	1	5	5
Amblyomma sculptum	*Amblyomma sculptum*	1	5	5
Amblyomma sculptum	*Amblyomma sculptum*	1	5	5
Amblyomma sculptum	*Amblyomma sculptum*	1	5	5
Amblyomma sculptum	*Amblyomma sculptum*	1	5	5
Amblyomma sculptum	*Amblyomma sculptum*	1	5	5
Amblyomma sculptum	*Amblyomma sculptum*	1	5	5
Amblyomma sculptum	*Amblyomma sculptum*	1	5	5
Amblyomma sculptum	*Amblyomma sculptum*	1	5	5
Amblyomma sculptum	*Amblyomma sculptum*	1	5	5
Amblyomma triste	*Amblyomma triste*	1	6	6
Amblyomma triste	*Amblyomma triste*	1	6	6
Amblyomma triste	*Amblyomma cajennense*	0	6	2
Amblyomma triste	*Amblyomma triste*	1	6	6

Amblyomma triste	*Amblyomma triste*	1	6	6
Amblyomma triste	*Amblyomma triste*	1	6	6
Amblyomma triste	*Amblyomma triste*	1	6	6
Amblyomma triste	*Amblyomma triste*	1	6	6
Amblyomma triste	*Amblyomma triste*	1	6	6
Amblyomma triste	*Amblyomma triste*	1	6	6
Amblyomma triste	*Amblyomma triste*	1	6	6
Amblyomma triste	*Amblyomma triste*	1	6	6
Amblyomma triste	*Amblyomma triste*	1	6	6
Amblyomma triste	*Amblyomma triste*	1	6	6
Amblyomma triste	*Amblyomma triste*	1	6	6
Amblyomma triste	*Amblyomma triste*	1	6	6
Amblyomma aureolatum	*Amblyomma ovale*	0	1	4
Amblyomma aureolatum	*Amblyomma aureolatum*	1	1	1
Amblyomma aureolatum	*Amblyomma aureolatum*	1	1	1
Amblyomma aureolatum	*Amblyomma aureolatum*	1	1	1
Amblyomma aureolatum	*Amblyomma ovale*	0	1	4
Amblyomma aureolatum	*Amblyomma aureolatum*	1	1	1
Amblyomma aureolatum	*Amblyomma aureolatum*	1	1	1
Amblyomma aureolatum	*Amblyomma aureolatum*	1	1	1
Amblyomma aureolatum	*Amblyomma aureolatum*	1	1	1
Amblyomma aureolatum	*Amblyomma aureolatum*	1	1	1
Amblyomma aureolatum	*Amblyomma aureolatum*	1	1	1
Amblyomma cajennense	*Amblyomma cajennense*	1	2	2
Amblyomma cajennense	*Amblyomma triste*	0	2	6
Amblyomma cajennense	*Amblyomma cajennense*	1	2	2
Amblyomma cajennense	*Amblyomma cajennense*	1	2	2
Amblyomma cajennense	*Amblyomma cajennense*	1	2	2
Amblyomma cajennense	*Amblyomma cajennense*	1	2	2
Amblyomma cajennense	*Amblyomma cajennense*	1	2	2
Amblyomma cajennense	*Amblyomma cajennense*	1	2	2
Amblyomma cajennense	*Amblyomma cajennense*	1	2	2
Amblyomma cajennense	*Amblyomma cajennense*	1	2	2
Amblyomma cajennense	*Amblyomma cajennense*	1	2	2
Amblyomma cajennense	*Amblyomma cajennense*	1	2	2
Amblyomma cajennense	*Amblyomma cajennense*	1	2	2
Amblyomma cajennense	*Amblyomma cajennense*	1	2	2
Amblyomma cajennense	*Amblyomma cajennense*	1	2	2
Amblyomma cajennense	*Amblyomma cajennense*	1	2	2
Amblyomma cajennense	*Amblyomma cajennense*	1	2	2
Amblyomma dubitatum	*Amblyomma triste*	0	3	6
Amblyomma dubitatum	*Amblyomma aureolatum*	0	3	1
Amblyomma dubitatum	*Amblyomma dubitatum*	1	3	3
Amblyomma dubitatum	*Amblyomma dubitatum*	1	3	3
Amblyomma dubitatum	*Amblyomma dubitatum*	1	3	3
Amblyomma dubitatum	*Amblyomma dubitatum*	1	3	3
Amblyomma dubitatum	*Amblyomma dubitatum*	1	3	3
Amblyomma dubitatum	*Amblyomma ovale*	0	3	4
Amblyomma dubitatum	*Amblyomma dubitatum*	1	3	3
Amblyomma dubitatum	*Amblyomma dubitatum*	1	3	3
Amblyomma ovale	*Amblyomma ovale*	1	4	4
Amblyomma ovale	*Amblyomma ovale*	1	4	4
Amblyomma ovale	*Amblyomma ovale*	1	4	4
Amblyomma ovale	*Amblyomma ovale*	1	4	4
Amblyomma ovale	*Amblyomma ovale*	1	4	4
Amblyomma ovale	*Amblyomma ovale*	1	4	4
Amblyomma ovale	*Amblyomma ovale*	1	4	4
Amblyomma ovale	*Amblyomma ovale*	1	4	4
Amblyomma ovale	*Amblyomma ovale*	1	4	4
Amblyomma ovale	*Amblyomma ovale*	1	4	4
Amblyomma ovale	*Amblyomma ovale*	1	4	4
Amblyomma ovale	*Amblyomma ovale*	1	4	4
Amblyomma sculptum	*Amblyomma sculptum*	1	5	5
Amblyomma sculptum	*Amblyomma sculptum*	1	5	5
Amblyomma sculptum	*Amblyomma sculptum*	1	5	5
Amblyomma sculptum	*Amblyomma sculptum*	1	5	5
Amblyomma sculptum	*Amblyomma sculptum*	1	5	5

Amblyomma sculptum	*Amblyomma cajennense*	0	5	2
Amblyomma sculptum	*Amblyomma sculptum*	1	5	5
Amblyomma sculptum	*Amblyomma sculptum*	1	5	5
Amblyomma sculptum	*Amblyomma sculptum*	1	5	5
Amblyomma sculptum	*Amblyomma sculptum*	1	5	5
Amblyomma sculptum	*Amblyomma sculptum*	1	5	5
Amblyomma sculptum	*Amblyomma sculptum*	1	5	5
Amblyomma sculptum	*Amblyomma sculptum*	1	5	5
Amblyomma sculptum	*Amblyomma ovale*	0	5	4
Amblyomma sculptum	*Amblyomma triste*	0	5	6
Amblyomma triste	*Amblyomma triste*	1	6	6
Amblyomma triste	*Amblyomma ovale*	0	6	4
Amblyomma triste	*Amblyomma triste*	1	6	6
Amblyomma triste	*Amblyomma triste*	1	6	6
Amblyomma triste	*Amblyomma triste*	1	6	6
Amblyomma triste	*Amblyomma triste*	1	6	6
Amblyomma triste	*Amblyomma triste*	1	6	6
Amblyomma triste	*Amblyomma triste*	1	6	6
Amblyomma triste	*Amblyomma triste*	1	6	6
Amblyomma triste	*Amblyomma triste*	1	6	6
Amblyomma triste	*Amblyomma triste*	1	6	6
Amblyomma triste	*Amblyomma triste*	1	6	6
Amblyomma triste	*Amblyomma triste*	1	6	6
Amblyomma triste	*Amblyomma triste*	1	6	6
Amblyomma triste	*Amblyomma triste*	1	6	6
Amblyomma aureolatum	*Amblyomma aureolatum*	1	1	1
Amblyomma aureolatum	*Amblyomma aureolatum*	1	1	1
Amblyomma aureolatum	*Amblyomma aureolatum*	1	1	1
Amblyomma aureolatum	*Amblyomma aureolatum*	1	1	1
Amblyomma aureolatum	*Amblyomma aureolatum*	1	1	1
Amblyomma aureolatum	*Amblyomma aureolatum*	1	1	1
Amblyomma aureolatum	*Amblyomma aureolatum*	1	1	1
Amblyomma aureolatum	*Amblyomma aureolatum*	1	1	1
Amblyomma aureolatum	*Amblyomma aureolatum*	1	1	1
Amblyomma aureolatum	*Amblyomma aureolatum*	1	1	1
Amblyomma cajennense	*Amblyomma triste*	0	2	6
Amblyomma cajennense	*Amblyomma cajennense*	1	2	2
Amblyomma cajennense	*Amblyomma cajennense*	1	2	2
Amblyomma cajennense	*Amblyomma cajennense*	1	2	2
Amblyomma cajennense	*Amblyomma cajennense*	1	2	2
Amblyomma cajennense	*Amblyomma cajennense*	1	2	2
Amblyomma cajennense	*Amblyomma cajennense*	1	2	2
Amblyomma cajennense	*Amblyomma cajennense*	1	2	2
Amblyomma cajennense	*Amblyomma ovale*	0	2	4
Amblyomma cajennense	*Amblyomma cajennense*	1	2	2
Amblyomma cajennense	*Amblyomma dubitatum*	0	2	3
Amblyomma cajennense	*Amblyomma cajennense*	1	2	2
Amblyomma cajennense	*Amblyomma cajennense*	1	2	2
Amblyomma cajennense	*Amblyomma triste*	0	2	6
Amblyomma cajennense	*Amblyomma cajennense*	1	2	2
Amblyomma cajennense	*Amblyomma dubitatum*	0	2	3
Amblyomma dubitatum	*Amblyomma dubitatum*	1	3	3
Amblyomma dubitatum	*Amblyomma dubitatum*	1	3	3
Amblyomma dubitatum	*Amblyomma dubitatum*	1	3	3
Amblyomma dubitatum	*Amblyomma dubitatum*	1	3	3
Amblyomma dubitatum	*Amblyomma dubitatum*	1	3	3
Amblyomma dubitatum	*Amblyomma dubitatum*	1	3	3
Amblyomma dubitatum	*Amblyomma dubitatum*	1	3	3
Amblyomma dubitatum	*Amblyomma dubitatum*	1	3	3
Amblyomma ovale	*Amblyomma ovale*	1	4	4
Amblyomma ovale	*Amblyomma ovale*	1	4	4
Amblyomma ovale	*Amblyomma ovale*	1	4	4
Amblyomma ovale	*Amblyomma ovale*	1	4	4
Amblyomma ovale	*Amblyomma ovale*	1	4	4
Amblyomma ovale	*Amblyomma ovale*	1	4	4
Amblyomma ovale	*Amblyomma ovale*	1	4	4
Amblyomma ovale	*Amblyomma ovale*	1	4	4

Amblyomma ovale	*Amblyomma ovale*	1	4	4
Amblyomma ovale	*Amblyomma ovale*	1	4	4
Amblyomma ovale	*Amblyomma ovale*	1	4	4
Amblyomma sculptum	*Amblyomma sculptum*	1	5	5
Amblyomma sculptum	*Amblyomma sculptum*	1	5	5
Amblyomma sculptum	*Amblyomma sculptum*	1	5	5
Amblyomma sculptum	*Amblyomma sculptum*	1	5	5
Amblyomma triste	*Amblyomma triste*	1	6	6
Amblyomma triste	*Amblyomma triste*	1	6	6
Amblyomma triste	*Amblyomma triste*	1	6	6
Amblyomma triste	*Amblyomma triste*	1	6	6
Amblyomma triste	*Amblyomma triste*	1	6	6
Amblyomma triste	*Amblyomma triste*	1	6	6
Amblyomma triste	*Amblyomma triste*	1	6	6
Amblyomma triste	*Amblyomma triste*	1	6	6
Amblyomma triste	*Amblyomma triste*	1	6	6
Amblyomma triste	*Amblyomma triste*	1	6	6
Amblyomma triste	*Amblyomma triste*	1	6	6
Amblyomma triste	*Amblyomma triste*	1	6	6
Amblyomma triste	*Amblyomma triste*	1	6	6
Amblyomma triste	*Amblyomma triste*	1	6	6
Amblyomma triste	*Amblyomma triste*	1	6	6
Amblyomma triste	*Amblyomma triste*	1	6	6
Amblyomma aureolatum	*Amblyomma aureolatum*	1	1	1
Amblyomma aureolatum	*Amblyomma aureolatum*	1	1	1
Amblyomma aureolatum	*Amblyomma aureolatum*	1	1	1
Amblyomma aureolatum	*Amblyomma aureolatum*	1	1	1
Amblyomma aureolatum	*Amblyomma aureolatum*	1	1	1
Amblyomma aureolatum	*Amblyomma aureolatum*	1	1	1
Amblyomma aureolatum	*Amblyomma aureolatum*	1	1	1
Amblyomma aureolatum	*Amblyomma aureolatum*	1	1	1
Amblyomma aureolatum	*Amblyomma aureolatum*	1	1	1
Amblyomma aureolatum	*Amblyomma triste*	0	1	6
Amblyomma aureolatum	*Amblyomma aureolatum*	1	1	1
Amblyomma cajennense	*Amblyomma cajennense*	1	2	2
Amblyomma cajennense	*Amblyomma cajennense*	1	2	2
Amblyomma cajennense	*Amblyomma cajennense*	1	2	2
Amblyomma cajennense	*Amblyomma cajennense*	1	2	2
Amblyomma cajennense	*Amblyomma cajennense*	1	2	2
Amblyomma cajennense	*Amblyomma cajennense*	1	2	2
Amblyomma cajennense	*Amblyomma cajennense*	1	2	2
Amblyomma cajennense	*Amblyomma cajennense*	1	2	2
Amblyomma cajennense	*Amblyomma cajennense*	1	2	2
Amblyomma cajennense	*Amblyomma cajennense*	1	2	2
Amblyomma cajennense	*Amblyomma cajennense*	1	2	2
Amblyomma cajennense	*Amblyomma cajennense*	1	2	2
Amblyomma cajennense	*Amblyomma cajennense*	1	2	2
Amblyomma cajennense	*Amblyomma cajennense*	1	2	2
Amblyomma cajennense	*Amblyomma cajennense*	1	2	2
Amblyomma cajennense	*Amblyomma cajennense*	1	2	2
Amblyomma dubitatum	*Amblyomma dubitatum*	1	3	3
Amblyomma dubitatum	*Amblyomma dubitatum*	1	3	3
Amblyomma dubitatum	*Amblyomma dubitatum*	1	3	3
Amblyomma dubitatum	*Amblyomma dubitatum*	1	3	3
Amblyomma dubitatum	*Amblyomma dubitatum*	1	3	3
Amblyomma dubitatum	*Amblyomma dubitatum*	1	3	3
Amblyomma dubitatum	*Amblyomma dubitatum*	1	3	3
Amblyomma dubitatum	*Amblyomma dubitatum*	1	3	3
Amblyomma dubitatum	*Amblyomma dubitatum*	1	3	3
Amblyomma ovale	*Amblyomma ovale*	1	4	4
Amblyomma ovale	*Amblyomma ovale*	1	4	4
Amblyomma ovale	*Amblyomma ovale*	1	4	4
Amblyomma ovale	*Amblyomma ovale*	1	4	4
Amblyomma ovale	*Amblyomma ovale*	1	4	4
Amblyomma ovale	*Amblyomma ovale*	1	4	4
Amblyomma ovale	*Amblyomma ovale*	1	4	4
Amblyomma ovale	*Amblyomma ovale*	1	4	4

Amblyomma ovale	*Amblyomma ovale*	1	4	4
Amblyomma ovale	*Amblyomma ovale*	1	4	4
Amblyomma ovale	*Amblyomma ovale*	1	4	4
Amblyomma sculptum	*Amblyomma sculptum*	1	5	5
Amblyomma sculptum	*Amblyomma sculptum*	1	5	5
Amblyomma sculptum	*Amblyomma sculptum*	1	5	5
Amblyomma triste	*Amblyomma triste*	1	6	6
Amblyomma triste	*Amblyomma triste*	1	6	6
Amblyomma triste	*Amblyomma triste*	1	6	6
Amblyomma triste	*Amblyomma triste*	1	6	6
Amblyomma triste	*Amblyomma triste*	1	6	6
Amblyomma triste	*Amblyomma triste*	1	6	6
Amblyomma triste	*Amblyomma triste*	1	6	6
Amblyomma triste	*Amblyomma triste*	1	6	6
Amblyomma triste	*Amblyomma dubitatum*	0	6	3
Amblyomma triste	*Amblyomma triste*	1	6	6
Amblyomma triste	*Amblyomma triste*	1	6	6
Amblyomma triste	*Amblyomma triste*	1	6	6
Amblyomma triste	*Amblyomma triste*	1	6	6
Amblyomma triste	*Amblyomma triste*	1	6	6
Amblyomma triste	*Amblyomma triste*	1	6	6
Amblyomma triste	*Amblyomma triste*	1	6	6
Amblyomma aureolatum	*Amblyomma aureolatum*	1	1	1
Amblyomma aureolatum	*Amblyomma aureolatum*	1	1	1
Amblyomma aureolatum	*Amblyomma dubitatum*	0	1	3
Amblyomma aureolatum	*Amblyomma aureolatum*	1	1	1
Amblyomma aureolatum	*Amblyomma aureolatum*	1	1	1
Amblyomma aureolatum	*Amblyomma aureolatum*	1	1	1
Amblyomma aureolatum	*Amblyomma aureolatum*	1	1	1
Amblyomma aureolatum	*Amblyomma aureolatum*	1	1	1
Amblyomma aureolatum	*Amblyomma aureolatum*	1	1	1
Amblyomma aureolatum	*Amblyomma aureolatum*	1	1	1
Amblyomma cajennense	*Amblyomma cajennense*	1	2	2
Amblyomma cajennense	*Amblyomma cajennense*	1	2	2
Amblyomma cajennense	*Amblyomma cajennense*	1	2	2
Amblyomma cajennense	*Amblyomma cajennense*	1	2	2
Amblyomma cajennense	*Amblyomma cajennense*	1	2	2
Amblyomma cajennense	*Amblyomma cajennense*	1	2	2
Amblyomma cajennense	*Amblyomma cajennense*	1	2	2
Amblyomma cajennense	*Amblyomma cajennense*	1	2	2
Amblyomma cajennense	*Amblyomma cajennense*	1	2	2
Amblyomma cajennense	*Amblyomma cajennense*	1	2	2
Amblyomma cajennense	*Amblyomma cajennense*	1	2	2
Amblyomma cajennense	*Amblyomma cajennense*	1	2	2
Amblyomma cajennense	*Amblyomma cajennense*	1	2	2
Amblyomma cajennense	*Amblyomma cajennense*	1	2	2
Amblyomma cajennense	*Amblyomma cajennense*	1	2	2
Amblyomma cajennense	*Amblyomma cajennense*	1	2	2
Amblyomma cajennense	*Amblyomma cajennense*	1	2	2
Amblyomma dubitatum	*Amblyomma sculptum*	0	3	5
Amblyomma dubitatum	*Amblyomma dubitatum*	1	3	3
Amblyomma dubitatum	*Amblyomma ovale*	0	3	4
Amblyomma dubitatum	*Amblyomma dubitatum*	1	3	3
Amblyomma dubitatum	*Amblyomma dubitatum*	1	3	3
Amblyomma dubitatum	*Amblyomma dubitatum*	1	3	3
Amblyomma dubitatum	*Amblyomma aureolatum*	0	3	1
Amblyomma dubitatum	*Amblyomma dubitatum*	1	3	3
Amblyomma dubitatum	*Amblyomma dubitatum*	1	3	3
Amblyomma ovale	*Amblyomma ovale*	1	4	4
Amblyomma ovale	*Amblyomma ovale*	1	4	4
Amblyomma ovale	*Amblyomma cajennense*	0	4	2
Amblyomma ovale	*Amblyomma aureolatum*	0	4	1
Amblyomma ovale	*Amblyomma ovale*	1	4	4
Amblyomma ovale	*Amblyomma aureolatum*	0	4	1
Amblyomma ovale	*Amblyomma ovale*	1	4	4
Amblyomma ovale	*Amblyomma ovale*	1	4	4
Amblyomma ovale	*Amblyomma ovale*	1	4	4

Amblyomma ovale	*Amblyomma ovale*	1	4	4
Amblyomma ovale	*Amblyomma ovale*	1	4	4
Amblyomma sculptum	*Amblyomma sculptum*	1	5	5
Amblyomma sculptum	*Amblyomma sculptum*	1	5	5
Amblyomma sculptum	*Amblyomma sculptum*	1	5	5
Amblyomma sculptum	*Amblyomma sculptum*	1	5	5
Amblyomma triste	*Amblyomma triste*	1	6	6
Amblyomma triste	*Amblyomma triste*	1	6	6
Amblyomma triste	*Amblyomma triste*	1	6	6
Amblyomma triste	*Amblyomma cajennense*	0	6	2
Amblyomma triste	*Amblyomma triste*	1	6	6
Amblyomma triste	*Amblyomma triste*	1	6	6
Amblyomma triste	*Amblyomma triste*	1	6	6
Amblyomma triste	*Amblyomma triste*	1	6	6
Amblyomma triste	*Amblyomma triste*	1	6	6
Amblyomma triste	*Amblyomma triste*	1	6	6
Amblyomma triste	*Amblyomma triste*	1	6	6
Amblyomma triste	*Amblyomma triste*	1	6	6
Amblyomma triste	*Amblyomma triste*	1	6	6
Amblyomma triste	*Amblyomma triste*	1	6	6
Amblyomma triste	*Amblyomma triste*	1	6	6
Amblyomma aureolatum	*Amblyomma aureolatum*	1	1	1
Amblyomma aureolatum	*Amblyomma aureolatum*	1	1	1
Amblyomma aureolatum	*Amblyomma aureolatum*	1	1	1
Amblyomma aureolatum	*Amblyomma ovale*	0	1	4
Amblyomma aureolatum	*Amblyomma aureolatum*	1	1	1
Amblyomma aureolatum	*Amblyomma aureolatum*	1	1	1
Amblyomma aureolatum	*Amblyomma aureolatum*	1	1	1
Amblyomma aureolatum	*Amblyomma aureolatum*	1	1	1
Amblyomma aureolatum	*Amblyomma ovale*	0	1	4
Amblyomma aureolatum	*Amblyomma aureolatum*	1	1	1
Amblyomma aureolatum	*Amblyomma aureolatum*	1	1	1
Amblyomma cajennense	*Amblyomma cajennense*	1	2	2
Amblyomma cajennense	*Amblyomma cajennense*	1	2	2
Amblyomma cajennense	*Amblyomma cajennense*	1	2	2
Amblyomma cajennense	*Amblyomma cajennense*	1	2	2
Amblyomma cajennense	*Amblyomma cajennense*	1	2	2
Amblyomma cajennense	*Amblyomma cajennense*	1	2	2
Amblyomma cajennense	*Amblyomma cajennense*	1	2	2
Amblyomma cajennense	*Amblyomma cajennense*	1	2	2
Amblyomma cajennense	*Amblyomma cajennense*	1	2	2
Amblyomma cajennense	*Amblyomma triste*	0	2	6
Amblyomma cajennense	*Amblyomma cajennense*	1	2	2
Amblyomma cajennense	*Amblyomma cajennense*	1	2	2
Amblyomma cajennense	*Amblyomma cajennense*	1	2	2
Amblyomma cajennense	*Amblyomma cajennense*	1	2	2
Amblyomma cajennense	*Amblyomma cajennense*	1	2	2
Amblyomma cajennense	*Amblyomma cajennense*	1	2	2
Amblyomma dubitatum	*Amblyomma aureolatum*	0	3	1
Amblyomma dubitatum	*Amblyomma aureolatum*	0	3	1
Amblyomma dubitatum	*Amblyomma triste*	0	3	6
Amblyomma dubitatum	*Amblyomma ovale*	0	3	4
Amblyomma dubitatum	*Amblyomma sculptum*	0	3	5
Amblyomma dubitatum	*Amblyomma dubitatum*	1	3	3
Amblyomma dubitatum	*Amblyomma dubitatum*	1	3	3
Amblyomma dubitatum	*Amblyomma dubitatum*	1	3	3
Amblyomma dubitatum	*Amblyomma triste*	0	3	6
Amblyomma ovale	*Amblyomma ovale*	1	4	4
Amblyomma ovale	*Amblyomma ovale*	1	4	4
Amblyomma ovale	*Amblyomma ovale*	1	4	4
Amblyomma ovale	*Amblyomma ovale*	1	4	4
Amblyomma ovale	*Amblyomma ovale*	1	4	4
Amblyomma ovale	*Amblyomma ovale*	1	4	4
Amblyomma ovale	*Amblyomma ovale*	1	4	4
Amblyomma ovale	*Amblyomma ovale*	1	4	4
Amblyomma ovale	*Amblyomma ovale*	1	4	4
Amblyomma ovale	*Amblyomma ovale*	1	4	4

Amblyomma ovale	*Amblyomma ovale*	1	4	4
Amblyomma sculptum	*Amblyomma sculptum*	1	5	5
Amblyomma sculptum	*Amblyomma sculptum*	1	5	5
Amblyomma sculptum	*Amblyomma dubitatum*	0	5	3
Amblyomma triste	*Amblyomma triste*	1	6	6
Amblyomma triste	*Amblyomma triste*	1	6	6
Amblyomma triste	*Amblyomma triste*	1	6	6
Amblyomma triste	*Amblyomma triste*	1	6	6
Amblyomma triste	*Amblyomma triste*	1	6	6
Amblyomma triste	*Amblyomma triste*	1	6	6
Amblyomma triste	*Amblyomma triste*	1	6	6
Amblyomma triste	*Amblyomma triste*	1	6	6
Amblyomma triste	*Amblyomma triste*	1	6	6
Amblyomma triste	*Amblyomma triste*	1	6	6
Amblyomma triste	*Amblyomma triste*	1	6	6
Amblyomma triste	*Amblyomma triste*	1	6	6
Amblyomma triste	*Amblyomma triste*	1	6	6
Amblyomma triste	*Amblyomma triste*	1	6	6
Amblyomma triste	*Amblyomma triste*	1	6	6
Amblyomma aureolatum	*Amblyomma aureolatum*	1	1	1
Amblyomma aureolatum	*Amblyomma aureolatum*	1	1	1
Amblyomma aureolatum	*Amblyomma aureolatum*	1	1	1
Amblyomma aureolatum	*Amblyomma aureolatum*	1	1	1
Amblyomma aureolatum	*Amblyomma aureolatum*	1	1	1
Amblyomma aureolatum	*Amblyomma aureolatum*	1	1	1
Amblyomma aureolatum	*Amblyomma aureolatum*	1	1	1
Amblyomma aureolatum	*Amblyomma aureolatum*	1	1	1
Amblyomma aureolatum	*Amblyomma aureolatum*	1	1	1
Amblyomma aureolatum	*Amblyomma aureolatum*	1	1	1
Amblyomma aureolatum	*Amblyomma aureolatum*	1	1	1
Amblyomma cajennense	*Amblyomma cajennense*	1	2	2
Amblyomma cajennense	*Amblyomma cajennense*	1	2	2
Amblyomma cajennense	*Amblyomma cajennense*	1	2	2
Amblyomma cajennense	*Amblyomma cajennense*	1	2	2
Amblyomma cajennense	*Amblyomma cajennense*	1	2	2
Amblyomma cajennense	*Amblyomma cajennense*	1	2	2
Amblyomma cajennense	*Amblyomma cajennense*	1	2	2
Amblyomma cajennense	*Amblyomma cajennense*	1	2	2
Amblyomma cajennense	*Amblyomma cajennense*	1	2	2
Amblyomma cajennense	*Amblyomma cajennense*	1	2	2
Amblyomma cajennense	*Amblyomma cajennense*	1	2	2
Amblyomma cajennense	*Amblyomma cajennense*	1	2	2
Amblyomma cajennense	*Amblyomma cajennense*	1	2	2
Amblyomma cajennense	*Amblyomma cajennense*	1	2	2
Amblyomma cajennense	*Amblyomma cajennense*	1	2	2
Amblyomma cajennense	*Amblyomma cajennense*	1	2	2
Amblyomma cajennense	*Amblyomma cajennense*	1	2	2
Amblyomma dubitatum	*Amblyomma dubitatum*	1	3	3
Amblyomma dubitatum	*Amblyomma dubitatum*	1	3	3
Amblyomma dubitatum	*Amblyomma dubitatum*	1	3	3
Amblyomma dubitatum	*Amblyomma dubitatum*	1	3	3
Amblyomma dubitatum	*Amblyomma dubitatum*	1	3	3
Amblyomma dubitatum	*Amblyomma dubitatum*	1	3	3
Amblyomma dubitatum	*Amblyomma triste*	0	3	6
Amblyomma dubitatum	*Amblyomma dubitatum*	1	3	3
Amblyomma dubitatum	*Amblyomma dubitatum*	1	3	3
Amblyomma ovale	*Amblyomma ovale*	1	4	4
Amblyomma ovale	*Amblyomma ovale*	1	4	4
Amblyomma ovale	*Amblyomma ovale*	1	4	4
Amblyomma ovale	*Amblyomma ovale*	1	4	4
Amblyomma ovale	*Amblyomma ovale*	1	4	4
Amblyomma ovale	*Amblyomma ovale*	1	4	4
Amblyomma ovale	*Amblyomma aureolatum*	0	4	1
Amblyomma ovale	*Amblyomma ovale*	1	4	4
Amblyomma ovale	*Amblyomma aureolatum*	0	4	1
Amblyomma ovale	*Amblyomma dubitatum*	0	4	3
Amblyomma ovale	*Amblyomma ovale*	1	4	4

Amblyomma sculptum	*Amblyomma sculptum*	1	5	5
Amblyomma sculptum	*Amblyomma triste*	0	5	6
Amblyomma sculptum	*Amblyomma sculptum*	1	5	5
Amblyomma triste	*Amblyomma triste*	1	6	6
Amblyomma triste	*Amblyomma triste*	1	6	6
Amblyomma triste	*Amblyomma triste*	1	6	6
Amblyomma triste	*Amblyomma triste*	1	6	6
Amblyomma triste	*Amblyomma triste*	1	6	6
Amblyomma triste	*Amblyomma triste*	1	6	6
Amblyomma triste	*Amblyomma triste*	1	6	6
Amblyomma triste	*Amblyomma triste*	1	6	6
Amblyomma triste	*Amblyomma triste*	1	6	6
Amblyomma triste	*Amblyomma triste*	1	6	6
Amblyomma triste	*Amblyomma triste*	1	6	6
Amblyomma triste	*Amblyomma triste*	1	6	6
Amblyomma triste	*Amblyomma triste*	1	6	6
Amblyomma triste	*Amblyomma triste*	1	6	6
Amblyomma triste	*Amblyomma triste*	1	6	6
Amblyomma aureolatum	*Amblyomma aureolatum*	1	1	1
Amblyomma aureolatum	*Amblyomma aureolatum*	1	1	1
Amblyomma aureolatum	*Amblyomma aureolatum*	1	1	1
Amblyomma aureolatum	*Amblyomma aureolatum*	1	1	1
Amblyomma aureolatum	*Amblyomma aureolatum*	1	1	1
Amblyomma aureolatum	*Amblyomma aureolatum*	1	1	1
Amblyomma aureolatum	*Amblyomma aureolatum*	1	1	1
Amblyomma aureolatum	*Amblyomma aureolatum*	1	1	1
Amblyomma aureolatum	*Amblyomma aureolatum*	1	1	1
Amblyomma aureolatum	*Amblyomma aureolatum*	1	1	1
Amblyomma aureolatum	*Amblyomma aureolatum*	1	1	1
Amblyomma aureolatum	*Amblyomma aureolatum*	1	1	1
Amblyomma aureolatum	*Amblyomma aureolatum*	1	1	1
Amblyomma cajennense	*Amblyomma cajennense*	1	2	2
Amblyomma cajennense	*Amblyomma cajennense*	1	2	2
Amblyomma cajennense	*Amblyomma cajennense*	1	2	2
Amblyomma cajennense	*Amblyomma cajennense*	1	2	2
Amblyomma cajennense	*Amblyomma cajennense*	1	2	2
Amblyomma cajennense	*Amblyomma cajennense*	1	2	2
Amblyomma cajennense	*Amblyomma cajennense*	1	2	2
Amblyomma cajennense	*Amblyomma cajennense*	1	2	2
Amblyomma cajennense	*Amblyomma cajennense*	1	2	2
Amblyomma cajennense	*Amblyomma cajennense*	1	2	2
Amblyomma cajennense	*Amblyomma cajennense*	1	2	2
Amblyomma cajennense	*Amblyomma cajennense*	1	2	2
Amblyomma cajennense	*Amblyomma triste*	0	2	6
Amblyomma cajennense	*Amblyomma cajennense*	1	2	2
Amblyomma cajennense	*Amblyomma cajennense*	1	2	2
Amblyomma cajennense	*Amblyomma cajennense*	1	2	2
Amblyomma cajennense	*Amblyomma cajennense*	1	2	2
Amblyomma dubitatum	*Amblyomma dubitatum*	1	3	3
Amblyomma dubitatum	*Amblyomma dubitatum*	1	3	3
Amblyomma dubitatum	*Amblyomma ovale*	0	3	4
Amblyomma dubitatum	*Amblyomma dubitatum*	1	3	3
Amblyomma dubitatum	*Amblyomma dubitatum*	1	3	3
Amblyomma dubitatum	*Amblyomma ovale*	0	3	4
Amblyomma dubitatum	*Amblyomma dubitatum*	1	3	3
Amblyomma dubitatum	*Amblyomma dubitatum*	1	3	3
Amblyomma dubitatum	*Amblyomma dubitatum*	1	3	3
Amblyomma dubitatum	*Amblyomma dubitatum*	1	3	3
Amblyomma dubitatum	*Amblyomma dubitatum*	1	3	3
Amblyomma dubitatum	*Amblyomma dubitatum*	1	3	3
Amblyomma dubitatum	*Amblyomma sculptum*	0	3	5
Amblyomma ovale	*Amblyomma ovale*	1	4	4
Amblyomma ovale	*Amblyomma ovale*	1	4	4
Amblyomma ovale	*Amblyomma ovale*	1	4	4
Amblyomma ovale	*Amblyomma ovale*	1	4	4
Amblyomma ovale	*Amblyomma ovale*	1	4	4
Amblyomma ovale	*Amblyomma ovale*	1	4	4

Amblyomma ovale	*Amblyomma ovale*	1	4	4
Amblyomma ovale	*Amblyomma ovale*	1	4	4
Amblyomma ovale	*Amblyomma ovale*	1	4	4
Amblyomma ovale	*Amblyomma ovale*	1	4	4
Amblyomma ovale	*Amblyomma cajennense*	0	4	2
Amblyomma ovale	*Amblyomma aureolatum*	0	4	1
Amblyomma ovale	*Amblyomma ovale*	1	4	4
Amblyomma sculptum	*Amblyomma sculptum*	1	5	5
Amblyomma sculptum	*Amblyomma sculptum*	1	5	5
Amblyomma sculptum	*Amblyomma sculptum*	1	5	5
Amblyomma sculptum	*Amblyomma sculptum*	1	5	5
Amblyomma sculptum	*Amblyomma sculptum*	1	5	5
Amblyomma sculptum	*Amblyomma sculptum*	1	5	5
Amblyomma sculptum	*Amblyomma sculptum*	1	5	5
Amblyomma sculptum	*Amblyomma sculptum*	1	5	5
Amblyomma sculptum	*Amblyomma sculptum*	1	5	5
Amblyomma sculptum	*Amblyomma sculptum*	1	5	5
Amblyomma sculptum	*Amblyomma ovale*	0	5	4
Amblyomma sculptum	*Amblyomma sculptum*	1	5	5
Amblyomma sculptum	*Amblyomma sculptum*	1	5	5
Amblyomma sculptum	*Amblyomma sculptum*	1	5	5
Amblyomma sculptum	*Amblyomma sculptum*	1	5	5
Amblyomma sculptum	*Amblyomma sculptum*	1	5	5
Amblyomma sculptum	*Amblyomma sculptum*	1	5	5
Amblyomma sculptum	*Amblyomma sculptum*	1	5	5
Amblyomma sculptum	*Amblyomma dubitatum*	0	5	3
Amblyomma sculptum	*Amblyomma sculptum*	1	5	5
Amblyomma sculptum	*Amblyomma sculptum*	1	5	5
Amblyomma sculptum	*Amblyomma ovale*	0	5	4
Amblyomma sculptum	*Amblyomma sculptum*	1	5	5
Amblyomma sculptum	*Amblyomma sculptum*	1	5	5
Amblyomma sculptum	*Amblyomma sculptum*	1	5	5
Amblyomma sculptum	*Amblyomma sculptum*	1	5	5
Amblyomma sculptum	*Amblyomma sculptum*	1	5	5
Amblyomma triste	*Amblyomma triste*	1	6	6
Amblyomma triste	*Amblyomma triste*	1	6	6
Amblyomma triste	*Amblyomma triste*	1	6	6
Amblyomma triste	*Amblyomma cajennense*	0	6	2
Amblyomma triste	*Amblyomma triste*	1	6	6
Amblyomma triste	*Amblyomma triste*	1	6	6
Amblyomma triste	*Amblyomma triste*	1	6	6
Amblyomma triste	*Amblyomma triste*	1	6	6
Amblyomma triste	*Amblyomma triste*	1	6	6
Amblyomma triste	*Amblyomma triste*	1	6	6
Amblyomma triste	*Amblyomma dubitatum*	0	6	3
Amblyomma triste	*Amblyomma triste*	1	6	6
Amblyomma triste	*Amblyomma aureolatum*	0	6	1
Amblyomma triste	*Amblyomma triste*	1	6	6
Amblyomma triste	*Amblyomma sculptum*	0	6	5
Amblyomma triste	*Amblyomma triste*	1	6	6
Amblyomma aureolatum	*Amblyomma aureolatum*	1	1	1
Amblyomma aureolatum	*Amblyomma aureolatum*	1	1	1
Amblyomma aureolatum	*Amblyomma aureolatum*	1	1	1
Amblyomma aureolatum	*Amblyomma aureolatum*	1	1	1
Amblyomma aureolatum	*Amblyomma aureolatum*	1	1	1
Amblyomma aureolatum	*Amblyomma aureolatum*	1	1	1
Amblyomma aureolatum	*Amblyomma ovale*	0	1	4
Amblyomma aureolatum	*Amblyomma dubitatum*	0	1	3
Amblyomma aureolatum	*Amblyomma aureolatum*	1	1	1
Amblyomma aureolatum	*Amblyomma aureolatum*	1	1	1
Amblyomma aureolatum	*Amblyomma aureolatum*	1	1	1
Amblyomma aureolatum	*Amblyomma aureolatum*	1	1	1
Amblyomma aureolatum	*Amblyomma aureolatum*	1	1	1
Amblyomma aureolatum	*Amblyomma aureolatum*	1	1	1
Amblyomma cajennense	*Amblyomma cajennense*	1	2	2
Amblyomma cajennense	*Amblyomma cajennense*	1	2	2
Amblyomma cajennense	*Amblyomma cajennense*	1	2	2

Amblyomma cajennense	*Amblyomma cajennense*	1	2	2
Amblyomma cajennense	*Amblyomma cajennense*	1	2	2
Amblyomma cajennense	*Amblyomma cajennense*	1	2	2
Amblyomma cajennense	*Amblyomma cajennense*	1	2	2
Amblyomma cajennense	*Amblyomma cajennense*	1	2	2
Amblyomma cajennense	*Amblyomma cajennense*	1	2	2
Amblyomma cajennense	*Amblyomma cajennense*	1	2	2
Amblyomma cajennense	*Amblyomma cajennense*	1	2	2
Amblyomma cajennense	*Amblyomma cajennense*	1	2	2
Amblyomma cajennense	*Amblyomma cajennense*	1	2	2
Amblyomma cajennense	*Amblyomma cajennense*	1	2	2
Amblyomma cajennense	*Amblyomma cajennense*	1	2	2
Amblyomma cajennense	*Amblyomma cajennense*	1	2	2
Amblyomma cajennense	*Amblyomma cajennense*	1	2	2
Amblyomma dubitatum	*Amblyomma dubitatum*	1	3	3
Amblyomma dubitatum	*Amblyomma dubitatum*	1	3	3
Amblyomma dubitatum	*Amblyomma dubitatum*	1	3	3
Amblyomma dubitatum	*Amblyomma dubitatum*	1	3	3
Amblyomma dubitatum	*Amblyomma dubitatum*	1	3	3
Amblyomma dubitatum	*Amblyomma dubitatum*	1	3	3
Amblyomma dubitatum	*Amblyomma dubitatum*	1	3	3
Amblyomma dubitatum	*Amblyomma dubitatum*	1	3	3
Amblyomma dubitatum	*Amblyomma dubitatum*	1	3	3
Amblyomma dubitatum	*Amblyomma dubitatum*	1	3	3
Amblyomma dubitatum	*Amblyomma aureolatum*	0	3	1
Amblyomma dubitatum	*Amblyomma dubitatum*	1	3	3
Amblyomma dubitatum	*Amblyomma dubitatum*	1	3	3
Amblyomma ovale	*Amblyomma ovale*	1	4	4
Amblyomma ovale	*Amblyomma ovale*	1	4	4
Amblyomma ovale	*Amblyomma ovale*	1	4	4
Amblyomma ovale	*Amblyomma dubitatum*	0	4	3
Amblyomma ovale	*Amblyomma ovale*	1	4	4
Amblyomma ovale	*Amblyomma ovale*	1	4	4
Amblyomma ovale	*Amblyomma aureolatum*	0	4	1
Amblyomma ovale	*Amblyomma aureolatum*	0	4	1
Amblyomma ovale	*Amblyomma ovale*	1	4	4
Amblyomma ovale	*Amblyomma ovale*	1	4	4
Amblyomma ovale	*Amblyomma ovale*	1	4	4
Amblyomma ovale	*Amblyomma ovale*	1	4	4
Amblyomma ovale	*Amblyomma ovale*	1	4	4
Amblyomma sculptum	*Amblyomma sculptum*	1	5	5
Amblyomma sculptum	*Amblyomma sculptum*	1	5	5
Amblyomma sculptum	*Amblyomma sculptum*	1	5	5
Amblyomma sculptum	*Amblyomma sculptum*	1	5	5
Amblyomma sculptum	*Amblyomma sculptum*	1	5	5
Amblyomma sculptum	*Amblyomma sculptum*	1	5	5
Amblyomma sculptum	*Amblyomma sculptum*	1	5	5
Amblyomma sculptum	*Amblyomma sculptum*	1	5	5
Amblyomma sculptum	*Amblyomma sculptum*	1	5	5
Amblyomma sculptum	*Amblyomma sculptum*	1	5	5
Amblyomma sculptum	*Amblyomma sculptum*	1	5	5
Amblyomma sculptum	*Amblyomma sculptum*	1	5	5
Amblyomma sculptum	*Amblyomma sculptum*	1	5	5
Amblyomma sculptum	*Amblyomma sculptum*	1	5	5
Amblyomma sculptum	*Amblyomma sculptum*	1	5	5
Amblyomma sculptum	*Amblyomma sculptum*	1	5	5
Amblyomma sculptum	*Amblyomma sculptum*	1	5	5
Amblyomma sculptum	*Amblyomma sculptum*	1	5	5
Amblyomma sculptum	*Amblyomma dubitatum*	0	5	3
Amblyomma sculptum	*Amblyomma sculptum*	1	5	5
Amblyomma sculptum	*Amblyomma sculptum*	1	5	5
Amblyomma sculptum	*Amblyomma dubitatum*	0	5	3
Amblyomma sculptum	*Amblyomma sculptum*	1	5	5
Amblyomma sculptum	*Amblyomma sculptum*	1	5	5
Amblyomma sculptum	*Amblyomma sculptum*	1	5	5
Amblyomma sculptum	*Amblyomma sculptum*	1	5	5
Amblyomma sculptum	*Amblyomma sculptum*	1	5	5

Amblyomma sculptum	*Amblyomma sculptum*	1	5	5
Amblyomma triste	*Amblyomma triste*	1	6	6
Amblyomma triste	*Amblyomma triste*	1	6	6
Amblyomma triste	*Amblyomma cajennense*	0	6	2
Amblyomma triste	*Amblyomma triste*	1	6	6
Amblyomma triste	*Amblyomma triste*	1	6	6
Amblyomma triste	*Amblyomma triste*	1	6	6
Amblyomma triste	*Amblyomma triste*	1	6	6
Amblyomma triste	*Amblyomma triste*	1	6	6
Amblyomma triste	*Amblyomma triste*	1	6	6
Amblyomma triste	*Amblyomma cajennense*	0	6	2
Amblyomma triste	*Amblyomma triste*	1	6	6
Amblyomma triste	*Amblyomma triste*	1	6	6
Amblyomma triste	*Amblyomma triste*	1	6	6
Amblyomma triste	*Amblyomma triste*	1	6	6
Amblyomma triste	*Amblyomma triste*	1	6	6
Amblyomma triste	*Amblyomma triste*	1	6	6
Amblyomma aureolatum	*Amblyomma aureolatum*	1	1	1
Amblyomma aureolatum	*Amblyomma aureolatum*	1	1	1
Amblyomma aureolatum	*Amblyomma aureolatum*	1	1	1
Amblyomma aureolatum	*Amblyomma aureolatum*	1	1	1
Amblyomma aureolatum	*Amblyomma aureolatum*	1	1	1
Amblyomma aureolatum	*Amblyomma aureolatum*	1	1	1
Amblyomma aureolatum	*Amblyomma aureolatum*	1	1	1
Amblyomma aureolatum	*Amblyomma aureolatum*	1	1	1
Amblyomma aureolatum	*Amblyomma aureolatum*	1	1	1
Amblyomma aureolatum	*Amblyomma aureolatum*	1	1	1
Amblyomma aureolatum	*Amblyomma aureolatum*	1	1	1
Amblyomma aureolatum	*Amblyomma aureolatum*	1	1	1
Amblyomma aureolatum	*Amblyomma aureolatum*	1	1	1
Amblyomma cajennense	*Amblyomma cajennense*	1	2	2
Amblyomma cajennense	*Amblyomma triste*	0	2	6
Amblyomma cajennense	*Amblyomma triste*	0	2	6
Amblyomma cajennense	*Amblyomma cajennense*	1	2	2
Amblyomma cajennense	*Amblyomma cajennense*	1	2	2
Amblyomma cajennense	*Amblyomma cajennense*	1	2	2
Amblyomma cajennense	*Amblyomma cajennense*	1	2	2
Amblyomma cajennense	*Amblyomma cajennense*	1	2	2
Amblyomma cajennense	*Amblyomma cajennense*	1	2	2
Amblyomma cajennense	*Amblyomma cajennense*	1	2	2
Amblyomma cajennense	*Amblyomma cajennense*	1	2	2
Amblyomma cajennense	*Amblyomma cajennense*	1	2	2
Amblyomma cajennense	*Amblyomma cajennense*	1	2	2
Amblyomma cajennense	*Amblyomma cajennense*	1	2	2
Amblyomma cajennense	*Amblyomma cajennense*	1	2	2
Amblyomma cajennense	*Amblyomma cajennense*	1	2	2
Amblyomma cajennense	*Amblyomma cajennense*	1	2	2
Amblyomma dubitatum	*Amblyomma sculptum*	0	3	5
Amblyomma dubitatum	*Amblyomma dubitatum*	1	3	3
Amblyomma dubitatum	*Amblyomma triste*	0	3	6
Amblyomma dubitatum	*Amblyomma dubitatum*	1	3	3
Amblyomma dubitatum	*Amblyomma dubitatum*	1	3	3
Amblyomma dubitatum	*Amblyomma triste*	0	3	6
Amblyomma dubitatum	*Amblyomma dubitatum*	1	3	3
Amblyomma dubitatum	*Amblyomma dubitatum*	1	3	3
Amblyomma dubitatum	*Amblyomma dubitatum*	1	3	3
Amblyomma dubitatum	*Amblyomma dubitatum*	1	3	3
Amblyomma dubitatum	*Amblyomma dubitatum*	1	3	3
Amblyomma dubitatum	*Amblyomma dubitatum*	1	3	3
Amblyomma dubitatum	*Amblyomma triste*	0	3	6
Amblyomma ovale	*Amblyomma ovale*	1	4	4
Amblyomma ovale	*Amblyomma ovale*	1	4	4
Amblyomma ovale	*Amblyomma ovale*	1	4	4
Amblyomma ovale	*Amblyomma ovale*	1	4	4
Amblyomma ovale	*Amblyomma ovale*	1	4	4
Amblyomma ovale	*Amblyomma ovale*	1	4	4
Amblyomma ovale	*Amblyomma ovale*	1	4	4

Amblyomma ovale	*Amblyomma ovale*	1	4	4
Amblyomma ovale	*Amblyomma ovale*	1	4	4
Amblyomma ovale	*Amblyomma ovale*	1	4	4
Amblyomma ovale	*Amblyomma aureolatum*	0	4	1
Amblyomma ovale	*Amblyomma cajennense*	0	4	2
Amblyomma ovale	*Amblyomma ovale*	1	4	4
Amblyomma ovale	*Amblyomma ovale*	1	4	4
Amblyomma sculptum	*Amblyomma sculptum*	1	5	5
Amblyomma sculptum	*Amblyomma sculptum*	1	5	5
Amblyomma sculptum	*Amblyomma sculptum*	1	5	5
Amblyomma sculptum	*Amblyomma sculptum*	1	5	5
Amblyomma sculptum	*Amblyomma sculptum*	1	5	5
Amblyomma sculptum	*Amblyomma sculptum*	1	5	5
Amblyomma sculptum	*Amblyomma sculptum*	1	5	5
Amblyomma sculptum	*Amblyomma sculptum*	1	5	5
Amblyomma sculptum	*Amblyomma sculptum*	1	5	5
Amblyomma sculptum	*Amblyomma sculptum*	1	5	5
Amblyomma sculptum	*Amblyomma sculptum*	1	5	5
Amblyomma sculptum	*Amblyomma sculptum*	1	5	5
Amblyomma sculptum	*Amblyomma sculptum*	1	5	5
Amblyomma sculptum	*Amblyomma sculptum*	1	5	5
Amblyomma sculptum	*Amblyomma sculptum*	1	5	5
Amblyomma sculptum	*Amblyomma sculptum*	1	5	5
Amblyomma sculptum	*Amblyomma sculptum*	1	5	5
Amblyomma sculptum	*Amblyomma sculptum*	1	5	5
Amblyomma sculptum	*Amblyomma aureolatum*	0	5	1
Amblyomma sculptum	*Amblyomma aureolatum*	0	5	1
Amblyomma sculptum	*Amblyomma sculptum*	1	5	5
Amblyomma sculptum	*Amblyomma sculptum*	1	5	5
Amblyomma sculptum	*Amblyomma sculptum*	1	5	5
Amblyomma sculptum	*Amblyomma sculptum*	1	5	5
Amblyomma sculptum	*Amblyomma sculptum*	1	5	5
Amblyomma sculptum	*Amblyomma sculptum*	1	5	5
Amblyomma sculptum	*Amblyomma sculptum*	1	5	5
Amblyomma sculptum	*Amblyomma sculptum*	1	5	5
Amblyomma triste	*Amblyomma triste*	1	6	6
Amblyomma triste	*Amblyomma triste*	1	6	6
Amblyomma triste	*Amblyomma triste*	1	6	6
Amblyomma triste	*Amblyomma triste*	1	6	6
Amblyomma triste	*Amblyomma triste*	1	6	6
Amblyomma triste	*Amblyomma triste*	1	6	6
Amblyomma triste	*Amblyomma triste*	1	6	6
Amblyomma triste	*Amblyomma triste*	1	6	6
Amblyomma triste	*Amblyomma triste*	1	6	6
Amblyomma triste	*Amblyomma triste*	1	6	6
Amblyomma triste	*Amblyomma triste*	1	6	6
Amblyomma triste	*Amblyomma triste*	1	6	6
Amblyomma triste	*Amblyomma triste*	1	6	6
Amblyomma triste	*Amblyomma triste*	1	6	6
Amblyomma triste	*Amblyomma triste*	1	6	6
Amblyomma aureolatum	*Amblyomma aureolatum*	1	1	1
Amblyomma aureolatum	*Amblyomma aureolatum*	1	1	1
Amblyomma aureolatum	*Amblyomma aureolatum*	1	1	1
Amblyomma aureolatum	*Amblyomma aureolatum*	1	1	1
Amblyomma aureolatum	*Amblyomma aureolatum*	1	1	1
Amblyomma aureolatum	*Amblyomma aureolatum*	1	1	1
Amblyomma aureolatum	*Amblyomma aureolatum*	1	1	1
Amblyomma aureolatum	*Amblyomma aureolatum*	1	1	1
Amblyomma aureolatum	*Amblyomma aureolatum*	1	1	1
Amblyomma aureolatum	*Amblyomma aureolatum*	1	1	1
Amblyomma aureolatum	*Amblyomma aureolatum*	1	1	1
Amblyomma aureolatum	*Amblyomma aureolatum*	1	1	1
Amblyomma aureolatum	*Amblyomma aureolatum*	1	1	1
Amblyomma aureolatum	*Amblyomma aureolatum*	1	1	1
Amblyomma cajennense	*Amblyomma cajennense*	1	2	2
Amblyomma cajennense	*Amblyomma cajennense*	1	2	2
Amblyomma cajennense	*Amblyomma cajennense*	1	2	2

Amblyomma cajennense	*Amblyomma cajennense*	1	2	2
Amblyomma cajennense	*Amblyomma cajennense*	1	2	2
Amblyomma cajennense	*Amblyomma cajennense*	1	2	2
Amblyomma cajennense	*Amblyomma cajennense*	1	2	2
Amblyomma cajennense	*Amblyomma cajennense*	1	2	2
Amblyomma cajennense	*Amblyomma cajennense*	1	2	2
Amblyomma cajennense	*Amblyomma cajennense*	1	2	2
Amblyomma cajennense	*Amblyomma cajennense*	1	2	2
Amblyomma cajennense	*Amblyomma cajennense*	1	2	2
Amblyomma cajennense	*Amblyomma cajennense*	1	2	2
Amblyomma cajennense	*Amblyomma cajennense*	1	2	2
Amblyomma cajennense	*Amblyomma cajennense*	1	2	2
Amblyomma cajennense	*Amblyomma cajennense*	1	2	2
Amblyomma dubitatum	*Amblyomma dubitatum*	1	3	3
Amblyomma dubitatum	*Amblyomma dubitatum*	1	3	3
Amblyomma dubitatum	*Amblyomma dubitatum*	1	3	3
Amblyomma dubitatum	*Amblyomma dubitatum*	1	3	3
Amblyomma dubitatum	*Amblyomma aureolatum*	0	3	1
Amblyomma dubitatum	*Amblyomma aureolatum*	0	3	1
Amblyomma dubitatum	*Amblyomma dubitatum*	1	3	3
Amblyomma dubitatum	*Amblyomma dubitatum*	1	3	3
Amblyomma dubitatum	*Amblyomma dubitatum*	1	3	3
Amblyomma dubitatum	*Amblyomma dubitatum*	1	3	3
Amblyomma dubitatum	*Amblyomma aureolatum*	0	3	1
Amblyomma dubitatum	*Amblyomma dubitatum*	1	3	3
Amblyomma dubitatum	*Amblyomma dubitatum*	1	3	3
Amblyomma ovale	*Amblyomma ovale*	1	4	4
Amblyomma ovale	*Amblyomma ovale*	1	4	4
Amblyomma ovale	*Amblyomma ovale*	1	4	4
Amblyomma ovale	*Amblyomma ovale*	1	4	4
Amblyomma ovale	*Amblyomma ovale*	1	4	4
Amblyomma ovale	*Amblyomma ovale*	1	4	4
Amblyomma ovale	*Amblyomma ovale*	1	4	4
Amblyomma ovale	*Amblyomma ovale*	1	4	4
Amblyomma ovale	*Amblyomma ovale*	1	4	4
Amblyomma ovale	*Amblyomma ovale*	1	4	4
Amblyomma ovale	*Amblyomma ovale*	1	4	4
Amblyomma ovale	*Amblyomma ovale*	1	4	4
Amblyomma ovale	*Amblyomma ovale*	1	4	4
Amblyomma sculptum	*Amblyomma sculptum*	1	5	5
Amblyomma sculptum	*Amblyomma sculptum*	1	5	5
Amblyomma sculptum	*Amblyomma sculptum*	1	5	5
Amblyomma sculptum	*Amblyomma sculptum*	1	5	5
Amblyomma sculptum	*Amblyomma sculptum*	1	5	5
Amblyomma sculptum	*Amblyomma sculptum*	1	5	5
Amblyomma sculptum	*Amblyomma sculptum*	1	5	5
Amblyomma sculptum	*Amblyomma sculptum*	1	5	5
Amblyomma sculptum	*Amblyomma sculptum*	1	5	5
Amblyomma sculptum	*Amblyomma sculptum*	1	5	5
Amblyomma sculptum	*Amblyomma sculptum*	1	5	5
Amblyomma sculptum	*Amblyomma sculptum*	1	5	5
Amblyomma sculptum	*Amblyomma sculptum*	1	5	5
Amblyomma sculptum	*Amblyomma sculptum*	1	5	5
Amblyomma sculptum	*Amblyomma sculptum*	1	5	5
Amblyomma sculptum	*Amblyomma sculptum*	1	5	5
Amblyomma sculptum	*Amblyomma sculptum*	1	5	5
Amblyomma sculptum	*Amblyomma sculptum*	1	5	5
Amblyomma sculptum	*Amblyomma sculptum*	1	5	5
Amblyomma sculptum	*Amblyomma sculptum*	1	5	5
Amblyomma sculptum	*Amblyomma dubitatum*	0	5	3
Amblyomma sculptum	*Amblyomma sculptum*	1	5	5
Amblyomma sculptum	*Amblyomma sculptum*	1	5	5
Amblyomma sculptum	*Amblyomma sculptum*	1	5	5
Amblyomma sculptum	*Amblyomma sculptum*	1	5	5
Amblyomma sculptum	*Amblyomma sculptum*	1	5	5
Amblyomma sculptum	*Amblyomma sculptum*	1	5	5
Amblyomma triste	*Amblyomma triste*	1	6	6

Amblyomma triste	*Amblyomma triste*	1	6	6
Amblyomma triste	*Amblyomma triste*	1	6	6
Amblyomma triste	*Amblyomma triste*	1	6	6
Amblyomma triste	*Amblyomma triste*	1	6	6
Amblyomma triste	*Amblyomma triste*	1	6	6
Amblyomma triste	*Amblyomma triste*	1	6	6
Amblyomma triste	*Amblyomma aureolatum*	0	6	1
Amblyomma triste	*Amblyomma aureolatum*	0	6	1
Amblyomma triste	*Amblyomma triste*	1	6	6
Amblyomma triste	*Amblyomma triste*	1	6	6
Amblyomma triste	*Amblyomma triste*	1	6	6
Amblyomma triste	*Amblyomma triste*	1	6	6
Amblyomma triste	*Amblyomma triste*	1	6	6
Amblyomma triste	*Amblyomma triste*	1	6	6
Amblyomma triste	*Amblyomma triste*	1	6	6
Amblyomma aureolatum	*Amblyomma aureolatum*	1	1	1
Amblyomma aureolatum	*Amblyomma aureolatum*	1	1	1
Amblyomma aureolatum	*Amblyomma aureolatum*	1	1	1
Amblyomma aureolatum	*Amblyomma aureolatum*	1	1	1
Amblyomma aureolatum	*Amblyomma aureolatum*	1	1	1
Amblyomma aureolatum	*Amblyomma dubitatum*	0	1	3
Amblyomma aureolatum	*Amblyomma aureolatum*	1	1	1
Amblyomma aureolatum	*Amblyomma aureolatum*	1	1	1
Amblyomma aureolatum	*Amblyomma aureolatum*	1	1	1
Amblyomma aureolatum	*Amblyomma aureolatum*	1	1	1
Amblyomma aureolatum	*Amblyomma aureolatum*	1	1	1
Amblyomma aureolatum	*Amblyomma aureolatum*	1	1	1
Amblyomma aureolatum	*Amblyomma aureolatum*	1	1	1
Amblyomma aureolatum	*Amblyomma aureolatum*	1	1	1
Amblyomma cajennense	*Amblyomma cajennense*	1	2	2
Amblyomma cajennense	*Amblyomma cajennense*	1	2	2
Amblyomma cajennense	*Amblyomma cajennense*	1	2	2
Amblyomma cajennense	*Amblyomma cajennense*	1	2	2
Amblyomma cajennense	*Amblyomma cajennense*	1	2	2
Amblyomma cajennense	*Amblyomma cajennense*	1	2	2
Amblyomma cajennense	*Amblyomma cajennense*	1	2	2
Amblyomma cajennense	*Amblyomma cajennense*	1	2	2
Amblyomma cajennense	*Amblyomma cajennense*	1	2	2
Amblyomma cajennense	*Amblyomma cajennense*	1	2	2
Amblyomma cajennense	*Amblyomma cajennense*	1	2	2
Amblyomma cajennense	*Amblyomma cajennense*	1	2	2
Amblyomma cajennense	*Amblyomma cajennense*	1	2	2
Amblyomma cajennense	*Amblyomma cajennense*	1	2	2
Amblyomma cajennense	*Amblyomma cajennense*	1	2	2
Amblyomma cajennense	*Amblyomma cajennense*	1	2	2
Amblyomma dubitatum	*Amblyomma dubitatum*	1	3	3
Amblyomma dubitatum	*Amblyomma dubitatum*	1	3	3
Amblyomma dubitatum	*Amblyomma dubitatum*	1	3	3
Amblyomma dubitatum	*Amblyomma dubitatum*	1	3	3
Amblyomma dubitatum	*Amblyomma dubitatum*	1	3	3
Amblyomma dubitatum	*Amblyomma dubitatum*	1	3	3
Amblyomma dubitatum	*Amblyomma dubitatum*	1	3	3
Amblyomma dubitatum	*Amblyomma dubitatum*	1	3	3
Amblyomma dubitatum	*Amblyomma dubitatum*	1	3	3
Amblyomma dubitatum	*Amblyomma dubitatum*	1	3	3
Amblyomma dubitatum	*Amblyomma dubitatum*	1	3	3
Amblyomma dubitatum	*Amblyomma sculptum*	0	3	5
Amblyomma dubitatum	*Amblyomma sculptum*	0	3	5
Amblyomma ovale	*Amblyomma ovale*	1	4	4
Amblyomma ovale	*Amblyomma ovale*	1	4	4
Amblyomma ovale	*Amblyomma ovale*	1	4	4
Amblyomma ovale	*Amblyomma ovale*	1	4	4
Amblyomma ovale	*Amblyomma ovale*	1	4	4
Amblyomma ovale	*Amblyomma cajennense*	0	4	2
Amblyomma ovale	*Amblyomma cajennense*	0	4	2
Amblyomma ovale	*Amblyomma ovale*	1	4	4
Amblyomma ovale	*Amblyomma ovale*	1	4	4

Amblyomma ovale	*Amblyomma ovale*	1	4	4
Amblyomma ovale	*Amblyomma aureolatum*	0	4	1
Amblyomma ovale	*Amblyomma aureolatum*	0	4	1
Amblyomma ovale	*Amblyomma dubitatum*	0	4	3
Amblyomma sculptum	*Amblyomma sculptum*	1	5	5
Amblyomma sculptum	*Amblyomma sculptum*	1	5	5
Amblyomma sculptum	*Amblyomma sculptum*	1	5	5
Amblyomma sculptum	*Amblyomma sculptum*	1	5	5
Amblyomma sculptum	*Amblyomma sculptum*	1	5	5
Amblyomma sculptum	*Amblyomma sculptum*	1	5	5
Amblyomma sculptum	*Amblyomma sculptum*	1	5	5
Amblyomma sculptum	*Amblyomma sculptum*	1	5	5
Amblyomma sculptum	*Amblyomma sculptum*	1	5	5
Amblyomma sculptum	*Amblyomma sculptum*	1	5	5
Amblyomma sculptum	*Amblyomma sculptum*	1	5	5
Amblyomma sculptum	*Amblyomma sculptum*	1	5	5
Amblyomma sculptum	*Amblyomma sculptum*	1	5	5
Amblyomma sculptum	*Amblyomma sculptum*	1	5	5
Amblyomma sculptum	*Amblyomma sculptum*	1	5	5
Amblyomma sculptum	*Amblyomma sculptum*	1	5	5
Amblyomma sculptum	*Amblyomma sculptum*	1	5	5
Amblyomma sculptum	*Amblyomma sculptum*	1	5	5
Amblyomma sculptum	*Amblyomma sculptum*	1	5	5
Amblyomma sculptum	*Amblyomma sculptum*	1	5	5
Amblyomma sculptum	*Amblyomma sculptum*	1	5	5
Amblyomma sculptum	*Amblyomma sculptum*	1	5	5
Amblyomma sculptum	*Amblyomma sculptum*	1	5	5
Amblyomma sculptum	*Amblyomma sculptum*	1	5	5
Amblyomma sculptum	*Amblyomma sculptum*	1	5	5
Amblyomma sculptum	*Amblyomma sculptum*	1	5	5
Amblyomma sculptum	*Amblyomma sculptum*	1	5	5
Amblyomma sculptum	*Amblyomma sculptum*	1	5	5
Amblyomma sculptum	*Amblyomma sculptum*	1	5	5
Amblyomma triste	*Amblyomma triste*	1	6	6
Amblyomma triste	*Amblyomma triste*	1	6	6
Amblyomma triste	*Amblyomma triste*	1	6	6
Amblyomma triste	*Amblyomma triste*	1	6	6
Amblyomma triste	*Amblyomma triste*	1	6	6
Amblyomma triste	*Amblyomma triste*	1	6	6
Amblyomma triste	*Amblyomma triste*	1	6	6
Amblyomma triste	*Amblyomma triste*	1	6	6
Amblyomma triste	*Amblyomma triste*	1	6	6
Amblyomma triste	*Amblyomma triste*	1	6	6
Amblyomma triste	*Amblyomma triste*	1	6	6
Amblyomma triste	*Amblyomma triste*	1	6	6
Amblyomma triste	*Amblyomma triste*	1	6	6
Amblyomma triste	*Amblyomma triste*	1	6	6
Amblyomma triste	*Amblyomma triste*	1	6	6
Amblyomma triste	*Amblyomma triste*	1	6	6
Amblyomma aureolatum	*Amblyomma aureolatum*	1	1	1
Amblyomma aureolatum	*Amblyomma aureolatum*	1	1	1
Amblyomma aureolatum	*Amblyomma aureolatum*	1	1	1
Amblyomma aureolatum	*Amblyomma aureolatum*	1	1	1
Amblyomma aureolatum	*Amblyomma aureolatum*	1	1	1
Amblyomma aureolatum	*Amblyomma aureolatum*	1	1	1
Amblyomma aureolatum	*Amblyomma aureolatum*	1	1	1
Amblyomma aureolatum	*Amblyomma aureolatum*	1	1	1
Amblyomma aureolatum	*Amblyomma aureolatum*	1	1	1
Amblyomma aureolatum	*Amblyomma aureolatum*	1	1	1
Amblyomma aureolatum	*Amblyomma aureolatum*	1	1	1
Amblyomma aureolatum	*Amblyomma aureolatum*	1	1	1
Amblyomma aureolatum	*Amblyomma aureolatum*	1	1	1
Amblyomma aureolatum	*Amblyomma aureolatum*	1	1	1
Amblyomma aureolatum	*Amblyomma aureolatum*	1	1	1
Amblyomma aureolatum	*Amblyomma aureolatum*	1	1	1
Amblyomma aureolatum	*Amblyomma aureolatum*	1	1	1
Amblyomma aureolatum	*Amblyomma aureolatum*	1	1	1
Amblyomma aureolatum	*Amblyomma aureolatum*	1	1	1

Amblyomma aureolatum	*Amblyomma aureolatum*	1	1	1
Amblyomma aureolatum	*Amblyomma aureolatum*	1	1	1
Amblyomma aureolatum	*Amblyomma aureolatum*	1	1	1
Amblyomma aureolatum	*Amblyomma aureolatum*	1	1	1
Amblyomma aureolatum	*Amblyomma aureolatum*	1	1	1
Amblyomma cajennense	*Amblyomma cajennense*	1	2	2
Amblyomma cajennense	*Amblyomma cajennense*	1	2	2
Amblyomma cajennense	*Amblyomma cajennense*	1	2	2
Amblyomma cajennense	*Amblyomma cajennense*	1	2	2
Amblyomma cajennense	*Amblyomma cajennense*	1	2	2
Amblyomma cajennense	*Amblyomma cajennense*	1	2	2
Amblyomma cajennense	*Amblyomma cajennense*	1	2	2
Amblyomma cajennense	*Amblyomma cajennense*	1	2	2
Amblyomma cajennense	*Amblyomma cajennense*	1	2	2
Amblyomma cajennense	*Amblyomma cajennense*	1	2	2
Amblyomma cajennense	*Amblyomma cajennense*	1	2	2
Amblyomma cajennense	*Amblyomma sculptum*	0	2	5
Amblyomma cajennense	*Amblyomma cajennense*	1	2	2
Amblyomma cajennense	*Amblyomma cajennense*	1	2	2
Amblyomma cajennense	*Amblyomma cajennense*	1	2	2
Amblyomma cajennense	*Amblyomma cajennense*	1	2	2
Amblyomma cajennense	*Amblyomma cajennense*	1	2	2
Amblyomma cajennense	*Amblyomma aureolatum*	0	2	1
Amblyomma cajennense	*Amblyomma cajennense*	1	2	2
Amblyomma cajennense	*Amblyomma cajennense*	1	2	2
Amblyomma cajennense	*Amblyomma cajennense*	1	2	2
Amblyomma cajennense	*Amblyomma cajennense*	1	2	2
Amblyomma cajennense	*Amblyomma cajennense*	1	2	2
Amblyomma cajennense	*Amblyomma cajennense*	1	2	2
Amblyomma cajennense	*Amblyomma cajennense*	1	2	2
Amblyomma cajennense	*Amblyomma cajennense*	1	2	2
Amblyomma cajennense	*Amblyomma cajennense*	1	2	2
Amblyomma cajennense	*Amblyomma cajennense*	1	2	2
Amblyomma cajennense	*Amblyomma cajennense*	1	2	2
Amblyomma cajennense	*Amblyomma dubitatum*	0	2	3
Amblyomma cajennense	*Amblyomma cajennense*	1	2	2
Amblyomma cajennense	*Amblyomma cajennense*	1	2	2
Amblyomma cajennense	*Amblyomma cajennense*	1	2	2
Amblyomma dubitatum	*Amblyomma dubitatum*	1	3	3
Amblyomma dubitatum	*Amblyomma dubitatum*	1	3	3
Amblyomma dubitatum	*Amblyomma dubitatum*	1	3	3
Amblyomma dubitatum	*Amblyomma sculptum*	0	3	5
Amblyomma dubitatum	*Amblyomma dubitatum*	1	3	3
Amblyomma dubitatum	*Amblyomma ovale*	0	3	4
Amblyomma dubitatum	*Amblyomma dubitatum*	1	3	3
Amblyomma dubitatum	*Amblyomma sculptum*	0	3	5
Amblyomma dubitatum	*Amblyomma dubitatum*	1	3	3
Amblyomma dubitatum	*Amblyomma dubitatum*	1	3	3
Amblyomma dubitatum	*Amblyomma dubitatum*	1	3	3
Amblyomma dubitatum	*Amblyomma dubitatum*	1	3	3
Amblyomma dubitatum	*Amblyomma dubitatum*	1	3	3
Amblyomma dubitatum	*Amblyomma dubitatum*	1	3	3
Amblyomma dubitatum	*Amblyomma dubitatum*	1	3	3
Amblyomma dubitatum	*Amblyomma dubitatum*	1	3	3
Amblyomma dubitatum	*Amblyomma dubitatum*	1	3	3
Amblyomma dubitatum	*Amblyomma dubitatum*	1	3	3
Amblyomma dubitatum	*Amblyomma dubitatum*	1	3	3
Amblyomma dubitatum	*Amblyomma dubitatum*	1	3	3
Amblyomma dubitatum	*Amblyomma ovale*	0	3	4
Amblyomma dubitatum	*Amblyomma dubitatum*	1	3	3
Amblyomma ovale	*Amblyomma ovale*	1	4	4
Amblyomma ovale	*Amblyomma triste*	0	4	6
Amblyomma ovale	*Amblyomma dubitatum*	0	4	3
Amblyomma ovale	*Amblyomma ovale*	1	4	4
Amblyomma ovale	*Amblyomma ovale*	1	4	4
Amblyomma ovale	*Amblyomma ovale*	1	4	4
Amblyomma ovale	*Amblyomma aureolatum*	0	4	1

Amblyomma ovale	*Amblyomma ovale*	1	4	4
Amblyomma ovale	*Amblyomma ovale*	1	4	4
Amblyomma ovale	*Amblyomma ovale*	1	4	4
Amblyomma ovale	*Amblyomma ovale*	1	4	4
Amblyomma ovale	*Amblyomma ovale*	1	4	4
Amblyomma ovale	*Amblyomma ovale*	1	4	4
Amblyomma ovale	*Amblyomma ovale*	1	4	4
Amblyomma ovale	*Amblyomma ovale*	1	4	4
Amblyomma ovale	*Amblyomma ovale*	1	4	4
Amblyomma ovale	*Amblyomma ovale*	1	4	4
Amblyomma ovale	*Amblyomma ovale*	1	4	4
Amblyomma ovale	*Amblyomma ovale*	1	4	4
Amblyomma ovale	*Amblyomma ovale*	1	4	4
Amblyomma ovale	*Amblyomma ovale*	1	4	4
Amblyomma ovale	*Amblyomma ovale*	1	4	4
Amblyomma ovale	*Amblyomma ovale*	1	4	4
Amblyomma ovale	*Amblyomma ovale*	1	4	4
Amblyomma sculptum	*Amblyomma sculptum*	1	5	5
Amblyomma sculptum	*Amblyomma sculptum*	1	5	5
Amblyomma sculptum	*Amblyomma sculptum*	1	5	5
Amblyomma sculptum	*Amblyomma sculptum*	1	5	5
Amblyomma sculptum	*Amblyomma sculptum*	1	5	5
Amblyomma sculptum	*Amblyomma sculptum*	1	5	5
Amblyomma sculptum	*Amblyomma sculptum*	1	5	5
Amblyomma sculptum	*Amblyomma sculptum*	1	5	5
Amblyomma sculptum	*Amblyomma sculptum*	1	5	5
Amblyomma sculptum	*Amblyomma sculptum*	1	5	5
Amblyomma sculptum	*Amblyomma sculptum*	1	5	5
Amblyomma sculptum	*Amblyomma sculptum*	1	5	5
Amblyomma sculptum	*Amblyomma sculptum*	1	5	5
Amblyomma sculptum	*Amblyomma dubitatum*	0	5	3
Amblyomma sculptum	*Amblyomma sculptum*	1	5	5
Amblyomma sculptum	*Amblyomma sculptum*	1	5	5
Amblyomma sculptum	*Amblyomma sculptum*	1	5	5
Amblyomma sculptum	*Amblyomma dubitatum*	0	5	3
Amblyomma sculptum	*Amblyomma sculptum*	1	5	5
Amblyomma sculptum	*Amblyomma sculptum*	1	5	5
Amblyomma sculptum	*Amblyomma sculptum*	1	5	5
Amblyomma sculptum	*Amblyomma dubitatum*	0	5	3
Amblyomma sculptum	*Amblyomma sculptum*	1	5	5
Amblyomma sculptum	*Amblyomma sculptum*	1	5	5
Amblyomma sculptum	*Amblyomma sculptum*	1	5	5
Amblyomma sculptum	*Amblyomma sculptum*	1	5	5
Amblyomma sculptum	*Amblyomma sculptum*	1	5	5
Amblyomma sculptum	*Amblyomma sculptum*	1	5	5
Amblyomma sculptum	*Amblyomma dubitatum*	0	5	3
Amblyomma sculptum	*Amblyomma sculptum*	1	5	5
Amblyomma sculptum	*Amblyomma sculptum*	1	5	5
Amblyomma triste	*Amblyomma triste*	1	6	6
Amblyomma triste	*Amblyomma triste*	1	6	6
Amblyomma triste	*Amblyomma triste*	1	6	6
Amblyomma triste	*Amblyomma triste*	1	6	6
Amblyomma triste	*Amblyomma triste*	1	6	6
Amblyomma triste	*Amblyomma triste*	1	6	6
Amblyomma triste	*Amblyomma triste*	1	6	6
Amblyomma triste	*Amblyomma triste*	1	6	6
Amblyomma triste	*Amblyomma triste*	1	6	6
Amblyomma triste	*Amblyomma triste*	1	6	6
Amblyomma triste	*Amblyomma triste*	1	6	6
Amblyomma triste	*Amblyomma triste*	1	6	6
Amblyomma triste	*Amblyomma triste*	1	6	6
Amblyomma triste	*Amblyomma triste*	1	6	6
Amblyomma triste	*Amblyomma triste*	1	6	6
Amblyomma triste	*Amblyomma triste*	1	6	6
Amblyomma triste	*Amblyomma triste*	1	6	6
Amblyomma triste	*Amblyomma triste*	1	6	6
Amblyomma triste	*Amblyomma triste*	1	6	6

Amblyomma triste	*Amblyomma triste*	1	6	6
Amblyomma triste	*Amblyomma triste*	1	6	6
Amblyomma triste	*Amblyomma triste*	1	6	6
Amblyomma triste	*Amblyomma triste*	1	6	6
Amblyomma triste	*Amblyomma triste*	1	6	6
Amblyomma triste	*Amblyomma triste*	1	6	6
Amblyomma triste	*Amblyomma triste*	1	6	6
Amblyomma triste	*Amblyomma triste*	1	6	6
Amblyomma triste	*Amblyomma triste*	1	6	6
Amblyomma triste	*Amblyomma triste*	1	6	6
Amblyomma triste	*Amblyomma triste*	1	6	6
Amblyomma triste	*Amblyomma triste*	1	6	6
Amblyomma aureolatum	*Amblyomma aureolatum*	1	1	1
Amblyomma aureolatum	*Amblyomma aureolatum*	1	1	1
Amblyomma aureolatum	*Amblyomma aureolatum*	1	1	1
Amblyomma aureolatum	*Amblyomma aureolatum*	1	1	1
Amblyomma aureolatum	*Amblyomma aureolatum*	1	1	1
Amblyomma aureolatum	*Amblyomma aureolatum*	1	1	1
Amblyomma aureolatum	*Amblyomma aureolatum*	1	1	1
Amblyomma aureolatum	*Amblyomma aureolatum*	1	1	1
Amblyomma aureolatum	*Amblyomma aureolatum*	1	1	1
Amblyomma aureolatum	*Amblyomma aureolatum*	1	1	1
Amblyomma aureolatum	*Amblyomma aureolatum*	1	1	1
Amblyomma aureolatum	*Amblyomma aureolatum*	1	1	1
Amblyomma aureolatum	*Amblyomma aureolatum*	1	1	1
Amblyomma aureolatum	*Amblyomma aureolatum*	1	1	1
Amblyomma aureolatum	*Amblyomma aureolatum*	1	1	1
Amblyomma aureolatum	*Amblyomma aureolatum*	1	1	1
Amblyomma aureolatum	*Amblyomma aureolatum*	1	1	1
Amblyomma aureolatum	*Amblyomma aureolatum*	1	1	1
Amblyomma aureolatum	*Amblyomma aureolatum*	1	1	1
Amblyomma aureolatum	*Amblyomma aureolatum*	1	1	1
Amblyomma aureolatum	*Amblyomma aureolatum*	1	1	1
Amblyomma aureolatum	*Amblyomma aureolatum*	1	1	1
Amblyomma aureolatum	*Amblyomma ovale*	0	1	4
Amblyomma aureolatum	*Amblyomma aureolatum*	1	1	1
Amblyomma cajennense	*Amblyomma cajennense*	1	2	2
Amblyomma cajennense	*Amblyomma cajennense*	1	2	2
Amblyomma cajennense	*Amblyomma cajennense*	1	2	2
Amblyomma cajennense	*Amblyomma cajennense*	1	2	2
Amblyomma cajennense	*Amblyomma cajennense*	1	2	2
Amblyomma cajennense	*Amblyomma cajennense*	1	2	2
Amblyomma cajennense	*Amblyomma cajennense*	1	2	2
Amblyomma cajennense	*Amblyomma cajennense*	1	2	2
Amblyomma cajennense	*Amblyomma cajennense*	1	2	2
Amblyomma cajennense	*Amblyomma cajennense*	1	2	2
Amblyomma cajennense	*Amblyomma cajennense*	1	2	2
Amblyomma cajennense	*Amblyomma cajennense*	1	2	2
Amblyomma cajennense	*Amblyomma cajennense*	1	2	2
Amblyomma cajennense	*Amblyomma cajennense*	1	2	2
Amblyomma cajennense	*Amblyomma cajennense*	1	2	2
Amblyomma cajennense	*Amblyomma cajennense*	1	2	2
Amblyomma cajennense	*Amblyomma cajennense*	1	2	2
Amblyomma cajennense	*Amblyomma cajennense*	1	2	2
Amblyomma cajennense	*Amblyomma cajennense*	1	2	2
Amblyomma cajennense	*Amblyomma cajennense*	1	2	2
Amblyomma cajennense	*Amblyomma cajennense*	1	2	2
Amblyomma cajennense	*Amblyomma cajennense*	1	2	2
Amblyomma cajennense	*Amblyomma cajennense*	1	2	2
Amblyomma cajennense	*Amblyomma cajennense*	1	2	2
Amblyomma cajennense	*Amblyomma cajennense*	1	2	2
Amblyomma cajennense	*Amblyomma cajennense*	1	2	2
Amblyomma cajennense	*Amblyomma cajennense*	1	2	2
Amblyomma cajennense	*Amblyomma cajennense*	1	2	2
Amblyomma cajennense	*Amblyomma cajennense*	1	2	2
Amblyomma cajennense	*Amblyomma cajennense*	1	2	2
Amblyomma cajennense	*Amblyomma cajennense*	1	2	2

Amblyomma cajennense	*Amblyomma cajennense*	1	2	2
Amblyomma cajennense	*Amblyomma cajennense*	1	2	2
Amblyomma dubitatum	*Amblyomma aureolatum*	0	3	1
Amblyomma dubitatum	*Amblyomma dubitatum*	1	3	3
Amblyomma dubitatum	*Amblyomma dubitatum*	1	3	3
Amblyomma dubitatum	*Amblyomma aureolatum*	0	3	1
Amblyomma dubitatum	*Amblyomma dubitatum*	1	3	3
Amblyomma dubitatum	*Amblyomma cajennense*	0	3	2
Amblyomma dubitatum	*Amblyomma dubitatum*	1	3	3
Amblyomma dubitatum	*Amblyomma dubitatum*	1	3	3
Amblyomma dubitatum	*Amblyomma dubitatum*	1	3	3
Amblyomma dubitatum	*Amblyomma dubitatum*	1	3	3
Amblyomma dubitatum	*Amblyomma dubitatum*	1	3	3
Amblyomma dubitatum	*Amblyomma dubitatum*	1	3	3
Amblyomma dubitatum	*Amblyomma dubitatum*	1	3	3
Amblyomma dubitatum	*Amblyomma dubitatum*	1	3	3
Amblyomma dubitatum	*Amblyomma dubitatum*	1	3	3
Amblyomma dubitatum	*Amblyomma aureolatum*	0	3	1
Amblyomma dubitatum	*Amblyomma dubitatum*	1	3	3
Amblyomma dubitatum	*Amblyomma dubitatum*	1	3	3
Amblyomma dubitatum	*Amblyomma dubitatum*	1	3	3
Amblyomma dubitatum	*Amblyomma dubitatum*	1	3	3
Amblyomma dubitatum	*Amblyomma dubitatum*	1	3	3
Amblyomma dubitatum	*Amblyomma dubitatum*	1	3	3
Amblyomma ovale	*Amblyomma ovale*	1	4	4
Amblyomma ovale	*Amblyomma ovale*	1	4	4
Amblyomma ovale	*Amblyomma ovale*	1	4	4
Amblyomma ovale	*Amblyomma ovale*	1	4	4
Amblyomma ovale	*Amblyomma ovale*	1	4	4
Amblyomma ovale	*Amblyomma ovale*	1	4	4
Amblyomma ovale	*Amblyomma ovale*	1	4	4
Amblyomma ovale	*Amblyomma ovale*	1	4	4
Amblyomma ovale	*Amblyomma ovale*	1	4	4
Amblyomma ovale	*Amblyomma ovale*	1	4	4
Amblyomma ovale	*Amblyomma ovale*	1	4	4
Amblyomma ovale	*Amblyomma ovale*	1	4	4
Amblyomma ovale	*Amblyomma ovale*	1	4	4
Amblyomma ovale	*Amblyomma ovale*	1	4	4
Amblyomma ovale	*Amblyomma ovale*	1	4	4
Amblyomma ovale	*Amblyomma ovale*	1	4	4
Amblyomma ovale	*Amblyomma ovale*	1	4	4
Amblyomma ovale	*Amblyomma ovale*	1	4	4
Amblyomma ovale	*Amblyomma ovale*	1	4	4
Amblyomma ovale	*Amblyomma ovale*	1	4	4
Amblyomma ovale	*Amblyomma ovale*	1	4	4
Amblyomma ovale	*Amblyomma ovale*	1	4	4
Amblyomma ovale	*Amblyomma ovale*	1	4	4
Amblyomma ovale	*Amblyomma ovale*	1	4	4
Amblyomma sculptum	*Amblyomma sculptum*	1	5	5
Amblyomma sculptum	*Amblyomma sculptum*	1	5	5
Amblyomma sculptum	*Amblyomma sculptum*	1	5	5
Amblyomma sculptum	*Amblyomma sculptum*	1	5	5
Amblyomma sculptum	*Amblyomma sculptum*	1	5	5
Amblyomma sculptum	*Amblyomma sculptum*	1	5	5
Amblyomma sculptum	*Amblyomma sculptum*	1	5	5
Amblyomma sculptum	*Amblyomma sculptum*	1	5	5
Amblyomma sculptum	*Amblyomma sculptum*	1	5	5
Amblyomma sculptum	*Amblyomma sculptum*	1	5	5
Amblyomma sculptum	*Amblyomma sculptum*	1	5	5
Amblyomma sculptum	*Amblyomma sculptum*	1	5	5
Amblyomma sculptum	*Amblyomma sculptum*	1	5	5
Amblyomma sculptum	*Amblyomma sculptum*	1	5	5
Amblyomma sculptum	*Amblyomma sculptum*	1	5	5
Amblyomma sculptum	*Amblyomma sculptum*	1	5	5
Amblyomma sculptum	*Amblyomma sculptum*	1	5	5
Amblyomma sculptum	*Amblyomma sculptum*	1	5	5
Amblyomma sculptum	*Amblyomma sculptum*	1	5	5

Amblyomma sculptum	*Amblyomma sculptum*	1	5	5
Amblyomma sculptum	*Amblyomma sculptum*	1	5	5
Amblyomma sculptum	*Amblyomma sculptum*	1	5	5
Amblyomma sculptum	*Amblyomma sculptum*	1	5	5
Amblyomma sculptum	*Amblyomma sculptum*	1	5	5
Amblyomma sculptum	*Amblyomma sculptum*	1	5	5
Amblyomma sculptum	*Amblyomma sculptum*	1	5	5
Amblyomma sculptum	*Amblyomma sculptum*	1	5	5
Amblyomma sculptum	*Amblyomma sculptum*	1	5	5
Amblyomma sculptum	*Amblyomma sculptum*	1	5	5
Amblyomma sculptum	*Amblyomma sculptum*	1	5	5
Amblyomma sculptum	*Amblyomma sculptum*	1	5	5
Amblyomma triste	*Amblyomma triste*	1	6	6
Amblyomma triste	*Amblyomma triste*	1	6	6
Amblyomma triste	*Amblyomma triste*	1	6	6
Amblyomma triste	*Amblyomma triste*	1	6	6
Amblyomma triste	*Amblyomma triste*	1	6	6
Amblyomma triste	*Amblyomma triste*	1	6	6
Amblyomma triste	*Amblyomma triste*	1	6	6
Amblyomma triste	*Amblyomma triste*	1	6	6
Amblyomma triste	*Amblyomma triste*	1	6	6
Amblyomma triste	*Amblyomma triste*	1	6	6
Amblyomma triste	*Amblyomma triste*	1	6	6
Amblyomma triste	*Amblyomma triste*	1	6	6
Amblyomma triste	*Amblyomma triste*	1	6	6
Amblyomma triste	*Amblyomma triste*	1	6	6
Amblyomma triste	*Amblyomma triste*	1	6	6
Amblyomma triste	*Amblyomma triste*	1	6	6
Amblyomma triste	*Amblyomma triste*	1	6	6
Amblyomma triste	*Amblyomma triste*	1	6	6
Amblyomma triste	*Amblyomma cajennense*	0	6	2
Amblyomma triste	*Amblyomma triste*	1	6	6
Amblyomma triste	*Amblyomma triste*	1	6	6
Amblyomma triste	*Amblyomma triste*	1	6	6
Amblyomma triste	*Amblyomma triste*	1	6	6
Amblyomma triste	*Amblyomma triste*	1	6	6
Amblyomma triste	*Amblyomma triste*	1	6	6
Amblyomma triste	*Amblyomma triste*	1	6	6
Amblyomma triste	*Amblyomma triste*	1	6	6
Amblyomma triste	*Amblyomma dubitatum*	0	6	3
Amblyomma triste	*Amblyomma triste*	1	6	6
Amblyomma triste	*Amblyomma triste*	1	6	6
Amblyomma triste	*Amblyomma triste*	1	6	6
Amblyomma aureolatum	*Amblyomma aureolatum*	1	1	1
Amblyomma aureolatum	*Amblyomma aureolatum*	1	1	1
Amblyomma aureolatum	*Amblyomma aureolatum*	1	1	1
Amblyomma aureolatum	*Amblyomma aureolatum*	1	1	1
Amblyomma aureolatum	*Amblyomma aureolatum*	1	1	1
Amblyomma aureolatum	*Amblyomma aureolatum*	1	1	1
Amblyomma aureolatum	*Amblyomma aureolatum*	1	1	1
Amblyomma aureolatum	*Amblyomma aureolatum*	1	1	1
Amblyomma aureolatum	*Amblyomma aureolatum*	1	1	1
Amblyomma aureolatum	*Amblyomma aureolatum*	1	1	1
Amblyomma aureolatum	*Amblyomma aureolatum*	1	1	1
Amblyomma aureolatum	*Amblyomma aureolatum*	1	1	1
Amblyomma aureolatum	*Amblyomma aureolatum*	1	1	1
Amblyomma aureolatum	*Amblyomma aureolatum*	1	1	1
Amblyomma aureolatum	*Amblyomma aureolatum*	1	1	1
Amblyomma aureolatum	*Amblyomma aureolatum*	1	1	1
Amblyomma aureolatum	*Amblyomma aureolatum*	1	1	1
Amblyomma aureolatum	*Amblyomma aureolatum*	1	1	1
Amblyomma aureolatum	*Amblyomma aureolatum*	1	1	1
Amblyomma aureolatum	*Amblyomma aureolatum*	1	1	1
Amblyomma aureolatum	*Amblyomma aureolatum*	1	1	1
Amblyomma aureolatum	*Amblyomma aureolatum*	1	1	1
Amblyomma aureolatum	*Amblyomma aureolatum*	1	1	1
Amblyomma aureolatum	*Amblyomma aureolatum*	1	1	1

Amblyomma cajennense	*Amblyomma cajennense*	1	2	2
Amblyomma cajennense	*Amblyomma cajennense*	1	2	2
Amblyomma cajennense	*Amblyomma triste*	0	2	6
Amblyomma cajennense	*Amblyomma cajennense*	1	2	2
Amblyomma cajennense	*Amblyomma cajennense*	1	2	2
Amblyomma cajennense	*Amblyomma cajennense*	1	2	2
Amblyomma cajennense	*Amblyomma cajennense*	1	2	2
Amblyomma cajennense	*Amblyomma cajennense*	1	2	2
Amblyomma cajennense	*Amblyomma cajennense*	1	2	2
Amblyomma cajennense	*Amblyomma cajennense*	1	2	2
Amblyomma cajennense	*Amblyomma cajennense*	1	2	2
Amblyomma cajennense	*Amblyomma cajennense*	1	2	2
Amblyomma cajennense	*Amblyomma cajennense*	1	2	2
Amblyomma cajennense	*Amblyomma cajennense*	1	2	2
Amblyomma cajennense	*Amblyomma cajennense*	1	2	2
Amblyomma cajennense	*Amblyomma cajennense*	1	2	2
Amblyomma cajennense	*Amblyomma cajennense*	1	2	2
Amblyomma cajennense	*Amblyomma cajennense*	1	2	2
Amblyomma cajennense	*Amblyomma cajennense*	1	2	2
Amblyomma cajennense	*Amblyomma cajennense*	1	2	2
Amblyomma cajennense	*Amblyomma cajennense*	1	2	2
Amblyomma cajennense	*Amblyomma cajennense*	1	2	2
Amblyomma cajennense	*Amblyomma cajennense*	1	2	2
Amblyomma cajennense	*Amblyomma cajennense*	1	2	2
Amblyomma cajennense	*Amblyomma cajennense*	1	2	2
Amblyomma cajennense	*Amblyomma cajennense*	1	2	2
Amblyomma cajennense	*Amblyomma cajennense*	1	2	2
Amblyomma cajennense	*Amblyomma cajennense*	1	2	2
Amblyomma cajennense	*Amblyomma cajennense*	1	2	2
Amblyomma cajennense	*Amblyomma cajennense*	1	2	2
Amblyomma cajennense	*Amblyomma cajennense*	1	2	2
Amblyomma cajennense	*Amblyomma cajennense*	1	2	2
Amblyomma cajennense	*Amblyomma sculptum*	0	2	5
Amblyomma dubitatum	*Amblyomma dubitatum*	1	3	3
Amblyomma dubitatum	*Amblyomma dubitatum*	1	3	3
Amblyomma dubitatum	*Amblyomma sculptum*	0	3	5
Amblyomma dubitatum	*Amblyomma sculptum*	0	3	5
Amblyomma dubitatum	*Amblyomma aureolatum*	0	3	1
Amblyomma dubitatum	*Amblyomma dubitatum*	1	3	3
Amblyomma dubitatum	*Amblyomma dubitatum*	1	3	3
Amblyomma dubitatum	*Amblyomma dubitatum*	1	3	3
Amblyomma dubitatum	*Amblyomma dubitatum*	1	3	3
Amblyomma dubitatum	*Amblyomma dubitatum*	1	3	3
Amblyomma dubitatum	*Amblyomma dubitatum*	1	3	3
Amblyomma dubitatum	*Amblyomma dubitatum*	1	3	3
Amblyomma dubitatum	*Amblyomma dubitatum*	1	3	3
Amblyomma dubitatum	*Amblyomma dubitatum*	1	3	3
Amblyomma dubitatum	*Amblyomma dubitatum*	1	3	3
Amblyomma dubitatum	*Amblyomma dubitatum*	1	3	3
Amblyomma dubitatum	*Amblyomma aureolatum*	0	3	1
Amblyomma dubitatum	*Amblyomma triste*	0	3	6
Amblyomma dubitatum	*Amblyomma aureolatum*	0	3	1
Amblyomma dubitatum	*Amblyomma dubitatum*	1	3	3
Amblyomma dubitatum	*Amblyomma dubitatum*	1	3	3
Amblyomma dubitatum	*Amblyomma dubitatum*	1	3	3
Amblyomma ovale	*Amblyomma ovale*	1	4	4
Amblyomma ovale	*Amblyomma aureolatum*	0	4	1
Amblyomma ovale	*Amblyomma ovale*	1	4	4
Amblyomma ovale	*Amblyomma ovale*	1	4	4
Amblyomma ovale	*Amblyomma ovale*	1	4	4
Amblyomma ovale	*Amblyomma ovale*	1	4	4
Amblyomma ovale	*Amblyomma ovale*	1	4	4
Amblyomma ovale	*Amblyomma ovale*	1	4	4
Amblyomma ovale	*Amblyomma ovale*	1	4	4
Amblyomma ovale	*Amblyomma ovale*	1	4	4
Amblyomma ovale	*Amblyomma aureolatum*	0	4	1
Amblyomma ovale	*Amblyomma ovale*	1	4	4

Amblyomma ovale	*Amblyomma ovale*	1	4	4
Amblyomma ovale	*Amblyomma ovale*	1	4	4
Amblyomma ovale	*Amblyomma ovale*	1	4	4
Amblyomma ovale	*Amblyomma ovale*	1	4	4
Amblyomma ovale	*Amblyomma ovale*	1	4	4
Amblyomma ovale	*Amblyomma aureolatum*	0	4	1
Amblyomma ovale	*Amblyomma aureolatum*	0	4	1
Amblyomma ovale	*Amblyomma sculptum*	0	4	5
Amblyomma ovale	*Amblyomma sculptum*	0	4	5
Amblyomma ovale	*Amblyomma ovale*	1	4	4
Amblyomma ovale	*Amblyomma ovale*	1	4	4
Amblyomma ovale	*Amblyomma ovale*	1	4	4
Amblyomma sculptum	*Amblyomma sculptum*	1	5	5
Amblyomma sculptum	*Amblyomma sculptum*	1	5	5
Amblyomma sculptum	*Amblyomma sculptum*	1	5	5
Amblyomma sculptum	*Amblyomma sculptum*	1	5	5
Amblyomma sculptum	*Amblyomma sculptum*	1	5	5
Amblyomma sculptum	*Amblyomma sculptum*	1	5	5
Amblyomma sculptum	*Amblyomma sculptum*	1	5	5
Amblyomma sculptum	*Amblyomma sculptum*	1	5	5
Amblyomma sculptum	*Amblyomma sculptum*	1	5	5
Amblyomma sculptum	*Amblyomma sculptum*	1	5	5
Amblyomma sculptum	*Amblyomma sculptum*	1	5	5
Amblyomma sculptum	*Amblyomma sculptum*	1	5	5
Amblyomma sculptum	*Amblyomma sculptum*	1	5	5
Amblyomma sculptum	*Amblyomma sculptum*	1	5	5
Amblyomma sculptum	*Amblyomma sculptum*	1	5	5
Amblyomma sculptum	*Amblyomma sculptum*	1	5	5
Amblyomma sculptum	*Amblyomma sculptum*	1	5	5
Amblyomma sculptum	*Amblyomma sculptum*	1	5	5
Amblyomma sculptum	*Amblyomma sculptum*	1	5	5
Amblyomma sculptum	*Amblyomma sculptum*	1	5	5
Amblyomma sculptum	*Amblyomma sculptum*	1	5	5
Amblyomma sculptum	*Amblyomma sculptum*	1	5	5
Amblyomma sculptum	*Amblyomma sculptum*	1	5	5
Amblyomma sculptum	*Amblyomma dubitatum*	0	5	3
Amblyomma sculptum	*Amblyomma sculptum*	1	5	5
Amblyomma sculptum	*Amblyomma sculptum*	1	5	5
Amblyomma sculptum	*Amblyomma sculptum*	1	5	5
Amblyomma sculptum	*Amblyomma sculptum*	1	5	5
Amblyomma sculptum	*Amblyomma sculptum*	1	5	5
Amblyomma sculptum	*Amblyomma sculptum*	1	5	5
Amblyomma sculptum	*Amblyomma sculptum*	1	5	5
Amblyomma triste	*Amblyomma triste*	1	6	6
Amblyomma triste	*Amblyomma triste*	1	6	6
Amblyomma triste	*Amblyomma triste*	1	6	6
Amblyomma triste	*Amblyomma triste*	1	6	6
Amblyomma triste	*Amblyomma triste*	1	6	6
Amblyomma triste	*Amblyomma triste*	1	6	6
Amblyomma triste	*Amblyomma triste*	1	6	6
Amblyomma triste	*Amblyomma triste*	1	6	6
Amblyomma triste	*Amblyomma triste*	1	6	6
Amblyomma triste	*Amblyomma triste*	1	6	6
Amblyomma triste	*Amblyomma triste*	1	6	6
Amblyomma triste	*Amblyomma triste*	1	6	6
Amblyomma triste	*Amblyomma triste*	1	6	6
Amblyomma triste	*Amblyomma aureolatum*	0	6	1
Amblyomma triste	*Amblyomma triste*	1	6	6
Amblyomma triste	*Amblyomma triste*	1	6	6
Amblyomma triste	*Amblyomma triste*	1	6	6
Amblyomma triste	*Amblyomma triste*	1	6	6
Amblyomma triste	*Amblyomma triste*	1	6	6
Amblyomma triste	*Amblyomma triste*	1	6	6
Amblyomma triste	*Amblyomma triste*	1	6	6
Amblyomma triste	*Amblyomma cajennense*	0	6	2
Amblyomma triste	*Amblyomma triste*	1	6	6
Amblyomma triste	*Amblyomma triste*	1	6	6

Amblyomma triste	*Amblyomma triste*	1	6	6
Amblyomma triste	*Amblyomma triste*	1	6	6
Amblyomma triste	*Amblyomma triste*	1	6	6
Amblyomma triste	*Amblyomma triste*	1	6	6
Amblyomma triste	*Amblyomma triste*	1	6	6
Amblyomma triste	*Amblyomma triste*	1	6	6
Amblyomma triste	*Amblyomma triste*	1	6	6
Amblyomma aureolatum	*Amblyomma aureolatum*	1	1	1
Amblyomma aureolatum	*Amblyomma aureolatum*	1	1	1
Amblyomma aureolatum	*Amblyomma aureolatum*	1	1	1
Amblyomma aureolatum	*Amblyomma aureolatum*	1	1	1
Amblyomma aureolatum	*Amblyomma triste*	0	1	6
Amblyomma aureolatum	*Amblyomma aureolatum*	1	1	1
Amblyomma aureolatum	*Amblyomma aureolatum*	1	1	1
Amblyomma aureolatum	*Amblyomma aureolatum*	1	1	1
Amblyomma aureolatum	*Amblyomma aureolatum*	1	1	1
Amblyomma aureolatum	*Amblyomma ovale*	0	1	4
Amblyomma aureolatum	*Amblyomma aureolatum*	1	1	1
Amblyomma aureolatum	*Amblyomma aureolatum*	1	1	1
Amblyomma aureolatum	*Amblyomma aureolatum*	1	1	1
Amblyomma aureolatum	*Amblyomma aureolatum*	1	1	1
Amblyomma aureolatum	*Amblyomma aureolatum*	1	1	1
Amblyomma aureolatum	*Amblyomma aureolatum*	1	1	1
Amblyomma aureolatum	*Amblyomma aureolatum*	1	1	1
Amblyomma aureolatum	*Amblyomma aureolatum*	1	1	1
Amblyomma aureolatum	*Amblyomma aureolatum*	1	1	1
Amblyomma aureolatum	*Amblyomma aureolatum*	1	1	1
Amblyomma aureolatum	*Amblyomma aureolatum*	1	1	1
Amblyomma aureolatum	*Amblyomma aureolatum*	1	1	1
Amblyomma aureolatum	*Amblyomma aureolatum*	1	1	1
Amblyomma aureolatum	*Amblyomma aureolatum*	1	1	1
Amblyomma aureolatum	*Amblyomma aureolatum*	1	1	1
Amblyomma cajennense	*Amblyomma cajennense*	1	2	2
Amblyomma cajennense	*Amblyomma cajennense*	1	2	2
Amblyomma cajennense	*Amblyomma cajennense*	1	2	2
Amblyomma cajennense	*Amblyomma cajennense*	1	2	2
Amblyomma cajennense	*Amblyomma cajennense*	1	2	2
Amblyomma cajennense	*Amblyomma cajennense*	1	2	2
Amblyomma cajennense	*Amblyomma cajennense*	1	2	2
Amblyomma cajennense	*Amblyomma triste*	0	2	6
Amblyomma cajennense	*Amblyomma cajennense*	1	2	2
Amblyomma cajennense	*Amblyomma cajennense*	1	2	2
Amblyomma cajennense	*Amblyomma cajennense*	1	2	2
Amblyomma cajennense	*Amblyomma triste*	0	2	6
Amblyomma cajennense	*Amblyomma cajennense*	1	2	2
Amblyomma cajennense	*Amblyomma cajennense*	1	2	2
Amblyomma cajennense	*Amblyomma cajennense*	1	2	2
Amblyomma cajennense	*Amblyomma cajennense*	1	2	2
Amblyomma cajennense	*Amblyomma cajennense*	1	2	2
Amblyomma cajennense	*Amblyomma cajennense*	1	2	2
Amblyomma cajennense	*Amblyomma cajennense*	1	2	2
Amblyomma cajennense	*Amblyomma cajennense*	1	2	2
Amblyomma cajennense	*Amblyomma cajennense*	1	2	2
Amblyomma cajennense	*Amblyomma cajennense*	1	2	2
Amblyomma cajennense	*Amblyomma cajennense*	1	2	2
Amblyomma cajennense	*Amblyomma cajennense*	1	2	2
Amblyomma cajennense	*Amblyomma cajennense*	1	2	2
Amblyomma cajennense	*Amblyomma cajennense*	1	2	2
Amblyomma cajennense	*Amblyomma cajennense*	1	2	2
Amblyomma cajennense	*Amblyomma cajennense*	1	2	2
Amblyomma cajennense	*Amblyomma cajennense*	1	2	2
Amblyomma cajennense	*Amblyomma cajennense*	1	2	2
Amblyomma cajennense	*Amblyomma cajennense*	1	2	2
Amblyomma cajennense	*Amblyomma cajennense*	1	2	2
Amblyomma cajennense	*Amblyomma cajennense*	1	2	2
Amblyomma dubitatum	*Amblyomma dubitatum*	1	3	3
Amblyomma dubitatum	*Amblyomma dubitatum*	1	3	3

Amblyomma dubitatum	*Amblyomma dubitatum*	1	3	3
Amblyomma dubitatum	*Amblyomma dubitatum*	1	3	3
Amblyomma dubitatum	*Amblyomma dubitatum*	1	3	3
Amblyomma dubitatum	*Amblyomma ovale*	0	3	4
Amblyomma dubitatum	*Amblyomma dubitatum*	1	3	3
Amblyomma dubitatum	*Amblyomma dubitatum*	1	3	3
Amblyomma dubitatum	*Amblyomma dubitatum*	1	3	3
Amblyomma dubitatum	*Amblyomma dubitatum*	1	3	3
Amblyomma dubitatum	*Amblyomma dubitatum*	1	3	3
Amblyomma dubitatum	*Amblyomma dubitatum*	1	3	3
Amblyomma dubitatum	*Amblyomma dubitatum*	1	3	3
Amblyomma dubitatum	*Amblyomma triste*	0	3	6
Amblyomma dubitatum	*Amblyomma dubitatum*	1	3	3
Amblyomma dubitatum	*Amblyomma ovale*	0	3	4
Amblyomma dubitatum	*Amblyomma dubitatum*	1	3	3
Amblyomma dubitatum	*Amblyomma aureolatum*	0	3	1
Amblyomma dubitatum	*Amblyomma dubitatum*	1	3	3
Amblyomma dubitatum	*Amblyomma dubitatum*	1	3	3
Amblyomma dubitatum	*Amblyomma dubitatum*	1	3	3
Amblyomma dubitatum	*Amblyomma dubitatum*	1	3	3
Amblyomma ovale	*Amblyomma ovale*	1	4	4
Amblyomma ovale	*Amblyomma aureolatum*	0	4	1
Amblyomma ovale	*Amblyomma ovale*	1	4	4
Amblyomma ovale	*Amblyomma triste*	0	4	6
Amblyomma ovale	*Amblyomma ovale*	1	4	4
Amblyomma ovale	*Amblyomma ovale*	1	4	4
Amblyomma ovale	*Amblyomma ovale*	1	4	4
Amblyomma ovale	*Amblyomma ovale*	1	4	4
Amblyomma ovale	*Amblyomma ovale*	1	4	4
Amblyomma ovale	*Amblyomma ovale*	1	4	4
Amblyomma ovale	*Amblyomma ovale*	1	4	4
Amblyomma ovale	*Amblyomma ovale*	1	4	4
Amblyomma ovale	*Amblyomma ovale*	1	4	4
Amblyomma ovale	*Amblyomma ovale*	1	4	4
Amblyomma ovale	*Amblyomma ovale*	1	4	4
Amblyomma ovale	*Amblyomma triste*	0	4	6
Amblyomma ovale	*Amblyomma triste*	0	4	6
Amblyomma ovale	*Amblyomma ovale*	1	4	4
Amblyomma ovale	*Amblyomma ovale*	1	4	4
Amblyomma ovale	*Amblyomma ovale*	1	4	4
Amblyomma ovale	*Amblyomma ovale*	1	4	4
Amblyomma ovale	*Amblyomma ovale*	1	4	4
Amblyomma ovale	*Amblyomma ovale*	1	4	4
Amblyomma ovale	*Amblyomma ovale*	1	4	4
Amblyomma ovale	*Amblyomma ovale*	1	4	4
Amblyomma sculptum	*Amblyomma sculptum*	1	5	5
Amblyomma sculptum	*Amblyomma sculptum*	1	5	5
Amblyomma sculptum	*Amblyomma sculptum*	1	5	5
Amblyomma sculptum	*Amblyomma sculptum*	1	5	5
Amblyomma sculptum	*Amblyomma sculptum*	1	5	5
Amblyomma sculptum	*Amblyomma sculptum*	1	5	5
Amblyomma sculptum	*Amblyomma sculptum*	1	5	5
Amblyomma sculptum	*Amblyomma sculptum*	1	5	5
Amblyomma sculptum	*Amblyomma sculptum*	1	5	5
Amblyomma sculptum	*Amblyomma sculptum*	1	5	5
Amblyomma sculptum	*Amblyomma dubitatum*	0	5	3
Amblyomma sculptum	*Amblyomma sculptum*	1	5	5
Amblyomma sculptum	*Amblyomma sculptum*	1	5	5
Amblyomma sculptum	*Amblyomma sculptum*	1	5	5
Amblyomma sculptum	*Amblyomma sculptum*	1	5	5
Amblyomma sculptum	*Amblyomma sculptum*	1	5	5
Amblyomma sculptum	*Amblyomma sculptum*	1	5	5
Amblyomma sculptum	*Amblyomma sculptum*	1	5	5
Amblyomma sculptum	*Amblyomma sculptum*	1	5	5
Amblyomma sculptum	*Amblyomma sculptum*	1	5	5
Amblyomma sculptum	*Amblyomma sculptum*	1	5	5
Amblyomma sculptum	*Amblyomma sculptum*	1	5	5

Amblyomma sculptum	*Amblyomma sculptum*	1	5	5
Amblyomma sculptum	*Amblyomma sculptum*	1	5	5
Amblyomma sculptum	*Amblyomma sculptum*	1	5	5
Amblyomma sculptum	*Amblyomma sculptum*	1	5	5
Amblyomma sculptum	*Amblyomma sculptum*	1	5	5
Amblyomma sculptum	*Amblyomma sculptum*	1	5	5
Amblyomma sculptum	*Amblyomma sculptum*	1	5	5
Amblyomma sculptum	*Amblyomma sculptum*	1	5	5
Amblyomma sculptum	*Amblyomma sculptum*	1	5	5
Amblyomma triste	*Amblyomma triste*	1	6	6
Amblyomma triste	*Amblyomma triste*	1	6	6
Amblyomma triste	*Amblyomma triste*	1	6	6
Amblyomma triste	*Amblyomma triste*	1	6	6
Amblyomma triste	*Amblyomma triste*	1	6	6
Amblyomma triste	*Amblyomma triste*	1	6	6
Amblyomma triste	*Amblyomma triste*	1	6	6
Amblyomma triste	*Amblyomma triste*	1	6	6
Amblyomma triste	*Amblyomma triste*	1	6	6
Amblyomma triste	*Amblyomma dubitatum*	0	6	3
Amblyomma triste	*Amblyomma triste*	1	6	6
Amblyomma triste	*Amblyomma triste*	1	6	6
Amblyomma triste	*Amblyomma ovale*	0	6	4
Amblyomma triste	*Amblyomma ovale*	0	6	4
Amblyomma triste	*Amblyomma triste*	1	6	6
Amblyomma triste	*Amblyomma triste*	1	6	6
Amblyomma triste	*Amblyomma triste*	1	6	6
Amblyomma triste	*Amblyomma triste*	1	6	6
Amblyomma triste	*Amblyomma triste*	1	6	6
Amblyomma triste	*Amblyomma triste*	1	6	6
Amblyomma triste	*Amblyomma triste*	1	6	6
Amblyomma triste	*Amblyomma triste*	1	6	6
Amblyomma triste	*Amblyomma triste*	1	6	6
Amblyomma triste	*Amblyomma triste*	1	6	6
Amblyomma triste	*Amblyomma triste*	1	6	6
Amblyomma triste	*Amblyomma triste*	1	6	6
Amblyomma triste	*Amblyomma triste*	1	6	6
Amblyomma triste	*Amblyomma triste*	1	6	6
Amblyomma triste	*Amblyomma triste*	1	6	6
Amblyomma triste	*Amblyomma triste*	1	6	6
Amblyomma triste	*Amblyomma triste*	1	6	6
Amblyomma aureolatum	*Amblyomma aureolatum*	1	1	1
Amblyomma aureolatum	*Amblyomma aureolatum*	1	1	1
Amblyomma aureolatum	*Amblyomma aureolatum*	1	1	1
Amblyomma aureolatum	*Amblyomma aureolatum*	1	1	1
Amblyomma aureolatum	*Amblyomma aureolatum*	1	1	1
Amblyomma aureolatum	*Amblyomma aureolatum*	1	1	1
Amblyomma aureolatum	*Amblyomma aureolatum*	1	1	1
Amblyomma aureolatum	*Amblyomma aureolatum*	1	1	1
Amblyomma aureolatum	*Amblyomma dubitatum*	0	1	3
Amblyomma aureolatum	*Amblyomma aureolatum*	1	1	1
Amblyomma aureolatum	*Amblyomma aureolatum*	1	1	1
Amblyomma aureolatum	*Amblyomma aureolatum*	1	1	1
Amblyomma aureolatum	*Amblyomma aureolatum*	1	1	1
Amblyomma aureolatum	*Amblyomma aureolatum*	1	1	1
Amblyomma aureolatum	*Amblyomma aureolatum*	1	1	1
Amblyomma aureolatum	*Amblyomma aureolatum*	1	1	1
Amblyomma aureolatum	*Amblyomma aureolatum*	1	1	1
Amblyomma aureolatum	*Amblyomma aureolatum*	1	1	1
Amblyomma aureolatum	*Amblyomma aureolatum*	1	1	1
Amblyomma aureolatum	*Amblyomma aureolatum*	1	1	1
Amblyomma aureolatum	*Amblyomma aureolatum*	1	1	1
Amblyomma aureolatum	*Amblyomma aureolatum*	1	1	1
Amblyomma aureolatum	*Amblyomma aureolatum*	1	1	1
Amblyomma aureolatum	*Amblyomma aureolatum*	1	1	1
Amblyomma cajennense	*Amblyomma cajennense*	1	2	2
Amblyomma cajennense	*Amblyomma cajennense*	1	2	2

Amblyomma cajennense	*Amblyomma cajennense*	1	2	2
Amblyomma cajennense	*Amblyomma cajennense*	1	2	2
Amblyomma cajennense	*Amblyomma cajennense*	1	2	2
Amblyomma cajennense	*Amblyomma cajennense*	1	2	2
Amblyomma cajennense	*Amblyomma cajennense*	1	2	2
Amblyomma cajennense	*Amblyomma cajennense*	1	2	2
Amblyomma cajennense	*Amblyomma cajennense*	1	2	2
Amblyomma cajennense	*Amblyomma cajennense*	1	2	2
Amblyomma cajennense	*Amblyomma cajennense*	1	2	2
Amblyomma cajennense	*Amblyomma cajennense*	1	2	2
Amblyomma cajennense	*Amblyomma cajennense*	1	2	2
Amblyomma cajennense	*Amblyomma cajennense*	1	2	2
Amblyomma cajennense	*Amblyomma cajennense*	1	2	2
Amblyomma cajennense	*Amblyomma cajennense*	1	2	2
Amblyomma cajennense	*Amblyomma cajennense*	1	2	2
Amblyomma cajennense	*Amblyomma cajennense*	1	2	2
Amblyomma cajennense	*Amblyomma cajennense*	1	2	2
Amblyomma cajennense	*Amblyomma cajennense*	1	2	2
Amblyomma cajennense	*Amblyomma cajennense*	1	2	2
Amblyomma cajennense	*Amblyomma cajennense*	1	2	2
Amblyomma cajennense	*Amblyomma cajennense*	1	2	2
Amblyomma cajennense	*Amblyomma cajennense*	1	2	2
Amblyomma cajennense	*Amblyomma cajennense*	1	2	2
Amblyomma cajennense	*Amblyomma cajennense*	1	2	2
Amblyomma cajennense	*Amblyomma cajennense*	1	2	2
Amblyomma cajennense	*Amblyomma cajennense*	1	2	2
Amblyomma cajennense	*Amblyomma cajennense*	1	2	2
Amblyomma cajennense	*Amblyomma cajennense*	1	2	2
Amblyomma cajennense	*Amblyomma cajennense*	1	2	2
Amblyomma cajennense	*Amblyomma cajennense*	1	2	2
Amblyomma cajennense	*Amblyomma cajennense*	1	2	2
Amblyomma cajennense	*Amblyomma cajennense*	1	2	2
Amblyomma dubitatum	*Amblyomma dubitatum*	1	3	3
Amblyomma dubitatum	*Amblyomma dubitatum*	1	3	3
Amblyomma dubitatum	*Amblyomma dubitatum*	1	3	3
Amblyomma dubitatum	*Amblyomma dubitatum*	1	3	3
Amblyomma dubitatum	*Amblyomma dubitatum*	1	3	3
Amblyomma dubitatum	*Amblyomma dubitatum*	1	3	3
Amblyomma dubitatum	*Amblyomma dubitatum*	1	3	3
Amblyomma dubitatum	*Amblyomma dubitatum*	1	3	3
Amblyomma dubitatum	*Amblyomma dubitatum*	1	3	3
Amblyomma dubitatum	*Amblyomma dubitatum*	1	3	3
Amblyomma dubitatum	*Amblyomma dubitatum*	1	3	3
Amblyomma dubitatum	*Amblyomma aureolatum*	0	3	1
Amblyomma dubitatum	*Amblyomma dubitatum*	1	3	3
Amblyomma dubitatum	*Amblyomma dubitatum*	1	3	3
Amblyomma dubitatum	*Amblyomma aureolatum*	0	3	1
Amblyomma dubitatum	*Amblyomma dubitatum*	1	3	3
Amblyomma dubitatum	*Amblyomma dubitatum*	1	3	3
Amblyomma dubitatum	*Amblyomma dubitatum*	1	3	3
Amblyomma dubitatum	*Amblyomma dubitatum*	1	3	3
Amblyomma dubitatum	*Amblyomma dubitatum*	1	3	3
Amblyomma dubitatum	*Amblyomma dubitatum*	1	3	3
Amblyomma ovale	*Amblyomma ovale*	1	4	4
Amblyomma ovale	*Amblyomma triste*	0	4	6
Amblyomma ovale	*Amblyomma sculptum*	0	4	5
Amblyomma ovale	*Amblyomma ovale*	1	4	4
Amblyomma ovale	*Amblyomma ovale*	1	4	4
Amblyomma ovale	*Amblyomma ovale*	1	4	4
Amblyomma ovale	*Amblyomma ovale*	1	4	4
Amblyomma ovale	*Amblyomma ovale*	1	4	4
Amblyomma ovale	*Amblyomma ovale*	1	4	4
Amblyomma ovale	*Amblyomma ovale*	1	4	4
Amblyomma ovale	*Amblyomma ovale*	1	4	4
Amblyomma ovale	*Amblyomma ovale*	1	4	4
Amblyomma ovale	*Amblyomma ovale*	1	4	4
Amblyomma ovale	*Amblyomma ovale*	1	4	4
Amblyomma ovale	*Amblyomma ovale*	1	4	4

Amblyomma ovale	*Amblyomma ovale*	1	4	4
Amblyomma ovale	*Amblyomma ovale*	1	4	4
Amblyomma ovale	*Amblyomma ovale*	1	4	4
Amblyomma ovale	*Amblyomma dubitatum*	0	4	3
Amblyomma ovale	*Amblyomma ovale*	1	4	4
Amblyomma ovale	*Amblyomma ovale*	1	4	4
Amblyomma ovale	*Amblyomma aureolatum*	0	4	1
Amblyomma ovale	*Amblyomma ovale*	1	4	4
Amblyomma ovale	*Amblyomma ovale*	1	4	4
Amblyomma sculptum	*Amblyomma sculptum*	1	5	5
Amblyomma sculptum	*Amblyomma sculptum*	1	5	5
Amblyomma sculptum	*Amblyomma sculptum*	1	5	5
Amblyomma sculptum	*Amblyomma sculptum*	1	5	5
Amblyomma sculptum	*Amblyomma sculptum*	1	5	5
Amblyomma sculptum	*Amblyomma sculptum*	1	5	5
Amblyomma sculptum	*Amblyomma sculptum*	1	5	5
Amblyomma sculptum	*Amblyomma dubitatum*	0	5	3
Amblyomma sculptum	*Amblyomma sculptum*	1	5	5
Amblyomma sculptum	*Amblyomma sculptum*	1	5	5
Amblyomma sculptum	*Amblyomma sculptum*	1	5	5
Amblyomma sculptum	*Amblyomma sculptum*	1	5	5
Amblyomma sculptum	*Amblyomma sculptum*	1	5	5
Amblyomma sculptum	*Amblyomma sculptum*	1	5	5
Amblyomma sculptum	*Amblyomma sculptum*	1	5	5
Amblyomma sculptum	*Amblyomma sculptum*	1	5	5
Amblyomma sculptum	*Amblyomma sculptum*	1	5	5
Amblyomma sculptum	*Amblyomma sculptum*	1	5	5
Amblyomma sculptum	*Amblyomma sculptum*	1	5	5
Amblyomma sculptum	*Amblyomma sculptum*	1	5	5
Amblyomma sculptum	*Amblyomma sculptum*	1	5	5
Amblyomma sculptum	*Amblyomma sculptum*	1	5	5
Amblyomma sculptum	*Amblyomma sculptum*	1	5	5
Amblyomma sculptum	*Amblyomma sculptum*	1	5	5
Amblyomma sculptum	*Amblyomma sculptum*	1	5	5
Amblyomma sculptum	*Amblyomma sculptum*	1	5	5
Amblyomma sculptum	*Amblyomma sculptum*	1	5	5
Amblyomma sculptum	*Amblyomma sculptum*	1	5	5
Amblyomma sculptum	*Amblyomma sculptum*	1	5	5
Amblyomma sculptum	*Amblyomma sculptum*	1	5	5
Amblyomma sculptum	*Amblyomma sculptum*	1	5	5
Amblyomma triste	*Amblyomma triste*	1	6	6
Amblyomma triste	*Amblyomma triste*	1	6	6
Amblyomma triste	*Amblyomma triste*	1	6	6
Amblyomma triste	*Amblyomma triste*	1	6	6
Amblyomma triste	*Amblyomma triste*	1	6	6
Amblyomma triste	*Amblyomma triste*	1	6	6
Amblyomma triste	*Amblyomma triste*	1	6	6
Amblyomma triste	*Amblyomma triste*	1	6	6
Amblyomma triste	*Amblyomma triste*	1	6	6
Amblyomma triste	*Amblyomma triste*	1	6	6
Amblyomma triste	*Amblyomma triste*	1	6	6
Amblyomma triste	*Amblyomma triste*	1	6	6
Amblyomma triste	*Amblyomma triste*	1	6	6
Amblyomma triste	*Amblyomma triste*	1	6	6
Amblyomma triste	*Amblyomma triste*	1	6	6
Amblyomma triste	*Amblyomma triste*	1	6	6
Amblyomma triste	*Amblyomma triste*	1	6	6
Amblyomma triste	*Amblyomma triste*	1	6	6
Amblyomma triste	*Amblyomma triste*	1	6	6
Amblyomma triste	*Amblyomma cajennense*	0	6	2
Amblyomma triste	*Amblyomma triste*	1	6	6
Amblyomma triste	*Amblyomma dubitatum*	0	6	3
Amblyomma triste	*Amblyomma triste*	1	6	6
Amblyomma triste	*Amblyomma triste*	1	6	6
Amblyomma triste	*Amblyomma triste*	1	6	6
Amblyomma triste	*Amblyomma triste*	1	6	6
Amblyomma triste	*Amblyomma triste*	1	6	6

Amblyomma triste	*Amblyomma triste*	1	6	6
Amblyomma triste	*Amblyomma triste*	1	6	6
Amblyomma triste	*Amblyomma triste*	1	6	6
Amblyomma triste	*Amblyomma triste*	1	6	6
