# Supplementary material for: Automated identification of spotted‐fever tick vectors using convolutional neural networks
Source: Med Vet Entomol. 2025 Jul 4;39(4):829–41. doi: 10.1111/mve.12822 (PMC12586270; doi:10.1111/mve.12822)
Supplement: Supplementary file 4 — Table S3. Hits, accuracy and 95% confidence interval in the identification of tick species images predicted by AlexNet, MobileNetV2 and ResNet‐50. [file MVE-39-829-s003.docx]

**Table S3.** Hits, accuracy and 95% confidence interval in the identification of tick species images predicted by AlexNet, MobileNetV2 and ResNet-50.

| **Algorithm** | **Group** | **Fold** | **Total** | **Correct** | **Accuracy** | **CI lower** | **CI upper** |
| --- | --- | --- | --- | --- | --- | --- | --- |
| AlexNet | All together | fold1 | 165 | 157 | 0.95 | 0.95 | 0.91 |
| AlexNet | All together | fold2 | 165 | 160 | 0.97 | 0.97 | 0.93 |
| AlexNet | All together | fold3 | 165 | 156 | 0.95 | 0.95 | 0.90 |
| AlexNet | All together | fold4 | 168 | 155 | 0.92 | 0.92 | 0.87 |
| AlexNet | All together | fold5 | 164 | 150 | 0.91 | 0.91 | 0.86 |
| AlexNet | Dorsal | fold1 | 83 | 73 | 0.88 | 0.88 | 0.79 |
| AlexNet | Dorsal | fold2 | 86 | 72 | 0.84 | 0.84 | 0.75 |
| AlexNet | Dorsal | fold3 | 84 | 73 | 0.87 | 0.87 | 0.78 |
| AlexNet | Dorsal | fold4 | 84 | 76 | 0.90 | 0.90 | 0.82 |
| AlexNet | Dorsal | fold5 | 86 | 81 | 0.94 | 0.94 | 0.87 |
| AlexNet | Female | fold1 | 75 | 62 | 0.83 | 0.83 | 0.73 |
| AlexNet | Female | fold2 | 74 | 71 | 0.96 | 0.96 | 0.89 |
| AlexNet | Female | fold3 | 72 | 63 | 0.88 | 0.88 | 0.78 |
| AlexNet | Female | fold4 | 73 | 66 | 0.90 | 0.90 | 0.82 |
| AlexNet | Female | fold5 | 74 | 65 | 0.88 | 0.88 | 0.78 |
| AlexNet | High resolution | fold1 | 99 | 95 | 0.96 | 0.96 | 0.90 |
| AlexNet | High resolution | fold2 | 101 | 90 | 0.89 | 0.89 | 0.82 |
| AlexNet | High resolution | fold3 | 100 | 93 | 0.93 | 0.93 | 0.86 |
| AlexNet | High resolution | fold4 | 99 | 95 | 0.96 | 0.96 | 0.90 |
| AlexNet | High resolution | fold5 | 100 | 91 | 0.91 | 0.91 | 0.84 |
| AlexNet | Low resolution | fold1 | 65 | 61 | 0.94 | 0.94 | 0.85 |
| AlexNet | Low resolution | fold2 | 66 | 61 | 0.92 | 0.92 | 0.83 |
| AlexNet | Low resolution | fold3 | 66 | 62 | 0.94 | 0.94 | 0.85 |
| AlexNet | Low resolution | fold4 | 65 | 58 | 0.89 | 0.89 | 0.79 |
| AlexNet | Low resolution | fold5 | 66 | 60 | 0.91 | 0.91 | 0.82 |
| AlexNet | Male | fold1 | 89 | 87 | 0.98 | 0.98 | 0.92 |
| AlexNet | Male | fold2 | 93 | 86 | 0.92 | 0.92 | 0.85 |
| AlexNet | Male | fold3 | 92 | 89 | 0.97 | 0.97 | 0.91 |
| AlexNet | Male | fold4 | 92 | 85 | 0.92 | 0.92 | 0.85 |
| AlexNet | Male | fold5 | 92 | 88 | 0.96 | 0.96 | 0.89 |
| AlexNet | Ventral | fold1 | 81 | 71 | 0.88 | 0.88 | 0.79 |
| AlexNet | Ventral | fold2 | 80 | 72 | 0.90 | 0.90 | 0.81 |
| AlexNet | Ventral | fold3 | 79 | 69 | 0.87 | 0.87 | 0.78 |
| AlexNet | Ventral | fold4 | 83 | 72 | 0.87 | 0.87 | 0.78 |
| AlexNet | Ventral | fold5 | 80 | 75 | 0.94 | 0.94 | 0.86 |
| MobileNetV2 | All together | fold1 | 165 | 152 | 0.92 | 0.92 | 0.87 |
| MobileNetV2 | All together | fold2 | 165 | 154 | 0.93 | 0.93 | 0.88 |
| MobileNetV2 | All together | fold3 | 165 | 152 | 0.92 | 0.92 | 0.87 |
| MobileNetV2 | All together | fold4 | 168 | 158 | 0.94 | 0.94 | 0.89 |
| MobileNetV2 | All together | fold5 | 164 | 158 | 0.96 | 0.96 | 0.92 |
| MobileNetV2 | Dorsal | fold1 | 83 | 74 | 0.89 | 0.89 | 0.81 |
| MobileNetV2 | Dorsal | fold2 | 86 | 75 | 0.87 | 0.87 | 0.79 |
| MobileNetV2 | Dorsal | fold3 | 84 | 76 | 0.90 | 0.90 | 0.82 |
| MobileNetV2 | Dorsal | fold4 | 84 | 75 | 0.89 | 0.89 | 0.81 |
| MobileNetV2 | Dorsal | fold5 | 86 | 77 | 0.90 | 0.90 | 0.81 |
| MobileNetV2 | Female | fold1 | 75 | 61 | 0.81 | 0.81 | 0.71 |
| MobileNetV2 | Female | fold2 | 74 | 66 | 0.89 | 0.89 | 0.80 |
| MobileNetV2 | Female | fold3 | 72 | 61 | 0.85 | 0.85 | 0.75 |
| MobileNetV2 | Female | fold4 | 73 | 64 | 0.88 | 0.88 | 0.78 |
| MobileNetV2 | Female | fold5 | 74 | 69 | 0.93 | 0.93 | 0.85 |
| MobileNetV2 | High resolution | fold1 | 99 | 85 | 0.86 | 0.86 | 0.78 |
| MobileNetV2 | High resolution | fold2 | 101 | 92 | 0.91 | 0.91 | 0.84 |
| MobileNetV2 | High resolution | fold3 | 100 | 88 | 0.88 | 0.88 | 0.80 |
| MobileNetV2 | High resolution | fold4 | 99 | 95 | 0.96 | 0.96 | 0.90 |
| MobileNetV2 | High resolution | fold5 | 100 | 89 | 0.89 | 0.89 | 0.81 |
| MobileNetV2 | Low resolution | fold1 | 65 | 58 | 0.89 | 0.89 | 0.79 |
| MobileNetV2 | Low resolution | fold2 | 66 | 63 | 0.95 | 0.95 | 0.87 |
| MobileNetV2 | Low resolution | fold3 | 66 | 61 | 0.92 | 0.92 | 0.83 |
| MobileNetV2 | Low resolution | fold4 | 65 | 58 | 0.89 | 0.89 | 0.79 |
| MobileNetV2 | Low resolution | fold5 | 66 | 61 | 0.92 | 0.92 | 0.83 |
| MobileNetV2 | Male | fold1 | 89 | 85 | 0.96 | 0.96 | 0.89 |
| MobileNetV2 | Male | fold2 | 93 | 85 | 0.91 | 0.91 | 0.84 |
| MobileNetV2 | Male | fold3 | 92 | 85 | 0.92 | 0.92 | 0.85 |
| MobileNetV2 | Male | fold4 | 92 | 89 | 0.97 | 0.97 | 0.91 |
| MobileNetV2 | Male | fold5 | 92 | 87 | 0.95 | 0.95 | 0.88 |
| MobileNetV2 | Ventral | fold1 | 81 | 69 | 0.85 | 0.85 | 0.76 |
| MobileNetV2 | Ventral | fold2 | 80 | 68 | 0.85 | 0.85 | 0.76 |
| MobileNetV2 | Ventral | fold3 | 79 | 69 | 0.87 | 0.87 | 0.78 |
| MobileNetV2 | Ventral | fold4 | 83 | 74 | 0.89 | 0.89 | 0.81 |
| MobileNetV2 | Ventral | fold5 | 80 | 75 | 0.94 | 0.94 | 0.86 |
| ResNet-50 | All together | fold1 | 165 | 151 | 0.92 | 0.92 | 0.86 |
| ResNet-50 | All together | fold2 | 165 | 158 | 0.96 | 0.96 | 0.92 |
| ResNet-50 | All together | fold3 | 165 | 148 | 0.90 | 0.90 | 0.84 |
| ResNet-50 | All together | fold4 | 168 | 152 | 0.90 | 0.90 | 0.85 |
| ResNet-50 | All together | fold5 | 164 | 154 | 0.94 | 0.94 | 0.89 |
| ResNet-50 | Dorsal | fold1 | 83 | 76 | 0.92 | 0.92 | 0.84 |
| ResNet-50 | Dorsal | fold2 | 86 | 71 | 0.83 | 0.83 | 0.73 |
| ResNet-50 | Dorsal | fold3 | 84 | 69 | 0.82 | 0.82 | 0.73 |
| ResNet-50 | Dorsal | fold4 | 84 | 75 | 0.89 | 0.89 | 0.81 |
| ResNet-50 | Dorsal | fold5 | 86 | 75 | 0.87 | 0.87 | 0.79 |
| ResNet-50 | Female | fold1 | 75 | 63 | 0.84 | 0.84 | 0.74 |
| ResNet-50 | Female | fold2 | 74 | 63 | 0.85 | 0.85 | 0.75 |
| ResNet-50 | Female | fold3 | 72 | 59 | 0.82 | 0.82 | 0.72 |
| ResNet-50 | Female | fold4 | 73 | 65 | 0.89 | 0.89 | 0.80 |
| ResNet-50 | Female | fold5 | 74 | 61 | 0.82 | 0.82 | 0.72 |
| ResNet-50 | High resolution | fold1 | 99 | 86 | 0.87 | 0.87 | 0.79 |
| ResNet-50 | High resolution | fold2 | 101 | 91 | 0.90 | 0.90 | 0.83 |
| ResNet-50 | High resolution | fold3 | 100 | 90 | 0.90 | 0.90 | 0.83 |
| ResNet-50 | High resolution | fold4 | 99 | 93 | 0.94 | 0.94 | 0.87 |
| ResNet-50 | High resolution | fold5 | 100 | 92 | 0.92 | 0.92 | 0.85 |
| ResNet-50 | Low resolution | fold1 | 65 | 60 | 0.92 | 0.92 | 0.83 |
| ResNet-50 | Low resolution | fold2 | 66 | 64 | 0.97 | 0.97 | 0.90 |
| ResNet-50 | Low resolution | fold3 | 66 | 58 | 0.88 | 0.88 | 0.78 |
| ResNet-50 | Low resolution | fold4 | 65 | 55 | 0.85 | 0.85 | 0.74 |
| ResNet-50 | Low resolution | fold5 | 66 | 61 | 0.92 | 0.92 | 0.83 |
| ResNet-50 | Male | fold1 | 89 | 82 | 0.92 | 0.92 | 0.85 |
| ResNet-50 | Male | fold2 | 93 | 87 | 0.94 | 0.94 | 0.87 |
| ResNet-50 | Male | fold3 | 92 | 88 | 0.96 | 0.96 | 0.89 |
| ResNet-50 | Male | fold4 | 92 | 83 | 0.90 | 0.90 | 0.82 |
| ResNet-50 | Male | fold5 | 92 | 89 | 0.97 | 0.97 | 0.91 |
| ResNet-50 | Ventral | fold1 | 81 | 71 | 0.88 | 0.88 | 0.79 |
| ResNet-50 | Ventral | fold2 | 80 | 74 | 0.93 | 0.93 | 0.85 |
| ResNet-50 | Ventral | fold3 | 79 | 74 | 0.94 | 0.94 | 0.86 |
| ResNet-50 | Ventral | fold4 | 83 | 72 | 0.87 | 0.87 | 0.78 |
| ResNet-50 | Ventral | fold5 | 80 | 70 | 0.88 | 0.88 | 0.79 |
