# Supplementary material for: Automated identification of spotted‐fever tick vectors using convolutional neural networks
Source: Med Vet Entomol. 2025 Jul 4;39(4):829–41. doi: 10.1111/mve.12822 (PMC12586270; doi:10.1111/mve.12822)
Supplement: Supplementary file 5 — Table S4. Sensitivity and specificity results observed in the study. [file MVE-39-829-s005.docx]

**Table S4**. Sensitivity and specificity results observed in the study.

| **Algorithm** | **Group** | **Species** | **"N"** | **Correct** | **Sensitivity** | | | **Specificity** | | |
| --- | --- | --- | --- | --- | --- | --- | --- | --- | --- | --- |
|  |  |  |  |  | Proportion | CI lower | CI upper | Proportion | CI lower | CI upper |
| AlexNet | Female | *A. aureolatum* | 58 | 52 | 0.90 | 0.79 | 0.95 | 0.96 | 0.94 | 0.98 |
|  |  | *A. cajennense* | 82 | 79 | 0.96 | 0.90 | 0.99 | 0.99 | 0.97 | 1.00 |
|  |  | *A. dubitatum* | 34 | 23 | 0.68 | 0.51 | 0.81 | 0.98 | 0.96 | 0.99 |
|  |  | *A. ovale* | 79 | 76 | 0.96 | 0.89 | 0.99 | 0.96 | 0.93 | 0.98 |
|  |  | *A. sculptum* | 63 | 53 | 0.84 | 0.73 | 0.91 | 0.98 | 0.96 | 0.99 |
|  |  | *A. triste* | 52 | 44 | 0.85 | 0.72 | 0.92 | 0.98 | 0.96 | 0.99 |
|  | Male | *A. aureolatum* | 63 | 63 | 1.00 | 0.94 | 1.00 | 0.99 | 0.97 | 1.00 |
|  |  | *A. cajennense* | 83 | 81 | 0.98 | 0.92 | 0.99 | 0.99 | 0.97 | 1.00 |
|  |  | *A. dubitatum* | 75 | 69 | 0.92 | 0.84 | 0.96 | 0.99 | 0.97 | 1.00 |
|  |  | *A. ovale* | 42 | 34 | 0.81 | 0.67 | 0.90 | 0.99 | 0.97 | 0.99 |
|  |  | *A. sculptum* | 92 | 90 | 0.98 | 0.92 | 0.99 | 0.99 | 0.98 | 1.00 |
|  |  | *A. triste* | 103 | 98 | 0.95 | 0.89 | 0.98 | 0.99 | 0.97 | 1.00 |
|  | Dorsal | *A. aureolatum* | 63 | 55 | 0.87 | 0.77 | 0.93 | 0.98 | 0.95 | 0.99 |
|  |  | *A. cajennense* | 83 | 79 | 0.95 | 0.88 | 0.98 | 0.99 | 0.97 | 0.99 |
|  |  | *A. dubitatum* | 56 | 39 | 0.70 | 0.57 | 0.80 | 0.99 | 0.97 | 0.99 |
|  |  | *A. ovale* | 61 | 51 | 0.84 | 0.72 | 0.91 | 0.99 | 0.98 | 1.00 |
|  |  | *A. sculptum* | 81 | 75 | 0.93 | 0.85 | 0.97 | 0.99 | 0.97 | 1.00 |
|  |  | *A. triste* | 79 | 76 | 0.96 | 0.89 | 0.99 | 0.94 | 0.91 | 0.96 |
|  | Ventral | *A. aureolatum* | 58 | 54 | 0.93 | 0.84 | 0.97 | 0.98 | 0.95 | 0.99 |
|  |  | *A. cajennense* | 82 | 78 | 0.95 | 0.88 | 0.98 | 0.98 | 0.96 | 0.99 |
|  |  | *A. dubitatum* | 52 | 44 | 0.85 | 0.72 | 0.92 | 0.98 | 0.96 | 0.99 |
|  |  | *A. ovale* | 60 | 49 | 0.82 | 0.70 | 0.89 | 0.98 | 0.95 | 0.99 |
|  |  | *A. sculptum* | 74 | 65 | 0.88 | 0.78 | 0.93 | 0.98 | 0.96 | 0.99 |
|  |  | *A. triste* | 77 | 69 | 0.90 | 0.81 | 0.95 | 0.97 | 0.94 | 0.98 |
|  | Low resolution | *A. aureolatum* | 53 | 45 | 0.85 | 0.73 | 0.92 | 0.98 | 0.96 | 0.99 |
|  |  | *A. cajennense* | 82 | 76 | 0.93 | 0.85 | 0.97 | 1.00 | 0.98 | 1.00 |
|  |  | *A. dubitatum* | 44 | 37 | 0.84 | 0.71 | 0.92 | 0.98 | 0.96 | 0.99 |
|  |  | *A. ovale* | 55 | 54 | 0.98 | 0.90 | 1.00 | 0.96 | 0.93 | 0.98 |
|  |  | *A. sculptum* | 17 | 16 | 0.94 | 0.73 | 0.99 | 1.00 | 0.98 | 1.00 |
|  |  | *A. triste* | 77 | 74 | 0.96 | 0.89 | 0.99 | 0.98 | 0.95 | 0.99 |
|  | High resolution | *A. aureolatum* | 68 | 66 | 0.97 | 0.90 | 0.99 | 0.97 | 0.95 | 0.98 |
|  |  | *A. cajennense* | 83 | 82 | 0.99 | 0.93 | 1.00 | 0.99 | 0.98 | 1.00 |
|  |  | *A. dubitatum* | 65 | 52 | 0.80 | 0.69 | 0.88 | 0.99 | 0.97 | 1.00 |
|  |  | *A. ovale* | 66 | 60 | 0.91 | 0.82 | 0.96 | 0.99 | 0.97 | 0.99 |
|  |  | *A. sculptum* | 138 | 133 | 0.96 | 0.92 | 0.98 | 0.99 | 0.98 | 1.00 |
|  |  | *A. triste* | 79 | 71 | 0.90 | 0.81 | 0.95 | 0.99 | 0.98 | 1.00 |
|  | All together | *A. aureolatum* | 121 | 117 | 0.97 | 0.92 | 0.99 | 0.98 | 0.97 | 0.99 |
|  |  | *A. cajennense* | 165 | 163 | 0.99 | 0.96 | 1.00 | 0.99 | 0.98 | 0.99 |
|  |  | *A. dubitatum* | 109 | 96 | 0.88 | 0.81 | 0.93 | 0.99 | 0.98 | 1.00 |
|  |  | *A. ovale* | 121 | 108 | 0.89 | 0.82 | 0.94 | 0.99 | 0.98 | 0.99 |
|  |  | *A. sculptum* | 155 | 143 | 0.92 | 0.87 | 0.96 | 0.99 | 0.98 | 0.99 |
|  |  | *A. triste* | 156 | 151 | 0.97 | 0.93 | 0.99 | 0.99 | 0.98 | 0.99 |
| MobileNetV2 | Female | *A. aureolatum* | 58 | 52 | 0.90 | 0.79 | 0.95 | 0.98 | 0.96 | 0.99 |
|  |  | *A. cajennense* | 82 | 79 | 0.96 | 0.90 | 0.99 | 0.96 | 0.93 | 0.98 |
|  |  | *A. dubitatum* | 34 | 20 | 0.59 | 0.42 | 0.74 | 0.97 | 0.95 | 0.98 |
|  |  | *A. ovale* | 79 | 72 | 0.91 | 0.83 | 0.96 | 0.98 | 0.96 | 0.99 |
|  |  | *A. sculptum* | 63 | 52 | 0.83 | 0.71 | 0.90 | 0.98 | 0.96 | 0.99 |
|  |  | *A. triste* | 52 | 46 | 0.88 | 0.77 | 0.95 | 0.97 | 0.95 | 0.98 |
|  | Male | *A. aureolatum* | 63 | 59 | 0.94 | 0.85 | 0.98 | 0.99 | 0.97 | 1.00 |
|  |  | *A. cajennense* | 83 | 81 | 0.98 | 0.92 | 0.99 | 0.98 | 0.97 | 0.99 |
|  |  | *A. dubitatum* | 75 | 71 | 0.95 | 0.87 | 0.98 | 0.99 | 0.97 | 0.99 |
|  |  | *A. ovale* | 42 | 35 | 0.83 | 0.69 | 0.92 | 0.99 | 0.97 | 0.99 |
|  |  | *A. sculptum* | 92 | 90 | 0.98 | 0.92 | 0.99 | 0.99 | 0.97 | 1.00 |
|  |  | *A. triste* | 103 | 95 | 0.92 | 0.85 | 0.96 | 0.99 | 0.98 | 1.00 |
|  | Dorsal | *A. aureolatum* | 63 | 55 | 0.86 | 0.75 | 0.92 | 0.97 | 0.94 | 0.98 |
|  |  | *A. cajennense* | 83 | 79 | 0.96 | 0.90 | 0.99 | 0.96 | 0.94 | 0.98 |
|  |  | *A. dubitatum* | 56 | 39 | 0.75 | 0.62 | 0.84 | 0.98 | 0.96 | 0.99 |
|  |  | *A. ovale* | 61 | 51 | 0.84 | 0.72 | 0.91 | 0.99 | 0.97 | 1.00 |
|  |  | *A. sculptum* | 81 | 75 | 0.94 | 0.86 | 0.97 | 0.99 | 0.97 | 0.99 |
|  |  | *A. triste* | 79 | 76 | 0.94 | 0.86 | 0.97 | 0.99 | 0.97 | 0.99 |
|  | Ventral | *A. aureolatum* | 58 | 56 | 0.97 | 0.88 | 0.99 | 0.97 | 0.95 | 0.98 |
|  |  | *A. cajennense* | 82 | 80 | 0.98 | 0.92 | 0.99 | 0.96 | 0.93 | 0.98 |
|  |  | *A. dubitatum* | 52 | 40 | 0.77 | 0.64 | 0.86 | 0.98 | 0.96 | 0.99 |
|  |  | *A. ovale* | 60 | 51 | 0.85 | 0.74 | 0.92 | 0.97 | 0.95 | 0.98 |
|  |  | *A. sculptum* | 74 | 62 | 0.84 | 0.74 | 0.90 | 0.99 | 0.97 | 1.00 |
|  |  | *A. triste* | 77 | 66 | 0.86 | 0.76 | 0.92 | 0.98 | 0.96 | 0.99 |
|  | Low resolution | *A. aureolatum* | 53 | 50 | 0.94 | 0.85 | 0.98 | 0.98 | 0.95 | 0.99 |
|  |  | *A. cajennense* | 82 | 77 | 0.94 | 0.87 | 0.97 | 0.99 | 0.97 | 1.00 |
|  |  | *A. dubitatum* | 44 | 40 | 0.91 | 0.79 | 0.96 | 0.98 | 0.95 | 0.99 |
|  |  | *A. ovale* | 55 | 43 | 0.78 | 0.66 | 0.87 | 0.99 | 0.97 | 1.00 |
|  |  | *A. sculptum* | 17 | 15 | 0.88 | 0.66 | 0.97 | 1.00 | 0.98 | 1.00 |
|  |  | *A. triste* | 77 | 76 | 0.99 | 0.93 | 1.00 | 0.97 | 0.94 | 0.98 |
|  | High resolution | *A. aureolatum* | 68 | 63 | 0.93 | 0.84 | 0.97 | 0.97 | 0.94 | 0.98 |
|  |  | *A. cajennense* | 83 | 82 | 0.99 | 0.93 | 1.00 | 0.98 | 0.96 | 0.99 |
|  |  | *A. dubitatum* | 65 | 47 | 0.72 | 0.60 | 0.82 | 0.99 | 0.97 | 1.00 |
|  |  | *A. ovale* | 66 | 53 | 0.80 | 0.69 | 0.88 | 0.98 | 0.96 | 0.99 |
|  |  | *A. sculptum* | 138 | 129 | 0.93 | 0.88 | 0.97 | 0.98 | 0.96 | 0.99 |
|  |  | *A. triste* | 79 | 75 | 0.95 | 0.88 | 0.98 | 0.99 | 0.97 | 0.99 |
|  | All together | *A. aureolatum* | 121 | 116 | 0.96 | 0.91 | 0.98 | 0.97 | 0.95 | 0.98 |
|  |  | *A. cajennense* | 165 | 162 | 0.98 | 0.95 | 0.99 | 0.99 | 0.98 | 1.00 |
|  |  | *A. dubitatum* | 109 | 95 | 0.87 | 0.80 | 0.92 | 0.99 | 0.97 | 0.99 |
|  |  | *A. ovale* | 121 | 102 | 0.84 | 0.77 | 0.90 | 1.00 | 0.99 | 1.00 |
|  |  | *A. sculptum* | 155 | 150 | 0.97 | 0.93 | 0.99 | 0.99 | 0.98 | 1.00 |
|  |  | *A. triste* | 156 | 149 | 0.96 | 0.91 | 0.98 | 0.99 | 0.98 | 0.99 |
| ResNet-50 | Female | *A. aureolatum* | 58 | 51 | 0.88 | 0.77 | 0.94 | 0.95 | 0.92 | 0.97 |
|  |  | *A. cajennense* | 82 | 78 | 0.95 | 0.88 | 0.98 | 0.97 | 0.94 | 0.98 |
|  |  | *A. dubitatum* | 34 | 21 | 0.62 | 0.45 | 0.76 | 0.97 | 0.94 | 0.98 |
|  |  | *A. ovale* | 79 | 72 | 0.91 | 0.83 | 0.96 | 0.97 | 0.95 | 0.99 |
|  |  | *A. sculptum* | 63 | 49 | 0.78 | 0.66 | 0.86 | 0.97 | 0.95 | 0.99 |
|  |  | *A. triste* | 52 | 40 | 0.77 | 0.64 | 0.86 | 0.98 | 0.96 | 0.99 |
|  | Male | *A. aureolatum* | 63 | 59 | 0.94 | 0.85 | 0.98 | 0.98 | 0.97 | 0.99 |
|  |  | *A. cajennense* | 83 | 80 | 0.96 | 0.90 | 0.99 | 0.99 | 0.98 | 1.00 |
|  |  | *A. dubitatum* | 75 | 67 | 0.89 | 0.80 | 0.94 | 0.99 | 0.97 | 1.00 |
|  |  | *A. ovale* | 42 | 36 | 0.86 | 0.72 | 0.93 | 0.99 | 0.97 | 0.99 |
|  |  | *A. sculptum* | 92 | 88 | 0.96 | 0.89 | 0.98 | 0.98 | 0.96 | 0.99 |
|  |  | *A. triste* | 103 | 99 | 0.96 | 0.90 | 0.98 | 0.99 | 0.97 | 1.00 |
|  | Dorsal | *A. aureolatum* | 63 | 56 | 0.89 | 0.79 | 0.95 | 0.95 | 0.92 | 0.97 |
|  |  | *A. cajennense* | 83 | 78 | 0.94 | 0.87 | 0.97 | 0.97 | 0.95 | 0.99 |
|  |  | *A. dubitatum* | 56 | 39 | 0.70 | 0.57 | 0.80 | 0.96 | 0.94 | 0.98 |
|  |  | *A. ovale* | 61 | 47 | 0.77 | 0.65 | 0.86 | 0.99 | 0.98 | 1.00 |
|  |  | *A. sculptum* | 81 | 72 | 0.89 | 0.80 | 0.94 | 0.98 | 0.96 | 0.99 |
|  |  | *A. triste* | 79 | 74 | 0.94 | 0.86 | 0.97 | 0.98 | 0.96 | 0.99 |
|  | Ventral | *A. aureolatum* | 58 | 52 | 0.90 | 0.79 | 0.95 | 0.98 | 0.96 | 0.99 |
|  |  | *A. cajennense* | 82 | 77 | 0.94 | 0.87 | 0.97 | 0.99 | 0.97 | 1.00 |
|  |  | *A. dubitatum* | 52 | 43 | 0.83 | 0.70 | 0.91 | 0.98 | 0.96 | 0.99 |
|  |  | *A. ovale* | 60 | 53 | 0.88 | 0.78 | 0.94 | 0.97 | 0.94 | 0.98 |
|  |  | *A. sculptum* | 74 | 64 | 0.86 | 0.77 | 0.92 | 0.98 | 0.96 | 0.99 |
|  |  | *A. triste* | 77 | 72 | 0.94 | 0.86 | 0.97 | 0.97 | 0.95 | 0.99 |
|  | Low resolution | *A. aureolatum* | 53 | 49 | 0.92 | 0.82 | 0.97 | 0.97 | 0.95 | 0.99 |
|  |  | *A. cajennense* | 82 | 76 | 0.93 | 0.85 | 0.97 | 0.99 | 0.97 | 1.00 |
|  |  | *A. dubitatum* | 44 | 34 | 0.77 | 0.63 | 0.87 | 0.98 | 0.95 | 0.99 |
|  |  | *A. ovale* | 55 | 49 | 0.89 | 0.78 | 0.95 | 0.98 | 0.96 | 0.99 |
|  |  | *A. sculptum* | 17 | 15 | 0.88 | 0.66 | 0.97 | 0.99 | 0.98 | 1.00 |
|  |  | *A. triste* | 77 | 75 | 0.97 | 0.91 | 0.99 | 0.97 | 0.94 | 0.98 |
|  | High resolution | *A. aureolatum* | 68 | 65 | 0.96 | 0.88 | 0.98 | 0.97 | 0.94 | 0.98 |
|  |  | *A. cajennense* | 83 | 80 | 0.96 | 0.90 | 0.99 | 0.98 | 0.97 | 0.99 |
|  |  | *A. dubitatum* | 65 | 52 | 0.80 | 0.69 | 0.88 | 0.98 | 0.96 | 0.99 |
|  |  | *A. ovale* | 66 | 54 | 0.82 | 0.71 | 0.89 | 0.99 | 0.97 | 1.00 |
|  |  | *A. sculptum* | 138 | 130 | 0.94 | 0.89 | 0.97 | 0.99 | 0.97 | 0.99 |
|  |  | *A. triste* | 79 | 71 | 0.90 | 0.81 | 0.95 | 0.99 | 0.97 | 0.99 |
|  | All together | *A. aureolatum* | 121 | 117 | 0.97 | 0.92 | 0.99 | 0.97 | 0.96 | 0.98 |
|  |  | *A. cajennense* | 165 | 158 | 0.96 | 0.92 | 0.98 | 0.99 | 0.98 | 1.00 |
|  |  | *A. dubitatum* | 109 | 89 | 0.82 | 0.73 | 0.88 | 0.98 | 0.97 | 0.99 |
|  |  | *A. ovale* | 121 | 104 | 0.86 | 0.79 | 0.91 | 0.99 | 0.98 | 0.99 |
|  |  | *A. sculptum* | 155 | 148 | 0.95 | 0.91 | 0.98 | 0.99 | 0.97 | 0.99 |
|  |  | *A. triste* | 156 | 147 | 0.94 | 0.89 | 0.97 | 0.98 | 0.97 | 0.99 |
